# 34th Annual Meeting & Pre-Conference Programs of the Society for Immunotherapy of Cancer (SITC 2019): part 1: National Harbor, MD, USA. 6-10 November 2019

**DOI:** 10.1186/s40425-019-0763-1

**Published:** 2019-11-06

## Poster Presentations

### Biomarkers, Immune Monitoring, and Novel Technologies

#### P1 Peritumoral neutrophil infiltration predicts recurrence of hepatocellular carcinoma following liver transplantation

##### Marc Najjar, MD^1^, Michael Ross^1^, Ayush Srivastava^1^, Robyn Gartrell, MD^1^, Emanuelle Rizk, BA^1^, Olivia Perez^1^, Evan Lieberman^1^, Charles Drake, MD, PhD^1^, Ladan Fazlollahi^1^, Helen Remotti^1^, Elizabeth Verna^1^, Karim Halazun^2^, Jean Emond^1^, Yvonne Saenger, MD^1^

###### ^1^Columbia University Medical Center, New York, NY, United States; ^2^Weill Cornell Medicine, New York, NY, United States

####### **Correspondence:** Marc Najjar (mn2594@cumc.columbia.edu)

**Background**

Hepatocellular carcinoma (HCC) is the most common liver malignancy and the 5th cause of cancer-related mortality worldwide. Though previous studies have found that serum neutrophil-to-lymphocyte ratio (NLR) is predictive of survival post liver transplant (LT), peritumoral neutrophil (PMN) infiltration in the tumor microenvironment (TME) of HCC has not been thoroughly investigated yet. In this study we sought to evaluate tissue based PMN infiltration in HCC post LT using quantitative multiplex immunofluorescence (qmIF), previously used to study the TME of several other tumor types[1].

**Methods**

A database of 634 patients was created at Columbia University Irving Medical Center (CUIMC) including adult patients with available clinical follow up who underwent liver transplantation (LT) for HCC between 1998 and 2018. We evaluated a preliminary cohort of 10 patients using qmIF, excluding patients with viral hepatitis. FFPE tumor sections were pre-selected by a GI pathologist. Slides were stained using qmIF for MPO (PMNs), CD3 (T cells), CD8 (cytotoxic T cells), CD68 (macrophages), HLA-DR (immune activation), and Hep-Par1 (hepatocytes/tumor). Multiplex images were visualized using Vectra (Akoya) and processed using inForm (Akoya). Data was analyzed using R Studio for concatenation, density, nearest neighbor and statistical analysis. Serum NLR was calculated using complete blood counts collected prior to LT(Figure 1).

**Results**

Preliminary cohort of 10 patients includes 4 with recurrence at a median of 2.4 years and 6 with no recurrence at a median of 12 years post-LT. We found that patients with recurrence post-LT have significantly higher densities of MPO+ PMNs compared to those with no recurrence. This difference is primarily driven by PMNs located within the peritumoral stroma (Median [interquartile range [IQR] 2.46 [1.99 - 2.92] vs 1.23 [0.723 -1.78], p=0.019). Intratumoral PMN infiltration was not associated with recurrence (Median [IQR] 0.91 [0.59 - 1.20] vs 1.33 [0.56 – 1.90], p=0.308). Moreover, density of CD3, both intratumoral and peritumoral, did not correlate with recurrence, nor did the tissue-derived NLR. Further, we found that the tissue-derived NLR did not correlate with NLR in blood.

**Conclusions**

Higher densities of peritumoral PMNs are associated with post-LT HCC recurrence. Evaluation of TME using qmIF can be used to predict recurrence in post-LT HCC. Further, tissue based analysis of PMNs does not correlate with serum NLR allowing potential for composite biomarkers. As this is preliminary, further analysis is underway and will be validated on the larger cohort of patients.

**Reference**

1. Gartrell RD, Marks DK, Hart TD, et al. Quantitative Analysis of Immune Infiltrates in Primary Melanoma. Cancer Immunol Res 2018;6:481-93.

**Fig. 1 (abstract P1).**
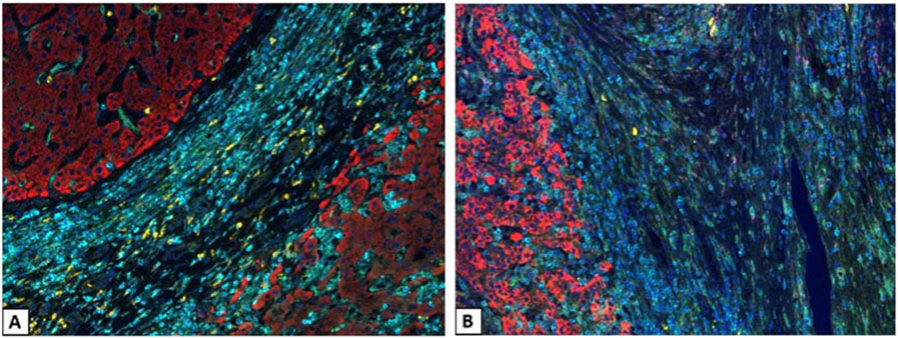
Quantitative multiplex immunofluorescence images of HCC

#### P2 Single-cell RNAseq analysis of the effects of cryopreservation on primary tumor tissue

##### Shawn Fahl (shawn.fahl@dls.com)

###### Discovery Life Sciences, Huntsville, AL, United States

**Background**

The tumor microenvironment is a complex mixture of multiple cell types, and numerous therapeutic interventions have been developed targeting distinct aspects of this environment. Tumor tissue samples are an integral part of identifying and understanding potential therapeutic targets within the tumor microenvironment of multiple cancer indications. As early biomarker discovery is often hindered by the logistical demands of sourcing fresh human tumor tissue, cryopreserved dissociated tumor cell suspensions provide a viable alternative for accessing multiple, highly-annotated tumor samples for complex studies. Previous evaluations of cryopreservation on viable tumor tissue have relied on flow or mass cytometry which, while powerful, are limited in the number of targets that can be analyzed. Single cell gene expression can analyze the expression of significantly more targets and provide a clearer picture on the effects of cryopreservation on the cellular composition of the tumor.

**Methods**

Multiple unique primary tumor samples were dissociated to the single-cell level and profiled by flow cytometry. These single cell suspensions were subsequently subjected to single cell RNASeq using the 10X Genomics platform prior to, and immediately following, cryopreservation. Data was subsequently analyzed to determine how cryopreservation impacted the cellular composition of the tumor microenvironment.

#### P3 Predicting patient response to checkpoint blockade therapy using in vitro 3D cultures

##### Kathryn Appleton, PhD, Ashley Elrod, Qi Jin Guo, Dennis Ruder, Tessa DesRochers, PhD

###### KIYATEC, Inc., Greenville, SC, United States

####### **Correspondence:** Tessa DesRochers (tessa.desrochers@kiyatec.com)

**Background**

Knowledge of immune responses that correlate with clinical outcome is essential for the development of strategies to harness a patient’s immune system to eradicate cancer. Pre-clinical platforms that recapitulate the immune response in the context of cancer are necessary for adequate understanding and detection of clinical efficacy, however, the technology to accurately test immuno-oncology (I/O) therapy response is lacking. Despite the value animal models provide in a pre-clinical setting, they lack matched patient tumor and immune cell interactions. To address this shortcoming, we developed in vitro 3D tissue models that maintain autologous patient tumor cells and immune cells for the testing and prediction of immune cell responses. We hypothesize that these 3D tissue models will recapitulate the patient tumor microenvironment and detect response to I/O agents.

**Methods**

Tumor cells and T-cells were obtained from seven melanoma patient biopsies and screened for PD-L1 and lymphocyte populations prior to incorporation into 3D culture. Effector cell to Tumor cell (E:T) optimization assays were conducted with expanded T-cells at different densities and co-cultured at different time points with tumor cells. Viability was measured using CellTiter-Glo® 3D. T-cell response was determined using flow cytometry following 24-hour co-culture with tumor cells. Microtumors were established using a biologically inert scaffold and extracellular matrix components. Microtumor viability was determined using PrestoBlue and T-cell infiltration was determined via flow cytometry. Analyte secretion was determined from supernatant using Milliplex MAP Human CD8+ T-cell Panel.

**Results**

We detected pembrolizumab binding to T-cells in a dose dependent manner and an increase in the activation marker CD69 on T-cells following tumor cell and pembrolizumab treatment in three of four patients tested. We devised an initial E:T optimization screen to identify a patient-specific ratio which renders our subsequent therapy response profiling highly personalized. CD3+CD8+ T-cell mediated tumor cell death and enhanced killing was detected in the presence of pembrolizumab. Immune cell infiltration as well as therapy related cell death was observed in our 3D microtumors. Altered patient specific cytokine secretion was measured when the cultures were treated with pembrolizumab and significantly correlated with pembrolizumab induced reduction of microtumor growth rates.

**Conclusions**

The data generated from these two complex 3D in vitro models allows us to better understand immune responses to autologous tumor cells and checkpoint blockade. Our models are therefore ideal and complimentary for preclinical testing of new I/O agents as well as patient response predictions to I/O based therapies.

**Ethics Approval**

Tissue was acquired with approval from Prisma Health's Institutional Review Board, PRO# 00069834.

#### P4 Novel immune competent murine glioblastoma models derived from Nestin-CreER^T2^; Quaking^L/L^; P53^L/L^; PTEN^L/L^ mice

##### Chao-Hsien Chen, MD^1^, Renee Chin, MS^1^, Genevieve Hartley, PhD^2^, Cheng-En Hsieh, MD^1^, Rishika Prasad, MS^2^, Takashi Shingu, PhD^2^, David Hong, MD^2^, Jian Hu, PhD^2^, Michael Curran, PhD^2^

###### ^1^The University of Texas MD Anderson Cancer Center UTHealth Graduate School of Biomedical Sciences, Houston, TX, United States; ^2^The University of Texas MD Anderson Cancer Center, Houston, TX, United States

####### **Correspondence:** Michael Curran (MCurran@mdanderson.org)

**Background**

The widely used glioblastoma multiforme (GBM) model GL261 is highly immunogenic and readily cured by checkpoint blockade limiting its use for pre-clinical modeling of immunotherapy for human GBM [1,2]. We developed four novel murine immunocompetent glioblastoma stem cell (QPP) lines derived from Nestin-CreER^T2^ Quaking (QKI)^L/L^; P53^L/L^; PTEN^L/L^ mice, reflecting a common set of alterations in patients [3-5]. The four QPP cell lines are syngeneic to C57BL/6J mice and exhibit distinct responses to T-cell checkpoint blockade.

**Methods**

The differential responsiveness of each QPP line was assessed through analysis of tumor growth in the brain versus the flank in untreated, αPD-1, or αCTLA-4 treated mice. The impact of tumor genomic landscape on responsiveness at each site was measured through whole exome sequencing. To understand cellular factors modulating responsiveness of these GBM lines to checkpoint blockade, the immune microenvironments of sensitive (QPP7) versus resistant (QPP8) lines were compared in the brain using high parameter flow cytometry. Drivers of flank sensitivity versus brain resistance were also measured for QPP8.

**Results**

QPP GBM lines demonstrate a range of sensitivities to CTLA-4 and PD-1 blockade when implanted on the flank ranging from complete sensitivity (QPP7) to complete resistance (QPP4). In the brain, QPP7 remains sensitive to both antibodies, but QPP4 and QPP8 fail to respond to blockade of either checkpoint (Figure 1). Analysis of the QPP8 immune infiltrate in skin reveals enhanced ratios of CD8s to Treg and myeloid suppressors in response to checkpoint blockade; however, none of these benefits manifest in the brain QPP8 except a very specific increase in CD8s relative to granulocytic suppressors (Figure 2). Brain-implanted QPP8 reacts adaptively to checkpoint blockade by upregulating PD-L1 expression across its myeloid stroma. In contrast, immune-responsive QPP7 does not induce PD-L1 and shows markers of enhanced CD8 T cell fitness. Consistent with these observations, genomic analysis reveals a higher mutation density in QPP7 versus the other QPP lines. Using checkpoint-insensitive QPP4/8, we have now identified agonists of the Stimulator of Interferon Genes (STING) pathway as highly promising therapeutics for treating these tumors in the brain.

**Conclusions**

We have developed novel syngeneic models of GBM with relevant genetics and immune sensitivities relative to human disease. Through comparing T cell checkpoint blockade sensitive versus insensitive variants of these QPP lines, and through comparing variant sensitivity dictated by site of implantation, we have begun to identify the genetic and cellular components that govern immunotherapeutic sensitivity of GBM.

**References**

1. Reardon DA, Omuro A, Brandes AA, Rieger J, Wick A, Sepulveda J, Phuphanich S, de Souza P, Ahluwalia MS, Lim M, Vlahovic G, Sampson J (2017) OS10.3 Randomized Phase 3 Study Evaluating the Efficacy and Safety of Nivolumab vs Bevacizumab in Patients With Recurrent Glioblastoma: CheckMate 143. Neuro-Oncology 19: iii21-iii21.

2. Reardon DA, Gokhale PC, Klein SR, et al. Glioblastoma Eradication Following Immune Checkpoint Blockade in an Orthotopic, Immunocompetent Model. Cancer Immunol Res. 2016;4(2):124-135.

3. Hu J, Ho AL, Yuan L, et al. From the Cover: Neutralization of terminal differentiation in gliomagenesis. Proc Natl Acad Sci U S A. 2013;110(36):14520-14527.

4. Brennan CW, Verhaak RG, McKenna A, et al. The somatic genomic landscape of glioblastoma. Cell. 2013;155(2):462-477.

5. Shingu T, Ho AL, Yuan L, et al. Qki deficiency maintains stemness of glioma stem cells in suboptimal environment by downregulating endolysosomal degradation. Nat Genet. 2017;49(1):75-86.

**Ethics Approval**

All experiments were conducted according to protocols approved by the University of Texas MD Anderson Cancer Center Institutional Animal Care and Use Committee.

**Fig. 1 (abstract P4).**
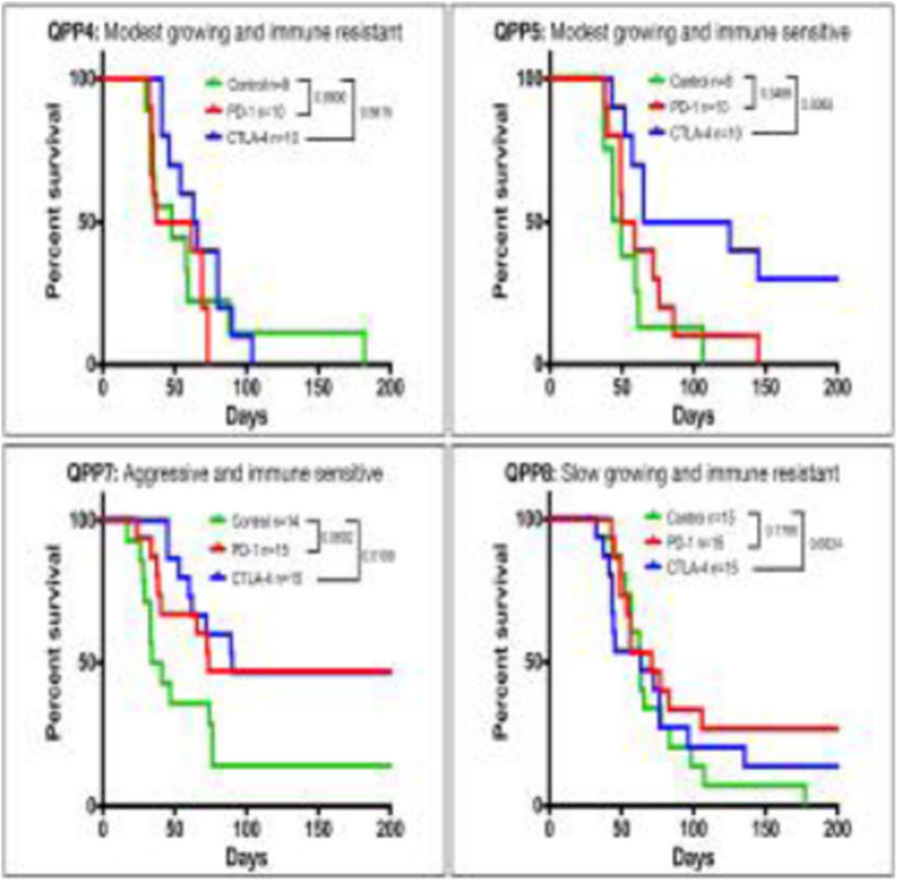
Orthotopic QPP survival and immune sensitivity

**Fig. 2 (abstract P4).**
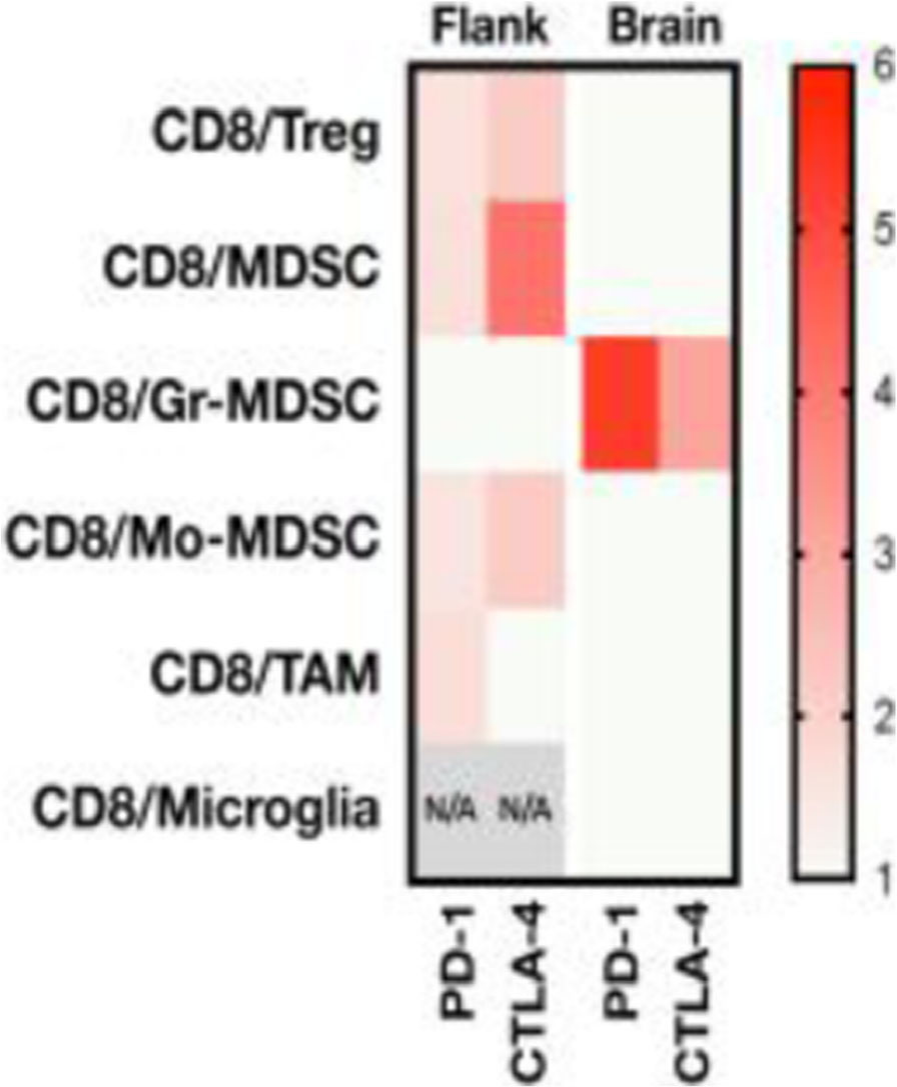
Immune landscape of QPP8 TME in different niches

#### P5 Laminar Wash™ AUTO system: a reliable walk-away sample preparation solution for better TIL recovery without centrifugation

##### Ira Kim^1^, Melvin Lye^1^, Roberta Zappasodi, PhD^2^, Isabell Schulze^2^, Christoph Eberle, PhD^3^, Chyan Ying Ke^1^, Kong Leong Cheng^1^, Ih Chin Kon^1^, Royce Pek^1^, Taha Merghoub, PhD^2^, Namyong Kim, PhD^1^

###### ^1^Curiox Biosystems, Boston, MA, United States; ^2^MSKCC, New York, NY, United States; ^3^Charles River Laboratories, Worcester, MA, United States

####### **Correspondence:** Namyong Kim (namyong@curiox.com)

**Background**

The naturally occurring tumor infiltrating lymphocytes (TILs) exist in a complex microenvironment containing the extracellular matrix, blood vessels, and stromal and endothelial components in addition to various immune cells. While a growing number of preclinical mouse models is under development, the heterogeneity of the cell composition in a solid tumor poses considerable technical challenges in isolating and characterizing the TILs for the downstream analysis. One common problem with TILs preparation occurs during solid tumor dissociation, whereby the TILs are left in a mixture with tissue debris and dead cells in suspension. Consequently, a preparation of autologous TILs often requires further costly and laborious processing such as density gradient centrifugation, immune cell sorting and enrichment, and dead cell and debris removal in combination with multiple centrifugation steps. We introduce a novel Laminar Wash™ technology, which can help overcome these technical challenges.

**Methods**

We performed pilot studies on syngeneic (MC38, CT26, CloudmanS91, 4T1) and humanized mouse tumor models as well as with human PBMCs and tumor biopsies using the Laminar Wash™ technology. Briefly, we evaluated various functional parameters of TILs such as polyfunctional CD8+ T cell responses and glucose update efficiency (2-NBDG) as well as conducting a side-by-side comparison of the TIL recovery rate and immunophenotypic characteristics of lymphoid and myeloid subsets on the Laminar Wash™ and the centrifugation-based systems. In addition, the cell retention rate, cell viability, debris removal, epitope preservation and the overall processing time were assessed and compared. Furthermore, we introduce a complete walk-away approach to sample preparation that eliminates operator-based variability while significantly enhancing reproducibility and consistency of downstream analysis.

**Results**

Our data demonstrate that the Laminar Wash™ method resulted in higher cell retention and viability, more clearly defined immune subsets, a lowered background signal, and an enhanced yield of the TILs from freshly dissociated tumor samples compared to the centrifugation-based counterparts. The Laminar Wash™ system can effectively remove the floating debris in suspension while keeping the live cells unperturbed, allowing the cell surface architecture and epitopes better retained for improved downstream analysis with flow cytometer. Additionally, the Laminar Wash™ AUTO system offers a completely automated sample processing solution for dissociated tumor samples, simplifying and expediting cell preparation with enhanced consistency and reproducibility.

**Conclusions**

Laminar Wash™ results in healthy, viable, and well defined population of TILs, while improving the overall quality of data. The AUTO station provides an automated, centrifuge-free, and walk-away workflow for dissociated tumor samples for cytometry-based assays.

**Acknowledgements**

Laminar Wash™ results in healthy, viable, and well defined population of TILs, while improving the overall quality of data. The AUTO station provides an automated, centrifuge-free, and walk-away workflow for dissociated tumor samples for cytometry-based assays.

#### P6 Development of a peripheral blood mononuclear cells (PBMC) ImmunoGraft platform to evaluate the pharmacodynamics of Immuno-oncology therapeutics

##### Bhavana Verma, PhD, Bruce Ruggeri, Amy Wesa, PhD

###### Champions Oncology, Rockville, MD, United States

####### **Correspondence:** Bruce Ruggeri (bruggeri@championsoncology.com); Amy Wesa (awesa@championsoncology.com)

**Background**

Humanized immune system (HIS) mouse models enable in vivo studies in the context of the human immune cells with a human tumor and are critical for the development of next generation immune-oncology (IO) agents. Humanization of immunodeficient mice through the adoptive transfer of normal adult PBMC leads to rapid engraftment of human T cells to study immune-modulatory agents in the context of human tumor xenografts, but is limited by the development of xenogeneic graft-versus host disease (xGVHD). In this study, we evaluated the engraftment of PBMC in β2microglobulin null super-immunodeficient mice NSG-B2M mice, that lack MHC Class I on host tissues. A cell line-derived xenograft model (CDX) co-engrafted with PBMC (PBMC-ImmunoGraft) was characterized for humanization, tumor infiltrating leukocytes (TIL) phenotype and tumor response to checkpoint inhibitors.

**Methods**

PBMC from healthy donors (N=7) were implanted and engraftment in peripheral blood was assessed by flow cytometry. T cell memory phenotypes were assessed over time in a small cohort, and costimulatory and inhibitory T cell subsets were evaluated at the terminal time point in blood and secondary lymphoid organs. Next, NSG-B2M mice were co-implanted with MDA-MB-231 breast cancer cell line s.c, humanized with PBMC and tumor growth kinetics were monitored. Efficacy studies evaluating check point inhibitors are currently ongoing.

**Results**

Successful PBMC engraftment without xGVHD was observed in NSG-B2M mice up to 8 weeks, in contrast with MHC Class I expressing immunodeficient mice that developed xGVHD within 4-5 weeks. Dose and donor- dependent chimerism was observed. T cells were detectable in the periphery starting at 2 weeks with stable levels (up to 40% of live cells to 8 weeks) with increasing T effector memory cells over the course of study. All tumors evaluated had high levels of TIL as measured by flow cytometry and immunohistochemistry. Costimulatory and inhibitory molecules evaluated on CD4 and CD8 included 4-1BB, TIM-3, LAG-3, OX-40, as well as PD-1, which was expressed on both peripheral blood cells and in TIL. Tumor growth kinetics was unaltered by PBMC humanization through a 5-week study window.

**Conclusions**

PBMC-humanized NSG-B2M mice may represent a model for evaluating of IO therapeutics with a long study window due to the lack of xGVHD. While PBMC engraftment kinetics are donor dependent, similar phenotypes are observed and T cell subsets expressing several relevant therapeutic targets, including PD-1 are present. This model may permit a rapid in vivo method to study checkpoint blockade and other T-cell-directed IO therapeutics.

**Ethics Approval**

The study was approved by Champions Oncology's Institutional Animal Care and Use Committee (IACUC).

#### P7 Development of a natural killer (NK) ImmunoGraft platform for the evaluation of the pharmacodynamics of immuno-oncology therapeutics

##### Bhavana Verma, PhD^1^, Bruce Ruggeri^1^, Jon Weidanz, PhD^2^, Amy Wesa, PhD^1^

###### ^1^Champions Oncology, Rockville, MD, United States; ^2^Abexxa Biologics, Arlington, TX, United States

####### **Correspondence:** Bruce Ruggeri (bruggeri@championsoncology.com); Amy Wesa (awesa@championsoncology.com)

**Background**

Harnessing NK cell anti-cancer cytotoxicity has gained interest as a therapeutic strategy, and consequently improved preclinical models supporting the translation of NK cell–mediated therapies to the clinic are desired. Reproducible models with human NK engraftment into immunodeficient mice co-engrafted with cell line-derived xenograft or patient-derived xenograft tumor models have been lacking due to an inability to support NK cell engraftment and persistence. Here we evaluated IL-15-NOG mice for the engraftment and sustained survival of both ex vivo expanded and primary human NK cell isolates for establishing models that engraft effectively with both human NK cells and a PDX or CDX tumor.

**Methods**

NK cells from normal adult peripheral blood mononuclear cells (PBMC) donors (N=3) were expanded using two different commercially available kits and evaluated for NK phenotype, expansion rates and yields. Titrated doses of ex vivo expanded NK cells were adoptively transferred into IL-15-NOG mice for human chimerism, and the persistence and survival of NK cells and their immunophenotype were assessed. In separate studies, naïve NK cells enriched from PBMC were also evaluated for NK cell persistence and expansion in vivo. To establish an NK ImmunoGraft, NK cells were engrafted in xenograft tumor bearing mice and tumor growth kinetics were characterized.

**Results**

Donor dependent NK expansion was observed ex vivo, with 28 to 50-fold expansion by two weeks. NK cells expanded ex vivo were CD3-CD16+CD56± and varied based on the expansion kit utilized. Nearly all CD45+ cells in circulation were NK cells, and these peaked by week 2, and were maintained for up to 10 weeks in IL-15-NOG mice. Primary NK cells engrafted with slower kinetics, with peak abundance at 3-4 weeks. NK cells expressed granzyme B, and further functional studies are in progress. For all NK cell populations, cell density-dependent engraftment was observed with a largely stable NK phenotype observed across the study. In the absence of any therapeutic treatment, NK cell persistence and expansion in vivo did not inhibit tumor xenograft growth kinetics in IL-15-NOG mice

**Conclusions**

IL-15-NOG mice support the survival and persistence of human NK cells from both ex vivo expanded and naïve NK cells, suggesting the universality of this platform for human NK engraftment. Our preliminary studies support IL-15 NOG mouse model as a suitable system for evaluation of NK cellular therapies or NK cell-modulating therapies in the context of patient-derived or cell-line derived xenograft (PDX or CDX) mouse models

**Ethics Approval**

The study was approved by Champions Oncology's Institutional Animal Care and Use Committee (IACUC).

#### P8 Monoclonal antibody detection from formalin-fixed paraffin-embedded tumor tissues using Fab-selective proteolysis nSMOL coupled with liquid chromatography and triple quadrupole mass spectrometry

##### Takashi Shimada, PhD^1^, Noriko Iwamoto, PhD^1^, Noriko Iwamoto, PhD^1^, Yoshinobu Koguchi, MD, PhD^2^, John Cha^2^, Brian Piening, PhD^2^, Eric Tran, PhD^2^, Hong-Ming Hu, PhD^2^, Bernard Fox, PhD^2^, William Redmond, PhD^2^

###### ^1^Shimadzu Scientific Instruments, Bothell, WA, United States; ^2^Providence Cancer Center, Portland, OR, United States

####### **Correspondence:** Takashi Shimada (tashimada@shimadzu.com)

**Background**

With the development of immune checkpoint inhibitors, the focus of cancer therapy is shifting to immunotherapy. Our purpose is to develop the drug efficacy index by rapid analysis of antibodies accumulating in cancer tissue using liquid chromatography and mass spectrometry (LC-MS/MS) and by characterization of the antibody distribution in the tumor microenvironment. Using a novel proteolysis method in which antibody molecules are collected on a 100 nm resin pore and trypsin is immobilized on a 200 nm nanoparticle surface, we have developed a method for physicochemically limiting trypsin access to antibody and identifying the structural specificity of complementarity-determining regions while minimizing extra peptides and protease without depending on the type of antibody. Using this method to detect antibodies from formalin-fixed paraffin-embedded (FFPE) tumor tissues, we aim to develop novel diagnostics that can aid in therapeutic dosing and predicting responses to antibody-based therapies.

**Methods**

To demonstrate the feasibility of these approaches, the human breast and epidermoid carcinoma cell lines SKBR3 and A431 were incubated with either trastuzumab and cetuximab, which bind to erbB2 and EGFR, respectively. FFPE cell blocks were then prepared and proteins were extracted from 8 μm sections after deparaffinization and decrosslinking. The extracted proteins were subjected to the Fab-selective proteolysis nSMOL, and the signature peptides of each antibody, IYPTNGYTR for trastuzumab and SQVFFK for cetuximab, were detected via triple-quadrupole LC-MS/MS. SCID mice were subcutaneously implanted with BT474 cells and 5 days later were infused with 10 mg/kg or 20 mg/kg trastuzumab. 24 h after administration, tumor and other tissues were harvested and FFPE block were prepared for trastuzumab quantitation in FFPE tissues.

**Results**

As a result of the pretreatment protocol using the cell block, the conditions of deparaffinization, decrosslinking, and protein extraction were optimized. Mass spectra of the signature peptides from trastuzumab and cetuximab could be detected using 20,000 cells. This condition was also applied to xenograft tissue and the degree of trastuzumab accumulation was detected in FFPE tumor tissue in a dose-dependent manner.

**Conclusions**

We show that these approaches can be utilized to quantify antibody concentrations in typically-challenging FFPE specimens with good sensitivity and as such could be utilized to assess efficacy of the monoclonal antibody administered. There are also potential applications related to rapid drug screening using the patient-derived xenograft model. Our future plans are focused on adapting these solutions to the characterization of immune checkpoint inhibitor therapeutics in standard-of-care FFPE tissues obtained from patients undergoing immunotherapy.

#### P9 Immune checkpoint biomarkers in hepatocellular carcinoma (HCC): Assessment of PD-L1 and tumour mutation burden in tumour samples from clinical patients

##### Hisani Horne, PhD, MPH^1^, Young Lee^1^, Todd Creasy^1^, Rebecca Fish^1^, Jonathan Cairns^1^, Paul Scorer^1^, Janine Feng^2^, Marietta Scott, PhD^1^, Mark Gustavson^1^, Aleksandra Dudek-Madej^1^, Craig Barker^1^, Nicholas Holoweckyi^1^, Rebecca Halpin^1^, Peiyi Wang^2^, Quinea Lassiter^2^, Xiaoling Xia^2^, Mohammed Abdelwahab^2^, Weimin Li^1^, Alejandra Negro^1^, Jill Walker^1^

###### ^1^AstraZeneca, Gaithersburg, MD, United States; ^2^Roche Tissue Diagnostics, Oro Valley, United States

####### **Correspondence:** Hisani Horne (hisani.madison@astrazeneca.com)

**Background**

Programmed cell death ligand-1 (PD-L1) expression and tumour mutation burden (TMB) have been shown to be predictive of response to anti-PD-1/PD-L1 immunotherapies in various cancers. The prevalence and distribution of PD-L1 expression and/or tumour mutations in HCC and correlation with clinical characteristics are poorly understood. A better understanding of these biomarkers may help inform appropriate patient selection strategies in HCC.

**Methods**

PD-L1 expression was evaluated on tumour cells (TC), immune cells (IC), or combined TC and IC using the VENTANA PD-L1 (SP263) Assay in three independent HCC sample sets: 2 from commercial tissue banks (n = 500 and n = 2417) and 1 from patients enrolled in NCT02519348, encompassing a wide range of stage and grade of disease. NCT02519348 is a phase 2 study evaluating safety, efficacy outcomes of durvalumab with or without tremelimumab in advanced HCC. TMB was assessed in tissue by whole exome sequencing in a subset of 70 patients from NCT02519348.

**Results**

At a cut-off date of Feb 28, 2019, 282/335 (84.2%) patients enrolled in NCT02519348 were successfully evaluated for PD-L1 (Table 1). Significant expression was seen in ICs relative to TCs. Patients in NCT02519348 showed higher PD-L1 expression in TCs than commercial cohorts. In a univariate analysis using 1% cut offs, higher TC (but not IC or combined TC and IC) PD-L1 expression was associated with patients with HCV infection (p=0.003). Of 70 study patients tested, 55 were evaluable for TMB (median 2.59, range 0.46 - 5.61 Mut/Mb). Among patients with available TMB data there was no observed correlation between TMB and PD-L1 expression.

**Conclusions**

PD-L1 expression was observed in both TC and ICs in HCC, with the latter being more prevalent. Viral status and disease stage may impact PD-L1 expression in this setting, but further work is needed to confirm this. TMB and PD-L1 appear to identify distinct patient subsets in HCC.

**Trial Registration**

NCT02519348

**Ethics Approval**

The study (NCT02519348) is performed in accordance with ethical principles that have their origin in the Declaration of Helsinki and are consistent with ICH/GCP, and applicable regulatory requirements.

**Table 1 (abstract P9).**
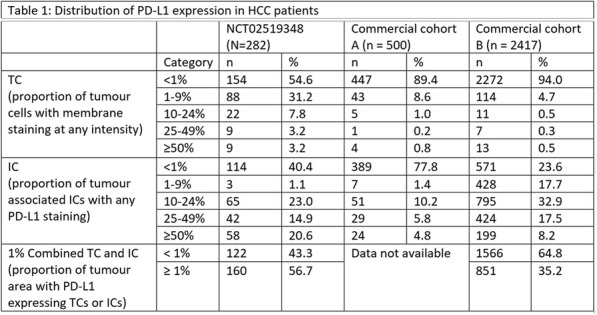
See text for description

#### P10 Inertial microfluidics enables highly consistent separation and concentration of leukocytes from human peripheral blood for downstream B-cell and T-cell functional assays

##### Sarah Mickool, Eric Smith, Aleksander Jonca, Gustavo Arnal, Mary Vincent Larcom, Melanie Scully, Peng Megn Kou, PhD, Nitin Kulkarni, Kyle Smith

###### MicroMedicine, Inc., Waltham, MA, United States

####### **Correspondence:** Kyle Smith (kyle@micromedicine.com)

**Background**

Cell separation plays a vital role in research and clinical settings for the development and monitoring of cutting-edge therapies. Despite its labor-intensiveness and variability, density gradient centrifugation-based method (DGM) has remained the primary method of upstream cell isolation for decades due to a lack of viable alternatives. This is problematic as DGM is a non-scalable, manual process. To address this lack of innovation, we have developed an automated Microfluidic System based on inertial focusing that enables label-free white blood cell (WBC) separation and concentration from 3-75mL of whole blood in short timescale with high consistency, providing reliable sample preparation for downstream functional assays.

**Methods**

WBCs were isolated from 15% ACD-A anticoagulated peripheral human blood using the Microfluidic System or DGM. Cell number, viability, and immune phenotype were evaluated by hematology analyzer and flow cytometry. To assess B-cell function, cells were cryopreserved post separation, thawed, and stimulated with IL-2 and R848, followed by Human IgG and IgM ELISPOT. To assess T-cell function, thawed cells underwent bead-based granulocyte depletion and stimulation with CEF peptide pool, followed by Human IFNγ ELISPOT.

**Results**

The prototype Microfluidic System consistently processed 40mL of anticoagulated blood in approximately 20 minutes with minimal hands-on time as opposed to 60–90 minutes for DGM with significant hands-on time. While DGM collects only peripheral mononuclear cells (PBMCs), the System isolates the total WBC population and may be beneficial for immunophenotyping. As shown in Table 1, the Microfluidic System consistently provided improved WBC or PBMC recovery, viability, purity, RBC depletion, and platelet depletion as compared to DGM. Immune phenotyping shows that the Microfluidic System also consistently resulted in improved recovery of lymphocyte subsets, including CD19+, CD3+, CD4+, and CD8+ cells (Table 2). B-cell and T-cell functionality were found to be equivalent between the two cell isolation methods based on IgM/IgG and IFNγ secretion, respectively. With the improved cell recovery using the Microfluidic System, more target cells from the same blood sample may be collected for downstream assays.

**Conclusions**

The Microfluidic System offers a faster, more reliable method than DGM for upstream cell separation from whole blood. The System consistently recovers more cells, including functional lymphocytes of different subsets, compared to DGM, potentially allowing more assays to be executed from the same blood sample. Overall, this technology has the potential to transform cell separation by automating a variable and labor-intensive processes, and therefore has utility in applications that require consistent cell quality and functionality.

**Table 1 (abstract P10).**
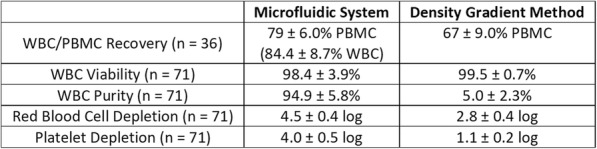
See text for description

**Table 2 (abstract P10).**
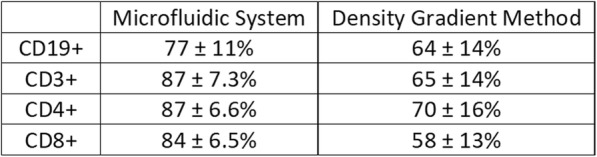
See text for description

#### P11 Early detection of breast cancer (BCa) through MDSC and lymphocyte immunophenotyping: from manual gating to pattern recognition neural networks

##### George Dominguez, PhD^1^, John Roop^1^, Alexander Polo, BS^1^, Anthony Campisi, BS^1^, Dmitry Gabrilovich, MD/PhD^2^, Amit Kumar, PhD^1^

###### ^1^Anixa Biosciences, San Jose, CA, United States; ^2^The Wistar Institute, Philadelphia, PA, United States

####### **Correspondence:** George Dominguez (george@anixa.com)

**Background**

Myeloid-derived suppressor cells (MDSCs) are contributors in supporting tumor progression and escape [1,2]. Studies have quantified MDSCs to detect tumor development, monitor progression, and/or predict therapeutic responses [3, 4]. Here, we compared several machine learning (ML) approaches to analyze flow cytometry data to detect breast cancer (stage I/II) through manual gating and hypervoxelation of cell events.

**Methods**

We used standard multiparametric flow cytometry techniques to measure myeloid-derived suppressor cell (MDSC), myeloid, and lymphocyte cell populations found in the peripheral blood of 99 biopsy-confirmed early stage BCa patients and 88 healthy donor female (HDF) controls. Manual gating was performed to generate gated values, and raw flow cytometry data were transformed using HyperVOX to generate hypervoxelated cytometry event counts. The ML algorithms used were: support vector machine (SVM), Bayes SVM, Ensemble SVM, k-nearest neighbor (kNN), and pattern recognition neural network (PRNN). All algorithms were trained using data from 64 BCa patients and 69 HDF controls. Predictions were evaluated using the performance of each trained ML algorithm on 35 early stage BCa patients and 19 HDF that were not used for training (holdout test set).

**Results**

Using manually gated counts, the resulting accuracies were: SVM = 75.4%, Bayes SVM = 71.3%, Ensemble SVM = 65.6%, and kNN = 69.7%. Using hypervoxelated event counts, the resulting accuracies were: SVM = 78.7%, Bayes SVM = 77.1%, Ensemble SVM = 57.4%, kNN = 67.2%, and PRNN = 92.6%. Hypervoxelated data analyzed using PRNN resulted in the highest accuracy with a sensitivity of 91.4% and a specificity of 94.7%; the resulting AUC = 0.9098 (95%CI = 0.8031 to 1.000). Additionally, we tested 26 samples collected from patients with confirmed ductal carcinoma in situ (DCIS) using hypervoxelated counts with a PRNN. Even though they are clinically deemed as pre-cancerous (stage 0), 18 out of 26 (AUC = 0.8421; 95%CI = 0.7163 to 0.9679) were classified as BCa suggesting utility for detecting the existence of even a non-invasive cancerous lesion.

**Conclusions**

Although further study is needed, we believe that using PRNN with MDSC immunophenotyping, in conjunction with other known clinical risk factors, would allow for clinicians to make a more informed diagnosis and treatment recommendation when screening and for recommending subsequent interventions for early stage breast cancer.

**References**

1. Kumar V, Patel S, Tcyganov E, Gabrilovich D. The nature of myeloid-derived suppressor cells in the tumor microenvironment. Trends Immunol. 2016; 37:208-220.

2. Marvel D, Gabrilovich D. Myeloid-derived suppressor cells in the tumor microenvironment: expect the unexpected. J Clin Invest. 2015; 125:3356-3364.

3. Elliott L, Doherty G, Sheahan K, Ryan E. Human tumor-infiltrating myeloid cells: phenotypic and functional diversity. Front Immunol. 2017; 8:86.

4. Okla K, Wertel I, Wawruszak A, Bobinski M, Kotarski J. Blood-based analyses of cancer: circulating myeloid-derived suppressor cells – is a new era coming? Crit Rev Clin Lab Sci. 2018.

**Ethics Approval**

The study was approved by the Virtua Oncology (#20161), University of Pennsylvania (#826544), and Cooper Health (#17-174) IRBs.

#### P12 Deep characterization of the depleted plasma proteome in subjects with NSCLC using data independent acquisition mass spectrometry reveal host immune response mechanisms

##### Nicholas Dupuis, PhD, Linda Sensbach, Sebastian Müller, Lukas Reiter

###### Biognosys AG, Schlieren, Switzerland

####### **Correspondence:** Nicholas Dupuis (nicholas.dupuis@biognosys.com)

**Background**

Measurement of circulating biomarkers in cancer has proven utility for early detection, differential diagnosis, and predicting pre-treatment response to therapy. More recently, circulating proteomic biomarkers for pre-treatment prediction of therapeutic response have received additional attention due to the heterogeneous responses to immunotherapies. To develop a greater understanding of the circulating plasma proteome in subjects with cancer we have optimized a depleted plasma proteomic workflow, based on label-free data independent acquisition (DIA) mass spectrometry, and applied it to plasma from subjects with late stage NSCLC. This approach provides a deep and unbiased description of the plasma proteome and the dysregulated biological pathways associated with lung cancer.

**Methods**

Plasma samples from subjects with Stage III-IV non-small cell lung cancer (NSCLC, n = 15) and age matched healthy donors (n = 15) were depleted of 14 high abundance proteins using MARS Hu-14 spin columns (Agilent). All samples were prepared for mass spectrometric acquisition using two-hour gradients on a C18 column coupled online to a Thermo Scientific Q Exactive HF-X operated in DIA mode. Targeted data extraction was performed using Spectronaut (Biognosys) with a hybrid library approach. Statistical analysis was conducted to identify disease associated biomarker candidates and pathway analysis highlights dysregulated biological functions.

**Results**

A comprehensive protein spectral library was created containing 1,827 unique proteins. In DIA acquisition, in total 1,304 proteins were quantified across all samples (1,105 average per sample). Univariate statistical testing identified 162 dysregulated proteins (125 up-regulated and 37 down-regulated; q-value > 0.05 and log2 fold change > 0.58). In addition to the acute phase proteins (e.g. CRP and SAA1) which were previously verified to be elevated in subjects with NSCLC, partial least squares discriminant analysis helped identify additional proteins that are differentially expressed between the sample groups. Most relevant to immune function was CLC (Galectin-10), which was elevated in NSCLC samples and has been identified as key component supporting the suppressive function of Tregs.[1] Furthermore, F13A1 was suppressed in the NSCLC samples which is known to be associated with macrophage activation.

**Conclusions**

162 proteins were identified as candidate biomarkers and reflect the host immune response via acute phase response signaling, innate immune response, and other proinflammatory stimuli. Several of these markers have been linked to patient outcomes and poor prognosis.

**Reference**

1. Kubach, J., et. al.; Blood 2007 110:1550-1558

#### P13 Immunomodulatory effects of Interleukin 2 in the circulation of melanoma patients and the added impact of VEGF inhibition with Ziv-aflibercept

##### Arjun Khunger, MD^1^, Ghanashyam Sarikonda^2^, Paul Frankel, PhD^3^, Jenn Tsau, PhD^2^, Zeni Alfonso, PhD^2^, Jane Gao, MS^2^, Anil Pahuja, BSc^2^, Christine Vaupel, PhD^2^, Naveen Dakappagari^2^, Shabnam Tangri, PhD^2^, Ahmad Tarhini, MD, PhD^4^

###### ^1^Memorial Hospital West, Pembroke Pines, FL, United States; ^2^Navigate BioPharma Services, Inc., a Novartis subsidiary, Carlsbad, CA, United States; ^3^City of Hope, Duarte, CA, United States; ^4^Emory University and Winship Comprehensive Cancer center, Atlanta, GA, United States

####### **Correspondence:** Ahmad Tarhini (tarhiniaa@gmail.com)

**Background**

Interleukin 2 (IL-2) plays a key role in antitumor immunity by enhancing survival of antitumor cytotoxic T lymphocytes and natural killer (NK) cells and promoting proinflammatory cytokines, that can lead to durable responses in patients with melanoma. High levels of vascular endothelial growth factor (VEGF) are associated with non-response to IL-2 and combination biotherapy with Ziv-aflibercept (inhibitor of the VEGF pathway) and high-dose IL-2 may lead to improved antitumor efficacy. Mechanistic studies utilizing peripheral blood of melanoma patients treated with this biotherapy may illuminate the underlying mechanisms of immune susceptibility and resistance [1].

**Methods**

Patients with stage III or stage IV inoperable melanoma were treated with high-dose IL-2 alone or in combination with Ziv-aflibercept in a phase 2 clinical trial [1] (NCI8628; Tarhini et al. Cancer. 2018). Peripheral blood mononuclear cells (PBMC) from treated patients (N=89) on this trial were tested at baseline (before initiating systemic immunotherapy), and 6-weeks (following immunotherapy initiation). High complexity (14-color) flow cytometry designed to detect key immunological biomarkers such as myeloid-derived suppressor cells (MDSCs), regulatory T cells (Tregs), proliferating T-cells, PD-1 and TIM3 expression on T-cells, and differentiation of T-cells into Th1, Th2 or Th17 phenotype were used to evaluate the correlation between immunological biomarker expression and efficacy. Statistical significance was determined using ANOVA or paired student’s t-test.

**Results**

Treatment with high dose IL-2 resulted in significant immune activation as detected by significant increases in both proliferating CD4+ (p<0.0001) and CD8+ (p<0.0001) T-cells at 6-weeks post-treatment in both treatment arms in addition to increase in Tregs (CD4+ CD25+ Foxp3+ T-cells; p<0.0001). Addition of VEGF inhibition showed a general trend towards decrease in classical monocytes (CD14+ CD16-; p=0.0769) as well as Th17 cells (defined as CD45RA- CCR6+ CXCR3- CCR4+; p=0.0597). In patients receiving combination therapy, a higher proportion of subjects experienced CBR (Clinically Beneficial Response = CR+PR+SD) compared to monotherapy and this CBR correlated with a decrease in CD4+ ICOS+ (p=0.0219), classical monocytes (CD14+ CD16-; p=0.0141), Th17 cells (CD45RA- CCR6+ CXCR3- CCR4+; p=0.0445) as well activated CD4+ T-cells (CD4+ CD38+ HLA-DR+; p=0.0285).

**Conclusions**

VEGF inhibition with Ziv-aflibercept adds significant immunomodulatory effects when combined with IL-2. Further correlative analyses determining the effect of combination therapy on progression-free survival and identifying predictive biomarkers of therapeutic efficacy are ongoing and will be presented at the meeting.

**Acknowledgements**

This United States (U.S.) National Cancer Institute (NCI)-sponsored study was initiated by the California Cancer Consortium under N01 contract NO1-CM-2011-00038. Laboratory correlatives were supported by Navigate BioPharma.

**Trial Registration**

https://clinicaltrials.gov/ct2/show/NCT01258855

**Reference**

1. Tarhini AA, Frankel P, Ruel C, Ernstoff MS, Kuzel TM, Logan TF, et al. NCI 8628: A randomized phase 2 study of ziv‐aflibercept and high‐dose interleukin 2 or high‐dose interleukin 2 alone for inoperable stage III or IV melanoma. Cancer. 2018;124(22):4332-41.

**Ethics Approval**

The study was initiated after approval by the ethics committee at the participating sites and was conducted in accordance with the Declaration of Helsinki.

#### P14 Validation of dendritic cell and natural killer cell signatures for clinical biomarker development

##### Bolan Linghu, PHD, Pei Zhang, PhD, Marylens Hernandez, Mingchao Xie, PhD, Christine Barbon, Srimathi Srinivasan, Deanna Russell, MS, Anna Coenen-Stass, Deanna Mele, PhD, Patricia McCoon, PhD, Jonathan Dry, Ben Sidders, Kris Sachsenmeier, PhD

###### AstraZeneca, Waltham, MA, United States

####### **Correspondence:** Ben Sidders (benjamin.sidders@astrazeneca.com); Kris Sachsenmeier (kris.sachsenmeier@astrazeneca.com)

**Background**

Quantification of immune cell abundance using gene signatures from mRNA profiling has the potential to inform clinical studies of cancer immunotherapy. However, few of the signatures reported in previous studies have been validated therefore the concordance of signature scores with corresponding immune cell abundance is unknown.

**Methods**

To tackle this challenge we designed a two-stage validation strategy. Firstly we validate signatures computationally using previously published datasets. Secondly we generate expression profiling data from an immune cell spike-in experiment with human PBMCs. As a proof of concept experiment, we implemented the method to validate two gene signatures for CD141+ dendritic cells (DC) and CD56+ natural killer (NK) cells.

**Results**

We demonstrate gene signatures for both CD56+ NK and CD141+ DC cell types show high and significant agreement to the corresponding immune cell abundance.

**Conclusions**

This work establishes a starting point for validating gene signatures through an approach that is tractable yet recapitulates real-world variability we might expect in clinical use.

#### P15 Microsatellite instability detection with cell-free DNA next-generation sequencing

##### Ariane Lozac’hmeur, MS, Jason Perera, PhD, Denise Lau, PhD, Aly Khan, PhD, Ariane Lozac’hmeur, MS

###### Tempus Labs, Chicago, IL, United States

####### **Correspondence:** Ariane Lozac’hmeur (ariane.lozachmeur@tempus.com)

**Background**

Microsatellite instability is a clinically actionable genomic indication for cancer immunotherapy. In microsatellite instability-high (MSI-H) tumors, defects in DNA mismatch repair (MMR) can cause a hypermutated phenotype where alterations accumulate in the repetitive microsatellite regions of DNA. MSI detection is typically performed by subjecting tumor tissue (“solid biopsy”) to clinical next-generation sequencing or specific assays, such as MMR IHC or MSI PCR. Circulating cell-free tumor DNA (cfDNA) testing (“liquid biopsy”) is rapidly emerging as a less invasive method for cancer detection and monitoring disease progression. Here, we explore the possibility of detecting MSI in cfDNA and develop a novel cfDNA MSI detection assay with high specificity.

**Methods**

The Tempus cfDNA targeted panel contains 39 highly informative microsatellite loci previously used by the clinically validated Tempus xT 595-gene panel. For each microsatellite locus, we identified all sequencing reads that mapped to the corresponding microsatellite region and quantified the number of repeat units contained within the sequencing read. Next, three distinct summary statistics were calculated to characterize the distribution of the number of repeat units for each locus. Finally, using 54 labeled patient samples (17 MSI-H, 37 microsatellite stable) sequenced with the Tempus cfDNA panel, a k-Nearest Neighbor (k-NN) classifier was trained to classify each locus for a new sample. Patient samples with more than 50% unstable loci were classified as MSI-H.

**Results**

We validated the ability of our model to detect MSI on a new independent validation dataset. MSI-H status was detected in 6 patient samples. In 3 of these patients (2 colorectal, 1 skin cancer), abnormal MMR IHC confirmed the detected MSI-H status. In the other 3 patients (1 colorectal, 1 non-small cell lung cancer, and 1 endometrial cancer), MSI-H status was confirmed by our clinically validated solid tumor MSI assay. Furthermore, the reliability of the model was validated in 10 technical replicates from 2 MSI-H patients in our training dataset. The results were 100% concordant with all 10 replicates classified as MSI-H.

**Conclusions**

These results demonstrate the ability of our assay to detect MSI in cfDNA with high specificity, providing a transformative opportunity to report a clinically actionable insight alongside other somatic changes detected from cfDNA.

#### P16 Circulating immunological biomarkers for predicting response to neoadjuvant chemotherapy in TNBC patients

##### Charlotte Milton, PhD, Thanussuyah Alaguthurai, Atousa khiabany, Mres, Sheeba Irshad, MD PhD

###### Kings College London, London, United Kingdom

####### **Correspondence:** Sheeba Irshad (sheeba.irshad@kcl.ac.uk)

**Background**

Triple negative breast cancer (TNBC) accounts for 10-20% of breast cancer and is associated with particularly poor prognosis. Patients are commonly treated with neoadjuvant chemotherapy (NAC) and response to treatment is a strong predictor of overall survival. Recently, the ability of chemotherapeutics to stimulate an anti-tumour immune response has been appreciated as an important mechanism of action; possibly contributing to the elimination of distant micro-metastatic disease by resetting of the attenuated functional immunity. In TNBCs, higher levels of tumour-infiltrating lymphocytes correlate with response to NAC and high intra-tumoral levels of immune-related genes, including those associated with type I interferon responses, and the presence of CD8+ cytotoxic T lymphocytes, correlate with improved disease outcome.

**Methods**

The underlying hypothesis of this study is that phenotypic profiling of peripheral blood cells have the potential to inform clinical decisions and help predict therapeutic response, with lower costs and higher compliance than serial tumour biopsies, due to their minimal invasiveness. Whilst significant research efforts have been made to assess circulating markers such as circulating tumour cells and circulating tumour DNA as potential biomarkers; understanding the evolving peripheral “immunological status” of TNBC patients on NAC is warranted.

We therefore set out to analyse serial blood samples from TNBC patients receiving NAC to monitor the changes in the peripheral immune response through deep analysis of functional and phenotypic immune markers. We investigated (1) whether chemotherapy affects the immune phenotype; and (2) whether a defined peripheral blood immune phenotypic profile relates to treatment response.

**Results**

Here we present preliminary results from 10 TNBC patients receiving NAC. Analysis of 39 PBMC populations using mass cytometry by time-of-flight (CyTOF), highlighted phenotypic changes in B cell populations in response to treatment, in particular a dramatic increase in circulating regulatory B cells (CD19+CD24+CD38+) post-chemotherapy (5.4% and 46.2% of B cells pre- and post-chemotherapy, respectively, p=0.0004). We also detected an increase in expression of exhaustion markers (CD38+CD39+) on CD8+ T cells which was associated with poor response to chemotherapy (0.8 and 2.7 fold increase from baseline in exhausted CD8+ T cells in patients with pathological complete response and residual disease, respectively, p=0.008).

**Conclusions**

We now plan to integrate these data with Luminex profiling of 36 serum cytokines, mass spectrometry analysis of circulating exosomes and clinicopathological and standard of care blood monitoring. Taken together, this study aims to provide a comprehensive analysis of the utility of immune monitoring to understand TNBC patient response to NAC.

**Ethics Approval**

The study was approved by NRES Committee London - Chelsea, approval number 13/LO/1248.

#### P17 Role of plasma-derived exosome in monitoring immunotherapy response and toxicity

##### Arnav Mehta, MD PhD^1^, Gyulnara Kasumova^2^, Alvin Shi^3^, Lina Hultin Rosenberg^4^, Emmett Sprecher^4^, Dennie Frederick^2^, Ryan Sullivan, MD^2^, Keith Flaherty^2^, Nir Hacohen^1^, Genevieve Boland^2^, Marijana Rucevic, PhD^4^

###### ^1^MGH and Broad Institute, Boston, MA, United States; ^2^MGH, Hanover, MA, United States; ^3^MIT, Cambridge, MA, United States; ^4^Olink Proteomics, Uppsala, MA, United States

####### **Correspondence:** Arnav Mehta (nawi214@gmail.com)

**Background**

Immune checkpoint blockade (ICB) has revolutionized the treatment of many solid tumors, including metastatic melanoma. Despite recent successes, many patients fail to respond or are overcome by severe toxicities that limit further treatment. To date, there are no non-invasive predictors of response and toxicity that can guide treatment decisions. In this work, we perform whole plasma and plasma-derived exosome proteomic profiling to construct a predictive model of immunotherapy response and toxicity, and to glean further biologic insight into the mechanisms underlying resistance to ICB.

**Methods**

Whole plasma was analyzed in a cohort of 55metastatic melanoma patients receiving anti-PD1 antibodies (MGH IRB #11-181) at baseline, and on-treatment at 6 week and 6 month time-points. Exosomes were analyzed in 15 of these patients for all time-points. Proteomic analysis was performed using an innovative multiplex proximity extension assay that enabled detection of more than 1000 proteins simultaneously. A linear mixed model with maximum likelihood estimation for model parameters was used to analyze differences between patient groups, and significant differences were determined after Benjamini and Hochberg multiple hypothesis correction.

**Results**

Between plasma baseline and on-treatment time-points, 67 differentially expressed proteins were identified including markers of inflammation such as PD1, CXCL9, CXCL10, CXCL11, IL10, CCL3 and TNFR2. Exosome samples had a distinct protein signature over the treatment period compared to plasma, including differential expression of CXCL16, CCL18, CCL20, and IL6, among others. 41 proteins were differentially expressed in plasma between ICB responders and non-responders including several inflammatory proteins such as CD28, TNFb, MCSFRa and IL8, and others implicated in melanoma resistance, such as MIA and ERBB2. Similarly, exosome revealed a distinct protein signature between responders and non-responders compared to plasma consisting of CXCL9, CXCL13, CXCL16, CCL19, CD8a, GZMA and CD5 expression. Whereas plasma proteins reflected a myeloid signature, exosome proteins reflected a lymphoid signature, suggesting that the two compartments may capture elements of different immune processes. Integrating data from both plasma and exosome proteomics, we applied machine learning tools to build a predictor of response. Further analysis to look for predictors of toxicity is currently underway.

**Conclusions**

Overall, our work suggests that plasma and exosome protein signatures are distinct and may reflect unique immunological processes. Proteomic analysis of these compartments may be an effective way for non-invasive liquid biopsy to predict ICB response.

#### P18 Liquid biopsy protein biomarkers to predict responses and elucidate resistance to cancer immunotherapy

##### Arnav Mehta, MD PhD^1^, Marijana Rucevic, PhD^2^, Gyulnara Kasumova^3^, Emmett Sprecher^2^, Lina Hultin Rosenberg^2^, Dennie Frederick^3^, Ryan Sullivan, MD^3^, Nir Hacohen^3^, Keith Flaherty^3^, Genevieve Boland^3^

###### ^1^MGH and Broad Institute, Boston, MA, United States; ^2^Olink Proteomics, Watertown, MA, United States; ^3^MGH, Hanover, MA, United States

####### **Correspondence:** Marijana Rucevic (m.rucevic@olink.com)

**Background**

The response of metastatic melanoma to anti-PD1 is heterogeneous. We performed proteomic profiling of patient plasma samples to build a predictor of immunotherapy response and uncover biological insights underlying primary resistance.

**Methods**

An initial cohort comprised 55 metastatic melanoma patients receiving anti-PD1 (Pembrolizumab or Nivolumab) at Massachusetts General Hospital (MGH), and 116 additional patients comprised a validation cohort. Plasma samples were collected baseline and on-treatment, at 6 weeks and 6 months’ time-points, and profiled for 1000 proteins by a multiplex Proximity Extension Assay (PEA, by Olink Proteomics). A subset of patients had single-cell RNA-seq (Smart-Seq2 protocol) performed on tumor tissue. Group differences and treatment effects were evaluated using linear mixed models with maximum likelihood estimation for model parameters, and Benjamini and Hochberg multiple hypothesis correction.

**Results**

At the baseline, 6 differentially expressed proteins were identified between responders (R) and non-responders (NR) whereas immune suppression marker ST2 and IL-6 were found significantly higher among NR. Kaplan-Meier survival curves stratified by the baseline differentially expressed proteins were highly predictive of overall survival (OS) and progression-free survival (PFS). At 6-weeks on-treatment time point, 80 proteins were found differentially expressed between R and NR including several proteins implicated in primary or acquired resistance (IL8, MIA, TNFR1 among others). Several 6-weeks differentially expressed proteins were highly predictive of survival (ICOSL, IL8, MIA). Furthermore, 160 significantly differentially expressed (DE) proteins were identified across the treatment period majority of which are reflective of immune activation under the pressure of the immunotherapy. Analysis of single-cell RNA-seq data of tumor tissue from a subset of these patients revealed that gene expression of most proteins predictive of response were enriched among tumor myeloid cells, with the remainder of proteins being reflective of exhausted T cell states.

**Conclusions**

These results unveil a putative role of myeloid cells within the tumor microenvironment in anti-PD1 response or primary resistance. Whole plasma proteomic profiling of anti-PD1 treated patients revealed DE proteins between R and NR that may enable a liquid biopsy to predict anti-PD1 response. Importantly, we demonstrate the relationship of serum biomarkers to OS and PFS and are currently attempting to build machine learning classifiers as predictors of response to checkpoint therapy leveraging early and late on-treatment time points.

#### P20 Semaphorin 4D in peripheral blood of head and neck squamous cell carcinoma reads the histological pattern of tumor inflammation in real time

##### Ioana Ghita, Manar Elnaggar, Risa Chaisuparat, John Papadimitriou, Joshua Lubek, Rania Younis, BDS, MDS, PhD, Soren Bentzen, PhD

###### University of Maryland, Baltimore, MD, United States

####### **Correspondence:** Rania Younis (ryounis@umaryland.edu)

**Background**

There is an urgent need for immune biomarkers that can monitor the status of inflammation of cancer patients. Soluble biomarkers represent a convenient prognostic and diagnostic method. Semaphorin 4D (Sema4D) is a glycoprotein that can function as a transmembrane protein or a cleaved soluble form (sSema4D), that we previously detected in peripheral blood [1]. The role of Sema4D as an inflammatory mediator in several pathological aspects and its role in tumor immune suppression [2,3], highlights its significance as a molecule to be further investigated for translational potential. The objective of this work was to investigate the level of sSema4D in plasma in relation to the histological pattern of tumor inflammation of head and neck squamous cell carcinoma (HNSCC) patients in real time.

**Methods**

Under University of Maryland institutional review board approval and upon patient consent, we obtained paired peripheral blood and tumor tissue of thirty-nine HNSCC patients, collected at the same time point to allow for real time correlative analysis. Thirty eight patients of classic autoimmune conditions, thirteen allergy patients, seven osteoarthritis patients and thirty-one healthy donors were included as controls. The level of Sema4D in plasma was detected using tailored direct ELISA assay. The histological pattern of tumor inflammation [4] was analyzed by three pathologists using the immunohistochemical staining of Sema4D of the tumor associated inflammatory cells (TAIs).

**Results**

sSema4D levels in plasma of HNSCC and the autoimmune individuals (p=0.18, independent-samples Mann-Whitney test), were not statistically significantly different, but sSema4D levels were significantly higher in the HNSCC and the autoimmune groups compared to healthy donors (p<0.001 for both comparisons). Three histological patterns of tumor inflammation were defined according to the extent of stromal inflammation and TAIs infiltrate into the tumor islands. First; the inflamed type (TAIs infiltrated the tumor cells), second the TAIs excluded type (inflamed stroma but TAIs did not infiltrate the tumor islands and/or were excluded by a thin peri-tumoral fibromyxoid zone) and third as deserted ( minimal to no TAIs in the peri-tumroal stroma or the tumor islands). The paired tumor tissue and blood samples collected at the same time point, showed that high levels of sSema4D in plasma, correlated directly with TAIs excluded histological pattern of tumor inflammation (p= 0.04).

**Conclusions**

Our data presents a novel role of Sema4D as a soluble immune biomarker that can read in real time the histological pattern of tumor inflammation. This opens new avenues for personalized immunotherapy and HNSCC patient stratification.

**References**

1. Derakhshandeh R, Sanadhya S, Lee Han K, Chen H, Goloubeva O, Webb TJ, Younis RH. Semaphorin 4D in human head and neck cancer tissue and peripheral blood: A dense fibrotic peri-tumoral stromal phenotype. Oncotarget. 2018; 9:11126-11144.

2. Younis RH, Han KL, Webb TJ. Human Head and Neck Squamous Cell Carcinoma-Associated Semaphorin 4D Induces Expansion of Myeloid-Derived Suppressor Cells. J Immunol. 2016; 196:1419-29.

3. Clavijo PE, Friedman J, Robbins Y, Moore EC, Smith E, Zauderer M, Evans EE, Allen CT. Semaphorin4D Inhibition Improves Response to Immune-Checkpoint Blockade via Attenuation of MDSC Recruitment and Function. Cancer Immunol Res. 2019; 7:282-291.

4. Ayers M, et al. IFN-gamma-related mRNA profile predicts clinical response to PD-1 blockade. J Clin Invest. 2017; 127:2930–40

**Ethics Approval**

The study was approved by University of Maryland institutional review board, Institutution‘s Ethics Board, approval number (HCR-HP-00073603)

#### P21 Activation Profiling of tumor infiltrating CD8+ T cells reveals CTLA-4 mean fluorescence intensity correlates with response in treatment naïve melanoma

##### Lauren Levine, MD, Katy Tsai, MD, James Lee, MD, Clinton Wu, BS, Kelly Mahuron, MD, Alain Algazi, MD, Michael Rosenblum, MD PhD, Adil Daud, MD

###### University of California, San Francisco, San Francisco, CA, United States

####### **Correspondence:** Adil Daud (daudai@gmail.com)

**Background**

Background: Activation markers such as PD-1 and PDL-1 as well as tumor mutation burden and IFN-gamma gene expression profiling have been explored as markers for response in melanoma and in other cancers. PD-1 inhibition activates checkpoint positive cytotoxic T lymphocytes (cpCTLs) inducing tumor regression. We have previously demonstrated that baseline peCTL frequency predicts response to anti–PD-1 monotherapy and combination CTLA4/PD-1 blockade in metastatic melanoma. We evaluated the frequency of this CD8+ T cell subset at baseline and after immunotherapy treatment and evaluated the utility of the intensity of expression activation marker expression as a surrogate for tumor response as assessed by flow cytometry.

**Methods**

We identified 490 patients with melanoma biopsied pre and post PD-1 therapy and available for analysis. Of these 148 patients had unresectable stage III or stage IV melanoma and were treatment naïve and started PD-1 therapy following biopsy. An additional 61 patients were identified with PD-1 resistant melanoma. Approximately 2 × 106 cells were stained with anti-hCD3, anti- hCD8, anti-hCD45, anti-CD4 , anti-Foxp3, anti–hCTLA-4 (14D3), anti–PD-1 , anti–HLA-DR, anti–PD-L1, and LIVE/DEAD Fix- able Aqua Dead Cell Stain (Life Technologies). Data were acquired by an LSRFortessa (BD Biosciences) and analyzed using FlowJo software (Tree Star, Inc.). Objective Responses were evaluated by RECIST 1.1, CR/PR were classified as “responders” and SD/PD as “non-responders.”

**Results**

: cpCTL percentage correlated with response. The mean cpCTL was 27.1% for treatment naïve responders (R), 16.52% for treatment naïve non-responders (NR) and 8.59% for PD-1 resistant patients post treatment (ANOVA p=0.0003 for R/NR, 801 (ANOVA p=0.0002).

**Conclusions**

PD-1 progressive patients are significantly depleted in cpCTL even compared to treatment naïve non-responders, suggesting that additional T cell influx may be needed for effective checkpoint blockade in these patients. In treatment naïve melanoma, CD8+ activation as shown by CTLA-4 MFI has an optimal range along the activation-dysfunction spectrum, and strongly correlates with response to PD-1 checkpoint therapy.

**Acknowledgements**

We gratefully acknowledge the patients who participated in this study

**Ethics Approval**

The study was approved by UCSF's Ethics Board approval number 138510

#### P22 Transcriptomic characterization of immune response within diverse tumor environments using the NanoString® nCounter® PanCancer IO 360™ assay

##### Jessica Perez, PhD, Lei Yang, David Henderson, PhD, Heather Brauer, PhD, Sarah Warren, PhD

###### NanoString, Seattle, WA, United States

####### **Correspondence:** Sarah Warren (swarren@nanostring.com)

**Background**

The efficacy of immune response in solid tumor settings is driven by many factors including the biology of the tumor, the immune system, and the microenvironment. The Tumor Inflammation Signature (TIS) is an 18-gene Research Use Only (RUO) signature that measures the presence of a preexisting immune response on the nCounter platform and enriches for response to pembrolizumab [1]. We have incorporated TIS into the PanCancer IO 360 panel, a 770-gene RUO expression assay containing 48 additional signatures of tumor-immune biology. To accompany this panel, we have created analysis software that associates the gene expression and signature scores with annotations of the samples to characterize the immune system, tumor, and stroma within the tumor microenvironment to give insight into underlying biology of response to treatment, disease progression, survival, and other sample characteristics.

**Methods**

The PanCancer IO 360 assay relies on gene signatures to describe biological processes, measure the presence of 14 different immune cell populations, or report the expression of key therapeutic targets. Data from The Cancer Genome Atlas (TCGA) was used for signature training and development. Signatures are either single genes, weighted linear sums of multiple genes with coregulated expression, or algorithms to determine under-expression of genes in a coregulated pathway [2,3]. The analysis software leverages differential expression analysis and Cox proportional hazard modeling to associate gene expression and signature scores with the clinical annotations.

**Results**

In the PanCancer IO 360 analysis, genes and signatures are compared to clinical annotations through heat maps, volcano plots, forest plots, box plots, waterfall plots, swim lane plots, Kaplan Myer plots, scatter plots, and the IO 360 wheel plot. The report is delivered in an HTML format that provides interactive visualizations, quality control, and downloadable results. Data are analyzed individually and as part of larger treatment groups.

**Conclusions**

The PanCancer IO 360 assay is a tool for characterizing transcriptional patterns associated with tumor-immune interactions that can be applied across a wide range of cancer types. Gene signatures enable robust characterization of immune activity from small sample cohorts, and the report simplifies the interpretation of results. This combination enables researchers to have insight into clinically relevant biology that will ultimately lead to help drive the immune-oncology field.

**References**

1. Ayers M, Lunceford J, Nebozhyn M, et al. IFN-γ-related mRNA profile predicts clinical response to PD-1 blockade. J Clin Invest. 2017;127(8):2930-2940.

2. Danaher P, Warren S, Dennis L, et al. Gene expression markers of Tumor Infiltrating Leukocytes. J Immunother Cancer. 2017;5:18.

3. Danaher P, Warren S, Lu R, et al. Pan-cancer adaptive immune resistance as defined by the Tumor Inflammation Signature (TIS): results from The Cancer Genome Atlas (TCGA). J Immunother Cancer. 2018;6(1):63.

#### P23 High dimensional immune monitoring of peripheral blood samples from breast cancer patients using mass cytometry (CyTOF)

##### Jose Villasboas, MD^1^, Kaitlyn McGrath, MS^1^, El-ad David Amir, PhD^2^, Roberto Leon-Ferre, MD^1^, Matthew Goetz, MD^1^, Judy Boughey, MD^1^, Jody Carter, MD, PhD^1^, Krishna Kalari, PhD^1^, Liewei Wang, MD, PhD^1^, Vera Suman, PhD^1^, Richard Weinshilboum, MD^1^, Stephen Ansell, MD, PhD^1^

###### ^1^Mayo Clinic, Rochester, MN, United States; ^2^Astrolabe Diagnostics, Fort Lee, NJ, United States

####### **Correspondence:** Jose Villasboas (Villasboas@mayo.edu)

**Background**

CyTOF produces high dimensional single cell data allowing simultaneous monitoring of multiple immune cell subsets. This enables characterization of the immune system in normal and disease states. We developed a standardized pipeline to study human peripheral blood mononuclear cells (PBMCs) of cancer patients. Here we detail our process and present early findings on a cohort of 40 patients with early-stage triple-negative breast cancer (TNBC) treated with neoadjuvant chemotherapy.

**Methods**

Thirty commercially-available metal-tagged antibodies were optimized to identify major cell subsets using a 4-point titration scheme. Replicates of cryopreserved PBMCs from a pool of 4 healthy donors were created for panel titration and used as longitudinal references. We studied 40 cryopreserved PBMCs from patients with TNBC. We stained samples individually using standard protocol, barcoded overnight during DNA intercalation, and pooled for acquisition. Debarcoded output data was normalized on a per-batch basis to the median intensity of EQbeads. We uploaded files to an automated platform for unbiased processing. Patient-level meta-data was added to experiment matrix to determine differential abundance of immune subsets across clinical and pathological groups.

**Results**

We required 7 rounds of titration to optimize antibody concentrations. Data was collected on over 23 million live single-cell events (Table 1) assigned to 31 canonical populations (Figure 1A). The median frequencies of main populations were: B cells (11.9%), T-CD4+ cells (34.3%), T-CD8+ cells (11.7%), NK cells (8.6%), and monocytes (11.3%). At the profiling level, 76 subsets were agnostically identified, with B, T, NK, and monocytes broken down into 10, 32, 8, and 13 subsets respectively. Activated (CD38+CD161+) CD16+NK cells (Figure 1B) were more prevalent in TNBC samples (median 5.2%, range 0.5%-11.9%) compared to normal blood (median 0.76%, range 0.1%-2.4%). A population with phenotype suggestive of myeloid derived suppressor cells (LineagenegHLA-DRLowCD66b+CD24+CD16+; Figure 1C) was also more prevalent in TNBC samples (median 1.2%, range 0.1%-17.3%) compared to normal blood (median 0.6%, range 0.2%-1.0%). These populations demonstrated opposite association trends when patients were stratified by clinical outcomes. Activated NK cells were more frequent in patients achieving pathological complete response while MDSC-like cells were more frequent in those with residual disease (Figures 1D-1E).

**Conclusions**

We demonstrated the feasibility of a complete pipeline for deep phenotyping of cryopreserved PBMCs in cancer patients. Our approach identified rare cell subsets using an unbiased analysis tool, linking specific populations to opposite clinical outcomes. High dimensional immune monitoring is feasible and should be applied to study the immune system of cancer patients at large.

**Acknowledgements**

This work was part of the Mayo Clinic Cancer Immunome Project which is supported by the Wohlers Family Foundation. Samples were obtained in collaboration with investigators from the Mayo Clinic Breast Cancer Genome Guided Therapy (BEAUTY) study. The BEAUTY study is funded in part by the Mayo Clinic Center for Individualized Medicine; Nadia’s Gift Foundation; John P. Guider; the Eveleigh Family; George M. Eisenberg Foundation for Charities; generous support from Afaf Al-Bahar; and the Pharmacogenomics Research Network (PGRN). Other contributing groups include the Mayo Clinic Cancer Center and the Mayo Clinic Breast Specialized Program of Research Excellence (SPORE).

**Ethics Approval**

The study was reviewed approved by the Mayo Clinic Institutional review board (IRB).

**Fig. 1 (abstract P23).**
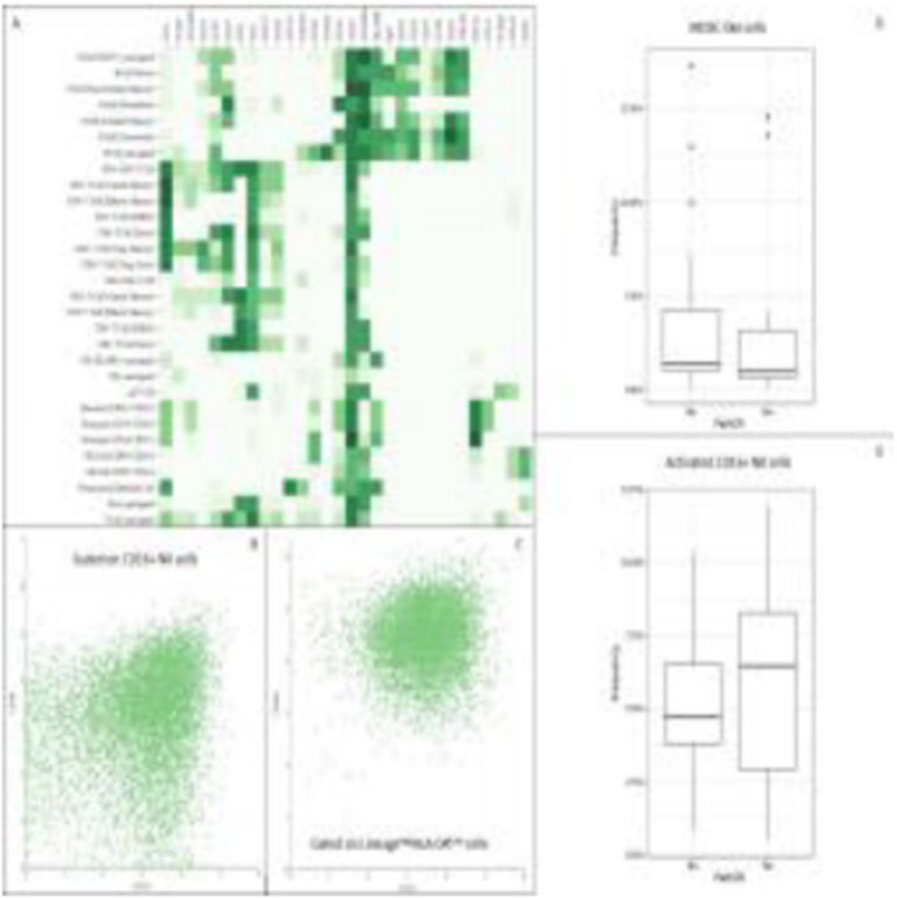
High dimensional immune monitoring of breast cancer PBMCs

**Table 1 (abstract P23).**
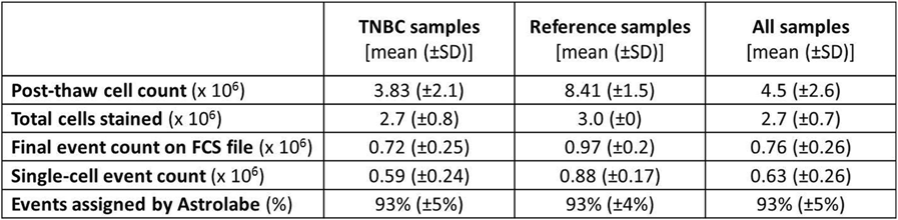
See text for description

#### P24 Molecularly guided digital spatial profiling for highly multiplexed analysis of gene expression with spatial and single cell resolution

##### Anushka Dikshit, PhD^1^, Chris Merritt, PhD^2^, Jamie Rose Kuhar^2^, Karen Nyugen^2^, Kristina Sorg^2^, Bingqing Zhang^1^, Courtney Anderson, PhD^1^, Xiao-Jun Ma^1^

###### ^1^Advanced Cell Diagnostics, Newark, CA, United States; ^2^NanoString Technologies, Seattle, WA, United States

####### **Correspondence:** Xiao-Jun Ma (xiao-jun.ma@bio-techne.com)

**Background**

The tumor microenvironment (TME) is a network of complex interactions between the tumor and surrounding immune cells. Immunotherapies including immune checkpoint blockade have demonstrated therapeutic efficacy and durable responses for several tumor types, however most patients are nonresponsive or develop resistance to such immunotherapies. To identify new predictive biomarkers to better stratify patients, it is essential to comprehensively characterize the immune cells within the TME at the molecular level. Traditional methods to assess gene expression in tissues lack either spatial information or sensitivity/specificity. To address this, we have developed a novel workflow combining the single molecule and single cell visualization capabilities of the RNAscope in situ hybridization (ISH) assay with the highly multiplexed spatial profiling capabilities of the GeoMx™ Digital Spatial Profiler (DSP) RNA assays (Research Use Only).

**Methods**

The fully automated RNAscope Multiplex Fluorescent assay was used to visually identify CD3E (T-cell)-enriched regions and CD19 and CD20 (B-cell)-enriched regions within FFPE human lung cancer tissues. Using the GeoMx DSP, 10 CD3E-enriched regions of interest (ROI) and 10 CD19-enriched ROI were spatially profiled for 78 genes related to immune-oncology research. The RNAscope Multiplex Fluorescence assay was used again to visually confirm the differentially expressed genes between the T and B-cell-enriched regions with single cell resolution.

**Results**

To show a workflow combining RNAscope molecularly guided visualization and GeoMx DSP profiling is feasible, we confirmed that both assay protocols are compatible. We then examined concordance between GeoMx DSP and RNAscope ISH data, demonstrating that RNAscope and GeoMx DSP data can be obtained on the same section. To test the full automated workflow, we compared the differentially expressed genes within the T cell and B cell-enriched ROI. The RNAscope assay confirmed that, while the expression of the immunoregulatory molecules CTLA4, PD-L1, PD-1, and ICOSLG were detected in both ROI, the CD3E (T-cell)-enriched ROI demonstrated significantly higher expression of these checkpoint markers. Compared to the CD19-enriched ROI, the CD3-enriched ROI also showed increased inflammatory signature, demonstrated by elevated levels of cytokines and chemokines such as CCL5, CXCL9 and IFNG.

**Conclusions**

We present a robust workflow that overcomes the historical limitations of ISH and IHC by combining high resolution imaging with high plex profiling. With this workflow, the RNAscope ISH technology can molecularly guide the GeoMx DSP to precisely profile ROI while retaining the morphological context of heterogenous tumors. Furthermore, RNAscope assays can be used to confirm GeoMx DSP-identified gene expression signatures at single cell resolution.

#### P25 A conserved MART-1 T cell receptor motif is predictive of responses to checkpoint blockade

##### Ariel Isser, BS^1^, Tatsuya Yoshida^2^, Junya Ichikawa^2^, Jeffrey Weber, MD, PhD^2^, Jonathan Schneck, MD, PhD^3^

###### ^1^Johns Hopkins University, Baltimore, MD, United States; ^2^New York School of Medicine, New York, NY, United States; ^3^Johns Hopkins School of Medicine, Baltimore, MD, United States

####### **Correspondence:** Jeffrey Weber (jeffrey.weber@nyulangone.org); Jonathan Schneck (jschnec1@jhmi.edu)

**Background**

Since the introduction of checkpoint blockade inhibitors for cancer immunotherapy, numerous studies have sought to identify biomarkers predictive of patient response [1]. However, the relevance of antigen-driven responses to the tumor has yet to be investigated. To address this question, we examined T cell responses to MART-1, an antigen overexpressed in melanoma cells and a target for melanoma clinical trials that have had variable degrees of success. We hypothesized that features of patients’ MART-1 CD8+ T cell repertoires could predict their response to checkpoint blockade.

**Methods**

To understand the MART-1 T cell repertoire, MART-1 CD8+ T cells were expanded from HLA-A2+ melanoma patients and healthy donors using artificial antigen presenting cells (aAPC) or peptide-pulsed dendritic cells. Tetramer positive cells were sorted after 14-22 days and CDR3β sequenced. Motif analysis based on sequence homology was performed using the Immunomap algorithm by clustering 11,252 unique MART-1 CDR3β sequences from 33 samples and 20 donors, including five nivolumab responders and five non-responders [2].

**Results**

No significant difference in the frequency of MART-1 expanded T cells was seen between healthy donors and melanoma patients with or without checkpoint therapy. There was no immunodominant Vβ gene usage and limited clonotype overlap between donors. However, sequence homology showed extensive overlap between donors, driven by two clusters present in 60% and 80% of samples at average frequencies of 10% and 14%, respectively. These clusters were homologous to each other as well as the DMF4 T cell receptor (TCR), one of the first clinically used genetically engineered T cells, with a known crystal structure [3,4]. The core region of these clusters contained a conserved amino acid motif that was identical to contact residues between the DMF4 TCR and MART-1 peptide bound to HLA-A2. The motif identified from the core region of these clusters was highly conserved across samples, present almost exclusively in the junctional region between the D and J genes of the CDR3β, and encoded by a diverse range of nucleotides, all evidence of selective pressure. Despite its conservation, the frequency of this motif was nearly six times lower in pre-therapy samples expanded from non-responders compared to responders (40% vs. 7%, p=0.0045, Figure 1).

**Conclusions**

Since the frequency of the identified MART-1 TCR motif is significantly lower in non-responders compared to responders, it could potentially be used as a biomarker to predict response of HLA-A2+ melanoma patients to checkpoint blockade prior to the onset of therapy.

**References**

1. Zappasodi R, Wolchok JD, Merghoub T. Strategies for Predicting Response to Checkpoint Inhibitors. Curr Hematol Malig Rep. 2018;13(5):383-395.

2. Sidhom J-W, Bessell CA, Havel JJ, Kosmides A, Chan TA, Schneck JP. ImmunoMap: A Bioinformatics Tool for T-Cell Repertoire Analysis. Cancer Immunol Res. January 2017; 6(2):151-162.

3. Rosenberg SA, Packard BS, Aebersold PM, et al. Use of Tumor-Infiltrating Lymphocytes and Interleukin-2 in the Immunotherapy of Patients with Metastatic Melanoma. N Engl J Med. 1988;319(25):1676-1680.

4. Borbulevych OY, Santhanagopolan SM, Hossain M, Baker BM. TCRs used in cancer gene therapy cross-react with MART-1/Melan-A tumor antigens via distinct mechanisms. J Immunol. 2011;187(5):2453-2463.

**Ethics Approval**

The protocol was approved by the NYU Institutional Review Board, i16-01975

**Fig. 1 (abstract P25).**
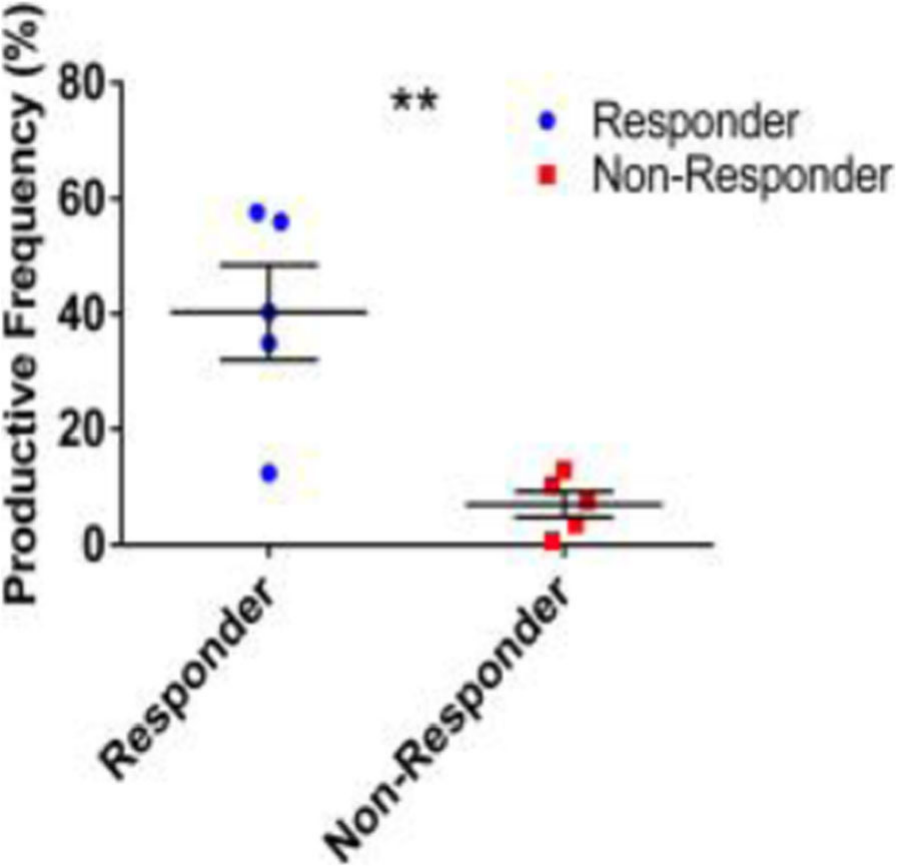
See text for description

#### P26 Murine T cell phenotype and function in a single-well format: a novel, multiplexed and high-throughput assay workflow using the iQue platform

##### Veronica Bruce, PhD, Caroline Weldon, John O'Rourke, Veronica Bruce, PhD

###### Sartorius, Albuquerque, NM, United States

####### **Correspondence:** John O'Rourke (John.ORourke@Sartorius.com)

**Background**

Immunotherapy is an actively growing arena in oncotherapeutics research and development. In this context, whether testing CAR-T cells, checkpoint inhibitors, or novel bispecific antibodies, the ultimate goal is to modulate the immune system to harness its tumor killing power. T cells play a critical role in immune-regulated clearance of both liquid and solid tumors. Upon antigenic stimulation and activation, T cells rapidly expand, secrete cytokines, and differentiate to various functional subsets (e.g. effector T cells, memory T cells). On the other hand, suppression of T cells (i.e. exhaustion) leads to immune escape and the spread of tumor cells. Mouse models remain the most commonly used animal system for in vivo and in vitro cancer biology research and drug discovery. As researchers move forward to either better understand the role of T cells in cancer biology or to develop novel immunotherapies, there is a need for improved methods to quickly gather comprehensive data on T cell biology in this model. To address this, we demonstrate a multiplexed, high-throughput, robust assay workflow capable of measuring multiple murine T cell biology endpoints quickly and reproducibly in a single-well format.

**Methods**

In our workflow, stimulated mouse T cells were assayed in a 96-well plate using fluorescent antibodies against CD3, CD4, CD8, CD69, CD44, CD62L, and PD-1, QBeads for cytokine detection, and markers for cell viability and proliferation. Data were acquired on the iQue3 technology (VBR configuration) and analyzed on a plate-based level using the integrated ForeCyt software.

**Results**

Our assay workflow enabled simultaneous evaluation of viability, interrogation of helper and cytotoxic T cells for markers of activation and exhaustion, and identification of key memory subsets. Proliferation and secreted cytokines (IFN-gamma and IL-2) were also quantified. Data analysis and visualization of multiple endpoints was streamlined and performed in real time using the ForeCyt software.

**Conclusions**

The assay was completed in four hours, including data analysis. This workflow saves the end user’s time and resources by combining multiple experiments into a single, multiplexed workflow, and helps minimize subject-to-subject variability. Altogether, our workflow allows for easy phenotype and functional profiling of murine T cells in a single-well format while generating actionable results in a matter of hours.

#### P27 Functional 3D-plEX quantitative multiplex immunofluorescence platform to assess IO drug impact on tumor microenvironment in ex vivo treated intact 3D-tumor organoids of fresh patient tumor tissue

##### Jenny Kreahling, PhD, Vijayendra Agrawal, PhD, Melba Page, PhD, Mibel Pabon, PhD, Soner Altiok, MD, PhD

###### Nilogen Oncosystems, Tampa, FL, United States

####### **Correspondence:** Soner Altiok (soner@nilogen.com)

**Background**

The tumor stroma consists of various components of the tumor microenvironment including tumor cells, fibroblasts, immune cells and the extracellular matrix. Spatial organization and dynamic interplay of the complex cell-to-cell interactions play an important role in cellular phenotypes that can result in permanent alterations in cellular functions and response to oncology as well as immuno-oncology drug treatments. While informative, conventional 2D tumor dissociated models do not maintain the stromal-stoichiometry of the tumor microenvironment, lacking vital support mechanisms necessary to accurately assess ex-vivo tumor cell viability and immune-cell activation after drug treatment. Here, we describe a functional quantitative multiplex immunofluorescence platform, 3D-plEX, to quantify drug-mediated changes in tumor immune microenvironment and tumor cell viability in intact 3D tumor organoids of patient tumor samples.

**Methods**

All patient tumor samples were obtained with patient consent and relevant IRB approval. Unpropagated live 3D tumoroids measuring 100-150 micron in size were prepared from fresh patient tumors using a proprietary technology, pooled together to represent tumor heterogeneity and equally distributed to different treatment groups including nivolumab, ipilimumab, atezolizumab and urelumab singly or in different combinations. Cell media was collected for multiplex cytokine release assay. Tumoroids were fixed and embedded for multiplex immunofluorescence studies. In addition to tumor cell killing, treatment-mediated changes in TME was analyzed in each treatment group side-by-side using multiplex immunofluorescence markers including CD4, CD8, FoxP3, CD68, Pan-CK, PD-L1 and Ki67.

**Results**

Our results demonstrated that 3D-plEX platform using clinically relevant intact, uniformly sized tumoroids of fresh patient tumor tissue is highly versatile and reliable approach to quantify drug-mediated changes in cellular composition and spatial organization of the tumor immune microenvironment.

**Conclusions**

Combination of this approach with multiplex cytokine release assay allows a comprehensive understanding of dynamic changes within the tumor tissue upon drug treatment. The impact of different immuno-oncology drug treatments ex vivo on TME will be discussed. Application of this platform in the clinical studies may also allow determining the most effective combinatorial therapeutic strategies for individual patients.

#### P28 Mass spectroscopy-based highly multiplexed super-resolution imaging method for fine details of tumor microenvironment monitoring and tumor-immune cell interactions

##### Yunhao Bai, BS^1^, Bokai Zhu^1^, Michael Angelo, MD, PhD^1^, Yongxin Zhao^2^, Sizun Jiang, PhD^1^, Xavier Rovira Clave, PhD^1^, Garry Nolan, PhD^1^

###### ^1^Stanford University, Stanford, CA, United States; ^2^Carnegie Mellon University, Pittsburgh, PA, United States

####### **Correspondence:** Sizun Jiang (sizunj@stanford.edu); Xavier Rovira Clave (xrovira@stanford.edu); Garry Nolan (gnolan@stanford.edu)

**Background**

In tumor microenvironment, tumor-immune interactions are indicated by cell surface proteins such as T cell receptor (TCR) and PD-L1. The key workhorse for studying these cellular interactions is via imaging; conventional imaging methods are limited by the number of channels and the spatial resolving capabilities.

A new modality of imaging, Multiplexed Ion Beam Imaging (MIBI) [1,2], can resolve >40 parameters simultaneously in biological samples. MIBI can current attain single cell resolutions but has difficulties in resolving fine subcellular features. Here, we present Expansion MIBI (ExMIBI), which combines a physical expansion of a biological sample with the MIBI imaging method. ExMIBI will be critical for the scientific community to obtain previously inaccessible insights into the fine details of tumor microenvironment and cancer-immune cell interactions, and promises to unravel fundamental insights in patient immunotherapy responses.

**Methods**

Expansion microscopy (ExM) [3,4] is a technique that can physical expansion of biological specimens 4 to 10 folds through polymer chemistry, three-color fluorescent imaging of cellular features with an apparent lateral resolution of 70 nm in diffraction-limited confocal microscopes has been achieved. However, the expanded gel is fragile and contains up to 99.9% water, which limits its usage in imaging method that requires high vacuum condition. We explored a way to collapse the tissue-containing gel on a complementary charged substrate to achieve a vacuum-compatible gel that can be imaged by the MIBIscope, with lateral resolution <100 nm. Various methods for sample charging removal are systematically tested for imaging a non-conductive gel in MIBI.

**Results**

We have established a robust method, ExMIBI, that allows ExM hydrogels to be compatible with the high vacuum imaging conditions of the MIBI. This method can achieve 40 parameters. A validated panel of MIBI compatible antibodies, focusing on the immune system, is being tested and established for ExMIBI in FFPE tissues (Figure 1).

**Conclusions**

The combination of ExM and MIBI, termed ExMIBI, permits highly multiplexed super resolution imaging of tissue samples. We will now be able to map previously inaccessible, finer details of the tumor microenvironment. The application of ExMIBI to dissect cellular immune interactions, in their spatial biological context, will allow a better understanding into the basic principles of our immune system in healthy and disease states.

**Acknowledgements**

We thank Matt Newgren for tireless technical support on the MIBI instrument. This research has received advice and help from Prof. Michael Angelo, Prof. Sean Bendall and their research group. B.Z. is supported by the Stanford Graduate Student fellowship. S.J is supported by a Stanford Dean’s Fellowship and the Leukemia & Lymphoma Society Career Development Program. X.R.-C. is supported by a long-term EMBO fellowship. This work was supported by grants from the FDA, NIH, Parker Institute for Cancer Immunotherapy, the Bill and Melinda Gates Foundation, as well as the Rachford and Carlota A. Harris Endowed Professorship to G.P.N.

**References**

1. Angelo M, Bendall SC, Finck R, et al. Multiplexed ion beam imaging of human breast tumors. Nature Medicine. 2014;20(4):436–42.

2. Keren L, Bosse M, Marquez D, et al. A Structured Tumor-Immune Microenvironment in Triple Negative Breast Cancer Revealed by Multiplexed Ion Beam Imaging. Cell. 2018;174(6):1373-87.E19.

3. Chen F, Tillberg PW, Boyden ES. Expansion Microscopy, Science. 2015;347(6221):543-8.

4. Tillberg PW, Chen F, Piatkevich KD, et al. Protein-retention expansion microscopy of cells and tissues labeled using standard fluorescent proteins and antibodies. Nature Biotechnology. 2016;34(9):987–92.

**Fig. 1 (abstract P28).**
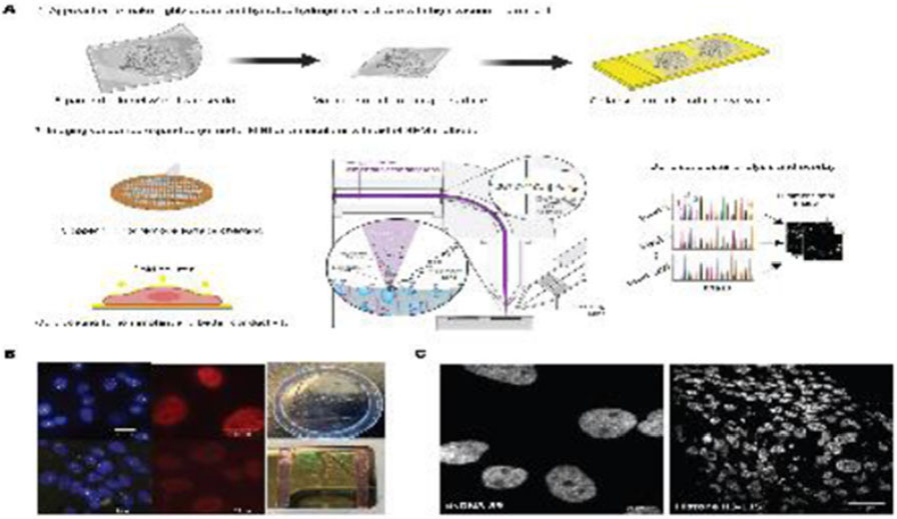
The workflow and sample images of ExM-MIBI

#### P29 Comprehensive image analysis of immunostained NSCLC tissues provides necessary context for immune oncology biomarker profiling

##### Charles Caldwell, PhD, Jenifer Caldara, BS, Will Paces, BS, Kelsey Weigel, PhD, Roberto Gianani, MD

###### Flagship Biosciences, Westminster, CO, United States

####### **Correspondence:** Charles Caldwell (ccaldwell@flagshipbio.com)

**Background**

Manual pathology assessments of Immunohistochemistry (IHC) markers in immune oncology (IO) is often challenging and results can be highly variable[1,2]. Measuring biomarker presence in IO must take in to account both immune and tumor environments and provide contextual information on the interaction between tumor and immune biomarker landscapes [3]. Due to the complex nature surrounding tissue biomarker interpretation in IO, digital image analysis (IA) solutions have been developed that layer complex artificial intelligence (AI) and machine learning algorithms to obtain full tissue biomarker profiles necessary for drug development and patient stratification[4].

Here, a comprehensive tissue analysis solution is presented in monoplex PD-L1 and CD8 stained slides that includes precise digital biomarker scoring in tumor and stromal compartments, recapitulation of common scoring paradigms, analysis of biomarker expression at the tumor/stroma interface (margin), and quantification, scoring, and spatial localization of leukocytes in the tumor and stroma. Aggregation of all cellular and biomarker data generates tissue phenotypes that characterize the IO landscape of each tissue.

**Methods**

Serial sections of 20 NSCLC samples were IHC stained for PD-L1 and CD8 expression. Stained slides were scanned at 20x magnification and analyzed using Flagship Biosciences’ image analysis solutions. Image analysis algorithms which quantify biomarker expression, separate tumor and stromal compartments, detect tumor/stroma margins, and identify leukocytes in immunostained tissues were implemented in each tissue analyzed. Resulting image markups of cell detection and biomarker expression measured by image analysis were reviewed by an MD pathologist for acceptance. Tissues not meeting acceptance criteria were re-analyzed until acceptable to the reviewing pathologist.

**Results**

We demonstrate the synergistic value of layered image analysis algorithms which provide context to biomarker expression in NSCLC tissues. Samples were grouped in to immune desert, excluded, and inflamed phenotypes based on total leukocyte and CD8 expression patterns in the tumor, stroma, and margin. PD-L1 expression was scored based on percentages of tumor and stromal expression, as well as digital representations of common PD-L1 scoring paradigms. Additionally, samples were stratified by PD-L1 patterns of constitutive, induced, immune, or ignorant expression.

**Conclusions**

Digital image analysis of IHC stained tissues creates comprehensive tissue biomarker profiles that are useful in assessment of tumor and immune interactions in IO drug development and patient stratification. Complex algorithms that utilize AI and machine learning can be overseen by MD pathologists to create clinically acceptable digital analysis solutions.

**References**

1. Rimm DL, Han G, Taube JM, et al. A Prospective, Multi-institutional, Pathologist-Based Assessment of 4 Immunohistochemistry Assays for PD-L1 Expression in Non–Small Cell Lung Cancer. JAMA Oncol. 2017;3(8):1051–1058.

2. Hendry S, Salgado R, Gevaert T, et al. Assessing Tumor-infiltrating Lymphocytes in Solid Tumors: A Practical Review for Pathologists and Proposal for a Standardized Method From the International Immunooncology Biomarkers Working Group: Part 1: Assessing the Host Immune Response, TILs in Invasive Breast Carcinoma and Ductal Carcinoma In Situ, Metastatic Tumor Deposits and Areas for Further Research. Adv Anat Pathol. 2017;24(5):235–251.

3. Taube JM, Galon J, Sholl LM, et al. Implications of the tumor immune microenvironment for staging and therapeutics. Mod Pathol. 2018;31(2):214–234.

4. Silva MA, Ryall KA, Wilm C, Caldara J, Grote HJ, et al. PD-L1 immunostaining scoring for non-small cell lung cancer based on immunosurveillance parameters. PLOS ONE 2018; 13(6): e0196464

#### P30 Deep spatial profiling of the immune landscape of MSI and MSS colorectal tumors

##### Sarah Church, PhD, Jason Reeves, Daniel Zollinger, Jill McKay-Fleisch, Andrew White, BSc, Michael Bailey, Arya Bahrami, PhD, Chris Merritt, PhD, Margaret Hoang, Sarah Warren, PhD, Joseph Beechem, PhD

###### NanoString Technologies, Everett, WA, United States

####### **Correspondence:** Sarah Church (schurch@nanostring.com)

**Background**

In colorectal cancer (CRC) there have been many recent advances in immune-related biomarkers that are both prognostic and predictive of response to immunotherapy. Microsatellite instability (MSI)/mismatch repair deficiency (dMMR) is present in ~15-20% of CRCs and corelates with increased immunogenic mutations that often augment lymphocyte infiltration into the tumor microenvironment (TME). Additionally, location of tumor infiltrating T-cells in two areas of the TME, the tumor center (CT) and invasive margin (IM) has also been shown to be prognostic and predictive of response to immunotherapy. Here we use multiplexed protein and RNA digital spatial profiling to elicit the immune landscape of MSI-MSS characterized CRC tumors.

**Methods**

Forty-eight CRC tumors were analyzed for gene expression (GX) using the NanoString® nCounter® PanCancer IO 360™ Research Use Only (RUO) Gene Expression Panel and assessed for 48 cell typing and biological signatures, including MMR loss/MSI predictor and the Tumor Inflammation Signature (TIS). A subset of 18 CRC tumors (6 MSI-TIS-hi, 6 MSS-TIS-hi, 6 MSS-TIS-lo) was selected for analysis with the RUO GeoMx™ Digital Spatial Profiler (DSP) using 40 antibodies, 84 or 1,600+ in situ probes. Selection of regions of interest (ROIs) in two locations, CT and IM were guided by staining with fluorescent markers (CD45, CD3, pan-CK, DNA). 300-600 μM diameter circle ROIs were selected, and in some cases segmented by pan-CK+/pan-CK-.

**Results**

Using whole tissue GX, we first confirmed MSI/dMMR characterization and TIS status of 48 CRC tumors using PanCancer IO 360 signatures. We selected 18 tumors within this cohort based on TIS status to further dissect the location-dependent immune contexture of the TME, with a particular emphasis on differentiating MSI-TIS-hi and MSS-TIS-hi CRCs. DSP confirmed loss of dMMR markers (MSH2/MLH1) and identified an increased amount of potentially suppressive macrophages (CD163+PD-L1+) in MSI-TIS-hi versus MSS-TIS-hi tumors. Segmentation of ROIs based on tumor versus stroma (pan-CK+/-) identified samples with high proportions of tumor-invading TILs. These samples were then further profiled using probes against 1600+ mRNA targets revealing distinct pathways related immune cell orientation within the TME.

**Conclusions**

Here we show the use of novel high-plex spatial profiling to profile location and pathways in the TME of MSI and MSS CRC tumors. These findings elicit unique biology related to the location and signaling of immune cells, which have the potential to unveil targets for therapeutic combinations.

#### P31 Applying multispectral unmixing and spatial analyses to explore tumor heterogeneity with a pre-optimized 7-color immuno-oncology workflow

##### Carla Coltharp, PhD, Bethany Remeniuk, PhD, Chichung Wang, Rachel Schaefer, Linying Liu, Glenn Milton, Victoria Duckworth, Michael McLane, Peter Miller, Yi Zheng, Carla Coltharp, PhD

###### Akoya Biosciences, Hopkinton, MA, United States

####### **Correspondence:** Yi Zheng (YZheng@akoyabio.com)

**Background**

The tumor microenvironment hosts a myriad of cellular interactions that influence tumor biology and patient outcomes. Multiplex immunofluorescence (mIF) provides the ability to investigate a large number of these interactions in a single tissue section, and has been shown to outperform other testing modalities for predicting response to immunotherapies [1].

Multispectral imaging (MSI) improves the capabilities of mIF by providing the ability to spectrally unmix fluorescence signals. This increases the number of markers that can be probed in the same scan and allows for separation of true immunofluorescence signals from tissue autofluorescence background.

Here, we apply MSI to explore spatial interactions observed in lung cancer samples using an end-to-end translational workflow based on the PhenopticsTM platform. The workflow includes a pre-optimized 7-color staining panel kit along with a pre-configured analysis algorithm for cell phenotyping.

Using tissue microarrays (TMA), we demonstrate the heterogeneity of spatial interactions observed among different lung cancer samples and the improved sensitivity of detection afforded by unmixing multispectral scans.

**Methods**

A lung cancer TMA was created using the 3DHistech TMA Master II from five formalin-fixed paraffin-embedded lung cancer tissue blocks. The TMAs were stained using the Opal Polaris 7-Color PD1/PD-L1 Lung Cancer Panel Kit on the Leica BOND RXTM automated stainer using the associated preloaded Opal 7-Color Panel Kit protocol. Whole slide MOTiFTM multispectral scans were acquired on Vectra Polaris® with pre-defined acquisition parameters. Scans were unmixed and analyzed with inForm® software using a pre-configured algorithm tailored to the PD1/PD-L1 Lung Cancer Panel Kit. Spatial analyses and visualizations were performed using the phenoptr and phenoptrReports R-based packages and custom scripts.

**Results**

The pre-optimized Opal Polaris 7-Color PD1/PD-L1 Lung Cancer Panel Kit was able to visualize the panel targets (PD-L1, PD-1, CD8, CD68, FoxP3, and Cytokeratin) across the variety of lung cancer samples in the TMA. Cell phenotyping and spatial analyses revealed core-to-core variations in cell densities and proximities among different markers. Measurement of the dynamic range of PD-L1 expression across different cores also revealed the improved sensitivity in PD-L1 detection provided by unmixing.

**Conclusions**

The end-to-end Phenoptics staining, imaging, unmixing, and spatial analysis workflow described here provides a robust and sensitive platform for exploring the immune landscape within the tumor microenvironment.

**Reference**

1. Lu S, Stein JE, Rimm DL, et al. Comparison of Biomarker Modalities for Predicting Response to PD-1/PD-L1 Checkpoint Blockade: A Systematic Review and Meta-analysis. JAMA Oncol. Published online July 18, 2019. doi:10.1001/jamaoncol.2019.1549

#### P32 Differential immune contexture of human colorectal carcinomas with mismatch repair deficiency (MSI-H) and increased DNA damage responses (DDR)

##### Shruti Desai, PhD^2^, Venkata Nagineni^2^, Micaela Morgada^2^, Aravind Kalathil^2^, Ila Datar^2^, Charles Fuchs, MD, MPH^1^, Patricia LoRusso, DO^2^, Ranjit Bindra^2^, Kurt Schalper, MD, PhD^2^

###### ^1^Yale University, New Haven, CT, United States; ^2^Yale University, School of Medicine, New Haven, CT, United States

####### **Correspondence:** Kurt Schalper (kurt.schalper@yale.edu)

**Background**

Tumor cells accumulate deleterious genomic alterations through sustained mutagenic exposure and defective DNA repair. Approximately 15% of human colorectal carcinomas (CRCs) display mismatch repair deficiency (MSI-H) associated with increased somatic mutations and sensitivity to immune checkpoint blockers. Advanced tumors can harbor additional DNA-repair alterations with functional/therapeutic implications. Increased double strand DNA breaks have been reported across solid tumors and can be detected by changes in Serine139-phosphorylated histone H2AX (γH2AX). We studied the immune composition of human CRCs with MSI-H and elevated DDR.

**Methods**

Using multiplexed quantitative immunofluorescence (QIF), we studied the level of major adaptive and innate immune markers in a retrospective collection of 265 stage I-IV CRCs from Yale represented in tissuemicroarrays. We used previously validated QIF panels including the markers DAPI, cytokeratin, γH2AX, CD3, CD4, CD8, CD20, PD-L1, CD15, myeloperoxidase (MPO), IL-8, Ki-67, granzyme-B (GZB), Beta-2 microglobulin (B2M), HLA-class I and HLA-class II. The MSI status was determined using clinical-grade immunohistochemistry detection of MLH1, MSH2, MSH6 and PMS2. We analyzed the association between localized measurement of markers and with major clinicopathologic variables/survival.

**Results**

From 252 evaluable cases, 12.1% were classified as MSI-H. Relative to MSS tumors, MSI-H CRCs showed significantly higher levels of PD-L1, lower CD20 and non-significant increases in CD3, CD4, CD8, T-cell Ki-67 and T-cell GZB. MSI-H cases displayed lower tumor-cell B2M and increased stromal HLA-class II expression. MSI-H tumors also showed significantly higher levels of IL-8 and MPO+ cells than MSS counterpart. The level of γH2AX was comparable between MSI-H and MSS malignancies. Cases with increased tumor-cell γH2AX (> cohort median) showed significantly higher levels of PD-L1 and all studied lymphocyte markers than cases with lower γH2AX. In addition, these tumors displayed significantly higher T-cell proliferation, mild increases in T-cell GZB and higher levels of HLA-class I/class II proteins. The levels of IL-8 and MPO+ cells were comparable across the γH2AX groups. The DNA repair and immune markers were variably associated with 5-year overall survival in the cohort.

**Conclusions**

DNA repair deficiency defines human CRCs with distinct innate and adaptive immune contexture. While mismatch repair deficiency is associated with mild/moderate intratumor T-cell responses and prominent myeloid cell features; elevated DDR display prominent adaptive immunity and unaltered myeloid-cell changes. Our data indicate that MSI-H and DDR phenotypes are independent features in human CRC and this could be used to design optimal therapeutic strategies.

**Ethics Approval**

All tissues were used after approval from the Yale Human Investigation committee protocol #9505008219 which approved the patient consent forms or waiver of consent.

#### P33 DNA damage response (DDR) is associated with increased adaptive anti-tumor responses and PD-L1 expression in human non-small cell lung cancer

##### Shruti Desai, PhD^2^, Aravind Kalathil^2^, Roy Herbst, MD, PhD^1^, Ranjit Bindra^2^, Patricia LoRusso, DO^1^, Kurt Schalper, MD, PhD^2^

###### ^1^Yale University, New Haven, CT, United States; ^2^Yale University, School of Medicine, New Haven, CT, United States

####### **Correspondence:** Kurt Schalper (kurt.schalper@yale.edu)

**Background**

Tumor cells accumulate genomic alterations as a consequence of sustained mutagenic events and defective DNA repair mechanisms, collectively called DNA damage response (DDR). Targeting DDR pathways can induce synthetic lethality and prominent anti-tumor responses in neoplasms with DNA repair deficiency. In addition, increased DNA damage could favor anti-tumor immune responses by increasing the neo-antigenic load and T-cell recognition. Despite its therapeutic implications, the frequency and significance of DDR alterations in human non-small cell lung cancer (NSCLC) remains poorly understood.

**Methods**

Using irradiated cell line preparations and expression controls, we standardized a multiplexed quantitative immunofluorescence (mQIF) panel for simultaneous and localized measurement of DAPI (all cells), cytokeratin for tumor epithelial cells (AE1/AE3, DAKO), γH2AX to map active DNA damage/repair responses (JBW301, Millipore), CD3 for T-lymphocytes (Rabbit polyclonal, DAKO) and PD-L1 (E1L3N, CST) in formalin-fixed paraffin-embedded (FFPE) tissue samples. We used this panel to interrogate 4 retrospective NSCLC cohorts from Yale represented in tissue microarray format including immunotherapy-naïve cases (Cohort#1: n=297 and #2:n=175); lung adenocarcinomas tested for major oncogenic mutations (Cohort #3, n=139); and baseline NSCLC samples from patients treated with immune checkpoint blockers (Cohort #4, n=84). We analyzed the levels of the markers in different tumor tissue compartments and their association with major clinicopathological variables.

**Results**

Detectable nuclear tumor-cell γH2Ax was recognized in 37-58% of NSCLCs. Elevated tumor-cell γH2Ax expression was consistently associated with smoking history, increased intratumor CD3+ T-cells and PD-L1 protein expression across the cohorts. The level of γH2Ax was significantly lower in KRAS mutant lung adenocarcinomas than in EGFR mutant or EGFR/KRAS wild type tumors. No additional clinicopathologic associations were found. γH2Ax was not prognostic as single marker. However, elevated simultaneous expression of γH2Ax and CD3 was associated with longer 5-year overall survival in the immunotherapy-naïve cohorts. In patients treated with PD-1 axis blockers, elevated baseline γH2Ax/CD3 was associated with a clear trend toward longer survival but did not reach statistical significance.

**Conclusions**

Active DDR as measured by tumor-cell γH2Ax expression occurs in a high proportion of human NSCLCs and is associated with T-cell inflamed tumors. Despite their association with smoking, lung adenocarcinomas harboring activating mutations in KRAS display lower DDR markers than EGFR mutant or EGFR/KRAS wild type malignancies. Collectively, our results support the use of combination therapy targeting DDR and immunostimulatory therapies in a fraction of NSCLC.

**Ethics Approval**

All tissues were used after approval from the Yale Human Investigation committee protocol #9505008219 which approved the patient consent forms or waiver of consent.

#### P34 A fully optimized end-to-end solution for I/O multiplex immunofluorescence staining using Opal Polaris 7-Color PD1/PD-L1 Panel Kits for lung cancer and melanoma

##### Yi Zheng, Rachel Schaefer, Linying Liu, Glenn Milton, Carla Coltharp, PhD, Victoria Duckworth, MS, Michael McLane, Peter Miller, MS

###### Akoya Biosciences, Hopkinton, MA, United States

####### **Correspondence:** Peter Miller (pmiller@akoyabio.com)

**Background**

Understanding cellular heterogeneity and spatial relationships between biomarkers within the tumor microenvironment (TME) is a key component to translational research in immuno-oncology. Multiplex immunofluorescence (mIF) on formalin-fixed, paraffin-embedded (FFPE) tissue is the multiparameter assay most frequently chosen across all current I/O clinical trials, as it allows for quantitative assessment of these relationships in situ. Running medium to large scale translational studies on FFPE tissue demands an assay that is reproducible, quantitative, easy-to-use, and standardized, yet still allows for flexibility when detecting differentially expressing biomarkers across samples. In this study, we demonstrate a fully developed, flexible, end-to-end workflow solution for tissue biomarker discovery by applying miF in lung cancer and melanoma. This newly developed Phenoptics™ solution provides an integrated MOTiF™ workflow including primary antibodies and image analysis algorithms enabling a more comprehensive and specific TME analysis with minimal user optimization.

**Methods**

FFPE samples from human lung cancer and melanoma were stained using Opal Polaris 7-Color PD1/PD-L1 Lung Cancer and Melanoma Panel Kits. Staining was performed on the Leica BOND RX™ automated stainer with the pre-loaded MOTiF protocol. Multispectral scans were acquired on Vectra Polaris® with pre-optimized acquisition parameters and analyzed with a pre-configured phenotyping algorithm in inForm®. Spatial analyses and visualizations were performed in R using phenoptr and phenoptrReports.

**Results**

This simplified end-to-end solution results in better quantification of cancer-immune interactions by providing:

- Well-optimized Opal Polaris 7-Color PD1/PD-L1 Lung Cancer and Melanoma Panel Kits. Along with the pre-loaded Leica BOND RX automation protocol, we provide a staining workflow with pre-defined primary antibody concentration, fixed staining order, and Opal™ dye-antibody pairs, leaving Opal concentrations as a flexible dial. Recommended image acquisition parameters on the Vectra Polaris® that significantly simplify visualization of multiple markers via multispectral isolation. Pre-configured image analysis algorithms that make quantitative analysis at a per-cell and per-slide level streamlined and standardized.

**Conclusions**

The 7-Color PD1/PD-L1 panel kits utilizing MOTiF whole slide scanning enable visualization of multiple biomarkers at the whole slide level, revealing distribution patterns and their spatial context across the entire tissue section. With these new assays, we have demonstrated an easy-to-use yet comprehensive end-to-end Phenoptics research workflow. We have radically simplified the Opal method and facilitated the development and optimization of translational multiplex fluorescent assays by providing pre-defined staining conditions while still giving researchers the flexibility to balance signals based on their tissue samples. Complementary pre-configured phenotyping provides researchers faster access to quantitative data across study samples.

#### P35 Pick-Seq®: a spatial analysis tool for immuno-oncology biomarker discovery utilizing multi-parameter imaging and RNA sequencing of tissue micro-regions

##### Nolan Ericson^1^, Rebecca Podyminogin^1^, Jennifer Chow, PhD^1^, Yu-An Chen^2^, Jia-Ren Lin^2^, Zoltan Maliga^2^, Peter Sorger^2^, Kyla Teplitz^1^, Melinda Duplessis, PhD^1^, Eric Kaldjian, MD^1^, Tad George, PhD^1^

###### ^1^RareCyte, Seattle, WA, United States; ^2^Harvard Medical School, Boston, MA, United States

####### **Correspondence:** Tad George (tgeorge@rarecyte.com)

**Background**

Pick-Seq is a novel workflow uniquely enabled by the RareCyte CyteFinder® Instrument that combines visualization of multiple protein markers with investigation of gene expression from selected micro-regions on tissue slides, providing spatial and contextual investigation of tumors and their microenvironment.

**Methods**

Frozen breast carcinoma and formalin-fixed, paraffin-embedded tonsil sections were stained by multi-parameter immunofluorescence (IF) for markers of T cells, B cells, and cytokeratin. Slides were imaged with the CyteFinder® Instrument and 40 μm micro-regions were retrieved with the integrated CytePicker® Retrieval Module. RNA was isolated and whole transcriptome amplified (SMART-seq v4), followed by Nextera XT library preparation, sequencing on Illumina MiSeq, and gene expression analysis. Differentially expressed genes were selected to create a Pick-Seq-informed IF staining panel to confirm RNA expression results. Cell compositions of each micro-region were deconvolved with CIBERSORT.

**Results**

Tonsil micro-regions from one T cell zone and two adjacent follicles were retrieved for RNA sequencing. Transcriptomic analysis confirmed increased expression of B cell markers in follicles and T cell markers in the T cell zone. CIBERSORT analysis revealed distinct cellular compositions between T cell zones and the B cell follicles. Principle component analysis of gene expression found that micro-regions retrieved from the two follicles clustered independently from each other, and from the T cell zone micro-regions. Differential expression analysis between the adjacent follicles revealed distinct patterns of CD21 expression, a marker which was not present in the original IF staining panel. Subsequent staining confirmed differential protein expression of CD21, indicating that only one follicle contained a germinal center. In breast carcinoma, ROI were identified for micro-region retrieval that included tumor cells, tumor cells with interspersed tumor infiltrating lymphocytes (TIL), or adjacent lymphoid aggregates. Micro-regions were picked and sequenced. Hierarchical clustering and differential expression analysis differentiated the three micro-region types and revealed tumor- and T cell-specific expression signatures. CIBERSORT demonstrated the presence of T cell-associated transcriptomic profiles in lymphoid aggregates and in TIL-containing micro-regions that were proportional to the number of T cells retrieved. Aligned RNA-seq reads were further analyzed via TraCeR to identify TCR α and β chain sequences from retrieved TILs.

**Conclusions**

These data establish the potential of combining multi-parameter IF microscopy with highly focused RNA sequencing as a powerful tool for investigation and biomarker discovery for immuno-oncology.

#### P36 The complexity of myeloid-derived suppressor cells in non-small cell lung cancer: A combinatorial multiplex IHC and flow cytometry approach

##### Amanda Finan, PhD^1^, Muriel Smet^2^, Maroua Tliba^1^, Manon Motte^1^, Jean-Philippe Coton^1^, Domenico Lazzaro^1^, Renaud Burrer^1^

###### ^1^Histalim, Montpellier, France; ^2^Barc Lab, Ghent, Belgium

####### **Correspondence:** Renaud Burrer (rburrer@histalim.com)

**Background**

Lung cancer is the most common cause of cancer-related deaths worldwide with non-small cell lung cancer (NSCLC) representing the gross majority of the cases. The immune microenvironment of NSCLC is diverse with many players that can impact tumor development and clinical outcomes. In particular, myeloid-derived suppressor cells (MDSC) are important components of the immunosuppressive network that can hinder the activity of T cells, natural killer cells, and dendritic cells. MDSC in the blood may represent prognostic markers for NSCLC patients and for monitoring a patient’s response to immunotherapies. There is a gap in the relevance of MDSC within the tissue context due to limitations with conventional immunohistochemistry. Multiplex immunofluorescence offers a technical advantage by allowing the detection of co-expression and spatial organization of multiple targets within a preserved tissue architecture on a single slide.

**Methods**

We have developed the multiplex immunofluorescence Histoprofile-MDSC panel to identify monocytic MDSC (M-MDSC) and polymorphonuclear MDSC (PMN-MDSC) in situ. Five human NSCLC tissue samples were investigated by multiplex immunofluorescence and H&E staining. After multispectral acquisition, the MDSC populations were evaluated with the imaging software HALO. Paired peripheral blood was analyzed for circulating M-MDSC by flow cytometry.

**Results**

The development and verification of the multiplex panel are presented. The NSCLC subtype of the samples was determined by a pathologist from the H&E sections. Monocytes, neutrophils, M-MDSC, and PMN-MDSC were evaluated in the five tissue samples. The neutrophils, monocytes, and M-MDSC in the peripheral blood could be assessed by flow cytometry. A varying distribution of the cell populations in the lung tissue and the peripheral blood of the different NSCLC subtypes can be appreciated. The two approaches are compared.

**Conclusions**

We present an in-depth combined approach for MDSC investigation in lung tissue and the peripheral blood of NSCLC patients. The approaches presented here demonstrate the power of multiplex immunohistochemistry and flow cytometry in the identification and quantification of multiple immune cell populations with a limited quantity of patient sample and the potential application of this method in both preclinical and clinical studies.

#### P37 ImmunoPET imaging of glioma-infiltrating myeloid cells using Zirconium-89-labeled anti-CD11b antibody

##### Alexandra Foster, BS, Rajeev Kumar, Shubhanchi Nigam, Lauren McCarl, Robert Edinger, Ian Pollack, Carolyn Anderson, Wilson Edwards, Gary Kohanbash, Alexandra Foster, BS

###### University of Pittsburgh, Pittsburgh, PA, United States

####### **Correspondence:** Gary Kohanbash (gary.kohanbash2@chp.edu)

**Background**

Gliomas are the most common primary central nervous system tumor, with malignant gliomas causing significant morbidity and mortality. Thirty percent of a glioma’s cellular mass may be attributed to immunosuppressive and pro-tumoral tumor-associated myeloid cells (TAMCs), primarily myeloid-derived suppressor cells (MDSCs) and tumor-associated macrophages (TAMs) [1-4]. Multiple preclinical studies and clinical trials have attempted to target these cells; however, monitoring responses to these therapies remains a challenge. Quantifying TAMCs within gliomas using an antibody-based tracer for non-invasive positron emission tomography (immunoPET) may allow for better patient stratification, monitoring of treatment efficacy, and ultimately improve survival rates [5-9]. Integrin CD11b is a cellular marker expressed on the surface of TAMCs frequently used to identify macrophages and microglia. We therefore hypothesized that radiolabeled anti-CD11b antibody (Ab) could be used for immunoPET imaging of TAMCs in a preclinical orthotopic syngeneic glioma model.

**Methods**

The human/mouse cross-reactive anti-CD11b Ab (clone M1/70) was conjugated with p-NCS-Bz-DFO chelator and radiolabeled with 89Zr for PET imaging with specific activity of 2 μCi/μg. PET/CT imaging, with or without a blocking dose of anti-CD11b Ab, was performed in mice bearing established orthotopic syngeneic GL261 gliomas. Flow cytometry and histology in tissues collected from post-imaging biodistribution validated targeting of CD11b+ TAMCs.

**Results**

Standard uptake values (SUV) indicated significant 89Zr-anti-CD11b Ab uptake in the tumor ipsilateral right brain (SUVmean = 2.6 ± 0.24) compared to contralateral left brain (SUVmean = 0.6 ± 0.11). Blocking with 10-fold lower specific activity 89Zr-anti-CD11b Ab reduced the SUV in right brain with (SUVmean = 0.11 ± 0.06). Spleen and lymph nodes also showed high uptake, while bone and muscle showed low uptake. Biodistribution analysis confirmed these results. Additionally, no uptake was observed in the brain of non-tumor bearing mice that received 89Zr-ant- CD11b. Flow cytometry with QuantiBRITE Fluorescence Quantitation Kit demonstrated that the majority of tumor-infiltrating immune cells expressed CD11b at an average of 54,076 CD11b molecules per cell in GL261.

**Conclusions**

Imaging TAMCs with 89Zr-labeled anti-CD11b Ab may be feasibility for preclinical studies, patient stratification, and monitoring of immunotherapy.

**References**

1. Gabrusiewicz K, Rodriguez B, Wei J, et al. Glioblastoma-infiltrated innate immune cells resemble M0 macrophage phenotype. JCI insight. 2016; 1(2).

2. Kennedy BC, Showers CR, Anderson DE, et al. Tumor-associated macrophages in glioma: friend or foe? J oncol; 2013. 2013.

3. Kohanbash G, Okada H. Myeloid-derived suppressor cells (MDSCs) in gliomas and glioma-development. Immunolo invest. 2012; 41:658–679.

4. Lapa C, Linsenmann T, Lückerath K, et al. Tumor-associated macrophages in glioblastoma multiforme-a suitable target for somatostatin receptor-based imaging and therapy? PloS one. 2015; 10.

5. Kohanbash G, McKaveney K, Sakaki M, et al. GM-CSF Promotes the Immunosuppressive Activity of Glioma-Infiltrating Myeloid Cells through Interleukin-4 Receptor-α. Cancer Res. 2013; 73:6413–6423.

6. Raychaudhuri B, Rayman P, Huang P, et al. Myeloid derived suppressor cell infiltration of murine and human gliomas is associated with reduction of tumor infiltrating lymphocytes. J Neurooncol. 2015; 122:293–301.

7. Okada H, Kohanbash G, Zhu X, et al. Immunotherapeutic approaches for glioma. Crit rev immunol. 2009; 29:1–42.

8. Otvos B, Silver DJ, Mulkearns-Hubert EE, et al. Cancer Stem Cell-Secreted Macrophage Migration Inhibitory Factor Stimulates Myeloid Derived Suppressor Cell Function and Facilitates Glioblastoma Immune Evasion. Stem Cells. 2016; 34:2026–2039.

9. Meyer C, Cagnon L, Costa-Nunes CM, et al. Frequencies of circulating MDSC correlate with clinical outcome of melanoma patients treated with ipilimumab. Cancer Immunol Immunother. 2014; 63:247–257.

**Ethics Approval**

The study was approved by University of Pittsburgh's Institutional Animal Care and Use Committee (IACUC).

#### P38 Sensitive methodologies for tracking T cell immunotherapy by MRI

##### Brooke Helfer, PhD^1^, Deanne Lister^2^, Charles O'Hanlon III^1^, Eric Ahrens^2^, Brooke Helfer, PhD^1^

###### ^1^Celsense, Inc, Pittsburgh, PA, United States; ^2^UCSD, La Jolla, CA, United States

####### **Correspondence:** Brooke Helfer (brooke@celsense.com)

**Background**

Cancer immunotherapies have made a great progress and hold much promise in the treatment of cancer. Specifically, in the case of B-cell malignancies (such as Acute Lymphoblastic Leukemia, or ALL), CAR (chimeric antigen receptor) and TCR (T-cell receptor) therapies have demonstrated encouraging clinical results. As we begin to target solid tumors with TCR and CAR T-cells, the hurdle of being able to select a suitable target and achieve successful cellular delivery/homing to the site of disease remains. With this in mind, being able to visualize a rapidly dividing cellular population is another obstacle to consider.

**Methods**

Here we demonstrate the application of two clinically applicable perfluorocarbon (PFC) tracers, one commercially available and a next-generation magnetic resonance imaging (MRI) probe called FETRIS. Both of these agents enable the migration and persistence of cellular therapies to be noninvasively imaged by 19F MRI, while the FETRIS reagent adds additional detection sensitivity.

**Results**

Using a general T-cell expansion protocol, we show that adding a cellular label does not alter the viability or release characteristics of T cells. By pairing the PFC signal with conventional proton MRI from the same imaging session, the images are able to be overlaid, allowing cells to be traced to their anatomical location. With nominal exogenous fluorine naturally present in tissue, labeled cells appear with little background.

**Conclusions**

Images of both reagents show the detection and sensitivity of the method and how they can be applied to monitor the distribution of cells over time.

#### P39 Looking beyond the assay: Comparison of multiplex chromogenic and fluorescent immunohistochemistry for standardized immune oncology profiling in non-small cell lung carcinoma patients

##### Ana Hidalgo Sastre, PhD^1^, Lorenz Rognoni, PhD^2^, Monika Baehner^2^, Marco Testori^2^, Jessica Chan^2^, Andreas Spitzmüller^2^, Nicolas Brieu, PhD^2^, Bonnie Phillips, PhD^3^, Katir Patel, PhD^3^, Sean Downing, PhD^3^, Alex Haragan^4^, John Field^4^, Florian Leiss, PhD^2^

###### ^1^Definiens, Munich, Germany; ^2^Definiens AG, Munich, Germany; ^3^Ultivue, Cambridge, MA, United States; ^4^Liverpool University Hospital, Liverpool, United Kingdom

####### **Correspondence:** Ana Hidalgo Sastre (ahidalgo@definiens.com)

**Background**

Given the heterogeneity of tumors and the variety of potential biomarkers in immune oncology, there is a need for quantitative standardized assays to reliably assess the immune status of a patient’s tumor to be able to extract the true biological information across cohorts. Here, two different tissue-based approaches have been compared: multiplex immunofluorescence (mIF) and multiplex chromogenic immunohistochemistry (mIHC). Independently of the technique used, assay reproducibility and standardized quantification of staining intensity are a prerequisite for obtaining consistent results. Using a cohort of non-small cell lung carcinoma (NSCLC) patients, we identified patterns of immune cell infiltration that were comparable, independent of the assay applied.

**Methods**

Formalin-fixed paraffin-embedded (FFPE) true consecutive slides from 7 NSCLC resections were stained with a multiplex chromogenic panel (including CD3, PD-L1, CD68, CD8, PD-1) at Mosaic Laboratories [1] and with the UltiMapper kits (I/O PD-L1 and I/O PD-1) from Ultivue. mIHC scans were acquired with an Aperio AT Turbo scanner (Leica), while mIF scans were acquired with a Zeiss Axio Scan.Z1 scanner (Zeiss) both as whole slide images. mIHC and mIF images were co-registered, and Definiens custom algorithms for digital image analysis were applied [2,3].

**Results**

Densities of immune cell populations and their locations in different compartments (invasive margin vs tumor center and tumor epithelium vs tumor stroma) were measured (Figure 1). For instance, CD3 cell density had a Pearson correlation of 0,91 and a Spearman correlation of 0,89 between both assays (mIHC vs mIF). Differentiation between tumor epithelium and tumor stroma was based on a histology-driven deep learning approach for mIHC and on pan Cytokeratin for mIF (Figure 1).

**Conclusions**

By applying mIHC and mIF in true consecutive tissue slides we retrieved the information of tumor immune cell infiltrates that was consistent across the different assays and distinguished it from information that is specific to either of the assays. We believe that being able to relate across staining techniques could help pathologists and research centers draw conclusions across cohorts that were stained with the same markers but with different assays.

**References**

1. Lisa M. Dauffenbach, Christopher A. Kerfoot, et al. Characterization of inflammatory cell patterns and densities using multiplex immunohistochemistry immuno-oncology assays [abstract]. In: Proceedings of the AACR-NCI-EORTC International Conference: Molecular Targets and Cancer Therapeutics; 2017 Oct 26-30; Philadelphia, PA. Philadelphia (PA): AACR; Mol Cancer Ther 2018;17(1 Suppl): Abstract nr B069.

2. Lorenz Rognoni, PhD; Vinay Pawar, PhD; Tze Heng Tanet, et al. Automated quantification of whole-slide multispectral immunofluorescence images to identify spatial expression patterns in the lung cancer microenvironment. SITC Annual Meeting; 2018 Nov 7-11; Washington, DC. Poster nr P442.

3. Brieu, Nicolas & Meier, Armin & Kapil, Ansh & Schönmeyer, Ralf & Gavriel, Christos & Caie, Peter & Schmidt, Günter. (2019). Domain Adaptation-based Augmentation for Weakly Supervised Nuclei Detection.

**Ethics Approval**

Ethical approval was granted by the Liverpool Research Ethics Committee, reference number 97/141.

**Fig. 1 (abstract P39).**
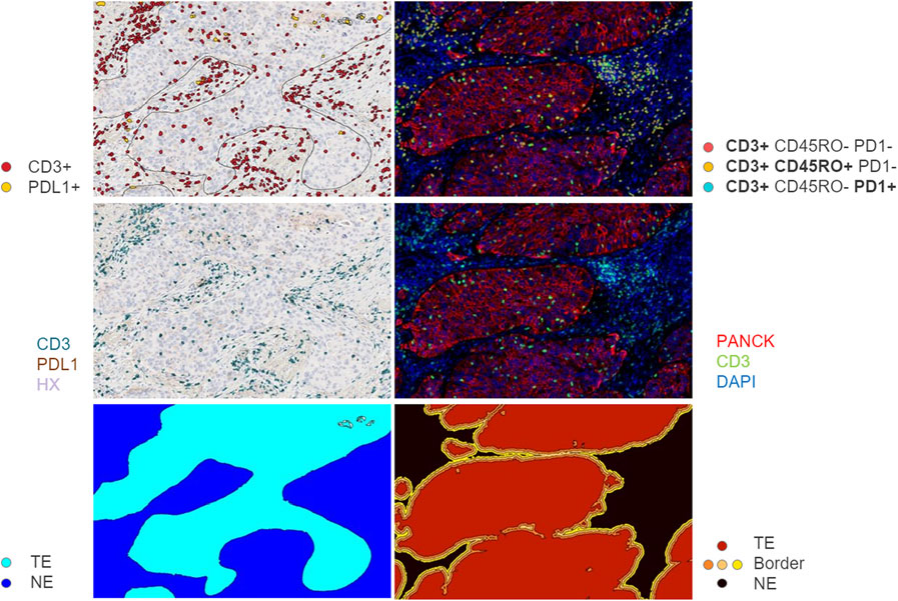
Consecutive slides from NSCLC resection

#### P40 Tumour immunity signatures to expand current diagnostic approaches in mismatch repair deficient cancers in the context of Lynch Syndrome through InSituPlex technology and Tissue Phenomics integration

##### Ryan Hutchinson, Fellow^1^, Armin Meier, PhD^2^, Bonnie Philips^3^, Katir Patel, PhD^3^, Sean Downing, PhD^3^, Karan Sharma^3^, Julia Como^1^, Simin Daneshvar^1^, Gillian Livock^2^, Ingrid Winship^4^, Christophe Rosty^1^, Mark Jenkins^5^, Gunter Schmidt^2^, Daniel Buchanan^1^, Ryan Hutchinson, Fellow^1^

###### ^1^Victorian Comprehensive Cancer Centre, Melbourne, Australia; ^2^Definiens AG, Munich, Germany; ^3^Ultivue, Cambridge, MA, USA, Boston, MA, United States; ^4^Royal Melbourne Hospital, Melbourne, Australia; ^5^The University of Melbourne, Melbourne, Australia

####### **Correspondence:** Daniel Buchanan (daniel.buchanan@unimelb.edu.au)

**Background**

Deficiency in the mismatch repair (dMMR) can result from inherited mechanisms (Lynch Syndrome (LS)) or from somatic inactivation caused by hypermethylation of the MLH1 gene promoter (MLH1 methylated). A third subtype of dMMR colorectal cancer (CRC) and endometrial cancer (EC) have neither LS nor MLH1 promoter methylation and are referred to as suspected Lynch syndrome (SLS). There remains a knowledge gap as to whether the tumour microenvironment (TiME) is different between LS, MLH1-methylated and SLS dMMR CRC and EC. The aim of this study was to characterise and identify immune patterns within the TiME that may enhance the current clinical triaging of LS, SLS and MLH1-methylated subtypes of dMMR CRC and EC.

**Methods**

Ten FFPE samples from seven individuals were studied: CRC (N=5; 1xLS, 2xMLH1 methylated, 1xSLS and 1x proficient MMR (pMMR)) and EC (N=2; 1xLS and 1xpMMR) and where available adjacent normal tissue (N=5: 3xcolon and 2xendometrium) were characterized using the Ultivue UltiMapper I/O portfolio (InSituPlex) on a Leica Bond autostainer and digitally acquired using the Zeiss Axio Scan Z1. We evaluated CD3, CD8, CD11c, CD20, CD45RO, CD68, CD163, Granzyme B, Ki-67, MHC II, PD-1, PD-L1, and pan-cytokeratin. Tissue phenomic approaches were developed to spatially characterize, quantify immune cell patterns and visualize heterogeneity within the TiME (Figure 1).

**Results**

InSituPlex technology enabled the visualization of the heterogenous infiltration and co-localization patterns across LS dMMR CRC (Figures 2&3). Tissue phenomic approaches demonstrated the following; within the SLS category the colon cancer had higher mean areas of intraepithelial (IE) PD-L1 (6% vs. 2%), CD8 (18% vs. 8%) and CD68 (28% vs. 12%) compared to the pMMR EC. The MLH1 methylated tumour with a high tumour mutation burden (33.84 mutations/MB) had a higher mean area of IE PD-L1 (8% vs. 2%) and CD8 (30% vs. 5%) while the tumour with low TMB had a higher mean area of IE CD68 (15% vs. 8%). Within LS, the EC had a higher mean area of IE PD-L1 compared to the CRC (14% vs. 0%), in contrast the CRC had a higher IE CD8 area (27% vs. 10%) (Figures 4&5).

**Conclusions**

This study evaluated the immune contexture within inherited and sporadic subtypes of dMMR CRCs and ECs, highlighting differing immune infiltration patterns and phenomic densities. Integration of multiplex technologies and Tissue Phenomics can enhance the understanding of the dMMR TiME and with potential utility in clinical triaging and to inform immune-oncology clinical trials.

**Acknowledgements**

We thank the investigators and participants of the ACCFR and ANGELS studies.

**Fig. 1 (abstract P40).**
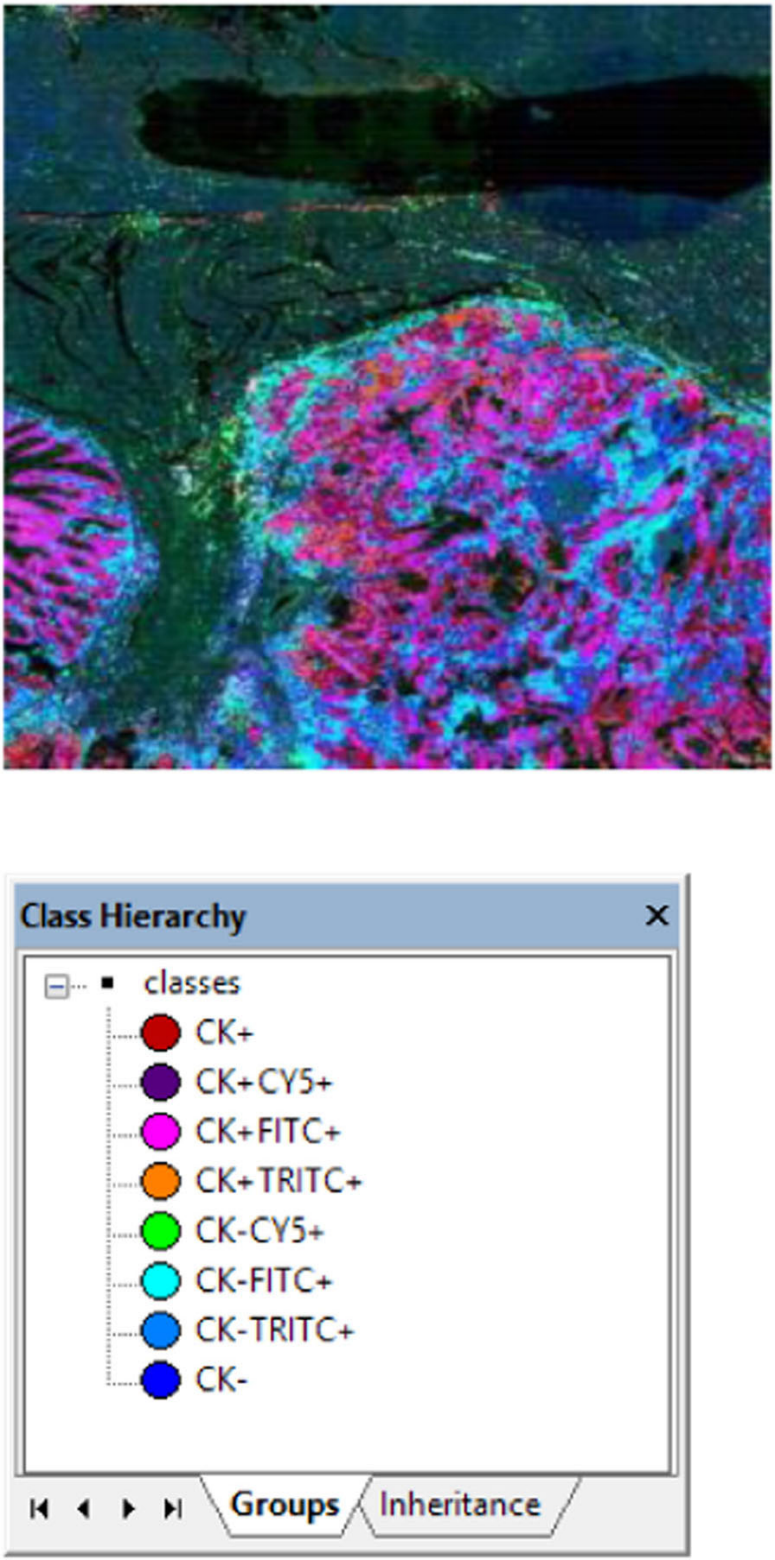
Phenotypes

**Fig. 2 (abstract P40).**
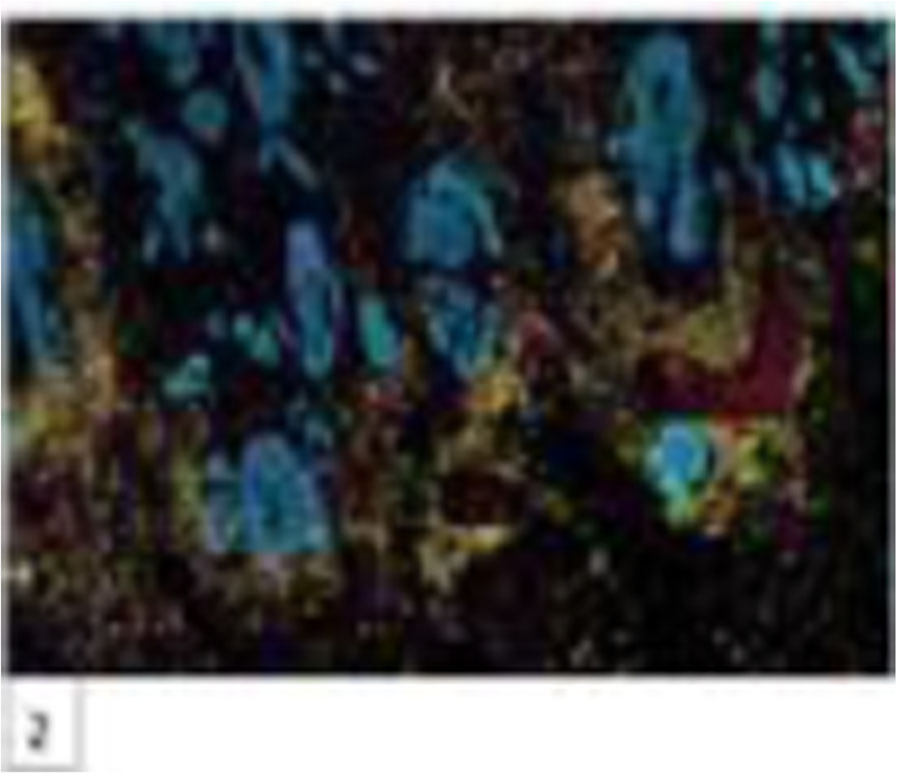
TiME regions of immune infiltration

**Fig. 3 (abstract P40).**
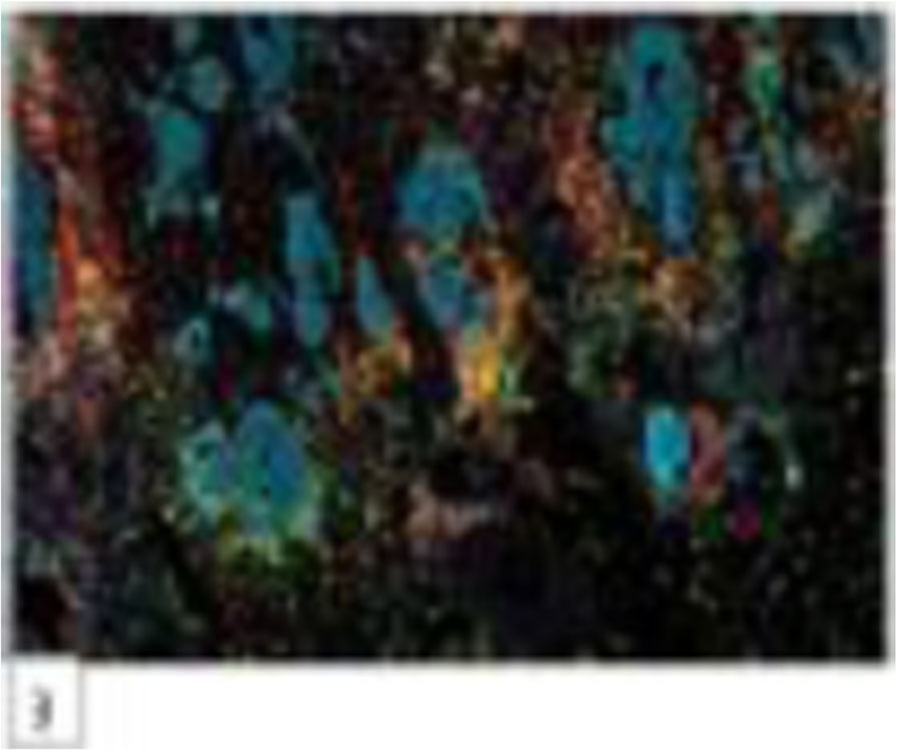
TiME regions of immune infiltration

**Fig. 4 (abstract P40).**
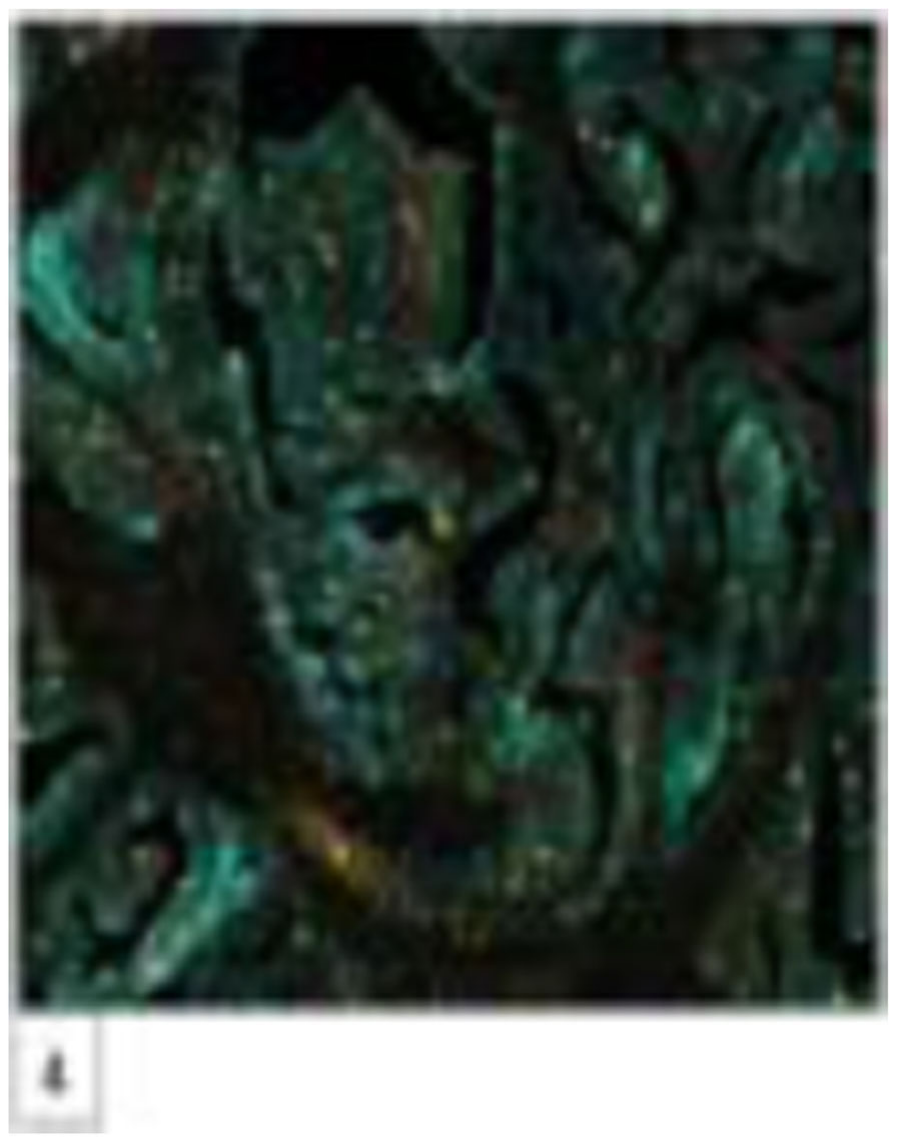
TiME regions of immune infiltration

**Fig. 5 (abstract P40).**
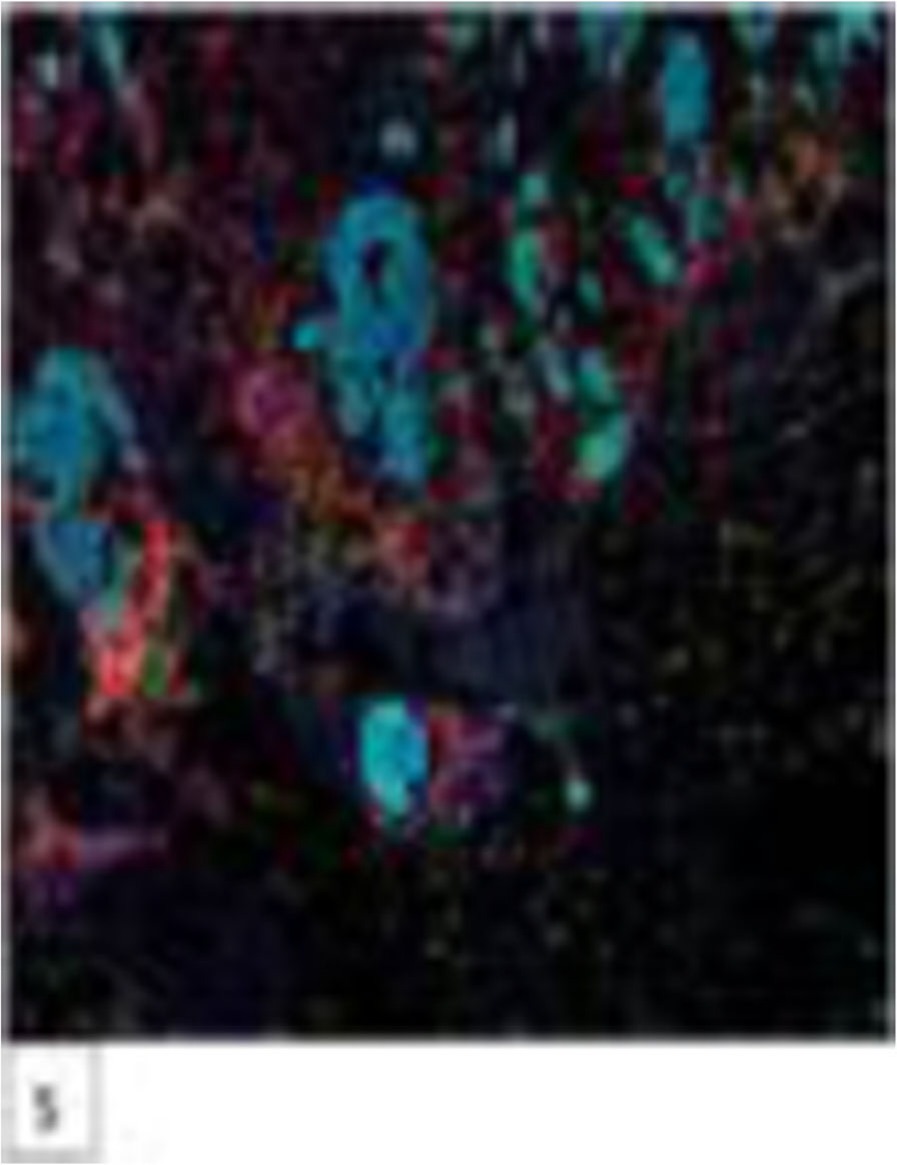
TiME regions of immune infiltration

#### P41 Multiplexed Imaging for the simultaneous detection of nucleic acids and proteins to dissect the tissue immune landscape and microenvironment of viral diseases

##### Sizun Jiang, PhD^1^, Xavier Rovira Clave, PhD^1^, Chi Ngai Chan, PhD^2^, Bokai Zhu^1^, Yunhao Bai, BS^1^, Marc Bosse, PhD^1^, David McIlwain, PhD^1^, Sean Bendall, PhD^1^, Michael Angelo, MD, PhD^1^, Jacob Estes, PhD^2^, Garry Nolan, PhD^1^

###### ^1^Stanford University, Stanford, CA, United States; ^2^Oregon Health and Sciences University, Stanford, CA, United States

####### **Correspondence:** Jacob Estes (estesja@ohsu.edu); Garry Nolan (gnolan@stanford.edu)

**Background**

Multiplexed Ion Beam Imaging (MIBI) is a novel imaging modality capable of resolving >40 parameters simultaneously in biological samples. Here, we developed viralMIBI, a highly sensitive method capable of detecting down to single copies of nucleic acids, in addition to protein epitopes. ViralMIBI enables the functional dissection of the immune landscape in viral driven diseases, such as that of tumor viruses (HBV, EBV, LCV) and others (HIV, SIV, Zika, Ebola). The combination of viralMIBI and cutting-edge cell neighborhood analytical methods will be paramount to better understand the immunological host-pathogen interactions for viral diseases, revealing insights into virus-induced immunodeficiency as well as virus-driven cancers.

**Methods**

To allow for the sensitive detection of nucleic acids, we took advantage of a customized branched DNA amplification method that can be easily adapted to a variety of multiplexed imaging platforms. Formalin-Fixed and Paraffin-Embedded (FFPE) tissue samples from Rhesus macaque animal models for a number of viral diseases were processed for viralMIBI nucleic acid and protein marker detection. Imaging was performed with the MIBIscope, a secondary ion mass spectrometry based device.

**Results**

We have established a robust method for highly multiplexed nucleic acid and protein epitope detection in FFPE tissue samples. As a proof of concept, we were able to detect down to single integrated copies of SIV. The establishment and validation of a Rhesus macaque specific antibody panel allowed for the in-depth characterization of cellular identities at the single-cell level, while maintaining their tissue geopositions.

**Conclusions**

ViralMIBI enables the MIBI to achieve highly sensitive nucleic acid detection, in addition to its multiplexed protein capabilities. Here, we leveage this method for the detection of various viral pathogens. ViralMIBI is also applicable to other targets, such as genomic amplifications frequently seen in cancers, or gene expression studies. The ability to image >40 parameters in tissue samples will vital for a better understanding of immune regulation of diseases, such as the establishment of viral related cancers as well as latent tissue reservoirs of pathogens. These discoveries can then be translated to better immunotherapy treatments against viral driven diseases.

**Acknowledgements**

We thank Matt Newgren for tireless technical support on the MIBI instrument, Rachel Finck, Xiao-Jun Ma and Bingqing Zhang for helpful discussions. S.J was supported by a Stanford Dean’s Fellowship and the Leukemia & Lymphoma Society Career Development Program. X.R.-C. was supported by a long-term EMBO fellowship. This work was supported by grants from the FDA, NIH, Parker Institute for Cancer Immunotherapy, the Bill and Melinda Gates Foundation, as well as the Rachford and Carlota A. Harris Endowed Professorship to G.P.N.

**Fig. 1 (abstract P41).**
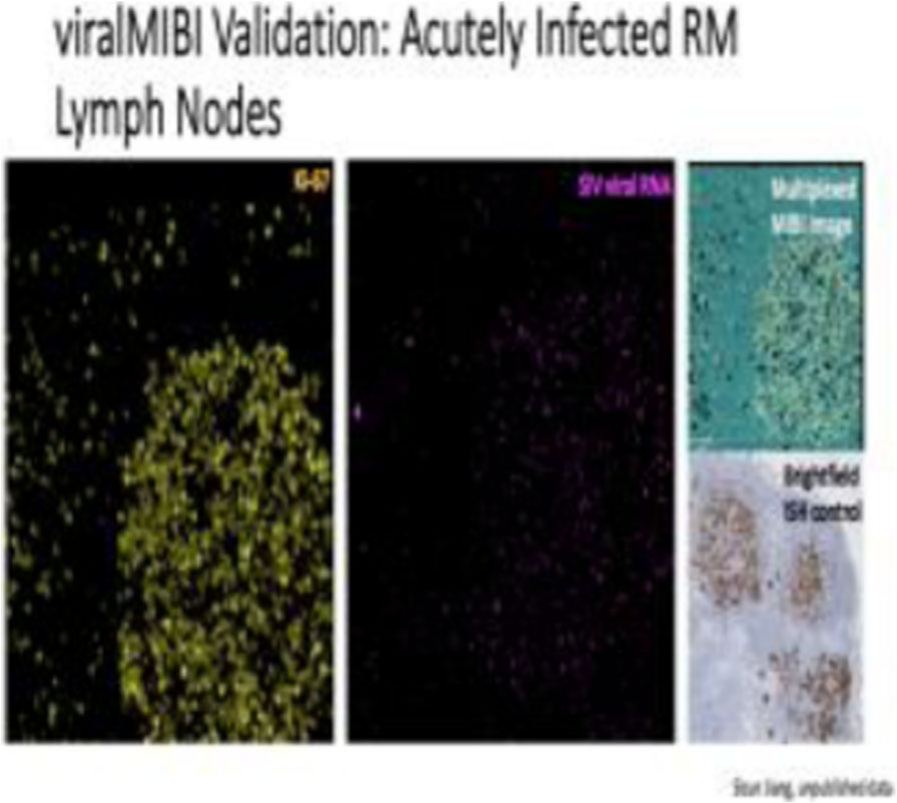
Validation of viralMIBI: Detection of SIV in infected tissue

**Fig. 2 (abstract P41).**
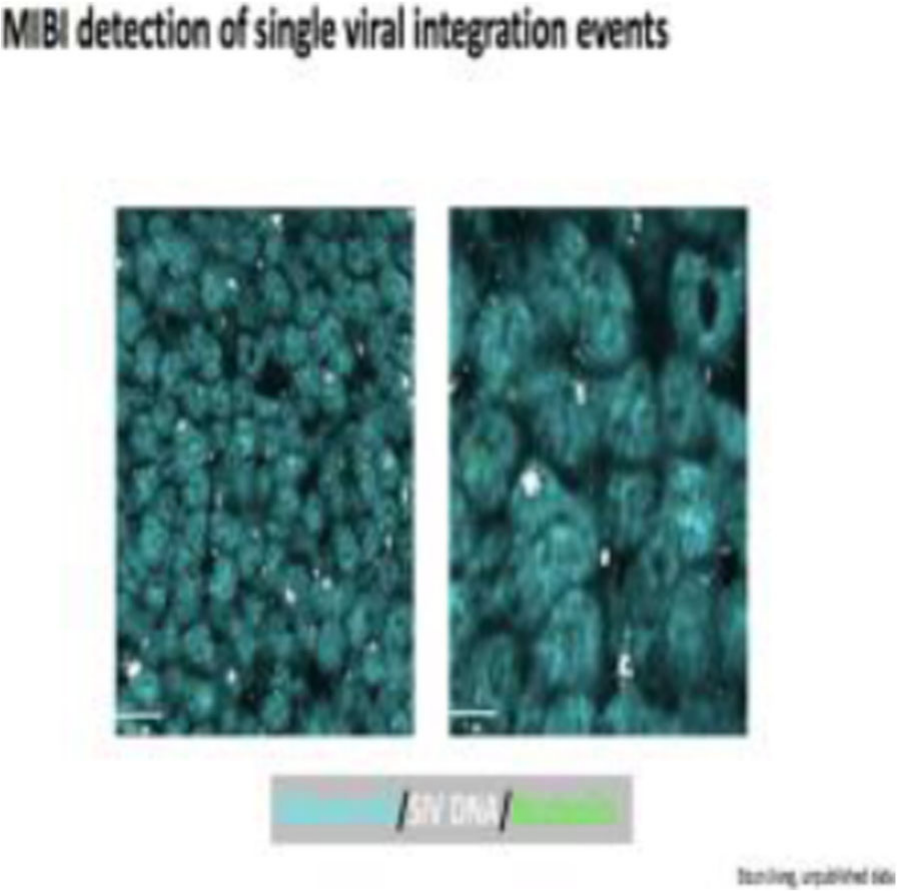
Detection of single integration events of SIV with viralMIBI

#### P42 An integrated multiplexing approach for the immunoprofiling of the tumor microenvironment of ovarian granulosa cell tumors

##### Juncker-Jensen, PhD^1^, Tyvette Hilliard^2^, Nicholas Stavrou^1^, Erinn Parnell^1^, Judy Kuo^1^, Eric Leones^1^, Flora Sahafi^1^, Josette William, PhD, MD^1^, Sharon Stack^2^, Anna Juncker-Jensen^1^

###### ^1^NeoGenomics, Aliso Viejo, CA, United States; ^2^University of Notre Dame, South Bend, IN, United States

####### **Correspondence:** Anna Juncker-Jensen (anna.juncker-jensen@neogenomics.com)

**Background**

Ovarian granulosa cell tumors (GCTs) are rare tumor accounting for 2-5% of all ovarian cancers. The main current treatment for GCT is surgery, however a subset require chemotherapy for residual and recurrent disease. GCT malignancies are often low-grade, however a clinical characteristic of these tumors is a tendency for late recurrence which is the most critical factor for GCT death. As the onset of recurrence is unpredictable, future research should focus on identifying both biomarkers for prognosis prediction, as well as targets that could help guide clinical trials in the development of targeted therapies for this rare indication. As GCTs are rare tumors making tissue availability very limited, we used a dual multiplexing approach in order to maximize the data output from a total of 14 FFPE tumor samples (6 primary tumors, and 8 recurrent tumors).

**Methods**

For protein multiplexing we have used MultiOmyx™, an immunofluorescence (IF) multiplexing assay utilizing a pair of directly conjugated Cyanine dye-labeled (Cy3, Cy5) antibodies per round of staining (Figure 1). Each round of staining is imaged and followed by dye inactivation enabling repeated rounds of staining and deactivation, while deep learning based cell classification algorithms identify positive cells for each biomarker. We generated a 15-marker panel consisting of CD3, CD4, CD8, FoxP3, CD68, CD163, HLA-DR, CD34, CTLA-4, PD-1, PD-L1, Ki67, vimentin, S100, and Pan Cytokeratin. For the gene expression analysis RNA was extracted from the adjacent 10 μm section and then analyzed using the Nanostring nCounter assay, specifically the 770 gene PanCancer Immune Panel. Hybridization, purification and immobilization and counts were based on manufacturer’s protocol.

**Results**

On protein level we confirmed previous findings that ovarian GCTs are so-called “cold” tumors, with a very low density of T cell infiltration. When we analyzed the presence of macrophages in the tumor microenvironment however, we found a 113% increase in TAM density in recurrent tumors compared to primary tumors. When searching for markers differentially expressed between primary and recurrent tumors we detected 4 genes in our PanCancer immune panel that were either significantly down-regulated (CCND3 or TOLLIP), or up-regulated (MAP3K and TNFSF4) in recurrent tumors. TNFSF4 encodes the protein OX40L, and interestingly a high expression of its receptor OX40 has previously been shown to be indicative for response to chemotherapy in recurrent ovarian cancer [1].

**Conclusions**

We have used a dual multiplexing approach on both gene and protein level in order to immunoprofile the tumor microenvironment of ovarian rare granulosa tumors.

**Reference**

1. Ramser M, Eichelberger S, Däster S, Weixler B, Kraljević M, Mechera R, Tampakis A, Delko T, Güth U, Stadlmann S, Terracciano L, Droeser RA, Singer G. High OX40 expression in recurrent ovarian carcinoma is indicative for response to repeated chemotherapy. BMC Cancer. 2018;18:425-433.

**Fig. 1 (abstract P42).**
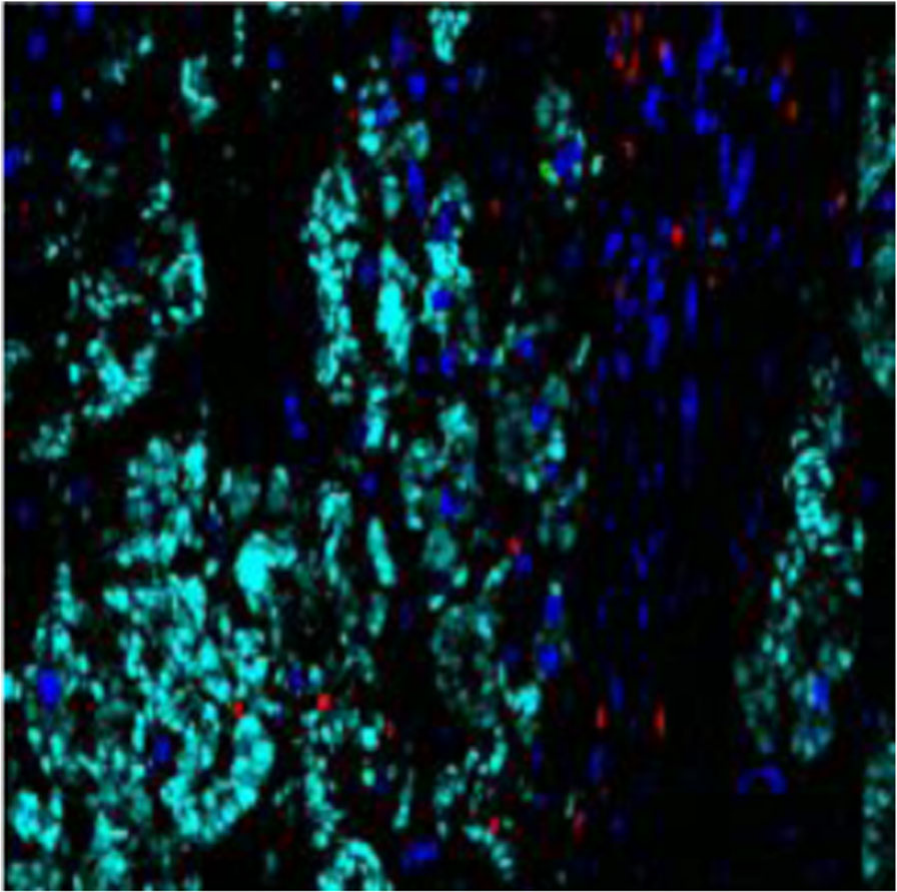
Immunofluorescent overlay image of GCT recurrent tumor

#### P43 A novel platform for highly multiplexed, single-cell imaging of cell suspensions

##### Anum Khan^1^, Won-Mean Lee^2^, Jon Mulholland^1^, Dhananjay Wagh^1^, John Coller^1^, Gabriel Mercado^2^

###### ^1^Stanford University, Palo Alto, CA, United States; ^2^Akoya Biosciences, Menlo Park, CA, United States

####### **Correspondence:** Won-Mean Lee (wmlee@akoyabio.com); Gabriel Mercado (gmercado@akoyabio.com)

**Background**

Analyzing populations at the single cell level has become increasingly important in the study of cancer and autoimmune disorders due to high levels of population heterogeneity and rare cell phenotypes that can drive disease pathogenesis and progression. Until recently, characterizing protein markers on single cells was limited to a handful of markers due to the technical and logistical challenges of flow cytometry platforms. New advancements in single cell analysis technologies have enabled researchers to study more than 30 parameters per cell. But these platforms are expensive and require significant panel design, thereby limiting access and usability. Here we demonstrate the use of the recently launched CODEX System to generate an in-depth immune profile of human PBMC samples.

**Methods**

The CODEX® System is an affordable, benchtop instrument that integrates with existing fluorescence microscopes and enables highly multiplexed imaging of over 40 markers in fresh frozen and FFPE tissue samples. The CODEX technology uses a DNA-based barcode library to label antibodies and iterative cycles of adding and removing cognate dye-labeled oligonucleotides to reveal the staining of three markers per cycle. Data acquisition is fully automated by the CODEX instrument. We tested a custom panel of more than 25 markers on the PBMC samples and acquired the images using a Keyence benchtop microscope.

**Results**

The CODEX system was used to generate highly multiplexed immune profiles of human PBMC samples using an optimized custom panel of CODEX antibodies. The images were processed using the CODEX Software Suite and cell phenotypes were clustered and annotated using the Multiplexed Analysis Viewer (MAV). Antibody specificity and panel performance were evaluated by assessing co-expression and mutually exclusive expression of relevant immune markers with the CODEX analysis pipeline.

**Conclusions**

Simultaneous analysis of tens of markers in blood or plasma samples can have several applications in the discovery of cellular biomarkers, immune monitoring and drug discovery and development. This preliminary study shows the compatibility of the CODEX system with cell suspensions for highly multiplexed, single-cell analysis and offers a more cost-effective method for immune profiling of blood samples.

#### P44 Solar-IHC: Cell-to-cell distances in the tumour immune microenvironment of Hepatocellular Carcinoma has the potential to prognosticate survival

##### Matthew Leong, NA^1^, Toh Han Chong^2^, Choo Su Pin^2^, Kiat Hon Lim Tony^3^, Joycelyn Lee^2^, David Wai^2^, Poh Sheng Joe Yeong^4^, Jin Miao Chen, PhD^5^

###### ^1^Lee Kong Chian School of Medicine, Nanyang Technological University, Singapore, Singapore, Singapore; ^2^National Cancer Centre Singapore, Singapore, Singapore; ^3^Singapore General Hospital, Singapore, Singapore, Singapore; ^4^Department of Anatomical Pathology, Singapore General hospital, Singapore, Singapore, Singapore; ^5^Singapore Immunology Network, Agency of Science, Technology and Research, Singapore, Singapore, Singapore

####### **Correspondence:** Jin Miao Chen (Chen_Jinmiao@immunol.a-star.edu.sg)

**Background**

Hepatocellular carcinoma (HCC) is a lethal cancer, being the fourth leading cause of cancer-associated mortality worldwide due to its low five-year survival and high reoccurrence rates [1], and identifying indicators of prognosis is key in developing novel treatments and improving survival of HCC patients. With the advent of digital pathology, the immune-architecture of solid tumours has become a central interest of cancer research and has been studied for the development of predictive and diagnostic applications. Here, we have assessed if intercellular Euclidean distances in the tumour immune microenvironment can possibly be used to predict patient prognosis.

**Methods**

In this study, biopsies were taken from 110 HCC patients who underwent surgical resection. The solar-IHC pipeline involves arranging the liver biopsies into tissue arrays and subsequently studying them using automated multiplex immunohistochemistry/immunofluorescence (mIHC/IF) protocol developed in Singapore General Hospital, with biomarkers Ecadherin, CD3, CD8, CD103 and PD1 [2], followed by image analysis software inForm version 2.4.2.

**Results**

Ecadherin was adopted as the tumour cell marker, and 26 immune cell phenotypes are defined by variable levels of immune markers CD8, CD103, and PD-1 (as shown in Table 1) Dimensionality reduction and unsupervised clustering of the distances between tumour and immune cell phenotypes showed distinct clusters of patients with significant differences in clinical outcomes. Long cell-cell distances between immune cell phenotypes and tumor cells was associated with an improved overall survival (p-value = 0.02) and disease-free survival (p-value = 0.01), while the opposite was true for short cell-cell distances between immune cell phenotypes and tumor cells. This was observed in the analysis of all CD8+, CD8-, CD103+, CD103-, PD1+ and PD1- cells, possibly due to the suppressive immunomodulatory effect Ecadherin+ tumour cells has on neighbouring immune cells [3]. Furthermore, machine learning enabled the prediction of clusters with considerable accuracy, with a K-fold cross-validation of 91%. This indicates a strong association of cell-cell distances with patient survival, and a robust reproducibility of distance pattern-based predictors.

**Conclusions**

In this study, our data suggests that the analysis of intercellular distances has the potential to be used as a prognostic indicator in HCC. Coupled with next generation machine learning techniques, this novel approach to cell-to-cell distance analysis has the potential to be an easily implementable algorithm to predict patient prognosis in HCC. This bioinformatics approach can also be utilized in the analysis of other biomarkers and cancer types, and this brings exciting prospects for the future of cancer research.

**References**

1. McGlynn KA, Petrick JL, London WT. Global epidemiology of hepatocellular carcinoma: an emphasis on demographic and regional variability. Clinics in liver disease. 2015;19(2):223-38.

2. Lim JCT, Yeong JPS, Lim CJ, Ong CCH, Wong SC, Chew VSP, Ahmed SS, Tan PH, Iqbal J: An automated staining protocol for seven-colour immunofluorescence of human tissue sections for diagnostic and prognostic use. Pathology. 2018 Apr;50(3):333-341

3. Nagl S, Haas M, Lahmer G, Büttner-Herold M, Grabenbauer GG, Fietkau R, et al. Cell-to-cell distances between tumor-infiltrating inflammatory cells have the potential to distinguish functionally active from suppressed inflammatory cells. Oncoimmunology. 2016;5(5):e1127494.

**Ethics Approval**

This study was approved by the Institutional Review Board (IRB), approval number 2014/590/B.

**Table 1 (abstract P44).**
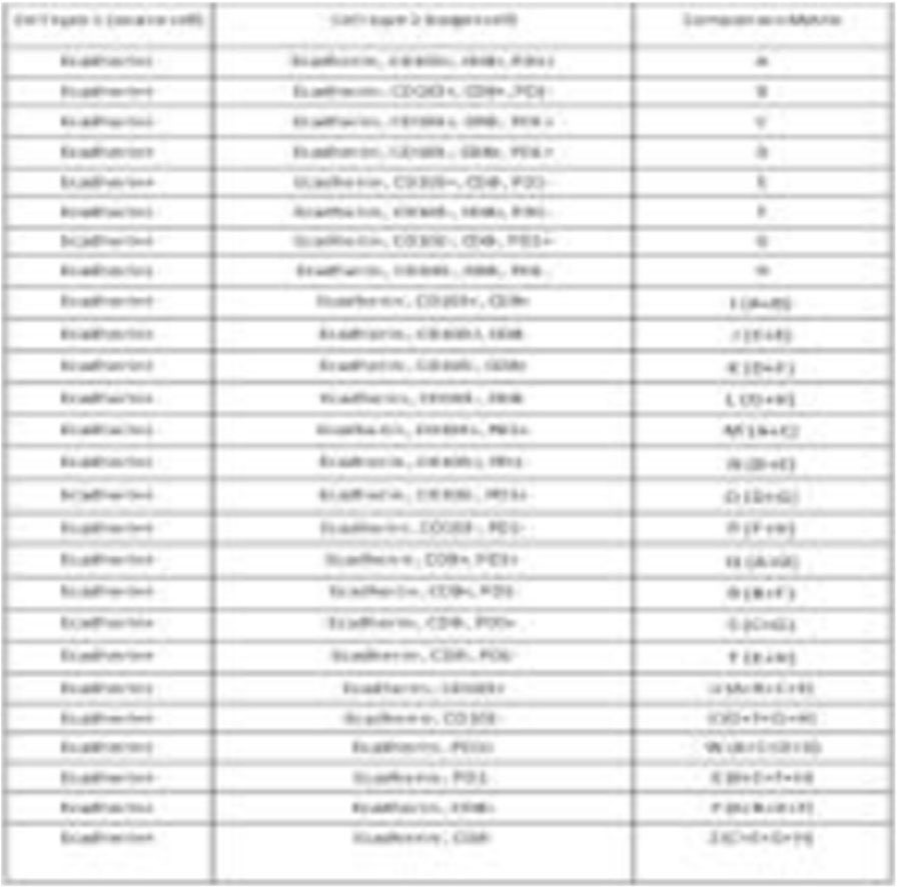
See text for description

#### P45 Multiplex immunofluorescence staining, whole slide imaging, and spatial phenotyping of T-cell exhaustion, regulatory T cells, and myeloid-derived suppressor cells in tumor FFPE samples

##### Kyla Teplitz^2^, Katir Patel, PhD^3^, Michael Tomac, MS^1^, Kate Lillard, PhD^1^, Mael Manesse, PhD^3^, Anne Hellebust, PhD^1^

###### ^1^Indica Labs, Inc., Alcester, United Kingdom; ^2^Rarecyte, Inc, Seattle, WA, United States; ^3^Ultivue, Inc, Cambridge, MA, United States

####### **Correspondence:** Kate Lillard (kate@indicalab.com)

**Background**

The immune cell milieu that comprises the tumor microenvironment (TME) is highly heterogeneous and complex. Depending on biological interactions and functional state, immune cell populations can either promote or suppress tumor progression. CD8+ T cells, for example, are the primary mediators of anti-tumor immunity; however, they are often ineffective either because they are unable to infiltrate the tumor or because they become functionally exhausted [1,2], Pathologically activated myeloid-derived suppressor cells (MDSCs) which infiltrate the tumor are also associated with tumor progression [3,4]. Multiple biomarkers are required to accurately identify these individual immune cell types and their functional states. In this work, we employ advanced multiplexing techniques to observe biologically and functionally distinct T cell and MDSC populations and to quantify their density and distribution within the TME of several tumor types.

**Methods**

UltiMapper assays were used to perform multiplex immunofluorescence on multiple tumor FFPE samples (lung, colorectal, breast). Three multilpex panels were run in this study: UltiMapper PD-1 [CD3, CD45RO, PD-1, CK/Sox1], UltiMapper T-reg[CD4, CD8, FoxP3, CK, Sox10], and UltiMapper MDSC[CD11b, CD14, CD15, HLA-DR].FFPE slides were stained using the BOND RX autostainer from Leica Biosystems and scanned on the CyteFinder® II HT Instrument from RareCyte, Inc. This instrument performs high-speed, whole-slide scanning in the 5 channels used in the UltiMapper Kits and outputs an open source, stitched, pyramidal TIFF. Image analysis was conducted using HALO 3.0 software to perform cell phenotyping, proximity analysis, image registration, and density mapping.

**Results**

Cell counts for relevant phenotypes were obtained for each panel to identify exhausted T cells, T-regs, cytotoxic T cells, M-MDSCs, and PMN-MDSCs. Spatial analysis was employed to map the degree of T-cell infiltration and exhaustion correlating to T-reg and MDSC expression in the tumor microenvironment.

**Conclusions**

Here we present a workflow for tackling the complexity of the tumor immune microenvironment by leveraging high-quality multiplex panels, high-speed whole-slide imaging, and quantitative spatial analysis. UltiMapper assays used in this study (PD-1, T-reg, and MDSC) were able to identify single-cell phenotypes through co-localization and negative selection of markers. Using HALO image analysis, cell populations were enumerated and quantified to measure the level of T-cell exhaustion caused by T-cell regulation and myeloid-derived immune cell suppression.

**References**

1. Fridman WH, Zitvogel L, Sautes-Fridman C, Kroemer G. The immune contexture in cancer prognosis and treatment. Nat. Rev. Clin. Oncol. 2017; 14:717–734.

2. Thommen DS, Schumacher TN. T Cell Dysfunction in Cancer. Cancer Cell. 2018; 33:547-562.

3. Gabrilovich DI, Ostrand-Rosenberg S, Bronte V. Coordinated regulation of myeloid cells by tumours. Nat. Rev. Immunol. 2012; 12:253–268.

4. Kumar V, Patel S, Tcyganov E, Gabrilovich DI. The Nature of Myeloid-Derived Suppressor Cells in the Tumor Microenvironment. Trends Immunol. 2016; 37:208–220.

#### P46 Same-slide multiplex immunofluorescence and brightfield histological staining as a new research tool for fast and comprehensive pathology assessment of the tumor microenvironment

##### Mael Manesse, PhD, Douglas Wood, PhD, Heike Boisvert, PhD, Sean Downing, PhD, Mael Manesse, PhD

###### Ultivue, Cambridge, MA, United States

####### **Correspondence:** Mael Manesse (mael.manesse@ultivue.com)

**Background**

Innovative and efficient translational research tools enabling a better understanding of the tumor and its microenvironment are a keystone of the development of digital pathology. Current immunohistochemistry (IHC) methods limit the depth of information from a single tissue sample to a single target in the case of chromogenic staining, or to sample morphology and general cell identification in the case of hematoxylin and eosin staining (H&E). True phenotyping requires the use of a single section, as serial sections may not contain the same cells, especially small immune cells such as T-cells. Multiplex immunofluorescence (mIF) methods have been established to provide insights into a wide number of markers of interest and their spatial context in a single sample. Here, we demonstrate a new research approach combining multiplexed detection of protein markers with standard H&E pathology review in tumor samples, in a streamlined, single-day sample-to-answer workflow.

**Methods**

InSituPlex technology was used to perform multiplex immunofluorescence staining of formalin-fixed, paraffin-embedded (FFPE) samples from human tonsil and primary tumor biopsies on the Leica Biosystems BOND RX autostainer. The tissues were then imaged in five distinct fluorescent channels (DAPI, FITC, TRITC, Cy5, Cy7) before being stained using standard H&E protocols and imaged again. Fluorescent and brightfield whole-slide images were acquired on a ZEISS AxioScan.Z1 slide scanner. Images of the same tissue section were co-registered and fused into a single image for analysis using Indica Labs HALO software.

**Results**

The InSituPlex technology enables deep phenotyping of immune cells through colocalization and co-expression of multiple protein markers in tumor samples. Phenotypic information was then overlaid with the H&E image of the same section to facilitate identification and immuno-profiling of specific cells in the tumor and its environment. The fused images were also analyzed to provide cell counts, distance mapping, and expression levels of each of the markers.

**Conclusions**

In this work, we present a new modality for pathology research with a convenient workflow that enables fast tissue review and deep immuno-profiling and phenotyping of the tumor via fusion of H&E and mIHC staining of the same tissue section.

#### P47 Imaging cancer immunology: Systemic tracking of immune cells in vivo with magnetic particle imaging

##### James Mansfield, Msc, Gang Ren^1^, Jeff Gaudet^1^, Yanrong Zhang^2^, Sara Ghobadi^2^, Max Wintermark^2^, Patrick Goodwill^1^

###### ^1^Magnetic Insight, Alameda, CA, United States; ^2^Stanford University, Palo Alto, CA, United States

####### **Correspondence:** James Mansfield (jim@jmansfield.com)

**Background**

The rapid growth of research into immuno-oncology research has fueled a need to track be able to determine the location of a variety of immune cells systemically and in solid tumors. However, existing methods for cell tracking that have generally been insufficient. Magnetic Particle Imaging (MPI) is a novel tomographic molecular imaging technique that can be used to non-invasively track iron-oxide tagged immune cells in 3D in vivo, with contrast similar to nuclear medicine but without the complex workflow, safety, and half-life limitations. In this study, we compared the behavior of monocytes loaded with nanoparticles in vitro and nanoparticles injected intravenously and subsequently taken up by phagocytic cells (in situ loading) and imaged using MPI the differences in biodistribution and migration of monocytes in in naïve and tumor-bearing and naïve (control) mice.

**Methods**

Twenty mice were implanted with 300,000 4T1 tumour cells in the 4th mammary fat pad. CD11b+ mouse monocytes were harvested using EasySep® Mouse CD11b positive selection kit II (StemCell Technologies). The isolated monocytes were prelabeled with Vivotrax® (100 μg/mL). On day 7 post-implantation, either 5 million prelabeled cells or free Vivotrax (6 mg/kg) were intravenously injected into normal or tumor-bearing mice for in vitro or in situ targeting experiments (N=5 mice for all four groups). 3D MPI images using a MOMENTUM MPI system (Magnetic Insight) were acquired 1, 4, 7 and 10 days after injection. MicroCT images (CT120, Trifoil Imaging) were acquired and co-registered using VivoQuant (Invicro). Tumors, liver, spleen and draining lymph nodes were then harvested, imaged, fixed, and stained with Prussian blue and analyzed for iron contents.

**Results**

Tumor-bearing mice showed a significant accumulation of nanoparticles for both the in situ and in vitro targeting methods, although the time and amount of accumulation was different. For both experiments, nanoparticles were predominately detected in the expanding margins of the tumor. For the in vitro labeled monocytes, accumulation was rapid, with the maximum accumulation being at 24 hours post-injection, while for the in situ labeled cells, accumulation was slower.

**Conclusions**

By combining the sensitivity, specificity as well as accurate quantitation potentials of MPI, information can be obtained on labeled monocytes and their biodistribution in tumour models. Other cells can also be labeled (dendritic cells, MDSCs, NKs, and T cells) and this information can be utilized to better understand the factors influencing immune cell migration in and around tumors.

#### P48 Turning ‘cold’ tumours ‘hot’: Guided magnetic hyperthermia for tumour immune stimulation

##### Patrick Goodwill^1^, Daniel Hensley^1^, Zhi Wei Tay^2^, Elaine Yu^1^, James Mansfield, Msc^1^, Blayne Kettlewell^1^, Ryan Orendorff^1^, Kyle Fields^1^, Steve Conolly^2^

###### ^1^Magnetic Insight, Alameda, CA, United States; ^2^University of California Berkeley, Berkeley, CA, United States

####### **Correspondence:** James Mansfield (jim@jmansfield.com)

**Background**

Cancer immunotherapy is now the “fifth pillar” of cancer therapeutics [1]. Although hugely successful, there are limitations. In many studies, less than half the patients are responsive to therapy. One hypothesis is that refractory tumours are immunologically ‘cold’ – i.e., there are insufficient immune cells in the tumour for the therapy to be efficacious [2]. Thus, methods to stimulate an immunogenic response in solid tumours to improve immunotherapy efficacy are desirable.

Hyperthermia is known to induce a local immunogenic response, making it a potential adjunct to radiation and immune therapies. One hyperthermia method is Magnetic Fluid Hyperthermia (MFH), which is based on electromagnetic heating of magnetic nanoparticles (MNPs) [3,4]. , However, poor control of heating localization and magnitude have prevented MFH’s widespread clinical adoption.

Magnetic Particle Imaging (MPI) is an emerging tracer imaging technique that directly detects and quantitates superparamagnetic iron-oxide nanoparticles with exceptional contrast and high sensitivity at millimeter-scale resolutions [5]. MPI’s contrast is similar to nuclear medicine, but without the complex workflow, safety, and half-life limitations of a radioactive tracer.

**Methods**

Here we describe how MPI and MFH can be combined to produce spatially localized heating and accurate control of heating magnitude. Spatial localization is achieved using a unique mechanism, magnetic localization. Localization is effected by using a strong magnetic field gradient to produce a “field-free region” (FFR) where nanoparticles are heated, while nanoparticles outside the FFR are quenched and do not heat. The use of an FFR thus enables millimeter-scale control over which MNPs are heated [6-8].

**Results**

MPI is first used to quantitate the MNPs prior to heating, to enable treatment planning and prediction of the heating dose. MFH can then be induced in target regions of interest located anywhere in the body while avoiding regions containing MNPs that should not be heated, such as the liver or lymph nodes.

**Conclusions**

Combined MPI-MFH enables new treatment workflows that exploit spatially localized MFH and accurate control of heating magnitude. These workflows may resemble image-guided radiation therapy or image-guided high-intensity focused ultrasound. Combined MPI-MFH also prevents damage to nearby healthy tissue while enabling new applications such as targeted immunogenic stimulation. MPI-MFH also enables new heat-actuation applications involving systemic injection of MNPs followed by local targeting such as local release of a drug [9] (break thermally labile bonds/nanocarriers) without requiring active chemical targeting. While currently only available for small animal use, its underlying physics does not prevent its translation to human sizes

**References**

1. Zaidi, N; Jaffee, E. J Clin Invest (2018).

2. Sharma, P. et al. Curr Opinion Immunol (2016).

3. Jordan, A. et al. Journal of Magnetism and Magnetic materials, (1999).

4. Latorre, M. Puerto Rico health sciences journal, 28(3) (2009).

5. Gleich, B. & Weizenecker, J. Nature 435,1214–1217 (2005).

6. Murase, K. Physica Medica, 29(6), 624-630 (2013).

7. Hensley, D. Physics in Medicine & Biology, 62(9), 3483 (2017).

8. Tay, Z. ACS nano, 12(4), 3699-3713 (2018).

9. Liu, J. F., Small, 14(44), 1802563 (2018).

#### P49 Use of Ultivue InSituPlex® multiplex immunofluorescence to localize and quantify regulatory T lymphocytes in formalin-fixed paraffin-embedded human tissue sections

##### Shawn O'Neil, DVM, PhD^1^, Renee Huynh^1^, Courtney Hebert^2^, Jamie Buell^2^, Sean Downing, PhD^2^, John Jakubczak, PhD^1^, Yutian Zhan, MS^1^

###### ^1^Pfizer, Cambridge, MA, United States; ^2^Ultivue, Inc., Cambridge, MA, United States

####### **Correspondence:** Shawn O’Neil (llospo@gmail.com)

**Background**

The inflammatory bowel diseases ulcerative colitis (UC) and Crohn’s disease (CD) are chronic, relapsing inflammatory disorders of the gastrointestinal tract (GIT) that affect millions of individuals worldwide [1]. The pathogenesis of these disorders is thought to involve dysregulation of mucosal immune homeostasis in the GIT in response to environmental factors in genetically susceptible individuals [2]. Regulatory T cells (Treg) are CD4+ T lymphocytes that play a central role in peripheral immune tolerance, actively inhibiting inflammation upon antigenic stimulation. There are two major populations of Treg: conventional Treg and TR1 cells [3]. Conventional Treg arise from the thymus (tTreg) or can be induced in the periphery (pTreg); both tTreg and pTreg constitutively express FoxP3 and CD25 (IL-2Rα). An imbalance in conventional Treg and effector T cells in the GIT microenvironment is thought to play a part in the pathogenesis of inflammatory bowel disease (IBD) [4]. Thus, we sought to quantify conventional Treg and CTL populations in GIT tissue sections from IBD patients versus normal individuals by multiplex immunofluorescence.

**Methods**

Conventional Treg are typically defined as lymphocytes with a CD3+/CD4+/CD25+/FoxP3+ immuno-phenotype. This complex antigenic signature has made it difficult to definitively label Treg populations in tissue sections by immunohistochemistry. We combined a 5-plex (CD3, CD4, CD8α, CD25, FoxP3) immunofluorescence assay using Ultivue InSituPlex® multiplex technology with image analysis using Indica Labs HaloTM software to identify, localize and enumerate: 1) total CD3+ T cells, 2) CD8α+ cytotoxic T lymphocytes (CTL) and 3) CD3+/CD4+/CD25+/FoxP3+ conventional Treg in formalin-fixed paraffin-embedded (FFPE) sections of GIT from patients with UC and CD versus controls. Using this approach, we were able to definitively identify and enumerate these immune cell populations on single FFPE tissue sections from each specimen.

**Results**

We found greater Treg and CTL cell densities (cells/mm2) in colon from CD and UC patients versus controls and higher densities of Treg and lower densities of CTL in small intestine from patients with CD versus controls.

**Conclusions**

The Ultivue InSituPlex© assay was capable of discretely localizing conventional Tregs and CTL in human tissues. This multiplex platform could be used to simultaneously localize Tregs and CTL in FFPE surgical resections and biopsies of neoplastic tissue as well.

**References**

1. Ng SC, Shi HY, Hamidi N, Underwood FE, Tang W, Benchimol EI, Panaccione R, Ghosh S, Wu JCY, Chan FKL, Sung JJY, Kaplan GG. Worldwide incidence and prevalence of inflammatory bowel disease in the 21st century: a systematic review of population-based studies. Lancet. 2018; 390:2769-78.

2. Corridoni D, Arseneau KO, Cominelli F. Inflammatory bowel disease. Immunol Lett. 2014; 161:231-5.

3. van Herk EH, Te Velde AA. Treg subsets in inflammatory bowel disease and colorectal carcinoma: Characteristics, role, and therapeutic targets. J Gastroenterol Hepatol. 2016; 31:1393-404.

4. Yamada A, Arakaki R, Saito M, Tsunematsu T, Kudo Y, Ishimaru N. Role of regulatory T cell in the pathogenesis of inflammatory bowel disease. World J Gastroenterol. 2016; 22:2195-205.

**Ethics Approval**

Human tissues were obtained from the National Disease Research Interchange (NDRI) with support from NIH grant U42OD11158. Tissues were collected for research purposes under IRB-approved informed consent and collection procedures and provided to Pfizer in accordance with applicable government regulations and guidelines.

#### P50 Rapid high-plex staining and simultaneous imaging for immunophenotyping of tissue sections

##### Benjamin Pelz, PhD^1^, Daniel Migliozzi^2^, Diego Dupouy^1^, Anne-Laure Leblond^3^, Alex Soltermann^3^, Martin Gijs^2^

###### ^1^Lunaphore Technologies, Lausanne, Switzerland; ^2^École Polytechnique Fédérale de Lausanne, Lausanne, Switzerland; ^3^Universitätsspital Zürich, Zurich, Switzerland

####### **Correspondence:** Benjamin Pelz (benjamin.pelz@lunaphore.com)

**Background**

The tumor microenvironment plays a vital role in cancer development. Multiplex immunostainings allow studying the interaction of different cell types in the tumor microenvironment using a single tissue slide. Though several techniques are available to perform high-plex stainings, they require intensive manual handling, are highly time consuming or not compatible with tissue sections on standard microscope slides. Here we present a fully automated microscope integrated method for rapid high-plex sequential fluorescent immunostaining and imaging of tissue sections.

**Methods**

Formalin-fixed, paraffin-embedded tissue sections underwent manual dewaxing and antigen retrieval step. All subsequent steps of staining, antibody elution and imaging were automated on the microscope integrated microfluidic device. A single tissue section was stained sequentially for 24 different immunophenotyping and tissue structural markers. Each staining cycle consisted of incubation of the tissue section with a pair of mouse and rabbit primary antibodies, followed by the corresponding fluorescently labelled secondary antibodies and DAPI. The section was imaged after each staining cycle and subsequently eluted before staining the next pair of markers.

**Results**

Our microscope integrated microfluidic system allowed automated 24-plex staining with conventional primary and fluorescently labelled secondary antibodies in less than five hours, including image acquisition steps. The microfluidic tissue processor enabled fast fluidic exchange and thereby resulted in reduced staining time down to 10-12min per marker. Integration of a window into the microfluidic chip allowed direct tissue imaging under the microscope avoiding the removal and mounting of the slide. Protocol optimization resulted in a high signal to background noise ratio for each marker and complete elution of antibodies from the previous staining step. A comparison of a 10-plex staining with standard chromogenic stainings on sequential sections showed high concordance for the stained area on tonsil as well as lung cancer tissue sections (Figure 1).

**Conclusions**

With the microscope integrated microfluidic system, it is possible to perform fast multiplex stainings including image acquisition without the need to handle the tissue slide. Moreover, due to the sequential nature of the system it would be easily possible to further increase the number of markers in the multiplex staining. We foresee this technique to greatly facilitates the execution of high-plex stainings and thereby the discovery of novel tumor-microenvironment interactions.

**Fig. 1 (abstract P50).**
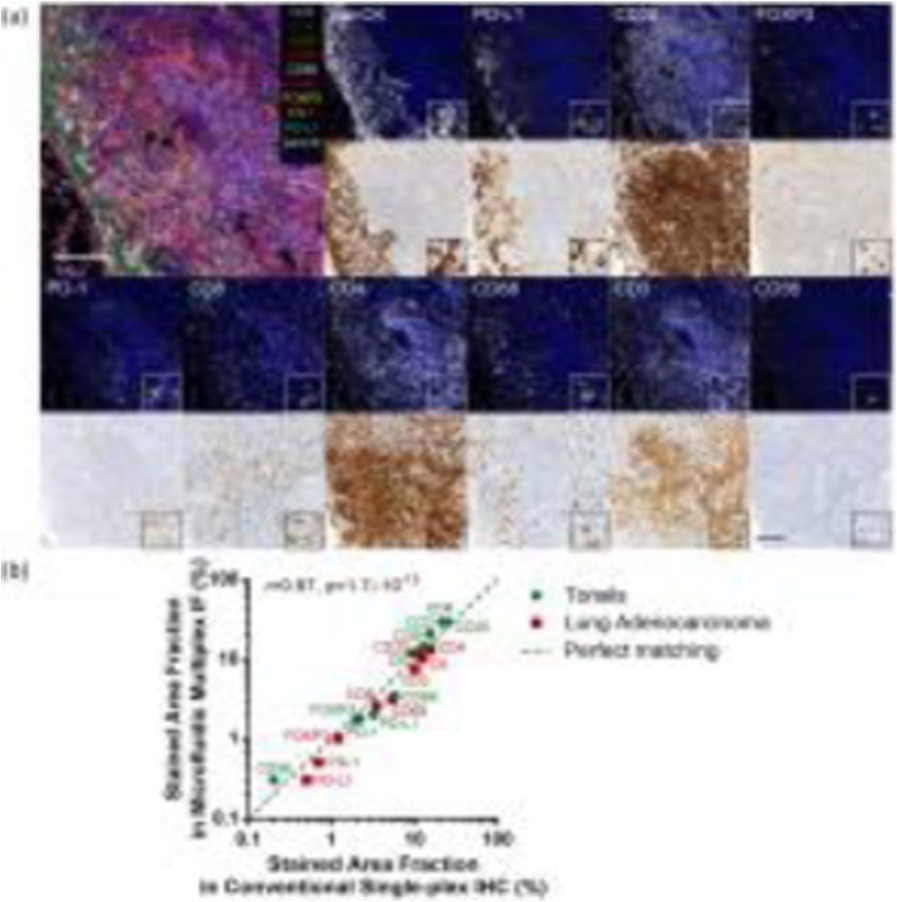
Automated microfluidics-assisted multiplexing

#### P51 Phenotypic and spatial analysis of inter- and intra-tumor heterogeneity using multiplexed ion beam imaging (MIBI)

##### Jason Ptacek, PhD^1^, Robert Johnson, PHD^2^, Joann Palma, PHD^2^, Jay Tarolli^1^, Rachel Finck^1^, Murat Aksoy^1^, Yi Zhang^1^, Jessica Finn^1^, Jason Ptacek, PhD^1^

###### ^1^Ionpath, Inc, Menlo Park, CA, United States; ^2^AbbVie, North Chicago, IL, United States

####### **Correspondence:** Jessica Finn (jessica.finn@ionpath.com)

**Background**

Elucidating both the cell types present in the tumor microenvironment and the spatial relationship between immune and cancerous cells is at the forefront of immunotherapy research. To address this, MIBI has been developed to image up to 40 markers at single cell resolution.

**Methods**

Staining of 10 NSCLC formalin-fixed paraffin embedded (FFPE) tissue sections was performed similarly to traditional IHC except that a panel of 20 metal labeled antibodies were stained simultaneously. The tissue was imaged at subcellular resolution using an ion beam and time-of-flight secondary ion mass spectrometry (ToF-SIMS). The masses of detected species were then assigned to target biomolecules given the unique label of each antibody and multi-step processing and segmentation were performed to create images of the TME and enable quantitative metrics of different cell subsets.

**Results**

Control samples imaged at study start and end showed consistent marker quantification (inter-run R2>0.99), indicating MIBI staining and acquisition is reproducible and robust. Each tumor sample was imaged across 10 regions of interest (ROIs) to assess heterogeneity of the TME. Highly expressed nuclear, membrane, and cytoplasmic markers were utilized in conjunction to accurately determine cell boundaries in tissue images. The resulting single cell segmentation enabled quantitative analyses of both marker expression and the spatial relationships between cells of different types. At the highest level, cells were classified as positive for markers that are indicative of immune and tumor cells based on measured intensities of marker expression, such as CD45+ and keratin+ cells in epithelial cancers, respectively. Co-expression of markers were used to classify immune cells into subsets, including T cells and macrophages (Figure 1). Cell types and their frequency were compared within the 10 ROIs collected per sample as well as between samples. Finally, distances between tumor and the closest immune cell were measured as a means for describing the spatial organization of the TME, which has been linked to patient survival.

**Conclusions**

MIBI offers high-parameter capability, at sensitivity and resolution uniquely suited to understanding the complex tumor immune landscape, including the spatial relationship of immune and tumor cells and the expression of immunoregulatory proteins.

**Fig. 1 (abstract P51).**
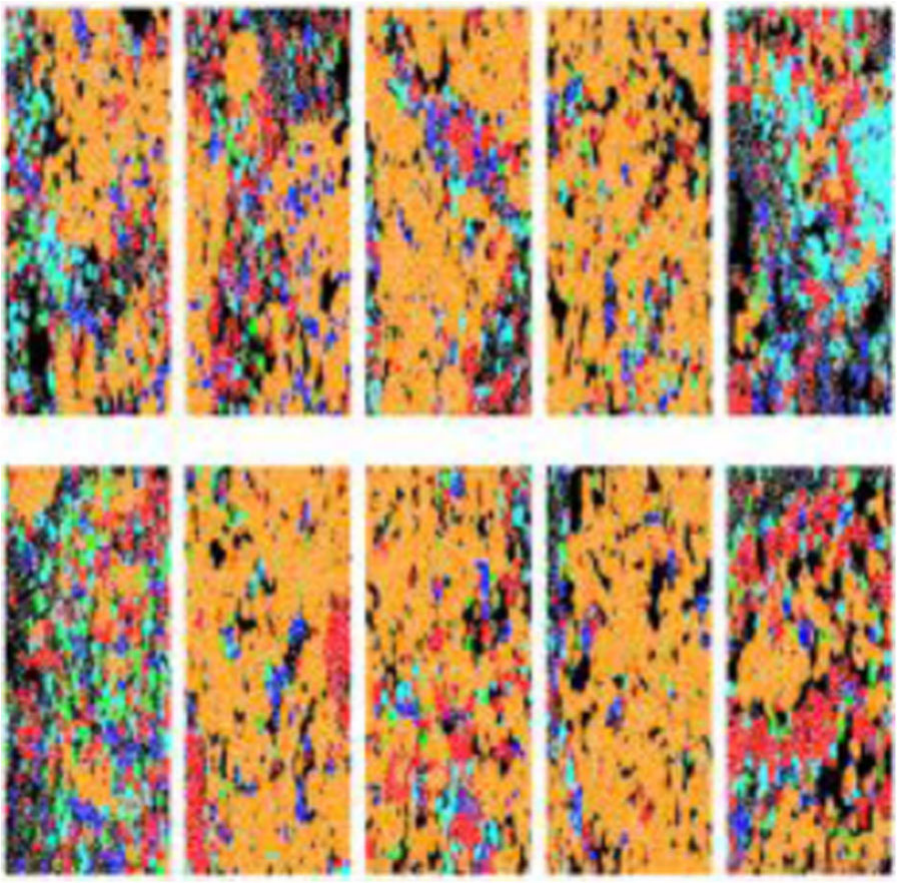
Cell segmentation and classification

#### P52 CyTOF in anti-ILT3 mAb drug discovery - humanized tumor model selection and in vivo PD/biomarker exploration

##### Yujie Qu, MD, Alan Byford, Caniga Michael, Ying Huo, Barbara Joyce-Shaikh, BS, Laurence Fayadat-Dilman, Veronica Juan, Carl Mieczkowski, Laura Bald, Jeanne Baker, Michael Meehl, Scott Pruitt, MD, PhD, Stephen Alves, Lily Moy, Philip Brandish, PhD, Jie Zhang-Hoover, Jie Zhang-Hoover

###### Merck, Boston, MA, United States

####### **Correspondence:** Jie Zhang-Hoover (jie.zhang-hoover@merck.com)

**Background**

ILT3 on human monocytic myeloid cells is linked to immune tolerance in transplantation and immune suppression in cancer. Anti-ILT3 mAb is being developed as a cancer immunotherapy to reverse the suppression and increase T cell activation. To evaluate the effect of an anti-ILT3 mAb in vivo, we sought to select an appropriate humanized tumor model and identify immune activation signatures in the model that are associated with the treatment efficacy.

**Methods**

Humanized tumor models were generated by subcutaneously implanting Panc 08.13 or SK-MEL-5 tumor cells in NSG mice engrafted with human cord-blood CD34+ hematopoietic stem cells (hu-NSG). Tumor-bearing mice were treated with a human-mouse chimeric anti-ILT3 mAb. Single cell mass cytometry (CyTOF) that simultaneously quantifies over 40 cell surface and intracellular markers was used to phenotype tumor infiltrating cells (TILs) in these tumor models.

**Results**

The CyTOF phenotyping of untreated mice showed an overall immune suppressive environment in the tumor in both Panc 08.13 and SK-MEL-5 hu-NSG models. ILT3 expression was detected in both models. However, the levels of ILT3 expression on CD14+ myeloid cells and percentage of CD14+ myeloid cells among TILs were higher in SK-MEL-5 compared to Panc 08.13 tumors, which led to the selection of the SK-MEL-5 model for further exploration. Anti-ILT3 mAb treatment in the SK-MEL-5 hu-NSG model increased activation of CD14+ myeloid sub-populations in TILs by viSNE CyTOF clustering analysis. Furthermore, the treatment increased levels of CD69 and HLA-DR expression on CD4+ T cells, while reducing the percentage of naïve CD4+ T suppressor cells in CD45+ TILs.

**Conclusions**

Anti-ILT3 mAb treatment induced a conversion from intra-tumoral immune suppression to activation in a SK-MEL-5 hu-NSG tumor model. CyTOF in anti-ILT3 drug discovery holds promise to effect a paradigm-shift in our ability to understand MOA and evaluate the impact of therapeutic interventions that can accelerate biomarker discovery and drug development.

The anti-ILT3 mAb activity in human immune cells in vitro and tumor efficacy in vivo is presented in a companion poster.

**Ethics Approval**

The study was approved by Merck Institutional Animal Care and Use Committee, approval number 2022-200518-FEB.

#### P53 Pixelwise H-score: a novel digital image analysis-based metric to quantify membrane biomarker expression from IHC images

##### Amy-Jackson Fisher, Pamela Whalen, Cory Painter, Pamela Vizcarra, Eric Powell, MD, Sripad Ram, PhD

###### Pfizer, Inc., San Diego, CA, United States

####### **Correspondence:** Sripad Ram (sripad.ram@gmail.com)

**Background**

Immunohistochemistry (IHC) assays play a central role in evaluating biomarker expression in tissue sections for diagnostic and research applications. Manual scoring of IHC images, which is the current standard of practice, is based on qualitative criteria and are known to have several shortcomings in terms of reproducibility and scalability to large scale studies. While digital image analysis (DIA) based approaches hold significant promise to overcome these limitations, current DIA methods pose several challenges that have limited their widespread use in analyzing clinical samples.

**Methods**

We introduce a novel DIA metric, the pixelwise H-score (pix H-score), that quantifies biomarker expression from whole-slide scanned IHC images. Pix H-score is unique in that it does not rely on the detection of individual cells or the delineation of subcellular compartments (e.g. nucleus and cell membrane) which are necessary for traditional scoring algorithms such as the H-score. All DIA metrics are calculated using either commercially available (HALO, Visiopharm) or open-source (QuPath) digital pathology software packages.

**Results**

We compute the pix H-score, the ATM score [1] and the traditional H-score [2] from IHC images for several biomarkers including PD-L1. Our results show that the pix H-score exhibit tight concordance to multiple orthogonal measurements such as mRNA levels and pathologist score, and provide consistently better performance over other DIA metrics.

**Conclusions**

We anticipate that the new metric introduced here will be broadly applicable to quantify biomarker expression from a wide variety of IHC images. Although not shown here, the new metric can also be applied to immunofluorescence images. Moreover, these results underscore the benefit of digital image analysis-based approaches which offer an objective, reproducible and highly scalable strategy to quantitatively analyze IHC images.

**References**

1. Choudhury KR, Yagle KJ, Swanson PE, Krohn KA, Rajendran JG, A Robust Automated Measure of Average Antibody Staining in Immunohistochemistry Images. J Histochem Cytochem. 2010; 58: 96-107.

2. Hatanaka Y, Hashizume K, Nitta K, Kato T, Itoh I, Tani Y, Cytometrical image analysis for immunohistochemical hormone receptor status in breast carcinomas. Pathol Int. 2003; 53: 693-699.

#### P54 Development of a 9-color immunofluorescence assay using tyramide signal amplification and multispectral imaging for high-throughput studies on FFPE tissue sections

##### Bethany Remeniuk, PhD, Carla Coltharp, PhD, Kristin Roman, MS, Chichung Wang, Clifford Hoyt, MS

###### Akoya Biosciences, Hopkinton, MA, United States

####### **Correspondence:** Clifford Hoyt (choyt@akoyabio.com)

**Background**

In cancer research, advancing our understanding of the underlying mechanisms driving disease progression is key to developing new therapeutic regimens and improving patient outcomes. Over the past several years, multiplex immunofluorescence (mIF) has played a vital role in elucidating novel immune-tumor interactions and identifying targets of interest for drug discovery and development.

Emerging studies utilizing mIF have revealed complex cell-to-cell interactions within the tumor microenvironment (TME), however, greater interrogation of the biology comprising these interactions, including cellular composition and functional status, require higher levels of multiplexing. With the rapidly increasing number of available multiplexing approaches, there is an inherent tradeoff between capability and throughput.

In this study, we demonstrate a streamlined workflow to develop and optimize a 9-color assay on the Leica BOND RX™ autostainer. This methodology offers an optimal balance between multiplexing and sample throughput to facilitate research and support translational studies on whole formalin-fixed paraffin-embedded (FFPE) tissue.

**Methods**

For the 9-color assay, Opal™ fluorophores were used on serial sections of lung cancer FFPE tissue. The panel was designed on Akoya’s Mantra 2 semi-automated multispectral microscope, which allows for rapid analysis of staining performance. Once optimized, multispectral images were acquired on both the Mantra 2 and Vectra Polaris of the same tissue regions and analyzed to show equivalence between the platforms. Cell counts, densities, and spatial parameters were generated using Akoya’s inForm image analysis software and the R script package phenoptrReports, which produces quick, summarized outputs of the image analysis data. These same analyses were also used to evaluate reproducibility of all markers when run in a high-throughput process.

**Results**

Dynamic range of measured per cell signals for all markers had a median of 200:1. Agreement between the Mantra 2 and Vectra Polaris-based measurements was generally >95% when comparing cellular expression signals and cell counts based on cell phenotyping classifiers. Cross talk was undetectable after spectral unmixing despite significant spectral overlap inherent in a 9-color assay. Reproducibility across three batches of five serial sections of lung cancer tissue was generally <10% coefficient of variation for all markers in the assay, supporting a high-through process of approximately 20 resection samples per day.

**Conclusions**

We have successfully established a standardized process for 9-color multiplexing that offers a balance between elucidating the intricate cellular biology driving disease progression and therapeutic responsiveness within the TME while simultaneously providing a practical and reliable assay that can be implemented to support translational, high-throughput studies in clinical research.

#### P55 Combining the best of two worlds: Transfer of multiplex immunofluorescence images from non-small cell lung carcinoma patients into pseudo multiplex chromogenic immunohistochemistry images

##### Lorenz Rognoni, PhD^1^, Ana Hidalgo Sastre, PhD^2^, Linda Brützel^1^, Philipp Wortmann^1^, Monika Baehner^1^, Marco Testori^1^, Jessica Chan^1^, Bonnie Phillips, PhD^3^, Katir Patel, PhD^3^, Sean Downing, PhD^3^, Alex Haragan^4^, John Field^4^, Florian Leiss, PhD^1^

###### ^1^Definiens AG, Munich, Germany; ^2^Definiens, Munich, Germany; ^3^Ultivue, Cambridge, MA, United States; ^4^Liverpool University Hospital, Liverpool, United Kingdom

####### **Correspondence:** Ana Hidalgo Sastre (ahidalgo@definiens.com)

**Background**

One of the biggest challenges in multiplex chromogenic IHC (mIHC) is to accurately identify and quantify double positive cells. Multiplex immunofluorescence (mIF) instead, allows for visualization of plenty of biomarkers at once with true co-localization. However, visualizing tissue morphology in mIF images can be challenging and the vast color combinations overwhelming. Pathologists are key to retrieve biological information from multiplex assays and provide annotations for assay validation. To support pathologist analysis, resections of non-small cell lung carcinoma (NSCLC) patients were stained with mIF and displayed as pseudo mIHC images. Additionally, consecutive slides were stained with a mIHC panel. PD-L1 positive macrophages from the pseudo mIHC images were quantified and compared to the readouts identified in the real chromogenic IHC.

**Methods**

7 formalin-fixed paraffin-embedded (FFPE) resections from NSCLC patients were stained using Ultivue’s UltiMapper I/O PD-L1 kit and I/O PD-1 kit and whole image scans were acquired with a Zeiss Axio Scan.Z1 scanner (Zeiss). Consecutive slides were stained with a multiplex chromogenic panel (including CD68, CD8, PD-1) at Mosaic Laboratories (1) and scanned with an Aperio AT Turbo scanner (Leica). Images were analyzed using an automated workflow for quantitative multiplex image analysis developed at Definiens (2). Afterwards, mIF images were converted into pseudo mIHC images. Pathologists annotated double positive macrophages for CD68 and PD-L1 on both images. Results were compared with automatically detected double positive cells and across assays. In addition, pathologists qualitatively assessed visual similarity of real and artificial chromogenic images.

**Results**

Pathologists annotated double positive macrophages for CD68 and PD-L1 markers on both images (mIF and pseudo mIHC). Results were compared with those obtained using artificial intelligence to automatically detect double positive cells and across assays. In addition, pathologists qualitatively assessed visual similarity of real and artificial chromogenic images (Figure 1).

**Conclusions**

Transferring mIF into pseudo mIHC images helps to combine the advantages from both approaches: true colocalization of biomarkers whilst maintaining tissue morphology, facilitating visual evaluation of digital images by pathologists. This technology could be used to complement research, clinical routine diagnostic, drug development and biomarker discovery.

**References**

(1) Lisa M. Dauffenbach, Christopher A. Kerfoot, et al. Characterization of inflammatory cell patterns and densities using multiplex immunohistochemistry immuno-oncology assays [abstract]. In: Proceedings of the AACR-NCI-EORTC International Conference: Molecular Targets and Cancer Therapeutics; 2017 Oct 26-30; Philadelphia, PA. Philadelphia (PA): AACR; Mol Cancer Ther 2018;17(1 Suppl): Abstract nr B069.

(2) Lorenz Rognoni, PhD; Vinay Pawar, PhD; Tze Heng Tanet, et al. Automated quantification of whole-slide multispectral immunofluorescence images to identify spatial expression patterns in the lung cancer microenvironment. SITC Annual Meeting; 2018 Nov 7-11; Washington, DC. Poster nr P442.

**Ethics Approval**

Ethical approval was granted by the Liverpool Research Ethics Committee, reference number 97/141.

**Fig. 1 (abstract P55).**
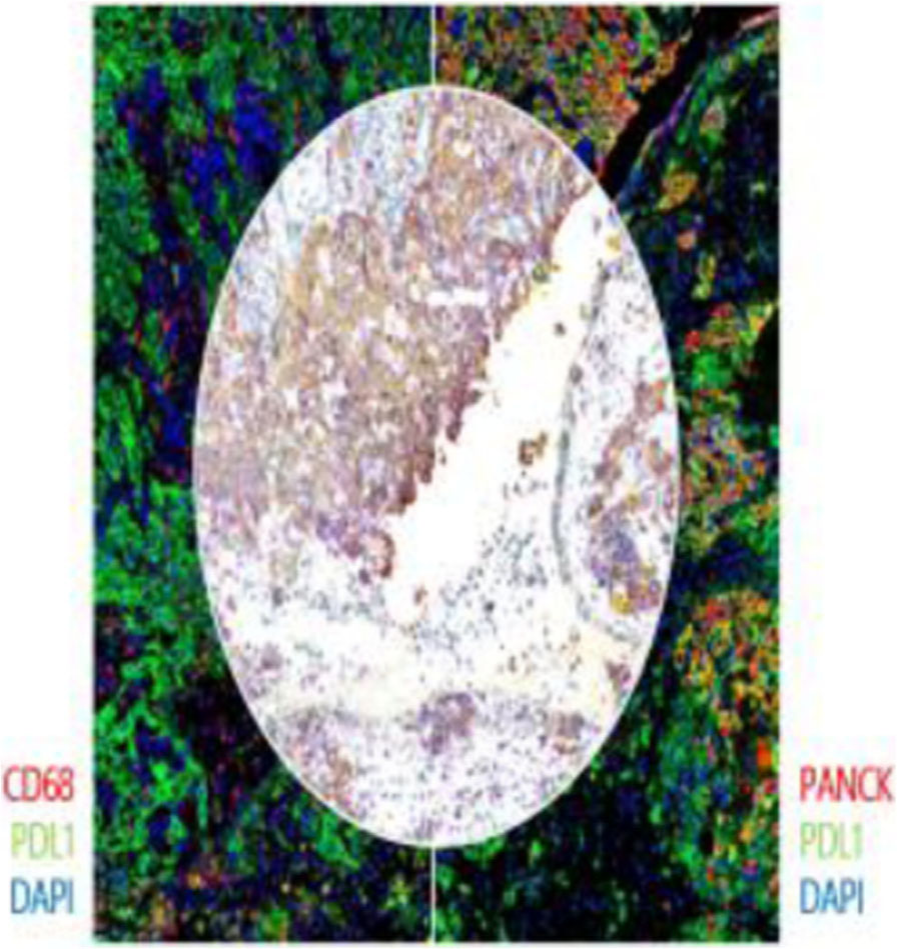
mIF and pseudo mIHC

#### P56 Interrogating the effect of oncolytic Herpes simplex virus-1 on spatial arrangement of myeloid cells in glioblastoma multiforme using an ex vivo human system and multiplex immunohistochemistry

##### Munisha Smalley, PhD^1^, Misti Jain, PhD^2^, Saravanan Thyiagarajan^2^, Emily Alonzo^3^, Katherine Crosby^3^, Douglas Best^4^, Hans Gertje, BS^2^, Basavaraja Shanthappa^2^, Ralph Pulchalski^5^, Charles Cobbs^5^, E. Antonio Chiocca^1^, Sean Lawler, PhD^1^, Aaron Goldman^1^, Munisha Smalley^1^

###### ^1^Brigham and Women's Hospital, Woburn, MA, United States; ^2^Mitra Biotech RxDx, Bangalore, India; ^3^Cell Signaling Technologies, Danvers, MA, United States; ^4^University of Birmingham, Birmingham, United Kingdom; ^5^Swedish Neuroscience Institute, Seattle, WA, United States

####### **Correspondence:** Aaron Goldman (goldman1@mit.edu)

**Background**

The role of myeloid cell populations within a tumor and their contribution to effective cancer immunotherapy is emerging with considerable interest. However, assessing the role of intratumoral myeloid cells under therapy pressure has it’s challenges. Here, we implemented a multiplex immunohistochemistry (mIHC) panel and a human ex vivo system to interrogate key myeloid subsets as they affiliate with infiltration and activation of an emerging immunotherapy for glioblastoma multiforme (GBM) – oncolytic Herpes simplex virus-1 (oHSV-1). mIHC combined with advancements in digital pathology and machine learning algorithms have enabled identification, quantification and spatial orientation of multiple cell types in a single field of view (FOV).

**Methods**

Cell Signaling Technology antibodies (CD3e,D7A6E™, ID: 85061), (CD68, D4B9C, ID: 76437), (CD11c, D3V1E, 49420), (MHC Class II (HLA-DRB) LGII-612.14), (Pan-Keratin, C11, 4545) were optimized for mIHC staining, using a tyramide signal amplification approach to pin-point, in a single FOV: intratumoral T-cells, defined by CD3e+; macrophages, defined by CD68 and conventional dendritic cells defined by CD11c+MHCII +, in relation to the surrounding tissue architecture defined by pan cytokeratin. These biomarkers were integrated with incidences of oHSV-1 infiltration and replication (via expression of green fluorescent protein).

**Results**

First, we confirmed an optimized protocol for treating GBM ex vivo with oHSV-1 such that tissue viability, infiltration and replication of the virus are optimal. The staining of the mIHC panel was optimized using matched 3,3′-Diaminobenzidine chromogenic and single biomarker fluorescent controls tonsil tissue, which were validated using tumor samples from patients with high grade glioma. We characterized the spatial arrangement of myeloid subpopulations, ex vivo and correlated the changes in spatial orientation, quantity and localization of cells to the tumor. We determined that dynamic re-arrangement of myeloid cells can be observed under pressure of immunotherapy within the tumor, and confirm both a time-dependent and dose-dependent effect of oHSV-1 on this immune cell modulation.

**Conclusions**

These data suggest a unique, multiplexed approach to study spatial arrangement of myeloid and T-cell populations and their spatial distribution within tumors under basal growth conditions or in the presence of anticancer immunotherapies, which may implicate the activity of myeloid cells with treatment responses. These findings could impact personalized cancer immunotherapy for patients receiving care.

**Ethics Approval**

The samples were collected under IRB approval.

**Consent**

The samples were collected under written patient consent for publication of this abstract.

#### P57 An ex-vivo human system elucidates a role for natural killer cells in the anticancer effect of drug combinations in triple negative breast cancer

##### Aaron Goldman^1^, Douglas Best^2^, Saravanan Thiyagarajan^2^, Misti Jain, PhD^2^, Basavaraja Shanthappa^2^, Munisha Smalley, PhD^1^, Hans Gertje, BS^2^, Aaron Goldman^1^

###### ^1^Brigham and Women's Hospital; ^2^Mitra Biotech, Woburn, MA, United States

####### **Correspondence:** Aaron Goldman (goldman1@mit.edu)

**Background**

Response and resistance to cancer therapy relies on the presence of active immune cells in the tumor microenvironment, which recalibrate the body’s own defense largely by modulating exhaustion of cytotoxic lymphocytes including T cells and natural killer (NK) cells. However, there is a critical gap in our understanding for the role of immune cells to drive response or resistance to drugs and immunotherapies at the individual patient level. This is primarily due to limitations in complex tumor-immune interfaces that exist in many current tumor models.

**Methods**

Here, we deployed an ex-vivo human system that uses an explant of native, patient-derived solid tumors including autologous immune cells. Utilizing biopsied tumor tissue from patients diagnosed with triple-negative (ER- PR- HER2-) breast cancers (TNBC, N=7), we studied drug-induced cell death (cleaved caspase-3) and spatial heterogeneity of NK cells (CD3-CD56+PanCK-) using multiplex immunohistochemistry (mIHC). Spatial orientation of cells in the microenvironment, including proximity of NK to tumor and NK cell density within regions of the tumor vs. stroma were performed using HALO-based quantitative analyses. Finally, we deployed in-vitro co-culture studies using 3-D TNBC organoids and human-derived NK cells (NK-92MI).

**Results**

First, we report the ability of the ex-vivo human system to retain the spatial orientation and total population of natural killer cells and T-cells over the course of a 72h explant culture. Next, using Spearman correlation analyses and principal component analysis (PCA), we determined that drug response to both immunotherapy and conventional cancer drugs, indicated by high incidence of cleaved caspase-3 after drug pressure, is directly associated with changes to the tumor-NK cell proximity and density of NK cells within the tumor bed vs. the stroma. Finally, using the 3-D tumor organoid cultures with NK cells, we determined that activity and tumor cytolysis by NK cells is hampered through cancer cell-activated cytokines, which diminish expression of activating biomarkers including NKG2D/C.

**Conclusions**

Taken together, these results provide a method to study the spatial arrangement of immune cells in an entirely human system, which can be perturbed with anticancer drugs to reliably influence the expression and growth patterns of immune cells. We further demonstrate that this strategy can help to guide in-vitro studies to further elucidate mechanisms of action of drugs, which influence response vs. resistance via immune cell activity.

**Ethics Approval**

Anonymous breast cancer tissue samples were collected under IRB approval with due written consent from each patient.

#### P58 Brain MRI performed within 4 weeks of PD-1 inhibitors as a potential prognostic marker for non-small cell lung cancer (NSCLC)

##### Ammar Sukari, MD, Misako Nagasaka, MD, Seongho Kim, PhD, Tahmida Chowdhury, Natasha Robinette, MD

###### Karmanos Cancer Institute, Detroit, MI, United States

####### **Correspondence:** Ammar Sukari (sukaria@karmanos.org)

**Background**

PD-1 inhibitors aim to re-instate the natural anti-cancer immune-mediated cytotoxicity. Although PD-1 inhibitors are now considered part of standard of care treatment in advanced metastatic NSCLC [1], little is known about the effects of PD-1 inhibitors on asymptomatic central nervous system (CNS) metastases. We hypothesized that early MRI brain imaging due to the development of neurological signs and symptoms following the initiation of PD-1 inhibitor may help delineate a subset of NSCLC patients with asymptomatic and undiagnosed CNS metastases prior to initiation of therapy and may predict for worse outcomes.

**Methods**

Data from NSCLC patients who received at least one dose of PD-1 inhibitors between September 2013 through the data cut-off of May 2017 were captured from our institution’s pharmacy database. The primary objective was to describe the characteristics of patients with MRI brain being performed within 4 weeks of the first dose of PD-1 inhibitors and the secondary objectives were estimation of progression free survival (PFS) and overall survival (OS) for the same population.

**Results**

140 NSCLC patients received at least one dose of PD-1 inhibitors prior to data cut-off. Median age was 64 (range: 24-86). 83 (59%) were male. 64 (46%) were treated on a clinical trial. There were 92 (66%) adenocarcinoma, 41 (29%) squamous cell carcinoma (SCC) and 7 (5%) poorly differentiated NSCLC. 84 (40%) had a pre-PD1 inhibitor MRI brain performed and 25 (18%) had been diagnosed with baseline CNS metastases. 128 (91%; Group 1) did not have an MRI brain performed within 4 weeks of starting PD-1 inhibitors, while 12 (9%; Group 2) patients did. 9 out of 12 patients had new or worsening CNS metastases. Of the 9, 1 had WBRT, 1 had gamma knife, 1 went onto hospice while a decision was made to monitor imaging and symptoms in 6 patients. The median PFS was 5.28 months (95% CI, 3.90 to 8.03) and 1.75 months (95% CI, 1.08 to NE) for Group 1 and Group 2, respectively. The median OS was not reached (95% CI, 15.38 to NE) and 5.77 months (95% CI, 2.85 to NE) for Group 1 and Group 2, respectively.

**Conclusions**

In this retrospective analysis, patients who had MRI brain within 4 weeks of starting PD-1 inhibitors had worse outcomes.

**Reference**

1. NCCN Clinical Practice Guidelines in Oncology. Non-Small Cell Lung Cancer. Version 5. 2019- June 7, 2019. https://www.nccn.org/professionals/physician_gls/default.aspx#site, last accessed 7/30/2019.

**Ethics Approval**

The study was approved by the Wayne State University Institution's Ethics Board, approval number 062616M1E.

#### P59 Highly consistent automated multiplex immunofluorescence for immunoprofiling of solid tumors in clinical trials: assay validation study using multispectral imaging and digital analysis

##### Michael Surace, PhD^1^, Lorenz Rognoni, PhD^2^, Farzad Sekhavati^2^, Andrew Fisher, PhD^2^, Andreas Spitzmueller^2^, Sara Batelli, PhD^2^, Karma Dacosta^1^, Vinay Pawar^2^, Clifford Hoyt^3^, Edwin Parra, MD, PhD^4^, Jaime Rodriguez-Canales, MD^1^

###### ^1^AstraZeneca, Gaithersburg, MD, United States; ^2^Definiens AG, Munich, Germany; ^3^Akoya Biosciences, Hopkinton, MA, United States; ^4^UT - MD Anderson Cancer Center, Houston, TX, United States

####### **Correspondence:** Jaime Rodriguez-Canales (rodriguezcanalesj@medimmune.com)

**Background**

Novel multiplex immunofluorescent (mIF) platforms have been developed for immunoprofiling of solid tumors to understand the tumor microenvironment and to identify biomarkers for immunotherapy. One of these methods employ IF and tyramide signal amplification (TSA) to generate between 4 to 9-marker multiplex panels analyzed with multispectral imaging. Although this method can provide reliable data, they can show variability in consistency depending on the markers [1], which affects the reliability of mIF for use in clinical trials. The goal of this study was to develop and validate a highly consistent mIF method for its use in clinical trials in the pharma and academic environments.

**Methods**

A mIF panel for the analysis of carcinomas was optimized using an automated stainer (Leica) and automated multispectral scanner (Polaris, Akoya Biosciences). The markers included keratins (AE1/AE3), CD68, PD-L1, PD1, CD8 and Ki67. Each primary antibody was first performed on standard chromogenic IHC according to previously validated protocols. The mIF panel was developed with a secondary antibody detection and TSA, using specific fluorophores for each marker. Once optimized, the miF panel was tested on serial sections of formalin-fixed human tonsil controls and six non-small cell lung carcinomas, including replicates, together with standard IHC staining of each individual marker for comparison. A set of three repeats on different days for each mIF with all cases was performed to test consistency and reproducibility of the mIF method. All slides were digitally scanned and analyzed using Automated Definiens Insights Platform with custom algorithms (Definiens AG, Munich, Germany), comparing the cell populations in the serial section slides between standard IHC and mIF, and between mIF repeated rounds. The data was statistically analyzed using Pearson’s correlations.

**Results**

Using an automated workflow, the mIF data compared with standard IHC showed correlations between 0.83 to 0.99. The data from the three rounds of mIF performed on different days showed correlations between 0.89 and 0.99. The marker that showed the lowest correlation was CD68 (r=0.83), the possible cause was the difficulties on cell segmentation due to morphologic irregularity shown by macrophages. Overall our present data showed a much higher consistency than our previously published results using a non-automated mIF protocol, which correlations ranged from 0.17 to 0.87[1].

**Conclusions**

Our data demonstrates that using an automated workflow including automated staining, scanning and analysis, and with properly validated IHC markers, mIF becomes a highly consistent methodology and it is compatible for its use with clinical trial tissue specimens.

**Trial Registration**

Not applicable

**Reference**

1. Parra ER, Uraoka N, Jiang M, Cook P, Gibbons D, Forget MA, Bernatchez C, Haymaker C, Wistuba II, Rodriguez-Canales J. Validation of multiplex immunofluorescence panels using multispectral microscopy for immune-profiling of formalin-fixed and paraffin-embedded human tumor tissues. Sci Rep. 2017; 7:13380-13391.

#### P60 Combining transcriptomic immune population inference with automated digital masking of H&E images finds immune effectors preferentially distribute within stroma regions

##### Christopher Szeto, PhD^1^, Mustafa Jaber, PhD^2^, Liudmila Beziaeva^2^, Kevin Kazmierczak^1^, Steve Benz^1^, Shahrooz Rabizadeh^1^

###### ^1^ImmunityBio, Santa Cruz, CA, United States; ^2^NantOmics, Culver City, CA, United States

####### **Correspondence:** Steve Benz (Steve.Benz@nantomics.com)

**Background**

Multiple methods to characterize immune-cell populations in tumor microenvironment (TME) are being assessed as potential biomarkers of immunotherapy response. These include manual pathological assessment of lymphocyte infiltration, immunohistochemical (IHC) staining for specific adaptive response markers such as CD8, and more recently transcriptomic-based deconvolutions of immune populations such as xCell and TIMER. Here we combined digital masking using deep-neural nets with transcriptomic deconvolution to infer where immune-subpopulations may reside in the TME.

**Methods**

An unselected set of 187 clinical samples from the ImmunityBio database were analyzed. Each had H&E stained diagnostic slides with pathologist-annotated tumor regions, as well as deep whole-transcriptomic sequencing (>200M reads). Deep neural networks previously trained on TCGA slide images were used to generate digital spatial masks for 3 characteristics: tumor-content, lymphocytes, and stroma. Patients were scored based on the presence of intratumoral lymphocytes (iTIL) and stromal lymphocytes (sTILs). Immune subpopulations were inferred from RNAseq expression of published immune-cell-specific genesets [1,2], as was Wnt-signaling level [3]. Significant associations between immune subpopulations and level of infiltration were analyzed.

**Results**

Manually annotated positive tumor regions were accurately digitally masked as >83% tumor or lymphocyte. Wnt signaling was strongly associated with overall stromal content (Rho=0.47, p<0.0001). Strong anti-correlation was observed between levels of sTILs and iTILs (Rho=-0.42, p<0.0001), and remained significant when including overall stroma area as a covariate. Digital lymphocyte masks somewhat correlated with RNAseq-based deconvolution of lymphocyte classes (Rho=0.30, p=0.0001) in line with reports from others [4], however this decreased when comparing lymphocyte count within annotated tumor regions only (Rho=0.17, p=0.03), despite high concordance of lymphocyte counts within and outside of annotated regions overall (Rho=0.82, p<0.0001). RNAseq-based lymphocyte levels were more associated with sTILs than iTILs (Rho=0.19 vs. -0.28, p<0.01 respectively).

**Conclusions**

Adaptive response effectors such as NK and T-cells are found more resident in surrounding stromal tissue than infiltrating tumor tissue. Increased Wnt/B-catenin signaling in stromal regions, reported by others as immunosuppressive, may sequester immune effectors and aid in immune escape.

**References**

1. Bindea G, et al. Spatiotemporal dynamics of intratumoral immune cells reveal the immune landscape in human cancer. Immunity 2013; 39:782-795.

2. Danaher P, et al. Gene expression markers of tumor infiltrating leukocytes. J Immunother Cancer. 2017; 5:18.

3. Slattery ML,et al. Expression of Wnt-signaling pathway genes and their associations with miRNAs in colorectal cancer. Oncotarget. 2018; 9:6075.

4. Pai SG, et al. Wnt/beta-catenin pathway: modulating anticancer immune response. J Hematol Oncol. 2017; 10:101.

#### P61 Segmentation and classification of single cells using multiplexed ion beam imaging

##### Jay Tarolli, Rachel Finck, Murat Aksoy, Yari Sigal, Noah Newgren, Jessica Finn, Jason Ptacek, PhD

###### IONpath, Menlo Park, CA, United States

####### **Correspondence:** Jay Tarolli (jay@ionpath.com)

**Background**

When studying the tumor microenvironment, knowing not only the types of immune cells present but also the spatial distribution and relationship of these immune cells to other immune and tumor cells provides crucial information. In the past, techniques used to analyze these spatial relationships have been limited by the number of biomarkers that could be simultaneously measured. Recently, with the development of multiplexed ion beam imaging (MIBI), 40+ biomarkers can be simultaneously measured in a single scan [1]. By probing with an ion beam, tissue sections can be imaged at a spatial resolution on the same order of magnitude as light based techniques, providing subcellular resolution. This combination of multiplexed biomarker measurements and subcellular spatial resolution enables segmentation of the image into individual cells, making possible subsequent cell type classification and quantification.

**Methods**

Samples of placenta, lung, tonsil, lymph node, thymus, and liver were imaged with MIBI. Segmentation of these images was performed in two steps. First, a MaskRCNN [2] model was trained to utilize multiplexed MIBI data to predict the location of cell instances in a MIBI image for a single class of objects by learning features from a set of nuclear, cytoplasmic, and membrane markers. The centroids of each predicted cell instance were used as seed points and, after manual refinement of these seed points, watershed segmentation was performed to determine boundaries between instances. Both the summed intensity of a marker as well as a weighted cell score which accounts for the spatial distribution of a marker’s expression throughout a cell instance were calculated and were used for cell type classification.

**Results**

Cell population and densities were calculated for a number of different cell types, including T cells, B cells, and macrophages based on a combination of one or more coexpressed biomarkers present within segmented cells. Figure 1 shows an example FOV with several cell types classified in a single image. Expression of immunoregulatory proteins including PD-1 and PD-L1 were quantified and assigned to specific cell types. Finally, nearest neighbor distances between various cell types were determined to characterize the spatial organization of cell populations within each tissue image.

**Conclusions**

The ability to characterize the many different cell types within the tumor microenvironment is made possible by the highly multiplexed nature of MIBI data, the subcellular spatial resolution of the image data, and downstream analysis tools, including computer vision approaches, which enable cell segmentation, classification, and spatial analysis.

**References**

1. Keren L, Bosse M, Marquez D, Angoshtari R, Jain S, Varma S, Yang S, Kurian A, Van Valen D, West R, Bendall S, Angelo M. A Structured Tumor-Immune Microenvironment in Triple Negative Breast Cancer Revealed by Multiplexed Ion Beam Imaging. Cell. 2018; 174:1373-1387.

2. He K, Gkioxari G, Dollár P, Girshick R. Mask R-CNN. IEEE International Conference on Computer Vision (ICCV). 2017; 2961-2969.

**Fig. 1 (abstract P61).**
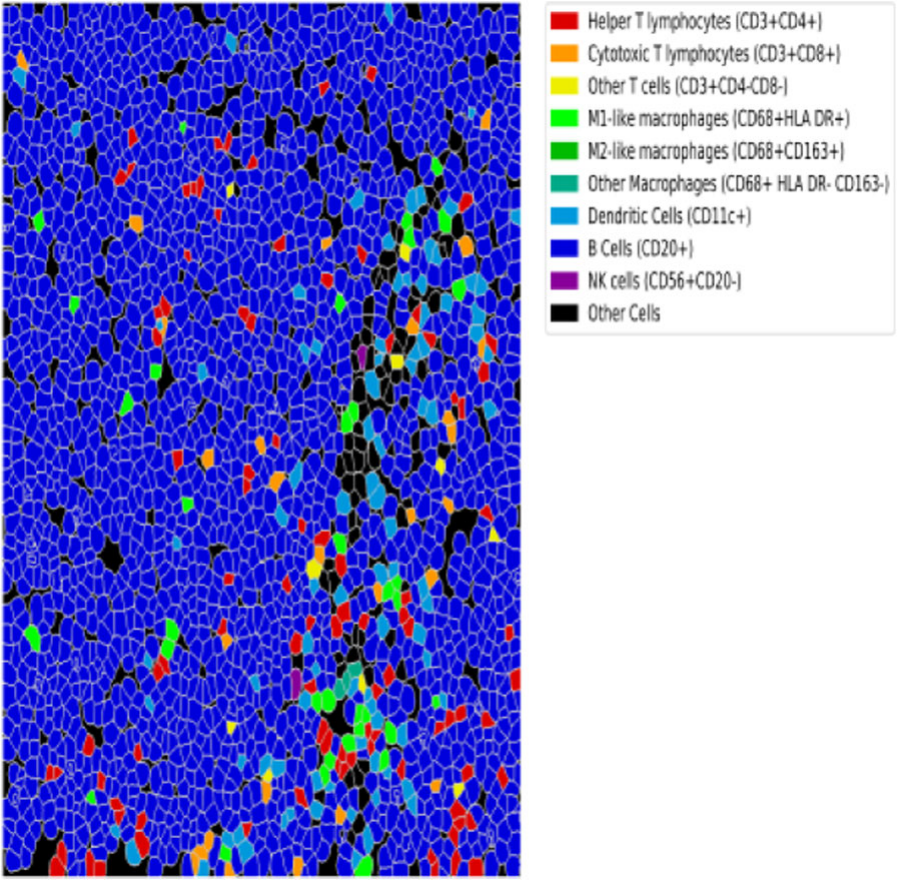
See text for description

#### P62 Bringing the tumor microenvironment into focus: Simplified development of seven-color multiplex immunohistochemistry-immunofluorescence (mIF) panels

##### Melissa Whiteman, PhD, Eric McIntush, PhD, Mike Spencer

###### Bethyl Laboratories, Montgomery, TX, United States

####### **Correspondence:** Mike Spencer (mspencer@bethyl.com)

**Background**

For the advancement of immunotherapeutics, the need to understand the tumor microenvironment has never been more pressing. Recent advances in mIF and multispectral imaging facilitate accurate simultaneous analysis of multiple tissue markers. This is critical in instances where sample is limited, such as a tumor biopsy or other clinical specimen. The applications of mIF are numerous, and span clinical, translational, and basic research applications. A seven-color mIF can take eight weeks, or more, to develop. Herein, we describe a simplified, faster approach.

**Methods**

FFPE human tissue was stained with PathPlex™ Panel 4 IHC validated primary antibodies (Bethyl Laboratories [A810-004]), mouse or rabbit HRP-conjugated secondary antibodies (Bethyl Laboratories [A90-116P, A120-501P]) and detected using Opal™ Polaris 7-color IHC kit fluorophores (Perkin Elmer [NEL861001KT]). Primary antibody order was optimized utilizing tissue microarray serial sections, and three slides per target by staining after the first, third, or sixth heat-induced epitope retrieval (HIER). All three slides were imaged using the same exposure time and analyzed for target/nucleus counts, signal intensity, and background. Finally, the order was tested in the seven-color mIF and compared to single stain for confirmation. Whole slide scans were generated using the Vectra Polaris® and analyzed using InForm® image analysis package.

**Results**

Development time of a seven-color mIF was reduced using IHC validated antibodies and the optimized dilution. Antibody order was guided by results of three slides stained after first, third or sixth HEIR. The ratio of target staining/DAPI nuclear counts, average intensity and overall background predicts the optimal order of staining. Some targets reveal larger average area staining, higher intensity and lower background when stained last, for example FOXP3 (Table 1, Figure 1), while the inverse may be true, or no effect for other targets. There are 720 possible combinations for a seven-color panel. Using this method, the number of slides was reduced to three per target (18) plus confirmation seven-color slides resulting in a panel containing CD3, CD8, CD68, Cytokeratin, FOXP3 and PD-L1 (Figure 2).

**Conclusions**

Multiplex IF is a powerful technique that allows for examination of spatial arrangement of proteins of interest as well as protein interaction/co-localization of multiple targets within a single tissue specimen. MIF panels can take eight or more weeks to optimize, however, researchers can save time and resources using validated antibodies and this antibody order guide.

**Fig. 1 (abstract P62).**
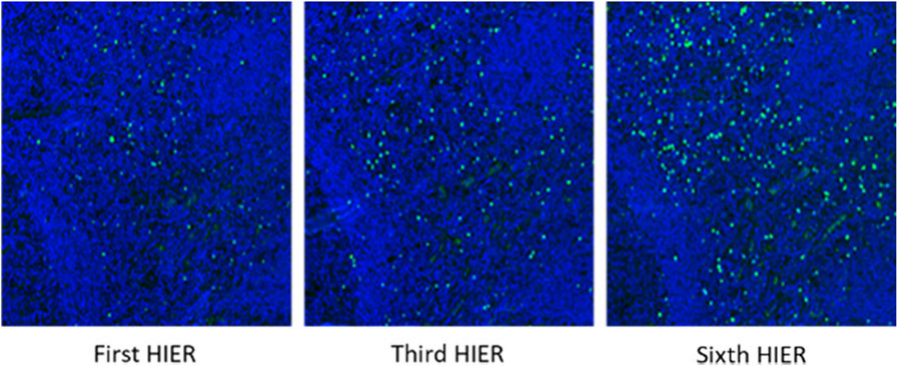
See text for description

**Table 1 (abstract P62).**
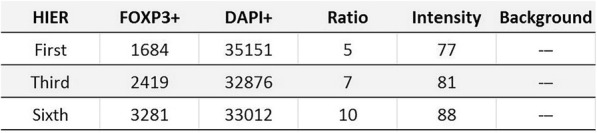
See text for description

**Fig. 2 (abstract P62).**
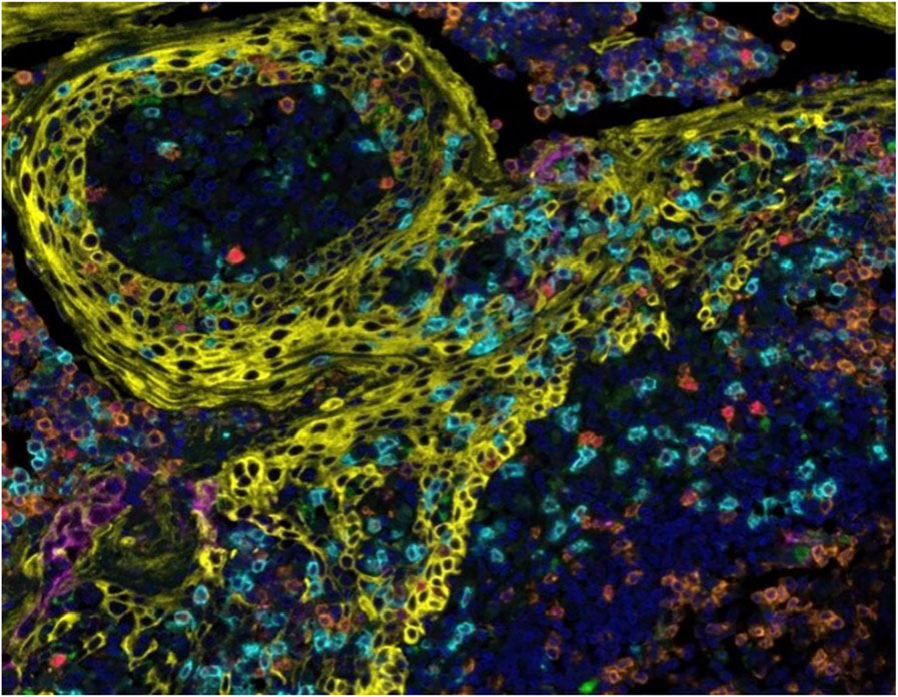
See text for description

#### P63 Clinical assay development and validation of multiplex immunofluorescent (mIHC) marker panel for evaluation of tumor infiltration myeloid cells in FFPE tissue sections

##### Lan Yi, PhD^1^, Jonathan Juco, MD^1^, Ashhad Mahmood, MD^2^, Omar Laterza^1^, Charo Garrido^1^, Lan Yi, PhD^1^

###### ^1^Merck & Co., Inc, Hillsborough, NJ, United States; ^2^Diagnostic Pathology Services, LLC, Skillman, NJ, United States

####### **Correspondence:** Omar Laterza (omar_laterza@merck.com); Charo Garrido (charo.garrido@merck.com)

**Background**

Myeloid-derived suppressor cells (MDSCs) are a group of leucocytes with myeloid origin and immune-suppressive function. Ample recent evidence supports key contributions of MDSC to tumor progression through immune-mediated mechanisms. MDSCs include two major subsets based on their phenotypic and morphological features: polymorphonuclear (PMN)-MDSC and monocytic (M)-MDSC. However, these cells remain less studied than T lymphocytes as their phenotypical, morphological and functional heterogeneity generate confusion in investigation and analysis of their role.

**Methods**

With the progresses on multiplex IHC assay technology, we are now able to develop multiplex immunofluorescent marker panels to evaluate the expression and localization of the main subpopulations of myeloid cells in the tumor microenvironment.

**Results**

We have developed and validated CD14, CD66b, CD163 and MHCII (HLA-DR) IHC multiplex marker panel to evaluate the main populations of myeloid cells, along with their activation status. After completing the validation for individual markers in a single chromogenic IHC platform, we optimized the incorporation of each marker into the multiplex platform. In parallel, a multiplex image analysis algorithm (APP) was generated and validated to quantify each subpopulation of myeloid cells in the tumor area. Last, fit for purpose analytical validation, including sensitivity, specificity and precision was successfully carried out.

**Conclusions**

This validated assay is currently being used to support multiple ongoing and future clinical trials.

#### P64 Highly multiplexed single-cell spatial analysis of FFPE tumor tissues using CODEX®

##### Jessica Yuan, PhD, Gajalakshmi Dakshinamoorthy, Joseph Kim, Sejal Mistry, Nadya Nikulina, Roya Bashier, Cassandra Hempel, Maria Elena Gallina, Julia Kennedy-Darling, Jessica Yuan, PhD

###### Akoya Biosciences, Menlo Park, CA, United States

####### **Correspondence:** Julia Kennedy-Darling (j.kennedy@akoyabio.com)

**Background**

Characterizing the complexities of the tumor microenvironment is elemental to understanding disease mechanisms. The spatial relationships between infiltrating immune cells and the remodeling of the cellular matrix is widely recognized as a key component to defining tumor heterogeneity. Current methodologies for analyzing the spatial dimension in tissues, like traditional immunofluorescence (IF) and immunohistochemistry (IHC), are limited to a few parameters at a time, restricting the scope of identifiable cells. Conversely, single-cell technologies like mass cytometry and NGS-based tools provide multiplexing capabilities, but at the expense of the associated spatial information. Here, we present the analysis of human lung cancer FFPE tissues with CODEX using a panel of more than 20 markers targeting the tumor microenvironment.

**Methods**

The CODEX technology, developed by Akoya, is comprised of a fluidics instrument that interfaces with existing microscope hardware, as well as a suite of reagents and associated control and analysis software. The CODEX technology involves labeling antibodies with oligonucleotide-based Barcodes followed by a single staining step. Around 40 parameters can be measured within a single tissue through fully-automated, iterative cycles of adding and removing corresponding dye-conjugated Reporters. Here, we apply this technology using a panel of antibodies targeting immune, cancer and other architectural features to measure cell subsets in cancer FFPE tissues. Image data is processed using the CODEX analysis pipeline, including clustering, annotation and mapping of cell types to the original image data with the Multiplexed Analysis Viewer (MAV).

**Results**

The CODEX technology was used to ascertain complex cellular niches and spatial associations between multiple cell types based on the staining pattern of more than 20 parameters. The high-parameter antibody panel used was optimized with human FFPE tonsil tissues. Human lung cancer FFPE tissues were then analyzed with this same panel. Cell clustering using the MAV software identified tens of cell types within the tumor tissues. Immune cell sub-types were mapped onto the original image data to assess infiltration and spatial associations.

**Conclusions**

Unlike other cyclic IF approaches involving multiple antibody staining and stripping steps, the CODEX platform involves a single initial staining step and subsequent gentle and relatively fast manipulation of the tissue thereafter. This provides a superior workflow and prevents tissue degradation. CODEX data from various normal and cancer human FFPE tissue types is shown here with corresponding single-cell analysis of key tissue features. Overall, the CODEX platform is an accessible and versatile technology for high parameter, spatial profiling of tissue specimens.

#### P65 Mutation-targeted T cell responses in blood from patients with solid tumors prior to treatment and which evolve with clinical benefit from anti-PD-1 therapies

##### Benjamin Yuen, PhD^1^, Fangfang Yin, PhD^2^, Duo An, PhD^1^, Boi Quach^1^, Linlin Guo, PhD^1^, Joanne Tan, PhD^2^, Songming Peng, PhD^1^, Zheng Pan, PhD^1^, Olivier Dalmas, PhD^1^, Robert Bao, PhD^1^, Kyle Jacoby, PhD^1^, Barbara Sennino, PhD^1^, Stefanie Mandl, PhD^1^, Matt Walters, PhD^2^, Juan Jaen, PhD^2^, Alex Franzusoff, PhD^1^, Benjamin Yuen, PhD^1^

###### ^1^PACT Pharma, South San Francisco, CA, United States; ^2^Arcus Biosciences, Hayward, CA, United States

####### **Correspondence:** Songming Peng (speng@pactpharma.com)

**Background**

T cells targeting tumor-exclusive neoepitopes (neoE) have been postulated to represent the primary mediators of clinical benefit for patients with solid tumors treated with immunotherapies. Identifying and tracking these T cells in patients can help to understand the mechanism for immune checkpoint inhibitor therapies, as well as provide new therapeutic candidates for personalized adoptive cell therapies. However, this has been hampered by the low frequency of neoE-specific T cells in peripheral blood. To this end, we demonstrate the use of the imPACT Isolation Technology®, an ultra-sensitive high-throughput technology, to capture neoE-specific CD8 T (neoE-T) cells from peripheral blood. In addition, this technology can be utilized to quantify and monitor neoE-T cells longitudinally during therapy. We show here preliminary data applying the imPACT technology to clinical trial samples for the characterization of mutation-targeted T cell responses from patients associated with clinical benefit.

**Methods**

Peripheral blood mononuclear cells (PBMC) from patients with non-small cell lung cancer (NSCLC) and treated with combinations containing an anti-PD-1 antibody were analyzed. Briefly, tumor-exclusive neoE-HLA target candidates were predicted and barcoded snare libraries comprising personalized neoE-HLA reagents were produced for capture of neoE-specific CD8+ T cells from PBMCs. Longitudinal analysis of neoE-T cells responses throughout the duration of treatment was performed to obtain valuable information on neoTCR sequences and neoE-T cell quantification & phenotype.

**Results**

A baseline neoE-specific CD8 T cell profile was identified in all subjects prior to treatment. Among NSCLC subjects exhibiting objective responses to therapy, some neoE-T cell clones identified at baseline persist in the blood and/or diversify in clonality over the course of treatment. In some circumstances, new neoE-T cell clones have emerged on treatment with anti-PD-1. Furthermore, phenotype analysis suggested the neoE-T cells captured from blood have been activated, indicating previous encounter with their respective neoE-HLA targets.

**Conclusions**

The imPACT technology was used to assess the phenotype & quantity of neoE-specific T cells in blood of trial participants over time. This approach revealed the evolution of mutation-targeted T cell responses in participants with clinical benefit and may prove to be a powerful tool to provide mechanistic understanding of immune responses associated with clinical benefit. These data support further testing of the neoE-T cell capture technology, with the potential to uncover the identity of neoE-specific T cells pre-existing in the blood of patients and the evolution of immune attack to cancer.

**Ethics Approval**

The study was approved by the institutional review boards or ethics committees of the participating sites in Arcus Biosciences’ clinical studies.

#### P66 A novel mass cytometry-based immunomonitoring platform for characterizing the peptide vaccine-induced immune response of HLA-A*0201+ patients with K27M+ diffuse midline gliomas

##### Jared Taitt, BA^1^, Payal Watchmaker, PhD^1^, Takahide Nejo, MD, PhD^1^, Neil Almeida^2^, Kaori Okada^1^, Sabine Mueller, MD PhD^1^, Hideho Okada, MD, PhD^1^, Jared Taitt, BA^1^

###### ^1^University Of California, San Francisco, San Francisco, CA, United States; ^2^George Washington University, Washington DC, United States

####### **Correspondence:** Sabine Mueller (sabine.mueller@ucsf.edu); Hideho Okada (hideho.okada@ucsf.edu)

**Background**

Diffuse midline glioma, including diffuse intrinsic pontine glioma (DIPG) constitutes up to 20% of pediatric brain cancer and has a median survival of 9-10 months. Given the proximity of DIPG to parenchymal regions that play vital homeostatic functions, surgical resections are often restricted in size and scope, leaving irradiation and chemotherapy as the primary management options. The ongoing development of immunotherapy has shown significant promise in many fields, including that of gliomas. Genetic studies revealed that greater than 70% of DIPG cases harbor an amino acid substitution from lysine (K) to methionine (M) at position 27 of histone 3 variant 3 (H3.3). We previously identified a novel HLA-A*02:01-restricted neoantigen epitope encompassing the H3.3K27M mutation. Accordingly, we have implemented a pilot vaccine through the Pacific Pediatric Neuro-Oncology Consortium (PNOC).

**Methods**

Twenty-nine newly diagnosed DIPG patients who are HLA-A2+ and H3.3K27M+ underwent radiation therapy, and then received the H3.3K27M peptide vaccine and tetanus toxoid (TT) peptide emulsified in Montanide in combination with poly-ICLC every 3 weeks for a total of 24 weeks. Our objective is to characterize vaccine-induced H3.3K27M-specific CD8+ T-cell and myeloid-derived immunosuppressive subpopulations in peripheral blood mononuclear cells utilizing a novel H3.3K27M-specific dextramer-based mass cytometry (CyTOF) method [1,2].

**Results**

Through this approach, the temporal expansion of vaccine-reactive CD8+ T-cells was observed in all patients who completed a minimum of 24 weeks on the study (n = 4). Simultaneously, this expansion was not observed in 4 of 5 patients who withdrew from the regimen due to progression. These T-cells were clustered on a tSNE plot using canonical CD8+ T-cell activation markers and further classified by their expression profiles, revealing distinct effector memory, central memory and transitional effector subpopulations. Chronological monitoring of these groups indicates the time course-dependent development and persistence of vaccine-reactive exhausted and effector memory CD8+ T-cells in 3 of the 4 initial patients analyzed. Furthermore, an analogous clustering and phenotyping approach was used for myeloid cells, allowing for the identification of myeloid-derived suppressor cell (MDSC) subpopulations. A comparative analysis revealed a positive correlation between two monocytic myeloid-derived suppressor cell (M-MDSC) subpopulations and progression-free survival.

**Conclusions**

Future plans include analyzing the remainder of patients enrolled in the trial and the utilization of CD8+ and MDSC-specific CyTOF panels to further classify the aforementioned subpopulations to further elucidate this relationship. This methodology offers insight into the progression of vaccine-induced patient immune responses and exhibits promise as a platform that may be extrapolated to other immunotherapies.

**References**

1. Louis DN, Perry A, Reifenberger G, von Deimling A, Figarella-Branger D, Cavenee WK, Ohgaki H, Wiestler OD, Kleihues P, Ellison DW. The 2016 World Health Organization Classification of Tumors of the Central Nervous System: A summary. Acta Neuropathol. 2016; 131:803–820.

2. Chheda Z, Kohanbash G, Okada K, Jahan N, Sidney J, Pecoraro M, Yang X, Carrera D, Downey K, Shrivastav S, Liu S, Lin Y, Lagisetti C, Chuntova P, Watchmaker P, Mueller S, Pollack I, Rajalingam R, Carcaboso A, Mann M, Sette A, Garcia C, Hou Y, Okada H. Novel and shared neoantigen derived from histone 3 variant H3.3K27M mutation for glioma T cell therapy. J Exp Med. 2017; 215:141-157.

**Ethics Approval**

The study was approved by UCSF IRB #: 16-20574

#### P67 Use of a regional integrated health record data network to identify patients who received checkpoint therapy following cancer diagnosis as a foundation for exploring immunotoxic events

##### Theresa Walunas, PhD, Carlos Galvez, Saya Jacob, Jeffrey Sosman, MD, Abel Kho

###### Northwestern University, Chicago, IL, United States

####### **Correspondence:** Theresa Walunas (t-walunas@northwestern.edu)

**Background**

Immune related adverse events (irAE) occur in >80% of patients receiving immune checkpoint inhibitors (ICI). Currently, most data about the incidence of irAE comes from clinical trials with restrictive eligibility requirements. With the wide use of ICI therapy as standard of care for many cancers, it is important to assess incidence of irAE in a general patient population. The Chicago Area Patient Reported Outcomes Research Network (CAPriCORN) is a clinical data research network containing medical records for >9.5M patients who receive care in 11 institutions spanning diverse patient populations and healthcare settings [1]. Using CAPriCORN, we wanted to determine whether we could identify a large, diverse cohort of patients who received ICIs as a foundation for exploring the incidence of irAE in a real-world data source.

**Methods**

We identified all patients within CAPriCORN who were 19-88 years old, had a diagnosis for an ICI-approved cancer, and received an ICI from 1/1/2011 through 12/31/2018. Clinical experts identified the International Classification of Disease 9 and 10 codes used to document cancer diagnosis and the RxNorm [2] codes for each ICI documented as a medication ordered in the medical record (Table 1). The query was developed against the PCORnet Common Data Model version 4.1 [3], validated on the Northwestern University site node in CAPriCORN and distributed to all CAPriCORN sites. Six of 9 sites returned counts. Data was centrally aggregated and stratified by age, race, sex and therapy. All data are aggregated counts.

**Results**

As shown in Table 2, we identified 6,541 patients within CAPriCORN who received ICI therapy for cancer. 45% are female, 75% identify as white, 13% African American, 2% Asian and 1% Native American, and 86% are 51-83 years of age. The most well represented cancers were Non-small Cell Lung Carcinoma (50%) and Metastatic Melanoma (18%). Overall, 67% received anti-PD1 therapies, followed by combination ICI therapies, anti-PDL1 and anti-CTLA4, though usage varied within cancer types.

**Conclusions**

Our results demonstrate that a large cohort of cancer patients who have received ICI therapy can be identified in an integrated medical record data environment that spans 11 institutions in a major urban center. This population is racially diverse, represents both sexes, a wide range of ages and includes all cancer types approved for ICI therapy as of 2018. This real-world cohort will be an effective foundation on which to explore the incidence of irAE, particularly rare irAE that require large sample sizes to investigate.

**Acknowledgements**

The investigators would like to acknowledge the CAPriCORN network for query support. This work was supported by Patient Centered Outcomes Research Institute (PCORI) CDRN-1306-04737.

**References**

1. Kho AN, Hynes DM, Goel S, et al. CAPriCORN: Chicago Area Patient-Centered Outcomes Research Network. J. Am Med Inform Assoc. 2014; 21:607-611.

2. https://pcornet.org/download/pcornet-common-data-model-v4-1-specification-5-may-2018/?wpdmdl=1919&refresh=5d42596fab3e11564629359

3. https://www.nlm.nih.gov/research/umls/rxnorm/

**Ethics Approval**

The study was approved under the CAPriCORN IRB, CHAIRb: Research Protocol #14120201 “CAPriCORN Clinical Data Research Network Master Protocol”.

**Table 1 (abstract P67).**
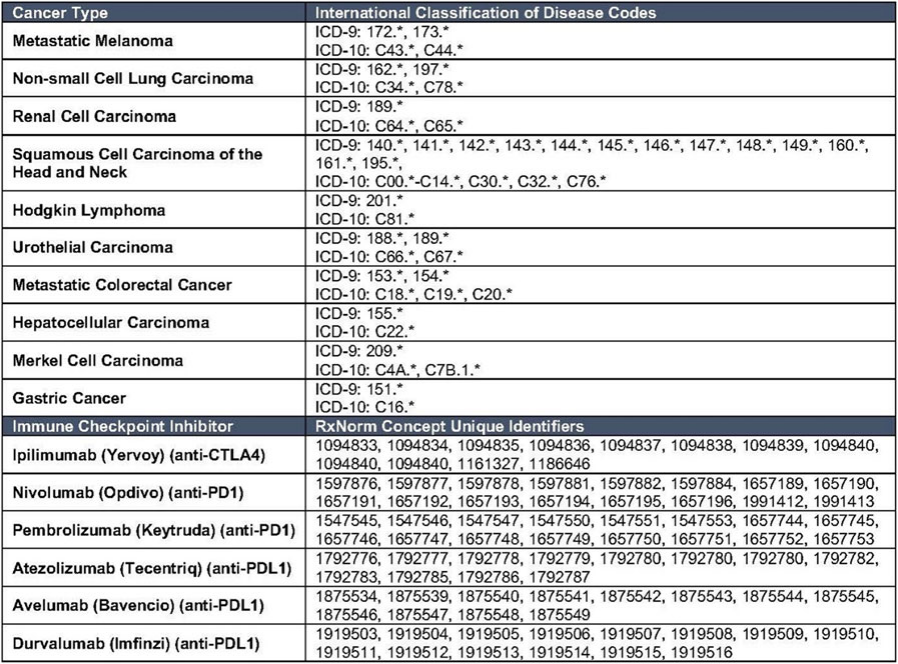
See text for description

**Table 2 (abstract P67).**
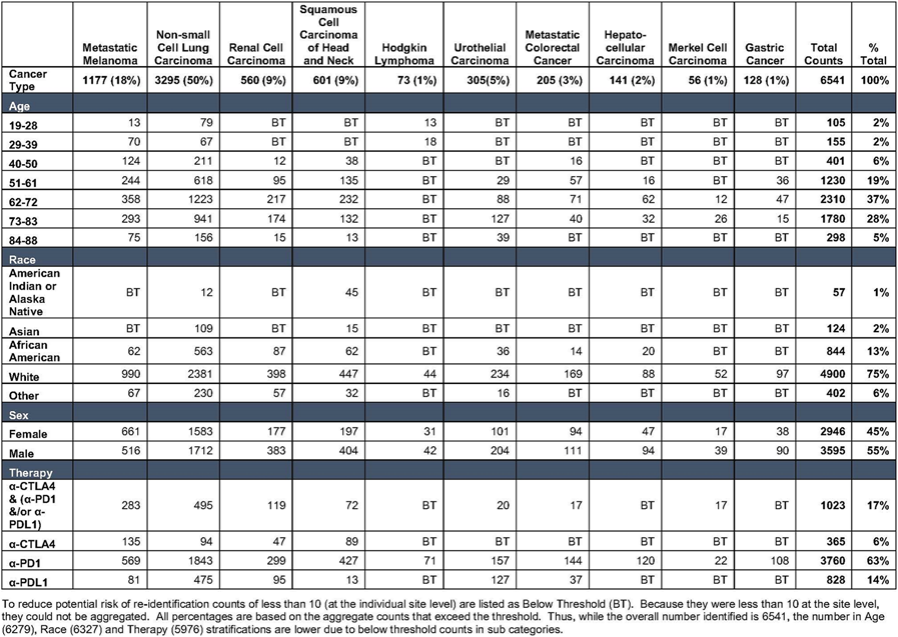
See text for description

#### P68 Innate inflammatory pathways are associated with TIL growth and response to adoptive immunotherapy

##### Suhendan Ekmekcioglu, PhD^1^, Dai Ogata, MD, PhD^1^, Caitlin Creasy, MS^1^, Marie Forget, PhD^1^, Sun-Hee Kim, PhD^1^, Jason Roszik, PhD^1^, Mike Spencer^3^, Patrick Hwu, MD^1^, Elizabeth Grimm, PhD^1^, Chantale Bernatchez, PhD^1^, Suhendan Ekmekcioglu, PhD^1^

###### ^1^The Univeristy of Texax MD Anderson Cancer Center, Houston, TX, United States; ^2^Bethyl Laboratories, Inc, Montgomery, TX, United States

####### **Correspondence:** Suhendan Ekmekcioglu (sekmekcioglu@mdanderson.org); Chantale Bernatchez (cbarnatchez@mdanderson.org)

**Background**

Immune infiltration of T cells (TIL) into the melanoma microenvironment has been associated with improved survival for some patients, and also has been exploited to grow TIL in vitro for adoptive therapy. However, prognostic significance of immune infiltrating cells in melanoma and other tumors remains a relatively new concept, and markers related to suppressive versus active functional TIL remain unclear. We previously reported that in Stage III melanoma patients’ tumors, positive expression of CD74 together with low or absent Macrophage Migration Inhibitory Factor (MIF) associates with favorable prognosis [1].

**Methods**

From an ongoing clinical trial using TIL intended for adoptive immunotherapy, we have studied the melanoma patient tumors specimens (FFPE) from 20 patients whose autologous TIL lines grew to sufficient number for possible use clinically. We also examined another 20 sets of melanoma tumor from which the TIL did not grow or not grow well. We analyzed the differences in the two groups of tumors (40 total FFPE) for CD74 regulated pathway features and inflammatory marker expression.

**Results**

CD74 regulated markers included CD44, MIF, and downstream inflammatory targets including inducible Nitric Oxide Synthase (iNOS) and Nitrotyrosine (NT). Our findings confirm our previous report in that tumor CD74 expression significantly associates with favorable OS and PFS (both, p=0.0038) and provides new data that in this set of patients the CD74 also correlates with best irRC of TIL treated patients. New findings include that the NT expression in tumor cells associated with poor TIL growth (p=0.014), as well as lack of clinical response to TIL treatment (p=0.02). We have also found that tumor cell-derived MIF and iNOS expression correlate with unfavorable prognosis for both OS and PFS (p=0.016 and 0.018, respectively).

**Conclusions**

We have identified the protein expression of CD74, MIF and of iNOS as providing survival information, and proposed that CD74+/MIF-/iNOS- together be considered to form a "signature" of good prognosis in general melanoma outcomes as well as TIL growth and favorable responses for these patients. Use of this signature for selecting patients for entry into TIL and possibly other immunotherapy trials, as well as research on the differential pathways of IFN-γ signaling in melanoma appear as important areas for future mechanistic research to improve patient outcome.

**Reference**

1. Ekmekcioglu S, Davies MA, Tanese K, Roszik J, Shin-Sim M, Bassett RL Jr, Milton DR, Woodman SE, Prieto VG, Gershenwald JE, Morton DL, Hoon DS, Grimm EA.Inflammatory Marker Testing Identifies CD74 Expression in Melanoma Tumor Cells, and Its Expression Associates with Favorable Survival for Stage III Melanoma. Clin Cancer Res. 2016 Jun 15;22(12):3016-24.

#### P69 Highly multiplexed single cell spatial analysis of the tumor microenvironment in lymphoma

##### Monirath Hav, MD, PhD^1^, Anthony Colombo^1^, Erik Gerdtsson^2^, Mohan Singh^2^, Denaly Chen^2^, Imran Siddiqi, MD PhD^2^, James Hicks^2^, Peter Kuhn, PhD^2^, Akil Merchant, MD^1^, Akil Merchant, MD^1^

###### ^1^Cedars Sinai Medical Center, Los Angeles, CA, United States; ^2^University of Southern California, Los Angeles, CA, United States

####### **Correspondence:** Akil Merchant (akil.merchant@cshs.org)

**Background**

Diffuse large B cell lymphoma (DLBCL) being the most subtype of non-Hodgkin lymphoma. Despite evidence of expression of PDL-1 on lymphoma cells, less than 10% of DLBCL patients respond to PD1 therapy [1]. We hypothesize that a better characterization of spatial architecture of the tumour microenvironment (TME) in lymphoma will help explain differences in responses to PD1/PDL-1 inhibitors and guide future targeted immunotherapies for these patients.

**Methods**

Here we characterized the TME in DLBCL using imaging mass cytometry (IMC), which allows high-dimensional, single-cell and spatial analysis of FFPE tissues at sub-cellular resolution [2]. Using a panel of 32 antibodies, IMC was performed 41 tissue microarray cores from 33 DLBCL cases. IMC images were analyzed for relevant immunophenotypes, the spatial architecture of those phenotypes and compared to clinical outcomes to identify immune contexture based biomarkers.

**Results**

Phenograph was used to cluster tumor and immune cells based on phenotype (Figure 1A). Immune cell represented 33% of the cells represented by CD4 (36%), CD8 (30%), macrophages (26%) and TREG (8%) (Figure 1B). Immune cell infiltration in individual tumor samples ranged from 7% to 75% with marked heterogeneity. (Figure 1C-D. Analysis of immune marker expression on tumor cells identified co-expression of PD-L1/CCR4/TIM3 to be highly prognostic for overall survival (p=0.003, Figure 1E)

To characterize the patterns of spatial interaction in the TME, we developed an unsupervised multivariate model to construct spatial meta-clusters based on average distances from CD8 to the centroids of 5 nearest endothelial cells, TREG, CD4 T cells, macrophages, and tumor cells (Figure 2A). Spatial analysis revealed 11 meta-clusters for CD8 T cell interactions (Figure 2B). Meta-clusters 2, 6, 8 and 11 were the 4 most dominant patterns of CD8 spatial interaction in the TME. Each CD8 spatial interaction pattern is distinctive with case to case heterogeneity (Figures 2C-D). Risk assessment analyses of spatial clusters 1, 2 and 4 (“hazardous”) had almost 3 times higher odds of being identified in refractory cases compared to clusters 3, 5 and 6 (“protective”) (Figure 2E). In the “protective” spatial neighborhoods, we observed the presence of activated CD8, Th1-like CD4, and less suppressive TREG phenotypes, with opposite in “hazardous” areas (Figures 3A-B). TIM-3 expression was high both on T cells and tumor cells in the “hazardous” neighborhoods.

**Conclusions**

Our novel approach to spatial analysis of the immune architecture reveals clinically relevant insights into the TME.

**References**

1. Ansell, S. M. et al. Nivolumab for Relapsed/Refractory Diffuse Large B-Cell Lymphoma in Patients Ineligible for or Having Failed Autologous Transplantation: A Single-Arm, Phase II Study. J. Clin. Oncol. 37, 481–489 (2019).

2. Giesen, C. et al. highly multiplexed imaging of tumor tissues with subcellular resolution by mass cytometry. Nat. methods | 11, 417 (2014).

**Ethics Approval**

The study was approved by USC IRB, approval number HS10-260

**Fig. 1 (abstract P69).**
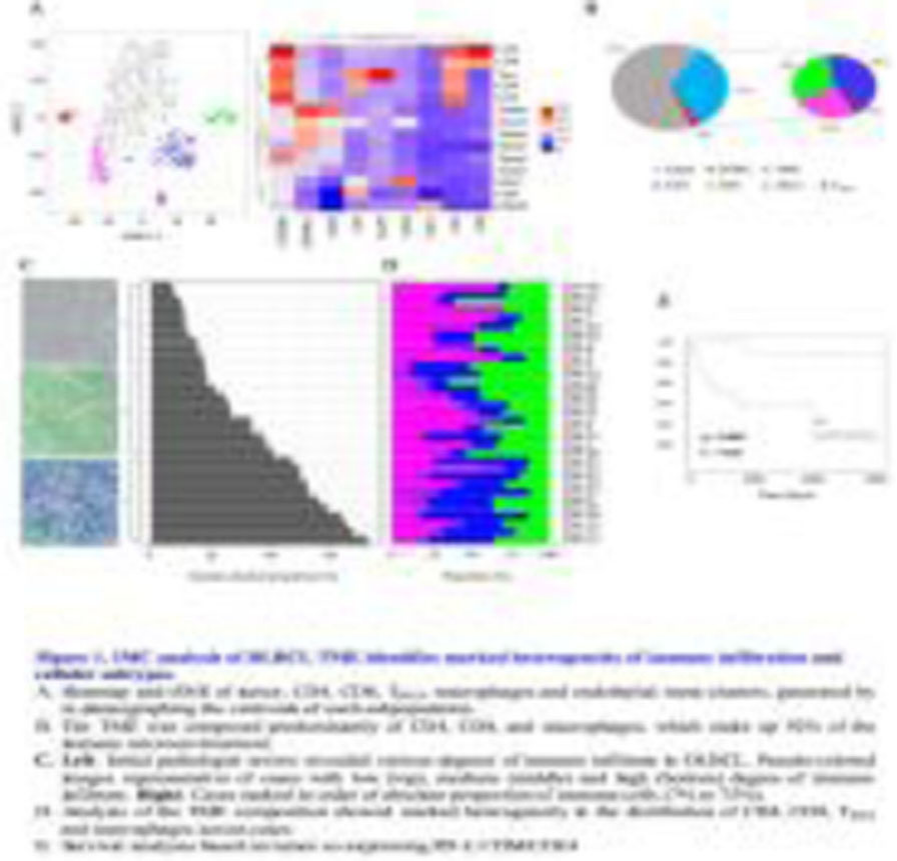
See text for description

**Fig. 2 (abstract P69).**
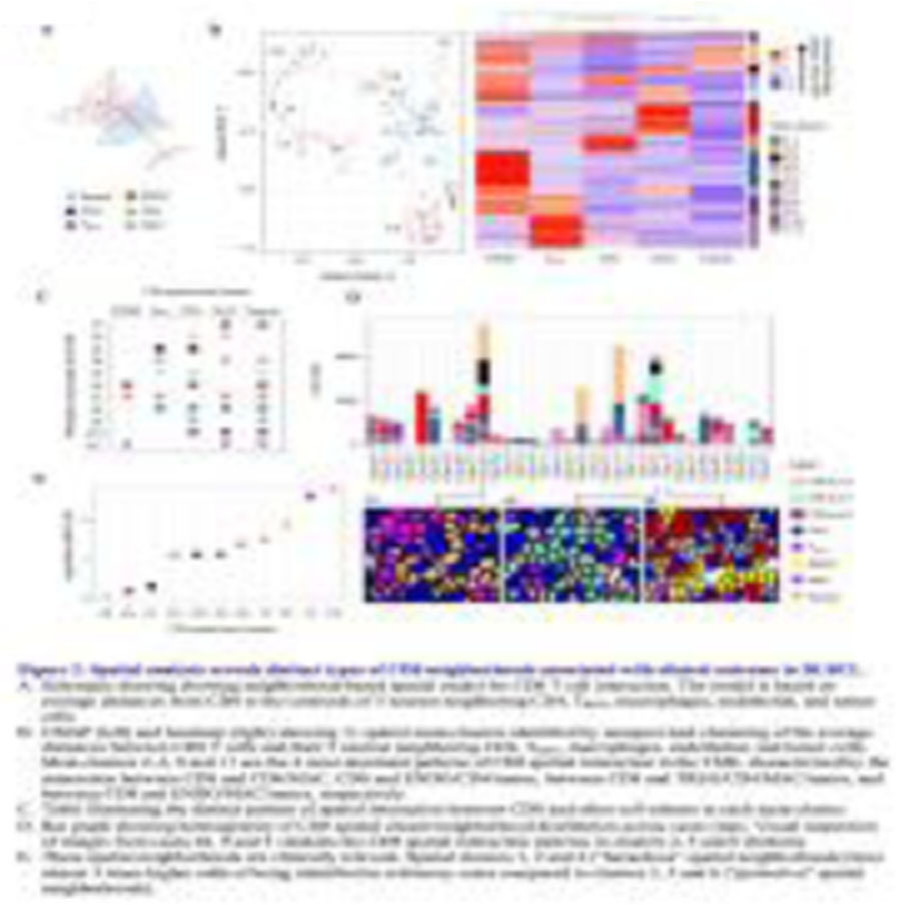
See text for description

**Fig. 3 (abstract P69).**
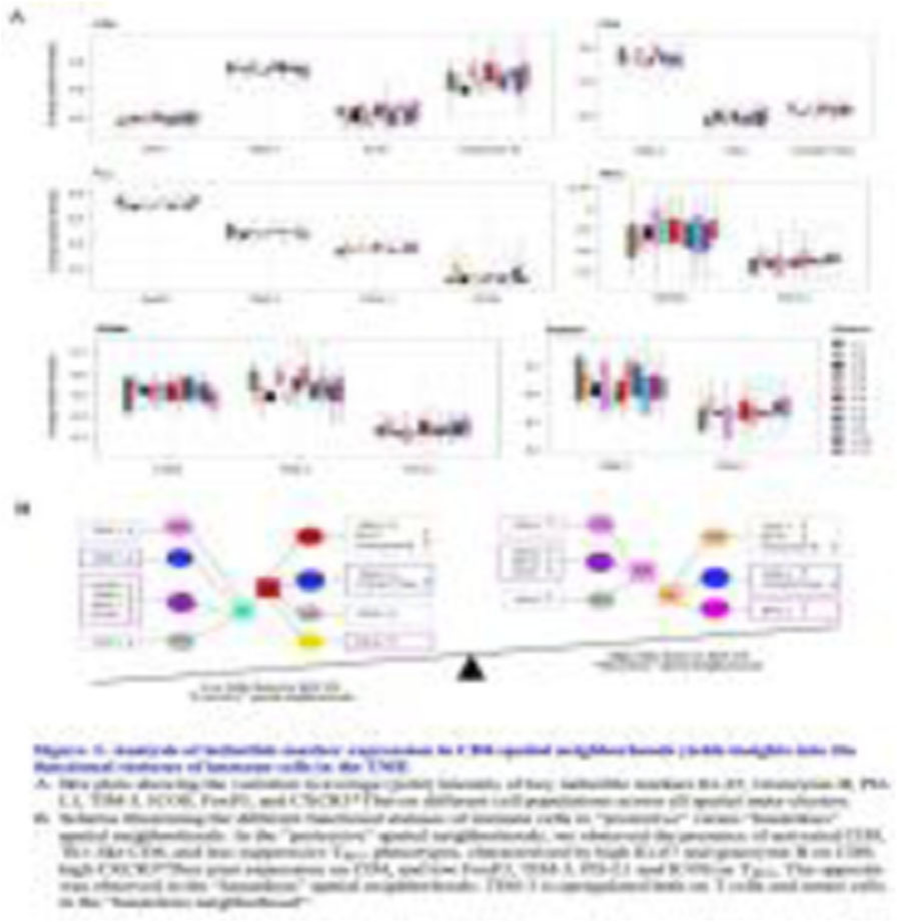
See text for description

#### P70 Tissue-based characterization of T cell exhaustion in inflammatory bowel disease and colorectal cancer using multiplex IHC

##### Marina Bleck, PhD, Diane Mierz^1^, Ania Mikucki^1^, Marie Marcher^1^, Sidharth Kerkar, MD^1^, Gerald Nabozny, PhD^1^, Sean Downing, PhD^2^, Alexander Klimowicz, PhD^1^, Alexander Klimowicz, PhD^1^

###### ^1^Boehringer Ingelheim, Ridgefield, CT, United States; ^2^Ultivue, Cambridge, MA, United States

####### **Correspondence:** Alexander Klimowicz (alexander.klimowicz@boehringer-ingelheim.com)

**Background**

T cell exhaustion and the PD-L1/PD-1 checkpoint axis has been extensively characterized in peripheral blood mononuclear cells and in human tumor tissues. This has provided a better understanding of the role this pathway plays in tumor immunology and of its clinical utility in predicting responsiveness to checkpoint inhibitor therapies. T cell exhaustion is not only associated with tumor progression, but has recently been associated with better prognosis and milder course of disease for a number of autoimmune and autoinflammatory disorders. We set out to characterize and contrast the T-cell exhaustion environment between colonic Crohn’s disease (CD) and colorectal cancer (CRC). We applied the Ultivue UltiMapper multiplex fluorescence IHC platform to capture complex immune cell phenotypes and provide a more in depth characterization than traditional IHC.

**Methods**

Commercially sourced FFPE surgical resections from n=5 colonic CD patients (matched lesional and non-lesional tissue) were compared to n=5 CRC tumor resections (3 hot and 2 cold tumors) using the Ultivue UltiMapper multiplex fluorescence immunohistochemistry platform. Two UltiMapper kits were used to evaluate the T cell environment in these tissues: UltiMapper I/O PD-L1 panel included the markers CD8, CD68, PD-L1, and pan-Cytokeratin/Sox10; UltiMapper I/O PD-1 panel included the markers CD3, CD45RO, PD-1, and pan-Cytokeratin/Sox10. All assays were stained on Leica BOND RX autostainers. Whole-slide images were acquired on a ZEISS Axio Scan.Z1 slide scanner. Image analysis was performed using Indica Labs HALO software.

**Results**

Contrasted to non-lesional CD tissues, several similarities were observed between CD lesional tissue and CRC, including the presence of PD-L1+ immunoreactivity in epithelial/tumor cells, increased immunoreactivity for PD-L1 in CD68+ cells, and a closer relationship between intra-epithelial and stromal CD8+ cells with PD-L1+ cells. In addition, areas in CRC and CD heavily infiltrated by immune cells or with tertiary lymphoid structures contained clusters of PD-L1+ cells that were negative for both CD68 and pan-Cytokeratin. Most of the CD3+ cells in non-lesional CD were PD-1 negative, except around tertiary lymphoid structures. In contrast, a greater percentage of CD3+ cells were also PD-1+ in CRC, and more so in lesional CD tissue.

**Conclusions**

The Ultivue UltiMapper multiplex fluorescence immunohistochemistry platform was effective in characterizing the PD-L1/PD-1 axis and T cell exhaustion environment in FFPE tissue, in part due to the ability to clearly identify more complex immune cell phenotypes than traditional multiplex IHC. The application of the UltiMapper assays demonstrated many similarities between marker and cell type distribution between lesional colonic CD and CRC.

#### P71 Refining tumor mutation burden values using variant expression

##### Shannon Bailey, PhD, Muhammad Ekram, PhD, Jim Lund, Jeffrey Gulcher

###### WuXi NextCODE Genomics, Arlington, MA, United States

####### **Correspondence:** Jeffrey Gulcher (jgulcher@wuxinetcode.com)

**Background**

Tumor mutation burden (TMB) is used as a surrogate marker for the neoantigen load of a tumor, and many studies have shown that TMB predicts the success of immune-oncology (IO) treatments for cancers, such as anti-PD-1 or anti-CTLA4 therapy. While IO treatment of patients with high TMB has led to success and excitement in the field, not all high TMB patients respond to IO treatment. TMB can be determined using next-generation sequencing, and both panel and whole-exome DNA sequencing have been used to measure the mutation load of a tumor.

Because all genes are not expressed in every cell, inclusion of the mutations found in unexpressed genes may confound the utility of TMB to predict neoantigen load. In this study, we explore improving TMB by taking into account both DNA mutation and RNA expression with the hypothesis that using both criteria will lead to a TMB biomarker that correlates better with neoantigen load.

**Methods**

We examined DNA and RNA sequencing data for different cancer types in The Cancer Genome Atlas (TCGA) to determine refined TMB values. The data were assessed to identify mutations with and without expression.

**Results**

We found that a significant faction mutations included in a standard TMB calculations reside in genes that are not expressed, and this fraction varies significantly among samples. A corrected TMB that incorporates gene expression is likely to be a better predictor of neoantigen load. In addition, our group has previously identified TCGA samples with allele specific expression in which up to 25% of the tumor mutations are not expressed despite significant expression of the gene. The fraction of unexpressed mutations is much higher in some cancer types. Adding this correction to the TMB calculation, including only variants that are expressed in the tumor in the TMB calculation, changes the average TMB values found among different cancer types, and corrects the TMB in individual samples. This refined TMB value provides a biomarker that reclassifies samples scored as high TMB to low TMB and is likely to better predict response to IO treatment.

**Conclusions**

As our results suggest, adding RNA sequencing can be used to improve the TMB biomarker to better separate treatment groups. The initial analysis was performed with TCGA exome data and is now being extended to refine TMB values generated from gene panels including TSO 500. We are also evaluating differences in tumor-infiltrating lymphocytes in based on the refined TMB value.

#### P72 Comprehensive and accurate prediction of presented neoantigens using ImmunoID NeXT and advanced machine learning algorithms

##### Dattatreya Mellacheruvu, PhD, Rachel Pyke, Charles Abbott, PhD, Nick Phillips, Rena McClory, John West, MBA, Richard Chen, Sean Boyle, PhD, Dattatreya Mellacheruvu, PhD

###### Personalis Inc., Menlo Park, CA, United States

####### **Correspondence:** Sean Boyle (sean.boyle@personalis.com)

**Background**

Comprehensive detection of potential neoantigens and accurate prediction of their MHC presentation are critical prerequisites for selecting neoepitopes that can be used for creating personalized cancer vaccines. However, prediction models developed using in-vitro MHC-peptide binding assays cannot model upstream presentation machinery, such as proteasome cleavage and peptide loading. Advances in immuno-affinity purification followed by mass spectrometry (IP-MS) have enabled direct detection of MHC-bound peptides and can therefore be used for modelling native MHC-peptide presentation. Further, genetically engineered cell lines that express a single HLA allele enable unambiguous HLA-peptide assignment. Here, we present an overview of our MHC presentation prediction framework based on a large collection of such mono-allelic cell lines and discuss its utility in conjunction with ImmunoID NeXT, our commercially available exome scale DNA and RNA sequencing and analytics platform specifically designed to enable the development of immuno-therapies.

**Methods**

Mono-allelic cell lines were generated from K-562 null-HLA parental cells by transfecting each of the selected alleles. Cells were grown, screened for surface expression, lysed and immuno-affinity purified using a column coated with HLA class I (W6/32) antibody. Peptides were gently eluted and analyzed using LC-MS/MS. Peptide-to-spectrum assignment was performed and filtered at 1% false discovery rate.

**Results**

The training data for our MHC presentation prediction framework were generated using a large collection of genetically engineered mono-allelic cell lines, encompassing approximately 60 HLA Class I alleles that are frequently present across various populations. The resulting immuno-peptidomics data were comprehensive and of high quality - the peptide yields were high (median of approx. 1600 unique peptides per allele) and the dominant motifs were in agreement with published motifs. Our prediction framework is based on multiple modelling algorithms, including a multi-layer neural network, and uses proprietary and standard features such as peptide sequence, peptide length, binding pocket sequence and abundance (measured by transcripts per million). We created allele-specific and pan-allele models and evaluated them on an independent hold-out dataset. Both our allele-specific and pan-allele models had superior performance compared to other public tools, with a higher precision across a range of recall (sensitivity) values.

**Conclusions**

Our integrated pipeline for neoepitope discovery, which includes the comprehensive profiling of putative neoantigens using ImmunoID NeXT and accurate and sensitive prediction of MHC presentation of such neoantigens across all HLA Class I alleles (using our pan-allelic models) enables the effective generation of neoepitopes that are critical for developing personalized cancer vaccines.

#### P73 Large scale multiomics reveals a marked bias in driver mutations toward areas not reliably presented to the immune system

##### Alex Powlesland, PhD, Michael Cundell, PhD, Floriana Capuano, PhD, Brandon Higgs, David Lowne, BS, Ricardo Carreira, PhD

###### Immunocore Ltd, Abingdon, United Kingdom

####### **Correspondence:** Alex Powlesland (alex.powlesland@gmail.com)

**Background**

The repertoire of HLA-peptides presented to the immune system which derive from cancer-associated, viral, and mutated proteins are attractive targets for immunotherapy. Identifying the full complement of peptides derived from a protein presented on a major class-I HLA restriction provides a vital step toward increasing the speed and viability of many immunotherapeutic strategies. Advances in next-generation sequencing (NGS) and single-cell technologies have enabled the accurate capture of somatic mutations accumulated by a tumour, yet a significant hurdle remains how this information can be utilized for immunotherapeutic benefit. Identifying which somatic mutations produce neoantigens is crucial in providing the link between genetic change and immunological impact.

**Methods**

Directly identifying potential neoantigens using mass spectrometry offers a significant improvement over traditional approaches based on prediction. However, the relatively high sample requirement of this approach inherently limits the depth of analysis that can be performed, with a significant risk that low abundance neoantigens are not detected.

By integrating multiomics data from over 1000 experiments in 200 immortalised cell lines, we have generated a database of over two million unique HLA-peptide sequences that offers near total coverage of the protein-coding genome. Our comprehensive HLA class-I peptide atlas has been used as a reference tool to aid direct identification of neoantigens by targeted mass spectrometry, to probe indirectly for the presence of neoantigens, and to explore how many common driver mutations associated with cancer interact with the immunopeptidome.

**Results**

We have identified hundreds of neoantigens directly by mass spectrometry and found that mutated proteins follow the same pattern of antigen processing and presentation as their unmutated equivalents. As a result, our HLA peptide atlas offers significant value in predicting the likelihood of a somatic mutation creating a neoantigen. Comparing predicted neoantigens with those directly identified by mass spectrometry, we show effective prioritization of mutations by accurately predicting the presence and relative abundance of neoantigens. Applying this process toward the five most commonly mutated genes in cancer reveals a marked bias toward mutations that either act negatively or are in ‘quiet’ areas of the immune landscape. As all mutated peptides contain novel amino acid sequence, and are hence able to elicit an immune response, this ability to convert ‘potential’ into ‘actual’ is crucial in establishing a mechanism for identifying false positive results observed in cell-based assays.

**Conclusions**

An integrative multiomics approach to neoantigen identification has delivered a powerful reference for developing novel immunotherapies

#### P74 Integrating CD8 and CD4 effector neo-epitope content with regulatory T cell epitope exclusion is a superior prognostic biomarker for bladder cancer patient compared to their tumor mutation burden

##### Guilhem Richard, PhD^1^, Randy Sweis, MD^2^, Matthew Ardito, BA^1^, Tzintzuni Garcia^2^, Leonard Moise, PhD^1^, Michael Princiotta, MS, PhD^3^, Dominique Bridon^3^, William Martin, BA MD^1^, Gad Berdugo, MSc, MBA^3^, Arjun Balar^4^, Gary Steinberg^4^, Anne de Groot, MD^1^

###### ^1^EpiVax, Inc., Providence, RI, United States; ^2^University of Chicago, Chicago, IL, United States; ^3^EpiVax Oncology, New York, NY, United States; ^4^NYU Langone Health, New York, NY, United States

####### **Correspondence:** Gad Berdugo (gberdugo@epivaxonco.com)

**Background**

We hypothesized that neo-epitope-based prediction using an advanced in silico T cell epitope screening system (Ancer™) may better identify patients with improved prognosis than tumor mutation burden. Analysis of genomic data derived from the muscle-invasive bladder cancer (BLCA) cohort of The Cancer Genome Atlas (TCGA) database for CD4, CD8, and Treg neo-epitopes was performed to determine whether Ancer™ would improve prognostic stratification compared to tumor mutational burden (TMB).

**Methods**

BLCA patient mutanomes (n=412) were retrieved from the TCGA and evaluated with Ancer™, an innovative and automated neo-epitope screening platform that combines proprietary machine learning-based HLA I and HLA II neo-epitope identification tools with removal of inhibitory regulatory T cell epitopes for neo-epitope ranking and personalized cancer vaccine design. BLCA patients were separated based on median TMB or neo-epitope burdens. We investigated the effect of integrating both CD8 and CD4 neo-epitope burdens as most mutanome pipelines exclusively focus on the identification of Class I neo-epitopes. Overall survival was analyzed using the Kaplan-Meier method and differences analyzed by log-rank testing.

**Results**

Compared to low TMB, high TMB was significantly associated with improved survival (p = 0.0001, difference of 38.5 months in median survival, Figure 1). Improved differentiation of median survival times was obtained when separating patients based on their Class I neo-epitope content, as estimated by Ancer™ (p < 0.0001, difference of 59.8 months in median survival). Adding Class II neo-epitope burden further increased separation of OS times, showcased by a 69.6-month increase in median survival for BLCA patients with both high CD8 and high CD4 neo-epitope contents compared to other patients (p = 0.0001). Since we discovered that Class II neo-epitopes can induce inhibitory responses, we further evaluated whether the screening of these detrimental sequences could improve our analysis. Upon identifying Class II neo-epitopes likely to induce T effector (Teff) responses, we found that the median survival of patients with high CD8 and high CD4 Teff contents was extended by nearly 4 months to 73.4 months compared, to the remainder of the cohort (p < 0.0001, Figure 2).

**Conclusions**

Our analysis suggests that optimal host-immune recognition of CD8+, CD4+, and Treg epitopes plays a key role in cancer survival. While defining CD8 neo-epitope burden enhanced associations with OS, the inclusion of CD4 Teff neo-epitope burden substantially helped identify long-term survivors. These results suggest that defining the number of true neo-epitopes using Ancer™ may represent a novel prognostic or predictive biomarker.

**Fig. 1 (abstract P74).**
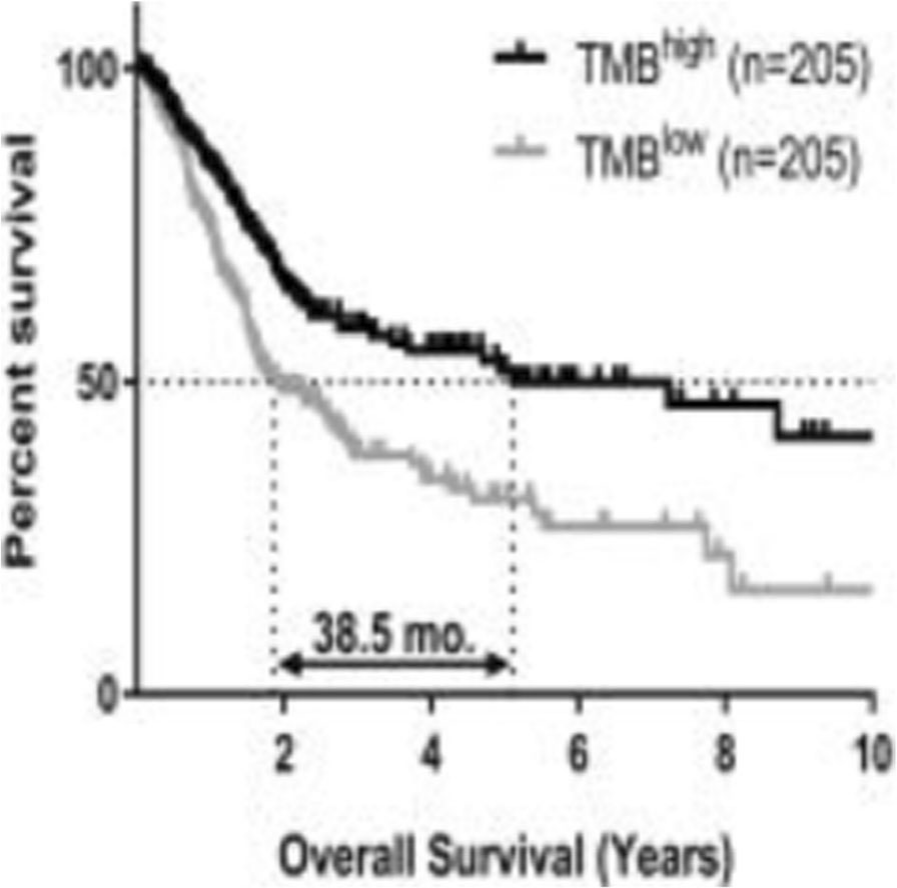
See text for description

**Fig. 2 (abstract P74).**
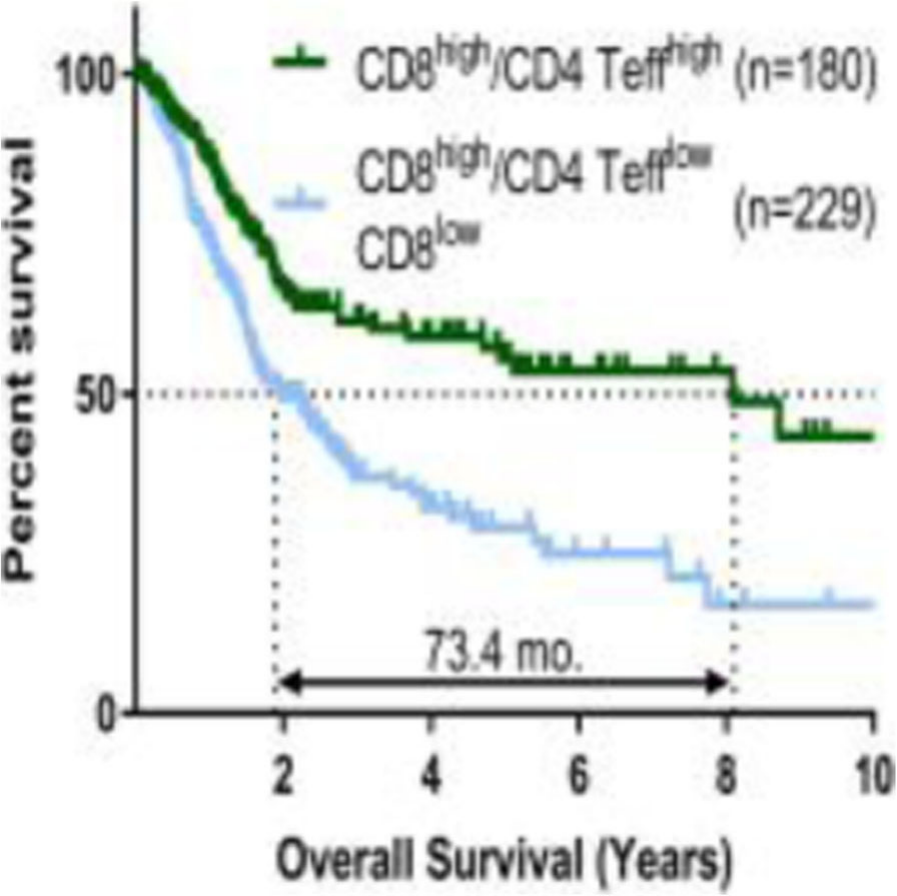
See text for description

#### P75 targetSCAPE and ultraSCAPE: Simultaneous identification and deep profiling of human antigen-specific T cells and other immune cell subsets by mass cytometry

##### David Roumanes, PhD^1^, Faris Kairi^1^, Alessandra Nardin, DVM^1^, Evan Newell, PHD^2^, Michael Fehlings^1^

###### ^1^immunoSCAPE, Cambridge, MA, United States; ^2^Fred Hutchinson Cancer Research Center, Singapore, Singapore

####### **Correspondence:** Alessandra Nardin (alessandra.nardin@immunoscape.com)

**Background**

During clinical trial immune monitoring, especially in the field of immunotherapy, it is critical to collect in-depth phenotypic information from multiple immune cell populations in order to assess the biological activity of the immunotherapy, to identify biomarkers of response or progression, and/or to identify new drug targets. However, patient samples, for example peripheral blood mononuclear cells (PBMC) or tissues, are often only available in small amounts and current methods face limitations in either depth of analysis and/or cell throughput.

**Methods**

In order to identify therapy-relevant antigens and to facilitate a concurrent in-depth characterization of cells directed towards these targets, immunoSCAPE leverages the high-dimensional immune profiling capabilities of cytometry by time of flight (CyTOF) and a unique methodology allowing the identification and characterization of rare antigen-specific T-cell subsets (targetSCAPE). By implementing a new technology (ultraSCAPE) that combines flow and mass cytometry together with a combinatorial live cell barcoding strategy, we further increased the high-dimensional phenotyping capacities to over 100 different marker molecules through simultaneous in-depth profiling of up to three additional immune cell subsets from the same sample.

**Results**

We isolated 4 different immune cell populations from a single sample and combined 3 different phenotypic panels consisting of 35 makers each together with a combinatorial tetramer multiplex and phenotyping panel for deep profiling of myeloid cells, NK cells, B cells and T cells. We demonstrate the potential of this novel immuno-phenotyping method, by tracking virus-specific T cells while simultaneously characterizing 4 immune cell subsets with over 100 distinct phenotypic markers from a single sample, which is currently impossible employing modern flow cytometers or classical mass cytometry methods.

**Conclusions**

With its ability to provide an unprecedented picture of the immune status within a single sample, including T cell specificity information and in depth profiling of relevant immune cell subsets, ultraSCAPE in combination with targetSCAPE can provide detailed insights on the effects of immunotherapy on the immune cell population. Information learned from in-depth immune phenotyping of several immune cell subsets such as T, NK and myeloid cell subsets can be leveraged for the development of novel diagnostics, for biomarker discovery and for monitoring therapeutic strategies in immunotherapy.

#### P76 Development of immunopeptidomic platform for human leucocyte antigens class I using microflow liquid chromatography and quadrupole time-of-flight mass spectrometry

##### Takashi Shimada, PhD^1^, Noriko Iwamoto, PhD^1^, Yoshinobu Koguchi, MD, PhD^2^, John Cha^2^, Brian Piening, PhD^2^, Eric Tran, PhD^2^, Hong-Ming Hu, PhD^2^, Bernard Fox, PhD^2^, William Redmond, PhD^2^

###### ^1^Shimadzu Scientific Instruments, Bothell, WA, United States; ^2^Providence Cancer Center, Portland, OR, United States

####### **Correspondence:** Takashi Shimada (tashimada@shimadzu.com)

**Background**

The highly complex population of peptides associated with human leucocyte antigens (HLA) is the human immunopeptidome. Comprehensive characterization of the immunopeptidome is key in predicting immunotherapeutic responses by evaluating targets of T cell interaction and in developing the next generation of cancer immunotherapies. Mass spectrometry (MS) is a technology that holds significant promise for untargeted and complete identification of the immunopeptidome. MS acquisition is mainly used an electrospray ionization (ESI) combined with nanoflow liquid chromatography (LC). However, the analysis time and retention reproducibility could be issues. Therefore, we tried to develop an MS platform that can ensure the coverage while increasing throughput using a microflow LC.

**Methods**

HLA class I complexes were purified from A431 cell lysate by W6/32 immunoaffinity. Purified HLA peptides were eluted with 5% formic acid. The peptides were fractionated by 10 kDa ultrafiltration. HLA peptides were separated with L-column2 ODS (0.3x150 mm) using a trap-elute protocol of microflow LC (Nexera-Mikros) and quadrupole time-of-flight MS (LCMS-9030). Flow rate was set at 5 μl/min with a gradient of acetonitrile in 0.1% formic acid for 18 min. MS/MS spectra were acquired using a data-dependent manner of top-10 precursor intensities. The precursor scan was first set from 400 to 600 Da. The charge states of precursors were set between 1 to 4, and MS/MS scan was from 200 to 1200 Da. The data were analyzed by Mascot proteome server and PEAKS sequencing software on SwissProt database. The mass tolerances of precursors and fragments were set at 0.05 Da and 0.3 Da. Minimal peptide length was set to 8 amino acids.

**Results**

An initial round of optimizations was performed to establish optimal parameters for immunopeptidome identifications. Using tryptic peptides from A431 lysate, we optimized the 50-100 msec repeat of MS/MS scanning, top-10 of data-dependent acquisitions per scan, 50 Da scan range, 35V±10V spread of collision electrode voltage, and 3.0 kV of electrospray voltage. These parameters were then applied for identification of HLA-associated peptides from A431 cells. From this, we identified 4,217 MS/MS and 801 sequences from 34,042 spectra.

**Conclusions**

From these data, we demonstrate that similar sensitivity can be sufficiently achieved with microflow platform as has been demonstrated preciously for nanoflow LC-MS. This has significant advantages in terms of throughput, instrument maintenance, and widespread applicability. Our future directions are to determine whether cancer neoepitopes identified by these approaches may be recognized and therapeutically targeted by patient T cells.

#### P77 Comprehensive profiling of tumor-immune interaction in anti-PD-1 treated melanoma patients reveals subject-specific tumor escape mechanisms

##### Charles Abbott, PhD^1^, Eric Levy, PhD^1^, Rachel Pyke^1^, Rena McClory^1^, Sekwon Jang, MD^2^, Richard Chen, PhD^1^, Sean Boyle, PhD^1^

###### ^1^Personalis, Menlo Park, CA, United States; ^2^Inova, Fairfax, VA, United States

####### **Correspondence:** Sean Boyle (sean.boyle@personalis.com)

**Background**

Checkpoint inhibitor therapy has demonstrated meaningful antitumor activity for many patients, though the majority fail to achieve complete response. Thus, it is of particular interest to identify biomarkers and mechanisms that promote positive response to immunotherapy. In the present study, we apply our comprehensive tumor immunogenomics platform (ImmunoID NeXT), integrating data from the tumor, tumor microenvironment and immune system to create a comprehensive biological signature of patient response to therapy.

**Methods**

We characterized the immunogenomics of 52 unresectable, stage III/IV melanoma patients who underwent anti-PD-1 therapy to assess factors influencing response. RECIST criteria were used to evaluate tumor response to therapy, with a median follow-up of 12 months. For each patient, a single paired FFPE tumor and normal blood sample was collected and profiled using Personalis’ ImmunoID NeXT platform; an augmented exome/transcriptome platform and analysis pipeline, which produces comprehensive tumor mutation information, gene expression quantification, neoantigen characterization, HLA typing and LOH, TCR repertoire profiling and tumor microenvironment profiling. Tumor molecular information was then analyzed together with clinical outcome.

**Results**

Comprehensive profiling demonstrated that elevated pretreatment neoantigen burden was predictive of response to PD-1 blockade, and significantly associated with progression-free survival. Additionally, we observed increased response to anti-PD-1 therapy in patients with elevated pretreatment TCR clonality. Patients with high neoantigen burden and TCR clonality that failed to achieve complete response revealed potential resistance mechanisms to anti-PD-1 therapy. Specifically, we identified two patients with high expression of IDO1 or CTLA4, which may facilitate PD-1-independent immune escape. Additionally, we found two patients with antigen presentation machinery (APM) mutations. The first patient had independent HLA-A and HLA-B mutations, likely leading to loss of surface expression of the proteins. In the second APM mutation patient we observed a high frequency (80% AF) frameshift variant in B2M, which potentially prevents proper HLA class I folding and antigen presentation. These APM mutations suggest reduced neoantigen presentation in these patients, which are probable mechanisms for tumor escape. By integrating neoantigen burden, HLA-LOH and APM mutational data into a corrected neoantigen burden, we were able to increase the predictive strength of this biomarker.

**Conclusions**

In summary, our comprehensive cancer immunogenomic analyses demonstrate that genomic and immune profiling of pretreatment patient samples can identify biomarkers and resistance mechanisms to immune checkpoint blockade, suggesting the potential efficacy of these as an integrated biomarker to optimize anti-PD-1 therapy patient selection.

#### P78 Optimization of tumor nutation burden measurement in FFPE DNA

##### Janice Au-Young, PhD, Iris Casuga, PhD, Vinay Mittal, Dinesh Cyanam, MS, Elaine Wong-Ho, Fiona Hyland, Seth Sadis, Warren Tom, PhD

###### Thermo Fisher Scientific, South San Francisco, CA, United States

####### **Correspondence:** Seth Sadis (Seth.Sadis@thermofisher.com)

**Background**

Tumor mutation burden (TMB) measures the number of somatic mutations and is a positive predictive factor for response to immune-checkpoint inhibitors in multiple cancer types. While whole exome sequencing (WES) is the gold standard for TMB measurement, it is not practical for routine use. TMB values measured using targeted sequencing have been shown to have good correlation with WES. However, during FFPE preservation, DNA may undergo cytosine deamination, resulting in false C>T substitutions and elevated TMB values. We have assessed the effect of DNA damage and repair on TMB values using the Oncomine Tumor Mutation Load Assay (OTMLA), a targeted next generation sequencing assay.

**Methods**

We measured TMB from 37 FFPE colon, lung, endometrial and gastric tumors using the OTMLA panel on Ion GeneStudio with 20ng of input DNA from tumor only samples. The informatics workflow utilizes a custom variant calling and germline variant filtering algorithm to accurately estimate somatic variants in tumor tissue. In parallel, TMB was measured by Whole Exome Sequencing (WES) targeting 50Mb using 100ng of tumor and matched normal DNA on a HiSeq X instrument. We examined factors that affect OTMLA measurements: Deamination signature, degree of deamination and allele ratio identify DNA samples with high levels of damage due to FFPE preservation. A Uracil-DNA glycosylase (UDG) repair step was introduced to eliminate damaged targets and improve usable TMB values of DNA from FFPE tumor tissue. At the variant level, samples with high deamination scores were analyzed dynamically as a function of allele frequency to study TMB values for correlation with WES.

**Results**

OTMLA TMB values showed good correlation with WES-derived TMB; however ~10% of tumor DNA samples had high TMB and deamination values outside the expected range. These samples were included as a subset of samples tested with and without the UDG repair step. UDG treatment decreased TMB and deamination scores, resulting in higher correlation with WES TMB values. Some samples with very high deamination scores were unable to be rescued; however, TMB values in samples with low deamination and minimal damage were not affected.

**Conclusions**

We show that deaminated cytosine bases can be enzymatically removed by treatment with UDG. In a subset of FFPE samples tested, UDG treatment was demonstrated to reduce the OTMLA estimated SNP proportion consistent with deamination. This results in consistent and effective reduction of C>T artifacts without affecting true variants and can provide TMB values in a biologically relevant range.

#### P79 Molecular comparison of tumor microenvironment in primary lung, melanoma, and kidney tumors versus paired lung metastases reveals shared perturbations of the immune milieu during oligoprogression

##### Davide Bedognetti, MD, PhD^1^, Jessica Roelands, Master^1^, Angelo Manfredi^2^, Norma Maugeri^2^, Francesca De Nicola^3^, Ludovica Ciuffreda^3^, Matteo Pallocca^3^, Maurizio Fanciulli^3^, Francesca Di Modugno, PhD^3^, Paolo Visca^3^, Barbara Antoniani^3^, Gabriele Alessandrini^3^, Darawan Rinchai, PhD^1^, Wouter Hendrickx, PhD^1^, Paola Nistico', MD^3^, Gennaro Ciliberto, MD^3^

###### ^1^Sidra Medicine, Doha, Qatar; ^2^IRCCS Ospedale San Raffaele, Milano, Italy; ^3^Istituto Nazionale Tumori Regina Elena, Rome, Italy

####### **Correspondence:** Gennaro Ciliberto (gennaro.ciliberto@ifo.gov.it)

**Background**

The importance of tumor-host interactions during cancerogenesis and metastatic progression has been now widely appreciated. More recently, the impact of the tumor immune microenvironment (TIME) to mold tumor evolution was convincingly demonstrated [1]. Solid tumors systemically reprogram the lung unique immune environment, dominated by intravascular neutrophil functions [2], to colonize this site. The concept of ‘oligoprogression’ has recently received mounting attention, due to its relevance and because it represents an interesting in vivo model to study TIME, although the specific mechanisms of oligometastatic process are relatively underinvestigated [3, 4].

**Methods**

RNA sequencing was performed on a retrospective collection of tissue samples from primary renal cell carcinoma, melanoma, and NSCLC and paired lung oligometastases of untreated patients (Figure 1). Enrichment of tumor-related pathways and transcripts that reflect the enrichment of immune cell subsets was assessed by single sample gene set enrichment analysis. Differentially expressed genes between primary tumors versus the corresponding lung metastases were used for pathway analysis. Neutrophils extracellular traps (NETs) were revealed by immunofluorescence, assessing extracellular DNA and citrullinated H4 histone co-localization and/or myeloperoxidase [5]. Autophagy was assessed the CYTO-ID® kit [5].

**Results**

While tumor-related pathway enrichment differed mostly according to the primary tumor histology, perturbations of immune-regulatory pathways was observed during oligoprogression in the lung. Deconvolution of immune cell subpopulations identified increased immature dendritic cells and reduced T cell abundance in oligometastatic lesions. Strikingly, a large proportion of differentially modulated pathways were “immune” rather than “cancer-cell”-related. Core analysis confirmed that the main transcriptomic network that is affected during disease progression is immune-based, centered on a cross-link between innate and adaptive immunity. Specifically, it was associated with decreased HLA, iCOS, IL-9, and IL-17 pathway activity and downregulation of interferon signaling. During progression, we observed coherent modulation of transcripts associated with NET generation, related to upregulation of key autophagic genes, to competition of the HMGB1 molecule with CXCL12 and CXCR4 and RAGE receptor (AGER) activation. Accordingly, NET expression was strikingly more abundant in lung metastases than in primary tumors.

**Conclusions**

Our results identify evident molecular mechanisms associated with suppression of the immune milieu during disease oligoprogression in the lung across different tumors. They include innate-adaptive immune dysfunction HLA-mediated, and interferon dysregulation associated with neutrophil-mediated immune suppression. Since these tumors are targeted by immune checkpoint blockade (ICB) our data highlights the relevance of characterizing the TIME composition in paired primary and oligometastatic lesions during ICB treatment to optimize treatment approaches.

**Acknowledgements**

Paola Nistico' and Gennaro Ciliberto are co-last authors.

**References**

1. Angelova M, Mlecnik B, Vasaturo A, Bindea G, Fredriksen T, Lafontaine L, et al. Evolution of Metastases in Space and Time under Immune Selection. Cell. 2018 Oct;175(3):751-765.e16.

2. Granton E, Kim JH, Podstawka J, Yipp BG. The Lung Microvasculature Is a Functional Immune Niche. Trends Immunol. 2018 Nov;39(11):890-899.

3. Weichselbaum RR. The 46th David A. Karnofsky Memorial Award Lecture: Oligometastasis-From Conception to Treatment. J Clin Oncol. 2018 Sep27:JCO1800847.

4. Stephens SJ, Moravan MJ, Salama JK. Managing Patients With Oligometastatic Non-Small-Cell Lung Cancer. J Oncol Pract. 2018 Jan;14(1):23-31.

5. Maugeri N, Campana L, Gavina M, Covino C, De Metrio M, Panciroli C, Maiuri L, Maseri A, D'Angelo A, Bianchi ME, Rovere-Querini P, Manfredi AA.Activated platelets present high mobility group box 1 to neutrophils, inducing autophagy and promoting the extrusion of neutrophil extracellular traps. J Thromb Haemost. 2014 Dec;12(12):2074-88. doi: 10.1111/jth.P689.

**Ethics Approval**

The study was approved by IFO Institution’s Ethics Board, approval number 561/03.

**Fig. 1 (abstract P79).**
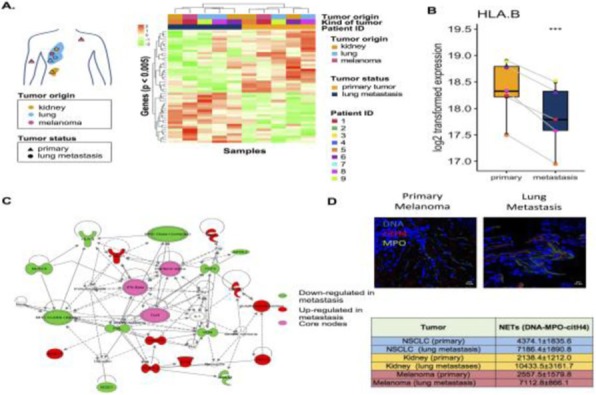
See text for description

#### P80 HER2 is associated with prolonged survival in advanced gastroesophageal adenocarcinoma patients treated with checkpoint blockade

##### Carrie Brachmann, PhD^1^, Emon Elboudwarej^1^, Manish Shah, MD^2^, David Cunningham^3^, Jean-Philippe Metges^4^, Eric Van Cutsem, MD, PhD^5^, Zev Wainberg, MD^6^, Jingzhu Zhou^1^, Dung Thai^1^, Pankaj Bhargava^1^, Daniel Catenacci, MD^7^

###### ^1^Gilead Sciences, Foster City, CA, United States; ^2^Weill Cornell Medicine, NY Presbyterian, New York, NY, United States; ^3^Sutton and London Hospital, London, United Kingdom; ^4^Brest University Hospital, Brest, France; ^5^University Hospitals Leuven & KU Leuven, Leuven, Belgium; ^6^UCLA School of Medicine, Santa Monica, CA, United States; ^7^University of Chicago Medical Center, Chicago, IL, United States

####### **Correspondence:** Carrie Brachmann (carrie.brachmann@gilead.com)

**Background**

The benefit of checkpoint blockade in advanced gastric cancer is limited and patient selection biomarkers are needed. In a randomized phase 2 study in >=2nd line advanced gastroesophageal adenocarcinoma (GEA) cancer in Europe, US and Australia, there was no clinical benefit for the addition of andecaliximab to nivolumab in the total population or evaluated subgroups (including PD-L1) [1,2]. Pharmacodynamic analyses demonstrated little to no impact of andecaliximab [3]. This exploratory biomarker analysis included all patients as a nivolumab-treated population.

**Methods**

Evaluation of archival tumor tissue was described [2,3]. Tumor mutation burden (TMB) was evaluated by whole exome sequencing with matched normal. Survival analyses (cox proportional hazards) were adjusted for age and sex.

**Results**

Overall survival (N=141, median 6.05 months) was not associated with PD-L1 positive (>=1% tumor or tumor+immune cells)/negative, diffuse/non-diffuse, gastric/gastroesophageal, prior therapy (median=2), tumor IFNgamma signature or CD8+ tumor infiltrate. Differential gene expression analysis identified GRB7, a downstream mediator of HER2 signaling and part of the HER2/ERBB2 amplicon in breast cancer [4], as one of two genes associated with survival >1 year (FDR=0.027). HER2-positivity (medical record) was associated with a 3.5-fold higher median expression of GRB7. Prolonged survival was associated with both HER2-positivity (n=43/132; HR=0.58, p=0.01) and the top quartile of GRB7 expression (n=25/94; HR=0.48, p=0.007). The median survival for HER2-positive patients was 10.1 months versus 5.95 months for HER2-negative. HER2 status was not associated with PD-L1 status or CD8+ infiltrate. Nearly all HER2-positive (n=40/43) and 2 HER2-negative patients received trastuzumab (median 62 days post-trastuzumab). Prior or best response was not related to 1 year survival and 2 of 3 HER2-positive patients that did not receive trastuzumab had >1 year survival. TMB was also evaluated and significantly associated with HER2-positivity (N=61, p=0.041). In the subset of 61 patients with TMB data, patients with high TMB plus HER2-positivity had the longest median survival of 15.4 months compared to all other patients at 6.7 months (N=14 vs 47; HR=0.47; p=0.04).

**Conclusions**

HER2 was associated with improved survival with checkpoint blockade in advanced GEA patients, regardless of response to prior trastuzumab. This study was limited by the lack of pre-treatment biopsies, but consistent with a recent report on Asian GEA patients [5]. The combination of HER2-positivity and relatively higher TMB in a limited dataset led to the greatest observed median survival time, suggesting an interaction between HER2 and TMB that warrants exploration in future GEA studies involving checkpoint inhibition.

**Acknowledgements**

The authors gratefully acknowledge the patients and their families who participated in this study.

**Trial Registration**

Clinicaltrials.gov NCT02862535

**References**

1. Shah M, Metges J-M, et al. A phase 2, open-label, randomized study to evaluate the efficacy and safety of andecaliximab combined with nivolumab versus nivolumab alone in subjects with unresectable or recurrent gastric or gastroesophageal junction adenocarcinoma. ASCO Gastrointestinal Cancers Symposium. 2019.

2. Metges J-M, Elboudwarej E, et al. Exploratory evaluation of baseline tumor biomarkers and their association with response and survival in patients with previously treated advanced gastric cancer treated with andecaliximab combined with nivolumab versus nivolumab. ASCO Gastrointestinal Cancers Symposium. 2019.

3. Brachmann C, Zhang Y, et al. Evaluation of intratumoral T cells in biopsies from advanced gastric patients treated with andecaliximab and nivolumab. ASCO Gastrointestinal Cancers Symposium. 2019.

4. Ferrari A, Vincent-Saloman A, et al. A whole-genome sequence and transcriptome perspective on HER2-positive breast cancers. Nature Communications. 2016.

5. Satoh T, Kang Y-K, et al. Exploratory subgroup analysis of patients with prior trastuzumab use in the ATTRACTION-2 trial: a randomized phase III clinical trial investigating the efficacy and safety of nivolumab in patients with advanced gastric/gastroesophageal junction cancer. Gastric Cancer. 2019.

**Ethics Approval**

This study was approved by the institutional review board or independent ethics committee appropriate for each site.

#### P81 Association of tumor mutational burden with clinical, genomic, and treatment characteristics in advanced non-small cell lung cancer

##### Connor Willis, PharmD^1^, Hillevi Bauer, PharmD^1^, Trang Au, PharmD^2^, Sudhir Unni, PhD, MBA^1^, Wallace Akerley, MD^1^, Ashley Sekhon, MD^3^, Firas Badin, MD^4^, John Villano, MD, PhD^5^, Matthew Schabath, PhD^6^, Bing Xia, MD^7^, Beth Gustafson, PharmD^8^, Komal Gupte-Singh, PhD^9^, Beata Korytowsky^9^, John-Michael Thomas, PharmD^9^, Gabriel Krigsfield, PhD^9^, Solomon Lubinga, PhD^9^, Diana Brixner, PhD^1^, David Stenehjem, PharmD^10^

###### ^1^University of Utah, Salt Lake City, UT, United States; ^2^Long Island University, Brooklyn, NY, United States; ^3^MetroHealth Medical Center, Cleveland, OH, United States; ^4^Baptist Health, Lexington, KY, United States; ^5^Markey Cancer Center, Lexington, KY, United States; ^6^H. Lee Moffitt Cancer Center, Tampa, FL, United States; ^7^University of Southern California, Los Angeles, CA, United States; ^8^Saint Luke's Cancer Institute, Kansas City, MO, United States; ^9^Bristol-Myers Squibb, Princeton, NJ, United States; ^10^University of Minnesota, Duluth, MN, United States

####### **Correspondence:** David Stenehjem (stene032@d.umn.edu)

**Background**

Tumor mutational burden (TMB) is emerging as a potential predictor of response to immunotherapy in various tumor types. However, the association of TMB data with clinical, demographic, genomic, and treatment characteristics warrants further investigation.

**Methods**

Nine U.S. Comprehensive Cancer Centers participated in this observational, cohort study; five centers are members of the Oncology Research Information Exchange Network (ORIEN). Adult patients with stage IV non-small cell lung cancer (NSCLC) with tissue-based TMB data from any testing platform were included and their treatment information was abstracted using a standardized case report form. TMB reporting ranged from September 2014 through March 2019. TMB-High and TMB-Low were defined as >10 mutations/megabase (mut/Mb) and <10 mut/Mb, respectively. Clinical, demographic, genomic, and treatment characteristics were compared by TMB level.

**Results**

There were 426 patients enrolled in the study across seven of the nine sites. TMB results from comprehensive genomic profiling (CGP) were available for 354 patients. CGP vendors included Foundation Medicine (79.9%), Caris Life Sciences (17.0%), Tempus (2.8%), and NantHealth (0.3%). The median time from diagnosis to CGP testing was 45 days. A comparison of clinical and demographic characteristics by TMB is presented in Table 1. TMB-High status was associated with male gender (p<0.01), and positive smoking history (p<0.01). No correlation was found between TMB and PD-L1 (Table 2). TMB-High was positively associated with multiple oncogenes including STK11, LRP1B, TP53, and KDM5C (Table 2). In addition, there were significant negative associations between TMB-High and individual occurrences of altered ALK (p=0.03), EGFR (p<0.01), and ROS1 (p=0.03). The proportion of patients receiving first-line immunotherapy increased yearly from 8.5% in 2015, 19% in 2016, 40% in 2017, and 46% in 2018.

**Conclusions**

These interim results demonstrate the feasibility of conducting multi-site observational electronic health record-based studies with CGP and TMB across a national cohort of comprehensive cancer centers. Immunotherapy utilization has been increasing in the first-line setting. Associations between TMB status and driver mutations are indicative of cancer etiology and informative for treatment decision-making. Updated results will be presented with an expanded cohort of patients and future publications with the final cohort (n~1000) will explore treatment, survival and response data.

**Acknowledgements**

This study was sponsored by Bristol-Myers Squibb. Recruitment efforts were supported by Mikaela Larson (Huntsman Cancer Institute) and M2Gen®

**Table 1 (abstract P81).**
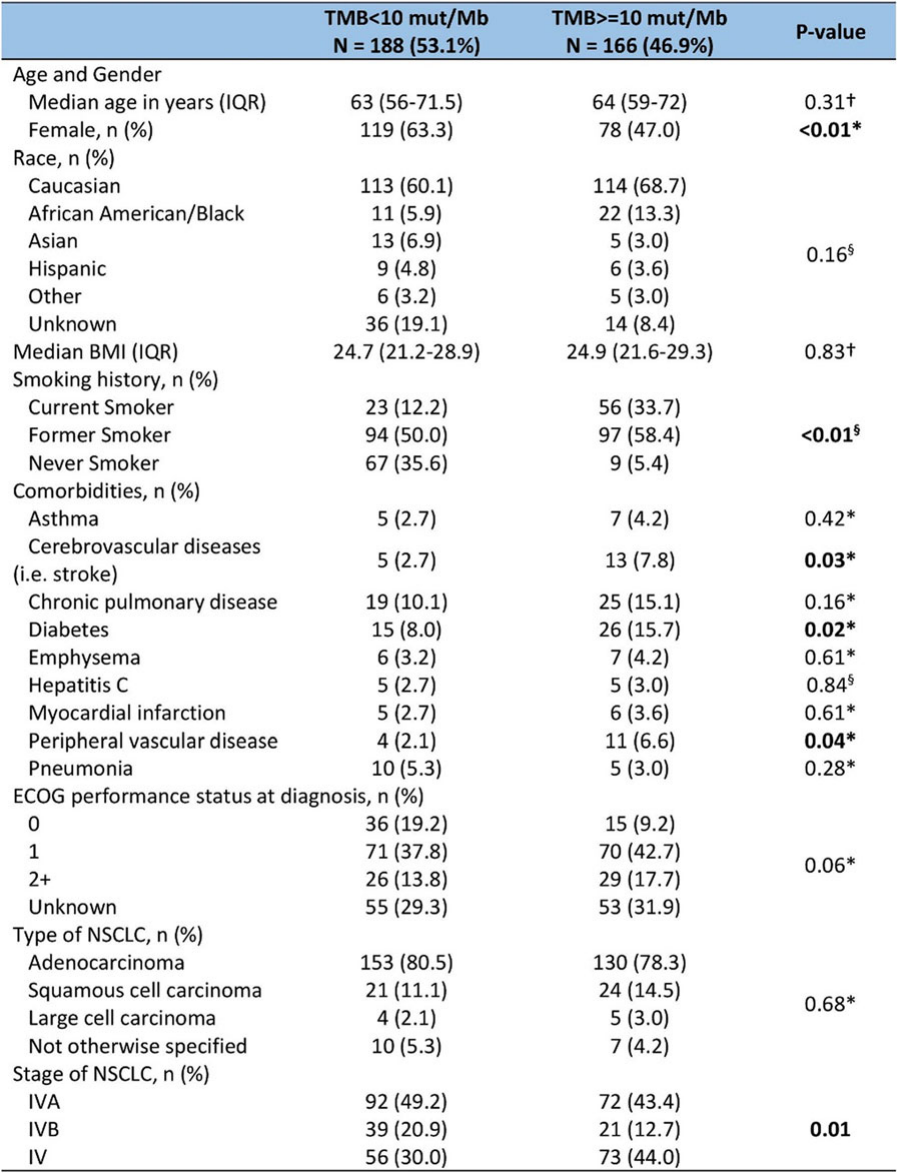
Associations between TMB and Baseline Characteristics

**Table 2 (abstract P81).**
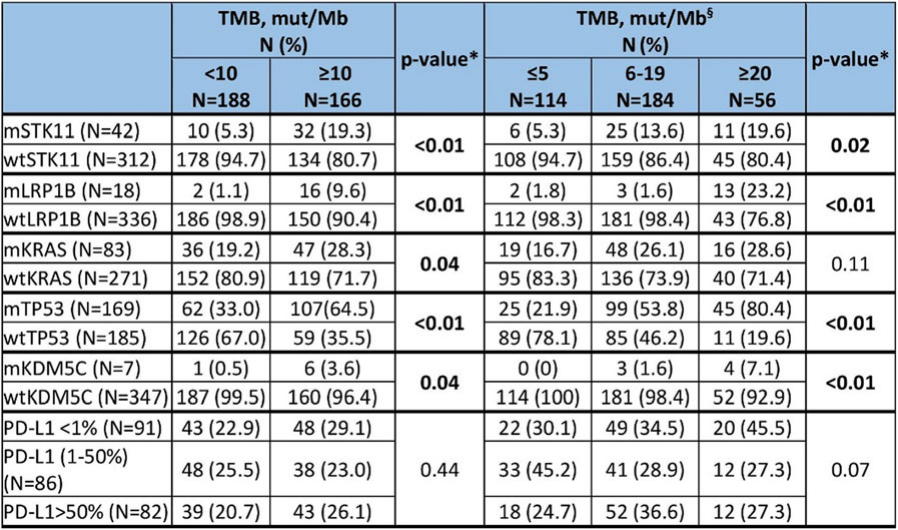
Associations between TMB and Select Oncogene Mutations

#### P82 High-throughput pairing of single T-cell α and β chains along with phenotypic expression profiling

##### Brittany Brown, BS^1^, Miranda Byrne-Steele, PhD^2^, Wenjing Pan, PhD^3^, Song Li^2^, Mary Eisenhower, BS^2^, Daniel Weber^2^, Mollye Depinet, MS^4^, Xiahong Hou, PhD, MD^2^, Alex Moore^2^, Jian Han, MD PhD^2^

###### ^1^iRepertoire, Inc., Huntsville, AL, United States; ^2^iRepertoire, Huntsville, AL, United States; ^3^HudsonAlpha Institute for Biotechnology, Huntsville, AL, United States; ^4^iRepertoire.com, Huntsville, AL, United States

####### **Correspondence:** Jian Han (jhan@irepertoire.com)

**Background**

The T-cell receptor (TCR) is responsible for recognizing antigens as peptides bound to a major histocompatibility complex. TCRs typically contain both an alpha (α) and beta (β) chain that contribute to antigen specificity; however, we have seen multiple cases of a single cell containing dual α or dual β chains as well. When analyzing bulk repertoires, information about endogenous pairing of α and/or β chains is lost after bulk lysis of T-cell populations. Pairing α and β chains from a single cell while also analyzing the phenotypic expression allows us to track TCR specificity and T cell function. This information can provide direct calculations of clonal frequency in various cell subsets, allow tracking of specific lymphocytes with treatment, and reveal paired information for both chains of the receptor for downstream Car-T development.

**Methods**

Here, we developed a method for high-throughput pairing of TCR α and β chains along with expression profiling. We examined, on average, around 15,000 CD4+ cells loaded onto the BD Rhapsody Express system. The receptor information is amplified from the same cDNA using iRepertoire’s proprietary method that incorporates a multiplex mix of primers associated with both the TCR α and β loci; phenotyping of the cell is obtained using the BD Rhapsody RNA-seq kit. Alongside the high throughput data, we also performed FACS-based single cell sequencing on the same individual’s samples through our iPair method (presented previously) and examined the overlapping receptor sequences between both methods.

**Results**

With this mid throughput method, we are able to accurately assess the frequency of single cells containing dual alpha or dual beta TCRs, which can help to evaluate the high throughput data.

**Conclusions**

The described high throughput application should be applicable to any oligo-dT based single cell strategy.

**Ethics Approval**

This study was approved by New England IRB, IRB number 120160202

#### P83 Pan-cancer assessment of composite genomic biomarkers in immuno-oncology to predict responses and resistances to immune checkpoint inhibitor therapy

##### Gustavo Cerqueira, Laurel Keefer, Kelly Gerding, PhD, Kenneth Valkenburg, Christina Oliveras, James White, Leila Ettehadieh, Christopher Gault, James Hernandez, Eric Kong, Isabell Loftin, Samuel Angiuoli, Abigail McElhinny, John Simmons, PhD

###### Personal Genome Diagnostics, Baltimore, MD, United States

####### **Correspondence:** Eric Kong (ekong@pgdx.com)

**Background**

Therapeutic response to immune checkpoint inhibitors (ICIs) requires a prior, suppressed immune response that is released via the interaction of the checkpoint receptors with their cognate ligands. Microsatellite instability (MSI) and tumor mutation burden (TMB) have emerged as composite genomic metrics that may better predict patient response to ICI treatment, compared to conventional PD-L1 expression. However, testing for MSI and TMB separately is labor intensive, increases turnaround time, and consumes valuable tumor tissue samples. Furthermore, recent studies have demonstrated that antigen presentation mechanisms may also play a role in predicting outcomes, wherein loss of heterozygosity in MHC class I genes (LOH-MHC) suggests low likelihood of ICI benefit.

**Methods**

Here, we propose that these varied immune-oncology metrics can be measured together in a single assay, utilizing next-generation sequencing (NGS) technologies. To test this hypothesis, we analyzed >200 pan-cancer FFPE tumor tissue samples using the PGDx elio™ tissue complete assay (currently in development; >500 genes panel) to measure MSI, determine TMB (across 1.3 Mb), and assess MHC status in a single assay. Detection of MSI was assessed for accuracy against a validated PCR approach and TMB determination from our targeted panel was compared to whole-exome sequencing (WES) derived TMB. LOH-MHC status reported from PGDx elio tissue complete was compared to MHC status determined by WES for accuracy. Additionally, TMB and MHC status were analyzed together in FFPE samples from ICI treated patients and correlated to clinical outcomes.

**Results**

115 pan-cancer samples were analyzed for microsatellite status and demonstrated an overall agreement of 100.0% with a PCR-based method. TMB was determined in 118 pan-cancer samples and displayed a high level of concordance with WES-derived TMB (Pearson correlation, p=0.903) across a range of TMB scores (0.2-89.7 muts/Mbp). FFPE tissue samples from 98 cancer patients previously treated with ICIs were then tested for LOH-MHC and demonstrated 88% accuracy of detection when compared to WES. Furthermore, analysis of TMB and MHC status in tandem found that patients with both high TMB and normal MHC status were found to have a significantly higher PFS, suggesting greater efficacy of ICI therapy.

**Conclusions**

These data demonstrate the feasibility of measuring MSI, TMB, and evaluating LOH-MHC with high accuracy in a single >500 gene NGS assay. Additionally, the results presented herein suggest that measuring TMB and MHC status concurrently can provide added utility in predicting patient response to ICI therapy.

#### P84 Panel-derived tumor mutational burden (TMB) correlates with immune checkpoint inhibitors (ICIs) response in gastrointestinal cancers

##### San-chi Chen, MD^1^, Kien-Thiam Tan, PHD^2^, Ming-Huang Chen^1^, Yi-Ping Hung, MD^1^, Yi-Lin Hsieh^2^, Yi-Hua Jan^2^, Yee chao^1^

###### ^1^Taipei Veterans General Hospital, Taipei, Taiwan, Province of China; ^2^ACT Genomics Co., Ltd., Taipei, Taiwan, Province of China

####### **Correspondence:** Yee chao (ychao@vghtpe.gov.tw)

**Background**

Tumor mutational burden (TMB) has been emerging as a relatively new biomarker that is independent of PDL1 for the prediction of response to the immune checkpoint inhibitor (ICI) treatment. A recent study has shown that whole exome sequencing (WES)-derived TMB correlates well panel-derived TMB that is estimated using targeted sequencing. Here, we evaluate the correlation between panel-derived TMB with response to ICI treatment in gastrointestinal cancers.

**Methods**

FFPE tumor and normal tissues from 18 patients with gastrointestinal cancers who had previously received ICI therapy at Taipei Veterans General Hospital were retrospectively underwent targeted next-generation sequencing (ACTOncoTM) for the identification of somatic variants across 440 genes and the calculation of TMB. NetMHC and IEDB were used to predict neopeptide bound to patient-specific HLA class one genotype. RECIST criteria were used to categorize tumor response.

**Results**

Patients were grouped into responder (PR or CR, n=10) and non-responder (PD or SD, n=7). Among all patients, responders had significantly higher TMB than non-responders (mean 7.37 muts/Mb vs. 1.24 muts/Mb, p=0.0007). The number of predicted neopeptides were significantly higher in responders than in non-responders (mean 10.8 vs. 3.6, p=0.0226). Notably, a non-responder harboring EGFR gain-of-function mutation did not respond to the ICI treatment despite high TMB. Furthermore, patients harboring MUC16 mutation demonstrated higher TMB than patients without MUC16 mutation (mean 17.38 muts/Mb vs. 3.9 muts/Mb, p=0.0005) (Figure 1).

**Conclusions**

Although the cohort size is small, our study showed that panel-based TMB is predictive of response to ICI. As in lung cancers, EGFR mutation is associated with decreased efficacy of ICI in the GI cancers.

**Ethics Approval**

The study was approved by TPEVGH intuition’s Ethics Board, approval number 2015-07-002BC.

**Fig. 1 (abstract P84).**
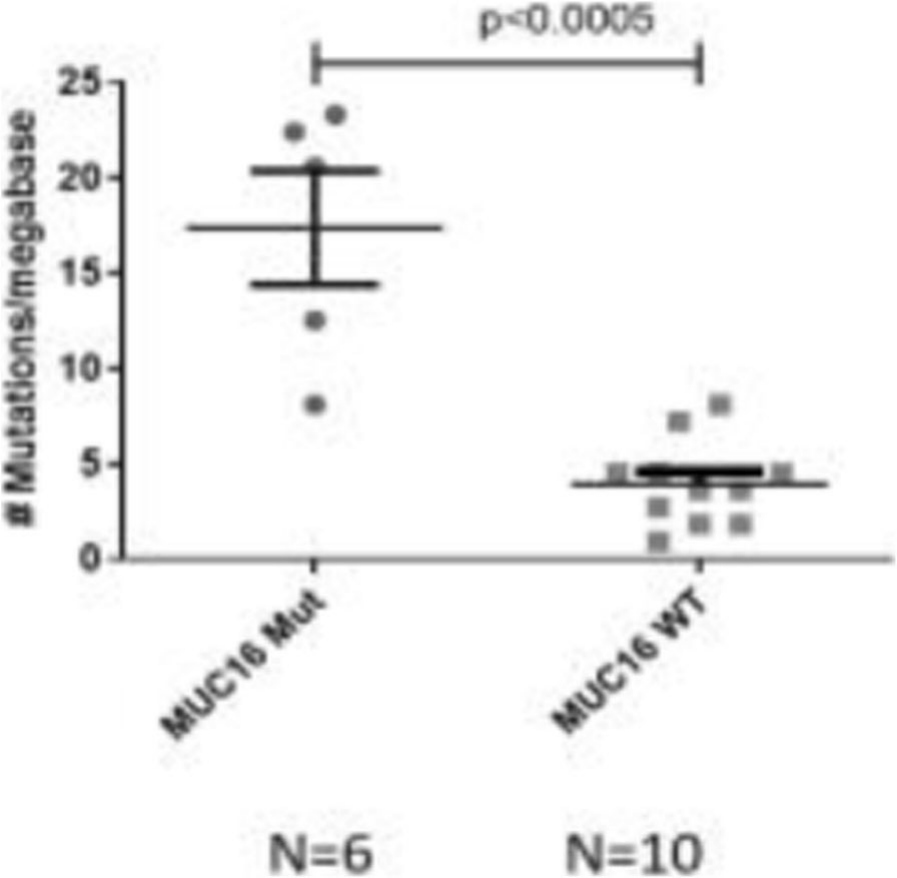
See text for description

#### P85 Development of a pan-cancer NGS assay for detection of tumor mutational burden and targeted biomarkers from FFPE samples

##### Dinesh Cyanam, MS, Vinay Mittal, Nickolay Khazanov, Paul Williams, Janice Au-Young, Gary Bee, Sameh El-Difrawy, Aren Ewing, Jennifer Kilzer, Anelia kraltcheva, PhD, Scott Myrand, MS, Yu-Ting Tseng, Cristina Van Loy, Elaine Wong-Ho, Chenchen Yang, Dinesh Cyanam, MS, Santhoshi Bandla, Warren Tom, PhD, Seth Sadis

###### Thermo Fisher Scientific, Ann Arbor, MI, United States

####### **Correspondence:** Seth Sadis (Seth.Sadis@thermofisher.com)

**Background**

Next-generation sequencing (NGS) is being applied to support routine clinical research in oncology with a primary focus on evaluating known oncogenic variants. However, the advent of cancer immunotherapies requires that clinical research solutions must also address biomarkers such as Tumor Mutational Burden (TMB) and Microsatellite Instability (MSI) for immune checkpoint inhibitors. Therefore, we developed a research use NGS solution for FFPE tissues that expanded upon our current Oncomine Tumor Mutation Load Assay by measuring biomarkers for both targeted and immune checkpoint therapies.

**Methods**

Gene content was prioritized based on the relevance and variant prevalence of biomarkers in solid tumors. Additional genomic regions were added to supplement the coding sequence footprint to support TMB. The assay used Ion AmpliSeq™ technology with automated templating on the Ion Chef™ system and sequencing on the Ion GeneStudio™ S5 sequencing platform. An automated tumor-only workflow for variant calling, TMB and MSI estimation and sample quality reporting was provided within Ion Reporter Software. Decision support tools were used for variant interpretation and evaluation of potential variant relevance.

**Results**

Over 500 genes with known DNA and RNA alterations were included. The panel has broad capability for variant calling, fusion detection, MSI status, in addition to TMB. Specifically, for TMB, DNA repair pathways were comprehensively represented as alterations in these genes may lead to high mutation burden. A coding sequence footprint to support TMB was generated. In development studies, the assay displayed high uniformity and consistent read depth to support robust variant calling. The automated workflow required minimal input of FFPE tumor only DNA and RNA material. Sample to report turnaround time was less than five days. In-silico assessment of TMB using publicly available whole-exome cancer sequencing data resulted in high correlation (R2 = 0.902, 0-40 mutations/Mb) and was parallel to the performance of our existing Oncomine Tumor Mutation Load Assay (R2 = 0.901, 0-40 mutations/Mb). Empirical analysis and performance of the assay on a common set of cell lines based on a universal reference standard also resulted in a positive correlation.

**Conclusions**

A larger tumor only NGS assay was developed to support comprehensive genomic profiling and routine clinical research in oncology. The assay design and informatics workflow support characterization of mutational signatures and provide normalized TMB estimates. Minimal input material requirement and rapid sample to report time will have a high impact on clinical research. More detailed information on the assay and an update on performance will be presented.

#### P86 Predictive Immune Modeling enables biomarker discovery in NSCLC patients treated with second line immunotherapy

##### Natalie LaFranzo, PhD^1^, Steve Daniel, PhD^1^, Walt Carney, PhD^2^, Milan Bhagat^3^, Natalie LaFranzo, PhD^1^

###### ^1^Cofactor Genomics, St. Louis, MO, United States; ^2^Walt Carney Biomarkers Consulting, LLC, Boston, MA, United States; ^3^TriStar Technology Group, LLC, Washington, DC, United States

####### **Correspondence:** Natalie LaFranzo (natalie_lafranzo@cofactorgenomics.com)

**Background**

While cancer checkpoint inhibitors have garnered much attention due to their ability to generate durable responses and improved survival, the actual number of patients who are eligible for, receive treatment with, and respond to these therapies remains modest [1]. This is driven by a dependency on legacy diagnostics, built on single-analyte biomarkers such as PD-L1, which have failed to capture the complexity of disease [2]. Even in the case of non-small cell lung cancer (NSCLC), an indication where the benefit of IO therapies is considered significant, there is much to be learned about the biology of the patients who respond, or do not respond to these therapies.

**Methods**

Multidimensional RNA models have emerged to move beyond these legacy methods to reveal the full scope of disease complexity, resulting in increased predictive accuracy. Leveraging a database of gene expression models built using Predictive Immune Modeling, immune context of the tumor microenvironment is quantified. In this study, a cohort of NSCLC patients who received second-line immunotherapies (checkpoint inhibitors) were evaluated retrospectively. Pre-treatment solid tumor FFPE tissue samples were processed using the ImmunoPrism immune profiling assay to generate comprehensive, individual immune profiles. Pathological, demographic, and survival data (including overall survival and progression-free survival, indicative of therapy response), was used to group patients for predictive biomarker discovery.

**Results**

Individual immune profiles of the patients are compared, both within and between relevant cohorts, and statistically-significant biological signals are reported. Machine-learning derived multidimensional biomarkers were also generated, which are defined by the optimal combination of all analytes measured in the assay, enabling improvements in predictive accuracy. This study represents the first data generated using the ImmunoPrism assay with patients receiving checkpoint inhibitor therapies.

**Conclusions**

Predictive Immune Modeling enables us to build multidimensional models of disease. When combined with well-curated patient cohorts, such as the NSCLC patients described here, predictive biomarkers may developed which capture more facets of the complex immune contexture than previously possible.

**References**

1. Haslam A, Prasad V. Estimation of the Percentage of US Patients With Cancer Who Are Eligible for and Respond to Checkpoint Inhibitor Immunotherapy Drugs. JAMA Netw Open. 2019 May 3;2(5):e192535.

2. Nishino M, Ramaiya NH, Hatabu H, Hodi FS. Monitoring immune-checkpoint blockade: response evaluation and biomarker development. Nat Rev Clin Oncol. 2017 Nov;14(11):655-668.

**Ethics Approval**

The human tissue samples utilized for this study were provided by TriStar Technology Group and have written, informed donor consent permitting academic and commercial research for publication, as well as approval from a competent ethical committee.

#### P87 All-in-One, quantitative immune repertoire profiling of PBMC and FFPE for renal cancer treatment evaluation

##### Mollye Depinet, MS^1^, Wenjing Pan, PhD^1^, Sang-gin Wu, MD, PhD^2^, Xiaohong Hou, MD PhD^1^, Brittany Brown, BS^1^, Mary Eisenhower, BS^1^, Daniel Weber^1^, Miranda Byrne-Steele, PhD^1^, Michael Lotze^3^, Jian Han, MD PhD^1^

###### ^1^iRepertoire, Huntsville, AL, United States; ^2^National Taiwan University, Taipei City, Taiwan, Province of China; ^3^UPMC Hillman Cancer Center, Pittsburgh, PA, United States

####### **Correspondence:** Jian Han (jhan@irepertoire.com)

**Background**

Next generation sequencing of the immune repertoire is a comprehensive immune profiling methodology that allows detailed, sequence-specific insight into the adaptive immune response. While immune repertoire analysis of bulk RNA typically focuses on a single receptor chain, understanding of the variable rearrangements of the immune repertoire as a whole provides a broader view of the immune landscape with potential prognostic value. This is accomplished through the study of all seven TCR and BCR chains together (i.e., TCR-alpha, TCR-beta, TCR-delta, TCR-gamma, and BCR-IgK and -IgL). One of the key challenges during immune receptor amplification is the formation of dimers, which can compete with the immune amplicons of interest during library preparation.

**Methods**

We therefore developed a novel PCR technique, dimer avoided multiplex PCR (dam-PCR), that effectively avoids dimer formation during PCR and incorporates unique molecular identifiers for direct RNA quantification and error removal. With one sample, dam-PCR allows for the amplification of all seven TCR and BCR loci in a single, quantitative multiplex reaction. Here, we apply this method to the amplification of both PBMC and FFPE RNA from renal cancer patients undergoing treatment.

**Results**

We found that both TCR-alpha and -beta diversity prior to treatment along with the expression ratio between B cells and T cells are good predictors of treatment efficacy.

**Conclusions**

Our study suggests that examining multi-chain immune repertoire composition can be valuable for predicting treatment response and evaluating treatment protocols. Additionally, this method shows promise for future applications in both clinical settings and basic research, as it allows for a cost effective, all-inclusive, and quantitative immune-profiling analysis of immune repertoires from a range of sample types, including FFPE, where sample RNA may be both limited in quantity and degraded in quality.

**Ethics Approval**

This study was approved by the University of University of Pittsburgh's Ethics Board.

#### P88 Evaluation of a tumor-only pan-cancer targeted semi-conductor based next-generation sequencing (NGS) test for microsatellite instability in FFPE samples

##### Sameh El-Difrawy, Ph D^1^, Anelia kraltcheva, PhD^2^, Vinay Mittal^2^, Elaine Wong-Ho^2^, Dinesh Cyanam, MS^2^, Seth Sadis^2^, Jennifer Kilzer^2^, Cristina Van Loy^2^, Janice Au-Young, PhD^2^, Aren Ewing^2^, Sameh El-Difrawy^2^

###### ^1^ThermoFisher Scientific, South San Francisco, CA, United States; ^2^Thermo Fisher Scientific, Carlsbad, CA, United States

####### **Correspondence:** Sameh El-Difrawy (Sameh.El-Difrawy@thermofisher.com)

**Background**

Comprehensive genomic profiling using next-generation sequencing (NGS) has become an essential tool to support routine clinical research in oncology. Advent of cancer immunotherapies also requires assessment of immune checkpoint inhibitor biomarkers such as microsatellite instability (MSI) and tumor mutational burden (TMB).

MSI arises from defects in the mismatch repair (MMR) system and is associated with hypermutability of short DNA sequence repeats, microsatellite locations, throughout the genome. Such defects are commonly observed in colorectal, gastric and endometrial cancers and have been shown to be predictive of response to immunotherapy treatment. Traditionally MSI testing has been done using single biomarker tests such as PCR/fragment analysis or immunohistochemistry (IHC) that require high sample input and are time consuming. Therefore, we developed an RUO NGS solution appropriate for FFPE tissues that addresses biomarkers for targeted and immune checkpoint therapies.

**Methods**

The performance of our RUO NGS based MSI approach was tested in the context of a large Ion AmpliSeq™ panel composed of more than 13,000 amplicons covering 500+ genes. The content includes a diverse set of microsatellite markers targeting MSI locations comprised of mono- and di-nucleotide repeats that range from 7 to 34 bp. Sequencing was carried out on the Ion 550™ chip and the Ion GeneStudio™ S5 system. In-sample standards were designed and incorporated as internal references utilized by the analysis pipeline and a novel algorithm was developed that leverages the unique signal processing properties inherent in semi-conductor sequencing. The test provides results for individual microsatellites and generates an MSI score and status for the sample of interest.

**Results**

The performance of the MSI solution was tested using a set of over 400 FFPE and cell-line samples from different tissue types and showed excellent concordance with orthogonal tests. We report on the sensitivity and specificity of our tumor only approach and propose ideas to utilize generated MSI score in combination with other bio markers.

**Conclusions**

An NGS assay was developed to support comprehensive genomic profiling and routine clinical research in oncology. The assay design and unique informatics workflow support precise characterization of mutational signatures and provides normalized MSI and TMB estimates. The performance of the assay was verified over a large cohort of colorectal, gastric and endometrial cancer samples with MSI status independently assigned by orthogonal tests. [For Research use Only. Not for use in diagnostic procedures]

#### P89 Obesity related changes in AXL-driven inflammatory signaling impact survival in melanoma

##### Alicia Gingrich, MD, Kylie Abeson, BS, Alexander Merleev, PhD, Robert Canter, MD, MAS, FACS, Emanual Maverakis, Amanda Kirane, MD, Alicia Gingrich, MD

###### University of California, Davis, Sacramento, CA, United States

####### **Correspondence:** Alicia Gingrich (agingrich@ucdavis.edu)

**Background**

The TYRO3, AXL and MERTK (TAM) receptor tyrosine kinase (RTK) family have been associated with a number of human cancers, including melanoma.[1-3] Effects attributed to oncogenesis and metastasis (epithelial-to-mesenchymal transition) of the TAM receptors have been described.[2] Recent evidence correlating obesity with a paradoxical improved response to immunotherapy in melanoma suggests both tumor microenvironment and clinical phenotype play a role in response.[4] Therefore, we sought to build a predictive model of response to therapy from biomarkers, using TAM receptors and conventional markers of checkpoint inhibition such as PD-1. This model was tested in the normal weight, overweight and obese populations.

**Methods**

TCGA-SKCM melanoma tumor mRNA expression and clinical data for metastatic melanoma patients were downloaded from the GDC legacy archive (https://portal.gdc.cancer.gov/legacy-archive) (n = 471).[5] Biomarkers were defined as “high” or “low” expression in each patient. Differences in Kaplan-Meier survival curves based on level of expression were tested using G-rho family tests. Strength of relationships between biomarkers were measured using Pearson’s correlation. All statistical analysis were performed using R package “survival”.

**Results**

Normal weight, overweight and obese patients had markedly different biomarker profiles associated with survival (Figure 1). In the normal weight population, high CD8 (p=0.0093), PD1 (p=0.0093) and CD84 (p=0.022) were associated with improved survival. In the overweight population, high CD8 (p=0.0098), PD1 (p=0.0004) and CD84 (p=0.0081) were associated with improved survival, while high Gas6 (p=0.029) and MERTK (p=0.043) were associated with decreased survival. And in the obese population, high AXL expression was associated with improved survival (p=0.004), while CD8 (p=0.91) and PD1 (p=0.89) demonstrated no association. In correlation analysis, AXL expression was most closely associated with macrophage markers CD163 (r=0.52), CD84(r=0.56) and MS4A4A(r=0.53) in the obese but not the normal weight population.

**Conclusions**

Taken together, these data suggest that immunologic response in metastatic melanoma patients is driven by separate immune profiles for obese and non-obese populations. AXL appears to mediate response in the obese population by a macrophage-driven mechanism as opposed to T cell mediation. Collectively, the significant differences in the transcriptomic profiles between obese and non-obese patients suggest potential clinical implications regarding targets for treatment and application to patients based on clinical phenotype.

**References**

1. Dransfield I, Farnworth S. Axl and Mer receptor tyrosine kinases: distinct and nonoverlapping roles in inflammation and cancer? Adv Exp Med Biol. 2016;930:113-32.

2. Verma A, Warner SL, Vankayalapati H, et al. Targeting Axl and Mer kinases in cancer. Mol Cancer Ther. 2011;10:1763-73.

3. Wu X, Liu X, Koul S, et al. AXL kinase as a novel target for cancer therapy. Oncotarget. 2014;5:9546-9563.

4. McQuade JL, Daniel CR, Hess KR, et al. Association of body-mass index and outcomes in patients with metastatic melanoma treated wtih targeted therapy, immunotherapy, or chemotherapy: a retrospective, multicohort analysis. Lancet Oncol. 2018;19:310-322.

5. Guan J, Gupta R, Filipp FV. Cancer systems biology of TCGA SKCM: efficient detection of genomic drivers in melanoma. Sci Rep. 2015;5:7857.

**Fig. 1 (abstract P89).**
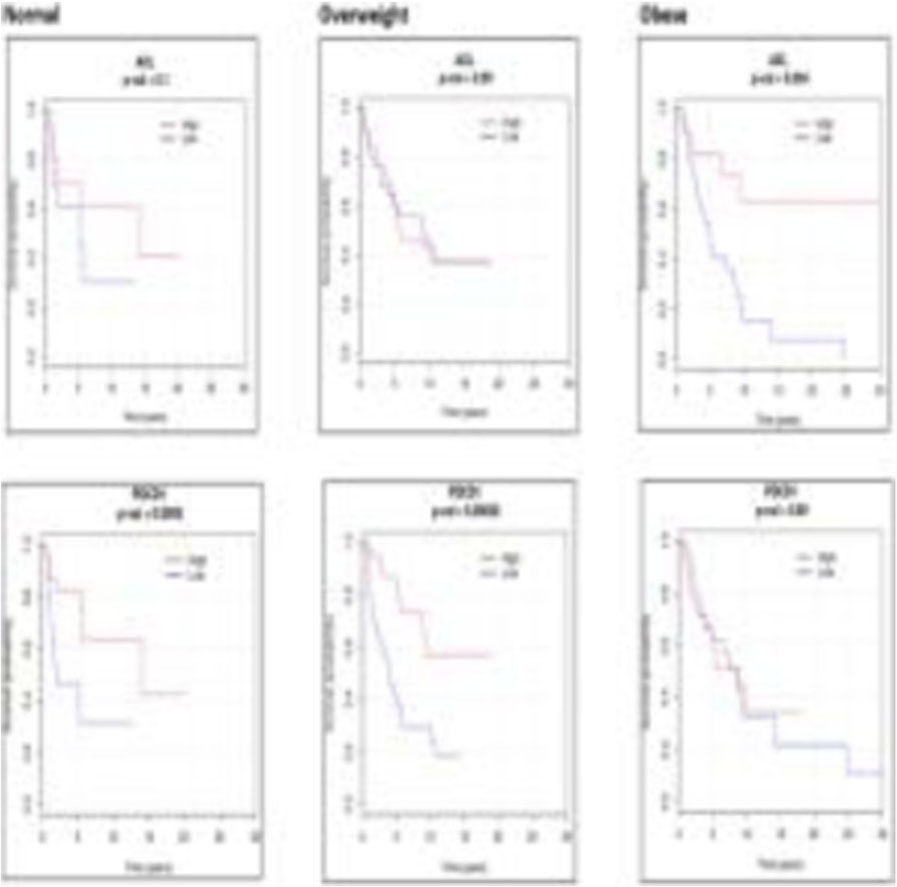
KM curves by clinical phenotype and biomarker

#### P90 Unique tumor immune microenvironments of potentially PD-L1/TGF-β trap responsive tumors

##### Sean Glenn, PhD, Sarabjot Pabla, MSc, PhD, BS, Erik Van Roey, Jonathan Andreas, MS, Blake Burgher, BS, RN, Jeffrey Conroy, BS, Mary Nesline, MS, Antonios Papanicolau-Sengos, MD, Vincent Giamo, BS, MS, Felicia Lenzo, Yirong Wang, MS, Carl Morrison, MD, DVM

###### OmniSeq, Inc., Buffalo, NY, United States

####### **Correspondence:** Sean Glenn (sean.glenn@omniseq.com)

**Background**

Tumors often do not respond to PD-1/PD-L1 axis inhibitors due to immune escape mechanisms present in the tumor microenvironment. Bi-functional antibody-based immunotherapies that simultaneously target immune checkpoints and immunosuppressive cells are being developed to slow tumor growth. Anti-PD-L1/ TGF-β trap fusion proteins are one approach being tested to counter the traditional immune checkpoint inhibition via PD-1/PD-L1 axes and simultaneously inhibit the pro-tumor/anti-inflammatory effects of TGF-β. In this study, we not only describe the tumor immune microenvironment of tumors expressing PD-L1 and TGF-β, but also describe potential patient selection strategies based on gene expression measurements of these tumor immune microenvironments from clinical samples.

**Methods**

RNA-seq was performed for 395 immune transcripts on 1323 FFPE tumors of diverse histologies. To find true TGF-β high expressing tumors, TGF-β gene expression was normalized by a tumor inflammatory score (average expression rank of 161 inflammation genes derived from co-expression signature of 1323 tumors spanning 35 tumor histologies). Proportion of PD-L1 IHC positive, tumor mutational burden (TMB) high and cell proliferation categories was estimated for TGF-β high expressing tumors. Inclusion and exclusion criteria were developed based on PD-L1 and normalized TGF-β expression.

**Results**

Gene expression revealed varying degrees of TGF-β high tumors in all tumor types studied. Sarcoma, pancreatic cancer and breast cancer had the highest proportion of TGF-β high tumors. Within these TGF-β high tumors, 41% were PD-L1 IHC+ (TPS≥1%), and 28% were TMB-high. 11% (n=147/1323) tumors were both TGF-β high and PD-L1 high making these tumor microenvironments ideal for a potential PD-L1/TGF-β trap treatment. Interestingly, 47% (n=69/147) of these tumors presented with strong/moderate inflammation, with 53% (n=78/147) being non-inflamed tumors. Conversely, there were 11.7% (n=155/1323) tumors that were TGF-β low and PD-L1 low presenting suboptimal tumor micro-environment for a potential treatment. Notably, only 26% (n=40/155) of these tumors presented with strong/moderate inflammation with clear majority (74%; n=115/155) being strongly or moderately inflamed tumors.

**Conclusions**

This large clinically tested tumor cohort suggests an immune phenotype of potentially PD-L1/TGF-β trap responsive tumors exists across multiple histologies. PD-L1/TGF-β high tumors have distinct immune profiles compared to PD-L1/TGF-β low tumors. A clinical immune gene expression assay described in this study could not only improve patient selection for anti-PD-L1/TGF-β trap treatment, but for other bi-specific fusion protein based immunotherapies.

**Ethics Approval**

De-identified specimens and data were analyzed by OmniSeq under IRB approved protocol BDR 080316 (Roswell Park Comprehensive Cancer Center, Buffalo, NY).

#### P91 Expression profiling of T cells using nanoscale automation with a full-length RNA sequencing library preparation kit on a microfluidic circuit platform

##### Thomas Goralski, PhD^1^, Sangpen Chamnongpol^1^, Michael Phelan^1^ , Jennifer Snyder-Cappione^2^, Julie Alipaz^1^, Joel Brockman^1^, Brian Fowler^1^, Jennifer A. Geis^1^, Christopher Kubu^1^, Raphael Kung^1^, Benjamin Lacar^1^, Naveen Ramalingam, PhD^1^, Mandi Wong^1^, Charles Park^1^, David King^1^

###### ^1^Fluidigm Corp, South San Francisco, CA, United States; ^2^Boston University School of Medicine, Boston, MA, United States

####### **Correspondence:** Christopher Kubu (chris.kubu@fluidigm.com)

**Background**

RNA sequencing (RNA-seq) provides hypothesis-free profiling of transcript levels and isoforms. This profiling captures a comprehensive view of the peripheral immune system or the tumor microenvironment. The resulting profiles can be used to characterize differential gene expression patterns that can further the understanding of the immune system. To further enable these types of studies we have developed a highly cost-effective, nanoliter-volume microfluidics-based workflow and chemistry compatible with Illumina® sequencing instruments to simultaneously generate RNA-seq libraries from up to 48 samples. This method fully automates solid-phase capture of polyadenylated RNA, reverse transcription, and index PCR within a compact nanoscale integrated fluidic circuit (IFC) on our Juno™ system. The workflow includes reagents necessary to generate full-length, random-primed RNA-seq libraries from as little as 10 ng of total RNA, while preserving strandedness information.

**Methods**

Multiple replicates of 10 ng and 100 ng of total RNA from control samples spiked with ERCC RNA Spike-In Mixes were used to prepare RNA-seq library using the Advanta™ RNA-Seq NGS Library Prep Kit. The performance was compared to a conventional library preparation kit. We also used our platform to profile total RNA purified from FACS-sorted CD3+, CD8+, CD28–, and CD25+/hi T suppressor cells.

**Results**

RNA-seq libraries from control RNAs at both 10 and 100 ng input have less than 10% rRNA reads, replicate correlations greater than 99%, and gene-level and transcript-level detection rates that are highly concordant with a conventional library preparation kit. Additionally, the data confirms comparable dynamic range and linearity of response of the ERCC spike-in controls. Libraries prepared from FACS-sorted T cells show differential expression profiles consistent with the expected patterns.

**Conclusions**

The Advanta RNA-Seq NGS Library Prep workflow simplifies the high-throughput generation of RNA-seq libraries, significantly minimizing hands-on time and costly reagent consumption, which will facilitate the incorporation of RNA sequencing into the immune-oncology research toolkit.

For Research Use Only. Not for use in diagnostic procedures.

#### P92 Survival benefits of comprehensive genomic profiling and treatment in metastatic non-small cell lung cancer

##### Alison Sexton Ward, PhD^1^, Jennifer Johnson, MD^2^, Komal Gupte-Singh, PhD^3^, Mohammad Ashraf Chaudhary, PhD^3^, Devender Dhanda, PhD^3^, Oliver Diaz, PhD^1^, Katherine Batt, MD, MSc^1^, John Fox^4^

###### ^1^Precision Health Economics, Oakland, CA, United States; ^2^Jefferson University Hospital, Philadelphia, PA, United States; ^3^Bristol-Myers Squibb, Princeton, NJ, United States; ^4^Priority Health, Grand Rapids, MI, United States

####### **Correspondence:** Komal Gupte-Singh (Komal.Singh@bms.com)

**Background**

Metastatic non-small cell lung cancer (mNSCLC) patients who receive comprehensive genomic profiling (CGP) at diagnosis may be more likely to receive optimal first line (1L) therapies than patients who receive panel testing (PT) with the enhanced ability to identify biomarkers with associated therapies. The incremental survival benefits of receiving optimal treatments following CGP testing at diagnosis have yet to be estimated.

**Methods**

A Markov simulation model of biomarker testing and treatment assignment for mNSCLC was built to estimate the survival outcomes associated with CGP versus PT. Biomarkers identified with PT were EGFR, ALK, ROS1, BRAF, and PD-L1 (≥50%). All biomarker tests were tested simultaneously using single gene testing or assay. CGP, which employed Next-Generation Sequencing, identified all the above biomarker changes and estimated tumor mutational burden (TMB). The model assumed that PD-L1 testing was conducted together with CGP. Biomarker identification, except for TMB and PD-L1, was assumed to be mutually exclusive and to occur at published prevalence rates. Incremental false-negative rates of each genetic test in PT relative to CGP were applied. Treatment pathways followed NCCN guidelines and current published clinical trial results. Key inputs and assumptions were tested in sensitivity analyses.

**Results**

Patient overall survival for each biomarker test within each testing strategy are shown in Table 1. Patients receiving CGP had 8.5% (1.4 months) longer survival on average than those who received PT. Patients receiving CGP testing at presentation spent more time on 1L therapies (40% vs. 33%), thereby less time on 2L therapies (23% vs. 26%) compared to patients receiving PT at presentation.

**Conclusions**

CGP testing among mNSCLC patients at the time of diagnosis resulted in survival gains in comparison to PT due to higher proportion of patients receiving optimal 1L treatment.

**Table 1 (abstract P92).**
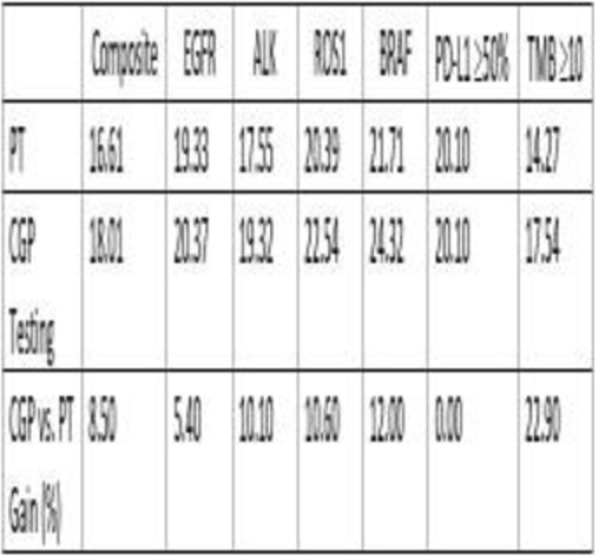
Overall survival (months) by testing strategy & biomarker

#### P93 The advanced immune-centric NGS cohort for colon cancer

##### Wouter Hendrickx, PhD^1^, Jessica Roelands, Master^1^, Peter Kuppen^2^, Francesco Marincola, MD^3^, Najeeb Syed^1^, Davide Bedognetti, MD, PhD^1^

###### ^1^Sidra Medicine, Doha, Qatar; ^2^Leiden University Medical Center, Leiden, Zuid-Holland, Netherlands; ^3^Refuge Biotechnologies, Half Moon Bay, CA, United States

####### **Correspondence:** Davide Bedognetti (dbedognetti@sidra.org)

**Background**

The immune system has a substantial effect on the progression of colon cancer. Typically, an immune response defined by a polarized Th1 phenotype, characterized by expression of chemokine-receptor ligands, activation of interferon-stimulated genes, production of cytotoxic molecules by effector immune cells, and upregulation of immune regulatory genes, has been associated with immune-mediated tumor rejection. We have previously introduced a gene signature, called Immunology Constant of Rejection (ICR), that reflects these immune components.[1–4] This signature was able to differentiate quite well the patients with an active immune environment and improved survival vs those who did not[5].

Virtually, all correlative analyses integrating exome and transcriptomic data in colon cancer based on publicly available date use the TCGA cohort. Although it is broadly accepted that T-cell infiltration influences prognosis in colon cancer[6], the association between transcriptomic immune signature and patient survival could not be observed in the TCGA colon cancer cohort. This is likely due to the per protocol exclusion of samples with low tumor purity (i.e., higher stromal/immune infiltration), as at that time TCGA consortium focused on defining cancer genetic makeup. To gain more insight into the underlying mechanism of cancer tissue rejection by the immune system in colon cancer, we build an extensive data repository from high quality snap frozen colon cancer samples unbiased for tumor purity.

**Methods**

RNA and DNA were isolated from a cohort of 366 colon cancer patients collected over the last decade at the University of Leiden Medical Center (LUMC), Netherlands. Tissue sections flanking the corresponding samples were hematoxylin- and eosin-stained. RNA-seq (HiSeq4000) data was obtained using HISAT2 alignment[7] and quantile normalized after GC-correction of the raw counts.[8] Whole Exome Sequencing (WES) (>100X) was performed for normal and cancer tissue (366 RNA-seq and 608 WES). T-cell repertoire was analyzed using Adaptive immunoSEQ in 125 samples. Tumor immune phenotype classification was done using unsupervised consensus clustering based on the expression of ICR genes.

**Results**

We have built one of the most extensive high-quality datasets for immunogenomic alterations available so far in colon cancer. Our preliminary data supports a positive impact of ICR gene expression in colon cancer cohort: patients with a Th-1 polarized microenvironment display better survival. Integrative analysis encompassing somatic mutation, copy number variations, and transcriptome is ongoing and will be presented at the conference (Figure 1).

**Conclusions**

This newly generated immune centric NGS dataset, generated in Qatar, will contribute dramatically to elucidating the genetic determinants of immune responsiveness in cancer.

**Acknowledgements**

This work was supported by Qatar National Research Fund (QNRF) with grant JSREP07-012-3-005

**References**

1. Wang, E., Worschech, A. & Marincola, F. M. The immunologic constant of rejection. Trends Immunol. 29, 256–262 (2008).

2. Spivey, T. L. et al. Gene expression profiling in acute allograft rejection: challenging the immunologic constant of rejection hypothesis. J. Transl. Med. 9, 174 (2011).

3. Bertucci, F. et al. The immunologic constant of rejection classification refines the prognostic value of conventional prognostic signatures in breast cancer. Br. J. Cancer (2018). doi:10.1038/s41416-018-0309-1

4. Galon, J., Angell, H. K., Bedognetti, D. & Marincola, F. M. The Continuum of Cancer Immunosurveillance: Prognostic, Predictive, and Mechanistic Signatures. Immunity 39, 11–26 (2013).

5. Roelands, J. et al. Genomic landscape of tumor-host interactions with differential prognostic and predictive connotations. bioRxiv 546069 (2019). doi:10.1101/546069

6. Pagès, F. et al. International validation of the consensus Immunoscore for the classification of colon cancer: a prognostic and accuracy study. The Lancet 391, 2128–2139 (2018).

7. Kim, D., Langmead, B. & Salzberg, S. L. HISAT: a fast spliced aligner with low memory requirements. Nat. Methods 12, 357–360 (2015).

8. Risso, D., Schwartz, K., Sherlock, G. & Dudoit, S. GC-Content Normalization for RNA-Seq Data. BMC Bioinformatics 12, 480 (2011).

**Ethics Approval**

Sidra Medicine IRB approval : #1602002725

**Fig. 1 (abstract P93).**
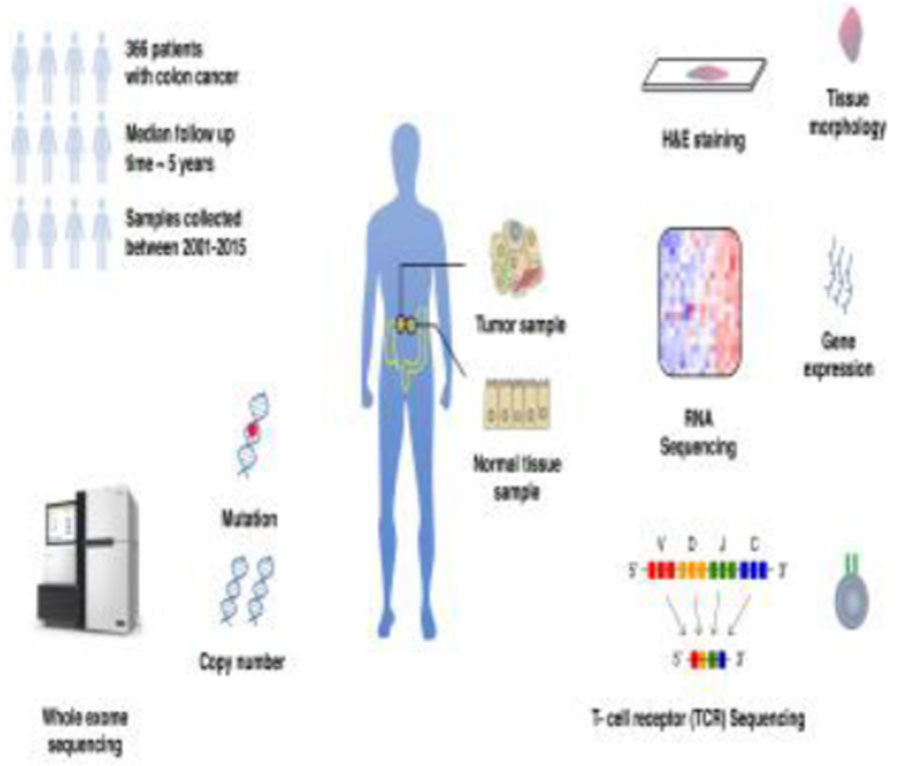
The advanced immune-centric NGS cohort for colon cancer

#### P94 A potential mechanism of anti-cancer immune response activated by immune-related adverse events (irAEs) in urological cancer patients

##### Taigo Kato, MD, PhD^1^, Motohide Uemura^1^, Koji Hatano^1^, Atsunari Kawashima^1^, Takeshi Ujike^1^, Kazutoshi Fujita^1^, Kazuma Kioytani^2^, Norio Nonomura^1^

###### ^1^Osaka University, Osaka, Japan; ^2^Japanese Foundation for Cancer Research, Tokyo, Japan

####### **Correspondence:** Taigo Kato (kato@uro.med.osaka-u.ac.jp)

**Background**

With the spread of usage of Immune checkpoint inhibitors (ICIs), a certain number of patients face discontinuation of ICIs due to severe immune-related adverse events (irAEs). Recently, some reports have shown encouraging efficacy among patients who discontinued ICIs, leading to the hypothesis that irAEs-experienced patients have strong and long-lasting anti-cancer immune responses. So far, the molecular mechanisms of the immune response, particularly for T cells that play pivotal roles in attacking cancer cells, still remain unclear. Thus, characterization of T cell repertoire and immune signatures in peripheral blood mononuclear cells (PBMCs) and tumors before and after ICIs treatment should contribute to better understanding of irAEs-related anti-cancer immune responses.

**Methods**

In this study, we collected PBMCs from 4 urological cancer patients, before ICIs treatment and at the onset of severe irAEs. For 1 kidney cancer patient who had long durable response after discontinuation of ICIs, we also collected metastatic tissue sample and applied a next generation sequencing approach to characterize T cell receptor (TCR) repertoires using RNAs isolated from tumors and PBMCs. We also measured mRNA expression levels of immune-related genes in the PBMCs of pre- and post-ICIs treatment.

**Results**

We found that elevated transcriptional levels of CD3, CD4, CD8, GZMA, PRF1, and FOXP3 along with high GZMA/CD3 and PRF1/CD3 ratio in the peripheral blood at the onset of irAEs. TCR repertoire analysis revealed drastic expansion of certain T cell clones in metastatic tissue after irAEs (Figure 1). Interestingly, some of these abundant TCR clonotypes were also increased in peripheral blood at the onset of irAEs (Figure 2).

**Conclusions**

Our findings revealed that a certain number of expanded- and irAEs-related T cell clones in cancer tissue may also circulate systemically and then attack tumor cells in distant regions, leading to durable response in the patients with irAEs.

**Fig. 1 (abstract P94).**
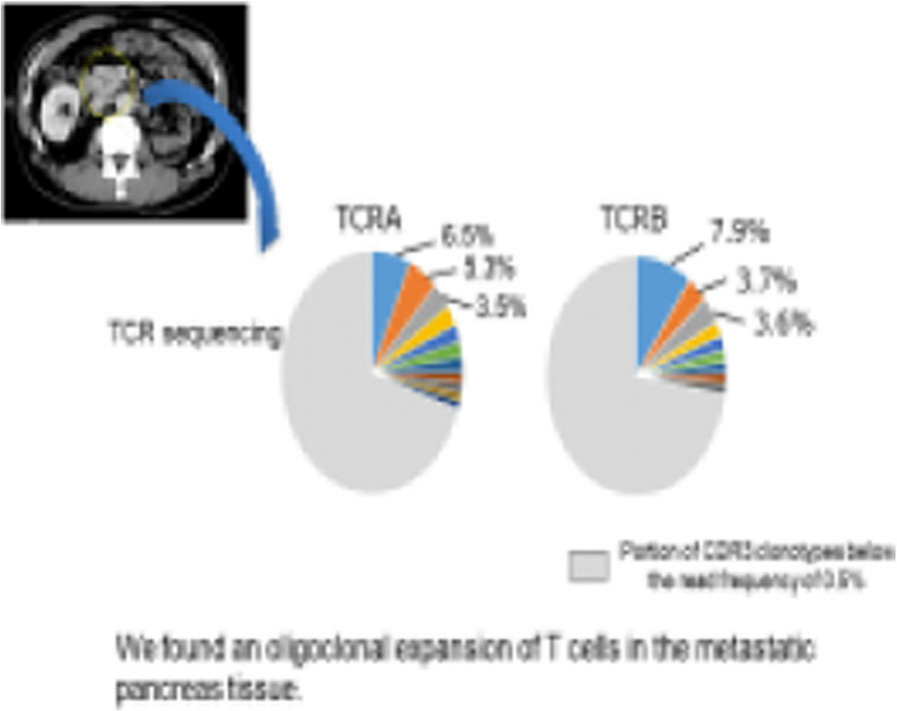
Clonal T cell expansion in pancreatic metastasis

**Fig. 2 (abstract P94).**
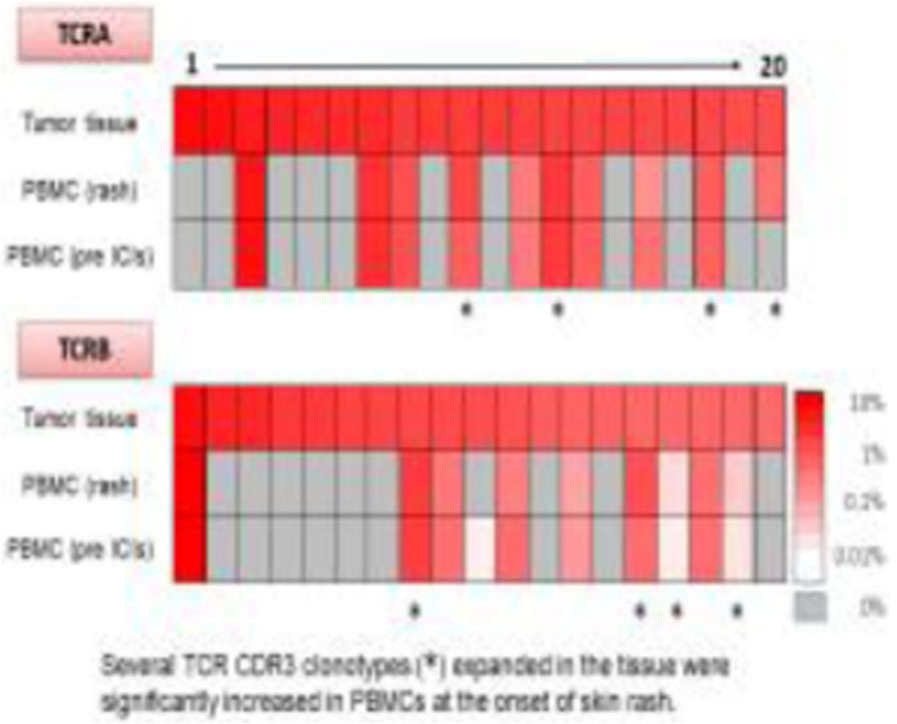
Expanded T cells in metastatic site are detected in systemic

#### P95 Single-cell RNA-sequencing from clinically relevant core needle biopsies for evaluation of tumor-immune cell interactions in the tumor microenvironment

##### Namit Kumar, PhD, Mohan Bolisetty, Peter Szabo, PhD, Xuan Li, Becky Penhallow, Ryan Golhar, Alice Walsh, Saumya Pant

###### Bristol-Myers Squibb, Princeton, NJ, United States

####### **Correspondence:** Namit Kumar (Namit.Kumar@bms.com)

**Background**

Elucidating biomarkers associated with immunotherapy response and resistance will allow for better informed patient selection and treatment decisions as well as enhanced drug development strategy. Current biomarker strategies are based on cellular markers (eg, immunohistochemistry) or bulk molecular averages (eg, whole-exome sequencing). However, there is limited ability to integrate cellular and molecular data. Single-cell RNAseq (scRNAseq) is a promising technology allowing for an unbiased analysis of the tumor microenviroment (TME) at cellular resolution. Despite the immense potential, implementation of this technology in clinical trials has been limited due to lack of methodologies applicable to clinically relevant specimens such as core-needle biopsies (CNB). Here, we describe the development of clinically applicable scRNAseq technology and analysis.

**Methods**

Treatment-naïve commercially sourced tumor resections were used to generate ex-vivo CNB for scRNAseq analysis with 10X genomics. Post-clustering, unsupervised cell-type identification was performed (SingleR), and downstream analyses were carried out (Seurat v2, custom R). Cells from multiple patients/tumor types (endometrial, TNBC, NSCLC, ccRCC, gastrointestinal; n=8), and healthy donors (peripheral blood mononuclear cells; n=3) were combined, batch-corrected and aligned using canonical correlation analysis (CCA); and differential gene expression was performed (MAST algorithm).

**Results**

CNB scRNAseq was optimized across 5 tumor types, and the resulting data from ~43,000 cells allowed for the unbiased identification of TME cellular components (stromal, epithelial, immune-cell subtypes). The cellular resolution of this dataset allowed us to identify cell populations with distinct gene signatures. For example, we identified 2 macrophage subclusters—a lung tumor-specific cluster and a tumor-independent cluster. Lung-specific macrophages showed upregulation of genes including SPP1, G0S2, RGCC, PHLDA1, and TREM. Differential gene expression analysis evaluated similarities and differences between TME vs healthy PB cells and allowed for surrogate pharmacodynamics marker assessment. In our analysis, 1197 genes were differentially expressed; the most enriched genes in tumor-derived monocytes included HSPA1A, IL8, APOE, and SPP1 whereas PB monocytes were enriched for genes including LGALS2, S100A12, S100A9, AHNAK, and CSTA.

**Conclusions**

We have demonstrated the feasibility of scRNAseq from single CNB through the development of protocols to enable identification of biomarkers related to pharmacodynamics, therapeutic response, or disease progression. Further, we have optimized the bioinformatics workflow to derive meaningful biological insights from these scRNAseq datasets, such as mechanisms involved in immune response or resistance that are tumor extrinsic or intrinsic. Our pilot study sets the groundwork to explore including scRNAseq in future prospective clinical studies.

**Acknowledgements**

Bristol-Myers Squibb.

#### P96 T-cell receptor alpha and beta repertoire profiling using an augmented transcriptome

##### Eric Levy, PhD, Pamela Milani, Sean Boyle, PhD, Gabor Bartha, Charles Abbott, PhD, Robert Power, Rena McClory, Robin Li, John West, MBA, Richard Chen

###### Personalis, Inc., Menlo Park, CA, United States

####### **Correspondence:** Richard Chen (richard.chen@personalis.com)

**Background**

The promise of immunotherapy has revealed the need for comprehensive profiling of the tumor and its immune microenvironment, including analysis of the T-cell receptor (TCR) repertoire. To address this challenge, we developed ImmunoID NeXT to provide a more comprehensive view of the tumor and tumor microenvironment (TME) from limited FFPE tumor biopsies. This includes profiling both the TCR alpha and beta chains. We show that ImmunoID NeXT accurately and reproducibly profiles abundant clones and provides information on the diversity of T-cells in tumor samples.

**Methods**

We first analyze the reproducibility of ImmunoID NeXT using replicates of PBMCs. Then, we compare the concordance of clones from ImmunoID NeXT to the top clones from a standalone TCR sequencing approach. We also analyze the reproducibility of clones in patient-derived FFPE samples, and compare to IHC quantification of CD3+ cells to highlight the intra-sample heterogeneity of T-cell abundance and diversity. We then analyze the clonal diversity of pre-treatment tumor samples in a cohort of melanoma patients who underwent PD-1 blockade. Finally, we use ImmunoID NeXT to profile the clonal diversity across over 100 solid tumor samples.

**Results**

Abundances of clones shared between replicates of PBMC samples have a very high concordance (R2>0.99 with both TRA and TRB). Compared to the standalone TCR approach, we identify over 96% of the top 1000 TRA clones, and over 99% of the top 1000 TRB clones, both with highly concordant abundances (R2>0.95 and R2>0.94 in TRA and TRB, respectively).

Subsequent curls of a tumor FFPE sample also have a high concordance of clonal abundances (R2>0.89 and R2>0.91 in TRA and TRB, respectively). TCR sequencing also provides a view of the clonal diversity of T-cells in a sample, which is not available with quantification via IHC. Finally, in a melanoma cohort, clonality based on either TRA or TRB is significantly different in responders to checkpoint inhibition.

**Conclusions**

The ImmunoID NeXT platform can provide insight into the diversity of the immune repertoire, highlighting the platform’s ability to provide comprehensive analysis of both the tumor and tumor microenvironment. We demonstrate that ImmunoID NeXT is reproducible, sensitive, and accurate at profiling high-abundance TRA and TRB clones, as well as feasible with FFPE samples. We also highlight how immune repertoire results from ImmunoID NeXT can be used to gain understanding about the immunological composition of the TME. Finally, we show how ImmunoID NeXT can profile the diversity of the TCR repertoire in tumor samples.

#### P97 TCRB repertoire convergence and clonal expansion define the NSCLC tumor microenvironment of responders to anti-PD-1 monotherapy

##### Timothy Looney, PhD^1^, Katharina Leonards^2^, Ilaria Alborelli^2^, Luca Quagliatta^1^, Philip Jermann^2^

###### ^1^Thermo Fisher Scientific, Austin, TX, United States; ^2^University of Basel, Basel, Switzerland

####### **Correspondence:** Philip Jermann (philipmartin.jermann@usb.ch)

**Background**

There is an outstanding need to identify predictive biomarkers for response to anti-PD-1 monotherapy for NSCLC. Here we investigated TCRB clonal expansion and TCR convergence within the pretreatment tumor microenvironment as predictors of response in a cohort of 37 FFPE-preserved biopsies. For context, we compared the predictive value of these features with TMB values from the same tumors.

**Methods**

Total RNA from FFPE-preserved pretreatment NSCLC biopsies (11 responders, 14 non-responders) was extracted for TCRB repertoire sequencing via the Oncomine TCRB-SR assay (15-265ng RNA input; average 164ng) and the Ion Torrent Gene Studio S5. TMB values were obtained from FFPE-preserved gDNA from the same biopsies using the Oncomine Tumor Mutation Burden Assay. TCR convergence and clonal expansion were evaluated independently or in a combined model as predictors of response.

**Results**

TCRB sequencing revealed increased TCR convergence (p = .02, Wilcoxon) and clonal expansion (p = .06, Wilcoxon) in those who benefited from anti-PD-1 therapy. A logistic regression classifier combining both features was able to discriminate responders from non-responders with a sensitivity of .91 and specificity of .71 at the optimal cutoff, per the Youden’s J method. The TCR-based classifier was able to identify responders who otherwise had low to intermediate (<10muts per Mb) TMB.

**Conclusions**

TCRB clonal expansion and convergence warrant further evaluation as potential predictive biomarkers of response. Importantly, TCRB sequencing may allow for identification of responders who are otherwise missed by TMB-based stratification.

#### P98 Automated rarefaction analysis for precision human and mouse B and T cell receptor repertoire profiling from peripheral blood and FFPE-preserved specimens

##### Timothy Looney, PhD, Geoffrey Lowman, PhD, Michelle Toro, Jayde Chang, Denise Topacio-Hall, BS, MA, Loni Pickle, PhD, Fiona Hyland, Timothy Looney, PhD

###### Thermo Fisher Scientific, Austin, TX, United States

####### **Correspondence:** Timothy Looney (timothy.looney@thermofisher.com)

**Background**

Identifying the optimal input amount and sequencing depth for B and T cell receptor repertoire profiling is challenging owing to variation in material quality and lymphocyte diversity in blood and FFPE preserved specimens. Rarefaction analysis has emerged as a potential approach for assessing whether immune repertoire libraries have been sequenced to saturation. Here we present a novel automated method for saturation analysis of IGH and TCRB chain libraries derived from sequencing of peripheral blood leukocytes (PBL) and FFPE-preserved RNA and DNA.

**Methods**

Human TCRB and IGH repertoire libraries were generated using the Oncomine TCRB-SR and BCR IGH-SR assays from: (1) 25ng PBL total RNA (2) 500ng PBL gDNA (3) 150ng RNA from FFPE preserved NSCLC and (4) 200ng gDNA from FFPE preserved brain tissue. Mouse TCRB and IGH libraries were generated using the Ion Ampliseq TCRB-SR and BCR IGH-SR assays and 25ng RNA or 500 ngDNA derived from spleen or lymph node. Libraries were sequenced on the Ion Torrent Gene Studio S5 then analyzed with Ion Reporter to identify clonotypes, quantify clonal expansion and diversity, and for IGH chain libraries, identify B cell clonal lineages and assess isotype usage. We then repeated clonotyping and analysis of secondary repertoire features using data that had been downsampled to fixed read depths.

**Results**

We observed an asymptotic relationship between the sequencing depth and the number of B and T cell clones detected, clone Shannon diversity, and B cell clonal lineage richness and diversity, indicating that libraries had been sequenced to saturation. By contrast, T and B cell normalized Shannon entropy appeared robust to sequencing depth.

**Conclusions**

Automated downsampling analysis may serve as a convenient tool for optimizing sequencing depth and input amount for B and T cell repertoire sequencing studies. We expect this approach to become a routine component of immune repertoire analysis.

#### P99 TMBler: a bioinformatic tool for measuring and optimizing Tumor Mutational Burden quantification from targeted sequencing panels

##### Laura Fancello, Luca Mazzarella, MD PhD, Alessandro Guida, Arnaud Ceol, Piergiuseppe Pelicci, Luca Mazzarella, MD PhD

###### IEO Istituto Europeo di Oncologia IRCCS, Milano, Italy

####### **Correspondence:** Luca Mazzarella (luca.mazzarella@ieo.it)

**Background**

Tumor mutational burden (TMB) is increasingly proposed as a predictive biomarker for immunotherapy response in cancer patients.

TMB assessed by Whole Exome Sequencing (WES) is considered the gold standard but remains confined to research settings. Targeted enrichment panels of various genomic sizes are emerging as a more sustainable methodology for assessing TMB in the clinical setting. However, panel-based TMB quantification has not been adequately standardized to date, leading to major heterogeneities in TMB measurement and a lack of uniformly accepted cutoff values, thus limiting the possibility to transfer results across settings. In particular, the choice of variants to include in TMB calculation (synonymous, cancer driver genes or low-allelic frequency mutations, or other features) may strongly affect results and in particular TMB predictive value [1]

**Methods**

We developed "TMBler", an R package to calculate TMB from targeted sequencing panels. TMBler allows to select multiple filters on mutation counts for TMB quantification. It also includes a set of functions to simulate custom panels on WES and calculate predictive value based on available data on immunotherapy response matched with sequencing data [2,3]. Finally, it allows to measure panel-based TMB concordance with WES-based TMB and its predictive value using Receiver Operating Characteristic (ROC) curves.

**Results**

By simulating custom and commercially available panels, we show that the application of specific filter combinations can significantly influence TMB calculation and its predictive value, and we identify instances where risk of erroneous assignment of patients to responder/nonresponder groups is highest

**Conclusions**

TMBler is a useful tool for quantifying TMB from targeted panels. It can analyze performance of existing panels, optimize analytical pipeline and design novel custom panels through simulations.

**References**

1. Fancello L, Gandini S, Pelicci PG, Mazzarella L. Tumor mutational burden quantification from targeted gene panels: major advancements and challenges. J Immunother Cancer. 2019 Jul 15;7(1):183

2. Hellmann MD, Nathanson T, Rizvi , et al. Genomic Features of Response to Combination Immunotherapy in Patients with Advanced Non-Small-Cell Lung Cancer. Cancer Cell. 2018

May 14;33(5):843-852

3. Samstein RM, Lee CH, Shoushtari AN. Tumor mutational load predicts survival after immunotherapy across multiple cancer types. Nat Genet. 2019 Feb;51(2):202-206

#### P100 Impact of obesity on immunity in gastroesophageal adenocarcinoma [GEAC]

##### Sarbajit Mukherjee, MD, MS^1^ , Sami Ibrahimi^2^, Yali Zhang^1^, Jianmin Wang^1^, Pawel Kalinski, MD, PhD^1^

###### ^1^Roswell Park Comprehensive Cancer Center, Buffalo, NY, United States; ^2^University of Oklahoma, Oklahoma City, United States

####### **Correspondence:** Sarbajit Mukherjee (sarbajit.mukherjee@roswellpark.org)

**Background**

Obesity is associated with an elevated risk of GEAC [1], but the molecular mechanism remains unknown. Paradoxically, however, obesity is associated with a superior response to anti-PD-1 treatment [2,3]. This may be explained by our recent observations that obesity enhances PD-1 mediated T-cell dysfunction in a mechanism involving leptin signaling. Prompted by this data, we aimed to identify obesity/leptin-regulated molecular biomarkers in GEAC.

**Methods**

Based on the body-mass index (BMI), we categorized patients into normal (BMI 18-24.9), overweight (BMI 25-29.9) and obese (BMI ≥30). We then retrospectively analyzed the clinical report of PD-L1 staining by IHC_22C3/Keytruda from metastatic GEAC patients treated at our institution between 2014-2019. Chi-squared test was used to determine the association between categorical variables. Next, we performed RNA-seq analysis of 13 gastric cancer FFPE specimens (8 obese and 5 normal weight) to identify differential gene expression between these two groups. Gene expression was quantified by log-fold changes. Differentially expressed genes were identified by using DESeq2. Then we looked at the association between the expression of leptin and immune-related genes from those specimens, using generalized linear model implemented in DESeq2. TCGA gastric cancer database (TCGA -STAD) was used to validate these associations (using Pearson test) independently. A p-value of <0.05.

**Results**

Our analysis of the clinical report of 77 patients with metastatic GEAC revealed that patients with a BMI =>25 were more likely to express PD-L1 than normal-weight individuals (p = 0.03)(Table 1). Our RNA-seq analysis identified the following genes to be up-regulated in the obese group: NOS2, FOXP3, IDO1, EOMES, CD160, and CXCR5 (p<0.05). Expression of these genes was positively correlated with leptin in our database; however, these associations did not reach statistical significance; possibly due to our small sample size. The same analysis within the TCGA-STAD database identified a strong positive correlation between the expression of all six genes and leptin (p <0.05)(Figure 1). GSEA identified several up-regulated immune-related pathways (Adaptive Immune System, Antigen Processing Cross Presentation etc.) in the obese group.

**Conclusions**

Our preliminary data suggest that obesity, and specifically leptin, is associated with several immune markers in GEAC. Our mechanistic studies will explore how obesity/ leptin regulates the immune system and promotes cancer. These studies may allow us to identify new leptin regulated pathways as therapeutic targets.

**References**

1. Garai J, Uddo RB, Mohler MC, Pelligrino N, Scribner R, Sothern MS et al. At the crossroad between obesity and gastric cancer. Methods Mol Biol.2015; 1238:689-707.

2. Ibrahimi S, Mukherjee S, Roman D, King C, Machiorlatti M, Aljumaily R. Effect of Body Mass Index and Albumin level on Outcomes of Patients Receiving Anti PD-1/PD-L1 Therapy. J Clin Oncol 36, 2018 (suppl 5S; abstr 213).

3. Wang Z, Aguilar EG, Luna JI, Dunai C, Khuat LT, Le CT et al. Paradoxical effects of obesity on T cell function during tumor progression and PD-1 checkpoint blockade. Nat Med. 2019 Jan; 25(1):141-151.

**Ethics Approval**

The study was approved by the Institutional Review Board at Roswell Park Comprehensive Cancer Center, approval number STUDY00000894 / BDR 109419.

**Table 1 (abstract P100).**
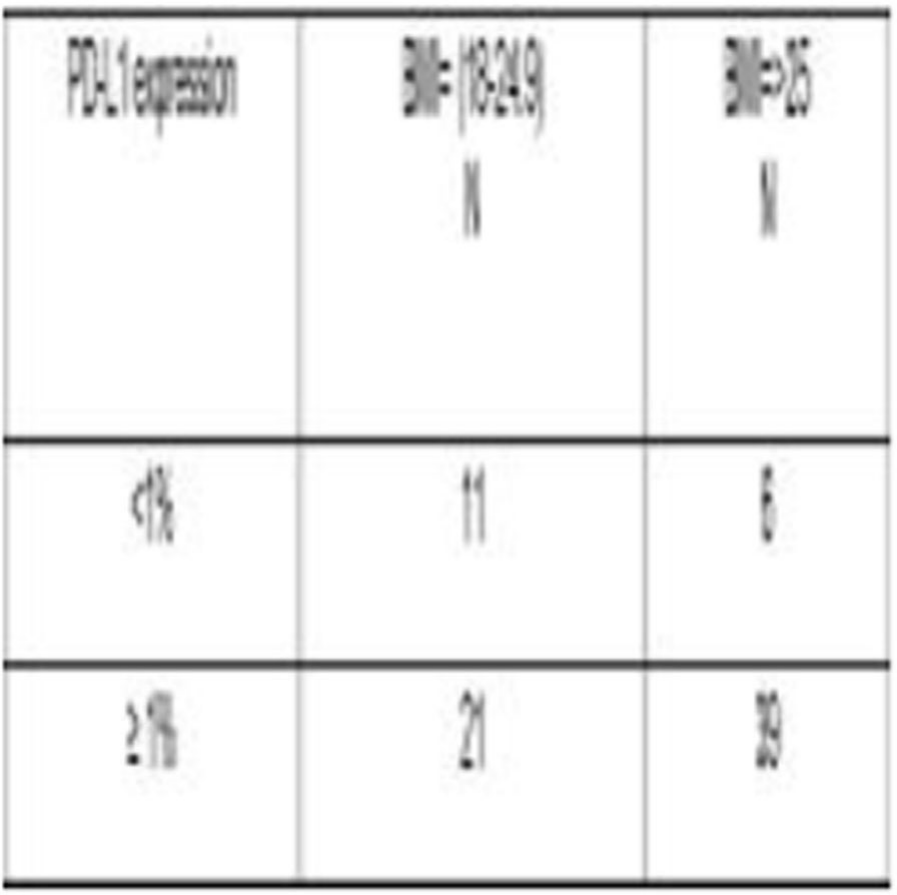
See text for description

**Fig. 1 (abstract P100).**
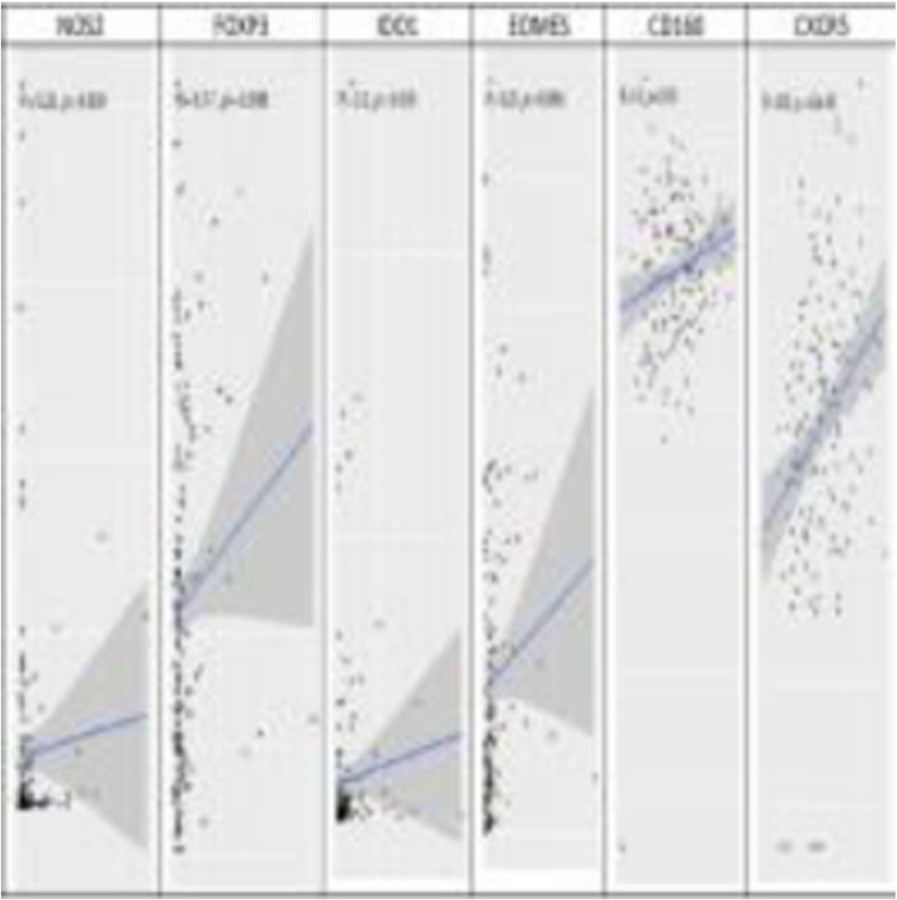
See text for description

#### P101 Immune-based classification of pleural malignant mesothelioma by using integrative transcriptome analysis

##### Ernest Nadal, MD, PhD^1^, Ania Alay^2^, David Cordero^1^, Elisabeth Aliagas^2^, José Ruffinelli^1^, Ramón Palmero^1^, Ricard Ramos^3^, Ivan Macía^3^, Anna Ureña^3^, Fran Rivas^3^, Xavier Solé^1^

###### ^1^Catalan Institute of Oncology, L'Hospitalet, Spain; ^2^Bellvitge Biomedical Research Institute, L'Hospitalet, Spain; ^3^Bellvitge University Hospital, L'Hospitalet, Spain

####### **Correspondence:** Xavier Solé (x.sole@iconcologia.net)

**Background**

Malignant pleural mesothelioma (MPM) is a rare and aggressive neoplasia. Immune checkpoint inhibitors in MPM demonstrated modest efficacy, partly due to lack of predictive biomarkers of clinical benefit from immunotherapy. The aims of this work were: to identify immune fractions associated with clinical outcome; to stratify MPM patients based on their immune contexture and to characterize the immune-based groups at the genomic and transcriptomic levels.

**Methods**

Seven gene-expression datasets of MPM were used to assess the immune microenvironment of 516 samples. The abundance of 20 immune fractions in each sample was inferred using Gene Set Variation Analysis. Identification of clinically-relevant fractions was performed with Cox Proportional-Hazards Models adjusted for age, stage, sex, and tumor histology.

**Results**

T-Helper 2 (Th2, HR=2.14, p=1.5x10-4) and cytotoxic T cells (CTC; HR=0.57, p=9.1x10-3) were found to be consistently associated with overall survival in multiple datasets. Three immune clusters (IG) were subsequently defined based on Th2 and CTC immune infiltration levels: IG1 (54.5% of samples) had high Th2/low CTC levels, IG2 (37%) had either low or high levels of both fractions, and IG3 (8.5%) had low Th2/high CTC levels. Immune clusters were associated with overall survival independently of tumor histology, with an improving survival from IG1 to IG3 (HR IG2=0.52, 95% CI 0.39–0.69; HR IG3=0.32, 95% CI 0.19–0.53; p=8.4x10-8; Figure 1). IG3 was significantly enriched in epithelioid tumors (90% IG3 vs. 62% IG1, p=0.001) and patients were younger compared to the other groups (60 years IG3 vs. 66 years IG1, p=0.021). These groups showed differential molecular profiles, being IG1 enriched for CDKN2A and IFN-related genes deletions. No statistically significant differences in the tumor mutational burden was observed, howerver IG3 tumours had fewer mutations than IG1 and IG2 groups. At the transcriptional level, IG1 samples showed upregulation of cell proliferation and DNA repair-related gene-sets, while IG3 samples presented upregulation of immune checkpoint inhibitors (Figure 2) and inflammation-related pathways. Finally, integration of gene expression with functional signatures of in vitro drug response showed that IG3 patients are more likely to respond to immune checkpoint inhibitors, while IG1 patients might be more sensitive to PARP inhibitors.

**Conclusions**

Analysis of publicly available gene-expression data of MPM reveals three major immune-based groups, based on Th2 and CTC composition. These clusters are associated with distinct genomic profiles and clinical outcome. Further validation of this classification is warranted in an independent cohort of MPM.

**Fig. 1 (abstract P101).**
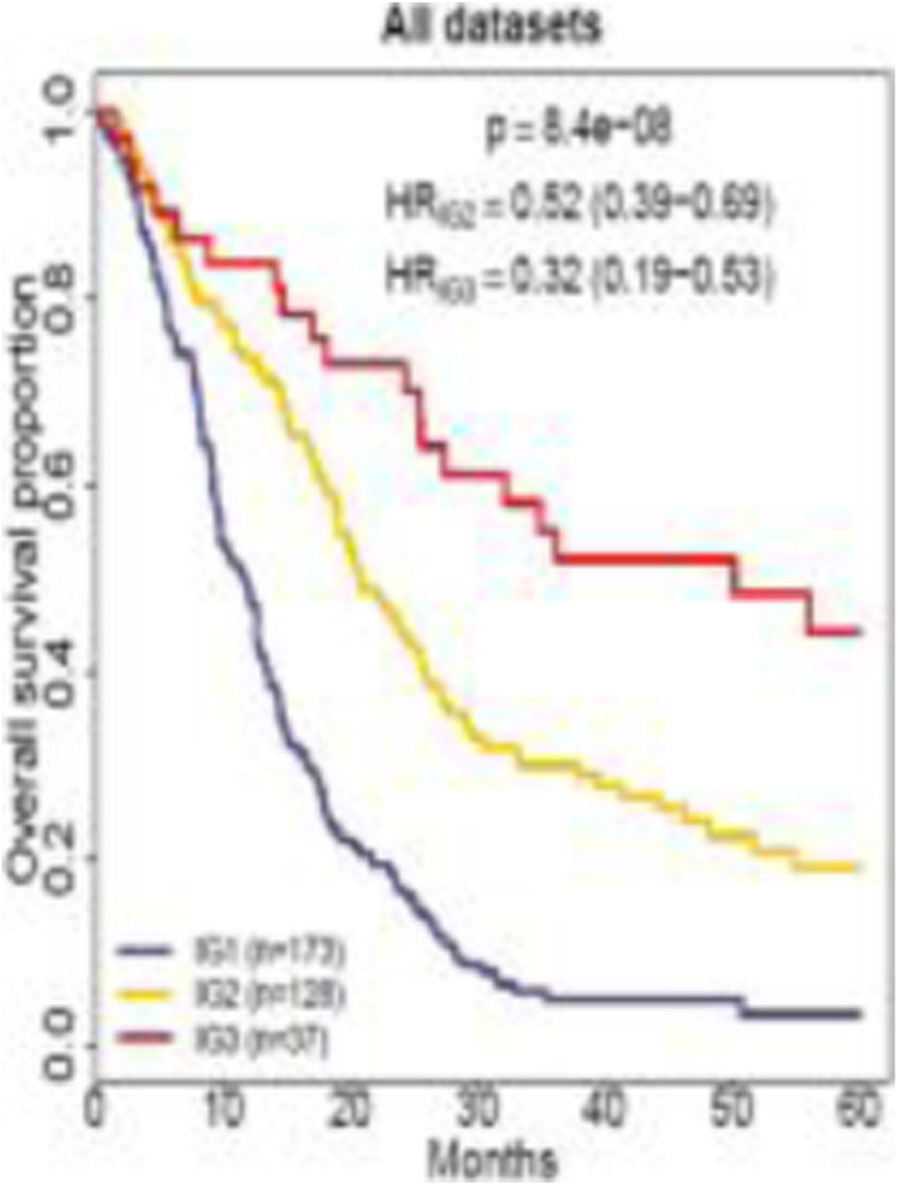
Overall survival analysis according to the immune groups

**Fig. 2 (abstract P101).**
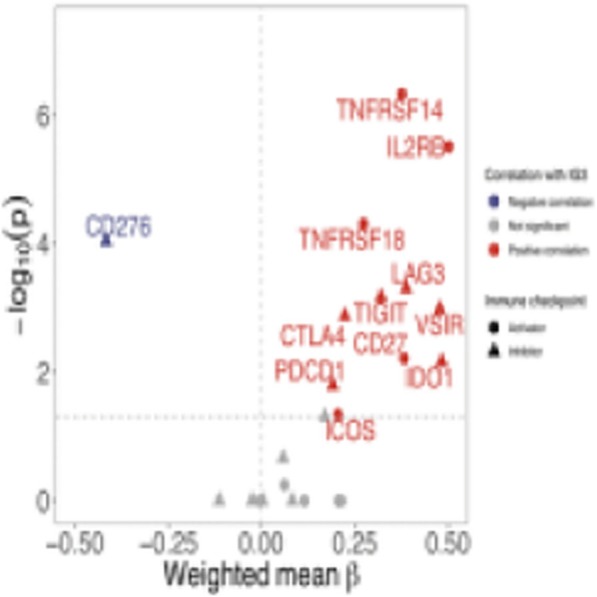
Expression of immune checkpoint markers

#### P102 CT antigens are frequently expressed non-inflamed tumors

##### Sarabjot Pabla, MSc, PhD, BS, Sarabjot Pabla, MSc, PhD, BS, Sarabjot Pabla, MSc, PhD, BS , Erik Van Roey, Sean Glenn, PhD, Jonathan Andreas, MS, Blake Burgher, BS, RN, Jeffrey Conroy, BS, Mary Nesline, MS, Antonios Papanicolau-Sengos, MD, Vincent Giamo, BS, MS, Felicia Lenzo, Yirong Wang, MS, Carl Morrison, MD, DVM

###### OmniSeq, Inc., Buffalo, NY, United States

####### **Correspondence:** Sarabjot Pabla (sarabjot.pabla@omniseq.com)

**Background**

Cancer testis (CT) antigens are tumor antigens that have a highly tissue restricted expression in germ cells but are often expressed in diverse malignancies. With their highly immunogenic expression limited to tumor cells, CT antigens have become a prime target for cancer vaccinations and T-cell based therapy with chimeric T-cell receptors. In this study, we investigated the association of two CT antigens (NY-ESO-1 and LAGE-1a) with the immune microenvironment of real-world clinical tumors spanning multiple histologies. Furthermore, we describe the association of CT antigens with traditional biomarkers of immunotherapy such as PD-L1 immunohistochemistry (IHC) and tumor mutational burden (TMB), with inflammatory status and cell proliferation status with confirmatory studies performed on a large TCGA pan-cancer cohort of 11,001 tumors.

**Methods**

Unsupervised clustering was performed on gene-expression data of 395 immune transcripts of 1323 FFPE tumors to reveal three inflammatory patient clusters and three distinct gene groups; CT-antigen, inflammatory and neoplastic clusters. Test for proportions was performed using Pearson’s chi-squared test to describe association of NY-ESO-1 and LAGE-1a with PD-L1 IHC, TMB, inflammatory cluster and cell-proliferation. A retrospective cohort (n=242) of checkpoint inhibition (CPI) treated tumors was utilized to perform overall survival (Kaplan-Meier curves) and response to CPI therapy for CT antigen+ tumors. Survival analysis was confirmed against the Pan-Cancer TCGA cohort (n=11,001).

**Results**

Unsupervised clustering showed clear co-expression sub-clustering of CTA genes differentiated from “immune” and from “neoplastic expression”. PD-L1 IHC status was not associated with NY-ESO-1 (p=0.71) or LAGE-1a (p=0.52) status. Interestingly, LAGE-1a positive cases were over-represented in TMB high cases (p=0.016), whereas, NY-ESO-1 status was not associated with TMB. NY-ESO-1 positive cases were highly over-represented in non-inflamed cluster (p=0.006), whereas, LAGE-1a status was not associated with inflammation status. Both NY-ESO-1 (p=0.031) and LAGE-1a (p=0.008) were significantly associated with cell-proliferation status. NY-ESO-1 positive tumors have significantly (p=0.014) higher response rate in retrospective cohort but this was not observed for LAGE-1a status. NY-ESO-1 and LAGE-1a status showed trend toward better (p=0.09 and p=0.06 respectively) survival in the retrospective and TCGA pan-cancer cohort.

**Conclusions**

This study presents an in-depth analysis of the immune landscape of CT antigen positive tumors across multiple histologies. CT antigen bearing tumors not only have unique immune profiles but also have significant associations with biologically relevant emerging biomarkers such as inflammatory signature, TMB and cell proliferation. CT antigens are a viable target for non-inflamed tumors for checkpoint inhibition therapy.

**Ethics Approval**

De-identified specimens and data were analyzed by OmniSeq under IRB approved protocol BDR 080316 (Roswell Park Comprehensive Cancer Center, Buffalo, NY).

#### P103 Detection of human leukocyte antigen class I loss of heterozygosity in solid tumor types by next-generation DNA sequencing

##### Jason Perera, PhD , Brandon Mapes, PhD, Denise Lau, PhD, Ameen Salahudeen, Aly Khan, PhD

###### Labs, Chicago, IL, United States

####### **Correspondence:** Jason Perera (jason.perera@tempus.com)

**Background**

Human leukocyte antigen (HLA) class I proteins are expressed on the surface of all nucleated cells and are vital for immune surveillance. When tumor-specific mutations (neoantigens) are presented on HLA molecules to CD8+ T cells, this recognition can drive immune responses against the tumor and lead to tumor destruction. One mechanism of immune escape for tumors is loss of heterozygosity in HLA genes (HLA-LOH), which reduces the total number of neoantigens available for presentation to T cells. Due to the highly polymorphic nature of HLA, the copy number status of HLA genes is extremely challenging to assess by standard bioinformatics approaches. To investigate the prevalence of HLA-LOH, we developed a specialized pipeline to detect HLA-LOH by DNA next-generation sequencing (NGS).

**Methods**

A cohort of colorectal and non-small cell lung cancer samples underwent DNA sequencing on the Tempus xT panel using paired, formalin-fixed, paraffin-embedded tumor and normal (blood or saliva) samples. To detect HLA-LOH from NGS data, we used NGS-based HLA typing to resolve the patient’s most probable HLA haplotype. Based on this haplotype, we adaptively realigned reads, extracted a number of features describing the relative allele coverage in the tumor and normal samples, and used these features to make a confident determination of allelic loss in the patient’s tumor sample.

**Results**

Evidence of HLA-LOH was detected in 16% of non-small cell lung tumor samples and 17% of colorectal tumor samples. We did not observe a significant association between LOH status and tumor mutational burden or neoantigen load. In the colorectal cancer cohort, HLA-LOH was observed in tumor samples classified as microsatellite instability-high (MSI-H); however, the association between HLA-LOH status and MSI status was not statistically significant.

**Conclusions**

We developed a novel method of determining HLA-LOH by DNA NGS and demonstrated that HLA-LOH is a readily detectable feature in human tumors. These results highlight the complexity of antigen presentation, the potential importance of HLA-LOH as a biomarker of immunotherapy response and resistance, and lays the groundwork for future investigations.

#### P104 Impact of chemotherapy (chemo) on peripheral T-cell diversity and implications for subsequent immunotherapy response in breast cancer

##### Joanna Pucilowska, PhD^1^, Paul Fields, PhD^2^, Valerie Conrad, BS^3^, David Page, MD^3^, Alison Conlin, MD^3^, Joanna Pucilowska, PhD^3^, Catherine Sanders, PhD^2^, Raina Tamakawa, MS^3^, Brie Chun, MD^3^, Isaac Kim, MD^3^, Mark Schmidt^3^

###### ^1^Providence Cancer Center, Portland, OR, United States; ^2^Adaptive Biotechnologies, Seattle, WA, United States; ^3^EACRI Providence Cancer Center, Portland, OR, United States

####### **Correspondence:** David Page (david.page2@providence.org)

**Background**

Immune checkpoint blockade is only modestly effective in metastatic breast cancer. One potential contributing factor is chronic lymphodepletion associated with preceding curative-intent chemo. Here, we evaluate the short and long-term effects of chemo on peripheral T-cell counts and clonal diversity in a cohort of breast cancer patients.

**Methods**

Stage I-III subjects (n=24) receiving curative-intent chemo (doxorubicin, cyclophosphamide, paclitaxel) were monitored longitudinally (mixed effects linear model) with serial peripheral blood mononuclear cell flow cytometry and quantitative immunosequencing of the T-cell receptor β locus (TCRseq) using the immunoSEQ® assay (Adaptive Biotechnologies, Seattle, WA). To evaluate for long-term chemo effects, these analyses were repeated in a cohort of recurrent breast cancer patients who received chemo >12 months preceding analysis (n=9). Wilcoxon rank sum and tests of slope were employed to screen for associations with chemo response, defined as complete pathologic response (pCR) at surgical resection.

**Results**

By TCRseq, chemo resulted in an acute decline in T-cell fraction (0-8 weeks, p12 months following chemo.

**Conclusions**

Curative-intent chemo is associated with T-cell death followed by reconstitution, with the resulting T-cell repertoire being more clonal and less abundant in naïve T cells. These findings persist at the time of metastatic recurrence, and therefore may contribute to immunotherapy non-response in metastatic disease. Conversely, we identified T-cell reconstitution as a potential biologic modifier of chemo response. T-cell reconstitution can be therapeutically targeted with inhibitors of androgen receptor signaling, which in experimental models enables thymic maturation of naïve T-cell clones and an increase in peripheral T-cell count. This hypothesis is being evaluated in an ongoing phase II clinical trial of bicalutamide (androgen receptor antagonist) plus ipilimumab and nivolumab in metastatic breast cancer (NCT03650894).

**Ethics Approval**

The study was reviewed and approved by Providence Heath and Services Internal review board, approval number 15-162.

#### P105 PD-L1 isoform as a potential biomarker to predict response for anti-PD-(L)1 treatment

##### Kunbin Qu (kunbin.qu@beigene.com)

###### BeiGene, USA, San Mateo, CA, United States

**Background**

anti-PD-1/anti-PD-L1 (anti-PD-(L)1) therapies have shown clinical activity across different cancers. However, predicting patient response remains challenging. Here we explore PD-L1 splicing isoforms as a potential predictive biomarker for anti-PD-(L)1 therapy response. Four PD-L1 splicing isoforms exist, including one dominant wildtype transcript and an alternative isoform which skips the second exon (deltaExon2_PD-L1).

**Methods**

TCGA normalized mRNA transcript counts were downloaded from Genomic Data Commons. anti-PD-1 treated melanoma RNA-Seq data was from Hugo et. al. [1]. Bioinformatics analyses were performed in statistical package R. Human wildtype and deltaExon2_PD-L1 isoforms were stably transfected into the mouse cell-line BW5147. A chimeric PD-1 receptor, P3Z, which fuses the extracellular and transmembrane domains of human PD-1 to the cytoplasmic domain of human CD3ζ, was stably transfected into HuT78 cells as a reporter assay for PD-1 signaling and IL-2 production [2].

**Results**

By examining protein crystal structures from Protein Data Bank, we found exon2 occupies the physical interface between PD-1 and PD-L1. It is also the interface between anti-PD-L1 therapeutics and PD-L1. Therefore, anti-PD-L1 molecules may not effectively target PD-L1 in patients harboring the deltaExon2_PD-L1 isoform and may lack clinical activity. The prevalence of the deltaExon2_PD-L1 isoform across TCGA tumors is shown in Figure 1. There are 8 cancers where the isoform is present above 5%, including liver and endometrial cancers.

The deltaExon2_PD-L1 isoform was successfully transfected into BW5147 as demonstrated by mRNA expression. In co-cultures of HuT78/P3Z with BW5147/PD-L1 (both the wildtype and deltaEexon2_PD-L1), IL-2 was secreted from the wildtype but not from the deltaExon2_PD-L1. Incubation with anti-PD-1 reduced IL-2 in a dose-dependent manner with the wildtype only (Figure 2), indicating deltaExon2_PD-L1 does not support PD-1 signaling.

Patients expressing only deltaExon2_PD-L1 or a higher ratio of deltaExon2_PD-L1/wildtype may not have optimal PD-(L)1 axis signaling and be less responsive to anti-PD-1 intervention. To test this hypothesis, a ratio metric between deltaExon2_PD-L1 and wildtype was applied to an anti-PD-1 treated melanoma cohort GSE78220. This biomarker ratio stratified responders from non-responders with a p-value of 0.027 (non-responders with no deltaExon2_PD-L1 isoform were excluded, Figure 3), whereas PD-L1 expression did not.

**Conclusions**

Patients with deltaExon2_PD-L1 isoform lack the interface between PD-L1 and PD-1, the same interface necessary for anti-PD-L1 therapeutic binding. This may lead to non-optimal signaling through the PD-(L)1 axis. Suboptimal signaling and inability to bind anti-PD-L1 potentially could reduce response to both anti-PD-1 and anti-PD-L1 treatments. This hypothesis needs to be further validated in additional anti-PD-L1 and anti-PD-1 treated cohorts.

**Acknowledgements**

The authors would like to thank Vanitha Ramakrishnan and Jessica Li for scientific discussions.

**References**

1. Hugo W. et. al. Genomic and Transcriptomic Features of Response to Anti-PD-1 Therapy in Metastatic Melanoma. Cell 2016; 165:35-44.

2. Zhang T. et. al. The binding of an anti-PD-1 antibody to FcγRΙ has a profound impact on its biological functions. Cancer Immunol Immunother. 2018; 67:1079-1090.

**Fig. 1 (abstract P105).**
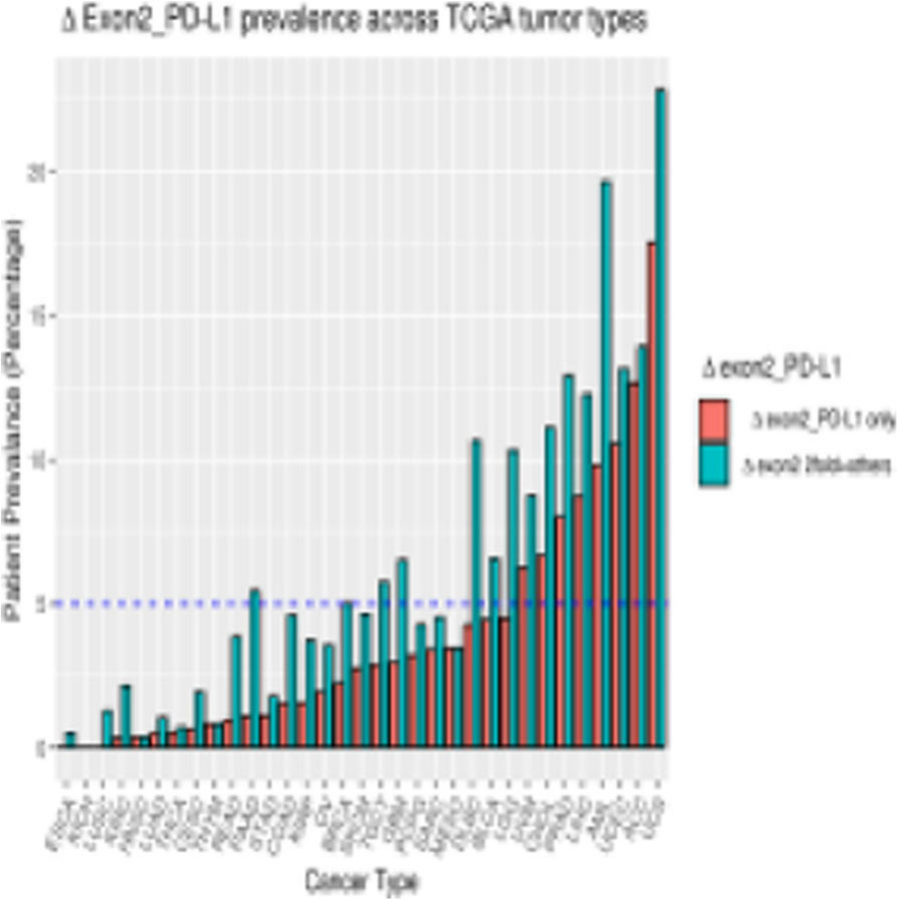
See text for description

**Fig. 2 (abstract P105).**
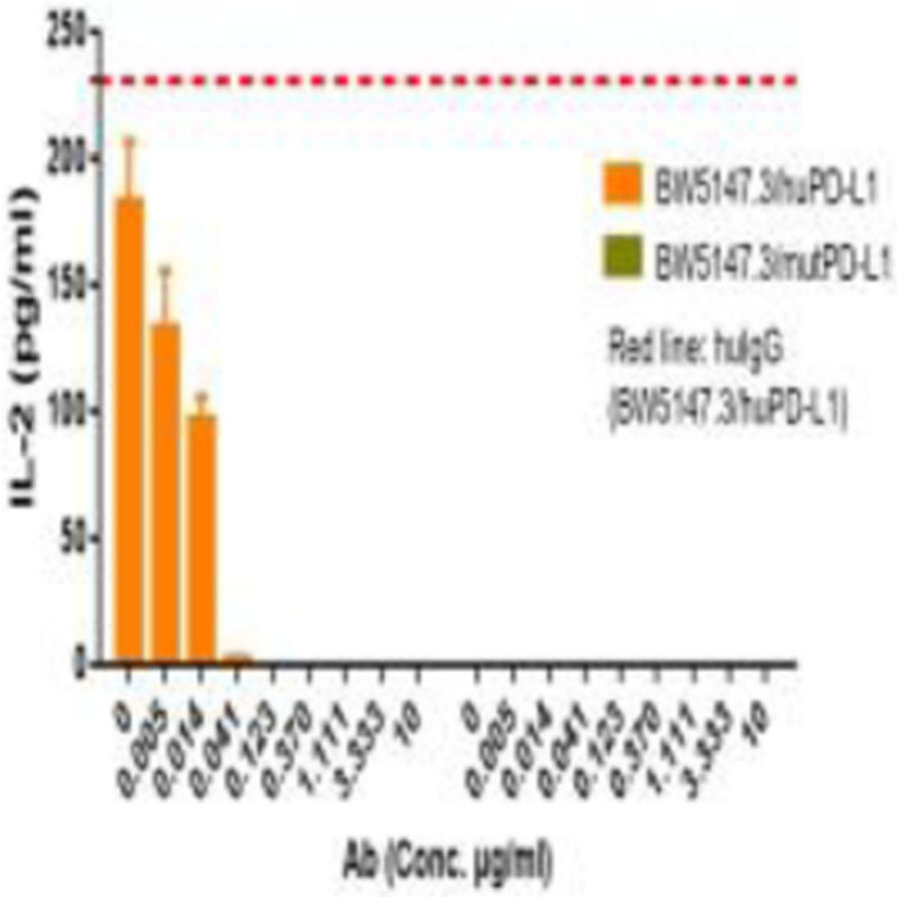
See text for description

**Fig. 3 (abstract P105).**
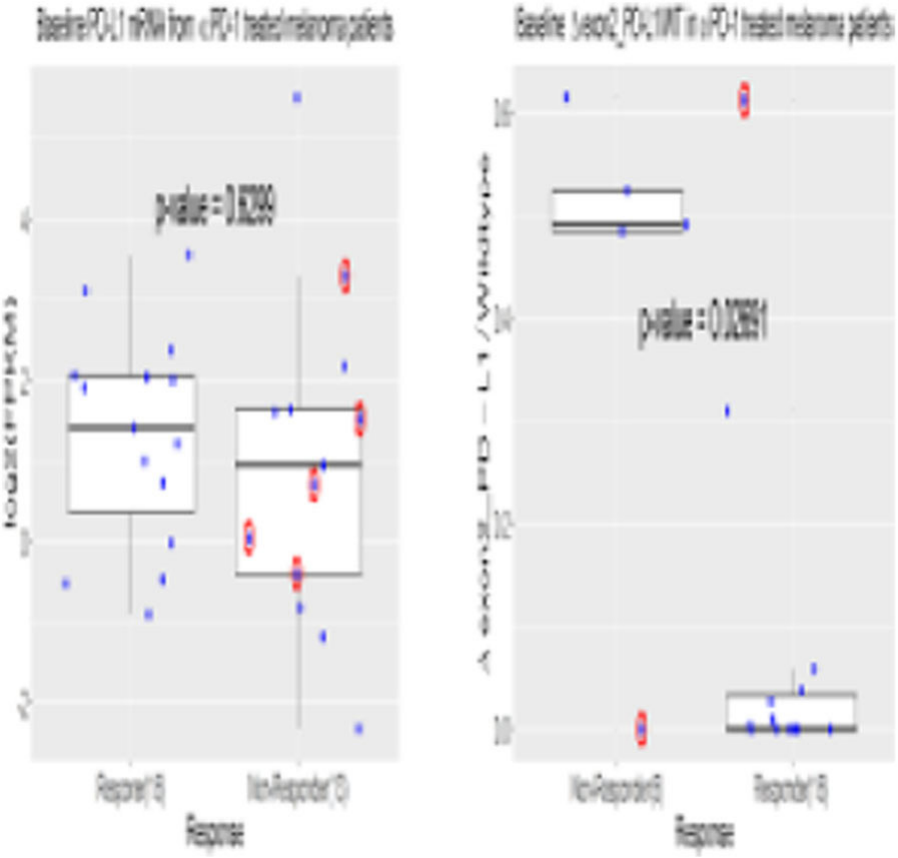
See text for description

#### P106 Tumor mutational burden profile (TMB) of oncogenic driver mutations in non small cell lung cancer

##### Paul Walker, MD, Nitika Sharma

###### East Carolina University, Greenville, NC, United States

####### **Correspondence:** Nitika Sharma (sharman@ecu.edu)

**Background**

Tumor mutational burden has emerged as a potential biomarker predictive of response to Immune checkpoint blockade (ICB) in lung cancer. The utility of this biomarker in oncogenic driver mutations, that account for nearly 20-50% of NSCLC, is still unknown. KRAS mutation in lung cancer is a prognostic biomarker whereas EGFR and BRAF pathogenic mutations are predictive of response to tyrosine kinase inhibitors (TKI). ICB with bevacizumab has demonstrated clinical benefit in EGFR mutated lung cancers per IMpower150 clinical trial [1]. TMB analysis between actionable/pathogenic EGFR mutations (i.e. exon 19 del, exon 21 L858R, T790M) and EGFR uncommon/variants mutations may provide therapeutic implications [2]. To explore the immunological basis for these findings, we evaluated the immune biomarker profile of NSCLC patients using Caris next-generation sequencing (NGS) platform.

**Methods**

We studied tissue samples on 446 patients with NSCLC from 2016-18. TMB was measured by counting all non-synonymous somatic mutations per megabase of the genome coding area using targeted NGS (592 genes). High TMB was defined as ≥ 10 mut/Mb. The analysis was conducted using SAS 9.4. Variables were tested using a Wilcoxon signed-rank test.

**Results**

KRAS mutations were found in 85 pts (19%), BRAF in 9 pts (2%), EGFR mutation in 36 pts (8%), EGFR pathogenic mutation in 22 pts (5%), EGFR variants in 14pts (3%). The median TMB of KRAS mutant vs KRAS wt (wild type) was 10 vs 7 mut/Mb (range 0-31, p<0.01).

**Conclusions**

This study highlights the unique immune profile of certain oncogenic driver mutations in NSCLC. Our results show that KRAS and BRAF mutant subsets have a significantly higher TMB than KRAS and BRAF wild type. In addition, EGFR variants have a higher TMB as compared to actionable pathogenic EGFR mutations. These findings could have therapeutic implications in guiding patient selection for ICB and merit a prospective investigation.

**References**

1. Socinski M, Jotte R,Cappuzzo F. Atezolizumab for First-Line Treatment of Metastatic Nonsquamous NSCLC. N Engl J Med. 2018; 378:2288-2301.

2. Offin M, Rizvi H,Tenet M.Tumor Mutation Burden and Efficacy of EGFR-Tyrosine Kinase Inhibitors in Patients with EGFR-Mutant Lung Cancers. Clin Cancer Res. 2018;1102.

**Ethics Approval**

The study was approved by ECU Institutional Review Board, approval number UMCIRB 15-001311.

#### P107 Changes in tumor mutational burden in serially biopsied non-small cell lung cancer

##### James Smithy, MD, MHS^1^, James Smithy, MD, MHS^1^, David Hwang, MD^1^, Yvonne Li^2^, Liam Spurr^2^, Andrew Cherniack, PhD^3^, Lynette Sholl^1^, Mark Awad, MD PhD^2^

###### ^1^Brigham and Women's Hospital, Boston, MA, United States; ^2^Dana-Farber Cancer Institute, Boston, MA, United States; ^3^Broad Institute of MIT and Harvard, Boston, MA, United States

####### **Correspondence:** Mark Awad (Mark_Awad@DFCI.harvard.edu)

**Background**

High tumor mutational burden (TMB) has been associated with response to checkpoint blockade in non-small cell lung cancer (NSCLC) and other malignancies. However, the degree to which TMB changes over time, across anatomical sites, and with intervening treatment remains unknown. To evaluate TMB changes across time points, we compared TMB in tissue specimens from patients with serially-biopsied NSCLC.

**Methods**

Clinicopathologic characteristics and changes in TMB were analyzed from patients with NSCLC and more than one tissue specimen that had undergone targeted next generation sequencing (NGS, OncoPanel) at the Dana-Farber Cancer Institute. Those representing distinct primary tumors by histologic or genomic analysis were excluded.

**Results**

193 NSCLC patients with more than one interpretable NGS result were identified; 30 were excluded due to separate primary tumors. Of the 163 remaining patients included in the analysis, the median time between samples was 14 months (range: 0 to 114 months). TMB was higher in current and former smokers (median TMB 10.7 v 6.4 mutations/megabase(Mb); p < 0.0001), patients without an identifiable oncogenic driver mutation (median TMB 14.5 v 8.5 mutations/Mb; p = 0.004), and patients with locoregional disease at the time of diagnosis (median TMB 10.8 v 8.0 mutations/Mb, p = 0.02). TMB correlated closely across all matched tumor pairs (Pearson’s r = 0.85, Figure 1). Significant increases or decreases in TMB were uncommon in paired samples, and we observed no significant change in median TMB with increasing time between specimen collection or with intervening chemotherapy, immunotherapy, radiation therapy, or targeted therapy.

**Conclusions**

In NSCLC, TMB correlated closely across tumor pairs, and increasing time between sample collections and intervening treatments were not correlated with significant changes in TMB.

**Ethics Approval**

This study was conducted under Dana-Farber/Harvard Cancer Center Protocol 02-180.

**Fig. 1 (abstract P107).**
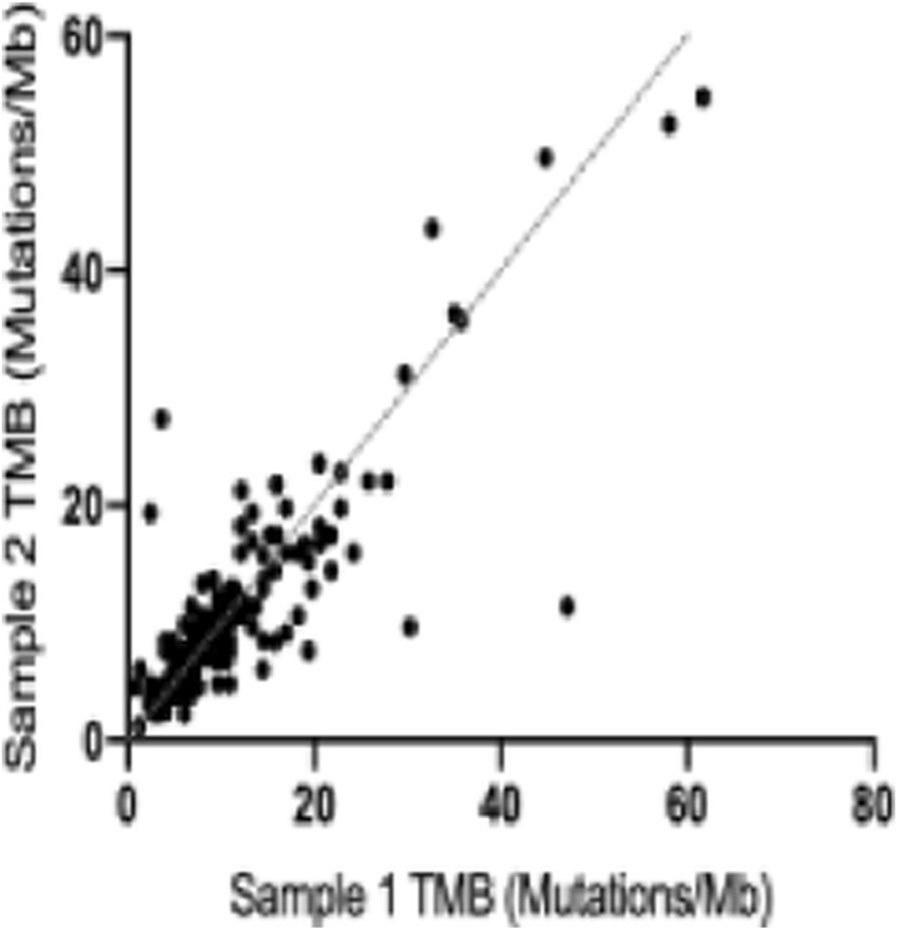
See text for description

#### P108 Working towards precision medicine of the tumor microenvironment

##### Kyung Kim, PhD^2^, Jeeyun Kim^2^, Seung-Tae Kim^2^, Jung-Yong Hong^2^, Laura Benjamin^1^, Kristen Strand-Tibbitts, PhD^1^

###### ^1^Oncologie Inc, Waltham, MA, United States; ^2^Samsung Medical Center, Seoul, Republic of Korea

####### **Correspondence:** Kristen Strand-Tibbitts (kristen@oncologie.international)

**Background**

Immune therapies for cancer have generated an enhanced focus on controlling cancer through modulation of biologies associated with the tumor microenvironment, rather than the traditional approach of targeting cancer cell biology. As more and more targeted therapies are designed to modulate the tumor microenvironment, we need a better understanding of microenvironmental heterogeneity in human cancer. Similar to what has been done to describe patient subsets based on their cancer biology using DNA and RNA signatures, we are working to describe patient subsets based on their microenvironment biology. The ultimate goal is to find effective means of identifying patients for novel therapeutic treatments that target biological pathways that regulate the non-neoplastic cells and drive cancer progression.

**Methods**

RNA from publicly available sources including microarray and RNASeq were analyzed with respect to gene signatures that describe four different microenvironmental phenotypes.

**Results**

These four microenvironmental subtypes are prognostic, but also show evidence of being predictive to existing modalities of cancer drugs when analyzed in retrospective analysis. We examined the impact of cancer stage on the distribution of these subtypes and find little variation.

**Conclusions**

Future clinical trials to prospectively test these four unique signatures as predictive biomarkers for therapy need to be designed.

#### P109 Predictive performance of a CD8-derived signature by gene expression profiling in patients with urothelial carcinoma from CheckMate 275

##### Peter Szabo, PhD^1^ , Padmanee Sharma, MD, PhD^2^, George Lee, PhD^1^, Scott Ely^1^, Vipul Baxi, MS^1^, Keyur Desai, PhD^1^, Lisu Wang^1^, Robin Edwards, PhD^1^, Saumya Pant^1^, Abdel Saci^1^, Neeraj Adya^1^, Matthew Galsky, MD^3^

###### ^1^Bristol-Myers Squibb, Lawrence Township, NJ, United States; ^2^MD Anderson Cancer Center, Houston, TX, United States; ^3^Tisch Cancer Institute, New York, NY, United States

####### **Correspondence:** Peter Szabo (Peter.Szabo@bms.com)

**Background**

Gene expression profiling (GEP) has been used to identify biomarkers of response to immunotherapy. Using a GEP-based inflammation assay, we derived and analytically validated a CD8 signature to assess T-cell infiltration in the tumor microenvironment (TME) [1]. Here, we retrospectively explore the association of the CD8 signature, alone and in relation to established biomarkers PD-L1 and tumor mutational burden (TMB), with clinical response to nivolumab treatment.

**Methods**

In the phase 2 CheckMate 275 trial, 270 patients with platinum-resistant metastatic urothelial carcinoma (UC) and evaluable tumor PD-L1 expression received nivolumab treatment. Responses were determined by blinded, independent review committee assessments [2]. Minimal follow-up time for the current analysis was ~3 years. T-cell infiltration in the TME was assessed using the CD8 signature and by immunohistochemistry (IHC) using an automated commercial proprietary assay (Dako mouse clone C8/144B; Agilent Technologies Co) [1]. PD-L1 expression on tumor cells was independently assessed by IHC using the PD-L1 IHC 28-8 pharmDx assay (Dako). TMB was measured by whole exome sequencing [3]. Cox proportional-hazards regression assessed the dependence of progression-free survival (PFS) or overall survival (OS), and logistic regression assessed the dependence of objective response (OR) on biomarker values. The linear effects of biomarkers and their multiplicative interaction were included when multiple biomarkers were evaluated. Likelihood-ratio tests (2-sided) were used to assess biomarker interaction effects. Associations with PFS and OS were investigated using Kaplan–Meier analyses with biomarker scores categorized by tertile.

**Results**

GEP was evaluable in 205 (76%) and GEP+TMB in 113 (42%) of 270 treated patients. Baseline characteristics, OR, PFS, and OS were similar between all treated patients and the GEP-evaluable cohort. CD8 signature scores showed a positive association with OR (P=0.005), PFS (P=0.005), and OS (P

**Conclusions**

These results suggest that the GEP-based CD8 signature may have utility as a potential biomarker for predicting clinical response to nivolumab treatment. The CD8 signature may be used alone and/or in combination with other relevant and potentially independent biomarkers such as PD-L1 expression or TMB to identify patients likely to benefit from anti–PD-1 therapies.

**Acknowledgements**

Bristol-Myers Squibb. Professional medical writing and editorial assistance were provided by Katerina Pipili, PhD, and Jay Rathi, MA, of Spark Medica Inc, funded by Bristol-Myers Squibb.

**Trial Registration**

NCT02387996

**References**

1. Szabo PM, Qi Z, Zerba K, et al. Association of an inflammatory gene signature with CD8 expression by immunohistochemistry (IHC) in multiple tumor types. J Clin Oncol. 2019; 37(Suppl): Abstract 2593.

2. Sharma P, Retz M, Siefker-Radtke A, et al. Nivolumab in metastatic urothelial carcinoma after platinum therapy (CheckMate 275): a multicentre, single-arm, phase 2 trial. Lancet Oncol. 2017; 18:312-322.

3. Galsky M, Saci A, Szabo P, et al. Impact of tumor mutation burden on nivolumab efficacy in second-line urothelial carcinoma patients: exploratory analysis of the phase II CheckMate 275 study. Ann Oncol. 2017; 28(Suppl 5): Abstract 848PD.

**Ethics Approval**

The protocol was approved by site institutional review boards or independent ethics committees and conducted according to Good Clinical Practice guidelines, per the International Conference on Harmonisation. Patients provided written informed consent based on Declaration of Helsinki principles.

#### P110 Tumor CD8+ T-cell infiltration assessed by gene expression profiling alone or by immunohistochemistry plus epithelial-mesenchymal transition gene expression in urothelial carcinoma in CheckMate 275

##### Peter Szabo, PhD^1^ , Abdel Saci^1^, Padmanee Sharma, MD, PhD^2^, George Lee, PhD^1^, Scott Ely^1^, Vipul Baxi, MS^1^, Keyur Desai, PhD^1^, Lisu Wang^1^, Scott Chasalow^1^, Michael Montalto^1^, Robin Edwards, PhD^1^, Saumya Pant^1^, Neeraj Adya^1^, Bruce Fischer, MD^1^, Matthew Galsky, MD^3^

###### ^1^Bristol-Myers Squibb, Lawrence Township, NJ, United States; ^2^MD Anderson Cancer Center, Houston, TX, United States; ^3^Tisch Cancer Institute, New York, NY, United States

####### **Correspondence:** Peter Szabo (Peter.Szabo@bms.com)

**Background**

Close proximity of CD8+ T cells to cancer cells has been associated with improved outcome with immunotherapy. Using a gene expression profiling (GEP)-based inflammation assay, we previously derived gene signatures that defined CD8+ T-cell infiltration (CD8 signature) and localization to tumor parenchymal and stromal compartments (CD8-topology signatures) in multiple tumor types [1,2]. In patients with urothelial carcinoma (UC), high stromal/epithelial-mesenchymal transition (EMT) gene expression has been associated with T-cell exclusion and poor response to immunotherapy [3]. Here, we assess three CD8-derived signatures and compare them with a CD8 immunohistochemistry (IHC)-derived score combined with EMT gene expression (CD8.IHC_EMT) to evaluate associations between these biomarkers and with response to nivolumab in patients with UC in CheckMate 275 [4].

**Methods**

270 patients with platinum-resistant metastatic UC received nivolumab, with response assessed by blinded central review [4]. CD8+ T-cell infiltration in the TME (assessed using the CD8 signature [1], CD8-topology signatures (parenchymal, stromal) [2], and by IHC using a proprietary commercial assay [Dako mouse clone C8/144B antibody; Agilent Technologies Co]) and PD-L1 expression on tumor cells (Dako PD-L1 IHC 28-8 pharmDx) were assessed on baseline tumor samples. Predictive performance of the CD8 signature and CD8-topology signatures individually, the combined CD8-derived signatures (triple CD8), and CD8.IHC_EMT, was evaluated using Cox proportional-hazards regression for overall and progression-free survival (OS, PFS) and with logistic regression for objective response (OR). Odds ratios were scaled to reflect the difference between the 75th and 25th biomarker percentiles. Two-sided likelihood-ratio tests were used to assess biomarker and interaction effects. Associations with PFS and OS were also investigated using Kaplan–Meier analyses with biomarker scores categorized by tertile.

**Results**

GEP was evaluable in 205/270 (76%) patients. Baseline characteristics and clinical outcomes were similar in the overall population and the GEP-evaluable cohort. Response and survival predictions from the triple CD8 and CD8.IHC_EMT overlapped, and both biomarkers predicted benefit from nivolumab independent of PD-L1 expression. Odds ratios for OR were 2.59 (95% CI, 1.59–4.21) for triple CD8, 2.12 (1.47–3.07) for CD8.IHC_EMT, 2.51 (1.42–4.43) for the CD8 signature, and 1.74 (1.22–2.49) for the parenchymal CD8-topology signature.

**Conclusions**

Combined CD8 and CD8-topology gene signatures (triple CD8) showed similar performance to CD8.IHC_EMT for predicting response and survival in nivolumab-treated patients with UC. These data suggest potential utility of testing biomarker combinations and support further evaluation of gene signatures associated with parenchymal vs stromal CD8+ T-cell localization for predicting response to immunotherapy in patients with cancer.

**Acknowledgements**

Bristol-Myers Squibb. Professional medical writing and editorial assistance were provided by Bernard Kerr, PGDipSci, and Jay Rathi, MA, of Spark Medica Inc, funded by Bristol-Myers Squibb.

**Trial Registration**

NCT02387996

**References**

1. Szabo PM, Qi Z, Zerba K, et al. Association of an inflammatory gene signature with CD8 expression by immunohistochemistry (IHC) in multiple tumor types. J Clin Oncol. 2019; 37(Suppl): Abstract 2593.

2. Szabo PM, Lee G, Ely S, et al. CD8+ T cells in tumor parenchyma and stroma by image analysis and gene expression profiling: potential biomarkers for immuno-oncology therapy. J Clin Oncol. 2019; 37(Suppl): Abstract 2594.

3. Wang L, Saci A, Szabo PM, et al. EMT- and stroma-related gene expression and resistance to PD-1 blockade in urothelial cancer. Nat Commun. 2018; 9:3503.

4. Sharma P, Retz M, Siefker-Radtke A, et al. Nivolumab in metastatic urothelial carcinoma after platinum therapy (CheckMate 275): a multicentre, single-arm, phase 2 trial. Lancet Oncol. 2017; 18:312-322.

**Ethics Approval**

The protocol was approved by site institutional review boards or independent ethics committees and conducted according to Good Clinical Practice guidelines, per the International Conference on Harmonisation. Patients provided written informed consent based on Declaration of Helsinki principles.

#### P111 Clinical and immunologic implications of a microsatellite instability score in lung cancer

##### Pedro Viveiros, MD^1^, Misuk Lee^1^, Bhoomika Sukhadia, MD^1^, Kyunghoon Rhee^1^, Victor Wang^2^, Jeffrey Chuang^2^, Young Kwang Chae, MD^1^

###### ^1^Northwestern University, Chicago, IL, United States; ^2^Jackson Laboratory For Genomic Medicine, Farmington, CT, United States

####### **Correspondence:** Misuk Lee (misuklee55@gmail.com)

**Background**

Microsatellite instability status is currently used to predict susceptibility to immunotherapy. MANTIS score was originally developed to identify microsatellite instability through next-generation sequencing (NGS). Although the 0.4 cutoff identifies MSI-high status, there is insufficient data for this score's repercussion for MSI-stable patients [1]. MSI-high status rarely occurs in lung cancer patients representing less than 1% of the cases. Therefore, we aim to identify how MANTIS score correlates with immune profile and clinical outcomes in MSI-stable lung cancer.

**Methods**

MANTIS score was calculated for two TCGA (The Cancer Genome Atlas) cohorts: squamous cell carcinoma (SqCC, n= 501) and adenocarcinoma (ADC, n=517). After excluding MSI-high patients (n=3 and 1, respectively) we stratified each cohort into quartiles. The highest quartile was named MANTIS-high (M-H) and the lowest quartile MANTIS-low (M-L). Immune profile (immune cell infiltration and PD-L1 expression), tumor mutational burden (TMB), neoantigen burden and survival outcomes were compared between M-H and M-L. Tumor immune landscape was identified using signatures from immune metagenes predicting infiltration for 31 immune cells.

**Results**

M-H was associated with higher activated CD4, gamma delta and Th17 T cell infiltration when compared with M-L in lung SqCC (all p <0.05). No statistically significant difference in tumor T cell infiltration was found in ADC (Figure 1,2). M-H patients had a higher TMB when compared with M-L patients in ADC (p<0.05) and the same tendency was observed for SqCC (p=0.10) (Figure 3). Additionally, M-H correlated with lower PD-L1 (CD274) expression in both SqCC and ADC (each p<0.05) when compared with M-L. No significant differences in neoantigen burden were demonstrated. M-H patients showed a trend towards lower median overall survival in SqCC and ADC (75 vs 63 months p=0.21; 53 vs 50 months, p=0.14, Figure 3C,3D).

**Conclusions**

This is the first report that illustrates the implications of a microsatellite instability score on immune landscape, PD-L1 expression, TMB and clinical outcome from a pool of more than a thousand MSS non-small cell lung cancer patients.

**Reference**

1. Angelova M, Charoentong P, Hackl H, Fischer ML, Snajder R, Krogsdam AM, Waldner MJ, Bindea G, Mlecnik B, Galon J, Trajanoski Z. Characterization of the immunophenotypes and antigenomes of colorectal cancers reveals distinct tumor escape mechanisms and novel targets for immunotherapy. Genome Biol. 2015; 16:64.

**Fig. 1 (abstract P111).**
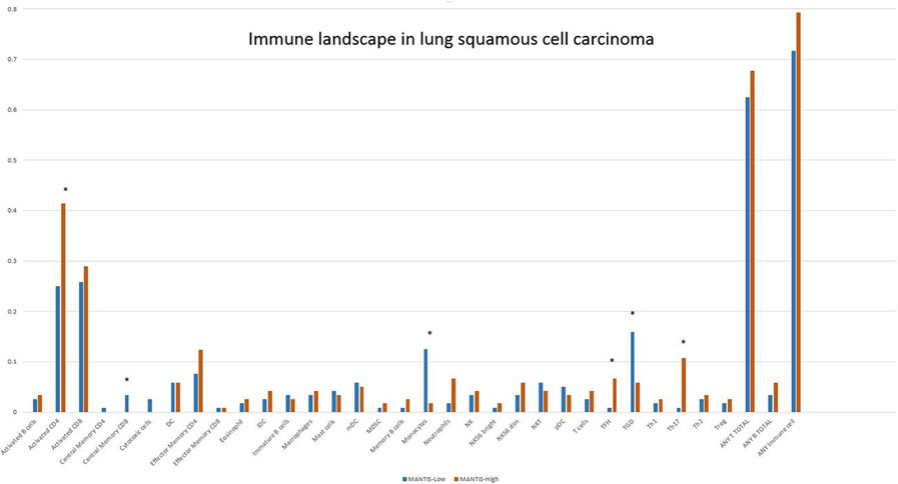
Immune landscape in squamous cell carcinoma

**Fig. 2 (abstract P111).**
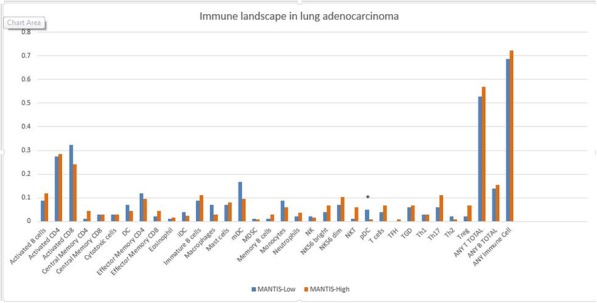
Immune landscape in adenocarcinoma

#### P112 A new way of immunity exploration by linking highly multiplexed antigen recognition to immune repertoire and phenotype

##### Dagmar Walter, PhD^1^, Stephane Boutet^1^, Michael Stubbington, PhD^1^, Katherine Pfeiffer^1^, Josephine Lee^2^, Luz Montesclaros^1^, Julia Lau^1^, Daniel Riordan^1^, Alvaro Martinez Barrio^1^, Liselotte Brix, PhD^3^, Kivin Jacobsen, PhD^3^, Bertrand Yeung^4^, Xinfang Zhao^4^, Tarjei Mikkelsen^1^

###### ^1^10x Genomics, Pleasanton, CA, United States; ^2^10x Genomics.com, Pleasanton, CA, United States; ^3^Immudex, Copenhagen, Denmark; ^4^Biolegend, San Diego,CA, United States

####### **Correspondence:** Tarjei Mikkelsen (tarjei@10xgenomics.com)

**Background**

Recent progress in cancer immunotherapy emphasizes the importance of understanding immune-regulatory pathways in cancer. It has been shown that immune cells play a crucial role in the tumor microenvironment and can be used for targeted therapeutics. Therefore, it is important to understand and characterize T cells and their antigen binding specificity and diversity in order to develop effective targeted immunotherapies. Recent technological advancements have enabled the integration of simultaneous cell-surface protein, transcriptome, immune repertoire and antigen specificity measurements at single cell resolution, providing comprehensive, scalable, high-throughput characterization of immune cells.

**Methods**

Using the 10x Genomics Single Cell Immune Profiling Solution with Feature Barcoding technology in conjunction with Biolegend oligo-conjugated antibodies and Immudex DNA barcoded peptide-MHC Dextramer® (pMHC), we performed multi-omic characterization of CD8+ T cell recognition of various virus and common cancer antigens in normal patients. Next generation sequencing libraries were made following the 10x Genomics workflow, where gene expression and immune repertoire libraries are generated alongside libraries from DNA barcodes conjugated to antibodies or pMHC, allowing quantification of cell surface proteins and identification of T cell receptor (TCR) specificities. Analysis was performed using the latest version of Cell Ranger (v3.0). The TCR-dist algorithm was used to identify clusters of related TCR sequences and enriched CDR3 motifs.

**Results**

We performed multi-omic characterization of ~100,000 CD8+ T cells from four MHC-matched donors. The multi-omic combination of gene expression, paired alpha/beta T cell receptor (TCR) repertoire, cell surface proteins and pMHC binding specificity allowed the identification of CD8+ T cell subpopulations with specificity for pMHCs within our panel. Within our data, we observed TCRs with cognate antigens that had been reported previously, while also identifying entirely new TCR–pMHC interactions. In addition, we observed specific expanded non-naïve T cell clones along with more diverse binding in the naïve compartment.

**Conclusions**

This rich and large dataset illustrates the power and scalability of the 10x Genomics Chromium Single Cell Immune Profiling Solution with Feature Barcoding technology and presents an exciting opportunity for researchers to explore and draw further conclusions about the mechanisms of TCR–pMHC interaction. Furthermore, this experiment serves as the next step on the path toward the even larger-scale experiments that will be necessary to fully comprehend the rules of antigen recognition in the adaptive immune system in response to cancer and infectious diseases and will be key in the development of successful immunotherapies.

**Acknowledgements**

This study was performed in collaboration with our 10x Genomics partners Immudex and Biolegend.

#### P113 Dynamic analysis and visualization of the immune infiltration in human cancer by integrating TCGA data

##### Mingchao Xie, PhD, Bolan Linghu, PhD, Zhongwu Lai, PhD, Jonathan Dry, Ben Sidders

###### AstraZeneca, Waltham, MA, United States

####### **Correspondence:** Jonathan Dry (Jonathan.Dry@astrazeneca.com); Ben Sidders (benjamin.sidders@astrazeneca.com)

**Background**

Understanding of the complex interplay between tumors and their immunologic microenvironment is critical for immune-oncology (IO) studies, which can facilitate the discovery of novel prognostic biomarkers, identification of new drug targets, and determination of drug resistance mechanisms. However, due to a lack of proper analysis tools and datasets, systematically exploring the tumor–immune interaction is still a big challenge.

**Methods**

Here, we deconvoluted the immune cell compositions and performed IO-related pathway/signature enrichment analysis for 9,721 primary tumor samples from 33 TCGA cancer types using transcriptomic data, and developed a web-based application, IO Browser.

**Results**

The browser allows the user to visualize the immune infiltrations of a sample or cohort, and to define disease segments or “immuno-types” based on the presence of single/multiple immune cell types or IO-related pathway/signatures. Users can then perform survival comparisons, explore gene expression of key cancer and IO genes as well as generate oncoprints in the different segments. The browser also provides statistical analysis to identify the gene or mutations enriched in the immuno-typed disease segment, and correlate gene expression or mutations with specific immune cell types in tumor microenvironment (TME).

**Conclusions**

In summary IO Browser enables comprehensive analysis and visualization of the dynamic interactions between tumor and immune landscape, and can aid our understanding of the interplay between tumor genomics and immune biology to facilitate line of sight and disease segmentation.

#### P114 Tissutal immune profile and pathological complete response in triple negative breast cancer

##### Andrea Botticelli, MD^1^, Bruna Cerbelli^1^ , Simone Scagnoli^1^, Maria Ida Amabile^1^, Alessandro De Luca^1^, Lucio Fortunato^2^, Leopoldo Costarelli^2^, Marianna Nuti, PhD^1^, Giulia D'Amati^1^, Paolo Marchetti^1^

###### ^1^Sapienza University of Rome, Rome, Italy; ^2^San Giovanni Addolorata Hospital, Rome, Italy

####### **Correspondence:** Bruna Cerbelli (bruna.cerbelli@uniroma1.it)

**Background**

In the neoadjuvant setting, pathological complete response (pCR) is more frequently achieved by triple negative breast cancer subtype and patients who attain this status show improved survival; However, standard neoadjuvant therapy results in pCR rates slightly over 30% of cases. The mechanism underlying the resistance to chemotherapy is still unclear and could be related both to the molecular heterogeneity of cancer cells and to the activation of the treatment-related immune response. For this reason, the search for immune biomarkers able to predict the response to chemotherapy represents a new promising frontier. Recent reports underscore the role of TILs, PDL-1 and CD73. The aim of our work is to define a novel tissutal immune profile (TIP) able to predict pCR [1,2].

**Methods**

We enrolled 61 pts who received NAC (EC for 4 cycles followed by Paclitaxel q7 for 12 cycles or q21 for 4 cycles) between Jan 2011 and June 2017 at Policlinico Umberto I and San Giovanni Addolorata Hospital of Rome. We performed, in basal paraffin-embedded biopsies, stromal TILS evaluation and immunohistochemistry for PD-L1 (Ventana SP142 clone) evaluated both on tumor cells (TC) and tumor-infiltrating immune cells (IC) and CD-73 assessed on TC. We defined “positive tissutal immune profile” (TIP+) the pts with “high TILS” (>50%), “PD-L1 positive” ( >1% both on TC and IC ) and “low CD73” (<40%), and the others as “negative tissutal immune profile” (TIP-).Statistical analysis was performed with T di Student test and χ2 test.

**Results**

We enrolled 61 females (median age: 50 y; range 28-75) affected by TNBC. The clinical stage before NAC was as follow: 3 pts cT3 (5%), 3 pts cT4 (5%) and 28 pts were cN+ (38%). Twenty-three patients (38%) showed pCR. No significant associations were found between pR and cT, cN, age, and KI-67. Seven patients (11%) were TIP+ and achieved pCR in 100% of cases; 54 patients were TIP- and pCR were showed in 16/54 of cases (30%) (p< 0,001).

**Conclusions**

TIP+ seems to be associated with higher pCR rate in TNBC patients .These preliminary results suggest the possibility of using novel profiles combining multiple immune- biomarkers.

**References**

1. Matsumoto H, Koo SL, Dent R, Tan PH, Iqbal J. Role of inflammatory infiltrates in triple negative breast cancer. J Clin Pathol. 2015;68:506–510. doi: 10.1136/jclinpath-2015-202944 139.

2. Jiang T, Xu X, Qiao M et al. Comprehensive evaluation of NT5E/CD73 expression and its prognostic significance in distinct types of cancers. BMC Cancer. 2018 Mar 7;18(1):267. doi: 10.1186/s12885-018-4073-7. PubMed PMID: 29514610; PubMed Central PMCID: PMC5842577.

**Ethics Approval**

CE 4181 Sapienza University of Rome

#### P115 Deep proteomic characterization of FFPE tumor samples from late-stage melanoma subjects treated with anti-PD-1 immunotherapy

##### Nicholas Dupuis, PhD^1^, Jakob Vowinckel, PhD^1^, Domenico Mallardo, MD^2^, Mariaelena Capone, MD^2^, Madonna Gabriele^2^, Antonio Sorrentino^2^, Vito Vanella^2^, Daniel Heinzmann^1^, Paolo Antonio Ascierto, MD^2^

###### ^1^Biognosys AG, Schlieren, Switzerland; ^2^Istituto Nazionale Tumori IRCCS, Naples, Italy

####### **Correspondence:** Paolo Antonio Ascierto (paolo.ascierto@gmail.com)

**Background**

Immune checkpoint inhibitors (ICI) have improved the treatment options for patients with advanced stage melanoma, with improved clinical responses and overall survival compared to standard systemic therapies. However, a large percentage of melanoma patients do not respond to ICIs, highlighting the need for a greater understanding of the tumor environment and host immune response. Here, we apply unbiased discovery proteomics, based on label-free data independent acquisition (DIA) mass spectrometry to deeply characterize global tumor proteomes to identify proteins and pathways that are associated with pre-treatment response to anti-PD-1 immunotherapy.

**Methods**

Unbiased, data-independent acquisition (DIA) mass spectrometry was used to analyze formalin fixed paraffin imbedded (FFPE) tumor tissue samples from subjects with Stage III-IV melanoma which were resected prior to initiation of first-line anti-PD-1 ICI therapy. The selected samples represent two distinct clinical subgroups; those who received clinical benefit, with a partial response or better (PR, SD and CR, n = 13), and those with no clinical benefit (PD, n = 9) and no observable response to therapy. Samples were prepared for mass spectrometry using standard procedures. All samples were analyzed using 2-hour gradients on a LC-MS/MS setup operated in DIA mode. Data was extracted using Spectronaut (Biognosys) with a sample specific spectral library which was combined with a large human tissue resource library. Statistical analysis was conducted to identify proteins that are either up- or down-regulated with respect to benefit group. Pathway analysis was also conducted to highlight dysregulated biological functions and pathways.

**Results**

7,590 proteins were quantified across all samples, with 6,627 quantified on average per sample. Univariate statistical testing between groups identified 254 proteins that are dysregulated (120 up-regulated and 134 down-regulated) in subjects who received clinical benefit. Through partial least squares discriminant analysis (PLS-DA) a set of 25 proteins was identified that describe the variance between the two sample groups. When annotated to their sub-cellular location, all up-regulated species are identified as mitochondrial proteins, indicating an enhanced metabolic environment, and the down-regulated species are cytosolic, lysosomal or membrane associated. This observation was also reflected in pathway analysis which identified up-regulation of arginine and citrulline metabolism and down-regulation of adhesion related processes driven by MHC-II and integrins.

**Conclusions**

Global profiling of the tumor proteome provides a unique characterization of melanoma tumor biology. A pathway level analysis shows increased metabolic processes combined with decreases in adhesion related proteins may underly the differences in benefit related to ICI therapy.

**Ethics Approval**

The study was approved by the Istituto Nazionale Tumori IRCCS Fondazione “G. Pascale” of Napoli Institutution‘s Ethics Board, approval number 33/17.

**Consent**

Written informed consent was obtained from the patient for publication of this abstract and any accompanying images. A copy of the written consent is available for review by the Editor of this journal.

#### P116 Centrifuge-free red blood cell lysis and immunostaining of whole blood for flow cytometry using Laminar Wash™ system

##### Ira Kim, Melvin Lye, Chyan Ying Ke, Nadiezda Fernandez Oropeza, Sigeeta Rajaram, Kong Leong Cheng, Ih Chin Kon, Royce Pek, Namyong Kim, PhD

###### Curiox Biosystems, Boston, MA, United States

####### **Correspondnce:** Namyong Kim (namyong@curiox.com)

**Background**

Blood cells are prime indicators of immuno-surveillance, and the ease of blood sampling makes blood analysis a key interest for clinical and research applications. While current flow cytometry methods are high-throughput and provide fine resolution in the segregation of white blood cell (WBC) populations, WBC enrichment involving red blood cell (RBC) lysis are laborious and typically performed manually, contributing to experimental variability especially as blood cells are sensitive to physical and chemical stress.

**Methods**

We describe RBC lysis and leukocyte immunostaining on a centrifuge-less platform Laminar Wash™, using a novel wall-less plate and laminar flow washer. The Laminar Wash™ 24-well plate consists of an array of hydrophilic spots surrounded by hydrophobic surface, which functions as a virtual wall that separates each spot. Each well is capable of staining and lysing 100uL of whole blood. During lysis, WBCs settle to the surface of the spot, allowing the spent lysis buffer to be removed by a gentle and continuous laminar-flow washing process on the Laminar Wash™ system, eliminating centrifugation and resuspension that may stress cells and disrupt antibody binding.

**Results**

We observed improved retention of CD45+ lymphocytes while lysing on Laminar Wash™ plates compared to conventional centrifuge tubes. In studies comparing mouse whole blood lysis and antibody staining by conventional tube centrifuge and Laminar Wash, Laminar Wash achieved dramatically higher staining index and improved resolution of cell cluster by flow cytometry.

**Conclusions**

In summary, Laminar Wash system provides gentle, fast and convenient blood lysis, while improving data quality with superior antibody staining.

#### P117 Immunogram to decipher PD1/L1 ICI resistance: a proof of concept in advanced Non-small cell lung cancer patients of the PIONeeR Project

##### Florence Monville, PhD^1^, Frederic Vely^2^, Joseph Ciccolini^3^, Florence Sabatier^2^, Stephane Garcia^2^, Vanina Leca^1^, Marion Fabre^4^, Christelle Piperoglou^4^, Pernelle Outters^1^, Laurent Arnaud^4^, Laurent Vanhille, PhD^1^, Caroline Lauge, BA^1^, Anna Martirosyan, Dr^1^, Aurelie Collignon^1^, Marie Roumieux^5^, Julien Mazieres^6^, Maurice Perol^7^, Françoise Dignat-George^2^, Eric Vivier^2^, Fabrice Barlesi, MD, PhD^2^, Jacques Fieschi, PhD^1^

###### ^1^HalioDx, Marseille, France; ^2^AMU, APHM, Marseille, France; ^3^AMU, APHM, IPC, Marseille, France; ^4^APHM, Marseille, France; ^5^AMU, Marseille, France; ^6^Toulouse Universitary Hospital, Toulouse, France; ^7^Centre Leon Berard, Lyon, France

####### **Correspondence:** Jacques Fieschi (jacques.fieschi@haliodx.com)

**Background**

In the management of advanced Non-Small Cell Lung Carcinoma (NSCLC), PD1/L1 immune checkpoint inhibitors (ICIs) have increased overall survival (OS) over standard second-line chemotherapy. While this long-term increase in OS is driven by about 20% of patients, others display disease progression during the first weeks. PIONeeR workpackage 2 aims to understand and eventually predict response and/or resistance to those ICIs in stage IV or recurrent NSCLC patients. For that purpose, an Immunogram was designed that integrates a comprehensive set of biomarkers measured in the tumor microenvironment.

**Methods**

The immune contexture from the PIONeeR trial’s patients is being characterized in a prospective manner and will be confronted to clinical data at the end of the study. This multi-modal approach, encompassing a range of immune scoring assays, is applied to blood and tumor biopsy from each patient, both sampled before and throughout anti-PD1/L1 ICI treatment. This work aims at describing pre-treatment samples profiling.

**Results**

We assessed the feasibility of such a profiling and provide descriptive multi-modal immune profiles for the 10 first PIONeeR-included patients. These profiles combine raw results from more than ten tests, corresponding to the following technologies and biomarkers: Genomic Next Generation Sequencing for Tumor Mutational Burden (TMB), DNA mismatch-repair deficiency (MSI/MSS status) and T Cell Clonality assessments ; dualplex and multiplex immunohistochemistry coupled to digital pathology analyses to assess Immune Cells Infiltration and PD-L1 mediated inhibition (Immunoscore® IC), Immune Suppression through Regulatory T cells and Myeloid-derives suppressor cells quantification, T-Cell Exhaustion status ; standardized methods for assessment of endothelial activation markers ; flow cytometry for circulating immune cell subtypes quantification; ICI plasma exposure levels. A multimodal integrative Immunogram presentation is proposed for each patients.

**Conclusions**

This preliminary study shows that multimodal immune profiling is feasible and could be a new tool to understand the biology and pharmacology of lung cancer resistance to anti-PD1/L1 ICIs and potentially guide patient management décisions.

**Acknowledgements**

This work is supported by the French National Cancer Agency, Agence Nationale du Cancer, through the PIONeeR project financing.

**Trial Registration**

ClinicalTrials.gov Identifier: NCT03493581

**Ethics Approval**

The study was approved by the French Ethic Comitee CPP Ouest II Angers, approval number 2018/08.

#### P118 HYDRA platform development to investigate Siglec-engaging tumor immunosuppressive glyco-codes

##### Li Peng, PhD, Adam Petrone, Adam Shoemaker, Jillian Prendergast, PhD, Zakir Siddiquee, Jenny Che, Lihui Xu, BS, Karl Normington, PhD, MBA, James Broderick, Li Peng, PhD

###### Palleon Pharmaceuticals, Waltham, MA, United States

####### **Correspondence:** Li Peng (lpeng@palleonpharma.com)

**Background**

The glyco-immune checkpoint (Siglec/sialoglycan axis) has emerged as a new mechanism of cancer immune escape and offers new therapeutic interventions to overcome resistance to current immunotherapies. Siglecs (sialic acid-recognizing Ig-superfamily lectins) are type I transmembrane sialoglycan binding proteins expressed on various immune cells (innate and adaptive). Humans express at least fourteen unique Siglecs which have distinct preferred sialoglycan ligands. Tumors upregulate certain sialoglycan patterns to facilitate immune cell evasion by engaging these inhibitory Siglec receptors. This tumor inhibitory “glyco-code” consists of a heterogenous mixture of numerous sialoglycans, binding to Siglecs through low affinity and high avidity interactions.

**Methods**

Deciphering the hypersialylation glyco-code of tumors is key to identifying cancer patients for glyco-immune checkpoint blockade therapies. However, the heterogeneity and complexity of sialoglycans make characterization of the tumor surface sialoglycome difficult with current technologies. To overcome this challenge, we developed a proprietary sialoglycan-probing reagent, HYDRA, to functionally detect inhibitory tumor sialoglycans engaging Siglecs. HYDRA mimics this natural avidity driven Siglec-sialoglycan interaction, consisting of multimeric fusions of a Siglec N-terminal extracellular domain containing the carbohydrate recognition domain (CRD), a trimerization motif, and a Fc dimerization domain.

**Results**

We have generated several HYDRA constructs with robust expression using a mammalian HEK293 system. Size-exclusion chromatography profiles of HYDRA demonstrate high purity and confirmed multimeric assembly. HYDRAs have greater than fifteen-fold increase in binding affinity compared to Siglec-Fc dimers as measured using bio-layer interferometry Octet. HYDRA also demonstrates sialoglycan-specific binding, as its binding was eliminated when cells were treated with sialidase (which removes terminal sialic-acids of sialoglycan) or using cells lacking sialoglycans from knocking out UDP-GlcNAc 2-Epimerase. Glycan array binding of HYDRA confirmed similar sialoglycan preferences of its Siglec counterpart as described in the literature, suggesting engineering did not alter glyco-recognition properties. These high-affinity and sialoglycan-specific HYDRAs enabled us to develop a robust immunohistochemistry (IHC) assay to analyze cancer patient samples. A cohort of tissues (>2,500 patients) from various indications were analyzed to enable indication prioritization for glyco-immune checkpoint therapies. HYDRA IHC on healthy and cancerous human tissues demonstrate unique binding patterns with concordance between duplicate primary tumor cores and primary tumor versus metastatic cores from the same patient in non-small cell lung, kidney and colon cancer samples.

**Conclusions**

In summary, the HYDRA technology distills the structural heterogeneity of tumor surface sialoglycans to a straightforward functional readout of immunosuppressive glyco-codes engaging inhibitory Siglecs, which may allow patient stratification based on deciphering a tumor-specific surface glycan pattern.

#### P119 Analytical validation of run-to-run and site-to-site performance of a human immune profiling assay and automated data analysis solution for CyTOF mass cytometry technology

##### Clare Rogers (clare.rogers@fluidigm.com)

###### Fluidigm, South San Francisco, CA, United States

**Background**

Immune profiling is an essential method for quantifying changes in immune population numbers and states over time in health and disease. A cornerstone in translational and clinical research, it is frequently used to investigate chronic inflammation, infectious disease, autoimmune diseases, and cancer. The diversity of immune populations demands a high parameter approach to more fully and efficiently quantify these changes. Mass cytometry, which utilizes CyTOF® technology, is a single-cell analysis platform that has used as many as 50 metal-tagged antibodies [1] to resolve discrete cell populations, all in a single tube of sample. It is an ideal solution for routine enumeration of immune cell populations.

**Methods**

We have developed a sample-to-answer solution for human immune profiling using mass cytometry: the Maxpar® Direct™ Immune Profiling System. It includes an optimized 30-marker immune profiling panel provided in a dried single-tube format, validated SOPs for human whole blood and PBMC staining, an instrument data acquisition template, instructions for data acquisition on a Helios™ system, and automated Maxpar Pathsetter™ software for data analysis.

**Results**

Here we present assay analytical validation data on repeatability, reproducibility, software precision, software accuracy, and site-to-site reproducibility. The repeatability of eight identical donor samples acquired on a single Helios instrument resulted in CVs 5% in frequency). Reproducibility of three identical samples acquired on three different Helios instruments resulted in CVs

**Conclusions**

We conclude that this assay provides a robust solution for broad immune profiling using mass cytometry, reducing sources of variability and subjectivity in sample preparation and data analysis.

**Reference**

1. Simoni Y, Becht E, Fehlings M et al. Bystander CD8+ T cells are abundant and phenotypically distinct in human tumour infiltrates. Nature. 2019; 557:575–579.

#### P120 Evaluation of CD8 score by automated quantitative image analysis in metastatic melanoma treated with PD1 blockade: preliminary results

##### Anjali Rohatgi, MD PhD, Douglas Hartman, Arivarasan Karunamurthy, Julie Burkette, Yana Najjar, MD, John Kirkwood, MD, Hassane Zarour, MD, Liron Pantanowitz, Diwakar Davar, MD

###### UPMC, Pittsburgh, PA, United States

####### **Correspondence:** Diwakar Davar (davard@upmc.edu)

**Background**

PD1 blockade produces responses in 30-40% of metastatic melanoma (MEL) with durable relapse-free benefit [1,2]. Pre-existing tumor-infiltrating CD8+T cell infiltrates (TIL), neoantigen burden and IFN-γ gene expression signature (GES) correlate with clinical anti-tumor response [3-5] to PD1 blockade. However, neoantigen burden and IFN-γ GES are cost-prohibitive and time-consuming assays that are not available for clinical use; while CD8 T cell analysis by immunohistochemistry (IHC) is cost-effective and operator-independent. The aim of this study is to develop and validate an image analysis algorithm to automatically quantify CD8+ T cells (CD8 score) in patients with metastatic MEL treated with PD1 blockade.

**Methods**

Included patients had advanced metastatic MEL treated with PD1 blockade. Radiographic response assessed using RECIST v1.1. For the purposes of this analysis, patients were defined as responders (R; complete, partial response, stable disease) or non-responders (NR; progressive disease). Pre-treatment tumor biopsies from 58 patients were utilized. Brightfield image analysis results were cross-validated with fluorescence-based quantification (AQUA™). A nuclear image algorithm designed to run on whole slide images was optimized to manual count. The algorithm was locked down and used on a cohort of whole tissue sections from MEL patients. All images were reviewed by independent pathologist blinded to clinical outcomes. Response and outcomes were statistically correlated with image analysis results.

**Results**

There were 40 R patients and 18 NR patients. Median CD8 score was 101 cells/mm3 in R and 48.7 cells/mm3 in NR (p=0.098). Median PFS were greater in R compared to NR (18 months vs. 2 months, p100 cells/mm3 (64%).

**Conclusions**

We report the successful technical development and clinical validation of an image algorithm to automate CD8 score for metastatic MEL treated with PD1 blockade. Preliminary results demonstrate CD8 score was directly associated with response and improved PFS. CD8 score is an assay that could be carried out using existing technology in pathology departments. Further analysis will focus on validating these results in a larger cohort to permit clinical use.

**References**

1. Ribas A, Hamid O, Daud A, Hodi FS, Wolchok JD, Kefford R, Joshua AM, Patnaik A, Hwu WJ, Weber JS, Gangadhar TC, Hersey P, Dronca R, Joseph RW, Zarour H, Chmielowski B, Lawrence DP, Algazi A, Rizvi NA, Hoffner B, Mateus C, Gergich K, Lindia JA, Giannotti M, Li XN, Ebbinghaus S, Kang SP, Robert C. Association of Pembrolizumab With Tumor Response and Survival Among Patients With Advanced Melanoma. JAMA. 2016; 315:1600-9.

2. Larkin J, Lao CD, Urba WJ, McDermott DF, Horak C, Jiang J, Wolchok JD. Efficacy and Safety of Nivolumab in Patients With BRAF V600 Mutant and BRAF Wild-Type Advanced Melanoma: A Pooled Analysis of 4 Clinical Trials. JAMA Oncol. 2015;4:433-40.

3. Tumeh P, Harview C, Yearley J, Shintaku I, Taylor E, Robert L, Chmielowski B, Spasic M, Henry G, Ciobanu V, West A, Carmona M, Kivork C, Seja E, Cherry G, Gutierrez A, Grogan T, Mateus C, Tomasic G, Glaspy J, Emerson R, Robins H, Pierce R, Elashoff D, Robert C, Ribas A. PD-1 blockade induces responses by inhibiting adaptive immune resistance. Nature. 2014;515:568-71

4. Cristescu R, Mogg R, Ayers M, Albright A, Murphy E, Yearley J, Sher X, Liu XQ, Lu H, Nebozhyn M, Zhang C, Lunceford JK, Joe A, Cheng J, Webber AL, Ibrahim N, Plimack ER, Ott PA, Seiwert TY, Ribas A, McClanahan TK, Tomassini JE, Loboda A, Kaufman D. Pan-tumor genomic biomarkers for PD-1 checkpoint blockade-based immunotherapy. Science. 2018;362:197

5. Rizvi NA, Hellmann MD, Snyder A, Kvistborg P, Makarov V, Havel JJ, Lee W, Yuan J, Wong P, Ho TS, Miller ML, Rekhtman N, Moreira AL, Ibrahim F, Bruggeman C, Gasmi B, Zappasodi R, Maeda Y, Sander C, Garon EB, Merghoub T, Wolchok JD, Schumacher TN, Chan TA. Mutational landscape determines sensitivity to PD-1 blockade in non-small cell lung cancer. Science. 2015;348:124-8.

**Ethics Approval**

The study was approved by University of Pittsburgh‘s Institutional Review Board, approval number PRO18080253.

**Consent**

Written informed consent was obtained from the patient for publication of this abstract and any accompanying images. A copy of the written consent is available for review by the Editor of this journal.

#### P121 Cancer Immunogram: combining multi-parameter approach and machine learning to capture the complexity of tumor immune contexture

##### Thomas Sbarrato, PhD^1^, Laurent Vanhille, PhD^1^, Mounia Filahi^1^, Anna Martirosyan, PhD^1^, Véronique Frayssinet^1^, Caroline Davin, BA^1^, Caroline Laugé^1^, Assil Benchaaben^1^, Alboukadel Kassambara^1^, Felipe Guimaraes^1^, Régis Perbost^1^, Jérôme Galon^2^, Hélène Girardi^1^, Jacques Fieschi, PhD^1^

###### ^1^HalioDx, Marseille, France, ^2^Centre de Recherche des Cordeliers, Paris, France

####### **Correspondence:** Jacques Fieschi (jacques.fieschi@haliodx.com)

**Background**

To tailor clinical care and personalized treatment of cancer patients, the scientific community together with the practitioners have focused into refining our understanding of cancer biology and resistance to treatments. In that perspective, the concept of Immunoscore proposed by Galon et al [1, 2, 3] has highlighted the crucial role of immune response to the tumor. In parallel, immunotherapies by immune checkpoint inhibitors (ICI) anti-PD-1/PD-L1 were approved in several cancer indications, such as Non-Small Cell Lung Cancer or melanoma, even if only a minority of these patients respond positively to the treatment. In addition, ICI are far less effective for other high-incidence indications like colorectal cancer (CRC), thus suggesting that multiple factors may be critical for capturing the exact nature of the tumour microenvironment (TME). In this context, the comprehensive identification and assessment of these factors could be key to stratify patients and allow the selection of the optimal treatment.

**Methods**

In order to support clinical researchers and biopharmaceutical companies in the evaluation of the efficacy of candidate drugs, HalioDx has developed the Cancer Immunogram, a solution based on Blank CU et al. [4]. Our multi-parameter approach encompassing a unique range of immune scoring assays is based on the analysis and the understanding of the immune contexture of tumors and offers a personalized and dynamic “fingerprint” of tumor-immune system interaction. To address this, the Cancer Immunogram combines different technologies and biomarkers to assess 1) the tumor characteristics (Tumor foreignness, MSI, PD-L1 expression, common mutation drivers), 2) the immune infiltration (Immunoscore®, CD8/PD-L1 proximity, TCR clonality, immune expression signature), 3) the immune checkpoint status (T Cell Exhaustion BrightPlex panel) and 4) the immune suppression status (Treg, MDSC and M1/M2 macrophage BrightPlex panels).

**Results**

Here, we consolidate our Proof of Concept for the Cancer Immunogram in the context of CRC [5] by leveraging this meta-analysis on a 20-patients cohort. Using machine learning algorithms to extract the most relevant features, we show that the Cancer Immunogram allows to identify patient-specific patterns which might improve the prediction of the response to therapy.

**Conclusions**

We believe that the Cancer Immunogram has the potential to facilitate drug development by providing a 360° vision of the tumour immune contexture and may also help clinicians to personalize advanced cancer patient care.

**References**

1. Galon J, Bruni D. Approaches to treat immune hot, altered and cold tumours with combination immunotherapies. Nat Rev Drug Discov. 2019;18:197-218.

2. Pagès F et al. International validation of the consensus Immunoscore for the classification of colon cancer: a prognostic and accuracy study. Lancet. 2018; 391:2128-2139.

3. Mlecnik B et al. Integrative Analyses of Colorectal Cancer Show Immunoscore Is a Stronger Predictor of Patient Survival Than Microsatellite Instability. Immunity. 2016; 44:698-711.

4. Blank CU et al. The "cancer immunogram". Science. 2016;; 352:658-60.

5. Sbarrato T et al, Combining multimodal biomarkers as an immunogram to guide immunotherapy use: A proof of concept. Proceedings: AACR Annual Meeting 2019; March 29-April 3, 2019; Atlanta, GA.

#### P122 Microfluidic-based cell separation method improves workflow for evaluation of rare lymphocytes from cancer patient samples

##### Jodi Stone, BS^1^, Megan Nichols^1^, Amy Austin^1^, Kala Bradshaw^1^, Jessica E. Norris, BS, MT^1^, Jennifer Montague, PhD^1^, Peng Meng Kou^2^, Nitin Kulkarni^2^, Nirav Sheth^*2*^, Anya Manning, MBA^*2*^, Sarah M. Mickool^*2*^, Kyle Smith^2^, Ravi Kapur^*2*^, John Powderly, MD, CPI^1^

###### ^1^Carolina BioOncology Institute, Huntersville, NC, United States; ^2^MicroMedicine, Waltham, MA, United States

####### **Correspondence:** John Powderly (jpowderly@carolinabiooncology.org)

**Background**

Isolation of rare lymphocyte populations from peripheral blood products of cancer patients can be challenging due to technician variability and substantial cell loss through standard cell separation methods such as Mononuclear Cell Preparation Tubes™ (CPTs). An automated microfluidic approach was evaluated to determine lymphocyte recovery, processing time, and ease of use. Furthermore, increased yields of rare cells from cancer patients’ peripheral blood could potentially substitute for leukapheresis when leukapheresis is not a viable option.

**Methods**

White blood cells (WBCs) or peripheral blood mononuclear cells (PBMCs) were isolated from human peripheral blood using either MicroMedicine’s Microfluidic System (MS) or CPTs. Cell viability and lymphocyte recovery were compared using a hematology analyzer and flow cytometry. Further, a rare lymphocyte population was positively immunomagnetically selected from healthy volunteers and cancer patients. Immunophenotyping was performed pre- and post-cell selection, followed by *in vitro* expansion of the rare lymphocytes.

**Results**

Using cells collected from healthy volunteers, the automated MS prototype consistently recovered 83.5 ± 10.1% lymphocytes in a total of 31 ± 5.8 minutes, including hands-on time, compared to the standard CPT process, which recovered 43.2 ± 7.6% lymphocytes in 72.2 ± 4.1 minutes, from 32 – 34 mL blood samples (n = 5). The viability was comparable at 97.9 ± 1.6% (MS) and 96.7 ± 3.0% (CPT). In further studies with both healthy donors and cancer patients, a rare lymphocyte population was successfully selected from WBCs isolated with the MS, enabling immunophenotyping of the rare cell population and subsequent *in vitro* expansion. Expansion of this lymphocyte population from a colon adenocarcinoma patient was found to be suppressed post-immunotherapy compared to pre-treatment. Cells isolated from patients with other malignancies were successfully expanded. Finally, peripheral blood collected from cancer patients yielded a greater number of rare lymphocytes using the MS for the cell expansion study in comparison to the CPT isolation method, which correlates to having a higher lymphocyte recovery.

**Conclusions**

The MS consistently recovered approximately twice the number of lymphocytes in half the time compared to the traditional CPT method. The automated cell separation process improves the consistency of cell isolation while freeing up technician time. Rare lymphocyte populations could be reliably recovered from peripheral blood with significantly higher yield compared to the CPT method. While leukapheresis enriches for MNCs and MS isolates all WBCs, these results suggest that peripheral blood collection-based MS has the potential to complement leukapheresis, especially for small- to medium-scale studies.

#### P123 Identification of mRNA signatures that predict response to immunotherapy in melanoma patients

##### Ioannis Vathiotis, MD^1^, Amy Sullivan^2^, Sarah Warren, PhD^2^, Nicole Gianino^1^, Sandra Martinez-Morilla, PhD^1^, Pok Fai Wong, MD, MPhil^1^, Harriet Kluger, MD^1^, Konstantinos Syrigos^3^, David Rimm, MD, PhD^1^

###### ^1^Yale University, New Haven, CT, United States; ^2^NanoString Technologies, Seattle, WA, United States; ^3^University of Athens, Athens, Greece

####### **Correspondence:** David Rimm (david.rimm@yale.edu)

**Background**

Currently, there is no diagnostic test that can accurately predict response in melanoma patients treated with immunotherapy. NanoString® nCounter® PanCancer IO 360™ panel (Research Use Only) measures mRNA from 770 genes related to the tumor and host immune response. Here, we used this panel to assess the predictive value of individual genes and weighted gene signatures in a cohort of immunotherapy (ITx) treated melanoma patients.

**Methods**

We used pretreatment, formalin-fixed paraffin-embedded (FFPE) whole tissue sections from 59 melanoma patients that received single agent or combination immunotherapy (pembrolizumab, nivolumab, or nivolumab plus ipilimumab). Two slides from each patient were macrodissected and RNA was extracted. The mRNA transcripts were hybridized and tagged by unique probes for the 770-plex PanCancer IO 360 panel and then measured on the nCounter platform. RNA counts were correlated with best overall response (BOR), clinical benefit (CB), progression free survival (PFS) and overall survival (OS).

**Results**

Indoleamine 2,3-dioxygenase 1 (IDO1) was the best single gene predictor of BOR (Area under the curve (AUC) = 0.73) and CB (AUC = 0.70). Among other genes, IDO1 mRNA was also found to be significantly associated with longer PFS (P < 0.01, False discovery rate (FDR) = 0.18) and OS (P < 0.01, FDR = 0.052). The previously described 18-gene tumor inflammation score (Ayers TIS) validated for the prediction of BOR (AUC = 0.68), PFS (P < 0.05, FDR = 0.18) and OS (P < 0.001, FDR = 0.025). TIS also predicted CB (AUC = 0.67). Its predictive value remained the same irrespective to immunotherapy agent administered. Nevertheless, it decreased for patients harboring the BRAF and NRAS mutations (AUC = 0.76 versus 0.51 and 0.44 for patients with BRAF and NRAS mutations respectively). The best signatures for this cohort were for Cytotoxicity, Immunoproteasome and CD56dim Cells which were predictive for BOR (AUC = 0.72, 0.71 and 0.70 respectively), CB (AUC = 0.69, 0.68 and 0.70 respectively), and OS (all FDRs < 0.05). Further work is underway to compare these Yale melanoma results with other cohorts.

**Conclusions**

Pretreatment mRNA counts of single genes or weighted signature scores are related to immunotherapy outcomes in melanoma patients. This work validated the Ayers TIS signature and highlighted the role of the immune microenvironment, especially NK cells, in mediating antitumor response after immune checkpoint inhibition.

NanoString nCounter is Intended for Research Use Only. Not for Use in Diagnostic Procedures.

**Ethics Approval**

The study was approved by Yale University Human Investigation Committee, approval number 9505008219.

#### P124 A novel cell-mediated immunotherapy for treatment of lung and breast cancer

##### Indu Venugopal, PhD, Kathlynn Brown, Michael McGuire, Claire Gormley

###### SRI International, Harrisonburg, VA, United States

####### **Correspondence:** Kathlynn Brown (kathlynn.brown@sri.com)

**Background**

Cancer Immunotherapies designed to generate a cell-mediated immune response against tumors are emerging as frontline treatment options for cancer; however, concerns regarding efficacy, safety and cost efficacy have limited the use of these treatments.

**Methods**

To address these weaknesses, we developed a novel immunotherapy capable of delivering previously encountered antigenic peptides specifically to cancer cells and facilitating their presentation through the MHC class I pathway. It utilizes a synthetic nanoparticle delivery system comprised of three components: a neutral stealth liposome, encapsulated synthetic immunogenic HLA class I restricted peptides derived from measles virus (MV), and a tumor-targeting peptide on the external surface of the liposome. The targeting peptide results in accumulation of liposomes specifically inside cancer cells, and facilitates presentation of MV-derived immunogenic peptides in HLA class I molecules (Figure 1). We refer to this system as TALL (Targeted Antigen Loaded Liposomes). Therefore, TALL can generate a secondary immune response specifically against the targeted tumor cells in a patient who has been previously vaccinated against or infected by MV. In short, we are attempting to trick the immune system into responding as though the cancer cell is infected with MV without the use of viral particles.

**Results**

We synthesized liposomes encapsulating H250, an immunogenic HLA class I restricted peptide identified from measles hemagglutinin protein. These liposomes were targeted to breast and lung cancer cells via our targeting peptide, which was identified using phage-display methodology. Treatment of lung cancer cells with TALL results in functional presentation of H250 in both MHC and HLA class I molecules. Our in-vitro and in-vivo studies indicate that presentation of H250 is dependent on the cancer targeting peptide; liposomes that lack the targeting peptide did not accumulate in the cancer cells and presentation of H250 was abrogated. Treatment with TALL substantially reduced growth of LLC1 and 4T1 tumors in vaccinated C57BL/6 and Balb/c mice respectively.

**Conclusions**

The outcome of our therapy is a robust cytotoxic T lymphocyte response directed specifically against the tumor. It's advantages include: 1) Bypassing the need to identify tumor-associated antigens or educate the immune system through a primary immune response; 2) It is anticipated to be effective against tumors with a low mutational load, making it efficacious on early-stage and metastatic cancer; 3) It does not use a live virus or biologically-derived material, allowing for complete synthetic manufacturing. It also does not require isolation or ex-vivo manipulation of patient’s cells, reducing production time and costs.

**Acknowledgements**

Research reported in this work was supported by DOD under grant number W81XWH-16-1-0262 and SRI internal research funds

**Fig. 1 (abstract P124).**
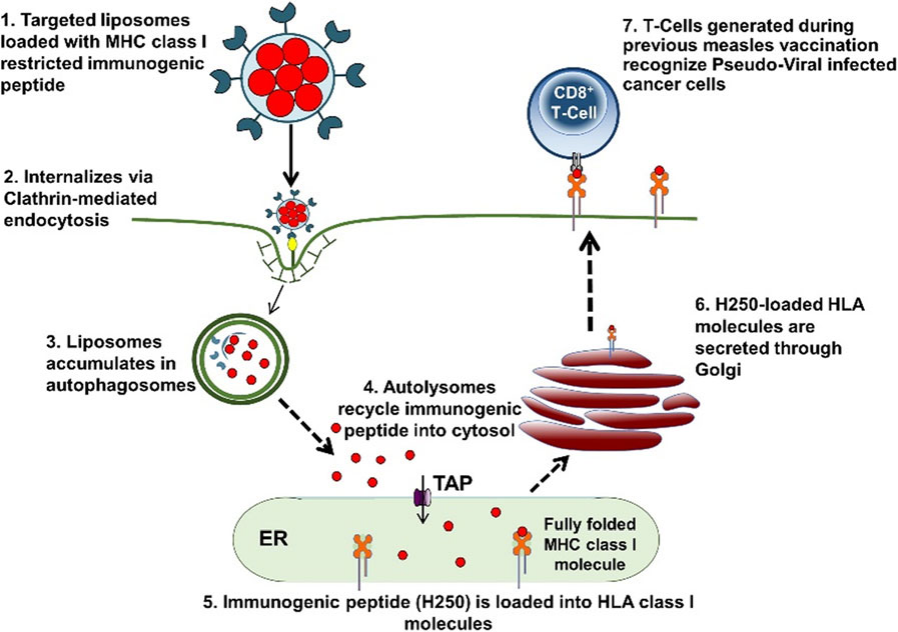
TALL mechanism of action

#### P125 Adenosine and AMP gene expression profiles predict response to adenosine pathway therapies and indicate a need for dual blockade of CD73 and A2AR with CD73 inhibitors

##### Stephen Willingham, PhD, Drew Hotson, PhD, Jessica Hsieh, Brian Munneke, Long Kwei, PhD, Joseph Buggy, Richard Miller, MD

###### Corvus Pharmaceuticals, Burlingame, CA, United States

####### **Correspondence:** Stephen Willingham (swillingham@corvuspharma.com)

**Background**

Extracellular adenosine in the tumor microenvironment generates an immunosuppressive niche that promotes tumor growth and metastasis by signaling through the A2A receptor (A2AR) on immune cells. Various agents targeting the adenosine pathway are now in clinical trials as cancer therapies. Ciforadenant is a selective A2AR antagonist and CPI-006 is an anti-CD73 antibody (Fc-mutant IgG1) that blocks the enzymatic conversion of AMP to adenosine and directly stimulates immunity. Both agents are now being studied in clinical trials (NCT02655822 and NCT03454451). In this report, we evaluate the role of adenosine and AMP-related gene expression profiles (GEPs) that may predict the response of patients receiving adenosine pathway therapies. Ex vivo studies reveal a requirement for dual blockade of CD73 and A2AR for optimal neutralization of AMP mediated immunosuppression.

**Methods**

Normal human PBMCs were stimulated ex vivo with NECA (stable adenosine analog) or AMP. RNA from tumor biopsies and PBMC was analyzed using NanoString. Renal cell cancer (RCC) tumor biopsies collected from patients treated with ciforadenant (100 mg BID) either as a single agent (n=18) or in combination with atezolizumab (n=14).

**Results**

Ex vivo A2AR agonism resulted in dose-dependent increases in CXCR2 ligands (CXCL1,2,3,5,8) and key mediators of neutrophil/MDSC biology (CSF3, IL-23). Increases in monocyte/macrophage inflammatory mediators such as IL-1beta and CCL2,3,7,8, 20 were also observed, as were increases in SERPINB2, S100A8, PTGS2, THBS1. Preliminary biomarker analysis suggests ciforadenant anti-tumor activity in RCC was associated with increased expression of select analytes (AdenoSig) in pretreatment biopsies (Figure 1).

Ex vivo AMP or AMPalphaS (a non-hydrolyzable AMP analog) stimulation induced a similar GEP (AMPSig), but included specific decreases in OAS3, BIRC5, CDK1, MX1, IFI27, and IFIT1. CD73 antibody and small molecule antagonists amplified the AMPSig by preserving AMP, which itself directly stimulates adenosine receptors. In contrast, ciforadenant inhibited induction of the AdenoSig and AMPSig in all experimental settings at the transcript and protein level.

**Conclusions**

A2AR agonists and AMP induce specific GEPs dominated by immunosuppressive mediators of MDSC and monocyte/macrophage biology. These GEPs may be used as biomarkers for patient selection. CD73 antagonists alone may be limited by the induction of compensatory immunosuppressive pathways mediated by AMP accumulation. Combination ciforadenant and CPI-006 treatment may synergize to activate anti-tumor immunity by 1) blocking adenosine production and signaling, 2) directly activating immune cells, and 3) blocking a compensatory induction of AMPSig. This combination strategy is being evaluated in an ongoing Ph1/1b clinical trial in patients with advanced solid tumors.

**Trial Registration**

NCT02655822 and NCT03454451

**Fig. 1 (abstract P125).**
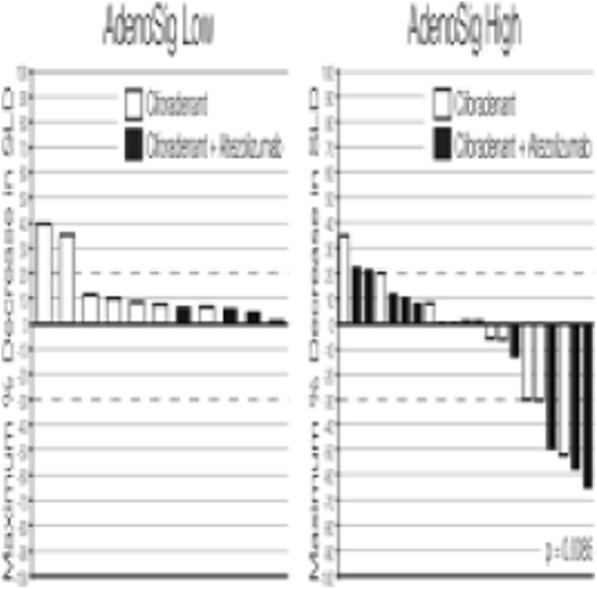
See text for description

#### P126 Discovery of biomarkers associated with benefit from PD-1 checkpoint blockade in non-small-cell lung cancer (NSCLC) using high-plex digital spatial profiling

##### Jon Zugazagoitia, MD, PhD^1^, Swati Gupta, PhD^1^, Kit Fuhrman, MS PhD^2^, Scott Gettinger, MD^1^, Roy Herbst, MD, PhD^1^, Kurt Schalper, MD, PhD^1^, David Rimm, MD, PhD^1^

###### ^1^Yale University School of Medicine, New Haven, United States; ^2^Nanostring, Inc., Seattle, WA, United States

####### **Correspondence:** David Rimm (david.rimm@yale.edu)

**Background**

Only a minority of patients with advanced NSCLC truly benefit from single-agent PD-1 checkpoint blockade, and more robust predictive biomarkers are needed to optimally deliver these therapies. The GeoMx Digital Spatial Profiler (DSP) (NanoString, Inc.) allows high-plex protein expression analysis in a quantitative and spatially-resolved manner from single formalin-fixed paraffin embedded tissue sections. Here we use this technology as a discovery tool to find protein markers associated with benefit from single-agent PD-1 checkpoint blockade in NSCLC.

**Methods**

We used the GeoMx DSP in a cohort of 63 immunotherapy-treated NSCLC cases represented in a tissue microarray, 52 of whom had pre-treatment samples and received single-agent PD-1 checkpoint blockade. A panel of 40 photocleavable oligonucleotide-labeled primary antibodies (NanoString Human IO panel) was used for protein detection. Proteins were measured in 4 independent molecularly-defined tissue compartments by fluorescence co-localization (tumor [panCK+], leucocytes [CD45+], macrophages [CD68+], and non-immune stromal cells [CK-/CD45-CD68-/DNA+]). The photocleaved oligos were hybridized and digitally counted with the nCounter platform. Two cut-points (median and top tertile) were explored for each maker. All statistical testing was performed using a two-sided significance level of α=0.05 without correction for multiple hypothesis testing.

**Results**

160 protein variables were generated per case (normalized counts within molecularly defined compartments). In univariate analyses using pre-specified cut-points, 10 markers were associated with clinical benefit (CB) or non-CB, 6 markers with PFS, and 13 markers with OS. Of these, CD56 (top tertile) and CD4 (median) measured in the CD45 compartment were the only markers that significantly predicted either CB (OR 6.7, p = 0.014 and OR 8.5, p = 0.014, respectively) longer PFS (HR 0.38, p = 0.011 and HR 0.33, p = 0.002, respectively) and longer OS (HR 0.44, p = 0.044 and HR 0.31, p = 0.002, respectively). After adjusting for 3 baseline clinical prognostic factors (performance status, liver metastasis, dNLR) in a multivariate Cox proportional hazard model, both CD56 and CD4 remained predictive for PFS (HR 0.39, p = 0.020 and HR 0.37, p = 0.017, respectively), while only CD4 was predictive for OS (HR 0.28, p = 0.006).

**Conclusions**

This pilot scale, discovery study shows the potential of the DSP technology in the identification of spatially-informed biomarkers of response to PD-1 checkpoint blockade in NSCLC. This works highlights a previously undescribed role for CD56+ immune cells and CD4+ T-cells as potential predictors of immunotherapy outcomes in NSCLC.

**Ethics Approval**

All tissue samples were collected and used with specific consent or waiver of consent under the approval from the Yale Human Investigation Committee protocol #9505008219.

#### P127 Development of a 12-marker immunofluorescence multiplex panel for the in-depth investigation of the tumor immune landscape analyzing 4,096 phenotypes

##### Courtney Hauck, Aditi Sharma, Monique Johnson, Wenya Yang, Bonnie Phillips, PhD, Mark Burton, HTL ASCP, Douglas Wood, PhD, Stephanie Hennek, PhD, Mael Manesse, PhD, J Kent Moore, PhD, Katir Patel, PhD, Jamie Buell, Sean Downing, PhD

###### Ultivue, Cambridge, MA, United States

####### **Correspondence:** Sean Downing (sean.downing@ultivue.com)

**Background**

Current IHC methods limit the depth of information from a single tissue sample to a single target in the case of chromogenic staining, or to sample morphology and general cell identification in the case of H&E. Multiplex immunofluorescence (mIF) methods provide insights into a wide number of markers of interest and their spatial context in a single sample but limit the level of marker co-localization detection possible because of multiple antigen retrieval or photobleaching steps. Here, we demonstrate the utility of a new 12-plex mIF panel using InSituPlex technology that can identify thousands of phenotypes and spatial behavior through the co-localization of markers that was once limited to the domain of flow cytometry.

**Methods**

The 12-Plex marker panel was developed including: CD3, CD4, CD8, CD20, Granzyme B, CD56, CD68, CD163, FoxP3, PD-1, PD-L1, and pan-Cytokeratin/Sox10 used the InSituPlex and DNA-Exchange technology to perform mIF staining of FFPE samples from tonsil and tumor biopsies on the Leica Biosystems BOND RX autostainer. The tissues were then imaged in five distinct fluorescent channels (DAPI, FITC, TRITC, Cy5, Cy7) in 3 rounds of image acquisition on the ZEISS Axio Scan.Z1. HALO analysis software was used to identify cell phenotypes and spatial interactions across the whole slide images. UMAP and PSDM were also used to characterize cellular phenotypes and similarities amongst samples in the cohorts. Downstream H&E staining was performed on the same slides with a fourth imaging round to produce a fused 12-plex fluorescent and brightfield image.

**Results**

The multiplex panel was able identify all 12 markers in FFPE samples and immunophenotype single cells through co-expression of several biomarkers. Of the 4,096 possible phenotypes, the relevant phenotypes mapped included, but were not limited to: T cells, T-regs, Cytotoxic T-cells, Exhausted T-cells, B cells, NK cells, M1 and M2 macrophages, tumor cells, and expression along the PD-L1 and PD-1 immune checkpoint axis. Distance mapping and infiltration indexes were measured in tumor regions, stroma compartments, and along invasive margins.

**Conclusions**

In this work, we introduce a tumor and immune cell phenotyping multiplex immunofluorescence panel for the comprehensive characterization of the tumor microenvironment and its applicability across a range of carcinoma and melanoma FFPE tissue samples for support of deep pathology assessment in drug discovery research. The ability to colocalize markers in the same compartment for the identification of thousands of phenotypes when combined with brightfield pathological assessment within a single sample has the potential to accelerate immunotherapy research.

#### P128 Pooled analysis of Programmed Death Factor Ligand 1 (PD-L1) expression as a predictive biomarker using individual data on 7,918 randomized study patients

##### Andrea Arfe, Geoffrey Fell, Brian Alexander, MD MPH, Mark Awad, MD PhD, Scott Rodig, MD, PhD, Lorenzo Trippa, Jonathan Schoenfeld, MD, MPH

###### Dana-Farber Cancer Institute, Boston, MA, United States

####### **Correspondence:** Jonathan Schoenfeld (jdschoenfeld@partners.org)

**Background**

PD-L1 expression is one of the most studied biomarkers to predict the efficacy of immune checkpoint inhibitors (ICIs), but its clinical significance is controversial. Several factors have limited the study of PD-L1 expression. Most trials use of hazard ratios (HRs) to measure treatment effects on survival outcomes, a questionable practice for immunotherapy studies. Additionally, trials use different cut-off values to dichotomize PD-L1 scores, complicating meta-analyses. Therefore, we performed a pooled analysis to: i) estimate the distribution of PD-L1 expression scores in clinical populations, and ii) assess the relationship between PD-L1 levels and ICIs’ effects on overall survival (OS). Instead of HRs, we used a more robust metric, i.e. differences in restricted mean survival times (ΔRMSTs).

**Methods**

Following PRISMA guidelines, we analyzed individual-level data reconstructed from the publications of 14 randomized clinical trials of ICIs. We used an imputation-based approach to estimate i) the distribution of PD-L1 scores, ii) the survival distribution in different PD-L1 classes, and iii) pooled ΔRMST estimates. We show the advantage provided by meta-analytic estimates such as ours for the design of future studies in a simulation study. We simulated 10,000 NSCLC trials (1:1 randomization; sample size: 500 patients) that compared ICIs with standard chemotherapy. Simulated trials followed either i) a design that does not use prior information on the distribution of PD-L1 levels and their association with ICIs’ effects, or ii) a design tailored to our meta-analytic estimates.

**Results**

We reconstructed data on 7,918 individual patients, 3,496 with NSCLC, 4,529 with other tumors. The estimated distribution of PD-L1 expression is U-shaped, with most patients presenting a low or high expression: only about 7% had an expression in the 5%-50% range. ΔRMST estimates suggest that i) ICIs provide an OS benefit to all patients, and ii) the magnitude of OS benefits increases along with PD-L1 score, although changes in ΔRMSTs were greater in NSCLC (Figure 1). In the simulations, the power to detect a positive treatment effect increased from 80% to 93% using a design tailored to meta-analytic information.

**Conclusions**

By highlighting that higher PD-L1 scores predict increasing OS benefits, our findings extend those of recent meta-analyses that evaluated PD-L1 expression scores as predictors of ICIs’ efficacy. They also illustrate how meta-analytic estimates like ours can improve the power of future trials to detect ICIs’ benefits. Our findings also suggest that the practice of dichotomizing the range of PD-L1 expression scores is inadequate for patient stratification.

**Fig. 1 (abstract P128).**
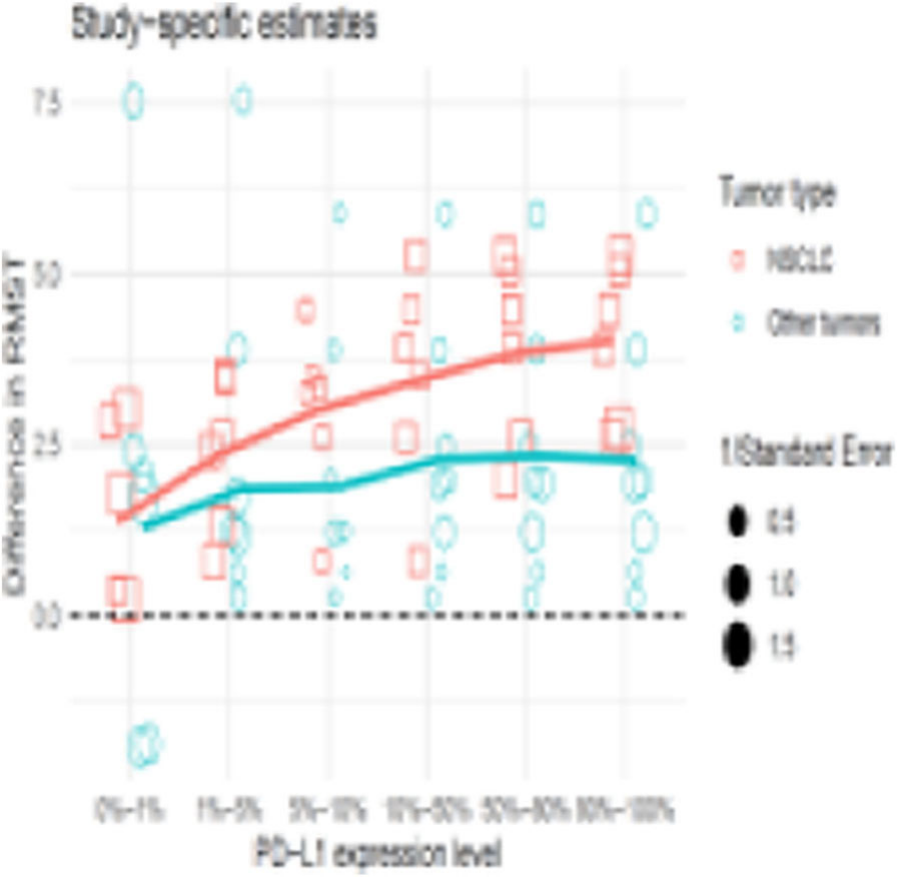
See text for description

#### P129 Correlation of circulating CD8 T cell activation with response to immunotherapy in advanced renal cell carcinoma

##### Jennifer Carlisle, MD, Caroline Jansen, BS, Adriana Reyes, Nataliya Prokhnevska, BS, Deborah Baumgarten, MD, Viraj Master, MD, PhD, R. Donald Harvey, PharmD, Bradley Carthon, MD, PhD, Omer Kucuk, MD, Mehmet Bilen, Haydn Kissick

###### Emory University, Atlanta, GA, United States

####### **Correspondence:** Jennifer Carlisle (jennifer.w.carlisle@emory.edu)

**Background**

Although immunotherapy with PD-1 or dual PD-1/CTLA-4 blockade can be a successful treatment for patients with advanced renal cell carcinoma (RCC), the majority do not respond [1, 2]. Prior peripheral blood immune profiling studies have shown that a transient rise in activated CD8 T cells correlates with clinical response to PD1 blockade in lung cancer [3, 4] and melanoma [5]. The effect of checkpoint blockade on circulating CD8 T cells in RCC is unknown, as is the T cell biology underlying clinical response.

**Methods**

Serial peripheral blood mononuclear cells were obtained from patients with RCC undergoing immunotherapy. Samples were obtained at baseline (cycle 1) and initiation of each subsequent cycle (up to cycle 6). Flow cytometry identified longitudinal changes in T cell subsets. Additionally, recently activated CD8 T cells, identified by surface expression of CD38 and HLA-DR, were sorted at baseline, post-cycle 1, and post-cycle 2, and analyzed by RNA seq. Clinical responses were determined at the first restaging scans using RECIST v1.1 criteria, to define those with clinical benefit (complete response, partial response, or stable disease) or no clinical benefit (progressive disease).

**Results**

Of 27 patients analyzed, 10 received nivolumab, 7 nivolumab + NKTR-214, and 10 nivolumab + ipilimumab. Median age was 58 years (range 33-78) with a male (70%), Caucasian (89%), and solely clear cell histology (83%) predominance. A burst in circulating activated CD8 T cells as defined by a ≥1.8 fold increase in CD38+HLA-DR+ CD8 T cells from baseline to post-cycle 1 (Figure 1A) was observed in 8/12 patients who had clinical benefit and 6/15 patients with no clinical benefit (Figure 1B). Transcriptional analysis revealed that in patients with the aforementioned immunological response, T-cells had upregulated TCR signaling, CD28 signaling, enhanced glycolysis and iron uptake, and reduced TGF-beta signaling compared to patients without an immunologic response.

**Conclusions**

Peripheral blood immune monitoring of RCC patients while on immunotherapy may provide an early predictor of response. One important limitation identified is the treatment specific cycle length defined sample collection timing and therefore may miss transient early immunologic changes. This study advances knowledge regarding the newly generated effector CD8 T cells that contain important information about the immunobiology underlying response to immunotherapy.

**Acknowledgements**

This work was supported by funding from the NCI grant 1-R00-CA197891 and Nektar Therapeutics. We would like to acknowledge The Yerkes NHP Genomics Core which is supported in part by NIH P51 OD011132, the Emory Flow Cytometry Core (EFCC) supported by the National Center for Georgia Clinical & Translational Science Alliance of the National Institutes of Health under Award Number UL1TR002378, and NIH/NCI under award number, 2P30CA138292-04.

**References**

1. Motzer RJ, Escudier B, McDermott DF, George S, Hammers HJ, Srinivas S, Tykodi SS, Sosman JA, Procopio G, Plimack ER, Castellano D, Choueiri TK, Gurney H, Donskov F, Bono P, Wagstaff J, Gauler TC, Ueda T, Tomita Y, Schutz FA, Kollmannsberger C, Larkin J, Ravaud A, Simon JS, Xu LA, Waxman IM, Sharma P, CheckMate I. Nivolumab versus Everolimus in Advanced Renal-Cell Carcinoma. N Engl J Med. 2015;373(19):1803-1813.

2. Motzer RJ, Tannir NM, McDermott DF, Aren Frontera O, Melichar B, Choueiri TK, Plimack ER, Barthelemy P, Porta C, George S, Powles T, Donskov F, Neiman V, Kollmannsberger CK, Salman P, Gurney H, Hawkins R, Ravaud A, Grimm MO, Bracarda S, Barrios CH, Tomita Y, Castellano D, Rini BI, Chen AC, Mekan S, McHenry MB, Wind-Rotolo M, Doan J, Sharma P, Hammers HJ, Escudier B, CheckMate I. Nivolumab plus Ipilimumab versus Sunitinib in Advanced Renal-Cell Carcinoma. N Engl J Med. 2018;378(14):1277-1290.

3. Kamphorst AO, Pillai RN, Yang S, Nasti TH, Akondy RS, Wieland A, Sica GL, Yu K, Koenig L, Patel NT, Behera M, Wu H, McCausland M, Chen Z, Zhang C, Khuri FR, Owonikoko TK, Ahmed R, Ramalingam SS. Proliferation of PD-1+ CD8 T cells in peripheral blood after PD-1-targeted therapy in lung cancer patients. Proc Natl Acad Sci U S A. 2017;114(19):4993-4998.

4. Kamphorst AO, Wieland A, Nasti T, Yang S, Zhang R, Barber DL, Konieczny BT, Daugherty CZ, Koenig L, Yu K, Sica GL, Sharpe AH, Freeman GJ, Blazar BR, Turka LA, Owonikoko TK, Pillai RN, Ramalingam SS, Araki K, Ahmed R. Rescue of exhausted CD8 T cells by PD-1-targeted therapies is CD28-dependent. Science. 2017;355(6332):1423-1427.

5. Huang AC, Postow MA, Orlowski RJ, Mick R, Bengsch B, Manne S, Xu W, Harmon S, Giles JR, Wenz B, Adamow M, Kuk D, Panageas KS, Carrera C, Wong P, Quagliarello F, Wubbenhorst B, D'Andrea K, Pauken KE, Herati RS, Staupe RP, Schenkel JM, McGettigan S, Kothari S, George SM, Vonderheide RH, Amaravadi RK, Karakousis GC, Schuchter LM, Xu X, Nathanson KL, Wolchok JD, Gangadhar TC, Wherry EJ. T-cell invigoration to tumour burden ratio associated with anti-PD-1 response. Nature. 2017;545(7652):60-65.

**Ethics Approval**

Samples are collected under an approved IRB protocol (The Urological Satellite Specimen Bank at Emory University, IRB00055316), and all patients provided informed consent.

**Fig. 1 (abstract P129).**
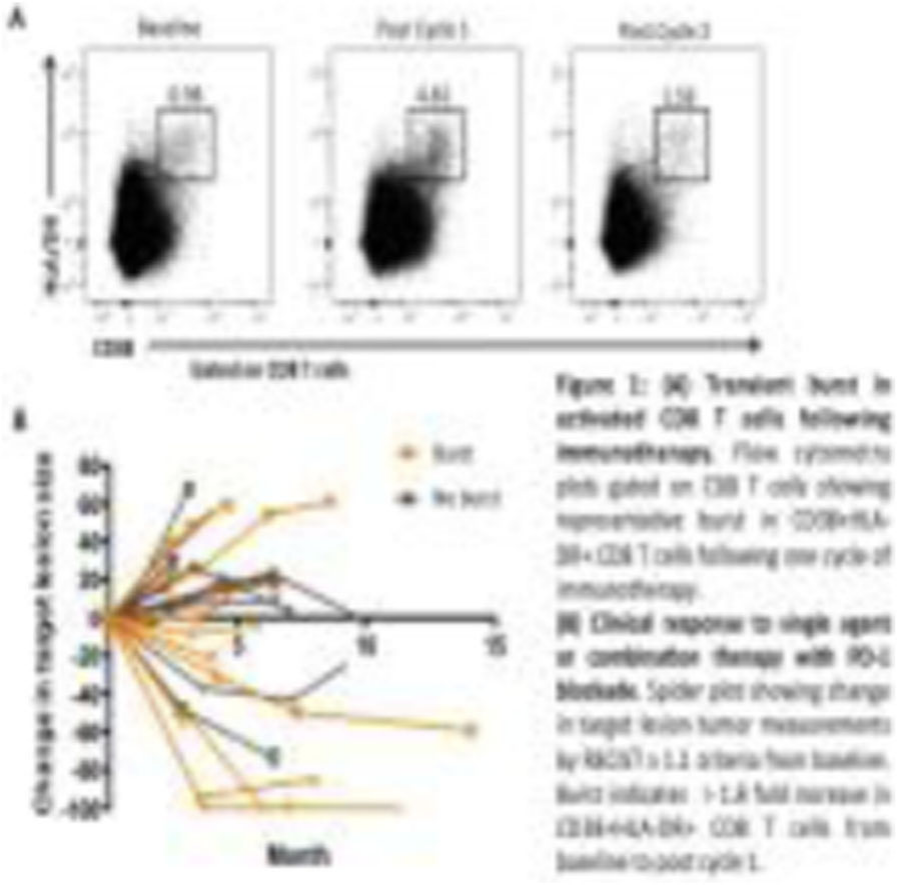
See text for description

#### P130 Melanoma patients harbor pre-existing IgG autoantibodies targeting neuronal proteins that associate with differential clinical outcomes following checkpoint blockade

##### Tyler Hulett, PhD^1^ , Keith Giles^2^, Michael Gowen, MD^2^, Danny Simpson^2^, Jeremy Tchack^2^, Una Moran^2^, Zarmeena Dawood^2^, Anna Pavlick, MD, MBA^2^, Shaohui Hu^1^, Hua Zhong^2^, Michelle Krogsgaard^2^, Tomas Kirchhoff, PhD^2^, Iman Osman^2^

###### ^1^CDI Laboratories, Portland, OR, United States; ^2^New York University School of Medicine, New York, NY, United States

####### **Correspondence:** Tyler Hulett (tyler.hulett@cdi-lab.com)

**Background**

Autoantibody landscapes are very specific to the individual, can remain stable for many years, and contain unique features reported in association with cancer, autoimmunity, infection, neurologic conditions, CD8+ T cell behavior, and checkpoint blockade adverse events [1–11].

The goal of this work was to determine whether pre-existing antigen-specific features in melanoma patient autoantibody landscapes would associate with clinical outcomes following checkpoint blockade.

**Methods**

Pre-treatment serum samples were collected from 117 melanoma patients prior to checkpoint blockade with anti-CTLA4 (N=60), anti-PD1 (N=38), or both in combination (N=16). All data was collected with approval of the NYU Institutional Review Board at the NYU Perlmutter Cancer Center with informed consent [11].

Serum samples were run on HuProt Human Proteome Microarrays containing >19,000 human proteins by CDI Laboratories. Raw serum IgG signal intensities were processed across staining cohorts via interquartile range normalization.

Pre-existing antibody responses were defined as patient-specific IgG signals >3.5 median absolute deviations above cohort median IgG background (modified Z-score). Group statistics were computed (GraphPad Prism), and gene ontology enrichment analysis was performed (Enrichr) [12].

**Results**

Several pre-existing antigen-specific IgG autoantibody targets were observed to have associations with good outcomes (SD/PR) or objective clinical responses (PR/CR) versus patients with progressive disease (POD). While final determination of the most predictive subsets is ongoing, many targets represent genes in an axis surrounding immune signaling pathways, hereditary neurodegenerative disease, and the ubiquitin proteasome pathway (ie, UBQLN1, UBQLN2).

An exemplary example was observed in the autoantibody responses shared by >10% of all patients regardless of clinical outcome. Gene ontology enrichment analysis of these shared melanoma-patient autoantibodies versus KEGG 2019 [12] demonstrates this set of proteins is strongly enriched for neurotrophin signaling-associated proteins after multi-sample correction (P=0.004) (Table 1). Several other associations were observed cohort-wide for ontologies with tissue-specific enrichment in the brain, neurons, and neuronal processes.

**Conclusions**

In this pilot study, we found strong associations across the cohort for autoantibodies against nerve-growth-inducing neurotrophins and genes like UBQLN1 and UBQLN2 which have strong associations with amyotrophic lateral sclerosis, frontotemporal dementia, Parkinson’s, and Alzheimer’s – neurodegenerative diseases that are known to have incidences which correlate with melanoma [14–16]; this hints at a potential immunologic connection between the conditions, perhaps related to an antitumor / autoimmune axis involving the targets reported here.

**Acknowledgements**

We thank the patients and their families who consented to participate in this study. Funding support for the study was provided by the NYU Cancer Center and NIH/NCI Cancer Center Support Grant P30CA016087, the Marc Jacobs campaign to support melanoma research, Goldberg Charitable Trust, Wings for Things Foundation and Clayman Family Foundation to I. Osman; the American Medical Association foundation, the Melanoma Research Foundation and the American Skin Association grants to M. Gowen.

**Trial Registration**

Patient samples included in this study were not part of a randomized controlled clinical trial.

**References**

1. Nagele EP, Han M, Acharya NK, DeMarshall C, Kosciuk MC, Nagele RG. Natural IgG Autoantibodies Are Abundant and Ubiquitous in Human Sera, and Their Number Is Influenced By Age, Gender, and Disease. Tsokos GC, editor. PLoS ONE. 2013;8:e60726.

2. Larman HB, Zhao Z, Laserson U, Li MZ, Ciccia A, Gakidis MAM, et al. Autoantigen discovery with a synthetic human peptidome. Nature Biotechnology. 2011;29:535–41.

3. Meyer S, Woodward M, Hertel C, Vlaicu P, Haque Y, Kärner J, et al. AIRE-Deficient Patients Harbor Unique High-Affinity Disease-Ameliorating Autoantibodies. Cell. 2016;166:582–95.

4. Graff JN, Puri S, Bifulco CB, Fox BA, Beer TM. Sustained Complete Response to CTLA-4 Blockade in a Patient with Metastatic, Castration-Resistant Prostate Cancer. Cancer Immunology Research. 2014;2:399–403.

5. Gnjatic S, Ritter E, Büchler MW, Giese NA, Brors B, Frei C, et al. Seromic profiling of ovarian and pancreatic cancer. Proceedings of the National Academy of Sciences. 2010;107:5088–5093.

6. Wongkulab P, Wipasa J, Chaiwarith R, Supparatpinyo K. Autoantibody to Interferon-gamma Associated with Adult-Onset Immunodeficiency in Non-HIV Individuals in Northern Thailand. Rottenberg ME, editor. PLoS ONE. 2013;8:e76371.

7. Anderson KS, Sibani S, Wallstrom G, Qiu J, Mendoza EA, Raphael J, et al. Protein Microarray Signature of Autoantibody Biomarkers for the Early Detection of Breast Cancer. Journal of Proteome Research. 2011;10:85–96.

8. Miersch S, Bian X, Wallstrom G, Sibani S, Logvinenko T, Wasserfall CH, et al. Serological autoantibody profiling of type 1 diabetes by protein arrays. Journal of Proteomics. 2013;94:486–96.

9. Srivastava RM, Lee SC, Andrade Filho PA, Lord CA, Jie H-B, Davidson HC, et al. Cetuximab-Activated Natural Killer and Dendritic Cells Collaborate to Trigger Tumor Antigen-Specific T-cell Immunity in Head and Neck Cancer Patients. Clinical Cancer Research. 2013;19:1858–72.

10. Hulett TW. Coordinated responses to individual tumor antigens by IgG antibody and CD8+ T cells following cancer vaccination. 2018;14.

11. Gowen MF, Giles KM, Simpson D, Tchack J, Zhou H, Moran U, et al. Baseline antibody profiles predict toxicity in melanoma patients treated with immune checkpoint inhibitors. Journal of Translational Medicine [Internet]. 2018 [cited 2018 Nov 4];16. Available from: https://translational-medicine.biomedcentral.com/articles/10.1186/s12967-018-1452-4

12. Chen EY, Tan CM, Kou Y, Duan Q, Wang Z, Meirelles GV, Clark NR, Ma'ayan A. Enrichr: interactive and collaborative HTML5 gene list enrichment analysis tool. BMC Bioinformatics. 2013;128(14).

13. Kuleshov MV, Jones MR, Rouillard AD, Fernandez NF, Duan Q, Wang Z, Koplev S, Jenkins SL, Jagodnik KM, Lachmann A, McDermott MG, Monteiro CD, Gundersen GW, Ma'ayan A. Enrichr: a comprehensive gene set enrichment analysis web server 2016 update. Nucleic Acids Research. 2016; gkw377 .

14. Olsen JH, Friis S, Frederiksen K. Malignant Melanoma and Other Types of Cancer Preceding Parkinson Disease: Epidemiology. 2006;17:582–7.

15. Freedman DM, Curtis RE, Daugherty SE, Goedert JJ, Kuncl RW, Tucker MA. The association between cancer and amyotrophic lateral sclerosis. Cancer Causes & Control. 2013;24:55–60.

16. Roe CM, Fitzpatrick AL, Xiong C, Sieh W, Kuller L, Miller JP, et al. Cancer linked to Alzheimer disease but not vascular dementia. Neurology. 2010;74:106–12.

**Ethics Approval**

All data collected for this study was collected with approval of the NYU Institutional Review Board at the NYU Perlmutter Cancer Center with informed consent.

**Consent**

No sensitive or patient identifiable information is included in the data presented.

**Table 1 (abstract P130).**
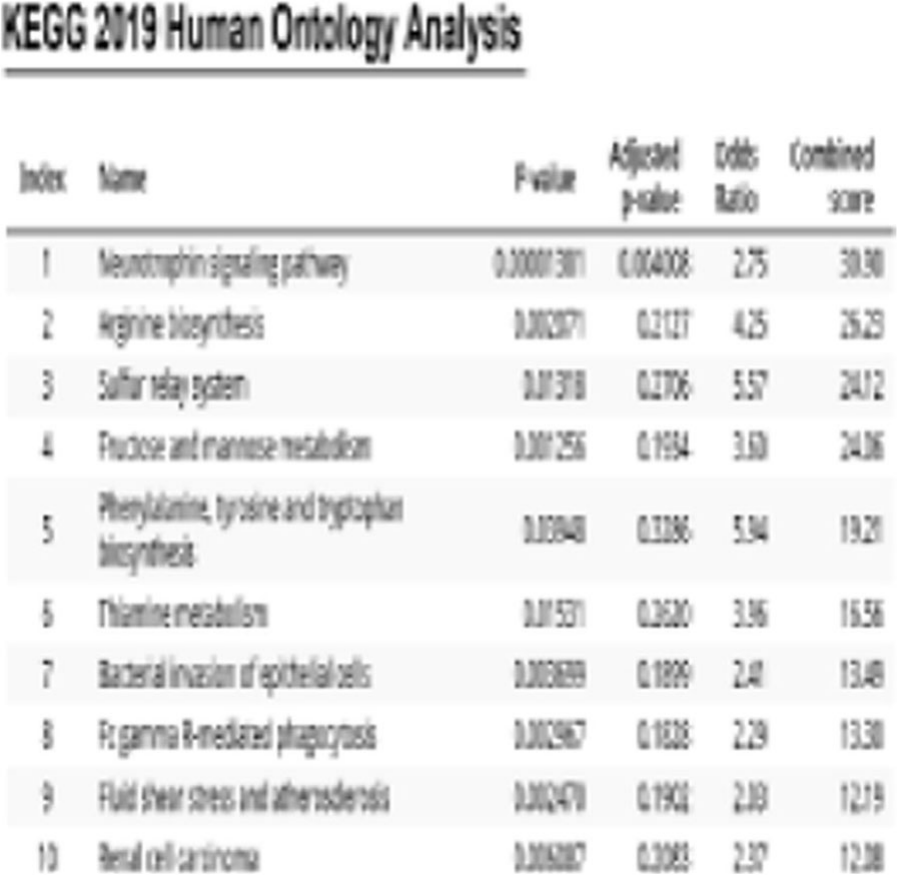
Enrichment of anti-neuronal growth autoantibodies

#### P131 Single cellular interrogation of tumor microenvironment enables diagnosis and prognostication of malignancies

##### Wei Jian Tan^1^, Jian Hang Lam^1^, Mona Meng Wang^2^, Paola Ricciardi-Castagnoli^1^, Anita Sook Yee Chan^2^, Tony Kiat Hon Lim^3^, Joe Poh Sheng Yeong^3^, Tong Seng Lim, PhD^1^

###### ^1^Menarini Biomarkers Singapore, Singapore, Singapore; ^2^Singapopre Eye Research Institute, Singapore, Singapore; ^3^Singapore General Hospital, Singapore, Singapore

####### **Correspondence:** Tong Seng Lim (tongseng.lim@mbiomarkers.com)

**Background**

Tumor microenvironment contains a diverse array of cell types with heterogeneous genomic and molecular profiles. Averaging the characteristics of all cells in a cancerous tissue no doubt obscures important variations in biomarkers among minority, but critical, pathogenic cell populations. High resolution, single-cell analyses are thus needed in the clinic to precisely delineate the inherent heterogeneity of tumor microenvironment underlying oncogenesis in each patient. Biopsies derived from tumor microenvironment are routinely used for medical diagnosis or prognostications. The number of cells typically available from a biopsy is limited and heterogeneous. The ability to distinguish, select, and sort rare malignant or pathogenic immune cells from either tissue or liquid biopsies poses a unique challenge for single-cell based diagnosis and prognostication. Heterogeneous cell populations with non-target immunoreactive cells in pausicellular biopsies complicate conventional bulk-cell analysis, and could lead to disease misdiagnosis or prognostication.

**Methods**

We adopted a state-of-the-art multi-modal strategy including the real-time imaging-based DEPArray with downstream molecular and genomic assays [1], and quantitative multiplex immunofluorescent technique [2] in order to predict clinical outcome or direct therapy, based on single-cell based diagnostic or prognostic biomarkers derived from tumor microenvironment.

**Results**

We provided proof-of concepts that DEPArray technology enabled automated isolation and recovery of rare malignant or pathogenic immune cells from liquid or tissue biopsies in several malignancies including vitreoretinal lymphoma (VRL), hepatocellular carcinoma (HCC) and colorectal carcinoma (CRC). Rare target B lymphoma cells were distinguished and sorted from pausicellular ocular vitreous biopsies with high resolution and purity required for sensitive single-cell based MYD88 mutational profiling to aid VRL diagnosis [1]. Single cellular imaging revealed the presence of large (>10μm), irregular shaped of a novel population of HCC-infiltrating macrophages in association with improved prognosis after surgery. A unique signature regulatory T-cells (Tregs) population was identified in both blood circulation and cancerous tissues of CRC. These signature Tregs expressed phenotypically distinct surface markers in association with better disease-free and overall survival of CRC patients.

**Conclusions**

Using real-time imaging-based, digital sorting DEPArray, we could distinguish, select and sort different types of malignant or target immune cells including B-cells, T-cells and macrophages from heterogeneous tumor microenvironment or liquid biopsies with low cellularity. Comprehensive genomic and molecular characterizations at single cell resolution revealed crucial biomarkers associated with clinicopathological features that impact clinical outcome of patients. The single cell interrogation using DEPArray technology provides a novel precision medicine tool for diagnostics and prognostications of malignancies in future.

**Acknowledgements**

This study was supported by research funding from the research collaboration between A. Menarini Biomarkers Singapore Pte Ltd, Singapore Eye Research Institute and Singapore General Hospital. Some data presented here are part of patent filed on 21 August 2018 (#10201807097T).

**References**

1. Tan, W.J., et al., Single-cell-MYD88 sequencing of isolated B cells from vitreous biopsies aids vitreoretinal lymphoma diagnosis. Blood, 2019.

2. Lim, J.C.T., et al., An automated staining protocol for seven-colour immunofluorescence of human tissue sections for diagnostic and prognostic use. Pathology, 2018. 50(3): p. 333-341.

**Ethics Approval**

This study was approved by the SingHealth Institutional Review Board in accordance with the Singapore Guidelines for Good Clinical Practice and the Declaration of Helsinki, approval number #2009/907/B, 2012/104/F and #2017/2494

#### P132 Harmony: Integrative tool to analyse and visualise multiplex-immunofluorescence single-cell data

##### Duoduo Wu^1^, Joe Yeong, MBBS, PhD^2^, Grace Tan^3^, Marion Chevrier^4^, Josh Loh^5^, Tony Lim^5^, Jinmiao Chen^4^

###### ^1^National University of Singapore, Singapore, Singapore; ^2^Department of Anatomical Pathology, Sing, Singapore, Singapore; ^3^Nanyang Technological University, Singapore, Singapore; ^4^Agency of Science, Technology and Resear, Singapore, Singapore; ^5^Singapore General Hospital, Singapore, Singapore, Singapore

####### **Correspondence:** Tony Lim (Lim.Kiat.Hon@singhealth.com.sg); Jinmiao Chen (Chen_Jinmiao@immunol.a-star.edu.sg)

**Background**

In the advent of immuno-technology, newer single-cell flow cytometry techniques have greatly increased the capacity for the maximum number of immunological parameters measured. Notably, multiplex-immunofluorescence (mIF) can perform measurements for 7 markers, flow cytometry can handle 20, and imaging mass cytometry can process up to 37 biomarkers simultaneously. Hence, dimensionality reduction techniques such as t-SNE and UMAP are becoming increasingly important for tumour single-cell data analysis. Using human hepatocellular carcinoma (HCC) tissue samples, we aim to compare and evaluate the use of a new technique, UMAP, as an alternative to t-SNE in mIF derived single-cell data.

**Methods**

We adopted an unsupervised clustering approach using FlowSOM to identify 8 major cell types present in human HCC tissues by staining them with 7 markers, including immune-checkpoint molecules and one nuclear counterstain. Following that, UMAP and t-SNE were ran independently on the dataset to qualitatively compare the distribution of clustered cell types in both dimensionality reduction tools.

**Results**

The key advantage of UMAP is its superior runtime – it takes approximately one-fifth the time required to run t-SNE. Both techniques provide similar arrangements of cell clusters, with the key difference being UMAP’s extensive characteristic branching. Also, increasing perplexity values in t-SNE results in a t-SNE visualisation with certain degrees of branching like that of UMAP’s, albeit limited. When parameters such as standard deviation, minimum and maximum intensity from the mIF image cytometry data were included, a t-SNE plot with virtually the same morphology as the resulting UMAP plot can be visualised. Most interestingly, UMAP’s branching highlighted biological lineages, especially in identifying potential hybrid tumour cells (HTC). Survival analysis shows patients with higher proportion of HTC have a worse prognosis (p-value = 0.019).

**Conclusions**

We conclude that both techniques are similar in their visualisation capabilities, but UMAP has a clear advantage over t-SNE in runtime, making it highly plausible to employ UMAP as an alternative to t-SNE in single-cell data analysis.

#### P133 High tumor expression of DKK1 is associated with improved clinical benefit and longer progression free survival across multiple solid tumors when treated with a targeted anti-DKK1 antibody (DKN-01)

##### Michael Kagey, PhD, Girish Naik, MD, Michael Haas, PhD, Heidi Heath, Franziska Schurpf-Huber, Walter Newman, PhD, Cynthia Sirard, MD

###### Leap Therapeutics, Cambridge, MA, United States

####### **Correspondence:** Cynthia Sirard (csirard@leaptx.com)

**Background**

Dickkopf-1 (DKK1), a secreted modulator of Wnt signaling, contributes to an immune suppressive tumor microenvironment and promotes tumor growth, angiogenesis and metastasis. DKN-01, a DKK1 neutralizing antibody, has demonstrated clinical activity across multiple solid tumors as both a monotherapy and in combination with checkpoint inhibitors and chemotherapies. Nonclinical studies indicate that DKN-01 efficacy depends on a functioning immune system, notably natural killer (NK) cells. High tumor expression of DKK1 correlates with a worse clinical prognosis in many solid tumors. As such, we evaluated tumor levels of DKK1 and association with clinical outcomes for DKN-01 based therapies.

**Methods**

DKK1 mRNA expression in patient tumor biopsies was evaluated with a RNAscope in situ hybridization assay. Expression levels were semi-quantified with QuPath or manually scored. Data was pooled from three separate clinical trials, DKN-01 as monotherapy or in combination with paclitaxel or pembrolizumab in esophagogastric cancer (EGC) (NCT02013154), DKN-01 as monotherapy or in combination with paclitaxel in epithelial endometrial cancer (EEC) or epithelial ovarian cancer (EOC) (NCT03395080) and DKN-01 in combination with gemcitabine/cisplatin in biliary tract cancer (BTC) (NCT02375880). Survival analysis was performed by the Kaplan-Meier method and multi-variable Cox proportional-hazards and logistic regression models were used to study the association of DKK1 H-score cutoffs (tertiles and quartiles) with survival and clinical benefit (CR, PR or SD per RECIST v1.1) outcomes.

**Results**

A total of 120 patients (59 EGC, 28 EEC, 20 EOC and 13 BTC) had DKK1 tumor expression with response and survival outcomes. Patients who had an H-score ≥ upper-quartile (≥50) of DKK1 expression versus < upper-quartile had a higher-odds of having clinical benefit/response with an adjusted OR of 4.46 (95% CI: 1.78, 11.71) and a longer PFS with an adjusted HR of 0.49 (95% CI: 0.29, 0.81). Patients who had an H-score ≥ upper-tertile (≥35) versus < upper-tertile had a higher-odds of having clinical benefit/response with an adjusted OR of 2.82 (95% CI: 1.21, 6.67) and a longer PFS with an adjusted HR of 0.53 (95% CI: 0.33, 0.85).

**Conclusions**

Elevated DKK1 tumor expression was associated with a higher clinical benefit/response rate and longer PFS for DKN-01 based treatments across multiple solid cancers. Tumor expression of DKK1 may represent an important patient selection criterion for further development of DKN-01 based therapies in solid cancers. The contribution of high levels of tumor DKK1 expression to an immune suppressive tumor microenvironment and the role of NK cells is currently under investigation.

**Ethics Approval**

Studies were approved by the Institutional Review Boards of each participating institution.

#### P134 Prognostic value of tumor microenvironment based on PD-L1 expression and CD8+ TILs density in locally advanced NSCLC treated with concurrent chemoradiotherapy

##### Lukas Käsmann^2^, Kathrin Gennen^1^, Julian Taugner^1^, Chukwuka Eze^1^, Monika Karin^1^, Olarn Roengvoraphoj^1^, Jens Neumann^1^, Amanda Tufman^3^, Michael Orth^1^, Simone Reu^1^, Claus Belka^1^

###### ^1^University Hospital, Munich, Germany; ^2^University of Munich, Munich, Germany; ^3^Thoracic Oncology Centre Munich, Munich, Germany

####### **Correspondence:** Lukas Käsmann (lkaesmann@gmail.com)

**Background**

The prognostic role of the tumor immunity microenvironment (TIME) in multimodal treatment for locally advanced non-small cell lung cancer (LA-NSCLC) is unclear. Increasing evidence suggests treatment benefit depending on tumor cell PD-L1 expression. The purpose of this retrospective single-center study was to investigate the prognostic value of PD-L1 expression on tumor cells in combination with CD8+ tumor stroma-infiltrating lymphocytes (TILs) density in inoperable LA-NSCLC treated with concurrent chemoradiotherapy (CRT).

**Methods**

We collected retrospectively clinical characteristics and initial tumor biopsy samples of 31 inoperable LA-NSCLC patients treated with concurrent CRT. PD-L1 expression on tumor cells (0% versus ≥1%), CD8+ TILs density (0-40% vs. 41-100%) and TIME according to classification by Zhang et al. were evaluated for potential prognostic value in terms of local control, progression-free (PFS) and overall survival (OS) as well as correlations with clinic-pathological features investigated.

**Results**

Median OS was 14 months (range: 3-167 months). The OS rates at 1- and 2 years were 68% and 20%. Local control rates for the entire cohort at 1 and 2 years were 74% and 61%, respectively. Median PFS and PFS at 1 and 2 years were 13±1.4 months, 58% and 19%. PD-L1 expression <1% on tumor cells was associated with improved OS, PFS and local control in patients treated with concurrent CRT. Univariate analysis showed a trend for improved OS and local control in patients with low CD8+ TILs density. Evaluation of TIME appears to be an independent prognostic factor for local control, PFS and OS. The longest and shortest OS were achieved in patients with type I (PD-L1neg/CD8low) and type IV (PD-L1pos/CD8low) tumors (median OS: 57±37 vs. 10±5 months, p=0.05), respectively.

**Conclusions**

Assessment of the tumor immunity microenvironment (TIME) by PD-L1 expression on tumor cells and CD8+ TILs density is a predictive biomarker in patients treated with concurrent CRT for inoperable LA-NSCLC.

**Acknowledgements**

The study was funded by the German Center for Lung Research (DZL).

**Ethics Approval**

The study was approved by the University Ethics Board, approval number 493-16.

**Consent**

Written informed consent was obtained from the patient for publication of this abstract and any accompanying images. A copy of the written consent is available for review by the Editor of this journal.

#### P135 Studying Loss of Y chromosome in colorectal and prostate cancers in males for non-invasive cancer biomarkers

##### Ambreen Asim, PhD, Sarita Agarwal, Rakesh Kapoor, Neeraj Rastogi

###### Sanjay Gandhi Postgraduate Institute of Medical Sciences, Lucknow, India

####### **Correspondence:** Sarita Agarwal (saritasgpgi@gmail.com)

**Background**

Loss of Y chromosome (LOY) is a well know established phenomenon associated with cancers and ageing. Recently, LOY in peripheral blood cells was suggested as a possible biomarker for different cancers in males. On the basis of previous findings, the present case-control study was conducted to evaluate the association of LOY in peripheral blood cells in prostate (PC) and colorectal cancers (CRC) in males [1-4].

**Methods**

30 CRC patients (mean age = 44.03±10.8), 36 PC patients (mean age = 60.8 ± 15.8 yrs) and 36 healthy control male cases (mean age = 54.6± 15.1 years) were recruited. DNA was extracted by using a standard phenol-chloroform method. Multiplex quantitative fluorescent (QF) PCR was used to co-amplify the homologous sequences present on the Y chromosome and other chromosome followed by their analysis on the genetic analyzer (ABI 3500) and finally the Y/X ratio was calculated on the basis of the peak height obtained from the electropherogram.

**Results**

The mean Y/X ratio was significantly lower in the whole group of cancer patients (0.709±0.02; p <0.0001) when compared to the controls (0.92±0.044). Also, the Y/X ratio when calculated separately was found to be lower in CRC (0.701±0.078; p <0.0001) and PC (0.717±0.044; p <0.0001) cases, when compared to controls (0.92±0.044). Multivariate logistic regression was performed by matching cancer and control subjects with age and the results suggest that LOY is not influenced by their age.

**Conclusions**

The results support the significant association of LOY in peripheral blood cells carcinogenesis in males. LOY can also serve as a non-invasive cancer biomarker to improve the early diagnosis and management of cancer patients in males.

**Acknowledgements**

We are highly grateful to Sanjay Gandhi Post Graduate Institute of Medical Sciences, Lucknow, Uttar Pradesh, India for providing the infrastructure and lab facilities for research work. The authors also thank all the consultant and residents of SGPGIMS, who helped in carrying out the study. Dr Ambreen Asim is the first author, who collected data, carried out all the practical work and drafted this abstract. Prof. Sarita Agarwal is the corresponding and second author, who helped in finalizing, correcting and critical review of the work. Prof.Rakesh Kapoor and Prof. Neeraj Rastogi are the oncologist consultant who has provided prostate and colorectal cancer patients blood samples after taking informed consent for this study.

**References**

1. Noveski P, Madjunkova S, Stefanovska E. Loss of Y Chromosome in Peripheral Blood of Colorectal and Prostate Cancer Patients. Plos One. 2016;11(1).

2. Chang Y M, Perumal R, Keat P Y, Rita Y.Y. Yong, Daniel L.C. Kuehn, Leigh Burgoyne. A distinct Y-STR haplotype for Amelogenin negative males characterized by a large Yp11.2 (DYS458-MSY1-AMEL-Y) deletion. Forensic Sci Int. 2007; 166(2-3):115-20.

3. Donaghue C, Mann K, Docherty Z and Ogilvie C M. Detection of mosaicism for primary trisomies in prenatal samples by QF-PCR and karyotype analysis.Prenat Diagn .2005; 25: 65–72.

4. Plaseski T, Noveski P, Trivodalieva S, Georgi D. Quantitative Fluorescent-PCR Detection of Sex Chromosome Aneuploidies and AZF Deletions /Duplications. Genetic Teasting, 2008 : 12: 4.

**Ethics Approval**

This study was approved by Sanjay Gandhi Post Graduate Institute of Medical Sciences Ethics Board; approval number IEC CODE – 2018- 53-IMP-103 dated 18th June 2018.”

**Consent**

Written informed consent was obtained from the patient for publication of this abstract and any accompanying images. A copy of the written consent is available for review by the Editor of this journal.

#### P136 Quantitative/spatial analysis of Tregs reveal a prominent biomarker role in human non-small cell lung cancer (NSCLC)

##### Richa Gupta^1^, Nicolas Rodriguez-Arriagada^1^, Shruti Desai, PhD^1^, Konstantinos Syrigos^2^, Roy Herbst, MD, PhD^1^, Vamsidhar Velcheti, MD FACP^3^, David Rimm, MD, PhD^1^, Sarah Goldberg, MD, MPH^1^, Kurt Schalper, MD, PhD^1^

###### ^1^Yale University, Burlington, CT, United States; ^2^Athens University, Athens, Greece; ^3^NYU-Langone Medical Center, Pepper Pike, OH, United States

####### **Correspondence:** Kurt Schalper (kurt.schalper@yale.edu)

**Background**

Regulatory T cells (Tregs) mediate potent tolerogenic signals, are involved in adaptive anti-tumor immune responses and T-cell reinvigoration using immune checkpoint blockers. Despite their prominent immune suppressive role, the tissue distribution and contribution of Tregs to clinical outcomes in human lung cancer is not well understood.

**Methods**

The levels and tissue distribution of Tregs and major tumor infiltrating lymphocyte (TIL) subsets were measured using simultaneous detection of FOXP3, CD4, CD8, pancytokeratin and DAPI by multiplexed quantitative immunofluorescence in 619 formalin-fixed paraffin embedded (FFPE) NSCLCs from 4 independent cohorts represented in tissue microarrays (cohort #1 [Yale, n=210], cohort #2 [Greece, n=192]; cohort #3] [80 immunotherapy-treated NSCLCs]; cohort #4 [Yale, n=137, adenocarcinomas with mutation testing). Markers were measured in different tissue compartments and cell phenotypes were used for individual cell counts and machine-learning-based spatial analysis. We studied the association between T-cell populations, tissue distribution, clinicopathologic/molecular characteristics and outcomes.

**Results**

Tregs (DAPI+/CD4+/FOXP3+ cells) were predominantly located in the stromal compartment and represented 3-10% of the total T-cell population. The level of Tregs was positively associated with higher CD8+ T-cell infiltration across the cohorts. There was no consistent association between Treg levels and patient age, gender, smoking status, clinical stage or tumor histology. However, Tregs were significantly higher in KRAS mutated lung adenocarcinomas than in EGFR mutant or KRAS/EGFR wild-type cases. As a single marker, the level of Tregs was not significantly associated with survival. However, the Treg to CD8 signal ratio was associated with shorter 5-year overall survival across the cohorts. Reduced survival was also seen in cases with a higher 5-nearest neighbor (5NN) mean distance between CD4+/Tregs and CD8+/CD4+ cells. Notably, the survival effect of the Treg-associated metrics was numerically higher in patients treated with immune checkpoint blockers.

**Conclusions**

Tregs are prominently less abundant than other TIL subsets in NSCLC microenvironments and they are increased in T-cell inflamed tumors. Their positive association with CD8+ cytotoxic TILs suggests their upregulation upon adaptive anti-tumor immune pressure and could explain the inconsistent reported relationship between Tregs and prognosis. Elevated Treg to CD8 signal ratio and reduced spatial clustering between CD4-Tregs and CD8-CD4 are indicative of poor outcome preferentially in NSCLC patients treated with checkpoint blockade suggesting a biomarker role.

**Ethics Approval**

All tissues were used after approval from the Yale Human Investigation committee protocol #9505008219 which approved patient consent forms or waivers of consent.

#### P137 Development and high specification validation of two recombinant rabbit monoclonal antibodies to accurately detect human PD-L2 expression in FFPE tissue sections by immunohistochemistry

##### Simon Renshaw, Will Howat, PhD, Subham Basu, PhD

###### Abcam, Cambridge, United Kingdom

####### **Correspondence:** Subham Basu (Subham.Basu@abcam.com)

**Background**

PD-L1 protein expression by immunohistochemistry (IHC) measurement is the only FDA-approved protein diagnostic biomarker for PD1/PD-L1 immunotherapies [1]. However, the tumor-immune interaction is complex: PD-L1 expression alone is not predictive of patient response [2]. This has led to the investigation of other PD-1 ligands such as PD-L2 [2]. PD-L2 even in the absence of PD-L1 has been associated with clinical response to PD-1 blockade in multiple tumor types [2]. PD-L2 status has also been investigated where immunotherapy based on PD-L1 has been less successful, such as prostate cancer, where PD-L1 expression is typically low [3]. Here, significantly higher levels of PD-L2 were associated with multiple survival and response measures [3]. Due to the diagnostic and therapeutic potential indicated by the presence of this key immune checkpoint ligand in patients irrespective of PD-L1 expression [2-4], dependable detection tools for investigating the presence and role of PD-L2 are crucial. To address this need, Abcam have developed and extensively characterized and validated a recombinant rabbit monoclonal antibody specific to PD-L2 (CAL28). For checkpoint inhibitors, Abcam already has research use only versions of three anti-PD-L1 RabMAb® antibodies employed in the clinical setting (73-10, 28-8 and SP142), co-developed with pharmaceutical and diagnostic companies.

**Methods**

A recombinant rabbit monoclonal antibody was generated using a direct B cell cloning process and characterized for IHC. The clone was tested using PD-L2-transfected and non-transfected HEK293 cells fixed in formaldehyde and processed into paraffin wax (FFPE) and further validated alongside In Situ Hybridization (ISH) for PD-L2 mRNA in FFPE commercial cell lines. Once specificity was determined, it was tested in positive and negative tissues and TMAs of Head & Neck Squamous Cell Carcinoma (HNSCC), Prostate Carcinoma (PC) and Renal Cell Carcinoma (RCC).

**Results**

CAL28 demonstrated positive IHC staining on PD-L2-overexpressed HEK293 cells processed in FFPE with a lack of staining in the parental line. Additionally, CAL28 demonstrated IHC staining in FFPE cell lines where PD-L2 expression was confirmed with ISH for PD-L2 mRNA. Expression in tumor tissue in TMAs from HNSCC, PC and RCC was evaluated with no non-specific background staining.

**Conclusions**

We have demonstrated sensitivity, specificity and reproducibility of a recombinant rabbit monoclonal antibody to PD-L2 in IHC (CAL28). The global, commercial availability of this recombinant clone to researchers, pathologists, clinicians and the biopharmaceutical industry will enable further progress to be made in understanding the clinical relevance and predictive value that PD-L2 promises for cancer immunotherapy.

**References**

1. Tsao MT, Kerr K, Yatabe Y, et al. PL 03.03 Blueprint 2: PD-L1 Immunohistochemistry Comparability Study in Real-Life, Clinical Samples. J Thor Oncol. 2017;12(Suppl 2):S1606

2. Yearley JH, Gibson C, Yu N, Moon C, Murphy E, Juco J, Lunceford J, Cheng J, Chow LQM, Seiwert TY, Handa M, Tomassini JE, McClanahan T. Clin Cancer Res. 2017;23:3158-3167

3. Zhao SG, Lehrer J, Chang SL, Das R, Erho N, Liu Y, Sjöström M, Den RB, Freedland SJ, Klein EA, Karnes RJ, Schaeffer EM, Xu M, Speers C, Nguyen PL, Ross AE, Chan JM, Cooperberg MR, Carroll PR, Davicioni E, Fong L, Spratt DE, Feng FY.The Immune Landscape of Prostate Cancer and Nomination of PD-L2 as a Potential Therapeutic Target. J Natl Cancer Inst. 2019;111:301-310

4. Takamori S, Takada K, Toyokawa G, Azuma K, Shimokawa M, Jogo T, Yamada Y, Hirai F, Tagawa T, Kawahara A, Akiba J, Okamoto I, Nakanishi Y, Oda Y, Hoshino T, Maehara Y. PD-L2 Expression as a Potential Predictive Biomarker for the Response to Anti-PD-1 Drugs in Patients with Non-small Cell Lung Cancer. Anticancer Res. 2018;38:5897-5901

#### P138 Review of evidence for predictive value of microsatellite instability/mismatch repair status in response to non-anti-PD-(L)1 therapies in patients with advanced or recurrent endometrial cancer

##### Cara Mathews, MD^1^, Ellie Im^2^, Liliana Alfaya^2^, Karin Travers^2^, Craig Gibson^2^

###### ^1^Women and Infants Hospital, Providence, RI, United States; ^2^TESARO: A GSK Company, Waltham, MA, United States

####### **Correspondence:** Cara Mathews(cmathews@wihri.org)

**Background**

Multiple immunotherapies have been evaluated in patients with advanced or recurrent endometrial cancer (EC) using molecular biomarkers, including microsatellite instability-high (MSI-H) and stable (MSS) status. Clinical outcomes appear to be different in patients with MSI-H/mismatch repair (MMR)-deficient status versus MSS/MMR-proficient status when receiving anti-programmed cell death (ligand) 1 (PD-[L]1) therapies [1,2]. It is unclear if these differences are due to the therapies themselves or to differences inherent to the patient populations.

We sought to evaluate the association between MSI-H/deficient MMR (dMMR) status and response among patients with advanced or recurrent EC.

**Methods**

We conducted a systematic review of the Embase, MEDLINE, and Cochrane Central Register of Controlled Trials databases from 2000 to present to identify publications (manuscripts and conference proceedings) on studies using chemotherapy, surgery, radiotherapy, hormonal therapy, or biological therapy (or any combination thereof) in adult patients (≥18 years) with stage III or IV advanced or recurrent EC, and where MMR or MSI status was identified (by any means). To better understand the prognostic value of MSI-H/MSS status, we excluded anti-PD-(L)1 therapies from the analysis, as recent evidence suggests that there is a positive predictive value for these agents in patients with MSI-H/dMMR status [2-4].

**Results**

Our systematic review of MSI/MMR status and recurrence-free survival (RFS), progression-free survival (PFS), and overall survival (OS) identified a total of 5 studies. One study reported dMMR status was associated with a reduction in RFS (hazard ratio, 2.02) [5], while another study found no significant effect [6]. A third study reported a trend towards a higher rate of recurrence among patients with advanced-stage EC with dMMR than among patients with MMR proficiency (P value not reported) [7]. Two studies reported no significant association between PFS and dMMR status [8,9]. Three studies found no statistically significant association between OS and dMMR status [5,6,8].

**Conclusions**

This review could not identify a consistent association between dMMR or MSI-H status and recurrence, RFS, PFS, or OS among patients with advanced or recurrent EC receiving therapy other than anti-PD-(L)1. For RFS, where differences were present, they trended towards worse outcomes for patients with MSI-H/dMMR status. Consequently, we have identified no evidence of a prognostic or predictive value of MSI-H or dMMR biomarker status for efficacy outcomes in patients with advanced or recurrent EC receiving non–anti-PD-(L)1 therapy. Further investigation into the prognostic or predictive value of MSI-H/dMMR status is warranted.

**Acknowledgements**

Clinical Trial Registration: N/A

**References**

1. Segal NH, Wainberg ZA, Overman MJ, et al. Safety and clinical activity of durvalumab monotherapy in patients with microsatellite instability–high (MSI-H) tumors [abstract]. J Clin Oncol. 2019; 37(Suppl 4):670.

2. Konstantinopoulos PA, Liu JF, Luo W, et al. Phase 2, two-group, two-stage study of avelumab in patients (pts) with microsatellite stable (MSS), microsatellite instable (MSI), and polymerase epsilon (POLE) mutated recurrent/persistent endometrial cancer (EC) [abstract]. J Clin Oncol. 2019; 37(Suppl 15):5502.

3. Oaknin A, Duska LR, Sullivan RJ, et al. Preliminary safety, efficacy, and pharmacokinetic/pharmacodynamic characterization from GARNET, a phase I/II clinical trial of the anti–PD-1 monoclonal antibody, TSR-042, in patients with recurrent or advanced MSI-H and MSS endometrial cancer. Gynecol Oncol. 2019; 154:17.

4. Antill YC, Kok PS, Robledo K, et al. Activity of durvalumab in advanced endometrial cancer (AEC) according to mismatch repair (MMR) status: The phase II PHAEDRA trial (ANZGOG1601). J Clin Oncol 37, 2019 (suppl; abstr 5501).

5. Djordjevic B, Bruegl A, Fellman B, et al. The prognostic effect of MLH1 loss in endometrial endometrioid adenocarcinoma. Lab Invest. 2014; 94:280A–281A.

6. Cohen JG, Goodman MT, Karlan BY, Walsh C. Genomic characterization of grade 3 endometrial carcinoma. Gynecol Oncol. 2014; 133:134–135.

7. Cosgrove CM, Cohn DE, Hampel H, et al. Epigenetic silencing of MLH1 in endometrial cancers is associated with larger tumor volume, increased rate of lymph node positivity and reduced recurrence-free survival. Gynecol Oncol. 2017; 146(3):588–595. doi:10.1016/j.ygyno.2017.07.003.

8. Kim SR, Pina A, Albert A, et al. Does MMR status in endometrial cancer influence response to adjuvant therapy? Gynecol Oncol. 2018; 151:76–81.

9. Aghajanian C, Filiaci V, Dizon DS, et al. A phase II study of frontline paclitaxel/carboplatin/bevacizumab, paclitaxel/carboplatin/temsirolimus, or ixabepilone/carboplatin/bevacizumab in advanced/recurrent endometrial cancer. Gynecol Oncol. 2018; 150:274–281.

#### P139 Expression of GITR and GITR-L by head and neck squamous cell cancer

##### Rachna Moudgil, MS^1^, Christopher Paustian, PhD^2^, Carmen Ballesteros-Merino, PhD^1^, Shawn Jensen, PhD^1^, Hong-Ming Hu, PhD^1^, Walter Urba, MD, PhD^1^, Carlo Bifulco, MD^1^, Marcus Couey, MD, DDS^1^, Traci Hilton, PhD^2^, Bernard Fox, PhD^1^, Rom Leidner, MD^1^, R. Bryan Bell, DDS, MD^1^

###### ^1^Earle A. Chiles Research Institute, Portland, OR, United States; ^2^UbiVac, Portland, OR, United States

####### **Correspondence:** Bernard Fox(foxb@foxlab.org)

**Background**

Head and neck squamous cell cancer (HNSCC) ranks as the 6th most common cancer afflicting humans and remains a significant unmet medical need. While interfering with the PD-1/PD-L1 axis improves outcomes, the majority of patients progress and die of their disease. To address this lack of efficacy our group has explored the immune makeup of HNSCC, hypothesizing that a better characterization of responders and non-responders will result in improved predictive biomarkers and insights into strategies to improve outcomes for the majority of patients.

**Methods**

Over the past 7 years we have collected and processed more than 350 HNSCC specimens. When sufficient tumor material was available, tumor-infiltrating lymphocytes (TIL) and primary tumor cultures were initiated and characterized for autologous tumor reactivity. Once established, tumor cell lines were characterized for phenotypic markers by flow cytometry. Flow cytometric analysis and RNASeq has been performed on some established cell lines and on FFPE tumor specimens.

**Results**

Consistent with previous reports increased expression of CD8 T cells was associated with improved outcome. In preliminary studies increased expression of GITR was also associated with improved outcome. Initial speculation was that GITR expression was coming from immune infiltrates. Subsequently a report suggested that GITR could be expressed by HNSCC. Using flow cytometry we detected low level GITR expression on 3 HNSCC cell lines and low to high level expression of GITR-L on a 8 HNSCC cell lines. Studies are continuing to expand on these preliminary observations.

**Conclusions**

Anti-GITR and GITR-L both have the potential to provide positive signals to immune cells. In addition to APC’s, GITR-L expression by some HNSCC cells may contribute to the make-up of the immune cells infiltrating these cancers.

**Acknowledgements**

Funding Support: The Harder Family, Robert and Elsie Franz, Wes and Nancy Lematta, Lynn and Jack Loacker, the Providence Portland Medical Foundation and the Oral and Maxillofacial Surgery Foundation, The Murdock Trust.

**Ethics Approval**

The study was approved by the institutional review board of the Providence Portland Medical Center (12-075A).

#### P140 Myeloid cell contexture and IL-8 expression as a candidate immunotherapy target in non-small cell lung cancer (NSCLC)

##### Venkata Vamsi Nagineni, MD^1^, Kurt Schalper, MD, PhD^1^, Shruti Desai, PhD^1^, Ignacio Melero, MD^2^, Miguel Sanmamed, MD, PhD^2^, Richa Gupta^1^, Roy Herbst, MD, PhD^1^, Venkata Vamsi Nagineni, MD^1^

###### ^1^Yale University, Waukegan, IL, United States; ^2^University of Navarra, Pamplona, Spain

####### **Correspondence:** Kurt Schalper (kurt.schalper@yale.edu)

**Background**

Interleukin-8 (IL-8) is a chemokine expressed in multiple cancer types, including NSCLC. It exerts various functions in shaping cancer vascularization, cell dedifferentiation and inflammation/immunity. IL-8 was described as a chemotactic factor for neutrophils and it has been proposed to mediate recruitment of tolerogenic myeloid cells favoring a pro-tumorigenic microenvironment. Although clinical trials targeting IL-8 are ongoing, its expression and role in NSCLC is unclear.

**Methods**

We developed a multiplexed quantitative immunofluorescence (QIF) panel for simultaneous and localized measurement of IL-8, myeloperoxidase (MPO), CD15, cytokeratin (CK) and DAPI. We analyzed the expression of these markers and their association with PD-L1, CD4 and CD8-positive cells in 3 retrospective NSCLC immunotherapy-naive cohorts represented in tissue microarrays (cohort #1, n=262; #2, n=145; and #3, n=132); 1 cohort of NSCLC patients treated with immune checkpoint blockers (#4, n=59) and 1 collection of lung adenocarcinomas (LAC) analyzed for activating mutations in EGFR and KRAS (#5, n=121). We studied the level of the targets, their distribution and association with immune features, clinicopathological variables and survival.

**Results**

IL-8 protein signal was detected in ~85% of cases with cytoplasmic staining pattern and was higher in tumor than in stromal cells. Elevated tumor IL-8 was consistently associated with higher MPO+ neutrophils and CD15+ tumor-associated myeloid cells across the cohorts, but not with CD4+ and CD8+ T-cells. Increased IL-8 expression was not associated with major clinicopathologic variables. Elevated MPO+ and CD15+ cells was significantly higher in KRAS mutated than in EGFR mutated LACs. High MPO and CD15 signal was associated with shorter 5-year overall survival in all NSCLC cohorts. The negative prognostic effect of MPO and CD15 was comparable in both immunotherapy-naïve and immunotherapy-treated NSCLC collections.

**Conclusions**

IL-8 protein is frequently expressed in NSCLCs associated with increased tumor-associated myeloid cells but independent from intratumor T-cell responses. KRAS mutated LACs have prominent MPO+/CD15+ expression, supporting an immune suppressive role of myeloid cells in these malignancies. CD15 and MPO are prognostic markers in NSCLC and IL-8 blockade could mediate favorable immunomodulatory effects.

**Ethics Approval**

All tissues were used after approval from Yale Human Investigation committee protocol #9505008219 which approved the patient consent forms or waivers of consent

#### P141 One-year progression-free survival in lung cancer patients treated with immune checkpoint inhibitors is significantly associated with a novel immunomodulatory signature but not PD-L1 staining

##### Harsha Ranganath, MD^1^, Amit Jain^1^, Justin Smith^1^, Julie Ryder^2^, Amina Chaudry^1^, Emily Miller^2^, Felicia Hare^2^, Poojitha Valasareddy^2^, Rob Seitz^3^, David Hout^3^, Brock Schweitzer^3^, Tyler Nielsen^3^, Janice Mullins^2^, Gregory Vidal^2^

###### ^1^University of Tennessee Health Sciences, Indianapolis, IN, United States; ^2^West Clinic Cancer Center, Memphis, TN, United States; ^3^Insight Genetics, Nashville, TN, United States

####### **Correspondence:** Gregory Vidal (gvidal@westclinic.com)

**Background**

Immune checkpoint inhibitors (PD-(L)1 inhibitors) have shown promising therapeutic outcomes and have been approved for multiple indications. However, widespread use of PD-(L)1 inhibitors has been limited by a low response rate and immune-related adverse events. Therefore, an improved method for predicting response to the immune checkpoint blockade would better identify patients misclassified by conventional testing. We have evaluated a proprietary algorithm which utilizes gene expression in solid tumors to assess the presence of an immunomodulatory (IM) signature intended to predict immunotherapy response. The purpose of this study was to evaluate the performance of the IM signature against progression-free survival (PFS) of patients treated with immune checkpoint inhibitors.

**Methods**

In this retrospective study, archival tumor tissue from metastatic lung cancer patients treated with one of three PD-(L)1 inhibitors (pembrolizumab, nivolumab, and atezolizumab) either as a single agent or in conjunction with standard chemotherapy, from whom response data was available, was tested for the IM signature. Patients were stratified into two groups based on IM signature classification as positive or negative, which was compared to immunohistochemistry PD-(L)1 testing with a primary endpoint of one-year progression-free survival. Additionally, the IM signature classification was compared with objective response by Spearman’s correlation as a continuous variable.

**Results**

A total of 71 metastatic lung cancer patients were included in the study with a median follow-up of 29 months. The one-year PFS hazard ratio for the IM positive group was 0.31 (95% CI 0.14 to 0.68; p=.004 - Figure 1). A total of 62 out of the 71 metastatic lung cancer patients had previous PD-L1 staining. Head-to-head analysis of PD-L1 and IM signature on these patients found the one-year PFS hazard ratio for the IM group to be 0.30 (95% CI 0.13 to 0.71; p=0.006) and the one-year PFS hazard ratio for PD-L1 positive staining to be 0.76 (95% CI 0.31 to 1.82; p=0.533). The mean IM correlation value with objective response for PD = -0.06; SD = -0.04; PR = 0.14; CR = 0.33; p

**Conclusions**

The IM signature was significantly associated with prolonged one-year progression-free survival among patients treated with PD-L1 inhibitors while PD-L1 staining failed to be significantly associated. Patients classified positive by the IM signature demonstrated a three-fold improved hazard ratio compared to those who were negative. Funding was provided by Insight Genetics working in cooperation with West Cancer Center and Research Institute.

**Ethics Approval**

This study was approved by the West Cancer Clinic Institutional Review Board.

**Fig. 1 (abstract P141).**
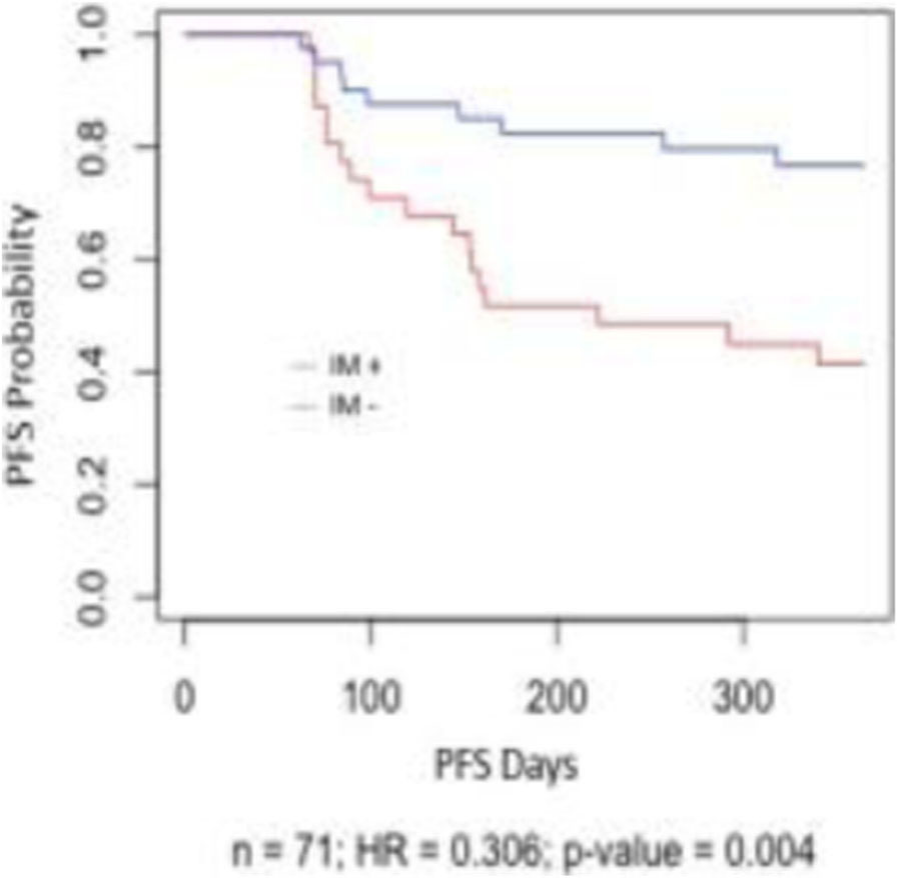
See text for description

#### P142 Development and validation of baseline predictive biomarkers for response to avelumab in second-line (2L) non-small cell lung cancer (NSCLC) using EpiSwitchTM epigenetic profiling

##### Parantu Shah, PhD^1^, Ewan Hunter^2^, Shobha Potluri^3^, Sen Zhang^1^, Mehrnoush Dezfouli^2^, Jennifer Back^2^, Louis James^2^, Navin Jandor^2^, Ryan Powell^2^, Matthew Salter^2^ , Aroul Ramadass^2^, Jayne Green^2^, Willem Westra^2^, Haidong Dong, MD, PhD^4^, Roxana Dronca, MD^4^, Svetomir Markovic, MD, PhD^4^, Alexandre Akoulitchev^2^, Ti Cai^1^, Paul Robbins^3^

###### ^1^EMD Serono, Inc, Billerica, MA, United States; ^2^Oxford Biodynamics, Oxford, United Kingdom; ^3^Pfizer, Inc, San Francisco, CA, United States; ^4^Mayo Clinic, Rochester, MN, United States

####### **Correspondence:** Parantu Shah (parantu.shah@emdserono.com); Matthew Salter (matthew.salter@oxfordbiodynamics.com)

**Background**

Development of baseline predictive classifiers for response to treatment can provide advantages for programs of targeted immunotherapies, development of successful combination therapies, and identification of responder populations to active therapies. Chromosome conformations represent strong systemic cellular network deregulations associated with differences in clinical phenotypes and outcomes [1].

**Methods**

Oxford Biodynamics, in collaboration with the EMD Serono, Inc., a business of Merck KGaA, Darmstadt, Germany/Pfizer alliance," has applied its proprietary technology EpiSwitchTM to monitor systemic epigenetic biomarkers for chromosome conformation signatures in baseline blood samples of patients with multiline anti–PD-L1 (avelumab) treatment of NSCLC. This application was based on the published methodology for validated predictive biomarkers for response to treatment [2], systemic blood-based monitoring of oncological conditions [3-5], and proprietary programs in collaboration with the Mayo Clinic for predictive and response biomarkers in melanoma patients treated with anti–PD-1 therapy (pembrolizumab).

**Results**

A 14-marker classifier was generated with 12 avelumab-treated patients in each response group; in this cohort, responders were defined as patients with complete or partial response, and non-responders were defined as patients with progressive disease. Validation of the developed predictive markers was performed on an independent cohort of 75 patients treated with avelumab as either first-line (1L) or 2L therapy. In the validation cohort, patients with stable disease were also considered as responders in addition to above. The classifier delivered stratifications for response vs nonresponse with 84% accuracy, 79% sensitivity, 92% specificity, 75% positive predictive value (PPV) and 95% negative predictive value (NPV). The associations of EpiSwitch™ response calls with overall survival (OS) and progressive free survival (PFS) in the independent cohort were significant (OS and PFS: log-rank p

**Conclusions**

The established EpiSwitchTM classifier contains strong binary markers of epigenetic deregulation with features normally attributed to genetic markers; the binary status of these classifying markers is statistically significant for survival. Altogether, these findings highlight the potential of the EpiSwitchTM approach for identifying responders and non-responders to immuno-oncology therapies.

**Acknowledgements**

The authors would like to thank patients enrolled in the EMR000070-001 JAVELIN Solid Tumor trial for agreeing for their samples to be used for research purposes. This work is funded by Merck KGaA, Darmstadt, Germany, as part of an alliance between Merck KGaA, Darmstadt, Germany and Pfizer Inc., New York, NY, USA.

**References**

1. Tordini F, Aldinucci M, Milanesi L, et al. The genome conformation as an integrator of multi-omic data: the example of damage spreading in cancer. Front Genet. 2016; 7:194.

2. Carini C, Hunter E; Scottish Early Rheumatoid Arthritis Inception Cohort investigators, et al. Chromosome conformation signatures define predictive markers of inadequate response to methotrexate in early rheumatoid arthritis. J Transl Med. 2018; 16:18.

3. Jakub JW, Grotz TE, Jordan P, et al. A pilot study of chromosomal aberrations and epigenetic changes in peripheral blood samples to identify patients with melanoma. Melanoma Res. 2015; 25:406-11.

4. Bastonini E, Jeznach M, Field M, et al. Chromatin barcodes as biomarkers for melanoma. Pigment Cell Melanoma Res. 2014; 27:788-800.

5. Yan H, Hunter E, Akoulitchev A, et al. Epigenetic chromatin conformation changes in peripheral blood can detect thyroid cancer. Surgery. 2019; 165:44-49.

**Ethics Approval**

The protocol was approved by the institutional review board or independent ethics committee at each center.

#### P143 Development and validation of baseline predictive biomarkers for response to immuno-checkpoint treatments in the context of multi-line and multi-therapy cohorts using EpiSwitchTM epigenetic profiling

##### Parantu Shah, PhD^1^, Ewan Hunter^2^, Shobha Potluri^3^, Sen Zhang^1^, Mehrnoush Dezfouli^2^, Jennifer Back^2^, Louis James^2^, Navin Jandor^2^, Ryan Powell^2^, Matthew Salter^2^, Aroul Ramadass^2^, Jayne Green^2^, Willem Westra^2^, Haidong Dong, MD, PhD^4^, Roxana Dronca, MD^4^, Svetomir Markovic, MD, PhD^4^, Alexandre Akoulitchev^2^, Ti Cai^1^, Paul Robbins^3^

###### ^1^EMD Serono, Inc, Billerica, MA, United States; ^2^Oxford Biodynamics, Oxford, United Kingdom; ^3^Pfizer, Inc, San Francisco, CA, United States; ^4^Mayo Clinic, Rochester, MN, United States

####### **Correspondence:** Parantu Shah (parantu.shah@emdserono.com)

**Background**

Development of baseline predictive classifiers for response to treatment can provide advantages for programs of targeted immunotherapies, development of successful combination therapies, and identification of responder populations to active therapies. Changes in chromosome conformations represent strong systemic cellular network deregulations associated with differences in clinical phenotypes and outcomes [1]. However, questions remain about the applicability of classifiers across treatment lines, indications, and drug combinations.

**Methods**

Oxford Biodynamics, in collaboration with the EMD Serono, Inc., a business of Merck KGaA, Darmstadt, Germany/Pfizer alliance, has applied its proprietary technology EpiSwitchTM to monitor systemic epigenetic biomarkers for chromosome conformation signatures at baseline in patients with multiline anti–PD-L1 (avelumab) treatment of non-small cell lung cancer (NSCLC). Additionally, epigenetic biomarkers to predict outcome and response in patients with melanoma treated with anti–PD-1 (pembrolizumab) and its combination with another agent were identified in collaboration with the Mayo Clinic.

**Results**

Three NSCLC classifiers predicting response to avelumab in first-line (1L), second-line (2L), and combined 1L + 2L cohorts were built and applied to test sets. Average accuracy, positive predictive value (PPV), and negative predictive value (NPV) for 10-fold cross-validation on data splits were reported. An NSCLC patient set treated with 2L pembrolizumab served as an independent test set. The 2L NSCLC classifier achieved high (defined hereafter as > 0.7) predictive power (PPV, NPV, and accuracy) in the 2L test set but not in the 1L test set. A reduced version of this classifier achieved a PPV of 0.71 in the 2L pembrolizumab population. The 1L classifier was not applicable in patients who received 2L treatment for NSCLC. The 1L + 2L composite classifier had high predictive power in both 1L and 2L cohorts and a high PPV for identifying responders in the 2L pembrolizumab population. A fourth classifier starting with preselected NSCLC markers had good predictive power for classifying responders in patients with melanoma treated with pembrolizumab. Finally, a 2L NSCLC classifier trained to classify response groups from pembrolizumab-treated patients also identified NSCLC responders with a high PPV from patients treated with pembrolizumab in combination with an epigenetic drug.

**Conclusions**

Collectively, these results suggest that a set of EpiSwitchTM biomarkers correlates with outcome on anti–PD-1/PD-L1 immunotherapies. Classifier signatures could be generated to work across treatment lines, indications, and combinations, and could be helpful for baseline patient stratification.

**Acknowledgements**

The authors would like to thank patients enrolled in the EMR000070-001 JAVELIN solid tumor trial for agreeing to consent usage of samples for research purposes. This work was funded by Merck KGaA, Darmstadt, Germany, as part of an alliance between Merck KGaA, Darmstadt, Germany and Pfizer Inc., New York, NY, USA.

**References**

1. Tordini F, Aldinucci M, Milanesi L, et al. The genome conformation as an integrator of multi-omic data: the example of damage spreading in cancer. Front Genet. 2016; 7:194.

**Ethics Approval**

The protocol was approved by the institutional review board or independent ethics committee at each enrolling center.

#### P144 Quantification of tumor-stroma-immune contexture by multiplex fluorescent immunohistochemistry and whole-slide digital image analysis

##### Adriana Racolta, PhD^1^, Mehrnoush Khojasteh, PhD^1^, Jennifer Giltnane^2^, Antony Hubbard, BS^1^, Hongjun Zhang^1^, Miriam Matei^1^, Jessica Baumann^1^, Wenjun Zhang, MD, PhD^1^, Tsu-Shuen Tsao, PhD^1^, Hartmut Koeppen^2^, Lisa Ryner^2^, Xingwei Wang^1^, Jim Martin^1^, Auranuch Lorsakul^1^, Ilya Ravkin^1^, Smadar Shiffman^1^, Lidija Pestic-Dragovich^1^, Lei Tang, PhD^1^, Yulei Wang, BA PhD^2^

###### ^1^Roche Tissue Diagnostics, Tucson, AZ, United States; ^2^Genentech, South San Francisco, CA, United States

####### **Correspondence:** Yulei Wang (wang.yulei@gene.com)

**Background**

Understanding response to immunotherapies in relation to tumor-immune contexture requires a paradigm shift from a single-marker test towards multiplexed immunohistochemistry (IHC). Here we report the development, early proof of concept of two fully automated 5-plex fluorescent multiplex IHC assays and accompanying digital pathology algorithms.

**Methods**

Tyramide signal amplification detection was used to inform on the tumor/stroma/immune contexture (CD8, PanCK, FAP, MHC-I, CD31) and to characterize T-cell functions (PD1, CD3, PanCK, GZMB, and PD-L1). Whole slide digital pathology scoring algorithms were developed to identify all phenotypes represented by the markers and their specificity and sensitivity was verified against the results by expert observers.

**Results**

Assay performance including accuracy, precision, and sequential markers detection was validated on > 200 unique cases of Gastric, Pancreatic, Breast, Lung, Urothelial and Colorectal carcinomas. For an early proof-of-concept, we analyzed paired pre- vs. post treatment tumor biopsies from three pancreatic cancer patients treated with a combination of atezolizumab and chemotherapy. Digital pathology algorithms identified biologically and clinically relevant features: MHC-I is highly expressed in tumor cells of the primary lesions and very rare tumor cells express MHC-I in the liver met samples. A patient with partial response (PR) showed a significantly increased tumor MHC-I upon treatment, while two patients with stable disease (SD) did not show significant changes. The PR patient also showed an increased density of CD3+, CD8+, and GZMB+ T cells within the tumor post-treatment, indicating an increased tumoral T cell infiltration and activation by the treatment.

**Conclusions**

The automated 5-plex IHC assays and digital pathology algorithms developed in this study provide a robust tool for quantitative and spatially resolved whole-slide characterization of the tumor-immune contexture. Applying these tools in large-scale clinical investigations may provide better understanding of the response/resistance mechanisms to cancer immunotherapies.

### Cellular Therapies

#### P145 Expanding Iovance’s tumor infiltrating lymphocytes (TIL) from core biopsies for adoptive T cell therapy using a 22-day manufacturing process

##### Michelle Abelson, PhD, Kenneth D'Arigo, Florangel Hilton, Maria Fardis, PhD, MBA, Cecile Chartier

###### Iovance Biotherapeutics, Inc., Tampa Bay, FL, United States

####### **Correspondence:** Cecile Chartier (cecile.chartier@iovance.com)

**Background**

Iovance’s TIL products Lifileucel and LN-145 have demonstrated remarkable clinical activity in melanoma and cervical cancer utilizing Iovance’s proprietary 22-day manufacturing process and surgically resected tumor lesions ~ 1.5-cm diameter [1, 2]. Using a core needle biopsy procedure to obtain tumor samples could allow for greater convenience of collecting the tumor from patients [3]. We asked whether a streamlined manufacturing process could be implemented to produce therapeutically relevant TIL from multiple histologies starting with a core biopsy.

**Methods**

Core biopsies obtained from 4 melanoma and 3 pancreatic, 2 breast, 2 ovarian, and 1 lung tumors were processed in vitro, using a 22-day expansion method termed ‘Core process’. Core biopsy-derived TIL were assessed for expansion, phenotype (lineage, youth/differentiation, activation, and exhaustion markers), function (IFN-gamma and CD107a mobilization), and TCR repertoire.

**Results**

Iovance’s Core process successfully generated TIL products from all tested samples. One to 2 cores yielded more than 10e9 T cells for 10 of the 12 preparations. Phenotypic analyses revealed no significant differences in terms of T cell lineages and memory subsets, or expression of activation, differentiation, and exhaustion markers when compared to Iovance’s current products. Core-derived TIL products responded to PMA and to anti-CD3 stimulations by inducing levels of CD107a mobilization and IFN-gamma secretion like those produced by TIL derived from excisional biopsies. Preliminary TCR sequencing data suggest that high-diversity products can be also be obtained from small samples, similar to what is obtained from TIL expansion.

**Conclusions**

This work demonstrates that the Iovance 22-day Core manufacturing method is highly robust and that it is feasible to expand TIL to therapeutically relevant numbers from as little as 1 to 2 core biopsies from multiple histologies with this method. Resulting products were shown to be phenotypically comparable to, and as potent as, products generated with Iovance’s process from excisional biopsy. Iovance anticipates implementing this process in the clinic in the near future.

**References**

1. Jazaeri AA, Zsiros E, Amaria RN, Artz AS, Edwards RP, Robert Michael Wenham RM, et al. Safety and efficacy of adoptive cell transfer using autologous tumor infiltrating lymphocytes (LN-145) for treatment of recurrent, metastatic, or persistent cervical carcinoma. Clin Oncol. 2019;37:15:2538 (suppl).

2. Sarnaik A, Khushalani NI, Chesney JA, Kluger HM, Curti BD, et al. Safety and efficacy of cryopreserved autologous tumor infiltrating lymphocyte therapy (LN-144, lifileucel) in advanced metastatic melanoma patients who progressed on multiple prior therapies including anti-PD-1. J Clin Oncol. 2019;37:15:2518 (suppl).

3. Ullenhag GJ, Sadeghi AM, Carlsson B, Ahlström H, Mosavi F, Wagenius G, Tötterman TH, et al. Adoptive T-cell therapy for malignant melanoma patients with TILs obtained by ultrasound-guided needle biopsy. Cancer Immunol Immunother. 2012;61:725–732.

#### P146 AUTO6NG: Next generation GD2-targeting CAR T-cell therapy with improved persistence and insensitivity to TGFb and checkpoint inhibition for relapsed/refractory neuroblastoma

##### Daniela Achkova, PhD^1^, Adrian Zarzoso^1^, Yusuf Demir^1^, Fernando Gallardo^1^, Maria Stavrou^1^, Marco Della Peruta^1^, Saket Srivastava^1^, Mathew Robson^1^, Shimobi Onuoha^1^, Simon Thomas^1^, Shaun Cordoba^1^, Martin Pule^1,2^

###### ^1^Autolus Ltd, London, United Kingdom; ^2^University College London Cancer Institute, London, United Kingdom

####### **Correspondence:** Martin Pule (m.pule@autolus.com)

**Background**

Neuroblastoma is the most common extracranial solid cancer in children with poor long-term survival in those with high-risk disease. A currently ongoing phase I clinical study of GD2-targeted CART for refractory/relapsed neuroblastoma (NCT02761915) shows activity against disseminated disease without inducing on target/off tumor toxicity. However, CART persistence was limited and clinical activity transient and incomplete.

Building on the GD2 CAR used in this study, we have developed a next generation T-cell product candidate termed AUTO6NG. The AUTO6NG product consists of 3 distinct populations of GD2-targeted CAR T-cells, produced by dual transduction of T-cells with two separate retroviral vectors. The first vector directs the expression of a GD2-targeting CAR, co-expressed with a constitutively signalling IL7 cytokine receptor (IL7R_CCR) (product A), while the second vector is a tri-cistronic retroviral vector encoding the same GD2 CAR, co-expressed with dominant negative TGFbRII (dnTGFbRII) and truncated SHP2 (dSHP2) (product B). dSHP2 confers resistance to inhibitory signals such as those from PD1.

**Methods**

Human T-cells were either dual transduced with both vectors yielding a mix of product A/B/A+B (AUTO6NG) or single transduced with each vector individually giving raise to product A or B. Both single and dual transduced CAR T-cells were extensively evaluated in vitro for redirected lysis, cytokine secretion, T-cell proliferation and survival and resistance to immunosuppressive pathways (including TGFb and PD1/PDL1 inhibition) in co-culture assays with GD2-positive and negative tumour cell lines. Additionally, anti-tumour activity of AUTO6NG was evaluated in vivo by intravenous administration in an established neuroblastoma xenograft model in NSG mice.

**Results**

AUTO6NG T-cells (product A/B/A+B) were highly potent in cytotoxicity assays against GD2 positive tumour cell lines with no differences observed compared with single transduced CAR T-cells (product A or B). Expression of the IL7R_CCR in both AUTO6NG and product A conferred exogenous-cytokine-independent viability and homeostatic proliferation of modified T-cells, without causing autonomous T-cell growth. Furthermore, AUOTO6NG T-cells and product B but not product A proved resistant to both TGFb- and PD1/PDL1-mediated immunosuppression in vitro due to the presence of dnTGFbRII and dSHP2 in those genetically engineered CAR T-cells. Finally, intravenous delivery of AUTO6NG exhibited potent anti-tumour activity and extended survival in NSG mice with established tumour burden.

**Conclusions**

These results demonstrate the feasibility, safety, and efficacy of AUTO6NG T-cells. The addition of IL7R_CCR, dnTGFbRII and dSHP2 modules to the AUTO6NG product augment its functions by extending T-cell persistence and rendering modified T-cells resistant to TGFb- and PD1/PDL1-driven immune inhibition.

#### P147 Effect of chemotherapy on cellular kinetics of NKG2D-based CAR T-cells in metastatic colorectal cancer patients

##### Erik Marcelo Alcantar Orozco, Eytan Breman, MSc, Marie-Sophie Dheur, PhD, Fabian Borghese, PhD, Emilie Cerf, PhD, Nathalie Braun, Caroline Lonez, PhD, Anne Flament, Frederic Lehmann, MD

###### Celyad, Mont-Saint-Guibert, Belgium

####### **Correspondence:** Frederic Lehmann (flehmann@celyad.com)

**Background**

Autologous and allogeneic Chimeric Antigen Receptor (CAR) T-cells are under thorough investigation to translate their success in B-cell malignancies to other types of cancer. Previous studies associated the anti-tumour effect of CAR T-cells to their long-term persistence. Most studies use cyclophosphamide and fludarabine (CyFlu) preconditioning chemotherapy to facilitate CAR T-cell persistence. However, the effect of CyFlu preconditioning was rarely compared to other chemotherapies or to CAR T-cells alone. The THINK, SHRINK and ALLOSHRINK trials evaluate the safety and clinical activity of NKG2D receptor-based CAR T-cells in metastatic colorectal cancer (mCRC) patients. THINK and SHRINK utilize autologous CAR T-cells, whereas ALLOSHRINK utilizes allogeneic CAR T-cells. In THINK, CAR T-cells are injected without preconditioning chemotherapy or after CyFlu. In SHRINK and ALLOSHRINK, FOLFOX chemotherapy is given before CAR T-cell injections. Herein we present cellular kinetics results from these three trials.

**Methods**

Whole blood samples were drawn at various timepoints from patients receiving at least one injection of CAR T-cells. Peripheral blood mononuclear cells (PBMCs) were isolated by ficoll gradient centrifugation at a central laboratory designated by the Sponsor. Genomic DNA was isolated using a commercially available kit. Engraftment of CAR T-cells was measured by digital droplet polymerase chain reaction (ddPCR) using transgene-specific primers and reported as transgene copies per microgram of genomic DNA. Long-term persistence of CAR T-cells was measured by calculating the area under the curve (AUC) using the linear trapezoidal rule.

**Results**

35 mCRC patients have been treated in THINK (14), SHRINK (9) and ALLOSHRINK (12). Preliminary results are available for 29 subjects. Cell kinetics for subjects having received one injection of autologous CAR T-cells show a seven-fold increase in mean peak levels of T-cell engraftment with CyFlu compared to FOLFOX. Mean AUC is four times higher with CyFlu compared to FOLFOX. Peak levels of engraftment and persistence observed with FOLFOX and without previous chemotherapy are similar. Additionally, allogeneic CAR T-cells exhibit a five-fold increase in mean AUC and a ten-fold increase in mean peak levels compared to autologous cells with the same prior chemotherapy regimen. Additional analyses will be presented during the congress.

**Conclusions**

Analyses of the initial 29 patients receiving either autologous or allogeneic NKG2D-based CAR T-cells demonstrate that CyFlu enhances peak levels and persistence of adoptively transferred cells. FOLFOX does not appear to influence engraftment or persistence of CAR T-cells. Allogeneic CAR T-cells show higher peaks and time-averaged persistence compared to autologous cells. Analysis of the results is ongoing.

**Ethics Approval**

The studies referred to in this abstract were approved by all relevant ethical committees and authorities.

#### P148 High affinity NK cells expressing a PD-L1 chimeric antigen receptor demonstrate anti-tumor activity in head and neck cancer through multiple distinct mechanisms

##### Yevtte Robbins^1^, Jay Friedman, PhD^1^, Sarah Greene^1^, Kellsye Fabian, PhD^1^, Michelle Padget^1^, John Lee, MD^2^, Patrick Soon-Shiong, MD^2^, Kayvan Niazi^3^, Lennie Sender^2^, Laurent Boissel^2^, Jeffrey Schlom, PhD^1^, James Hodge, PhD, MBA^1^, Clint Allen, MD^1^

###### ^1^NIH, Bethesda, MD, United States; ^2^NantKwest, Culver City, CA, United States; ^3^Nantworks, Culver City, CA, United States; ^4^NIH/NIDCD, Bethesda, MD, United States

####### **Correspondence:** Clint Allen (clint.allen@nih.gov)

**Background**

A significant portion of head and neck cancers (HNCs) harbor genomic alterations that render them insensitive to T cell detection. For these patients, natural killer (NK) cellular therapy may be an effective complementary treatment approach. We studied the anti-tumor activity of a novel, off the shelf, NK cellular therapy consisting of high affinity NK cells engineered to express a chimeric antigen receptor (CAR) targeting PD-L1 (PD-L1 t-haNKs).

**Methods**

Irradiated (15 Gy) PD-L1 t-haNK cells were assessed for direct cytotoxicity of five human and two murine HNC cell lines by real-time impedance analysis. PD-L1 knockout by CRISPR/Cas9 gene editing was performed in select cells to assess PD-L1-specific killing. Co-culture assays with PD-L1 t-haNKs and murine or human peripheral and tumor infiltrating leukocytes were performed to determine selective elimination of cells. Wild-type C57BL/6 (B6) or NSG mice were engrafted with parental or PD-L1 knockout murine or human tumors and assessed for tumor growth inhibition (TGI) following PD-L1 t-haNK treatment.

**Results**

PD-L1 CAR expression on PD-L1 t-haNKs was verified. PD-L1 t-haNKs killed all human and murine HNC cell lines at low effector:target ratios. Killing of cells was significantly enhanced with increased PD-L1 expression following IFN-γ pre-treatment. Baseline killing was partially reversed and IFN-γ -enhanced killing was completely abrogated in PD-L1 knockout cells. Ex vivo co-culture of PD-L1 t-haNKs with peripheral and tumor infiltrating leukocytes from tumor bearing mice or with peripheral leukocytes from HNC patients revealed selective elimination of PD-L1 high macrophages and myeloid derived suppressor cells (MDSC) but not lymphocyte subsets. Treatment of B6 mice bearing murine oral cancers with PD-L1 t-haNKs in vivo resulted in ≥50% reduction in PD-L1 high macrophages and MDSC but no reduction in lymphocytes. Treatment of NSG mice bearing parental human HNC or B6 mice bearing parental murine oral cancer resulted in significant TGI after PD-L1 t-haNK treatment. TGI was completely abrogated in mice bearing PD-L1 knockout tumors.

**Conclusions**

PD-L1 t-haNKs mediated potent PD-L1-specific cytotoxicity against HNC cells and selectively eliminate immunosuppressive macrophages and MDSC expressing high levels of PD-L1 from the periphery and tumor microenvironment. PD-L1 t-haNK monotherapy resulted in PD-L1-specific TGI in xenograft and syngeneic models. These data provide the pre-clinical rationale for the clinical study of PD-L1 t-haNKs in solid tumors. Evidence that PD-L1 t-haNKs selectively eliminate immunosuppressive macrophages and MDSC support the clinical study of PD-L1 t-haNKs as a monotherapy or in combination with treatments designed to activate T cell immunity.

**Ethics Approval**

The study was approved by the NIH Animal care and Use Committee, approval number 1464-18.

#### P149 Silencing PD-1 using self-delivering RNAi PH-762- to improve Iovance TIL effector function using Gen 2 manufacturing method

##### Inbar Azoulay-Alfaguter, PhD^1^, Michelle Abelson, PhD^1^, Krit Ritthipichai, DVM, PhD^1^, Kenneth D’Arigo^1^, Florangel Hilton^1^, Marcus Machin, BS^2^, Dingxue Yan^2^, James Cardia^2^, Maria Fardis, PhD, MBA^1^, Cecile Chartier^1^

###### ^1^Iovance Biotherapeutics, Inc., Tampa, FL, United States; ^2^Phio Pharmaceuticals, Tampa, FL, United States

####### **Correspondence:** Cecile Chartier (cecile.chartier@iovance.com)

**Background**

Adoptive T-cell transfer with tumor infiltrating lymphocytes (TIL) is an investigational immunotherapy for advanced solid cancers. Ongoing Phase II clinical trials of Iovance’s lifileucel and LN-145 TIL products have demonstrated efficacy with ORRs of 38% and 44% in patients with melanoma and cervical cancer, respectively [1,2]. Anti-PD-1 therapy has been widely used as a first-line therapy in several types of cancer. TIL infusion products from the patients previously treated with anti-PD-1 therapy still sustain PD-1 expression, especially the subset of tumor antigen-specific TIL [3]. Building on the therapeutic efficacy of PD-1 blockade, we reasoned that intrinsic silencing of PD-1 in our TIL products, may provide similar benefits to systemic administration of anti-PD-1 therapy, while decreasing the side effects associated with systemic anti-PD-1 [3]. Self-delivering small interfering RNA (sd-rxRNA) is a chemically modified siRNA molecule, which has ability to penetrate cell types with high knockdown efficiency of specific target genes [4]. Furthermore, a knockdown approach yields a transient effect, which may prove a more favorable approach when compared with permanent genetic modification. Here, we tested the silencing efficiency of a PD-1-targeted sd-rxRNA, termed PH-762, in TIL and its effect on TIL phenotype and function.

**Methods**

TIL from melanoma, breast cancer, lung cancer, H&N cancer, and sarcoma were expanded ex vivo with Iovance’s proprietary 22-day process in the presence of PH-762. Resulting TIL products were assessed for PD-1 knockdown, cell expansion and viability, phenotype (T-cell lineage, differentiation, activation, and exhaustion), and effector functions (IFN-gamma induction).

**Results**

Average silencing of the PD-1 levels was 85%. Sixteen of the 19 tumors tested demonstrated >80% silencing at the surface of PH-762-treated TIL relative to control sd-rxRNA-treated TIL. The remaining 3 samples had ~70% silencing efficiency. Expression of T-cell activation markers including 4-1BB and OX40 was significantly increased in TIL expanded with PH-762. Importantly, other inhibitory and exhaustion molecules remained unaffected, suggesting that compensatory mechanisms were not triggered by PD-1 silencing. Functionally, PD-1 knockdown TIL displayed elevated IFN-gamma secretion when co-cultured with autologous tumor cells, indicating improved effector function upon specific T-cell re-stimulation.

**Conclusions**

sd-rxRNA-mediated silencing of PD-1 with PH-762 in TIL was highly efficient and generated TIL products with elevated effector function, providing a strong rationale for clinical testing.

**Acknowledgements**

PH-762 was kindly provided by Phio Pharmaceuticals.

**References**

1. Sarnaik A. et al. Safety and efficacy of cryopreserved autologous tumor infiltrating lymphocyte therapy (LN-144, lifileucel) in advanced metastatic melanoma patients who progressed on multiple prior therapies including anti-PD-1. J Clin Oncol. 2019;37:2518-2518.

2. Jazaeri A A, et al. Safety and efficacy of adoptive cell transfer using autologous tumor infiltrating lymphocytes (LN-145) for treatment of recurrent, metastatic, or persistent cervical carcinoma. J ClinOncol. 2019;37:2538-2538.

3. Gros A, et al. PD-1 identifies the patient-specific CD8(+) tumor-reactive repertoire infiltrating human tumors. J Clin Invest. 2014;124:2246-2259.

4. Ligtenberg M A, et al. Self-Delivering RNAi Targeting PD-1 Improves Tumor-Specific T Cell Functionality for Adoptive Cell Therapy of Malignant Melanoma. Mol Ther. 2018;26:1482-1493.

#### P150 1st-in-human CAR T clinical trial for metastatic breast cancers

##### Cynthia Bamdad, PhD , Andrew Stewart, PhD, Pengyu Huang, PhD, Benoit Smagghe, PhD, Scott Moe, PhD, Tyler Swanson, Thomas Jeon, Danica Page, Ketan Mathavan, PhD, Trevor Grant, PhD, Rachel Herrup

###### Minerva Biotechnologies, Waltham, MA, United States

####### **Correspondence:** Cynthia Bamdad (cbamdad@minervabio.com)

**Background**

Minerva will open a 1st-in-human CAR T clinical trial for metastatic breast cancers at the Fred Hutchinson Center September, 2019. huMNC2-CAR44 targets a novel form of MUC1; no therapeutic that targets this form has ever been tested in humans. All previous, failed attempts to therapeutically target MUC1 have targeted the tandem repeat domains, which are cleaved and shed from the surface of cancer cells. Cleavage and shedding of the tandem repeat domain increases as tumor stage increases. huMNC2-CAR44 targets the truncated extra cellular domain of MUC1* (muk 1 star), also known as MUC1-C, which is the transmembrane cleavage product that remains after MUC1 is cleaved and the tandem repeat domain is shed from the cancer cells. The MNC2 antibody, which is the targeting head of the CAR, cannot bind to full-length MUC1. It binds to an ectopic epitope that is only unmasked by cleavage and release of the MUC1 tandem repeat domain. MUC1* growth factor receptor is activated when onco-embryonic growth factor NME7AB dimerizes its truncated extracellular domain. NME7AB and the huMNC2 antibody both compete for the same binding site, which is masked in full-length MUC1.

**Methods**

Monoclonal antibody MNC2 was selected because it recognizes a conformational epitope within MUC1* that is created by cleavage by MMP9, which is overexpressed in breast cancers and is an indicator of poor prognosis. The luminal edge of some normal tissues express a cleaved MUC1*-like form; however, on normal tissues, MUC1 is cleaved by a different cleavage enzyme, which alters the conformation of the truncated extra cellular domain and it is not recognized by the MNC2 antibody.

**Results**

huMNC2-scFv recognizes 95% of breast cancers, across all subtypes, wherein the average percent staining for each tissue specimen is ~80%. Despite this robust staining of cancerous tissues, huMNC2-scFv showed almost no binding to normal tissues and no staining of critical organs. In vitro, huMNC2-CAR44 T cells killed cancer cells, but not non-cancer cells even if they expressed MUC1 or a cleaved MUC1. In NSG mice (n>300), huMNC2-CAR44 T cells eliminated MUC1* positive tumors from implanted naturally occurring breast cancer cells. A single CAR T cell injection eliminated tumors for 100 days; control animals had to be sacrificed at Day 20. Further, huMNC2-CAR44 T cell mediated killing increased as MUC1* density increased (Figure 1).

**Conclusions**

If successful, huMNC2-CAR44 could treat a wide variety of solid tumors. huMNC2-scFv binds to 95% of breast, 83% ovarian, 78% pancreatic and 71% of lung cancers.

**Fig. 1 (abstract P150).**
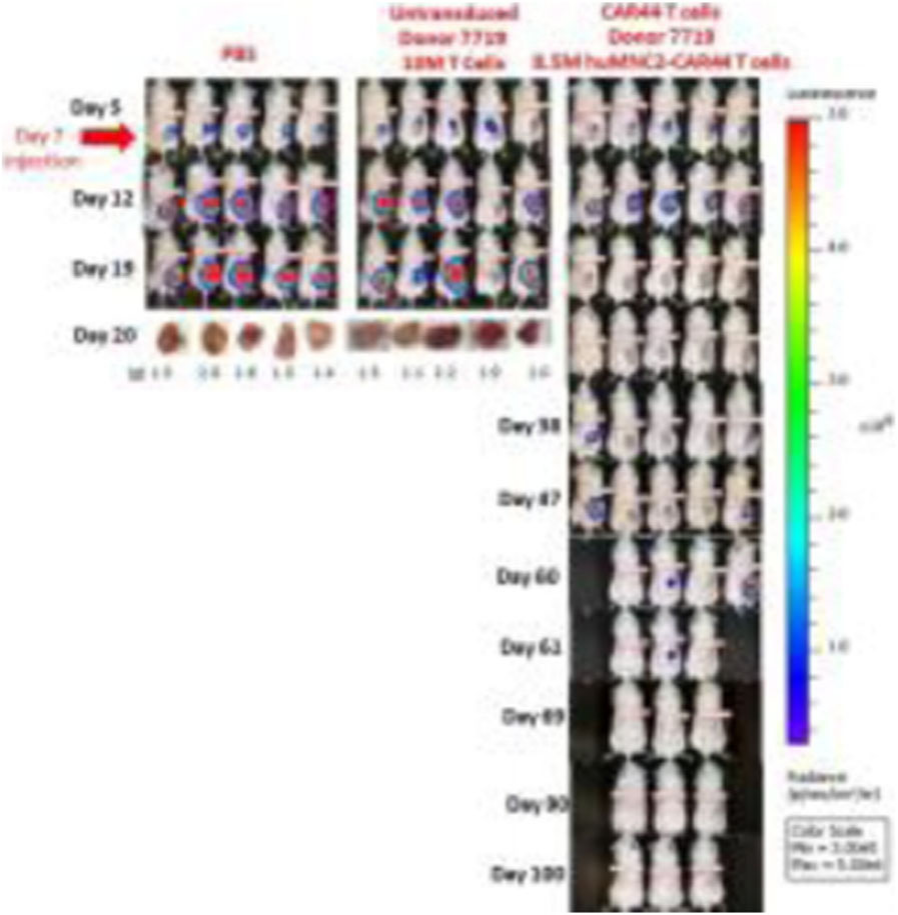
huMNC2-CAR44 T cells kill MUC1* positive tumors

#### P151 Solid tumor cytotoxicity by natural killer cells expressing a HER2-directed chimeric antigen receptor enhanced by MyD88/CD40 (MC)

##### Xiaomei Wang, PhD, Daniel Jasinski, PhD, Jan Medina, David Spencer, PhD, Aaron Foster, PhD, Joseph Bayle, PhD

###### Bellicum Pharmaceuticals, Houston, TX, United States

####### **Correspondence:** Aaron Foster (afoster@bellicum.com); Joseph Bayle (jhbayle@bellicum.com)

**Background**

The potent, innate anti-tumor cytotoxicity of natural killer (NK) cells combined with their low risk of inducing graft-versus-host disease have made NK cells an emerging platform for allogeneic, off-the-shelf CAR-based cell therapies. However, adoptive transfers of NK cells have shown limited expansion and persistence which may impact their ability to induce durable anti-tumor responses. Here, we demonstrate that constitutive expression of a novel chimeric costimulatory protein, comprised of the signaling domains from MyD88 and CD40 (MC) and secreted IL-15 dramatically improves the proliferation and anti-tumor efficacy of HER2 CAR-redirected NK cells.

**Methods**

Human CD56-positive cells were enriched from PBMCs derived from healthy donors and activated with irradiated K562 cells in the presence of IL-15. NK cells were subsequently transduced with retroviral vector encoding inducible Caspase-9 (iC9), a HER2-specific CAR (HER2.ζ), MyD88/CD40 (MC) [1] and IL-15. Gene-modified NK cells were evaluated for expansion, cytotoxicity, cell phenotype and cytokine production, in vitro and in an HER2 positive OE-19 NSG mouse xenograft model.

**Results**

NK cells were efficiently transduced (>50%) and demonstrated robust ex vivo expansion (150 fold, 14 days post-activation) in culture relative to transduced NK cells. In coculture assays with HER2-expressing OE19 and SKOV3 tumor cells, MC-enhanced CAR-NK cells showed potent cytotoxicity with elevated expression of pro-inflammatory cytokines and chemokines including MIP1α, IFN-γ, and GM-CSF. In addition, NK cells expressing the iC9 safety switch could be rapidly ablated by treatment with 1 nM rimiducid to initiate apoptosis. In animals engrafted with OE-19 tumor cells, iC9-CAR.ζ-MC-IL15 modified NK cells demonstrated significantly improved control of tumor expansion compared with control NK cells.

**Conclusions**

MyD88/CD40 and IL-15 enhance the proliferation and anti-tumor potency of CAR-modified NK cells. Further, inclusion of the iC9 safety switch can be used to mitigate potential toxicities. These technologies have the potential to provide a potent, off-the-shelf allogeneic cell therapy to treat solid tumors.

**Reference**

1. Collinson-Pautz MR, Chang WC, Lu A, Khalil M, Crisostomo JW, Lin PY, Mahendravada A, Shinners NP, Brandt ME, Zhang M, Duong M, Bayle JH, Slawin KM, Spencer DM, Foster AE. Constitutively active MyD88CD40 costimulation enhances expansion and efficacy of chimeric antigen receptor T cells targeting hematological malignancies. Leukemia. 2019; 33:2195-2207.

**Ethics Approval**

This study was approved by Bellicum's IACUC and performed in its AAALAC approved vivarium.

#### P152 Antigen delivery to PBMCs by microfluidic squeezing primes anti-tumor immunity

##### Matthew Booty, PhD, Kelan Hlavaty, Emrah Ozay, PhD, Carolyne Smith, PhD, Katherine Seidl, PhD, Howard Bernstein, MD, PhD, Armon Sharei, Scott Loughhead

###### SQZ Biotechnologies, Watertown, MA, United States

####### **Correspondence:** Matthew Booty (matt.booty@sqzbiotech.com)

**Background**

The presentation of sufficient antigen on major histocompatibility complex class I (MHC-I) is a potential barrier to generating potent cancer immunizations. We use microfluidics-based squeezing to deliver antigen directly to the cytosol of target antigen presenting cells (APCs) – resulting in the enhanced presentation of antigen on MHC-I. In addition to facilitating potent CD8+ T cell priming by professional APCs, this approach can make unfractionated peripheral blood mononuclear cells (PBMCs) effective, unorthodox APCs capable of priming CD8+ T cell responses in mouse and human systems.

**Methods**

Protein and peptide antigens were delivered to the cytosol of murine splenocytes or human PBMCs by microfluidic squeezing. The response to in vivo immunization was assessed by flow cytometry in a series of experiments in mice. Tumor experiments were conducted with the TC-1 cell line, which expresses the viral antigens E6 and E7 from human papilloma virus type 16 (HPV16).

Human PBMCs were loaded with synthetic long peptides (SLPs) containing MHC-I restricted epitopes from cytomegalovirus (CMV) or HPV16. These PBMCs were co-cultured with epitope-reactive human responder CD8+ T cells, and interferon gamma production was quantified to assess antigen-specific responses in vitro.

**Results**

In mice, we demonstrate that microfluidic squeezing enables delivery to all cell subsets within the spleen and that delivered protein antigen is rapidly processed and presented on MHC-I. In vivo immunization using splenocytes squeezed with a HPV16-derived E7 SLP primes E7-specific responses. Prophylactic immunization of mice implanted with TC-1 resulted in complete protection and these responses were durable, as mice were protected upon TC-1 re-challenge. Therapeutic immunization following TC-1 implantation reduced tumor growth and extended survival compared to unimmunized mice (25 days vs 50 days). Following therapeutic immunization, 85% of tumor infiltrating CD8+ T cells were found to be E7-specific compared to 3% in unimmunized mice.

In human cells, we demonstrate that squeezing of primary PBMCs enables delivery to all cell subsets. Delivery of CMV and HPV16 SLPs leads to presentation on MHC-I, as demonstrated by in vitro responses of both CD8+ T cell clones and patient-derived memory populations. Delivery of CMV antigens at the manufacturing scale (~1 x 10^9 cells) also results in presentation and activation of CD8+ T cells.

**Conclusions**

Through the direct cytosolic delivery of antigen, we engineered unfractionated PBMCs to function as potent APCs. This strategy has demonstrated significant potential to generate CD8+ T cell responses in both mouse and human systems and has been scaled for clinical implementation.

**Ethics Approval**

Human samples were supplied by an approved vendor and animal studies were conducted in accordance with SQZ Biotech's Animal Care Program and IACUC which operate according to principles set forth in PHS Policy and the Guide for the Care and Use of Laboratory Animals - 8th edition.

#### P153 Memory CD8+ T cells are more resistant to cancer stem cell (CSC) suppression than effector CD8+ T cells and are more effective at targeting CSC in a murine melanoma model

##### Brooke Bredbeck, MD^1^, Shibin Qu^2^, Alicia Kevelin^2^, Ashley Pepple^2^, Amy Felsted^1^, Anutosh Ganguly^2^, Clifford Cho, MD, FACS^1^

###### ^1^University of Michigan Medical School, Ann Arbor, MI, United States; ^2^Ann Arbor VA Medical Center, Ann Arbor, MI, United States

####### **Correspondence:** Clifford Cho (cliffcho@med.umich.edu)

**Background**

The ability to suppress immune reactivity is a defining hallmark of cancer [1-3]. Both the administration and disinhibition of CD8+ T cells, through adoptive immunotherapy and checkpoint inhibition respectively, have yielded unprecedented responses in patients with advanced melanoma [4-8]. However, a majority of patients remain stubbornly unresponsive to T cell-based therapy [9,10]. A better knowledge of cancer-induced T cell suppression is needed improve efficacy. Memory CD8+ T cells (Tmem) are more effective than effector CD8+ T cells (Teff) at controlling melanoma growth after adoptive cell transfer (ACT) in a murine melanoma model [11,12]. Melanoma cancer stem cells (CSC) are primarily responsible for tumor growth and metastasis [13,14]. We hypothesized that Tmem are both more resistant to CSC suppression and more effective at targeting CSC after ACT.

**Methods**

The B16F10 melanoma cell line was stably transfected to express low levels of lymphocytic choriomeningitis virus (LCMV) peptide antigen GP33 (B16GP33). Ly5.1+/C57BL/6 mice were infected with LCMV to isolate Teff and Tmem on post-infection days 8 or > 30, respectively. Ly5.2+/C57BL/6 mice were inoculated with subcutaneous B16GP33 tumors followed by either no treatment or ACT with Teff or Tmem on days 1 or 7. On day 18-20, tumors were harvested for flow cytometric analysis (FACS) to characterize tumor-infiltrating lymphocytes (TIL) and composition of melanoma CSC versus non-CSC (NCSC) based on expression of the CSC-specific marker aldehyde dehydrogenase (ALDH).

**Results**

Tumor inhibition was observed after ACT, with greatest treatment effect found after Tmem ACT (Figure 1). FACS analysis of CD8+ TIL showed a predominant exhausted and non-activated phenotype after Teff ACT; in contrast, CD8+ TIL exhibited a highly activated phenotype as well as superior endogenous CD8+ T cell recruitment (Figure 2) after Tmem ACT. FACS analysis of tumor cells after ACT demonstrated that ALDHhigh CSC fractions were markedly expanded after Teff ACT, but diminished after Tmem ACT (Figure 3).

**Conclusions**

Tmem-based ACT resulted in optimal tumor growth suppression, a more activated TIL phenotype with superior CD8+ T cell recruitment, and substantially stronger clearance of CSC compared to Teff ACT and controls. These observations suggest that use of Tmem may enable cellular therapies to more effectively evade the suppressive effects of melanoma while selectively targeting CSC.

**References**

1. Marincola F, Wang E, Herlyn M, et al. Tumors as elusive targets of T-cell-based active immunotherapy. Trends Immunol. 2003; 24:335-342.

2. Dunn GP, Old LJ, et al. The three E’s of cancer immunoediting. Ann Rev Immunol. 2004; 22:329-360.

3. Rabinovich GA, Gabrilovich D, Sotomayor EM. Immunosuppressive strategies that are mediated by tumor cells. Ann Rev Immunol. 2007; 25:267-295.

4. Rosenberg SA, Yang JC, Sherry RM, et al. Durable complete responses in heavily pretreated patients with metastatic melanoma using T-cell transfer immunotherapy. Clin Cancer Res. 2011; 17:4550-4557.

5. Dudley MS, Wunderlich JR, Yang JC, et al. Adoptive cell transfer therapy folloing non-myeloablative but lymphodepleting chemotehrapy for the treatemnt of patients with refractory metastatic melanoma. J Clin Oncol. 2005; 23:2346-2357.

6. Hodi FS, O’Day SJ, McDermott DF, et al. Improved survival with ipilimumab in patients with metastatic melanoma. N Engl J Med. 2010; 363:711-723.

7. Larkin J, Chiarion-Sileni V, Gonzalez R, et al. Combined nivolumab and ipilimumab or monotherapy in untreated melanoma. N Engl J Med. 2015; 373:23-34.

8. Wolchok JD, Kluger H, Callahan MK, et al. Nivolumab plus ipilimumab in advanced melanoma. N Engl J Med. 2013; 369:122-33.

9. Brahmer JR, Tykodi SS, Chow LQM, et al. Safety and activity of anti-PD-L1 antibody in patients with advanced cancer. N Engl J Med. 2012; 366:2455-2465.

10. Royal RE, Levy C, Turner K, et al. Phase 2 trial of single agent ipilimumab (anti-CTLA-4) for locally advanced or metastatic pancreatic adenocarcinoma. J Immunother. 2010; 33:828-33.

11. Contreras A, Sen S, Tatar AJ, et al. Enhanced local and systemic anti-melanoma CD8+ T cell responses after memory T cell-based adoptive immunotherapy in mice. Cancer Immunol Immunother. 2016; 65:601-611.

12. Contreras A, Beems MV, Tatar AJ, et al. Co-transfer of tumor-specific effector and memory CD8+ T cells enhances the efficacy of adoptive melanoma immunotherapy in a mouse model. J Immunother Cancer. 2018; 6:41.

13. Maccalli C, DeMaria R. Cancer stem cells: perspectives for therapeutic targeting. Cancer Immunol Immunother. 2015; 64:91-97.

14. Pan Q, Li Q, Liu S, Ning N, Zhang X, Yu Y, Chang AE, Wicha MS. Concise review: targeting cancer stem cells using immunological approaches. Stem Cells. 2015; 33:2085-2092.

**Ethics Approval**

This study was approved by University of Michigan’s ethics board (IACUC), approval #1608-004.

**Fig. 1 (abstract P153).**
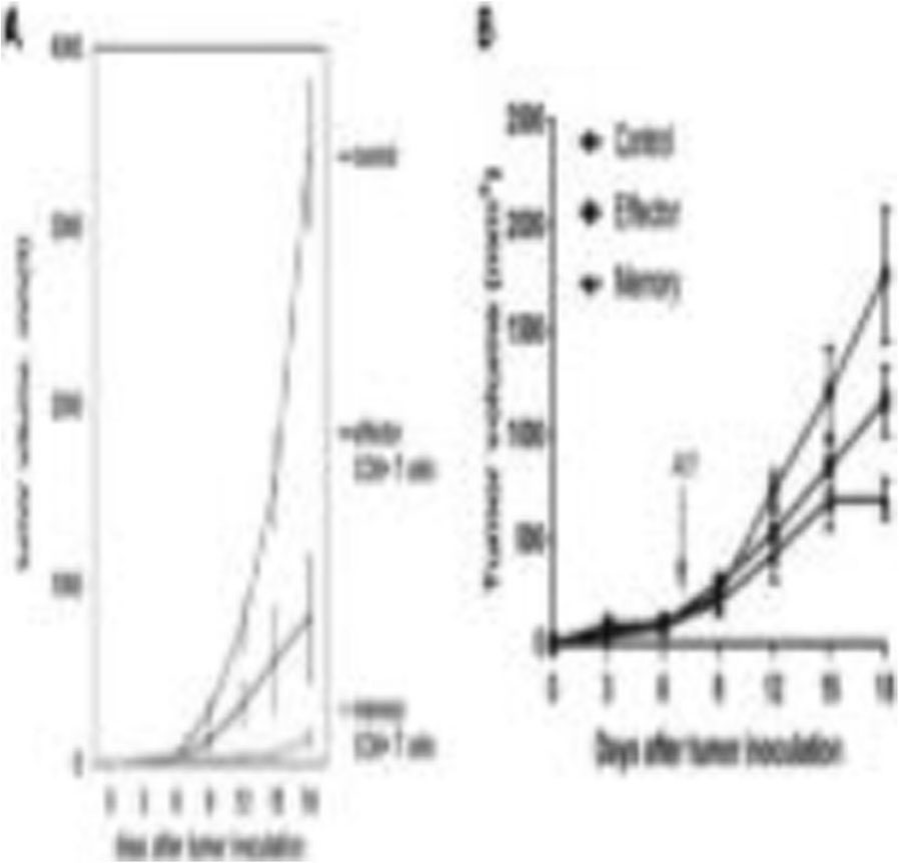
Memory CD8+ T cells vs effector CD8+ T cells in melanoma

**Fig. 2 (abstract P153).**
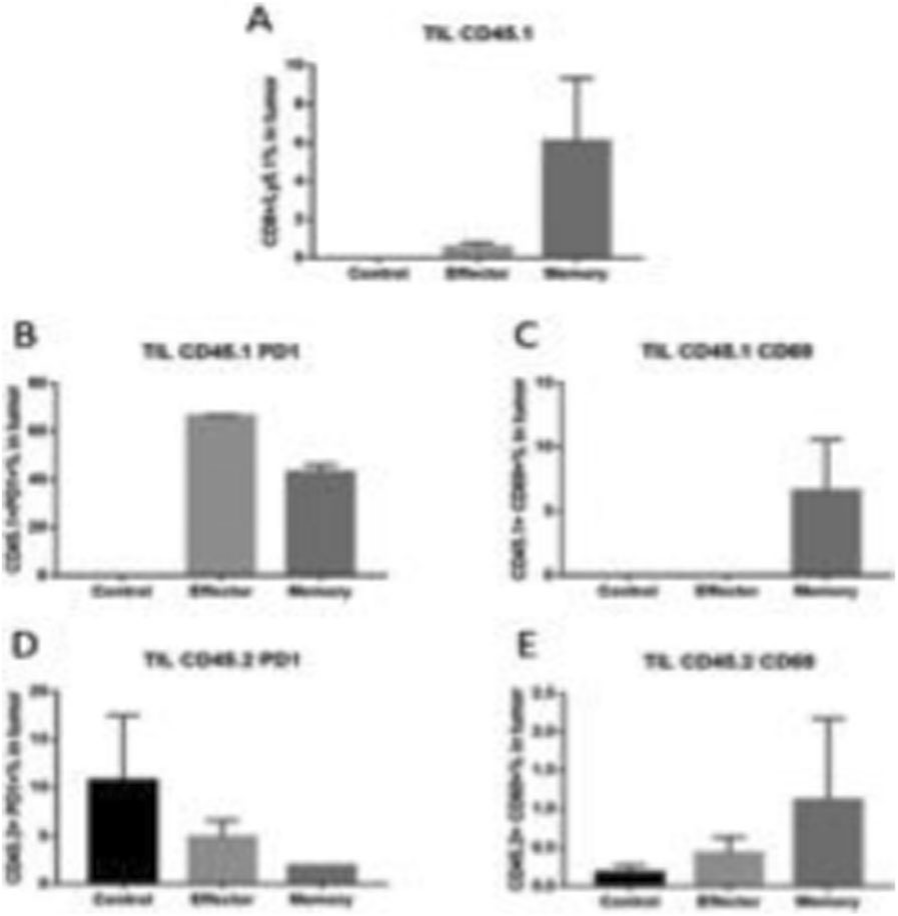
CD8+ TIL exhibit a more activated phenotype after memory ACT

**Fig. 3 (abstract P153).**
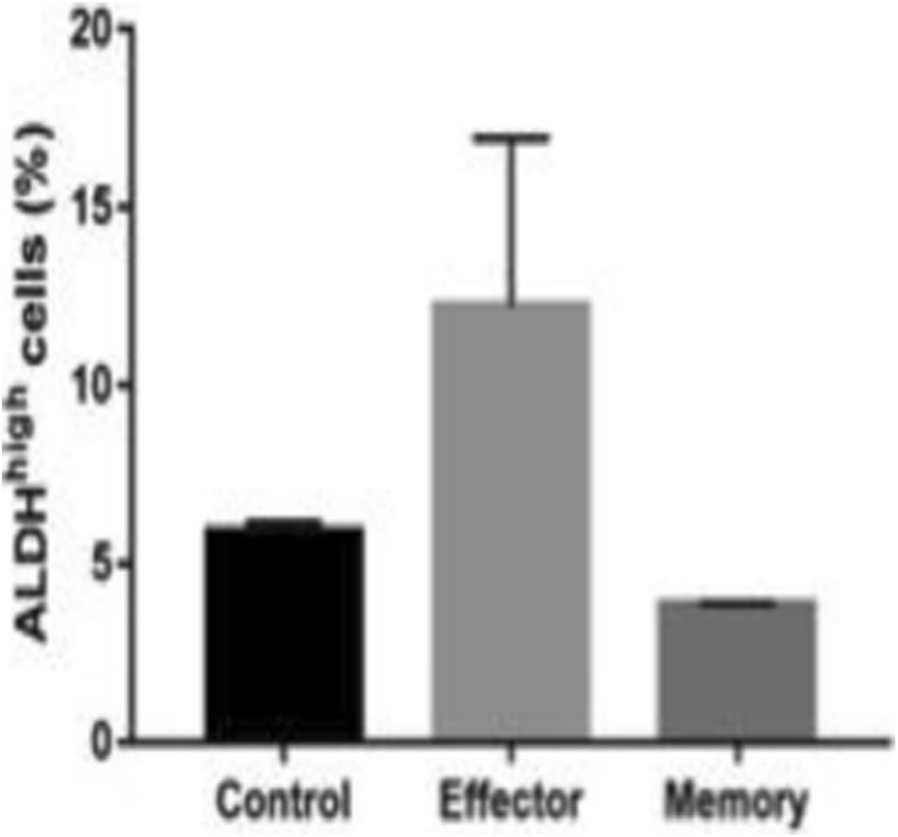
Memory CD8+ T cells target melanoma CSC

#### P154 Development of an antigen-presenting bead kit for activation and expansion of human antigen-specific T cells

##### Yelena Bronevetsky, PhD (yelena.bronevetsky@gmail.com)

###### Berkeley Lights Inc, Alameda, CA, United States

**Background**

Immunogenicity validation of peptide neoantigens represents a critical bottleneck in the tumor antigen discovery process. Current bioinformatics platforms for antigen prediction are unsatisfactory, forcing researchers to screen many peptides per protein target in order to identify the few bona fide antigens. Standard immunogenicity assays also cannot discriminate antigen-Human Leukocyte Antigen (HLA) binding from T cell receptor (TCR) recognition, leading to unnecessary screening of non-HLA binders in expensive and lengthy T cell reactivity assays that require large numbers of expensive primary cells. Further, the use of donor-derived antigen-presenting cells to assay T cell immunogenicity and expand rare, antigen-specific cells from the endogenous repertoire has inherent variability and a minimal degree of quality control. Berkeley Lights has developed an artificial antigen-presenting bead kit that expands antigen-specific T cells from peripheral blood.

**Methods**

Peptide binding to HLA Class I and stability of the peptide-HLA complex is assayed by loading peptides onto beads and staining with antibody. Following validation of peptide-HLA binding, primary CD8+ T cells from peripheral blood are stimulated by antigen-presenting beads twice over the course of two weeks. Frequencies of antigen-specific T cells in the resulting cells is assayed by tetramer staining. Antigen-specific T cells can be loaded onto the Berkeley Lights (BLI) Lightning platform, a novel microfluidic platform that enables thousands of single cell experiments in parallel. On the BLI Optoselect chip, IFNg secretion and CD137 upregulation of antigen-specific T cells is assayed in response to antigenic stimulation. Following analysis, single cells can be exported for further analysis.

**Results**

Berkeley Lights has developed an artificial antigen-presenting bead that expands antigen-specific T cells from peripheral blood 10 times more effectively than autologous dendritic cells. This system allows users to load peptides of choice onto magnetic beads and use them to assay peptide-HLA binding and stability, and to efficiently stimulate and expand antigen-specific T cells. Finally, in conjunction with the Berkeley Lights Lightning platform and the T cell Phenotype and Functional Analytics workflow, multiple functional parameters can be assayed from as few as 1000s of T cells, linking peptide-HLA binding and recognition to antigen-specific effector function.

#### P155 Prospective translational study evaluating vaccine-enhanced adoptive T cell therapy for treatment of osteosarcoma in companion dogs

##### Tammie Wahaus, BSBA^1^, Noe Reyes, DVM^1^, Jeffrey Bryan, DVM, MS, PhD, DACVIM-Oncology^2^, Jeffrey Bryan, DVM, MS, PhD, DACVIM-Oncology^2^, Gary Wood, PhD^3^, Brian Flesner, DVM, MS, DACVIM-Oncology^2^, Lindsay Donnelly, DVM, MS, DACVIM-Oncology^2^, Debbie Tate, RVT, VTS^2^

###### ^1^ELIAS Animal Health, Olathe, KS, United States; ^2^University of Missouri, Columbia, MO, United States; ^3^TVAX Biomedical, Olathe, KS, United States

####### **Correspondence:** Tammie Wahaus (twahaus@eliasah.com)

**Background**

Canine osteosarcoma (OSA) is an aggressively metastatic primary bone malignancy with a 90% mortality rate. Many naturally occurring canine cancers are genetically and biologically similar to their human counterparts. All cancers express neoantigens and therefore are potentially susceptible to vaccine-enhanced adoptive T cell therapy. Syngeneic rodent studies demonstrated that metastases could be permanently eliminated with vaccine-enhanced adoptive T cell therapy. Canine OSA studies provide an excellent translational bridge between experimental metastatic rodent cancer studies and metastatic human cancer clinical trials. We hypothesized that dogs with OSA could be safely treated at diagnosis with surgery, autologous cancer cell/P. acnes vaccination, adoptive T cell transfer (ACT) of ex vivo-activated T cells, and low dose human interleukin-2 (IL-2) resulting in improved survival compared to carboplatin. We further hypothesized that significant efficacy would be achieved by treating dogs with intact immune systems and minimal residual disease [1,2].

**Methods**

14 client-owned cancer bearing dogs were enrolled in a one-arm prospective trial. Dogs were staged with bloodwork, limb and thoracic radiographs, histopathology, and bone scans prior to amputation to remove the primary bone tumor. Autologous cancer cell/P. acnes vaccinations were administered intradermally weekly for three weeks. Dogs underwent leukapheresis. Mononuclear white blood cell products were stimulated ex vivo with a T cell-specific superantigen. Dogs received ACT of ex vivo-activated T cells followed by five subcutaneous IL-2 injections. Dogs were monitored for development of metastases via thoracic radiographs every three months.

**Results**

All 14 patients received autologous vaccinations. Due to early metastasis, 11 dogs received ACT. One dog did not receive adjuvant IL-2; ten dogs completed the entire protocol. Toxicity was minimal after pre-medicants (NSAID, antihistamine, and antiemetic) were instituted prior to ACT. With premedication, all toxicities were VCOG grade I/II. Median disease-free interval for all dogs was 213 days. Median survival time (MST) for all dogs was 415 days. Five dogs have survived for over 621 days and are disease-free (Figure 1.) In addition, the results included at least one dog with complete regression of distant macroscopic metastasis.

**Conclusions**

This immunotherapy protocol is safe and tolerable. Compared to MST for historical amputation alone with or without adjuvant chemotherapy (MST of 307(1) and 134(2) days, respectively), a significant survival benefit is noted in this group of patients. Further prospective studies are warranted to gain additional immunologic insight to the protocol, further improve disease response and survival, and evaluate the translational impact this treatment could have in advancing human medicine.

**References**

1. Phillips B, Powers BE, Dernell WS, Straw RC, Khanna C, Hogge GS, Vail DM. Use of single-agent carboplatin as adjuvant or neoadjuvant therapy in conjunction with amputation for appendicular osteosarcoma in dogs. J Am Anim Hosp Assoc. 2009; 45:33-8.

2. Spodnick GJ, Berg J, Rand WM, Schelling SH, Couto G, Harvey HJ, Henderson RA, MacEwen G, Mauldin N, McCaw DL, Moore AS, Morrison W, Norris AM, O’Bradovich, J, O’Keefe DA, Page R, Ruslander D, Klausner J, Straw R, Thompson JP, Withrow SJ. Prognosis for dogs with appendicular osteosarcoma treated by amputation alone: 162 cases (1978-1988). J Am Vet Med Assoc. 1992; 200(7):995-9.

**Ethics Approval**

The study was approved by University of Missouri’s IACUC, approval number 8280.

**Fig. 1 (abstract P155).**
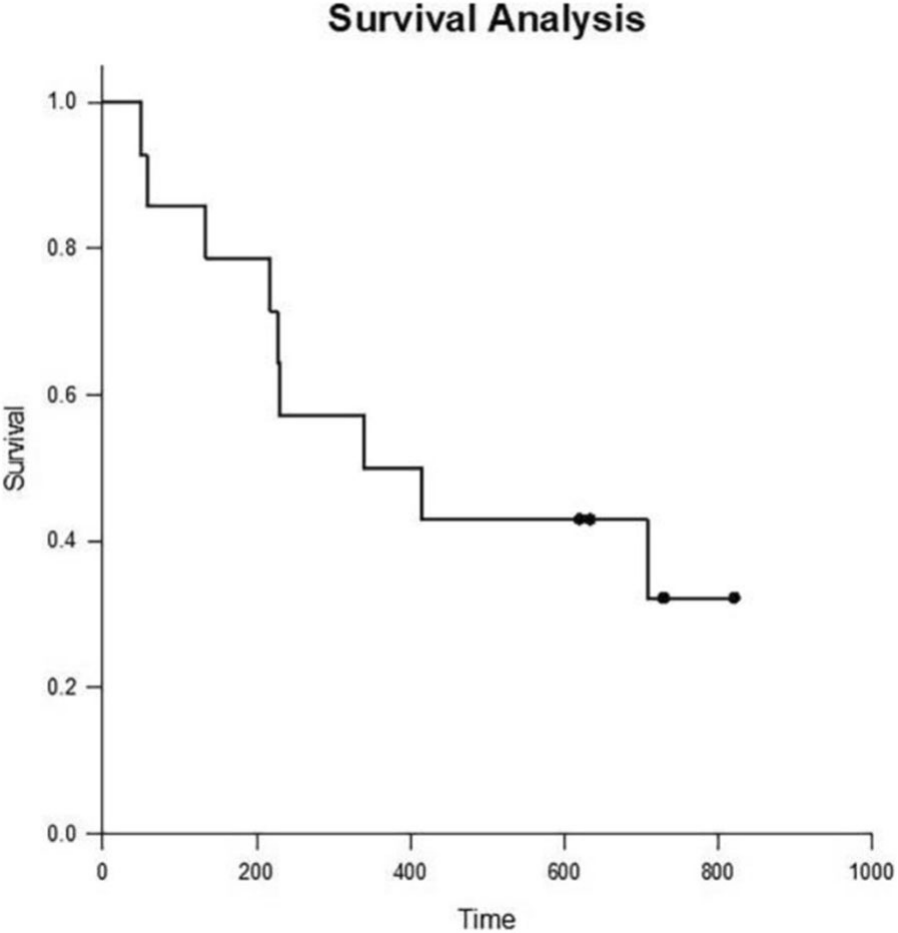
Survival Analysis of Dogs Completing Protocol

#### P156 Lung cancer sub-types exhibit differential susceptibility to natural killer cell cytotoxicity

##### Jason Cahoon, BS, Shilan Dong, MS, Rafet Amoor, Donna Sonntag, MS, Alexander Spurrell, Rachit Ohri, PhD

###### Enable Life Sciences, Worcester, MA, United States

####### **Correspondence:** Rachit Ohri (rachit@enablelifesciences.com)

**Background**

Natural Killer (NK) cells hold great promise in immunotherapy, particularly for lung cancer [1]. However, there is a paucity of literature which organizes the susceptibility of various lung cancer subtypes to NK cells. We evaluated the cytotoxicity (necrosis and apoptosis) of the NK cell line KHYG-1 (Effector) against cell-lines of 3 different subtypes of lung cancer i.e. H1975, H1703, A549 (Target). We also determined the levels of biomarkers relevant to NK cell activation and function [2] i.e. the cell-surface biomarker CD107a and 5 soluble biomarkers [Perforin, Granzyme-A, Granzyme-B, IFN-gamma and TNF-alpha].

**Methods**

Three lung cancer cell lines (H1975, H1703, A549) (ATCC, Virginia) were plated at 100% confluency in 96-well plates (1.25 cells/cm^2, 3.2*10^5 cells/mL), while K562 cells (ATCC, Virginia), used as a positive control, were suspended in the plate wells at 3.2*10^5 cells/mL. KHYG-1 cells (JCRB, Japan) were added at a density of 6.4*10^6 cells/mL, at a 20:1 Effector:Target (E:T) ratio. Cells incubated for 5 hours at 5% CO2, 37 °C. Subsequently:

[i] Cytotoxicity (necrosis and apoptosis) in target cells were quantified using flow cytometry with PerCP-Cy™5.5 Annexin V and Propidium Iodide (PI).
[ii] CD107a expression on KHYG-1 cells was evaluated using flow cytometry with PE-labeled anti-human CD107a Ab.
[iii] Expression levels of 5 soluble biomarkers (Perforin, Granzyme-A, Granzyme-B, IFN-gamma and TNF-alpha) were determined in the cell supernatants using Luminex.

**Results**

The cytotoxicity data suggests that for necrosis, all target cell-lines were significantly different (all p values < 0.0001) from each other in terms of susceptibility to the effector cells (A549 > H1703 > H1975 > K562) (Figure 1). K562 cells are significantly higher in late apoptosis than all three lung cancer cell lines (Figure 2). Amongst the 3 lung cancer cell-lines, the H1703 cell line is significantly higher in late apoptosis than H1975 and A549 cells (p-value < 0.05) (Figure 3). Although differences were seen in the necrotic and late apoptotic profiles of target cells, the CD107a expression on the KHYG-1 effector cells was similar across all co-cultures (Figure 4). The soluble biomarker data (Luminex) is being collected.

**Conclusions**

In conclusion, cell-lines corresponding to different lung cancer subtypes, i.e. A549 (Carcinoma), H1703 (Squamous Cell) and H1975 (Adenocarcinoma) exhibit significant differences in both their necrosis and late apoptosis susceptibility when co-cultured with NK cells. Such insight could be used to better guide NK cell based immunotherapy development.

**References**

1. Aktaş O, Öztürk A, Erman B, Erus S, Tanju S, Dilege S. Role of Natural Killer Cells in Lung Cancer. J Clin Cancer Res Clin Oncol. 2018; 144:997-1003.

2. Hodge G, Barnawi J, Jurisevic C, Moffat D, Holmes M, Reynolds PN, Jersmann H, Hodge S. Lung cancer is associated with decreased expression of perforin, granzyme B and interferon(IFN)-γ by infiltrating lung tissue T cells, natural killer (NK) T-like and NK cells. Clin Exp Immunol. 2014; 178:79–85.

**Fig. 1 (abstract P156).**
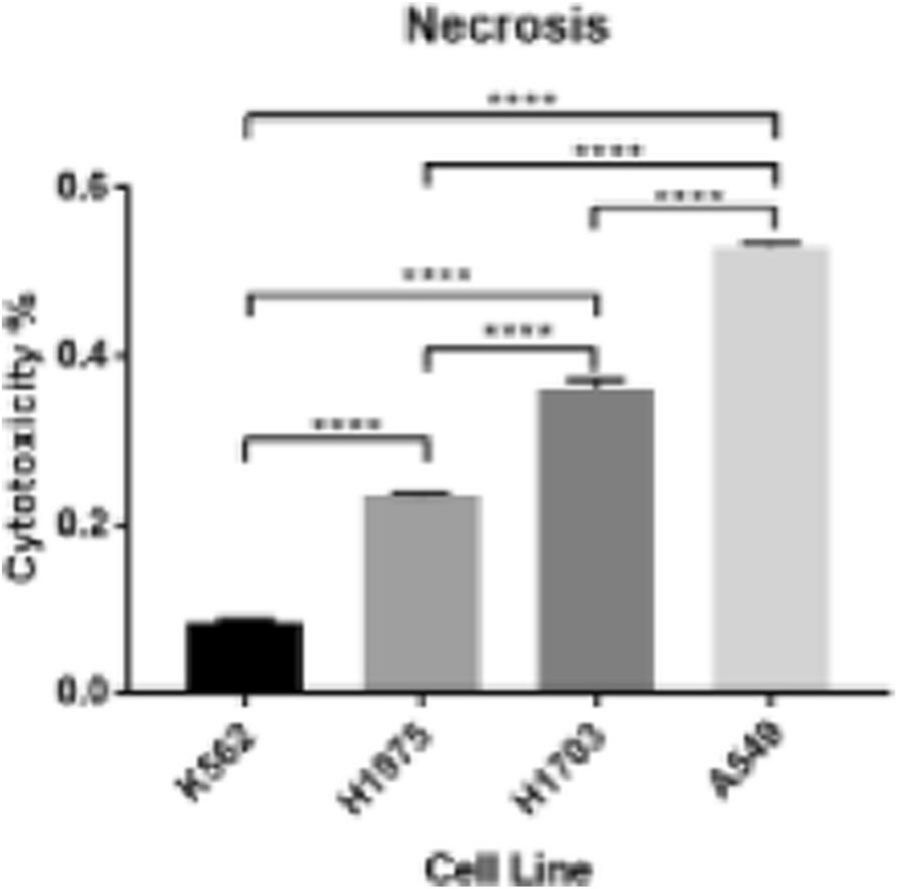
Necrosis

**Fig. 2 (abstract P156).**
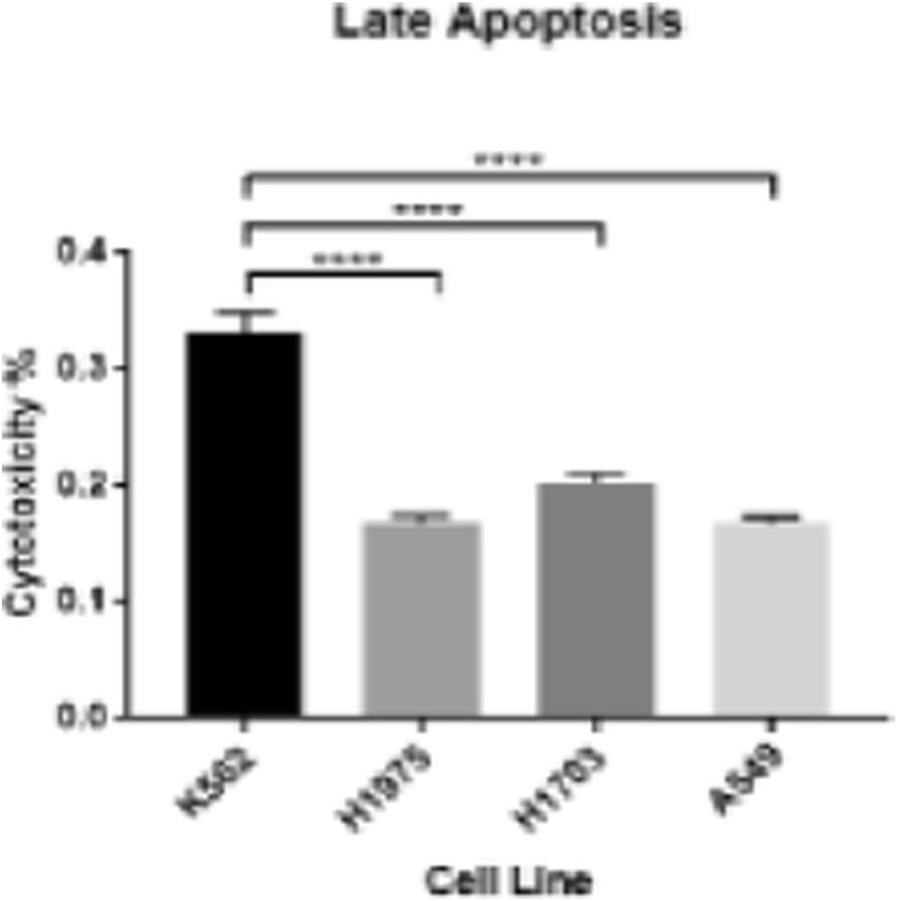
Late Apoptosis

**Fig. 3 (abstract P156).**
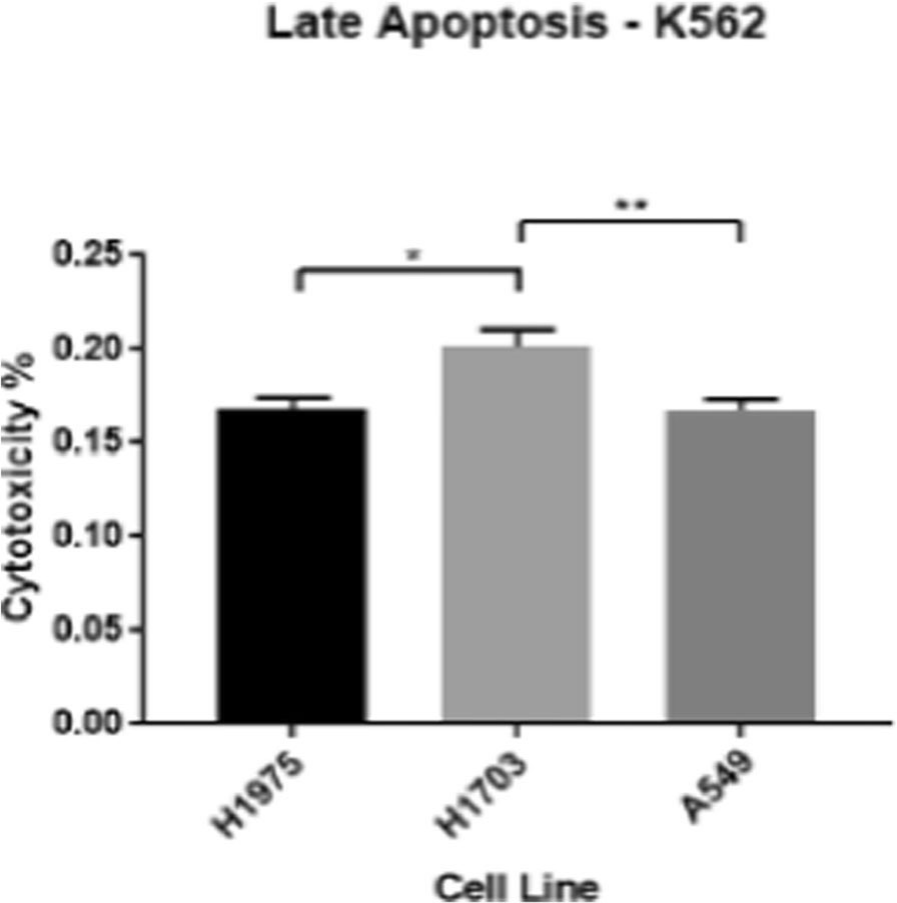
Late Apoptosis minus K562

**Fig. 4 (abstract P156).**
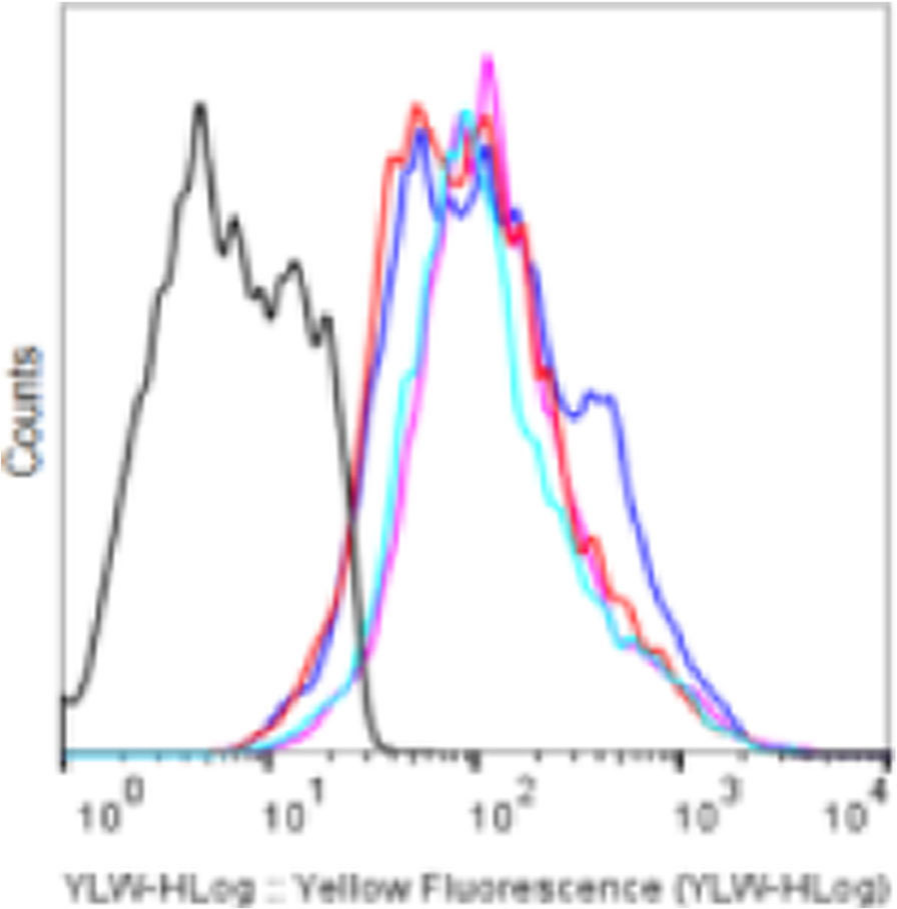
CD107a Expression

#### P157 Optimized process for manufacturing Deep-Primed™ T cells creates product with improved functional characteristics and reactivity against multiple tumor-associated antigens

##### Shawn Carey, PhD, Christine McInnis, PhD, Alicia Worthylake, Angela Forte, Elisabeth Brown, Darren Smith, Kate Sackton, PhD, Rosemary Soucy, Tap Maniar, MD, Karsten Sauer, PhD, Thomas Andresen, PhD, Andy Rakestraw, PhD

###### Torque Therapeutics, Cambridge, MA, United States

####### **Correspondence:** Thomas Andresen (tandresen@torquetx.com)

**Background**

Adoptive cell transfer (ACT) of tumor-targeted T cells has demonstrated encouraging clinical efficacy in some hematological cancers. However, in solid tumors, targeting a single antigen (e.g., CAR-T and TCR therapies) can lead to antigen escape and development of resistance. Furthermore, although support provided by lymphodepletion or cytokine administration can enhance responses to ACT, these systemic treatments are often associated with significant toxicities.

Torque’s Slipstream™ T cell manufacturing platform is a high-efficiency process for generating Deep-Primed™ T cells: polyclonal non-genetically engineered T cells that (1) are targeted against multiple tumor-specific antigens and (2) carry immunomodulating cytokine payloads to provide prolonged and locally directed immune support without systemic toxicities. The Slipstream™ process is designed to resolve the manufacturing challenge of generating high yields of early memory phenotype tumor-reactive T cells, which are associated with clinical benefit. Here, we show that the Slipstream™ process drives robust expansion while preserving favorable memory characteristics of natural tumor-reactive T cells, and we demonstrate that Deep-Priming™ T cells with Deep IL-15 or Deep IL-12 improves function.

**Methods**

Multi-targeted T cells (MTC) were comparatively generated from donors via either a first-generation process or the new Slipstream™ process that leverages ex vivo expansion conditions optimized for MTC production. T cell reactivity against tumor-associated antigens, memory, polyfunctionality, cytotoxicity, and response to Deep IL-15 and Deep IL-12 were measured. The modularity of Slipstream™ was tested by training MTC against antigen cassettes including cancer or viral antigens and measuring reactivity against antigen subsets.

**Results**

Compared to a first-generation process, MTC generated with Slipstream™ exhibited >20-fold improvement in antigen-specific reactivity and a substantial improvement in the yield of memory-phenotype antigen-specific T cells including a 10-fold increase in Tcf1-positive cells. Furthermore, the Slipstream™ process yielded MTC with increased polyfunctionality and specificity as measured by cytokine production and TCR sequencing, respectively. Notably, T cells expanded using the Slipstream™ process showed potent cytotoxicity against human cancer cells as well as responsiveness to Deep IL-15 and Deep IL-12. The Slipstream™ process can also be adapted for simultaneous training of MTC against different antigens including virus-associated tumor antigens.

**Conclusions**

The Slipstream™ process is optimized to produce Deep-Primed™ MTC with substantive increases in characteristics associated with clinical efficacy: antigen reactivity, memory phenotype, and polyfunctionality. Modularity of the Slipstream™ process has been demonstrated by simultaneously training T cell clones reactive to cancer and virus-associated antigens, and Deep-Primed™ MTC with cell-associated Deep IL-15 or Deep IL-12 drives enhanced T cell function in vitro.

#### P158 Suboptimal er stress induced autophagy regulates anti-tumor T cell response

##### Shilpak Chatterjee, Danh Tran, Kim Dosung, Satish Nadig, Carl Atkinson, Hongjun Wang, J. Alan Dieh, Shikhar Mehrotra, PhD, Paramita Chakraborty, PhD

###### Medical University of South Carolina, Charleston, SC, United States

####### **Correspondence:** Shikhar Mehrotra (mehrotr@musc.edu)

**Background**

Endoplasmic reticulum (ER) stress induced by external or internal stimuli activates a number of well-orchestrated cellular signaling processes aimed to promote either cell apoptosis or to restore cellular function and resolve the stress. In tumor microenvironment, induction of ER stress is known to dampen the antitumor activity of T cells by reducing their mitochondrial function. However, if magnitude of ER stress governs the T cell fate and function is unknown.

**Methods**

We performed our study on B16 murine melanoma model and used standard immunological techniques like flow cytometry, immunoblot analysis, microscopy, real time PCR

**Results**

Using melanoma antigen gp100 reactive T cells, we found that low level of ER stress enhances T cell stemness and promotes mitochondrial biogenesis, whereas high level of ER stress triggers T cell death. Moreover, upon adoptive transfer, T cells treated with low dose ER stress inducer are able to form long-lived memory in vivo, express reduced level of co-inhibitory molecule, and demonstrate superior anti-tumor immunity by increasing overall survival of B16 murine melanoma bearing mouse. Mechanistically, we discovered that, upon ER insult at suboptimal level, a protective autophagy pathway is induced to promote cell survival and maintain stemness through the protein kinase R-like endoplasmic reticulum kinase (PERK)/ activating transcription factor-4 (ATF4)-dependent manner. Conversely, knockdown of PERK abrogates autophagy activation, hampers mitochondrial biogenesis in response to suboptimal ER stress, which in-turn compromises the antitumor function of melanoma antigen specific T cells. Furthermore, we demonstrated that blocking autophagy in T cells hampers T cell anti-tumor activity. Lastly, T cells which initiates autophagic process due to suboptimal ER stress show better potential to control tumor compared to those, that do not enter into the process

**Conclusions**

Overall, these preclinical data highlights that, low level of ER stress response is important for healthy cellular function and therapeutically, ER stress pathways can be manipulated in T cells in order to regulate their antitumor potential.

**Ethics Approval**

The study was approved by Medical University of South Carolina‘s Ethics Board, approval number 2018-00628.

#### P159 TCR fingerprinting and off-target peptide identification

##### Armen Karapetyan, Chawaree Chaipan, Katharina Winkelbach, Sandra Wimberger, Jun Seop Jeong, Bishnu Joshi, Robert Stein, MD PhD, Dennis Underwood, PhD, Eleni Chantzoura, PhD, Alvaro Yague, Jan Bergmann, John Castle, PhD, Marc Van Dijk, PhD, Volker Seibert

###### Agenus Inc, Lexington, MA, United States

####### **Correspondence:** John Castle (john.castle@agenusbio.com); Marc Van Dijk (marc.vandijk@agentustherapeutics.com)

**Background**

Adoptive T cell therapy using patient T cells redirected to recognize tumor-specific antigens by expressing genetically engineered high-affinity T-cell receptors (TCRs) has therapeutic potential for melanoma and other solid tumors. Clinical trials implementing genetically modified TCRs in melanoma patients have raised concerns regarding off-target toxicities resulting in lethal destruction of healthy tissue, highlighting the urgency of assessing which off-target peptides can be recognized by a TCR.

**Methods**

As a model system we used the clinically efficacious NY-ESO-1-specific TCR C259, which recognizes the peptide epitope SLLMWITQC presented by HLA-A*02:01. We investigated which amino acids at each position enable a TCR interaction by sequentially replacing every amino acid position outside of anchor positions 2 and 9 with all 19 possible alternative amino acids, resulting in 134 peptides (133 altered peptides plus epitope peptide). Each peptide was individually evaluated using three different in vitro assays: binding of the NY-ESO C259 TCR to the peptide, peptide-dependent activation of TCR-expressing cells, and killing of peptide-presenting target cells. To represent the TCR recognition kernel, we defined Position Weight Matrices (PWMs) for each assay by assigning normalized measurements to each of the 20 amino acids in each position. To predict potential off-target peptides, we applied a novel algorithm projecting the PWM-defined kernel into the human proteome, scoring NY-ESOc259 TCR recognition of 336,921 predicted human HLA-A*02:01 binding 9-mer peptides.

**Results**

Of the 12 peptides with high predicted score, we confirmed 7 (including NY-ESO-1 antigen SLLMWITQC) strongly activate human primary NY-ESO C259-expressing T cells. These off-target peptides include peptides with up to 7 amino acid changes (of 9 possible), which could not be predicted using the recognition motif as determined by alanine scans.

**Conclusions**

Thus, this replacement scan assay determines the “TCR fingerprint” and, when coupled with the algorithm applied to the database of human 9-mer peptides binding to HLA-A*02:01, enables identification of potential off-target antigens and the tissues where they are expressed. This platform enables both screening of multiple TCRs to identify the best candidate for clinical development and identification of TCR-specific cross-reactive peptide recognition and constitutes an improved methodology for the identification of potential off-target peptides presented on MHC class I molecules. We used this platform and demonstrate screening of multiple TCRs targeting tumor antigens.

#### P160 Engineered natural killer cells redirected against adenosinergic immunometabolic suppression for the immunotherapy of lung carcinoma

##### Andrea Chambers, MS, Kyle Lupo, BS, Jiao Wang, PhD, Sandro Matosevic, PhD

###### Purdue University, Lafayette, IN, United States

####### **Correspondence:** Sandro Matosevic (sandro@purdue.edu)

**Background**

NK cells are powerful effectors in cancer immunotherapy and have potential to treat various cancers; however significant challenges remain in the treatment of solid tumors. Energy availability is compromised surrounding solid tumors and NK cell metabolic reprogramming can occur to inhibit NK effector functions [1]. Accumulation of adenosine in the tumor microenvironment (TME) from the activity of ectoenzymes CD39 and CD73 on cancer cells is one mechanism that leads to impaired NK cell function. Our previously published data has established that the effects of TME adenosine on NK cells cause specific reorganization of the cells’ metabolism and effector signatures to suppress NK cell function, and the cytokine combination of IL-12/15 was hyperresponsive to adenosine [2]. One way to combat immunosuppression induced by cancer-produced adenosine is to engineer NK cells to overcome this inhibition. To that end, we engineered NK cells to directly target CD73 by imparting NK-specific signaling to enhance anti-tumor activity against CD73+ lung carcinoma.

**Methods**

Peripheral blood-derived NK cells were isolated from healthy human donors and expanded using feeder cells. NK cells were electroporated using mRNA or transduced with lentivirus expressing the CD73-targeting construct which bears signaling domains derived from FcγRIIIa. Engineered NK cells expressing the construct were tested for their killing ability against lung carcinoma A549 cells. The engineered NK cells were then adoptively transferred into a CD73+ lung cancer xenograft into NSG mice. Circulating CD73-CAR NK cells were quantified for their expression of activating markers NKG2D, DNAM, and NKp30 and visualized using immunohistochemistry to determine infiltration into tumors, and mice were assessed for tumor growth.

**Results**

We showed NK cells can be efficiently redirected against CD73 to block the generation of immunosuppressive adenosine and rescue impaired NK cell anti-tumor immunity. Specifically, primary human NK cells were successfully engineered to express the synthetic CD73-FCyRIIIa construct. Retargeted NK cells showed enhanced anti-tumor functions in vitro against CD73-expressing A549 cells. Engineered primary NK cells also showed promise in stunting CD73+ lung cancer tumor growth for up to 3 weeks in vivo. Current and future studies include evaluation of off target effects and local injection to further evaluate infiltration of the NK cells in vivo.

**Conclusions**

The microenvironment of solid tumors is highly immunosuppressive and adenosine has been shown to impair NK cell anti-tumor immunity. A novel anti-CD73 targeting construct using NK cell signaling components has been developed and shown to prevent tumor growth of CD73+ lung carcinoma.

**References**

1. Chambers A *, Lupo K*, Matosevic S. Tumor-microenvironment-induced immunometabolic reprogramming of natural killer cells. Front Immunol. 2018; 9:2517.

2. Chambers A, Wang J, Lupo K, and Matosevic S. Adenosinergic signaling alters natural killer cell functional responses. Front Immunol, 2018; 9:2533.

**Ethics Approval**

The study was approved by Purdue University Institution's Review Board, approval number 1804020540.

#### P161 High-efficiency CAR-T cell manufacturing by improved scalable electroporation

##### Jian Chen, PhD, George Sun

###### Celetrix LLC, Manassas, VA, United States

####### **Correspondence:** Jian Chen (jchen@celetrix.com)

**Background**

CAR-T cells are currently manufactured for clinical use by infection of human T cells with viral vectors containing the CAR gene. The current viral CAR-T manufacturing process is lengthy and costly and electroporation has emerged as a promising alternative. However, clinical use of electroporation technology in CAR-T has been difficult and several clinical trials have met significant problems due to the low transfection efficiency and/or high cell mortality.

**Methods**

Our novel understanding of the electroporation mechanism revealed that the current widely-used electroporation methods have significant mistakes in the physical design as well as electroporation buffer design. The first problem is the electroporation sample container design. It is well known that electrochemical reaction generates gas bubbles that are harmful to the cells and there was no good solution to the problem. Here we used a novel pressurization approach to largely eliminate the effect.

**Results**

Combined with other improvements including electroporation buffer design and post-electroporation cell culture strategy, we have been able to achieve over 80% plasmid transfection efficiency in unstimulated T cells and over 90% plasmid transfection efficiency in stimulated T cells. The viability in survived cells is over 95% measured by live/dead staining and the true survival rate measured by survived cell number is over 66%. The new electroporation method can achieve over 90% in gene editing and the method is also widely applicable in electroporation of NK cells, DC cells and monocytes.

**Conclusions**

The new method is also scalable as billions of cells can be processed in the large volume electroporation setting. Our method can potentially eliminate the need for expensive cell expansion and virus production altogether, therefore cutting the huge economic burden of CAR-T therapy.

#### P162 Development of CD4+ and CD8+ TCRαβ-deficient bioluminescent reporter T cells for screening and characterization of neoantigen-specific TCRs

##### Zhi-jie Cheng, PhD, Jamison Grailer, PhD, Michael Slater, Pete Stecha, Jim Hartnett, Frank Fan, PhD, Mei Cong, PhD

###### Promega Corporation, Madison, WI, United States

####### **Correspondence:** Zhi-jie Cheng (jey.cheng@promega.com)

**Background**

Adoptive cancer antigen-specific T cell therapy currently comprised of chimeric antigen receptor (CAR-) and T cell receptor (TCR) engineered T cells. Clinical results from CAR-T cells have demonstrated promising results in treating leukemia, while TCR-engineered T cells which have the advantage of recognizing intracellular tumor antigens is still in very early development.

**Methods**

Here, we report the development of two CD4+ or CD8+ TCRαβ-KO reporter T cell lines for the screening and characterization of transgenic TCRs. A TCRαβ-KO reporter T cell line was first developed by knocking out the endogenous TCR α and β chains in the reporter T cell line using CRISPR/Cas9 and the successful knockout is confirmed by phenotypic assays and TCR v chain locus sequencing.

**Results**

We demonstrated that re-introduction of HA peptide-specific HA1.7 TCR α and β chains into TCRαβ-KO reporter T cell lines results in HA peptide-dependent TCR activation and luciferase reporter expression when HA peptide is presented by a MHCII+ cell line. Furthermore, the select expression of CD4 or CD8 variants in the TCRαβ-KO reporter T cell line could enable the development of TCRs for both MHCI- and MHCII-restricted tumor antigen targets.

**Conclusions**

The CD4+ and CD8+ TCRαβ-deficient reporter T cells can serve as valuable tools for screening and characterization of neoantigen-specific TCRs

#### P163 Effect of common gamma-chain cytokines on myeloid-derived suppressor cell and M2 macrophage suppressive function: Implications for cellular immunotherapy

##### Anna Cole, BA^1^, Charlotte Rivas^2^, Josue Pineda^2^, Corrine Baumgartner^2^, Stephanie Fetzko^2^, Robin Parihar^2^

###### ^1^Rice University, Houston, TX, United States; ^2^Baylor College of Medicine, Houston, TX, United States

####### **Correspondence:** Robin Parihar (rxpariha@texaschildrens.org)

**Background**

Immunotherapy using antigen-redirected lymphocytes such as chimeric antigen receptor (CAR)-T or -NK cells in patients with solid tumors has shown poor efficacy. Cell therapies are hindered by immunosuppressive cells such as inhibitory macrophages (M2s) and myeloid-derived suppressor cells (MDSCs) that contribute to a highly suppressive tumor microenvironment (TME) [1]. Researchers have armed redirected lymphocytes with the ability to secrete cytokines in hopes of promoting their proliferation and function in suppressive TMEs [2,3]. However, the effect of these cytokines on other immune cells within the TME, such as MDSCs and M2s, is unknown.

**Methods**

To determine how the human common gamma-chain cytokines, interleukin(IL)-2, IL-7, IL-15, and IL-21 affect human MDSCs and M2s, we exposed ex vivo enriched M2s and MDSCs to each cytokine separately and assessed changes in MDSC and M2 phenotype and ability to dampen T-cell activation and proliferation. To further define cytokine-induced changes in MDSC/M2 function in a more clinically relevant system, we tested the ability of cytokine-exposed MDSCs/M2s to impair CAR-T cell proliferation and anti-tumor activity in a TME co-culture. As a clinical correlate, we assessed common gamma-chain cytokine receptor expression on MDSCs and M2s within neuroblastoma and sarcoma patient tumors and tested the effects of cytokine exposure on their suppressive capacity.

**Results**

Subsets of ex vivo enriched M2s and MDSCs expressed common gamma-chain cytokine receptors. MDSCs expressed receptors for IL-2 (22%, avg. MFI=67, n=3), IL-7 (43%, avg. MFI=375, n=3), IL-15 (23%, avg. MFI=310, n=3), and IL-21 (65%, avg. MFI=124, n=4); whereas M2s expressed receptors for IL-2 (17%, avg. MFI=59), IL-7 (98%, avg. MFI=543), IL-15 (36%, avg. MFI=619), and IL-21 (91%, avg. MFI=296). Exposure of human MDSCs or M2s to IL-2, IL-7, IL-15, or IL-21 did not alter their cell-surface phenotype. Exposure of these suppressive myeloid cells to IL-2, IL-7, and IL-15 did not change their ability to suppress T-cell proliferation. In contrast, exposure of M2s and MDSCs to IL-21 increased their ability to suppress T-cell proliferation and activation (98% suppression by IL-21 exposed MDSCs vs. 72% suppression by control MDSCs; 98% suppression by IL-21 exposed M2 vs. 79% suppression by control M2 at a 2:1 T cell:MDSC/M2 ratio).

**Conclusions**

These results suggest that IL-21 increases the suppressive capacity of human MDSCs and M2s. Ongoing experiments will define the mechanisms by which IL-21 alters MDSC and M2 suppression and further define the effect of IL-21 exposed MDSCs and M2s on tumor growth and CAR-T cell therapeutic efficacy in vivo.

**References**

1. Martinez M, Moon EK. CAR T cells for solid tumors: New strategies for finding, infiltrating, and surviving in the tumor microenvironment. Front. Immunol. 2019; 10:128.

2. Yeku OO, Purdon TJ, Koneru M, Spriggs D, Brentjens RJ. Armored CAR T cells enhance antitumor efficacy and overcome the tumor microenvironment. Sci Rep. 2017; 7:10541.

3. Liu D, Song L, Wei J, Courtney AN, Gao X, Marinova E, Guo L, Heczey A, Asgharzadeh S, Kim E, et al. IL-15 protects NKT cells from inhibition by tumor-associated macrophages and enhances antimetastatic activity. J Clin Invest. 2012; 122:2221-2233.

**Ethics Approval**

Tumor tissue use was approved by Baylor College of Medicine IRB study protocol #26691, and samples were de-identified prior to laboratory evaluation.

#### P164 Effect of NK cell treatment on PD-L1 expression and anti-PD-L1 response

##### Alicja Copik, PhD, Jeremiah Oyer, Sarah Gitto, Deborah Altomare, PhD

###### University of Central Florida, Orlando, FL, United States

####### **Correspondence:** Deborah Altomare (deborah.altomare@ucf.edu)

**Background**

PD-1 axis blockade therapies have shown success but responses are limited to ~15% of cancer patients. These responses correlate with presence of lymphocyte infiltrated, PD-L1 positive tumors. Strategies that increase PD-L1 expression may improve outcomes of PD-1 axis blockade. PD-L1 on tumor cells is induced by IFNγ, secreted by NK cells. We developed a method for producing therapeutic quantities (>1,000 fold expansion within two weeks) of hyper-activated NK cells with high anti-tumor cytotoxicity and enhanced IFNγ secretion. The utilizes particles from Plasma Membrane of K562 cells expressing membrane bound IL21 (PM21-particles). Herein, the ability of PM21-particle expanded NK cells to induce PD-L1 expression on various tumors was tested in vitro and in vivo in ovarian cancer model. Furthermore, the effect of anti-PD-L1 on NK cell anti-tumor activity was tested in vitro and in vivo.

**Methods**

NK cells were expanded with PM21-particles as described. For in vivo experiments, NSG mice were implanted with 1x10^6 SKOV-3 cells i.p.. Mice were treated with 10^7 PM21-NK cells (n=6) or with vehicle control (n=6) on days 8 and 13. Mice were sacrificed on day 20 to collect tumors. Tumors were perfused and retrieved tumor cells were analyzed for PD-L1 expression while infiltrating immune cells were phenotyped.

**Results**

PM21-NK treatment induced PD-L1 on >30% of tumor cells across multiple cell lines. PM21-NK cells are negative for PD-1 and addition of anti-PD-L1 had no effect on their cytotoxicity or cytokine production. In in vivo experiment, PM21-NK cell treated mice had increased PD-L1+ tumors vs. the untreated group (29.7% vs 14.5%, p<0.0001). Despite T-cell depletion, T-cells made up ~22% of hCD45+ events in perfused tumors, 83% of which were Tregs. PM21-NK cells are PD-1-, but are inhibited by Tregs. Untreated and anti-PD-L1 alone mice had median survival of 24 days. Treatment with PM21-NK cells improved survival over untreated (p=0.0003) and PD-L1 alone (p=0.0002) groups having median survival of 40 days. Combination of PM21-NK cells with anti-PD-L1 further improved of survival over the PM21-NK cells alone group (48 days, p=0.042) with 25% of mice still remaining in good health at day 58.

**Conclusions**

These data support the use of anti-PD-L1 in NK cell therapy, regardless of initial tumor PD-L1 status. PM21-NK cells can be used for tumor treatment and to prime tumors to express PD-L1. The PD-L1 induced upon NK cell treatment can serve as “universal targetable ligand” if used with humanized anti-PD-L1 antibodies to cause tumor killing by ADCC.

#### P165 Robust, reproducible and highly scalable manufacturing of P-BCMA-ALLO1, an allogeneic CAR-T stem cell memory product for multiple myeloma, from numerous healthy donors

##### Stacey Cranert, PhD, Maximilian Richter, PhD, Min Tong, MS, Leslie Weiss, MS, Yening Tan, MS, Eric Ostertag, MD, PhD, Julia Coronella, PhD, Devon Shedlock, PhD

###### Poseida Therapeutics, San Diego, CA, United States

####### **Correspondence:** Devon Shedlock (dshedlock@poseida.com)

**Background**

Autologous Chimeric Antigen Receptor (CAR) T cell therapy for relapsed/refractory Multiple Myeloma (MM), such as Poseida’s anti-B cell maturation antigen (BCMA) product candidate, P-BCMA-101, have shown significant efficacy in the clinic. P-BCMA-101 is comprised of a high percentage of stem cell memory T cells (TSCM), resulting in a product that is much safer and potentially more durable than other anti-BCMA autologous product candidates. However, individualized products have expensive and time-consuming manufacturing and significant variability in input patient T cells characteristics. We are developing P-BCMA-ALLO1, an off-the-shelf anti-BCMA allogeneic (allo) CAR-T product candidate manufactured from serial healthy donor material that circumvents many of the downsides of an individualized CAR-T product.

**Methods**

P-BCMA-ALLO1 is produced using two key platform technologies: the nonviral piggyBac® (PB) DNA Modification System and the high-fidelity Cas-CLOVER™ (CC) Site-Specific Gene Editing System. The PB transposase mRNA and DNA encoding the PB-based transgene are electroporated along with the components of the CC system needed to knockout (KO) the T Cell Receptor (TCR) and beta-2 microglobulin, thereby eliminating expression of Major Histocompatibility Complex (MHC) class I. The T cells are then expanded using our proprietary "booster molecule.” The resulting product demonstrates expression of the transgene in nearly all cells, and after a purification step, have eliminated all TCR expression and most MHC class I expression.

**Results**

We have produced P-BCMA-ALLO1 at both research and near-clinical scale from >35 donors with >97% manufacturing success. Efficiencies of TCR-KO ranged from ~50-90%, with final product always demonstrating >99% TCR-KO. T cell expansion varied from ~0.5-20 fold. At clinical production scale, this translates to up to 250 doses of CAR-T per manufacturing run at a dose of 150x10e6 cells/patient. P-BCMA-ALLO1 demonstrated a high-percentage of TSCM cells (CD45RA+CD62L+CD45RO-). Furthermore, P-BCMA-ALLO1 generated from multiple donors demonstrated potent efficacy in the RPMI-8226 xenograft model in NSG mice, thus establishing the feasibility of using serial individual donors in our manufacturing process.

**Conclusions**

In summary, these data demonstrate a robust, reproducible and highly scalable manufacturing process. Moreover, this production process can be expanded for use with additional targets for treatment of other heme or solid tumors.

#### P166 ET140202 T-cell therapy for the treatment of liver cancer is built upon a novel antibody-T cell receptor (AbTCR) ARTEMIS™ T-cell platform

##### Jun Cui, PhD, Pengbo Zhang, Hongruo Yun, Yiyang Xu, Lucas Horan, PhD, Shaohua Xu, Sean Xu, Hong Liu

###### Eureka Therapeutics, Inc., Emeryville, CA, United States

####### **Correspondence:** Hong Liu (hong.liu@eurekainc.com)

**Background**

The use of engineered T cells for the treatment of solid cancers remains challenging. Recently, we developed a novel antibody-T cell receptor (AbTCR) ARTEMIS™ T-cell platform, which combines antibody-based target recognition with gamma/delta TCR-based cellular activation [1]. In contrast to chimeric antigen receptors (CARs), the AbTCR forms a natural multimeric receptor with the endogenous CD3 complex, which feeds into a network of signaling pathways that regulate T-cell activation. In addition, the inclusion of gamma/delta TCR chains within the AbTCR avoids the formation of mispaired receptors with unknown cross-reactivity, which is a potential risk associated with current alpha/beta TCR-based therapies.

Using the core design of the AbTCR ARTEMIS™ T-cell platform, we developed ET140202 for the treatment of hepatocellular carcinoma (HCC). ET140202 features an AbTCR targeting alpha-fetoprotein (AFP)peptide/MHC complexes (specifically AFP158-166/HLA-A2) expressed on HCC cancer cells. To optimize T-cell activation and expansion, the AbTCR is co-expressed with a CD28-based co-stimulatory molecule engineered to target Glypican 3 (GPC3) expressed on HCC cancer cells.

**Methods**

To test the specificity and potency of ET140202 T cells in vitro, ET140202 T cells were co-incubated with either target-positive or target-negative cells. Lactate Dehydrogenase release was used to quantify target cell lysis. CFSE assay was used to measure cell proliferation. Expression of differentiation and exhaustion markers were determined by flow cytometry. The in vivo anti-tumor activity of ET140202 T cells was tested in an AFP+/HLA-A2+ Hep G2 liver cancer xenograft model. We also engineered the same anti-AFP158-166/HLA-A2 binding moiety onto a CD28-based CAR (AFP-CAR) and compared AFP-CAR-T cells to ET140202 T cells in various assays.

**Results**

ET140202 T cells specifically lysed AFP-positive tumor cells. Compared to AFP-CAR-T cells, ET140202 T cells displayed enhanced in vitro cell killing and proliferation even after repetitive antigen stimulations. ET140202 T cells also display a less exhausted surface phenotype (e.g. lower PD-1 expression) and a higher percentage of central memory T cells (CCR7+ CD45RA-) after antigen stimulation. In vivo, both intravenous and intratumoral single administration of ET140202 T cells led to significant tumor growth inhibition.

**Conclusions**

ET140202 is built upon our novel AbTCR ARTEMIS™ T-cell platform, which was designed to harness the natural biology of T cells to fight cancer. Both in vitro cellular assays and in vivo mouse studies support the safety and efficacy of ET140202 T cells. Whether these pre-clinical findings for AbTCR-based ET140202 T-cell therapy translate into the clinical setting is currently being tested (clinicaltrial.gov, NCT0399803).

**Reference**

1. Xu Y, Yang Z, Horan LH, Zhang P, Liu L, Zimdahl B, Green S, Lu J, Morales JF, Barrett DM et al. A novel antibody-TCR (AbTCR) platform combines Fab-based antigen recognition with gamma/delta-TCR signaling to facilitate T-cell cytotoxicity with low cytokine release. Cell Discovery 2018, 4(1):62.

**Ethics Approval**

All animal experiments were conducted according to protocols approved by their Institutional Animal Care and Use Committee (IACUC) and in accordance with the Guide for the Care and Use of Laboratory Animals (National Research Council, National Academy Press, Washington, DC, 1996) and the Policy on Humane Care and Use of Laboratory Animals (Department of Health and Human Services, Bethesda, MD).

#### P167 Improved efficacy leveraging CAR-T therapeutics that produce tightly controlled, tumor proximal, immunomodulatory outputs

##### Michon Pinnix, Krista McNally, Jay Danao, BS, Melissa Fardy, Rachel Hovde, MS, Charlotte Davis, Nicole Grant, Dianna Lester-Zeiner, David Mai, Ben Wang, PhD, Gus Zeiner, PhD

###### Chimera Bioengineering, Emeryville, CA, United States

####### **Correspondence:** Gus ZeinerGus Zeiner (gus@chimera.bio)

**Background**

Current chimeric antigen receptor (CAR) T cell therapies are clinically efficacious against several B cell malignancies, but are less effective at eliminating solid tumors. A key contributor to this observed lack of efficacy is the tumor microenvironment (TME) that is erected by solid tumors to impose immunosuppressive physical and chemical barriers to T cell function and survival. To break TME-driven immunosuppression, attempts have been made to arm CAR-T cells with the ability to produce immunomodulatory payloads that target features of the TME. By their nature, these immunomodulators (e.g. Interleukin 12) are frequently toxic when systemically delivered. Due to a limited repertoire of existing programmable gene regulators, constitutive expression and systemic distribution of immunomodulators by armed CAR-T cells is common.

Chimera Bioengineering has characterized a novel, post-transcriptional gene regulatory node that strictly governs effector outputs in human T cells. This gene regulatory node, termed Gold, has been used to create enhanced CAR-T therapeutics that produce tightly controlled immunomodulatory outputs only upon tumor engagement.

**Methods**

Methods include: design and characterization of a proprietary bicistronic GoldCAR lentiviral vector to simultaneously deliver both CAR and IL12 transgenes; production of lentivirus and infection of primary human T cells; in vitro characterization of GoldCAR-T cell function utilizing cell based assays, immunoassays and flow cytometry; implantation of subcutaneous xenograft tumors in NSG mice with tumor growth monitored by caliper and bioluminescent imaging (BLI) to assess in vivo efficacy; and ex vivo analysis of blood, tumor and lymphatic organs to characterize safety profile.

**Results**

CD19 targeted GoldCAR-T cells delivering IL12 demonstrate improved efficacy over standard CD19 CAR-T cells in a subcutaneous Daudi B cell xenograft mouse model. These GoldCAR-T cells exhibit comparable efficacy to CAR-T cells with constitutive IL12 expression, but levels of pro-inflammatory cytokines in the peripheral blood are lower in the mice treated with GoldCAR-T cells.

**Conclusions**

GoldCAR-T cells with tumor proximal IL12 delivery demonstrate enhanced efficacy over standard CAR-T cells and an improved safety profile compared to CAR-T cells with constitutive IL12 expression. The implications of this study point to an amplified CAR-T cell response in an immunosuppressive tumor microenvironment.

**Trial Registration**

Not applicable

**Ethics Approval**

The animal study was conducted under the Institutional Animal Care and Use Committee (IACUC) of LumiGenics, LLC, 750 Alfred Nobel Drive, Suite 103, Hercules CA 94547

#### P168 Role and function of T-cell immunoglobulin– and mucin domain–containing (TIM)–3 receptor on natural killer cells in solid tumors

##### Tram Dao, Sandro Matosevic, PhD

###### Purdue University College of Pharmacy, West Lafayette, IN, United States

####### **Correspondence:** Sandro Matosevic (smatosev@purdue.edu)

**Background**

Natural killer (NK) cells are part of the innate immune system, but are capable of participating in both innate and adaptive immune responses due to their wide range of cytolytic activities, from degranulation, secretion of cytokines to antibody-dependent cell-mediated cytotoxicity. These are possible due to the cells’ ability to recognize self and non-self-entities via the net signal generated from their activating and inhibitory receptors upon engagement. One such receptor is TIM-3, which is expressed on various lymphocytes. In T-cells, TIM-3 is an exhaustion marker [1], but on NK cells, results are conflicting in regards to its function as the receptor exhibits both activating and inhibitory effects depending on disease type and activation status [2-6].

**Methods**

NK cells were isolated from peripheral blood of healthy donors. After expansion, they were co-cultured for 4 hours with glioblastoma (U87) at effector:target (E:T) ratios of 2.5:1 and 10:1, and various receptors were screened by flow cytometry, including PD-1, NKG2A, LAG-3, CD158b, CEAMCAM-1 and TIM-3. Then, expression of TIM-3 was measured when in the presence of patient-derived primary glioblastoma cells (GBM43) and prostate cancer (PC3) for 4 hours. To determine the effect of TIM-3 expression, killing assay are being carried out by blocking TIM-3 on NK cells. Further investigation is being performed by blocking one of TIM-3’s primary ligands, Galectin-9, on cancer cells to determine its impact on NK cell cytotoxicity. Statistical analyses are completed in SAS JMP Pro 14.

**Results**

We found that TIM-3 is significantly downregulated on primary human NK cells, in both frequency and surface density, when exposed to solid tumor cells such as U87, GBM43 and PC3 at multiple E:T ratios. Unlike other inhibitory NK receptors, this downregulation was unique to TIM-3. However, it is not known why the downregulation occurs with solid tumors, and whether this change in expression affects NK killing capacity. Here, we report the role of TIM-3 on NK cell cytotoxicity against solid tumor cell lines and the role of Galectin-9 in mediating NK cell activity.

**Conclusions**

We found that Tim-3 was significantly downregulated on NK cells in response to solid tumor cells. Understanding the complex roles of Tim-3 expression on NK cells allows us to better understand the nuanced immunomodulatory role of Tim-3 on NK cell anti-tumor responses, and provide a basis for the development of immunotherapies targeting impaired NK cell function in solid tumors

**References**

1. Sánchez-Fueyo A, Tian J, Picarella D, Domenig C, Zheng XX, Sabatos CA, Manlongat N, Bender O, Kamradt T, Kuchroo VK, Gutiérrez-Ramos JC, Coyle AJ, Strom TB. Tim-3 inhibits T helper type 1-mediated auto- and alloimmune responses and promotes immunological tolerance. Nat Immunol. 2003; 4:1093-101.

2. Ndhlovu LC, Lopez-Vergès S, Barbour JD, Jones RB, Jha AR, Long BR, Schoeffler EC, Fujita T, Nixon DF, Lanier LL. Tim-3 marks human natural killer cell maturation and suppresses cell-mediated cytotoxicity. Blood. 2012; 119:3734-43.

3. Jost S, Moreno-Nieves UY, Garcia-Beltran WF, Rands K, Reardon J, Toth I, Piechocka-Trocha A, Altfeld M, Addo MM. Dysregulated Tim-3 expression on natural killer cells is associated with increased Galectin-9 levels in HIV-1 infection. Retrovirology. 2013; 10:74.

4. Gleason MK1, Lenvik TR, McCullar V, Felices M, O'Brien MS, Cooley SA, Verneris MR, Cichocki F, Holman CJ, Panoskaltsis-Mortari A, Niki T, Hirashima M, Blazar BR, Miller JS. Tim-3 is an inducible human natural killer cell receptor that enhances interferon gamma production in response to galectin-9. Blood. 2012; 119:3064-72.

5. Ju Y, Hou N, Meng J, Wang X, Zhang X, Zhao D, Liu Y, Zhu F, Zhang L, Sun W, Liang X, Gao L, Ma C. T cell immunoglobulin- and mucin-domain-containing molecule-3 (Tim-3) mediates natural killer cell suppression in chronic hepatitis B. J Heptatol. 2010; 52:322-9.

6. da Silva IP, Gallois A, Jimenez-Baranda S, Khan S, Anderson AC, Kuchroo VK, Osman I, Bhardwaj N. Reversal of NK-cell exhaustion in advanced melanoma by Tim-3 blockade. Cancer Immunol Res. 2014; 2:410-22.

**Ethics Approval**

This study was approved by Purdue Intuition’s Ethics Board, approval number 1804020540.

#### P169 Enriching non-genetically modified antigen specific marrow infiltrating lymphocytes (MILs) to target HPV+ oropharyngeal squamous cell carcinoma

##### Danielle Dillard^1^, Vanessa Chan^1^, Lakshmi Rudraraju, MS^2^, Elizabeth DeOliveira^2^, Amy Thomas^1^, Ervin Griffin^1^, Megan Heimann^1^, Luca Biavati, MD^1^, Elizabeth Zawidzka^1^, Marguerrita El Asmar^1^, Drew Pardoll, MD, PhD^1^, Kellie Smith, PhD^1^, Carole Fakhry, MD^3^, Ivan Borrello, MD^1^

###### ^1^Johns Hopkins School of Medicine, Baltimore, MD, United States; ^2^WindMIL therapeutics, Baltimore, MD, United States; ^3^Otolaryngology, Head and Neck Surgery, Baltimore, MD, United States

####### **Correspondence:** Danielle Dillard (danielle.dillard@jhmi.edu)

**Background**

Human papillomavirus positive (HPV+) oropharyngeal squamous cell carcinoma (HPV-OPSCC) accounts for ~80% of OPSCCs in the United States. Although HPV-positivity confers improved survival relative to HPV(-) OPSCC, outcomes for metastatic HPV-OPSCC remain dismal. High tumor infiltrating lymphocytes (TILs) are associated with better outcomes in HPV-OPSCC and have the promise of a therapeutic role based upon their intrinsic enhanced tumor specificity. However, not all tumors possess TILs. To date, therapeutic expansions involve surgical excision of the tumor, expansion of TILs with high-dose IL-2 for up to 4 weeks and clinical product success rate between 40-60%. Marrow infiltrating lymphocytes (MILs) represent a novel and distinct T cell population obtained from the bone marrow (BM) of patients that possess significant tumor-specificity over peripheral blood lymphocytes (PBLs). Although the early work was done in myeloma, the unique nature of the BM microenvironment makes it a reservoir of antigen-experienced memory T cells in numerous solid tumors. As such, we sought to determine whether HPV-specific MILs could be identified and expanded ex vivo.

**Methods**

We obtained bone marrow and peripheral blood from patients with localized, HPV-OPSCC. A modified version of the MANAFEST assay was used to evaluate proliferation of peripheral and bone marrow-derived CD8+ T cells in response to HPV early 6 (E6) and early 7 (E7) peptides (HPVFEST) in a HPV-OPSCC patient.

**Results**

T cell receptor sequencing and bioinformatic analysis of each peptide-stimulated culture revealed a markedly increased frequency of HPV-specific T cells in MILs compared to peripheral blood. HPV-specific MILs showed a higher average TCR clone frequency relative to PBLs (p value = 0.0037). Additionally, of the 5 highest expanded TCR clones for both compartments HPV-specific MILs possessed an increased average representative clone frequency (p value = 0.0001). To investigate general responsiveness to shared tumor antigens, we incubated autologous BM with lysate from an HPV+ OPSCC and then added ex-vivo activated MILs and PBLs. Tumor specificity was defined as IFN훾 production in CD3 cells. MILs possessed an average of 50.73% IFN훾 production as compared to 0.11% in PBLs to HPV-OPSCC lysate.

**Conclusions**

Collectively, these data indicate that HPV-specific T cells exist in the BM of patients with localized disease and possess a greater clonoytpic frequency and functional tumor recognition compared to PBLs. These data provide the rationale for developing this novel adoptive T cell approach using MILs in this patient population.

**Ethics Approval**

This study was approved by Johns Hopkins University Institution Review Board, approval number IRB00128334 and NA_00028682.

#### P170 Sequential anti-CD19, anti-CD22, and anti-CD20 autologous chimeric antigen receptor T cell (CAR-T) therapies treating a child with relapsed refractory Burkitt lymphoma

##### Juan Du, MD, Yonghong Zhang

###### Beijing Boren Hospital, Beijing, China

####### **Correspondence:** Yonghong Zhang (yhzhang58@126.com)

**Background**

Currently, the prognosis of children with relapsed refractory(r/r) Burkitt lymphoma(BL) remains dismal . New therapies are exlored to achieve a higher remission rate such as immunotherapy for these patients .We have successfully treated a case by adopting sequential autologous chimeric antigen receptor T cell(CAR-T) therapies, targeting antigen CD19,CD22,and CD20.

**Methods**

An 8-year-old boy was studied,who presented with a mass on the right side of the neck and was diagnosed with BL by pathology. The child was treated with standard chemotherapy but suffered from relapse. Subsequently, anti-CD19, anti-CD22, and anti-CD20 autologous CAR-T cell treatments were sequentially administered. We observed the clinical manifestations and response to the three cycles of CAR-T treatments, values of peripheral CAR-T cells were also monitored and side effects were assessed.

**Results**

The patient displayed no response to anti-CD19-CART treatment .After CD-22 directed CART the patient got partial remission (PR),but relapse occurred quickly. Finally, after the use of anti-CD20 CAR-T cell therapy, the child achieved complete remission (CR) and has currently achieved a 6-month event-free survival (EFS). During the CD19 and CD20 CAR-T cell treatments, only mild cytokine release syndrome (CRS) were observed in the patient (grade 1) while he developed a grade 3 CRS during CD22 CAR-T therapy, the symptoms included fever and hypoxemia.

**Conclusions**

Autologous CAR-T cell therapies targeting multplei tumor antigens could be novel and safe treatments for children with r/r BL.

**Consent**

Written informed consent was obtained from the patient for publication of this abstract and any accompanying images. A copy of the written consent is available for review by the Editor of this journal.

#### P171 T cell receptor gene therapy for a public neoantigen derived from mutated PIK3CA, a dominant driver oncogene in breast and endometrial cancers

##### Jiaqi Ma^2^, Martin Klatt, PhD^1^, Friederike Dundar, PhD^3^, Paul Zumbo^3^, Matthew Femia^1^, Doron Betel, PhD^3^, David Scheinberg^1^, Brian Baker, PhD^2^, Christopher Klebanoff, MD^1^, Smita Chandran, PhD^1^

###### ^1^MSKCC, New York, NY, United States; ^2^University of Notre Dame, Notre Dame, IN, United States; ^3^Weill Cornell Medicine, New York, NY, United States

####### **Correspondence:** Christopher Klebanoff (klebanoc@mskcc.org); Smita Chandran (chandrs1@mskcc.org)

**Background**

“Public” neoantigens represent immunogenic epitopes encompassing hotspot mutations in driver oncogenes that are also restricted by common HLA alleles. In contrast with patient-specific “private” neoantigens, public neoantigens are conceptually attractive because they are tumor-specific, clonally conserved, and shared across patients. Whether PIK3CA, the most common driver oncogene in breast and endometrial cancer, can yield public neoepitopes that may be exploited for cancer immunotherapy is unknown.

**Methods**

We have developed a high-throughput, single-cell functional assay for the discovery and retrieval of TCR alpha/beta gene sequences that confer specific recognition of endogenously processed and presented public neoantigens and not the corresponding wild type (WT) sequence. In this approach, donor-derived T cells are sensitized with autologous antigen presenting cells (APCs) electroporated with RNA encoding PIK3CA hotspot mutations. Expanded T cells are subsequently divided into paired daughter wells for short-term co-culture with APCs electroporated with minigenes containing either mutant or WT PIK3CA sequences. Acutely re-stimulated T cells from paired wells are subject to single-cell alpha/beta TCR VDJ and RNA sequencing. TCR alpha/beta gene sequences associated with selective upregulation of TCR signaling transcripts to mutant but not WT PIK3CA stimulation are subsequently cloned into retroviral vectors to confirm reactivity.

**Results**

Using this method, we have retrieved multiple TCRs that confer specific recognition of a public neoepitope derived from a PIK3CA hotspot mutation (H1047L). These TCRs are restricted by HLA-A*03:01, an allele present in 20.5% of the North American population. Immune-precipitation/tandem mass spectrometry analysis determined that the endogenously processed and presented public neoepitope is a 9 amino acid sequence containing a His to Leu substitution at position 2. To understand the mechanistic basis for the immunogenicity of this public neoepitope, we generated x-ray crystallography structures of mutant and WT epitopes bound to HLA-A*03:01 at ~2Å resolution. These studies revealed significant topologic overlap in the bound peptides. By contrast, the thermal and kinetic stability of the mutant peptide/HLA-A*03:01 complex was significantly enhanced relative to the WT complex, as measured by differential scanning fluorimetry and fluorescence anisotropy assays. Peripheral blood T cells genetically engineered with PIK3CA public neoantigen-specific TCRs cytolytically cleared target cells in a HLA/mutation-specific manner, leaving HLA-mismatched or WT target cells unperturbed.

**Conclusions**

These findings reveal for the first time the existence of an endogenously processed and presented public neoantigen derived from a PIK3CA hotspot mutation. These results open the possibility of targeting this common driver oncogene using adoptively transferred and genetically redirected T cells.

**Ethics Approval**

The study was approved by Smita Chandran's Institution‘s Ethics Board, approval number IRB 17-250.

#### P172 Shape and material properties increase artificial antigen presenting cell effectiveness

##### Savannah Est Witte, BS, Kaitlyn Calebrisi, Jordan Green, PhD

###### Johns Hopkins University, Baltimore, MD, United States

####### **Correspondence:** Jordan Green (green@jhu.edu)

**Background**

Particulate delivery of artificial antigen presenting cells (aAPCs) is a promising cell-free strategy to initiate selective T cell stimulation for immunotherapy in vivo. While 2D aAPC strategies aim to optimize T cell proliferation and selection in vitro for subsequent cell therapy, it would be advantageous to deliver “off-the-shelf” biodegradable aAPCs directly as an in vivo therapeutic. 3D aAPC effectiveness has been limited due to inefficiencies in stimulating T cells as well as rapid clearance of delivered particles. To improve bioavailability as well as increase particulate aAPC effectiveness, we have developed a soft, biodegradable, microparticle aAPC (Figure 1). To create a new platform technology for immunoengineering, material and shape are investigated as parameters for improving T cell stimulation.

**Methods**

One micron size particles were synthesized using a poly(ethylene glycol) diacrylate (PEGDA) or using a poly(lactic-co-glycolic acid) emulsion method [1,2]. Anti-CD3 and anti-CD28 conjugated particles were incubated with primary mouse T cells and proliferation was quantified at 3 and 7 days. For macrophage uptake studies, particles were incubated with macrophages at 37 °C to analyze particle uptake and 4 °C to evaluate binding of particles to cells. To synthesize soft, ellipsoidal aAPCs, a novel thin-film stretching technique was developed where emulsified PEGDA droplets were frozen then cast into films and stretched [3].

**Results**

Protein conjugation efficiency and T cell proliferation were 10-fold higher for PEGDA particles than PLGA particles (Figure 2a-b). Uptake studies indicate a ~20-fold decrease in binding and uptake by the PEGDA particles (Figure 2c). Ellipsoidal aAPCs stimulate T cells 3 times more effectively than spherical particles (Figure 3b,d). Uptake studies indicate a ~10-fold decrease in nonspecific uptake of ellipsoidal particles (Figure 3c).

**Conclusions**

Particle material and shape are significant factors in designing particulates as biomimetic aAPCs for in vitro T cell stimulation. Ongoing work on further decoupling these parameters and optimizing in vivo efficacy has the potential to unleash a promising biomimetic platform technology for immunoengineering of T cells.

**References**

1. Anselmo AC, Mitragotri S. Impact of Particle Elasticity on Particle-Based Drug Delivery Systems. Adv. Drug Deliv. Rev. 2017; 108:51–67.

2. Meyer RA, Sunshine JC. Biodegradable Nanoellipsoidal Artificial Antigen Presenting Cells for T Cell Activation. Small. 2016; 11:1519–1525.

3. Meyer RA, Meyer RS, Green JJ. An Automated Multidimensional Thin Film Stretching Device for the Generation of Anisotropic Polymeric Micro- and Nanoparticles. J. Biomed. Mater. Res. A. 2015; 103:2747–2757.

**Fig. 1 (abstract P172).**
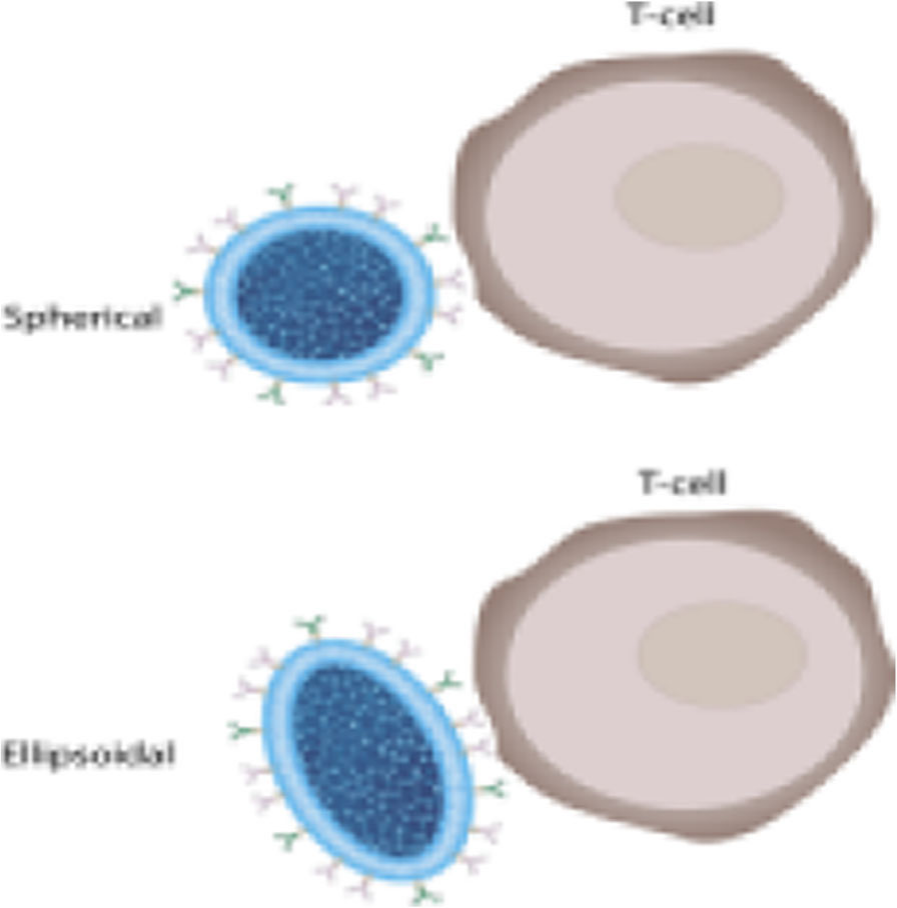
See text for description

**Fig. 2 (abstract P172).**
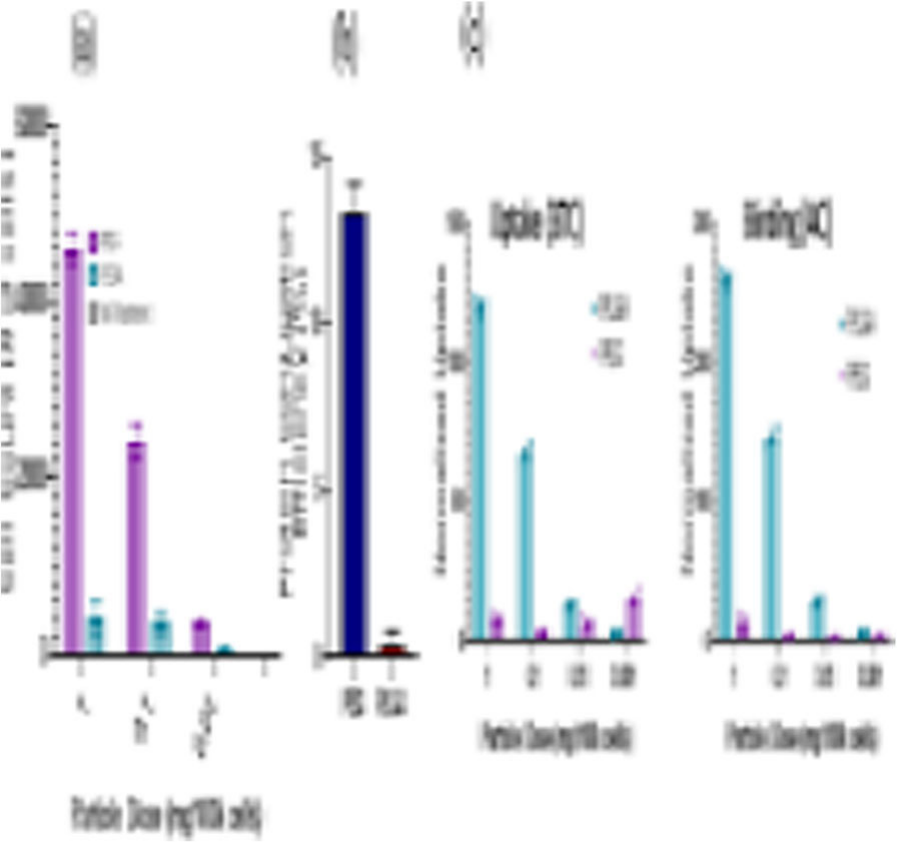
See text for description

**Fig. 3 (abstract P172).**
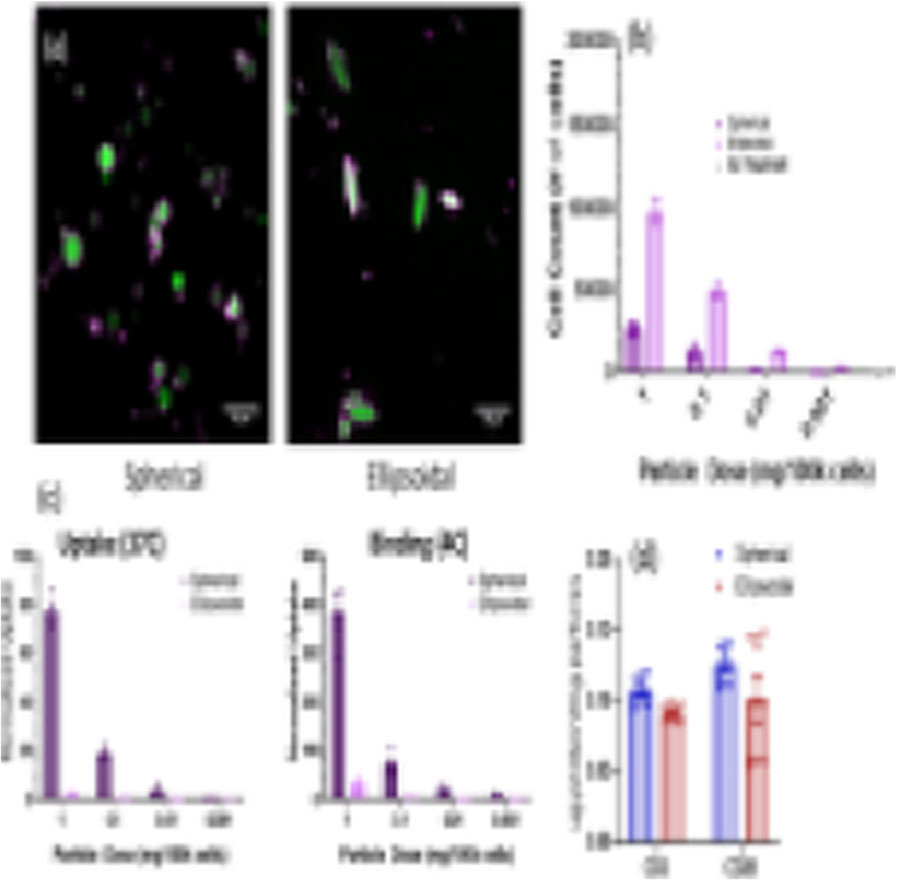
See text for description

#### P173 Case reports: Correlates of response following adoptive transfer of ADP-A2M4, affinity-enhanced T-cells targeting MAGE-A4, in synovial sarcoma

##### Svetlana Fayngerts, PhD^1^, Zohar Wolchinsky^1^, Shravani Shitole^1^, Joana Senra^1^, Rebecca Dryer-Minnerly, PhD^1^, Ruoxi Wang^1^, Jean-Marc Navenot, PhD^1^, Olga Ochkur^1^, Gareth Betts, PhD^1^, Natalie Bath, MSc^1^, Erin Van Winkle^1^, Tom Holdich^1^, Malini Iyengar, PhD^1^, Rafael Amado, MD^1^, Marcus Butler, MD^2^, David Hong, MD^3^, Alex Tipping, PhD^1^, Samik Basu, MD^1^, Indu Ramachandran, PhD^1^

###### ^1^Adaptimmune, Philadelphia, PA, United States; ^2^Princess Margaret Cancer Centre, Toronto, Ontario, Canada; ^3^MD Anderson Cancer Center, Houston, TX, United States

####### **Correspondence:** Indu Ramachandran (indu.ramachandran@adaptimmune.com)

**Background**

ADP-A2M4 is a genetically engineered autologous affinity-enhanced receptor immunotherapy (SPEAR T-cells) directed towards a MAGE-A4 peptide expressed in the context of HLA-A*02 on tumor cells. Clinical responses with ADP-A2M4 have been reported in patients with advanced MAGE-A4+ synovial sarcoma (SS) tumors. Here, we describe intra-tumoral and peripheral correlates associated with clinical response and resistance in two patients with SS.

**Methods**

Transduced T-cell persistence was determined by qPCR in PBMCs. Serum cytokines were measured via a multiplexed electrochemiluminescence-based immunoassay (MSD). Immunohistochemistry for antigen and immune markers was performed on FFPE tumor biopsies collected from patients prior to and following ADP-A2M4 transfer. A digital PCR-based assay was performed on FFPE tumor biopsies to detect the presence of SPEAR T-cells in the tumor. T-cell cytotoxicity assays were performed in vitro using the IncuCyte® platform. Clinical responses were assessed by RECIST v1.1.

**Results**

In the first patient, the best overall response (BOR) following ADP-A2M4 treatment was a partial response. Multiple correlates previously shown to be associated with response were observed. The patient’s pre- and post-infusion tumor biopsies expressed high levels of MAGE-A4 protein. Post-infusion, high levels of persisting transduced cells were observed in peripheral blood. Additionally, the patient had a grade 2 CRS event associated with a high level of serum IFN-g and IL-15 induction. In the post-infusion tumor sample, a notable increase in CD3+ T-cell infiltration, including SPEAR T-cells, was observed along with PD-L1 induction.

In the second patient, the BOR was stable disease; then the disease progressed. MAGE-A4 protein expression was lower prior to ADP-A2M4 infusion, compared to the 1st patient. Minimal peripheral induction of IFN-g and IL-15 was observed post-infusion along with a lower level of transduced T-cell persistence, compared with the 1st patient. No CRS was reported in this patient. Both patients’ manufactured products contained transduced CD8+ T-cells capable of killing antigen-expressing targets in vitro. In the responding patient, effective target killing was observed in transduced CD8+ T-cells isolated from the tumor site post-infusion. Eight additional SS patients have been treated, and we continue to analyze biomarkers in these patients.

**Conclusions**

Based on these two cases, we have identified some factors that may contribute to the anti-tumor activity of ADP-A2M4. High antigen expression levels, IL-15 and IFN-g cytokine induction, good engraftment, tumor site trafficking, and cytolytic function of SPEAR T-cells may be associated with favorable responses in SS patients treated with ADP-A2M4.

**Trial Registration**

NCT03132922

#### P174 Combinatorial tumor targeting using a novel switchable RevCAR system

##### Anja Feldmann, PhD^1^, Anja Hoffmann^1^, Ralf Bergmann^1^, Liliana Loureiro, PhD^1^, Enrico Kittel-Boselli^2^, Nicola Mitwasi^1^, Stefanie Koristka^1^, Justyna Jureczek^3^, Nicole Berndt^1^, Claudia Arndt^1^, Michael Bachmann^1^

###### ^1^Helmholtz-Zentrum Dresden-Rossendorf, Dresden, Germany; ^2^University Hospital Carl Gustav Carus, Dresden, Germany; ^3^German Cancer Research Center (DKFZ), Heidelberg, Germany

####### **Correspondence:** Anja Feldmann (a.feldmann@hzdr.de)

**Background**

Although T-cells genetically modified to express chimeric antigen receptors (CARs) are successfully used to treat hematological malignancies, patients still suffer from several drawbacks of conventional CAR (cCAR) therapy. CAR-T-cells can cause severe to life-threatening adverse reactions like on-target, off-tumor toxicities which cannot be controlled in patients. Moreover, cCAR therapy often fails to successfully affect solid tumors and bears the risk to encourage tumor escape variants upon targeting of only one single tumor-associated antigen (TAA). In order to overcome these problems, we have established a novel on/off-switchable RevCAR system facilitating combinatorial targeting strategies.

**Methods**

For combinatorial targeting one T-cell has to be modified with two separate CARs recognizing different TAAs. The first CAR mediates the activation and the second CAR the costimulatory signal. In case of ‘AND’ gate targeting, dual-CAR-T-cells have to recognize both TAAs on the surface of the target cells to get activated. However, such combinatorial targeting strategies are struggling with several challenges including the adjustment of signal strength and affinity of both split CARs as well as the CAR size limiting the number of transduced specificities. In order to overcome these obstacles, our idea was to construct small RevCARs comprising only a small peptide epitope as extracellular domain. By removing the extracellular single-chain variable fragment (scFv) of cCARs, RevCARs avoid tonic signaling induced by scFv dimerization. As RevCARs do not have an extracellular antigen binding moiety, they cannot bind to any antigen per se. Thus, actually they are switched off. Only in the presence of a bispecific target module (RevTM), RevCAR-T-cells can be redirected to tumor cells and switched on. Finally, short-living RevTMs allow a repeatedly on/off-switch and controllability of RevCAR-T-cells and furthermore a flexible redirection of RevCAR-T-cells to any target.

**Results**

For proof of concept two small peptide epitopes were selected to construct the respective RevCARs. Additionally, a series of different RevTMs was generated recognizing one of the two peptide epitopes and simultaneously any potential TAA. RevTMs were able to efficiently redirect RevCAR-T-cells specifically against different tumor targets. Moreover, we show that combinatorial targeting can be achieved using our RevCAR system. Here, dual-RevCAR-T-cells were efficiently activated only after engagement by two RevTMs targeting the activating or costimulatory RevCAR and different TAAs.

**Conclusions**

Taken together, we developed a switchable RevCAR platform showing high effectiveness, increased specificity, improved safety, easy controllability, and small size facilitating combinatorial tumor targeting.

**Ethics Approval**

The study was approved by local authorities and the Ethics Board.

#### P175 PD-L1: A side-effect of T cell engagement or a main player in MDS tumor immune evasion?

##### Valentina Ferrari, BA^1^, Alison Tarke^1^, Hannah Fields^1^, Tiffany Tanaka^2^, Rafael Bejar^2^, Thomas Lane, MD^1^, Antonella Vitiello, PhD^1^, Maurizio Zanetti, MD^2^

###### ^1^PersImmune, San Diego, CA, United States; ^2^University Of California San Diego, San Diego, CA, United States

####### **Correspondence:** Antonella Vitiello (avitiello@persimmune.com)

**Background**

Immune checkpoint inhibitors (ICIs) are being tested in myelodysplastic syndromes (MDS) based on pre-clinical data suggesting that the relevant targets are expressed on tumor and immune cells. Here we study both tumor cells and T cells from patients with higher-risk MDS to assess the role of PD-L1.

**Methods**

Patients’ CD3+ control cells, CD34+ stem cells, and their autologous MDS cell lines (MCLs) were analyzed by DNA and RNA sequencing to identify somatic variants present in the tumor cells and absent from the control cells. From all somatic variants identified, we generated and tested neopeptides in vitro for their ability to induce tumor-specific T cell responses. A T cell killing assay was performed to assess which neopeptide-specific T cells were capable of mediating tumor cell lysis (Figure 1A). In parallel, tumor PD-L1 expression levels were measured by flow cytometry before and after 24-hour incubation with tumor-specific autologous T cells. As a control for background tumor cell lysis and PD-L1 expression, tumor cells were also incubated with CEF-specific T cells (CEF: CMV, EBV, and flu peptides).

**Results**

Patients’ tumor cells did not express PD-L1 at baseline. Remarkably, after co-culture, PD-L1 expression on the tumor cells ranged from 20% to 70% (Figure 1B). Tumor cells, when incubated with CEF-specific T cells, did not upregulate PD-L1, suggesting that PD-L1 expression may be linked to target recognition by neoantigen-specific T cells (Figure 2). Interestingly, tumor cell lysis was independent of PD-L1 expression on tumor cells (Figure 1C). Additionally, IFNg neutralization did not affect PD-L1 expression nor ability to lyse tumor cells (Figure 3). These data show that when tumor cells are incubated with autologous tumor-specific T cells, the tumor cells upregulate PD-L1 expression yet do not escape lysis by T cells.

Since lysis of tumor cells may occur prior to their upregulating PD-L1, we pre-incubated tumor cells with soluble IFNg prior to co-culture with T cells. Tumor cells were 96% PD-L1+, and were lysed by tumor-specific T cells at the same level of target cells that had not been treated with IFNg (Figure 4).

**Conclusions**

Collectively, these data lend support to the notion that, in this system, PD-L1 is not a main player in MDS tumor immune evasion, suggesting that tumor immune evasion might function in a PD-L1-independent way. They also suggest that cognate recognition of tumor cells by neoantigen-specific T cells can cause the upregulation PD-L1 on target cells via a yet to be identified mechanism.

**Ethics Approval**

The study was approved by the University of California, San Diego's Institutional Review Board, HRPP #161345.

**Fig. 1 (abstract P175).**
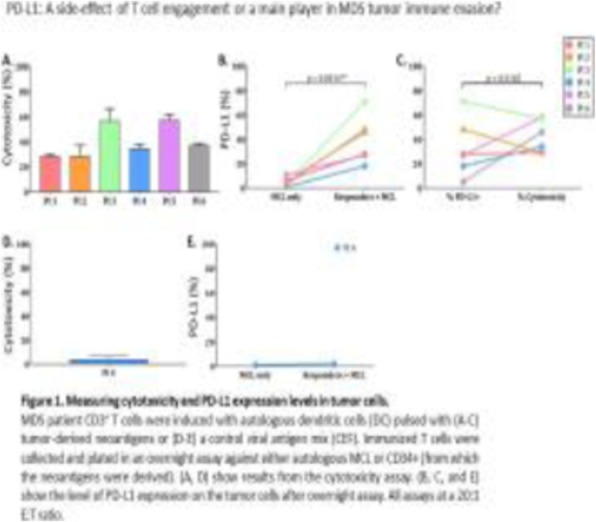
Ferrari, V SITC 2019 abstract slide 1

**Fig. 2 (abstract P175).**
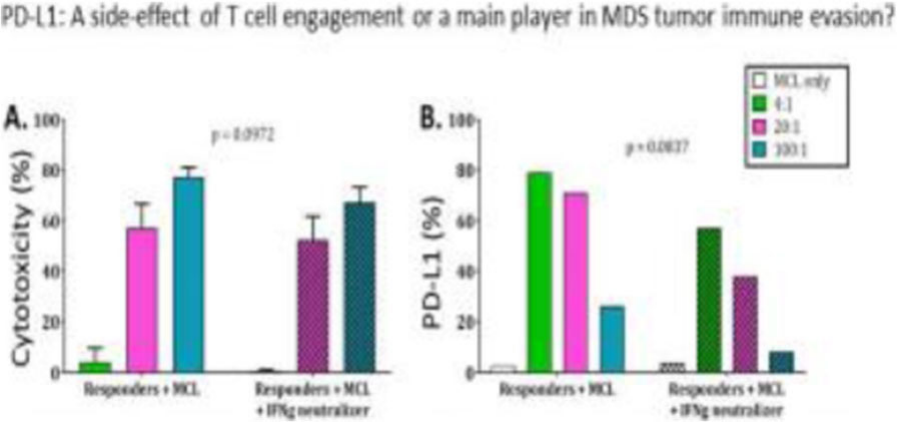
Ferrari, V SITC 2019 abstract slide 2

**Fig. 3 (abstract P175).**
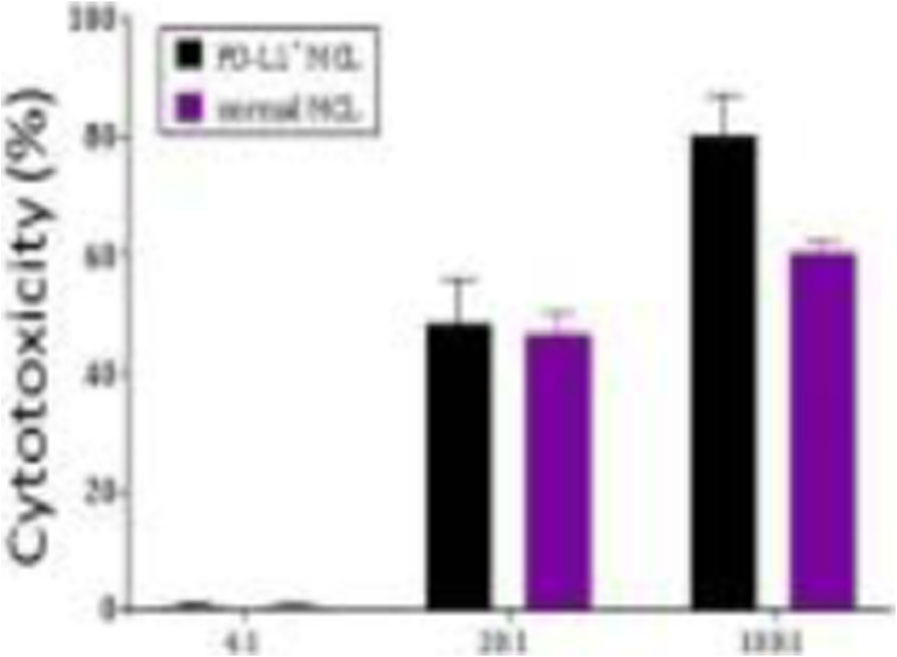
Ferrari, V SITC 2019 abstract slide 2

#### P176 Adoptive cell therapy using tumor-infiltrating lymphocytes (TIL) for metastatic uveal melanoma: feasibility of treatment using a product generated from the primary tumor

##### Marie-Andree Forget, PhD, Cara Haymaker, PhD, Orenthial Fulbright, BS, Shawne Thorsen, BS, Esteban Flores, BS, Arely Wahl, MSc, Rene Tavera, BS, Benjamin Tintera, BS, Timothy Woody, Michelle Williams, Yun Shin Chun, Patrick Hwu, MD, Dan Gombos, Sapna Patel, MD, Rodabe Amaria, MD, Chantale Bernatchez

###### MD Anderson Cancer Center, Houston, TX, United States

####### **Correspondence:** Chantale Bernatchez (CBernatchez@mdanderson.org)

**Background**

MD Anderson Cancer Center’s tumor-infiltrating lymphocyte (TIL) program has expanded TIL from tumor fragments of cutaneous metastatic melanoma using high dose IL-2 from over 900 patients with a growth success averaging 62% [1]. Surprisingly, this growth success plunges to 45% for metastatic uveal melanoma tumor fragments [2] and less than 20% from primary uveal tumors. The reason for this drop is unclear as uveal melanomas have an infiltration of CD8+TIL comparable to cutaneous melanoma [2], which in theory makes it an attractive candidate for immunotherapy. However, limited success observed with checkpoint therapy prompted us to explore ex vivo manipulation of the TIL. A previous report demonstrated the feasibility of TIL adoptive cell therapy for metastatic uveal patients using a TIL product expanded from metastases [3].

Since the primary and metastatic sites of uveal melanoma display preserved gene mutations, indicating a potential shared antigen landscape, one could propose generating a TIL product from the primary tumor when the patient undergoes enucleation and to utilize this product for treatment at time of recurrence.

**Methods**

Given the challenge of propagating TIL from a primary uveal tumor in high dose of IL-2 only, we hypothesized that our new TIL3.0 method to propagate TIL from tumor fragments (1st phase of expansion), based on the 3-signals required for optimal activation of a T-cell (TCR engagement, costimulation and cytokine exposure) would enable TIL growth from a higher percentage of primary uveal tumors given the success obtained with metastatic sites [1]. This product can be banked and accessed later for treatment at recurrence.

**Results**

The TIL3.0 expansion platform was shown to be optimal for T-cell propagation allowing for successful expansion of TIL from primary uveal melanoma tumors in >90% of the cases (n=20). This expansion was rapid (less than 3 weeks) and consistently composed of CD8+CD3+TIL. This later observation is attributed to the use of the agonistic anti-CD137/4-1BB, Urelumab, as of costimulation signal in our TIL3.0 method.

The TIL3.0 method applied to primary tumors could be scaled and adapted for GMP. This process, followed by a rapid expansion protocol, was applied to treat the first metastatic uveal patient with a TIL product generated from the primary tumor. The patient was infused with a total of 14.4 billion TIL with a viability of 99%.

**Conclusions**

This study demonstrates the feasibility of generating a TIL product from a primary uveal tumor to be used for treatment at recurrence.

**Trial Registration**

NCT00338377

**References**

1. Tavera RJ, Forget MA, et al. Utilizing T-cell Activation Signals 1, 2, and 3 for Tumor-infiltrating Lymphocytes (TIL) Expansion: The Advantage Over the Sole Use of Interleukin-2 in Cutaneous and Uveal Melanoma. J Immunother. 2018; 41:399-405.

2. Qin Y, de Macedo MP, Reuben A, et al. Parallel profiling of immune infiltrate subsets in uveal melanoma versus cutaneous melanoma unveils similarities and differences: A pilot study. OncoImmunology. 2017; 6:e1321187.

3. Chandran SS, et al. Treatment of metastatic uveal melanoma with adoptive transfer of tumour-infiltrating lymphocytes: a single-centre, two-stage, single-arm, phase 2 study. Lancet Oncol. 2017; 18:792-802.

**Ethics Approval**

Institutional review board (IRB)-approved protocol# 2004-0069

#### P177 NF-kB p50-deficient immature myeloid cell (p50-IMC) adoptive transfer slows the growth of murine prostate and pancreatic ductal carcinoma

##### Rahul Suresh, PhD, David Barakat, PhD, Theresa Barberi, PhD, Lei Zheng, PhD MD, Elizabeth Jaffee, MD, Kenneth Pienta, MD, Alan Friedman, MD

###### Johns Hopkins University School of Medicine, Baltimore, MD, United States

####### **Correspondence:** Alan Friedman (afriedm2@jhmi.edu)

**Background**

NF-kB p50 binds DNA but, unlike p65, lacks a trans-activation domain and recruits co-repressors. Macrophages and dendritic cells lacking NF-kB p50 are skewed towards a pro-inflammatory phenotype, with increased cytokine expression and enhanced T cell activation; additionally, murine melanoma, fibrosarcoma, colon carcinoma, and glioblastoma grow slower in p50-/- mice. Given these data, we evaluated efficacy of p50-deficient immature myeloid cells (p50-IMC) adoptively transferred into tumor-bearing hosts. Immature cells were utilized to maximize tumor localization, and pretreatment with 5-fluorouracil (5FU) was examined due to its potential to impair marrow production of myeloid cells, to target tumor myeloid cells, and to potentially release tumor neoantigens.

**Methods**

WT-IMC or p50-IMC were generated by culturing lineage-negative marrow cells from WT or p50-/- mice in media containing TPO, SCF, and FL for six days followed by M-CSF for one day on ultra-low attachment plates. Mice inoculated with Hi-Myc prostate cancer (PCa) or K-Ras(G12D) pancreatic ductal carcinoma (PDC)-luciferase cells received 5FU followed five days later by three doses of 1E7 IMC every three to four days. Some groups also received four doses of anti-PD-1 antibody twice weekly alone or with p50-IMC.

**Results**

PCa grew slower in p50-/- mice, and absence of host p50 led to prolonged survival of mice inoculated orthotopically with PDC. 5FU followed by p50-IMC slowed PCa and PDC tumor growth ~3-fold in contrast to 5FU followed by WT-IMC, 5FU alone, or p50-IMC alone. Slowed tumor growth was evident for 93% of PCa tumors but only 53% of PDC tumors. In PCa, p50-IMC predominantly generated tumor and draining lymph node F4/80+ macrophages, but also CD11b+F4/80-CD11c+ conventional dendritic cells. A subset of tumor and nodal macrophages co-expressed Ly6C and MHCII and had reduced MR compared to host macrophages, collectively indicating a pro-inflammatory phenotype. p50-IMC also produced a 5-fold increase in activated PCa tumor CD8 T cells, and antibody-mediated CD8 T cell depletion obviated slower tumor growth induced by 5FU followed by p50-IMC. Anti-PD-1 markedly slowed PCa growth but had little efficacy against PDC, whereas anti-PD-1 combined with p50-IMC slowed PDC tumor growth to prolong survival more effectively than either alone in an initial experiment.

**Conclusions**

5FU followed by p50-IMC slows the growth of murine prostate and pancreatic ductal carcinoma and depends upon CD8 T cell activation. Deletion of p50 in patient-derived marrow CD34+ cells and subsequent production of IMC for adoptive transfer may contribute to the therapy of these and additional cancers, alone or with additional immunotherapies.

**Ethics Approval**

The study was approved by the Johns Hopkins University Animal Care and Use Committee, protocol MO19M10.

#### P178 Dual inhibition of PI3Kdelta and PI3Kgamma to enhance mitochondrial mass and ex vivo expansion of central and stem cell memory T cells from CLL patients

##### Christopher Funk, BS, , Shuhua Wang, MD, Alexandra Waller, BS, Lauren Fleischer, BS, Aditi Sharma, Harold Spencer, PhD, Vikas Gupta, MD, PhD, Sruthi Ravindranathan, PhD, Mala Shanmugam, PhD, Christopher Flowers, MD, MS, Edmund Waller, MD, PhD, FACP

###### Emory University School of Medicine, Atlanta, GA, United States

####### **Correspondence:** Edmund Waller (ewaller@emory.edu)

**Background**

For chimeric antigen receptor T-cell (CAR T) therapy to treat chronic lymphocytic leukemia (CLL), recent work associates remissions with infusion of sufficient non-exhausted memory CAR T, capable of oxidative phosphorylation [1]. Our work aims to modulate metabolic pathways during ex vivo expansion of T-cells for translation of these findings to clinical adoptive therapies. Class I catalytic PI3K enzymes, such as PI3Kdelta and PI3Kgamma, regulate T-cell differentiation, regulatory T cell formation, and TCR signaling [2]. In this study, we hypothesized that pharmacological inhibition of these pathways during ex vivo culture would increase populations of early memory CAR T with enhanced metabolic and survival potential.

**Methods**

Healthy- and CLL- donor peripheral blood mononuclear cells were isolated and cryopreserved. Thawed cells were sorted for CD3 expression prior to culture (or transduction) and expanded in G-Rex plates using anti-CD3/CD28 beads, 30 U/mL interleukin-2, and investigational drugs: idelalisib, duvelisib and ibrutinib for 9 days.

**Results**

Class I catalytic enzymes in T-cells, PI3Kdelta and PI3Kgamma, exhibit domain homology (Figure-1A). Accordingly, maximum cell yields for both idelalisib (Figure 1B) and duvelisib (Figure 1C) occurred upon inhibition of both isoforms. Comparing doses of duvelisib, idelalisib, and ibrutinib that yielded optimal T-cell expansion, we showed potent PI3Kdelta/gamma dual antagonism maximizes live T-cell yields (Figure 1D) out-performing interleukin-2-inducible kinase inhibition. PI3K antagonists increased frequencies of cells expressing co-stimulatory molecules (Figure 2A-B). A dose-dependent increase in cells expressing FAS/FAS-L (Figure 2C) and an increased expression of pro-survival BCL-2 (Figure 2D) after anti-CD3/28 stimulation suggests the increase in live cells with a TSCM phenotype is likely due to enhanced cell survival.

Next, we confirmed the positive effect of dual PI3Kdelta/gamma inhibition in expansion of T cells from CLL donors (Figure 3A-B). PI3K antagonists increased frequencies of CD8 cells (Figure 3C) and co-stimulatory molecule expressing cells (Figure 3D-E). Interestingly, addition of idelalisib to T cell cultures resulted in a dose-dependent decrease in immune checkpoint molecules LAG-3, Tim-3, and PD-1 (Figure 3F-J). Given the importance of T cells with the stem cell memory (TSCM) phenotype in adoptive T-cell therapy, T-cell differentiation was studied. PI3K antagonists increase the frequency of early, T-memory, and effector memory cells (Figure 4A). Notably, the frequency of TSCM doubled (Figure 4B). Lastly, PI3K antagonists significantly increased the mitochondrial mass (Figure 4C) within total CD3 (Figure 4D) and CD8 (Figure 4E) subsets.

**Conclusions**

Dual inhibition of PI3Kdelta and PI3Kgamma modulates aspects of T-cell biology relevant to CAR T remissions. PI3Kdelta/gamma antagonism enhances CD27 expression and mitochondrial mass, decreases immune checkpoint expression, and enriches the TSCM phenotype.

**Acknowledgements**

The authors thank the patients and healthy volunteers for their blood donations. CR Funk is supported by a Howard Hughes Medical Research Fellowship. The authors thank Emory’s Clinical Lymphoma Research Team for requesting patient consent and aiding in sample collection.

**References**

1. Fraietta JA, et al. Determinants of response and resistance to CD19 chimeric antigen receptor (CAR) T cell therapy of chronic lymphocytic leukemia. Nat. Med. 2018; 24:563-571.

[2]2. Fruman DA, Chiu H, Hopkins BD, Bagrodia S, Cantley LC, and Abraham RT. Leading Edge The PI3K Pathway in Human Disease. Cell. 2017; 170:605-635.

**Ethics Approval**

The study was approved by Emory University's Institutional Review Committee, approval number 00057236.

**Fig. 1 (abstract P178).**
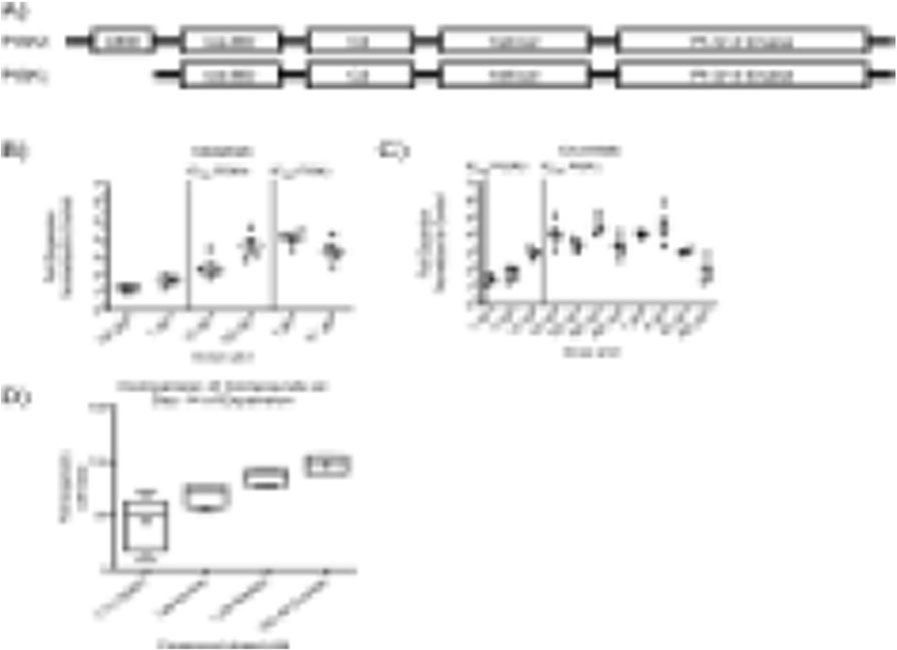
See text for description

**Fig. 2 (abstract P178).**
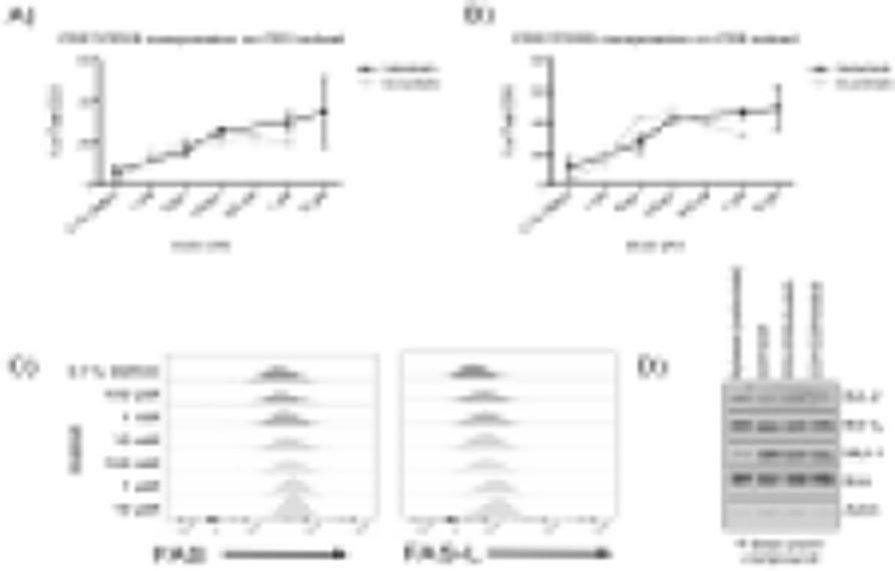
See text for description

**Fig. 3 (abstract P178).**
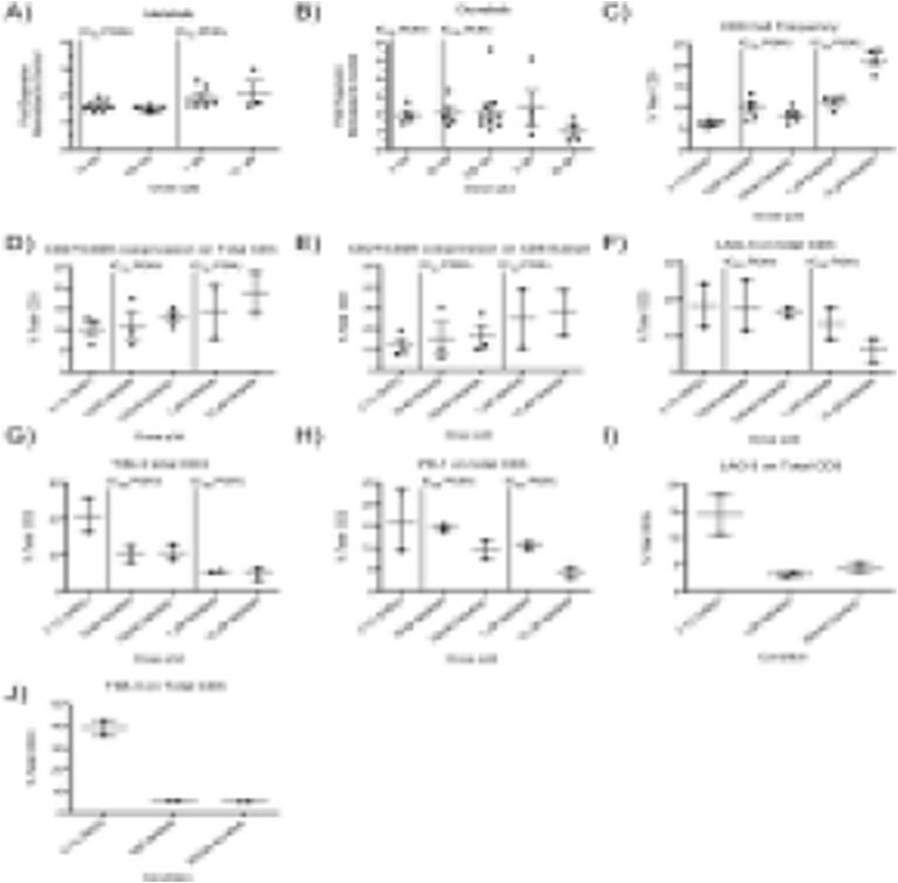
See text for description

**Fig. 4 (abstract P178).**
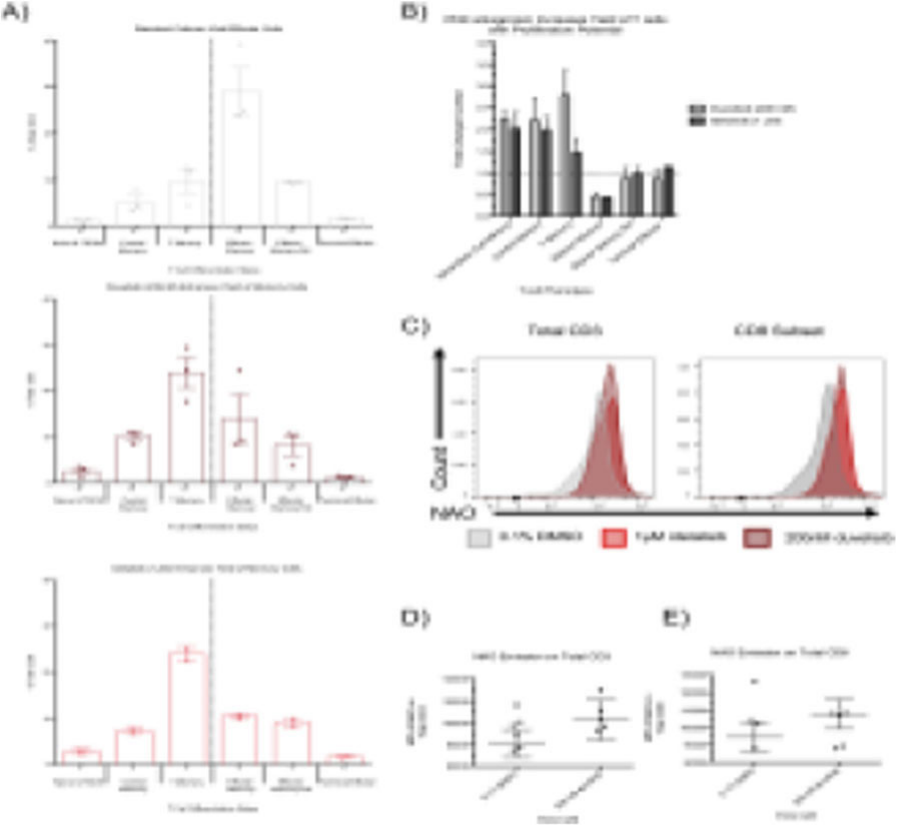
See text for description

#### P179 Checkpoint Cbl-b siRNA-based APN401 adoptive cell therapy: superior efficacy & immune memory induction in murine hepatocellular carcinoma following APN401 monotherapy and synergism with anti-PD1

##### Anderson Gaweco, MD, PhD^1^, Kathrin Thell^1^, Maria Urban^1^, Julia Harrauer^1^, Isabella Haslinger^1^, Josef Penninger^2^, Vincent Chung^3^, Anthony El-Khoueiry, MD^4^, Carlos Becerra, MD^5^

###### ^1^Apeiron Biologics, Vienna, Austria; ^2^University of British Columbia, Vancouver, BC, Canada; ^3^City of Hope, Duarte, CA, United States; ^4^USC Norris Comprehensive Cancer Center, Los Angeles, CA, United States; ^5^Baylor University Medical Center, Dallas, TX, United States

####### **Correspondence:** Anderson Gaweco (anderson.gaweco@apeiron-biologics.com)

**Background**

The intracellular master checkpoint Cbl-b, an E3 ubiquitin ligase, negatively regulates the innate and adaptive anti-tumor immune responses. Selective cell-based targeting of Cbl-b not only induces anti-tumor activity in vivo but also overrides immune regulation by the PD-L1/PD-1 pathway in vitro [1]. Human APN401 is an autologous adoptive cellular therapy of ex vivo Cbl-b-silenced human PBMCs currently in a clinical Phase 1b multiple dose study demonstrating early clinical safety and tolerability in patients with advanced solid tumors. Herein the preclinical Proof of Concept efficacy of murine APN401 immunotherapy is established in the syngeneic mouse hepatocellular carcinoma Hepa1-6 tumor rechallenge study.

**Methods**

Hepa1-6-C57/BL6-tumor bearing mice were treated for 19 days with murine APN401, ex vivo silenced immune cells with Cbl-b specific siRNA, as monotherapy or in combination with anti-PD1 (clone RMP1-14) versus control siRNA on D7 post-inoculation. Mice were later rechallenged on D29 with Hepa1-6 s.c. on the contralateral flank in the absence of any further APN401 treatment.

**Results**

Significant tumor growth inhibition (TGI) was observed following 19 days of treatment with APN401 alone or in combination with anti-PD1 starting on D7 after s.c. inoculation (p<0.0001). Following rechallenge, significant TGI of 86% (p<0.001) was observed in prior APN401-treated mice versus control siRNA-treated mice. Mice that received APN401 in combination with anti-PD1 prior to rechallenge demonstrate profound synergistic anti-tumor efficacy (p<0.001) versus anti-PD1 alone with control siRNA. APN401 was well tolerated and APN401-treated mice were unremarkable with optimal body conditions.

**Conclusions**

APN401 monotherapy demonstrates striking anti-tumor efficacy in the murine hepatocellular carcinoma Hepa1-6 model. The preclinical synergistic effects of APN401 with anti-PD1 support its therapeutic utility as a combination therapy with immune checkpoint anti-PD1 treatment. The significant TGI observed following tumor rechallenge indicate that prior selective cell-based Cbl-b-silencing with APN401 alone or in combination with anti-PD1 induced systemic and durable anti-tumor immune memory responses. These findings highlight the potential promise of a selective adoptive cell-based Cbl-b silencing by APN401 as a novel immunotherapy for cancer.

**Reference**

1. Fujiwara M, Anstadt EJ, Clark RB. Cbl-b Deficiency Mediates Resistance to Programmed Death-Ligand 1/Programmed Death-1 Regulation. Front Immunol. 2017 Jan 26;8:42.

#### P180 SENTI-101, an allogeneic cell product, induces potent and durable anti-tumor immunity in pre-clinical models of peritoneal carcinomatosis

##### Alba Gonzalez Junca, PhD, Gary Lee, PhD, Archana Nagaraja, PhD, Alyssa Mullenix, Russell Gordley, PhD, Daniel Frimannson, PhD, Anissa Benabbas, Chen-Ting Lee, PhD, Tiffany Truong, Allison Quach, Mengxi Tian, Rishi Savur, Rowena Martinez, Alyssa Perry-McNamara, Don-Hong Wang, PhD, Ori Maller, PhD, Dharini Iyer, PhD, Ashita Magal, Christina Huynh, Carmina Blanco, Jack Lin, PhD, Brian Garrison, PhD, Philip Lee, PhD, Timothy Lu, MD, PhD, Sravani Mangalampalli

###### Senti Biosciences Inc., South San Francisco, CA, United States

####### **Correspondence:** Gary Lee (gary.lee@sentibio.com)

**Background**

More effective therapies for disseminated peritoneal carcinomatosis, including high-grade serous ovarian cancer, remain a major medical need [1]. Although several treatments offer initial responses to localized disease, patients with disseminated peritoneal tumors face poor overall survival [2].

SENTI-101 is a novel therapeutic agent comprising allogeneic mesenchymal stromal cells (MSCs) genetically modified to express a potent combination of immunomodulatory cytokines: IL12 and IL21. Upon administration, SENTI-101 innately homes to peritoneal tumors, secretes IL12 and IL21 in a localized and sustained fashion, and induces a robust anti-tumor immune response.

**Methods**

Two syngeneic pre-clinical models of disseminated peritoneal carcinomatosis with distinct immune phenotypes were established by implanting cells in the peritoneal cavities of mice (CT26-fLUC = immune-inflamed; B16-F10-fLUC = immune-excluded) [3]. A library of over 50 murine MSC lines engineered to express immune effectors (cytokines, chemokines, growth factors), either individually or in combination, was administered intraperitoneally and evaluated for anti-tumor activity via bioluminescence and tumor weight measurements. Immune phenotype was characterized by flow-cytometry and multiplexed immunohistochemistry.

**Results**

MSCs expressing the combination of IL12 and IL21 (SENTI-101) were selected based on significant tumor-burden reduction and immune profile changes in both syngeneic models. Notably, the combination outperformed each individual cytokine in extending survival (p=0.02).

Intraperitoneal administration of SENTI-101 into tumor-bearing mice led to preferential co-localization with tumors (>10-fold higher vs. normal tissues, p=0.001). Local concentrations of IL12 and IL21 were ~100-fold greater in the peritoneal space vs. serum (p=0.002). SENTI-101 treatment reduced tumor-burden more than 200-fold (p50% of the mice were tumor-free after 90 days, while control groups and groups treated with anti-PD1 antibody had a median survival of 21 to 30 days. Surviving mice were able to reject newly implanted tumor cells, demonstrating anti-tumor immune memory.

Anti-tumor effects of SENTI-101 are mediated by a multi-modal immune response. The frequency of antigen-presenting cells in peritoneal tumor-draining lymph nodes was more than doubled vs. controls (p=0.01). This correlated with increased T-cell and B-cell tumor infiltrates forming tertiary-lymphoid structures, which are associated with improved prognosis in cancer [4]. T-cell activation markers (CD38, IFNg, GranzymeB) were significantly increased locally.

**Conclusions**

SENTI-101 induces localized immune-modulation, regulates multiple steps of the cancer immunity cycle, and results in durable anti-tumor responses. These data warrant further development of SENTI-101 for the loco-regional treatment of advanced solid tumors.

**References**

1. Desai JP, Moustarah F. Cancer, Peritoneal Metastasis. StatPearls Publishing. 2019 [Updated 2019 Jun 30]

2. Lengyel E. Ovarian Cancer Development and Metastasis. Am J Pathol. 2010; 177(3): 1053–1064

3. Mosely SI, Prime JE, Sainson RC, et al. Rational Selection of Syngeneic Preclinical Tumor Models for Immunotherapeutic Drug Discovery. Cancer Immunol Res. 2017; 5(1):29-41

4. Sautès-Fridman C, Petitprez F, Calderaro J, Fridman WH. Tertiary lymphoid structures in the era of cancer immunotherapy. Nat Rev Cancer. 2019;19(6):307-325

#### P181 Multi-phenotype CRISPR-Cas9 screens identify p38 kinase as a target for adoptive immunotherapies

##### Devikala Gurusamy, PhD, Suman Vodnala, Amanda Henning, Rigel Kishton, Tori Yamamoto, Arash Eidizadeh, Li Jia, Christine Kariya, Mary Black, Robert Eil, Douglas Palmer, Zhiya Yu, Jenny Pan, MD, Madhusudhanan Sukumar, Shashank J. Patel, Nicholas Restifo, MD

###### National Institutes of Health, Bethesda, MD, United States

####### **Correspondence:** Nicholas Restifo (restifo@nih.gov)

**Background**

Adoptive T cell transfer immunotherapy (ACT) using tumor-infiltrating lymphocytes (TIL) and gene-modified T cells can induce complete and durable regression of metastatic human malignancies that are otherwise refractory to treatment. While successful T cell-based treatments of patients with widely metastatic melanoma, synovial sarcoma, cholangiocarcinoma and cancers of the breast, colon, and cervix have been reported in recent years, most patients with common epithelial cancers fail to respond to treatment. While several factors can contribute to the efficacy of ACT, a major inherent limitation is the induction of terminally differentiated phenotype coupled with the loss of proliferative capacity in TIL during current ex vivo expansion protocols. Individual gene knockout approaches for enhancing T cell-based cancer immunotherapies are low-throughput and can improve one desired function (T cell memory) at the expense of another equally important function (expansion). Thus, there is significant interest in identifying T-cell intrinsic negative regulatory circuits that limit their ability to expand robustly ex vivo, while dampening their terminal effector differentiation along with the reduction of oxidative stress and genomic damage.

**Methods**

To identify the T cell intrinsic negative regulatory circuits, we developed a multi-phenotype genetic screen to systematically target 29 major kinases screen to concurrently measure the impacts of individual gene knockouts on T cell expansion, differentiation, oxidative stress and genomic stress. Using CRISPR-Cas9-based gene perturbation combined with high-throughput flow cytometry, we developed and validated a multi-phenotype screen, which identified Mapk14/p38 kinase as a target that improved all four phenotypes in CD8+ T cells. We used murine and human ex vivo T cell expansion models to validate the results from our genetic screen.

**Results**

Results from our genetic screen identified p38 kinase as a unique multi-phenotypic regulator of cellular differentiation, oxidative, and genomic stress while achieving improved cellular expansion. Furthermore, pharmacological inhibition of p38 kinase in murine and human ex vivo T cell expansion models validated the results from our genetic screen. Cells cultured in the presence of a p38 inhibitor had increased capacity for cytokine production, specifically interferon-γ and demonstrated improved in vivo persistence. Additionally, cells cultured in the presence of the p38 inhibitor demonstrated enhanced in vivo cell-expansion, tumor infiltration, and anti-tumor efficacy in an immunocompetent tumor mouse model.

**Conclusions**

This study establishes p38 inhibition in T cells as a potentially important strategy for improving ACT immunotherapy for cancer patients.

**Ethics Approval**

All human samples were isolated in accordance with approved clinical protocols and in accordance with NIH institutional review board approval and informed consent from patients and healthy donors.

#### P182 GAIA-102: a new class NK cell-like phenotype manufactured in accordance with GMP/GCTP that can eliminate solid tumors

##### Yui Harada, PhD, Yoshikazu Yonemitsu, MD, PhD

###### Kyushu University, Fukuoka, Japan

####### **Correspondence:** Yui Harada (rkfraile@med.kyushu-u.ac.jp)

**Background**

[Background] Cancer immunotherapy has been established as a new therapeutic category since the recent success of immune checkpoint inhibitors and a type of adoptive immunotherapy, namely chimeric antigen receptor-modified T cells (CAR-T). Although CAR-T demonstrated impressive clinical results, serious adverse effects (cytokine storm and on-target off-tumor toxicity) and undefined efficacy on solid tumors are important issues to be solved. We’ve developed a cutting-edge, simple, and feeder-free method to generate highly activated and expanded human NK cells from peripheral blood (US9404083, PCT/JP2018/018236, PCT/JP2019/012744), and have been conducting further investigation why our new type of NK cells, named as GAIA-102, are so effective to kill malignant cells.

**Methods**

[Materials and Methods] Cryopreserved PBMCs purchased from HemaCare Corporation were mixed and processed by using LOVO and CliniMACS® Prodigy (automated/closed systems). CD3+ and CD34+ cells were depleted, and the cells were cultured at a concentration of 1 x 106 cells/ml with high concentration of hIL-2 and 5% UltraGRO® for 14 days in our original closed system. Then, we confirmed the expression of surface markers, CD107a mobilization and cell-mediated cytotoxicity against various tumor cells and normal cells with or without monoclonal antibody drugs in vitro and antitumor effects against peritoneal dissemination model using SKOV3 in vivo.

**Results**

[Results and Discussion] Importantly, we’ve found that our GAIA-102 exhibited CD3-/CD56bright/CD57- immature phenotype that could kill various tumor cells efficiently from various origins, including Raji cells that was highly resistant to NK cell killing. More importantly, massive accumulation, retention, infiltration and sphere destruction by GAIA-102 were affected neither by myeloid-derived suppressor cells nor regulatory T-lymphocytes. GAIA-102 was also effective in vivo to murine model of peritoneal dissemination of human ovarian cancer.

**Conclusions**

Thus, these findings indicate that GAIA-102 has a potential to be an ‘upward compatible’ modality over CAR-T strategy, and would be a new and promising candidate for adoptive immunotherapy against solid tumors. We now just started GMP/GCTP production of this new and powerful NK cells and first-in-human clinical trials in use of GAIA-102 will be initiated on 2020.

**Ethics Approval**

[Ethics Approval] Written informed consent was obtained from all healthy volunteers, in accordance with the Declaration of Helsinki. Upon the approval of the institutional ethical committee (approval no. 29-315) of Kyushu University, peripheral blood samples were collected from healthy volunteers. The animal experiments were reviewed and approved by the Institutional Animal Care and Use Committee of Kyushu University (approval nos. A30-234-0 and A30-359-0).

#### P183 Development of novel chimeric antigen receptor T cells for immunotherapy of hepatocellular carcinoma

##### Yukai He, PhD, Leidy Caraballo Galva, Xiaotao Jiang

###### Augusta University, Augusta, GA, United States

####### **Correspondence:** Yukai He (yhe@augusta.edu)

**Background**

Immunotherapy has a great potential for hepatocellular carcinoma (HCC). Several human glypican 3 (hGPC3)-specific chimeric antigen receptor T cells (CARTs) are being tested for HCC. But, most, if not all, are constructed from one monoclonal antibody (mAb). It is unknown whether targeting different epitopes of hGPC3 will create more effective CARTs. Here, we aim to develop novel CARTs that target different regions of hGPC3.

**Methods**

BalB/C mice were immunized with hGPC3 protein. Hybridomas and mAbs were generated and characterized. Then, CARTs were built from the novel mAbs and their antitumor effect was studied.

**Results**

Twenty-two hGPC3-specific mAbs were identified by ELISA. Out of them, 14 bound HepG2 cells. Five mAbs were further characterized by immunohistochemical staining. Three of them (6G11, 8F8, and 12D7) were found to specifically stain HCC tumor but not adjacent normal tissues. The 3 mAb’s affinity were in the nanomolar range. 6G11 and 8F8 bound to hGPC3 epitope aa25-39 and aa463-496, respectively. No specific epitope was identified for 12D7 though it bound to the N-fragment (25-358aa). CARTs built from the 3 mAbs underwent expansion in response to HepG2 cell stimulation. However, their effector function was significantly different. 8F8 CARTs possessed the strongest effector function. 6G11 CARTs generated the greatest expansion, but with slightly weaker function. In contrast, 12D7 CART had the weakest effector function. Soluble hGPC3 did not activate CARTs, nor blocked CART activation by tumor cells. Adoptive transfer of 8F8 and 6G11, but not 12D7, CARTs generated potent antitumor effects with complete regression of HCC xenografts, which correlated to their expansion in vivo.

**Conclusions**

The three novel CARTs that target different hGPC3 regions possess significantly different effector function and antitumor effects. Adoptive transfer of CARTs targeting the hGPC3 N- or C-epitope results in complete eradication of HCC xenografts.

#### P184 Activating marrow infiltrating lymphocytes in hypoxia enhances their efficacy in adoptive T-cell therapy

##### Megan Heimann^1^, Ervin Griffin^1^, Luca Biavati, MD^1^, Amy Thomas^1^, Danielle Dillard^1^, Elizabeth Zawidzka^1^, Brianna Richardson^2^, Robert Leone^1^, Gregory Szeto^2^, Kimberly Noonan, PhD^3^, Ivan Borrello, MD^1^

###### ^1^Johns Hopkins University School of Medicine, Baltimore, MD, United States; ^2^University of Maryland Baltimore County, Baltimore, MD, United States; ^3^WindMIL Therapeutics, Baltimore, MD, United States

####### **Correspondence:** Ivan Borrello (iborrell@jhmi.edu)

**Background**

Marrow infiltrating lymphocytes (MILs) are a promising candidate for adoptive cell therapy (ACT) due to their broader anti-tumor specificity and persistence. These characteristics are due to intrinsic properties of the bone marrow (BM); known to be a reservoir for long-lived memory T cells. It has also been established that naïve and memory T cells are metabolically quiescent, favoring oxidative phosphorylation (OXPHOS) over glycolysis, while effector T cells favor glycolysis to fuel their rapid proliferation.

**Methods**

We examined how activation and expansion of MILs in hypoxia could be used to better understand the inherent properties of the BM, to exploit these properties, and enhance the efficacy of MILs in ACT, especially when compared to that of peripheral blood lymphocytes (PBLs). By activating MILs in hypoxia, we can select for and/or alter the cells best suited to mount an effective anti-tumor response.

**Results**

Activation under hypoxic conditions alters MILs in several unique ways. MILs show greater overall expansion, enhanced tumor-specificity, and a unique metabolic profile—upregulating both OXPHOS and glycolytic machinery. This metabolic profile suggests that hypoxia-activated MILs possess properties of both effector and memory cells. PBLs grown under the same conditions fail to expand significantly and show no metabolic differences or specificity. Following activation in hypoxia we have found that MILs have upregulated metabolism-related genes such as CPT1A and GLUT1 and 3, as well as anti-apoptotic factors such as BCL2 and BCL2L1 at an RNA level. The post-expansion MILs product, using intracellular staining and FACs analysis, shows an increase in mitochondrial proteins, including TOMM20, CPT1a, and SDHa, increased mTOR signaling, and increased glycolytic machinery—HK2 and GLUT1. Additionally, while cell-cycle analysis with Ki67 and PI shows that MILs are more resting at baseline and arrested in G0, upon activation in hypoxia they become more proliferative—moving into S and G2/M phase—than normoxic MILs or PBLs and maintain this phenotype over time. This finding is supported by our RNAseq data showing a lack of transcriptional activity at baseline but a significant increase by day 3 of activation. Gene expression analysis has also shown that there is a substantial increase in expression of IL2 and IL15Ra with a significant decrease in the expression of IL10 transcripts.

**Conclusions**

These findings suggest that hypoxia contributes to the unique properties of MILs through modification of their metabolic profile and is being uniquely employed to generate more effective MILs, and not PBLs, for adoptive cell therapy.

#### P185 T cell antigen presenting cell (tAPC) is a strategy to induce CAR T expansion in vivo in the absence of a tumor for on-target toxicity studies

##### Yen Ho, BS, Jon Jones, BA, Patrick Carlson, BS, Cyr de Imus, Rebekah Turk, Dina Alcorn, Kyle Kolaja, Ruth Salmon, PhD, Thomas Long

###### Celgene Corporation, Seattle, WA, United States

####### **Correspondence:** Thomas Long (thomas.long@junotherapeutics.com)

**Background**

Preclinical safety evaluation of chimeric antigen receptor (CAR) T cells presents a number of unique challenges. One of those challenges is the development of a Cynomolgus macaque toxicology model as a tool to understand potential on-target CAR T cell toxicity. This is important for CAR T cell programs where the lead binder is cyno cross-reactive and the target has known normal tissue expression conserved across species. Unlike many mouse models, non-human primates lack target-expressing tumors that can drive CAR T cell activation and expansion; it would be advantageous to recapitulate that expansion in the context of a toxicity model.

**Methods**

T cell antigen presenting cells (tAPCs) have been reported to drive measurable CAR T cell expansion in Rhesus macaques [1]. The advantages of this strategy include the ease of manufacturing tAPCs in parallel to CAR T cells and co-engraftment of tAPCs with CAR T cells in hematological niches to ensure antigen availability. The optimal tAPC dosing strategy to drive a strong and persistent CAR T cell activity has not been determined. Here, we generated human anti-CD19 CAR T cells expressing firefly luciferase and human T cells expressing a truncated human CD19 (CD19t) lacking the intracellular domain as tAPCs. We then dosed mice either with CAR and tAPC concurrently, or with CAR first then followed by tAPCs three days later.

**Results**

(Figure 1) shows bioluminescent imaging (BLI) measurements that indicate concurrent and delayed tAPC dosing have similar CAR T cell expansion kinetics; however, the delayed tAPC dosing exhibits a greater magnitude of CAR T cell expansion. In both cases, the CAR T cell expansion occurs in a tAPC dose-dependent manner. Imaging shows that concurrent dosing leads to CAR T cell proliferation primary in the lungs, whereas delayed tAPC dosing leads to more systemic CAR T cell expansion. In addition, flow cytometry data show a significant depletion of tAPCs in the peripheral blood between day 7 and day 14.

**Conclusions**

Follow-up studies delivering additional tAPC doses three, six, and nine days after CAR T cells show that repeated tAPC administration can significantly increase the CAR T cell exposure over time compared to a single tAPC dose. Overall, these data demonstrate that tAPCs can be used to induce CAR T cell expansion in vivo in the absence of a tumor and will enable us to design a tAPC strategy for use in a Cynomolgus macaque model to evaluate the safety of CAR T cell candidates.

**Reference**

1. Berger C, Sommermeyer D, Hudecek M, Berger M, Balakrishnan A, Paszkiewicz PJ, Kosasih PL, Rader C, Riddell SR. Safety of targeting ROR1 in primates with chimeric antigen receptor-modified T cells. Cancer Immunol Res. 2015;3(2):206-16.

**Ethics Approval**

All animal studies were conducted in accordance with protocols approved by the Institutional Animal Care and Use Committee.

**Fig. 1 (abstract P185).**
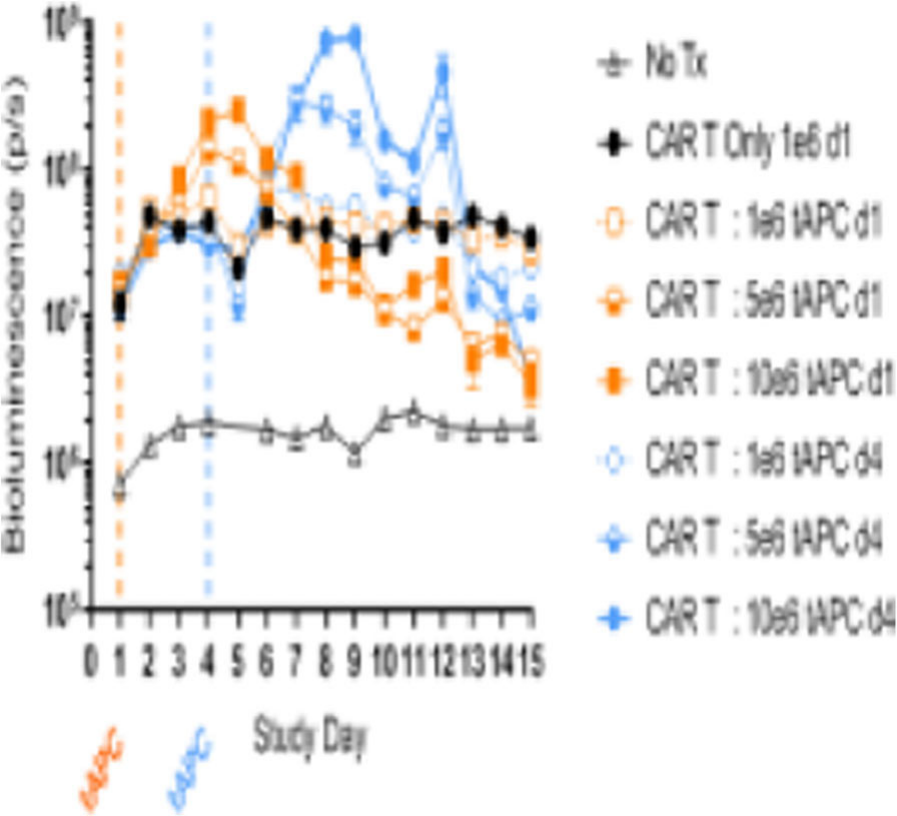
See text for description

#### P186 Evaluation of antigen-specific T-cell immunity at the single cell level using large panels of DNA barcoded MHC multimers

##### Kivin Jacobsen, PhD^1^, Dagmar Walter^2^, Michael Stubbington^2^, Katherine Pfeiffer^2^, Charlotte Halgreen^1^, Liselotte Brix, PhD^1^, Stephane Boutet^2^, Kivin Jacobsen, PhD^1^

###### ^1^Immudex, Copenhagen, Denmark; ^2^10x genomics, Pleasanton, CA, United States

####### **Correspondence:** Liselotte Brix (lb@immudex.com)

**Background**

Identification of disease-specific T-cell epitopes is key to developing novel cancer vaccines and immunotherapies. Profiling disease-specific T cells, emerging during an induced cellular immune response is important for understanding anti-tumor immunity and guide personalized therapy. The MHC dCODE™Dextramer® technology enables simultaneous screening of high numbers of T-cell specificities in the same sample using MHC multimer-specific DNA barcodes and next generation sequencing as readout. Combining this technology with 10x Genomics Chromium single cell assay further enables the simultaneous analysis of antigen specific T-cells, sequencing of the cognate T-cell receptors and single cell gene expression profiling.

**Methods**

Panels of up to 50 dCODE™Dextramer® reagents were used for screening antigen-specific T-cells in human blood samples. Single cell sequencing was performed on the isolated T-cell subpopulations, and their gene expression profile analyzed in combination with cell phenotype and TCR sequences.

**Results**

The experiment generated a large dataset and we show one example of how such a dataset can be analyzed to generate useful information. By combining the gene expression profile, cellular phenotypes and Dextramer specificity we identified expanded populations of antigen-specific T cells in the memory T cell compartment and characterized their individual TCR clonotypes based on TCR sequence. pMHC-specific T-cells were also detected in the naïve T cell compartment, showing a more diverse TCR sequence profile.

**Conclusions**

This experiment demonstrates a novel method of exploring antigen-specific T-cell responses. Linking TCR sequences with pMHC specificity, cellular phenotypes and gene expression at this scale and resolution provide a more comprehensive analysis of the antigen-specific T cell response than previously available in a single workflow. Novel biomarkers and improved strategies of T cell based immunotherapeutic development will result from T cell analysis at this scale and resolution.

#### P187 Single-cell RNA sequencing and functional assessment of healthy donor- and cancer patient-derived T and CAR-T cells

##### Zinkal Padalia, MS, Konstantinos Karagiannis, PhD, Brigid Mcewan, MA, Vahan Simonyan, PhD, Jonathan Terrett, PhD, Demetrios Kalaitzidis, PhD

###### CRISPR Therapeutics, Cambridge, MA, United States

####### **Correspondence:** Demetrios Kalaitzidis (d.kalaitzidis@crisprtx.com)

**Background**

Autologous chimeric antigen receptor T (CAR-T) cell therapies have shown remarkable success in treating relapsed/refractory B-cell malignancies. However, even in indications with high complete response rates, not all patients respond or have durable responses after CAR-T treatment. Furthermore, autologous CAR-T treatments have not yielded the same impressive outcomes in solid malignancies to date. A major limitation of autologous CAR-T therapy may be the dysfunctional state of a patient’s T cell populations used for manufacturing of a drug product. Allogeneic therapeutics can bypass this limitation by enabling the use of healthy donor starting material. Moreover, healthy donor material that exhibits specific T cell attributes can be selected for drug product manufacturing.

**Methods**

To identify attributes that can be associated with improved performance of CAR-T cells we have characterized T cells from healthy donors as well as cancer patients, in particular from chronic lymphocytic leukemia (CLL) patients as these have been described previously to be dysfunctional.

**Results**

We show impaired function of cancer patient-derived CAR-T cells when compared to healthy donor-derived cells utilizing both in vitro and in vivo assays. We have performed single-cell RNA sequencing (scRNA seq) on both starting material T cells and CAR-T cells from multiple healthy and CLL donors used in functional assays to uncover both gene expression and population differences associated with CAR-T cell performance. scRNA seq analysis revealed marked heterogeneity among starting populations as well as CAR-T lots from the cancer patient-derived T cells.

**Conclusions**

Our analysis has allowed us to associate distinct cellular subpopulation and gene expression profiles with preclinical functional outputs.

#### P188 Enhanced anti-tumor activity of human placental CD34+ derived natural killer cells in combination with ACY-241 for multiple myeloma immunotherapy

##### Lin Kang, PhD, Xiaokui Zhang, Shuyang He, Vanessa Voskinarian-Berse, Bhavani Stout, Valentina Rousseva, William van der Touw, James Edinger, Robert Hariri

###### Celularity Inc., Warren, NJ, United States

####### **Correspondence:** Xiaokui Zhang (xiaokui.zhang@celularity.com)

**Background**

Celularity, Inc. is developing human placental CD34+ derived, off-the-shelf, and allogeneic natural killer (PNK) cells for various hematologic malignancies and solid tumors. ACY-241 is an orally bioavailable and selective histone deacetylase (HDAC) 6 inhibitor in MM clinical development. ACY-241 has been shown to sensitize MM cells to endogenous NK cell killing [1]. Here, we investigated the potential augmentation of PNK mediated anti-MM activity by ACY-241 treatment.

**Methods**

Placental CD34+ cells were cultivated in the presence of cytokines including thrombopoietin, SCF, Flt3 ligand, IL-7, IL-15 and IL-2 for 35 days to generate PNK cells. MM cell lines were treated with different doses (0, 0.1, 0.3, 1, 3, 10 and 30μM) of ACY-241 over 24h, 48h, or 72h. Cytotoxicity of PNK against different doses of ACY-241 pretreated MM cell lines was assessed by a PKH26/TO-PRO-3 FACS based assay. Ligands to NK activating receptors of ACY-241 treated MM cells were evaluated by flow cytometry. The RPMI8226 subcutaneously(SubQ) xenografted NOD scid gamma (NSG) mouse model was used for in vivo efficacy study.

**Results**

ACY-241 treatment of MM cell lines for >48h resulted in significant inhibition of cancer cell growth and decreased cell viability at doses >10μM. In a dose dependent manner, ACY-241 further enhanced cytotoxicity of PNK against MM cells. In a 4h cytotoxicity assay at effector to target (E:T) ratio of 10:1, relative to vehicle control, PNK (n=3 donors) showed increased cytotoxicity to ACY-241 pretreated MM cell lines: RPMI8226 (16.3% to 34.9%), MM.1S (15.1% to 26.0%), OPM2 (12.7% to 37.2%), and U266 (0% to 9.1%). Increased expression of ligands to activating NK receptors, MIC A/B, CD56, CD54, and CD155 was detected from ACY-241 treated MM cells, suggesting that engagement of NKG2D, CD11a or DNAM-1 of NK cells leads to enhancement of the anti-MM effect.

In vivo anti-MM activity of PNK in combination with ACY-241 was assessed in a RPMI8226 SubQ xenograft NSG model. Single intravenous dosing of 1.0E7 PNK in combination with ACY-241 significantly reduced the tumor growth compared to vehicle control (P<0.001).

**Conclusions**

Our data demonstrated that enhanced in vitro anti-MM activity of PNK in combination with ACY-241. In vivo efficacy of PNK in combination with ACY-241 was further demonstrated in a RPMI8226 SubQ xenograft NSG model. Taken together, our results demonstrate the synergistic effects of combining an HDAC inhibitor with an NK cell therapy for anti-MM enhancement. Further development of a combinatorial PNK and ACY-241 therapy for MM treatment is warranted.

**Reference**

1. Ray A, Das DS, Song Y, Hideshima T, Tai YT, Chauhan D, Anderson KC. Combination of a novel HDAC6 inhibitor ACY-241 and anti-PD-L1 antibody enhances anti-tumor immunity and cytotoxicity in multiple myeloma. Leukemia. 2018 Mar;32(3):843-846.

#### P189 Tumor Treating Fields (TTFields) induce immunogenic cell death resulting in enhanced antitumor efficacy when combined with anti-PD-1 therapy

##### Noa Kaynan, PhD^1^, Tali Voloshin^1^, Shiri Davidi^1^, Yaara Porat^1^, Anna Shteingauz^1^, Mijal Munster^1^, Rosa Schnaiderman^1^, Catherine Tempel Brami^1^, Yaniv Alon^1^, Einav Zeevi^1^, Karnit Gotlib^1^, Roni Blat^1^, Orni Tal Yitzhaki^1^, Shay Cahal^1^, Aviran Itzhaki^1^, Eilon Kirson^1^, Uri Weinberg, MD PhD^1^, Adrian Kinzel^2^, Yoram Palti^1^, Moshe Giladi^1^

###### ^1^Novocure Ltd., Haifa, Israel; ^2^Novocure GmbH, Munich, Germany

####### **Correspondence:** Moshe Giladi (mgiladi@novocure.com)

**Background**

Tumor Treating Fields (TTFields) are a clinically applied anti-neoplastic treatment modality delivered via noninvasive application of low-intensity, intermediate-frequency, alternating electric fields. In this study we evaluated whether TTFields-induced cell death can be immunogenic and therefore suitable for combination with anti-PD-1 therapy.

**Methods**

Cancer cells were treated with TTFields using the inovitro(TM) system. Immunogenic cell death (ICD) was characterized by the exposure of calreticulin on the cell surface, secretion of ATP, and release of HMGB1. For detection of ER stress, phosphorylation of eIF2α was assessed. TTFields effect on autophagy was evaluated using electron microscopy, and evaluation of LC3. Bone marrow derived dendritic cells (DCs) were co-incubated with TTFields treated cells and phagocytosis by DCs and DCs maturation were evaluated. The combination of TTFields and anti-PD-1 was evaluated in short duration treatment protocol in orthotopic lung cancer model and long duration treatment protocol in subcutaneous colon cancer model. Analysis of infiltrating cells was performed using flow cytometry.

**Results**

We demonstrate that cancer cells that die during TTFields application exhibit ER stress leading to calreticulin translocation to the cell surface, as well as release of damage-associated molecular patterns including HMGB1 and ATP. Moreover, we show that TTFields treated cells promote phagocytosis by DCs, DCs maturation in vitro, and promote immune cells recruitment in vivo. We also show that the combined treatment of TTFields plus anti-PD-1 led to a significant decrease in tumor volume and significant increases in CD45+ tumor infiltrating cells in both tumor models. In the lung tumors, these infiltrating cells, specifically macrophages and DCs, demonstrated upregulation of surface PD-L1 expression following short treatment duration. Correspondingly, cytotoxic T-cells isolated from these tumors have shown higher levels of IFN-γ production relative to untreated mice. In the colon cancer tumors, significant increases in T-cell infiltration was observed following long treatment duration with TTFields plus anti-PD-1.

**Conclusions**

Our results demonstrate the potential of TTFields therapy to induce ICD. We also demonstrate robust efficacy of concurrent application of TTFields and anti PD-1 therapy in mouse models of cancer. These data suggest that combining TTFields with anti-PD-1 might achieve tumor control by further enhancing antitumor immunity.

**Acknowledgements**

The authors would like to thank Dr. Kenneth Swanson from Beth Israel Deaconess Medical Center, and Dr. Ilan Volovitz from Tel Aviv Sourasky Medical Center for their helpful and constructive comments.

**Ethics Approval**

This study was approved by Novocure’s Ethics Board and by the Israel National Ethics Board; approval numbers 160816, 21015, IL-17-3-131 and IL-19-1-38.

#### P190 Single-day CAR manufacturing platform using mRNA and Flow Electroporation Technology

##### Michael Kuo^1^, Robert Keefe, PhD^1^, Linhong Li, PhD^1^, Angelia Viley^1^, Mary Loveras^1^, Brian Mulhern^2^, Melanie Hartsough^3^, Claudio Dansky Ullmann, MD^4^, Dhana Chinnasamy^1^

###### ^1^MaxCyte Inc, Gaithersburg, MD, United States; ^2^Scilucent, Washington DC, United States; ^3^Hartsough Nonclinical Consulting, Gaithersburg, MD, United States; ^4^MaxCyte, Cambridge, MA, United States

####### **Correspondence:** Dhana Chinnasamy (dhanac@maxcyte.com)

**Background**

MaxCyte has developed a rapid and potent cell therapy that utilizes mRNA in transient Flow Electroporation (FEP) to produce gene-modified cell products, termed CARMA™. This proprietary CARMA platform modifies peripheral blood mononuclear cells (PBMC) from apheresis to generate a cryopreserved drug product in a single-day manufacturing process using the cGMP-compliant, closed MaxCyte GT® Transfection System, dramatically reducing the labor, facilities investment, and cost of raw materials typically required for such products. The CARMA one-day manufacturing process using cGMP grade mRNA has the potential to revolutionize cell therapy strategies by significantly reducing the wait time for patients receiving treatment.

**Methods**

We report here the implementation of the CARMA platform to manufacture MCY-M11, a PBMC cell therapy product expressing an anti-mesothelin chimeric antigen receptor (Meso-CAR) designed to target mesothelin-expressing solid malignancies. MCY-M11 expresses the Meso-CAR in all cells in the PBMC preparation, which are processed and cryopreserved without the need for prior activation or selective expansion. MCY-M11 for clinical application is manufactured under the appropriate cGMP quality systems and controls by MaxCyte at HCATS, a Contract Development Manufacturing Organization (CDMO). Manufacturing release specifications are preliminarily assigned, with multiple For Information Only (FIO) data points being accumulated during clinical production, while sufficient clinical data is being generated to establish meaningful release criteria.

**Results**

A total of 20 CARMA product development and engineering runs were performed during the technology transfer campaign, with analytical test methods and supply chain established. The viable cell yield from pre-FEP to post-FEP samples averaged around 92%. Meso-CAR expression in T and NK cell subsets in MCY-M11 ranged from 42-83% (average 73%) and 28-75% (average 59%), respectively. The anti-tumor bioactivity and target specificity of MCY-M11 was successfully established in vitro by demonstrating antigen-specific cytotoxicity and inflammatory cytokine release in co-culture assays with various mesothelin-expressing human tumor cell lines. Increased survival and efficacy were also demonstrated in vivo using a human mesothelin expressing ovarian syngeneic mouse tumor model.

**Conclusions**

The CARMA one-day manufacturing process using cGMP grade mRNA has the potential to revolutionize cell therapy strategies by significantly reducing the wait time for patients receiving treatment. MCY-M11 is currently being tested in a first-in-human clinical trial for advanced epithelial ovarian cancer and peritoneal mesothelioma (ClinicalTrials.gov Identifier: NCT03608618).

**Trial Registration**

NCT03608618

#### P191 IL-6 fuels durable memory for Th17-mediated responses to tumors

##### Hannah Knochelmann, BS^1^, Connor Dwyer, PhD^1^, Aubrey Smith, BS^1^, Megan Wyatt, MS^1^, Guillermo Rangel RIvera^1^, Jacob Bowers^1^, Michelle Nelson, PhD^1^, Gregory Lesinski, PhD, MPH^2^, Zihai Li, MD, PhD^3^, Mark Rubinstein, PhD^1^, Chrystal Paulos, PhD^1^

###### ^1^Medical University of South Carolina, Charleston, SC, United States; ^2^Emory University, Atlanta, GA, United States; ^3^Ohio State University, Columbus, OH, United States

####### **Correspondence:** Hannah Knochelmann (knochelm@musc.edu)

**Background**

Accessibility of T cell transfer therapies for most patients is hindered by cost and time required for product development. Our lab has shown that shortening ex vivo expansion of Th17 cells licenses a proinflammatory cell product which induces cytokine storm with high levels of systemic IL-6 in tumor-bearing hosts. Despite potential toxicity, briefly expanded Th17 cells eradicate large established tumors in low doses and generate durable memory against tumor rechallenge, suggesting a therapeutic benefit to the inflammatory state. Prior reports show that IL-6 promotes functional CD4+ T cell memory formation. Given that IL-6 is blocked clinically to manage cytokine release syndrome, we addressed the physiologic impact of IL-6 on efficacy and durability of Th17 cell therapy.

**Methods**

Th17 cells were expanded ex vivo using the TRP-1 transgenic mouse model in which CD4+ T cells express a TCR that recognizes tyrosinase-related protein 1 on melanoma. Naïve CD4+ T cells were polarized to the Th17 phenotype and infused into mice with B16F10 melanoma after a nonmyeloablative total body irradiation (5 Gy) preparative regimen. Serum cytokine levels were obtained by multiplex array and IL-6 signaling was inhibited with antibodies targeting the IL-6R and neutralizing IL-6 cytokine.

**Results**

Acute IL-6 blockade post Th17 cell transfer did not impact the primary response against melanoma nor the engraftment of Th17 cells. However, blocking IL-6 abrogated long-term responses increasing the frequency of tumor relapse upon secondary challenge and reduced survival. Mechanistically, IL-6 blockade reduced phosphorylation of STAT3 in transferred T cells associating with diminished Bcl-2 expression. The CD4+ compartment was reshaped by IL-6 blockade via promoting a greater frequency of FoxP3+ Treg cells in the peripheral blood, tumor and draining lymph nodes. Given the plasticity of Th17 and Treg cells, we assessed FoxP3 expression within the cell product 10 days post transfer and found that the frequency of FoxP3+ transferred cells was significantly heightened through IL-6 blockade.

**Conclusions**

IL-6 induced by Th17 cell therapy promotes an inflammatory over regulatory phenotype in vivo permitting durable memory against tumors. The expansion of tumor-specific regulatory cells from the transferred product is enhanced in the absence of IL-6 signaling. This work implies that the universal strategy of IL-6 inhibition for cytokine release syndrome may come at the expense of long-term efficacy for cell therapy approaches.

**Ethics Approval**

All animal studies were approved by MUSC's IACUC committee, approval number 0488.

#### P192 Anti-HLA-G antigen receptor T-cells exhibit potent anti-tumor effects against human solid tumors

##### Alan Epstein, MD, PhD, Aida Kouhi, Aida Kouhi

###### University of Southern California, Los Angeles, CA, United States

####### **Correspondence:** Alan Epstein (aepstein@usc.edu)

**Background**

HLA-G is highly expressed on human placenta during pregnancy and has been found to suppress the NK response to cells that lose their HLA and/or beta2-microglobulin expression. [1-2]. In addition, except for pregnancy, HLA-G is rarely expressed in normal adult tissues. Moreover, roughly 50% of human solid tumors lose their HLA expression to avoid detection by the human immune system. [3] HLA-G is therefore an outstanding target for CAR T-cells since, like the placenta, it is up-regulated in HLA-negative tumors to suppress NK destruction. [4] We have successfully generated anti-HLA-G CAR T-cells to treat solid tumors that express HLA-G.

**Methods**

An anti-HLA-G CAR construct was generated by fusing anti HLA-G scFv to a second generation CAR containing the CD8α leader sequence, 4-1BB co-stimulation sequence, and CD3ζ signaling domain. The CAR vector was then fused with a lentivirus vector in-frame with the CAR backbone, and was used to transduce primary human CD3 positive T-cells. After transduction, expanded CAR-T cells were characterized for their ability to bind HLA-G antigen and HLA-G positive SKOV-3 cells (human ovarian cancer model) using flow cytometry.

**Results**

Expanded CAR-T cells were able to bind successfully both the HLA-G antigen and SKOV-3 cells in vitro. Expanded CAR-T cells were then co-cultured with SKOV3-Luc cells and studied for their epitope-driven cytotoxicity. Anti HLA-G CAR T-cells displayed dose-dependent cytotoxicity when co-cultured with tumor cells. We have recently developed an in vivo model of ovarian cancer that can be used for testing the efficacy of our CAR-T cells. In this model, NSG mice are injected with 2 million SKOV3-Luc cells intraperitoneally (ip). Seven-10 days after injection, tumors are visible when observed by bioluminescence imaging, at which time the treatment group will receive an ip injection of anti HLA-G CAR-T cells. Since ovarian cancer rapidly metastasizes to the peritoneum, the aforementioned model should provide relevant clinical data that can be translated to patients, and like hematopoietic cancers, will present antigen quickly after injection of CAR T-cell to keep them stimulated and functional.

**Conclusions**

We are currently testing the efficacy of anti-HLA-G CAR-T cells in vivo using this ip model, and plan to show that HLA-G as a pan tumor target will provide selective and specific cell based therapy which may in the near future be clinically relevant for chemotherapy resistant ovarian cancer and other tumors.

**Acknowledgements**

This work is supported by Cell Biotherapy, Inc., Los Angeles, CA.

**References**

1. Apps R, Gardner L, Moffett A. A critical look at HLA-G. Trends Immunol. 2008; 29:313–321.

2. Jurisicova A, Casper RF, MacLusky NJ, Mills GB, Librach CL. HLA-G expression during preimplantation human embryo development. Proc. Natl. Acad. Sci. U. S. A. 1996; 93:161–5.

3. de Kruijf EM et al. HLA-E and HLA-G expression in classical HLA class I-negative tumors is of prognostic value for clinical outcome of early breast cancer patients. J. Immunol. 2010; 185:7452–9.

4. Lin, A. & Yan, W.-H. Human Leukocyte Antigen-G (HLA-G) Expression in Cancers: Roles in Immune Evasion, Metastasis and Target for Therapy. Mol. Med. 2015; 21:782–791.

**Ethics Approval**

This study was approved by the IRB of the University of Southern California protocol #HS-16-00029 on 2-29-16.

#### P193 CoStAR (Costimulatory Antigen Receptor) enhancement of tumour infiltrating lymphocyte therapy

##### Gray Kueberuwa, PhD, John Bridgeman, PhD, Martina Sykorova, Milena Kalaitsidou, Michelle Le Brocq, Robert Hawkins

###### Immetacyte, Manchester, Manchester County, United Kingdom

####### **Correspondence:** Robert Hawkins (r.hawkins@immetacyte.com)

**Background**

The efficacy of TIL therapy is limited in some patients due to the failure of the cells to respond to tumour sufficiently or persist long enough to have a necessary anti-tumour effect. We have addressed this issue by developing Co-stimulatory receptors (CoStARs) that provide enhanced signaling to tumour-specific T-cells upon encountering tumour associated antigens.

Since tumour reactivity is determined by natural TCRs that have undergone thymic selection, this approach does not bear with it the risks of other therapies targeting tumour antigens expressed on the cell surface

**Methods**

In order to identify optimal signalling domain for Co-StAR molecules, several iterations of our prototype receptor were synthesised. The ability of each to enhance T-cell activation, proliferation, secretion of cytokines and increase resistance to apoptosis were assessed.

To explore if this approach has the potential of wide applicability, we went on to assess targeting of two additional ovarian cancer tumour associated antigens. To achieve this, the antigen binding moiety was exchanged and signaling domain kept constant.

**Results**

We show that colorectal cancer specific Co-StAR significantly enhances the number of T-cells expressing IL2, TNFα, 41BB, CD107a and bcl-xL by factors ranging from 2-4 fold in model systems. This shows an increase in activation, effector function and resistance to apoptosis. We also identified an optimal signaling domain that caused the greatest magnitude of enhancement for the above factors.

Observations of Co-StAR enhancement were mirrored in model systems for ovarian cancer, targeting two separate ovarian cancer tumour associated antigens.

In addition, stimulation assays showed that Co-StAR with optimal signaling domain increased T-cell proliferation over 3 weeks in comparison to prototype Co-StAR, Co-StAR that binds an irrelevant target, or indeed, mock transduced T-cells.

**Conclusions**

Our optimal Co-StAR provides a means to effectively deliver “signal 2” to T-cells. Enhancing activation, effector functions and resistance to apoptosis upon contact with target tumour cells.

Since T-cell activation primarily requires “signal 1”, application to of Co-StAR to enhance TIL therapy, which works through natural, thymically selected TCRs, provides a means to increase the activity of tumour-reactive TIL without risking severe off-tumour side effects.

Immetacyte is assessing this approach in our current Phase I/II clinical trial of TIL in ovarian cancer patients (EudraCT–2019-000106-30).

**Acknowledgements**

This research was supported by IUK projects 133299 and 104468

**Ethics Approval**

This study was approved by the South Central Research Ethics Committee : 19/SC/0355

#### P194 Use of stimulatory cells in conjunction with IL-12 and IL-18 augments NK cell expansion and transduction, drives a memory phenotype, and improves in vitro and in vivo CAR NK activity

##### Anmol Vohra, MS, Katherine Jamboretz, MS, Sasha Lazetic, Denise Gonzalez, Daofeng Liu, PhD, Ivan Chan, PhD, James Trager, PhD

###### Nkarta Inc, South San Francisco, CA, United States

####### **Correspondence:** James Trager (jtrager@nkartatx.com)

**Background**

NK cells have been expanded on K562 stimulatory cells expressing membrane-bound (mb) IL-15 and 41BBL for clinical use, and can be genetically modified to express activating chimeric receptors [1,2,3]. Engineered NK cells targeting CD19 or ligands of NKG2D show in vitro and in vivo cytotoxicity against relevant tumor targets that can overcome endogenous resistance to NK cells. NK cells activated in the presence of IL-12, IL-15 and IL-18 develop cytokine induced memory-like phenotype and function; these cells have shown clinical promise [4]. Here we describe NK cell function and phenotype achieved by combining the robust driven by K562-mbIL15-41BBL with the induction of a cytokine-induced memory phenotype achieved after exposure to IL-12 and IL-18.

**Methods**

Healthy donor PBMC NK were expanded on K562-mbIL15-41BBL stimulatory cells with IL-2 alone or with IL-2 plus IL-12 and IL-18 (12-18). We compared NK cell expansion, cytokine secretion, cytotoxicity against tumor lines at various time points, and persistence in culture over 4 weeks. The expanded NK were transduced with CD19 and NKG2D CAR constructs, and the resulting cells evaluated for CAR expression, cytotoxicity and in vivo efficacy against relevant cell lines.

**Results**

Addition of 12-18 to the K562-mbIL15-41BBL stimulatory cells improves NK expansion 2-3 fold [If we have a p value, it’s better to give it:<br>‘…significantly improves NK expansion 2-3 fold (p<x) relative to that…’][Will put together later for the poster]relative to that achieved using the stimulatory cell line alone, while NK cell cytotoxicity is unchanged. IFNγ and TNFα production and transduction efficiency are also improved in this setting. Over 3 weeks of culture following brief exposure to 12-18, NK cell phenotype changes, with an increased percentage of CD62L+ and NKG2C+ cells, and increased NKG2C expression per NK cell. While the NKG2D-CAR driven cytotoxic activity is unchanged by 12-18 at 14 days post-exposure, cytotoxic activity increases in these cells by day 21. In addition, this improved cytotoxicity at day 21 is reflected by improved CD19-CAR driven in vivo activity against the CD19+ tumor target [Raji or Nalm6?]Nalm6 [Oops, Nalm6, thx.] with an increased presence of circulating NK cells over 4 weeks in the mice.

**Conclusions**

The data demonstrates that the addition of IL-12 and IL-18 to K562-mbIL15-41BBL stimulatory cells during NK expansion maintains in vitro cytotoxicity and improves expansion, transduction, persistence, and in vivo efficacy. Further, IL-12 and IL-18 drive development of a memory phenotype and more sustained NK cell function over the course of several weeks. This activation system may allow for development of more robust and potent engineered NK cells for clinical use.

**References**

1. Lapteva N, Durett AG, Sun J, Rollins LA, Huye LL, Fang J, Dandekar V, Mei Z, Jackson K, Vera J, Ando J, Ngo MC, Coustan-Smith E, Campana D, Szmania S, Garg T, Moreno-Bost A, Vanrhee F, Gee AP, Rooney CM. Large-scale ex vivo expansion and characterization of natural killer cells for clinical applications. Cytotherapy. 2012;14(9):1131-1143

2. Chihaya I, Iwamoto S, Campana D. Genetic modification of primary natural killer cells overcomes inhibitory signals and induces specific killing of leukemic cells. Blood. 2005; 106:376-383.

3. Yang Y, Connolly J, Shimasaki N, Mimura K, Kono K, Campana D. A Chimeric Receptor with NKG2D Specificity Enhances Natural Killer Cell Activation and Killing of Tumor Cells. Cancer Res. 2013;73(6):1777-1786

4. Romee R, Rosario M, Berrien-Elliott MM, Wagner JA, Jewell BA, Schappe T, Leong JW, Abdel-Latif S, Schneider SE, Willey S, Neal CC, Yu L, Oh ST, Lee YS, Mulder A, Claas F, Cooper MA, Fehniger TA. Cytokine-induced memory-like natural killer cells exhibit enhanced responses against myeloid leukemia. Sci Trans Med. 2016;8(357): 357ra123

**Ethics Approval**

Animal studies were conducted and approved by the Explora IACUC committee.

#### P195 Loss of function of the TSC1-TSC2 complex renders tumors eligible for GD3 CART therapy

##### Ancy Thomas^1^, Saurav Sumughan^1^, Zhussipbek Mukhatayev^1^, Emilia Dellacecca^1^, Nicola Lancki^1^, Levi Barse^2^, Jesus Zamora-Pineda^2^, Suhail Akhtar^2^, Maria Picken^2^, Denise Scholtens^1^, Daniel Dilling^2^, Richard Junghans, PhD, MD^3^, Caroline Le Poole^1^

###### ^1^Northwestern University, Chicago, IL, United States; ^2^Loyola University, Maywood, IL, United States; ^3^Boston University, Boston, MA, United States

####### **Correspondence:** Caroline Le Poole (caroline.lepoole@northwestern.edu)

**Background**

Benign tumors can arise from bi-allelic mutations in a single gene. In tuberous sclerosis complex (TSC) and lymphangioleiomyomatosis (LAM), tumors do not acquire additional mutations, and patients are not eligible for therapeutics that rely on neoantigen formation. However, the affected gene is responsible for several predictable phenotypic changes. As mTOR hyperactivity resulting from mutations in TSC1 or TSC2 is associated with overexpression of some melanoma-associated antigens, de novo expression of ganglioside D3 expression may render the resulting, benign tumors eligible for immunotherapy.

**Methods**

We probed the expression of GD3 in human TSC lesions of the lungs, kidneys, skin and brain by immunostaining and monitored anti-GD3 titers in serum by ELISA. Infiltration by NK cells and NKT was measured to look for natural responses to the cell surface antigen. We next isolated tumors cells from TSC2 heterozygote mice and confirmed loss of heterozygosity by genotyping before challenging groups of 10 SCID/beige mice in 2 repeat experiments, and subjecting them to adoptive transfer by GD3-CART cells and measured tumor sizes over time. Similarly we treated groups of 8 ageing TSC mice >16 months of age by adoptive GD3 CART-cell transfer, and measured surface tumor growth on internal organs.

**Results**

We found consistent overexpression of GD3 in tissues from TSC patients compared to healthy controls. GD3 overexpression was not accompanied by an influx of NK(T) cells, and anti-GD3 titers were reduced rather than elevated in patients, supporting the concept that slow growing tumors in TSC patients are not immunogenic. However, CART cells responsive to GD3, supplemented by IL-2, mediated prolonged and significant anti-tumor responses in both immunodeficient and immune competent hosts. The majority of TSC2 heterozygote mice treated by CAR T cells displayed no tumors at end point, versus all mice treated with untransduced T cells.

**Conclusions**

These promising results infer that adoptive transfer of transgenic T cells can offer an effective strategy to not only prevent further tumor growth as rapamycin therapy does, but also to treat and even eliminate arising tumors. This strategy might offer a cure for patients with LAM, a disease that hits women in the prime of their lives.

**Acknowledgements**

Studies supported by a DoD Tuberous Sclerosis Complex Research Program Clinical Translational Research Award to CLP.

**Ethics Approval**

All animal experiments were approved by the Animal Care and Use Committee of Northwestern University and followed the institutional guidelines; protocol number IS00008259.

#### P196 The first step toward the universal cell therapy: Simultaneous removal of HLAs (Human leukocyte antigens) using CRISPR-mediated quadruple genome editing in allogeneic T cells

##### Jeewon Lee, Ph D, Munkyung Kim, Joong Hyuk Sheen, Jihye Ryu, Yu Young Kim, Okjae Lim

###### MOGAM Institute for Biomedical Research, Yongin-si, Gyeonggi-do, Republic of Korea

####### **Correspondence:** Okjae Lim (blubelle@mogam.re.kr)

**Background**

Chimeric antigen receptor (CAR) T cell therapy is the revolutionary treatment of choice for hematologic malignancies. Currently approved CAR T therapies require patients’ own immune cells, and this autologous T cell manufacturing process involves certain limitations primarily derived from the nature of individualized therapy. Thus, engineering allogeneic donor cells to evade host immune rejection is required for a broader clinical application of the therapy.

**Methods**

In this study, we attempted to inhibit expression of both HLA I and II through the CRISPR/Cas9 gene editing system to reduce allo-reactive immune rejection response. First, we screened 60 gRNAs targeting B2M and 60 gRNAs each targeting alpha chains of HLA-II molecules (DP, DQ and DR, respectively) to find gRNA sequences efficiently ablate expression of HLA molecules on T cell surface. Next, we investigated whether the absence of HLA-I/II expression in donor T cells could alleviate immune response from allogeneic responders using in vitro mixed lymphocyte reaction (MLR) assays.

**Results**

We have identified gRNA sequences highly efficient in targeting B2M and alpha chains of HLA-II molecules without carrying off-target effects. Selected gRNA sequences for HLA-II ablation covered the vast majority of each HLA-II alpha chain allele. HLA-I/II double negative T cells generated by simultaneous quadruple genome editing with the selected gRNAs maintained their phenotypes and cytotoxicity upon TCR stimulations compared to the control cells treated with non-target gRNA. Furthermore, the MLR assays showed that IFN- and TNF-α production in allo-responder T cells was significantly decreased in the absence of donor HLA-I alone and was further diminished in response to HLA-I/II double negative donor T cells compared with the control cells, implicating prolonged survival of the adoptively transferred immune cells.

**Conclusions**

In conclusion, we have identified novel gRNA sequences ablating expression of HLA molecules on donor T cell surfaces to dramatically reduce donor-derived allo-responses, establishing an essential cornerstone towards the universal T cell therapy.

**Ethics Approval**

Human PBMCs were obtained from healthy volunteers by leukapheresis from the Samsung Medical Center (SMC) under IRB approval (SMC IRB no.2018-01-089).

#### P197 The development of an autologous neoantigen specific T cell product from peripheral blood, NEO-PTC-01, through the ex-vivo induction protocol, NEO-STIM™

##### Divya Lenkala, MS^1^, Marit Van Buuren, PhD^1^, Brian McCarthy^1^, Jessica Kohler, PhD^1^, Michael Nelson^1^, Flavian Brown^1^, Yvonne Ware, MS^1^, Yuting Huang, MS^1^, Janani Sridar^1^, Yusuf Nasrullah, MS, United Kingdom^1^, Dewi Harjanto^1^, Joost Van Den Berg, PharmD^2^, Matthew Goldstein, MD, PhD^3^, Richard Gaynor, MD^1^

###### ^1^Neon Therapeutics, Cambridge, MA, United States; ^2^Netherlands Cancer Institute, Amsterdam, Netherlands; ^3^Tango Therapeutics, Cambridge, MA, United States

####### **Correspondence:** Marit Van Buuren (mvanbuuren@neontherapeutics.com)

**Background**

Neoantigens are tumor-specific antigens that have been shown to be important in the anti-tumor immune response. These antigens are not subject to central immune tolerance and are therefore potentially more immunogenic than tumor-associated antigens. The goal of our studies is to generate neoantigen specific T cell responses and perform detailed characterization of the induced T cell responses towards these neoantigen targets to assess the applicability of the approach for adoptive cell therapy.

**Methods**

Patient-specific neoantigens were predicted using our RECON® bioinformatics platform, and the predicted high-quality neoantigens were utilized in our proprietary ex-vivo stimulation protocol, NEO-STIM to assess immunogenicity. NEO-STIM is used to prime, activate and expand memory and de novo T cell responses from both the CD4+ as well as the CD8+ compartment. In-depth analysis was performed to characterize the specificity, functionality (cytokine production and cytolytic capacity) and diversity of the induced T cell responses through high throughput flow cytometric analysis.

**Results**

Here we present the successful induction of memory and de novo CD8+ and CD4+ T cell responses in peripheral blood mononuclear cells isolated by leukapheresis from five melanoma patients using NEO-STIM. We then extensively characterized these T cell responses and show that these responses are functional, specific and have cytolytic capacity.

**Conclusions**

NEO-STIM is a novel platform to understand in detail the immunogenic potential of high-quality neoantigen-targets. Moreover, this platform can be utilized to generate T cell products from peripheral blood for adoptive cell therapy for patients with a variety of solid tumors.

**Ethics Approval**

The samples for the study were collected under ClinicalTrials.gov: NCT02897765 and N16NEON protocol

#### P198 Short-lived and extended half-life target modules for redirecting UniCAR T-cells against sialyl-Tn expressing cancer cells

##### Liliana Loureiro, PhD^1^, Anja Feldmann, PhD^1^, Ralf Bergmann^1^, Stefanie Koristka^1^, Nicole Berndt^1^, Nikolett Hegedüs^2^, Domokos Máthé^2^, Paula Videira^3^, Michael Bachmann^1^, Claudia Arndt^1^

###### ^1^Helmholtz-Zentrum Dresden-Rossendorf, Dresden, Germany; ^2^Semmelweis University, Budapest, Hungary; ^3^Faculdade de Ciências e Tecnologia/UNL, Caparica, Portugal

####### **Correspondence:** Liliana Loureiro (l.loureiro@hzdr.de)

**Background**

The development of chimeric antigen receptors (CARs) has rapidly emerged as a promising approach in cancer immunotherapy. Nonetheless, drawbacks associated with CAR T-cell therapies include on-target/off-tumor effects and cytokine release syndrome. Aiming an increased clinical safety while preserving the efficacy of such therapy, we developed a novel modular universal CAR platform termed UniCAR. UniCAR T-cells are exclusively activated in the presence of a target module (TM), which establishes the cross-link between antigen-specific cancer cells and UniCAR T-cells in an individualized time- and target-dependent manner. The carbohydrate antigen sialyl-Tn (STn) is a particularly interesting target due to its expression in several types of cancer and absence in normal healthy tissues. Given the small size of such TMs, they are rapidly eliminated and thus, possible side effects and activation of UniCAR T-cells can be easily controlled by TM dosing. In late phases of treatment, TMs with extended half-life may play an important role by improving the eradication of residual tumor cells.

**Methods**

In this work, a novel longer-lasting TM against STn was developed, characterized and compared to the previously developed short-lived anti-STn TM [1]. Short-lived TMs are composed of a tumor-specific binding moiety fused to the La peptide epitope (E5B9) which is recognized by UniCAR T-cells. In extended half-life TMs, these two components are fused via an Fc domain derived from the human IgG4 molecule. Functional and pharmacokinetic properties were assessed using in vitro and in vivo assays.

**Results**

The developed anti-STn IgG4-based TM efficiently activates and redirects UniCAR T-cells to STn-expressing tumors in a highly efficient target-specific and target-dependent manner, promoting the secretion of pro-inflammatory cytokines, tumor cell lysis of breast and bladder cancer cells in vitro and of breast cancer cells in experimental mice. A comparable or increased killing efficiency was obtained at a lower concentration range in comparison to the results obtained for the anti-STn scFv-based TM. Additionally, PET studies demonstrate the specific enrichment of the anti-STn IgG4-based TM at the tumor site presenting a prolonged serum half-life compared to the scFv short-lived TM.

**Conclusions**

Taken together, these data demonstrate the effective and potential application of this CAR T-cell-derived modular system to target STn in different types of cancer using different TM formats. The use and combination of such molecules with different formats and half-lives provides highly promising and customized tools for retargeting of UniCAR T-cells in a flexible, individualized and safe manner at different stages of treatment.

**Reference**

1. Loureiro L R, Feldmann A, Bergmann R, Koristka S, Berndt N, Arndt C, Pietzsch J, Novo C, Videira P, Bachmann M. Development of a novel target module redirecting UniCAR T cells to Sialyl Tn-expressing tumor cells. Blood Cancer J. 2018; 8(9): 81.

**Ethics Approval**

All animal activities and procedures were performed in accordance with the protocols approved by the Institutional Review Board at Semmelweis University - Budapest, approval number PE/EA/50-2/2019.

#### P199 Generation of functionally and phenotypically mature, allogeneic natural killer cells from human induced pluripotent stem cells under chemically-defined, feeder- and serum- free culture conditions

##### Kyle Lupo, BS, Andrea Chambers, MS, Sandro Matosevic, PhD

###### Purdue University, Lafayette, IN, United States

####### **Correspondence:** Sandro Matosevic (sandro@purdue.edu)

**Background**

While targeted immunotherapy with engineered natural killer (NK) cells has emerged as a promising approach for the treatment of solid tumors, challenges in sourcing, processing, and genetically modifying blood-derived NK cells limit the potential for developing life-saving treatments for cancer patients. As an alternative, the use of induced pluripotent stem cells (iPSCs) offers a promising approach to overcoming existing challenges faced when engineering NK cell-based immunotherapies. However, approaches for generating NK cells from iPSCs described so far have several shortcomings: they utilize sera or feeder layers to adapt iPSCs or culture hematopoietic progenitors, take months to complete, and rely on individualized, and thus highly variable, iPSC reprogramming protocols, limiting their utility.

**Methods**

We have generated NK cells from iPS cells using a novel feeder-free differentiation protocol starting from either centrally-validated and banked iPSC lines or iPSCs reprogrammed from donor fibroblasts. Our protocol utilizes a two-step, entirely feeder-free procedure involving hematopoietic progenitor generation followed by NK differentiation. These differentiated NK cells have been characterized for inhibitory and activating marker expression, IFN-γ production, degranulation, and cytotoxicity against a number of solid tumor targets, including primary patient-derived glioblastoma cells. Moreover, these cells were expanded in culture and manipulated to generate a cytotoxic infusible cell therapy product.

**Results**

iPS cells were differentiated into hematopoietic progenitor cells, yielding CD34+/CD45+ and CD34+/CD43+ cell populations at yields consistent with results described in literature using feeder-based protocols [1]. Following four weeks of NK cell differentiation, cells showed high expression of several NK cell maturation markers as well as inhibitory and activating receptors (CD56+/CD3-, NKG2D, NKp30, NKp44, NKp46, DNAM-1, CD16, CD94/NKG2A, and CD158b). iPSC-NK cells derived using our protocol were also similar to blood-derived NK cells in morphology, expansion rate, and functionality (in terms of cytotoxicity and degranulation potential). By using centrally-validated iPSC lines, we further demonstrate our ability of avoiding donor-specific reprogramming protocols.

**Conclusions**

We developed a new protocol for the generation of NK cells from iPSCs that is entirely feeder and serum-free and can be extended to the use of validated iPSC lines avoiding donor and reprogramming variability. iPSC-derived NK cells using our protocol exhibit characteristics of mature blood-derived NK cells and powerful cytotoxicity against solid tumor targets. Additionally, these cells offer the advantage of increased expansion rates, improved ease of transfection while in the iPSC state, and are free of contaminating T-cells associated with GvHD risk, overcoming many limitations of existing NK cell based immunotherapies.

**Reference**

1. Bock A, Knorr D, and Kaufman D. Development, Expansion, and In Vivo Monitoring of Human NK Cells from Human Embryonic Stem Cells (hESCs) and Induced Pluripotent Stem Cells (iPSCs). Journal of Visualized Experiments : JoVE 74 (2013): 50337.

#### P200 CART-engineered Marrow-infiltrating Lymphocytes (MILsTM) are more polyfunctional than their matched peripheral blood counterparts

##### Eric Lutz, PhD^1^, Lakshmi Rudraraju, MS^1^, Elizabeth DeOliveira^1^, Srikanta Jana^1^, Jing Zhou, MD, PhD^2^, Sean Mackay, MBA^2^, Ivan Borrello, MD^3^, Kimberly Noonan, PhD^1^

###### ^1^WindMIL Therapeutics, Baltimore, MD, United States; ^2^Isoplexis, Branford, CT, United States; ^3^Johns Hopkins University, Baltimore, MD, United States

####### **Correspondence:** Eric Lutz (lutz@windmiltherapeutics.com)

**Background**

WindMIL Therapeutics is developing Marrow-infiltrating Lymphocytes (MILsTM), a novel form of adoptive T cell therapy composed of bone marrow-derived, patient-autologous, polyclonal CD4 and CD8 T cells [1]. Genetically unmodified MILsTM have demonstrated antitumor activity in patients with multiple myeloma [2] and are being developed for several other tumor types. Distinguishing features of T cells from bone marrow compared to T cells from peripheral blood lymphocytes (PBLs) include their memory phenotype, inherent tumor antigen-specificity, higher CD8:CD4 ratio and ability to persist long-term [3]. Based on these differences, we hypothesized that MILsTM would provide a more robust platform for CAR-T therapy compared to PBLs. We have previously shown that CAR-modified MILsTM (CAR-MILsTM) demonstrate superior killing of tumor target cells in vitro compared to CAR-T cells generated from patient-matched PBLs (CAR-PBLs) [4]. In this study, we compared, at the single cell level, functionality of patient-matched CAR-MILsTM and CAR-PBLs following antigen-specific in vitro stimulation.

**Methods**

CAR-MILsTM and CAR-PBLs engineered to express a BCMA-specific, 4-1BB/CD3z-signaling CAR were produced using cryopreserved lymphocytes from the bone marrow and blood of six patients with multiple myeloma. CD4 and CD8 T cells isolated from the CAR-MILsTM and CAR-PBLs products were stimulated with K562 cells transduced with either BCMA (K562-BCMA) or nerve growth factor receptor (K562-NGFR) at a ratio of 1:2 for 20 hrs. After 20 hrs of co-culture, T cells were enriched and loaded into IsoCode chips containing ~12,000 microchambers pre-patterned with a 32-plex antibody array. Protein secretion from 1000-2000 single T cells per product was detected by a fluorescence ELISA-based assay and single cell polyfunctional profiles analyzed using IsoPeak (IsoPlexis).

**Results**

CD4 and CD8 T cells from both CAR-MILsTM and CAR-PBLs demonstrated an antigen-specific increase in polyfunctionality (secretion of 2+ cytokines per cell) and polyfunctional strength index (PSI) in response to BCMA stimulation compared to NGFR control. When compared to CAR-PBLs, CAR-MILsTM demonstrated increased polyfunctionality and increased PSI in both CD4 and CD8 T cells. The enhanced PSI in CAR-MILsTM was predominated by effector, stimulatory and chemoattractive proteins associated with antitumor activity including Granzyme B, IFNg, IL-8, MIP1a and MIP1b. Coincidentally, increased PSI and enhanced secretion of these same proteins was reported to be associated with improved clinical responses in patients with Non-Hodgkin lymphoma treated with CD19-specific CAR-T therapy [5].

**Conclusions**

Based on these data and the inherent antitumor properties of MILsTM, we speculate that CAR-MILsTM would be more potent and effective than currently approved CAR-T products derived from PBLs.

**References**

1. Borrello I and Noonan KA, Marrow-Infiltrating Lymphocytes – Role in Biology and Cancer Therapy. Front Immunol. 2016 March 30; 7(112)

2. Noonan K.A., Huff C.A., Davis J., Lemas M. V., Fiorino S., Bitzan J., Ferguson A., Emerling A., … Borrello I. Adoptive transfer of activated marrow-infiltrating lymphocytes induces measurable antitumor immunity in the bone marrow in multiple myeloma. Sci. Transl. Med. 2015; 7: 288ra78.

3. Noonan KA, Rudraraju L, Hoyos V, Lutz E and Borrello I. Persistence of Non Gene-Modified Adoptively Transferred Marrow Infiltrating Lymphocytes (MILs) More Than Five Years Post Transfer. Blood 2016 128:4552.

4. Lutz ER, Hoyos V, Rudraraju L, DeOliveira E, Jana S, Weiss I, Borrello IM, Noonan K. Marrow-infiltrating Lymphocytes (MILs) provide a robust platform for CAR-T cell therapy. Blood 2018 132:3337.

5. Rossi J, Paczkowski P, Shen Y, … Bot A. Preinfusion polyfunctional anti-CD19 chimeric antigen receptor T cells are associated with clinical outcomes in NHL. Blood 2018 132(8):804-814.

**Ethics Approval**

The study was approved by the Johns Hopkins University IRB.

#### P201 Comparison of phenotype and anti-tumor profile of CD19-CAR-T cells generated from either umbilical cord blood- or peripheral blood-derived T lymphocytes

##### Cristina Maccalli, PhD^1^, Dhanya Kizhakayil, PhD^1^, Shilpa Ravindran, BSc^1^, Saad Rasool^1^, Rebecca Mathew^1^, Valentina Mattei^1^, Monica Casucci, PhD^2^, Sara Deola, MD, PhD^1^, Chiara Cugno, MD^1^, Damien Chaussabel^1^, Sara Tomei, PhD^1^, Christof von Kalle^1^,

###### ^1^Sidra Medicine, Doha, Qatar; ^2^San Raffaele Scientific Institute, Milan, Italy

####### **Correspondence:** Cristina Maccalli (cmaccalli@sidra.org)

**Background**

T lymphocytes expressing antigen-specific chimeric receptors (CARs) have been revealed as a powerful therapeutic approach for aggressive and refectory childhood and adult B cell malignancies. Umbilical cord blood cells (UCB), with their unique capacity of broad leukocyte antigen (HLA)-matching, can represent an appealing starting material for the generation of “off-the shelf” CAR-T cells to render this type of therapy accessible to a large number of cancer patients.

**Methods**

CAR-T cells have been generated from either UCB (N=5, ALLCELLS, USA) and peripheral blood lymphocytes (PBLs; N=2) from healthy donors. In vitro enriched T cells have been transduced with CD19-CD28z-CD3z and CD19-4-1BBz-CD3z encoding lentiviral vectors (LVs). Deep phenotype characterization of these CAR-T cells has been performed utilizing an in-house designed IF multiparametric (28 markers) panel. Functional assays have been performed to assess cytokine (IFN-gamma, IL2, IL-5 and IL-17), perforin and granzyme B release (EliSpot or multicolor FluoroSpot) and cytotoxic activity (Delfia assay) by CAR-T cells following the co-culture with CD19+ or CD19- target cells. In addition, transcriptomic modular repertoire [1-3] has been applied by parallel quantitative PCR using the high throughput BioMark HD platform to determine gene expression profile of CAR-T cells described above.

**Results**

Efficient LV transduction was achieved for UCB-T cells, although requiring higher MOI as compared to PBL (25 vs. 5; 66-80 vs. 70-88 % of transduction, respectively). The frequency of CD4+ transduced T cells (45-59% of positive cells) was superior in UCB as compared to PBL (27-36% of positive cells) while transduced CD8+ T cells were 18-20 and 40-67%, respectively. CB-CAR-T cells were enriched of CD45RA+CCR7+CD27+CD62L+ T cells. These cells co-expressed ICOS and 4-1BB but these molecules were not detectable on PBL-CAR-T cells. Markers associated with late differentiation/exhaustion of T cells, such as LAG-3 and TIM-3, were found only on PBL-CAR-T cells. PD-1 was expressed at higher levels in CB- vs. PBL-derived CAR-T cells (15-40% and 40-60% in CD45RA+ and CD45RO+ T cells, respectively).

Antigen-specific reactivity was shown by CAR-T cells either isolated from UCB or PBL against acute lymphoblastic leukemia or EBV-B cells overexpressing CD19.

Interestingly, differential gene expression profiles were assessed through the comparison of UCB- vs. PBL-derived CAR-T cells and their co-culture with antigen-specific target cells. Genes differentially expressed in CD19-CD28z-CD3z vs. CD19-4-1BBz-CD3z CAR-T cells were also found.

**Conclusions**

Taken together, these results proved that anti-tumor early differentiated/central memory CAR-T cells can be efficiently isolated from UCB with distinctive phenotype as compared to PBL-CAR-T cells.

**References**

1 Chaussabel, D. and N. Baldwin. Democratizing systems immunology with modular transcriptional repertoire analyses. Nat Rev Immunol, 2014, 14:271-80;

2 Altman MC., Rinchai D., Baldwin N., Whalen E., Garand M., Ahamed Kabeer B., et al. A Novel Repertoire of Blood Transcriptome Modules Based on Co-expression Patterns Across Sixteen Disease and Physiological States. 2019, bioRxiv; doi: https://doi.org/10.1101/525709.

3 Altman MC., Baldwin N., Whalen E., Al-Shaikhly T., Presnell S., Khaenam P., et al. A Transcriptome Fingerprinting Assay for Clinical Immune Monitoring. 2019, bioRxiv; doi: https://doi.org/10.1101/587295.

#### P202 Mechanisms underlying human placental CD34+-derived natural killer cell cytotoxicity against glioblastoma

##### Tanel Mahlakoiv, PhD, Bhavani Stout, Valentina Rousseva, Irene Raitman, Lin Kang, PhD, Robert Hariri, Xiaokui Zhang, William van der Touw

###### Celularity, Warren, NJ, United States

####### **Correspondence:** William van der Touw (william.vandertouw@celularity.com)

**Background**

Natural killer (NK) cells are innate immune cells with a critical role in immune surveillance against cell transformation and tumor development. NK cells express an array of unique activating and inhibitory receptors whose aggregate signaling determine activation of NK cell effector function. Adoptive transfer of NK cells has demonstrated the potential to induce antitumor responses in the clinic. Celularity has developed a platform for generating cytotoxic NK cells from placental CD34+ cells (PNK cells) for adoptive cancer immunotherapy. Although PNK cells demonstrate cytotoxicity against diverse cancer cell types, their activating mechanisms are little characterized. In this study, we explore the contribution of specific signaling pathways and upstream NK cell receptors involved in PNK cell cytotoxicity against glioblastoma multiforme (GBM) cell targets.

**Methods**

PNK cells were transcriptionally profiled using scRNAseq and qRT-PCR to identify candidate pathways regulating cytolytic function. Expression of major receptors and intracellular signaling molecules were analyzed using flow cytometry and western blot. PNK cell phenotype was compared to circulating NK cells. PNK cytotoxicity was evaluated against GBM cell lines (LN-18 and U251) in xCELLigence platform and a degranulation assay using CD107a staining. The role of key signaling pathways driving PNK effector functions was analyzed in cytolysis assays using small molecule inhibitors of Src kinases, SYK, PLC-γ, PI3K and MAP kinases, including JNK, p38 and ERK.

**Results**

PNK cells highly express genes mediating NK cell effector functions, including NCR1, NCR2, NCR3, KLRK1 and CD226. Flow cytometry demonstrated increased expression of NKp44 (99.7±0.2% vs. 69.3±5.2%), NKG2D (68.7±7.9% vs. 44.6±5.8%) and GITR 99.7±0.2% vs. 15.0±3.2%) on PNK cells when compared to circulating NK cells. PNK cells demonstrated strong cytolytic activity against multiple GBM cell lines. While inhibitors of Src, SYK, PLC-γ, p38 and ERK did not modulate PNK cytotoxicity, inhibitors targeting JNK and PI3K pathways significantly suppressed PNK cell cytotoxicity, specifically, 64.9±4.7% inhibition by SP600125 (JNK) and 25.2±3.3% inhibition by Ly294002 (PI3K) on LN-18 cells; 100% inhibition by SP600125 and 45.5.5±8.9% by Ly294002 on U251. JNK and PI3K inhibitors also reduced degranulation (70.6±3.2% by SP600125 and 58.4±3.6% by Ly294002). Furthermore, PI3K pathway controlled PNK cytokine production upon coculture with U251 cell line, whereas JNK inhibition had minimal effect.

**Conclusions**

Our results demonstrate the importance of PI3K and JNK pathways in mediating PNK cytotoxicity to GBM cell line targets. These data combined with our receptor profiling on PNK cells establish the rationale for further investigating receptor-ligand interactions that directly modulate PI3K and JNK activity.

#### P203 Discovery and characterization of the first fully human Phosphopeptide Tumor Target-specific T cell receptor

##### Xavier Michelet, PhD^1^, Eleni Chantzoura, PhD^1^, Ekaterina Breous-Nystrom, PhD^2^, Alessandra Franchino^2^, Rachel Smith^1^, Daniel Pollacksmith^1^, Jan Bergmann^1^, Alvaro Sebastian Yague^1^, Paisley Myers, PhD^2^, Erin Jeffrey^2^, Benjamin Wolf^2^, Dennis Underwood, PhD^2^, Marc Van Dijk, PhD^1^, Arthur Hurwitz, PhD^1^

###### ^1^Agentus Therapeutics, Lexington, MA, United States; ^2^Agenus, Basel, Switzerland

####### **Correspondence:** Arthur Hurwitz (andy.hurwitz@agentustherapeutics.com)

**Background**

AgenTus Therapeutics is developing innovative adoptive cell therapies to target a novel class of neoantigens called Phosphopeptide Tumor Targets (PTTs). These post-translational modification-based neo-antigens arise in tumor cells through dysregulated kinase and phosphatase activities. PTTs represent one of the most promising cell therapy targets, as they are shared within and between cancer indications. Using a mass-spectrometry-based approach that analyzes MHC I-bound peptides, we have analyzed PTTs from several indications. This approach allows us to survey the TCR ligandome of tumor cells and healthy tissues.

Phospho-ligandome analysis identified the phosphopeptide EPRpSPSHSM presented by HLA*B07:02+ cancer cells in a patient with Acute Myeloid Leukemia (AML). This phosphopeptide results from the phosphorylation of the Mixed Lineage Leukemia-1 (MLL1) protein, a histone lysine methyl transferase that functions as a transcriptional regulator and has been associated with tumorigenesis.

**Methods**

Using proprietary platforms consisting of primary T cell expansion from the central compartment and a mammalian display platform containing TCR α and β chain libraries from the expanded T cells, we isolated the first fully-human PTT-specific TCR: agenT-04002.

**Results**

Functional characterization demonstrated that target recognition by agenT-04002 is dependent on the phosphoseryl-moiety. Furthermore, agenT-04002 shows potent cytotoxic activity against numerous human hematologic tumor cell lines in vitro and AML tumor control in vivo in a mouse xenograft model. Activated T cells harboring the recombinant TCR display a pro-inflammatory phenotype in vitro and in vivo following tumor challenge. Most importantly, when co-cultured with AML cancer cells from patients, agenT-04002 T cells specifically recognize and kill tumor cells while sparing healthy myeloid cells.

**Conclusions**

AgenTus is developing the next generation of TCRs by targeting a unique class of neo-antigens with multi-cancer potential. Our data demonstrate feasibility, specificity, and potency of PTT-specific TCRs. Targeting PTTs across diverse indications will enable us to have broader applicability of cellular therapies.

#### P204 Ex vivo-activated allogeneic CD4^+^ T-cells disrupt immunosuppressive tumor microenvironment, and induce host tumor-specific cytotoxic T-cells in mice

##### Kazuhiro Mochizuki, MD, PhD^1^, Shogo Kobayashi^1^, Nobuhisa Takahashi^1^, Hideki Sano^1^, Yoshihiro Ohara^1^, Shin Mineishi, MD^2^, Yi Zhang^3^, Atsushi Kikuta^1^

###### ^1^Fukushima Medical University, Fukushima City, Japan; ^2^Penn State Cancer Institute, Hershey, PA, United States; ^3^Temple University, Philadelphia, PA, United States

####### **Correspondence:** Kazuhiro Mochizuki (mochi-k@fmu.ac.jp)

**Background**

Cancer immunotherapies that target tumor-specific or tumor-associated antigens are promising treatments for patients with incurable cancers [1,2]. However, relapses due to the loss of target antigens challenge the success of these therapies [1,3]. Multitargeted immunotherapies, such as cancer vaccinations specific to multiple cancer-associated peptides, are possible approaches. However, clinical studies have shown that they have limited efficacy with respect to the induction of objective responses [4]. The graft-versus-leukemia effect observed after allogeneic hematopoietic stem cell transplantation (allo-HSCT) is another example of strong multitargeted antitumor immunity mediated by donor T-cells that recognize and react to multiple allo-antigens [5]. In the present study, we demonstrated a novel approach for attaining alloreactive CD4^+^ T-cell-induced multitargeted cancer immunity that does not utilize allo-HSCT.

**Methods**

Cluster of differentiation (CD)4^+^ and CD8^+^ T-cells isolated form the spleen of BALB/c mice were separately activated in cultures by dendritic cells (DCs) generated from the bone marrow of C57BL/6 (B6) mice. The resultant host-reactive donor T-cells were injected into B6 mice bearing pre-established B16 melanoma. Host T-cells activated by syngeneic DCs were used as the control.

**Results**

Whereas the intratumoral injection of host-reactive donor CD4^+^ T-cells elicited potent antitumor immunity against established B16 melanoma in an alloantigen-dependent manner, intratumoral injection of host-reactive donor CD8^+^ T-cells or host-type syngeneic T-cells failed to induce antitumor responses. The number of injected donor-type host-reactive CD4^+^ T-cells diminished after tumor regression and did not induce graft-versus-host disease-like complications. Interestingly, early after injection, the alloreactive CD4^+^ T-cells underwent marked expansion and produced higher levels of interferon-gamma compared to syngeneic CD4^+^ T-cells. This was accompanied by markedly increased infiltration of host macrophages within the tumors as early as four hours after injection. These tumor-infiltrating macrophages secreted higher levels of interleukin (IL)-1β, IL-12 and IL-23, which are critical for inducing effector T-cell responses. Indeed, 24 hours after injection of alloreactive CD4^+^ T-cells, the infiltration of host effector CD8^+^ T-cells into tumors significantly increased, as evidenced by their production of high levels of perforin and granzyme B. Furthermore, the melanoma B6 mice that survived alloreactive CD4^+^ T-cell therapy developed host memory T-cells specific to the B16 melanoma and acquired complete resistance to the tumor rechallenge.

**Conclusions**

Results showed that immune reactions triggered by ex vivo-generated alloreactive CD4^+^ T-cells disrupt immunosuppressive tumor microenvironments and establish long-term host antitumor memory T-cell responses. Our findings may help develop new strategies for significantly enhancing the efficacy of cancer immunotherapy.

**Acknowledgements**

This work was supported by JSPS KAKENHI Grant Numbers JP15K09659, and JP19K07754.

**References**

1. Maude SL, Laetsch TW, Buechner J, Rives S, Boyer M, Bittencourt H, Bader P, MR. Verneris MR, Stefanski HE, Myers GD, Qayed M, Moerloose BD, Hiramatsu H, Schlis K, Davis KL, Martin PL, Nemecek ER, Yanik GA, Peters C, Baruchel A, Boissel N, Mechinaud F, Balduzzi A, Krueger J, June CH, Levine BL, Wood P, Taran T, Leung M, Mueller KT, Zhang Y, Sen K, Lebwohl D, Pulsipher MA, Grupp SA. Tisagenlecleucel in Children and Young Adults with B-Cell Lymphoblastic Leukemia. NEJM. 2018; 378: 439-448.

2. Ali SA, Shi V, Maric I, Wang M, Stroncek DF, Rose JJ, Brudno JN, Stetler-Stevenson M, Feldman SA, Hansen BG, Fellowes VS, Hakim FT, Gress RE, and Kochenderfer JN. T cells expressing an anti–B-cell maturation antigen chimeric antigen receptor cause remissions of multiple myeloma. Blood. 2016;128:1688-1700.

3. Majzner RG, Mackall CL. Tumor Antigen Escape from CAR T-cell Therapy. Cancer Discov. 2018; 8: 1219–1226.

4. Bezu L, Kepp O, Cerrato G, Pol J, Fucikovag J, Spisekg R, Zitvogel L, Kroemer G, Galluzzi L. Trial watch: Peptide-based vaccines in anticancer therapy. Oncoimmunol. 2018; 7: e1511506 (15 pages).

5. Negrin RS. Graft-versus-host disease versus graft-versus-leukemia. Hematology Am Soc Hematol Educ Program. 2015; 2015: 225-230

**Ethics Approval**

Experimental protocols were approved by the Fukushima Medical University’s committee on Use and Care of Animals; approval number 28054, 29039, and 2019048.

#### P205 Automated, closed bioreactors for T cell processing and dendritic cell-T cell co-culture

##### Lekhana Bhandary, BS, PhD, Andrew Kozbial, BS, PhD, Shashi Murthy, BS, PhD

###### Northeastern University, Boston, MA, United States

####### **Correspondence:** Shashi Murthy (s.murthy@northeastern.edu)

**Background**

Functionally closed and affordable automated cell culture systems are critical to the success of cell-based immunotherapy. Despite major advances in these therapies, there are few systems available that are practical for use at both the pre-clinical and clinical stages. To address this need, we have designed a system called BATON which is designed for optimal culture of both adherent and suspension cell types (Fig. 1). Cells are cultured via continual perfusion and the fluid flow loop also enables automated cell loading and harvest. This poster will describe two application areas, namely T cell expansion relevant to autologous CAR-T and TCR therapies and dendritic cell (DC)-T cell co-culture for neo-antigen-based T cell therapies.

**Methods**

The T cell expansion capability of the BATON system was evaluated by seeding BATON cartridges each having a surface area of 40 cm2 and volume of 25 mL with 23 million PBMCs along with CD3/28 Dynabeads. Cells were continually perfused with Irvine Scientific Prime XV xeno-free T cell medium with 33 U/mL IL-2 for 9 days. For comparison, a similar culture was performed in a G-Rex 6 well plate. For DC-T cell co-culture experiments enriched monocytes (MOs) were seeded into the BATON system at a seeding density of approximately 600k MOs/cm2 into two cartridges. Monocytes were differentiated into immature DCs by continually perfusing the seeded MOs for 6 days with CellGenix DC Medium supplemented with 350 U/mL IL-4 and GM-CSF (CellGenix). On Day 6, the DCs from one cartridge were harvested for flow cytometry. The other cartridge was drained without removal of the DCs and seeded with approximately 23 million PBMCs. This cartridge was then perfused with Irvine Scientific Prime XV xeno-free T cell medium with 33 U/mL IL-2. Cells were harvested following 7 days of co-culture. In addition to flow cytometry characterization, the cytotoxicity of the T cells was evaluated via co-culture with Jurkat cells.

**Results**

BATON achieved high levels of T cell expansion, comparable to G-Rex (Fig. 2-3) and harvested cells showed strong cytotoxic ability (Fig. 4). For DC-T cell co-culture experiments, the BATON system generated DCs from monocytes at high yield (27% of seeded monocytes converted into DCs) (Fig. 5A). Expansion of T cells from the seeded PBMCs was robust, with 26-fold expansion achieved in 7 days (Fig. 5B). Harvested T cells showed strong cytotoxic ability relative to control (Fig. 6).

**Conclusions**

The BATON system is an effective platform for reagent- and DC-mediated T cell expansion.

**Acknowledgements**

Funding from the NSF via grant 1645205 is gratefully acknowledged.

**Fig. 1 (abstract P205).**
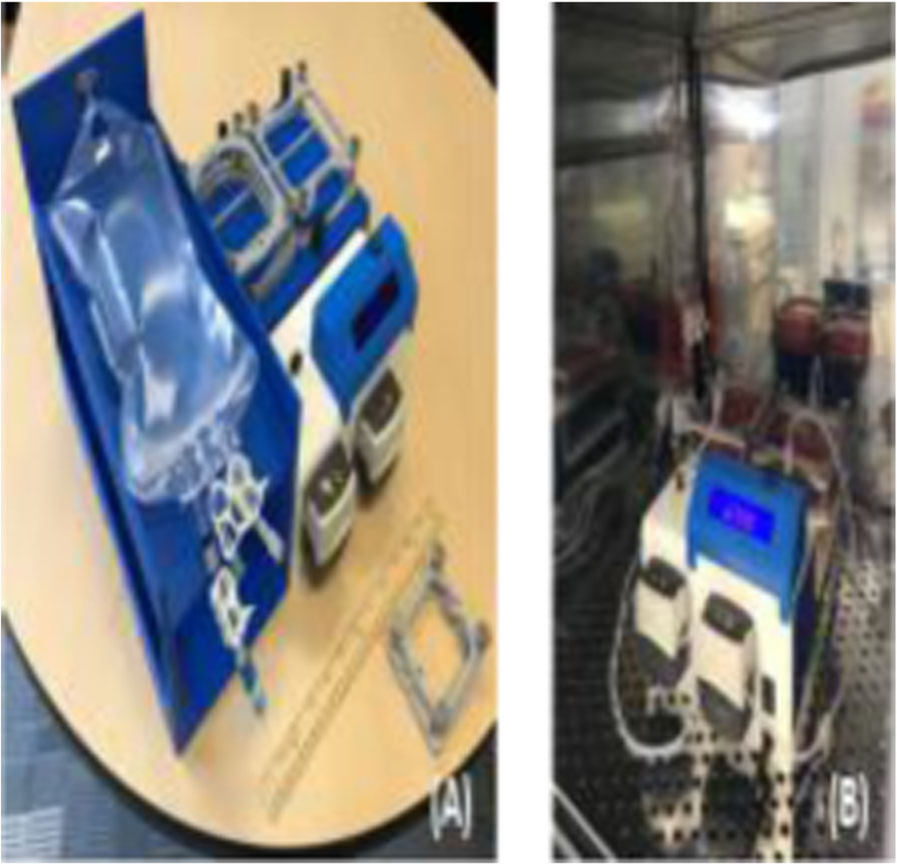
See text for description

**Fig. 2 (abstract P205).**
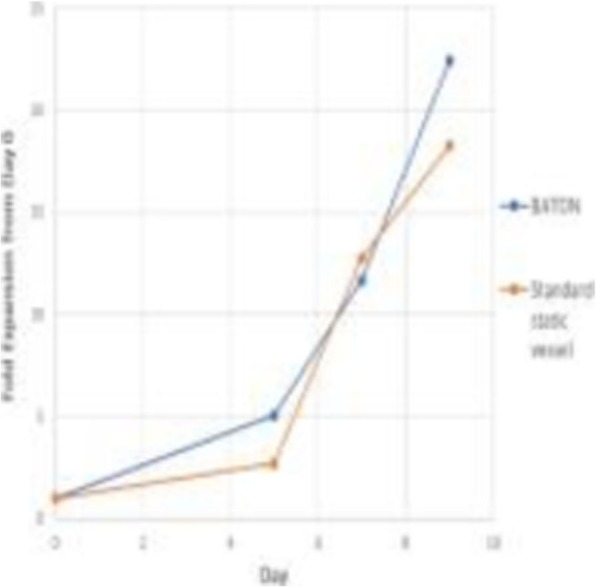
See text for description

**Fig. 3 (abstract P205).**
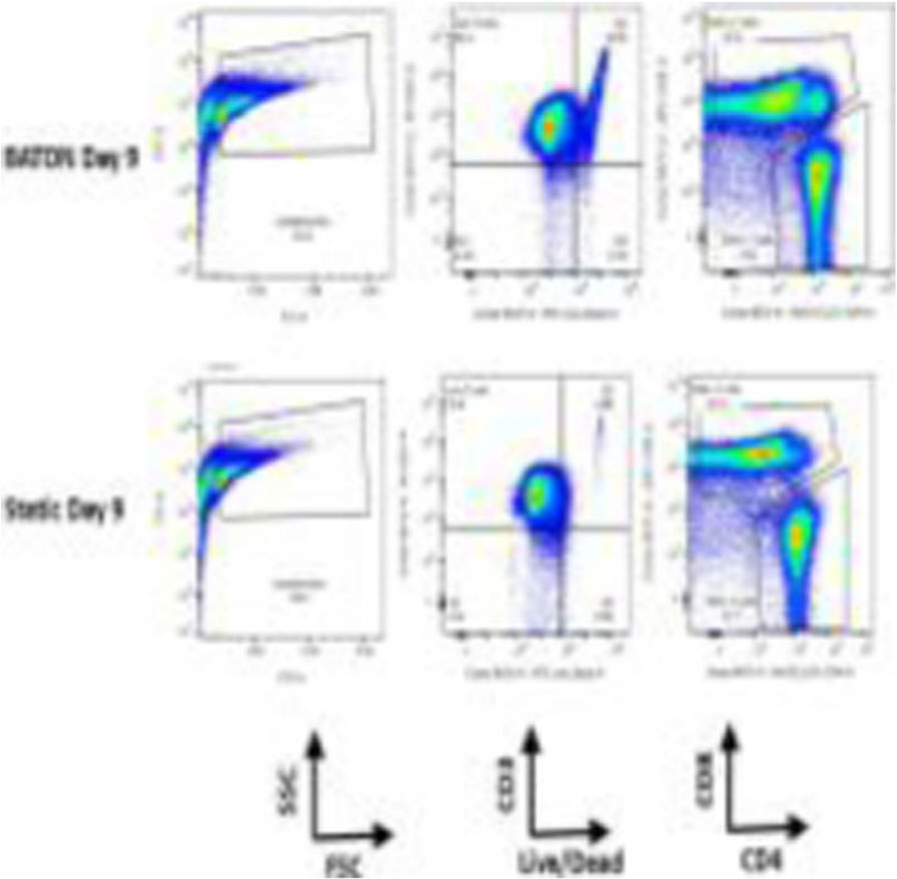
See text for description

**Fig. 4 (abstract P205).**
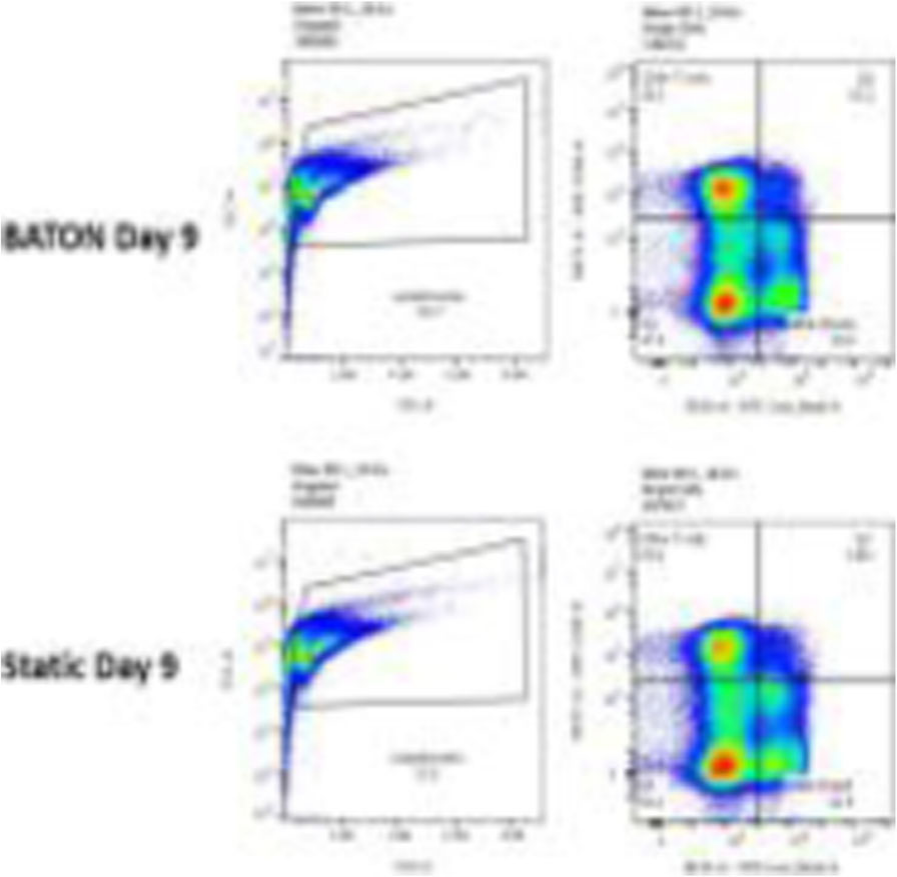
See text for description

**Fig. 5 (abstract P205).**
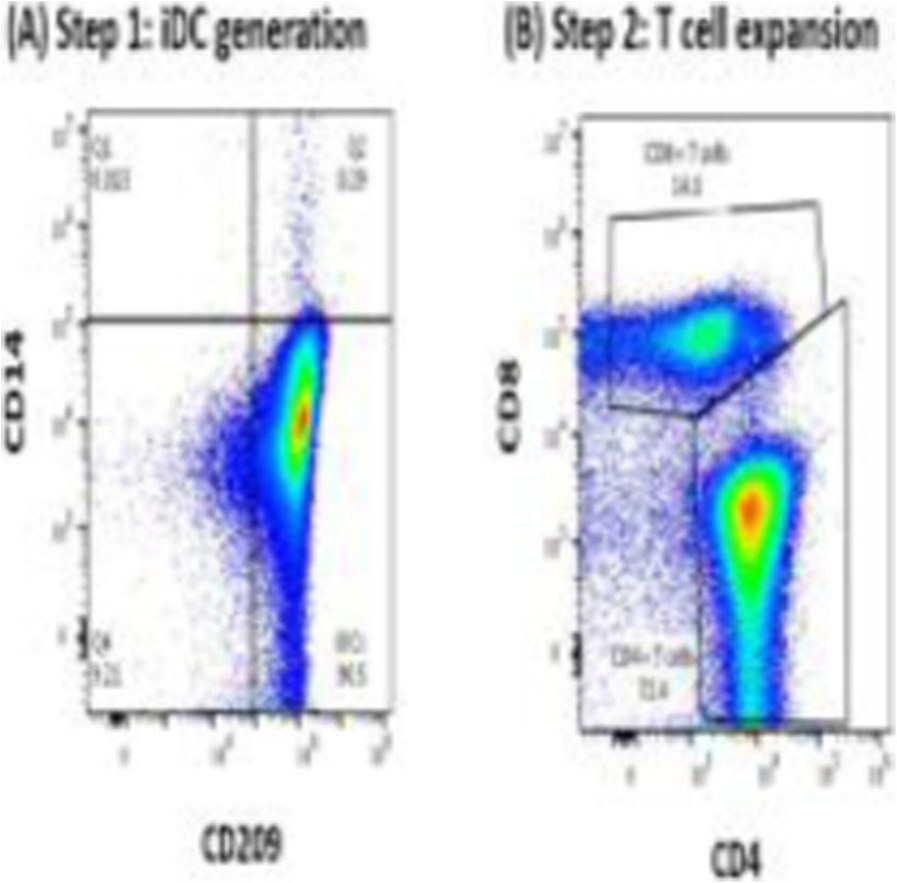
See text for description

**Fig. 6 (abstract P205).**
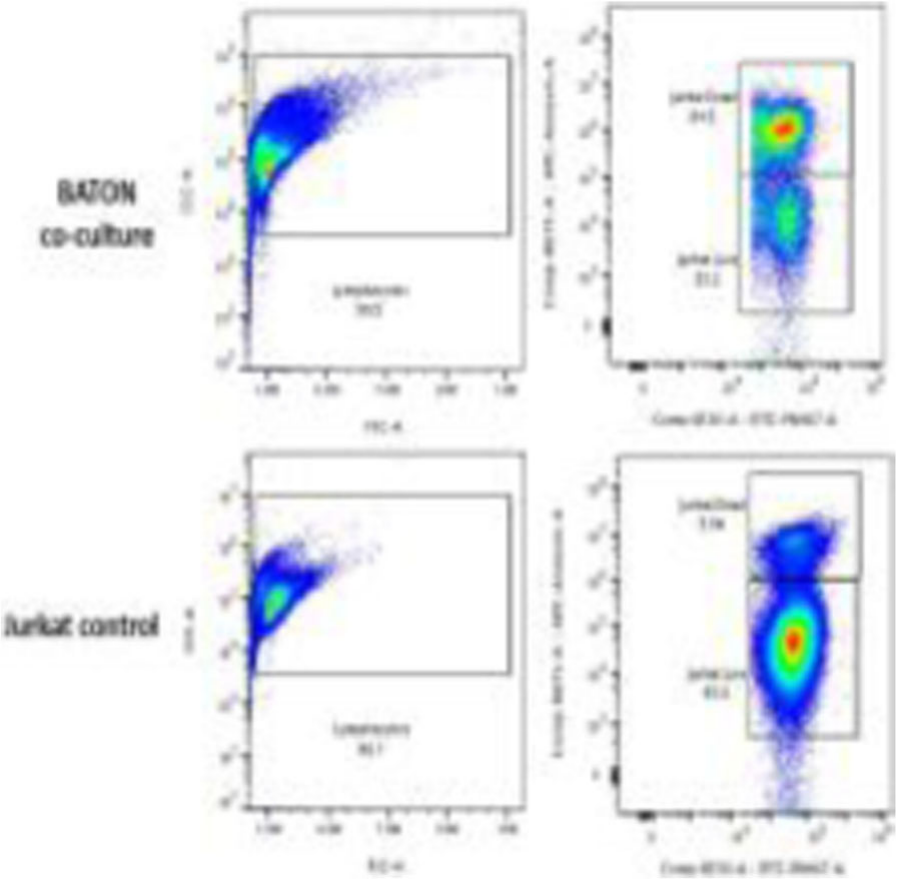
See text for description

#### P206 Transmembrane and linker domain amino acid composition alters chimeric antigen receptor (CAR) membrane residence and may conceal detection of novel functional CAR formats

##### Dina Schneider, PhD^1^, Virginia Hoglund, MS^2^, Ying Xiong, PhD^1^, Darong Wu, MS^1^, Boro Dropulic, PhD, MBA^1^, Rimas Orentas, PhD^2^

###### ^1^Lentigen Technology, Miltenyi Biotec, Gaithersburg, MD, United States; ^2^Seattle Children’s Research Institute, Seattle, WA, United States

####### **Correspondence:** Rimas Orentas (rimas.orentas@seattlechildrens.org)

**Background**

The relationship between the structure of the extracellular linker (L) and transmembrane (TM) domains, and CAR-T function has not been fully described. In previous studies we used L and TM domains derived from CD8. To better define amino acid sequences governing cell surface expression and anti-tumor activity, we altered the sequence and length of these domains and tested the impact on CAR T biology.

**Methods**

TM domains from glycoproteins expressed on the T cell surface were aligned to CD8 and those with a high degree of similarity were used to create new CARs. In some constructs the extracellular sequence proximal to the membrane of those proteins (L) was also included. CAR function was tested using LV-transduced human T cells. Protein expression was analyzed by flow cytometry and western blot, in vitro function by cytokine release and cell-mediated cytolysis, and in vivo function in xenograft models. CAR protein expression was also analyzed by immunofluorescent microscopy.

**Results**

Sequences from T cell-expressed CD antigens, the CD3 complex, activation markers, and members of the tumor necrosis factor receptor superfamily (TNFRSF) were analyzed. Based on sequence conservation we created new CARs expressing combinations of CD4 and CD8 TM domains, as well as TNFRSF9, TNFRSF16, and TNFRSF19 (CD137, NGFR, TROY/TAJ). All constructs were detected by western blot. Strong T cell surface expression was seen for CD8L/CD4TM, CD8L/CD4TM, CD8L/TNFRSF19TM, and TNFRSF16L/TNFRSF16TM. Intermediate surface expression was seen for TNFRSF9L/TNFRSF9TM. Constructs with TNFRSF19L/ TNFRSF19TM had very poor surface expression. However, these “undetectable” CARs by flow cytometry had the highest level of cytotoxicity and cytokine release vs Raji lymphoma. Immunofluorescence studies with transduced T cells on their own, or in the presence of Raji target cells, demonstrated that TNFRSF19 sequence may mediate an intracellular residence profile. Association of CARs with the CD3 complex was also noted.

**Conclusions**

The production of CARs for clinical use generally requires detection of the CAR protein on the cell surface. We found that high-activity CAR-T constructs can be created using the linker and TM domains of TNFRSF19, even though these constructs are expressed on the cell surface at low to undetectable levels. The mechanism by which these CARs are functionally active, while in a primarily intracellular state, is under investigation. Intracellular residence of CARs may be a novel mechanism to prevent undesired activation or T cell exhaustion and represents a novel locus of CAR-T activity control.

#### P207 A patient-driven ex vivo 3D tumor organoid model to assess efficacy of tumor infiltrating T-cell adoptive cell therapy

##### Jenny Kreahling, PhD, Mibel Pabon, PhD, Melba Page, PhD, Vijayendra Agrawal, PhD, Soner Altiok, MD, PhD

###### Nilogen Oncosystems, Tampa, FL, United States

####### **Correspondence:** Soner Altiok (soner@nilogen.com)

**Background**

Adoptive cell transfer (ACT) of ex vivo expanded tumor-infiltrating lymphocytes (TILs) has shown promising therapeutic efficacy in subsets of patients with several solid tumors including NSCLC. However, to improve the anti-tumor efficacy of TIL ACT in solid tumors it is critical to develop rational combination strategies and to identify biomarker(s) predictive of patients who would respond favorably to TIL therapy. Here we describe a high content imaging approach using a fresh tumoroid model with intact tumor stroma for quantitative assessment of autologous TIL infiltration and target tumor cell killing.

**Methods**

All human tumor samples were obtained with patient consent and relevant IRB approval. For the ex vivo assays 3D tumoroids measuring 100-150 micron in size were prepared and cryopreserved during the process of ex vivo propagation of autologous TILs. Allogeneic peripheral blood mononuclear cells (PBMCs) were used as control. Ex vivo propagated TILs were fluorescently labeled and their growth and functional characteristics in the presence or absence of CD3/CD28 tetramer were assessed via flow cytometry. High content confocal analysis was used to quantify TILs infiltration into the tumoroids and target tumor cell killing using Nilogen’s 3D-ACT platform. Multiplex cytokine assays and flow cytometry analysis were performed to assess TIL activation upon exposure to tumoroids.

**Results**

We successfully prepared matched autologous TILs and unpropagated 3D tumoroids from NSCLC patient tumors. The characteristics of tumor immune microenvironment and tumor cell viability was evaluated in previously cryopreserved tumor organoids using a custom image analysis algorithm that was developed for the collection of data in a structurally relevant environment on quantification of marker-specific cell number, cell viability and apoptosis in addition to structural and functional analysis of cells in intact 3D tumoroids. High content confocal imaging analysis demonstrated that CD3/CD28 pre-activated TILs with increased activation phenotypes and enhanced pro-inflammatory cytokine release had marked infiltration into the 3D tumor organoids compared to untreated TILs and PBMCs. The data was correlated with quantitative tumor cell killing assessment for tumoroids.

**Conclusions**

These results demonstrate that 3D-ACT model using ex vivo expanded TILs and 3D tumoroid models is an effective tool for the therapeutic assessment of autologous TILs and indicate that it can also be used to assess efficacy of other cellular therapy applications. Furthermore, implementation of this platform in the clinical studies may also allow determining the most effective combinatorial cellular therapy strategies for individual patients.

#### P208 Impact of combined blockade of PD1 and activation of CD137 on tumor infiltration and tumor cell killing efficacy of TILs in an ex vivo autologous 3D tumoroid model of NSCLC patient samples

##### Jenny Kreahling, PhD, Melba Page, PhD, Mibel Pabon, PhD, Vijayendra Agrawal, PhD, Soner Altiok, MD, PhD

###### Nilogen Oncosystems, Tampa, FL, United States

####### **Correspondence:** Soner Altiok (soner@nilogen.com)

**Background**

Adoptive cell therapy (ACT) with TILs has been of growing interest as anti-cancer treatment in solid tumors. This therapy consists of the outgrowth and expansion of tumor resident T cells from tumor material and their transfer back into the same patient to achieve tumor cell killing. However, existence of intrinsic immune escape mechanisms may diminish the efficacy of therapeutic applications of TILs. Here we describe an ex vivo patient derived 3D tumoroid platform utilizing powerful high content confocal imaging modalities to monitor the impact of PD1 inhibition and CD137 activation on autologous TIL infiltration and ACT mediated tumor cell killing.

**Methods**

Human tumor samples were obtained with patient consent and relevant IRB approval. Fresh patient tumor samples were processed into tumoroids measuring 100-150 μm in size. For these studies, autologous TILs were propagated from each tumor sample. TILs were fluorescently labeled and incubated together with 3D tumoroids in the presence or absence of the PD1 inhibitor nivolumab and/or an agonist anti-CD137 mAb urelumab. TIL infiltration into tumoroids and killing of metabolically labeled tumor cells were quantified by advanced confocal microscopy and a custom image analysis algorithm that was correlated with flow cytometry and cytokine profiling.

**Results**

We show that nivolumab and urelumab treatments had significant impacts on TIL infiltration in subsets of NSCLC tumoroids. Flow cytometry analysis demonstrated treatment-mediated activation of TILs accompanied by marked changes in the release of pro-and anti-inflammatory cytokine profiles. Furthermore, we documented the effect of TIL transfer and drug treatment on resident T-cells, Tregs and myeloid cell populations within the tumoroids. No correlation was found between TIL activity and composition of propagated TILs or PD-L1 expression on tumor cells.

**Conclusions**

This data suggests that combined blockade of PD1 and activation of CD137 may enhance the therapeutic efficacy of TIL ACT in NSCLC. Overall, this study also shows that our 3D-ACT tumoroid model allows comprehensive analysis of compensatory mechanisms and selection of rational combinatorial treatment using adaptive cellular therapy with autologous TILs and likely with other types of cellular therapies.

#### P209 Hijacking CAR-CD19 T cells to potently control Her2-positive solid tumors in vitro and in vivo through the use of unique and selective bridging proteins

##### Paul Rennert, PhD, Christine Ambrose, PhD, Fay Dufort, PhD, Lihe Dufort, Alyssa Birt, Thomas Sanford, Lan Wu, Roy Lobb, PhD

###### Aleta Biotherapeutics, Natick, MA, United States

####### **Correspondence:** Paul Rennert (paul.rennert@aletabio.com)

**Background**

Cell therapy success is limited by two critical issues. One is loss of antigen expression. This accounts for the ~50% relapse rate seen in CAR-treated B cell malignancies. Solid tumors have highly variable antigen expression and CARs targeting a single antigen fail as antigen-negative tumor cells escape, driving tumor resistance.

A related issue is that most cell therapeutics fail to persist in the patient. This is a particularly true of solid tumor treatment with CAR T cells. The persistence failure may result from unproductive CAR-T interaction with the targeted tumor cell.

We have developed CAR-CD19 T cells (CAR19s) that secrete bridging proteins to address these two critical issues. We leverage the ability of CAR19s to persist independently of the target tumor cell while simultaneously endowing these CARs with potent targeting technology. Here we illustrate this technology using the CD19-based bridging protein that binds both EGFR and Her2. These data demonstrate that CAR19 T cells can be redirected to kill solid tumors in vivo.

**Methods**

We cloned a highly stable CD19 extracellular domain (ECD) in frame with an anti-Her2 scFv to create, express and purify CD19-ECD-anti-Her2 bridging proteins. The sequence was also cloned downstream of a CAR19 domain and P2A cleavage site in a lentiviral vector. Transduced primary T cells expressed the CAR19 and secreted the bridging protein. These bridging protein formats were evaluated with in vitro cytotoxicity assays and Her2+ tumors in vivo. Finally, we added an anti-EGFR scFv sequence, creating an extremely potent multi-antigen targeting module.

**Results**

Incorporating a stabilized CD19 ECD in bridging proteins improved protein expression, including from CAR19 T cells. CAR19 T cells secreting stabilized CD19-anti-Her2 bridging proteins were highly potent in vitro and in vivo targeting CD19+ or Her2+ cells. An anti-EGFR scFv was added to the CD19-ECD-anti-Her2 bridging protein. This novel multi-antigen targeting bridging protein supports highly potent cytotoxicity against single and dual antigen-expressing tumor cells while retaining intrinsic anti-CD19 activity, providing these unique CARs with a tumor-independent and self-renewing antigen depot in CD19-positive normal B cells. For specific indications a third binding domain is added.

**Conclusions**

We have created a robust system for targeting multiple tumor antigens simultaneously with a single CAR T cell. Further the use of CAR19s supports CAR-T cell persistence independently of tumor antigen expression. This unique technology addresses critical issues in cell therapy using a potent technology whose modular nature allows for rapid program and pipeline development.

#### P210 Deep phenotypic and functional analysis of transduced anti-CD19 CAR T cells and untransduced T cells in patients treated with axi-cel by single cell mass cytometry

##### Yohei Arihara, MD, PhD^1^, Caron Jacobson, MD^1^, Philippe Armand, MD^1^, John Rossi, MS^2^, Nathalie Scholler, MD, PhD^2^, Stuart Sievers, PhD^2^, Edmund Chang, PhD^2^, Mauro Avanzi, MD, PhD^2^, Adrian Bot, MD, PhD^2^, Jerome Ritz, MD^1^

###### ^1^Dana-Farber Cancer Institute and Harvard Medical School, Boston, MA,United States; ^2^Kite, a Gilead Company, Santa Monica, CA, Santa Monica, CA, United States

####### **Correspondence:** Jerome Ritz (jerome_ritz@dfci.harvard.edu)

**Background**

Axi-cel, an autologous anti-CD19 chimeric antigen receptor (CAR) T cell therapy, has shown high efficacy in relapsed/refractory (R/R) diffuse large B cell lymphoma (DLBCL). Axi-cel contains heterogeneous populations of transduced and untransduced T cells. We used single cell mass cytometry (CyTOF) to analyze the impact of this heterogeneity on proliferation and expansion of these cells after infusion.

**Methods**

CyTOF examined CAR T cell products from 12 patients with R/R DLBCL and peripheral blood mononuclear cells obtained 7 days after axi-cel infusion. We identified anti-CD19 CAR T cells (CAR+ cells) using antibodies directed against the scFv extracellular region. The analytic panel included 29 cell surface, activation, exhaustion, and cell-cell adhesion markers to identify/characterize lymphocyte subsets; and 9 intracellular markers to characterize functional status and activation of signaling pathways. viSNE was used to visualize high-dimensional data on a 2D map and quantify CyTOF data.

**Results**

Axi-cel products contained a median of 63% (range, 20-86%) transduced CAR (CAR+) T cells, and included relatively undifferentiated T cell subsets: a median of 0.4% and 52% of CD4 CAR+ cells were T stem cell memory (SCM) and central memory (CM) cells, respectively, and 7% and 34% of CD8 CAR+ cells were SCM and CM cells, respectively. CAR+ T cells in products had significantly higher expression of proliferation, activation, and exhaustion markers (Ki67, CD25, HLA-DR, ICOS, OX-40, Tim3, LAG3) and higher expression of cell-cell adhesion molecules (CD49d, CD29) compared with CAR-negative (CAR–) T cells. On day 7, a median of 11% of circulating T cells (range 0.6-58.4%) were CD4 CAR+ and 3% (range 0.6-44.1%) were CD8 CAR+. Both CAR– and CAR+ T cells showed evidence of activation, but circulating CAR+ T cells expressed higher levels of Ki67, 4-1BB, Tim3, PD-1, PD-L1, CXCR3, CD29, pZAP70, pSTAT3 and pSTAT5, compared to CAR– T cells.

**Conclusions**

CyTOF enables detailed characterization of CAR T cell products comprising heterogeneous T cell subsets. Axi-cel comprises both transduced and untransduced T cells at various stages of differentiation, including SCM cells. CAR+ T cells showed higher expression of a broad range of proliferation and activation/exhaustion markers, compared to CAR– cells, both in axi-cel products and in peripheral blood 7 days after CAR T cell infusion. These data shed light on phenotypic and functional diversity of CAR T cells, pre- and post-infusion, influenced by the manufacturing process, conditioning-related homeostatic cytokines and antigen-driven activation. Future studies may explore associations between product composition and clinical outcomes.

**Trial Registration**

NCT02926833

#### P211 Combining Deep™ IL-12 Primed and Deep™ IL-15 Primed T cells leverages complementary mechanisms to enhance anti-tumor activity

##### Katharine Sackton, PhD, Elena Geretti, PhD, Pengpeng Cao, PhD, Shawn Carey, PhD, Xiaoyan Liang, Jonathan Nardozzi, PhD, Zishu Gui, Alicia Worthylake, Glenn Leary, Becker Hewes, MD, Tap Maniar, MD, Jonathan Fitzgerald, PhD, Andy Rakestraw, PhD, Karsten Sauer, PhD, Douglas Jones, PhD, Thomas Andresen, PhD

###### Torque Therapeutics, Cambridge, MA, United States

####### **Correspondence:** Thomas Andresen (tandresen@torquetx.com)

**Background**

Interleukin-15 (IL-15) and Interleukin-12 (IL-12) play complementary roles as immunomodulators. IL-15 induces T cell memory and supports survival, activation and proliferation of CD8+ T and NK cells. IL-12 promotes T cell cytotoxicity and innate immune responses in the tumor microenvironment. Both cytokines have been explored as cancer immunotherapies, but clinical success has been limited due to severe side effects. To limit systemic toxicities, Torque has developed its Deep-Primed™ T cell therapy technology. Multi-targeted T cells (MTC) specific for multiple tumor antigens are generated from patient apheresis. Cytokines are tethered to MTCs to support MTC persistence and activity following adoptive transfer into patients, while limiting systemic cytokine exposure. This study evaluates the combination of cytotoxic T lymphocytes (CTL) Deep-Primed with Deep™ IL-15 and Deep™ IL-12 to leverage their complementary biology for superior efficacy.

**Methods**

CTLs reactive against MART-1 antigen were generated from healthy donors (MART-1 CTLs). Next, expansion and cytotoxicity of MART-1 CTLs loaded with Deep IL-12, Deep IL-15 or both against MART-1 expressing SKMEL-5 melanoma cells were assessed. In addition, murine PMEL CD8+ T cells reactive against the B16-F10 melanoma antigen gp100 were loaded with Deep IL-12, Deep IL-15 or both and evaluated for in vitro expansion, activation and cytotoxicity against B16-F10 melanoma cells, as well as for anti-tumor activity in B16-F10 tumor-bearing mice.

**Results**

Loading with Deep IL-15 promoted MART-1 CTL proliferation and preserved antigen reactivity over time. Deep IL-12 loaded MART-1 CTLs displayed enhanced IFN-γ secretion and cytotoxicity, particularly at low effector:target ratios. Combination of MART-1 CTLs loaded with Deep IL-12 and Deep IL-15 further enhanced T cell expansion, IFN-γ secretion and cytotoxicity. Similarly, combination of murine PMEL T cells loaded with Deep IL-12 and Deep IL-15 resulted in persistent T cell activation, improved memory, and enhanced cytotoxicity over individually loaded T cells. Coadministration of Deep IL-12 and Deep IL-15 loaded PMEL T cells to B16-F10 melanoma-bearing mice was well-tolerated, with minimal and reversible body weight loss, and elicited superior anti-tumor activity.

**Conclusions**

Modular tethering of Deep™ IL-12 and Deep™ IL-15 to T cells uniquely leverages their complementary functions as immunomodulators to maximize anti-tumor activity without notable toxicity in preclinical models. A Phase I clinical trial of Deep IL-15 Primed MTCs (TRQ15-01) in solid cancers and lymphoma is enrolling (NCT03815682). Torque is initiating clinical evaluation of Deep IL-12 Primed MTCs (TRQ12-01), including a combination arm with TRQ15-01.

#### P212 Potential clinical application of tumor-infiltrating lymphocyte therapy for ovarian epithelial cancer prior or post-resistance to chemotherapy

##### Donastas Sakellariou-Thompson, BS, Marie-Andree Forget, PhD, Emily Hinchcliff, MD, Joseph Celestino, Patrick Hwu, MD, Amir Jazaeri, MD, Cara Haymaker, PhD, Chantale Bernatchez

###### MD Anderson Cancer Center, Houston, TX, United States

####### **Correspondence:** Cara Haymaker (chaymaker@mdanderson.org); Chantale Bernatchez (cbernatchez@mdanderson.org)

**Background**

Background: Epithelial ovarian cancer (OvCa) is the deadliest gynecological cancer, and is estimated to account for almost 14,000 deaths in 2018. Traditional management of advanced stage OvCa includes tumor reductive surgery and adjuvant platinum-taxane chemotherapy, which results in high rates of initial complete response. However, nearly 90% of patients recur and the 5-year survival rate for late-stage disease is only 28. Immunotherapy has become a powerful treatment option for several solid tumor types. The presence of tumor-infiltrating lymphocytes (TIL) is correlated with better prognosis in ovarian cancer, pointing at the possibility to benefit from harnessing their anti-tumor activity. The effectiveness of adoptive cell therapy (ACT) with TIL has already been shown in metastatic melanoma with objective response rates of 40-50%. This preclinical study explores the feasibility of transposing TIL ACT to OvCa using an improved culture method.

**Methods**

Methods: High-grade serous ovarian cancer (n=84), pre- or post-chemotherapy and primary or metastatic, samples were accrued. TIL were cultured using either high-dose IL-2 only, high-dose IL-2 with an agonistic antibodies targeting 4-1BB (a41BB), or a combination of IL-2, a41BB, and an agonistic anti-CD3 mAb. The cells were phenotyped using flow cytometry in the fresh tissue and after expansion. Tumor reactivity was assessed against HLA-matched ovarian cancer cell lines via IFN-γ ELISPOT.

**Results**

Results: Ovarian cancer is highly infiltrated with CD8+ TIL that are preferentially and robustly expanded with IL-2 and the two agonistic antibodies. With a 95% success rate, the TIL are grown to ≥100x10^6 cells in 2-3 weeks without over differentiation. In addition, the CD8+ TIL grown with this method showed HLA-restricted tumor recognition. TIL growth and tumor recognition was independent of surgery site or chemotherapy exposure.

**Conclusions**

Conclusions: These results indicate the viability of TIL ACT for refractory ovarian cancer by allowing for the large expansion of anti-tumor TIL in a short time and consistent manner. A Phase II clinical trial based on this work is currently open at UTMDACC to evaluate the feasibility of TIL ACT in recurrent or refractory OvCa (NCT03610490).

**Ethics Approval**

Ethics approval: Ethical approval and tissue from surgical resections used to expand TIL were both obtained under a protocol (PA16-0912 and LAB02-188) approved by the Institutional Review Board of UTMDACC.

#### P213 Conversion of peripheral blood mononuclear cells into tumor-specific cytolytic cell populations using tumor cells engineered with multiple immunomodulatory factors

##### Joshua Keegan^1^, James Lederer^1,2^, Frank Borriello, MD, PhD^2^, Nathan Schomer, MS^2^

###### ^1^BWH Harvard Medical School, Boston, MA, United States; ^2^Alloplex Biotherapeutics, Inc., Winchester, MA, United States

####### **Correspondence:** James Lederer (jlederer@alloplexbio.com); Frank Borriello (fborriello@alloplexbio.com)

**Background**

Numerous studies and two recent clinical approvals have demonstrated the efficacy and safety of cellular immunotherapies to treat diverse cancer subtypes. To date, the only approved cytotoxic cellular therapies in the U.S. are T-cell based. However, ongoing clinical trials using gamma delta T cells and natural killer cells have suggested not only clinical efficacy, but also synergistic beneficial effects when combined with checkpoint inhibitor antibody therapies. Our group developed novel immune stimulatory allogeneic tumor cells engineered with multiple immunomodulatory factors as a novel approach to generate heterologous tumor cell lytic populations of human peripheral blood mononuclear cells (PBMCs) for cellular immunotherapy.

**Methods**

SK-MEL-2 cells obtained from the NIH were engineered to express a set of immunomodulatory proteins with the aim of expanding oncolytic cell populations. Cells were engineered using lentiviral vectors prepared by VectorBuilder, and sorted by flow cytometer for high expression of desired immunomodulators. These engineered cells were then mixed with freshly thawed human PBMCs and co-incubated for up to 14 days. Cells were collected at time points during the incubation period for phenotypic analysis using mass cytometry (CyTOF). Functional characterization of stimulated PBMCs was conducted using a cytotoxicity assay against targets from several cancer subtypes.

**Results**

CyTOF analysis of stimulated PBMCs revealed expansion of natural killer cells, gamma-delta T cells, and CD8 T cells by 8 days after stimulation (Figure 1). All these populations expressed high levels of NKG2D and Granzyme B, suggesting widespread recognition of tumor antigens and cytotoxic capability. In contrast, PBMCs that were activated and expanded by CD3/CD28 activation beads showed less heterogenous expansion. PBMCs expanded with immunomodulatory cell lines demonstrated potent cytotoxic activity towards both the parental cell line, as well as other melanoma and non-melanoma cancer cell lines (Figure 2). As a control for specific cytotoxicity, both autologous and allogeneic PBMCs were tested as targets and displayed no detectable cytotoxicity in our assay.

**Conclusions**

We developed a novel approach to expand immune cell populations from normal human PBMCs demonstrating anti-tumor activity against multiple cancer cell types. This strategy is being developed to activate and expand PBMCs from cancer patients that will be used for autologous cellular immunotherapy. Taken together, the results from this study demonstrate our ability to generate multiple populations of cytotoxic effector cells from PBMCs, which should provide a straight-forward approach to generate clinically-relevant cells for adoptive cellular immunotherapy.

**Fig. 1 (abstract P213).**
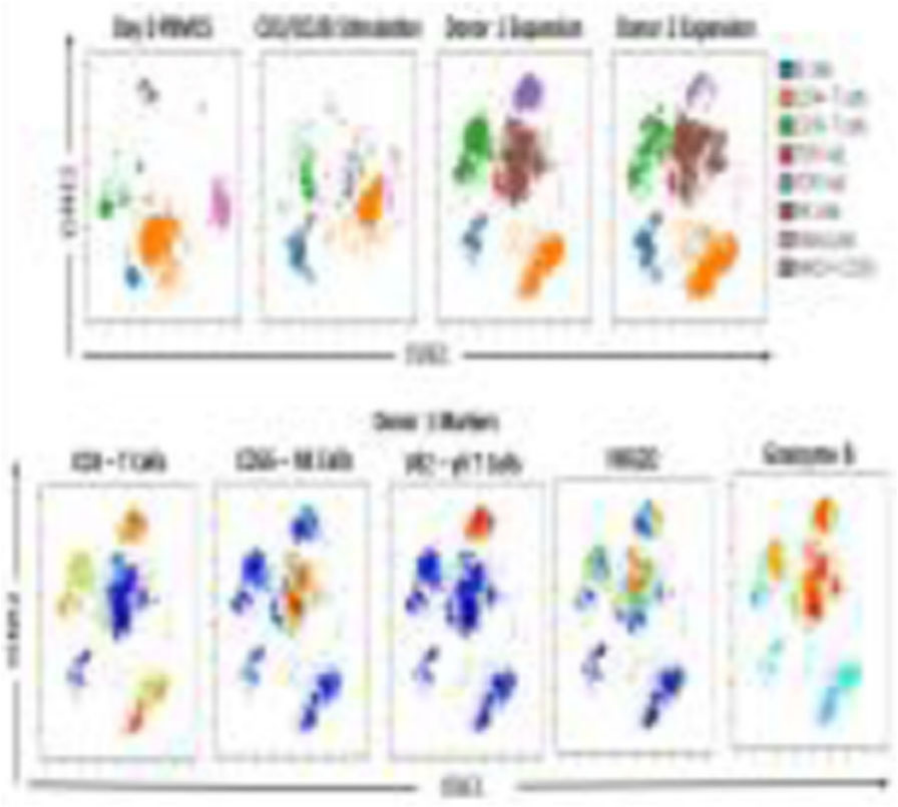
See text for description

**Fig. 2 (abstract P213).**
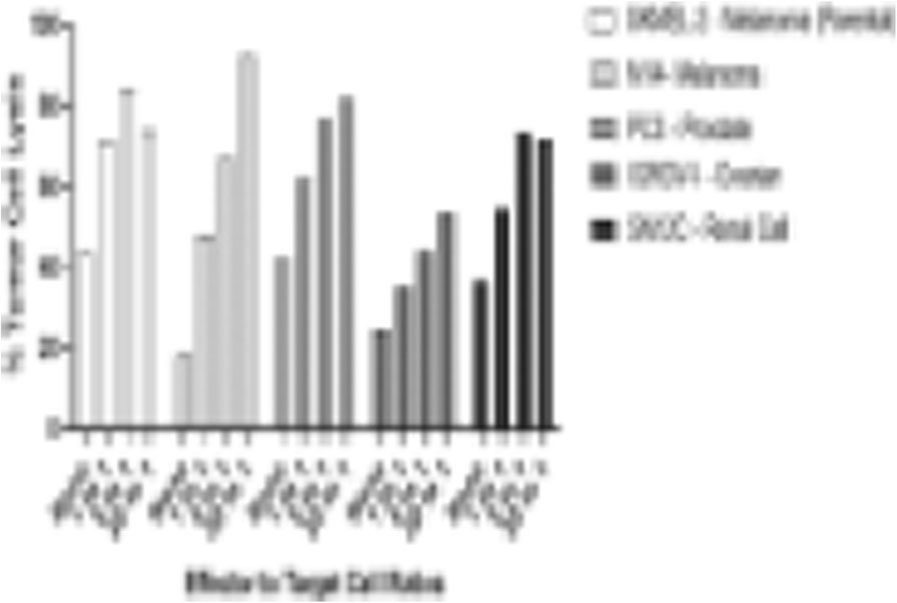
See text for description

#### P214 T cells precision engineered to express neoepitope-specific TCRs cloned from a patient with colorectal cancer specifically target and kill relevant neoantigen-expressing tumor cells

##### Barbara Sennino, PhD, Adam Litterman, John Gagnon, Andrew Conroy, Bhamini Purandare, Zheng Pan, Dalmas Olivier, Kyle Jacoby, Songming Peng, Alex Franzusoff, Stefanie Mandl, PhD

###### PACT Pharma, South San Francisco, CA, United States

####### **Correspondence:** Alex Franzusoff (afranzusoff@pactpharma.com); Stefanie Mandl (smandl@pactpharma.com)

**Background**

Neoepitopes (neoE) derived from private tumor-exclusive mutations represent compelling targets for personalized TCR-T cell therapy to eradicate tumor cells throughout the body. The imPACT Isolation Technology® is an ultra-sensitive and high-throughput process for the capture of mutation-targeted CD8 T cells from patient blood. NeoTCRs of native sequence, cloned from the captured T cells, were evaluated for tumor-targeted functionality by non-viral precision genome engineering of fresh human CD8 and CD4 T cells for neoTCR expression [1,2].

**Methods**

NeoTCRs were isolated from the blood of a patient with colorectal cancer using the imPACT Isolation Technology® [3]. Subsequently, healthy donor CD8 and CD4 T cells were precision genome engineered to replace endogenous TCRs with the native neoTCR sequence in each edited T cell for expression at native TCR levels. Precision genome engineering of a colon cancer tumor cell line (SW620) was used to express the patient-specific neoantigen (COX6C-R20Q) at native levels. neoTCR-T cells were co-cultured with SW620 expressing the COX6C-R20Q mutation or the COX6C wild-type peptide. Functional readouts were T cell proliferation, cytokine secretion and tumor cell killing.

**Results**

Seven neoTCR clonotypes against the mutated COX6C peptide (COX6C-R20Q) presented in the context of HLA-A2 were cloned from imPACT-captured neoE-specific CD8 T cells. Primary human T cells were engineered with the 7 different TCR specificities against the COX6C-R20Q. Each of the seven candidate neoTCR-engineered T cells displayed specific cytotoxicity against tumor cells expressing endogenous levels of the COX6C-R20Q neoantigen. At 96 hours, using Effector to Tumor cell ratio (E:T) 1:1, 85-90% tumor elimination was observed (p < 0.000001 for each comparison). Significant tumor cell killing was detected with an E:T ratio as low as 1:5. neoTCR-T cells also proliferated and secreted interferon-gamma in response to co-culture with the relevant tumor target. Importantly, neoTCR-T cell activity was absent when co-cultured with tumor cells expressing wild type COXC6 protein.

**Conclusions**

These results demonstrate that the imPACT Isolation Technology used to capture antigen-experienced, neoE-specific T cells from the blood of patients with cancer authenticates that these neoE-HLA targets are relevant for engineering neoTCR-T cells therapies. Leveraging this approach, PACT is developing autologous personalized adoptive T cell therapy (NeoTCR-P1 product). A Phase 1 clinical trial to test NeoTCR-P1 T cells in subjects with solid tumors is currently ongoing (NCT03970382).

**References**

1. Jacoby K, Moot R, Lu W, Nguyen D, Sennino B, Conroy A, Purandare B, Litterman A, Urbinati F, Foy S, Hunter T, Tai A, Bethune M, Peng A, Dalmas O, Franzusoff A and Mandl S. Abstract 4783: Highly efficient, non-viral precision genome engineering for the generation of personalized neoepitope-specific adoptive T cell therapies. Cancer Res 2019;79(13 Suppl).

2. Sennino B, Conroy A, Purandare B, Litterman A, Jacoby K, Moot R, Lu W, Nguyen D, Urbinati F, Foy S, Hunter T, Dalmas O, Bethune M, Park T, Peng S, Franzusoff A and Mandl S. Abstract 1433: NeoTCR-P1, a novel neoepitope-specific adoptive cell therapy, consists of T cells with ‘younger’ phenotypes that rapidly proliferate and kill target cells upon recognition of cognate antigen. Cancer Res 2019;79(13 Suppl).

3. Peng S, Quach B, An D, Sandoval S, Bao R, Pan Z, Bethune M, Dalmas O, Yi M, Meadows C, Heeringa K, Guo L, Yuen B, Sorfleet J, Jacoby K, Moot R, Lu W, Nguyen D, Sennino B, Conroy A, Purandare B, Litterman A, Mandl S and Franzusoff A. Abstract 1435: An ultra-sensitive and high-throughput technology (imPACT) for the identification and isolation of intrinsic and emergent neoepitope-specific T cells from the peripheral blood and TILs of cancer patients. Cancer Res 2019;79(13 Suppl).

**Ethics Approval**

Human samples in this study were procured from a commercial vendor who collected them according to their established ethics policies.

#### P215 Depletion of CD45RA-positive cells potentiates the reactivation of EBV-specific T-cells from EBV positive lymphoma patients

##### Sandhya Sharma, BSc, Naren Mehta, Kathan Parikh, RA, Cliona Rooney, PhD

###### Baylor College of Medicine, Houston, TX, United States

####### **Correspondence:** Sandhya Sharma (me.sandhya@gmail.com)

**Background**

Epstein-Barr virus (EBV)-positive lymphomas express viral type-2 latency proteins (T2-Ags). Autologous EBV-specific T-cells (EBVSTs) directed to T2-Ags have produced complete responses in ~50% lymphoma patients[1]. However, in our ongoing clinical trial, we failed to generate EBVSTs from 24% of the patients procured; manufacturing failures associated with lack of T-cell expansion and T2-Ag-specificity. Further, in lines successfully expanded, many EBVST lines demonstrated low T2-Ag specificity and some contained a high frequency of NK-cells. Our goal is to improve both the manufacturing success rate and clinical efficacy of EBVSTs. In EBV-exposed individuals, EBVSTs reside in CD45RA-CD45RO+ memory compartment, while CD45RA-positive population includes unwanted naïve T-cells, suppressive regulatory-T cells, and NK-cells[2,3]. We hypothesized that removal of CD45RA-positive cells from PBMCs prior to EBV-antigen specific stimulation would improve EBVST generation and specificity by eliminating competing naïve T-cells, while reducing potentially inhibitory cells capable of inhibiting the outgrowth of antigen-specific T-cells. We, therefore investigated the effects of selective depletion of CD45RA-positive cells from PBMCs prior to EBV T2-Ags stimulation.

**Methods**

EBVSTs were generated from whole PBMCs and CD45RA depleted (RAD) PBMCs and we measured proliferation by counting, T2-Ag specificity using IFN-gamma ELIspot assays and cell-phenotype by flow cytometric analysis. To compare their in-vivo efficacy, EBVSTs were adoptively transferred into immunodeficient mice bearing autologous EBV-transformed lymphoblastoid tumor cells and tumor clearance was evaluated.

**Results**

RAD-EBVSTs produced greater expansion of EBVSTs from PBMCs and decreased the frequency of NK cells, which dominated some of our patient lines. T2-Ag specificity increased by 3-5 fold as measured by gamma-IFN release in response to T2-Ags stimulation in both healthy donors and patients. RAD-EBVSTs maintained antigen specificity over multiple rounds of weekly T2-Ags stimulation. Phenotypic analysis demonstrated decreased expression of exhaustion markers in RAD-EBVSTs. Most importantly, this approach restored responsiveness to antigen stimulation in most unresponsive patients enabling the successful reactivation and expansion of EBVSTs from lymphoma patients that failed previously. This advantage extends to VSTs with specificity for HPV, VZV and adenoviral antigens, suggesting that we have identified a general approach for improving the activity of VSTs for immunotherapy. EBVSTs generated from RAD-PBMCs demonstrated more rapid tumor clearance and superior control of metastatic tumors in our xenograft model.

**Conclusions**

This approach to the generation of VSTs is being translated to the clinic for use in multiple clinical trials. In future, we aim to elucidate the mechanisms underlying the inhibitory effects of the CD45RA positive population in the reactivation and expansion of EBVSTs.

**References**

1. Bollard CM, Gottschalk S, Torrano V, et al. Sustained Complete Responses in Patients With Lymphoma Receiving Autologous Cytotoxic T Lymphocytes Targeting Epstein-Barr Virus Latent Membrane Proteins. Journal of Clinical Oncology. 2016;32(8):798-808. doi:10.1200/JCO.2013.51.5304.

2. Krzywinska E, Cornillon A, Allende-Vega N, et al. CD45 Isoform Profile Identifies Natural Killer (NK) Subsets with Differential Activity. Bobé P, ed. PLoS ONE. 2016;11(4):e0150434. doi:10.1371/journal.pone.0150434.

3. Krzywinska E, Allende-Vega N, Cornillon A, et al. Identification of Anti-tumor Cells Carrying Natural Killer (NK) Cell Antigens in Patients With Hematological Cancers. EBioMedicine. 2015;2(10):1364-1376. doi:10.1016/j.ebiom.2015.08.021.

**Ethics Approval**

The study was approved by Baylor College of Medicine's Ethics Board, approval number H7634 and H7666 for human subjects. Animal protocol AN5551.

#### P216 CD8+ T cells break tolerance to tumors in a B cell-dependent manner via TLR9 signaling

##### Aubrey Smith, BS, Hannah Knochelmann, BS, Connor Dwyer, PhD, Megan Wyatt, MS, Guillermo Rangel Rivera, Jessica Thaxton, PhD, MSCR, Mark Rubinstein, PhD, Bei Liu, MD MPH, Eric Bartee, PhD, Chrystal Paulos, PhD

###### Medical University of South Carolina, Mount Pleasant, SC, United States

####### **Correspondence:** Aubrey Smith (butchera@musc.edu)

**Background**

Administration of Toll-like receptor agonists with adoptively transferred T cells augment tumor immunity. However, systemic or local administration of TLR agonists could heighten inflammation and lead to toxic side effects when coupled with CAR or TIL-based therapies. Thus, we hypothesized that TLR agonists could be used ex vivo during T cell expansion and ultimately generate a cell product with enhanced anti-tumor properties for adoptive cell transfer therapy while reducing potential toxicity to patients.

**Methods**

To test our hypothesis, we employed the Pmel-1 transgenic mouse model, in which CD8+ T cells harbor a TCR specific for the gp100 epitope expressed on melanoma and healthy melanocytes. Pmel splenocytes were stimulated with hgp100 peptide in the presence or absence of TLR9 agonist CpG (ODN-1668) and expanded in IL-2 for one week. T cell phenotype was analyzed via flow cytometry and anti-tumor activity assessed in mice bearing B16F10 melanoma tumors.

**Results**

T cells expanded with CpG possessed a unique phenotype (IL-2Ralpha-high, ICOS-high, CD39-low) and mediated more potent responses against melanoma in vivo than traditionally expanded T cells. Interestingly, this phenotype and anti-tumor function was dependent on the presence of B cells at the start of culture, as their removal resulted in a loss of this unique phenotype and anti-tumor efficacy in vivo. Conversely, removal of CD4+ T cells, NK cells, dendritic cells, or macrophages from culture did not ablate the phenotype or anti-tumor activity of CpG-generated T cells. The CpG-elicited T cell effects were also dependent on the peptide-mediated interaction between the T cell and APC in culture as activating with plate-bound or bead-bound antibody strategies resulted in a T cell population that was similar to the ineffective vehicle treated cells both phenotypically and therapeutically. We further found that CpG-treated B cells expressed heightened levels of CD40, suggesting that induction of a CD40-CD40L axis between B and T cells may account for the generation of potent IL-2Ralpha-high, ICOS-high, CD39-low T cells.

**Conclusions**

The TLR9 agonist CpG can be used in ex vivo culture to potentiate an anti-tumor T cell product for ACT. The CpG-associated T cell phenotype (IL-2Ralpha-high, ICOS-high, CD39-low) and anti-tumor ability was dependent on the presence of B cells and a direct interaction between the anti-tumor T cells and APC via peptide activation. Post CpG stimulation B cells express heightened CD40, which may promote the B cell/T cell interaction and thus the generation of a potent T cell product for ACT.

**Ethics Approval**

This study was approved by the Institutional Animal Care & Use Committee of the Medical University of South Carolina, protocol number 0488.

#### P217 An NK cell line (PD-L1 t-haNK) engineered to target PD-L1 efficiently kills tumor cells and myeloid derived suppressor cells

##### Kellsye Fabian, PhD^1^, Michelle Padget^1^, Renee Donahue, PhD^1^, Kristen Solocinski, PhD^1^, Clint Allen, MD^2^, John Lee, MD^3^, Patrick Soon-Shiong, MD^3^, Jeffrey Schlom, PhD^1^, James Hodge, PhD, MBA^1^

###### ^1^NCI/NIH, Bethesda, MD, United States; ^2^NIDCD/NIH, Bethesda, MD, United States; ^3^NantKwest, Culver City, CA

####### **Correspondence:** James Hodge (jh241d@nih.gov)

**Background**

The ability of natural killer (NK) cells to lyse tumor targets without prior sensitization and without human leukocyte antigen (HLA)-restriction make them promising candidates for “off the shelf” cell-based immunotherapy. Here we investigate the anti-tumor efficacy of a novel NK cell platform, the PD-L1-targeted high-affinity NK (PD-L1 t-haNK), which lacks killer inhibitory receptors (KIRs), carries a high payload of granzyme and perforin granules, and has been designed with a chimeric antigen receptor (CAR) to target PD-L1 expressing cells on cells.

**Methods**

Frozen, irradiated (15 Gy) PD-L1 t-haNK cells were thawed and characterized via flow cytometry and RNAseq. PD-L1 t-haNK lytic activity was assessed in vitro using MDA-MB-231, BT549, T47D, MCF7, SUM149, H460, H441, HCC4006, SW480, SW620, DU145, HTB1, CaSki, and CH22 cell lines as targets in indium-based and flow cytometry-based killing assays. The effect of pre-treating tumor targets with IFNγ on PD-L1 t-haNK targeting was also examined. PD-L1 t-haNK cells were co-cultured with human peripheral blood mononuclear cells (PBMCs) and purified human myeloid derived suppressor cells (MDSCs) to investigate the effects of PD-L1 t-haNK on immune subsets. The therapeutic activity of PD-L1 t-haNK in vivo was studied by adoptive transfer of PD-L1 t-haNK cells to NOD scid gamma (NSG) mice engrafted with parental MDA-MB-231 (PD-L1+) and MDA-MB-231/PD-L1 CRISPR knockout (PD-L1-null).

**Results**

Here, we show that irradiated PD-L1 t-haNK cells express PD-L1-specific CAR, and high levels of perforin and granzyme B. PD-L1 t-haNKs lysed all 14 human tumor cell lines tested in vitro, and increased cell lysis corresponded with increasing levels of PD-L1 expression on tumor cells. Increasing PD-L1 expression on tumor cells through IFNγ treatment improved PD-L1 t-haNK-mediated lysis by up to 100%. In vivo, adoptive transfer of PD-L1 t-haNK cells inhibited the growth of engrafted parental MDA-MB-231 but not PD-L1 null MDA-MB-231 tumors. Finally, when co-cultured with human PBMCs and purified human MDSCs expressing PD-L1, PD-L1 t-haNK cells preferentially lysed the MDSC population but not other PBMC subsets.

**Conclusions**

This study provides a rationale for the potential use of adoptively transferred irradiated PD-L1 t-haNK cells as a unique immunotherapeutic platform against a range of human tumor types. In addition to lysing the tumor cells, MDSC killing may be a novel mechanism of action of PD-L1 t-haNK cells that may potentiate their impact especially in cancers where MDSC’s limit immune approaches. The safety and clinical benefit of PD-L1 t-haNK cells in cancer patients are being assessed in ongoing clinical trials.

**Ethics Approval**

The study was approved by the NCI IRB, NIH protocol 99-CC-0168.

#### P218 A novel, bioluminescent assay for the selective detection of target cell killing in mixed cultures

##### Richard Somberg, PhD, Brock Binkowski, Aileen Paguio, Peter Stecha, Chris Eggers, Braeden Butler, Michael Beck, Julia Gilden, PhD, Jey Cheng, Mei Cong, PhD, Frank Fan, PhD

###### Promega, Madison, WI, United States

####### **Correspondence:** Brock Binkowski (brock.binkowski@promega.com)

**Background**

Efforts to develop and commercialize cellular immunotherapies would benefit from assays that selectively monitor target cell death that are sensitive and easy-to-use. To address this, we have developed an approach to selectively quantify target cell death using a gain-of-signal assay format and bioluminescence read-out.

**Methods**

The method relies on the release of a HiBiT-tagged protein from target cells following cell lysis. HiBiT, an 11 a.a. peptide tag, binds to cell-impermeable Large BiT (LgBiT), a 17.6 kDa protein, to reconstitute NanoBiT Luciferase. Target cells are engineered to express a HiBiT-tagged protein using either ectopic expression or CRISPR/Cas9 to tag endogenous lactate dehydrogenase (LDH), and cell lysis is quantified by adding a detection reagent containing LgBiT and furimazine substrate (no medium removal).

**Results**

The signal is proportional to the amount of target cell death, and measurements can be made using endpoint or kinetic formats. Cell lines have low rates of spontaneous release and fusion proteins that are stable in the extracellular medium, enabling assays up to 24 hours or more. The bright signal from NanoBiT Luciferase allows the use of low numbers of target cells per well (e.g. 2,500), and the assay can detect very low levels of target cell death (e.g.

**Conclusions**

The assay provides a sensitive, simple read-out to support discovery research and/or QC lot release applications.

#### P220 Targeting neoantigens with immunotherapy: Are we limited to pre-existing autologous neoantigen-specific T-cells?

##### Aline Bracher, Slavoljub Milosevic, Daniel Sommermeyer

###### Medigene Immunotherapies GmbH, Planegg-Martinsried, Germany

####### **Correspondence:** Daniel Sommermeyer (d.sommermeyer@medigene.com)

**Background**

Several facts shape our considerations regarding the use of neoantigens as highly specific targets for immunotherapy of cancer. First, adoptive T-cell therapy using TILs seems to be most successful if the TILs include T-cells specific for antigens resulting from individual mutations. Second, the success of checkpoint inhibitors is often correlated with the mutational load of tumors. However, a mutation must fulfill several criteria to be effective as a neoantigen that can be recognized by T-cells. Obviously, the mutation must lead to a novel amino acid sequence (e.g. single amino acid substitution, fusion- or frameshift-sequence), and be located in a gene expressed in tumor cells. Furthermore, a peptide spanning the new sequence needs to be efficiently processed and presented by HLA molecules. Finally, a T-cell response must be triggered that can specifically recognize the mutated epitope.

Targeting neoantigens as patient-individualized epitopes requires robust processes for rational and rapid selection and validation of neoantigens as T-cell targets. Currently, the most challenging step is predicting specific T-cell responses. Huge efforts have been made to analyze the reactivity of patients’ T-cells against mutations. However, this approach is limited to the T-cell repertoire present in patients at the time of tumor resection or blood draw and might miss potential potent T-cell responses that were lacking or no longer present in the patient. In our opinion, only screening the T-cell repertoire of several healthy donors can answer the question if a specific mutation can trigger T-cell responses. We present proof-of-concept data how we use our high-throughput T-cell receptor (TCR) platform technologies and automated processes for fast and efficient screenings of T-cells isolated from several partially HLA-matched healthy donors.

**Methods**

Promising neoantigens were predicted and T-cells responses after stimulation with antigen-presenting cells either transfected with minigene constructs or loaded with peptides were compared.

**Results**

Peptide stimulation triggered specific T-cells for most tested mutations, indicating that T-cell repertoires of healthy donors can recognize neoantigens when they are forced to be presented. Also, with the clinically relevant approach using endogenously processed antigen encoded by minigenes, specific T-cell responses against neoantigens presented on different HLAs were efficiently triggered, although only against some of the tested epitopes.

**Conclusions**

This screening strategy has the aim to develop future TCR-based therapies and can be used for the identification of promising mutations for vaccination or as a source for TCRs for adoptive T-cell therapy. Furthermore, generated data can subsequently improve algorithms predicting the immunogenicity of neoantigens.

#### P221 Superior anti-tumor activity of metabolically enhanced bispecific antibody armed CAR-less Bionic T cells

##### Archana Thakur, PhD^1^, John Scholler^2^, Edwin Bliemeister^1^, Carl June, MD^2^, Lawrence Lum, MD, DSc^1^

###### ^1^University of Virginia, Charlottesville, VA, United States; ^2^University of Pennsylvania, Philadelphia, PA, United States

####### **Correspondence:** Archana Thakur (at2fx@virginia.edu)

**Background**

The major challenges of T cell-based therapies in solid tumors are the metabolic insufficiency of T cells to exhibit effector functions and persist in an altered metabolic landscape of the tumor microenvironment (TME). To overcome these challenges, we developed the next generation of chimeric antigen receptors-less (CAR-less) Bionic T cells for the treatment of solid cancers. We hypothesize that metabolically enhanced Bionic T cells armed with bispecific antibody (BiAb) will show enhanced anti-tumor responses and controlled off-tumor on-target toxicity in solid cancer.

**Methods**

We engineered metabolically enhanced “Bionic T cells” (BTC) that are devoid of CAR but contain a transmembrane and an intracellular domain of a co-stimulatory molecule and TCR signaling domain of CD3z. T cells were transduced with CAR-less constructs without co-stimulatory domain-FLAG-ζ (Control) or with co-stimulatory domain-FLAG-4-1BB-ζ, FLAG-CD28-ζ, FLAG-ICOS-ζ and FLAG-OX40-ζ, FLAG-27-ζ and were tested for their hypoxic tolerance, anti-tumor activity, cytokine production and exhaustion phenotype in the presence or tumor targets.

**Results**

Our data show that hypoxia differentially affected BTC survival depending on the co-stimulatory endodomain. Under normoxia Bionics with CD28ζ (28z) endodomain show 5% apoptosis versus 61% apoptosis under hypoxic (5% oxygen) condition. On the other hand, Bionics with 4-1BBζ showed only 13% apoptosis under hypoxic condition suggesting enhanced hypoxic tolerance. HER2 and EGFR BiAb armed BTC were tested against various low-high HER2 and EGFR expressing cancer cell lines. Specific cytotoxicity of anti-HER2 BiAb (HER2Bi) and anti-EGFR BiAb (EGFRBi) armed BTC against MDA-MB-231, SK-BR-3, BT-20, MiaPaCa-2 cell lines measured by real time cell analysis using xCELLigence ranged between 75-100% at 2:1 E/T ratio at 72 hours. Sequential killing by HER2Bi armed BTC followed by (f/b) EGFRBi armed BTC showed efficient killing against target cells (86.2%) or EGFRBi-BTC f/b HER2Bi-BTC (88.2%) compared to the killing by HER2Bi-BTC (49.7%) or EGFRBi-BTC (43.5%) alone at low E/T ratio in the presence of 100 IU/ml IL-2 at 96 hours. Cytokine levels of IFN-γ, IL-15, IL-2R, and GM-CSF were significantly higher in culture supernatants of tumor cells (SK-BR-3) and HER2Bi-BTC or EGFRBi-BTC co-cultures compared to the control condition with unarmed BTC. Phenotypic data show increased expression of 4-1BB, ICOS and OX40 on CD4+ and CD8+ T cells after short antigenic exposure and continuous antigenic exposure.

**Conclusions**

Data show that armed BTC: 1) exhibit superior survival in hypoxic condition; 2) can effectively kill multiple tumor targets in serial killing assay; 3) cytokines and chemokines show immune modulating and tumor killing profile.

**Acknowledgements**

This study was made possible by start-up funds for LGL from the University of Virginia. AT is a co-founder of NOVA Immune Platform. CJ is a co-founder of T Immunity. LGL is a co-founder of Transtarget, Inc. and sits on the Scientific Advisory Board for Rapa Therapeutics.

#### P222 Modulating BRD4 in T cells using self-delivery RNAi to improve adoptive cell therapy of cancer

##### Jeroen Melief, PhD^1^, Laura Van Leeuwe Kirsch, BSc^1^, Esmeralda Hemme^1^, John Barrett, PhD^2^, Simon Fricker, PhD^2^, Gerrit Dispersyn, PhD^2^, Rolf Kiessling, MD, PhD^1^

###### ^1^Karolinska Institute, Stockholm, Sweden; ^2^Phio Pharmaceuticals, Marlborough, MA, United States

####### **Correspondence:** Rolf Kiessling (rolf.kiessling@ki.se)

**Background**

Ex vivo expansion of T cells for adoptive cell therapy (ACT) of cancer is commonly done with cytokines and stimuli for efficient activation and expansion of large numbers of tumor-specific T cells. This inevitably leads to excessive T cell differentiation ex vivo, and decreased T cell functionality and persistence in vivo. Importantly, T cell differentiation is tightly controlled by epigenetic mechanisms that could be targeted therapeutically. The BET protein BRD4 has been reported to regulate expression of the transcription factor BATF that drives CD8+ T cells towards a more effector-like phenotype, while BRD4 inhibition by the drug JQ1 prevented this and thereby enhanced in vivo T cell persistence and function in adoptive immunotherapy models [1]. Ph-29089 is a chemically modified self-delivery RNA inhibitor containing an asymmetric duplex structure (≤ 15 base pair duplex) and a single-strand phosphorothioate tail targeting the BET protein BRD4. PH-29089 is efficiently delivered to immune cells, including T cells, without the need for specialized formulations or mechanical transfection as is observed with current RNAi’s.

**Methods**

Purified human CD8+ T cells were expanded using the rapid expansion protocol (REP) developed by the National Cancer Institute. Flow cytometry was used to study Ph-29089 for its ability to knock down BRD4 at the protein level in expanding T cells, and to determine T cell differentiation status during and immediately after ex vivo expansion. Release of IFNγ by T cells cocultured with tumor cells was assessed by ELISA.

**Results**

Ph-29089 elicited a concentration dependent silencing of BRD4 protein with an IC50 of 1-2 μM. The BRD4 silencing persisted at least 5 days post-treatment, whereas media and non-targeting control (NTC) did not significantly affect BRD4 protein levels. Compared to untreated, NTC-treated and JQ1-treated CD8+ T cells, Ph-29089-treated CD8+ T cells contained higher percentages of central memory and stem cell-like memory T cells, as determined by expression of CD45RA, CCR7, CD62L and CD95. Moreover, Ph-29089-treated CD8+ T cells displayed superior functionality, as indicated by enhanced IFNγ production when exposed to the allogeneic melanoma cell lines A375 and ROAL.

**Conclusions**

These findings support the hypothesis that BRD4 silencing by Ph-29089 is a viable approach for expanding T-cells with superior anti-tumor potential for adoptive cell therapies.

**Reference**

1. Kagoya Y, Nakatsugawa M, Yamashita Y, Ochi T, Guo T, Anczurowski M, et al. BET bromodomain inhibition enhances T cell persistence and function in adoptive immunotherapy models. J Clin Invest. 2016 126(9):3479–94.

**Ethics Approval**

This study was carried out in accordance with the recommendations of Karolinska Institutet review board with written informed consent from all subjects. All subjects gave written informed consent in accordance with the Declaration of Helsinki. The protocol was approved by the Stockholm Regional Ethics Committee, with approval number (2011/143-32/1).

#### P223 Withdrawn

#### P224 Case studies of sarcoma and MRCLS following treatment with NY-ESO-1 TCR T Cells (GSK3377794): correlates of predictable response characteristics

##### Brian Van Tine, MD, PhD^1^, Sandra D'Angelo, MD^2^, Alexandra Gyurdieva^3^, Laura Johnson^3^, David Turner^3^, Jenna Tress^3^, M. Phillip DeYoung^3^, Yuehui Wu^3^, Aisha Hasan, MBBS MD^3^, Dejka Araujo, MD^4^

###### ^1^Washington University in St. Louis, St. Louis, MO, United States; ^2^Memorial Sloan Kettering Cancer Center, New York, NY, United States; ^3^GlaxoSmithKline, Collegeville, PA, United States; ^4^MD Anderson Cancer Center, Houston, TX, United States

####### **Correspondence:** Brian Van Tine (bvantine@wustl.edu)

**Background**

Genetically engineered NY-ESO-1 specific T cells (NY-ESO-1 T Cells; GSK3377794) are autologous CD4+ and CD8+ T cells transduced with a self-inactivating lentiviral vector to express an affinity-enhanced NY-ESO-1-specific T-cell receptor. Phase 1 and 2 trials are evaluating GSK3377794 in solid tumors and hematologic malignancies. This study will review biomarker data for eight patients from two ongoing phase 1/2 pilot studies of GSK3377794 in synovial sarcoma (SS; NCT01343043; N=7) and myxoid/round-cell liposarcoma (MRCLS; NCT02992743; N=1) with prolonged response and stable disease (SD).

**Methods**

Patients who were progression free ≥4 months following first infusion were selected. All received the same lymphodepletion (30 mg/m2 x3D fludarabine, 600 mg/m2 x3D cyclophosphamide) followed by GSK3377794 infusion. Six patients with SS were eligible for second infusion and received higher-dose lymphodepletion (30 mg/m2 x4D fludarabine, 1800 mg/m2 x2D cyclophosphamide) before second infusion. Pretreatment biopsies were analyzed for CD3 infiltration by RNAScope. Transduced cell persistence was measured by quantitative PCR of transgene vectors peripheral blood mononuclear cell (PBMC) DNA. Cytokine expression was measured by Meso Scale Discovery immunoassay. PBMC phenotypes were characterized by flow cytometry.

**Results**

Five of seven patients with SS had SD for 17.8–105 weeks; two had partial response/complete response (PR/CR) per RECIST1.1. The patient with MRCLS had PR per RECIST1.1 lasting 8.8 months. Six of seven patients with SS received second infusion; 2/7 had SD, 3/7 had PR, 1/7 had CR. Immunohistochemistry revealed ≥50% of cells with 2+/3+ NY-ESO-1 expression; this was maintained before second infusion. Baseline tumor samples consistently showed 10 fold over first infusion; of these, there was one PR and one CR. Cytokine increases reflecting immune cell activation (eg, IFN gamma, IL-6, and IL-2R alpha were observed 4–7 days after both infusions. Transduced T cells within manufactured product showed increased expression of activation markers (eg, CD28, ICOS, and CD40L) versus T cells from apheresis. In 2 patients, transduced CD8 cells primarily had T effector memory RA+ (CD45RA+CCR7-) and T effector memory (CD45RA-CCR7-) phenotype. In one, 34.3% transduced CD8 cells had T stem cell memory (CD45RA+CCR7+) phenotype.

**Conclusions**

SS and MRCLS tumors initially show low immune cell infiltration. Upon GSK3377794 infusion, increased expression of activated immune cell cytokines was observed in serum from selected patients. Further analysis can provide insights into clinical response characteristics and identification of predictive biomarkers.

**Acknowledgements**

Medical writing assistance was provided by provided by Fiona Woodward and Chloe Stevenson of Fishawack Indicia Ltd, UK. These studies (NCT01343043 and NCT02992743) were funded by GlaxoSmithKline (GSK).

**Trial Registration**

NCT01343043 and NCT02992743

**Ethics Approval**

This study was approved by the appropriate institutional review boards and independent ethics committees.

#### P225 Generating iPSC-derived CAR T cells with an endogenous T cell phenotype and conventional CAR T functionality

##### Zhiqiang (Daniel) Wang, PhD^1^, Hellen McWilliams-Koeppen, MS^1^, Christian Huynh, BS^1^, Hernan Reza^1^, Vibhuti Vyas^1^, Xiuli Wang, PhD^1^, Wen-Chung Chang, MS^1^, Julie Ostberg, PhD^1^, Renate Starr, MS^1^, Jamie Wagner^1^, Brenda Aguilar, BS^1^, Xiwei Wu^1^, Jinhui Wang^1^, Wei Chen^1^, Chris Seet^2^, Gay Crooks^2^, Christine Brown, PhD^1^, Stephen Forman, MD^1^

###### ^1^City of Hope, Duarte, CA, United States; ^2^UCLA, Los Angeles, CA, United States

####### **Correspondence:** Zhiqiang (Daniel) Wang (zhwang@coh.org); Christine Brown (cbrown@coh.org); Stephen Forman (sforman@coh.org)

**Background**

Chimeric Antigen Receptor (CAR) T cell therapy is a revolutionary cancer treatment that genetically alters T cells to redirect and harness their cancer killing potential. Currently FDA approved CAR T cell products are autologous-based, requiring individualized blood apheresis and manufacture. Deriving patient-specific CAR T cell products is expensive, laborious, and time consuming, with logistical and regulatory challenges. The success rate of autologous CAR T cell therapy is also limited by the urgency of acute and aggressive cancers, uncertainty over T cell number, and intrinsic differences in T cell functionality. Generating CAR T cells from induced pluripotent stem cells (iPSC) holds encouraging prospect for generating ‘off-the-shelf’ CAR T cell products and overcoming these challenges. iPSCs can proliferate almost infinitely while keeping their pluripotency and lineage differentiation potential. However, the complexity of T cell development and disturbance of T cell differentiation by CAR expression creates a challenge for successful iPSC-derived CAR T cell generation. Previously reported iPSC-derived CAR T cells showed innate like phenotypes with weak antigen-specific cytotoxicity and compromised cytokine production [1].

**Methods**

In our current study, we generated iPSC lines from healthy donor T cells by an integration-free method using iPSC reprograming episomal vectors. The iPSC cells were transduced with clinical grade lentivirus to express CD19-specific CARs (CD19CAR), sorted and colonized to generate a homogeneous CAR+ iPSC cell bank. By using a 3D co-culture system, we successfully generated iPSC-derived CD19CAR T cells.

**Results**

The produced iPSC-derived CD19CAR T cells have a surface marker phenotype (CD3+CD5+CD7+TCRalphabeta+CD8alphabeta+ and CD3+CD5+CD7+TCRalphabeta+CD4+) and gene expression signatures typical of natural T cells. These iPSC-derived CD19CAR T cells expanded robustly within two weeks (~100 fold), and showed potent antigen-specific cytotoxicity against CD19+ parental tumor cells such as NALM6 and Raji comparing to their CD19 knockout control cells. It is intriguing that the in vitro cytotoxicity potency of iPSC-derived CD19CAR T cells was superior to conventional PBMC-derived CAR T cells generated from the same donor. These iPSC-derived CD19CAR T cells also demonstrated efficient degranulation activity and a Th1 cytokine profile (e.g. IFNgamma and TNFalpha when challenged with CD19+ target cells. Furthermore, these cells demonstrated potent anti-tumor activity in vivo in a NSG mouse model using NALM6 as target tumor.

**Conclusions**

Our study demonstrates the feasibility of generating naturalistic and functional CAR T cells from iPSCs, which may provide utility in the development of ‘off-the-shelf’ CAR T cell manufacturing strategies.

**Acknowledgements**

This research is supported by Mustang Bio. Inc.

**Reference**

1. Themeli, M., et al., Generation of tumor-targeted human T lymphocytes from induced pluripotent stem cells for cancer therapy. Nat Biotechnol, 2013. 31(10): p. 928-33.

**Ethics Approval**

The study was approved by the COH Institutional Review Board (IRB) and Office of Human Subjects Protection.

#### P226 Iovance Gen2 TIL manufacturing process produces drug products that exhibit favorable quality attributes for adoptive cell transfer across 5 solid tumor indications

##### Seth Wardell, Maritza Lienlaf-Moreno, Lavakumar Karyampudi, Anand Veerapathran, PhD, Ian Frank, Michelle Blaskovich, BS, Kenneth Onimus, Arvind Natarajan, Maria Fardis, PhD, MBA, Joe Wypych

###### Iovance Biotherapeutics, Inc., Tampa, FL, United States

####### **Correspondence:** Joe Wypych (joe.wypych@iovance.com)

**Background**

The Iovance Gen2 manufacturing process is a robust T-cell expansion platform that produces a cryopreserved drug product after a 22-day manufacturing period. Gen2 represents a flexible closed cell production process that is scalable to meet commercial demand. Drug products generated by this process display favorable quality attributes for adoptive transfer and the method is reproducible across 5 solid tumor indications at clinical scale.

**Methods**

Methods to assess proliferation, phenotype, and function were applied to in-process and final drug products generated with Gen2 at clinical scale to determine fit within the internally defined target product profile. TIL expansion was assessed through automated enumeration of total and viable nucleated cells. Culture health was assessed through cellular viability determined by DAPI exclusion. Immunophenotyping was performed to determine identity and purity as well as relative levels of activation and differentiation of the cell product. Cellular function was evaluated as the ability of the cell product to secrete IFN-ɣ in response to CD3, CD28, and 4-1BB receptor engagement.

**Results**

Reported herein is the collective experience at Iovance for expansion of TIL from five tumor types. The Iovance Gen 2 manufacturing process achieved doses comparable to lifileucel (LN-144, melanoma) and previously published methods across 5 primary tumor indications (Melanoma: mean 2.83 x 10e10 viable cells, n=82; Cervical: mean 2.31 x 10e10 viable cells, n=53; Head & Neck: mean 5.82 x 10e10 viable cells, n=12; Lung: mean 2.09 x 10e10 viable cells, n=3; Sarcoma: mean 1.12 x 10e10 viable cells, n=5). Quality attributes of drug products generated with Gen 2 were comparable across all 5 primary tumor indications evaluated in terms of T-cell purity, expression of costimulatory molecules, and memory subsets. Gen 2 drug products across all 5 additional indications continued to exhibit robust capacity to produce INF-ɣ upon reactivation, comparable to lifileucel.

**Conclusions**

The Iovance Gen2 manufacturing process allows for the rapid generation of clinical scale doses for patients in urgent need of therapy. The cryopreserved drug product introduced critical logistical efficiencies and flexibility in distribution that overcame traditional barriers to the commercialization of TIL therapy. Gen 2 drug products exhibit favorable quality attributes for adoptive transfer including high levels of co-stimulatory molecules, and a robust capability to secrete cytokine upon reactivation. These characteristics are reproducible across a broad range of solid tumor indications at a high manufacturing success rate opening the door for many more patients to benefit from this highly beneficial therapy. [1,2,3]

**References**

1. Donia M, Junker N, Ellebaek E, Andersen MH, Straten PT, Svane IM. Characterization and comparison of ‘Standard’ and ‘Young’ tumor infiltrating lymphocytes for adoptive cell therapy at a Danish Translational Research Institution. Scand. J. Immunol. 2011;75:157–67.

2. Besser MJ, Shapira-Frommer R, Treves AJ. et al. Clinical responses in a phase II study using adoptive transfer of short-term cultured tumor infiltration lymphocytes in metastatic melanoma patients. Clin Cancer Res. 2010;16:2646–55.

3. Jin J, Sabatino M, Somerville R, Wilson JR, Dudley ME, Stroncek DF, et al. Simplified method of the growth of human tumor infiltrating lymphocytes in gas-permeable flasks to numbers needed for patient treatment. J Immunother 2012;35:283–92.

#### P227 Co-expression of the metabolic enzyme GOT2 with a GPC3-targeted CAR-T overcomes challenges of the solid tumor microenvironment, substantially improving therapeutic efficacy in solid tumor xenografts

##### Kathleen Whiteman, MS, Tapasya Pai, Eugene Choi, Taylor Hickman, Tyler Johnson, Luke Barron, PhD, Taylor Friedman, Madaline Gilbert, Binzhang Shen, Seth Ettenberg, Kathleen McGinness, Greg Motz

###### Unum Therapeutics, Cambridge, MA, United States

####### **Correspondence:** Greg Motz (greg.motz@unumrx.com)

**Background**

The metabolic demands of cancer cells in the solid tumor microenvironment (TME) create an unfavorable T cell environment through depletion of critical nutrients and amino acids and accumulation of waste products. This drives T cell dysfunction and inhibits the effectiveness of immunotherapies. To overcome these and other TME challenges, we developed the BOXR (bolt-on chimeric receptor) platform in which engineered T cells co-express both a chimeric-targeting receptor and a “bolt-on” transgene [1]. In a screen of 100+ genes for enhanced T cell function when co-expressed with an anti-glypican-3 (GPC3) CAR, we identified the first candidate of our BOXR platform, BOXR1030, which co-expresses the transgene glutamic-oxaloacetic transaminase 2 (GOT2), a critical enzyme involved in mitochondrial metabolism.

**Methods**

We compared functional and phenotypic readouts of second-generation GPC3 CAR-T cells with BOXR1030. Broad transcriptome profiling, metabolic characterization, and comprehensive phenotypic assessments were performed; T cell proliferation and cytokine production under TME-stress conditions (limiting nutrients and hypoxia) were evaluated. In vivo, we assessed T cell anti-tumor activity, expansion and phenotype using GPC3-expressing solid tumor xenograft models in mice.

**Results**

The addition of GOT2 had pleiotropic effects on BOXR1030 T cells, improving multiple T cell functions relative to parent GPC3 CAR-T cells. BOXR1030 CD4+ T cells had greater polyfunctionality relative to parent CAR-T. BOXR1030 showed improved proliferation in vitro, including against TME-challenges. BOXR1030 CD8+ T cells had a greater proportion of less differentiated CD27+ cells following production, and CD8+ T cells evaluated ex vivo from xenograft tumors had substantially diminished level of inhibitory receptors (PD-1, Tim-3) suggesting resistance to exhaustion in the TME (Figure 1). Further, BOXR1030 was highly efficacious against GPC3-expressing solid tumor models that resisted parental CAR-T therapy (Figure 2), and activity was associated with improved T cell expansion and persistence in peripheral blood.

**Conclusions**

Co-expression of a metabolic gene to enhance T cell function is a novel approach to cell therapy for solid tumors. BOXR1030 had substantially improved T cell phenotype and function in diverse ways relative to the parent GPC3 CAR, and GOT2 conferred superior activity against numerous TME challenges both in vitro and in vivo. These results demonstrate that engineering of T cell immunometabolism is an effective and potent strategy to overcome the challenges of the solid tumor microenvironment. IND-enabling studies with BOXR1030 are underway with the expectation that BOXR1030 will be evaluated clinically in the treatment of GPC3+ malignancies.

**Reference**

1. Barron L, Whiteman K, Gilbert M, Pai T, Snyder M, Fray M, Nelson A, Johnson T, Lakeman K, Shin J, Boomer R, Ettenberg S, McGinness K, Motz G. Select metabolic and costimulatory “bolt-on” transgenes enhance chimeric receptor-bearing T cell activity against solid tumors. J Immunother Cancer. 2018;6(Suppl 1):114:110-111, abstract P216.

**Ethics Approval**

This study was approved by Unum Therapeutics’ Institutional Animal Care and Use Committee (IACUC); approval number 2016-04-004.

**Fig. 1 (abstract P227).**
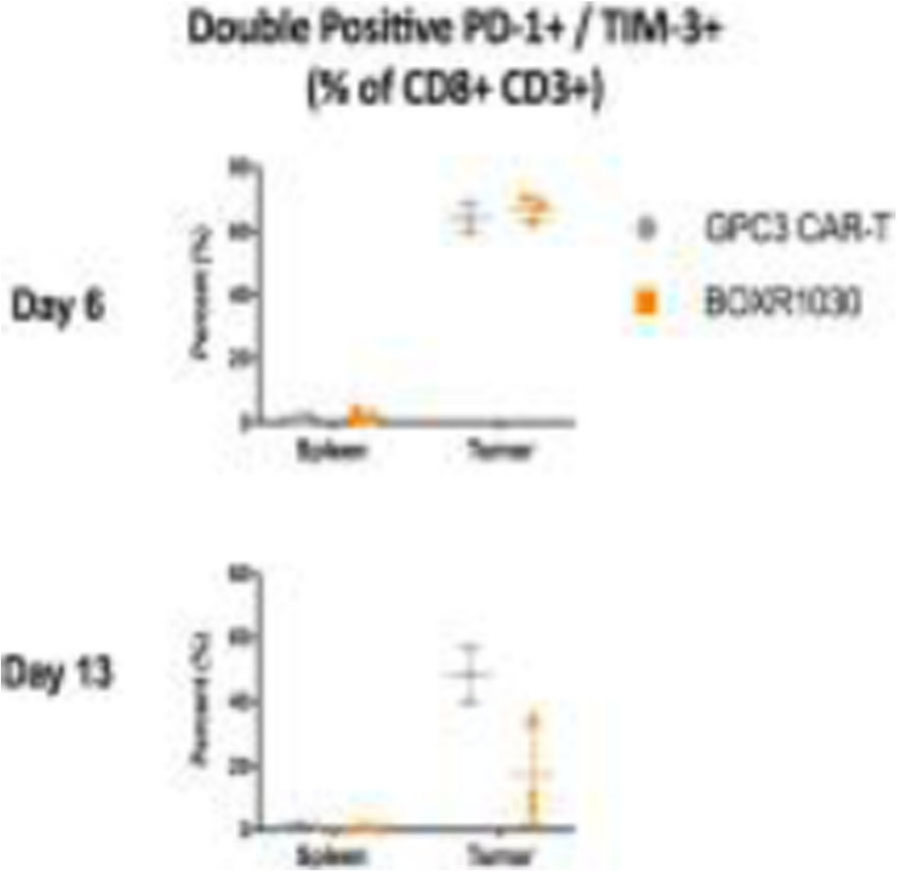
See text for description

**Fig. 2 (abstract P227).**
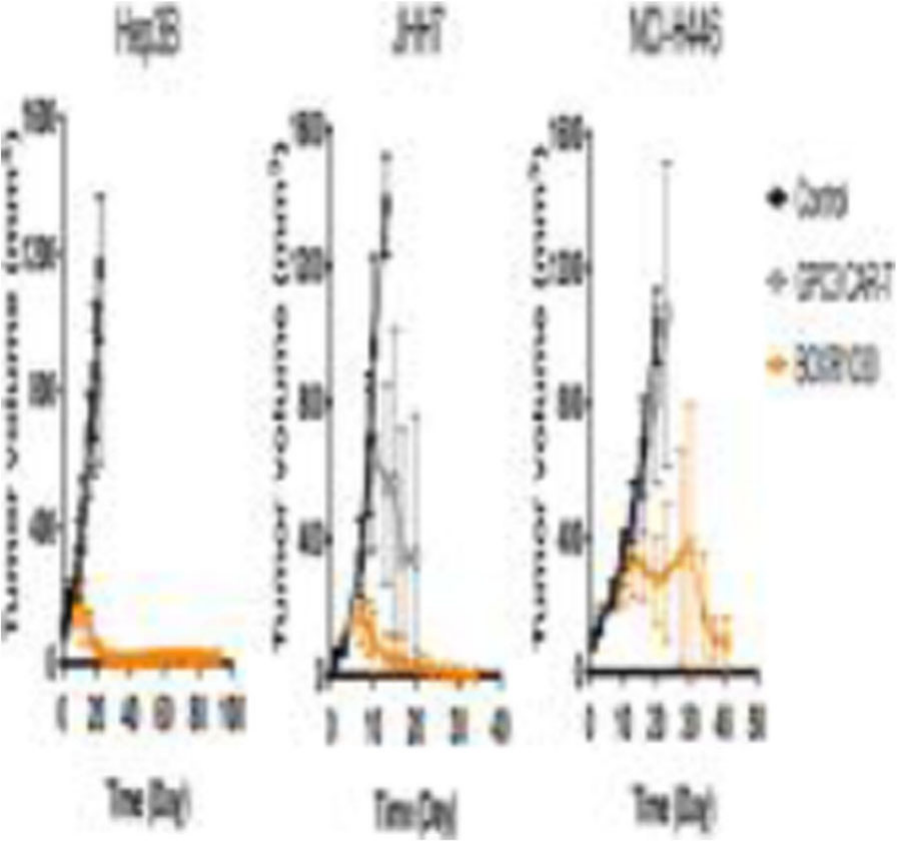
See text for description

#### P228 Tumor infiltrating lymphocyte recognition of shared neoantigens from mutated DNA repair/remodeling proteins in a patient with metastatic pancreatic adenocarcinoma

##### Ghanshyam Singh Yadav, PhD^1^, Chetana Bhaskarla, PhD^1^, Smriti Chaurasia, PhD^1^, Joshua Tobin^1^, Xinming Zhuo^2^, Yinghong Pan^2^, Annerose Berndt^2^, Udai Kammula, MD, FACS^1^

###### ^1^University of Pittsburgh, Pittsburgh, PA, United States; ^2^UPMC Genome Center, Pittsburgh, PA, United States

####### **Correspondence:** Udai Kammula (kammulaus@upmc.edu)

**Background**

Defective DNA repair, a hallmark of cancer, results in genomic instability and accumulation of genetic abnormalities in many malignancies. Adoptive cell transfer (ACT) using autologous tumor infiltrating lymphocytes (TIL) represents a personalized cancer immunotherapy capable of targeting shared and private neoantigens resulting from tumor somatic mutations. We sought to interrogate TIL neoantigen reactivity in a tumor harboring a somatic mutation in the DNA repair gene, ATM (Ataxia-Telangiectasia Mutated).

**Methods**

TIL cultures were generated from a surgically resected pancreatic cancer metastasis harboring a somatic ATM mutation. Anti-tumor reactivity of TIL culture was assessed by coculturing TIL with autologous tumor cells and measurement of IFN-gamma release and upregulation of 4-1BB by flow cytometry. Tumor specific mutations were identified by whole genome sequencing (WGS). DNA fragments encoding the altered gene sequences were synthesized and expressed in autologous dendritic cells by RNA electroporation to enable neoantigen reactivity screening. T cell receptor (TCR) sequencing was performed after single-cell sorting of tumor reactive TIL followed by primer specific PCR for TCR alpha and beta chains.

**Results**

Analysis of the pancreatic cancer TIL revealed high level reactivity against autologous tumor. Tumor WGS identified 141 somatic mutations (107 SNVs; 19 frameshifts; 15 other). Screening for neoantigen reactivity identified CD8+ T-cell responses against a missense mutation in ATM (23% of TIL) and a frameshift mutation in ARID1A (32% of TIL) but not against respective wild type gene products. TCR sequencing identified a single unique TCR specific for ATM and ARID1A, respectively. Genes encoding the ATM specific TCR were retrovirally transduced into healthy donor T-cells and found to confer strong ATM mutation reactivity without recognition of wild type ATM.

**Conclusions**

Over 50% of the TIL expanded from a patient with metastatic pancreatic cancer were found to recognize neoantigens from either mutated ATM or ARID1A, which play a crucial role in DNA repair and chromatin remodeling. ACT using T-cells genetically engineered with these TCRs represents an attractive immunotherapy for patients harboring these shared tumor mutations.

**Acknowledgements**

The study was supported by UPMC Immune Transplant and Therapy Center (ITTC).

**Ethics Approval**

This study was reviewed and approved by University of Pittsburgh Institutional Review Board. IRB#18010273.

#### P229 The next generation “off-the-shelf” universal CAR for adoptive immunotherapy

##### Weichih Yang, PhD^1^, Yun Ji^1^, Xiaobing Luo^1^, Huijuan Cui^1^, Michael Patrick^1^, Yutian Wei^1^, Shigui Zhu^1^, Jiaqi Huang^1^, Xin Yao^1^, Yihong Yao^1^, Aibing Liang^2^, Ping Li^2^

###### ^1^Cellular BioMedicine Group, Gaithersburg, MD, United States; ^2^Tongji Hospital of Tongji University, Shanghai, China

####### **Correspondence:** Yun Ji (yun.ji@cellbiomedgroup.com)

**Background**

Adoptive immunotherapy using autologous T cells redirected with chimeric antigen receptors (CARs) has emerged as a powerful means of treating cancer, such as B-cell malignancy. However, this approach is limited by the availability of autologous T cells especially for infant patients or patients undergoing multi rounds of chemotherapy.

**Methods**

Here we show that employment of the CRISPR-Cas9 system allows highly efficient multiplex gene editing of T Cell Receptor Alpha Constant and Beta-2-Microglobulin in primary human T cells, which is intended to avoid graft-versus-host-disease and minimize the immunogenicity of transferred cells. Furthermore, redirecting the gene-engineered cells with a B cell maturation antigen (BCMA) CAR led to their efficient destruction of BCMA+ tumor targets. To further improve the efficacy of these universal BCMA CAR T cells, we use a strategy to generate the next generation universal CAR T cells by starting from naive precursors and producing the CAR T cells in conditions favoring T memory stem (Tscm) cell expansion.

**Results**

These Tscm enriched gene-engineered BCMA CAR T cells demonstrated superior activity compared to the conventional universal CAR T cells based on their expansion, phenotype, IFN-gamma release, and cytotoxicity. An early phase clinical trial using this BCMA CAR in an autologous setting has demonstrated promising clinical readout (NCT03815383).

**Conclusions**

Therefore, we believe this next generation Tscm-enriched universal CAR T cells employing the same BCMA vector will provide another alternative choice for multiple myeloma patients in an “off-the-shelf” manner similar to other biological drugs.

#### P230 Activating antigen carriers for cancer therapy: preclinical immune responses drive tumor regression

##### Defne Yarar, PhD, Amritha Ramakrishnan, Katarina Blagovic, PhD, Katherine Seidl, Howard Bernstein, MD, PhD, Armon Sharei

###### SQZ Biotechnologies, Watertown, MA, United States

####### **Correspondence:** Defne Yarar (defne.yarar@sqzbiotech.com)

**Background**

Productive activation of the immune system by antigen presenting cells (APCs) loaded ex vivo has proven to be challenging. To overcome this, we have developed an approach that harnesses the natural process of red blood cell (RBC) clearance from the body to activate the immune response in vivo. Using the CellSqueeze® microfluidics platform, we have generated activating antigen carriers (AACs), engineered from RBCs, that are highly loaded with antigen and adjuvant and potently activate APCs in vivo. Here, we show that AAC-mediated antigen and adjuvant targeting to APCs drives antigen presentation in vivo and primes potent anti-tumor T cell responses.

**Methods**

To generate AACs, we loaded proteins or synthetic long peptide antigens together with adjuvants into murine or human RBCs with CellSqueeze®. Following intravenous AAC injection into mice, we measured AAC clearance kinetics from the blood and characterized the site and cell type of AAC uptake. In addition, we quantified endogenous immune responses to AAC administration by flow cytometry. To determine the ability of AACs to control subcutaneously implanted tumors, we measured tumor growth rates in mice treated either prophylactically or therapeutically with AACs. Finally, to assess if AACs could be engulfed by antigen-presenting cells in a human system, we quantified in vitro uptake of human AACs by monocyte-derived dendritic cells using flow cytometry and fluorescence microscopy.

**Results**

We demonstrate that CellSqueeze® loads antigen and adjuvant into AACs effectively. When administered into a mouse, these carriers are cleared from circulation within one hour and are engulfed by professional phagocytes in both the spleen and liver. Moreover, we find that murine SQZ AACs processed with CellSqueeze® stimulate model antigen CD4+ and CD8+ responses and cancer-associated antigen-specific CD8+ T cell responses in vivo. We observe that prophylactic and therapeutic AAC administration to mice strongly impedes tumor growth and extends survival. Following therapeutic immunization, the anti-tumor responses correlate with an over 10x increase in antigen-specific CD8+ tumor-infiltrating lymphocytes compared to untreated mice. Finally, in an in vitro human system, we demonstrate that AACs can be highly loaded with antigen and adjuvant using CellSqueeze®, and that these AACs can be engulfed by human monocyte-derived dendritic cells.

**Conclusions**

In summary, these results indicate that antigen and adjuvant delivery to APCs in vivo can effectively prime a potent anti-tumor response in mice and support the further study of SQZ AACs as an immunotherapy for cancer treatment.

#### P231 Invariant natural killer T cells as an allogeneic cell therapy platform

##### Burcu Yigit, PhD, Xavier Michelet, PhD, Simon Yue, Darrian Moskowitz, Mark Exley, Burcu Yigit, PhD

###### AgenTus Therapeutics, Lexington, MA, United States

####### **Correspondence:** Burcu Yigit (burcu.yigit@agentustherapeutics.com)

**Background**

AgenTus Therapeutics is developing innovative allogeneic and ‘off-the shelf’ cell therapies by utilizing invariant natural killer T cells (iNKT) to target solid and liquid tumors. iNKT cells are innate-like lymphocytes that bridge innate and adaptive immune responses. They can be activated via their invariant T cell receptor recognizing lipid antigens (e.g. alpha-Galactosylceramide) presented on CD1d molecules, through NKG2D - NKG2D ligand interactions, and by cytokines. Upon activation, large amounts of IFN-gamma production leads to recruitment and activation of T cells and NK cells. iNKT cells also exert potent direct cytolytic activity. While they are found in very low numbers in human blood (~ 0.01% of T lymphocytes), some of their unique properties make them valuable for cell therapy platforms. Due to their invariant antigen receptor, their ability to cause GvHD is minimal, and in fact, they have been demonstrated to suppress GvHD in BMT settings. This facilitates the use of iNKT cells in an allogeneic cell therapy setting. In addition, iNKT cells are very efficient in infiltrating solid tumors to exert their cytotoxic function and activate other anti-tumor immune cells.

**Methods**

Due to low frequency of circulating iNKT cells, we have developed and optimized a method to isolate and generate large numbers of these cells in vitro for use in ‘off-the-shelf’ and allogeneic setting.

**Results**

We can achieve over 40,000-fold expansion of iNKT cells through stimulation of the invariant TCR in less than 30 days. Importantly, after such massive expansion, iNKT cells retain their inherent cytotoxic capacity and cytokine production in response to tumor cells.

**Conclusions**

AgenTus is applying its proprietary Antigen Receptor platforms to identify novel CARs and TCRs directed against tumor-specific antigens. We believe that through modification with tumor-targeted CARs and TCRs, iNKT cells will serve as potent allogeneic cell therapy vehicles. This should enable an ‘off-the-shelf’ approach for improving patient access to cell therapy.

#### P232 Characterization of ADCC resistance in multiple cancer types

##### David Zahavi, MS, BS, Yongwei ZHANG, Sandra Jablonski, PhD, Louis Weiner, MD

###### Georgetown University, Washington, DC, United States

####### **Correspondence:** Louis Weiner (weinerl@georgetown.edu)

**Background**

Antibody-dependent cell-mediated cytotoxicity (ADCC) is an important mechanism of action in targeted monoclonal antibody (mAb) cancer immunotherapy. The majority of patients who receive targeted mAbs develop resistance to therapy and there remains a great need to understand resistance mechanisms. In vitro modeling of ADCC provides an experimental system for uncovering tumor cell based immune resistance mechanisms.

**Methods**

Utilizing our in vitro model system of continuous selection pressure with NK92-CD16V effector cells and the mAbs Cetuximab and Trastuzumab we have generated three ADCC resistant cell lines from parental A431, SK-OV-3, and FaDu cells.

**Results**

We show that the induction of ADCC resistance in all three cells lines involves a loss of target cell adhesion properties required for the establishment of an immune synapse, NK cell activation, and target cell cytotoxicity. Remarkably, ADCC-resistant cells possess reduced cell surface expression of multiple proteins that contribute to intercellular interactions and immune synapse formation. We have termed the loss of a selection of cell surface proteins which contributes to ADCC resistance Testudinidosis. This phenomenon is characterized by dysregulation of protein trafficking and subcellular localization of the cell surface molecules. Additionally, ADCC resistant cell lines exhibit aberrant IFN/STAT1 signaling.

**Conclusions**

Using multiple cell lines to model ADCC resistance has led to the discovery of a shared mechanism of resistance across cancer types that may reveal potential therapeutic targets for combination immunotherapy.

#### P233 RTX-321, an allogeneic artificial antigen presenting red cell therapeutic, expressing MHC I-Peptide, 41BBL and IL12, promotes antigen-specific T cell expansion and anti-tumor activity in HPV16+ tumors

##### Xuqing Zhang, PhD, Tiffany Chen

###### Rubius Therapeutics, Cambridge, MA, United States

####### **Correspondence:** Tiffany Chen (tiffany.chen@rubiustx.com)

**Background**

Autologous CAR-T therapy has demonstrated efficacy in a small subset of hematological cancers. The wider adoption of antigen-specific therapies has been limited by significant toxicity and a lack of effectiveness in solid tumors. Manufacturing is costly, time-consuming and difficult to scale. To address these limitations, Rubius Therapeutics has genetically engineered red cells to create an allogeneic artificial antigen-presenting cell (aAPC), called RTX-321, for the treatment of HPV16+ advanced solid tumors. RTX-321 presents an HPV E7 peptide on major histocompatibility complex I (MHC I [HPV]), a costimulatory signal (4-1BBL) and a membrane-bound cytokine (IL-12) on the cell’s surface to mimic the human immunobiology of T cell APC interactions. RTX-321 is designed to activate and expand tumor-specific T cells present within the patient and eliminates the need for manufacturing patient-derived T cells.

**Methods**

As a proof of principle, red cells engineered to express mouse MHC I H-2Kb loaded with OVA 257-264 peptide (H-2Kb OVA), murine 4-1BBL (m4-1BBL) and murine IL-12 (mIL-12) were used to stimulate OT1 cells.

**Results**

Compared to cells expressing H-2Kb OVA or m4-1BBL alone, these cells induced up to a 14-fold expansion of OVA antigen-specific OT1 cells in vivo. These expanded cells displayed a memory phenotype and enhanced antigen-specific tumor killing of EG7.OVA tumor cells. To test in vivo efficacy, a mouse surrogate, mRBC-321, was created using murine red cells chemically conjugated with H-2Kb OVA, m4-1BBL and mIL-12. Naïve OT1 cells were transferred into tumor bearing mice followed by mRBC-321 treatment. Dramatic dose-dependent expansion of OT1, comprised of functional effector and central memory phenotypes, was observed in peripheral blood (9468-fold Tem; 146-fold Tcm expansion) and secondary lymphoid organs (3323-fold Tem; 725-fold Tcm expansion in spleen) on day 7. mRBC-321 treatment using an EG7.OVA tumor model resulted in tumor regressions in 12/16 mice, 6 of which were cured, and increased survival (p

**Conclusions**

Based on these results, human RTX-321 expressing h4-1BBL, hIL-12, and HLA-A*02 with an HPV E7 peptide was generated to activate and expand HPV E7-specific T cells. RTX-321 activated TCR signaling in an engineered HPV E7-specific TCR Jurkat line and stimulated 4-1BB and IL-12R signaling in respective reporter cell lines. These results support the clinical development of RTX-321, which is currently in IND-enabling studies for the treatment of HPV16+ advanced solid tumors.

#### P234 DAP10 and DAP12 signaling based CAR circumvents ligand-dependent tonic signaling and mediates potent anti-tumor response in vivo

##### Alan Epstein, MD, PhD, Long Zheng, Long Zheng

###### University of Southern California, Los Angeles, CA, United States

####### **Correspondence:** Alan Epstein (aepstein@usc.edu)

**Background**

The Lym-1 antibody which targets a unique, discontinuous epitope on the HLA-Dr protein expressed in human B-cell lymphomas and leukemias developed in the late 1970’s by co-author ALE has been found to be clinically safe and effective as an I-131 radioimmunoconjugate [1-3]. Based upon these early clinical data, we have generated a humanized version (huLym-1-B) to construct chimeric antigen receptors (CARs) using the single chain variable fragment (ScFv) of huLym-1-B to treat Lym-1 positive tumors.

**Methods**

The antibody ScFv was ligated to a lentivirus vector in frame with CAR backbone and CAR lentiviruses were produced and used to transduce primary CD3+ human T-cells. After transduction, we characterized the expansion, the effector function, and the immune-phenotypes of CAR-T cells. The in vivo efficacy of the CAR-T cells was measured in a metastatic Raji lymphoma xenograft 8 days after the iv injection of 1 million tumor cells in NSG mice.

**Results**

huLym-1-BCAR was successfully transduced at an efficiency between 50-80%. Surprisingly, the CAR transduced T-cells showed limited expansion, reaching approximately 8-fold expansion after 11 days of culture compared to mock T-cells which had a robust 310 to 380-fold expansion. In expanded CAR-T cells, over 28% of cells showed phosphorylated CD3z and increased PD-1 and LAG3 expression indicative of T-cell exhaustion which was not detected in mock T-cells. Since Lym-1 recognizes a conformational epitope on HLA-Dr which may be weakly expressed by activated T-cells, we speculated that the huLym-1-BCAR may stimulate the transduced T-cells to cause tonic signaling and expansion failure. This was confirmed by flow cytometry that detected huLym-1-B but not huLym-1-Bmut, a huLym-1-B mutant that lost its binding ability, binding on T cells. Meanwhile, neither huLym-1-Bmut based CAR or huLym-1-BCAR with inactive CD3z domains showed decreased expansion or increased CD3z phosphorylation.

**Conclusions**

Although the proliferation issue did not compromise CAR-T cells’ effector function, this would impose a potential challenge for large-scale manufacture. We discovered that replacing the BB3z in huLym-1-BCAR with signaling domains from DAP10 and DAP12 addressed this proliferation issue but enabled potent anti-tumor efficacy in vivo. In addition, the expanded huLym-1-B DAPCAR-T cells enabled a lower dose of injected CAR T-cells to induce durable control of metastatic lymphoma in mice with large tumor burdens. Further testing of DAP signaling sequences in other CAR T-cells may show that this change also improves the clinical efficacy of CAR T-cell therapy directed against other antigens targeted in both hematopoietic and solid tumors.

**Acknowledgements**

This work is supported by Cell Biotherapy, Inc., Los Angeles, CA.

**References**

1. Epstein AL, et al. Two new monoclonal antibodies, Lym-1 and Lym-2, reactive with human B-lymphocytes and derived tumors, with immunodiagnostic and immunotherapeutic potential, Cancer Res. 1987; 47:830-840.

2. DeNardo GL, et al. Low-dose, fractionated radioimmunotherapy for B-cell maligancies using 131I-Lym-1 antibody. Cancer Biother Radio. 1998; 13:239-254.

3. Hu E, et al. A phase 1a clinical trial of LYM-1 monoclonal antibody serotherapy in patients with refractory B cell malignancies. Hematol Oncol. 1989; 7:155-166.

**Ethics Approval**

This study was approved by the IRB of the University of Southern California, protocol number HS-16-00029 approved 2-29-16, and by IACUC protocol 20585.

### Checkpoint Blockade Therapy

#### P235 A dense, proliferative myeloid and T cell-rich, immune infiltrate characterizes immunotherapy-induced skin rash

##### Cormac Cosgrove, PhD, Cory Kosche, Suyeon Hong, Caroline Le Poole, Jennifer Choi

###### Northwestern University, Chicago, IL, United States

####### **Correspondence:** Cormac Cosgrove (cormac.cosgrove@northwestern.edu)

**Background**

Checkpoint inhibitor immunotherapy is associated with a unique toxicity profile, collectively known as immune related adverse events (irAEs). One of the earliest and most common irAEs is skin rash. Interestingly, development of rash has been associated with improved survival, and is likely an early indicator of a successfully activated immune response. More severe toxicities also occur, can affect nearly every organ and are clinical justification for dose reduction or termination of immunotherapy. However, the mechanism underpinning rash development and its link to disease outcome or toxicities is not known. Thus, we examined the makeup of immunotherapy-associated skin rash infiltrates with the goal of uncovering mechanistic insights behind rash development.

**Methods**

Immunohistochemistry was used to describe the immune infiltrate in skin biopsies from healthy subjects and from a lesional site of patients who developed rash secondary to immunotherapy. Rash samples were obtained from 7 patients receiving α-PD1, α-CTLA4/α-PD1 combination or α-PDL1/α-NKG2A combination. Acetone-fixed sections from frozen biopsies were stained for CD3, CD4, CD8, CD68, CD11c, CD1a, CD207, or Ki67. Cell abundance in the dermis was compared among groups.

**Results**

: Rash samples showed significant enrichment of T cells (CD3 p=0.01), CD8 T cells (p=0.03) and dendritic cells (CD11c p=0.03) in the dermis vs controls. More moderate enrichment of macrophages (CD68) was observed in rash versus control samples while the abundance of dermal Langerhans cells (CD1a or CD207) was comparable among groups. We observed more proliferating cells in the dermis of rash vs control samples (Ki67 p=0.024), associated with areas of dense immune infiltration. Ki67 expression was highly correlated with both CD68 (r=0.89; p=0.012) and CD11c (r=0.786; p=0.048), suggesting myeloid cell proliferation. Finally, stable or partial response to therapy tracked with either a dense T cell- or myeloid-cell infiltrate while patients who progressed displayed low infiltrate levels.

**Conclusions**

In conclusion, immunotherapy associated skin rash contains a complex immune infiltrate, with increased cellular proliferation among sites of dense infiltration. The highest levels of T cell or myeloid cell infiltrate were seen in stable or responding patients, suggesting a link between the immune profile of skin rash and therapy response.

**Ethics Approval**

The study was approved by The Northwestern University Ethics Board

#### P236 Nivolumab related side effects based on patient-reported outcomes: A multicenter study from real-life setting

##### Canan Karadas^1^, Zehra Gok Metin, Assoc Prof, PhD, RN^1^, Nur Izgu, PhD, RN^1^, Canan Porucu, MSc, RN^2^, Nuri Karadurmus, Prof Dr, MD^2^, Sadettin Kilickap, Prof Dr, MD^3^, Umut Demirci, MD^4^

###### ^1^Hacettepe University, Ankara, Turkey; ^2^Gulhane Training and Research Hospital, Ankara, Turkey; ^3^Hacettepe University Cancer Institute, Ankara, Turkey; ^4^Dr. A.Y. Ank Onco Tra and Res Hospital, Ankara, Turkey

####### **Correspondence:** Canan Karadas (karadas.canan@gmail.com)

**Background**

Immune checkpoint inhibitors provide an effective treatment option for patients with melanoma and other cancer types. Nivolumab, as an immune checkpoint inhibitor, acts via blockade of the PD-1 receptor, and limits immune responses against tumors. As reported for nivolumab, the anti-PD-1 antibodies can induce immune-related adverse events [1, 2]. Nivolumab related adverse effects compose of general, pulmonary, gastrointestinal, neurologic, skin, and infusion reactions. These adverse effects may be life threatening, so following of patients receiving nivolumab is essential for early diagnosis and management. Therefore, this study aimed to examine nivolumab related adverse effects based on patient- reported outcomes (PROs) from a real-life setting.

**Methods**

In total, 40 patients receiving first cycle of nivolumab were included from three hospitals located in Turkey. Patient Information Form and Patient Monitoring Checklist-Nivolumab were used for data collection. Patient Monitoring Checklist-Nivolumab included totally 46 questions: (21-general adverse effects, 3-pulmonary, 8-gastrointestinal, 5-neurological, 2-skin, and 7-infusion reactions) and two options in each item that evaluating presence of related adverse effect, as “yes” or “no”. PROs were analyzed by frequency and percentages.

**Results**

The mean age of patients was 55.47±16.48 years, and the majority of them (82.5%) had diagnosed with cancer longer than one year. The cancer diagnosis composed of melanoma (52.5%), renal cell carcinoma (17.5%), nasopharyngeal carcinoma (7.5%) and other cancer types (23.5%). Considering differences in terms of gender, only two items including 8th item related general adverse effects, quiring loss of hair, and 37th item evaluating changes in mental status, perception, judgement, and memory had a significant difference. Female patients reported higher adverse effects than male regarding aforementioned items (p

**Conclusions**

PROs related nivolumab in this study included fatigue, rash and itching, and diarrhea as consisted with previous reports. A comprehensive approach is needed to reduce and manage nivolumab related adverse effects.

**References**

1. Naidoo J, Page DB, Li BT, Connell LC, Schindler K, Lacouture ME, Wolchok JD. Toxicities of the anti-PD-1 and anti-PD-L1 immune checkpoint antibodies. Ann Oncol. 2015; 26:2375–2391.

2. Spain L, Diem S, Larkin J. Management of toxicities of immune checkpoint inhibitors. Cancer Treat Rev. 2016; 44:51–60.

**Ethics Approval**

The study was approved by clinical trials ethics committee of the University of Health Sciences Ankara Oncology Training and Research Hospital (decision number: 2018–06/52) and performed in accordance with the Helsinki Declaration.

#### P237 A case of anti-Zic4 antibody-mediated cerebellar toxicity induced by dual checkpoint inhibition in HNSCC

##### Sunil Iyer, MD, Nidah Khakoo, MD, Gabriella Aitcheson, MD, Marina Kushnirsky, MD, Cesar Perez, MD

###### University of Miami, Miami Beach, FL, United States

####### **Correspondence:** Sunil Iyer (sunil.iyer@jhsmiami.org)

**Background**

Combined checkpoint inhibition therapy targeting the PD-L1 and CTLA4 pathways has been a successful approach in the treatment of metastatic melanoma, leading to its investigation in the treatment of head and neck squamous cell carcinoma (HNSCC) with PD-L1 expression [1]. Despite the potential for excellent responses, an increased rate of autoimmune neurological toxicity and paraneoplastic conditions has been observed [2]. We present the case of a patient with metastatic HPV-positive HNSCC treated with ipilimumab/nivolumab who experienced severe cerebellar ataxia, with a positive screen for the anti-Zic4 antibody, which has been associated with cerebellar degeneration in small cell lung cancer (SCLC) and has not been reported in HNSCC [3].

**Results**

A 40-year-old man diagnosed with HPV-positive HNSCC with metastatic recurrence after radiation treatment of the initial tumor was started on a clinical trial of a DNA-PK inhibitor. His disease progressed, and given his PD-L1 tumor proportion score of 70% he was initiated on ipilimumab/nivolumab. After his second cycle, he presented with sudden blurred vision and mild ataxia, which rapidly progressed to severe ataxia and dysarthria. Autoimmune toxicity was suspected; initial brain imaging and serum testing were unremarkable. While awaiting the results of complex autoimmune and paraneoplastic CSF testing, he was treated with multiple modalities in an escalating fashion with minimal improvement, including pulse-dose corticosteroids, IVIG, and plasmapheresis. The paraneoplastic panel returned negative for common autoimmune culprits in cerebellar encephalopathy including anti-Hu and anti-Yo; however, anti-Zic4 was detected at borderline levels. Repeat MRI showed an enhancing lesion in the cerebellum. Finally, rituximab was initiated, and the patient is slowly improving. Notably, restaging scans show a mixed response with resolution of previously extensive metastatic disease in the thorax, however with worsening osseous lesions.

**Conclusions**

We present a case of anti-Zic4-mediated cerebellar toxicity in the setting of dual PD-L1/CTLA4 inhibition in the treatment of metastatic HNSCC. Anti-Zic4 has been historically associated with cerebellar-predominant paraneoplastic neurological disorders in SCLC [3], and to our knowledge, has not been described in HNSCC. Although the patient experienced an impressive partial response, he suffered grade 4 cerebellar neurotoxicity. Cases have demonstrated excellent clinical responses utilizing dual PD-L1/CTLA4 inhibition in HNSCC [4], however high-grade adverse events have also been reported in this regimen’s more established use in metastatic melanoma [2]. Despite the exciting advances in cancer immunotherapy, clinicians must be aware of the rare, debilitating, and possibly previously undescribed paraneoplastic and autoimmune toxicities that may occur.

**References**

1. Forster MD, Devlin MJ. Immune Checkpoint Inhibition in Head and Neck Cancer. Front Oncol. 2018; 8:310.

2. Pollack MH, Betof A, Dearden H. Safety of resuming anti-PD-1 in patients with immune-related adverse events (irAEs) during combined anti-CTLA-4 and anti-PD1 in metastatic melanoma. Ann Oncol. 2018; 29:250-255.

3. Bataller L, Wade DF, Graus F. Antibodies to Zic4 in paraneoplastic neurologic disorders and small-cell lung cancer. Neurology. 2004; 62.

4. Schwab KS, Kristiansen G, Schild HH. Successful Treatment of Refractory Squamous Cell Cancer of the Head and Neck with Nivolumab and Ipilimumab. Case Rep Oncol 2018;11:17–20.

**Consent**

Consent was obtained from the patient for publication of this abstract.

#### P238 Longitudinal immune and genomic monitoring reveals signatures of response and Immune-related adverse events in cancer patients receiving checkpoint inhibitor therapy

##### Shaheen Khan, PhD, David Gerber

###### UT Southwestern Medical Center, Dallas, TX, United States

####### **Correspondence:** David Gerber (david.gerber@utsouthwestern.edu)

**Background**

Despite the remarkable success of immune checkpoint inhibitor (ICI) therapy, a significant number of patients develop severe and unpredictable immune-related adverse events (irAEs) affecting a wide variety of organs. Concerns over irAE have led to the exclusion of patients with autoimmune disease from ICI clinical trials. Role of host genetic and immune factors in mediating irAEs remain unclear and it is not clear if the manifestations of irAEs is associated with response to therapy.

**Methods**

We used multi-faceted approach to identify blood-based biomarkers predictive of irAEs and response to ICI therapy. We characterized changes in host immune system in 200 patients receiving ICI therapy at baseline and post-immunotherapy (100 with irAEs and 100 without irAEs). We assessed genetic predisposition to autoimmunity in these patients using the Illumina GSA SNP array and via targeted resequencing of over 150 immunoregulatory loci including the HLA region. We evaluated serum levels of cytokines/chemokines, Antinuclear autoantibodies (ANA) and 124 autoantibodies and performed RNA sequencing and flow cytometry on peripheral blood mononuclear cells (PBMCs) at baseline and post immunotherapy in patients with and without irAEs.

**Results**

Our preliminary data analysis identified signatures of autoantibodies and cytokines correlating with response and toxicity. We also identified HLA haplotypes linked with autoimmunity in selected patients that developed immune-related adverse events. In this meeting, we will present immune and genetic correlates of irAEs and response to therapy in our patient cohort.

**Conclusions**

In-depth analysis of immune and genetic datasets is currently underway. We hope that our studies can help identify blood-based biomarker signatures predictive of irAEs and/or response and reveal novel insights into the mechanisms underlying irAE. Our current genetic data suggests further expanding the genetic studies in larger patient population to identify the role of underlying genetic predisposition to autoimmunity in mediating irAEs. We hope our findings may ultimately help identify customize therapy, expand use of immunotherapy and prevent toxicities.

#### P239 Immune checkpoint inhibitor-associated arthritis: a systematic literature review of case series and case reports

##### Michael Tiongson, BA^1^, Nilasha Ghosh, MD^2^, Carolyn Stewart, BA^3^, Karmela Chan, MD^2^, Bridget Gatto, MLIS^2^, Anne Bass, MD^2^

###### ^1^Albany Medical College, Nanuet, NY, United States; ^2^Hospital for Special Surgery, New York, NY, United States; ^3^Weill Cornell Medicine, New York, NY, United States

####### **Correspondence:** Nilasha Ghosh (GhoshN@HSS.edu)

**Background**

As immune checkpoint inhibitors (ICI) continue to revolutionize cancer treatment, immune-related adverse events (irAE) are becoming more prevalent. Inflammatory arthritis occurs in approximately 4% of ICI-treated patients [1] but remains poorly characterized. We performed a systematic literature review to identify all reports of ICI-associated inflammatory arthritis in order to describe it phenotypically and serologically.

**Methods**

PubMed, Embase and Cochrane databases were searched for publications reporting musculoskeletal irAEs secondary to ICI treatment through the search date, May 31, 2019. Publications were included if they provided individual patient-level data regarding the pattern of joint involvement. Two reviewers screened all abstracts and full texts to extract demographics, clinical features, serologies, treatment data and outcomes. Descriptive statistics were used to summarize results.

**Results**

4339 articles were screened, of which 67 were included (42 case reports, 15 case series, 10 retrospective chart reviews) encompassing 372 patients (Table 1). Mean age was 63 +/- 11 years; 61% patients were male. The majority of patients had metastatic melanoma (57%) and were treated with anti-PD1 or anti-PDL1 therapy (78%). Median time to onset of arthritis was 4 months (range: 1 day-53 months). 49% had polyarticular arthritis, 17% oligoarthritis, 3% monoarthritis, 10% arthralgia and 21% polymyalgia rheumatica (PMR). 9% tested positive for rheumatoid factor (RF) or cyclic citrullinated peptide (CCP) antibodies. 74% required corticosteroids and 45% required additional therapies, including 5% requiring a TNF inhibitor. 63% of patients achieved control of their musculoskeletal symptoms with treatment, and 32% were ultimately able to discontinue anti-rheumatic treatments. ICI were continued in 49%, transiently withheld in 11%, and permanently discontinued due to musculoskeletal irAEs in 13%. At last follow-up, 27% had progression of their cancer.

**Conclusions**

Half of reported ICI-associated arthritis cases have a polyarthritis (often in an RA distribution) but only 9% are seropositive. PMR is also commonly seen. The vast majority of ICI-arthritis cases are in melanoma patients treated with anti-PD1/PDL1 therapy. Most patients respond to steroids alone but about half require additional anti-rheumatic agents. Further studies are needed to determine long-term musculoskeletal outcomes in these patients and the impact of arthritis treatment on cancer survival.

**Reference**

1. Kostine M, Rouxel L, Barnetche T, Veillon R, Martin F, Dutriaux C, Dousset L, Pham-Ledard A, Prey S, Beylot-Barry M, Daste A. Rheumatic disorders associated with immune checkpoint inhibitors in patients with cancer—clinical aspects and relationship with tumour response: a single-centre prospective cohort study. Ann Rheum Dis. 2018; 77:393-8.

**Table 1 (abstract P239).**
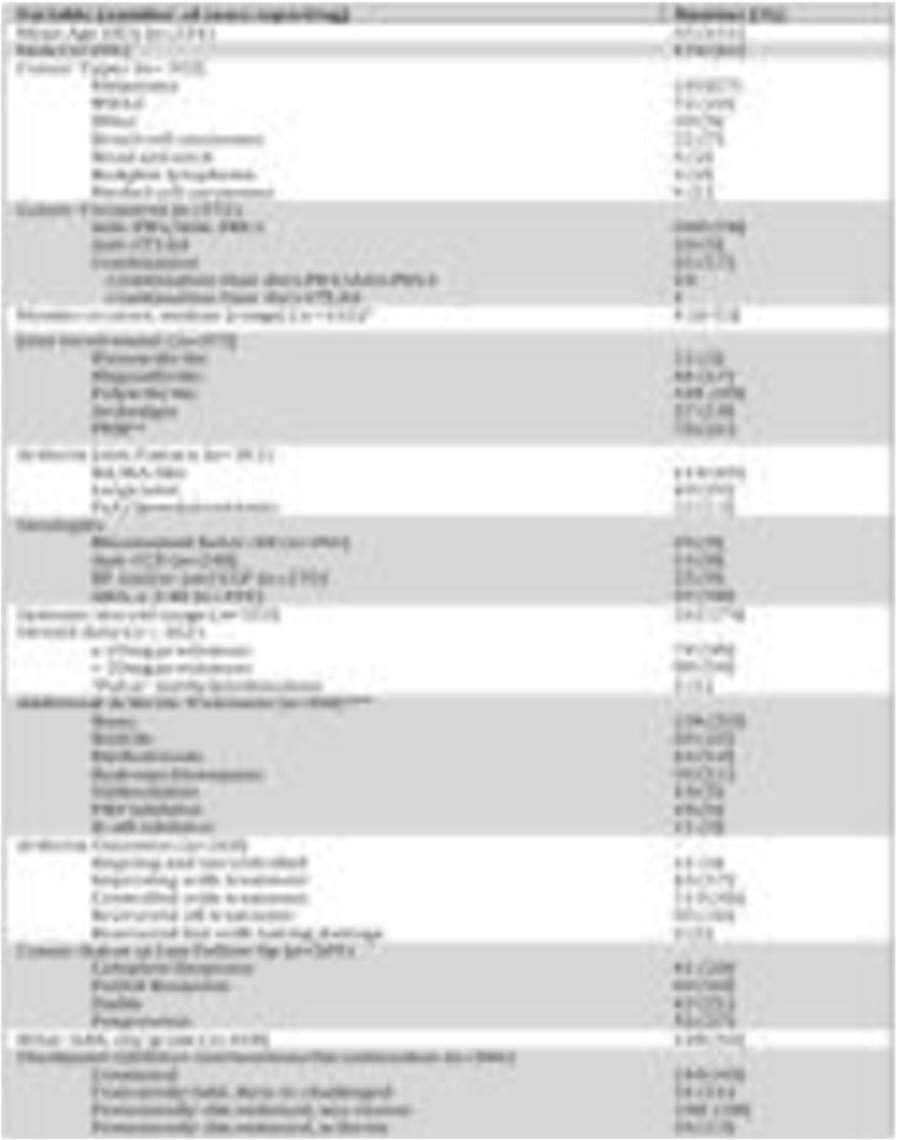
ICI-arthritis patient characteristics (total n=372)

#### P240 Quantitative cell-based bioassays to advance immunotherapy programs targeting immune checkpoint receptors

##### Vanessa Ott, PhD, Jamison Grailer, PhD, Jun Wang, Julia Gilden, PhD, Pete Stecha, Denise Garvin, Michael Beck, Jim Hartnett, Frank Fan, PhD, Mei Cong, PhD, Zhi-jie Jey Cheng

###### Promega, Madison, WI, United States

####### **Correspondence:** Zhi-jie Jey Cheng (jey.cheng@promega.com)

**Background**

The human immune system is comprised of a complex network of immune checkpoint receptors that are promising new immunotherapy targets for the treatment of a variety of cancers and autoimmune-mediated disorders. Immunotherapies designed to block co-inhibitory receptors (e.g. PD-1, CTLA-4) are showing unprecedented efficacy in the treatment of cancer. However, not all patients and tumor types respond to this approach. This has resulted in broadening of immunotherapy research programs to target additional co-inhibitory (e.g. LAG-3, TIM-3) and co-stimulatory (e.g. 4-1BB, GITR, OX40, ICOS) receptors individually and in combination.

**Methods**

A major challenge in the development of biologics is access to quantitative and reproducible functional bioassays. Existing methods rely on primary cells and measurement of complex functional endpoints. These assays are cumbersome, highly variable and fail to yield data quality required for drug development in a quality-controlled environment. To address this need, we have developed a suite of cell-based functional bioassays to interrogate modulation of immune checkpoint receptors individually (e.g. PD-1, CTLA-4, LAG-3, TIM-3, GITR, 4-1BB) and in combination (e.g. PD-1+CTLA-4, PD-1+LAG-3). These assays consist of stable cell lines that express luciferase reporters driven by response elements under the precise control of mechanistically relevant intracellular signals.

**Results**

The bioassays reflect mechanisms of action for the drug candidates designed for each immune checkpoint receptor and demonstrate high specificity, sensitivity and reproducibility. For example, using the PD-1 blockade bioassay, TCR-mediated luciferase activity is recovered with anti-PD-1 and PD-L1 blocking Abs but not with unrelated control antibodies. Similarly, TCR and CD28-mediated luciferase activity is recovered in the CTLA-4 blockade bioassay with an anti-CTLA-4 blocking antibody (ipilimumab), but not with unrelated control antibodies. In a PD-1+CTLA-4 combination bioassay, anti-PD-1 (nivolumab) and anti-CTLA-4 (ipilimumab) blocking antibodies individually induced luciferase activity (3.0- and 5.6-fold, respectively), the combination of both antibodies resulted in a synergistic 18-fold increase in luciferase activity. Similar antibody-induced luciferase activity is observed in a panel of bioassays specific for agonist antibodies, including GITR, 4-1BB, OX40 and CD40. This response can be enhanced following Fc receptor cross-linking, reflecting in vivo activity.

**Conclusions**

Cell-based reporter bioassays overcome the limitations of primary cell-based assays for functional characterization of antibody and other biologics drugs targeting individual or combination immune checkpoint receptors. Here we show a portfolio of mechanism of action-based bioassays for co-inhibitory and co-stimulatory immune checkpoint receptors that can be used for antibody screening, characterization, potency and stability studies.

#### P241 Single-cell deconvolution identifies T-cell correlates of response to PD-1 blockade treatment in AML

##### Xingyue An, BS^1^, Jay R Adolacion^1^, Mansour Alfayez^2^, Jairo Matthews^2^, Wilmer Flores^2^, Steven Kornblau, MD^2^, Arash Saeedi^1^, Sreyashi Basu^2^, Naval Daver, MD^2^, Navin Varadarajan, PhD^1^

###### ^1^University of Houston, Houston, TX, United States; ^2^Univ. of Texas MD Anderson Cancer Center, Houston, TX, United States

####### **Correspondence:** Navin Varadarajan (nvaradarajan@uh.edu)

**Background**

The combination of the αPD-1 (nivolumab) and hypomethylating agent azacytidine demonstrated encouraging response in R/R acute myeloid leukemia (AML) patients, but the percentage of patients who achieved IWG 2016 responses was limited[1]. Early predictive biomarkers to facilitate future trials patient selection are desirable. A better understanding of T cells in AML pre-therapy and on-therapy should yield valuable insights on the treatment-induced anti-tumor response.

**Methods**

We performed RNA-sequencing on T cells from a cohort of AML patients who were treated with azacytidine and nivolumab (Table 1). By leveraging subset definitions based on single-cell RNA-sequencing results from T cells of cancer patients, we implemented deconvolution of our bulk T-cell RNA-sequencing data to obtain the relative abundance of different T-cell subsets (in-silico dissection).

**Results**

For validation purpose, we compared the gene expression of peripheral blood (PB) T cells from AML patients and healthy donors (HD)[2,3]. The deconvolution results were consistent with previously published flow-cytometry data profiling cancer patients[4,5] (Figure 1). Compared with HD T cell, circulating AML CD4 T cells consisted of a higher frequency of Treg[4]. PB CD8 T cells from AML patients were with a significantly lower frequency of naïve, and higher frequencies of effector and exhausted phenotypes[5].

Independent of the clinical responses, comparison of the pre-treatment CD8 T cells from bone marrow (BM) and PB from all AML patients using both gene set enrichment analysis and deconvolution indicated that the BM CD8 T cells were more activated/differentiated compared with PB CD8 T cells, likely reflective of an ongoing immune response against the AML. We also found treatment-induced gene expression changes in the AML circulating CD8 T cells, characterized by increased cell metabolism and cell proliferation. Deconvolution identified that pre-therapy relative abundance of exhausted (CD3+CD8+PD-1+CD45RO+) and effector (CD3+CD8+CD45RA+Tbet+PD-1lo) CD8 T cells (plasticity) could serve as subpopulations relevant for patient stratification (Figure 2). We further validated these results using CyTOF wherein these same subpopulations were differentially abundant between responders and non-responders at the pre-therapy time-point.

**Conclusions**

Collectively, our results revealed that (1) PD-1 blockade-based treatment-induced gene expression profiling changes (increase cell proliferation, metabolism and activation) of CD8 T cells are detectable as early as EOC1 in the PB; (2) specific subpopulations of plastic CD8 T cells identified have the potential to serve as an actionable biomarker to select AML patients most likely to benefit from such immune checkpoint therapies. These findings need to be confirmed in larger studies with αPD-1 based therapies in AML.

**Acknowledgements**

NIH (R01CA174385), CPRIT (RP180466), MRA Established Investigator Award to NV (509800), Welch Foundation (E1774), NSF (1705464), CDMRP (CA160591), and Owens foundation. We would like to acknowledge the MDACC Flow Cytometry and Cellular Imaging Core facility for the FACS sorting (NCI P30CA16672), UH Seq-N-Edit core for RNA-sequencing service, and Intel for the loan of computing cluster.

**Trial Registration**

NCT02397720

**References**

1. Daver, N. et al. Efficacy, Safety, and Biomarkers of Response to Azacitidine and Nivolumab in Relapsed/Refractory Acute Myeloid Leukemia: A Nonrandomized, Open-Label, Phase II Study. Cancer Discov. 2019; 9: 370–383.

2. Corces, M. R. et al. Lineage-specific and single-cell chromatin accessibility charts human hematopoiesis and leukemia evolution. Nat. Genet. 2016; 48: 1193–1203.

3. Linsley, P. S., Speake, C., Whalen, E. & Chaussabel, D. Copy number loss of the interferon gene cluster in melanomas is linked to reduced T cell infiltrate and poor patient prognosis. PLoS One. 2014; 9.

4. Szczepanski, M. J. et al. Increased frequency and suppression by regulatory T cells in patients with acute myelogenous leukemia. Clin. Cancer Res. 2009; 15: 3325–3332.[5. Knaus, H. A. et al. Signatures of CD8+ T cell dysfunction in AML patients and their reversibility with response to chemotherapy. JCI Insight 2018; 3.

**Ethics Approval**

All patients signed an informed consent form approved by the Institutional Review Board (IRB) from The University of Texas MD Anderson Cancer Center. The study was conducted in accordance with the Declaration of Helsinki. The study was approved by the IRB from University of Houston.

**Table 1 (abstract P241).**
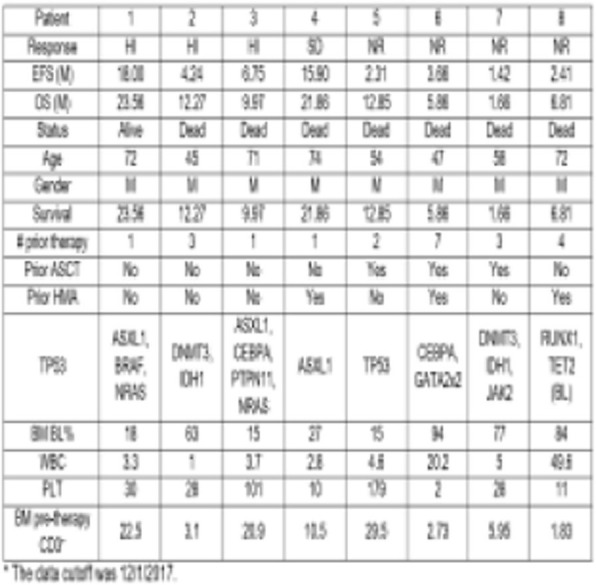
See text for description

**Fig. 1 (abstract P214).**
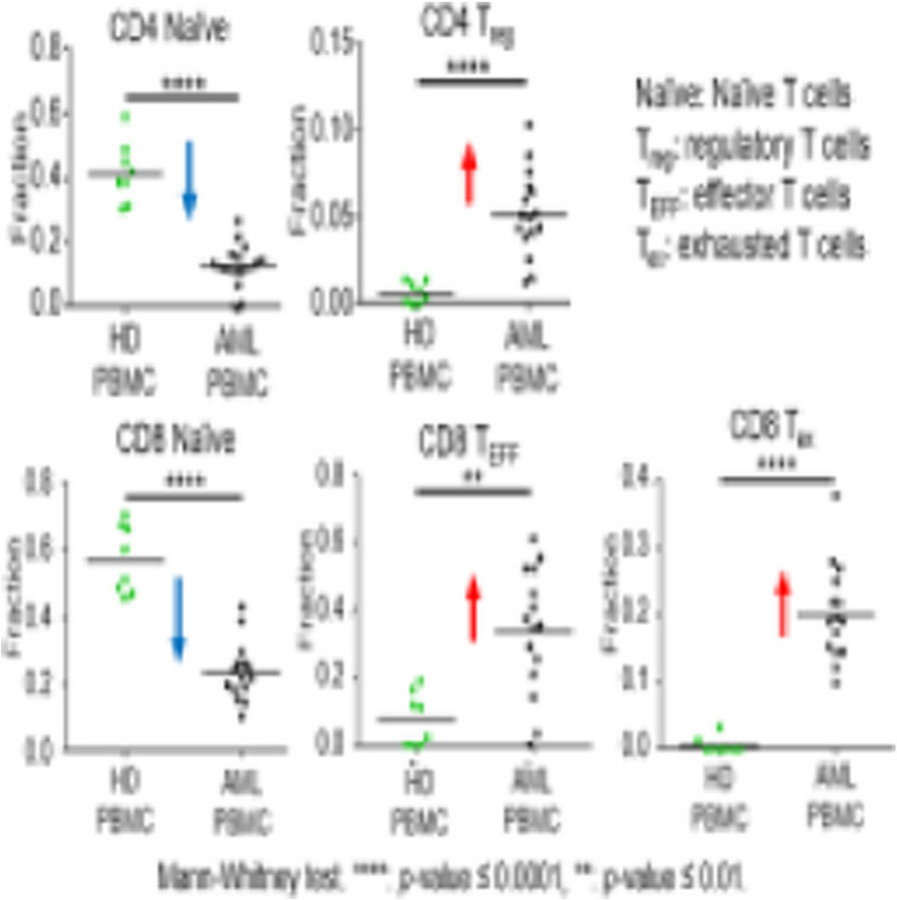
See text for description

**Fig. 2 (abstract P214).**
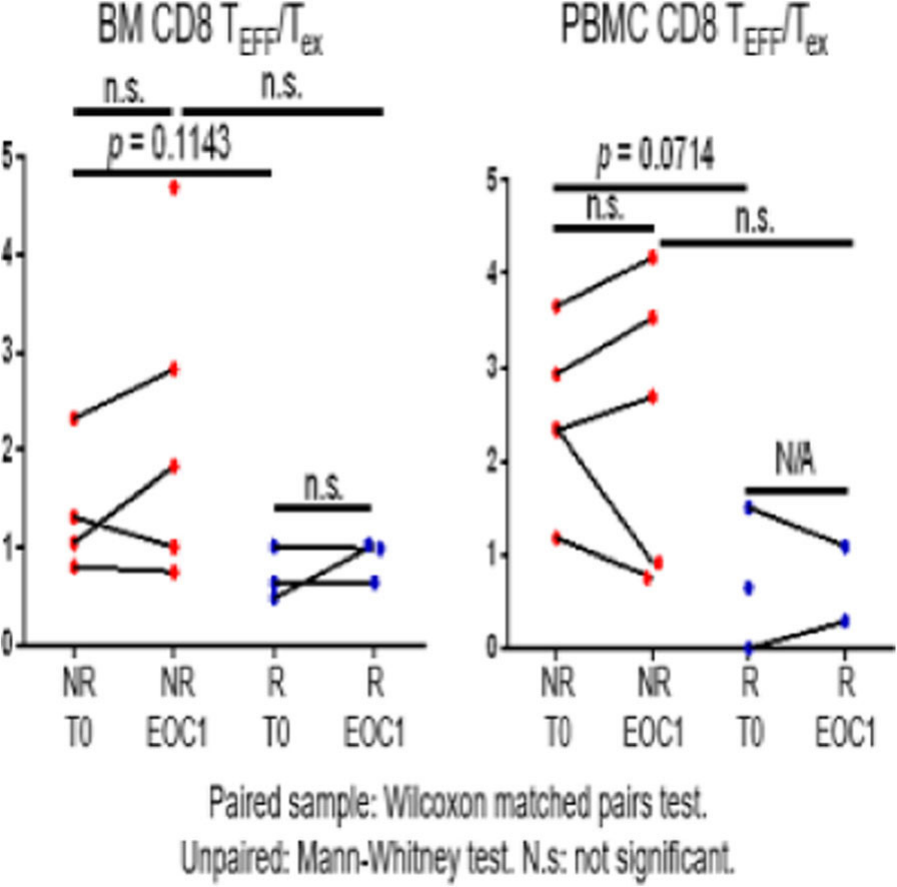
See text for description

#### P242 Combination of the angiogenesis inhibitor lucitanib with immune checkpoint blockade augments anti-tumor activity in syngeneic models

##### Rachel Dusek, PhD, Liliane Robillard, PhD, Thomas Harding, PhD, Andrew Simmons, PhD, Minh Nguyen

###### Clovis Oncology, Inc., San Francisco, CA, United States

####### **Correspondence:** Rachel Dusek (rdusek@clovisoncology.com)

**Background**

Lucitanib is an anti-angiogenic, small molecule multi-tyrosine kinase inhibitor that has demonstrated potent tumor growth inhibition in multiple cancer xenograft models. Because angiogenic factors contribute to immune suppression in the tumor microenvironment, inhibiting angiogenic pathways may normalize the tumor vasculature and relieve immunosuppression to augment antitumor immunity, especially with concurrent immune checkpoint inhibition. Therefore, we characterized lucitanib’s selectivity and investigated the antitumor efficacy and mechanisms of action of lucitanib combined with anti-PD-1 in nonclinical models.

**Methods**

The kinase inhibition profile of lucitanib was evaluated using functional biochemical assays. Kinase phosphorylation in cancer cells or mouse tissues was assessed by western blot. In vivo efficacy studies were conducted in syngeneic mouse models with monotherapy or combinations of lucitanib (10 mg/kg daily), DC101 (mouse VEGFR2 monoclonal antibody; 40 mg/kg every 2 days), or anti-PD-1 (5−10 mg/kg biweekly). Treated tumors were analyzed for gene and protein expression and immune composition.

**Results**

In vitro, lucitanib demonstrated selective and potent inhibition of the tyrosine kinases VEGFR1-3, PDGFRalpha/beta, FGFR1-3, CSF1R, DDR1, and RET. Lucitanib caused dose-dependent inhibition of VEGFR2 phosphorylation in vivo; one 10 mg/kg dose sustained inhibition for 12 hours. Compared with DC101, lucitanib significantly enhanced tumor growth inhibition and survival in MC38 colon tumor-bearing mice. Lucitanib combined with anti-PD-1 significantly increased antitumor activity relative to single agents and to DC101 plus anti-PD-1. Lucitanib-treated MC38 tumors exhibited gene expression changes beyond those observed with DC101 treatment. Across multiple syngeneic mouse models, tumor growth was significantly inhibited 81%−98% by lucitanib combined with anti-PD-1 and 57%−88% by lucitanib alone. The combination significantly extended survival by 90% to >186% and 15% to >35% compared with vehicle or the best monotherapy, respectively. Lucitanib combined with anti-PD-1 increased gene expression associated with T-cells, cytotoxic cells, and T-cell signaling in BR5FVB1-Akt ovarian tumors, relative to each monotherapy. However, lucitanib alone appeared sufficient to modulate innate and adaptive immunity-related gene expression in MC38 tumors. Additional tumor profiling and mechanism of action studies are ongoing.

**Conclusions**

Lucitanib, a potent and selective angiogenesis inhibitor, is differentiated from DC101 and displays enhanced antitumor activity in combination with PD-1 inhibition in multiple syngeneic models. Gene expression changes associated with tumor immune infiltration and increased antitumor immunity were observed in the combination-treated tumors and may contribute to the increased antitumor activity. Results from these studies support the clinical development of the combination of lucitanib and immune checkpoint blockade as a potential treatment for patients with solid tumors.

**Ethics Approval**

The studies were conducted in accordance with the Shanghai Medicilon Inc.Guidelines for Use and Care of Animals or an approved IACUC protocol at Crown Biosciences.

#### P243 Modulating the immunogenicity of low-mutation soft tissues sarcomas with epigenetic targeted therapy

##### Himavanth Gatla, PhD, Maggie Phillips, Brian Ladle

###### Johns Hopkins Medicine, Owings Mills, MD, United States

####### **Correspondence:** Brian Ladle (bladle@jhmi.edu)

**Background**

Sarcomas account for 13% of all cancers in young adults under the age of 20. For recurrent and metastatic sarcomas, the very poor survival rate despite surgery and chemotherapy warrants better systemic therapies. As opposed to cancers that show good responses to immune checkpoint blockade which have high mutation burden, pediatric sarcomas present with low mutation burden, and a complete ineffectiveness of immune checkpoint blockade as a monotherapy, suggesting poor immune system activation, and immune infiltration.

**Methods**

We have used a mutated Kras-driven murine sarcoma syngeneic tumor model (KP Sarc) and GM-CSF-secreting whole cell tumor cell vaccine approach (GVAX) to investigate if epigenetic targeted therapy induced tumor associated antigens are capable of generating sustained anti-tumor immunity

**Results**

We show that sequential combination of epigenetic modifying drugs - DNA methyl transferase inhibitor (decitabine) and HDAC inhibitor (entinostat) significantly increases the expression of cancer testis antigens (CTAs), compared to either drugs alone. In addition, these drugs modify chemokine expression including increased expression of CXCL10. Significantly improved immune responses can be generated to decitabine and entinostat pre-treated KP sarc tumor cells as assessed by slowed tumor growth, increased T cell infiltrates, and increased cytokine production. Furthermore, immune checkpoint blockade therapy potentiates the tumor regression mediated by the combination of decitabine and entinostat more effectively than the regression mediated by either drugs alone. This suggests that combination therapy induced antigenicity generates better anti-tumor immune responses. Rechallenging the mice which rejected epigenetically modified KP Sarc tumor formation with similarly treated KP Sarc cells did not result in tumor formation, whereas untreated KP Sarc cells grew uninhibited, suggesting that epigenetic therapy induced tumor associated antigens are capable of generating a sustained memory immune response. By depleting CD4 and CD8 T lymphocytes, we show that epigenetic targeted therapy induced anti-tumor responses are mediated by both CD4 and CD8 T lymphocytes. Difficulty with in vivo treatment includes the myelosuppressive side effects of decitabine and entinostat which can inhibit T cell responses immediately after treatment. Proper sequencing of the drugs when given in vivo will be crucial to generate successful adaptive T cell responses to newly expressed antigens.

**Conclusions**

Epigenetic targeted therapy induced tumor associated antigens are capable of generating sustained anti-tumor immune responses.

#### P244 The genomic architecture of serous carcinomas shapes the tumor microenvironment and modulates responses to targeted and immunotherapies

##### Sonia Iyer, PhD^1^, Shuang Zhang^2^, Anniina Farkkila^3^, Sean Smith^4^, David Pepin^5^, Raghav Mohan^5^, Tian Xia^1^, Ferenc Reinhardt^1^, Tony Chavarria^1^, Esmee Hoefsmit^1^, Shailja Pathania^6^, Yunlan Zhou^3^, Kevin Elias^7^, Benjamin Neel^8^, Robert Weinberg, PhD^1^

###### ^1^Whitehead Institute for Biomedical Research, Cambridge, MA, United States; ^2^NYU Langone Health, New York, NY, United States; ^3^Dana-Farber Cancer Institute, Boston, MA, United States; ^4^Massachusetts Institute of Technology, Cambridge, MA, United States; ^5^Massachusetts General Hospital, Boston, MA, United States; ^6^University of Massachusetts, Boston, MA, United States; ^7^Brigham and Women's Hospital, Boston, MA, United States; ^8^NYU-Langone Medical Center, New York, NY, United States

####### **Correspondence:** Sonia Iyer (iyers@wi.mit.edu); Robert Weinberg (weinberg@wi.mit.edu)

**Background**

High-grade serous ovarian cancer (HGSOC) is the most frequent and most aggressive histologic subtype of ovarian cancer. The cornerstone of the existing treatment of HGSOC is DNA-damaging chemotherapy; however, practically all patients eventually develop the progressive disease and the 5-year survival is only 40%. Immunotherapy would seem to be an attractive alternative treatment to chemotherapy, yet existing immunotherapies perform poorly in ovarian cancer, with only ~10% of patients responding to checkpoint blockade. Why this is the case remains poorly understood and there is a pressing need to understand the underlying biology of immune evasion in ovarian cancer. One critical area of interest is the role of homology dependent DNA repair (HR) in immune evasion. Unfortunately, the preclinical tools required to explore the relationship between the types of DNA damage repair deficiencies and immune evasion have been lacking. Hence, we have modeled the biology of ovarian cancer using patient-relevant mutational landscapes in an immune-proficient, syngeneic mouse model in order to help us identify the contribution of common driver mutations to the immune repertoire in the tumor microenvironment, and thus to responses of HGSOC tumors to immunotherapy.

**Methods**

We hypothesize that the immune composition and gene expression signatures of the resulting tumors will vary based on the combination of genetic alterations and the DNA repair proficiency of the transformed cells. To this end, we have engineered novel syngeneic mouse models from murine-fallopian-tube epithelium using CRISPR/Cas9 technology. These tumors capture the most common combinations of co-occurring mutations observed in HR-deficient and -proficient patient samples.

**Results**

To validate the DNA repair proficiency of the transformed cells, we measured Rad51 nuclear focus formation after ionizing radiation (IR) and PARP inhibitor and DNA-damaging agent sensitivity. The HR-deficient cell lines had significantly fewer Rad51 nuclear foci and were more sensitive to PARP inhibition in comparison to HR-proficient cells. Initial immune /stromal analysis using flow cytometry, scRNA seq transcriptomic and immunofluorescence analysis revealed substantial differences in the myeloid and T-cell regulatory compartments between HR-proficient and -deficient primary and metastatic tumors and within the ascitic fluid. Preliminary results also suggest that inhibition of the DNA damage response (DDR), checkpoint kinase 1 (Chk1) in combination with immune checkpoint inhibitors, potentiates antitumor effects and augments cytotoxic T-cell infiltration.

**Conclusions**

Understanding the genetic basis of these complex cellular interactions will be critical to better tailor combinations of existing targeted treatments and immunotherapies in ovarian cancer to fight this devastating disease.

#### P245 Durvalumab after concurrent chemoradiotherapy in inoperable stage III non-small cell lung cancer (NSCLC) – a German radiation oncology survey

##### Lukas Kaesmann, MD, Chukwuka Eze, Julian Taugner, Olarn Roengvoraphoj, Claus Belka, Farkhad Manapov

###### University Hospital, Munich, Germany

####### **Correspondence:** Lukas Kaesmann (lkaesmann@gmail.com)

**Background**

Consolidation PD-L1 inhibition with durvalumab after platin-based concurrent chemoradiotherapy (CRT) has become the standard of care in inoperable stage III non-small cell lung cancer (NSCLC) based on the excellent PACIFIC trial results. Treatment recommendations need time for implementation in nationwide settings and require the close interaction of different medical specialities. In this nationwide survey, we questioned the distribution and clinical settings of durvalumab treatment after concurrent CRT, observed side effects of this treatment and summarize follow-up management.

**Methods**

We surveyed radiation oncology institutions in Germany via an anonymous online questionnaire sent by e-mail to all members of the German Radiation Oncology Society.

**Results**

We received a total of 255 responses (response rate: 18%). Of which 203 (80%) were completed and returned and thus eligible for further evaluation. The respondents work in 87 different cities and 44% in a private medical practice, 29% in university and 22% in a general hospital. Responses of the same department were analysed for congruence. Durvalumab was implemented in clinical routine by 143 (70%) respondents. Reasons for failed implementation in clinical practice were patient ineligibility, decision of medical oncologists or absence of **updated** German evidence review (S3-guidelines) regarding this treatment approach. **Durvalumab was generally administered according to the respondents by private oncological practices in 32%, general or university hospital in 57% and in the radiation oncology department, which delivered the CRT in 11% of cases.** Importantly, according to **36%** of all respondents initial PD-L1 status was present in ≤30% of all patients. 82% of respondents have treated 1-15 patients with durvalumab and 14% of respondents >15 patients. Furthermore, no respondent had applied durvalumab in less than 14 days after the completion of CRT. 65 (**46%**) and 49 (34%) respondents started durvalumab 14-28 days and later than 28 days after CRT, respectively. The majority of respondents (>80%) re-staged the patients with CT (thorax/upper abdomen) prior to durvalumab. Severe side effects requiring hospital admission in more than 10% of all patients were reported by only 12% of all respondents.

**Conclusions**

Durvalumab was implemented in the multimodal treatment of inoperable stage III NSCLC and administered by the absolute majority of respondents. Low testing rates of PD-L1 at initial diagnosis were observed and should be considered a major barrier to universal adoption and integration in the clinical work-flow. Durvalumab appears to be well tolerated. However, treatment-related side effects need to be considered during and after multimodal therapy.

**Acknowledgements**

We would like to thank the Board of the German Society for Radiation Oncology (DEGRO) for their approval and their office team for providing the mailing list.

**Ethics Approval**

The Board of the German Society for Radiation Oncology (DEGRO) approved the survey.

#### P246 Prospective evaluation of outcome and toxicity of durvalumab treatment after chemoradiotherapy in inoperable stage III non-small cell lung cancer (NSCLC) patients

##### Lukas Kaesmann, MD, Julian Taugner, Chukwuka Eze, Olarn Roengvoraphoj, Claus Belka, Farkhad Manapov

###### University hospital, Munich, Germany

####### **Correspondence:** Lukas Kaesmann (lkaesmann@gmail.com)

**Background**

Consolidation PD-L1 inhibition with durvalumab after concurrent chemoradiotherapy (CRT) has become the standard of care in inoperable stage III non-small cell lung cancer (NSCLC) based on the excellent PACIFIC trial results. The aim of this prospective single center study was to evaluate the outcome and toxicity of durvalumab treatment after CRT in a tertiary cancer center.

**Methods**

We prospectively collected clinical characteristics, toxicity and outcome of all patients with inoperable stage III NSCLC treated with durvalumab after CRT/RT since 9th November 2018. Toxicity was collected using the Common Terminology Criteria for Adverse Events version 5 before and during treatment. Re-staging after CRT and before the start of durvalumab consisted of a CT scan (thorax/upper abdomen). 18F-FDG-PET-CT was performed 3 months and CT 6 months after start of consolidation treatment.

**Results**

Data of 16 patients treated with durvalumab after CRT/RT were evaluated. Three patients (19%) were female and 13 (68%) male, median age at treatment start was 64 years. 10 (53%) patients had T4 or T3 tumors, four (25%) patients had N3 and 9 (56%) N2 disease. 15 Patients had CRT with a medium radiation dose of 63.20 Gy and were treated with two concurrent cycles of platin-based chemotherapy. One patient was treated with moderate hypofractionated radiotherapy without chemotherapy. Median follow-up was 7 (range:2-16) months. All patients were alive at the time of evaluation. Four (25%) patients have developed oligoprogression. Metastastic sites were bone, brain, adrenal gland and distant lymph nodes. Two patients received second-line chemotherapy after distant failure. Another two received stereotactic body radiotherapy for all metastatic sites and continued on durvalumab. Common toxicity during durvalumab was dermatitis (I-II° CTCAE) which occurred earliest after 2 cycles in 10 (65%) patients and pneumonitis II° CTCAE in 2 (13%) and III° CTCAE in 2 (13%) patient between 2-7 months after completion of CRT. In total, 3 (19%) patients discontinued durvalumab treatment after a median of 4 months due to distant progression or unacceptable toxicity.

**Conclusions**

Durvalumab was well tolerated with reversible acute toxicity. 25% of patients develop oligoprogression after a mean time of 5.5 months after the end of CRT.

**Ethics Approval**

The study was approved by the University Ethics Board, approval number 17-230.

**Consent**

Written informed consent was obtained from the patient for publication of this abstract and any accompanying images. A copy of the written consent is available for review by the Editor of this journal.

#### P247 TLR3-targeting combinatorial chemokine modulation sensitizes “Cold” tumors for the therapeutic effectiveness of immune checkpoint inhibition

##### Kathleen Kokolus, MS, PhD^1^, Natasa Obermajer, PhD^2^, Per Basse, MD, PhD^1^, Pawel Kalinski, MD, PhD^1^

###### ^1^Roswell Park Comprehensive Cancer Center, Buffalo, NY, United States; ^2^UPMC Hillman Cancer Center, Pittsburgh, PA, United States

####### **Correspondence:** Pawel Kalinski (Pawel.Kalinski@roswellpark.org)

**Background**

Immune checkpoint inhibition (ICI) has emerged as life-prolonging and occasionally curative treatment for many cancer patients, but their activity remains disappointing in many common tumors. ICI therapies are effective against “hot” tumors infiltrated with cytotoxic T lymphocytes (CTLs) but inefficient against “cold” tumors lacking CTLs. The importance of CTLs, availability of CTL targets and local expression of PD-L1 and PD-L2 (induced by CTL-produced IFNγ) in the overall effectiveness of ICI remain controversial. We have observed that chemokine-modulatory (CKM) regimens combining TLR3 ligands with type-1 IFNs are up to 100-fold more effectiveness in inducing CTL attractants (CXCL9, CXCL10, CCL5) compared to either factor alone. Moreover, CKM suppresses local Treg attractants, and targets tumor microenvironment (TME) rather than healthy tissues [1-3]. Thus, we tested whether local or systemic CKM treatments enhance CTL infiltration in “cold” tumors and determined the feasibility of short-term CKM to sensitize poorly immunogenic, αPD-1 resistant, tumors to PD-1 blockade.

**Methods**

C57BL/6 mice inoculated with MC38 (colorectal) or ID8 (ovarian) cancer cells were treated starting on day three (low-stage disease) or eight (late-stage disease). A two dose course of CKM (IFNα and rintatolimod [2]) followed by three doses of αPD-1 in two distinct regimens: 1) Sequentially (following CKM) or 2) Concurrent with CKM. Mice were monitored for intratumoral CTL, tumor growth and survival.

**Results**

We observed strong effectiveness of CKM in promoting intratumoral increases in CTL and PD-L1 expression in the TME. CKM aids in the sensitization of the largely αPD1-resistant tumors to PD1 blockade. Both sequential and concurrent CKM allowed antitumor effectiveness of αPD-1, resulting in overall prolongation of survival and 30-100% cures, depending on treatment initiation. Sensitizing tumors to αPD-1 did not require intratumoral CKM administration and was observed with systemic application at distant sites, consistent with the preferential activation of tumor tissues by CKM observed in tumor explant model [3]. Although strong antitumor effects were seen in the absence of any vaccination component, a stronger effect could be observed by vaccination with tumor-loaded dendritic cells.

**Conclusions**

We demonstrate that local or systemic CKM sensitizes mice with poorly immunogenic tumors for subsequent effectiveness of PD-1 blockade. Thus, promoting intratumoral CTL accumulation may be sufficient for therapeutic effectiveness of ICI against “cold” tumors with low mutational load. Our data provides rationale for clinical testing of sequential regimens where short-term CKM is followed by routine ICI, limiting the inconvenience for patients and facilitating the inclusion of CKM into routine immunotherapy plans.

**Acknowledgements**

Funded by 1P01CA132714, Rustum Family Foundation and Institutional Support.

**References**

1. Muthuswamy R, Berk E, Junecko BF et al. Cancer Res. 2012; 72:3735-3743.

2. Theodoraki MN, Yernei S, Sarkar et al. Cancer Res. 2018; 78:4292-4302.

3. Obermajer H, Urban J, Wieckowski E et al. Nat Protoc. 2018; 13:335-357.

**Ethics Approval**

This study was approved by Roswell Park Comprehensive Cancer Center's IACUC (protocol 1398M).

#### P248 Pembrolizumab plus axitinib (P+A) versus other first-line (1L) systemic therapies for advanced/metastatic clear-cell renal cell carcinoma (ccmRCC) by IMDC Risk Status – a network meta-analysis (NMA)

##### Ian McGovern, MPH^1^, Rohan Shirali, MS^1^, Andrew Simon, ScM^1^, Yichen Zhong, PhD^2^, Rodolfo Perini, MD^2^, Maria Lorenzi, MSc^1^, Oluwakayode Adejoro, MD, MPH^2^

###### ^1^Precision Xtract, Boston, MA, United States; ^2^Merck & Co. Inc., Kenilworth, NJ, United States

####### **Correspondence:** Oluwakayode Adejoro (oluwakayode.adejoro@merck.com)

**Background**

The International Metastatic Renal Cell Carcinoma Database Consortium (IMDC) risk group classification is an important prognostic factor for efficacy outcomes of first-line systemic treatment of advanced/metastatic mRCC. IMDC risk is predictive of outcomes including overall survival (OS), progression-free survival (PFS) and overall response rate (ORR). Pembrolizumab in combination with axitinib showed superior and clinically meaningful improvements in OS, PFS and ORR versus sunitinib in subjects with untreated ccmRCC in the KEYNOTE-426 trial. This NMA synthesized evidence from randomized clinical trials (RCTs) to indirectly compare the relative treatment effects of P+A vs other therapies in subjects with favorable and intermediate + poor IMDC risk groups.

**Methods**

Fixed-effect Bayesian NMA was conducted to determine the relative efficacy of treatments. Hazard ratios (HRs) for PFS and OS were estimated with 95% credible intervals (CrIs). Analyses were conducted among subjects with favorable risk, and intermediate + poor risk disease.

**Results**

Among subjects with favorable IMDC risk, the estimated HRs for OS favored P+A over the other 3 interventions evaluated [nivolumab+ipilimumab (N+I) (HR=0.53, 95% CrI: 0.18-1.60), sunitinib (HR=0.64, 95% CrI: 0.24-1.70) and pazopanib (HR=0.73, 95% CrI: 0.26-2.03)] but none were statistically significant. For PFS, P+A had statistically significant benefit over 2 out of 9 interventions evaluated [interferon-alpha (IFN) (HR=0.30, 95% CrI 0.15-0.60) and bevacizumab (B)+ temsirolimus (HR=0.41, 95% CrI 0.18-0.98)]. The results numerically favored P+A over 5 out of 9 interventions evaluated [ranging from B+IFN (HR=0.50, 95% CrI 0.23-1.10) through, atezolizumab, pazopanib, N+I, to sunitinib (HR=0.81, 95% CrI: 0.53-1.23)], but were not statistically significant. PFS benefits favored B+atezolizumab (HR=1.07, 95% CrI: 0.42-2.73) and avelumab+axitinib (HR=1.49, 95% CrI: 0.76-2.96) over P+A but were not statistically significant.

In the intermediate + poor IMDC risk group, P+A showed a significant OS benefit over sunitinib (HR=0.52, 95% CrI: 0.37-0.74) and was favored over the other two interventions evaluated, but not statistically significant [cabozantinib (HR=0.65, 95% CrI: 0.38-1.11) and N+I (HR=0.79, 95% CrI: 0.53-1.18)]. P+A showed a significant PFS benefit over sunitinib (HR=0.67, 95% CrI: 0.53-0.85) and was favored over N+I (HR=0.87, 95% CrI: 0.65-1.17), but not statistically significant. PFS benefit favored cabozantinib (HR=1.40, 95% CrI: 0.85-2.31) over P+A, but was not statistically significant.

**Conclusions**

The results of this analysis suggest that pembrolizumab+axitinib may have PFS and OS advantages over most alternative first-line treatment options for mRCC, irrespective of IMDC risk groups.

#### P249 Time-dependent blood transcriptomic perturbations differentially associated with mono and combination checkpoint inhibitor therapy

##### Darawan Rinchai, PhD^1^, Emily Hinchcliff, MD^2^, Wouter Hendrickx, PhD^1^, Jessica Roelands, Master^1^, Damien Chaussabel^1^, Davide Bedognetti, MD, PhD^1^, Amir Jazaeri, MD^2^

###### ^1^Sidra Medicine, Doha, Qatar; ^2^The University of Texas MD Anderson Cancer Center, Houston, TX, United States

####### **Correspondence:** Amir Jazaeri (aajazaeri@mdanderson.org)

**Background**

The rationale for combination immunotherapy is based on presumed additive or synergistic properties of each individual drug [1-2]. Understanding the molecular mechanisms modulated by a given drug is critical to implement more efficient therapeutic approaches. We conducted a study to determine 1) whether response to anti- anti-CTLA4 monotherapy (Tremelimumab) or combination of anti-PDL1(Durvalumab) and anti-CTLA4 (Tremelimumab) therapy could be measured via blood transcriptome profiling; and 2) whether differences between both treatment groups could be observed. In blood transcriptomic correlative studies performed so far, samples have been collected at limited time points (i.e., before and after treatments), preventing the description of the kinetic and dynamic changes associated with specific treatment modalities [3].

**Methods**

An adaptively randomized phase II trial of sequential versus combination administration of Tremelimumab and Durvalumab (MEDI4736) in patients with recurrent platinum resistant ovarian, peritoneal or fallopian tube cancers at the M.D. Anderson Cancer Center. Peripheral blood samples were collected serially before treatment and at 6 time points post-treatment from patients receiving Tremelimumab, alone or in combination with Durvalumab, administered every 28 days. A total of 91 samples were analyzed. Time points include C1D01 (baseline), C1H12 (12 hours after treatment), C1D08 (cycle one day 8), C1D15 (cycle one day 15), C2D01 (cycle 2 day one) and C3D01 (cycle 3 day one). Blood transcriptome profiles were generated by RNA-seq (Illumina HiSeq4000) at Sidra medicine. A set of 382 transcriptional modules was used for the analysis of this dataset using a pre-defined framework [4-5]. A module is considered to be “responsive” to the treatment when significant changes in abundance are observed for a proportion of its constitutive transcripts that is greater that what could be expected by chance [4-5].

**Results**

We identified changes in blood transcript abundance in both treatment groups, the modular perturbation peaking at cycle 1 day 15 post-treatment (Figure 1). Perturbations of Cell cycle, Protein synthesis and Gene transcription modules were observed in both groups. But important qualitative differences were observed as well. Most notably, abundance of gene sets associated with Interferon, Tumor necrotic factor, cytotoxic lymphocyte and erythroid signatures was specifically increased in patients receiving combination immunotherapy

**Conclusions**

We characterized differential, time-dependent, systemic perturbations associated with mono vs combination immune checkpoint blockade. The peak of this immune modulation is observed at day 15 after treatment. The mechanistic and clinical relevance of these findings remains to be explored in a larger group of patients

**Trial Registration**

NCT03026062

**References**

1. Naumann RW, Coleman RL. Management strategies for recurrent platinum-resistant ovarian cancer. Drugs 2011; 71: 1397-412.

2. Davis A, Tinker AV, Friedlander M. "Platinum resistant" ovarian cancer: what is it, who to treat and how to measure benefit? Gynecologic oncology 2014; 133: 624-31.

3. Friedlander P, Wassmann K, Christenfeld AM, Fisher D, Kyi C, Kirkwood JM, Bhardwaj N, Oh WK. Whole-blood RNA transcript-based models can predict clinical response in two large independent clinical studies of patients with advanced melanoma treated with the checkpoint inhibitor, tremelimumab. J Immunother Cancer 2017 Aug 15;5(1):67. doi: 10.1186/s40425-017-0272-z.

4. Chaussabel D, Baldwin N. Democratizing Systems Immunology with Modular Transcriptional Repertoires Analyses. Nat Rev Immunol. 2014 Apr;14(4):271–80.

5. Altman MC, Rinchai D, et al. A Novel Repertoire of Blood Transcriptome Modules Based on Co-expression Patterns Across Sixteen Disease and Physiological States. bioRxiv 525709; doi: https://doi.org/10.1101/525709

**Ethics Approval**

The study was approved by the MD Anderson Cancer Center Ethics Board, approval number IRB 5 IRB00006023 and Sidra Medicine's IRB, approval 1804022877

**Fig. 1 (abstract P249).**
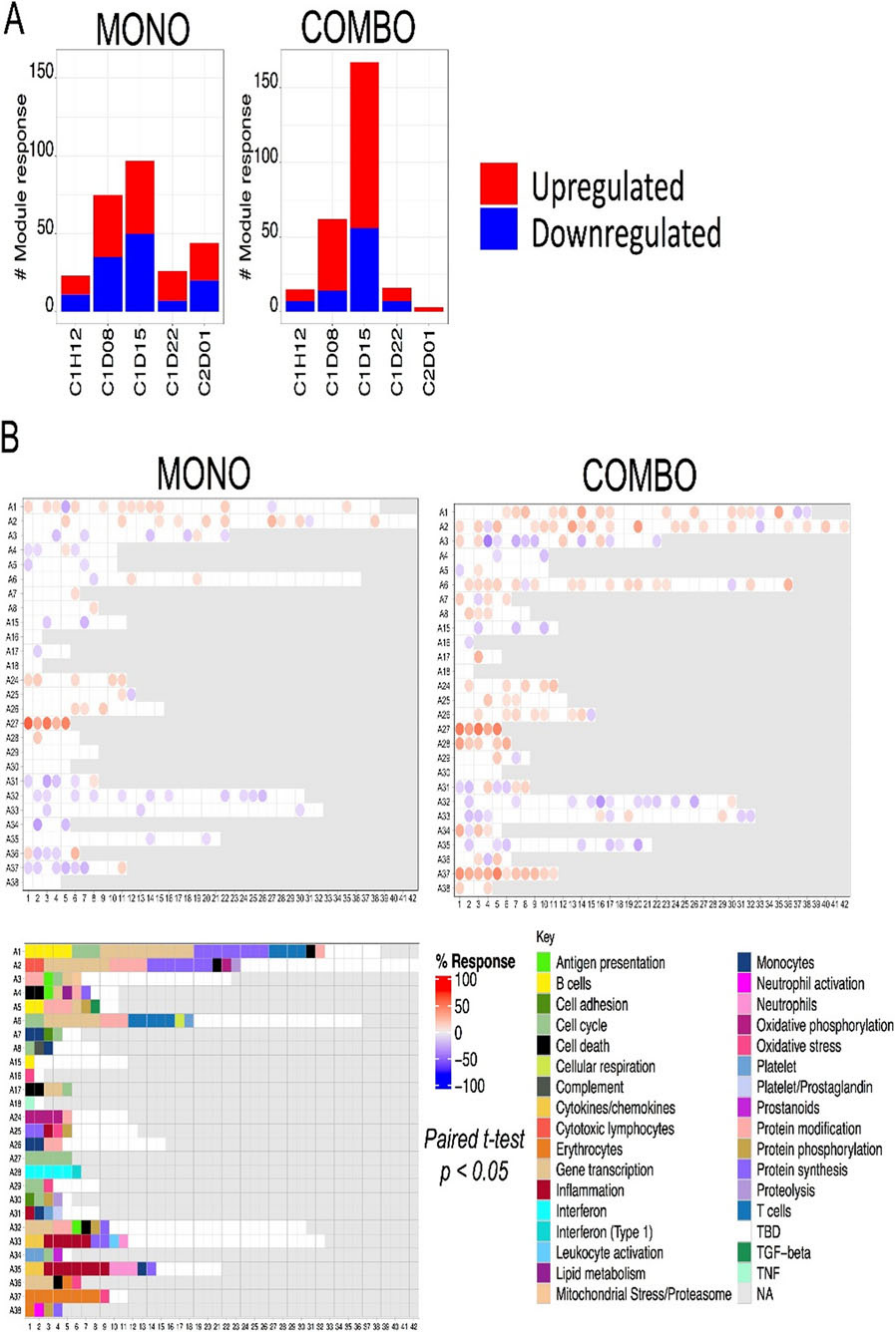
Modular repertoire analysis

#### P250 Combination treatment of the oral CHK1 inhibitor, SRA737 and low dose gemcitabine, enhances the effect of PD-L1 blockade by modulating the immune microenvironment in small cell lung cancer

##### Triparna Sen, PhD (triparnasen@gmail.com)

###### Memorial Sloan Kettering Cancer Center, New York, NY, United States

**Background**

Small cell lung cancer (SCLC), the most aggressive form of lung cancer, shows poor response rates to immunotherapy targeting the programmed cell death protein 1 pathway (PD-(L)1). Our group previously discovered that SCLC exhibits high expression of checkpoint kinase 1 (CHK1) and that the CHK1 inhibitor SRA737 activates the innate immune STING pathway, demonstrating robust anti-tumor activity and synergy in combination with anti-PD-L1 in an SCLC model.

**Methods**

As SRA737 is being tested in SCLC patients in combination with low dose gemcitabine (LDG), we evaluated the efficacy and immune correlates (including macrophages associated with resistance to immune checkpoint blockade) of the SRA737+LDG regimen in combination with anti-PD-L1 in an SCLC model.

**Results**

Trp53, Rb1 and p130 (RPP) triple knockout SCLC cells were implanted into the flank of B6129F1 immunocompetent mice. After the mice developed tumors, they were treated with single agents or various drug combinations. Anti-PD-L1 and LDG demonstrated minimal effect on tumor growth as single agents

and only a modest effect as a combination. Moderate to strong anti-tumor activity was however observed with SRA737 monotherapy which directly correlated with dosing intensity. The most profound and synergistic anti-tumor activity was observed when anti-PD-L1 was combined with the SRA737+LDG regimen, with all animals showing durable regressions. Analysis of tumor infiltrating immune cells at the end of this treatment regimen showed a dramatic induction of cytotoxic T-cells and a reduction of exhausted and regulatory T cells. Similarly, pro-inflammatory M1 type macrophages and dendritic cells were increased while immunosuppressive M2 type macrophages and MDSC cells were dramatically decreased. As monotherapy, the more dose intensive SRA737 schedule resulted in similar effects on lymphocytes when combined with anti-PD-L1. These effects are consistent with our previous data showing that SRA737 treatment leads to an induction of STING and type I interferon signaling in tumors, which is associated with the establishment of an anti-tumor immune microenvironment.

**Conclusions**

Our findings suggest that the combination of anti-PD-L1 with the SRA737+LDG regimen may represent the optimal implementation of these agents, leading to a dramatic anti-tumor activity accompanied by the establishment of a strong anti-tumor immune microenvironment. Given that anti-PD-(L)1 drugs are approved but show limited efficacy in SCLC, our preclinical data provide a strong rationale for combining these agents with the SRA737+LDG regimen

to enhance clinical response rates.

#### P251 CTX-8371, a novel bispecific targeting both PD-1 and PD-L1, is more potent than combination anti-PD-1 and PD-L1 therapy and provides enhanced protection from tumors in vivo

##### Diana Albu, PhD, Pearl Bakhru, Wilson Guzman, BS, Michael Ophir, Rachel McCrory, BS, William Carson, Jason Kong, Beata Bobrowicz, Pia Muyot, Amanda Oliphant, Dalton Markrush, Rachel Rennard, Cheuk Lun Leung, Sara Haserlat, Michael Schmidt, Jose Gonzalo, Bing Gong, Robert Tighe, BS, Diana Albu, PhD, Benjamin Wolf

###### Compass Therapeutics, Cambridge, MA, United States

####### **Correspondence:** Benjamin Wolf (benjamin.wolf@compasstherapeutics.com)

**Background**

Monoclonal antibody immunotherapies targeting immune checkpoint receptors have shown great promise for a subset of cancer patients. However, novel combination therapies are still needed to increase the benefit of cancer immunotherapy and bring it to broader patient populations. Here we describe the preclinical evaluation of CTX-8371, which combines PD-1 and PD-L1 targeting in one bispecific, tetravalent molecule.

**Methods**

Our proprietary Stitchmabs®™ bispecific screening platform was used to conduct an unbiased screen of bispecifics comprised of various checkpoint blocking antibodies. This screen yielded the surprising discovery that a bispecific containing both PD-1 and PD-L1 binding arms was more potent than the combination of parent monoclonal antibodies. We then generated common light chain bispecifics containing compatible anti-PD-1 and PD-L1 antibodies and used multiple in vitro assays to identify our lead, CTX-8371. Additional in vitro and in vivo experiments confirmed CTX-8371’s reactivity across species, in vivo anti-tumor effects, and the underlining mechanisms driving its distinctive activity.

**Results**

We found that CTX-8371 binds to human and cynomolgus monkey PD-1 and PD-L1 targets with sub-nanomolar affinities and is cross-reactive to mouse PD-1 and PD-L1. Compared to Keytruda®, CTX-8371 increased T cell activation and tumor cell killing in vitro, significantly delayed tumor growth, and prolonged survival in human cell transfer tumor models. Additionally, CTX-8371 demonstrated efficacy in transplantable mouse syngeneic models. Investigation into the mechanisms responsible for the enhanced efficacy of CTX-8371 unexpectedly found that the bispecific causes a massive loss of PD-1 from the T cell surface, which was not observed in response to monoclonal antibodies alone or combined. This robust PD-1 downregulation, potentially mediated through bridging together the T cell and tumor cell, may explain the ability of CTX-8371 to reverse PD-1 suppression more potently than standard blocking antibodies.

**Conclusions**

Taken together, the results demonstrate that the bispecific, tetravalent antibody CTX-8371 has increased potency in vitro and in vivo as compared to clinical checkpoint blockade agents. Some of its effects are likely attributable to its unique mechanism of action, driving robust downregulation of cell-surface PD-1. Thus, CTX-8371 has the potential to increase the number of patients that benefit from PD-1/PD-L1 checkpoint blockade.

#### P252 A retrospective study to evaluate real-world clinical outcomes in patients with metastatic renal cell carcinoma (mRCC) treated with Ipilimumab and Nivolumab

##### Landon Brown, MD, Emily Kinsey, MD, Chester Kao, MD, Patrick Healy, Andrew Armstrong, MD, Megan McNamara, MD, Sundhar Ramalingam, MD, Michael Harrision, MD, Daniel George, MD, Tian Zhang, MD

###### Duke University, Durham, NC, United States

####### **Correspondence:** Landon Brown (landon.brown@duke.edu)

**Background**

The combination of nivolumab at 3mg/kg plus ipilimumab at 1mg/kg (I+N) followed by maintenance nivolumab has greatly improved outcomes in patients with intermediate or poor-risk untreated mRCC [1]. Real-world series of patients treated with this combination are scarce. In this retrospective analysis, we present a real-world experience with this combination immunotherapy.

**Methods**

A search was performed to identify all mRCC patients treated in the Duke Cancer Institute network with I+N. An extensive chart review was conducted. Patient characteristics are summarized with descriptive statistics; Kaplan Meier analysis was performed for progression free survival (PFS) and overall survival (OS).

**Results**

From 10/2017 to 2/2019, 83 patients received I+N for mRCC and were included. Demographics are shown in Table 1. By International Metastatic RCC Database Consortium (IMDC) risk criteria [2], 20.5% were favorable, 61.4% intermediate, and 18.1% poor risk. 65% were stage IV at diagnosis, 63.9% were untreated, and 16.9% patients had prior nivolumab exposure. 77.1% of patients had clear cell pathology. 12/83 (14.4%) have sarcomatoid differentiation: 2 have an ongoing response and 7 have died. At the data cutoff date, 44/83 (53%) patients have progressed or died. Median PFS was 5.3 months (95% CI 3-8.5) (Figure 1). OS rates at 6, 12, and 18 months were 76.2%, 63.8%, and 51.5%, respectively (Figure 2). Rates of best radiographic response were CR 4.8%, PR 22.9%, SD 18.1%, PD 32.5%, and unknown 21.7%. 44/83 (53%) patients experienced no adverse event (AE). 18/83 (21.7%) patients experienced a grade 3/4 AE (most commonly diarrhea, n=7), 20/83 (24%) patients experiencing a grade 1-2 AE at worst (most commonly hypothyroidism, n=14), and one grade 5 AE occurred. 23/83 (27.7%) patients have died, with 10/83 (12.0%) patients dying within 90 days of receiving the first dose of I+N. 4/83 patients have achieved a complete response. Two of these patients discontinued treatment at 11 and 12 months with a sustained response at 1 and 5 months, respectively. Three other patients remain off therapy for AEs and have not progressed after 11, 5, and 3 months.

**Conclusions**

In our real-world cohort of mRCC patients, I+N has similar clinical efficacy as previously described; however, the cohort is more frail, with 16.9% of patients treated in the nivolumab refractory setting. Five patients remain off therapy. This forms the basis for larger prospective treatment discontinuation trials (ie. Alliance A031704 phase 3 trial) with prospective treatment discontinuation for complete response patients at 1-year.

**References**

1. Motzer RJ, Tannir NM, McDermott DF, et al. Nivolumab plus Ipilimumab versus Sunitinib in Advanced Renal-Cell Carcinoma. N Engl J Med. 2018;378(14):1277-1290.

2. Heng DY, Xie W, Regan MM, et al. Prognostic factors for overall survival in patients with metastatic renal cell carcinoma treated with vascular endothelial growth factor-targeted agents: results from a large, multicenter study. J Clin Oncol. 2009;27(34):5794-5799.

**Ethics Approval**

This study was approved by the Duke University IRB (#Pro00101984)

**Fig. 1 (abstract P252).**
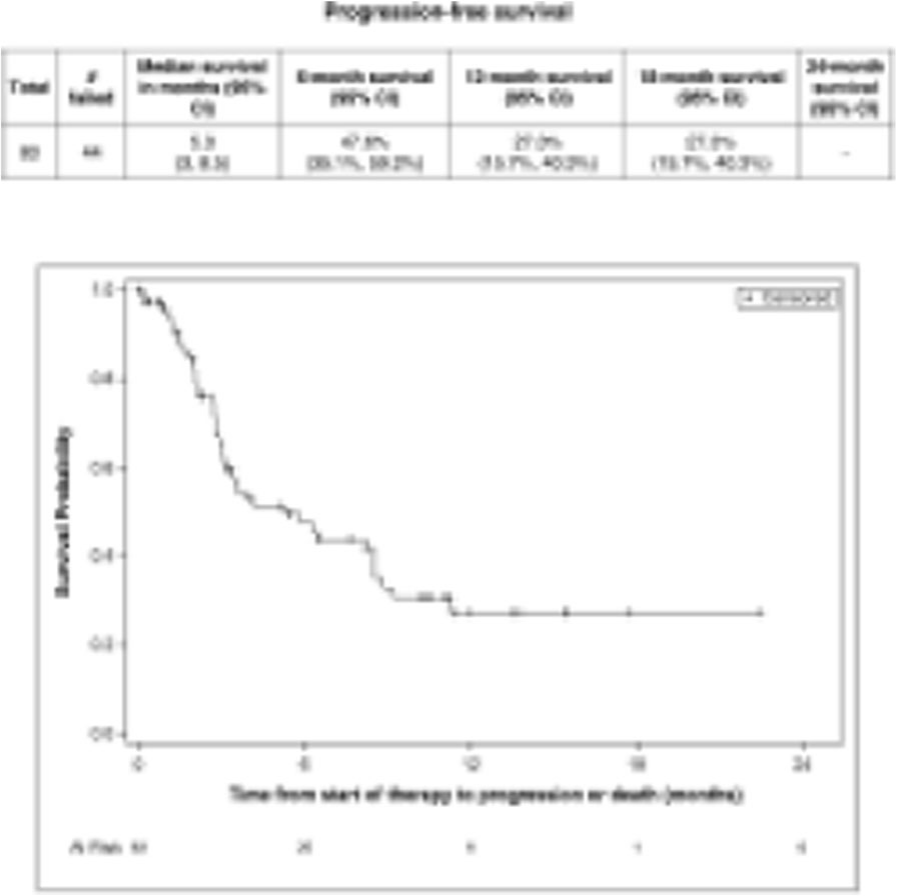
See text for description

**Fig. 2 (abstract P252).**
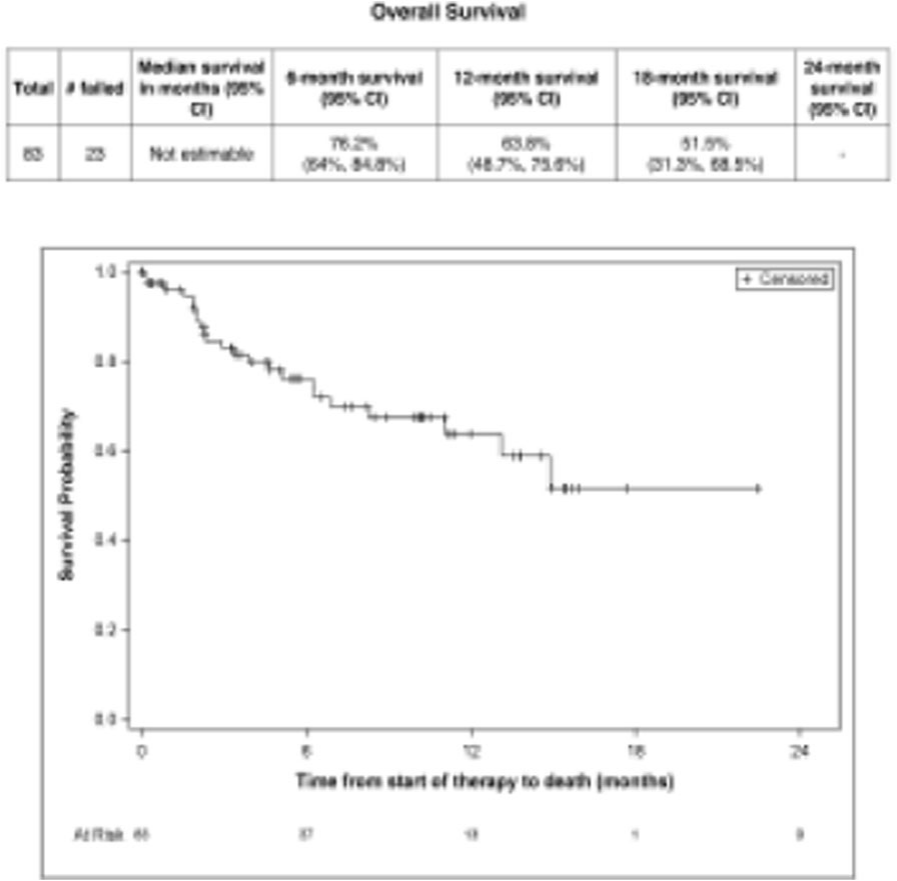
See text for description

**Table 1 (abstract P252).**
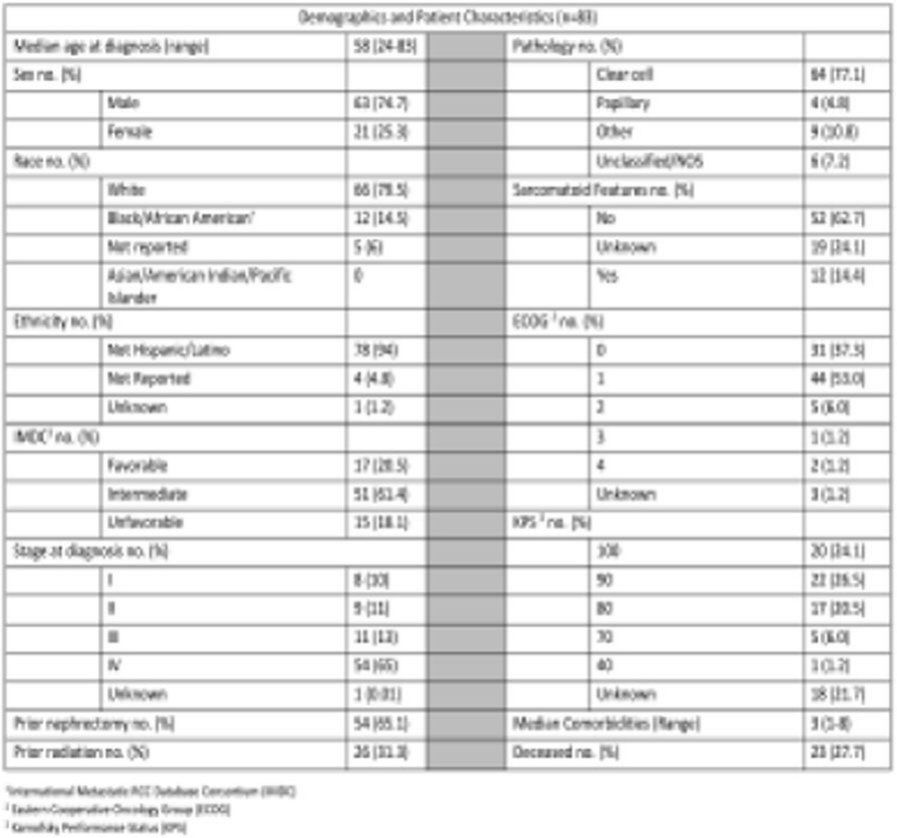
See text for description

#### P253 Distinct clinical and immunological responses to αPD-1, ⍺PD-L1 and ⍺PD-L2 immunotherapy in B16 melanoma in aged versus young hosts includes T-cell stem cell effects and PD-L2 expression differences

##### Myrna Garcia, BS, Alvaro Padron, Yilun Deng, MD, PhD, Harshita Gupta, PhD, Aravind Kancharla, Tyler Curiel, MD

###### University of Texas Health Science Center at San Antonio, San Antonio, TX, United States

####### **Correspondence:** Tyler Curiel (curielt@uthscsa.edu)

**Background**

Aging is the biggest risk factor for cancer, yet little is known about cancer immunotherapy effects. αPD-1 can block PD-L1 and PD-L2 while ⍺PD-L1 blocks PD-1 and CD80 [1]. A recent key finding in young hosts including humans is that melanoma response to αPD-1/αPD-L1 correlates with CD8+TCF-1+ T cell stem cell (TCSC) generation [2].

**Methods**

We tested αPD-1 (100 μg/mouse), αPD-L1 (100 μg/mouse) or αPD-L2 (200 μg/mouse) in aged (18-24 months) and young (3-8 months) mice challenged orthotopically with B16. Tumors and draining lymph nodes (TDLN) were analyzed by flow. Bone marrow-derived DC were generated with GM-CSF.

**Results**

We reported that αPD-1 treats young and aged with B16 and αPD-L1 only treats young [3]. αPD-L2 treated B16 in aged but, remarkably, not young, the first anti-cancer single agent immunotherapy exhibiting this property. Efficacy in young (αPD-1, αPD-L1) and aged (αPD-1, αPD-L2) correlated with increased TCSC and total TIL, but TCSC differed by age and treatment (e.g., distinct CCR2, CXCR5, CXCR3, PD-1 and TIM-3 expression). Aged expressed significantly more T-cell PD-1 and up to 40-fold more PD-L2 versus young in myeloid and NK cells, and TCSC. Bone marrow-derived DC experiments suggest aged DC are destined for high PD-L2 versus young.

**Conclusions**

Treatment differences in aged versus young could depend on immune checkpoint or TCSC differences, which could be related to CD8+ T-cell infiltration, including TCSC. PD-L2 expression differences could be a mechanism for treatment differences. We are now identifying mechanisms for increased PD-L2 and contributions to αPD-L2 efficacy in aged, and testing TCSC effects on treatments (Figure 1-3). Our work can improve cancer immunotherapy in aged hosts and further provide important insights even in young hosts.

**Acknowledgements**

South Texas MSTP training grant (NIH T32GM113896), TL1TR002647, R01 CA231325.

**References**

1. Schildberg FA, Klein SR, Freeman GJ, Sharpe AH. Coinhibitory Pathways in the B7-CD28 Ligand-Receptor Family. Immunity. 2016;44(5):955-72.

2. Im SJ, Hashimoto M, Gerner MY, Lee J, Kissick HT, Burger MC, et al. Defining CD8+ T cells that provide the proliferative burst after PD-1 therapy. Nature. 2016;537(7620):417-21.

3. Padron A, Hurez V, Gupta HB, Clark CA, Pandeswara SL, Yuan B, et al. Age effects of distinct immune checkpoint blockade treatments in a mouse melanoma model. Exp Gerontol. 2018;105:146-54.

**Ethics Approval**

All animal work was done under UTHSA Institutional Animal Care and Use Committee approved studies in compliance with the Guide for the Care and Use of Laboratory Animal Resources (published by National Research Council of the National Academies), Animal Welfare Act (AWA) (published by USDA), Public Health Service Policy on Humane Care and Use of Laboratory Animals (published by NIH) and US Government Principles for Utilization and Care of Vertebrate Animals Used in Testing, Research, and Training. Approval number: 20180021AR.

**Fig. 1 (abstract P253).**
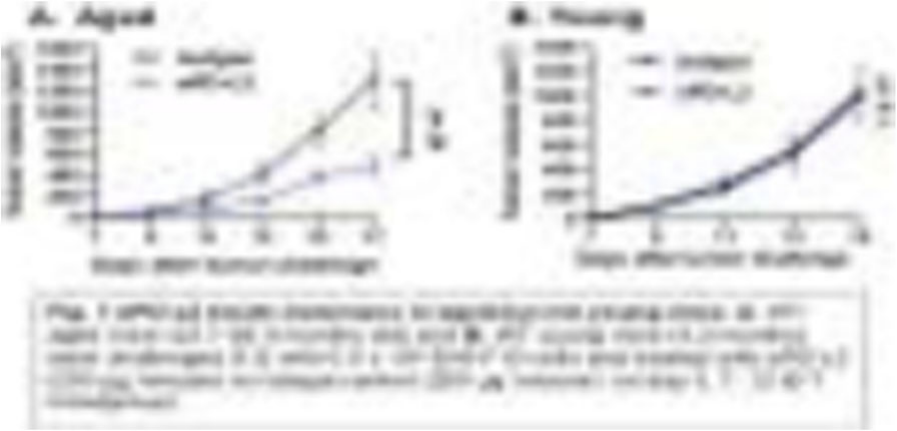
⍺PD-L2 treats B16 melanoma in aged mice but not young mice

**Fig. 2 (abstract P253).**
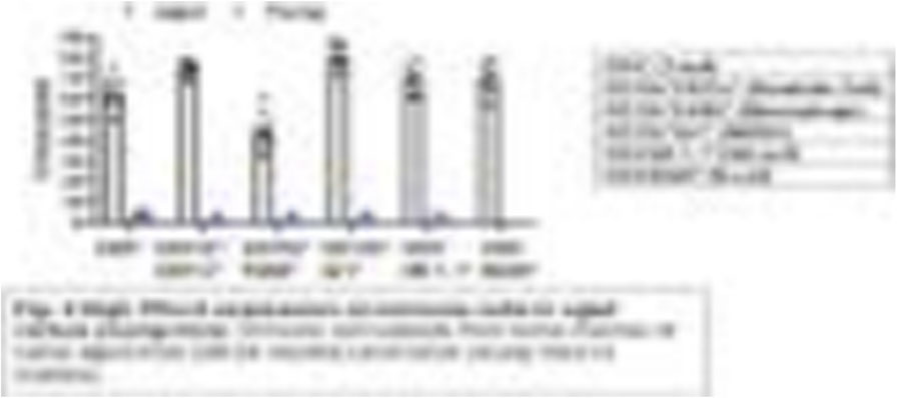
PD-L2 expression is significantly higher in aged mice when compared to young

**Fig. 3 (abstract P253).**
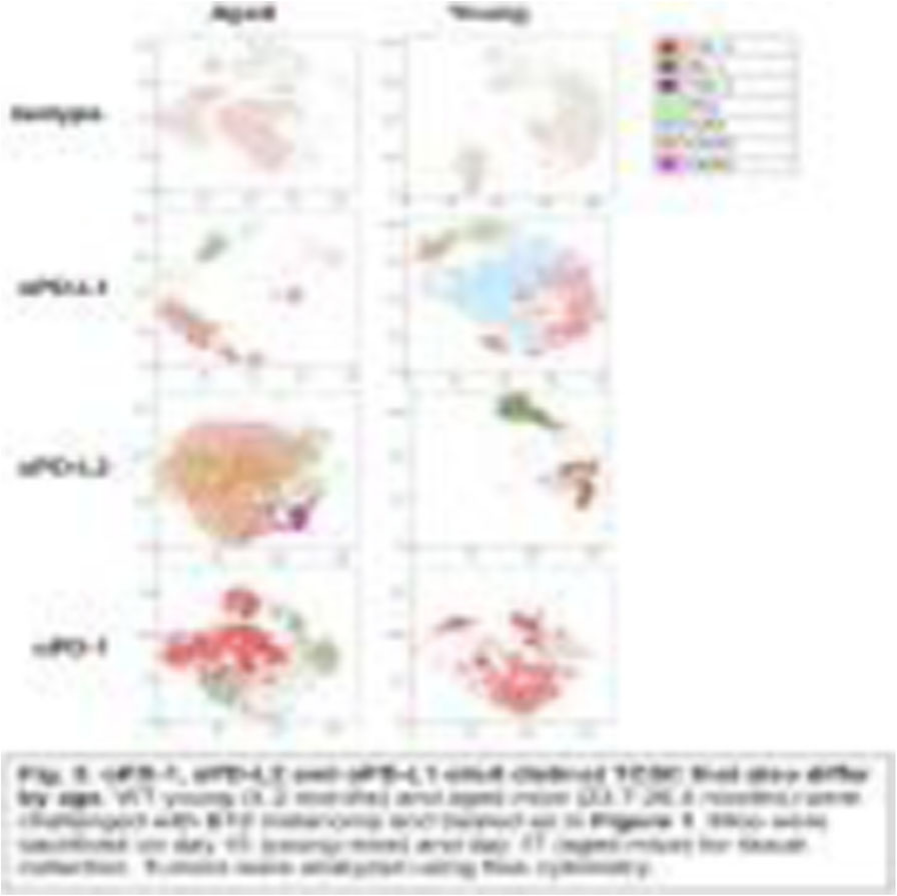
αPD-1, αPD-L2 and αPD-L1 elicit distinct TCSC that also differ by age

#### P254 Requirement of Fc gamma receptor-mediated myeloid-cell activation for effective cancer immunotherapy with an anti-TIGIT antibody

##### Jin-hwan Han, PhD, Mingmei Cai, Jeffery Grein, Samanthi Perera, PhD, Hongmei Wang, Mike Bigler, Roenna Ueda, Thomas Rosahl, Elaine Pinheiro, PhD, Drake LaFace, Wolfgang Seghezzi, Sybil Williams, PhD

###### Merck Research Laboratories, South San Francisco, CA, United States

####### **Correspondence:** Sybil Williams (sybil_williams@merck.com)

**Background**

The molecule “T cell immunoreceptor with immunoglobulin and ITIM domain”, or TIGIT, has recently received much attention as a promising target in the treatment of various malignancies. In spite of the quick progression of anti-TIGIT antibodies into clinical testing both as monotherapy and in combination with programmed death-1 (PD-1)–directed immune checkpoint blockade, the molecular mechanism behind the observed therapeutic benefits remains poorly understood.

**Methods**

Anti-Mouse TIGIT (mTIGIT) blocking antibodies of two distinct isotypes (mouse IgG1 with D265A mutation and mouse IgG2a) and TIGIT-deficient mice were generated and used to demonstrate the requirement of IgG-Fc gamma receptor interaction for effective anti-tumor response in vivo studies. Gene expression profiling of whole tumors after in vivo treatment of anti-mTIGIT antibody with functional or non-functional Fc as monotherapies or in a combination with anti-PD-1 was carried out in order to better understand the effects of anti-TIGIT antibody with functional Fc at molecular level at different time points.

**Results**

Here we demonstrate using mouse tumor models that anti-mTIGIT antibodies require interactions with Fc gamma receptors on myeloid cells in the tumor microenvironment for effective anti-tumor response. Our observations reveal that the anti-mTIGIT therapeutic effect is not achieved by depletion of intratumoral regulatory T cells, but instead is mediated by “reverse activating signals” through Fc gamma receptors on myeloid cells, inducing expression of various mediators such as cytokines, including TNF-alpha and IL-23, and chemokines, such as CXCL10 and CXCL11, thus generating the conditions for potentially promoting immune infiltrates into the tumor microenvironment. In addition, up-regulation of co-stimulatory molecules, such as CD80, CD86, and CD40, has been observed, consistent with the heightened anti-tumor activity of Fc gamma receptor binding competent anti-mTIGIT antibodies. Furthermore, we discovered induction of a robust and persistent granzyme B and perforin response from the in vivo treatment of anti-mTIGIT antibody with a functional Fc, distinct from a predominantly interferon-gamma-driven anti-PD-1 blockade.

**Conclusions**

Our observations for the first time provide mechanistic insights into the requirement for Fc engagement of anti-mTIGIT monoclonal antibodies for effective anti-tumor activity in vivo which has implications for the various human antibodies of various isotypes are currently under intense clinical investigations.

#### P255 Preclinical characterization and efficacy of MG1124, a novel immune checkpoint blockade targeting CEACAM1 for cancer therapy

##### Jae-Chul Lee, Master degree^1^, Minkyu Hur^1^, Hye-Young Park^1^, Mi-Young Oh^1^, Hye-mi Nam^1^, Hye In Yum^1^, HyungSuk Choi^2^, Jaehwan Kim^3^, Byoung Chul Cho, MDphD^3^, Yangmi Lim^1^

###### ^1^MOGAM Institute for Biomedical Research, Yong-in, Korea, Republic of; ^2^GC Pharma, Yongin-si, Korea, Republic of; ^3^Yonsei Cancer Center, Seoul, Korea, Republic of

####### **Correspondence:** Yangmi Lim (ymlim@mogam.re.kr)

**Background**

CEACAM1 is one of the several immune checkpoint receptors expressed on T cells and NK cells that mediate suppression of inflammatory T cell response. It is known that CEACAM1-CEACAM1 homophilic interaction induces downregulation of ZAP70 phosphorylation in response to T cell receptor (TCR) stimulation. CEACAM1 is also highly expressed on non-small cell lung cancer (NSCLC) and its expression is correlated with cancer progression and poor prognosis. We developed a fully human monoclonal antibody MG1124, targeting human CEACAM1.

**Methods**

T cell activation of MG1124 was determined by an NFAT-luciferase reporter assay with CEACAM1 overexpressing Jurkat stable cells. Evaluation of the homophilic interaction of CEACAM1 or interaction of CEACAM1 with CEACAM6 was performed by protein ELISA. In vitro efficacy of MG1124 was examined using an NK-mediated tumor cell killing assay. The anti-tumor efficacy of MG1124 alone or in combination was studied in vivo in a humanized mouse model engrafted with NSCLC patient-derived tumor xenografts.

**Results**

Anti-CEACAM1 antibody MG1124 bound to CEACAM1 but not to other CEA family members. MG1124 blocked CEACAM1-CEACAM1 homophilic interaction and CEACAM1-CEACAM6 heteropilic interation by binding to the N domain of CEACAM1. Especially CEACAM1-CEACAM1 homophilic interaction induced downregulation of ZAP70 phosphorylation in response to TCR stimulation in a CEACAM1 overexpressing Jurkat stable cell line, which was rescued by MG1124 resulting in augmentation of NFAT activity and IL-2 expression. NK cell-mediated tumor lysis was increased by MG1124 in a CEACAM1 expression-dependent manner. In an NSCLC PDX-huNSG mouse model, MG1124 suppressed tumor progression as a monotherapy and combination with pembrolizumab in a CEACAM1 high expressing model. In single mouse trial analysis, MG1124 suppressed tumor progression more than 30% as monotherapy (53%, 10/19) as well as in combination (73%, 16/22) with pembrolizumab (5 mpk, 2qW). Moreover, PDXs of adenocarcinoma origin with more than 50% of CEACAM1 expression were more efficiently prohibited for progression with MG1124, suggesting the potential therapeutic use of MG1124 in patients with NSCLC.

**Conclusions**

MG1124, an anti-CEACAM1 antibody, blocked CEACAM1-mediated negative regulation and restored T/NK cell activities. MG1124 showed effective anti-tumor activity in in vivo mouse models and its combination with PD-1 blockade further enhanced treatment efficacy. MG1124 is a potential therapeutic candidate for immune checkpoint blockade in cancer therapy.

#### P256 A novel bifunctional anti-PD-1 / IL-7 fusion protein potentiates effector function of exhausted T cell and disarms Treg suppressive activity

##### Aurore Morello, PhD^1^, Justine Durand^1^, Caroline Mary^1^, Virginie Thepenier^1^, Margaux Seite^1^, Géraldine Teppaz^1^, Nicolas Poirier^2^

###### ^1^OSE immunotherapeutics, Nantes, France; ^2^Poirier Household, Nantes, France

####### **Correspondence:** Nicolas Poirier (nicolas.poirier@ose-immuno.com)

**Background**

Despite the clinical success of anti-PD(L)1 therapies, most patients remain unresponsive or fail to develop a durable response. We explored a second generation of PD-1 antibody by fusing IL-7 cytokine to the Fc portion. IL-7 is an optimal target for immunotherapy to preferentially stimulate effector T-cell (Teff) functions over regulatory T-cells (Treg), due to the differential expression of IL-7R. Moreover, It has been published that PD-1 blockades increase IL-7R expression and improve IL-7 signaling in exhausted T-cells rationalizing our combinatorial approach.

**Methods**

Proliferation (H3 thymidine), IFN-γ, IL-7R signaling (pSTAT5) and NFAT (PD-1 bioassay, Promega) assays were tested to determine anti PD-1/IL-7 efficacy on naïve and/or exhausted-like T-cells. For the suppressive assay, CD4 Treg and autologous CD8 Teff were co-cultured (1:1) and proliferation was assessed on Day 5. Tumor infiltrating T cells (TILs) were isolated from orthotopic tumor-bearing mice (Hepa1.6, LLC-1,AK7) and subjected to IL-7 ex vivo, IL-7R signaling pSTAT5 was determined by flow cytometry.

**Results**

Our anti PD-1/IL-7 bispecific antibody efficiently blocks the PD-1/PD-L1 and PD-L2 interactions and the PD-1-mediated inhibitory signal (pSHP1). Importantly, we observed that the IL-7 portion synergizes with the anti-PD-1 to enhance TCR mediated signaling (NFAT). Although IL-7R expression on T cells decrease over repeated antigen stimulation, we demonstrated that IL-7 still efficiently activate partially and fully-exhausted human T-cells (pSTAT5) and maintain their proliferation capacity. We next characterized sensitivity of TILs to IL-7 in multiple orthotopic mouse models. In PD-1 sensitive tumor (Mesothelioma), only 10% of TILs express IL-7R whereas in PD-1 resistant model (Hepatocarcinoma and Lung carcinoma), 40-60% of TILs (CD4 and CD8) express IL-7R and respond to IL-7 stimulation ex vivo as measured by pSTAT5 signaling. These data suggest that the anti PD-1/IL-7 bispecific can reactivate TILs that are resistant to PD-1 therapy. Knowing that Tregs have a key suppressive function, we also explored the possibility that the anti-PD-1/IL-7 fusion protein affect Treg functions. In a human Treg/Teff coculture assay, we observed that the anti PD-1/IL-7 molecule abrogate the Treg capacity to inhibit proliferation and IFN-gamma secretion of CD8+ Teff. Moreover, IL-7 and the anti-PD-1/IL-7 does not stimulate Treg proliferation, in contrast to IL-2 and IL-15 cytokines.

**Conclusions**

Our data validate the therapeutic potential of providing IL-7 signals to overcome PD-1 resistance. The bifunctional anti-PD1/IL-7 favors the T-cell effector over T-regulatory immune balance by stimulating effector and exhausted T-cells while disarming Tregs suppressive functions.

#### P257 Obesity is associated with diminished anti-PD-1-based immunotherapy response rates in renal cancer

##### Rachael Orlandella, BS^1^, Shannon Boi^1^, Justin Gibson, BS^1^, William Turbitt^1^, Gal Wald^2^, Lewis Thomas^2^, Katlyn Norris^1^, Lakshminarayanan Nandagopal, MD^1^, Peng Li, PhD^1^, Eddy Yang, MD, PhD^1^, Tatiana Marquez-Lago, PhD^1^, Lyse Norian, PhD^1^

###### ^1^University of Alabama, Birmingham, AL, United States; ^2^University of Iowa, Iowa City, IA, United States

####### **Correspondence:** Lyse Norian (lnorian@uab.edu)

**Background**

Obesity is a regarded as a major risk factor for developing renal cell carcinoma (RCC). Despite the success of anti-PD-1 checkpoint blockade in RCC, response rates remain low (20-30%). Recent studies have observed that obesity is associated with heightened frequencies of PD-1+ CD8 T cells [1] and favorable outcomes and responses to immunotherapy in melanoma [1, 2]. However, the effects of obesity on anti-tumor immunity and immunotherapeutic efficacy in renal cancer remain unknown.

**Methods**

PD-1 expression on tumor-infiltrating CD8 T cells from treatment-naive RCC subjects with (BMI >30 kg/m2) or without (BMI < 30 kg/m2) obesity (n = 18) was determined via flow cytometry. In a separate retrospective study, outcome data were queried for RCC patients with (BMI >30 kg/m2) or without (BMI < 30 kg/m2) obesity that were treated with anti-PD-1 as standard of care and had at least 6 months of follow-up (n=58). Overall survival (OS) was analyzed using Kaplan-Meier methods and Cox proportional hazards regression after controlling for patients’ age, sex, and number of prior treatments. For murine studies, BALB/c mice were randomized to and maintained on either standard chow or high-fat diet for 20 weeks to generate age-matched lean or diet-induced obese (DIO) mice. Mice were then given an orthotopic renal tumor challenge with syngeneic Renca cells and treated with an anti-PD-1-based combination immunotherapy or saline.

**Results**

Obesity was associated with reduced frequencies of intratumoral PD-1highCD8+ T cells in treatment-naive murine and human renal tumors. Although the majority (73%) of lean mice responded to immunotherapy, DIO mice exhibited a reduced response rate (44%). Lean and DIO responders exhibited favorable ratios of activated CD8+ T cells to myeloid-derived suppressor cells (MDSC), reduced PD-1 expression on CD8+ T cells, and elevated concentrations of CCL5 in renal tumors. Neutralization of CCL5 in lean immunotherapy-treated mice yielded a reduced response rate (43%), unfavorable ratios of activated CD8+ T cells to MDSCs, and diminished IFNg secretion from intratumoral CD8+ T cells. The translational relevance of our murine findings was reflected in metastatic RCC patients, as patients with obesity had a trending reduction in OS following standard of care nivolumab (p= 0.06) and a 10.2 month reduction in OS.

**Conclusions**

Our data suggest that obesity is associated with reduced responses to anti-PD-1 based immunotherapies in the context of renal cancer. Continued study of this critical issue is needed to better inform patient care.

**Acknowledgements**

Financial support was provided by NIH grant R01CA181088 to LAN; CPCTP T32 fellowship #T32CA047888 to RMO; CPCTP R25 fellowship #R25CA047888 to SKB; CMDB T32 fellowship #T32GM008111 to JTG; and Oncology T32 fellowship #T32CA183926 to WJT.

**References**

1. McQuade, J.L., et al., Association of body-mass index and outcomes in patients with metastatic melanoma treated with targeted therapy, immunotherapy, or chemotherapy: a retrospective, multicohort analysis. Lancet Oncol, 2018. 19(3): p. 310-322.

2. Wang, Z., et al., Paradoxical effects of obesity on T cell function during tumor progression and PD-1 checkpoint blockade. Nat Med, 2019. 25(1): p. 141-151.

**Ethics Approval**

Human subject studies were approved by the UAB IRB (protocol # X151013003); murine studies were approved by UAB IACUC (protocol #20233).

#### P258 Combination of NK Cells and anti-PD-L1 Ab with ADCC enhances the anti-tumor effects in PD-L1 high cancer cells

##### Ji-Eun Park, BS^1^, Bhumsuk Keam, MD, PhD^2^, Ha-ram Park^1^, Soyeon Kim, PhD^3^, Chan-Young Ock, MD, PhD^2^, Miso Kim^2^, Tae Min Kim, MD, PhD^2^, Dong-Wan Kim, MD PhD^2^, Dae Seog Heo^2^

###### ^1^Seoul National University Cancer Research Institute, Seoul, Korea, Republic of; ^2^Seoul National University Hospital, Seoul, Korea, Republic of; ^3^Biomedical Research Institute, SNUH, Seoul, Korea, Republic of

####### **Correspondence:** Bhumsuk Keam (bhumsuk@snu.ac.kr)

**Background**

Although Programmed cell death-1 (PD-1)/ Programmed death-ligand 1 (PD-L1) inhibitors showed remarkable antitumor activity, a large portion of cancer patients do not response to PD-1/PD-L1 inhibitors even in the PD-L1 high tumor. Most of PD-L1 inhibitors were modified in FcR binding site to prevent antibody-dependent cellular cytotoxicity (ADCC) against PD-L1 expressing non-tumor cells. IMC-001, developed by ImmuneOncia, is a fully human PD-L1 recombinant monoclonal antibody that did not modify FcR binding and preserved ADCC. Therefore, IMC-001 would be synergistic with NK compared to other PD-L1 monoclonal antibodies (mAbs). We evaluate anti-tumor efficacy of IMC-001 and NK cells against several PD-L1 high cancer cell lines through ADCC.

**Methods**

PD-L1 expression was measured by flow cytometry. Standard 51Cr-release and CD107a degranulation assays were performed to evaluate the in vitro ADCC efficacy of 3 groups: control, anti-PD-L1 Ab without ADCC (atezolizumab), anti-PD-L1 Abs with ADCC (IMC-001, Anti-hPD-L1-hIgG1 [atezolizumab with wild type FcR binding site, hPD-L1mab]). Various cancer cell lines were used as target cells, including head and neck squamous carcinoma (HNSCC), lung cancer, stomach cancer, ovarian cancer, bladder cancer and lymphoma cell lines. 51Cr-release assay was performed using NK-92-CD16 as an effector cell with effector to target ratio (E:T) of 30:1. CD107a degranulation assay was performed using peripheral blood mononuclear cells (PBMC) from healthy donors with E:T ratio of 1:1. PBMC was activated by IL-15 and grouped by CD16 V158F genotyping individually.

**Results**

The expression of PD-L1 is high in several cell lines including SNU-1076 (8.8±1.3), FaDu (15±0.1), HN31 (21.8±1.1) and H1975 (11.3±1.7). NK cell cytotoxicity in PD-L1 high cell lines was more potent in IMC-001 or anti-hPD-L1-hIgG1 compared to control treatment or atezolizumab. The PD-L1 high or PD-L1 low tumor cell specific lysis was detected by 51Cr-release assay in control group (isotype and atezolizumab) vs. and anti-PD-L1Ab with ADCC groups (IMC-001 vs. Anti-hPD-L1-hIgG1) (Figure 1). Besides, in CD107a degranulation assay, activated PBMC cytotoxicity was increased when target cells are opsonized by anti-PD-L1 Abs with ADCC. NK cells that characterized with CD16 high affinity genotype (V/F) are more enhancing ADCC than (F/F) low affinity genotype. Two genotyped (V/F vs. FF) NK cell lysis induced by IMC-001 in FaDu cells was 39.3% vs. 12.8%.

**Conclusions**

Anti-PD-L1 Abs with ADCC, such as IMC-001, enhanced the cytotoxic activity of NK cells on various PD-L1 high cell lines. This study provides rationale that NK92-CD16 or NK cell immunotherapy for PD-L1 high tumor through combination with ADCC preserved anti-PD-L1 Ab.

**Fig. 1 (abstract P258).**
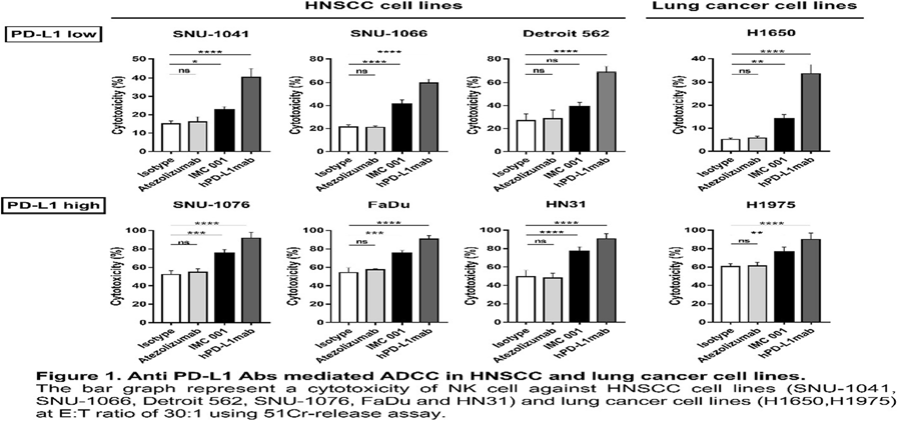
See text for description

#### P259 Hydrogel-enabled intratumoral co-delivery of anti-PD-1 antibody and adenosine deaminase in a mouse model of renal cell carcinoma

##### Ketki Velankar, MS, Ngoc Pham, BS, Wilson Meng, PhD, Ellen Gawalt, Nathan Schueller

###### Duquesne University, Pittsburgh, PA, United States

####### **Correspondence:** Wilson Meng (meng@duq.edu)

**Background**

It is hypothesized that tumor resistance to anti-PD-1 monoclonal antibodies is due in part to the accumulation of adenosine (ADO) generated in the tumor microenvironment (TME). ADO impairs the activation and proliferation of effector T cells while expanding regulatory T cell (Treg) population, which is inversely related to the overall survival of cancer patients, including those with renal cell carcinoma (RCC). We propose to develop an injectable system by which ADO are degraded in the TME in order to enhance the efficacy of anti-PD1 treatment. To this end, we have developed a hydrogel to co-deliver anti-PD-1 antibody with adenosine deaminase (ADA), which catabolizes ADO. The hydrogel contains a bioaffinity module (named “Z15_EAK”) to retain the anti-PD-1 antibody in tumors while limit the diffusion of ADA in TME for extended durations. We have previously shown that Z15_EAK hydrogel can retain IgG at subcutaneous injection site for at least two weeks [1]. The expectation is that persistent co-localization of anti-PD-1 and ADA in the TME will expand Th1 T cells and reduce Treg in draining lymph nodes (DLN) and systemic lymphoid tissues. This postulation was tested in an immunocompetent mouse model of RCC.

**Methods**

A mouse RCC cell line (RENCA) was cultivated for in vitro assays and in vivo inoculation into BALB/c mice. Beginning three days after tumor inoculation, the hydrogel loaded with an anti-PD-1 IgG antibody and ADA was injected subcutaneously in the peri-tumoral region for three doses three days apart. DLN, spleen, and tumors were collected for flow cytometric analysis and ELISA measurements.

**Results**

After three doses, DLN in mice received the hydrogel loaded with anti-PD-1 antibody and ADA were five times larger than those in mice received saline control (Figure 1). In addition, the lymph nodes in treated mice contained fewer CD4+CD25+FoxP3+ Treg cells compared to controls (Figure 2). After ex vivo re-stimulation with RENCA for expansion, lymphocytes in treated mice exhibited higher interferon-gamma levels than controls, indicating elevated Th1 phenotype in the DLN.

**Conclusions**

The preliminary data indicate that the local delivery of anti-PD-1 and ADA with the hydrogel shifted the local T cell population toward an effector phenotype (Th1) while limiting the Treg expansion. An extended co-localization of anti-PD-1 and ADA in the TME not only modulates immune events in the local lymphoid tissues but can also enhance the anti-tumor response systemically. Furthermore, the localized delivery reduces off-target toxicities of anti-PD-1 antibody.

**Reference**

1. Pham, N.B., et al. Toward reducing biomaterial antigenic potential: a miniaturized Fc-binding domain for local deposition of antibodies. Biomaterials Science. 2019: 7(3): 760-772.

**Ethics Approval**

The animal study was approved by Duquesne University's Ethics Board, approval number 180604.

**Fig. 1 (abstract P259).**
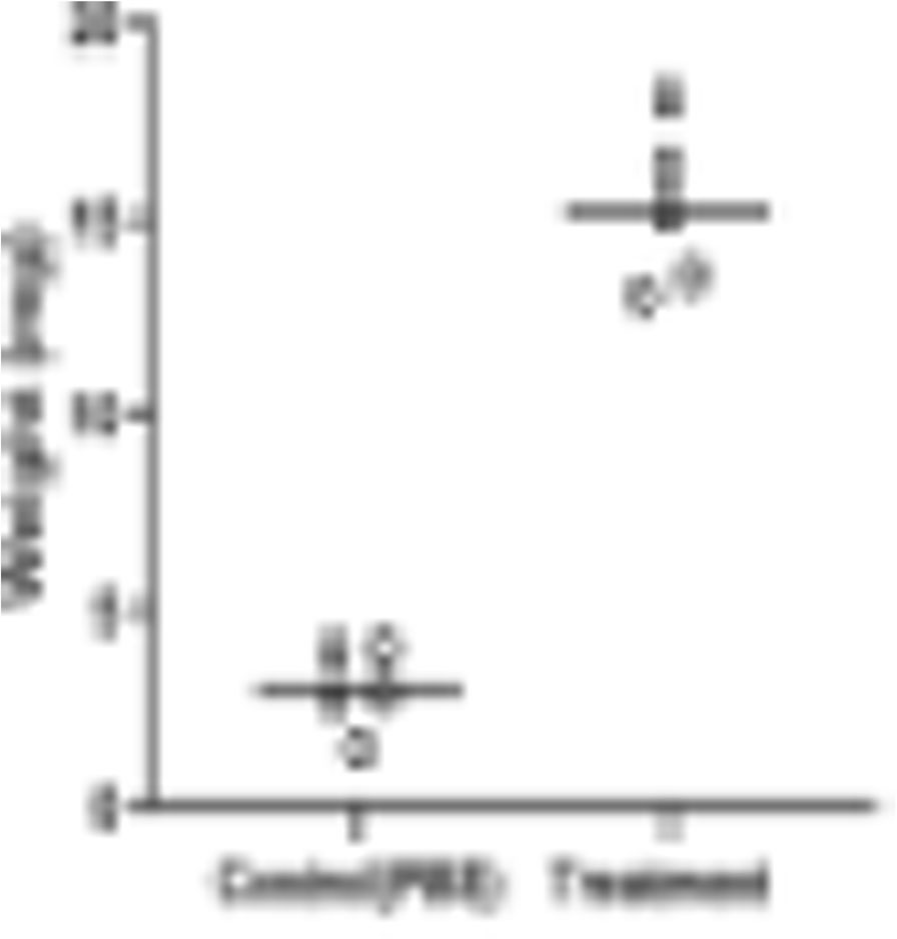
See text for description

**Fig. 2 (abstract P259).**
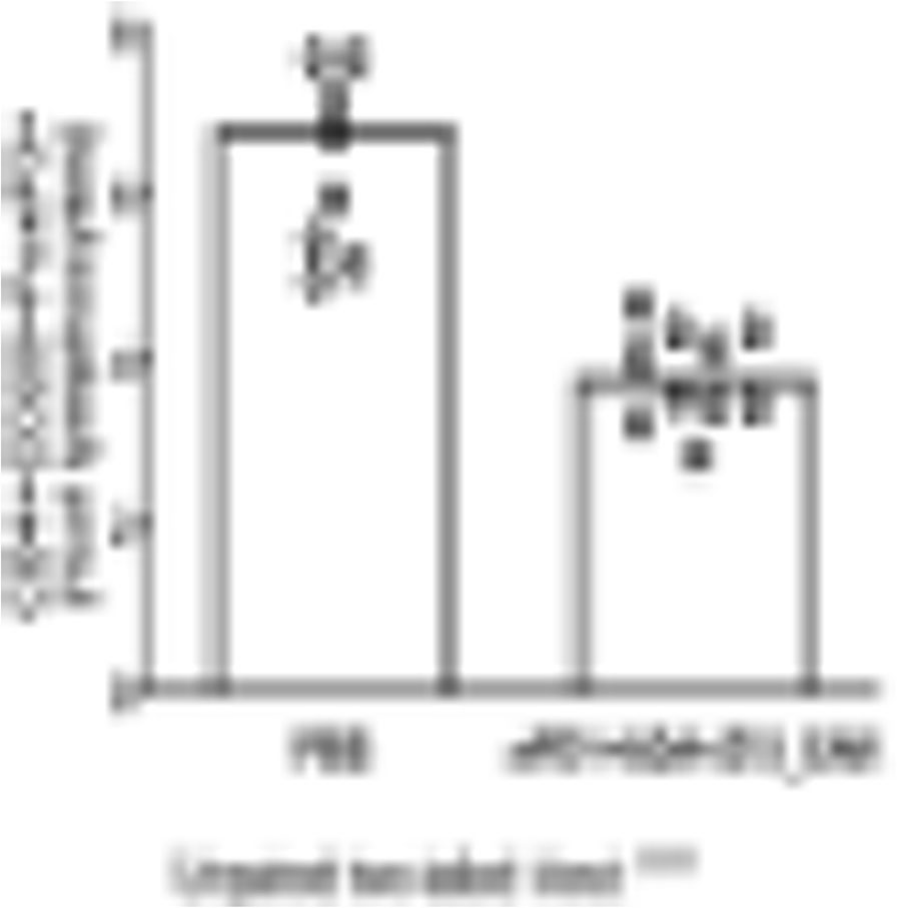
See text for description

#### P260 Characterization of AB154, a humanized, non-depleting α-TIGIT antibody undergoing clinical evaluation in subjects with advanced solid tumors

##### Alejandra Lopez, BSc, Joanne Tan, PhD, Amy Anderson, PhD, Akshata Udyavar, PhD, Nell Narasappa, MSC, Susan Lee, PhD, Daniel DiRenzo, PhD, Kristen Zhang, BS, Hema Singh, Sharon Zhao, Kimberline Gerrick, Adam Park, Lisa Seitz, MA, Nigel Walker, PhD, Matthew Walters, PhD

###### Arcus Biosciences, Inc., Hayward, CA, United States

####### **Correspondence:** Joanne Tan (jtan@arcusbio.com)

**Background**

TIGIT (T-cell immunoreceptor with Ig and ITIM domains) is an inhibitory receptor expressed on natural killer (NK) cells, CD8+ T cells, CD4+ T cells and regulatory T cells (Treg). CD226 is an activating receptor found on NK cells, monocytes and a subset of T cells. TIGIT and CD226 are paired receptors that compete for shared ligands CD155 and CD112, which are expressed by cancer and antigen-presenting cells. Binding of CD155 to TIGIT results in immune suppression, whereas binding of the same ligand to CD226 promotes immune activation. AB154, designed to lack FcɣR binding, blocks human TIGIT with minimal risk of depleting intra-tumoral antigen-experienced CD8+ T cells.

**Methods**

Translational studies quantifying TIGIT, CD226 and CD155 expression in various tumor types and normal tissues were performed using flow cytometry, immunohistochemistry (IHC) and by mining publicly available RNASeq datasets. TIGIT occupancy (RO) and Ki-67 levels from Ph1 dose escalation cohorts were quantified by flow cytometry. Downstream transcriptional effects of TIGIT/CD155 interaction in CD8+ T cells and Treg were assessed using Nanostring®. AB154, AB154 modified to restore wild-type (wt) IgG1 effector function or to display enhance FcɣR binding via Fc mutations, were used in functional assays and antibody-dependent cell cytotoxicity (ADCC) studies.

**Results**

AB154, regardless of IgG1 variant, effectively abrogated the TIGIT-mediated inhibitory effects on activated T cells. In contrast, only non-depleting AB154 lacked ADCC activity in mixed cultures containing NK cells and activated T cells. Data assembled from TCGA, confirmed by flow cytometry as well as IHC, identified multiple tumor types bearing high TIGIT and CD155 expression. In particular, antigen-experienced T cells isolated from late stage head and neck squamous cell carcinoma tumors express higher levels of TIGIT and PD-1 than of CD226. Levels of TIGIT expression in this subset was equivalent, if not higher, than intra-tumoral Treg. Preliminary results from our Phase-1 dose escalation study demonstrated near complete target engagement by AB154 in T cells, NK cells, and NKT cells, coupled with concomitant increases in Ki-67 expression within the aforementioned subsets.

**Conclusions**

Blockade of multiple immune checkpoint proteins can confer effective and durable responses in the treatment of cancer. The data presented here provide: 1) rationale for clinical development of a non-depleting a-TIGIT blocking antibody (AB154), 2) evidence of AB154-related immune activation in subjects with advanced solid tumors, 3) evidence supporting AB154 as a rational combination partner with a-PD-1 (AB122).

**Trial Registration**

NCT03628677

#### P261 Recruitment of CD103+ DCs via tumor stroma-targeted chemokine delivery enhances efficacy of checkpoint inhibitor immunotherapy

##### John-Michael Williford, PhD, Jun Ishihara, PhD, Ako Ishihara, Aslan Mansurov, BChen, Tiffany Marchell, Melody Swartz, PhD, Jeffrey Hubbell

###### University of Chicago, Chicago, IL, United States

####### **Correspondence:** Jeffrey Hubbell (jhubbell@uchicago.edu)

**Background**

Checkpoint inhibitor antibody (CPI) therapy has demonstrated significant clinical benefit in a number of tumor types. Unfortunately, certain tumor characteristics, such as the lack of immune cell infiltration, often correlate with poor responses to CPI therapy. Studies have identified C-C Motif Chemokine Ligand 4 (CCL4) as a key molecule necessary for the recruitment of cross-presenting, CD103+ dendritic cells (DCs) to the tumor; tumors lacking CCL4 expression exhibit a “cold tumor” phenotype and respond poorly to immunotherapy [1]. Based on these results, we hypothesized that tumor-targeted CCL4 could enhance immune cell infiltration into the tumor and synergize with CPI therapy.

**Methods**

We generated a fusion protein comprised of CCL4 and a collagen binding domain (CBD) derived from von Willebrand factor, a tumor-stroma targeting strategy developed in our lab [2]. Anti-tumor efficacy studies were performed in mouse syngeneic models, including B16F10 melanoma, EMT6 breast cancer, and PyMT breast cancer. Flow cytometry was employed to evaluate the tumor immune infiltrate.

**Results**

Utilizing exposure of collagen in leaky tumor vasculature due to its disordered structure, we observed that intravenous (i.v.) infusion of CBD-CCL4 fusion proteins, but not native CCL4, can enhance infiltration of CD103+ DCs, CD8+ T cells, and natural killer cells and slow B16F10 tumor growth when combined with CPI therapy consisting of anti-cytotoxic T-lymphocyte antigen 4 antibody (CTLA4) + anti-programmed death-ligand 1 antibody (PD-L1) (Figure 1, A-B) Further analysis showed strong correlations between the presence of CD103+ DCs and CD8+ T cells and tumor regression. Similarly, in the EMT6 breast cancer model, tumor-targeted CCL4 in combination with CPI, but not native form CCL4, enhanced recruitment of CD103+ DCs, CD8+ T cells, and led to a reduction in tumor growth. To confirm the importance of CD103+ DCs in mediating anti-tumor responses, we utilized Batf3 knockout mice bearing B16F10 tumors; in this instance, anti-tumor efficacy of CPI + CBD-CCL4 was completely lost. Efficacy studies in PyMT breast cancer models highlighted the therapeutic benefit of CBD-CCL4 delivery (Figure 1C); CPI therapy alone led to complete tumor remission in only 10% of mice, whereas combination therapy of CPI + CBD-CCL4 cured 50% of the treated mice (Figure 1D).

**Conclusions**

These results highlight the utility of recruiting CD103+ DCs to the tumor to improve the efficacy of CPI therapy. This engineered chemokine delivery strategy demonstrates significant translational potential by targeting the tumor stroma following systemic administration.

**References**

1. Spranger S, Gajewski T. Impact of oncogenic pathways on evasion of antitumor immune responses. Nat Rev Cancer. 2018; 18:139-147.

2. Ishihara J et al. Targeted antibody and cytokine cancer immunotherapies through collagen affinity. Sci Transl Med. 2019; 11:eaau3259.

**Fig. 1 (abstract P261).**
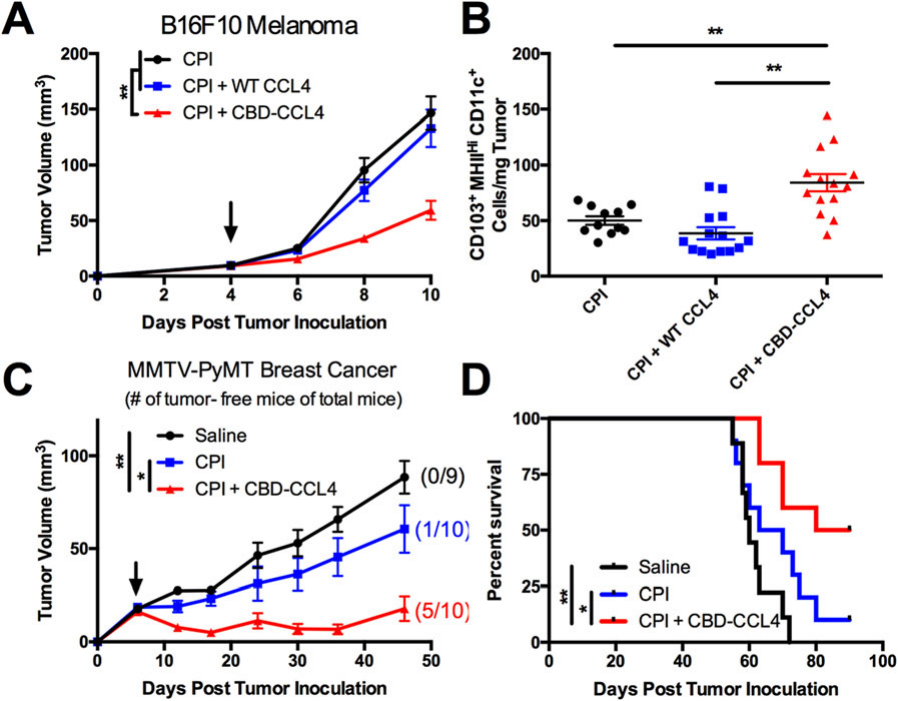
See text for description

#### P262 Schweinfurthins Cause Rapid Induction of Ecto-calreticulin Expression

##### Raymond Hohl, MD, Jeffrey Neighbors, PhD, Ruoheng Zhang, MBBS

###### Penn State College of Medicine, Hershey, PA, United States

####### **Correspondence:** Raymond Hohl (rhohl@pennstatehealth.psu.edu)

**Background**

Our previous study demonstrated that schweinfurthin analogs improve anti-PD-1 immunotherapy in a murine melanoma model by inducing sustainable in vivo anti-tumor immunity.[1] We speculate that the induction of immunogenic cell death (ICD) contributes to these effects. The release of immunogenic danger-associated molecular patterns (DAMPs) from tumor cells during ICD directly activate anti-cancer immunity.[2] Cell surface exposure of calreticulin (ecto-CRT) is the major determinative DAMP because it stimulates cancer cell phagocytosis by dendritic cells and further activates anti-cancer immunity.[3] Phosphorylation of eukaryotic translation inhibition factor eIF2α, an indicator of ER stress, is highly correlated to CRT exposure on the cell surface in some forms of ICD.[4] Previous studies of the schweinfurthin family of compounds have shown that they increase the phosphorylation of eIF2α and have complex effects on lipid homeostasis.[5] We hypothesize that schweinfurthin analogs enhance CRT exposure during induction of ICD via eIF2α phosphorylation to improve anti-cancer immunity.

**Methods**

B16.F10 murine metastatic melanoma cells were cultured with increasing concentrations of the schweinfurthin analog, TTI-3114 or vehicle for 24 hours, followed by measurement of ecto-CRT expression by flow cytometry. Kinetics of ecto-CRT exposure was also assessed. To determine the importance of lipids in this process, studies were carried out with normal or charcoal-stripped lipid-free media. In addition, the role of eIF2α phosphorylation in TTI-3114-induced CRT exposure was evaluated using western blots for total and phosphorylated eIF2α with thapsigargin acting as a positive control for the induction of ER stress.

**Results**

- TTI-3114 induces rapid surface calreticulin exposure on B16.F10 murine melanoma cells in a concentration-dependent manner, starting at 30nM.
- Lipid depletion sensitizes melanoma cells to TTI-3114-induced calreticulin exposure.
- TTI-3114 causes eIF2α phosphorylation before CRT exposure in melanoma cells, indicating potential ER stress response.

**Conclusions**

Based on the above results, we hypothesize that schweinfurthins induce CRT exposure on the cell surface likely by promoting a form of ER-stress, and this effect is augmented by lipid depletion. Future experiments will investigate the function of various lipids in schweinfurthin-induced CRT exposure and subsequent immunogenic cell death. We will also explore the exact mechanisms which are responsible for the CRT cell surface exposure phenomena.

**References**

1. Kokolus, K. M. et al. Schweinfurthin natural products induce regression of murine melanoma and pair with anti-PD-1 therapy to facilitate durable tumor immunity. Oncoimmunology 8, 1–13 (2019).

2. Zhou, J. et al. Immunogenic cell death in cancer therapy: Present and emerging inducers. J. Cell. Mol. Med. 4854–4865 (2019). doi:10.1111/jcmm.14356

3. Obeid, M. et al. Calreticulin exposure dictates the immunogenicity of cancer cell death. Nat. Med. 13, 54–61 (2007).

4. Bezu, L. et al. eIF2α phosphorylation: A hallmark of immunogenic cell death. Oncoimmunology 7, 1–3 (2018).

5. Kuder, C. H. et al. Functional Evaluation of a Fluorescent Schweinfurthin: Mechanism of Cytotoxicity and Intracellular Quantification. Mol. Pharmacol. 82, 9–16 (2012).

#### P263 Targeting EZH2 enhances antigen presentation, antitumor immunity and circumvents anti-PD-1 resistance in head and neck cancer

##### Liye Zhou, PhD^1^, Ravindra Uppaluri, MD, PhD^2^, Tenny Mudianto, BS^1^, Xiaojing Ma, PhD^1^, Rachel Riley, BS^1^

###### ^1^Dana-Farber Cancer Institute, Boston, MA, United States; ^2^Brigham and Women's Hospital/DFCI, Boston, MA, United States

####### **Correspondence:** Ravindra Uppaluri (ravindra_uppaluri@dfci.harvard.edu)

**Background**

Anti-programmed death-1 (PD-1) receptor-based therapeutics improve survival in recurrent head and neck squamous cell carcinoma (HNSCC) patients but many do not benefit due to a low response rate. Multiple mechanisms of immunoevasion have been identified in HNSCCs including in the antigen presentation machinery. Herein, we identified enhancer of zeste homolog 2 (EZH2) as a therapeutic target in HNSCCs that enhanced tumor cell antigen presentation and subsequently sensitized resistant tumors to anti-PD-1 therapy.

**Methods**

EZH2 regulation of antigen presentation was defined using EZH2 inhibitors (GSK126 and EPZ6438) in human and mouse HNSCC cell lines. Mechanistic dissection of EZH2 in regulation of antigen presentation was investigated using flow cytometry, qRT-PCR, ELISA and chromatin-immunoprecipitation assays. EZH2 deficient cell lines were generated using CRISPR-CAS9. GSK126 and anti-PD-1 blocking antibody were used in testing combinatorial therapy in vivo.

**Results**

EZH2 expression was negatively correlated with antigen processing machinery (APM) pathway components in HNSCC TCGA datasets. EZH2 inhibition resulted in significant upregulation of MHC class I expression in both human and mouse HNSCC lines and increased antigen presentation in mouse models. This increased antigen presentation on the tumor cell by EZH2 inhibitors or CRISPR mediated EZH2 deficiency, increased antigen specific CD8+ T cell proliferation, IFNγ production and tumor cell cytotoxicity. Mechanistically, EZH2 inhibition reduced the histone H2K27me3 modification on the β-2-microglobulin promoter to regulate antigen presentation. Finally, in an anti-PD-1 resistant model of HNSCC, combination EZH2 inhibition with anti-PD-1 suppressed tumor growth at least partially due to the upregulation of antigen presentation capacity of tumor cells.

**Conclusions**

Our results demonstrated that targeting EZH2 enhanced antigen presentation and circumvented anti-PD-1 resistance. Thus, combining EZH2 targeting with anti-PD-1 may increase therapeutic susceptibility in HNSCC.

#### P264 Characterization of a human CD137 (4-1BB) receptor binding monoclonal antibody with differential agonist properties that promotes antitumor immunity

##### Helen Kotanides, Rose Marie Sattler, Maria Lebron, Carmine Carpenito, PhD, Juqun Shen, Jingxing Li, David Surguladze, Jaafar Haidar, Colleen Burns, Leyi Shen, Ivan Inigo, BS, Anthony Pennello, Amelie Forest, MSc, Xinlei Chen, Darin Chin, Andreas Sonyi, Michael Topper, Lauren Boucher, Prachi Sharma, Yiwei Zhang, Douglas Burtrum, Ruslan Novosiadly, Dale Ludwig, Gregory Plowman, Michael Kalos

###### Eli Lilly and Company, Indianapolis, IN, United States

####### **Correspondence:** Helen Kotanides (helen.kotanides@lilly.com)

**Background**

CD137 (4-1BB) is a member of the TNFR receptor superfamily that plays a key role in mediating immune response through costimulatory signals that promote T cell proliferation, survival and memory. CD137 agonism has the potential to reinvigorate potent antitumor immunity either alone or in combination with other immune checkpoint therapies. We hypothesized that an antibody with a unique binding mode could activate T cells in an Fc effector-less format. Hence, we developed 7A5, a CD137 agonist monoclonal antibody which potentially has a ligand-like structural binding mode and demonstrated that it effectively engages the CD137 receptor in preclinical studies.

**Methods**

7A5 was identified from a human Fab phage display library screen and engineered to an IgG1 Fc effector null antibody. Solid phase binding assays with recombinant CD137 protein and cell-based assays in CD137 expressing cells were used to evaluate binding and functional activity in vitro. To assess agonist activity, 7A5 was tested in NF-kB luciferase reporter, PBMC co-stimulation and Treg suppression assays. To determine antitumor activity in vivo, human tumor xenograft mouse models (NSG mice harboring human NCI-H292 or HCC827 NSCLC tumors) reconstituted with human PBMCs or T cells, were used. Treatments included 7A5 monotherapy and the combination with anti-PD-L1 antibody in these models.

**Results**

In this study, we characterized 7A5, a fully human IgG1 Fc effector null monoclonal antibody. We showed 7A5 binds CD137 and the binding epitope overlaps with the CD137 ligand binding site. 7A5 engages the CD137 receptor and activates signaling independent of cross-linking or Fc effector function. It binds to activated primary T cells and leads to T cell stimulation in cell-based assays. Monotherapy with 7A5 inhibits tumor growth in humanized mouse models and this activity is enhanced when combined with a PD-L1 antagonist antibody. Furthermore, changes to the intra-tumoral immune gene expression signature in response to 7A5 is highly suggestive for a mechanism of enhanced T cell infiltration and activation.

**Conclusions**

In summary, CD137 antibody 7A5 represents a differentiated agonist with preclinical biological properties that support its further development as an anti-cancer immunotherapy.

#### P265 Immune checkpoint inhibitors induce response in a dose-dependent manner while their immune related adverse events are dose-independent, a meta-analysis

##### Osama Rahma, MD^1^, Joshua Reuss, MD^2^, Anita Giobbie-Hurder, MS^1^, Ghazaleh Razavi, MD^3^, Pooja Mehra, MD^4^, Seema Gupta^5^, Rawad Elias, MD^6^, Samir Khleif, MD^5^

###### ^1^Dana-Farber Cancer Institute, Boston, MA, United States; ^2^John Hopkins, Baltimore, MD, United States; ^3^Georgia Cancer Center, Augusta, GA, United States; ^4^University of Virginia, Charlottesville, VA, United States; ^5^Georgetown University, Washington, DC, United States; ^6^Hartford Healthcare, Hartford, CT, United States

####### **Correspondence:** Samir Khleif (snk48@georgetown.edu)

**Background**

Despite the expansion of Immune Checkpoint Inhibitor (ICI) indications, the relationship between ICI dose-escalation and toxicity or response has not been established. To understand this correlation, we performed a meta-analysis of all available clinical trials investigating ICIs.

**Methods**

We searched PubMed and abstracts presented at (inter)national meetings for trials (T) using FDA-approved ICIs including ipilimumab, atezolizumab, nivolumab, and pembrolizumab. The reported rates of treatment-related grade 3-5 adverse events (G3-5AEs), immune-related adverse events (irAEs), and response were collected. For each ICI, comparisons of incidence rates between doses or diseases were based on marginal, exact generalized linear models.

**Results**

A total of 74T (7469 patients (pts)) published between 1/2010 – 1/2017 were included (15T-ipilimumab (1058 pts), 30T-nivolumab (2281 pts), 29T-pembrolizumab (4130 pts)) (Figure 1). For ipilimumab, the overall incidence of G3-5AEs was 34%. A significant 27% reduced risk of G3-5AEs was seen with 3 mg/kg compared to 10 mg/kg (p=0.002) (Figure 2). However, there was no relationship observed between dose of ipilimumab and incidence of irAEs or response to therapy (Figure 3). With nivolumab, the overall incidence of G3-5AEs was 20.1%. Incidence of G3-5AEs was significantly lower in NSCLC, with risk reductions of 24-38% when compared to RCC or melanoma (p≤0.05). No dose-toxicity relationship was seen for G3-5AEs or irAEs (Figure 4). In both melanoma (6T) and NSCLC (7T), a dose-response association was observed, with significantly decreased odds of response of 17% and 64% for 1mg/kg compared to 3mg/kg in melanoma and NSCLC, respectively (Figure 5,6) with no further increase in response for doses above 3 mg/kg. This association was not observed in RCC (Figure 7). For pembrolizumab, the overall incidence of G3-5 AEs was 13.3%. Risk of G3-5AEs was 17% lower in melanoma than in NSCLC (p=0.03). No dose-toxicity relationship was seen for G3-5AEs or irAEs (Figure 8). In melanoma (7T), 2mg/kg every 3 weeks (q3w) had 22% decreased odds of response compared to 10mg/kg (q2w) (p=0.01) (Figure 9). For NSCLC (5T), no dose-response relationship was noted (Figure 10).

**Conclusions**

We found no correlation between dose of ipilimumab and odds of G3-5iAEs or response. For pembrolizumab and nivolumab, no dose-toxicity correlation was seen but a dose-response correlation was observed suggesting that, for the PD-1 inhibitors, efficacy appears to be dose-dependent while toxicity does not. Accordingly, future clinical trial design of ICIs should use a dose escalation method with a primary objective of identifying an effective dose rather than a maximum tolerated dose.

**Acknowledgements**

Merck and BMS for providing input

**Fig. 1 (abstract P265).**
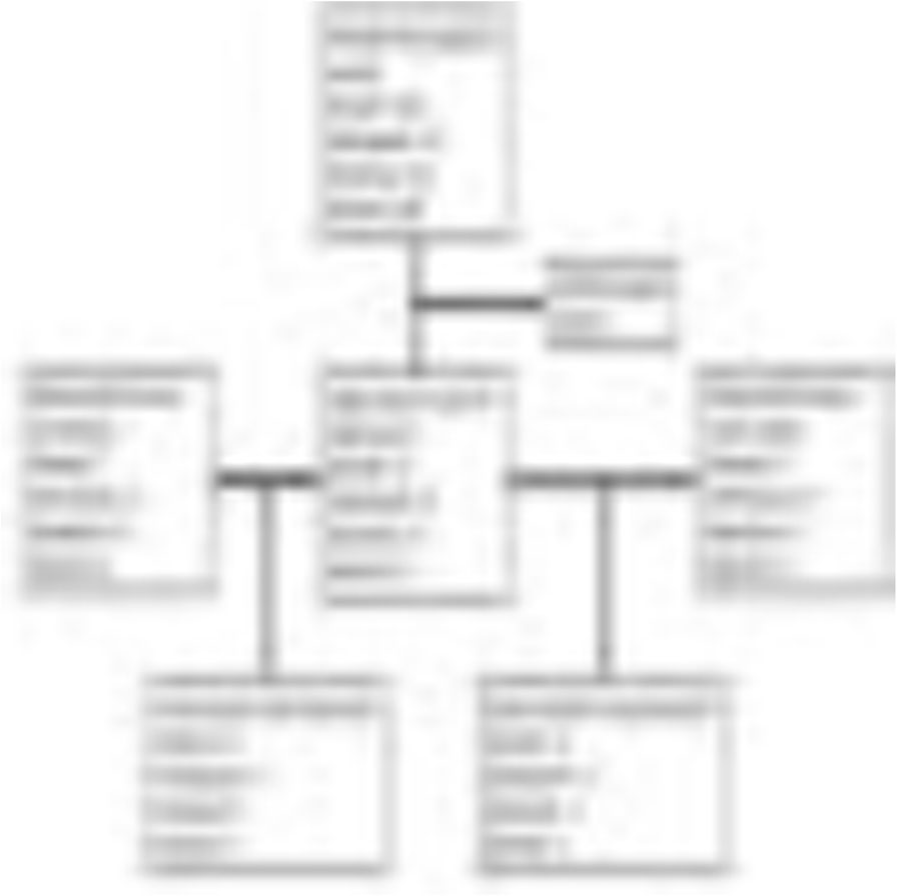
Consort Diagram of Literature Search

**Fig. 2 (abstract P265).**
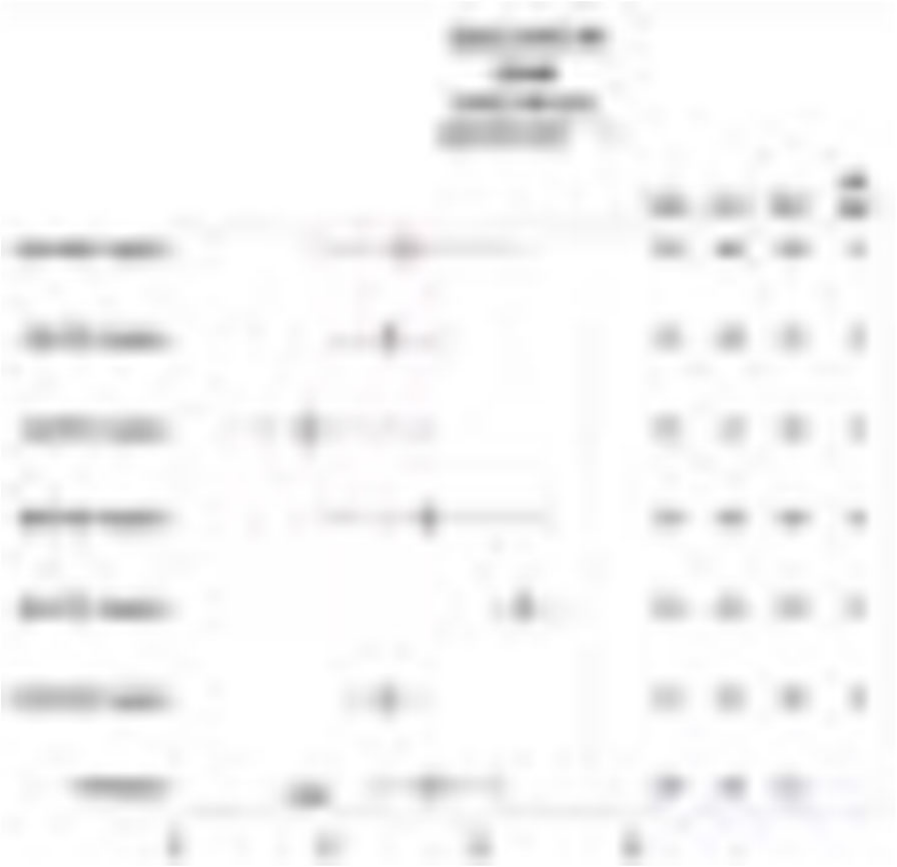
Bootstrap analysis for G3-4 AEs of ipilimumab

**Fig. 3 (abstract P265).**
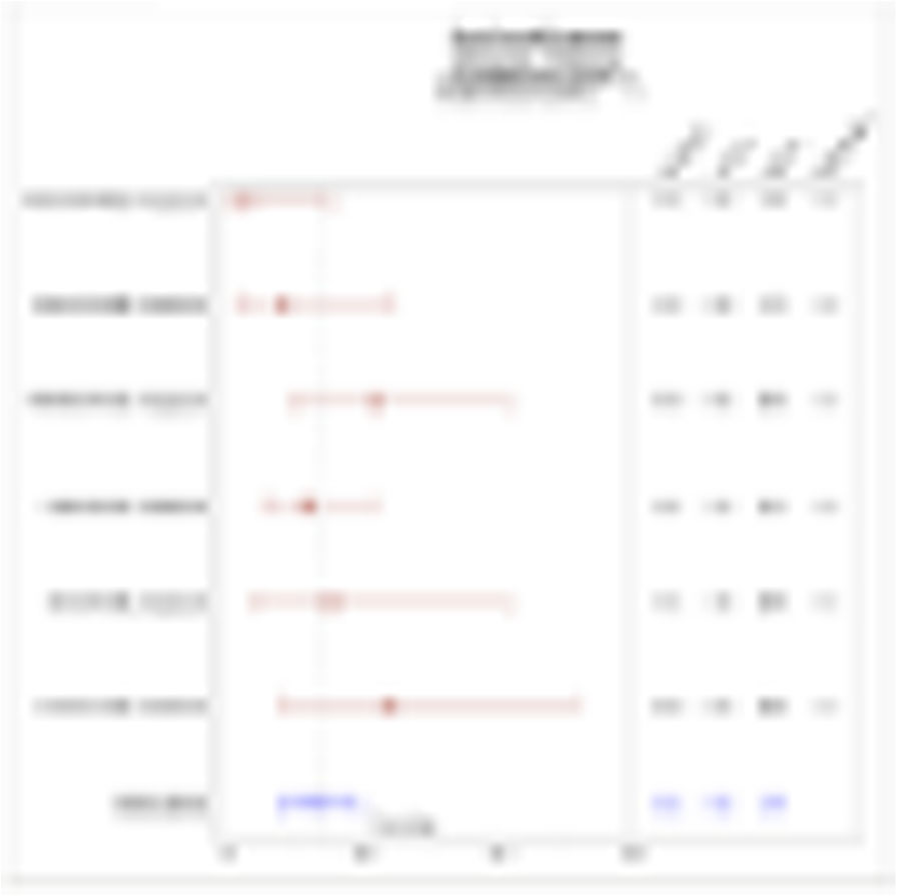
Bootstrap analysis for ORR for ipilimumab

**Fig. 4 (abstract P265).**
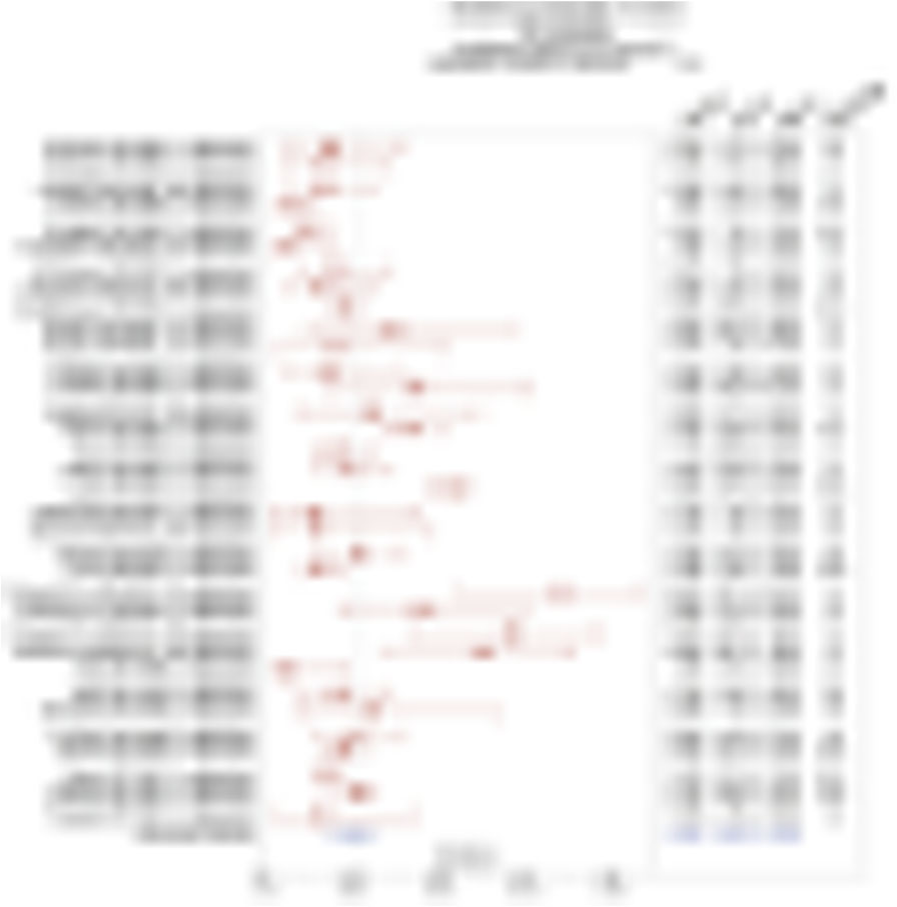
Bootstrap analysis for G3-4 AEs for nivolumab

**Fig. 5 (abstract P265).**
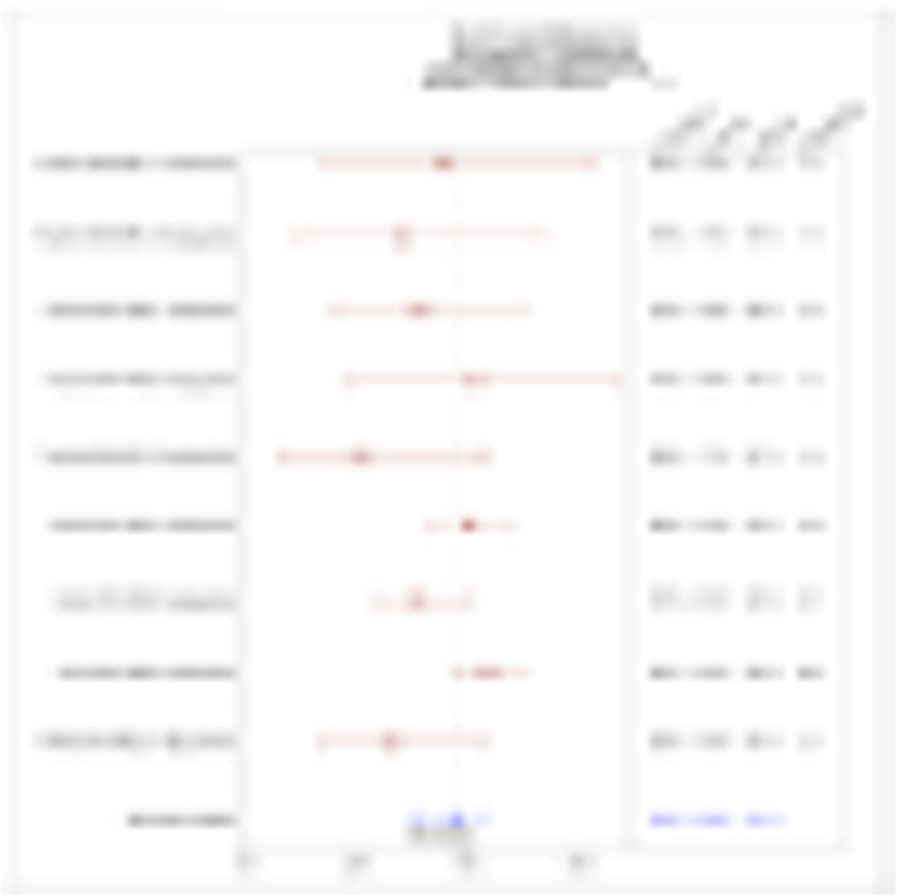
Bootstrap analysis for ORR for nivo in melanoma

**Fig. 6 (abstract P265).**
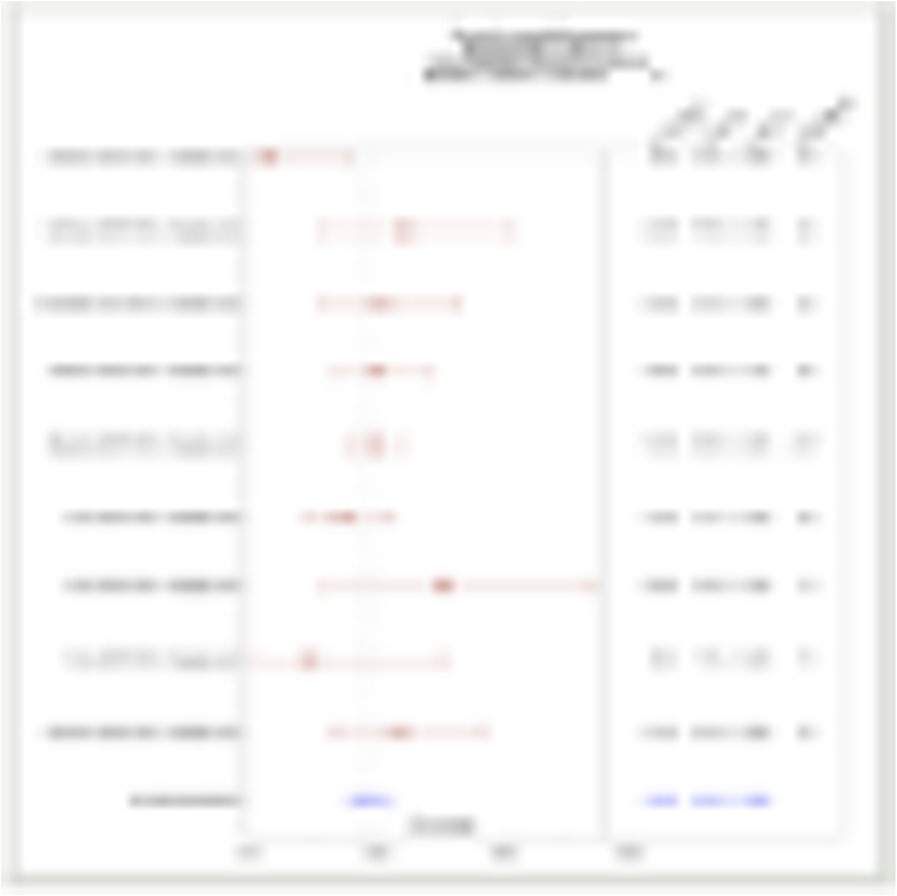
Bootstrap analysis for ORR for nivolumab in NSCLC

**Fig. 7 (abstract P265).**
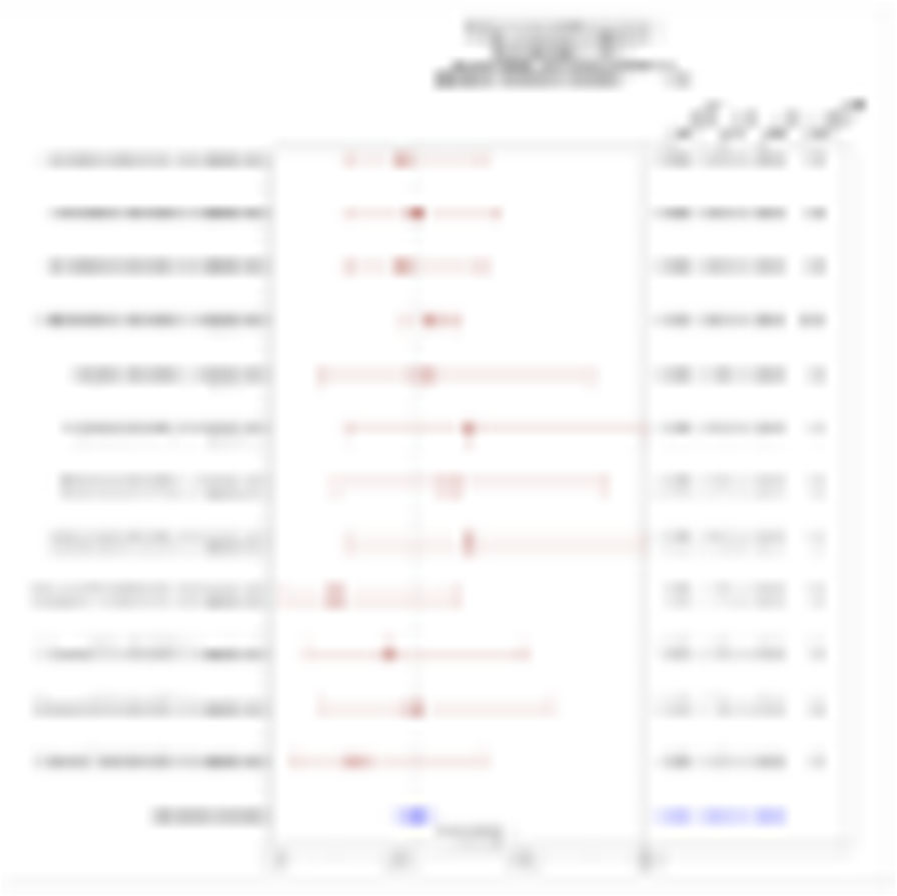
Bootstrap analysis for ORR for nivolumab in RCC

**Fig. 8 (abstract P265).**
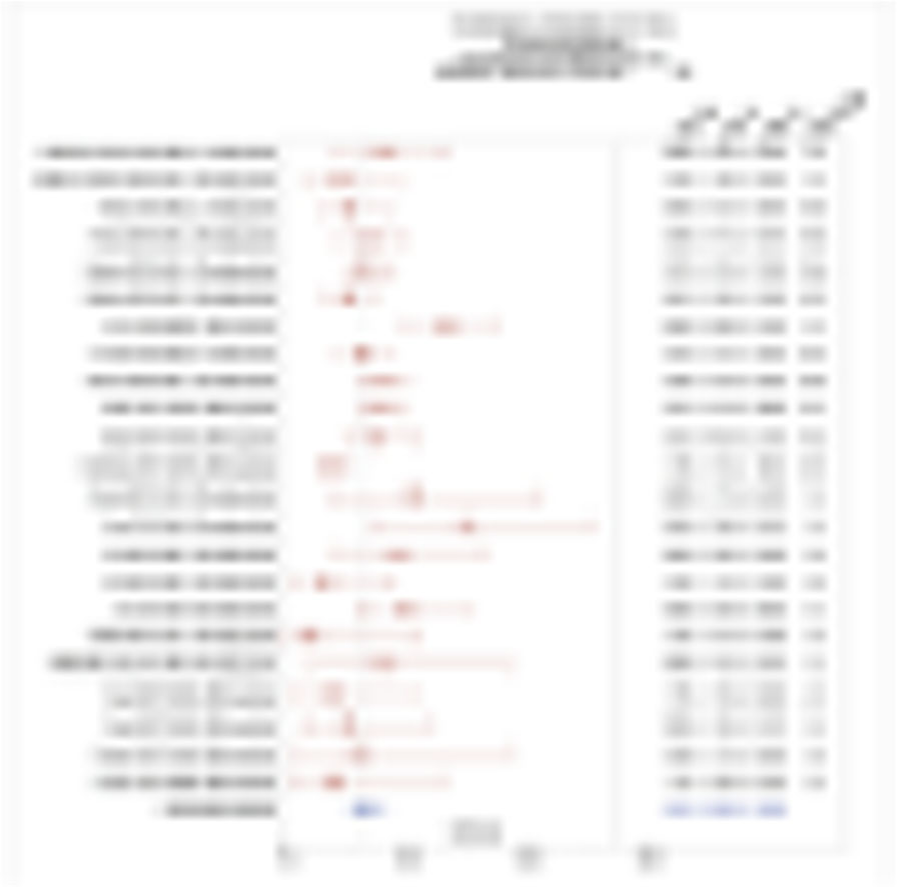
Bootstrap for incidence of G3-4 AE in pembrolizumab

**Fig. 9 (abstract P265).**
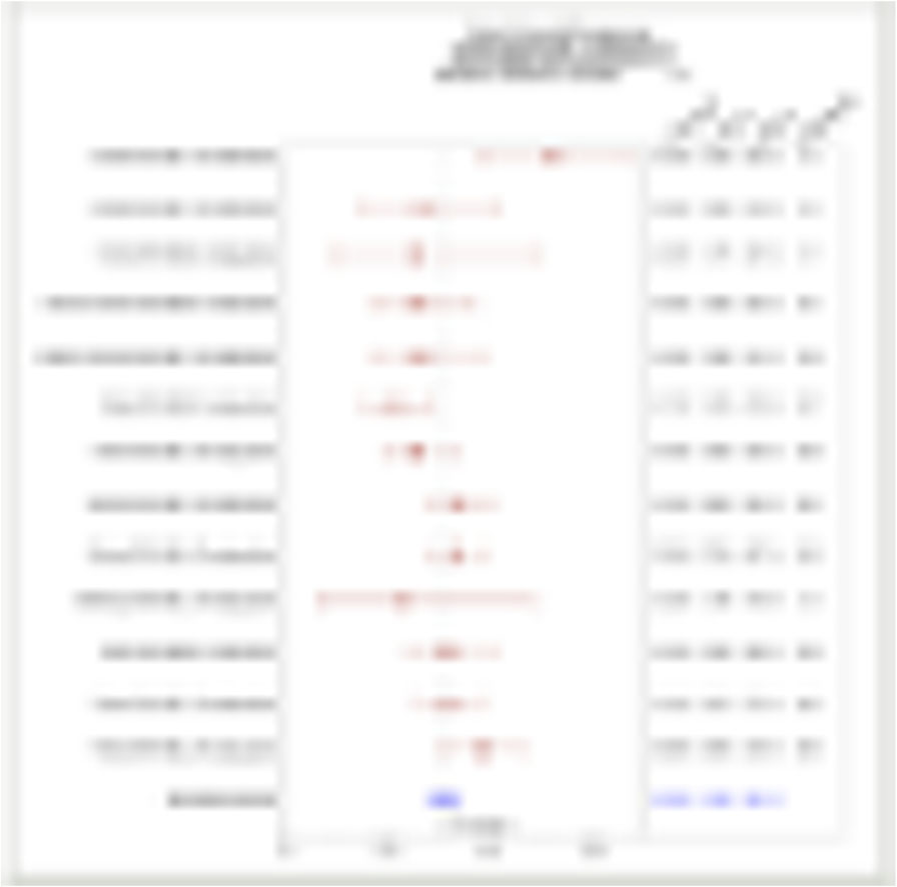
Bootstrap for ORR for pembrolizumab in melanoma

**Fig. 10 (abstract P265).**
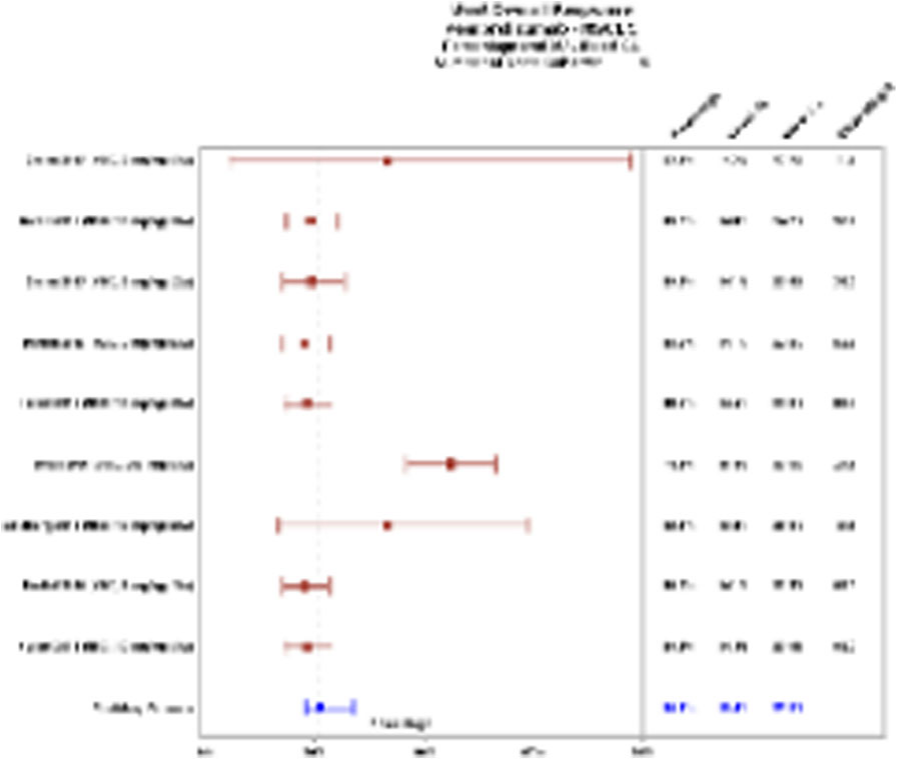
Bootstrap for ORR for pembrolizumab in NSCLC

#### P266 Evaluation of immunomodulatory receptor/ligand expression on matched human biospecimens

##### Shawn Fahl (shawn.fahl@dls.com)

###### Discovery Life Sciences, Huntsville, AL, United States

**Background**

The integration of immunomodulatory receptor signaling is crucial for the activation status of responding T cells, and modulation of these receptors, and their ligands, may be of therapeutic benefit. Indeed, recent breakthroughs in checkpoint inhibitor therapies, and in particular those that target the PDL1/PD1 interaction, have demonstrated success in numerous oncological indications. Understanding the expression of these receptors and their cognate ligands within the complex cellular architecture of solid tumors will be fundamentally important to the design on the next-generation of immunotherapies.

**Methods**

Bulk RNASeq analysis of primary human tumor tissue revealed the expression of numerous co-stimulatory (LIGHT/HVEM, 41BB/41BBL, OX40/OX40L, GITR/GITRL) and co-inhibitory (Lag3, VISTA, PVR/PVRL2/TIGIT, Tim3/Galectin-9) receptors and ligands within the tumor microenvironment. Using multiparametric flow cytometry, we have profiled the expression of these immunomodulatory receptors and their respective ligands on the major cellular components of the tumor microenvironment and correlated it with expression on cellular subsets within matched peripheral blood.

#### P267 Phenotyping of TIGIT pathway members may be used for cancer selection in the clinical application of anti-TIGIT antibody EOS884448

##### Noemie Wald, PhD, Julia Cuende, PhD, Marjorie Mercier, Florence Nyawouame, MSc, Margreet Brouwer, MSc, Erica Houthuys, PhD, Gregory Driessens, PhD, Veronique Bodo, PhD, Catherine Hoofd

###### iTeos Therapeutics, Gosselies, Belgium

####### **Correspondence:** Gregory Driessens (gregory.driessens@iteostherapeutics.com)

**Background**

TIGIT is a T cell co-inhibitory receptor that drives tumor cell mediated immunosuppression. Predominantly expressed on CD4+ Tregs, CD8+ T and NK cells in healthy individuals, TIGIT is further upregulated in these cells in cancer patients. In patients, it is frequently co-expressed with exhaustion markers such as PD-1. DNAM-1/CD226, a co-stimulatory receptor, is expressed on NK and T cells and competes with TIGIT for PVR/CD155 binding, but with a lower affinity. Since cancer cells express high level of CD155 and because TIGIT expression is increased on TILs, the TIGIT pathway represents a major mechanism for immunosuppression within the tumor. We developed EOS884448, an antagonist anti-TIGIT antibody, to prevent TIGIT-mediated immunosuppression in cancer patients.

**Methods**

To support selection of indications for clinical application of EOS884448, we used flow cytometry and immunohistochemistry (IHC) to characterize peripheral and tumoral expression of TIGIT, CD155 and CD226 in healthy or cancer donors.

**Results**

TIGIT is expressed on multiple immune subsets in healthy donors. Similar analysis on matched PBMCs and TILs from 15 cancer donors highlighted the overexpression of TIGIT on cells from those samples. Interestingly, ex vivo polyfunctional analysis of cytokine production demonstrated immunosuppression of TIGIT+ TILs versus their TIGIT- counterparts. Among PBMCs and TILs assessed by flow cytometry, tumor-infiltrating Tregs exhibit the highest TIGIT expression (frequency of positive cells and receptor density). This finding was confirmed by IHC on tumor samples, supporting the potential value of an ADCC-competent antibody targeting preferentially tumor-infiltrating Tregs. Finally, intrinsic expression of TIGIT on tumor cells was detected on several haematological malignancies, opening the potential for EOS8844488 to directly kill tumor cells in addition to its activities to reinvigorate immunity. The expression of the TIGIT ligands CD155 and CD226 co-receptor were also analysed by IHC in tissues (n=284-307) from 9 cancer indications. CD155 is mostly expressed by tumor cells, ranging from a median of 2% of CD155high tumor cells for cervix to 50% for pancreatic cancer. CD155 expression is highest in pancreatic, prostate, kidney, gastric and colon cancers. CD226 is detected on immune cells infiltrating tumors. The median percentage of tissue area positive for CD226 ranged from 0.07% in head and neck to 0.98% in gastric cancer, with gastric, lung and renal cancer showing the highest CD226 expression.

**Conclusions**

Together, these findings strongly support the relevance of targeting TIGIT with an ADCC-competent antibody and provide a method to select cancer types that may benefit from treatment with EOS884448.

**Ethics Approval**

The study was approved by UCL‘s Ethics Board, approval number Biobank2019/09MAI/005

#### P268 IPH5401 anti-human C5aR antibody targets suppressive myeloid cells in the TME

##### Joanna Fares, Léa Simon, Caroline Soulas, Marion Loivet, Elodie Bonnet, Luciana Batista, Romain Remark, PhD, Cécile Bonnafous, Robert Zerbib, MSc, Mathieu Bléry

###### Innate Pharma, Marseille, France

####### **Correspondence:** Robert Zerbib (robert.zerbib@innate-pharma.fr)

**Background**

A hallmark mechanism of synergy in immunotherapy is the elimination of immunosuppressive cells, such as myeloid cells and neutrophils, to allow for the reactivation of effector cells. These immunosuppressive cells are indeed associated with poor prognosis in many cancer types as well as resistance to checkpoint blockade. From a therapy perspective, we aimed to specifically target the recruitment of these major mediators of pro-tumoral inflammation into the tumor microenvironment (TME).

The complement system consists of a network of more than 50 different plasma and membrane associated proteins. It is a part of the innate immune system and plays a key role in host defense against pathogens as well as in tissue homeostasis. The anaphylatoxin C5a is formed upon cleavage of C5 during the process of complement activation. C5a is the most potent chemoattractant and induces recruitment and activation of different immune cells to inflamed tissue, among which are neutrophils, eosinophils, monocytes, basophils, and mast cells. C5a binds to the seven transmembrane-spanning receptors C5aR1 (CD88) and C5aR2 (C5L2). Blocking C5aR1 thus appears as a potent mean to control the myeloid suppressive cells in the TME. In this context we developed IPH5401, a fully human blocking anti-C5aR1 monoclonal antibody that prevents binding to C5a.

**Results**

We first explore further the expression profile of C5aR1 in the TME both at mRNA and protein levels in several solid cancer indications showing various levels of infiltration by C5aR1 positive immune cells. Then, we demonstrated that IPH5401 can block activation and migration of Human neutrophils and macrophages in vitro.

**Conclusions**

Altogether, these results support our ongoing multi-center, open label, dose-escalation and dose expansion Phase I/II clinical trial (STELLAR-001) evaluating the safety and efficacy of IPH5401 in combination with durvalumab, an anti-PD-L1 immune checkpoint inhibitor, as a treatment for patients with advanced solid tumors.

#### P269 IL-15 together with TIGIT blockade reverses PVR-mediated NK cell dysfunction in melanoma

##### Joe-Marc Chauvin, PhD^1^, Mignane Ka^1^, Ornella Pagliano^1^, Carmine Menna^1^, Quanquan Ding^1^, Cindy Sander^1^, Jiajie Hou^2^, Soldano Ferrone, MD, PhD^1^, Diwakar Davar, MD^1^, John Kirkwood, MD^1^, Robert Johnston, PhD^3^, Alan Korman, PhD^3^, Mark Smyth^2^, Hassane Zarour, MD^1^

###### ^1^University of Pittsburgh, Pittsburgh, PA, United States; ^2^QIMR Berghofer Medical Research Institute, Queensland, Australia; ^3^Bristol-Myers Squibb, Redwood City, CA, United States

####### **Correspondence:** Joe-Marc Chauvin (chauvinj@upmc.edu)

**Background**

Natural Killer cells (NKs) play a critical role in tumor immunosurveillance. Multiple activating and inhibitory receptors regulate NK cell-mediated tumor cytotoxicity. The inhibitory receptor TIGIT and its counter-receptor CD226 exert opposite effects on NK cell function, with TIGIT blockade reinvigorating NK cell-mediated tumor reactivity. Whether and how the manipulation of the TIGIT/CD226/PVR axis may reactivate NK-cell mediated antitumor activity in melanoma patients (MPs) has not yet been thoroughly evaluated.

**Methods**

Flow cytometry was used to evaluate the phenotype and function of NKs in the periphery (cNKs) and tumor sites (TiNKs) in MPs. CD226 mRNA was evaluated by RT-PCR. CD226 and TIGIT internalization was evaluated on isolated cNKs by Imagestream analysis. Melanoma lung metastasis tumor models in WT and TIGIT-/- mice were used to evaluate in vivo effects of TIGIT and CD226 blockades on NK-mediated tumor control with or without IL-15 treatment.

**Results**

In sharp contrast with CD8+ T cells, TiNKs downregulated both TIGIT and CD226 expression as compared to cNKs. TiNKs exhibited decreased expression of activation markers, lytic potential and melanoma killing capacity as compared to cNKs in MPs. Membrane-bound PVR, but not soluble PVR, triggered CD226 internalization and degradation, leading to NK dysfunction. IL-15 stimulation increased CD226 and TIGIT expression levels and NK cell function, and TIGIT blockade further increased TiNK proliferation and function against MHC class-I deficient tumors as compared to IL-15 or TIGIT blockade alone. TIGIT blockade promotes tumor antigen-specific CD8+ T cells proliferation independently of NK cells. TIGIT blockade impeded metastasis in mice in a CD226-dependent manner and only in presence of IL-15.

**Conclusions**

Membrane-bound PVR plays a critical role in the tumor microenvironment by modulating TIGIT/CD226 expression and the function of TiNKs. IL-15 together with TIGIT blockade, counteracts PVR-mediated TiNK dysfunction in melanoma, and prevent metastasis occurrence in mice in a CD226 dependent manner. Our findings support the development of novel combinatorial immunotherapy with IL-15 and TIGIT blockade to promote NK cell-mediated destruction of MHC class I-deficient melanoma, which are refractory to CD8+ T cell-mediated immunity and PD-1 blockade.

#### P270 Expression and clinical significance of the CD47/SIRPα pathway as a candidate immunotherapy target in non-small cell lung cancer (NSCLC)

##### Shruti Desai, PhD^1^, Franz Villarroel-Espindola^1^, Patricia Gaule, PhD^1^, Adam Ducler^1^, Marisa Peluso, MS^2^, Benjamin Lee, MD PhD^2^, Kurt Schalper, MD, PhD^1^, Pamela Holland, PhD^2^

###### ^1^School of Medicine, Yale University, New Haven, CT, United States; ^2^Surface Oncology, Cambridge, United States

####### **Correspondence:** Kurt Schalper (kurt.schalper@yale.edu)

**Background**

Immunostimulatory therapies have revolutionized the treatment of NSCLC. Multiple studies show that activation of the CD47/SIRPα pathway can mediate cancer immune evasion by blocking phagocytic activity of macrophages. Although early stage clinical trials blocking this pathway are ongoing, the expression, tissue distribution and clinical significance of the CD47 axis in NSCLC remains unknown.

**Methods**

Using control tissue samples/cell line transfectants, we validated antibodies to reliably detect CD47 and SIRPα protein in FFPE tissue and standardized a multiplexed quantitative immunofluorescence panel for simultaneous measurement of DAPI, pan cytokeratin, CD8/CD47/SIRPα. We used this panel to interrogate two retrospective NSCLC Yale cohorts represented in tissue microarrays (#1: n=297 and #2: n=175). Cohort #3, n=139 adenocarcinomas with mutation testing were also analyzed. We studied the levels of the targets, tissue distribution, association with clinicopathologic/molecular variables and survival.

**Results**

Predominant tumor CD47 expression (cytoplasmic/membranous) was recognized in 82 -88% of cases in the NSCLC cohorts. SIRPα protein was detected in 94- 98% of cases located in the stroma. Elevated expression of CD47/SIRPα was significantly associated with high CD8+ tumor infiltrating lymphocytes in the cohorts. The targets showed no consistent associations between major clinicopathologic variables. Lung adenocarcinomas with KRAS mutation showed significantly lower levels of CD47 than EGFR mutated or EGFR/KRAS WT tumors. Although individual CD47/SIRPα levels were not prominently associated with outcome, their co-localization in stroma was positively associated with longer 5-year overall survival.

**Conclusions**

CD47 and SIRPα are frequently expressed in NSCLCs with higher levels in CD8+/T-cell inflamed tumors, suggesting adaptive upregulation of this pathway associated with anti-tumor immune pressure. CD47 levels are associated with major oncogenic signaling events in lung adenocarcinomas. Localized measurement of stromal CD47/SIRPα co-expression in intact tumor specimens could provide valuable information about the pathway activation.

**Ethics Approval**

All tissues were used after approval from the Yale Human Investigation committee protocol #9505008219 which approved the patient consent forms or waiver of consent.

#### P271 Anti-SIRPalpha antibodies exert anti-tumor activity in both CD47-dependent and CD47-independent manners

##### Hongtao Lu, PhD, Xiaofeng Niu, PhD, Qinglin Du, PhD, Jingfeng Yu, Roumei Xing, Yanfen Hu, Jinfeng Zhao, Fengli Wang, Zhihao Wu, PhD, Yangsheng Qiu, Hongtao Lu, PhD

###### Elpiscience Biopharma, Shanghai, China

####### **Correspondence:** Hongtao Lu (hongtaolu@elpiscience.com)

**Background**

Signal-regulatory protein alpha (SIRPα), is an inhibitory receptor expressed on myeloid cells and dendritic cells. Ligation of CD47 to SIRPα delivers a “don’t eat me” signal to suppress phagocytosis. Tumor cells frequently overexpress CD47 to evade macrophage-mediated destruction. Currently, agents targeting CD47 have proceeded to clinical trials and demonstrated promising anti-tumor results. However, these agents have been associated with hemolytic anemia and thrombocytopenia. In addition, universal expression of CD47 causes antigen sink, which leads to reduced efficacy. We therefore consider targeting SIRPα to achieve an improved efficacy with a better safety profile. We have developed 2 classes of anti-SIRPα antibodies: CD47-SIRPα interaction “blocker” and “non-blocker”. Both groups of antibody functionally stimulate phagocytosis of multiple cancer cell types by macrophages.

**Methods**

Using SIRPα extracellular domain (ECD), SIRPα overexpression stable cell line and plasmid encoding SIRPα as immunogens, anti-SIRPα antibodies were generated by traditional hybridoma technology. Pan-allele/SIRP family homologue binding properties, and species cross-reactivity profile were evaluated by ELISA and FACS. In vitro function activity was determined by phagocytosis assay. In vivo safety profile was assessed in hCD47/hSIRPα double knock-in mice. Lead clone was humanized via CDR grafting and back mutation screening. Stress tests were carried out to evaluate the developability of candidate antibody.

**Results**

A panel of anti-SIRPα antibodies that stimulate phagocytosis of multiple cancer cells were developed. One lead clone (B4) was selected from the “Blocker” group based on ranking of in vitro binding/function properties. Subsequently, ES004-B4 was identified as the candidate antibody after humanization, affinity maturation, and property characterization. It recognized pan-allele human SIRPα with high affinity (KD hSIRPα V1/V2, 0.86nM/1.43nM). ES004-B4 was shown to stimulate phagocytosis of multiple cancer cell types. Another lead antibody (N4) was selected from the CD47/SIRPα interaction “non-blocker” group. ES004-N4 also induced strong phagocytosis of multiple cancer cell types by macrophages, albeit it did not disrupt CD47/SIRPα interaction, suggesting an unique mode of action. ES004-B4 and ES004-N4 didn’t trigger hemagglutination and had no negative impact on T cell proliferation in vitro. No severe hemolytic anemia and thrombocytopenia were observed in hCD47/hSIRPα double knock-in mice treated with 10mg/kg of each antibody, suggesting a low safety risk in vivo.

**Conclusions**

In summary, we have developed 2 anti-SIRPα antibodies with “Best-in-Class” potential: 1) CD47/SIRPα interaction “Blocker” ES004-B4, 2) CD47/SIRPα interaction “Non-Blocker” ES004-N4. Both antibodies greatly enhance macrophage-mediated tumor cell destruction, possibly through different mechanisms of action. We are currently advancing the development of ES004-B4 and ES004-N4 into clinical candidates.

#### P272 SRF231, a fully human high-affinity anti-CD47 antibody, exerts potent preclinical antitumor activity through engagement of the Fc receptor (FcR), CD32a

##### Marisa Peluso, MS, Kshama Doshi, PhD, Caroline Armet, BS, Li Zhang, Rachel O'Connor, BS, Matthew Rausch, PhD, Jonathan Hill, PhD, Benjamin Lee, MD PhD, Pamela Holland, PhD, Vito Palombella, PhD, Alison Paterson, PhD

###### Surface Oncology, Inc., Cambridge, MA, United States

####### **Correspondence:** Marisa Peluso (mpeluso@surfaceoncology.com)

**Background**

CD47 is a transmembrane protein that acts as a “Don’t Eat Me” signal to evade immune recognition. It is overexpressed in multiple cancer subtypes and is associated with poor prognosis. Several anti-CD47 molecules designed to antagonize the CD47 axis are being tested in the clinic. Preclinical characteristics and antitumor mechanisms of the investigational agent SRF231, a fully human antibody targeting CD47, are described.

**Methods**

SRF231 monovalent affinity and binding properties were evaluated by Surface Plasmon Resonance (SPR) technology and in vitro agglutination assays. Directly labeled SRF231 was used to profile tumor vs normal cell binding. SRF231-mediated antitumor activity was assessed in tumor using macrophage coculture systems designed to evaluate the impact of SRF231 on tumor cell phagocytosis, cell death, and cell depletion. Receptor occupancy (RO)/activity relationships and dependency on FcR were also assessed. A xenograft tumor model was used to characterize the pharmacokinetic (PK)/pharmacodynamic (PD)/tumor exposure/efficacy relationship of SRF231 following single dose administration.

**Results**

SRF231 is a fully human, high-affinity anti-CD47 antibody with a slow off-rate and binding mode that does not lead to agglutination of patient-derived red blood cells or tumor cells. Increased binding to several tumor vs normal cells is observed with SRF231. Analyses probing the relationship between FcR and SRF231 activity revealed that SRF231 leads to antitumor activity through both phagocytosis and cell death mechanisms in a manner largely dependent on the activating receptor FcγRIIa (CD32a), predominantly expressed by myeloid cells. Additionally, SRF231-mediated antitumor activity is retained in longer-term assay conditions and in washout conditions. Moreover, submaximal SRF231 RO is sufficient for maximal phagocytosis induction in vitro. In a B-cell lymphoma xenograft model, a single dose of SRF231/mouse yields tumor stasis out to 21 days with submaximal tumor exposure. Antitumor activity is associated with an increase of host macrophage infiltration and cytokine induction suggestive of an innate immune response.

**Conclusions**

SRF231 is a high affinity, CD47-targeting antibody that delivers an activating signal to myeloid cells via CD32a and displays favorable preclinical characteristics regarding its RO/tumor exposure/efficacy relationship. SRF231 is currently being evaluated in a Phase 1 clinical trial [NCT03512340] in advanced solid tumors and lymphomas. Understanding these PK/PD/tumor exposure/efficacy relationships, as well as the role of target vs FcR affinity, offers guidance on the development of CD47 antagonists for patients with cancer.

**Ethics Approval**

Mice were used in compliance with protocols approved by the IACUC of Mispro Biotech Services, Cambridge, MA (#2017-03-21SUR-1), Charles River Accelerator and Development Lab, Cambridge, MA (#CR-008), Charles River Laboratories, Worcester, MA (#I023), or MI Bioresearch, Ann Arbor, MI (VUF #26).

#### P273 Blockade of glyco-immune checkpoint using EAGLE to potentiate anticancer immunity

##### Lizhi Cao, Jenny Che, Abhishek Das, PhD, Sujata Nerle, Wayne Gatlin, Robert Leblanc, Zakir Siddiquee, Hui Xu, Karl Normington, PhD, MBA, Weiguo Yao, James Broderick, Li Peng, PhD

###### Palleon Pharmaceuticals, Waltham, MA, United States

####### **Correspondence:** Li Peng (lpeng@palleonpharma.com)

**Background**

The glyco-immune checkpoint (Siglec/sialogylcan) axis has recently emerged as a new mechanism of cancer immune evasion. We have previously described a bifunctional antibody-sialidase fusion platform named EAGLE (Enzyme-Antibody Glyco-Ligand Editing) to inhibit this axis by selectively removing the terminal sialic acids of sialoglycans on tumor cells. Using a bacterial sialidase for proof-of-concept studies, we have shown that EAGLE leads to enzyme-dependent robust monotherapy efficacy with complete regressions and immune memory in syngeneic mouse tumor models.

**Methods**

Since the bacterial sialidase poses immunogenicity concerns, we engineered and optimized a human sialidase for EAGLE overcoming the low expression yield and poor stability of human sialidases. We confirmed the antitumor activity of human sialidase-containing EAGLE in vitro and in vivo using coculture of cancer cells and primary human immune cells and immunocompetent tumor models. Furthermore, we explored and identified predictive and correlative pharmacodynamic (PD) biomarkers to EAGLE treatment in preclinical tumor models.

**Results**

The expression yield of human sialidase was improved by ~400 fold compared to the wild type through protein engineering, which enables the production of human sialidase-based EAGLE. We constructed EAGLE-408, consisting of the engineered human sialidase and anti-HER2 antibody trastuzumab, and confirmed its robust desialylation efficiency using various HER2-expressing tumor cells in vitro. EAGLE-408 enhanced macrophage-mediated phagocytosis of tumor cells in vitro. EAGLE-408 also demonstrated enzyme-dependent monotherapy efficacy with complete regressions and immune memory in syngeneic EMT6-HER2 mouse tumor models. Furthermore, in the PD study, we observed EAGLE-408 treatment enhanced CD8+ T cell infiltration into tumors, increased CD8+ T cells in the draining lymph nodes, and augmented NK cells and myeloid cells in circulation.

**Conclusions**

In summary, EAGLE with engineered human sialidase offers a new immunomodulatory approach to overcome resistance to current immunotherapies by effectively inhibiting Siglec/sialoglycan axis in the tumor microenvironment.

#### P274 Antibody targeting of tumor associated macrophages in pancreatic cancer and melanoma remodel the tumor microenvironment and revives immune targeting of tumor cells

##### Dhifaf Sarhan, PhD (dhifaf.sarhan@ki.se)

###### Karolinska Institute, Stockholm, Sweden

**Background**

Immunotherapy for cancer has revolutionized clinical practice and enabled cures for previously lethal cancers. However, the clinical responses are variable and highly influenced by immune regulatory compartments in the tumor microenvironment (TME). This is especially true for pancreatic cancer (PC) where clinical trials aiming to recover T cell anti-tumor activity have been disappointing. Thus, in PC and other cancers there is a clinical need for alternative treatments We have previously shown that antibodies targeting the scavenger receptor MARCO expressed on tumor-associated macrophages (TAMs), reduces tumor growth and impair metastasis in murine cancer models. Here we investigated targeting of the scavenger receptor MARCO on human TAMs in pancreatic ductal adenocarcinoma (PDAC) and hypothesized that targeting this receptor will remodel the suppressive environment and relive the anti-tumor responses to increase the efficacy of immunotherapy.

**Methods**

To test our hypothesis, analysis of MARCO gene expression data from the Human Protein Atlas (HPA) project was performed investigating pancreatic tumors (n=176), as these consist of up to 80% stroma, compared with healthy pancreatic tissues (n=171). In vitro, cytokine differentiated macrophages alternatively cultured with PC cell lines under hypoxia and normoxia conditions were co-cultured with cytotoxic cells to mimic their interaction in the TME. Later, macrophages were treated with anti-MARCO Abs and their phenotype and function were examined prior and following interaction with immune effector cells. Subsequently, anti-MARCO ab anti-tumor efficacy was tested in vitro and in vivo in PC and melanoma models.

**Results**

We found a 30-fold increase in MARCO expression in pancreatic tumors compared to healthy tissues. Also, a significant (p=0.03) correlation between high expression and decreased survival was noted in pancreatic cancer patients. Furthermore, pancreatic cancer cell lines induced MARCO expression on macrophages and dedifferentiated them towards myeloid-derived suppressor cells (MDSC). This effect was amplified by hypoxic condition. Notably, MARCO+ MDSC in contrast to control monocytes and macrophages suppressed T- and Natural Killer (NK) cell anti-tumor activities, which was reversed by treatment with anti-human MARCO Abs. In addition, targeting TAMs with anti-MARCO Abs, abolished their anti-inflammatory phenotype in vitro and in vivo and normalized their metabolism towards pro-inflammatory. Moreover, in B16 melanoma tumor model the anti-MARCO Ab mediated an anti-tumor effect that was dependent on NK cells and their TRAIL-mediated killing mechanism.

**Conclusions**

Thus, our study reveals a novel interaction between TAMs and cytotoxic cells as a result of TAM targeting with monoclonal Ab demonstrating a promising approach as immunotherapy that remodel the immune TME.

**Ethics Approval**

The study was approved by Institutional Ethics Board, approval number Dnr 2013.977-31.1.

#### P275 Pan-tumor pathologic scoring of response to PD-(L)1 blockade

##### Julie Stein, Evan Lipson, MD, Tricia Cottrell, MD, PhD, Patrick Forde, MD, Robert Anders, MD, PhD, Ashley Cimino-Mathews, Elizabeth Thompson, MD PhD, Mohamad Allaf, MD, Mark Yarchoan, Josephine Feliciano, MD, Elizabeth Jaffee, MD, Drew Pardoll, MD, PhD, Suzanne Topalian, MD, Janis Taube, MD, MSC

###### Johns Hopkins University School of Medicine, Baltimore, MD, United States

####### **Correspondence:** Janis Taube (jtaube1@jhmi.edu)

**Background**

Pathologic response assessment of tumor specimens from patients receiving systemic treatment provide an early indication of therapeutic efficacy and predict long-term survival. Grading systems for pathologic response were first developed for chemotherapy in select tumor types. Immunotherapeutic agents have a mechanism of action distinct from chemotherapy and are being used across a broad array of tumor types. A standardized, universal scoring system for pathologic response that encompasses features characteristic for immunotherapy and spans tumor types is needed.

**Methods**

Hematoxylin and eosin-stained slides from neoadjuvant surgical resections and on-treatment biopsies were assessed for features of immune-related pathologic response (irPR). 258 specimens from patients with 11 tumor types as part of ongoing clinical trials for anti-PD-1 were evaluated. An additional 98 specimens from patients receiving anti-PD-(L)1 in combination with other treatments were also reviewed, including those from three additional tumor types.

**Results**

Common irPR features (immune activation, cell death, tissue repair, regression bed) were present in all tumor types reviewed, including melanoma, non-small cell lung, head and neck squamous cell, Merkel cell, and renal cell carcinoma, amongst others. Features were consistent across primary tumors, lymph nodes, and distant metastases. Specimens from patients treated with anti-PD-(L)1 in combination with another agent also exhibited irPR features.

**Conclusions**

irPR features are consistent across tumor types and treatment settings. Standardized, pan-tumor immune-mediated pathologic response criteria (irPRC) are defined and associated specimen-handling considerations are described. Future, prospective studies are merited to validate irPRC in larger datasets and to associate pathologic features with long-term patient outcomes.

**Ethics Approval**

The study was approved by the Johns Hopkins University Institutional Review Board.

#### P276 Efficacy of PD-1/PDL-1 Immune Checkpoint Inhibitors in Elderly Patients with Non-Small-Cell Lung Cancer; A Subgroup Meta-analysis of Randomized Controlled Trials

##### Faisal Ali^1^, Maryam Hussain^2^, Arafat Farooqui^3^, Syed Jafri, MD^4^

###### ^1^Saint Joseph Hospital, Chicago, IL, United States; ^2^Icahn School of Medicine at Mount Sinai, New York, NY, United States; ^3^King Edward Medical University, Lahore, Pakistan; ^4^The University of Texas, Houston, TX, United States

####### **Correspondence:** Syed Jafri (syed.h.jafri@uth.tmc.edu)

**Background**

PD-1/PDL-1 immune-checkpoint inhibitors (ICPIs) have emerged as an efficacious drug class for the treatment of non-small-cell lung cancer (NSCLC). The efficacy of PD-1/PD-L1 ICPI therapy in the elderly (patients age ≥65 and ≥75) has not been thoroughly investigated. The aim of this study was to assess the efficacy of PD-1/PDL-1 ICPIs compared to chemotherapy in elderly patients with NSCLC.

**Methods**

A systematic review of the literature to identify randomized controlled trials (RCTs) which reported overall survival (OS) and progression free survival (PFS) of elderly patients with NSCLC who were randomized to receive PD-1/PDL-1 ICPIs or docetaxel/investigator’s choice chemotherapy. The hazard ratios (HR) of OS and PFS in elderly patients (along with their 95% confidence intervals; CI) were extracted to compute a pooled (HR) to report the efficacy of PD-1/PDL-1 versus chemotherapy stratified by patient age (≥65 and ≥75). A random effects model was employed only when there was significant heterogeneity among studies (>40%, as assessed by I-squared).

**Results**

Screening of 15,092 studies yielded four RCTs (two with patients age ≥75) which enrolled a total of 2,429 patients. No significant difference in PFS with PD-1/PDL-1 treatment versus chemotherapy was found among patients aged ≥65 (HR 0.8, 95% CI 0.53-1.06, I2 78.30%) or patients aged ≥75 (HR 1.1, 95% CI 0.43-1.77,I2 0.00%). Patients aged ≥65 had an improved OS with PD-1/PD-L1 ICPI treatment versus chemotherapy (HR 0.67, 95% CI 0.51-0.84, I2 62.50%). Patients aged ≥75 did not show any significant difference in OS when treated with PD-1/PD-L1 versus chemotherapy (HR 1.02, 95% CI 0.35-1.69,I2 0.00%).

**Conclusions**

Patients aged ≥65 show an improved OS with PD-1/PD-L1 therapy. However, NSCLC patients ≥75 do not show a significant difference in OS or PFS when treated with PD-1/PD-L1 ICPI versus chemotherapy (Figure 1). This may be a consequence of aging and its impact on individuals’ capability to mount an anti-tumor response. Further studies assessing the efficacy of PD-1/PD-L1 ICPI use in patients ≥75 are warranted.

**Fig. 1 (abstract P276).**
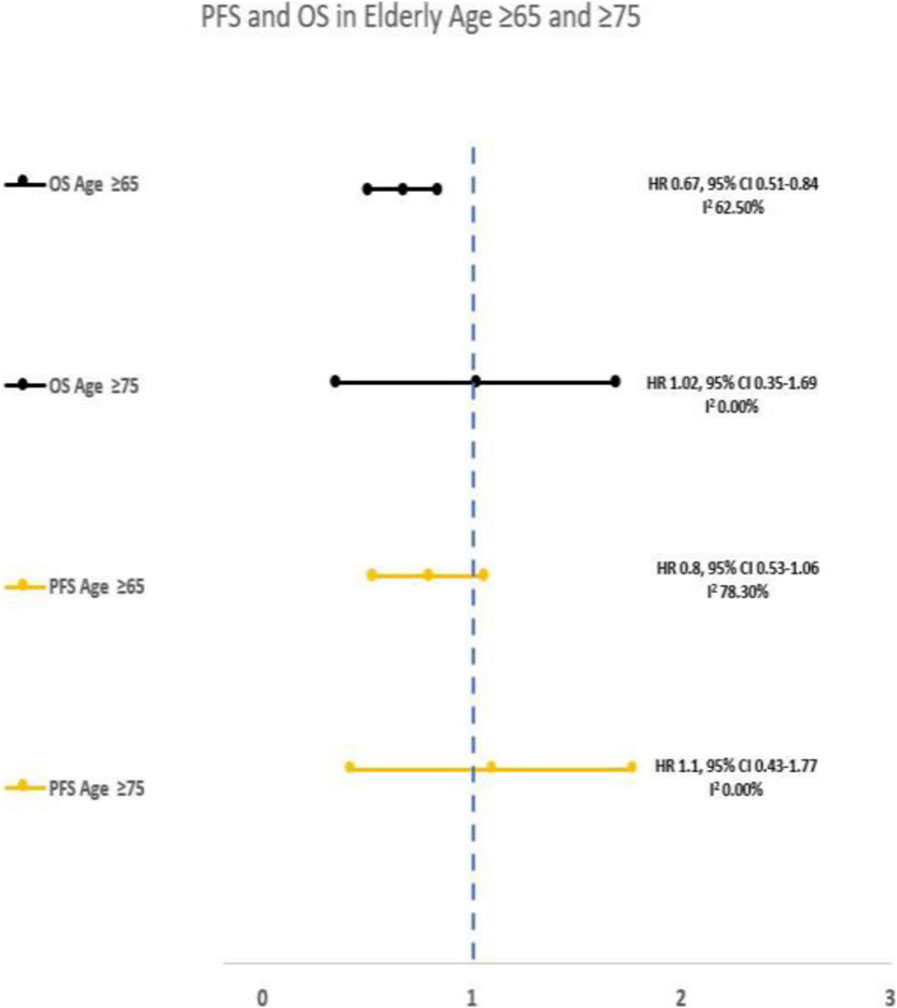
OS and PFS in Elderly Patients with NSCLC

#### P277 Utility of PD-L1 Expression in Non-Small-Cell Lung Cancer Patients Treated with PD-1/PDL-1 Immune Checkpoint Inhibitors; A Subgroup Meta-analysis of Randomized Controlled Trials

##### Faisal Ali^1^, Maryam Hussain^2^, Arafat Farooqui^3^, Syed Jafri, MD^4^

###### ^1^Saint Joseph Hospital, Chicago, IL, United States; ^2^Icahn School of Medicine at Mount Sinai, New York, NY, United States; ^3^King Edward Medical University, Lahore, Pakistan; ^4^The University of Texas, Houston, TX, United States

####### **Correspondence:** Syed Jafri (syed.h.jafri@uth.tmc.edu)

**Background**

PD-1/PDL-1 immune-checkpoint inhibitors (ICPIs) have emerged as an efficacious drug class for the treatment of non-small-cell lung cancer (NSCLC) evident by findings of multiple randomized controlled trials (RCTs). The utility of PD-L1 expression analysis in treatment planning and prognosis remains questionable. The aim of this study was to assess the efficacy of PD-1/PDL-1 ICPIs compared to chemotherapy in patients with NSCLC stratified by PD-L1 expression

**Methods**

A systematic review of the literature to identify RCTs which reported overall survival (OS) and progression free survival (PFS) of patients with NSCLC who were randomized to receive PD-1/PDL-1 ICPIs or docetaxel/investigator’s choice chemotherapy and underwent PD-L1 expression analysis. The hazard ratios (HR) of various degrees of PD-L1 expression (along with their 95% confidence intervals; CI) were extracted to compute a pooled (HR) to report the efficacy of PD-1/PDL-1 versus chemotherapy stratified by the degree of PD-L1 expression. A random effects model was employed only when there was significant heterogeneity among studies (>40%, as assessed by I-squared

**Results**

Screening of 15,092 studies yielded four RCTs (two reporting only PD-L1 expression ≥50%) which enrolled a total of 2,429 patients. An improved PFS with PD-1/PDL-1 treatment versus chemotherapy was found among patients with PD-L1 expression ≤1% (HR 0.78, 95% CI 0.57-0.98, I2 25.30%) ≥1% (HR 0.62, 95% CI 0.46-0.78, I2 0.00%), ≥5% (HR 0.46, 95% CI 0.31-0.6, I2 0.00%), and ≥10% (HR 0.44, 95% CI 0.29-0.58, I2 0.00%). Similarly, an improved OS was observed with PD-1/PDL-1 treatment versus chemotherapy was found among patients with PD-L1 expression ≥1% (HR 0.7, 95% CI 0.52-0.87, I2 0.00%), ≥5% (HR 0.54, 95% CI 0.38-0.69, I2 0.00%), and ≥10% (HR 0.51, 95% CI 0.35-0.68, I2 0.00%), but not with PD-L1 expression of ≤1%, ≤5%, and ≤10% (figure 1). No significant difference in OS and PFS was observed with PD-1/PD-L1 treatment versus chemotherapy among patients with ≥50% PD-L1 expression. Of the two RCTs which reported ≥50% PD-L1 expression, one used Nivolumab (and reported a negative outcome in OS and PFS) whereas the other used Pembrolizumab (and reported a favorable OS and PFS).

**Conclusions**

NSCLC patients who express PD-L1 have an improved OS and PFS with PD-1/PD-L1 ICPI use versus chemotherapy. However, high PD-L1 expression (≥50%) may not translate into a better response to all PD-1/PD-L1 ICPIs (Figure 1). Further studies are needed to assess the intra-class efficacy of different PD-1/PD-L1 ICPIs in NSCLC patients with a high PD-L1 expression.

**Fig. 1 (abstract P277).**
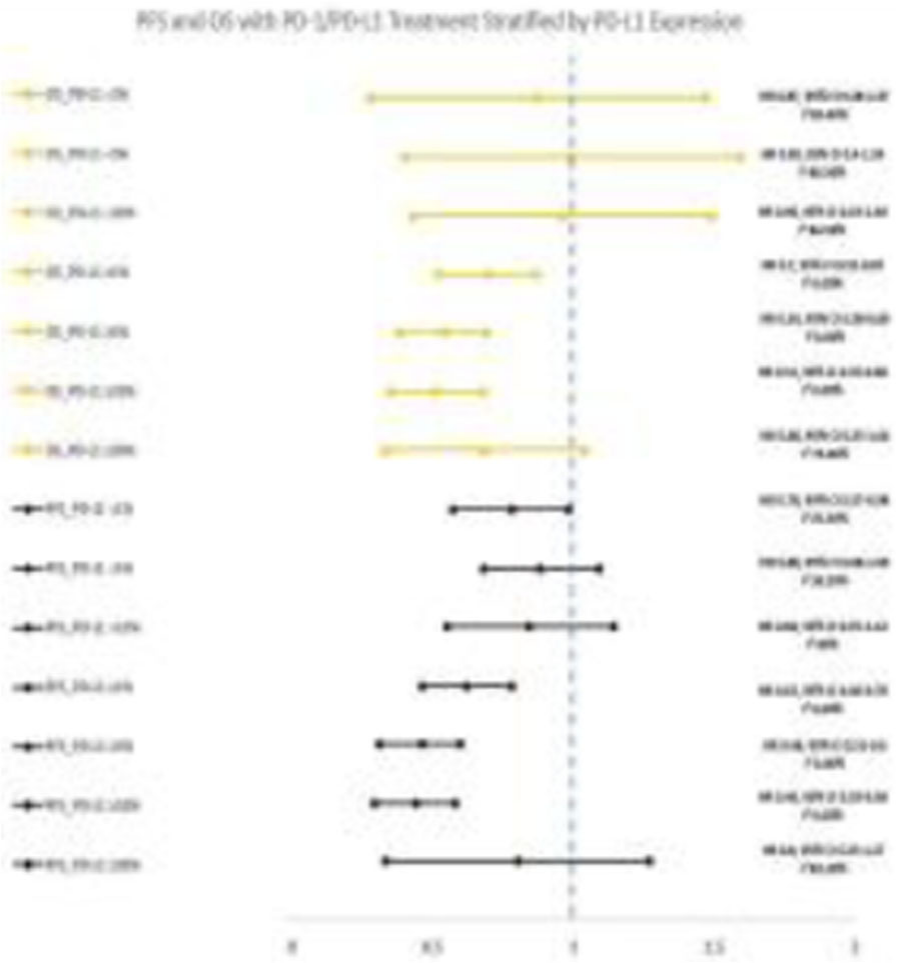
OS and PFS Stratified by PD-L1 Expression

#### P278 PD-1/PDL-1 immune checkpoint inhibitors in smokers with non-small-cell lung cancer; a subgroup meta-analysis of randomized controlled trials

##### Faisal Ali^1^, Bibek Pannu^2^, Maryam Hussain^3^, Arafat Farooqui^4^, Taha Alrifai^1^, Phyo Myint^1^, Syed Jafri, MD^5^

###### ^1^Saint Joseph Hospital, Chicago, IL, United States; ^2^John H Stroger Jr Hospital, Chicago, IL, United States; ^3^Icahn School of Medicine at Mount Sinai, New York, NY, United States; ^4^King Edward Medical University, Lahore, Pakistan; ^5^The University of Texas, Houston, TX, United States

####### **Correspondence:** Syed Jafri (syed.h.jafri@uth.tmc.edu)

**Background**

PD-1/PDL-1 immune-checkpoint inhibitors (ICPIs) have emerged as an efficacious drug class for the treatment of non-small-cell lung cancer (NSCLC) evident by findings of multiple randomized controlled trials (RCTs). However, the efficacy of PD-1/PDL-1 ICPIs in patients with NSCLC who are current or former smokers remains to be debated. The aim of this study was to assess the efficacy of PD-1/PDL-1 ICPIs compared to chemotherapy in patients with NSCLC who were active or former smokers

**Methods**

A systematic review of the literature to identify RCTs which reported overall survival (OS) and progression free survival (PFS) among former or active smokers with NSCLC who were randomized to receive PD-1/PDL-1 ICPIs or docetaxel or investigator’s choice chemotherapy. The hazard ratios (HR) of these subgroups (along with their 95% confidence intervals; CI) were extracted to compute a pooled (HR) to report the efficacy of PD-1/PDL-1 versus chemotherapy in former or active smokers with NSCLC. A random effects model was employed only when there was significant heterogeneity among studies (>40%, as assessed by I-squared)

**Results**

Screening of 15,092 studies yielded six RCTs which enrolled a total of 3,584 patients. PFS did not differ significantly with PD-1/PDL-1 treatment or chemotherapy among non-smokers (HR 1.33, 95% 0.80-1.80; I2 20.6%) and former or active smokers (HR 0.83, 95% CI 0.63-1.04; I2 67.5%). Survival analysis revealed that former or active smokers tended to have an improved OS when treated with PD-1/PDL-1 inhibitors versus chemotherapy (HR 0.72, 95% CI 0.54-0.90; I2 70.5%), whereas OS did not differ significantly among non-smokers when treated with PD-1/PDL-1 inhibitors versus chemotherapy (HR 0.76, 95% CI 0.48-1.04; I2 0.0%)

**Conclusions**

NSCLC patients who are former or active smokers have an improved OS when treated with PD-1/PDL-1 ICPIs, though the PFS does not differ significantly (Figure 1). This may be a consequence of a higher mutation burden among smokers. Further research into the interaction between carcinogens, toxins and proinflammatory substances of smoking and ICPIs are warranted.

**Fig. 1 (abstract P278).**
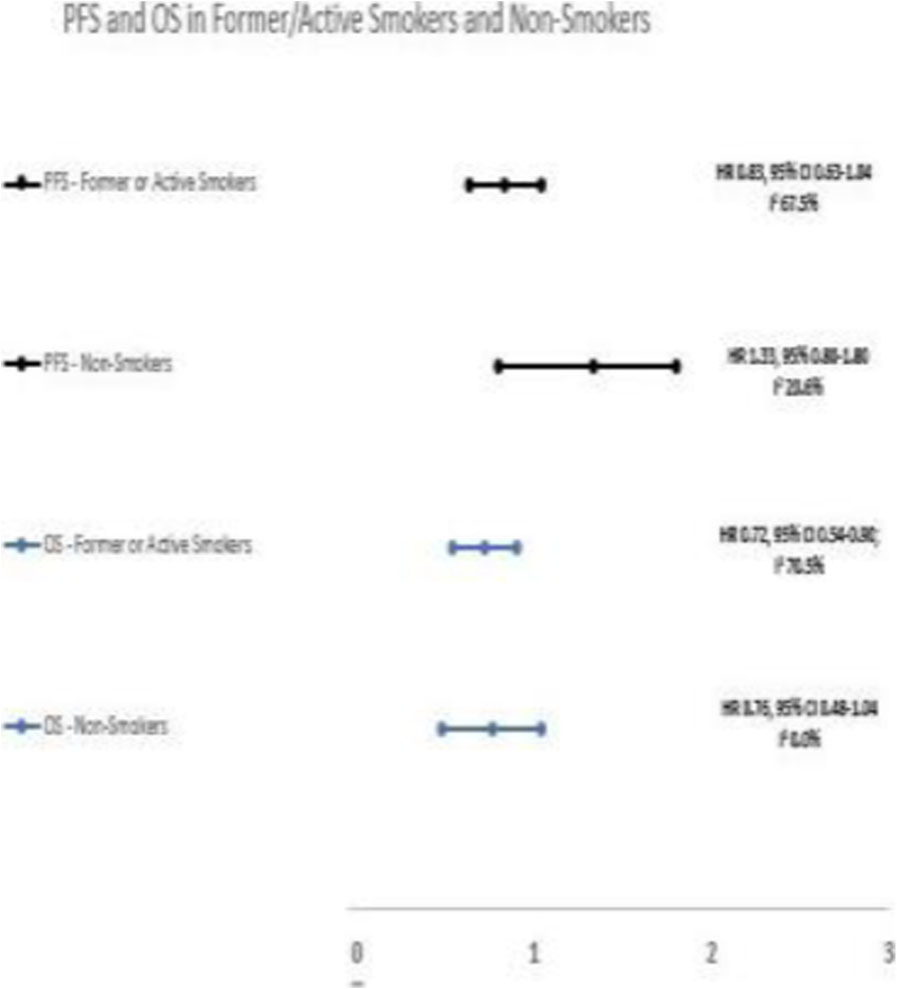
OS and PFS in Smokers and Non-Smokers

#### P279 PDL-1 is highly expressed in ovarian germ cell tumor and associated with cancer stem cells population expressing CD44

##### Salmah Alamri, Miral Mashhour, Kholoud Alwosaibai, PhD

###### King Fahad Specialist Hospital, Dammam, Saudi Arabia

####### **Correspondence:** Kholoud Alwosaibai (kh20978@gmail.com)

**Background**

Immunotherapy using checkpoint inhibitors have proposed beneficial effects for some types of cancer such as lung cancer and melanoma. However, using PD-1/PDL-1 inhibitors to treat ovarian cancer was limited and further studies are needed to identify the patients that will benefit from this treatment. In this study we have explored predictive biomarkers in ovarian cancer that might associate with checkpoint treatment outcome. For the first time, we have investigated the role of PDL-1 expression in the tumor microenvironment cells that includes immune cells and cancer stem cells in different types of ovarian cancer.

**Methods**

66 surgical samples of different types of ovarian cancer have been collected from pathology department. IHC staining has been performed using PD-L1 IHC 22C3 pharmDx to detect PDL-1, CD8 and CD4 to detect tumor infiltrating lymphocyte (TIL), and CD44, CD117 and OCT3/4 to detect stem cell markers.

**Results**

We found that 47% of ovarian cancer patients express PDL-1. The expression of PDL-1 have been detected in different types of ovarian cancer including, serous carcinoma, germ cell tumor, endometrioid. The majority (73%) of germ cell tumor tissues express PDL-1 whereas serous cancer and endomitrioid express PDL-1 in 46% and 50% of the cancer tissue, respectively. However, PDL-1 protein was undetectable in some histological type of ovarian cancer such as granulosa tumor and mucinous tumor.

Also we determined the expression levels of TIL in the ovarian cancer tissue that either PDL-1 positive or negative. We found that 81% of ovarian cancer samples have TIL that express CD8 and 92% of these ovarian cancer samples are associated with PDL-1 expression.TIL that express CD4 have been found in 66% of all ovarian cancer samples and 77% of these samples are associated with PDL-1 expression.

Further, we have studied the association between PDL-1 expression and cancer stem cell markers such as CD44, CD117, OCT 3/4. We found that all PDL-1 positive samples are expressing CD44 and also, we found strong association between CD117 expression and PDL-1 expression.

**Conclusions**

Immunotherapy treatment using PDL-1 inhibitor could be considered for ovarian cancer patients that expressing PDL-1 particularly, germ cell ovarian cancer. In addition, PDL-1 expression is strongly associated with CD44. Inhibiting PDL-1 using immunotherapy might downregulate stem cell populations and decrease the chemotherapy resistance and recurrence that derived by stem cell residual in the cancer tissue.

**Ethics Approval**

The study was approved by the IRB at King Fahd Specialist Hospital- Dammam. approval number ONC0340

#### P280 Impact of tumor mutational burden on overall survival in patients with non-small cell lung cancer treated with immunotherapy

##### Mark Awad, MD PhD^1^, Navin Mahadevan^2^, Andrew Polio^1^, Natalie Vokes^1^, Elizabeth Aguilar^1^, Biagio Ricciuti, MD^1^, Giuseppe Lamberti, MD^1^, Gonzalo Recondo, MD^1^, Giulia Leonardi^1^, Anika Adeni^1^, Pasi Janne, MD PhD^1^, Eliezer Van Allen, MD^1^, Adem Albayrak, MS^1^, Renato Umeton^1^, Lynette Sholl^2^

###### ^1^Dana-Farber Cancer Institute, Boston, MA, United States; ^2^Brigham and Women’s Hospital, Boston, MA, United States

####### **Correspondence:** Mark Awad (Mark_Awad@DFCI.harvard.edu)

**Background**

In non-small cell lung cancer (NSCLC), tumor mutational burden (TMB) has been proposed as a biomarker of response to immune checkpoint inhibitors, but the optimal TMB cutpoint associated with a survival benefit remains unclear.

**Methods**

We collected clinicopathologic data from patients with NSCLC sequenced by the OncoPanel NGS platform at the Dana-Farber Cancer Institute. The relationship between TMB and clinical outcomes after treatment with immune checkpoint inhibitors was investigated in the subset of patients treated with immunotherapy.

**Results**

Among 2690 patients identified, median TMB was significantly higher among current smokers compared to former (P<0.0001) and never smokers (P<0.0001) and among squamous tumors compared to smoking-related nonsquamous tumors (P=0.01) (Figure 1). Patients without oncogenic drivers and those harboring BRAF or KRAS mutation had the highest median TMB (10.6, 11.1 and 9.8 mutations/megabase [mut/Mb], respectively), while tumors with ROS1, MET, RET and ALK alterations had the lowest median TMB (6.7, 6.1, 5.3, 5.3 mut/Mb, respectively) (Figure 2). Among patients treated with immunotherapy (N=489), a recursive partitioning algorithm identified an optimal TMB cut-off for PFS and OS of 18.5 mut/Mb, which represents the 88th percentile for TMB in this cohort. Baseline clinicopathologic characteristics were well-balanced between patients with a TMB of ≥18.5 and <18.5 mut/Mb (Table 1). Patients with a TMB of ≥18.5 mut/Mb had a significantly higher response rate (43.3% vs. 17.5%, P<0.0001), a longer median progression-free survival (8.2 vs. 2.7 months, HR:0.52 [95%CI:0.38-0.72], P<0.0001), and a longer median overall survival (20.7 vs. 10.2 months, HR:0.55 [95%CI:0.38-0.79], P=0.001) compared to those with a TMB <18.5 mut/Mb (Figure 3). After adjusting for performance status, smoking history, and PD-L1 expression, a TMB of ≥18.5 mut/Mb was associated with a significantly longer PFS (HR:0.56 [95%CI: 0.41-0.78], P =0.001) and OS (HR: 0.57 [95%CI: 0.40-0.82], P =0.003) in multivariate analysis.

**Conclusions**

Tumor-only NGS identifies clinical and genomic correlates of high TMB in NSCLC. Patients with a TMB ≥88th percentile are most likely to experience a survival benefit when treated with immune checkpoint inhibitors.

**Fig. 1 (abstract P280).**
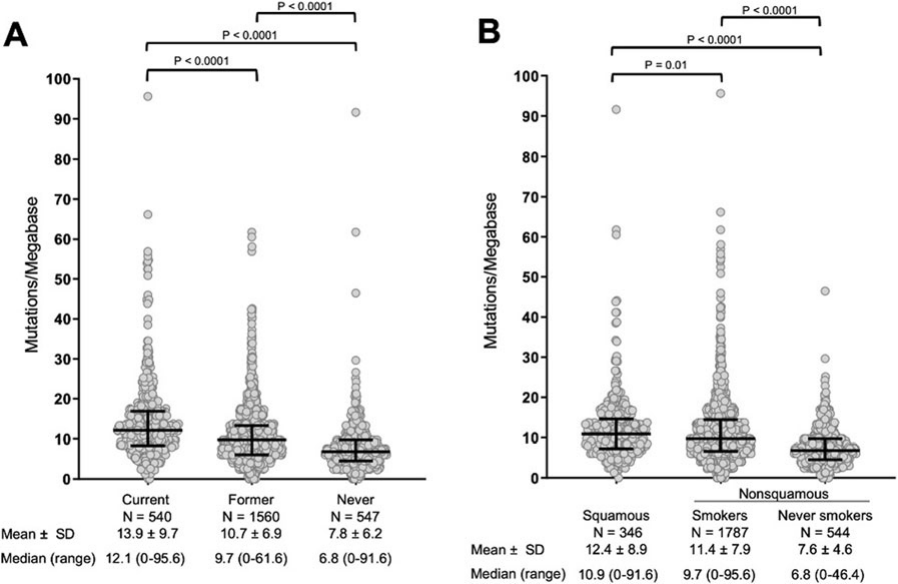
See text for description

**Fig. 2 (abstract P280).**
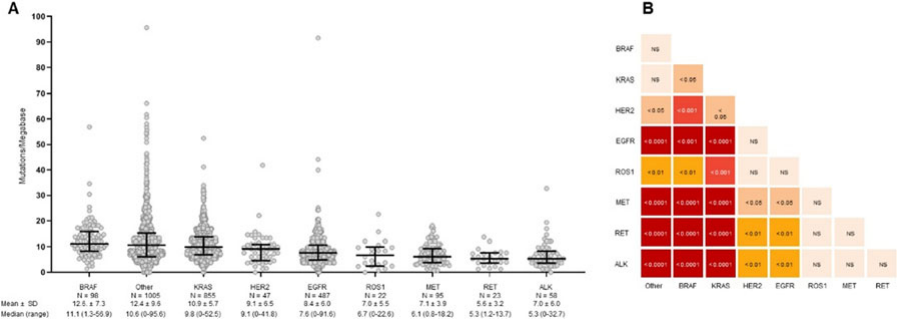
See text for description

**Fig. 3 (abstract P280).**
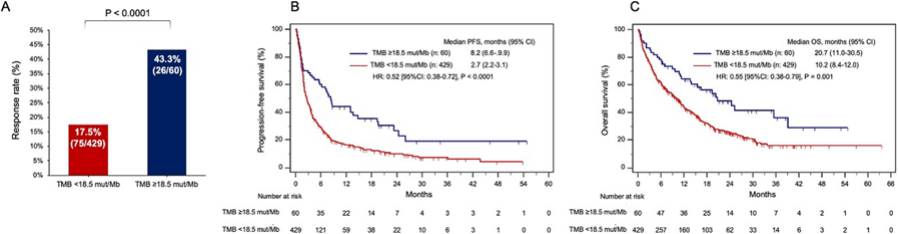
See text for description

**Table 1 (abstract P280).**
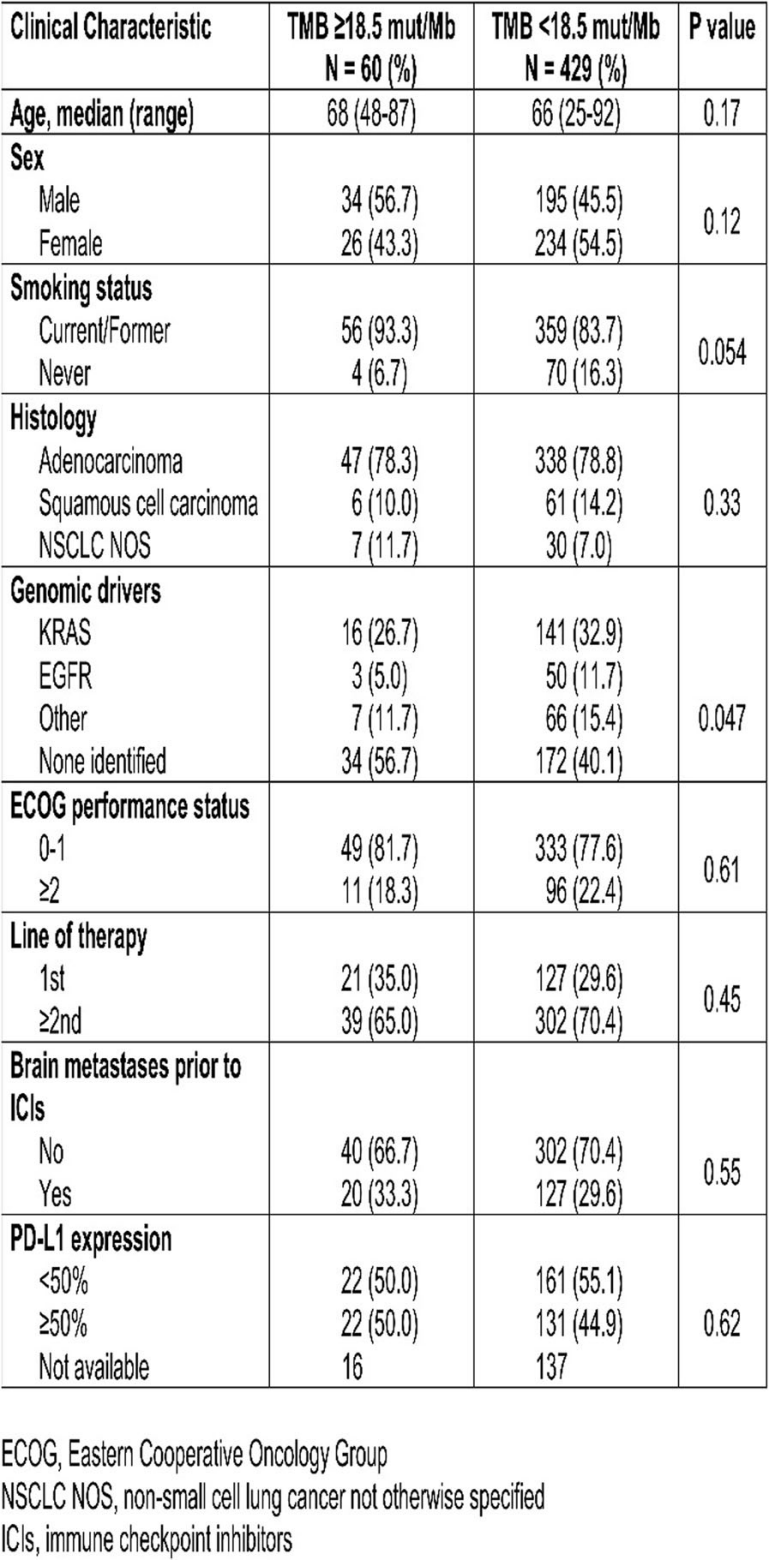
See text for description

#### P281 Durable response after immunotherapy discontinuation: a multicenter real-life experience

##### Maria Bassanelli, MD, PhD^1^, Diana Giannarelli, MD^2^, Marco Russano^3^, Fabiana Letizia Cecere^2^, Maria Rita Migliorino^4^, Silvana Giacinti^5^, Viola Barucca^4^, Emilio Bria^6^, Enzo Maria Ruggeri^5^, Fabio Calabrò^4^, Alain Gelibter^7^, Daniele Santini^3^, Anna Ceribelli^1^

###### ^1^San Camillo de Lellis Hospital, Rieti, Italy; ^2^Regina Elena National Cancer Institute, Rome, Italy; ^3^University Campus Bio-Medico, Rome, Italy; ^4^San Camillo Forlanini Hospital, Rome, Italy; ^5^Belcolle Hospital, Viterbo, Italy; ^6^Fondazione Pol. Universitario A.Gemelli, Rome, Italy; ^7^Policlinico Umberto I, Rome, Italy

####### **Correspondence:** Maria Bassanelli (maria.bassanelli@yahoo.it)

**Background**

Immune checkpoint inhibitors (ICIs) have significantly improved overall survival (OS) in several cancer types. Unlike chemotherapy, the optimum treatment duration with ICIs is not clearly established [1,2]

**Methods**

We conducted an observational, retrospective analysis of 46 consecutive patients (pts) with advanced solid tumors who discontinued immune-based therapies for any reason except progressive disease. The aim of this study was to assess the outcome and the antitumor activity of ICIs after treatment discontinuation. Treatment-free survival (TFS) was defined as the time from interruption of immunotherapy for any reason except progressive disease to start of subsequent anticancer therapy or best supportive care or death. Median OS, progression free survival (PFS), TFS and the 95% confidence interval (CI) were estimated with the Kaplan -Meier method

**Results**

46 pts (median age 68 years [range 41-86]; male: 65.2%) with advanced cancer (n.39 non-small-cell lung cancer, n.15 renal cell carcinoma and n.2 melanoma) were treated with ICIs, as clinically indicated, at eight Italian institutions: 44 pts received programmed death 1 (PD-1) inhibitors (n.31 nivolumab, n.13 pembrolizumab) and 2 pts programmed death ligand 1 (PD-L1) (n.1 durvalumab, n.1 atezolizumab). A median of 8 cycles were administered [range 1 to 52]. 36 pts discontinued ICIs due to toxicities (diarrhoea, pneumonitis, hepatotoxicity)and 10 pts for reasons non immune-related. The median PFS from the beginning of ICIs was 12.4 months (mo) [95% CI: 8.2-16.6] and the median OS was 20.0 mo (95% CI: 11.8-28.2). Median PFS from ICIs completion was 5.0 mo [95% CI: 2.7-7.3] and median OS was 16.1 mo (95% CI: 5.4-26.8). Median TFS was 7.4 (95% CI: 5.8-8.9) mo

**Conclusions**

This study shows the durable cancer-specific immune response in pts with advanced cancer even after stopping the ICIs for any reason except progressive disease

**References**

1. Kim PS, Ahmed R. Features of responding T cells in cancer and chronic infection. Curr Opin Immunol 2010; 22: 223–230.

2. Schadendorf D, Hodi FS, Robert C et al. Pooled analysis of long-term survival data from phase II and phase III trials of ipilimumab in unresectable or metastatic melanoma. J Clin Oncol 2015; 33: 1889–1894.

#### P282 Tumor-targeted T-cell activation via an investigational PD-L1 x CD137 bispecific molecule

##### Alexey Berezhnoy, PhD, Ling Huang, Daorong Liu, Jennifer DiChiara, Jonathan Li, PhD, Douglas Smith, PhD, Jill Rillema, BS, Valentina Ciccarone, PhD, James Tamura, PhD, Ralph Alderson, PhD, Gundo Diedrich, Ezio Bonvini, PhD, Paul Moore, PhD

###### MacroGenics, Inc, Rockville, MD, United States

####### **Correspondence:** Paul Moore (moorep@macrogenics.com)

**Background**

Blockade of the PD-1/PD-L1 axis can improve outcome in a variety of cancers; yet, many patients, including subsets of patients with PD-L1+ tumors, do not benefit. The magnitude of immune activation promoted by PD-1/PD-L1 axis blockade can be further enhanced through concomitant T-cell co-stimulation such as that achieved through CD137 agonism; however, clinical applications of such an approach may be limited by toxicity associated with the systemic effects of CD137 agonists. Here we characterize PD-L1 and CD137 tumor expression supporting the development of a PD-L1xCD137 bispecific molecule that provides PD-1 axis blockade coupled with tumor-targeted CD137 co-stimulation.

**Methods**

In situ hybridization (ISH) and multicolor flow cytometry was performed to characterize PD-L1 and CD137 expression in tumor biopsies. A PD-L1xCD137 bispecific molecule (PD-L1xCD137) was constructed based on PD-L1 blocking mAbs and CD137-engaging mAbs and was evaluated for binding to respective antigens. Its functional activity was evaluated in CD3 or SEB-driven T-cell activation systems, MLR assays and tumor microenvironment models. Anti-tumor activity in vivo was evaluated in combination with tumor targeted anti-CD3 based bispecific DART® molecules.

**Results**

ISH revealed expression of PD-L1 in significant proportion of surgically resected carcinomas; noteworthy, many such tumors contained CD137+ immune infiltrate adjacent to PD-L1+ cells. Moreover, ex vivo co-incubation of tumor and immune cells in the presence of CD3-based bispecifics or Fc-enhanced antibodies further induces PD-L1 and CD137 expression. PD-L1xCD137 binds and blocks PD-L1, reversing PD-1-mediated T-cell inhibition equipotently to the effect of approved PD-L1 benchmark mAbs; it also binds CD137, but absent clustering supported by PD-L1+ cells, fails to induce CD137 signaling. In the presence of PD-L1-expressing cells, however, PD-L1xCD137 drives CD137 activation and immune cell co-stimulation. Robust T-cell activation and cytokine secretion was induced by PD-L1xCD137, with significantly greater activity than that observed with the combination of PD-L1 blocking and CD137 agonistic mAbs. Notably, when combined with tumor targeted immunotherapies, PD-L1xCD137 supports enhanced activation of effector cells and anti-tumor activity.

**Conclusions**

These data show that an investigational PD-L1xCD137 bispecific can switch on CD137 co-stimulation in a PD-L1-dependent fashion. While tumor adaptive resistance via PD-L1 induction promotes tumor immune escape, PD-L1xCD137 can exploit the checkpoint ligand up-regulation by contributing a co-stimulatory signal in addition to checkpoint blockade. PD-L1xCD137 provides a potential therapeutic approach to overcome limitations of existing PD-1/PD-L1-targeting strategies either as monotherapy or in combination with complementary immune based therapeutic modalities, such as CD3 based bispecifics or Fc-enhanced mAbs.

#### P283 Generation and characterization of a PD-1 resistant mouse tumor model

##### Marie Bernardo, PhD, Tatiana Tolstykh, Yu-An Zhang, Dinesh Bangari, Hui Cao, PhD, Joon Sang Lee, PhD, Natalia Malkova, Jack Pollard, PhD, Fangxian Sun, Dmitri Wiederschain, Timothy Wagenaar

###### Sanofi Oncology Research, Cambridge, MA, United States

####### **Correspondence:** Timothy Wagenaar (timothy.wagenaar@sanofi.com)

**Background**

Immune checkpoint blockade elicits durable anti-cancer responses in the clinic, however a large proportion of patients do not benefit from treatment. Several mechanisms of innate and acquired resistance to checkpoint blockade have been defined and include mutations of MHC I and IFNg signaling pathways. However, such mutations occur in a low frequency of patients and additional mechanisms have yet to be defined. In an effort to better understand acquired resistance to checkpoint blockade, we generated a mouse tumor model exhibiting in vivo resistance to anti-PD-1 antibody treatment.

**Methods**

A PD-1 resistant mouse tumor model was generated by serially passaging MC38 tumors in mice treated with anti-PD-1. The resistant tumor line was characterized using a range of molecular techniques.

**Results**

MC38 tumors acquired resistance to PD-1 blockade following serial in vivo passaging. Lack of sensitivity to PD-1 blockade could not be attributed to dysregulation of PD-L1 or B2M expression, as both were expressed at similar levels in parental and resistant cells. Similarly, IFNg signaling and antigen processing and presentation pathways were functional in both parental and resistant cell lines. Unbiased gene expression analysis was used to further characterize potential resistance mechanisms. RNA-sequencing revealed substantial differences in global gene expression with PD-1 resistant tumors displaying a marked reduction in expression of immune-related genes relative to parental MC38 tumors. Transcriptomic data revealed that PD-1 resistant tumors exhibit reduced immune infiltration across multiple cell types, including T and NK cells, while pathway analysis identified activation of two major tumor promoting signaling pathways in PD-1 resistant tumors. Pharmacological inhibition of these pathways in combination with PD-1 blockade inhibited tumor growth and extended the survival of mice bearing resistant tumors.

**Conclusions**

This study describes a novel PD-1 resistant mouse tumor model and underscores the importance of two well defined signaling pathways to response to immune checkpoint blockade.

#### P284 Pitfalls in preclinical development of immunotherapies for ER+ breast cancer: estrogen as an immunomodulator potentially influencing pembrolizumab efficacy in a breast cancer model in humanized mice

##### Tiina Kähkönen^1^, Mari Suominen^1^, Jenni Mäki-Jouppila^1^, Emrah Yatkin^2^, Philip Dube^3^, Azusa Tanaka^3^, Jussi Halleen^1^, Jenni Bernoulli, PhD^1^

###### ^1^Pharmatest Services, Turku, Finland; ^2^University of Turku, Turku, Finland; ^3^Taconic Biosciences, Rensselaer, NY, United States

####### **Correspondence:** Jenni Bernoulli (jenni.bernoulli@pharmatest.com)

**Background**

Immunotherapies have the potential to improve outcomes in triple-negative breast cancer patients but evidence is less consistent in estrogen receptor -positive (ER+) patients. To advance preclinical development and understand the effects of immunotherapies against ER+ breast cancer, we aimed to establish a novel orthotopic ER+ breast cancer model in humanized mice and to study efficacy of pembrolizumab in the model.

**Methods**

Female CIEA NOG® (NOG) mice and NOG mice engrafted with human CD34+ hematopoietic stem cells (huNOG, Taconic Biosciences) were implanted with 5 μg/day estradiol (E2)-releasing implants, and one week later inoculated with ER+ MCF-7 human breast cancer cells into the mammary fat pad. One group of huNOG mice did not receive E2 implants. Orthotopic tumor growth was followed by caliper measurements. At study week 2, the E2 supplemented mice were stratified to receive human IgG4 isotype control or pembrolizumab (5 mg/kg, i.p., Q5D) until the end of the study. The study was terminated at study week 7 and tumors were processed for histological and immunohistochemical analysis of tumor infiltrating lymphocytes (TILs). Changes in blood cell counts were assessed by flow cytometry and hematology.

**Results**

ER+ orthotopic breast tumors grew only in the presence of E2 supplement. However, general condition of huNOG mice started to decrease after 3 weeks on E2 supplementation and their survival was decreased compared to huNOG mice without E2 supplement. Hematological analysis indicated that estrogen decreased the levels of white and red blood cells, hemoglobin and hematocrit that were further decreased by concomitant pembrolizumab treatment. Flow cytometry analysis confirmed lower numbers of CD3+, CD4+ and CD8+ T cells in the blood of E2 supplemented huNOG mice. Tumor immunohistochemistry showed low number of TILs and very low expression of programmed death-ligand 1 (PD-L1). No clear anti-tumor effects were observed with pembrolizumab treatment compared to the isotype control group.

**Conclusions**

In conclusion, estrogen supplementation was needed to support orthotopic ER+ breast cancer growth. However, estrogen decreased survival of female huNOG mice. Estrogen had immunomodulatory effects and induced adverse effects including anemia. Due to these deleterious effects and decreased number of immune cells, no direct conclusions can be drawn for the possible anti-tumor effects of pembrolizumab in this orthotopic ER+ breast cancer model. Caution should be taken when evaluating efficacy of immunotherapies in hormone-dependent preclinical cancer models, and using ER+ breast cancer models where tumor growth is supported by local microenvironment, such as in bone metastasis models, should be considered.

**Ethics Approval**

This study was approved by the National Animal Experiment Board in Finland; license number ESAVI-2331-04 10 07-2017.

#### P285 Integrated molecular characterization of primary resistance mechanisms to immune checkpoint blockade in advanced non-small cell lung carcinoma (a-NSCLC)

##### Benjamin Besse, MD PhD^1^, Caroline Fraslon, PhD^2^, Hui Cao, PhD^2^, Joon Sang Lee, PhD^2^, Stephanie Malyszka, MS^2^, Marielle Chiron, PhD^2^, Anne Caron, PhD^2^, Souâd Naimi, PhD^2^, Laura Mezquita, MD^1^, David Planchard, MD, PhD^1^, Jean-Yves Scoazec, MD^1^, Ludovic Lacroix, PharmD^1^, Etienne Rouleau, MD^1^, Naima Imam-Sghiouar, PhD^1^, Aurélien Marabelle, MD PhD^1^, Eric Angevin, MD^1^, Christophe Massard, MD^1^, Jack Pollard, PhD^2^

###### ^1^Gustave Roussy Cancer Center, Villejuif, France; ^2^Sanofi, Vitry sur Seine, France

####### **Correspondence:** Caroline Fraslon (caroline.fraslon@sanofi.com)

**Background**

Reinvigoration of anti-tumor immunity via immune checkpoint blockade (ICB) has transformed outcomes in a-NSCLC. However, a majority of patients are innately resistant to ICB, and a better understanding of the resistance mechanisms may guide the development of new treatment strategies and patients therapies.

**Methods**

Biopsies performed immediately before treatment with single agent ICB in patients with a-NSCLC (MATCH-R trial [NCT02517892]) were analyzed. The stromal microenvironment and immune context was characterized via an integrated analysis of whole transcriptome (RNA-seq), whole exome sequencing (WES), and immunohistochemistry (IHC) of CD3, CD8, FOXP3 and PDL1. Specifically, the immune context and the relative abundance of 10 immune and stromal cell types were assessed with integrated IHC and Cell Populations-counter (MCP-counter) [1] analysis of the RNA-seq. Somatic mutations and Tumor Mutation Burden (TMB) were evaluated. The transcriptional state of the tumor and its microenvironment was assessed by GSVA analysis [2] of the MSigDB collection [3]. Patient’s outcome was associated to molecular data. Primary resistance to ICB was defined as PD in the first radiological examination, or a median PFS inferior to 3 months.

**Results**

Thirty-one patients with adenocarcinoma were enrolled: Median age was 60 (34-80), 13 were female, 28 were smokers, 10 were responders, and 21 were non-responders. Median tumor cellularity was 60% (30%-90%).

Responders had higher TMB and immune infiltration compared to non-responders. Non-responders could be divided into two classes: those with equal infiltration to responders “hot tumors” and those with less infiltration “cold tumors”. Neutrophil infiltration was associated with resistance to ICB in both “hot” and “cold” resistant tumors. Mutations in the IFN-γ and/or KRAS/STK11/KEAP pathways were associated to ICB resistance. Increased activation of hypoxia-response, transforming growth factor (TGF-β) and MYC pathways from the GSVA analysis were also associated to ICB resistance. TGF-β pathway activation was associated to the “cold tumor” ICB-resistant tumor class.

**Conclusions**

ICB sensitivity was associated to TMB, IFN-γ pathway mutation, and immune infiltration. We have further refined our understanding of the primary ICB resistance mechanisms in that there are both “hot” and “cold” non-responsive tumors, which suggests that different therapeutic approaches be may be required in these two subtypes, i.e. targeting the TGF-β pathway in the “cold” non-responding tumor class. We are continuing to increase our baseline cohort size and will include post-treatment biopsies collected with this protocol.

**References**

1. Becht E, Giraldo NA, Lacroix L, Buttard B, Elarouci N, Petitprez F, Selves J, Laurent-Puig P, Sautès-Fridman C, Fridman WH, de Reyniès A. Estimating the population abundance of tissue-infiltrating immune and stromal cell populations using gene expression. Genome Biol. 2016; 17(1):218-238.

2. Hänzelmann S, Castelo R, Guinney J. GSVA: gene set variation analysis for microarray and RNA-seq data. BMC Bioinformatics. 2013;14:7-22.

3. Liberzon A, Subramanian A, Pinchback R, Thorvaldsdóttir H, Tamayo P, Mesirov JP. Molecular signatures database (MSigDB) 3.0. Bioinformatics. 2011; 27(12):1739-40.

**Ethics Approval**

This study was approved by Gustave Roussy Scientific Board, the French National Health Agency and Ethical Committee (ID-RCB : 2014-A01147-40)

#### P286 In vivo genetic screens in PD-1 resistant mouse models identified modulators of anti-PD1 response with relevance to pembrolizumab-treated human metastatic melanoma

##### Aurélie Docquier, PhD, Cécile Déjou, Gilles Alberici, Armand Bensussan, PhD, Jeremy Bastid, Nathalie Bonnefoy, PhD

###### OREGA Biotech, Ecully, France

####### **Correspondence:** Jeremy Bastid (jeremy.bastid@orega-biotech.com)

**Background**

We previously reported the identification of CD39 as a regulator of anti-PD-1 response in combination with oxaliplatin in resistant models using our screening approach [1].

**Methods**

Similar approach was used to identify novel targets that modulate anti-PD-1 response. Here we report the discovery of a novel potent regulator of anti-PD-1 therapy in MCA205 fibrosarcoma and B16K1 melanoma syngeneic mouse models harboring no or low response rates to PD-1 immune checkpoint inhibitor.

**Results**

Remarkably, the Knock-Out (KO) of the identified target had limited, if any, impact on tumor growth in various mouse models. Whereas the mouse models used are broadly resistant to anti-PD1 therapy, treatment in the KO background markedly improved the efficacy of anti-PD-1 mAb by increasing the response rates and by increasing the rate of complete vs partial responses, which translated into improved mouse survivals. We generated various human-mouse cross-reactive blocking antibodies to the target, including a humanized mAb. The neutralizing antibodies mimicked the KO phenotype and markedly improved the response to anti-PD1 therapy in preclinical mouse models. The mechanism of action is being investigated. We found that the expression of target within the tumor induces an immunosuppressive tumor immune microenvironment by upregulating several immunoregulatory cytokines and chemokines. Interestingly, we re-analyzed transcriptomic data from 28 metastatic melanoma tumors prior to anti-PD-1 pembrolizumab therapy [2] to validate our target and related signaling pathways. We found a stepwise increased expression of our target, its receptor and the identified targets of the pathway from complete responders (low expression) to partial responders and non-responders (high expression), thereby suggesting that the identified target is relevant to the clinical situation.

**Conclusions**

The mAb is currently under preclinical development as a novel immunotherapeutic antibody to overcome resistance to anti-PD-1 immune checkpoint blockade.

**References**

1. Perrot I, Michaud HA, Giraudon-Paoli M, et al. Blocking Antibodies Targeting the CD39/CD73 Immunosuppressive Pathway Unleash Immune Responses in Combination Cancer Therapies. Cell Rep. 2019 May 21;27(8):2411-2425.e9. doi: 10.1016/j.celrep.2019.04.091

2. Hugo W, Zaretsky JM, Sun L et al. Genomic and Transcriptomic Features of Response to Anti-PD-1 Therapy in Metastatic Melanoma. Cell. 2016 Mar 24;165(1):35-44. doi: 10.1016/j.cell.2016.02.065. Epub 2016 Mar 17.

#### P287 A novel bispecific checkpoint inhibitor antibody to preferentially block PD-1 and LAG-3 on dysfunctional TILs whilst sparing Treg activation

##### Laura Codarri Deak^1^, Patrick Weber, Mr^1^, Stefan Seeber, Dr^2^, Mario Perro, Dr^1^, Xavier Miot, Mr^1^, Heidi Poulet, Mrs^1^, Iryna Dekhtiarenko, Dr^1^, Matthias Füth^3^, Henry Kao, Dr^3^, Christine McIntyre, Dr^4^, Francesca Michielin, Dr^3^, Christian Klein, Dr rer nat^1^, Pablo Umana, PhD^1^, Lin-Chi Chen, Dr^5^, Christoph Markert, Dr^2^, Merlind Mücke, Dr^3^

###### ^1^Roche Innovation Center Zurich, Zurich, Switzerland; ^2^Roche Innovation Center Munich, Munich, Germany; ^3^Roche Innovation Center Basel, Basel, Switzerland; ^4^Roche Innovation Center Welwyn, Welwyn, United Kingdom; ^5^Roche Innovation Center New York, New York, NY, United States

####### **Correspondence:** Laura Codarri Deak (laura.codarri_deak@roche.com)

**Background**

Check point inhibitors targeting PD-1 have shown unprecedented clinical efficacy in several cancer indications and therefore have, revolutionized the standard of care. However, despite this advancement in cancer immunotherapy, only ~20-30% of the patients derive durable benefit from such a treatment. One of the suggested reasons for this limited success is the expression/activation of compensatory inhibitory pathways such as LAG-3 on tumor-reactive T cells. These pathways compensate for the loss of function of PD-1 upon its blockade. Therefore, it is envisioned that simultaneous antagonism of PD-1 and LAG-3 receptors would overcome this adaptive resistance mechanism and allow a more profound reinvigoration of dysfunctional tumor-reactive T cells. Conversely, a recent report has highlighted that blockade of LAG-3 on regulatory T cells (Tregs) increases their suppressive function and, therefore may off-set its benefit on the reinvigoration of dysfunctional tumor-reactive T cells.

**Methods**

We therefore developed a 1+1 PD1-LAG3 bispecific antibody (BsAb) with a 10-20 fold higher affinity for PD-1 than for LAG-3, allowing an avidity driven selectivity gain to PD-1 and LAG-3 double positive T cells.

**Results**

Hence, PD1-LAG3 BsAb is assumed to have the following advantages over monospecific and other bispecific aPD/L1 and aLAG-3 antibodies: 1) improved targeting to dysfunctional T cells rather than Tregs due to the selectivity gain and different expression patterns of PD-1 and LAG-3 on these two T cell types, 2) reduced internalization, 3) Fc silent-mediated resistance to drug-shaving by macrophages.

**Conclusions**

These characteristics translated in a significant increase in 1) in vitro T cell effector functions even in the presence of Tregs, and 2) in vivo tumor control/eradication in mouse models compared to combination of monospecific anti-PD1 and anti-LAG3 antibodies.

#### P288 CCR5+CTLA4+ Treg cell subset characterizes renal tumors (RCC) immunosuppressed by PD1 blockade

##### Agathe Dubuisson^1^, Agathe Dubuisson^1^, Charles Bayard^1^, Sebastien Lofek^1^, Nicolas Voisin^1^, Séverine Mouraud^1^, Delphine Bredel^1^, Sandrine Susini^1^, Mathieu Rouanne, MD^1^, Hervé Beaumert^1^, Bastien Parier^2^, Aurélien Marabelle, MD PhD^1^, Laurence Zitvogel, MD, PhD^1^, Mélodie Bonvalet^1^, Mélodie Bonvalet^1^

###### ^1^Gustave Rousy, Villejuif, France; ^2^APHP, Le Kremlin Bicetre, France

####### **Correspondence:** Mélodie Bonvalet (bonvaletmelodie@gmail.com)

**Background**

A small subset of patients undergoing PD1 blockade therapy develop a hyper-progressive disease (HPD), corresponding to an acceleration of tumor growth rate [1]. However, the mechanisms underlying HPD are unknown, and the identification of predictive biomarkers remains an unmet clinical need. We aimed at better understanding the mechanism of hyper-progression following nivolumab treatment by examining the tumor immune infiltrate of fresh RCC using an ex vivo model system.

**Methods**

Fifteen fresh primary RCC were processed according to methods previously reported [2] and stimulated for 3 days with nivolumab. At baseline, we monitored the phenotype of the immune infiltrate by flow cytometry. After 3 days of culture, we measured 26 soluble factors which were released into the supernatant. We then examined the relationship between baseline phenotype and functional immune reactivity to nivolumab, considering variations of at least 2 independent soluble factors in the range of

**Results**

Nivolumab induced a strong inhibition of soluble factor release in 5 out of 15 RCC. The decreased soluble factors were IL1RA (3 out of 5 tumors (3/5)), IFNg, CXCL10, IL10, G-CSF, GM-CSF (2/5), IL-4, IL-5, IL-6, IL-8, IL-9, CCL4, CCL5, PDGFbb (1/5). In these hypo-sensitive tumors (HS), nivolumab induced a significant decrease of IL-4, IL-8, G-CSF (p<0.05).

**Conclusions**

The TME of HS renal tumors that are immunosuppressed by nivolumab display high levels of the CCR5 ligands and CCR5+ CTLA4+ Treg cells, compared to NHS tumors. As reported by Kamada et al. [3], distinct Treg subset might be involved in the deleterious effects of nivolumab observed in HPD. Prospective clinical studies should determine if nivolumab-associated HPD is caused by a pre-existing pool of highly immunosuppressive CCR5+ CTLA4+ Treg cells.

**Acknowledgements**

This work was supported by Bristol-Myers Squibb.

**References**

1. Champiat S, Dercle L, Ammari S, Massard C, Hollebecque A, Postel-Vinay S, Chaput N, Eggermont A, Marabelle A, Soria JC, Ferté C. Hyperprogressive Disease Is a New Pattern of Progression in Cancer Patients Treated by Anti-PD-1/PD-L1. Clin Cancer Res. 2017;23(8):1920-1928.

2. Jacquelot N, Roberti MP, Enot DP, Rusakiewicz S, Ternès N, Jegou S, Woods DM, Sodré AL, Hansen M, Meirow Y, Sade-Feldman M, Burra A, Kwek SS, Flament C, Messaoudene M, Duong CPM, Chen L, Kwon BS, Anderson AC, Kuchroo VK, Weide B, Aubin F, Borg C, Dalle S, Beatrix O, Ayyoub M, Balme B, Tomasic G, Di Giacomo AM, Maio M, Schadendorf D, Melero I, Dréno B, Khammari A, Dummer R, Levesque M, Koguchi Y, Fong L, Lotem M, Baniyash M, Schmidt H, Svane IM, Kroemer G, Marabelle A, Michiels S, Cavalcanti A, Smyth MJ, Weber JS, Eggermont AM, Zitvogel L. Predictors of responses to immune checkpoint blockade in advanced melanoma. Nat Commun. 2017 ;8(1):592.

3. Kamada T, Togashi Y, Tay C, Ha D, Sasaki A, Nakamura Y, Sato E, Fukuoka S, Tada Y, Tanaka A, Morikawa H, Kawazoe A, Kinoshita T, Shitara K, Sakaguchi S, Nishikawa H. PD-1+ regulatory T cells amplified by PD-1 blockade promote hyperprogression of cancer. Proc Natl Acad Sci U S A. 2019 May 14;116(20):9999-10008.

**Ethics Approval**

The study was approved by the Comité de Protection des Personnes Ile de France III Ethics Board, approval number 2016-A00732-49.

#### P289 Macrophages modulate patient response to immune checkpoint inhibition in a novel lung tumour explant model

##### Lauren Evans, BSc, MSc^1^, Kate Milward, DPhil^1^, Richard Attanoos, BSc, MB BS, FRCPath^2^, Aled Clayton, PhD^1^, Rachel Errington, PhD^1^, Zsuzsanna Tabi, PhD^1^

###### ^1^Cardiff University, Cardiff, United Kingdom; ^2^University Hospital Wales, Cardiff, United Kingdom

####### **Correspondence:** Lauren Evans (Evansl57@cardiff.ac.uk)

**Background**

The tumour microenvironment (TME) consists of a dynamic interplay between the tumour and stroma. Various stroma-resident and tumour-infiltrating immune cells are associated with pro-tumour activity. Whilst immunotherapies may trigger anti-tumour immune responses, anti-inflammatory M2-like tumour-associated macrophages (TAMs) within the TME present an obstacle for effective treatment [1]. Pre-clinical research has predominantly used mouse models to represent the in vivo TME. Unfortunately, such models do not faithfully replicate the human immune system therefore, providing an inadequate measure of immunotherapy response. Human tumour-derived explants maintain the original 3D tumour architecture and combination of multiple cell types. Therefore, we established an ex vivo tumour explant model of non-small cell lung cancer (NSCLC) incorporating TAMs, to determine their role in immunotherapeutic response.

**Methods**

Tumour explants (ca. 1mm³) were generated from fresh tumour tissue. Autologous CD14^+^ peripheral blood mononuclear cells (PBMCs) were added to explants for 48h followed by flow cytometry phenotyping using a 7-marker macrophage panel (CD14; CD64; PDL-1; CD163; CD206; CD23; CD200R). The functional contribution of macrophages to explant-mediated immunosuppression was assessed through measuring IFNγ/TNFα production from CD4^+^/CD8^+^ T cells by flow cytometry. T cells, in the presence of explants, were stimulated with a viral peptide pool and incubated for 6 days ±250μg/mL Atezolizumab, prior to intracellular cytokine staining. Culture supernatants were collected and the Th1/Th2 cytokine profile determined using a LEGENDplex bead-based immunoassay (BioLegend). Transcriptomic analysis was performed on patient tumour tissue and peripheral blood using the NanoString PanCancer IO360 gene expression panel.

**Results**

Tumour explants significantly promoted M2-like macrophage differentiation and suppressed T cell activity ex vivo. The PDL-1 inhibitor, Atezolizumab significantly improved T cell function in some patients and reduced explant-mediated immunosuppression, particularly in the presence of macrophages. This suggests that response or resistance to anti-PDL-1 therapies may be partially TAM dependent. The differential effect of Atezolizumab observed in the presence of patient-derived tumour explants indicates the potential of this model in predicting immunotherapy responses. Ongoing transcriptomic analysis of lung cancer tissue aims to reveal associations between the TME and T cell function. Variations in the cytokine secretion profile of tumour explant co-cultures are also being studied under different experimental conditions.

**Conclusions**

Using the tumour explant model, we found that alleviation of explant-mediated immunosuppression by Atezolizumab may be macrophage dependent. This model could be used to predict patient response to anti-PDL-1 immunotherapy, and explore combination therapies. Ongoing research aims to improve immunotherapy responses through reprogramming TAMs using immune-modifying drugs.

**Acknowledgements**

Study supported by research funds from Cardiff University, School of Medicine. We would like to thank the Wales Cancer Bank and Lung Multidisciplinary Research Group for their ongoing involvement in patient recruitment and sample acquisition, and to the patients who donated their samples for our research.

**Reference**

1. Cassetta L, Kitamura T. Targeting Tumor-Associated Macrophages as a Potential Strategy to Enhance the Response to Immune Checkpoint Inhibitors. 2018;6.

**Ethics Approval**

Ethical approval for this project was provided through the Wales Cancer Bank (WCB). The WCB has ethics approval as a Research Tissue Bank from the Wales Research Ethics Committee 3, reference 16/WA/0256 (previous approval references – 06/MRE09/26 and 11/WA/0279). This approval covers the collection of samples (including consent), processing and storing samples across multiple collection and storage sites. The approval also allows release of anonymised samples to researchers carrying out cancer related activity, following successful application approval from the WCB External Review panel (project 17/016). Sample collection of blood and tissue, from non-small cell lung cancer patients undergoing surgical resection, was performed at the University Hospital of Wales, Cardiff.

#### P290 Interferon gamma production by regulatory T cells is necessary for response to cancer immunotherapy

##### Angela Gocher, PhD, Dario Vignali, PhD, Creg Workman, PhD, Sanah Handu

###### University of Pittsburgh, Pittsburgh, PA, United States

####### **Correspondence:** Dario Vignali (dvignali@pitt.edu)

**Background**

Our lab has shown that murine regulatory T cells (Tregs) produce the cytokine interferon gamma (IFNγ) during anti-PD1 therapy and that Treg response to IFNγ is necessary for tumor eradication by anti-PD1 therapy [1]. However, the role of this cellular source of IFNγ in the tumor microenvironment (TME) and how Treg derived IFNγ dictates response to other cancer immunotherapies has yet to be studied. In addition, it has been shown that IL-12-induced production of IFNγ is necessary for response to anti-PD1 therapy [2]. However, it has yet to be determined whether Tregs are a key responder to IL-12 during cancer immunotherapy. Thus, elucidating the interplay of IL12, IFNγ and Tregs in the TME is essential for maximizing efficacy and minimizing the clinical resistance to current cancer immunotherapies.

**Methods**

Our lab has generated two novel murine models that allow for Treg-restricted deletion of Ifng (IfngL/LFoxp3Cre-YFP) or Il12rb2 (Il12rb2L/LFoxp3Cre-YFP). These murine models were validated and used to assess the contribution of Treg-derived IFNγ on the growth of syngeneic MC38 (colon adenocarcinoma) and B16 (melanoma) tumors and response to a various cancer immunotherapies (checkpoint blockade, vaccination and tumor-specific antibodies). Flow cytometry was conducted on tumor and control non-draining lymph nodes to phenotype the immune infiltrate in response to immunotherapy.

**Results**

Mice with Tregs either unable to generate IFNγ (IfngL/LFoxp3Cre-YFP) or respond to the IFNγ-inducing cytokine IL-12 (Il12rb2L/LFoxp3Cre-YFP) were unable to eradicate tumors in response to immunotherapy. In addition, preliminary results suggest that Il12rb2-deficient Tregs may be more suppressive as indicated by an increase in tumor growth compared to control.

**Conclusions**

These data suggest that IFNγ producing Tregs are necessary to shift the balance from an immunosuppressive TME to one that favors the reinvigoration of the anti-tumor response generated by current cancer immunotherapeutic agents. Future studies will determine whether the capacity of Tregs to produce IFNγ can predict response to immunotherapy.

**References**

1. Overacre-Delgoffe A, Chikia M. Interferon-γ Drives Treg Fragility to Promote Anti-tumor Immunity. Cell. 2017; 169: 1130-1141.

2. Garris C, Arlauckas S. Successful Anti-PD1 Cancer Immunotherapy Requires T Cell-Dendritic Cell Crosstalk Involving the Cytokines IFN-γ and IL-12. Immunity. 2018; 49: 1148-1161.

#### P291 Driving T cell dysfunction in vitro for rational immunotherapy design

##### Matthew Hancock, Cailin Joyce, PhD, Thomas Horn, Simarjot Pabla, Benjamin Duckless, Andrew Basinski, Dhan Chand, PhD, Jeremy Waight, PhD, Mariana Manrique, PhD, Nicholas Wilson, Alex Duncan, PhD, Jennifer Buell, PhD, David Savitsky, PhD, Lukasz Swiech, John Castle, PhD

###### Agenus Inc, Lexington, MA, United States

####### **Correspondence:** John Castle (john.castle@agenusbio.com)

**Background**

Immune checkpoint blockade (ICB) elicits durable responses in some cancer patients, but novel targets and combination approaches are needed to address resistance and broaden clinical benefit. Here, we present a system for characterizing mechanisms of T cell dysfunction in the tumor microenvironment (TME) and apply the system to uncover novel approaches to ICB resistance.

**Methods**

We developed a long-term human co-culture system comprised of primary T cells and cancer cells that enables controlled differentiation of naïve T cells to effector, memory and dysfunctional states. We longitudinally monitored T cell effector functions, protein and RNA expression across states and single cells. Finally, we challenged the system with PD1 antibody to uncover biomarkers and mechanisms of therapeutic resistance.

**Results**

T cells in our system become activated and then gradually progress to a terminally dysfunctional state driven by multiple cancer antigen exposures. T cell cytotoxicity is maintained over several antigen exposures before sharply decreasing whereas cytokine secretion begins to decrease with only one prior antigen exposure. The expression of known T cell regulators and novel factors is altered over the time course, with known factors reflecting previous observations in vivo. Anti-PD1 prolongs cytotoxic capacity but T cells eventually fail to respond. Single cell mapping in the presence of anti-PD1 reveals an expanded population of T cells that co-express PD1, TIGIT and activation markers. Consistent with this, the combination of PD1 and TIGIT blockade enhances cytotoxicity relative to monotherapies.

**Conclusions**

These findings demonstrate the utility of our system to deeply interrogate therapeutic response and resistance. Moreover, its scalability and modularity enable genome-scale screening to discover novel targets that could enhance antitumor activity of both natural and adoptively transferred T cells.

#### P292 Tissue site of tumor growth dictates anti-tumor immunity and response to checkpoint blockade

##### Brendan Horton, PhD, Stefani Spranger, PhD

###### Massachusetts Institute of Technology,, Cambridge, MA, United States

####### **Correspondence:** Stefani Spranger (spranger@mit.edu)

**Background**

An emerging area of clinical importance is differential responsiveness to checkpoint blockade immunotherapy across different tissues sites of tumor growth, leading to partial responses and ultimately cancer-related deaths [1, 2]. However, tissue-specific anti-tumor immune responses are not well understood. We used mouse models to determine how anti-tumor immune responses differ across tissue sites and how this relates to immunotherapy efficacy.

**Methods**

Mice were inoculated subcutaneously or intravenously with syngeneic KP lung cancer cells, then treated with anti-CTLA-4 and anti-PD-L1 antibodies, and analyzed for tumor burden. Response to checkpoint blockade was correlated with tumor-infiltrating T cells (TIL) and systemic immune responses using flow cytometry and immuno-histochemistry. Mice were also tested for their ability to generate systemic and protective immunity against a second tumor challenge.

**Results**

Comparing lung and subcutaneous tumors, we observed striking differences within the TIL compartment. Lung tumors contained more TIL with higher expression of PD-1. Despite this, lung tumors did not respond to anti-CTLA-4 + anti-PD-L1, while subcutaneous tumors did. TIL expanded in subcutaneous tumors after immunotherapy, while lung tumors had no change in the number of TIL. We further tested for concomitant immunity and found that subcutaneous KP tumors generated an immune response that could protect against a second tumor. However, KP lung tumors failed to generate a protective systemic immune response. These results were reproduced using a pancreatic cancer cell line, indicating that tumors growing in the lung generated weaker systemic immune responses than subcutaneous tumors. Protective concomitant immunity required CD8+ T cells, and to a lesser extent CD4+ T cells, but not NK cells. Therefore, we investigated differences in the generation of systemic, antigen-specific CD8+ T cell responses between subcutaneous and lung KP tumors. Using KP.SIY tumors, elispot assays found that lung tumors generated fewer antigen-specific T cells in the spleen. Transferred 2C T cells proliferated less in the spleens of lung tumor-bearing hosts than in hosts with subcutaneous tumors, even though proliferation in draining lymph nodes was similar. Consistently, KP.SIY lung tumors generated weaker concomitant immunity than subcutaneous KP.SIY tumors.

**Conclusions**

The tissue site of tumor growth determines the number and phenotype of TIL, the generation of systemic immunity, and the response to checkpoint blockade. Response to immunotherapy correlates not with the number of TIL, or their phenotype, but with the ability of the host to mount a systemic anti-tumor CD8+ T cell response.

**References**

1. Khoja, L., et al., Patterns of response to anti-PD-1 treatment: an exploratory comparison of four radiological response criteria and associations with overall survival in metastatic melanoma patients. Br J Cancer, 2016. 115(10): p. 1186-1192.

2. Lee, J.H.J., et al., Metastasis-specific patterns of response and progression with anti-PD-1 treatment in metastatic melanoma. Pigment Cell Melanoma Res, 2018. 31(3): p. 404-410.

**Ethics Approval**

The animal work in these experiments was reviewed and approved by the MIT Committee on Animal Care. The approved animal protocol number for this work is 0117-012-20.

#### P293 Tumor mutational burden (TMB) and genomic predictors of clinical outcomes to PD-1/PD-L1 checkpoint blockade in high-grade gliomas

##### Bryan Iorgulescu, MD^1^, Mehdi Touat^1^, Yvonne Li^1^, Liam Spurr^1^, Gareth Grant^1^, William Pisano^1^, Mary Jane Lim-Fat^1^, Eudocia Lee^1^, Lakshmi Nayak^1^, E Chiocca^2^, Raymond Huang^2^, Andrew Cherniack^3^, Patrick Wen^1^, Ahmed Idbaih^1^, Franck Bielle^4^, David Reardon, MD^1^, Keith Ligon^1^

###### ^1^Dana-Farber Cancer Institute, Boston, MA, United States; ^2^Brigham and Women's Hospital, Boston, MA, United States; ^3^Broad Institute, Cambridge, MA, United States; ^4^Sorbonne Université, Paris (UPMC), Paris, France

####### **Correspondence:** David Reardon (david_reardon@dfci.harvard.edu); Keith Ligon (Keith_Ligon@dfci.harvard.edu)

**Background**

High tumor mutational burden (TMB) is an emerging biomarker for predicting response to PD-1/PD-L1 immune checkpoint blockade (ICB) in a spectrum of cancer patients, however, its clinical value and therapeutic implications in high-grade gliomas (HGG) is not yet established.

**Methods**

We retrospectively reviewed all HGGs at our institutions from 2013-2018 that underwent genomic characterization. TMB was determined from clinical targeted exome next-generation sequencing (DFCI-Profile, ~500 cancer causing genes) as the number of nonsynonymous coding mutations per megabase and the optimal cut-off for TMBhigh was determined using the 98th percentile of newly-diagnosed tumors and segmented linear regression analysis. Patients were stratified by histomolecular subtype, IDH mutation, 1p/19q co-deletion, TMB, and ICB treatment under clinical trials and expanded access. Overall (OS) and progression-free (PFS) survival were estimated by the Kaplan-Meier method and evaluated using multivariable Cox regression.

**Results**

We identified 1,223 HGG patients with genomics, including 64 hypermutated tumors. Overall the cohort consisted of 79% newly-diagnosed and 21% recurrent gliomas and subclass distribution was 75% IDH-wildtype glioblastomas/anaplastic astrocytomas,16% IDH-mutant glioblastomas/anaplastic astrocytomas, and 6% 1p/19-codeleted anaplastic oligodendrogliomas. Hypermutated HGG were predominantly seen in the setting of recurrence (18% of recurrent HGGs vs 2% of newly diagnosed, 5% overall incidence). Comparisons of biomarkers and genomics in hypermutated versus non-hypermutated HGGs showed distinct characteristics in glioma subtypes with implications for differential mechanisms of TMBhigh acquisition in HGGs with the most important biomarkers of differential hypermutation risk being MGMT promoter methylation (22% of methylated vs. 6% of unmethylated cases, p=0.02) and IDH1/2 mutation (25% of IDH-mutant vs. 12% of IDH-wildtype HGGs, p=0.007).

129 (11%) of HGGs (mostly IDH-wildtype GBMs/AAs) received PD-1/PD-L1 ICB therapy, including 13% (n=8) of hypermutated HGGs. Immunohistopathologic assessment of tumor responses and immune infiltrates was conducted and correlated with the genomic profiling. Because the majority of ICB-treated cases were IDH-wildtype HGGs, this subset was further evaluated in analyses which were risk-adjusted by age, sex, histomolecular subgroup, MGMT promoter methylation, and prior therapy (i.e. RT/TMZ/CCNU/bevacizumab). Correlations between TMB, molecular genotypes, and clinical response to ICB in HGGs will be presented.

**Conclusions**

Our study represents the largest set of genomically characterized gliomas and gliomas with hypermutation with data related to responses to PD-1/PD-L1 ICB therapy. Our data support systematic TMB characterization and clinical molecular genotyping to assist therapy-decision making in gliomas and provide foundational data essential to future clinical trial designs of immunotherapeutics.

**Ethics Approval**

This study was approved by the Partners HealthCare (#2015P002352) and Dana-Farber Cancer Institute (10-417) institutional review boards.

#### P294 Long survival associated with receipt of anti-CTLA-4 in patients with metastatic melanoma from acral lentiginous, mucosal and uveal primary tumors

##### Nicholas Klemen, MD, Melinda Wang, BS, Kelly Olino, MD, James Clune, MD, Stephan Ariyan, MD, Charles Cha, MD, Sarah Weiss, MD, Harriet Kluger, MD, Mario Sznol, MD

###### Yale University School of Medicine, New York, NY, United States

####### **Correspondence:** Mario Sznol (mario.sznol@yale.edu)

**Background**

Metastatic melanoma from acral lentiginous, mucosal and uveal primary tumors responds poorly to checkpoint inhibitors (CPI), potentially due to a low burden of immunogenic neoantigens [1-7]. Long-term outcomes of these patients after treatment with CPI have not been established.

**Methods**

We performed a retrospective review of a single institutional experience using antibodies against CTLA-4, PD-1 and PD-L1 for patients with stage IV melanoma. Primary tumor histology was categorized as cutaneous, acral, mucosal or uveal. Patients with unknown primary were excluded. We measured overall survival (OS) after the first dose of CPI using the Kaplan-Meier method.

**Results**

We treated 428 patients with metastatic melanoma from 2007-2019. Primary tumors were cutaneous in 283 (66%) patients, unknown in 55 (13%), acral in 22 (5%), mucosal in 38 (9%) and uveal in 30 (7%)(Table 1). Mucosal patients had a slight female preponderance. The proportion staged M1c was higher in mucosal and uveal patients. Patients with cutaneous primary tumors had median OS after CPI of 45 months, compared with 17 months for acral (P = 0.047), 18 months for mucosal (P = 0.003) and 12 months for uveal (P < 0.001)(Figure 1). Five-year survival for cutaneous, acral, mucosal and uveal patients was 46%, 34%, 21% and 22% respectively.

Next we combined the patients with acral, mucosal and uveal melanoma (n = 90) and performed survival analysis stratified by the first type of CPI treatment. Median OS after anti-PD-1 or anti-PD-L1 was 9 months, compared with 18 months after anti-CTLA-4 (P = 0.010) and 20 months after combination therapy with anti-CTLA-4 plus anti-PD-1 (P = 0.003)(Figure 2). While 21 of 31 (68%) patients treated with anti-CTLA-4 later were treated with anti-PD-1, only 5 of 18 (28%) patients treated with anti-PD-1 later received anti-CTLA-4 (P = 0.02). There were 21 patients who survived at least three years after CPI, all of whom were treated with anti-CTLA-4 with or without anti-PD-1. Of the 10 patients with actual five-year survival, 3 had complete responses while the other 7 all required local and/or regional therapies to control progressive disease (Table 2).

**Conclusions**

Long survival in patients with metastatic melanoma from acral, mucosal and uveal primary tumors was uniformly associated with receipt of anti-CTLA-4. Our experience shows that while acral, mucosal and uveal melanomas have worse outcomes than cutaneous melanoma, with an aggressive multidiscliplinary approach five-year survival is still possible for 25-32% of these patients.

**References**

1. Luke JJ, Callahan MK, Postow MA, Romano E, Ramaiya N, Bluth M, et al. Clinical activity of ipilimumab for metastatic uveal melanoma. Cancer. 2013;119:3687–95.

2. Algazi AP, Tsai KK, Shoushtari AN, Munhoz RR, Eroglu Z, Piulats JM, et al. Clinical outcomes in metastatic uveal melanoma treated with PD-1 and PD-L1 antibodies. Cancer. 2016;122:3344–53.

3. D’Angelo SP, Larkin J, Sosman JA, Lebbé C, Brady B, Neyns B, et al. Efficacy and Safety of Nivolumab Alone or in Combination With Ipilimumab in Patients With Mucosal Melanoma: A Pooled Analysis. JCO. 2017;35:226–35.

4. Postow MA, Luke JJ, Bluth MJ, Ramaiya N, Panageas KS, Lawrence DP, et al. Ipilimumab for Patients With Advanced Mucosal Melanoma. The Oncologist. 2013;18:726–32.

5. Bello DM, Chou JF, Panageas KS, Brady MS, Coit DG, Carvajal RD, et al. Prognosis of acral melanoma: a series of 281 patients. Ann Surg Oncol. Springer US; 2013;20:3618–25.

6. Krauthammer M, Kong Y, Ha BH, Evans P, Bacchiocchi A, McCusker JP, et al. Exome sequencing identifies recurrent somatic RAC1 mutations in melanoma. Nat Genet. Nature Publishing Group; 2012;44:1006–14.

7. Lawrence MS, Stojanov P, Polak P, Kryukov GV, Cibulskis K, Sivachenko A, et al. Mutational heterogeneity in cancer and the search for new cancer-associated genes. Nature. 2013;499:214–8.

**Ethics Approval**

The study was approved by the Yale-New Haven Hospital Institutional Review Board, approval number 2000021595.

**Table 1 (abstract P294).**
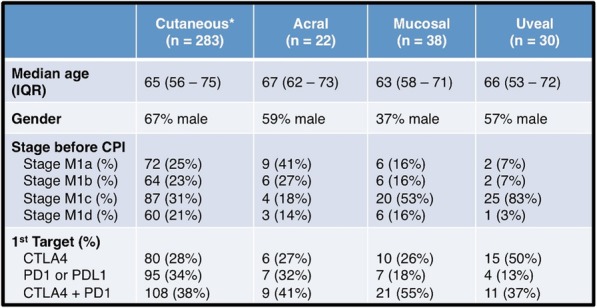
Demographics and first treatment

**Fig. 1 (abstract P294).**
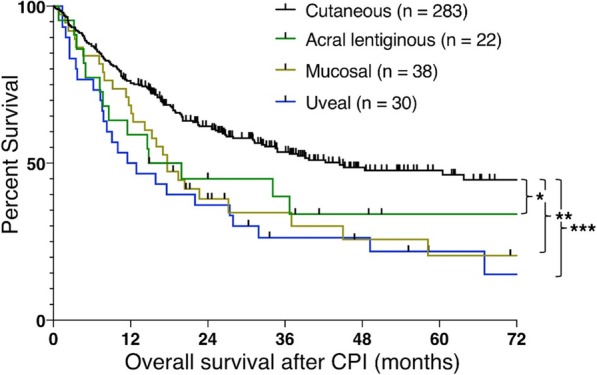
Overall survival stratified by histology

**Fig. 2 (abstract P294).**
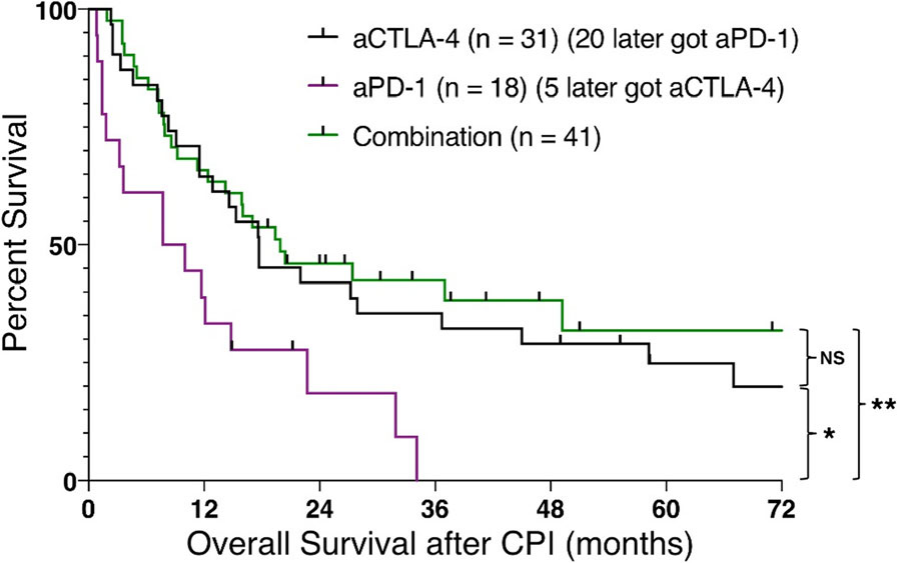
Overall survival stratified by treatment

**Table 2 (abstract P294).**
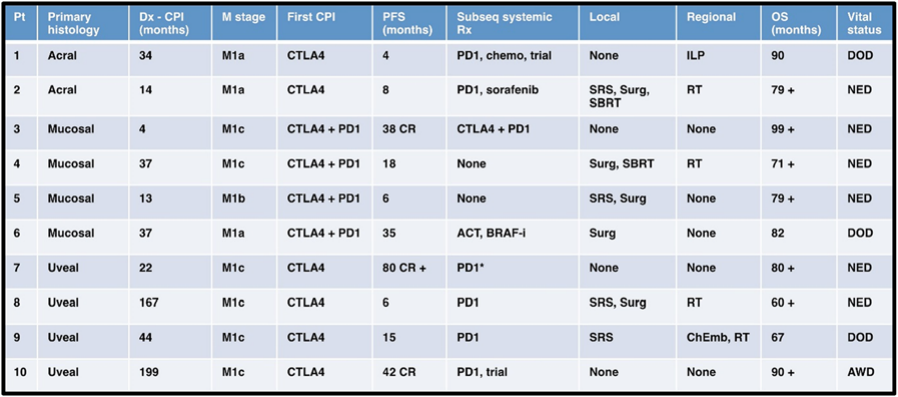
Characteristics of five-year survivors

#### P295 The impact of metastatic sites on checkpoint inhibitor outcomes in patients with cutaneous and unknown primary melanoma

##### Penina Krieger, MPhil, Francisco Sanchez-Vega, Nikolaus Schultz, Alexander Shoushtari, MD

###### Memorial Sloan Kettering Cancer Center, Bronx, NY, United States

####### **Correspondence:** Penina Krieger (peninakrieger@gmail.com)

**Background**

Clinical biomarkers of response to Programmed Death-1 (PD-1) based therapies are sorely needed in melanoma. The American Joint Committee on Cancer (AJCC) 8th Edition Staging system is derived from the pre-checkpoint inhibitor era and divides patients into locally advanced or soft tissue disease (M0/M1a), lung metastases (M1b), other visceral metastases (M1c), and brain metastases (M1d). It is unclear whether this prognostic classification remains valid in the modern therapeutic era. The number of metastatic sites influences outcomes with BRAF-MEK therapy [1], but its prognostic importance with PD-1-based therapy is unknown.

**Methods**

All patients with melanoma who had prospective tumor molecular profiling at a single center (MSK-IMPACT) and subsequently received frontline PD-1 blockade as single agent (Nivolumab or Pembrolizumab) or combination therapy with Ipilimumab (combo) were included. Demographic and clinical data were collected, including metastatic sites present at time of PD-1 therapy. Overall survival (OS) and time to treatment failure (TTF) were calculated from the onset of PD-1 therapy using Kaplan-Meier methodology. The impact of specific metastatic sites on TTF and OS was determined using Cox Proportional Hazards Regression Models. Tumor mutational burden (TMB) was calculated as previously described [2].

**Results**

309 patients received frontline PD-1 monotherapy (n=179) or PD-1 combo (n=130). Overall survival varied by AJCC stage (p<0.0001, Figure 1); M1b and M0/M1a groups had similar OS (median=NR, p = 0.27) followed by M1c (median=39 mo) and M1d (median=28 mo). Patients with 3+ metastatic sites had worse median OS than those with 0-2 metastatic sites (45 vs 39 mo, p<0.0001, Figure 2).

Among patients with M1c disease, those with bone or liver metastases had worse median OS than those without them (39 vs NR mo, Liver HR=2.4, p=0.036, Bone HR=2.6, p=0.021, Figure 3). Among patients with M1d disease, those with liver metastases had shorter TTF than those without when treated with PD-1 combination therapy (HR=2.4, p=0.044). There was no significant difference in median TMB by AJCC M stage at treatment or by site of metastasis.

**Conclusions**

AJCC 8th edition M1b disease has a similar prognosis to M0/M1a disease in the era of PD-1-based therapy. The presence of liver or bone metastases at time of PD-1 therapy portends worse OS and TTF even within M1c and M1d disease. Patients with 0-2 metastatic sites live longer than those with 3+ sites. These clinical observations are not explained by differences in TMB. Future trials of PD-1 blockade in advanced melanoma should account for these differences.

**Acknowledgements**

Research reported in this abstract was supported by the National Cancer Institute of the National Institutes of Health under Award Number R25CA020449 and by National Cancer Institute Cancer Center Core Grant P30CA008748, Kravis Center for Molecular Oncology. The content is the responsibility of the authors and does not necessarily represent the official views of the National Institutes of Health.

**References**

1. Long G, Grob J, Nathan P, Ribas A, Robert C, Schadendorf D, Lane S, Mak C, Legenne P, Flaherty K, Davies M. Factors predictive of response, disease progression, and overall survival after dabrafenib and trametinib combination treatment: a pooled analysis of individual patient data from randomised trials. Lancet Oncol. 2016; 17:1743–54.

2. Samstein RM, Lee CH, Shoushtari AN, Hellmann MD, Shen R, Janjigian YY, Barron DA, Zehir A, Jordan EJ, Omuro A, Kaley TJ, Kendall SM, Motzer RJ, Hakimi AA, Voss MH, Russo P, Rosenberg J, Iyer G, Bochner BH, Bajorin DF, Al-Ahmadie HA, Chaft JE, Rudin CM, Riely GJ, Baxi S, Ho AL, Wong RJ, Pfister DG, Wolchok JD, Barker CA, Gutin PH, Brennan CW, Tabar V, Mellinghoff IK, DeAngelis LM, Ariyan CE, Lee N, Tap WD, Gounder MM, D’Angelo SP, Saltz L, Stadler ZK, Scher HI, Baselga J, Razavi P, Klebanoff CA, Yaeger R, Segal NH, Ku GY, DeMatteo RP, Ladanyi M, Rizvi NA, Berger MF, Riaz N, Solit DB, Chan TA, Morris LGT. Tumor mutational load predicts survival after immunotherapy across multiple cancer types. Nat Genet. 2019; 51:202–206

**Ethics Approval**

The study was approved by Memorial Sloan Kettering's Ethics Board, approval number 18-244.

**Fig. 1 (abstract P295).**
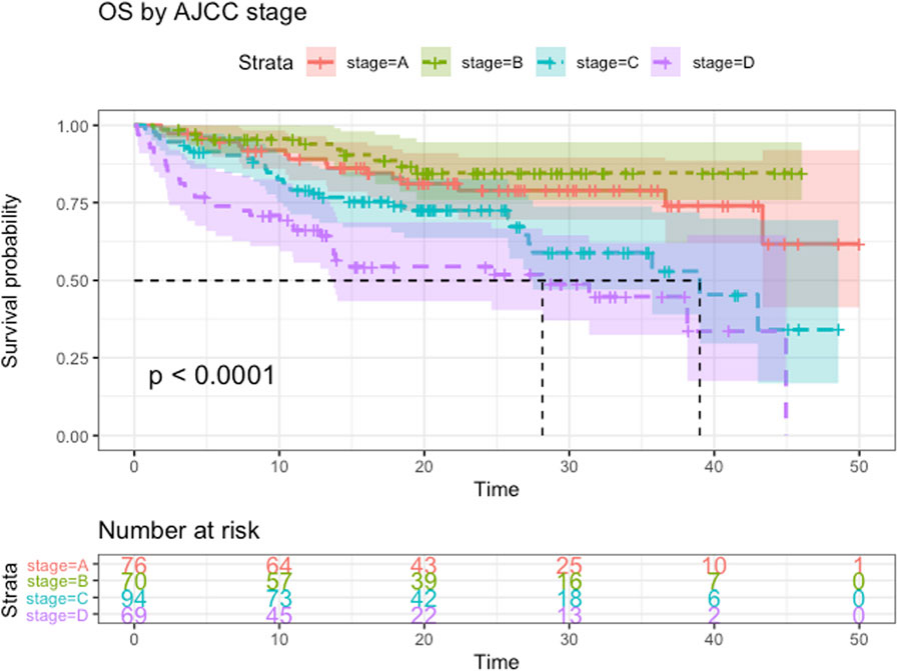
See text for description

**Fig. 2 (abstract P295).**
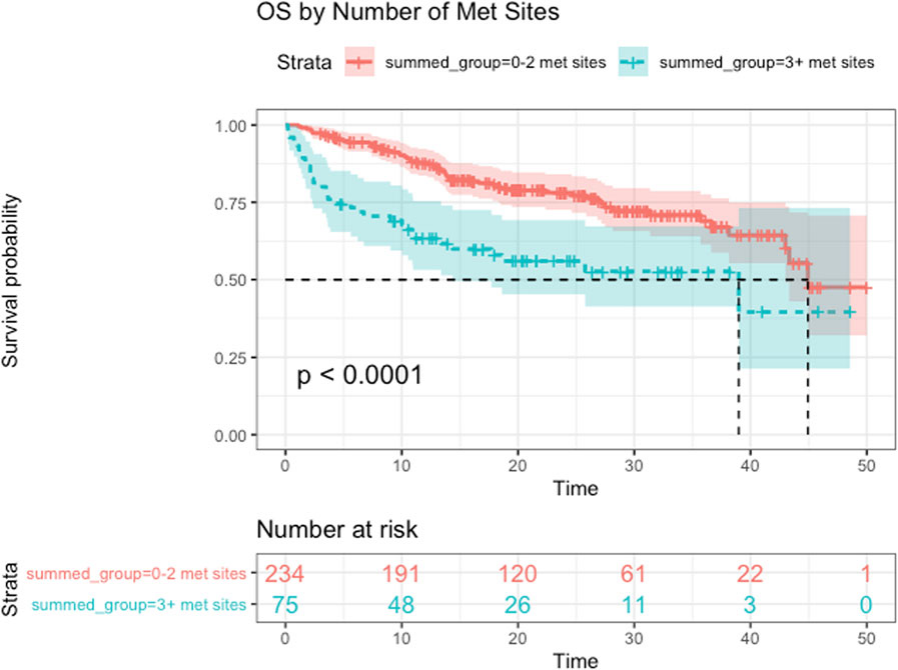
See text for description

**Fig. 3 (abstract P295).**
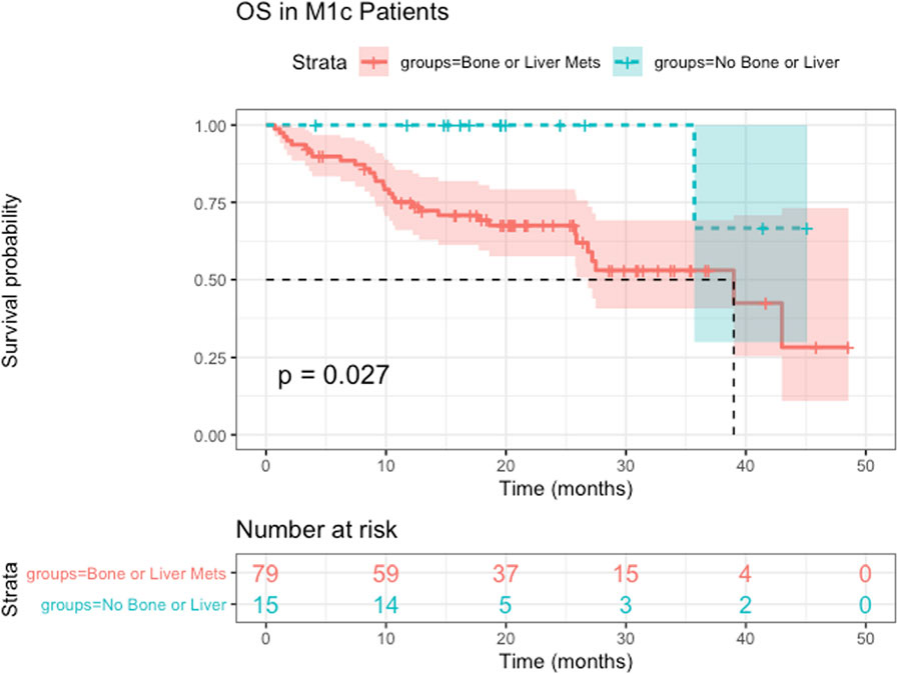
See text for description

#### P296 BMI, irAE and gene expression signatures predict resistance and survival to immune-checkpoint inhibition in renal cell carcinoma

##### Brian Labadie, MD^1^, Ping Liu, PhD^1^, Riyue Bao, PhD^1^, Michael Crist, MD^2^, Ricardo Fernandes, MD^3^, Laura Ferreira Freire^4^, Scott Graupner^5^, Andrew Poklepovic, MD^5^, Ignacio Duran^4^, Saman Maleki Vareki^3^, Arjun Balar, MD^6^, Jason Luke, MD, FACP^7^

###### ^1^University of Chicago, Chicago, IL, United States; ^2^NYU School of Medicine, New York, NY, United States; ^3^Western University, London, Ontario, Canada; ^4^HUMV, Santander, Spain; ^5^Virginia Commonwealth University, Richmond, VA, United States; ^6^New York University, New York, NY, United States; ^7^University of Pittsburgh, Pittsburgh, PA, United States

####### **Correspondence:** Jason Luke (lukejj@upmc.edu)

**Background**

Treatment with immune-checkpoint inhibition (ICI) has changed the treatment paradigm in ccRCC however many do not respond to these treatments and no reliable molecular biomarker exists to predict response to ICI in individual patients. Clinical variables may correlate with lack of response to treatment (primary resistance) or clinical benefit.

**Methods**

Via an international multi-institution collaboration, clinical characteristics from patients with ccRCC treated with anti-PD-1/L1 therapy were collected. Patients with primary resistance (defined as progression on initial computed tomography scan) were compared to patients with clinical benefit. Multivariable analysis was performed to identify factors associated with improved time to progression or death. The Cancer Genome Atlas Kidney Renal Clear Cell Carcinoma cohort (TCGA-ccRCC) was examined for the correlation between gene expression patterns, clinical factors, and survival outcomes.

**Results**

Of 90 patients, 38 (42.2%) had primary resistance and 52 (57.8%) had clinical benefit. Compared with the cohort of patients with initial benefit, primary resistance was more likely to occur in patients with worse ECOG performance status (p=0.03), earlier stage at diagnosis (p=0.04), no prior nephrectomy (p=0.04) and no immune-related adverse events (irAE) (p=0.02). In the entire cohort, improved overall survival was significantly correlated with lower International Metastatic RCC Database Consortium risk score (p=0.02) and lower neutrophil:lymphocyte ratio (p=0.04). In patients with clinical benefit, improved progression free survival was significantly associated with increased BMI (p=0.007) and irAE occurrence (p=0.02) while improved overall survival was significantly correlated with overweight BMI (BMI 25-30)(p=0.03) and no brain metastasis (p=0.005). In the TCGA-ccRCC analysis, higher expression of angiogenesis gene signature was found to be correlated with lower neoplasm histologic grade and better survival (p < 0.05). Angiogenesis and T cell-inflamed gene signatures were inversely correlated in tumors of high T cell-inflamed gene expression (p=0.008), a pattern not observed in non-T cell-inflamed tumors. (Figure 1)

**Conclusions**

Identification of BMI, performance status and prior nephrectomy as predictors of response to PD1/L1 in ccRCC may help inform treatment selection. The inverse association of angiogenesis gene signatures with ccRCC histologic grade highlight opportunities for adjuvant combination VEGFR2 TKI and ICI.

**Fig. 1 (abstract P296).**
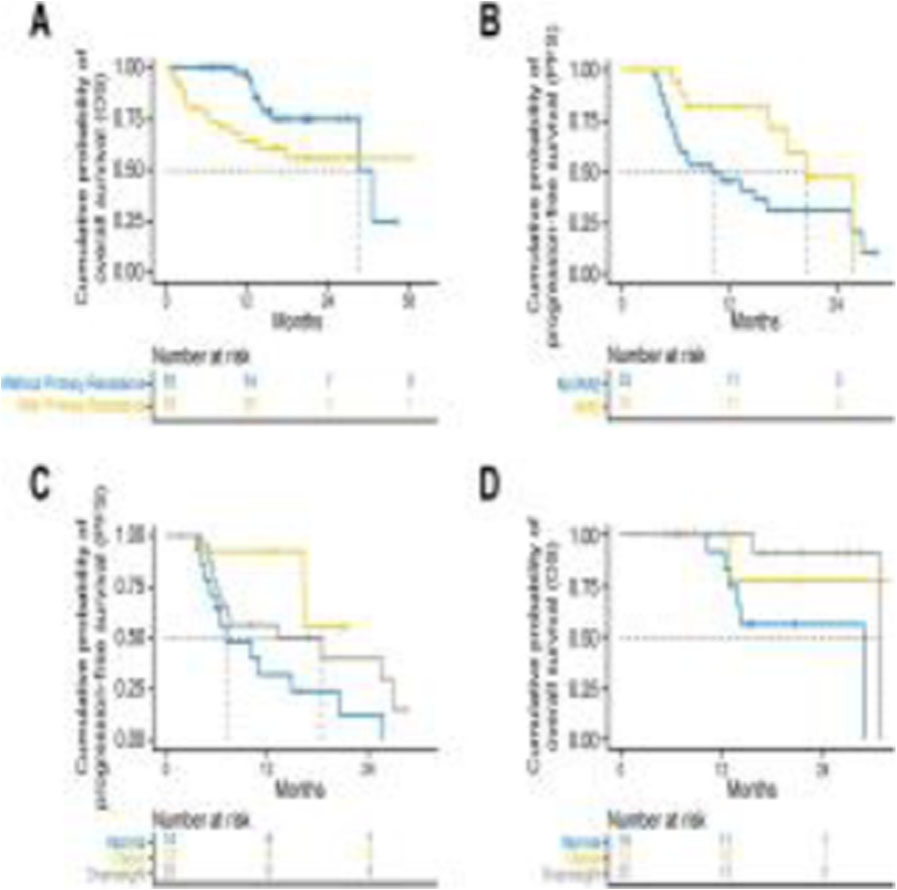
Kaplan-Meier curves depicting survival outcomes

#### P297 A semi-mechanistic platform model to capture individual animal responses to checkpoint inhibitors in a syngeneic mouse model

##### Lin Lin, PhD^1^, Alison Betts^2^, Carissa Young^1^, Wendy Qiao^2^, Jatin Narula^2^, Peter O’Brien^2^, Derek Bartlett^2^, Andrea Hooper^2^, Jason Williams^2^, John Burke^1^, Joshua Apgar^1^, Lore Gruenbaum, PhD^1^, Fei Hua, PhD^1^

###### ^1^Applied BioMath, LLC, Concord, MA, United States; ^2^Pfizer Worldwide R&D, Cambridge, MA, United States

####### **Correspondence:** Fei Hua (fei.hua@appliedbiomath.com)

**Background**

Syngeneic mouse models have been widely employed in preclinical discovery of checkpoint inhibitors as they enable study of drug impact on the intact immune system. However, the interpretation of such studies remains challenging partly due to the large variability in individual animal responses to drug treatment.

**Methods**

In this work, we describe the generation of a model platform that captures essential aspects of the pharmacokinetics, cellular and tumor growth effects of murine surrogates of two checkpoint therapeutic antibodies, anti-PD1 and anti-CTLA4, in the CT26 syngeneic tumor model. The model describes individual animal responses with regard to drug exposure, key intra-tumoral cell kinetics and tumor volume changes and provides biologically plausible explanations for the observed differences between good and poor responders to treatment with anti-PD1 or anti-CTLA4.

**Results**

We used the model to predict the antibody dose-response relationships for individual animals and to identify dose thresholds above which complete tumor elimination can be achieved in good responders. In contrast, our models predict that poor responders would not achieve complete response even with much higher drug doses. The parameters in our model that impact the response in poor responders are not drug-related. This finding suggests that immune-cell related barriers have to be crossed in order to achieve a therapeutic response in these animals - possibly via combination therapy.

In addition, we identified the net tumor cell doubling rate, one potential parameter that contributes to individual variability in response to treatment, as the most sensitive biological parameter determining tumor volume changes upon treatment with anti-PD1 or anti-CTLA4. Measuring individual animal tumor cell growth characteristics may help with the experimental design and qualification of animals for studies (in addition to absolute tumor volume), and thereby reduce inter-animal variability and enhance the interpretability of study results, especially in combination with a model such as the one presented here.

**Conclusions**

This model platform can be adapted to capture and compare checkpoint drug effects in different syngeneic tumor models. Moreover, it can be expanded to add additional drug mechanisms and can serve as a tool to inform the experimental design of mouse studies.

#### P298 PBRM1 loss defines distinct tumor phenotype associated with immunotherapy resistance in renal cell carcinoma

##### Xiande Liu, PhD^1^, Wen Kong^1^, Christine Peterson^1^, Daniel McGrail^1^, Anh Hoang^1^, Xuesong Zhang^1^, Truong Lam^1^, Patrick Pilie, MD^1^, Haifeng Zhu, PhD^1^, Kathryn Beckermann^2^, Scott Haake^2^, Sevinj Isgandrova^3^, Margarita Martinez-Moczygemba^3^, Nidhi Sahni^1^, W. Kimryn Rathmell^2^, Eric Jonasch, MD^1^

###### ^1^MD Anderson Cancer Center, Houston, TX, United States; ^2^Vanderbilt University Medical Center, Nashville, TN, United States; ^3^Texas A&M Health Science Center, Houston, TX, United States

####### **Correspondence:** Eric Jonasch (ejonasch@mdanderson.org)

**Background**

Polybromo-1 (PBRM1), encoding a mammalian specific subunit of the switch/sucrose non fermenting (SWI/SNF) chromatin remodeling complex, is the second most frequently mutated gene in clear cell renal cell carcinoma (ccRCC). Data thus far on the effect of PBRM1 loss on immune responsiveness are inconsistent. The impact of PBRM1 mutation on response to immunotherapy in patients with renal cell carcinoma (RCC) has become a topic of intense debate. RCC-specific mechanistic and large-scale clinical data are needed to precisely further characterize the influence of PBRM1 loss on response to immunotherapy.

**Methods**

An immunocompetent murine RCC model was applied to investigate the response to anti-PD-1 therapy. Multiple human RCC datasets (TCGA, IMmotion150 and ICGC), a murine pre-malignant dataset and a Renca tumor dataset were used to perform gene signature enrichment analysis (GSEA). Immunohistochemistry and Multiplex Opal Immunofluorescence were performed to assess immune cell infiltration. Real-time PCR, western blot, ELISA and chromatin immunoprecipitation (ChIP) were used to study the activity of the interferon gamma signaling pathway.

**Results**

Pbrm1 knockout in murine RCC Renca cells impaired the binding of brahma-related gene 1 (BRG1), the adenosine-triphosphate-dependent enzyme subunit of the SWI/SNF complex, to the promoter of IFN gamma receptor 2 (Ifngr2) and reduced Ifngr2 expression. PBRM1/Pbrm1 deficiency impaired IFN gamma-induced phosphorylation of Janus kinase 2 (JAK2) and STAT1, and the subsequent expression of downstream target genes involved in tumor microenvironment (TME) modulation, such as Cxcl9, Icam1, Irf1, and Stat1 itself. In both human and murine RCC tumors, PBRM1/Pbrm1 loss was associated with lower expression of immune-related profiles and reduced T cell infiltration. PBRM1/Pbrm1 loss tumors also demonstrated higher expression of angiogenesis-related profiles and increased CD31 expression. Furthermore, Pbrm1 deficient Renca subcutaneous tumors in mice demonstrated longer latency but more resistance to programmed death-1 (PD-1) blockade. Analysis of the IMmotion150 cohort revealed that ccRCC patients with PBRM1 mutations were associated with decreased immune infiltrates and a reduced response rate to atezolizumab monotherapy or combination therapy with bevacizumab.

**Conclusions**

Pbrm1 and PBRM1 loss reduced IFN gamma-STAT1 signaling. Pbrm1 inactivation was associated with a less immunogenic tumor microenvironment in animal and human tissue samples. Response to PD-L1 blockade is reduced in patients with PBRM1 mutations in the IMmotion 150 study. This study forms a framework for future mechanistic and clinical studies on the interaction between genomic features in RCC and response to immunotherapy.

**Acknowledgements**

We acknowledge the TCGA Research network. This work was supported by funding from DOD grant W81XWH-17.1.0307, DOD grant CA160728P1, UT MD Anderson Cancer Center CCSG grant 5 P30 CA016672 (Biostatistics shared resource group) and the Adopt-a-Scientist Foundation.

**Ethics Approval**

The animal protocols (2018-0376) were approved by Institutional Animal Care and Use Committee (IACUC) of The Health Science Center, Texas A&M University. Human subject protocol (2007-0511) was approved by Institutional Research Board at M.D. Anderson Cancer Center.

#### P299 Neuropilin-1 is a T cell memory checkpoint limiting long-term tumor immunity

##### Chang Liu, PhD^1^, Ashwin Somasundaram, MD^2^, Sasikanth Manne^3^, Angela Gocher, PhD^1^, Andrea Szymczak-workman^1^, Kate Vignali^1^, Daniel Normolle, PhD^2^, Robert Ferris, MD, PhD^1^, Tullia Bruno, PhD^1^, E. John Wherry, PhD^3^, Creg Workman, PhD^1^, Dario Vignali, PhD^1^

###### ^1^University of Pittsburgh, Pittsburgh, PA, United States; ^2^UPMC Hillman Cancer Center, Pittsburgh, PA, United States; ^3^University of Pennsylvania, Philadelphia, PA, United States

####### **Correspondence:** Dario Vignali (dvignali@pitt.edu)

**Background**

Robust CD8+ T cell memory is essential for long-term protective immunity but is often impaired in cancer due to T cell exhaustion, which causes a loss of memory precursors. Immunotherapy via checkpoint blockade does not effectively reverse this defect in the majority of patients, potentially underlying disease relapse. Resistance mechanisms that underlie poor CD8+ memory development remain unknown.

**Methods**

The development of post-surgical tumor immunity [1] was interrogated in the CD8+ T cell-restricted Neuropilin-1 (Nrp1)-deficient mice (E8ICreNrp1L/L), by surgically removing the primary B16F10 tumor followed by re-challenge 30- or 60-days post resection. The synergy between CD8-specific Nrp1 deficiency and anti-PD1 blockade was investigated with the MC38 tumor model. In a competitive setting, the Nrp1–/– and Nrp1+/+ pMel-T cells [2] were co-transferred into the same host (CD45.1) followed by gp100-B16 tumor inoculation, where the in vivo long-term persistence of the donor cells was assessed. The transcriptomic modulation by Nrp1 deficiency was studied using bulk population RNA sequencing (bpRNAseq) on the pMel-T cells (Nrp1–/– vs. Nrp1+/+) recovered from various phase of in vivo activation (effector, memory and recall). Lastly, the physiological relevance of NRP1 expression on CD8+ T cells in cancer patients was studied in a cohort of peripheral blood leukocyte (PBL) samples from treatment-naïve patients with head and neck squamous cell carcinoma (HNSCC).

**Results**

The E8ICreNrp1L/L mice exhibited substantially enhanced protection from tumor re-challenge, despite unchanged primary tumor growth. Enhanced responsiveness to anti-PD1 immunotherapy was also observed. NRP1 was co-expressed with multiple inhibitory receptors (IRs) on CD8+ T cells and restrained memory differentiation and development by repressing an Id3-dependent transcriptional program. NRP1 was also highly expressed on the exhausted CD8+ T cells found in the HNSCC patients and negatively associated with the size of memory T cell pool and disease prognosis.

**Conclusions**

These data reveal NRP1 as a unique “immune memory checkpoint” with a mode of action that is distinct from other immune checkpoints. NRP1 blockade may promote the establishment of long-term T cell memory that is essential for durable anti-tumor immunity.

**References**

1. Zhang, P., et al., Induction of postsurgical tumor immunity and T-cell memory by a poorly immunogenic tumor. Cancer Res, 2007. 67(13): p. 6468-76.

2. Overwijk, W.W., et al., Tumor regression and autoimmunity after reversal of a functionally tolerant state of self-reactive CD8+ T cells. J Exp Med, 2003. 198(4): p. 569-80.

**Ethics Approval**

All animal experiments were performed in the American Association for the Accreditation of Laboratory Animal Care-accredited, specific-pathogen-free facilities in Division of Laboratory Animal Resources, University of Pittsburgh School of Medicine (UPSOM). Animal protocols were approved by the Institutional Animal Care and Use Committees of University of Pittsburgh. Patients diagnosed with head and neck squamous cell carcinoma (HNSCC) electing to undergo treatment were offered the option to participate in the University of Pittsburgh Cancer Institute (UPCI) protocol for research. All patients signed an informed consent that was approved by the Institutional Review Board (IRB) of the University of Pittsburgh.

#### P300 Real World data analysis related to metastatic melanoma patients treated with immunotherapy from 2012 to 2018 at Istituto Nazionale Tumori IRCCS Fondazione “G. Pascale” of Napoli, Italy

##### Gabriele Madonna, Medical Biotechnology (LS)^1^, Mariaelena Capone, MD^1^, Marilena Tuffanelli^1^, Marcello Curvietto, PhD^1^, Miriam Paone, PhD^1^, Assunta Esposito, PhD^1^, Antonio Sorrentino^1^, Marco Palla^1^, Luigi Scarpato^1^, Domenico Mallardo, MD^1^, Ester Simeone, MD^1^, Antonio Grimaldi, MD^1^, Kristina Viktorsson^2^, Lisa Villabona^2^, Rolf Lewensohn^2^, Giuseppe Masucci, MD, PhD^2^, Paolo Antonio Ascierto, MD^1^

###### ^1^Istituto Nazionale Tumori IRCCS Fondazione G. Pascale, Naples, Italy; ^2^Karolinska Institutet, Stockholm, Sweden

####### **Correspondence:** Paolo Antonio Ascierto (paolo.ascierto@gmail.com)

**Background**

Immuno checkpoint inhibitors (ICI) have improved the prognosis for patients with advanced malignancy [1-5]. Their real-life application may give different outcome compared to the benefit presented by clinical trials as the inclusion and exclusion criteria might be selective and give overoptimistic survival rates. Here we present the analysis of cutaneous metastatic melanoma patients treated with check-point inhibitors at Istituto Nazionale Tumori IRCCS Fondazione “G. Pascale” of Napoli Italy (INT-NA).

**Methods**

We investigated retrospectively, from 2012 to 2018, 578 stage IV melanoma patients received ipilimumab, pembrolizumab or nivolumab as monotherapy at the INT-NA. Ipilimumab was administered intravenously at the dosage of 3 mg/kg every 3 weeks for four doses, pembrolizumab at the dosage of 200 mg every 3 weeks and nivolumab at the dosage of 3 mg/kg every 2 weeks until disease progression or unacceptable toxicity appeared. Disease evaluation was performed at baselineand then every 12 weeks until progression or the discontinuation of treatment according to the Response Evaluation Criteria in Solid Tumors (RECIST 1.1) [6]. Survival analysis was performed using the Kaplan Meier method and with the log-rank test. Cox regression was used in the univariate and multivariate analysis. The results were considered significant if p

**Results**

Patients treated at INT-NA with nivolumab and pembrolizumab showed comparable better clinical benefit toward patients treated with ipilimumab (RR 44.5% vs 20.7%; p=0.01). The anti-tumoural medications received before recruitment to INT-NA had significant relevance in the outcome. Naïve patients or previously challenged by Immunotherapy had the best clinical benefit compared to those receiving first line BRAF/MEK inhibitors (RR 40% vs 10%; p=0.001). Again, after disease progression treatment with target therapy in naïve and immunotherapy previously treated patients, showed a different beneficial pattern compared to patients received BRAF/MEK inhibitors before immunotherapy. The clinical parameters correlated with an increase of clinical benefit were genus (female vs male), age (older [+60] vs younger), LDH score (normal vs high and very high), neutrophil/lymphocyte ratio (elevated vs normal). The introduction of an algorithm including the clinical variables above mentioned could define four predictable cohorts of benefit with a 95% of accuracy.

**Conclusions**

From the real-life analysis, we generated a simple algorithm that might drive clinical decision. Our finding clearly showed how previous treatment impacted outcome: patients treated with iBRAF/iMEK after treatment with ICI showed better clinical outcome respect patients treated with iBRAF/iMEK before treatment with ICI.

**Acknowledgements**

The study was supported by the Institutional Project "Ricerca Corrente" of Istituto Nazionale Tumori IRCCS Fondazione “G. Pascale” of Napoli, Italy

**References**

1. Balar AV, Weber JS. PD-1 and PD-L1 antibodies in cancer: current status and future directions. Cancer Immunol Immunother 2017; 66(5):551–564.2.

2. Larkin J, Minor D, D’Angelo S et al. Overall survival in patients with advanced melanoma who received nivolumab versus investigator’s choice chemotherapy in CheckMate 037: a randomized, controlled, open-label phase III trial. J Clin Oncol 2018; 36(4): 383–390.

3. Schachter J, Ribas A, Long GV et al. Pembrolizumab versus ipilimumab for advanced melanoma: final overall survival results of a multicentre, randomised, open-label phase 3 study (KEYNOTE-006). Lancet 2017;390: 1853–1862.4.

4. Larkin J, Chiarion-Sileni V, Gonzalez R et al. Combined nivolumab and ipilimumab or monotherapy in untreated melanoma. N Engl J Med2015; 373: 23–34.5.

5. Robert C, Long GV, Brady B et al. Nivolumab in previously untreated melanoma without BRAF mutation. N Engl J Med 2015; 372(4):320–330.

6. Wolchok JD, Hoos A, O'Day S, Weber JS, Hamid O, Lebbé C, et al: Guidelines for the evaluation of immune therapy activity in solid tumors: immune-related response criteria. Clin Cancer Res 2009, 15:7412–20.

**Ethics Approval**

The study was approved by the internal ethics board of the Istituto Nazionale Tumori IRCCS Fondazione “G. Pascale” in Napoli Italy, approval number of registry 33/17

#### P301 Expression of tumor matrix metalloproteases ADAM10 and ADAM17 correlates with low PD-L1 protein-to-mRNA ratio in multiple tumors, predicting poor outcomes

##### Aaron Mansfield, MD, Jacob Orme, MD PhD, Roxana Dronca, MD, Haidong Dong, MD, PhD

###### Mayo Clinic, Rochester, MN, United States

####### **Correspondence:** Jacob Orme (orme.jacob@mayo.edu)

**Background**

ADAM10 and ADAM17 portend poor prognosis in many malignancies [1]–[6]. We previously showed these proteases cleave Programmed death-ligand 1 (PD-L1) from tumors in soluble form (sPD-L1) [7]. sPD-L1 engages immune cell Programmed death 1 (PD-1) to inhibit tumor immunity. It is unknown how broadly this mechanism occurs in solid malignancies. We hypothesized (1) ADAM10 and/or ADAM17 may be elevated in tumors with low PD-L1 protein despite high PD-L1 (CD274) mRNA and (2) this low tumor PD-L1 protein-to-mRNA ratio may predict lower overall survival.

**Methods**

We queried the Cancer Genome Atlas (TCGA) for all solid tumors with Level 3 reverse phase protein array (RPPA) PD-L1 protein levels and RNA-seq sequence per million mapped fragments (FPKM) PD-L1 (CD274), ADAM10, and ADAM17 mRNA levels. We calculated a PD-L1 protein-to-mRNA ratio for each sample. Groups of high and low PD-L1 protein-to-mRNA ratios were evaluated for (1) ADAM10 and ADAM17 expression and (2) overall survival by Cox proportional hazards modeling, adjusting for age and stage at diagnosis.

**Results**

Tumor samples demonstrating low PD-L1 protein-to-mRNA ratios expressed significantly more ADAM10 and/or ADAM17 in 23 of 25 cancer types (Table 1). Cox proportional hazards ratios for death in each group were calculated and reported as a forest plot including hazard ratios and 95% confidence intervals for overall survival for each cancer subtype adjusted for patient age and tumor stage (Figure 1). Patients with tumors demonstrating low PD-L1 protein-to-mRNA ratio experienced significantly worse outcomes in 8 of 25 tumor types and improved outcomes in 2 tumor types (Table 2).

**Conclusions**

In this work we report that reduced human PD-L1 protein-to-mRNA ratios are associated with (1) high ADAM10 and/or ADAM17 expression and (2) poor outcomes in multiple cancers. We previously showed in multiple cell lines that ADAM10 and ADAM17 cleave PD-L1 from the surface of tumor cells [7]. Our results suggest that ADAM10 and/or ADAM17 may cleave PD-L1 to cause a low PD-L1 protein-to-mRNA ratio in these tumors. This process may explain poorer survival of patients with low PD-L1 protein-to-mRNA ratios in some cancers.

Our findings may explain why some tumors that do not have detectable PD-L1 expression on immunohistochemistry respond to PD-(L)1 inhibitor therapy given the solubilization of PD-L1 and its subsequent systemic negative regulatory effects on T cells. Further, ADAM10/ADAM17 inhibition may prevent PD-L1 shedding and sensitize tumors to therapy. While this work is correlative in nature, studies exploring this mechanism of tumor immune system evasion are ongoing.

**Acknowledgements**

The results shown here are in whole or part based upon data generated by the TCGA Research Network: https://www.cancer.gov/tcga.

**References**

1. M. Uhlen et al., “A pathology atlas of the human cancer transcriptome.,” Science, vol. 357, no. 6352, p. eaan2507, Aug. 2017.

2. P. C. Buchanan et al., “Ectodomain shedding of the cell adhesion molecule Nectin-4 in ovarian cancer is mediated by ADAM10 and ADAM17.,” J. Biol. Chem., vol. 292, no. 15, pp. 6339–6351, Apr. 2017.

3. M. E. Powers, H. K. Kim, Y. Wang, and J. Bubeck Wardenburg, “ADAM10 Mediates Vascular Injury Induced by Staphylococcus aureus α-Hemolysin,” J. Infect. Dis., vol. 206, no. 3, pp. 352–356, 2012.

4. S.-S. Ni, J. Zhang, W.-L. Zhao, X.-C. Dong, and J.-L. Wang, “ADAM17 is overexpressed in non-small cell lung cancer and its expression correlates with poor patient survival,” Tumor Biol., vol. 34, no. 3, pp. 1813–1818, Jun. 2013.

5. Y.-Y. Wang, Z.-Y. Ye, L. Li, Z.-S. Zhao, Q.-S. Shao, and H.-Q. Tao, “ADAM 10 is associated with gastric cancer progression and prognosis of patients,” J. Surg. Oncol., vol. 103, no. 2, pp. 116–123, Feb. 2011.

6. B. You, Y. Shan, S. Shi, X. Li, and Y. You, “Effects of ADAM10 upregulation on progression, migration, and prognosis of nasopharyngeal carcinoma,” Cancer Sci., vol. 106, no. 11, pp. 1506–1514, Nov. 2015.

7. J. J. Orme et al., “Tumor-associated ADAM10 and ADAM17 produce soluble PD-L1 (sPD-L1, sB7-H1) and affect downstream tumor immunity – a resistance mechanism to PD-1 checkpoint blockade in melanoma,” in CRI-CIMT-EATI-AACR International Cancer Conference, 2018.

**Ethics Approval**

Lab studies involving human subjects are approved by Mayo Clinic‘s Institutional Review Board (IRB), approval number 15-000934.

**Table 1 (abstract P301).**
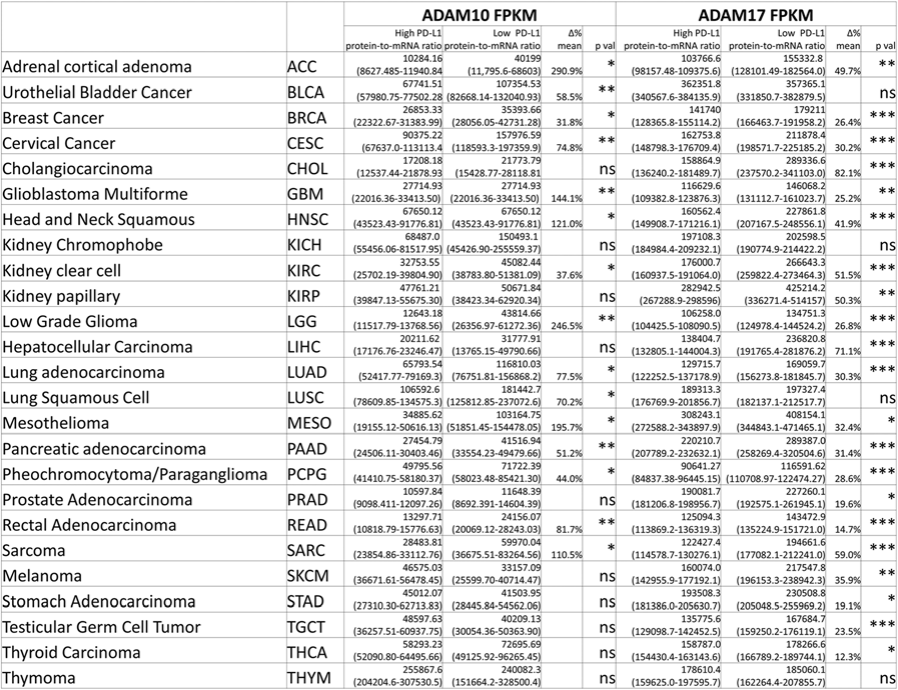
See text for description

**Table 2 (abstract P301).**
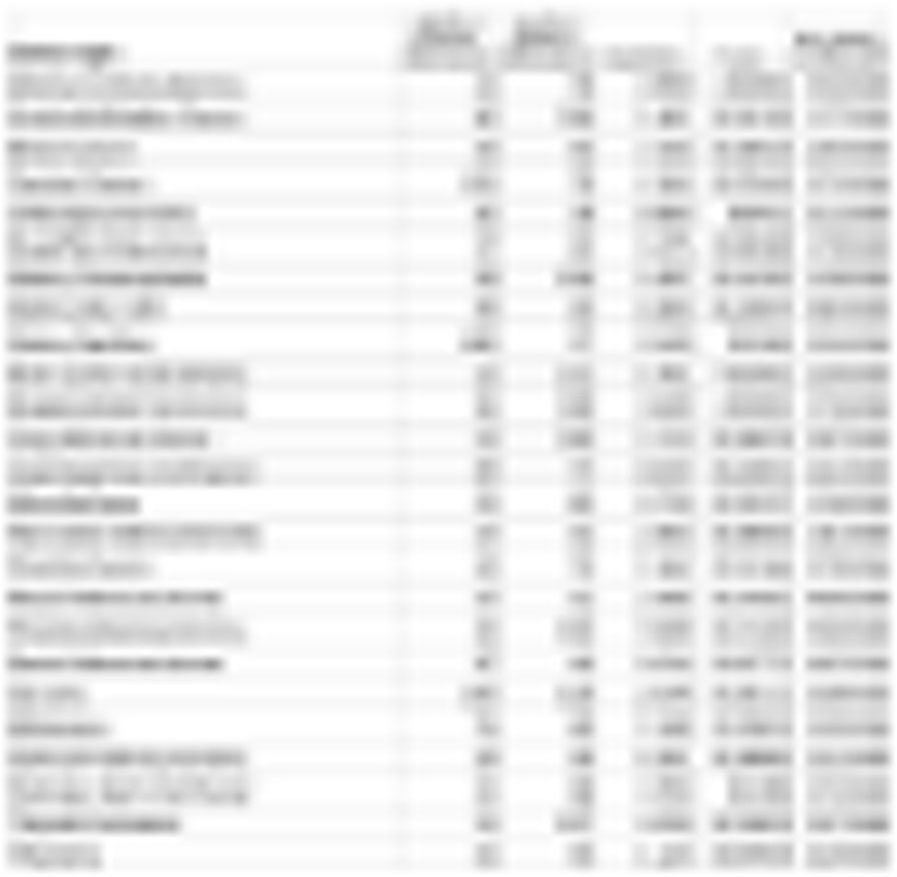
See text for description

**Fig. 1 (abstract P301).**
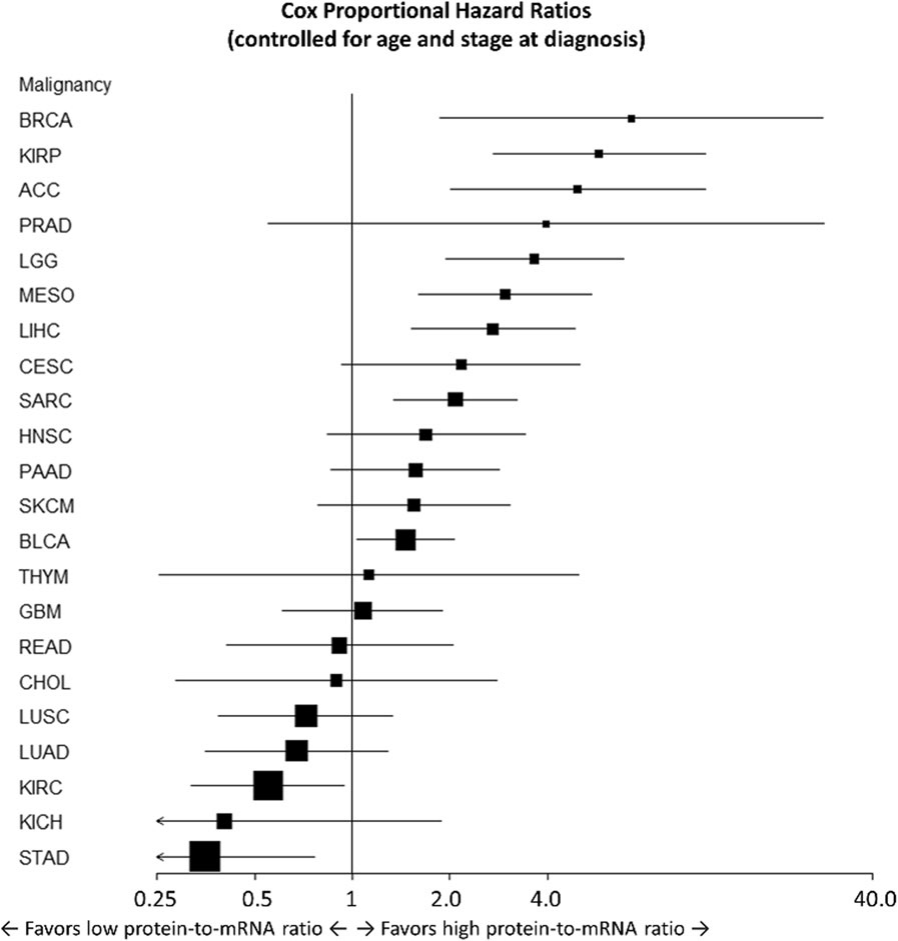
See text for description

#### P302 A reversible T cell exhaustion-like in vitro assay to screen candidate drugs

##### Wushouer Ouerkaxi^1^, Eden Kleiman^2^, Pirouz Daftarian^2^

###### ^1^MBL International, Woburn, MA, United States; ^2^JSR Lifesciences, Sunnyvale, CA, United States

####### **Correspondence:** Pirouz Daftarian (pdaftarian@jsrlifesciences.com)

**Background**

T cells undergo different layers of suppression, with a spectrum of events that may overlap such as dysfunction, anergy, unresponsiveness, tolerance, and exhaustion. Many factors have been attributed to this including multiple co-inhibitory receptor surface expression, altered transcription factor expression, epigenetic rewiring and dysregulated metabolism. Antigen persistence is necessary for driving TEX maintenance in both the chronic viral infection setting and cancer. In addition to persistent antigen exposure, tumor-infiltrating lymphocytes (TILs) within the tumor microenvironment (TME) encounter numerous tumor-mediated immunosuppressive metabolic byproducts, suppressive cytokines, hypoxia and cellular debris which converge to suppress T cell function and uniquely alter is transcription factor profile. These suppressed or dysfunctional T cells are incapable of mounting an optimal anti-tumor response in part due to lack of fitness in competing for glucose and oxygen. Our team has utilized the recall antigen potency assay as a tool for function-based screening of immune checkpoint inhibitor (ICI) drug candidates.

**Methods**

A recall antigen potency assay has been used as a tool for function-based screening of immune checkpoint inhibitor (ICI) drug candidates. In this assay, healthy human PBMCs are stimulated with peptide(s) derived from either viruses or tumor proteins and grown in culture for one week. Day 4 supernatants are functionally assayed by ELISA for IFN-γ secretion and cells are assayed on day 7 by flow cytometry for CD8+ or CD4+ T cell expansion by using a single or cocktail of pMHC tetramers.

**Results**

We have shown that in roughly 30% of donor PBMCs, ICI drugs such as pembrolizumab are able to boost both IFN-γ secretion and antigen-specific recall. One potential explanation for this effect is that the observed increase in T cell co-inhibitory receptor expression and presumed co-inhibitory receptor downstream signaling is ameliorated with ICI drugs releasing these T cells from the repressive effects of these co-inhibitory receptors. Further, this assay has the potential to screen for donors who are in vitro “responders” and “non-responders” to ICI drugs and is also being used to screen co-stimulatory agonists, peptide biologics and other drug classes.

**Conclusions**

Taken together, our data indicate that the recall antigen potency assay we established is highly potential to screen the drug candidates for immune checkpoint inhibitor and enhancer drug candidates.

#### P303 PD-1 checkpoint blockade in advanced melanoma patients: Neutrophils, NK cells, monocytic subsets and host PD-L1 expression as predictive biomarker candidates

##### Yago Pico de Coaña, Maria Wolodarski, MD, Irene van der Haar Àvila, Takahiro Nakajima, Stamatina Rentouli, Andreas Lundqvist, PhD, Giuseppe Masucci, MD, PhD, Johan Hansson, Rolf Kiessling, MD, PhD

###### Karolinska Institute, Stockholm, Sweden

####### **Correspondence:** Yago Pico de Coaña (yago.pico.de.coana@ki.se)

**Background**

Blockade of the PD-1 receptor has revolutionized the treatment of metastatic melanoma, with significant increases in overall survival and a dramatic improvement in patient quality of life. Despite the success of this therapeutic approach, the number of benefitting patients is limited and there is a need for predictive biomarkers and a deeper mechanistic analysis of the cellular populations involved in a clinical response.

**Methods**

With the aim to find predictive biomarkers for PD-1 checkpoint blockade, an in-depth immune monitoring study was conducted in 36 advanced melanoma patients undergoing treatment with pembrolizumab (n=7) or nivolumab (n=30) treatment at Karolinska University Hospital. Blood samples were collected from patients at the following time points: Before treatment and at the time of the second and fourth doses. Peripheral blood mononuclear cells (PBMCs) were isolated by density gradient centrifugation and stained for flow cytometric analysis within two hours of sample collection.

**Results**

Two distinct cellular populations were inversely correlated with survival: Neutrophils, and Monocytic myeloid derived suppressor cells (MDSCs). Furthermore, overall survival and progression free survival were also found to be inversely correlated with the activation status of NK cells. Finally, PD-L1 expression in different monocytic subsets was significantly increased in patients with shorter progression free survival and was correspondingly correlated inversely with overall survival.

**Conclusions**

Our results suggest that cellular populations other than T cells can be critical in the outcome of PD-1 blockade treatment. Specifically, the frequencies of activated NK cells and monocytic MDSCs are inversely correlated with survival and clinical benefit and their role as predictive biomarkers should be further evaluated.

**Ethics Approval**

The protocol was approved by the local Ethics Committee and the Institutional Review Board at Karolinska Institute (approval number 2015/1862-32) and all patients provided written informed consent in accordance with the Declaration of Helsinki.

#### P304 DNA damage response gene alterations are associated with high tumor mutational burden and clinical benefit from programmed death 1 axis inhibition in non-small cell lung cancer

##### Biagio Ricciuti, MD^1^, Gonzalo Recondo, MD^1^, Renato Umeton^1^, Giuseppe Lamberti, MD^1^, Mizuki Nishino^2^, Lynette Sholl^2^, Michael Cheng^1^, Mark Awad, MD PhD^1^

###### ^1^Dana Farber Cancer Institute, Boston, MA, United States; ^2^Brigham and Women’s Hospital, Boston, MA, United States

####### **Correspondence:** Biagio Ricciuti (biagio_ricciuti@dfci.harvard.edu)

**Background**

DNA damage response (DDR) gene alterations are associated with increased tumor infiltrating lymphocytes, higher genomic instability, and higher tumor mutational burden (TMB) in cancer. Whether DDR alterations are associated with benefit from immune-checkpoint inhibitors (ICIs) in non-small cell lung cancer (NSCLC) is unknown.

**Methods**

Clinicopathologic and genomic data were collected from patients (pts) with advanced NSCLC at the Dana-Farber Cancer Institute (DFCI) treated with PD-(L)1 inhibitors. Targeted next-generation sequencing (NGS) by OncoPanel was used to determine DDR gene mutation status and TMB. Patients were categorized based on the presence or absence of deleterious DDR gene alterations in a panel of 53 DDR genes. All loss-of-function alterations in DDR genes (including nonsense, frameshift, or splice site) were classified as pathogenic. Missense mutations were evaluated using the Catalogue of Somatic Mutations in Cancer (COSMIC) [1], and ClinVar databases [2], as well as the PolyPhen-2 (Polymorphism Phenotyping v2) functional prediction tool [3]. Missense mutations were classified as pathogenic if annotated as pathogenic by either COSMIC or ClinVar and damaging by Polyphen-2. Because only tumor tissue was sequenced, common single nucleotide polymorphisms (SNPs) were filtered if present at ≥0.1% in Genome Aggregation Database (gnomAD) version 2.1.1 [4]. Clinical outcomes to immunotherapy were evaluated according to DDR mutation status.

**Results**

Among 256 pts with successful NGS who received ICIs, 134 (52.3%) were identified as having deleterious DDR mutations (DDR-positive). DDR-positive and DDR-negative groups were well balanced in terms of baseline clinicopathological characteristics (Table 1). The median TMB was significantly higher in the DDR-positive group compared to the DDR-negative group (12.17 vs 8.36 mutations/megabase, P< 0.0001), as well as among never smokers (9.40 versus 5.70 mut/Mb, P = 0.035, Figure 1B). Compared to DDR-negative pts (N=122), DDR-positive pts had a significantly higher objective response rate (28.6% vs 16.4%, P=0.025, Figure 2A), longer median progression-free survival (4.2 vs 2.2 months, HR: 0.64 [95%CI: 0.49-0.84], P=0.001, Figure 2B) and overall survival (17.5 vs 9.9 months, HR: 0.60 [95%CI: 0.43-0.82], P=0.002, Figure 2C) with PD-(L)1 therapy. DDR-positive status was associated with significantly longer PFS (HR: 0.70 [0.51-0.95], P=0.024) and OS (HR: 0.61 [95%CI: 0.43-0.85], P=0.004) in multivariate analysis (Table 2).

**Conclusions**

Deleterious DDR alterations are frequent in NSCLC and are associated with higher TMB and improved clinical outcomes in NSCLC pts treated with PD-1 axis inhibition.

**References**

1. Forbes SA, Beare D, Boutselakis H, et al. COSMIC: somatic cancer genetics at high-resolution. Nucleic acids res. 2017; 45:D777-D783.

2. Landrum MJ, Lee JM, Benson M, et al. ClinVar: public archive of interpretations of clinically relevant variants. Nucleic acids res. 2016; 44:D862-D868

3. Adzhubei IA, Schmidt S, Peshkin L, et al. A method and server for predicting damaging missense mutations. Nat. Methods. 2010; 7:248

4. https://gnomad.broadinstitute.org

**Fig. 1 (abstract P304).**
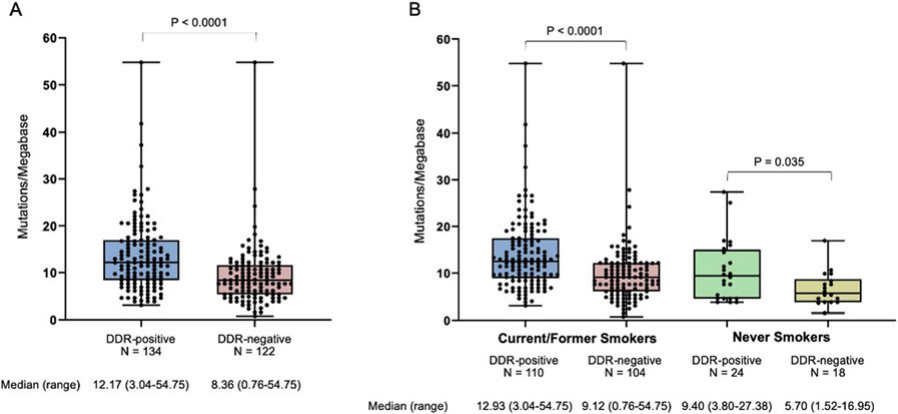
See text for description

**Fig. 2 (abstract P304).**
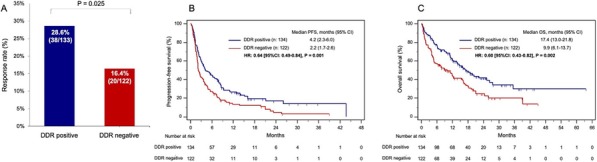
See text for description

**Table 1 (abstract P304).**
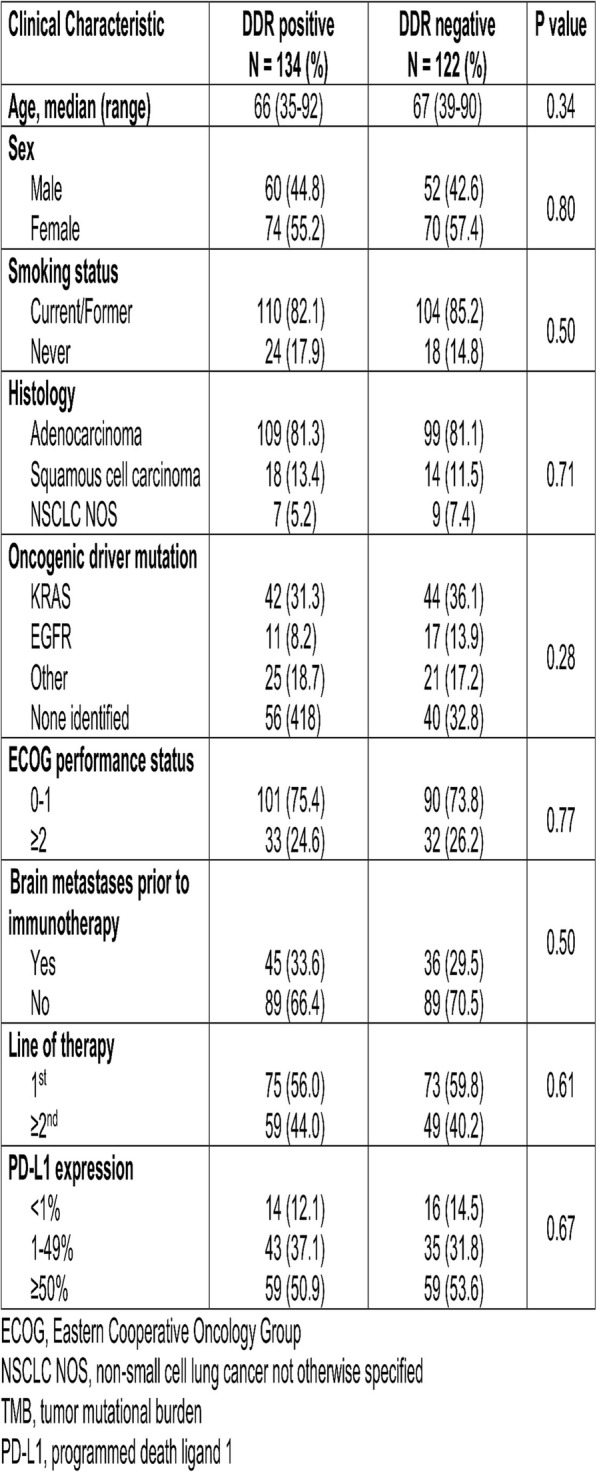
See text for description

**Table 2 (abstract P304).**
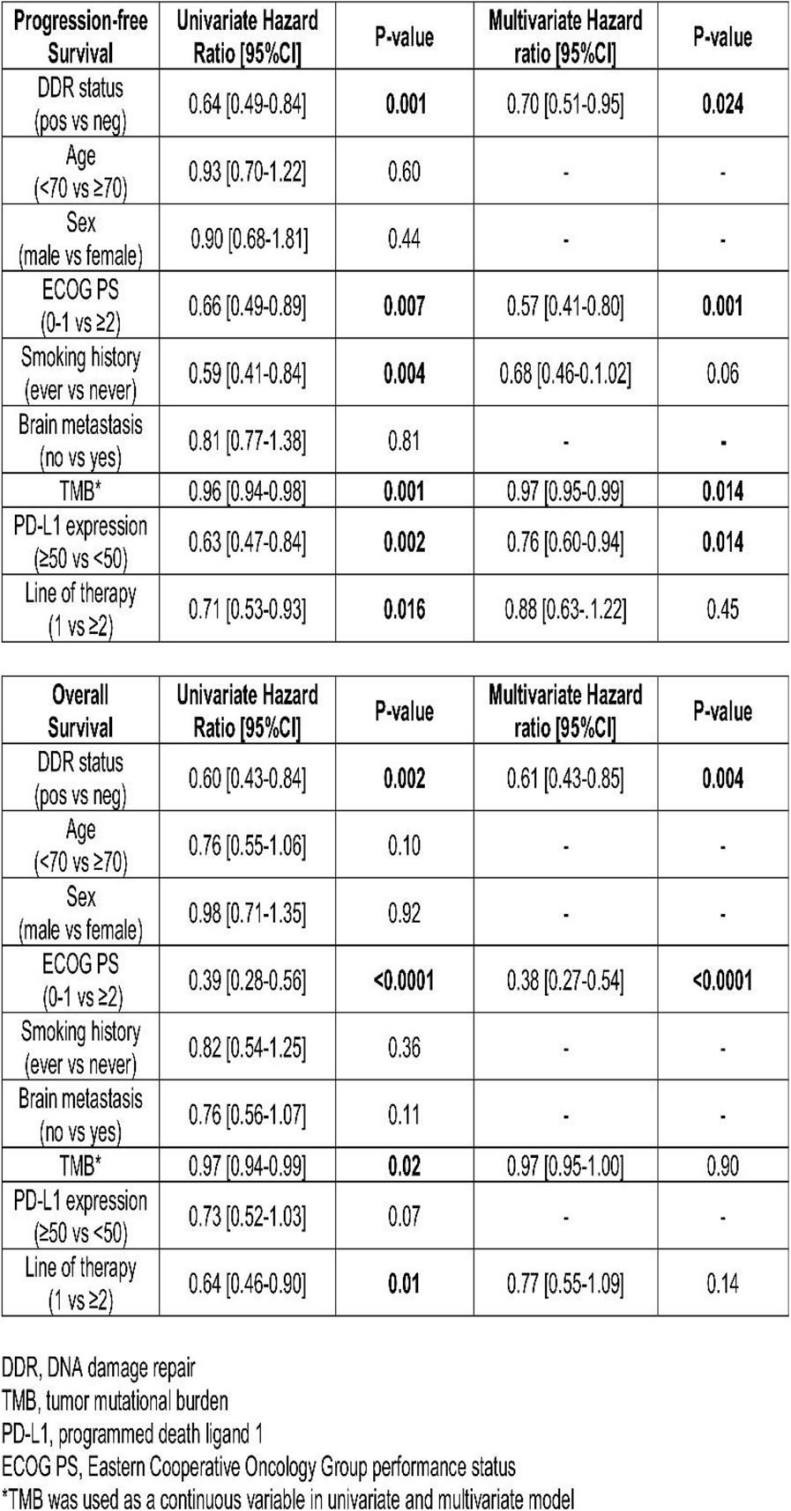
See text for description

#### P305 Response to Pembrolizumab and tumor microenvironment composition is associated with IL8 expression in a head and neck squamous-cell carcinoma cohort

##### Arun Khattri, PhD^1^, Jason Reeves^2^, SuFey Ong^2^, Riyue Bao, PhD^1^, Arya Bahrami, PhD^2^, Yi-Hung Carol Tan, PhD^1^, Andrew White, BSc^2^, Michael Bailey^2^, Heather Brauer, PhD^2^, Sarah Warren, PhD^2^, Joseph Beechem, PhD^2^, Tanguy Seiwert, MD^3^

###### ^1^University of Chicago, Chicago, IL, United States; ^2^NanoString Technologies, Seattle, WA, United States; ^3^Johns Hopkins University, Baltimore, MD, United States

####### **Correspondence:** Tanguy Seiwert (tseiwert@jhmi.edu)

**Background**

A subset of head and neck squamous-cell carcinomas are known to respond to immune checkpoint inhibitors. To better understand response to therapy in these tumors, a cohort of samples treated with Pembrolizumab was examined to determine if specific cell types are associated with response to intervention by combined profiling of standard bulk expression assays, ISH staining, and spatially resolved multiplexed protein analysis.

**Methods**

RNA was extracted from archival FFPE samples (n = 107) collected prior to therapeutic intervention and profiled using the NanoString® nCounter® PanCancer IO 360™ panel (Research Use Only). Gene expression signatures were calculated for immune cell subsets, as well as the Tumor Inflammation Signature (TIS; Ayers 2017 JCI). Individual gene expression and signatures were compared to patient outcome. Subsequently, expression of IL8 was validated by RNAScope in a subset of responders and non-responders (n = 9). To determine whether the IL8 staining pattern observed was consistent with specific cell types, a set of six tumor samples (three IL8+ and three IL8-) were further characterized by multiplexed protein expression analysis on the GeoMx™ digital spatial profiling (DSP) platform to quantitate expression of 40 antibodies. IL8 staining was used to guide DSP selection of regions of interest (ROI) within the tumors that were either IL8+ or IL8-. Protein expression was specifically measured from tumor or stromal areas based on Pan-cytokeratin immunofluorescence.

**Results**

Initial analysis of the head and neck cohort found that previously reported signatures of response, including TIS, were not associated with patient outcome in this cohort. In contrast, IL8 expression was observed to be highest in patients with progressive disease. Further investigation demonstrated that IL8 expression was most specifically associated with neutrophil markers/expression signatures. DSP profiling confirmed that tumor and stromal segments from IL8+ regions were associated with high expression of CD66B and ARG1 and lower expression HLA-DR consistent with neutrophil/granulocytic MDSCs presence. Furthermore, these regions were shown to have lower expression of T-cell markers including CD3, CD8 and CD4.

**Conclusions**

These results demonstrate that, in addition to previously reported biomarkers, IL8 expression and neutrophil presence may be related to response to checkpoint therapy in head and neck cancers. Decreased T-cell marker expression in IL8+ regions may reflect decreased response in the larger cohort.

**Ethics Approval**

The study was approved by the University of Chicago‘s Ethics Board, approval number 8980 and 16-1269

#### P306 Innate immune cells play a role in therapy resistance to anti-PD1 in Hu-mice melanoma model

##### Raj Somasundaram, PhD, Meenhard Herlyn, DVM PhD

###### The Wistar Institute, Philadelphia, PA, United States

####### **Correspondence:** Raj Somasundaram (shyam@wistar.org)

**Background**

Immune checkpoint inhibitor therapy is rapidly emerging as a front-line treatment option for many solid tumors. However, only a third of melanoma patients respond to immune checkpoint blockade. Currently available mouse models have many short comings and are unable to address the basis of therapy resistance and immune non-responsiveness that are observed in patients.

**Methods**

Our laboratory has developed a novel humanized mouse melanoma model. Immuno-deficient NSG mice were reconstituted with human CD34+ cells and after 8-12 weeks, mice are fully reconstituted with human innate and adaptive immune cells. Humanized mice were then challenged with HLA-matched melanoma cells and the functional ability of human immune cells to restrict tumor growth was monitored.

**Results**

Restricted tumor growth was observed in humanized mice indicating in vivo sensitization of human immune cells to melanoma.

In therapy studies, tumor-bearing humanized mice treated with anti-PD-1 showed restricted tumor growth. Anti-PD-1 therapy resulted in enhanced infiltration of T-cells that correlated with tumor response. MassCyTOF studies was performed using a panel of immune markers to understand the mechanism of therapy non-responsiveness in some tumors. Results indicated downmodulation of HLA-class I molecules and increased presence of mast cells cells in the tumor region. In tumor-bearing mice, combination of therapy drugs targeting c-kit+ mast cells and anti-PD1 caused complete regression of tumor lesions. Tumor free mice were able to reject freshly challenged melanoma cells indicating the presence of memory T-cell responses.

**Conclusions**

Our results suggest that humanized mouse melanoma model can be explored further to understand the therapy resistance mechanisms to immune-based treatments. Further, model will be useful for developing new therapeutic strategies for treating melanoma patients.

#### P307 The impact of metformin (M) on the response rates of checkpoint inhibitors (CPI)

##### Philip Haddad, MD, MPH^1^, David Sommerhalder, MD^2^

###### ^1^Overton Brooks VA/ LSUHSC, Shreveport, LA, United States; ^2^Louisiana State University Health Science, Bossier City, LA, United States

####### **Correspondence:** Philip Haddad (haddad8838@msn.com)

**Background**

Immunotherapies in oncology have brought significant change and hope to the field over the last several years. Responses, however, remain unpredictable and low in most cases. One of the aspects thought to be limiting immunotherapies is the tumor microenvironment which favors tumor growth and immunosuppression. It has been hypothesized that dysregulated tumor metabolism creates a hypoxic tumor microenvironment which acts as a barrier to antitumor immunity. Metformin has been shown to reduce oxygen consumption and subsequently reduce the microenvironment hypoxia, leading to improved response rates of checkpoint inhibitors in murine in vitro and in vivo models [1]. We performed a retrospective review to evaluate response rates in our patients who were treated with CPI+M.

**Methods**

We reviewed all adult cancer patients who were treated with a CPIs. Patients treated with M and CPIs were compared to those who were treated with CPIs only. All tumor types and all CPI drugs were included. The primary endpoint was overall response rate, which included stable disease, partial response, and complete response. Additional data was captured for subgroup analysis. Patients were excluded if they had never received the treatment, or if they were never assessed for response.

**Results**

As of this date, 144 patients had been included in the study data-set. Of those, 24 patients were treated with M and CPIs and 120 patients received CPIs only. Both groups were comparable with respect to sex. However, the M+CPI group was slightly older. Lung cancer constituted the majority of cases in both groups. The overall response rates of the CPI+M group were significantly higher than those treated with CPI only (75% vs 53.3%, p = 0.05).

**Conclusions**

The data from this chart review shows an apparent benefit with respect to overall response rates in patients treated with M and CPIs. The cohorts remain small in this study, but the data is significant. These results align well with data from mouse studies and two small retrospective human studies in melanoma [2] and lung cancer [3], hence, demonstrating an increased immune response and higher response rates. Currently, we are expanding our cohort number and data-set as we gained access to a bigger data warehouse. More prospective data are awaited from an ongoing phase Ib (UMIN000028405), and phase II (NCT03800602, NCT03048500) trials that should shed more light on the effects of metformin on immuno-oncologic therapies.

**References**

1. Scharping N, Menk A, Whetstone R, Zeng X, Delgoffe G. Efficacy of PD-1 blockade is potentiated by metformin-induced reduction of tumor hypoxia. Cancer Immunol Res. 2017;5(1):9-16.

2. Afzal M, Mercado R, Shirai K. Efficacy of metformin in combination with immune checkpoint inhibitors (anti-PD-1/anti-CTLA-4) in metastatic malignant melanoma. J Immunother Cancer. 2018;6(1):64.

3. Afzal M, Dragnev K, Sarwar T, Shirai K. Clinical outcomes in non-small-cell lung cancer patients receiving concurrent metformin and immune checkpoint inhibitors. Lung Cancer Manag. 2019:1-12 (online publication).

**Ethics Approval**

The study was approved by Louisiana State University Health Science Center of Shreveport’s Institutional Review Board separately for each site, IRB numbers are STUDY00000891, and STUDY00001017.

#### P308 The impact of obesity on the response rates of checkpoint inhibitor (CPI) cancer immunotherapy

##### Philip Haddad, MD, MPH^1^, David Sommerhalder, MD^2^

###### ^1^Overton Brooks VA Medical Center/LSUHSC-S, Shreveport, LA, United States; ^2^Louisiana State University Health Science, Bossier City, LA, United States

####### **Correspondence:** Philip Haddad (haddad8838@msn.com)

**Background**

With durable responses, improved clinical benefit, and relatively fewer toxicities, CPIs targeting cytotoxic T-lymphocyte-associated protein 4 (CTLA4), programmed death-1 (PD-1), and its ligand (PD-L1) have established themselves as essential components of cancer immunotherapy across multiple cancer types. Obesity is a known risk factor for several cancer types. It is associated with increased progression and cancer-related death. This is thought to be the result of inflammaging and PD-1 mediated immune suppression. Recently, two large retrospective studies found that obesity conferred a survival advantage for cancer patients treated with CPIs which may be independent of sex [1,2]. However, the mechanistic explanation of this observed obesity paradox, assuming it is real, has been the subject of many scientific conjectures. These studies focused on the impact of obesity on CPI overall survival which can be affected by many confounding variables. Instead, we explored the effect of obesity on CPI response rates.

**Methods**

We retrospectively reviewed every cancer patient that received CPIs at Overton Brooks VA Medical Center (OBVAMC) between 2015 and 2019. Patient’s BMI scores at the beginning of CPI therapies were calculated. Based on the WHO definition, the patients were grouped according to their BMIs into overweight and obese (Group A) versus normal and underweight (Group B). Our primary outcome of interest was defined as the presence or absence of CPI response. Patients who attained stable disease, partial response, and complete response were categorized as responders. Those who progressed on CPI were labeled as non-responders. The significance of the association between the grouped BMI categories and the occurrence of any response was analyzed statistically.

**Results**

Between 2015 and 2019, 65 patients were treated with CPIs and had documented responses. Both groups were comparable with respect to age, sex, race, and types of CPIs. Lung cancer constituted the majority of cases in both groups. Head and neck cancers were more prevalent in Group B while renal and bladder cancer and melanoma were more prevalent in Group A. Group B had a significantly higher response rate (80% vs 50%, p=0.01). Furthermore, a higher response rate was observed between normal BMI and overweight patients (p=0.001) and normal BMI and obese patients (p=0.06). No difference in response rates was observed between underweight and obese patients.

**Conclusions**

This is the first report to show a detrimental effect of overweight and obesity on CPI response rates in a retrospective cohort of non-selected consecutive cancer patients in a real-world clinical setting.

**References**

1. McQuade J, et al. Association of body-mass index and outcomes in patients with metastatic melanoma treated with targeted therapy, immunotherapy, or chemotherapy: a retrospective, multicohort analysis. Lancet Oncol. 2018;19(3):310-322.

2. Xu H, Cao D, He A, Ge W. The prognostic role of obesity is independent of sex in cancer patients treated with immune checkpoint inhibitors: A pooled analysis of 4090 cancer patients. Int Immunopharmacol. 2019;74:1-10.

**Ethics Approval**

The study was approved by the Louisiana State University Health Science Center of Shreveport Institutional Review Board, IRB number STUDY00001017.

#### P309 Older age predicts better outcome to neoadjuvant immune checkpoint blockade in metastatic melanoma

##### Rohit Thakur^1^, Stephen Douglass, PhD^2^, Beth Helmink, MD PhD^1^, Rodabe Amaria, MD^1^, Hussein Tawbi, MD, PhD^1^, Jennifer McQuade^1^, Eliza Rozeman^3^, Elizabeth Burton^1^, Sangeetha Reddy, MD, MSci^4^, John Wherry^5^, Christian Blank, MD PhD^3^, Georgina Long^6^, Jeffrey Gershenwald, MD^1^, Michael Davies, MD, PhD^1^, Michael Tetzlaff, MD PhD^1^, Ashani Weeraratna^7^, Jennifer Wargo, MD, MMSc^1^

###### ^1^MD Anderson Cancer Center, Houston, TX, United States; ^2^The Wistar Institute, Philadelphia, PA, United States; ^3^The Netherlands Cancer Institute, Amsterdam, Netherlands; ^4^UT Southwestern Medical Center, Houston, TX, United States; ^5^University of Pennsylvania, Philadelphia, PA, United States; ^6^Melanoma Institute Australia, Sydney, Australia; ^7^Johns Hopkins School of Medicine, Baltimore, MD, United States

####### **Correspondence:** Jennifer Wargo (JWargo@mdanderson.org)

**Background**

There is a growing appreciation for studying the impact of host and environmental factors on response to immune checkpoint blockade (ICB). Recently it was reported that older age correlated with better outcome in stage IV melanoma patients treated with ICB. We examined the association of age with response to ICB and anti-tumor immunity in melanoma patients treated neoadjuvantly (NCT02519322) and interrogated underlying mechanisms in a murine model.

**Methods**

Tumor samples were obtained from neoadjuvant ICB trial patients pre-treatment, on-treatment and on-surgery. Transcriptome profiling was performed using Illumina NextSeq platform and whole exome sequencing using Illumina HiSeq 2500 platform. Univariable and multivariable analyses were performed using logistic regression modeling. Immune profiling was performed by Immunohistochemistry (IHC). The effects of age on anti-tumor immunity were examined by implanting 2x105 Yumm1.7 cells subdermally in young (8wks) and aged (>12months) male mice. Tumors were harvested after 30 days of growth and immune surface markers were analyzed by flow cytometry.

**Results**

In the neoadjuvant ICB cohort, increasing age correlated with better response to immunotherapy (OR= 0.91, P=0.039) and remained a significant predictor of response in the multivariable model (OR=0.88, P=0.03) after adjusting for sex, tumor site, stage, prior therapies, and toxicity; similar results were observed in the OpACIN neoadjuvant trial study (NCT02977052). Tumor mutational load was positively correlated with age (r=0.4) but did not reach statistical significance (P=0.1). Differential gene expression analysis revealed genes associated with MHC class II antigen expression (HLA-DQB1, HLA-DRB1) as significantly overexpressed in younger patients upon treatment with ICB. Furthermore, MHC class II regulator interferon-gamma signaling was also observed upregulated in younger patients (P=0.03). PD-1 expression (by IHC) was lower in older patients upon treatment with ICB (r=-0.47, P=0.035). In keeping with human cohort, mice injected with Yumm1.7 melanoma cells showed significant increase in immune cell populations expressing MHC class II antigen in the young mice versus aged mice (P=0.0048).

**Conclusions**

Increased age was associated with improved outcomes in melanoma patients receiving neoadjuvant ICB, similar to results in stage IV. The mechanisms behind this association are likely multifactorial, and may relate in part to MHC class II antigen expression mediated immune evasion in younger patients, though further studies are needed to delineate contributing factors.

**Trial Registration**

NCT02519322

**Ethics Approval**

This trial was approved by the MD Anderson Cancer Center Institutional Review Board. The trial was conducted in accordance with the ethical principles of the Declaration of Helsinki and with adherence to the Good Clinical Practice guidelines, as defined by the International Conference on Harmonization. This protocol was conducted with compliance with all relevant ethical regulations.

**Consent**

Written informed consent was obtained from all participants. The MD Anderson Data Safety Monitoring Board reviewed the data at 12-month increments.

#### P310 Optimal priming prevents the induction of dysfunctional CD8 T-cells in subprimed conditions, reversing resistance to anti-PD-1

##### Vivek Verma, PhD, Rahul Nandre, PhD, Jose Lopez, Seema Gupta, PhD, Samir Khleif, MD

###### Georgetown University Medical Center, Washington, DC, United States

####### **Correspondence:** Samir Khleif (snk48@georgetown.edu)

**Background**

Suboptimal-priming of lymphocytes by low-affinity antigens is a mechanism for prevention of generation of strong immune responses against self-antigens. However, we recently found that the suboptimally-primed CD8 cells have a pre-disposition to develop a dysfunctional phenotype that is marked by expression of CD38 on PD1+CD8+T-cells. Interestingly, the number of these dysfunctional cells were significantly increased upon PD-1 blockade in these suboptimally-primed CD8 cells that served as a reason for resistance to anti-PD-1 therapy. On the other hand, anti-PD-1 blockade of optimally-primed CD8 cells did not generate these dysfunctional cells and led to cell activation and generation of effector functions [1]. Hence, here we investigated the effect of optimal-priming on reversing the resistance to anti-PD-1 therapy.

**Methods**

Mice were inoculated with TC-1 cells (a mouse lung epithelial cell-line, expressing human papillomavirus-specific E7-peptide) in the presence or absence of concomitant priming with gp100 peptide, a non-cognate tumor vaccine. Seven days later mice were treated with anti-PD-1 either alone or followed by combination of tumor-specific E7-peptide vaccine+anti-PD-1. Tumor growth rates, mice survival, and immune responses in the TME were estimated.

**Results**

We found that in suboptimally-primed TC-1 tumor-bearing mice, anti-PD-1 treatment did not show any anti-tumor effects. Therefore, to check if the optimal priming of CD8 T-cells could reverse this resistance, we vaccinated mice with gp100 at the time of tumor implantation with TC-1 cells. We found that compared to suboptimally-primed mice, anti-PD-1 treatment of primed-mice resulted in significant retardation of tumor growth and a prolonged mice survival. Interestingly, tumor-specific vaccination (E7-peptide) of these primed-mice at the time of anti-PD-1 treatment further enhanced the therapeutic efficacy of PD-1 blockade. Moreover, in gp100-primed-mice we found a significant reduction in the number of PD-1+CD38hi dysfunctional cells compared to suboptimally-primed mice. The number of these dysfunctional cells was further reduced upon anti-PD-1 treatment of primed-mice. In addition, the priming state also affected the functionality of CD8 T-cells. Although gp100 alone prevented the induction of these dysfunctional cells, the functionality of CD8 T-cells was only increased when anti-PD-1 was given subsequent to priming with gp100 peptide.

**Conclusions**

Here we demonstrate that optimal priming of CD8 cells reverses resistance to anti-PD-1 therapy. The suboptimal-priming of the CD8+ T-cells induces higher numbers of dysfunctional PD-1+CD38hi CD8+ T-cells and their frequency further increases upon anti-PD-1 therapy, leading to therapeutic failure. Since in most tumors, T-cells are suboptimally-primed [2,3], our mouse data demonstrate the importance of appropriately primed T-cells in responding to anti-PD-1 treatment.

**References**

1 Verma, V. et al. PD-1 blockade in subprimed CD8 cells induces dysfunctional PD-1(+)CD38(hi) cells and anti-PD-1 resistance. Nat Immunol, doi:10.1038/s41590-019-0441-y (2019).

2 Vonderheide, R. H. The Immune Revolution: A Case for Priming, Not Checkpoint. Cancer Cell 33, 563-569, doi:10.1016/j.ccell.2018.03.008 (2018).

3 Vonderheide, R. H., Domchek, S. M. & Clark, A. S. Immunotherapy for Breast Cancer: What Are We Missing? Clin Cancer Res 23, 2640-2646, doi:10.1158/1078-0432.CCR-16-2569 (2017).

#### P311 Dual antagonism of prostaglandin receptors EP2 and EP4 by TPST-1495 suppresses tumor growth and stimulates anti-tumor immunity

##### Chan Whiting, PhD^1^, Kim Fischer, PhD^2^, Bryan Laffitte, PhD^2^, Lisa Rahbaek, PhD^2^, Nick Stock, PhD^2^, Davorka Messmer, PhD^2^, Austin Chen, PhD^2^, Traci Olafson^2^, Natalie Nguyen^2^, Amanda Enstrom, PhD^1^, Derek Metzger^1^, Brian Francica^1^, Dingzhi Wang, PhD^3^, Raymond Dubois, PhD, MD^3^, Ginna Laport, MD^1^, Peppi Prasit, PhD^2^, Thomas Dubensky, PhD^1^

###### ^1^Tempest Therapeutics, San Francisco, CA, United States; ^2^Inception Sciences, San Diego, CA, United States; ^3^Medical University of South Carolina, Charleston, SC, United States

####### **Correspondence:** Brian Francica (bfrancica@tempesttx.com)

**Background**

Progression of diverse malignancies is promoted by elevated levels of Prostaglandin E2 (PGE2). High PGE2 levels results from dysregulation of Cyclooxygenase-2 (COX-2), the enzyme that produces this lipid. PGE2 stimulates tumor cell proliferation, survival, evasion and metastasis along with host angiogenesis. PGE2 suppresses anti-tumor immunity through inhibiting the function of critical immune effectors such as NK and T cells, and M1 macrophages, while promoting the activity of suppressive immune cells including myeloid derived suppressor cells, M2 macrophages, and regulatory T cells. PGE2 signals through a family of four homologous E-prostanoid (EP) G-coupled receptors, known as EP1, EP2, EP3 and EP4; which,are activated via distinct signal transduction pathways. Published literature and experimental results presented here demonstrate that selective antagonism of both EP2 and EP4 receptor signaling, but not EP1 and EP3, effectively overcomes PGE2-mediated immune suppression and results in anti-tumor efficacy. TPST-1495 is a first-in-class, orally available, small molecule, selective dual antagonist of the human PGE2 receptors EP2 and EP4, currently under development by Tempest.

**Methods**

The effects of TPST-1495 as monotherapy or in combination with anti-PD1 were evaluated in the syngeneic mouse colon models CT26 and Apcmin/+. The mechanism of anti-tumor immunity of TPST-1495 was evaluated using in vitro primary dendritic cell (DC) differentiation and activation assays. Characterization of in vitro differentiated immune cells or tumor infiltrating lymphocytes were performed using flow cytometry. ELISA was used for measurement of cytokine production.

**Results**

Treatment with TPST-1495 reversed PGE2 immune suppression in vitro and in vivo compared to antagonism of EP4 alone or all 4 EP receptors. TPST-1495 prevented PGE2 inhibition in vitro of DC differentiation and activation from human donor monocytes; single EP2 or EP4 antagonists were sub-optimal in this assay. Significantly, combination with EP1 and/or EP3 antagonists reversed the effect of dual EP2 and EP4 blockade on PGE2 immune suppression, suggesting that COX-2 inhibition is not optimal for blocking the effects of PGE2. TPST-1495 induced potent anti-tumor immune responses and significant tumor regression as a monotherapy in two different murine tumor treatment models of colon cancer, CT26 and Apcmin/+. CT26 tumors analyzed from mice treated with TPST-1495 alone revealed a significant increase of infiltrating effector T cells. TPST-1495 combination with anti-PD1 synergistically inhibited CT26 tumor progression.

**Conclusions**

TPST-1495 is a differentiated highly potent selective dual antagonist of EP2 and EP4 that overcomes prostaglandin-mediated immune suppression and promotes anti-tumor efficacy.

#### P312 Ipilimumab treatment immunophenotypic changes are associated with progression of disease with sequential nivolumab therapy in metastatic melanoma

##### David Woods, PhD^1^, Andressa Sodre Laino, PhD^1^, Aidan Winters^2^, Jason Alexandre^1^, Jeffrey Weber, MD, PhD^1^, Pratip Chattopadhyay^1^

###### ^1^NYU Langone Health, New York, NY, United States; ^2^UCSF, San Francisco, CA, United States

####### **Correspondence:** Pratip Chattopadhyay (Pratip.Chattopadhyay@nyulangone.org)

**Background**

Nivolumab (nivo) and ipilimumab (ipi) combination immunotherapy has a ~60% response rate in metastatic melanoma patients. However, the impact of these therapies on immune cell phenotypes and the relationship of those changes to patient outcomes remains under-investigated.

**Methods**

High dimension flow cytometry on baseline and at week 13 (e.g. after initial nivo or ipi therapy) was performed for peripheral blood samples from 33 metastatic melanoma patients receiving sequential nivo-ipi or the reverse sequence. We used a novel computational approach to analyze the data through semi-comprehensive Boolean gating in which immune cell lineages (e.g. CD3+CD4+) were evaluated for all possible combinations for up to 15 markers.

**Results**

3,844 measured immunophenotypes were significantly altered post-nivo, and 7,133 immunophenotypes were altered post-ipi. The frequency of 584 immunophenotypes were significantly changed in both treatments, with 59 of those changing in opposing directions. In the nivo-ipi cohort, 260 baseline and 662 post-nivo immunophenotypes were significantly associated with response and survival (outcomes). In the ipi-nivo cohort, 432 baseline and 668 post-ipi immunophenotypes were associated with outcomes. Two highly similar immunophenotypes associated with outcomes overlapped between the cohorts, CD14+CD11C+CD33+CD15-CD19-PDL1-PDL2+CD163+GAL9-CD80-CD86-41BBL+CD40+OX40L+ cells. While lower levels of these cells were associated with response and improved survival in nivo-ipi treated patients, lower levels were associated with better outcomes in the ipi-nivo treated patients. Of the 3,844 immunophenotypes altered post-nivolumab, 100 were also associated with ipi-nivo response. Of these 100, 97% were altered in a manner positively associated with response (e.g. upregulated by nivolumab and higher in responders). For example, nivo upregulated CD4+CD45RO-CCR7+ frequencies, which were associated with response and longer survival in ipi-nivo treated patients. Of the 7,133 immunophenotypes altered post-ipi, 110 were also associated with nivo-ipi response. Of these, 95% were altered in a manner negatively associated with response. This includes ipi associated upregulation of a population of CD4+CD38+CD39+CD127-GARP- cells that are negatively associated with coutcomes in nivo-ipi treated patients as well as downregulation of a CD4+CD127+CD45RO+CD95+CCR7+ population of cells positively associated with outcomes.

**Conclusions**

These results demonstrate that nivo and ipi altered the peripheral immune landscape in distinct ways. While immunophenotypic changes post-nivo favored response in ipi-nivo treated patients, changes associated with ipi treatment favored progression with ipi-nivo. These data suggest that the immunophenotypic impact of ipi alters the immune landscape in a manner that may impair a subsequent response to nivo. These data also highlight several novel immune cell populations that are associated with both treatment effects and patient outcomes.

**Ethics Approval**

The study was approved by NYU Langone Health's IRB, approval number S16-00035.

#### P313 Single-cell secretome assessment of metastatic melanoma patient peripheral T-cells reveals a pharmacokinetic signature of patient response to nivolumab therapy

##### David Woods, PhD, Andressa Sodre de Castro Laino, Daniel Freeman, Jeffrey Weber, MD, PhD, Pratip Chattopadhyay

###### NYU Langone Health, New York, NY, United States

####### **Correspondence:** David Woods (David.Woods@nyumc.org)

**Background**

Therapies targeting T-cell co-inhibitory molecules (e.g. PD1) have demonstrated unprecedented efficacy in the treatment of metastatic melanoma. However, not all patients respond to checkpoint inhibition, so there is an unmet need to identify mechanisms of resistance/response.

**Methods**

Using IsoLight, a platform for assessing the secretion of 32 analytes at single-cell resolution, we evaluated previously frozen peripheral blood T-cells from six responding and six progressing patients (according to RECIST 1.1 criteria) treated with nivolumab. Baseline and week 13, post-treatment CD4+ and CD8+ T-cell samples were assessed for each patient. T-cells were stimulated with CD3 and CD28 activating antibodies overnight and subsequently placed on capture chips for 20 hours. Approximately 400 single-cell events were assessed for each sample.

**Results**

CD4+ T-cells from progressing patients, compared to those from responding patients, had significantly (p<0.05) higher mean production of IL-17F post-nivolumab and an increased proportion of cells secreting IL-13, RANTES, IL-6, soluble CD137, TNF, MIP1a and MIP1b relative to baseline. Using an elastic net machine learning algorithm with cross validation, we assessed the ability of a manually curated list of analytes to predict patient outcomes. Delta values (post-treatment minus baseline values) were used for 15 parameters. A receiver operating characteristic with an area under the curve of 0.898 was achieved. The most important features in these models were the percentage of CD4+ T-cells expressing IL-6, MIP1a and soluble CD137 along with the percentage of CD8+ T-cells expressing IL-13.

**Conclusions**

These results demonstrate the ability of single-cell, high-dimension technologies coupled with machine learning to reveal complex associations between immune cell function and clinical outcomes. Specifically, these data show that changes in secretome production potential of T-cells after treatment with the PD1 blocking antibody nivolumab are associated with metastatic melanoma patient outcomes. We identified a signature of secreted molecules that were associated with patient outcomes, providing rationale for targeting these molecules to increase the efficacy of nivolumab. Work is underway to validate the observed associations in an independent set of patient samples.

**Ethics Approval**

The study was approved by NYU Langone Health's IRB, approval number S16-00035.

#### P314 Alterations of DNA damage response signaling in the development of antibody-dependent cellular cytotoxicity (ADCC) resistance

##### Louis Weiner, MD, Yongwei Zhang, Dalal Aldeghaither, David Zahavi, MS, BS

###### Lombardi Cancer Center, Washington, DC, United States

####### **Correspondence:** Louis Weiner (weinerl@georgetown.edu)

**Background**

Accumulating evidence has shown that DNA damage response (DDR) is closely associated with immune response. Innate immune responses, such as Natural killer (NK) cell-mediated killing, are dependent on the DDR essential kinases Ataxia telangiectasia mutated (ATM) or ATM- and RAD3-related (ATR) [1]. However, DNA damage inducing agents activate the NF-kappaB-regulated interferon immune response pathway [2]. Moreover, DDR inhibition resulting from DNA repair deficiency or cell cycle checkpoint inhibition can potentiate efficacy of antibody-based immunotherapy, such as immune checkpoint blockade and ADCC [3-5]. However, the mechanistic role of DNA damage response signaling in the development of immunotherapy resistance remains unclear. A NK cell-mediated cetuximab-dependent killing in vitro ADCC model was used to study this involvement. Our previous studies have suggested a loss of cell surface adhesion molecules in ADCC resistant cells [6]. Here we study the alterations of DNA damage response signaling in the process of ADCC resistance development.

**Methods**

A431, a human epidermoid carcinoma cell line, develops resistance to cetuximab and NK92-CD16v cell-mediated killing after 35 challenges of continuous exposure. Surviving cells following each ADCC challenge were collected and studied for protein expression by western blot, immunofluorescence and flow cytometry. Neutral comet assay was used to measure the level of DNA double strand breaks. Fluorescence-based cytotoxicity methods were used to determine the activity of ADCC. Apoptosis was measured by Annexin-V-PI staining. Small interference RNA (siRNA) were used to knockdown gene expression.

**Results**

Levels of gammaH2AX, a marker of DNA damage signaling, were significantly increased with exposure to ADCC, but no increased DNA breaks were detected in ADCC resistant cells. p53, phosphorylated-p53 and signal transducer and activator of transcription 1 (STAT1) were also enhanced during the process of ADCC resistance development. Interestingly, phosphorylated-STAT1 reached a peak prior to the emergence of ADCC resistance and then decreased until cells became entirely resistant. There was less apoptosis induction, no caspase activation, less induction of gammaH2AX and no activation of p53 in response to ADCC in resistant cells. Inhibition of p53 or STAT1 by siRNA, and ATM/ATR inhibitors enhanced ADCC activity in A431 cells but not in ADCC resistant cells. TP53 knockdown activated STAT1 in A431 cells, but reduced activation of STAT1 in ADCC resistant cells.

**Conclusions**

DNA damage response signaling was altered during the development of ADCC resistance, which is involved in ADCC activity regulation and might become be a signature of cell sensitivity in response to immunotherapy. Further DDR-related multiplex mechanisms are being investigated.

**References**

1. Gasser S, Orsulic S, Brown EJ, Raulet DH. The DNA damage pathway regulates innate immune system ligands of the NKG2D receptor. Nature. 2005;436(7054):1186-90.

2. Brzostek-Racine S, Gordon C, Van Scoy S, Reich NC. The DNA damage response induces IFN. J Immunol. 2011;187(10):5336-5345.

3. Sen T, Rodriguez BL, Chen L, Corte CMD, Morikawa N, Fujimoto J, Cristea S,Nguyen T, Diao L, Li L, Fan Y, Yang Y, Wang J, Glisson BS, Wistuba II, Sage J, Heymach JV, Gibbons DL, Byers LA. Targeting DNA Damage Response Promotes Antitumor Immunity through STING-Mediated T-cell Activation in Small Cell Lung Cancer. Cancer Discov. 2019,9(5):646-661.

4. Fenerty KE, Padget M, Wolfson B, Gameiro SR, Su Z, Lee JH, Rabizadeh S, Soon-Shiong P, Hodge JW. Immunotherapy utilizing the combination of natural killer- and antibody dependent cellular cytotoxicity (ADCC)-mediating agents withpoly (ADP-ribose) polymerase (PARP) inhibition. J Immunother Cancer. 2018,6(1):133-136.

5. Germano G, Lamba S, Rospo G, Barault L, Magrì A, Maione F, Russo M, CrisafulliG, Bartolini A, Lerda G, Siravegna G, Mussolin B, Frapolli R, Montone M, MoranoF, de Braud F, Amirouchene-Angelozzi N, Marsoni S, D'Incalci M, Orlandi A,Giraudo E, Sartore-Bianchi A, Siena S, Pietrantonio F, Di Nicolantonio F,Bardelli A. Inactivation of DNA repair triggers neoantigen generation and impairstumour growth. Nature. 2017,552(7683):116-120.

6. Aldeghaither DS, Zahavi DJ, Murray JC, Fertig EJ, Graham GT, Zhang YW,O'Connell A, Ma J, Jablonski SA, Weiner LM. A Mechanism of Resistance to Antibody-Targeted Immune Attack. Cancer Immunol Res. 2019;7(2):230-243.

**Fig. 1 (abstract P314).**
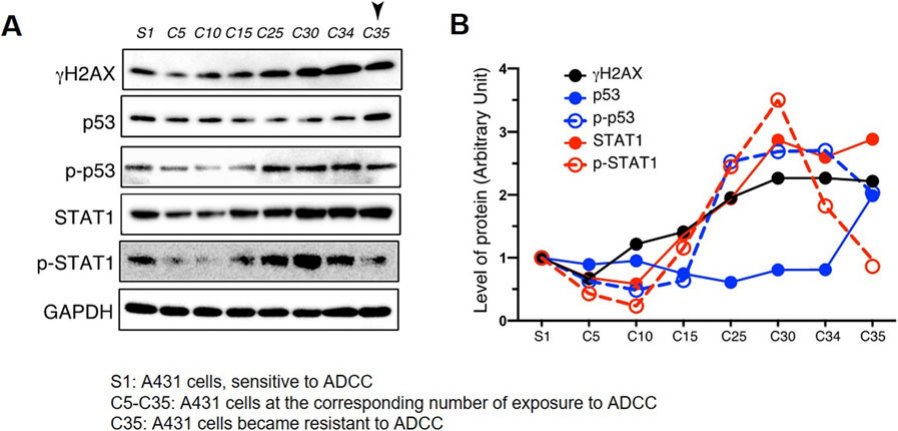
Enhancement of DDR signaling pathway molecules

#### P315 The expression of programmed death ligand 2 (PD-L2) in immunosuppressive tumor microenvironment of non-small cell lung cancer (NSCLC) and its potential association with immunotherapy

##### Qu Zhang, PhD^1^, Stefan Bentink^2^, Vinay Pawar^2^, Farzad Sekhavati, PhD^2^, Keith Steele, DVM, PhD^1^, Jason Hipp^1^, Song Wu, PhD^1^

###### ^1^AstraZeneca, Gaithersburg, MD, United States; ^2^Definiens AG, Munich, Germany

####### **Correspondence:** Qu Zhang (zhangq@medimmune.com)

**Background**

In the tumor microenvironment (TME), programmed death-1 receptor (PD-1), combined with its ligands PD-L1 and PD-L2, play an important suppressive role in the immune response to cancer. PD-L1 expression has been shown to be a key predictive biomarker of response to PD1/PD-L1 therapies. PD-L2 expression is observed in multiple tumor types, and animal studies suggest PD-L2 may be involved in T-cell suppression. However, the role of PD-L2 expression in the TME and the role as a predictive biomarker in immunotherapy is not as well understood as PD-L1. Here, we investigated the expression of PD-L2 in multiple cellular components of the TME and its potential impact on the efficacy of PD-L1 blockade in cancer patients.

**Methods**

Single-cell RNA sequencing (scRNAseq) was performed using 10X genomics for two commercial NSCLC samples and ~4,500 cells were clustered by their expression pattern using shared nearest neighbor. Gene expression data for lung adenocarcinoma (LUAD) and squamous carcinoma (LUSC) in TCGA were analyzed. Baseline tumor transcriptomes were profiled for 97 1L+ NSCLC patients treated with PD-L1 inhibitor durvalumab (NCT01693562). PD-L1 and PD-L2 immunostaining was performed on baseline samples in CP1108. Gene expression signatures for macrophage, fibroblast, dendritic cells, cancer associated fibroblast (CAF) and interferon gamma were curated in-house or adopted from previous studies.

**Results**

In scRNAseq data, PD-L2 mRNA was found in over 10% of macrophage and fibroblast cells, and

**Conclusions**

PD-L2 mRNA expression is mainly in immunosuppressive cellular components of the TME in NSCLC, including macrophage and cancer associated fibroblast cells and low PD-L2 expression in PD-L1 high NSCLC patients may associate with improved OS.

**Trial Registration**

NCT01693562

**Ethics Approval**

This study was conducted according to the Declaration of Helsinki and approved by the independent ethics committee/institutional review board at each participating center, with informed consent obtained from all patients.

#### P316 MG1131, a novel TIGIT-targeted monoclonal antibody, induces T cell activation and anti-tumor immune response and suppresses Treg cell activity

##### Hyemi Nam, MS^1^, Hye-Young Park^1^, Eun Jung Song^2^, Eunhee Lee^1^, Hye In Yum^1^, Munkyung Kim^1^, Jeewon Lee, Ph D^1^, So Jung Lim^1^, Okjae Lim^1^, Yangmi Lim^1^

###### ^1^MOGAM Institute for Biomedical Research, Yongin-si, Korea, Republic of; ^2^GC Pharma, Yongin-si, Gyeonggi-do, Korea, Republic of

####### **Correspondence:** Yangmi Lim (ymlim@mogam.re.kr)

**Background**

T cell immunoreceptor with Ig and ITIM domain (TIGIT) is a co-inhibitory receptor expressed on CD8+ T cells, CD4+ T cells, NK cells, and regulatory T cells (Treg). TIGIT binds two ligands PVR (CD155) and PVRL2 (CD112) and these ligands are expressed by T cells, APCs, and tumor cells. As malignancies progress, PVR over-expressed in tumor cells interacts with TIGIT expressed on tumor infiltrating lymphocytes (TIL) and suppresses TIL activity by sending an inhibitory signal to immune cells, which is an immune escape mechanism in cancer. In cancer, TIGIT blockade results in improved effector CD8+ T cell and NK cell function as well as decreased Treg-cell-mediated suppression. Therefore, we developed MG1131, a novel anti-TIGIT antibody, to modulate the tumor microenvironment towards a more effective anti-cancer response.

**Methods**

TIGIT-targeting antibody candidates were screened out of a phage display library. The TIGIT antigen binding affinity and PVR blocking of anti-TIGIT antibodies were evaluated through both protein-based and cell-based assays. Functional consequences of MG1131 were determined using a cell-based reporter assay for T cell activation, a Treg cell functional assay and an NK-mediated tumor killing assay. Treg cells and NK cells were isolated from healthy donor PBMC.

**Results**

We screened out a few of the clones which stood out showing high affinity binding to TIGIT and significant blocking of the TIGIT-PVR interaction. Among the tested antibodies, MG1131 was identified to have the strongest affinity for human TIGIT and significant blocking activity, resulting in competition with PVR in a dose dependent manner. Furthermore, MG1131 was cross-reactive with cynomolgus monkey TIGIT, but not with mouse TIGIT. Our in vitro efficacy data demonstrated that MG1131 significantly enhances T cell activation and NK-mediated tumor killing activities in a PVR-dependent manner and MG1131 induces IFN-γ secretion and proliferation of CD8+ T cells by inhibiting Treg suppressive function.

**Conclusions**

We developed an anti-TIGIT antibody, MG1131, with pronounced inhibitory activity on the TIGIT-PVR signaling axis. In this study, MG1131 significantly enhanced T cell activation and NK-mediated tumor killing activity, and efficiently suppressed Treg cell function. Therefore, MG1131 is a potential candidate for cancer immunotherapy.

#### P317 Utilization of second-line immuno-oncology agents and associated health outcomes among united states veterans with advanced non-small cell lung cancer

##### Mina Allo, PharmD, MPH^1^, Lin Gu, MS^2^, Vishal Vashistha, MD^3^, Ashlyn Press, MPH^2^, Michael Kelley, MD^2^, Christina Williams, PHD, MPH^2^

###### ^1^Bristol-Myers Squibb, Princeton, NJ, United States; ^2^Durham Veterans Affair, Duke Cancer Ins, Durham, NC, United States; ^3^ Dept of Medicine, Duke University, Durham, NC, United States

####### **Correspondence:** Mina Allo (mina.allo@bms.com); Christina Williams (christina.williams4@va.gov)

**Background**

Studies describing treatment safety, effectiveness and patterns of use are needed to evaluate the real-world impact of immuno-oncology (IO) in advanced non-small cell lung cancer (NSCLC) relative to chemotherapy (CT). This retrospective cohort analysis assessed utilization of IO and CT agents and associated outcomes in second-line (2L) treatment among stage IV NSCLC patients receiving care in the Veterans Affairs (VA).

**Methods**

The VA Corporate Data Warehouse (CDW) oncology database was used to determine survival and 14 common adverse events (AE) of adult patients with stage IV NSCLC diagnosed from 2012 to 2017 who received systemic non-targeted (ALK, EGFR) therapy within 120 days of diagnosis and were followed until death or end of the study period in June 2019. Descriptive statistics were used to summarize treatment and AE occurrence. Kaplan-Meier methodology and multivariate Cox regression were used to evaluate survival.

**Results**

We identified 1655 patients who received 2L therapy, with 42% (n=695) receiving IO monotherapy (nivolumab, pembrolizumab, atezolizumab, and durvalumab), 56.5% (n=935) receiving CT only, and 1.5% (n=25) receiving IO+CT (not included in the current analysis due to limited sample). Greater than 99% of 2L IO users used CT only in 1L setting, and >96% of 2L CT only group used CT in 1L (~ 3% used IO monotherapy, 0.6% IO+CT). Median age was 67 vs. 65 years in the IO and CT groups, respectively (p=0.006). No statistically significant differences between the IO and CT groups were observed by sex (~97% male), race (~77% White), ethnicity (~98% non-Hispanic), smoking history (~95% current/former smoker), or histology (~58% adenocarcinoma). The most common AEs in the IO group were dyspnea (50%), colitis/enterocolitis (40%), and anemia (28%); most common AEs in the CT group were hypertension (32%), anemia (29%), colitis/enterocolitis (26%), and nausea/vomiting (25%). Median survival was significantly longer in the IO group relative to the CT group (18.6 months and 15.1 months, respectively; adjusted HR 0.73, 95% CI 0.66-0.81, Figure 1).

**Conclusions**

Among the veteran population in real-world settings, patient characteristics were similar for 2L IO or CT therapy, with the exception of age and geographic region. Rates of common AEs were as expected. Our findings indicate improved survival among patients receiving IO versus CT in the 2L setting. More population-based studies are needed to confirm these findings in other healthcare settings.

**Acknowledgements**

The team would like to thank Daniel Lane, PharmD, PhD, MBA for his support of this study.

**Ethics Approval**

This study was approved by the Durham VA Institutional Review Board (IRB #02009).

**Fig. 1 (abstract P317).**
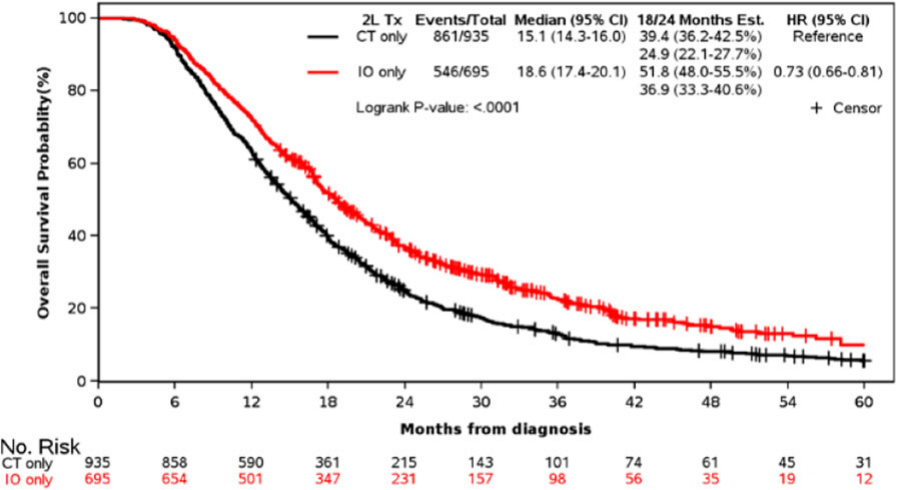
KM curve of overall survival of patients in 2L

#### P318 Large-scale evaluation of concordance of genomic scores in whole exome sequencing and Foundation Medicine comprehensive genomic platform across cancer types

##### Deepti Aurora-Garg^1^, Andrew Albright, PhD^1^, Ping Qiu, PhD^1^, Yongjin Li^1^, Xiaoqiao Liu^1^, David Fabrizio, PhD^2^, Lixin Lang^1^, Jared Lunceford, PhD^1^, Razvan Cristescu, PhD^1^

###### ^1^Merck & Co., Inc., Kenilworth, NJ, United States; ^2^Foundation Medicine, Cambridge, MA, USA, Cambridge, MA, United States

####### **Correspondence:** Deepti Aurora-Garg (deepti.aurora-garg@merck.com)

**Background**

Whole exome sequencing (WES) is a comprehensive method to evaluate the clinical relevance of DNA molecular characteristics, including pan-exomic genomic scores (eg, tumor mutational burden [TMB] and homologous recombination deficiency–loss of heterozygosity [HRD-LOH]) and individual alterations (eg, BRCA1/2). Although comprehensive targeted genomic panels are available to measure TMB and HRD-LOH, including FoundationOne® CDx (F1CDx), implementation of WES as a diagnostic approach in clinical practice can be challenging. To assess the feasibility of translating findings using WES as an exploratory tool into a practical diagnostic device such as F1CDx, we evaluated the concordance of genomic scores (TMB and HRD-LOH) and single-gene alterations between WES and F1CDx in a large pan-tumor data set.

**Methods**

This analysis used solid tumor samples from patients with advanced disease who received pembrolizumab monotherapy in the second line or later during single-arm clinical trials. WES and F1CDx (Dx1 baitset) were used to analyze samples from 436 patients across 22 tumor types. Spearman rank-order correlation and linear regression were used to determine concordance and cutoff equivalence for TMB and HRD-LOH, each calculated by both WES [1] and F1CDx (Foundation Medicine proprietary pipeline QSR_F1Dx_v1.0.3).

**Results**

Using WES and F1CDx, high concordance was observed in the pan-tumor assessment of TMB (Spearman correlation, 0.7; n=413) and HRD-LOH (Spearman correlation, 0.5; n=364). When individual indications were considered, the concordance was further improved for indications with higher distribution medians. TMB concordance was higher when restricted to non–small cell lung cancer (Spearman correlation, 0.8; n=38). HRD-LOH concordance was higher when restricted to ovarian cancer (Spearman correlation, 0.7; n=54) and breast cancer (Spearman correlation, 0.6; n=80). Regression analysis of TMB using both platforms identified F1CDx (Foundation Medicine proprietary pipeline QSR_F1Dx_v1.0.3) TMB cutoffs of 10 and 13 mutations/megabase to correspond to WES TMB of ~150 and ~175 mutations/exome, respectively. Assessment of BRCA1/2 deleterious mutations also demonstrated agreement between WES and F1CDx, with 305 of 309 (98.7%) samples showing agreement; 282 samples showed wild-type status by both methods and 23 samples showed mutant status by both methods.

**Conclusions**

The high level of concordance between WES and F1CDx suggests that molecular biomarker discoveries, including clinically relevant cutoffs and molecular epidemiology findings evaluated on the translational WES platform, may be translated successfully in the diagnostic setting. To our knowledge, this is the first evaluation of concordance of genomic scores performed in the context of clinical trial data across many indications and in a large data set.

**Reference**

1. Cristescu R, Mogg R, Ayers M, et al. Pan-tumor genomic biomarkers for PD-1 checkpoint blockade-based immunotherapy. Science. 2018;362. pii: eaar3593.

**Ethics Approval**

The studies in which patient samples were collected were approved by an independent ethics committee before being initiated at each site.

#### P319 Changes in fatigue severity, health related, and dermatology related quality of life in melanoma patients receiving nivolumab: Preliminary results of a prospective study from real-life experience

##### Canan Karadas^1^, Nur Izgu, PhD, RN^1^, Zehra Gok Metin, Assoc Prof, PhD, RN^1^, Canan Porucu, MSc, RN^2^, Nuri Karadurmus, Prof Dr, MD^2^, Sadettin Kilickap, Prof Dr, MD^3^, Umut Demirci, MD^4^

###### ^1^Hacettepe University, Ankara, Turkey; ^2^Gulhane Training and Research Hospital, Ankara, Turkey; ^3^Hacettepe University Cancer Institute, Ankara, Turkey; ^4^Dr. A.Y. Ank Onco Tra and Res Hospital, Ankara, Turkey

####### **Correspondence:** Canan Karadas (karadas.canan@gmail.com)

**Background**

Previous studies have reported that nivolumab, as an immune checkpoint inhibitor, may enhance survival, reduce therapy related toxicities and improve quality of life (QoL) among melanoma patients compared with traditional cytotoxic chemotherapy [1,2]. However, nivolumab is frequently associated with fatigue, increased risk of skin toxicity and autoimmune-related adverse events. Thus, patients may experience important changes in daily living activities, health related QoL, and skin integrity during nivolumab treatment. In view of this issue, studies are needed evaluating these important changes related to nivolumab, concurrently. Therefore, this study aimed to investigate fatigue severity, health related QoL, and dermatology related QoL in melanoma patients receiving nivolumab.

**Methods**

A total of 20 patients, scheduled to receive first dose of nivolumab, in three leading hospitals located in Ankara, was included in this descriptive, prospective and multicenter study. All the patients received at least four cycle of nivolumab infusion between October 2018 and June 2019. Patient Information Form, Brief Fatigue Inventory (BFI), Functional Living Index-Cancer (FLIC), and Dermatology Life Quality Index (DLQI) were used for data collection at 1st, 2nd, 3rd, and 4th cycles of nivolumab. Descriptive statistics, Mann Whitney U and Friedman tests were utilized for data analysis.

**Results**

The majority of the patients was male (60%), and the mean age of patients was 54.10±18.88 years. The mean time of diagnosis for patients with melanoma was 40.50±47.84 months (range 2-168). There were no significant differences between patients’ BFI, FLIC, and DLQI total scores in terms of age and gender (p>.05). The mean scores of BFI scores were 4.15±2.90 at the 1stcycle, and 3.75±2.98 at the 4th cycle; FLIC scores were 88.15±9.69 and 95.26±12.07; and DLQI scores were 2.60±6.30 at 1.90±3.11, respectively. Considering the changes within time in terms of all scale scores, no significant differences were found in BFI (p=.29), and DLQI (p=.49). With regard to FLIC scores a significant difference was found from the 1st to the 4th cycle of nivolumab (p=.05).

**Conclusions**

The present study may be the first effort to evaluate changes in BFI, FLIC and DLQI scores during nivolumab treatment in patients with melanoma from the 1st to the 4th cycle. The study findings revealed that improvements in BFI, and DLQI scores following nivolumab, even not statistically significant. Lastly supporting literature [3,4], significant increase has been found in FLIC scores with nivolumab treatment.

**References**

1. Dine J, Gordon R, Shames Y, Kasler MK, Barton-Burke M. Immune checkpoint inhibitors: an innovation in immunotherapy for the treatment and management of patients with cancer. Asia Pac J Oncol Nurs. 2017; 4(2):127-135.

2. Shepherd FA, Douillard JY, Blumenschein GR. Immunotherapy for non-small cell lung cancer: Novel approaches to improve patient outcome. J Thorac Oncol. 2011; 6(10): 1763-1773.

3. Ramirez RA, Lu J, Thomas KE. Quality of life for non-small cell lung cancer patients in the age of immunotherapy. Transl Lung Cancer Res. 2018; 7(Suppl 2):149-152.

4. Schadendorf D, Larkin J, Wolchok J, Hodi FS. Chiarion-Sileni V, Gonzalez R, Wagstaff J. Health-related quality of life results from the phase III Check Mate 067 study. Eur J Cancer. 2017; 82: 80-91.

**Ethics Approval**

The study was approved by clinical trials ethics committee of the University of Health Sciences Ankara Oncology Training and Research Hospital (decision number: 2018–04/52) and performed in accordance with the Helsinki Declaration.

#### P320 Novel in vivo preclinical humanized models for the evaluation of human specific immune checkpoint inhibitors

##### Anya Avrutskaya^1^, Fabienne Sonego^2^, Jacob Hauser^1^, Emily O’Koren^1^, Robin Ball^1^, Gaëlle Martin^2^, Julie Chaix^2^, Thi Bui^1^, Ian Belle^1^, Elizabeth Reap^1^, Patrick Fadden, PhD^1^, Chassidy Hall^1^, Kader Thiam^2^, Paula Miliani de Marval^1^

###### ^1^Charles River Discovery, Wilmington, MA, United States; ^2^Genoway, lyon, France

####### **Correspondence:** Paula Miliani de Marval (paula.milianidemarval@crl.com)

**Background**

In the last few years, there has been an increasing demand for suitable pre-clinical mouse models for evaluating the efficacy of checkpoint inhibition-based cancer immunotherapies. During tumor progression, immune cells can become unresponsive and evade immune surveillance upon chronic activation and expression of the programmed cell death protein-1 (PD-1) the ligand PD-L1 on tumor cells or expression of the T lymphocyte associated antigen 4 (CTLA4) in T-cells cells resulting in tumor immune-tolerance. We have previously demonstrated that murine anti-PD-1, anti-PD-L1 and CTLA-4 blockade can effectively enhance immune normalization and re-activate the antitumor response against multiple syngeneic tumor models. While these models proved instrumental for evaluating murine immune-checkpoint inhibitors (ICI), there is a clear need for additional mouse models to evaluate the efficacy of ICI specific for human targets.

**Methods**

To address this need, we describe the development of humanized PD-1 and CTLA-4 knock-in (KI) mouse models. The main advantage of these models is that human PD-1 or CTLA-4 proteins are expressed in the context of a fully functional immune system. To validate these models we evaluated the response to pembrolizumab or ipilimumab in a colorectal carcinoma and a glioblastoma preclinical tumor models.

**Results**

We observed significant tumor growth inhibition and growth delay in the MC38 tumor model with either monotherapy, but not when treated with the murine counterparts: anti-PD-1 (clone RPM1-14) or CTLA-4 (clone 9H10). To extend our validation studies to other tumor models, we implanted GL261 glioblastoma orthotopically in the brain of PD-1 KI mice and achieved a significant increased life span in the group treated with pembrolizumab compared to both the control group and the group treated with murine anti-PD-1 antibody. Furthermore, we found that pembrolizumab and ipilimumab therapy results in enhanced effector functions of CD4+and CD8+ T cells associated with increased expression of Granzyme B.

**Conclusions**

In summary, the results shown here underscore the value of resourcing to humanized knock-in (KI) mouse models as tools to evaluate human specific immune-checkpoint based therapeutics alone and in combination with other agents.

#### P321 Real-world clinical outcomes among patients with advanced non-small cell lung cancer who initiated first-line regimens

##### Eric Nadler, MD^1^, Bhakti Arondekar, PhD^2^, Kathleen Aguilar^3^, Jie Zhou^3^, Jane Chang^2^, Xinke Zhang^4^, Vivek Pawar^4^

###### ^1^Texas Oncology, Medical Oncology, Dallas, TX, United States; ^3^Pfizer Inc., New York, NY, United States; ^4^McKesson Life Sciences, The Woodlands, TX, United States; ^4^EMD Serono, Inc., Billerica, MA, United States

####### **Correspondence:** Eric Nadler (eric.nadler@USONCOLOGY.COM)

**Background**

While clinical trials have demonstrated the clinical benefit of immuno-oncology (IO) regimens for the treatment of advanced non-small cell lung cancer (aNSCLC), either in combination or as monotherapies, limited research has evaluated clinical outcomes with these therapies in a real-world setting. This retrospective observational study evaluated time to treatment discontinuation (TTD) and overall survival (OS) in patients with aNSCLC receiving care in US community oncology clinics. Previous research suggests that TTD is a pragmatic real-world efficacy endpoint, as TTD and progression-free survival (PFS) are associated across different types of therapy in NSCLC clinical trials [1], therefore TTD was explored in this study.

**Methods**

Patients with aNSCLC who initiated first-line (1L) treatment with systemic chemotherapies, targeted therapies, or IO regimens in the US Oncology Network between 3/1/15 and 8/1/18 were included in the study population. Electronic health record data for these patients was captured through 2/1/19. Descriptive analyses were performed to assess baseline characteristics and treatment patterns, and the Kaplan-Meier method was used to evaluate TTD and OS from the start of 1L treatment.

**Results**

In total, 7,746 patients were included in this analysis (Table 1): 5,859 (75.6%) initiated 1L systemic chemotherapies, 656 (8.5%) targeted therapies, 907 (11.7%) IO monotherapies, and 324 (4.2%) IO combination regimens (with chemotherapies or targeted therapies). Of these, 51.8%, 50.3%, 21.7%, and 17.6%, respectively, proceeded to a subsequent treatment following 1L discontinuation. Median TTD ranged from 2.0 months (95% CI 1.9-2.1) in patients who received systemic chemotherapies to 3.5 months (95%CI: 2.8-4.2) in patients who received IO monotherapies (Figure 1). Similarly, median OS was longest in patients who received IO monotherapies (19.9 months [95%CI: 16.6-24.1]; Figure 2).

**Conclusions**

While heterogeneous patient characteristics may have influenced the results of this study, the trends observed suggest favorable outcomes in patients with aNSCLC treated with IO monotherapy in the 1L setting. Further research should explore whether this is related to a predominance of patients with high PD-L1 expression among those who received IO monotherapies. TTD for IO-based therapies in this real-world setting appears to be shorter than PFS reported in previous trials, indicating an unmet need may remain and needs to be explored. However, these results could be influenced by effects of informative censoring or other underlying clinical factors. Additionally, future studies should investigate differences in the tolerability profiles of 1L regimens, as well as how treatment sequences contribute to outcomes.

**Acknowledgements**

This study was funded by Merck KGaA, Darmstadt, Germany, as part of an alliance between Merck KGaA, Darmstadt, Germany and Pfizer Inc., New York, NY, USA.

**Reference**

1. Blumenthal GM, Gong Y, Kehl K, et al. Analysis of time-to-treatment discontinuation of targeted therapy, immunotherapy, and chemotherapy in clinical trials of patients with non-small-cell lung cancer. Ann Oncol. 2019;30(5):830-8.

**Ethics Approval**

The study was reviewed and granted exception and waiver of consent by the US Oncology, Inc. Institutional Review Board.

**Table 1 (abstract P321).**
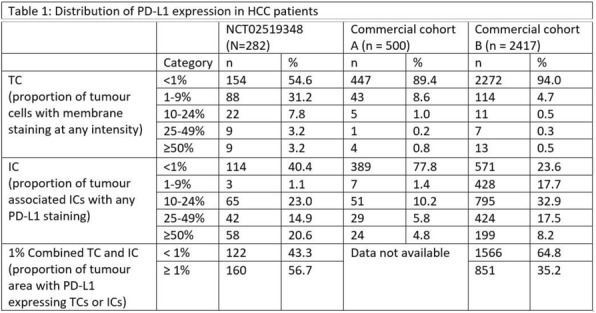
See text for description

**Fig. 1 (abstract P321).**
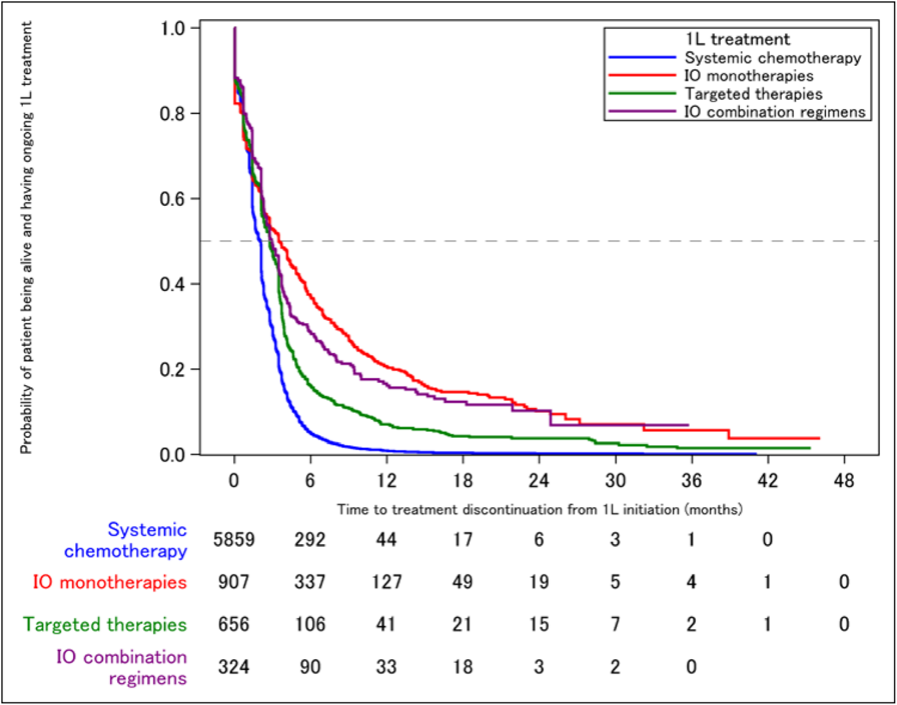
See text for description

**Fig. 2 (abstract P321).**
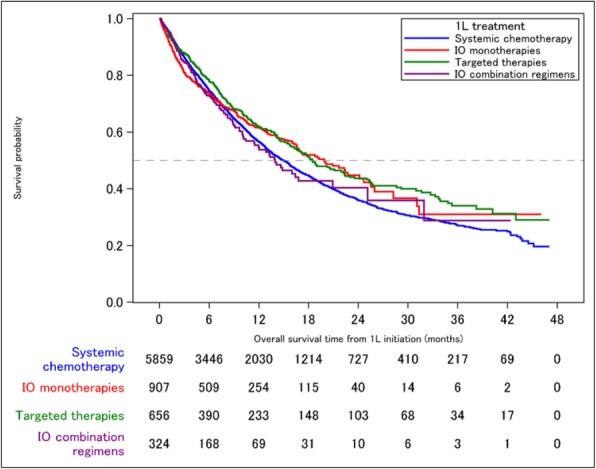
See text for description

#### P322 COM902, a novel therapeutic antibody targeting TIGIT augments T cell function and the activity of PVRIG pathway blockade in vitro and in vivo

##### Maya Kotturi, BS, PhD, Eran Ophir, PhD, Sarah Whelan, PhD, Spencer Liang, Kathryn Logronio, BS PhD, Kyle Hansen, BS, Zoya Alteber, PhD, Mark White, BS, PhD

###### Compugen, South San Francisco, CA,United States

####### **Correspondence:** Eran Ophir (erano@cgen.com)

**Background**

TIGIT is a coinhibitory receptor that is highly expressed on tumor infiltrating lymphocytes (TILs), including effector and regulatory (Treg) CD4+ T cells, effector CD8+ T cells, and NK cells. Engagement of TIGIT with its cognate ligand PVR directly suppresses lymphocyte activation. TIGIT and PVR are broadly expressed in different types of solid tumors, suggesting that TIGIT-PVR signaling may be a dominant immune escape mechanism for cancer. Utilizing COM902, a therapeutic antibody targeting TIGIT, we demonstrate that co-blockade of TIGIT and a new checkpoint inhibitor, PVRIG, augments T cell responses in vitro and in vivo.

**Methods**

Multi-color flow cytometry analysis of dissociated tumors was used to quantify TIGIT and PVRIG expression on TILs. Membranous PVR and PVRL2 expression was characterized by immunohistochemistry. The ability of COM902 to promote T cell responses in vitro, alone and in combination with an anti-PVRIG antibody, COM701, was evaluated in a primary TIL assay. To examine the in vivo effects of TIGIT blockade with COM902 a chimeric antibody with the constant region of mouse IgG1 was generated. The anti-tumor activity of the chimeric COM902 antibody in combination with an anti-mouse PVRIG antibody was assessed in the mouse CT26 colon carcinoma model.

**Results**

COM902 is a fully human antibody that binds TIGIT with high affinity and specificity and disrupts the binding of TIGIT to PVR. This antibody binds to TIGIT on human CD8+ T cells with higher affinity than tested benchmark antibodies. In dissociated tumor samples, TIGIT expression was highest on TILs in endometrial, head and neck, kidney and lung tumors, and directly correlated with PVRIG expression. Except for breast tumors, PVR was moderately to highly expressed in all tumor types examined, while PVRL2 expression was highest in prostate, ovarian, liver and endometrial tumors. Combination of COM902 and COM701 resulted in enhanced CD3+ TIL activity in vitro. Furthermore, the combination of chimeric COM902 and anti-PVRIG resulted in significant CT26 tumor growth inhibition and enhanced overall survival, which was comparable to the combination of chimeric COM902 and anti-PD-L1.

**Conclusions**

We describe the development of a very high affinity antagonistic TIGIT antibody, COM902, that is currently in preclinical development. Co-expression of TIGIT with PVRIG in TILs and their non-redundant inhibitory effects on T cell activation suggest a potential therapeutic advantage in clinical combinations targeting both pathways. Towards this end we are planning a trial that will eventually incorporate combinations of COM902 with the anti-PVRIG antibody, COM701.

#### P323 IPH5301, a CD73 blocking antibody targeting the adenosine immunosuppressive pathway for cancer immunotherapy

##### Ivan Perrot, Caroline Denis, PhD, Marc Giraudon-Paoli, Severine Augier, Rachel Courtois, Diana Jecko, Violette Breso, Thomas Arnoux, Nicolas Gourdin, PhD, Romain Remark, PhD, Cecile Bonnafous, Ariane Morel, PhD, Eric Vivier, Yannis Morel, PhD, Pascale Andre, Carine Paturel, PhD

###### Innate Pharma, Marseille, France

####### **Correspondence:** Pascale Andre (pascale.andre@innate-pharma.fr)

**Background**

CD73 is an extracellular ectonucleotidase highly expressed by tumoral or stromal cells in the tumor microenvironment. By inducing tumor cell death, conventional anti-cancer therapies induce extracellular release of adenosine triphosphate (ATP), which is degraded by CD39 into adenosine monophosphate (AMP) and then by CD73 into adenosine, an inhibitor of immune response. Blockade of CD73-mediated degradation of AMP may therefore stimulate anti-tumor immunity across a wide range of tumors through preventing the production of adenosine. IPH5301 is a humanized effector-silent IgG1 monoclonal antibody that selectively binds to and inhibits the activity of both membrane-bound and soluble human CD73. IPH5301 is designed to enhance anti-tumor immune responses by inhibiting the enzymatic activity of CD73 in the tumor microenvironment, thus releasing tumor-infiltrating lymphocytes from adenosine-mediated suppression. Here, we described the expression of CD73 in several human solid tumors, characterized IPH5301 antibody properties and its efficacy in vitro.

**Methods**

CD73 expression was assessed by immunochemistry on cohorts of solid tumors i.e breast, ovarian, lung, melanoma, pancreatic and head and neck cancer. In vitro efficacy of IPH5301 was evaluated (1) in human T cell proliferation assays; and (2) in enzymatic assays with lymphocytes and serum from healthy donors and human CD73-knock-in (huCD73KI) mice. To get more insight into the mechanism of action of IPH5301, CD73-IPH5301 complex was analyzed using electron microscopy and the crystal structure of IPH5301 Fab in complex with CD73 ectodomain was determined.

**Results**

Whereas inter-patient variability was observed in all tested indications, CD73 expression was always detected mainly on tumor cells and did not correlate with the expression of CD39 or PD-L1. In vitro IPH5301 efficiently restored T cell proliferation and blocked adenosine-mediated suppression of T cell proliferation in a mixed lymphocyte reaction in a dose-dependent manner. IPH5301 did not induce CD73 down-modulation and did not directly activate B cells. Furthermore, IPH5301 efficiently blocked CD73 enzymatic activity in human serum and whole blood as well as in serum and splenocytes from huCD73KI mice. Finally, we showed that IPH5301 contrains CD73 in an intermediate inactive form.

**Conclusions**

These results indicate that IPH5301 blocks CD73 with a differentiated mechanism of action compared to benchmarked anti-CD73 clinical candidates and support the clinical development of IPH5301 for cancer immunotherapy, potentially in combination with chemotherapy or immune checkpoint inhibitors.

**Acknowledgements**

The research leading to CD73 results were obtained within the TumAdoR collaborative consortium that received funding from the European Community's Seventh Framework Program (FP7/2007-2013) under grant agreement n°602200.

### Clinical Trial Completed

#### P324 Pan-tumor analysis of the association of cancer and immune biology-related gene expression signatures with response to pembrolizumab monotherapy

##### Razvan Cristescu, PhD^1^, Michael Nebozhyn, PhD^1^, Chunsheng Zhang, PhD^1^, Andrew Albright, PhD^1^, Julie Kobie, PhD^1^, Lingkang Huang^1^, Qing Zhao, MD PhD^1^, Anran Wang^1^, Hua Ma^1^, Andrea Webber^1^, Petar Jelinic^1^, Mohini Rajasagi^1^, Sandra Souza^1^, Raluca Predoiu^1^, Z. Alexander Cao^1^, Junshui Ma^1^, Michael Morrissey^1^, Clemens Krepler, MD^1^, Stephen Keefe^1^, Jonathan Cheng, MD^1^, Vassiliki Karantza^1^, Sukrut Shah^1^, Rodolfo Perini, MD^1^, Antoni Ribas, MD, PhD^2^, Petros Grivas, MD, PhD^3^, David Cescon^4^, Terrill McClanahan, PhD^1^, Alexandra Snyder, MD^1^, Mark Ayers, PhD^1^, Jared Lunceford, PhD^1^, Andrey Loboda, PhD^1^

###### ^1^Merck Inc., Boston, MA, United States; ^2^University of California, Los Angeles, Los Angeles, CA, United States; ^3^University of Washington, Seattle, WA, United States; ^4^Princess Margaret Cancer Centre, Toronto, Canada

####### **Correspondence:** Razvan Cristescu (razvan_cristescu@merck.com)

**Background**

RNA sequencing (RNASeq) data on baseline tumor biopsies from patients in pembrolizumab monotherapy studies were used to explore potential relationships between key biological gene expression signatures and objective response rate (ORR) in the trials.

**Methods**

A canonical set of 10 consensus signatures representative of key tumor biology and tumor microenvironment (TME) elements beyond the 18-gene T-cell inflamed gene expression profile (GEP [1]) was defined using the independent Merck-Moffitt and TCGA databases [2,3], external to any pembrolizumab trial and prior to relating RNASeq data to clinical outcomes from studies evaluated. These signatures (Angiogenesis, Hypoxia, Glycolysis, Proliferation, MYC, RAS, gMDSC, mMDSC, Stroma/EMT/TGFβ, WNT) were evaluated in the trial dataset blinded to clinical outcome, to test the association with ORR (RECIST 1.1; where response=PR or CR). Studies with available RNASeq data (N=1188) included: KN001/KN006-Melanoma (N=476; pembrolizumab-treated and ipilimumab-naïve), KN052-urothelial (N=186), KN012/KN055-HNSCC (N=147; HPV-negative by whole exome sequencing), KN086-TNBC (N=132), KN059-Gastric (N=92), and KN427-RCC (N=78), KN100-Ovarian (N=77). Pan-cancer logistic regression analysis of ORR for consensus signatures included terms adjusting for cancer type, ECOG performance status, and the T-cell inflamed GEP, an approach equivalent to evaluating association between ORR and the residuals of consensus signatures after detrending them for their relationship with the T-cell inflamed GEP and cancer type. Testing of the 10 pre-specified consensus signatures for negative association (except Proliferation with a hypothesized positive-association) with ORR was adjusted for multiplicity.

**Results**

Covariance patterns of the 11 signatures (including GEP) in Merck-Moffitt and TCGA showed highly concordant co-expression patterns in the RNASeq data from pembrolizumab trials. As anticipated, the T-cell inflamed GEP demonstrated the strongest association with ORR to pembrolizumab. Beyond the positive association seen with T-cell inflamed GEP, three other RNA signatures, Angiogenesis, mMDSC and Stroma/EMT/TGFβ, exhibited negative associations at the 0.05 level after adjusting for multiple testing (Table 1).

**Conclusions**

Pan-cancer testing of exploratory gene expression signatures using the RNASeq platform in 1188 patients from single-arm pembrolizumab trials suggests that features beyond interferon gamma-related T-cell inflammation may be relevant to response to anti-PD1 monotherapy and may define other axes of tumor biology as rational candidates for pembrolizumab combinations. These features (Angiogenesis, mMDSC and Stroma/EMT/TGFβ) have been previously hypothesized to represent immune-suppressive axes with potential negative impact on immunotherapy efficacy. Future evaluation of the association of these signatures with response and survival outcomes in other cancer types and in randomized settings will provide additional insight into their prognostic or predictive character.

**Acknowledgements**

Joanne E Tomassini for writing support and Sheila Erespe for editorial support, both employees of Merck & Co., Inc.

**Trial Registration**

NCT01295827, NCT01866319, NCT02335411; NCT02335424; NCT01848834; NCT02255097; NCT02447003; NCT02674061; NCT02853344

**References**

1. Ayers M, Lunceford J, Nebozhyn M, et al. IFN-gamma-related mRNA profile predicts clinical response to PD-1 blockade. J Clin Invest 2017;127:2930-2940.

2. Cristescu R, Mogg R, Ayers M, et al. Pan-tumor genomic biomarkers for PD-1 checkpoint blockade-based immunotherapy. Science 2018;362:eaar3593.

3. Ayers M, Nebozhyn M, Cristescu R, et al. Molecular Profiling of Cohorts of Tumor Samples to Guide Clinical Development of Pembrolizumab as Monotherapy. Clin Cancer Res 2019;25:1564-1573.

**Ethics Approval**

The clinical trials included in this analysis were approved by the appropriate ethics committees at each participating study center.

**Table 1 (abstract P324).**
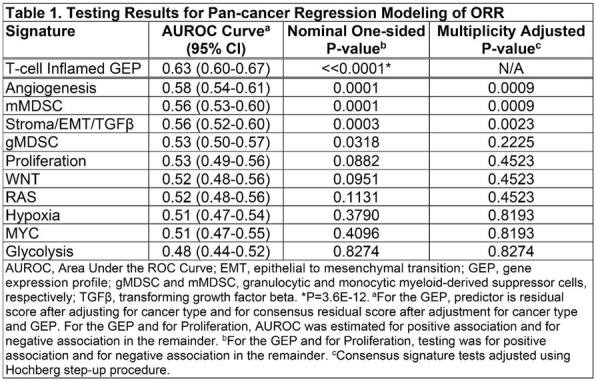
See text for description

#### P325 Tumor Infiltrating Lymphocytes (TILs) in triple-negative breast cancer: High Immunoscore is associated with pathological CR in patients receiving neoadjuvant chemotherapy.

##### Bernardo Rapoport, MD^1^, Simon Nayler^2^, Jerome Galon, Dr^3^, T Mlecnik^4^, Teresa Smit^1^, Jacqui Barnard-Tidy^1^, Aurelie Fugon^4^, Marine Martel^5^, Ronald Anderson, Professor^6^, Carol Benn^2^

###### ^1^The Medical Oncology Centre of Rosebank, Johannesburg, South Africa; ^2^Prof., Johannesburg, South Africa; ^3^LABORATORY OF INTEGRATIVE CANCER IMMUNOL, France, France; ^4^Luminy Biotech enterprises 163 Ave de Lu, Marseille, France; ^5^HalioDx, Marseille, France; ^6^Immunology,University of Pretoria, Pretoria, South Africa

####### **Correspondence:** Teresa Smit (pharmacist@rapoport.co.za)

**Background**

The presence of high levels of tumor infiltrating lymphocytes (TILs) has been associated with better prognosis in early triple-negative breast cancer (TNBC). Immunoscore is a prognostic tool, which categorizes the densities of spatially positioned CD3 and CD8 cells in both invasive margins (IM) and the center of the tumor (CT) yielding a five-tiered classification (0–4). High immunoscores have been reported to be associated with improved outcomes in patients with colorectal cancer.

**Methods**

We performed the Immunoscore in a cohort of 103 breast cancer (BC) patients previously receiving neo-adjuvant chemotherapy. There were triple-negative (TNBC)=53, Luminal=32, Her2+=18 who received treatment with anthracycline and/or taxane- and/or trastuzumab-based neo-adjuvant chemotherapy. Pre-treatment tumor samples were immune-stained for CD3 and CD8 T-cell markers. Quantitative analysis of the immune cells was carried out using a computer-assisted image analysis in different tumor locations.

**Results**

The pathological complete response (pCR) rate of the entire cohort was 44%. On univariate analysis factors associated with higher pCR included primary tumor size (T1=43.48% vs. T2=52.31% vs. T3+T4 6.67%, Chi2=10.3201, p40=56.86% vs.15-39=40.54% vs.

T-cell density subsets (CD3, CD8) and Immunoscore were significantly higher in TNBC compared to non-TNBC patients. Receiver-operating characteristic (ROC) curve analysis was used to determine the optimal cut-off points for CD3 and CD8. A high density of CD3 (> than 800mm2) and CD8 (> than 400mm2) positive T-cells in the CT was associated with higher pCR (CD3 CT:60% vs.25%, p=0.00035 and CD8 CT: 64% vs.27%, p=0.00016). Analysis of CD3 (> than 1400mm2) (CD3 IM:63% vs.19%, p=0.0001) and CD8 densities in the IM (> than 500mm2) was also significantly associated with pCR (CD8 IM:63% vs. 15%, p=0.00003). High immunoscore (24/38 pts (63%)) vs. intermediate (17/48 pts (35%)) vs. low (4/17 pts (24%)) was significantly associated with pCR (p=0.00674). In a logistic regression model, Ki-67 (p

**Conclusions**

The results of this study show a significant prognostic and potentially predictive role for the Immunoscore and Ki-67 in BC patients, particularly in the TNBC subset.

**Acknowledgements**

Dr Ronwyn van Eeden

**Ethics Approval**

Ethics Approval was obtained from Pharmaethics SA and University of PTA, approval no 517/2017

**Consent**

Written informed consent was obtained from the patient for publication of this abstract and any accompanying images. A copy of the written consent is available for review by the Editor of this journal.

#### P326 A retrospective analysis of DNA plasmid and peptide-based vaccine therapy in treatment of HER-2/neu+ breast cancer

##### Aaron Stewart, BS^1^, William Gwin, MD^1^, Mary Disis, MD, FACP^1^, Jennifer Childs^1^, James Dai^2^, Doreen Higgins, RN, BSN, OCN^1^, Angela Kask^1^

###### ^1^University of Washington, Seattle, WA, United States; ^2^Fred Hutchinson Cancer Research Center, Seattle, WA, United States

####### **Correspondence:** William Gwin (wrgwin@medicine.uw.edu)

**Background**

Patients with HER-2/neu+ overexpressing breast cancer often lose immunity toward the HER2 antigen [1]. Vaccines are capable of inducing a cytotoxic T lymphocyte immune response toward overexpressing antigens, leading to targeted tumor destruction [2-4]. Clinical trials have explored peptide-based and DNA-based vaccines as possible vehicles for vaccine delivery, though a direct comparison of safety and immunogenicity has not been previously studied[5-6]. We hypothesize that the DNA-based vaccine will produce superior immunogenicity due to stable plasmid persistence within the tissue leading to prolonged HER2 immune response[7].

**Methods**

We retrospectively analyzed adverse events and ELISpot data from 104 patients treated with vaccines targeting the intracellular domain of HER2/neu using either DNA or peptide fragments.

**Results**

Adverse event profiles of the 104 patients analyzed were similar with no reported grade 3, 4, or 5 events. There was no significant effect on left ventricular ejection fraction (p=0.88 and p=0.59). Patients with low initial immunity, characterized as 0.05. Furthermore, patients with low initial immunity who received the DNA-based vaccine showed improved immunogenicity towards the unvaccinated extracellular domain of HER2 at 1 month and 6 months, p

**Conclusions**

A comparison of DNA-based and peptide-based vaccines targeting HER2 intracellular domain epitopes revealed a more robust and long-lasting immunogenic response with the DNA-based vaccine while maintaining an excellent safety profile. Additionally, immune responses to extracellular domain regions of HER2 demonstrate the intra-epitope spreading potential in the DNA-based vaccine.

**Acknowledgements**

A special thanks to the Boyd Scholarship for financial assistance during my research.

**Trial Registration**

Clinicaltrials.gov: NCT00436254 and NCT0034310

**References**

1. Datta J., et. al. Progressive loss of anti-HER2 CD4+ T-helper type 1 response in breast tumorigenesis and the potential for immune restoration. Oncoimmunology. 2015;4(10):e1022301.

2. Mittendorf EA, et. al. Clinical trial results of the HER-2/neu (E75) vaccine to prevent breast cancer recurrence in high-risk patients: from US Military Cancer Institute Clinical Trials Group Study I-01 and I-02. Cancer. 2012;118(10):2594-602.

3. Disis, M.L., et al., Generation of T cell immunity of the HER-2/neu protein after active immunization with HER-2/neu peptide based vaccine. J. Clin Onc, 2002;20(11): 26424-2632

4. Disis, M.L., et al., Generation of immunity to the HER-2/neu oncogenic protein in patients with breast and ovarian cancer using a peptide-based vaccine. Clin Cancer Res, 1999;5(6):1289-97.

5. Disis ML, et. al. A phase I trial of the safety and immunogenicity of a DNA- based vaccine encoding the HER-2/neu (HER2) intracellular domain in subjects with HER2+ breast cancer. ASCOAnnual Meeting. 2014;

6. Disis ML, et. al. Phase II study of a HER-2/neu (HER2) intracellular domain (ICD) vaccine given concurrently with trastuzumab in patients with newly diagnosed advanced stage breast cancer. SABCC. 2009;

7. Aurisicchio L., Ciliberto G. Genetic cancer vaccines: Current status and perspectives. Expert Opin. Biol. Ther. 2012;12:1043–1058.

#### P327 Evaluation of PD-L1 and cutoff selection to define a predictive biomarker for pembrolizumab monotherapy in esophageal cancer using KEYNOTE-180

##### Mary Savage, Serafino Pantano, Qi Liu, Jared Lunceford, PhD, Peter Kang, Pooja Bhagia, MBBS, MD, Kenneth Emancipator, MD

###### Merck & Co., Inc., Kenilworth, NJ, USA, Kenilworth, NJ, United States

####### **Correspondence:** Kenneth Emancipator (kenneth.emancipator@merck.com)

**Background**

Interim analysis of the KEYNOTE-180 study (NCT02559687) was used to establish a relationship between PD-L1 expression levels and objective response rate (ORR) in patients with esophageal cancer whose disease progressed after ≥2 lines of therapy and to select a PD-L1 cutoff for further validation in the randomized setting (ie, KEYNOTE-181).

**Methods**

KEYNOTE-180 was a single-arm, open-label, phase 2 study of pembrolizumab in patients with previously treated, advanced esophageal cancer. Pembrolizumab 200 mg was given intravenously every 3 weeks. The primary objective was ORR. Patients were required to provide a tumor sample for retrospective analysis of biomarkers, which may predict response to pembrolizumab. PD-L1 expression was measured using the PD-L1 IHC 22C3 PharmDx assay and evaluated using a combined positive score (CPS). CPS is the ratio of PD-L1–expressing cells (tumor cells, lymphocytes, macrophages) to viable tumor cells. Testing for a relationship between CPS and ORR (per RECIST v1.1 by central review) was conducted using logistic regression. Cutoff selection proceeded by joint evaluation of the ORR enrichment profile, the sensitivity and specificity profile (receiver operating characteristic analysis), the prevalence of patients with late-line esophageal cancer selected by the cutoff, and trends in overall survival (OS) by Kaplan-Meier (KM) curves.

**Results**

There were 8 responders among 105 patients with available PD-L1 results at the time of interim analysis (March 1, 2017). CPS was statistically significantly associated (P=0.03) with probability of response. A cutoff of CPS 1 did not show enrichment of ORR, whereas higher cutoffs did (Table). CPS 10 showed >3-fold enrichment in ORR above versus below the cutoff. Although higher cutoffs indicated further enrichment for ORR, attendant drops in sensitivity and prevalence occurred. Separation between KM OS curves for CPS ≥10 versus CPS

**Conclusions**

CPS was useful for identifying patients who responded to pembrolizumab monotherapy. Through the evaluation of several clinical utility dimensions, CPS 10 was chosen for further validation in the randomized setting based on its ability to enrich for ORR and simultaneously to preserve sensitivity. The preservation of sensitivity, along with prevalence, was considered particularly important because of the safety profile of pembrolizumab and the paucity of treatment options in this patient population.

**Trial Registration**

ClinicalTrials.gov, NCT02559687

**Ethics Approval**

The study and the protocol were approved by the Institutional Review Board or ethics committee at each site.

**Consent**

All patients provided written informed consent to participate in the clinical trial.

**Table 1 (abstract P327).**
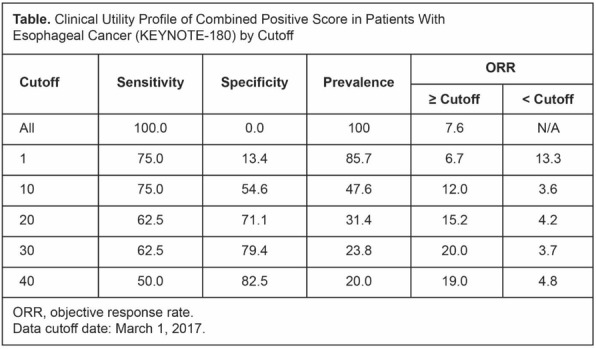
See text for description

#### P328 A phase IIB study of Pembrolizumab plus BL-8040 in metastatic pancreatic cancer: Clinical outcomes and biological correlates

##### David Fogelman, MD^1^, Michael Overman, MD^1^, Robert Wolff^1^, Milind Javle, MD^1^, Shubham Pant, MBBS^1^, Gauri Varadhachary^1^, Rachna Shroff^2^, Ignacio Wistuba, MD^1^, Carmelia Barreto, PhD^1^, Renganayaki Pandurengan, MS^1^, Sandesh Subramanya, PhD^3^, Debora Ledesma, PhD^3^, Abi Vainstein-Haras, MD^4^, Ella Sorani, PhD^4^, Tzipora Lustig^4^, Osnat Kashtan^4^, Yosi Gozlan^4^, Steven Townson, PhD^5^, Jeanne Fahey, PhD^6^, James Yao, MD^1^

###### ^1^M.D. Anderson Cancer Center, Houston, TX, United States; ^2^University of Arizona, Tuscon, AZ, United States; ^3^MD Anderson Cancer Center, Houston, TX, United States; ^4^Bioline Rx, Modi'in, Israel; ^5^Merck & Co., Inc., Shoreline, WA, United States; ^6^Merck & Co., Inc, Boston, MA, United States

####### **Correspondence:** David Fogelman (dfogelman@mdanderson.org)

**Background**

BL-8040 (BL), a CXCR4 antagonist, increases T cell entry into the bloodstream and thereafter into tumor in humans. We hypothesized that BL in combination with pembrolizumab (P) may thereby promote efficacy in metastatic pancreatic cancer (mPC).

**Methods**

Methods: This phase IIb open label study enrolled patients with progression after at least one prior chemotherapy for mPC. Two weeks of single agent BL (1.25 mg/kg) was followed by 3-week cycles of P (200 mg IV d1) plus BL (d1,4,8,11). Biopsies for tumor biology were performed before treatment, after BL monotherapy (optional), and after BL/P combination

**Results**

As of July 2019, 20 pts enrolled; 15 were evaluable for the primary endpoint of radiologic response. Baseline characteristics: median age 66, 10M/10F, median 2 prior lines of therapy (range 1-3). Best overall response includes 1 PR, 2 SD, 12 PD yielding 21.4% disease control (1PR+2SD). Median TTP was 2 months overall and 7 months for the PR/SD pts. Median OS was 7 months overall and 12 months in PR/SD pts. The combination was well tolerated with most AEs being injection site discomfort. Five patients experienced grade 3/4 toxicities. Grade 3 toxicities included HTN (n = 1), Alk Phos (n = 1), N/V (n = 2), ascites (n = 1), dyspnea (n = 1), and abd pain (n = 2). One pt had grade 4 dyspnea. Paired biopsies have been analyzed for six patients (1 PR 2 SD 3 PD). Patients with PR/SD had, at baseline, trends towards greater T cell, especially cytotoxic CD8+ T cell counts within the tumor niche than patients with PD (T cells: 188-627 cells/mm2 vs 7-41 cells/mm2, Cytotoxic CD8+ T cells: 18-137 cells/mm2 vs 0-2 cells/mm2). The PR patient demonstrated an increase in cytotoxic CD8+ T cell number in the tumor niche and reduction in the stroma following treatment. Additional molecular profiling data from multiplex IF will be available at the meeting.

**Conclusions**

This combination of immunotherapy with pembrolizumab plus BL-8040, without cytotoxic chemotherapy, shows clinical activity in patients with pancreatic cancer even in this heavily pretreated population. The combination was well tolerated. We noted a trend towards greater CD8+ T cell infiltrate at baseline within the tumor cell niche among patients who demonstrated clinical benefit. OS for all comers was longer than expected in this heavily pretreated patient group suggesting that P + BL might have a salutary effect on survival time if used earlier in the disease course.

**Trial Registration**

NCT02907099

**Ethics Approval**

This study was approved by the M.D. Anderson Institutional Review Board, approval number 2016-0410.

#### P329 PolyPEPI1018 off-the shelf vaccine as add-on to maintenance therapy achieved durable treatment responses in patients with microsatellite-stable metastatic colorectal cancer patients (MSS mCRC)

##### Joleen Hubbard, MD^1^, Chiara Cremolini, MD^2^, Rondell Graham, MD^1^, Roberto Moretto, MD^2^, Jessica Mitchell, CNP^1^, Jaclynn Wessling^1^, Eniko Toke^3^, Zsolt Csiszovszki, PhD^3^, Orsolya Lőrincz^3^, Levente Molnár^3^, Eszter Somogyi^3^, Mónika Megyesi^3^, Kata Pántya^3^, József Tóth^3^, Péter Páles^3^, István Miklós^3^, Alfredo Falcone, MD^2^, Joleen Hubbard, MD^1^

###### ^1^Mayo Clinic, Rochester, MN, United States; ^2^Azienda Ospedaliera Universitaria Pisana, Pisa, Italy; ^3^Treos Bio, Budapest, Hungary

####### **Correspondence:** Joleen Hubbard (joleenhubbard@gmail.com)

**Background**

PolyPEPI1018 is an off-the-shelf, multi-peptide vaccine against CRC, containing 12 immunogenic epitopes derived from 7 conserved cancer antigens frequently expressed in mCRC based on the analysis of 2,931 biopsies. Here we report the results of the phase I study of PolyPEPI1018 vaccine as an add-on to maintenance therapy in MSS mCRC patients.

**Methods**

11 patients with MSS mCRC in the first-line setting were vaccinated with PolyPEPI1018 just after the transition to maintenance therapy with a fluoropyrimidine and a targeted agent (bevacizumab). (Part A: n= 5, single dose, 12 weeks follow-up; Part B: n= 6, 3 doses, Q12W). Primary endpoints were safety and immunogenicity. Multiple analysis of vaccine-induced immune responses in blood and tumor were performed. Both immune response and clinical benefit were predicted using the autologous HLA-genotype determined from patient’s saliva sample.

**Results**

The vaccine was well tolerated; most common side effects were transient skin reactions and flu-like syndrome. No vaccine-related SAE occurred. 90% of patients had vaccine-specific CD8+ T-cell responses of memory-effector type against at least 2 of the 7 vaccine antigens, 5 on average. Vaccine specific CD4+ T-cell responses were detected in all patients. Ex vivo CD8+ T cell responses of effector type were detected in 71% of patients, as well as increased fractions of CRC-reactive, polyfunctional, circulating CD8+ and CD4+ T cells in patient’s PBMC after vaccination. Among the 11 patients 3 patients had objective tumor response according to RECIST v1.1, one of them received a single dose and 2 of them received 3 doses. For the Part B of the study, the Objective Response Rate (ORR) was 33% (2/6) and the Disease Control Rate (DCR) was 67% (4/6). Notably, one patient experienced complete tumor shrinkage on 2 of 3 target lesions and partial response on 1 lesion after 25 weeks of treatment, qualifying for curative surgery. Median duration of disease control was 9 months (95%CI 6.3-11.5) (mPFS not reached during the study). The 10 month PFS was 50% (3/6). Predicted vaccine antigen-specific CD8+ T cell responses were confirmed in vitro with a PPV of 79% (p=0.01). Predicted multiantigenic immune responses tend to correlate with both PFS and tumor volume reduction.

**Conclusions**

Treatment with PolyPEPI1018 vaccine and maintenance therapy was safe, well-tolerated, and demonstrated evidence of immunological and clinical activity in MSS mCRC tumors. In addition predicted multiantigenic immune responses indicated treatment benefit, which supports further development of a companion diagnostic together with the vaccine.

**Trial Registration**

NCT03391232

**Ethics Approval**

This study was approved by Mayo Clinic Institutional Review Board and by Central Ethics Committee, Italy (Protocol number: OBERTO-101).

#### P330 Results from the completed dose-escalation of the alloSHRINK phase I study evaluating the allogeneic NKG2D-based CAR T-cell therapy CYAD-101 in metastatic colorectal cancer patients

##### Hans Prenen, MD^1^, Marika Rasschaert, MD^1^, Alain Hendlisz, MD^2^, Leila Shaza, MD^2^, Erik Alcantar-Orozco, MD, PhD^3^, Emilie Cerf, PhD^3^, Florence Renard^3^, Caroline Lonez, PhD^3^, Anne Flament^3^, Jeroen Dekervel, MD^4^, Eric Van Cutsem, MD, PhD^4^

###### ^1^University Hospital Antwerp (UZ Antwerp), Edegem, Belgium; ^2^Institut Jules Bordet, BRUSSELS, Belgium; ^3^Celyad, Mont-Saint-Guibert, Belgium; ^4^University Hospital Leuven (UZ Leuven), Leuven, Belgium

####### **Correspondence:** Caroline Lonez (clonez@celyad.com)

**Background**

Current success of chimeric antigen receptor T-cell (CAR-T) therapy in hematological malignancies has been achieved using autologous cell products. Whilst feasible in such relatively small patient populations, delivering an autologous product to large cohorts of patients is likely beyond the current logistical capabilities. In the phase 1 alloSHRINK study, we tested the first-in-class non-gene edited allogeneic CAR T-cell therapy, CYAD-101, administered concurrently with chemotherapy, for the treatment of metastatic colorectal cancer (mCRC). The NKG2D-based CAR of CYAD-101 targets eight ligands present at high frequencies in mCRC, not only on tumor cells but also cells from the tumor microenvironment, and co-express a T-cell receptor (TCR) inhibiting molecule (TIM) that interferes with TCR signaling in an attempt to avoid the main issue of allogeneic T-cell therapy, the graft versus host disease (GvHD).

**Methods**

The alloSHRINK study (NCT03692429) evaluates the safety and clinical activity of multiple infusions of CYAD-101, administered concurrently with standard of care FOLFOX chemotherapy, in patients with non-resectable mCRC who received prior chemotherapy lines (i.e. rechallenge population). Three dose-levels (DL; 1x10E8, 3x10E8 and 1x10E9 T-cells per infusion) were evaluated through a 3+3 design.

**Results**

In total 12 patients have been enrolled in the dose escalation segment, now completed (3 at DL1, 3 at DL2 and 6 at DL3). At the time of submission, only data from the first two DLs were available. At DL1 and DL2, there was no report of dose-limiting toxicity (DLT) and no patient experienced Grade ≥ 3 related adverse events (uncleaned database). No clinical evidence of GvHD has been recorded. Best overall response ≥ 3 months include 1 partial response and 3 stable disease over the first 6 patients (DL1 and 2). At DL1 and 2, preliminary data show a dose-dependent effect on the cell kinetics and control of the host-versus-graft response against CYAD-101 cells as evidenced by the similar levels of CYAD-101 engraftment after 2nd and 3rd infusions.

**Conclusions**

As of August 2019, no GvHD has been observed following infusions of non-gene edited allogeneic CAR T-cells to mCRC patients at the first two DLs, with preliminary signals of clinical activity. The study will have reached protocol-specified endpoints for analysis at the time of presentation and safety, clinical and cell engraftment will be presented. The results from this study, in comparison with a study evaluating the autologous analog of CYAD-101 in mCRC will provide critical information to support the development of CAR-T therapy in solid tumors.

**Trial Registration**

NCT03692429

**Ethics Approval**

The study was approved by all relevant Belgian Institution‘s Ethics Boards and authorities.

#### P331 Results from the completed dose-escalation phase I SHRINK study evaluating the autologous NKG2D-based CAR T-cell therapy CYAD-01 in metastatic colorectal cancer patients

##### Leila Shaza, MD^1^, Alain Hendlisz, MD^1^, Ahmad Awada, MD, PhD^1^, Jean-Luc Canon, MD^2^, Javier Carrasco^2^, Eric Van Cutsem, MD, PhD^3^, Jeroen Dekervel, MD^3^, Erik Alcantar-Orozco, MD, PhD^4^, Florence Renard^4^, Emilie Cerf, PhD^4^, Caroline Lonez, PhD^4^, Anne Flament^4^, Marc Van den Eynde, MD^5^, Jean-Pascal Machiels, MD, PhD^5^

###### ^1^Institut Jules Bordet, Brussels, Belgium; ^2^Grand Hôpital de Charleroi (GHdC), Charleroi, Belgium; ^3^University Hospital Leuven (UZ Leuven), Leuven, Belgium; ^4^Celyad, Mont-Saint-Guibert, Belgium; ^5^Cliniques Universitaires Saint Luc, Brussels, Brussels, Belgium

####### **Correspondence:** Caroline Lonez (clonez@celyad.com)

**Background**

Chimeric antigen receptor T-cell (CAR-Ts) therapies have yet to demonstrate positive results in the context of solid tumors largely due to the lack of suitable target antigens. NKG2D-based CARs target 8 stress ligands notably expressed to a very high frequency across the metastatic colorectal cancer (mCRC) patient population. The autologous NKG2D-based CAR-T therapy CYAD-01 achieved stable disease in several patients with mCRC when given as a monotherapy in a multiple injection setting without any other supportive therapy (THINK study). In the SHRINK phase 1 study, CYAD-01 was given concurrently with FOLFOX chemotherapy.

**Methods**

The SHRINK phase 1 study (NCT03310008) evaluated the safety and clinical activity of multiple infusions of CYAD-01, administered concurrently with FOLFOX chemotherapy in mCRC patients. Three dose-levels (DL; 1x10E8, 3x10E8 and 1x10E9 T-cells per infusion) were evaluated through a 3+3 design in two different mCRC patient populations: (i) resectable liver dominant mCRC with FOLFOX chemotherapy as 1st line treatment (i.e. neoadjuvant population), and (ii) non-resectable mCRC with prior chemotherapy lines for mCRC including FOLFOX and/or FOLFIRI (i.e. rechallenge population).

**Results**

The three DL have been completed with 9 patients in total (3 at each DL), without any report of dose-limiting toxicity (DLT). Only 1 patient experienced Grade 3 related adverse event (AE) and no patient experienced Grade 4 related AE (uncleaned database as of August 2019). Best overall response ≥ 3 months includes 1 partial response and 6 stable disease out of 9 patients. Preliminary data show a dose-dependent effect on the cell kinetics. The study will have reached protocol-specified endpoints for analysis at the time of presentation.

**Conclusions**

Early data show preliminary signs of clinical activity with the concurrent administration of CYAD-01 and FOLFOX chemotherapy in the present SHRINK study. Safety, clinical and translational research data (cell engraftment) will be presented. The results from this study, in comparison with the results from a similar Phase I study evaluating the allogeneic analog of CYAD-01 in mCRC patients (i.e. CYAD-101), will provide critical information to support the development of CAR T-cell therapy in solid tumors.

**Trial Registration**

NCT03310008

**Ethics Approval**

The study was approved by all relevant Belgian Institution‘s Ethics Boards and authorities.

#### P332 Cancer vaccine against prostate cancer antigen TARP induces antigen-specific CD8+ T cells with upregulation of activation marker PD1 in patients with decreased PSA velocity in D0 prostate cancer

##### Hoyoung Maeng, MD^1^, Brittni Moore^1^, Lauren Wood, MD^2^, Seth Steinberg^1^, Katherine McKinnon, MS^1^, Masaki Terabe, PhD^1^, Purevdorj Olkhanud^1^, Ira Pastan, MD^1^, Jay Berzofsky, MD, PhD^1^

###### ^1^National Cancer Institute, Bethesda, MD, United States; ^2^PDS Biotechnology, Berkeley Heights, NJ, United States

####### **Correspondence:** Hoyoung Maeng (hoyoung.maeng@nih.gov)

**Background**

With the success of immune checkpoint inhibitors, anti-tumor immunity is at the focus of cancer therapy. The pursuit of the mechanism to boost anti-tumor T-cell responses is critical to improve the suboptimal response rate to checkpoint inhibitors. Cancer vaccines can be used to induce such tumor-specific T-cell responses as one of the solutions to low responses to checkpoint inhibitors.

**Methods**

The antigen-specific T cell response in the patients who received a vaccine targeting the prostate cancer antigen, TARP (TCRγ alternate reading frame protein)[1] for biochemically recurrent (D0) prostate cancer on NCT00972309 was assessed. Patients with HLA-A0201 received vaccination at weeks 3, 6, 9, 12 and 15 following 1:1 randomization between a vaccine consisting of TARP peptides, montanide ISA 51 VG and GM-CSF versus autologous dendritic cell (DC) pulsed with TARP peptides. Both vaccines used two types of peptides; wild type (WT) TARP 27-35 (TARP2735) and epitope-enhanced TARP 29-37 peptides (TARP2937-9V). The peripheral blood mononuclear cells were collected at baseline and study weeks following vaccination to be stored in liquid nitrogen until analysis. Cells were thawed and stimulated in vitro with TARP peptides with cytokine support for multicolor flow cytometry.

**Results**

CD8+ T cell subsets were analyzed for association with the disease response in 5 patients each who were responders and non-responders whereas response was defined as slowing of PSA slope log value as previously published by Wood et al [3]. The proportion of PD1-expressing CD8+ cells showed statistically important differences from baseline with an increase in responders and a decrease in non-responders at week 12 after stimulation with TARP 2735 (p= 0.016), TARP 2937 (p=0.032) and TARP 2937-9V (p= 0.016) peptides. Other CD8+ subsets with granzyme A, IFN-γ, IL-2, TNFα and perforin positive cells were investigated. CD8+TNFα+ cells at week 12 (p=0.032) and CD8+perforin+ cells at week 18 (p=0.032) stimulated with TARP 2937 also showed statistically large differences with an increase in responders and a decrease in non-responders.

**Conclusions**

Antigen-specific CD8+ T cells from responders as defined by reduced PSA slope log showed statistically greater activation as assessed by expression of activation marker PD-1 than those from non-responders after the vaccination in a first-in-human trial targeting TARP. The antigen-specific T cell response to the vaccine peptides is an immune correlate of vaccine-induced protection and may be used to guide the ongoing study NCT02362451 that does not have HLA restrictions.

**Acknowledgements**

This work was supported by the Center for Cancer Research, National Cancer Institute, National Institue of Health.

**Trial Registration**

NCT00972309

**References**

1. Wolfgang CD, Essand M, Vincent JJ et al. TARP: a nuclear protein expressed in prostate and breast cancer cells derived from an alternate reading frame of the T cell receptor gamma chain locus. Proc Natl Acad Sci U S A. 2000;97(17):9437–9442.

2. Oh S, Terabe M, Pendleton CD et al. Human CTLs to wild-type and enhanced epitopes of a novel prostate and breast tumor-associated protein, TARP, lyse human breast cancer cells. Cancer Res 2004;64(7):2610-2618.

3. Wood LV, Fojo A, Roberson BD, et al. TARP vaccination is associated with slowing in PSA velocity and decreasing tumor growth rates in patients with Stage D0 prostate cancer. Oncoimmunology. 2016;5(8):e1197459.

**Ethics Approval**

The study was approved by the National Cancer Institute Ethics Board assigned a local number 09C0139, approval number P08397.”

#### P333 Timed anti-tumor vaccination during chemotherapy induces strong T-cell immunity and prolonged survival of late stage cervical cancer patients

##### Marij Schoenmaekers-Welters, PhD^1^, Marij Welters, PhD^1^, Cornelis Melief, MD, PhD^2^, Ignace Vergrote^3^, Judith Kroep, MD, PhD^1^, Gemma Kenter, MD,PhD^4^, Nelleke Ottevanger^5^, Wiebren Tjalma^6^, Hannelore Denys^7^, Mariette van Poelgeest, MD, PhD^1^, Hans Nijman^8^, Anna Reyners^8^, Thierry Velu^9^, Frederic Goffin^9^, Roy Lalisang^10^, Nikki Loof^1^, Sanne Boekestijn^1^, Willem Jan Krebber^2^, Leon Hooftman^2^, Sonja Visscher^2^, Brent Blumenstein, PhD^11^, Richard Stead^2^, Winald Gerritsen^5^, Sjoerd van der Burg, PhD^1^

###### ^1^Leiden University Medical Center, Leiden, ZA, Netherlands; ^2^ISA Pharmaceuticals, Leiden, Netherlands; ^3^University Hospital Leuven, Leuven, Belgium; ^4^Center for Gynecological Oncology, Amsterdam, Netherlands; ^5^Nijmegen University Medical Center, Nijmegen, Netherlands; ^6^University Hospital Antwerp, Antwerp, Belgium; ^7^University Hospital Gent, Gent, Belgium; ^8^University Medical Center Groningen, Groningen, Netherlands; ^9^Chirec Cancer Institute, Maastricht, Netherlands; ^10^University Medical Center Maastricht, Maastricht, Netherlands; ^11^Trial Architecture Consulting, Washington, DC, United States

####### **Correspondence:** Marij Schoenmaekers-Welters (M.J.P.Schoenmaekers-Welters@lumc.nl)

**Background**

High-risk human papilloma virus type 16 (HPV16) is the major cause of inducing cervical cancer. The oncoproteins E6 and E7 are responsible for the cancer development and therefore targeted by the therapeutic synthetic long peptide (SLP) vaccine ISA101. Monotherapy induced HPV16 E6/E7-specific T cells and was clinically effective in half of the HPV16-SLP vaccinated patients with HPV16+ high-grade premalignant lesions of the vulva. However, in HPV16+ cervical cancer patients additional measures need to be taken as the vaccine-induced T cells encounter an immunosuppressive milieu in the tumor microenvironment. Therefore, in the current study the ISA101 vaccination is combined with standard-of-care chemotherapy.

**Methods**

Late stage cervical cancer patients (n=77) were treated 3 times with ISA101 with a 3-week interval in a single arm dose escalation study testing 4 different doses of ISA101 and with the addition or not of pegylated IFN alpha (PegIntron). The start of ISA101 vaccination was at day 15 after the second cycle of standard-of-care chemotherapy, which consisted of carboplatin (AUC6)/paclitaxel (175mg/m2). Blood samples taken during the study were subjected to a set of complementary immune assays to determine the vaccine-induced T-cell responses.

**Results**

In 43% of the 72 evaluated patients an objective clinical response was observed. Carboplatin/paclitaxel depleted myeloid suppressive cells (p

**Conclusions**

Our study demonstrates that chemotherapy combined with immunotherapy, in this case HPV16-SLP vaccination, can be exploited to effectively treat HPV16+ cervical cancer patients and warrants confirmation in a randomized controlled trial.

**Acknowledgements**

This work was financially supported by the Dutch Cancer Society grant 2009-4400 (to C.J.M. Melief and S.H. van der Burg). ISA Pharmaceuticals sponsored the trial.

**Trial Registration**

ClinicalTrials.gov NCT02128126.

**Ethics Approval**

This study was approved by the Central Committee of Human Investigations and by the ethical board of the Leiden University Medical Center (LUMC): EudraCT 2013-1804-12.

#### P334 Concurrent cetuximab (CTX) and nivolumab (NIVO) in patients with recurrent and/or metastatic (R/M) head and neck squamous cell carcinoma (HNSCC): Safety results of a phase I/II study

##### Christine Chung, MD^1^, Marcelo Bonomi, MD^2^, Conor Steuer, MD^3^, Michael Schell^1^, Jiannong Li^1^, Matthew Johnson^1^, Caitlin McMullen^1^, J. Trad Wadsworth^1^, Krupal Patel^1^, Julie Kish, MD^1^, Jameel Muzaffar, MD^1^, Kedar Kirtane^1^, James Rocco^4^, Nabil Saba, MD^3^

###### ^1^Moffitt Cancer Center, Tampa, FL, United States; ^2^Ohio State University Medical Center, Columbus, OH, United States; ^3^Emory University, Atlanta, GA, United States; ^4^Ohio State University, Columbus, OH, United States

####### **Correspondence:** Christine Chung (christine.chung@moffitt.org)

**Background**

Use of anti-Programmed Death-1 (anti-PD-1) inhibitors is a standard of care for patients (pts) with R/M HNSCC, but only limited numbers of pts achieve long term clinical benefits. Improving its efficacy and maintaining low toxicity profile are critical in combination strategies. We report the safety results of a phase I/II trial of CTX and NIVO in pts with R/M HNSCC.

**Methods**

Pts were treated with CTX 500 mg/m2 IV on Day (D) -14 as a lead-in followed by CTX 500 mg/m2 IV and NIVO 240 mg/m2 IV on D1 and D15 every 28-D cycle (C). Pts with CTX infusion reaction or who did not receive C1D1 for any reason were considered to be non-evaluable and were replaced. The toxicities with possible, probable, and definite attribution were included in treatment-related adverse events (TRAEs) and immune-related adverse events (IRAEs) analyses. NIVO dose reduction was not allowed.

**Results**

For the phase I cohort, 3 pts were enrolled. No dose limiting toxicities were observed during 4 weeks of observation period after C1D1, and no dose reduction was required. An additional 44 pts were enrolled, and 2 pts were non-evaluable. A total of 45 pts were analyzed. The median age was 64 (range 24-77), with 37 males and 8 females. The ECOG performance status at baseline was 0 (9 pts, 20%), 1 (33 pts, 73.3%), and 2 (3 pts, 6.7%). The primary sites were oral cavity 10 (22%), oropharynx 24 (53%), hypopharynx 3 (7%), larynx 6 (13%), and unknown primary 2 (4%). The p16 status was positive 22 (49%), negative 11 (24%), and unknown 12 (27%). The p16 status of the subsite, oropharynx, was positive 20 (83%) and negative 4 (17%). The smoking status was current 6 (13%); former 27 (60%) and never 12 (27%) with median pack years of 20 (range 0-185). Prior chemotherapy was given in 44 (98%). Prior radiotherapy was given in 36 (80%). The most common grade 3 TRAEs occurring >2% were fatigue 5 (11%) and rash-acneiform 2 (4.4%). The only grade 4 TRAE was CTX infusion reaction in 1 (2.2%). The most common grade 3 and 4 IRAEs occurring >2% were fatigue 2 (4.4%). No grade 5 TRAEs or IRAEs occurred. TRAEs led to CTX dose reduction in 4 (9%) of pts: infusion reaction, diarrhea, hypomagnesemia, fatigue (one each).

**Conclusions**

The combination of CTX and NIVO is well tolerated and remains to be an option for future studies.

**Ethics Approval**

The study was approved by Advarra CIRBI, approval number 00000971

#### P335 The tumor immune microenvironment and its association with pCR in NRG Oncology/NSABP B-52: Quantification of PD-1, PD-L1, CD8, FOXP3, and CD68 by multiplex fluorescent-immunohistochemistry

##### Marion Joy, PhD^1^, Ying Wang^1^, Rim Kim^1^, Nan Song^1^, Ashok Srinivasan^1^, Huichen Feng^1^, Corey Lipchik^1^, Reena Cecchini^2^, Samuel Jacobs^1^, Joseph Costantino^2^, Sandra Swain^3^, Eleftherios Mamounas^4^, Priya Rastogi^5^, Soonmyung Paik^6^, C. Kent Osborne^7^, Norman Wolmark^2^, Peter Lucas^8^, Mothaffar Rimawi^9^, Katherine Pogue-Geile^1^

###### ^1^NRG Oncology/NSABP, Pittsburgh, PA, United States; ^2^NRG Oncology/NSABP and the University of Pittsburgh, Pittsburgh, PA, United States; ^3^NRG Oncology/NSABP and Georgetown Lombardi Comprehensive Cancer Center, Georgetown University Medical Center, Washington, DC, United States; ^4^NRG Oncology/NSABP and Orlando Health, UF Health Cancer Center, Orlando, FL, United States; ^5^NRG Oncology/NSABP and the University of Pittsburgh Cancer Institute, Pittsburgh, PA, United States; ^6^NRG Oncology/NSABP and Yonsei University College of Medicine, Pittsburgh, PA, United States; ^7^NRG Oncology/NSABP and The Baylor College of Medicine/Dan L Duncan Comprehensive Cancer Center, Houston, TX, United States; ^8^NRG Oncology/NSABP and the University of Pittsburgh School of Medicine, Pittsburgh, PA, United States; ^9^NRG Oncology/NSABP The Baylor College of Medicine/Dan L Duncan Comprehensive Cancer Center, Houston, TX, United States

####### **Correspondence:** Katherine Pogue-Geile (katherine.pogue-geile@nsabp.org)

**Background**

The NRG Oncology/NSABP B-52 neoadjuvant clinical trial was conducted to test whether addition of estrogen deprivation (ED) would improve pCR rate in HER2+/ER+ breast cancer patients treated with docetaxel, carboplatin, trastuzumab, and pertuzumab (TCHP). A numerical increase in pCR rate was observed with ED (46.1% v 40.9%), but the difference was not statistically significant. B-52 provided the opportunity to explore potential predictive markers of pCR to possibly guide new treatment strategies. We examined the tumor immune microenvironment (TME) of B-52 baseline biopsy tumors to determine its association with pCR.

**Methods**

Pretreatment (N=238) biopsies were assessed for CD8, FOXP3, CD68, PD-L1, and PD-1, with multiplex fluorescent immunohistochemistry (mf-IHC) utilizing the Vectra® Quantitative Pathology Imaging System and inForm® Advanced Image Analysis software. Tumor and stromal regions were defined with a panCK antibody (included in the same multiplex) and annotated by a pathologist. Digital quantitation of all markers was assessed in the tumor and stromal regions (defined by panCK). In our pre-specified, CTEP-approved analysis, we tested the association of PD-L1 in tumor+stroma with a cut-point optimized by ROC curves (Table1). In exploratory analyses, we also tested a clinically meaningful PD-L1 cut-point of >1%; other markers were tested for associations with pCR using chi-square tests and a median cut-off (Table 1).

**Results**

Based on our pre-specified analysis, total PD-L1 (assessed in both stroma+tumor) was positively associated with pCR across trial arms (45% v 30%, p=0.038). PD-L1 was not significantly associated with pCR with a clinically utilized cut-off of >1% within the stromal-immune cell compartment (CD8+FOXP3+CD68). Surprisingly, in both stromal and tumor cell compartments, CD68 was positively associated with pCR when the two arms are evaluated together. When the treatment arms are examined separately, CD68 in the stromal compartment correlates positively with pCR. FOXP3 was also positively correlated with pCR in the stromal cell compartment across arms and in the TCHP+ED arm. PD-1 and CD8 were not significantly associated with pCR.

**Conclusions**

B-52 showed a positive association of PD-L1 expression with pCR based on our pre-specified analysis but the extremely low cut-off (0.05%) makes the clinical utility of this observation doubtful. Surprisingly, FOXP3+, and CD68+ staining cells were positively associated with pCR. Additional analyses currently being conducted to assess during treatment biopsies and the spatial relationships between different immune markers may provide further mechanistic insights.

**Acknowledgements**

U10CA180868; U24CA196067; UG1CA189867; Genentech, BCRF

**Trial Registration**

NCT02003209

**Ethics Approval**

Chesapeake IRB: Samples are exempt based on the Determination for NSABP Foundation, Inc. Protocol TB-2 “NSABP TB-2: Comprehensive Survey of Prognostic and Predictive Markers for Breast and Colon Cancer” (Pro00005069). All patients provided written informed consent to the NSABP B-52 Clinical Study, which was reviewed and approved by the NCI CIRB. No personal identifiable information is included.

**Table 1 (abstract P335).**
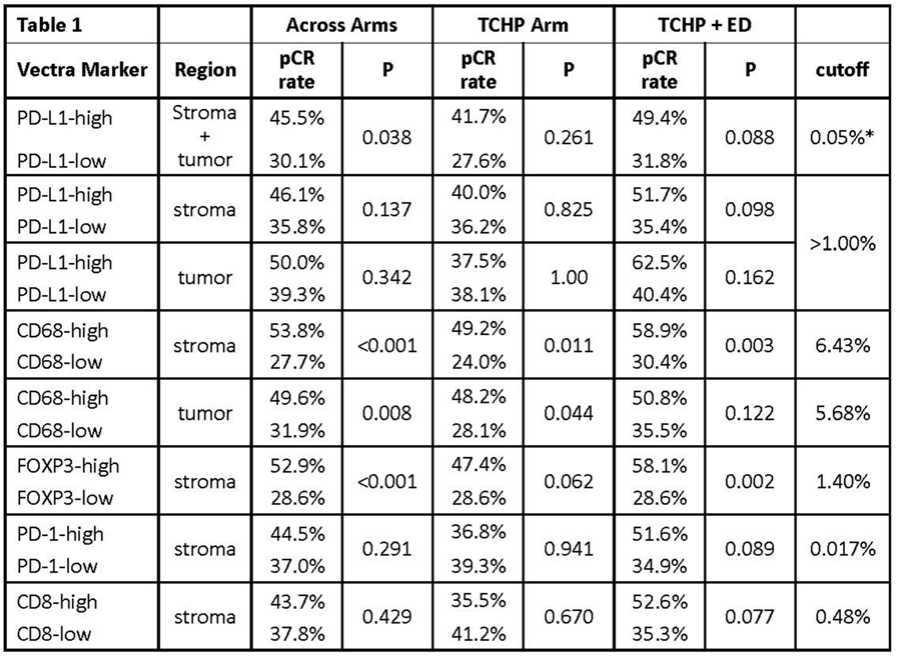
See text for description

#### P336 Impact of cytokine release syndrome on cardiac function following CD19 CAR-T cell therapy in children and young adults with acute lymphoblastic leukemia

##### Amita Kulshrestha^1^, Haneen Shalabi, Do^1^, Vandana Sachdev^2^, Douglas Rosing^2^, Stanislav Sidenko^2^, Crystal Mackall, MD^3^, Brandon Wiley^4^, Daniel Lee^5^, Nirali Shah^6^

###### ^1^National Institutes of Health, Bethesda, MD, United States; ^2^National Heart, Lung and Blood Institute, Bethesda, MD, United States; ^3^Stanford, Stanford, CA, United States; ^4^Mayo Clinic, Rochester, MN, United States; ^5^University of Virginia, Charlottesville, VA, United States; ^6^National Cancer Institute, Kensington, MD, United States

####### **Correspondence:** Nirali Shah (nirali.shah@nih.gov)

**Background**

Cytokine release syndrome (CRS) is the main toxicity of CAR-T cell therapy, which may require hemodynamic support. The impact of CRS on cardiac function has not been well described.

**Methods**

We report on cardiac toxicity seen in children and young adults with ALL treated on our phase I trial of CD19 CAR-T cell therapy (clinicaltrials.gov NCT01593696). All patients had a baseline echocardiogram. Cumulative anthracycline exposure was calculated from prior exposure. Additional studies included increased frequency of echocardiograms upon ICU transfer, and serial troponin and proBNP.

**Results**

From July 2012 to March 2016, 52 patients, with a median age of 13.4 years (range, 4.2-30.3) were treated on-study; 23 underwent at least one prior allogeneic stem cell transplantation. CRS was seen in 37/52 (71%), which was grade 3-4 CRS in 8 subjects (21.6%). The median prior anthracycline exposure was 205 mg/m2 (range, 70-620 mg/m2) in doxorubicin equivalents. The median baseline LV ejection fraction (LVEF) was 62% (range 52%-71%). The median LV global longitudinal strain (GLS), at baseline was abnormal: -17 (range, -14 to -24, n=35). ICU transfers occurred in 20 patients, 11 of whom required vasoactive hemodynamic support, with 5 necessitating more than 1 pressor. Seven patients received tocilizumab and 4 patients received steroids. Six (16%) patients developed cardiac dysfunction, amongst whom 4 had grades 3-4 CRS. (Figure 1) Severe cardiac dysfunction, (LVEF < 30%) was seen in 3, with one patient developing cardiac arrest with subsequent full recovery following placement of an intra-aortic balloon pump, steroids, and tocilizumab. In 2 of these patients, anthracycline exposures exceeded > 360 mg/m2. All but 2 patients had full resolution of cardiac dysfunction by day 28 post CAR. Troponin elevations were seen in 4 of 6 patients with low LVEF. In a limited cohort of patients with pre/post pro-BNP, pro-BNP was higher during CRS, with the highest levels correlating with more severe cardiac dysfunction. (Figure 2)

**Conclusions**

Patients with higher-grade CRS are more likely to experience significant cardiac side effects from CAR T-cell therapy. In most cases, resolution to near baseline was seen coinciding with resolution of CRS, with most having near complete resolution by day 28 post infusion. Implementation of more frequent echocardiogram monitoring and incorporation of BNP and troponin into daily laboratory panel may help to identify those at highest risk of severe cardiac dysfunction at an earlier time point, allowing for earlier intervention in CRS to potentially limit acute cardiac toxicity.

**Acknowledgements**

This research was supported by the Intramural Research Programs of the Center of Cancer Research, National Cancer Institute, NIH and the Clinical Center.

**Trial Registration**

The clinical trial is registered at clinicaltrials.gov NCT01593696

**Ethics Approval**

This study was approved by the National Cancer Institute Institutional Review Board.

**Fig. 1 (abstract P336).**
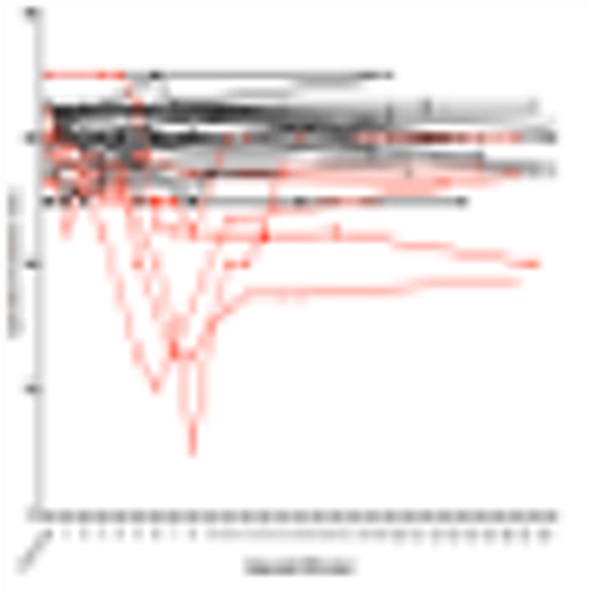
Change in Ejection Fraction Post CRS Onset

**Fig. 2 (abstract P336).**
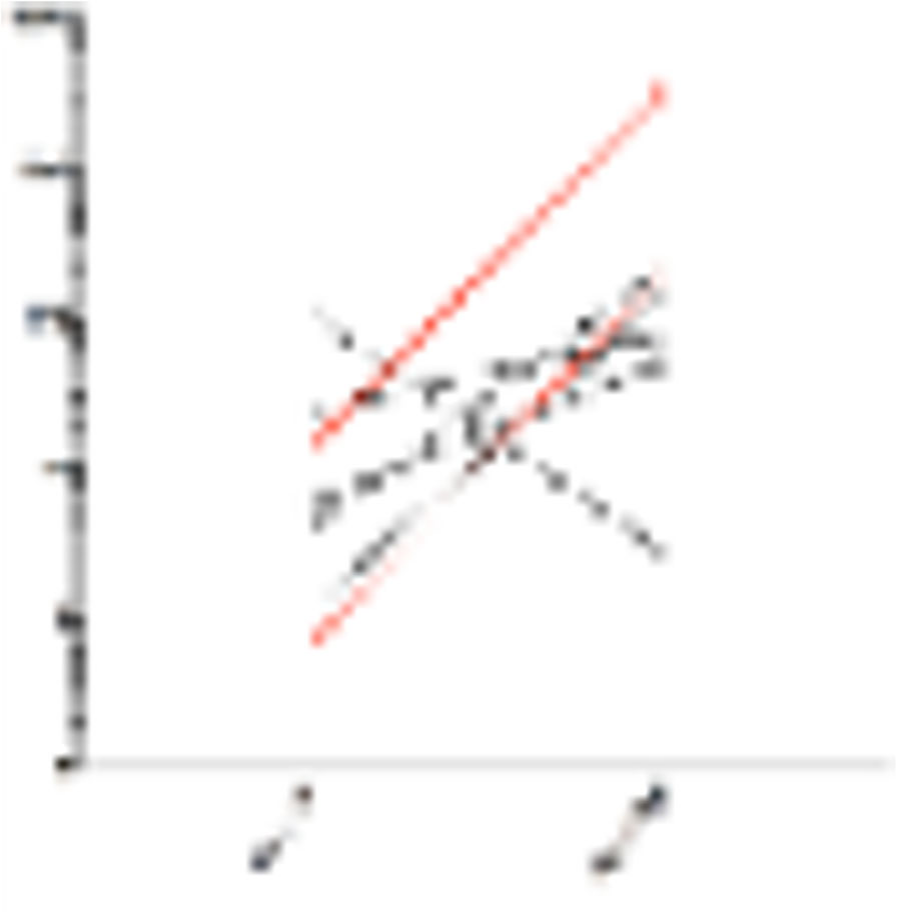
Changes in BNP pre and post-CAR infusion

#### P337 Survival prolongation by dendritic cell vaccination in combination with OK-432, gemcitabine and/or S-1 in patients with advanced pancreatic cancer

##### Masahiro Ogasawara, MD, PhD, Shuichi Ota, MD PhD

###### Sapporo Hokuyu Hospital, Sapporo, Japan

####### **Correspondence:** Masahiro Ogasawara (ogasawara@hokuyu-aoth.org)

**Background**

Pancreatic cancer is the most fatal human cancer, with a 5-year overall survival rate of less than 5%. In the current study, we have evaluated the clinical and the immunological responses in patients with advanced pancreatic cancer who received dendritic cell (DC) vaccination in combination with a toll like receptor (TLR) 4 agonist, OK-432 and chemotherapeutic agents, gemcitabine (GEM) and/or S-1.

**Methods**

Twenty three patients (13 males, 10 females; aged 37-83 years, median 64 year old) with advanced pancreatic cancer refractory to standard treatment were treated with DC vaccination in combination with OK-432, GEM and/or S-1 from 2012 to 2013 at Sapporo Hokuyu Hospital. Autologous DCs were generated by culturing adherent mononuclear cells with interleukin-4 and granulocyte-macrophage colony stimulating factor. DCs were then loaded with synthetic peptides derived from cancer antigens such as Wilms’ tumor 1 (WT1) and MUC1 following maturation by prostaglandin E2 and OK-432. Peptide-loaded mature DCs and OK-432 were administered intradermally every 2 weeks, 7 times. The induction of vaccine-induced T cell responses was monitored by using HLA-tetramer and ELISPOT assays.

**Results**

The treatment was well tolerated and none of the patients experienced more than grade 3 adverse events during the treatment period. Of 23 patients, 1 had partial response (PR), 8 had stable disease (SD) and 14 had progressive disease after one course of vaccination. The median overall survival (OS) was 9.6 months. Survival of patients achieving PR or SD (responders) was longer than those who did not respond to the treatment (non-responders) (median OS; 18.0 vs 5.8 months). An HLA-tetramer assay showed an increase in the positivity of WT1-specific CD8+ T cells in both responders and non-responders after vaccination. However, the increment in the positivity was remarkable in responders in comparison with non-responders; 46.3 and 10.7 fold in responders and non-responders, respectively. Similarly, an ELISPOT assay showed marked increase in spot-positive cells in responders. The median OS in patients showing the positivity in both assays was longer than those who were positive in either assay or who were negative in both assays; a median OS was 18.4 months, 9.7 months and 4.7 months, respectively, suggesting a correlation between an immune response and a clinical outcome.

**Conclusions**

DC vaccination combined with a conventional chemotherapy in patients with advanced pancreatic cancer was demonstrated to be safe and can elicit immune responses against tumor antigens, which was correlated with clinical effects.

**Ethics Approval**

This study was approved by the Ethics and Internal Review Board at Sapporo Hokuyu Hospital, approval number 131111.08

#### P338 2-year follow-up from JAVELIN Lung 200, an open-label, randomized, phase 3 study of avelumab vs docetaxel in patients with platinum-treated advanced non-small cell lung cancer (NSCLC)

##### Fabrice Barlesi, MD, PhD^1^, Mustafa Özgüroğlu^2^, Johan Vansteenkiste^3^, David Spigel^4^, James Yang^5^, Hidenobu Ishii^6^, Marina Garassino^7^, Filippo de Marinis^8^, Aleksandra Szczesna^9^, Andreas Polychronis^10^, Ruchan Uslu^11^, Maciej Krzakowski^12^, Jong-Seok Lee^13^, Luana Calabro, MD^14^, Osvaldo Aren Frontera^15^, Barbara Ellers-Lenz^16^, Marcis Bajars^17^, Mary Ruisi^17^, Keunchil Park^18^

###### ^1^Aix-Marseille University, Assistance Publique - Hôpitaux de Marseille, Livon, France; ^2^Cerrahpaşa Medical Faculty, Istanbul University, Leuven, Belgium; ^3^University Hospital KU Leuven, Leuven, Belgium; ^4^Sarah Cannon Research Institute, Nashville, TN, United States; ^5^National Taiwan University Hospital, Taipei, Taiwan, Province of China; ^6^Kurume University School of Medicine, Kurume, Japan; ^7^Fondazione IRCCS Istituto Nazionale dei Tumori, Milan, Italy; ^8^Istituto Europeo di Oncologia, Milan, Italy; ^9^Regional Lung Disease Hospital, Otwock, Poland; ^10^Mount Vernon Cancer Centre, Northwood, Middlesex, United Kingdom; ^11^Ege University Hospital, Izmir, Turkey; ^12^Centrum Onkologii-Instytut Im. M. Skłodowskiej-Curie w Warszawie, Warszawa, Poland; ^13^Seoul National University Bundang Hospital, Seoul National University College of Medicine, Seongnam, Korea, Republic of; ^14^University Hospital of Siena, Siena, Italy; ^15^Instituto Nacional del Cáncer, Santiago, Chile; ^16^Merck KGaA, Darmstadt, Germany; ^17^EMD Serono Inc, Billerica, MA, United States; ^18^Samsung Medical Center, Sungkyunkwan University School of Medicine, Seoul, Korea

####### **Correspondence:** Fabrice Barlesi (Fabrice.BARLESI@ap-hm.fr)

**Background**

Avelumab, a human IgG1 anti–PD-L1 monoclonal antibody, is approved as monotherapy for metastatic Merkel cell carcinoma and platinum-treated urothelial carcinoma in various countries, and in combination with axitinib to treat advanced renal cell carcinoma in the United States. In the JAVELIN Lung 200 study, avelumab did not significantly prolong overall survival (OS) vs docetaxel in patients with platinum-treated PD-L1+ NSCLC (primary objective); however, prespecified exploratory analyses showed longer OS with avelumab vs docetaxel in patients with higher PD-L1+ tumors. We report updated data from JAVELIN Lung 200.

**Methods**

Patients with stage IIIB/IV or recurrent NSCLC and disease progression following platinum-doublet chemotherapy were randomized 1:1 to avelumab 10 mg/kg Q2W or docetaxel 75 mg/m2 Q3W. The primary endpoint was OS; the primary analysis population was patients with PD-L1+ tumors (≥1% tumor cell expression; PD-L1 IHC 73-10 pharmDx assay).

**Results**

792 patients were enrolled (intention-to-treat [ITT] population), including 529 with PD-L1+ tumors (primary analysis population) and 263 with PD-L1− tumors. (n=263). At data cut-off (March 4, 2019) in the PD-L1+ population, median duration of follow-up for OS was 35.4 months in the avelumab arm (n=264) and 34.7 months in the docetaxel arm (n=265); study treatment was ongoing in 25 (9.5%) vs 0 patients, and 17 (6.4%) vs 74 (27.9%) had received a posttreatment checkpoint inhibitor (CPI), respectively. 2-year OS rates with avelumab vs docetaxel in different PD-L1+ subgroups are shown (Table 1). Of patients with PD-L1+ tumors alive at 2 years, 67% had received a posttreatment CPI in the docetaxel arm compared with 13% in the avelumab arm. In patients with PD-L1+ tumors who had an objective response with avelumab (50 [18.9%]) or docetaxel (28 [10.6%]), median duration of response (DOR; investigator assessed) was 19.1 months (95% CI: 10.8-34.8) vs 5.7 months (95% CI: 4.1-8.3), and proportions with a response lasting ≥6 months were 86.0% (95% CI: 72.9%-93.1%) vs 48.1% (95% CI: 28.7%-65.2%), respectively. Safety profiles of avelumab and docetaxel were similar to those in previous analyses.

**Conclusions**

Updated data from JAVELIN Lung 200 showed that although avelumab did not significantly prolong OS vs docetaxel in the primary confirmatory analysis, 2-year OS rates were doubled with avelumab vs docetaxel in higher PD-L1+ subgroups, and median DOR was >12 months longer with avelumab vs docetaxel.

**Acknowledgements**

This study was funded by Merck KGaA, Darmstadt, Germany, as part of an alliance between Merck KGaA, Darmstadt, Germany and Pfizer Inc., New York, NY, USA.

**Trial Registration**

NCT02395172

**Ethics Approval**

The study protocol was approved by institutional review boards and ethics committees at each institution. The study was done in accordance with the trial protocol, Good Clinical Practice guidelines, and the Declaration of Helsinki. All patients provided written informed consent.

**Table 1 (abstract P338).**
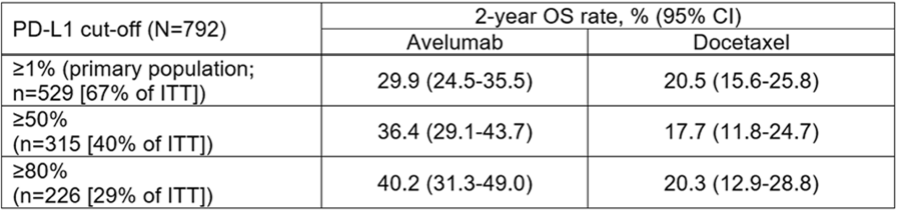
See text for description

#### P339 Survival is improved by antigen-specific cytotoxic T lymphocytes (CTL) responses after treatment with the vaccine Tedopi in HLA-A2 positive advanced non-small cell lung cancer (NSCLC) patients

##### Benjamin Besse, MD PhD^1^, Enriqueta Felip, MD PhD^2^, Giuseppe Giaccone^3^, Rafal Dziadziuszko^4^, Elisabeth Quoix, MD^5^, Werner Hilgers^6^, Federico Cappuzzo^7^, Christophe Borg^8^, Jordi Remon^9^, Nicolas Poirier^10^, Dominique Costantini^10^, Bérangère Vasseur^10^, Santiago Viteri^11^

###### ^1^Gustave Roussy, Villejuif, France; ^2^Vall d'Hebron University Hospital, Barcelona, Spain; ^3^Georgetown University, Washington, DC, WA, United States; ^4^Medical University of Gdańsk, Gdańsk, Poland; ^5^Nouvel Hôpital Civil, Strasbourg, France; ^6^Institut Sainte Catherine, Avignon, France; ^7^AUSL Romagna, Ravenna, Italy; ^8^Centre Hospitalier Universitaire, Besançon, France; ^9^CIOCC- Barcelona, Barcelona, Spain; ^10^OSE Immunotherapeutics, Nantes, France; ^11^University Hospital Dexeus, Barcelona, Spain

####### **Correspondence:** Benjamin Besse (benjamin.besse@gustaveroussy.fr)

**Background**

Tedopi (OSE2101) is a multiple epitope vaccine restricted to HLA-A2 positive patients (45%), targeting five tumor-associated antigens (TAA) frequently expressed in solid tumors: carcinoembryonic antigen (CEA), human epidermal growth factor receptor 2 (HER-2/neu), melanoma-associated antigen type 2 and 3 (MAGE2 and MAGE3), and p53. Tedopi is composed by 2 wild type and 7 chemically modified peptides to increase HLA-A2 or T cell receptor (TCR) affinity. A pan-DR epitope (PADRE) of helper T-lymphocyte (HTL) has been added to increase the Cytotoxic T Lymphocyte (CTL) responses. In previously treated advanced NSCLC patients, Tedopi showed a strong CTL immune response, which correlated with overall survival (OS) [1]. The aim of the current translational study was to explore the predictive effect of the epitope type and number of epitopes on OS.

**Methods**

Out of 64 previously treated HLA2+ advanced NSCLC patients enrolled in a phase II trial testing the efficacy of Tedopi (1mL subcutaneously Q3W for 6 cycles, then Q8W for the reminder year 1 and Q12W up to year 2), 33 patients were assessed for epitope-specific cytotoxic response and HTL responses using an interferon gamma enzyme-linked immunosorbent assay. Leukapheresis was performed at baseline, at week 9, 18 and 30 for immunogenicity assays. Predictive analyses of OS were performed using Cox regression.

**Results**

Patients were stage IV (64%), or locally advanced stage IIIb (36%). Eleven patients were assessed for all 10 epitopes, and 33 for 6 selected epitopes (2 CEA, 1 HER-2, MAGE2, MAGE3, PADRE). Median survival was 30 months.

There was at least one CTL response to one vaccine epitope in >90% of patients. Eight epitopes were highly immunogenic (from 55% to 91%), while HER-2 wild type and one p53 analogue shown a lower response (respectively 36% and 9%).

In patients evaluated for 6 selected epitopes, the best cut-off of number of CTL responses to discriminate OS were 1-6 versus 0, 2-6 vs 0-1 or 3-6 vs 0-2. All of three were statistically significant. As an example, patients with CTL responses to 3-6 epitopes (n=23) had a median OS of 38 months compared to 15 months in patients (n=10) with CTL responses to 0-2 epitopes (HR=0.39; p=0.04) (Figure 1). CTL response to HER-2 analogue, MAGE3, PADRE and one p53 analogue were predictive of better OS.

**Conclusions**

In NSCLC patients, survival was significantly prolonged in patients immunized to epitope specific Tedopi vaccine. HER-2, MAGE3, PADRE and p53 were identified as vaccine predictive epitopes for prolonged survival.

**Acknowledgements**

We thank François Montestruc and Constant Josse (eXYSTAT, Malakoff, France) for the statistical analysis

**Reference**

1. Barve M, Bender J, Senzer N, Cunningham C, Greco FA, McCune D, Steis R, Khong H, Richards D, Stephenson J, Ganesa P, Nemunaitis J, Ishioka G, Pappen B, Nemunaitis M, Morse M, Mills B, Maples PB, Sherman J and Nemunaitis JJ. Induction of Immune Responses and Clinical Efficacy in a Phase II Trial of IDM-2101, a 10-Epitope Cytotoxic T-Lymphocyte Vaccine, in Metastatic Non-Small-Cell Lung Cancer. J Clin Oncol. 2008;26(27):4418–25.

**Ethics Approval**

The study protocol and its related documents (including the patient information and informed consent form) received approval from the Institutional Review Board (IRB), and the Competent Authority prior to study initiation.

**Consent**

Each patient gave his/her written informed consent prior to study enrolment.

**Fig. 1 (abstract P339).**
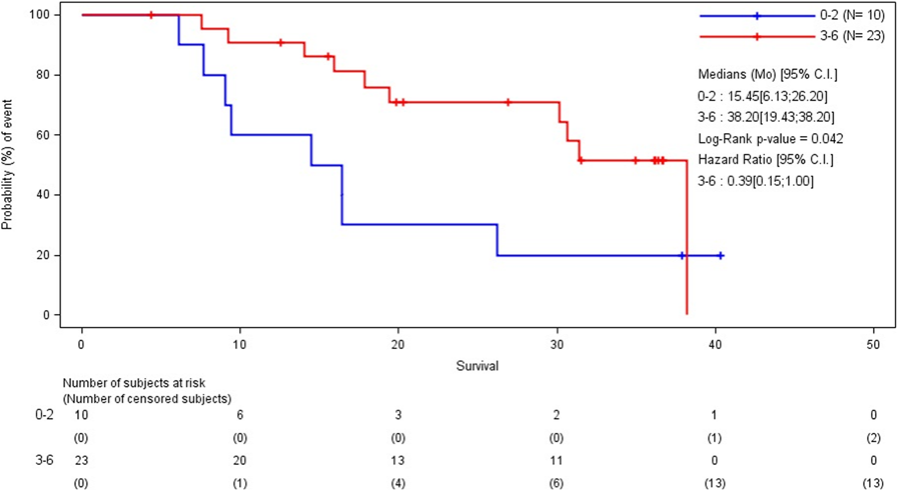
Overall survival in patients with 3-6 vs 0-2 CTL responses

#### P340 Region-focused deep survival learning on PD-L1 stained tissue samples for data-driven stratification of durvalumab-treated NSCLC patients

##### Nicolas Brieu, PhD^1^, Ansh Kapil^1^, Armin Meier, PhD^1^, Keith Steele, DVM, PhD^2^, Marlon Rebelatto, DVM, PhD, DACVP^2^, Guenter Schmidt, PhD^1^

###### ^1^Definiens AG, Munich, Germany; ^2^AstraZeneca, Gaithersburg, MD, United States

####### **Correspondence:** Nicolas Brieu (nbrieu@definiens.com)

**Background**

The selection of metastatic non-small cell lung cancer (NSCLC) patients that are likely to respond to an anti-PD-L1 checkpoint monotherapy can be guided by the visual assessment by pathologists of the Tumor Cell (TC) score on PD-L1 stained tissue samples [1]. Deep learning approaches have recently enabled the computer-based replication of this visual TC score [2,3] and of its ability to predict overall survival (OS) [4]. Because these methods try to reproduce as close as possible the visual scoring methodology, they are built on extensive prior hypotheses (e.g. definition of cell positivity, score and cut-off) and do not enable the data-driven discovery of novel stratification rules. We present here a novel region-focused end-to-end deep-learning approach that enables the data-driven generation of survival risk heatmaps and the stratification of patients into two risk groups.

**Methods**

On a subset (N=151) of core needle biopsies and tissue resections from the NCT01693562 clinical trial (NSCLC), epithelium regions are automatically segmented within the manually delineated tumor area [3]. A patch-based convolutional neural network (CNN) is trained on selected patches in a two-fold pre-validation procedure to maximize a log partial likelihood derived from the Cox proportional hazards model [5,6]. To avoid a disproportionately large number of patches from tissue resections, a random subset of up to 10K patches is selected for each patient within the segmented regions. The overall survival risk is predicted and aggregated by mean on the detected epithelium regions only. Patients are finally stratified based on the cohort median of the resulting aggregated risk scores. For baseline comparison, the same steps are repeated considering the complete delineated tumor area instead of the sole segmented epithelium regions.

**Results**

The proposed epithelium-focused and data-driven survival CNN yields similar patient stratification (HR=0.525, p=0.003) as obtained with 25% cut-off on visual (HR=0.574, p=0.01) or automated (HR =0.539, p=0.004) TC score (Figure 1), while releasing prior hypotheses on PD-L1 region positivity, score methodology, and cut-off value. As expected on durvalumab-treated patients, high and low risks are associated with low and high PD-L1 staining respectively. No relevant risk groups are identified if the analysis is performed on the full tumor area instead.

**Conclusions**

Our results suggest, for the first time on core needle biopsies and tissue resections, (i) the ability of end-to-end deep survival learning to directly learn relevant patient stratification as well as (ii) the necessity, in case of small patient cohorts, to restrict the analysis to automatically detected meaningful regions.

**References**

1. Rebelatto et al., Development of a programmed cell death ligand-1 immunohistochemical assay validated for analysis of non-small cell lung cancer and head and neck squamous cell carcinoma, Diagnostic Pathology 2016

2. A. Kapil et al., Deep Semi Supervised Generative Learning for Automated Tumor Proportion Scoring on NSCLC Tissue Needle Biopsies, Scientific reports, 2018

3. A. Kapil et al., DASGAN - Joint Domain Adaptation and Segmentation for the Analysis of Epithelial Regions in Histopathology PD-L1 Images, arXiv preprint arXiv:1906.11118, 2019

4. N. Brieu et al., Deep learning-based PD-L1 tumor cell (TC) scoring improves survival prediction compared to pathologists on durvalumab-treated NSCLC patients, SITC 2018.

5. P. Mobadersany et al., Predicting cancer outcomes from histology and genomics using convolutional networks, PNAS 2018.

6. A. Meier et al., End-to-end learning to predict survival in patients with gastric cancer using convolutional neural networks, Annals of Oncology (ESMO), 2018.

**Ethics Approval**

For the Phase 1/2 durvalumab trial (NCT01693562), the study protocol was reviewed and approved by the Institutional Review Board of the participating centers and informed consent was obtained from all patients.

**Fig. 1 (abstract P340).**
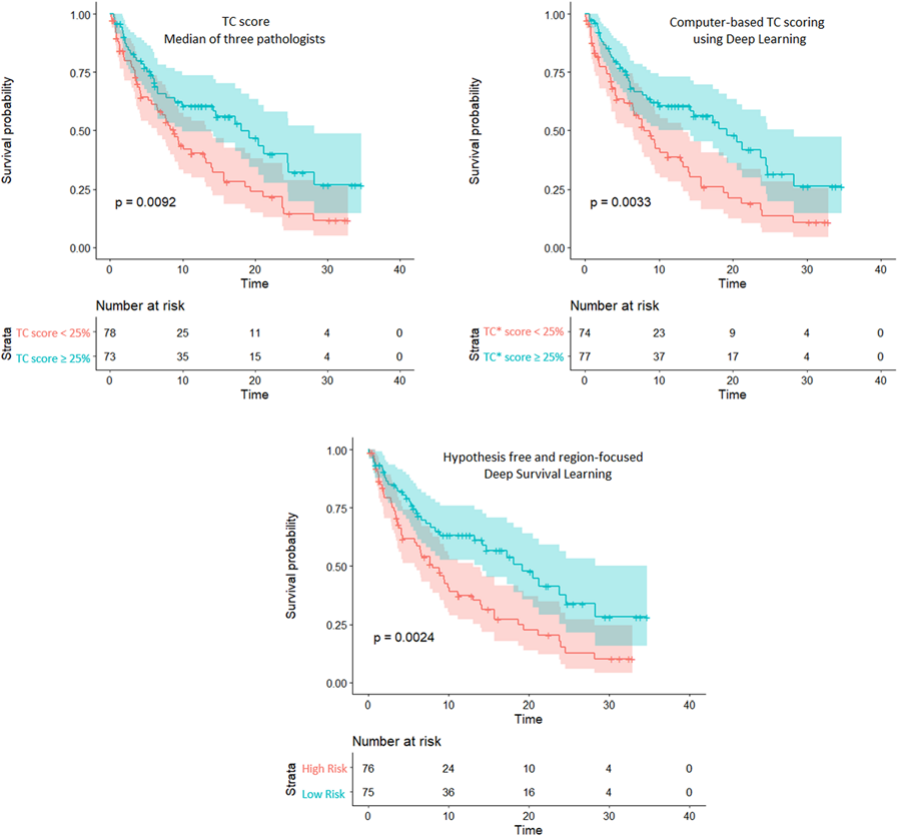
See text for description

#### P341 Activation of toll-like receptors via PS-targeting monoclonal antibodies

##### Rolf Brekken, PhD (rolf.brekken@utsouthwestern.edu)

###### UT Southwestern Medical Center, Dallas, TX, United States

**Background**

Multifocal immune suppression in the tumor microenvironment is a major underlying cause for the limited efficacy of immune checkpoint blockade. Persistent immune suppression prevents the development of a robust T cell response to tumor specific antigens that is required for effective downstream immune checkpoint blockade. An underappreciated but significant contributor to immune suppression in tumors is the exposure of the membrane phospholipid phosphatidylserine (PS) on the surface of tumor cells and tumor-derived microvesicles. PS is recognized by receptors on immune cells where it triggers the secretion of immune suppressive cytokines, prevents the differentiation of myeloid-derived suppressor cells (MDSCs) and inhibits dendritic cell (DC) maturation; events that prevent a productive anti-tumor T cell response. Bavituximab, a chimeric monoclonal antibody (mAb) that targets PS and inhibits PS-mediated immunosuppressive signaling, drives immune activation by reducing the levels of MDSCs, by polarizing tumor-associated macrophages towards an immune stimulatory phenotype and by promoting the maturation of dendritic cells (DCs). Bavituximab and other PS-targeting mAbs (2aG4 and 1N11) bind to PS via beta-2 glycoprotein 1 (β2GP1). β2GP1, an abundant serum glycoprotein, was recently identified as a novel component of innate immunity via activation of Toll-like receptors (TLRs).

**Methods**

Human β2GP1. Monoclonal-antibodies: Bavituximab and 1N11. qPCR, WB, IP, ICC/IHC, CD, TEM, MST and ELISA

**Results**

We investigated whether the innate immune changes induced by PS-targeting mAbs is mediated in part by β2GP1-induced activation of TLRs in myeloid cells. β2GP1 has a closed conformation that prevents its interaction with PS and cell surface receptors. However, interaction of β2GP1 with LPS can induce an open conformation of the protein that allows interaction of β2GP1 with PS and cell surface receptors, including the TLRs. We found through circular dichroism and transmission electron microscopy that the PS-targeting antibodies also induce conformational changes in β2GP1, shifting it to an open conformation. In addition, we demonstrate that PS-targeting mAb-mediated dimerization of β2GP1 stimulated pro-inflammatory polarization of bone marrow-derived macrophages (BMDMs) and induced TLR2 signaling. Finally, we investigated the expression of a newly characterized TLR-induced transcription factor, Spi-C in bone marrow progenitor cells after stimulation with TLR specific agonists or PS-targeting mAbs +/- β2GP1.

**Conclusions**

These studies demonstrate that the PS-targeting mAbs stimulate Spi-C expression in a β2GP1-dependent manner. Overall these data support that one mechanism of innate immune activation induced by PS-targeting mAbs is through TLR2 stimulation on myeloid cells. Future studies are focused on validating these results in vivo using Tlr-deficient and β2gp1-deficient mice.

**Trial Registration**

NCT01999673, NCT03139916, NCT03519997

**Ethics Approval**

All animal experiments were done according to the Animal Research Center (ARC) at UT Southwestern Medical Center. All clinical trials were conducted in accordance with all Federal and State Laws.

#### P342 Long term outcomes of a phase I study with UV1, a second generation telomerase based vaccine, in patients with advanced non-small cell lung cancer

##### Wenche Rasch, PhD^1^, Paal Brunsvig, MD PhD^2^, Martha Nyakas, MD^2^, Clau Reisse, MD^2^, Jon Amund Kyte^2^, Hedvig Vidarsdotter Juul^2^, Steinar Aamdal^1^, Gustav Gaudernack, PhD^3^, Else Marit Inderberg^2^

###### ^1^Ultimovacs ASA, Oslo, Norway; ^2^Oslo University Hospital, Oslo, Norway; ^3^Ultimovacs ASA, Prof. Emeritus, Oslo University Hospital, Oslo, Norway

####### **Correspondence:** Wenche Rasch (wenche.rasch@ultimovacs.com)

**Background**

A first generation hTERT vaccine (GV1001) showed evidence of clinical efficacy in patients with advanced non-small cell lung cancer (NSCLC). We have now tested a second generation hTERT vaccine, UV1. This vaccine is designed to give high population coverage and is composed of three synthetic long peptides containing multiple epitopes identified by epitope spreading data from long-term survivors who participated in previous hTERT vaccination trials.

**Methods**

Eighteen non-HLA-typed patients with stage III/IV NSCLC with no evidence of progression after prior treatments, were enrolled in a phase I dose-escalation study of UV1 vaccination with GM-CSF as adjuvant, evaluating safety, immune response, and long term clinical outcome. The present study also aimed to provide a rationale for combining UV1 vaccine with PD-1/PD-L1 blockade.

**Results**

Treatment with GM-CSF and UV1 was well tolerated with no serious adverse events observed. All patients experienced one or more adverse events, the majority grade 1, such as injection site reactions and fatigue. Seventeen patients were evaluable for tumor response; 15 patients had stable disease as best response, while 2 patients had progressive disease. The median progression free survival (PFS) was 12.3 months and the median overall survival (OS) was 28.2 months. The OS at 3 years was 44%. None of the 7 long-term surviving patients (median survival 4.96 years, range 4.04-5.51) have received checkpoint blockade therapy after UV1 vaccination. UV1-vaccination induced specific T helper 1 (Th1) immune responses in the majority (67%) of patients. Both immune responses and OS were dose related.

**Conclusions**

The highest dose of UV1 (700 μg) resulted in the highest proportion of immune responses. These responses occurred more rapidly and were stronger compared to lower doses and the patients in this group had a 3-year OS of 83%. This, together with the safety and clinical outcome data, favours 700 μg as the preferred UV1 dose in this patient population. These results provide a rationale for further clinical studies in NSCLC with UV1 vaccination in combination with immune checkpoint blockade.

**Acknowledgements**

We thank all the patients for their participation in the study

**Trial Registration**

The clinical trial UV1/hTERT-L was performed with NoMA approval and is registered with Clinicaltrials.gov on February 11, 2013 (NCT01789099). Patients provided written informed consent to participate. Enrollment started on April 8, 2013.

**Ethics Approval**

The study was approved by the institutional protocol board, the regional Ethical Committee (REC 2012/1114, EudraCT 2012- 001852-20) and the Norwegian Medicines Agency (NOMA) and the study was registered at clinicaltrials.gov (NCT01789099).

**Consent**

Individual patient consent was not applicable as no information in this abstract/poster can be categorized as identifiable.

#### P343 Initial results from a Phase II study (TACTI-002) in non-small cell lung cancer, or head and neck cancer patients receiving eftilagimod alpha (LAG-3 fusion protein) and pembrolizumab

##### Julio Peguero, MD^1^, Enriqueta Felip, MD PhD^2^, Bernard Doger^3^, Margarita Majem^4^, Enric Carcereny^5^, Tim Clay^6^, Pawan Bajaj^7^, Matthew Krebs, MD PhD^8^, Frederic Triebel, MD, PhD^9^

###### ^1^Oncology Consultants, Houston, TX, United States; ^2^Vall d’ Hebron Institute of Oncology, Barcelona, Spain; ^3^Fundación Jimenez Díaz, Madrid, Spain; ^4^Hospital de la Santa Creu i Sant Pau, Barcelona, Spain; ^5^Institut Català d'Oncologia Badalona, Barcelona, Spain; ^6^St John of God Subiaco Hospital, Perth, Australia; ^7^Tasman Health Care, Queensland, Australia; ^8^The University of Manchester and The Christie NHS Foundation Trust, Manchester, United Kingdom; ^9^Immutep, Orsay, France

####### **Correspondence:** Frederic Triebel (ftriebel@immutep.com)

**Background**

Eftilagimod alpha (efti; previously IMP321) is a recombinant LAG-3 Ig fusion protein that binds to MHC class II molecules to mediate antigen presenting cell (APC) and CD8 T-cell activation. The stimulation of the dendritic cell network and subsequent T cell recruitment at the tumor site with efti may lead to stronger anti-tumor CD8 T cell responses than observed with pembrolizumab alone. Combining an APC activator with an immune checkpoint inhibitor (ICI) aims to increase efficacy without additional toxicity. We hereby report initial results of stage 1 of this phase II trial (NCT03625323).

**Methods**

The study is based on a Simon's optimal two-stage design, with objective response rate (ORR) as primary endpoint. Secondary endpoints include progression free survival and overall survival. Blood for PK/PD assessments and anti-drug antibody evaluation is collected. During the first stage of the study, patients (pts) are recruited into each of three indications: A: 1st line, PD-X naïve NSCLC; B: 2nd line, PD-X refractory NSCLC; C: 2nd line PD-X naïve HNSCC. Additional patients (N2) will be recruited for each part if the pre-specified threshold for ORR is met. In total 109 patients are planned to be enrolled. Eftilagimod alpha is administered as 30 mg subcutaneous injection every 2 weeks for the first 8 cycles and every 3 weeks for the 9 following cycles. Pembrolizumab is administered at a standard dose of 200 mg intravenous infusion every 3 weeks for maximum 2 years. The study was approved by all relevant ethics committees and institutional review boards.

**Results**

Between 05 March and 24 July 2019, 27 pts were enrolled and treated in the study. The mean age was 67 (range 53-84) and 74% were male. The ECOG PS was 0 in 59% of the pts and 1 in 41% of the pts. The treatment has been well tolerated with the most common AEs being cough (9%), dyspnea (9%), diarrhea (6%) and asthenia (5%). Eleven treatment related SAEs were reported in ten pts. Thirteen (13) pts of part A are evaluable (data cut-off 24th July 2019) for efficacy. The vast majority had only one post-baseline tumor assessment. Four pts of 13 (31%) had a partial response and six (46%) pts had stable disease according to iRECIST at data cut-off.

**Conclusions**

Thirty (30) mg efti s.c. every 2 weeks in combination with standard dose of pembrolizumab is safe and shows encouraging antitumor activity.

**Trial Registration**

EudraCT: 2018-001994-25

NCT: 03625323

**Ethics Approval**

The study was approved by Advarra IRB (US), approval number N/A, approval date: 13/08/2018; London - Harrow Research Ethics Committee (UK), approval number 18/LO/1889; Instituto de Investigación Hospital 12 de Octubre (Spain), approval number 18/376; Belberry HREC (Australia), approval number 2018-08-636; St John of God Health Care (Australia), approval number 1450.

#### P344 A randomized multi-center phase 2 study of combined PD-L1/CTLA-4 inhibition with or without radiation in non-small cell lung cancer patients who progressed on PD-(L)1 directed therapy: ETCTN 10021

##### Arta Monjazeb, MD, PhD^1^, Anita Giobbie-Hurder, MS^2^, Ana Lako^3^, Mark Awad, MD PhD^3^, Ryan Gentzler, MD^4^, Carrie Lee^5^, Joleen Hubbard^6^, James Abbruzzese, MD^7^, Salma Jabbour, MD^8^, Nataliya Uboha^9^, Kevin Stephans^10^, Jennifer Johnson, MD^11^, Haeseong Park^12^, Liza Villaruz, MD^13^, Katrina Kao^3^, Elad Sharon, MD, MPH^14^, David Raben, MD^15^, Raymond Mak^3^, Howard Streicher, MD^14^, Helen Chen, MD^14^, Mansoor Ahmed, PhD^14^, Scott Rodig, MD, PhD^16^, F. Stephen Hodi, MD^3^, Jonathan Schoenfeld, MD, MPH^3^

###### ^1^UC Davis Cancer Center, Sacramento, CA, United States; ^2^Dana-Farber Cancer Institute, Boston, MA, United States; ^3^Dana-Farber Cancer Institute, Boston, MA, United States; ^4^University of Virginia, Charlottesville, VA, United States; ^5^University of North Carolina, Chapel Hill, United States; ^6^Mayo Clinic, Rochester, United States; ^7^Duke, Durham, United States; ^8^Rutgers Cancer Institute of New Jersey, New Brunswick, NJ, United States; ^9^University of Wisconsin, Madison, WI, United States; ^10^Cleveland Clinic, Cleveland, United States; ^11^Jefferson Medical Center, Philadelphia, PA, United States; ^12^Washington University in St. Louis, St. Louis, United States; ^13^University of Pittsburgh Medical Center, Pittsburgh, PA, United States; ^14^National Institutes of Health, Bethesda, MD, United States; ^15^University of Colorado, Greenwood Village, CO, United States; ^16^Brigham and Women's Hospital, Boston, MA, United States

####### **Correspondence:** Jonathan Schoenfeld (jdschoenfeld@partners.org)

**Background**

Preclinical data support combined PD-L1/CTLA-4 inhibition and suggest synergy between PD-L1/CTLA-4 inhibition and targeted radiation via enhanced systemic anti-tumor immune responses. We aimed to evaluate combined PD-L1/CTLA-4 inhibition in NSCLC patients who progressed on prior PD-(L)1 inhibitors and determine whether high- or low-dose radiation could increase objective responses outside the radiation field.

**Methods**

ETCTN 10021 is a multicenter randomized phase 2 study evaluating the addition of repeated low-dose fractionated radiotherapy (0.5 Gy BID x 2 days) or hypofractionated radiation (8 Gy x 3) concurrently with PD-L1/CTLA-4 inhibition (durvalumab 1500mg/tremelimumab 75mg q4w for 4 cycles followed by durvalumab monotherapy) in NSCLC patients progressive on prior PD-(L)1 inhibitors (intervening therapy allowed). Patients were randomized 1:1:1 to durvalumab/tremelimumab alone or with low-dose or hypofractionated radiotherapy. The primary endpoint was objective response per RECIST v1.1 excluding irradiated lesions with planned interim analysis. Correlative analyses were performed on tissue obtained following progression on prior PD-(L)1 inhibitors using PD-L1 immunohistochemistry and multiplex immunofluorescence (IF) evaluating CD8, CD4, PD1, Ki67, and cytokeratin in tandem.

**Results**

We randomized 78 patients (26 per each of 3 arms) who received >=1 cycle of study therapy between August 2017 and March 2019 across 18 sites. Patients received PD-(L)1 inhibitors for a median of 1 cycles (range 1-5) prior to enrollment; 68% had prior radiation. Treatment related adverse events (TRAE) of any grade were observed in 53 subjects (68%), and grade ≥3 events in 18 subjects (23%), including 1 grade 5 respiratory failure. Response rate across all cohorts were 10% (n=8, 95% exact CI: 5%-19%), and disease control 19% (n=15, 95% exact CI: 11-30%). Median duration of response was 10.3 months (95% CI: 1.4 months – not reached). Response and disease control weren’t significantly different between arms (Table 1, p=0.99/0.52, respectively), nor was time to progression (p=0.88, overall mTTP 3.3mos), or overall survival (p=0.40, overall mOS 11.4 mos); therefore, the study closed to enrollment at interim analysis. PD-L1 expression was not associated with response (p=0.52). IF demonstrated an association between intratumoral CD4+/PD1+/Ki67+ cells and response (p=0.02).

**Conclusions**

We did not observe a benefit adding targeted radiotherapy concurrently with combined PD-L1/CTLA-4 therapy in a PD-(L)1 inhibitor refractory NSCLC population. However, across cohorts PD-L1/CTLA-4 was generally tolerable and led to response/disease control in some patients, including responses >6 months. Multiplex IF suggests tumor infiltration by Ki-67+/PD-1+ CD4 T cells is associated with response and worthy of further investigation. Additional correlative genomic and immune analyses are planned.

**Trial Registration**

ClinicalTrials.gov Identifier: NCT02888743

**Ethics Approval**

This study was approved by the NCI Central IRB.

**Table 1 (abstract P344).**
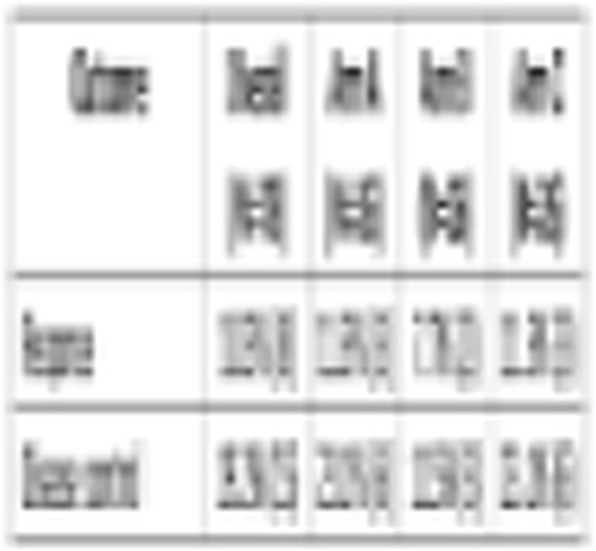
See text for description

#### P345 PARP inhibition when combined with PD-L1 inhibition has a suppressive effect on T cells in patients with relapsed or recurrent small cell lung cancer

##### Nobuyuki Takahashi, MD, PhD, Vinodh Rajapakse, Min-Jung Lee, Akira Yuno, Sunmin Lee, Sehyun Kim, Rasa Vilimas, Samantha Nichols, Jane Trepel, Anish Thomas

###### National Cancer Institute, Bethesda, MD, United States

####### **Correspondence:** Anish Thomas (anish.thomas@nih.gov)

**Background**

Poly ADP-ribose polymerase (PARP) inhibition increased PD-L1 expression, augmented cytotoxic T-cell infiltration and potentiated the anti-tumor effect of PD-L1 blockade in small cell lung cancer (SCLC) in vivo [1]. Yet in clinical studies, PARP inhibitor plus PD-L1 inhibitor did not improve responses in relapsed or recurrent SCLC patients compared to historical controls of PD-L1 inhibitor alone [2, 3]. Given the role of PARPs in activating inflammatory gene expression [4], we investigated the effects of PARP inhibition plus PD-L1 blockade on the adaptive immune system in SCLC patients.

**Methods**

NCT02484404 SCLC cohort is an open label phase 2 study evaluating the combination of durvalumab (1500 mg iv, Q4W) and olaparib (300 mg BID) in patients with relapsed or recurrent SCLC [2]. Peripheral blood lineages (pretreatment [C1D1], 2 weeks [C1D15] and 6 weeks [C3D1] after treatment) were serially assessed by flow cytometry.

**Results**

20 patients were evaluated. Activated Ki67+ HLA-DR+ T cells significantly decreased post-treatment (median [interquartile range] on C1D1 and C3D1: 3.5% [2.2–5.9] vs. 2.1% [1.8–3.3], p=0.033 among CD4+ T cells; 2.6% [1.8–5.1] vs. 1.4% [1.0–2.3], p=0.002 among CD8+ T cells). Activated Ki67+ PD-1- T cells also significantly decreased post-treatment (1.9% [1.2–3.9] vs. 1.3% [1.1–2.4], p=0.020 among CD4+ T cells; 3.2% [2.0–5.0] vs. 2.4% [2.0–2.7], p=0.025 among CD8+ T cells). By contrast, exhausted Ki67- TIM-3+ CD8+ T cells significantly increased post-treatment (0.8% [0.5–1.3] vs. 1.2% [0.8–2.0], p=0.002). PD-1 expression on regulatory Foxp3+ CD25+ T cells (Treg) and effector regulatory CD45RA- Foxp3hi T cells (eTreg) significantly increased post-treatment (median [IQR] of mean fluorescence intensity [MFI] ratio on C1D1 and C3D1: 2.2 [1.6–2.8] vs. 3.1 [2.2–3.4], p=0.002 among Treg; 2.4 [1.7–2.8] vs. 3.4 [2.3–4.2], p=0.002 among eTreg). CTLA-4 expression on non-regulatory Foxp3- CD4+ T cells (non-Treg) also increased post-treatment (0.18 [0.16–0.20] vs. 0.21 [0.18–0.23], p=0.007).

**Conclusions**

The combination of olaparib and durvalumab resulted in significant decrease in peripheral blood activated T cells, whereas exhausted T cells and inhibitory markers on Treg, eTreg and non-Treg cells significantly increased. These findings contrast with the expected changes under PD-L1 inhibitor treatment alone. These paradoxical changes of immune subsets likely reflect the anti-inflammatory effect of olaparib, which may have attenuated the antitumor immune efficacy of durvalumab.

**Trial Registration**

NCT02484404

**References**

1. Sen T, Rodriguez BL, Chen L, Corte CMD, Morikawa N, Fujimoto J, et al. Targeting DNA Damage Response Promotes Antitumor Immunity through STING-Mediated T-cell Activation in Small Cell Lung Cancer. Cancer Discov. 2019;9(5):646-61.

2. Thomas A, Vilimas R, Trindade C, Erwin-Cohen R, Roper N, Xi L, et al. Durvalumab in Combination with Olaparib in Patients with Relapsed SCLC: Results from a Phase II Study. J Thorac Oncol. 2019.

3. Krebs M, Ross K, Kim S, De Jonge M, Barlesi F, Postel-Vinay S, et al. P1.15-004 An Open-Label, Multitumor Phase II Basket Study of Olaparib and Durvalumab (MEDIOLA): Results in Patients with Relapsed SCLC. J Thorac Oncol. 2017;12(11):S2044-S5.

4. Rosado MM, Bennici E, Novelli F, Pioli C. Beyond DNA repair, the immunological role of PARP-1 and its siblings. Immunology. 2013;139(4):428-37.

**Ethics Approval**

The trial was conducted under a National Cancer Institute Center for Cancer Research–sponsored investigational new drug application with institutional review board approval; approval number 15-c-0145.

#### P346 Comparison of TMEs from melanoma and NSCLC patients refractory or resistant to anti–PD-(L)1 therapies

##### George Locke, Cherie Taglienti, PhD, Laureen S. Ojalvo, Christoph Helwig, MSc, Alex Rolfe, Olaf Christensen, Isabelle Dussault, PhD

###### EMD Serono Research & Development, Billerica, MA, United States

####### **Correspondence:** Isabelle Dussault (isabelle.dussault@emdserono.com)

**Background**

Bintrafusp alfa (M7824), an innovative first-in-class bifunctional fusion protein composed of the extracellular domain of the TGF-βRII receptor (a TGF-β “trap”) fused to a human IgG1 mAb blocking PD-L1, has shown evidence of clinical activity in phase 1 studies of patients with advanced solid tumors. Preliminary efficacy from a cohort of NSCLC patients who received prior immune checkpoint therapy was previously presented (SITC-2017); baseline tumor samples were collected to assess mechanisms of resistance to prior therapy. Here we describe the baseline tumor microenvironments (TMEs) of 2 cohorts of patients, melanoma and NSCLC, who experienced progressive disease on prior anti–PD-(L)1 treatment before starting bintrafusp alfa administration.

**Methods**

Fresh tumor biopsies from melanoma (n=29) and NSCLC (n=64) patients refractory or resistant to prior anti–PD-(L)1 therapy were processed for FFPE and subjected to RNA sequencing. Based on the mechanism of action of bintrafusp alfa, genes and gene signatures related to immune and TGF-β pathways were evaluated and compared between tumor types.

**Results**

Melanoma had elevated CD8+ T-cell gene signatures but low levels of predicted tumor neo-antigens. NSCLC had elevated gene signatures suggesting increased infiltration of inhibitory immune cells and gene signatures related to the TGF-β pathway. Lastly, certain mesenchymal markers were expressed at a higher level in melanoma compared with NSCLC—in particular, the gene encoding vimentin, which is associated with metastasis and poor prognosis, was 4-fold higher in melanoma.

**Conclusions**

The results suggest that this cohort of melanoma patients may have resisted prior immune therapy due to a low neo-antigen count and/or a mesenchymal phenotype. In contrast, the mechanisms of resistance in NSCLC patients in this study may have been related to high levels of inhibitory immune and TGF-β pathway genes. Collectively, these results provide novel insights into TMEs of melanoma and NSCLC in patients refractory or resistant to anti–PD-(L)1 agents.

**Ethics Approval**

This study was performed with IRB (#15C0179) and FDA approval and is registered with Clinicaltrials.gov on August 7, 2015 (NCT02517398). Patients provided written informed consent to participate.

#### P347 Immunomodulation in tumor and peripheral blood following Toca 511 & Toca FC treatment in patients with solid tumors

##### Gerald Falchook, MD^1^, Jordi Rodon^2^, Shree Venkat^3^, Arthur Donahue^4^, Peder Horner^4^, Amber Thomassen^3^, William Accomando^5^, Maria Rodriquez-Aguirre^5^, Cornelia Bentley^5^, Daniel Hogan^5^, Derek Ostertag^5^, Sharon Yavrom^5^, Thian Khoeh^5^, Douglas Jolly, PhD^5^, Harry Gruber, MD^5^, Jolene Shorr^5^, Jaime Merchan^3^

###### ^1^Sarah Cannon Research Institute, Denver, CO, United States; ^2^MD Anderson Cancer Center, Barcelona, Spain; ^3^University of Miami, Miami, FL, United States; ^4^Diversified Radiology of Colorado, Denver, CO, United States; ^5^Tocagen Inc., San Diego, CA, United States

####### **Correspondence:** Gerald Falchook (gerald.falchook@sarahcannon.com)

**Background**

Toca 511 (vocimagene amiretrorepvec) is a cancer-selective, gamma-retroviral replicating vector encoding yeast cytosine deaminase, an enzyme that converts 5 fluorocytosine (5-FC) into 5-fluorouracil in the tumor microenvironment. Preclinical models indicated that Toca 511 and 5-FC treatment kills dividing cancer and nearby immunosuppressive cells, leading to T-cell priming and antitumor immune activity [1]. A Phase 3 trial of Toca 511 & Toca FC (extended-release 5-FC) for treatment of recurrent high grade glioma is ongoing, following Phase 1 observations of prolonged survival and durable complete responses in some patients [2].

**Methods**

This Phase 1b, single-arm, multicenter study (Toca 6) was designed to investigate immunological changes following Toca 511 & Toca FC treatment in patients with advanced solid tumors. Patients received intravenous (IV) Toca 511 for 3 days (Week 1), underwent biopsy of metastatic tumor (Week 2), were dosed with oral Toca FC (Weeks 5 and 6), underwent follow-up biopsy (~Week 9), and then repeated oral Toca FC every 4-6 weeks. Longitudinal peripheral blood mononuclear cell (PBMC) samples were immunophenotyped by flow cytometry, and tumor biopsies were analyzed by immunohistochemistry (IHC).

**Results**

A total of 21 patients with a median 4 lines of prior chemotherapy were enrolled (17 colorectal cancer, 2 sarcoma, 1 each non-small cell lung and pancreas cancer). PBMC results suggest T-cell shifts from naïve to effector phenotypes, CD4+ memory T-cell expansion, and/or B-cell increases after Toca FC in 41% of patients with pre- and post-Toca FC blood samples. Following treatment with Toca FC, some patients showed marked changes in tumor infiltrating immune populations assessed by IHC, including decreases in CD11b+ myeloid cells, Tregs, and exhausted T-cells, and increases in CD8+ T-cells. In addition, IV Toca 511 led to viral expression in tumor, which was decreased post-Toca FC. Treatment has been generally well tolerated, with no related Grade 4 adverse events. At data cut-off, 9 patients were alive (median follow-up 10.4 months); median overall survival was 9.6 months (95% CI 6.3, 16.4). A patient receiving concomitant panitumumab had a partial response.

**Conclusions**

Results suggest Toca 511 infects metastatic tumor following IV administration, and subsequent Toca FC induces tumor and immunosuppressive cell killing. Preliminary analyses indicate Toca 511 & Toca FC treatment may be associated with T-cell mediated immune activity in peripheral blood and metastatic tumor, consistent with the immunologic mechanism of action observed in preclinical models. Preliminary clinical data suggest a signal of activity in these heavily pretreated patients warranting further investigation.

**Trial Registration**

NCT02576665

**References**

1. Mitchell LA, Lopez Espinoza FL, Mendoza D, et al. Toca 511 gene transfer and treatment with the prodrug, 5-fluorocytosine, promotes durable antitumor immunity in a mouse glioma model. Neuro Oncol. 2017;19:930-939.

2. Cloughesy TF, Landolfi J, Vogelbaum, et al. Durable complete responses in some recurrent high-grade glioma patients treated with Toca 511 + Toca FC. Neuro Oncol. 2018;20:1383-1392.

**Ethics Approval**

This study was approved by the institutional review boards of University of Miami Hospitals and Clinics, The University of Texas MD Anderson Cancer Center, and Sarah Cannon Research Institute at HealthONE.

#### P348 A phase 1 dose escalation study to evaluate the safety and tolerability of evofosfamide in combination with ipilimumab in advanced solid malignancies

##### Aparna Hegde, MD^1^, Priyamvada Jayaprakash, PhD^1^, Elizabeth Sumner^1^, Di Nguyen^1^, Hira Zain^1^, Sarina Piha-Paul, MD^1^, Daniel Karp^1^, Jordi Rodon^1^, Shubham Pant, MBBS^1^, Siqing Fu, MD, PhD^1^, Ecaterina Dumbrava, MD^1^, Timothy Yap, MD PhD^1^, Vivek Subbiah, MD^1^, Priya Bhosale, MD^1^, Jack Higgins, PhD^2^, Eric T.Williams^2^, Thomas F. Wilson^2^, Funda Meric-Bernstam, MD^1^, Michael Curran, PhD^1^, David Hong, MD^1^

###### ^1^The University of Texas MD Anderson Cancer Center, Bee Cave, TX, United States; ^2^Molecular Templates, Austin, TX, United States

####### **Correspondence:** Michael Curran (MCurran@mdanderson.org); David Hong (dshong@mdanderson.org)

**Background**

The anti-tumor responses to immune checkpoint inhibitors (ICI) is limited in malignancies such as pancreatic adenocarcinoma (PA), head and neck squamous cell carcinoma (HNSCC) and castration resistant prostate cancer (CRPC) through establishment of inaccessible hypoxic regions [1,2]. Under hypoxic conditions, evofosfamide (EVO) releases the alkylating agent Br-IPM, which decreases hypoxia, reduces density of MDSC, restores T cell infiltration and increases tumor antigen presentation [3]. Across syngeneic models, EVO demonstrates strong therapeutic cooperativity with ICI [4].

**Methods**

A phase 1, dose-escalation trial using 3+3 design was conducted to determine the safety, tolerability and activity of EVO in combination with ipilimumab (IPI) for the treatment of 4 tumor types: metastatic or locally advanced PA, HPV negative HNSCC, ICI-refractory melanoma and CRPC (NCT03098160). The study drugs (EVO, IPI) were given at the following doses respectively: level 1 (400mg/m2, 3mg/kg), level 2 (480mg/m2, 3mg/kg), level 3 (560mg/m2, 3mg/kg), level 4 (640mg/m2, 3mg/kg). EVO was administered on days 1 and 8 in cycles 1 and 2. IPI was administered on day 8 of each 3 week cycle for a maximum of 4 doses after which retreatment was allowed in those with irCR/ irPR/ irSD or irPD anytime after study initiation. Tumor response was assessed using irRECIST. Change from baseline in peripheral blood and tumor tissue immune and hypoxia parameters were evaluated as potential biomarkers of activity for this combination.

**Results**

Twenty-one patients with a median age of 67 years were enrolled in the study, of whom a majority had CRPC (n=11) followed by PA (n=7), melanoma (n=2) and HNSCC (n=1). Three patients were enrolled at level 1 and six in level 2, 3 and 4 each. The most common any grade adverse events were rash (n=17), anemia (n=16) and leukopenia (n=12). The most common grade 3 adverse events were transaminitis (n=4), lymphopenia (n=3) and anemia (n=3). One patient required treatment discontinuation and 3 required EVO dose reduction for toxicities. Out of 18 patients with measurable disease at baseline, three (16.7%) had PR (2 with CRPC and 1 with HNSCC) and twelve (66.7%) had SD. The best responses were observed at dose level 3 (Figure 1). Reduced hypoxic exposure of myeloid stroma, correlating with reduced suppressive polarization was observed.

**Conclusions**

No new or unexpected safety signals were observed with combined EVO and IPI. The combination showed evidence of activity in heavily pretreated refractory solid tumors. Dose expansion is planned at EVO 560mg/m2 and IPI 3mg/kg.

**References**

1. Blank CU, Haanen JB, Ribas A, et al: CANCER IMMUNOLOGY. The "cancer immunogram". Science 352:658-60, 2016

2. Chouaib S, Noman MZ, Kosmatopoulos K, et al: Hypoxic stress: obstacles and opportunities for innovative immunotherapy of cancer. Oncogene 36:439-445, 2017

3. Duan JX, Jiao H, Kaizerman J, et al: Potent and highly selective hypoxia-activated achiral phosphoramidate mustards as anticancer drugs. J Med Chem 51:2412-20, 2008

4. Ai M, Budhani P, Sheng J, et al. Tumor hypoxia drives immune suppression and immunotherapy resistance. J Immunotherapy of Cancer 2015;3(Suppl 2):P392.

**Ethics Approval**

The study was approved by University of Texas MD Anderson Cancer Center's Ethics Board, approval number IRB00000121.

**Consent**

Written informed consent was obtained from the patient for publication of this abstract and any accompanying images. A copy of the written consent is available for review by the Editor of this journal.

**Fig. 1 (abstract P348).**
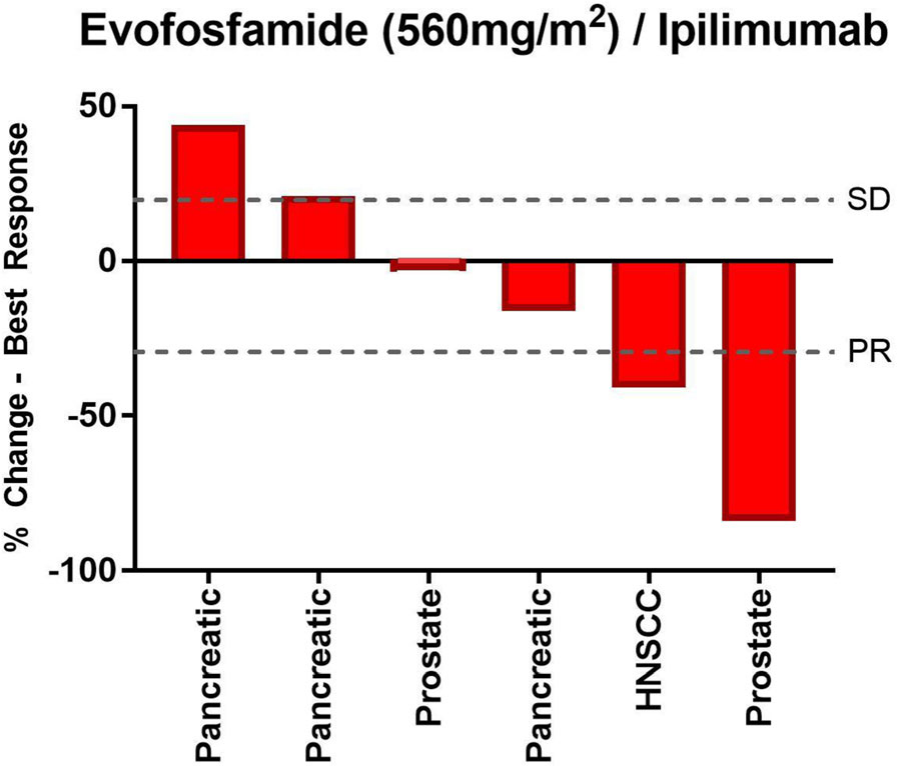
See text for description

#### P349 Effects of bintrafusp alfa (M7824) and radiation combination therapy on antitumor activity, immune response, and radiation-induced fibrosis in multiple cancer models

##### Yan Lan, MD, Chunxiao Xu, PhD, Huakui Yu, Guozhong Qin, Bo Marelli, Jin Qi, Rachel E. Fontana, Amit Deshpande, George Locke, Alex Rolfe, Molly H. Jenkins, Joern-Peter Halle, Kin-Ming Lo

###### EMD Serono Research & Development, Billerica, MA, United States

####### **Correspondence:** Yan Lan (yan.lan@emdserono.com)

**Background**

We recently reported the enhanced preclinical antitumor activity of bintrafusp alfa (M7824), an innovative first-in-class bifunctional fusion protein composed of the extracellular domain of the TGF-βII receptor (a TGF-β “trap”) fused to a human IgG1 mAb blocking PD-L1. In phase 1 and 1b expansion studies in patients with advanced solid tumors, bintrafusp alfa showed early evidence of clinical activity. Bintrafusp alfa is a particularly rational combination partner for radiation therapy (RT) because RT induces expression of TGF-β, which can promote epithelial-to-mesenchymal transition (EMT), fibrosis, and metastasis, and the expression of PD-L1. Furthermore, the induction of abscopal effects requires the combination of RT with immunotherapy in mouse models, and abscopal responses have been reported in patients receiving RT in combination with an immune checkpoint inhibitor.

**Methods**

The combination of bintrafusp alfa and RT was compared with bintrafusp alfa monotherapy or RT alone in MC38 colorectal carcinoma, GL261-luc2 glioma, and 4T1 breast cancer murine models. Antitumor activity was evaluated via tumor growth, survival, and lung metastases. Enzyme-linked immune absorbent spot (ELISpot) and immunohistochemistry were used to measure the function and infiltration of CD8+ T cells and the quantity of α-SMA, a marker of cancer-associated fibroblasts. Gene expression signature scores of different pathways were calculated from targeted RNAseq analysis.

**Results**

The combination therapy enhanced antitumor activity in all three murine models, increased tumor-specific and tumor-infiltrating CD8+ T cells in the MC38 and 4T1 models, respectively, and potentiated an abscopal effect in secondary MC38 tumors. In the 4T1 model, combination therapy decreased lung metastases vs either monotherapy and decreased the expression of EMT and VEGF pathway gene signatures vs RT. Expression of α-SMA significantly decreased with bintrafusp alfa monotherapy in this model, whereas it significantly increased with RT monotherapy. However, the combination with bintrafusp alfa was able to reduce α-SMA expression vs RT, suggesting that bintrafusp alfa can reduce RT-induced fibrosis, presumably via TGF-β blockade.

**Conclusions**

Collectively, these preclinical findings support the clinical development of bintrafusp alfa and RT combination therapy and support the rationale for a clinical trial investigating bintrafusp alfa in combination with chemoradiation (CRT) in stage III non-small cell lung cancer (NSCLC; NCT03840902). In addition, the enhanced efficacy seen in multiple murine models supports the broad application of this combination for treatment of additional cancer indications.

**Ethics Approval**

This study was approved by the Institutional Animal Care and Use Committee at EMD Serono, Inc.; approval number [17-008].

#### P350 Clinical signal/profile in a phase I study of T cCell receptor (TCR) affinity-enhanced specific T cells (TAEST) in advanced cancer patients

##### Yi Li, PhD^1^, Zhaosheng Han, PhD^1^, Xing Zhang, MD^2^, Jian Zhang^3^, Chengzhi Zhou^4^, Haiping Gong^1^, Desheng Weng, MD^2^, Jianchuan Xia, PhD MD^2^, Johnson Lau^5^, Shiyue Li^4^, Weiliang Zhu^3^

###### ^1^Guangdong Xiangxue Life Sciences, Ltd., Guangzhou, China; ^2^Sun Yat-sen University Cancer Center, Guangzhou City, Guangdong Provi, Peoples Republic of China; ^3^Zhujiang Hospital, Guangzhou, China; ^4^Guangzhou Institute of Respiratory Healt, Guangzhou, China; ^5^Axis Therapeutics Ltd., Hongkong, Hong Kong

####### **Correspondence:** Shiyue Li (lishiyue@188.com)

**Background**

T-cell triggering thresholds can be improved by engineered TCR with enhanced binding affinity. TAEST for NY-ESO-1 was designed for potentially better efficacy and good safety profile.

**Methods**

Preclinical: Determined the TCR affinities. Expression of CD3, CD4, CD8, and TCR were traced by antibodies/tetramer. Specificity/efficacy in vitro/in vivo and TAEST infiltration in tumor/lymph node were evaluated. Clinical: Phase I study – 14 advanced cancer patients were treated with TAEST.

**Results**

Preclinical: TAEST had higher affinity to its antigen vs wild type T-cells and with great expression (80-90% positive engineered TCR-T cells), with ~5-6X more CD8+ over CD4+ cells. There was great in vitro and in vivo efficacy with strong evidence of tumor specific TAEST infiltration. Clinical: Stage I - TAEST alone was dosed in 3 NSCLC patients with demonstrated safety and stable disease (SD) observed for 28-165 days (OS: 77-308 days). Stage II - lymphodepletion was added to TAEST in 11 patients (NSCLC 4, thyroid CA 1, liver CA 1, breast CA 1, colon CA 1, melanoma CA 1, synovial sarcoma 1,and fibrotic sarcoma 1,) and treated with 0.8-2.15x1010 TAEST cells: the synovial sarcoma patient had PR (> 70% tumor size reduction) with > 12 months duration; the breast CA patient had a > 40% tumor shrinkage with healing of skin metastatic ulcers during Rx; Two other patients (liver, thyroid CA) showed SD but significant tumor necrosis (>50%) with symptomatic relief of local pain. Three NSCLC patients had SD with 59-188 days (Survival 129-392 days). The fibrotic patient had SD for 87 days (Survival: 273 days); the melanoma patient had SD with 105 days (Survival: 176 days); last 2 patients ( NSCLC 1, colon CA 1) had PD at 14-16 days post infusion ( OS: 92-129 days) .

The treatment was tolerated well with fever (12/14), chills (4/14), neutropenia (5/14), thrombocytopenia(1/14), diarrhea(2/14), chest pain (1/14), and skin rash (3/14) observed. Expected cytokine response, TCR-gene detection/persistence (>60 days), were also observed in the patients above (particularly, >362 day for synovial sarcoma patient).

**Conclusions**

(1) TAEST, with its enhanced TCR binding affinity, is safe and tolerable in a clinical phase I study; (2) TAEST exhibits encouraging efficacy (DCR: 85.7%, 12/14) with a near CR for synovial sarcoma patient (duration >12 months), and marked tumor necrosis with two more patients (liver CA, thyroid CA); (3) Lymphodepletion pretreatment appeared to be critical for efficacy/cytokine response/persistence of TAEST cells.

**Acknowledgements**

The National key R&D Program of China, 2016YFC1303404; The Sciences and Technology Program of Guangzhou, No. 201704020220.

**Trial Registration**

ClinicalTrials.gov Identifier: NCT03159585; NCT03029273; NCT03462316

**Ethics Approval**

The study of bone sarcoma and soft tissue sarcoma was approved by Sun Yat-sen University Cancer Center, approval number B2017-023-01.

The study of NSCLC was approved by The First Affiliated Hospital of Guangzhou Medical University, approval number 2016 No.63.

The study of multiple solid tumor was approved by Zhujiang Hospital of Southern Mediacl University, approval number 2017-ZLZX-001.

**Consent**

Written informed consent was obtained from the patient for publication of this abstract and any accompanying images. A copy of the written consent is available for review by the Editor of this journal.

#### P351 Association between response assessment using RECIST and irRECIST in 1765 patients with advanced solid tumors treated with avelumab monotherapy

##### Juliane Manitz^1^, Peter Eggleton^2^, Marcis Bajars^1^, Oliver Bohnsack, MD, PhD, MBA^3^, James Gulley, MD, PhD, FACP^4^

###### ^1^EMD Serono Research and Development Institute, Inc, Billerica, Massachusetts, United States; ^2^Merck KGaA, Darmstadt, Germany, Darmstadt, Germany; ^3^PAREXEL Informatics, Berlin, Germany, Berlin, GERMANY; ^4^Center for Cancer Research, National Cancer Institute, National Cancer Institutes of Health, Bethesda, MD, United States

####### **Correspondence:** Juliane Manitz (juliane.manitz@emdserono.com)

**Background**

A subset of patients receiving immune checkpoint inhibitor (ICI) treatment may have unconventional response patterns, such as pseudoprogression, which can be classified as best overall response (BOR) of progressive disease (PD) by Response Evaluation Criteria in Solid Tumors (RECIST) v1.1; therefore, immune-related (ir) response criteria, irRECIST, have been proposed. This analysis reports the differences in response assessment by RECIST v1.1 and irRECIST and their association with overall survival (OS) in patients with advanced solid tumors treated with avelumab monotherapy (anti–PD-L1).

**Methods**

Data from patients with metastatic or locally advanced solid tumors (n=1677, data cutoff, February 15, 2017) enrolled in the phase 1, open-label JAVELIN Solid Tumor trial (NCT01772004), and data from patients with metastatic Merkel cell carcinoma with disease progression after prior chemotherapy (n=88, data cutoff, March 24, 2017) enrolled in part A of the phase 2 open-label JAVELIN Merkel 200 trial (NCT02155647) were pooled. Patients with castration-resistant prostate cancer from the JAVELIN Solid Tumor study were excluded. All patients received avelumab 10 mg/kg every 2 weeks by intravenous infusion. BOR, disease control rate, and progression-free survival (PFS) were evaluated. Concordance of disease control rates, Kaplan-Meier, landmark OS, and correlation analyses were performed.

**Results**

A total of 1765 patients were included. All patients had ≥3 months of follow-up. The pooled data set included 12 tumor types. The discordance between the tumor assessment criteria for disease control rate was 8.3% (n=147), i.e. complete response + partial response + stable disease (SD) per irRECIST and PD + not evaluable per RECIST; most patients (n=135) had a BOR of PD by RECIST and irBOR of SD by irRECIST. The Kaplan-Meier analysis according to Wolchok et al exhibited clear separation of the respective (dis)concordant subgroups. The rank correlations between OS and PFS and OS and irPFS were 0.73 (95% CI, 0.70-0.75) and 0.75 (95% CI, 0.72-0.78), respectively.

**Conclusions**

The discordance between disease control per irRECIST and RECIST suggests that for approximately one in 12 patients, irRECIST is a better indicator of clinical benefit from ICI treatment than RECIST. However, overall no stronger association was observed between OS and irPFS compared with between OS and PFS. Thus, neither RECIST nor irRECIST showed a clear advantage for predicting OS for clinical decisions or regulatory purposes.

**Acknowledgements**

This study was funded by Merck KGaA, Darmstadt, Germany, as part of an alliance between Merck KGaA, Darmstadt, Germany and Pfizer Inc., New York, NY, USA.

**Trial Registration**

All trials were registered at clinicaltrials.gov, trial numbers NCT01772004 and NCT02155647.

**Ethics Approval**

The trials were approved by the institutional review board or independent ethics committee at each participating center.

#### P352 Workflow for Immune Monitoring during Clinical Trials by using unsupervised high dimensional augmented intelligence assisted analysis

##### Alessandra Metelli, PhD, Carsten Krieg, PhD, Luis Cardenas, BS

###### Medical University of South Carolina, Charleston, SC, United States

####### **Correspondence:** Carsten Krieg (KriegC@musc.edu)

**Background**

Following checkpoint inhibitors, which significantly improved cancer treatment, an increasing number of combination therapeutics is being tested in clinical trials. To find possible clinical or biological correlates of response or intervention, high throughput multi-omic approaches are necessary to catch all features of possible immune responses during treatment.

**Methods**

To this aim, we present a portfolio of multi-omic approaches including high-dimensional mass cytometry (CyTOF) and single cell sequencing in combination with unsupervised machine-learning bioinformatics to perform in depth characterization of immune responses during clinical (immuno)therapy. The analysis is data driven, can be adapted to high throughput approaches and can model arbitrary trial designs.

**Results**

We here show three proof of concept projects using biobanked peripheral blood mononuclear cells (PBMCs). In the first study, 51 patients with stage IV melanoma before and after 12 weeks of anti-PD-1 therapy were analyzed. We observed a clear T cell response on therapy. The most evident difference in responders before therapy was an enhanced frequency of CD14+ CD16+HLA-DRhi classical monocytes. We validated our results using conventional flow and found a clear correlation of enhanced monocyte frequencies before therapy initiation with clinical response such as lower hazard and extended progression-free and overall survival. In a second study, we used CyTOF to monitor immune response in 21 non-small cell lung cancer (NSCLC) patients that initially responded and then progressed under anti-PD-1 to a novel combination immunotherapy of anti-PD-1 plus an IL-15 super-agonist (ALT-803). In this phase Ib clinical study a response in the CD8+ T cell compartment was observed. Unexpectedly, our high dimensional unbiased analysis was able to detect and characterize a strong expansion of innate tumor-reactive effector NK cells starting around day 4 of therapy. In our third unpublished study we were able to identify neutrophils as predictors of outcome in lung cancer patients.

**Conclusions**

Taken together, our unbiased artificial intelligence-driven immune workflow is an extraordinary instrument to monitor immune responses during (immuno)therapy and serves as a novel approach for therapeutic target identification.

**Trial Registration**

ClinicalTrials.gov Identifier: NCT02523469

**Ethics Approval**

This study was approved by the MUSC and Zurich institutional review board.

#### P353 Phase 1 pilot study of RRx-001 + nivolumab in advanced metastatic cancer (PRIMETIME)

##### Corey Carter, MD^1^, Bryan Oronsky, MD PhD^2^, Mary Quinn^1^, Jane Trepel^3^, Nacer Abrouk, PhD^4^, Jeff Skinner, MD^5^

###### ^1^EpicentRx, Inc., La Jolla, CA, United States; ^2^EpicentRx Inc, La Jolla, CA, United States; ^3^NIH, Bethesda, MD, United States; ^4^Clinical Trials Innovations, Mountain View, CA, United States; ^5^Walter Reed National Mil Medical Center, Bethesda, MD, United States

####### **Correspondence:** Mary Quinn (mquinn@epicentrx.com)

**Background**

RRx-001 is a minimally toxic small molecule that downregulates CD47 and repolarizes tumor associated macrophages (TAMs) as well as normalizes aberrant tumor perfusion. On the premise that the interaction between a CD-47 downregulator like RRx-001 and an anti-PD-1 inhibitor like nivolumab may serve to activate both arms of the immune system, a phase 1 pilot study was undertaken to determine the safety and feasibility of RRx-001 and nivolumab in patients with advanced cancer and no standard options.

**Methods**

This single arm, open-label pilot study (NCT02518958) called PRIMETIME was designed to evaluate the safety profile of RRx-001 and nivolumab in patients with advanced malignancies and no other standard therapeutic options. A 3+3 trial design was used to establish safety of the combination at each dose level and guide the decision to escalate dose. RRx-001 is infused once weekly while nivolumab is given at 3mg/kg once every 2 weeks. The RRx-001 starting dose was 2 mg IV weekly with 4 dose level escalations up to 16 mg IV weekly. From January 2015 to November 2015, twelve patients received treatment for only 4 cycles (total 12 weeks) with the combination due to unavailability of nivolumab, which was not supplied to the Sponsor. Treatment-emergent (all cause, TEAEs) and treatment-related (TRAEs) adverse events that occurred within 16 weeks of the first dose of RRx-001 and nivolumab were characterized according to CTCAE v4.03.

**Results**

Twelve patients received >1 dose of RRx-001 and nivolumab. One discontinuation occurred due to pneumonitis and one to voluntary withdrawal after a post-procedural infection. There were no DLTs. The main adverse event related to RRx-001 was infusion reaction (33.3%). The main adverse event related to the combination was pseudoprogression manifested by larger tumors in patients that were symptomatically improved (25%). The most common immune-related treatment-emergent AEs were pneumonitis (8.3%), and hypothyroidism (8.3%). The objective response rate at 12 weeks was 25% and the disease control rate (DCR) consisting of > SD was 67% by Response Evaluation Criteria in Solid Tumors (RECIST) version 1.1 25% of the patients progressed on the combination.

**Conclusions**

The combination of RRx-001 and nivolumab was safe and well-tolerated with preliminary evidence of anti-cancer activity. Further analyses with a larger sample size will be required to confirm the activity of the combination and to determine the optimum schedule for RRx-001 and nivolumab.

**Ethics Approval**

The study was approved by all the revelant Institution‘s Ethics Boards.

#### P354 Exploring correlates of clinical and immune response to cancer immunotherapy using FAUST, a novel unbiased cell population discovery method, in whole blood flow cytometry

##### Steven Fling, PhD, Nirasha Ramchurren, PhD, Leonard D'Amico, Martin Cheever, MD, Evan Greene, Greg Finak, Raphael Gottardo, PhD

###### Fred Hutchinson Cancer Research Center, Seattle, WA, United States

####### **Correspondence:** Steven Fling (sfling@fredhutch.org)

**Background**

The purpose of this study is to describe an immune monitoring approach that combines multi-parameter whole blood flow cytometry with an automated, unbiased, cell population evaluation method to facilitate discovery of informative correlative biomarkers. The Cancer Immunotherapy Trials Network (CITN) coordinates multi-center cancer immunotherapy trials, wherein multiparameter flow cytometry is performed in real time on longitudinally collected whole blood samples.

**Methods**

We recently reported results from two CITN multi-center clinical trials. We also reported a non-parametric method for unbiased cell population discovery that annotates cell populations with biologically interpretable phenotypes through a new procedure called Full Annotation Using Shape-constrained Trees (FAUST). We used FAUST to analyze extensive flow cytometry data in these two CITN clinical trials and demonstrate that candidate biomarkers can be associated with clinical outcome. Here we compare flow cytometry data analyzed both by conventional manual gating strategies as well as by the FAUST method.

**Results**

We highlight the value of FAUST in identifying predictive biomarkers of clinical responses to immunotherapy within fresh whole blood. By combining whole blood flow staining with FAUST, our results demonstrate the ability to capture important minor cell subpopulations, including within the CD8 T cell compartment, that otherwise are missed by manual gating. Manual gating can be biased and limited to characterizing cell populations considered a-priori to be significant.

**Conclusions**

Our results emphasize the unique value of performing flow cytometry in multi-center trials using fresh whole blood which preserves the minor cell populations identified by FAUST which may be lost or compromised by standard cryopreservation methods.

#### P355 Multicenter, open-label, phase 1 study of DSP-7888 Dosing Emulsion (DSP-7888) in patients with advanced malignancies

##### Morris Groves^2^, Aaron Hansen^3^, Wael Harb^4^, Kelly Curtis, MD^5^, Erina Koga-Yamakawa^6^, Makoto Origuchi^7^, Zhonggai Li^7^, Jose Iglesias^8^, Walid Shaib, MD^9^, Alexander Spira, MD, PhD, FACP^1^

###### ^1^Virginia Cancer Specialists, Fairfax, VA, United States; ^2^Texas Oncology-Austin Midtown, Austin, TX, United States; ^3^UHN Princess Margaret Cancer Centre, Toronto, Canada; ^4^Horizon Oncology Research, LLC, Lafayette, IN, United States; ^5^Syneos Health, Phoenix, AZ, United States; ^6^Sumitomo Dainippon Pharma Co., Ltd, Cambridge, MA, United States; ^7^Boston Biomedical, Inc., Cambridge, MA, United States; ^8^Former employee, Boston Biomedical, Inc., Cambridge, MA, United States; ^9^Emory University, Atlanta, GA, United States

####### **Correspondence:** Alexander Spira (Alexander.Spira@USOncology.com)

**Background**

DSP-7888, a cancer vaccine composed of 2 synthetic peptides derived from Wilms’ tumor 1 (WT1) protein, may induce WT1-specific cytotoxic T-lymphocytes (CTLs) and helper T-lymphocytes–mediated immune responses against WT1-expressing tumors. This dose-escalation study (NCT02498665) evaluated DSP-7888 in patients with recurrent or progressive advanced malignancies, despite receiving standard therapy, or in patients intolerant to standard therapy or for whom no standard of therapy existed for their malignancy. The primary objectives were safety, tolerability, and identification of the recommended phase 2 dose (RP2D). Secondary and exploratory objectives included overall survival and WT1-specific CTLs induction.

**Methods**

Patients who failed or were intolerant to prior lines of treatment and tested positive for HLA-A*02:01, HLA-A*02:06, or HLA-A*24:02 received escalating doses of intradermal (ID) or subcutaneous (SC) DSP-7888 in a rolling study design: 3.5, 10.5, or 17.5 (ID only) mg every week for 4 weeks, then every 1–2 weeks for 6 weeks, and every 2–4 weeks thereafter until progression or other discontinuation event was met. Dose-limiting toxicities (DLTs) were evaluated over days 1–29. The dose at which ≤1 of 6 patients had a DLT was eligible to be the RP2D. WT1-specific CTL inductions were assessed by HLA Tetramer with peripheral blood.

**Results**

Twenty-four patients received ID (3.5 mg, n=4; 10.5 mg, n=3; 17.5 mg, n=3) or SC DSP-7888 (3.5 mg, n=9; 10.5 mg, n=5). The most frequent adverse event (AE) was injection site reaction (ISR; n=15; 62.5% [ID: 100% of patients, SC: 36%]); all were grade 1 or 2. No DLT was observed. ID DSP-7888 10.5 mg was determined to be the dose level for further study based on the RP2D identified in a phase 1/2 study of DSP-7888 in patients with myelodysplastic syndrome (NCT02436252). Four patients (ID 17.5 mg, n=1; SC 3.5 mg, n=1; SC 10.5 mg, n=2) had stable disease, 16 had progressive disease, and 4 were not evaluable. Twenty-one patients were evaluable for WT1-specific CTL detection. In evaluable patients, WT1-specific CTL induction was observed in 6 of 9 ID patients (66.7%) and 5 of 12 SC patients (41.7%).

**Conclusions**

DSP-7888 was well tolerated, with no DLTs, in patients with advanced malignancies, supporting further evaluation of DSP-7888. The 10.5 mg ID dose was identified as a dose level and route of administration for further evaluation.

#### P356 First-in-human study of the cancer peptide vaccine, TAS0313, in patients with advanced solid tumors: phase I dose finding part results

##### Noboru Yamamoto, MD, PhD, Jun Sato, MD, PhD, Satoru Iwasa, MD, PhD, Kan Yonemori, MD, PhD, Takafumi Koyama, MD, Kenji Tamura, MD, PhD, Toshio Shimizu, MD, PhD, Syunsuke Kondo, Shigehisa Kitano

###### National Cancer Center Hospital, Tokyo, Japan

####### **Correspondence:** Toshio Shimizu (tosshimi@ncc.go.jp)

**Background**

TAS0313 is a cancer vaccine cocktail containing three long peptides, with a total of 12 cytotoxic T lymphocyte (CTL) epitope peptides. These peptides were derived from eight cancer-associated antigens that are highly expressed in various cancers. We report the results of a phase I part examining the tolerability, safety, potential efficacy, and immunological responses of 9 mg and 27 mg TAS0313 in patients with advanced solid tumors.

**Methods**

The enrolled patients had ECOG PS 0-1 and at least one of the following HLA types: HLA-A*02:01, -A*02:06, -A*02:07, -A*11:01, -A*24:02, -A*31:01, or -A*33:03. Emulsified TAS0313 solution with an immunological adjuvant (Montanide ISA-51) was subcutaneously administered on Days 1, 8, and 15 of Cycles 1 and 2 and on Day 1 of Cycle 3 or later in 21-day cycles until disease progression, or unacceptable toxicity.. Tolerability was assessed in at least six patients during the first cycle. Tumor response was evaluated using RECIST v1.1. Blood samples were collected pre- and post-treatment for the analysis of antigen specific CTL and IgG. Optional serial tumor biopsies were performed for tumor infiltrating leukocyte (TIL) analysis. CTL, IgG, and TIL were measured by ELISPOT assay, Luminex assay, and IHC (CD8 positive), respectively.

**Results**

Seventeen patients were enrolled in 9mg (n=10) and 27mg (n=7) groups; the median age was 65 years, and 53% of the patients had ECOG PS 1. There was no serious adverse drug reaction (ADR) in any patient. All ADRs were of grade 1 or 2, with the most frequent being dermatological injection site reaction, in 7/10 (70%) and 6/7 (86%) patients and pyrexia, in 1/10 (10%) and 2/7 (29%) for the 9mg and 27mg groups, respectively. The best overall response was stable disease, in 2/10 (20%) and 2/7 (28%) patients. One patient with cancer of unknown origin received prolonged administration (over 10 months) of the 9mg dose. In the 9mg and 27mg groups, antigen specific IgG was augmented in 9/10 (90%) and 7/7 patients (100%), antigen specific CTL was detected in 2/10 (20%) and 3/7 patients (43%), and TIL counts were increased in 2/3 (67%) and 3/4 patients (75%), respectively.

**Conclusions**

TAS0313 demonstrated safety, tolerability, and immunological responses in patients with advanced solid tumors in the 9mg and 27mg groups. A phase II part, evaluating the efficacy of combination therapy with pembrolizumab in patients with urothelial carcinoma and monotherapy in glioblastoma patients, is currently underway.

**Trial Registration**

JapicCTI-183824

**Ethics Approval**

The study was approved by National Cancer Research Center Central Hospital’s Ethics Board, approval number T4499.

**Consent**

Written informed consent was obtained from the patients for publication of this abstract. A copy of the written consent is available for review by the Editor of this journal.

#### P357 Phase I/II clinical and immune responses for locally advanced or metastatic pancreatic cancer using anti-CD3 x anti-EGFR bispecific antibody armed T cells (BATs)

##### Lawrence Lum, MD, DSc^1^, Tri Le^1^, Minsig Choi^2^, Archana Thakur, PhD^1^, Matthew Reilly^1^, Paul Kunk, MD^1^, Abhinav Deol, MD^3^, Karen Ballen, MD^1^, Tamila Kindwall-Keller, DO^1^, Dana Schalk^1^, Ewa Kubicka^1^, Manley Huang, PhD^1^, Philip Philip, MD^3^, Hussein Aoun^3^, Gregory Dyson, PhD^3^, Qin Liu^4^, Anthony Shields, MD PhD^3^

###### ^1^University of Virginia, Charlottesville, VA, United States; ^2^Stony Brook University, Stony Brook, NY, United States; ^3^Karmanos Cancer Institute, Wayne State U, Detroit, MI, United States; ^4^Wistar Institute, Philadelphia, PA, United States

####### **Correspondence:** Lawrence Lum (lawrenceglum@cs.com)

**Background**

Chemotherapy for locally advanced pancreatic cancer (LAPC) and metastatic pancreatic cancer (MPC) has poor responses and survival rates. Retargeting anti-CD3 activated T cells (ATC) by arming them with anti-CD3 x anti-EGFR bispecific antibody (EGFRBi) makes ATC into specific cytotoxic T lymphocytes (EGFR BATs). Targeting pancreatic cancer cell lines induces cytokine secretion, proliferation, cytotoxicity, and inhibits tumor growth. We present 5 phase I and 13 phase II patients for a total of 18 evaluable patients out of 21 who underwent apheresis.

**Methods**

In the phase I, LAPC or MPC patients at Karmanos Cancer Institute (KCI) on Protocol #2011-025, in a dose escalation, were given 10, 20, and 40 x 10^9 BATs/infusion weekly for 3 weeks, followed by a booster infusion 3 months later if patients were stable or better. There were no dose limiting toxicities, and all infusions were given in the outpatient setting. In the phase II portion, 13 PC patients at KCI (NCT02620865) and University of Virginia (NCT03269526) received twice weekly infusions of 10 x 10^9 BATs/infusion over 4 weeks for a total of 80 x 10^9 EGFR BATs.

**Results**

Eighteen patients were evaluable. Four patients were stable at 6.1, 6.5, 5.3, and 39 months. Two patients developed complete responses (CR) when chemotherapy was restarted after their BATs infusions. Patient IT20104 was stable for 1 year on capcitabine, developed “pseudoprogression,” achieved a CR after restarting capcitabine, and was off therapy until 54 months after enrollment when relapse occurred. The median overall survival is 14.8 months with a time to progression of 6.6 months. Specific cytotoxicity mediated by peripheral blood mononuclear cells (PBMC) peaked at 31% two weeks after the third infusion, and IFN-γ EliSpots rose from

**Conclusions**

EGFR BATs infusions were safe and induced specific adaptive anti-tumor responses. This phase I/II study strongly suggests that multiple infusions of EGFR BATs may provide a survival benefit in patients with pancreatic cancer, and that BATs therapy may increase the effectiveness of subsequent chemotherapy, which will drive the design of future combination trials of BATs and other modalities of therapy.

**Acknowledgements**

These studies were made possible thanks to philanthropy from Karmanos Cancer Institute and start-up funds for LGL from the University of Virginia. LGL and MH are co-founders of Transtarget, Inc. LGL is a member of the Scientific Advisory Board for Rapa Therapeutics. AT is a co-founder of NOVA Immune Platform.

**Trial Registration**

Protocol #2011-025; NCT02620865; NCT03269526

**Ethics Approval**

These studies were approved by the Karmanos Cancer Institute / Wayne State University IRB, approval numbers 2011-25 and 2015-100, and the University of Virginia IRB, approval number HSR 19236.

#### P358 Phase 1 trial of NY-ESO-1-specific adoptive T-cell therapy with GSK3377794 in patients with advanced synovial sarcoma

##### Sandra D'Angelo, MD^1^, George Demetri^2^, Brian Van Tine, MD, PhD^3^, Mihaela Druta^4^, John Glod, MD^5^, Warren Chow^6^, Jenna Tress^7^, M. Phillip DeYoung^7^, Aisha Hasan, MBBS MD^7^, Yuehui Wu^7^, David Turner^7^, Ran Ji^7^, Alexandra Gyurdieva^7^, Dejka Araujo, MD^8^

###### ^1^Memorial Sloan Kettering Cancer Center, New York, NY, United States; ^2^Dana-Farber Cancer Institute, New York, NY, United States; ^3^Washington University in St. Louis, St. Louis, MO, United States; ^4^H. Lee Moffitt Cancer Center, Tampa, FL, United States; ^5^National Cancer Institute, Bethesda, MD, United States; ^6^City of Hope Comprehensive Cancer Center, Duarte, CA, United States; ^7^GlaxoSmithKline, Collegeville, PA, United States; ^8^MD Anderson Cancer Center, Houston, TX, United States

####### **Correspondence:** Sandra D'Angelo (dangelos@mskcc.org)

**Background**

Genetically-engineered NY-ESO-1 specific T-cells (NY-ESO-1 T-Cells; GSK3377794) are autologous CD4+ and CD8+ T cells transduced with a self-inactivating lentiviral vector to express affinity-enhanced NY-ESO-1-specific T-cell receptors (TCRs). Ongoing phase 1 and 2 trials are evaluating GSK3377794 in solid tumors and multiple myeloma. Study NCT01343043 (208466) is a phase 1 clinical trial assessing GSK3377794 in patients with previously treated, advanced metastatic synovial sarcoma (SS), stratified into 4 cohorts (Table 1). Of 12 patients receiving GSK3377794 infusion in Cohort 1, responses were observed in 6 patients (1 complete response [CR]/5 partial responses [PR]), with an overall response rate (ORR) of 50% (95% confidence interval [CI]: 0.21–0.79). Median progression-free survival (PFS) was 15.2 weeks (95% CI: 7.6–37.9); median duration of response (DoR) was 30.9 weeks (95% CI: 14–72). As of October 15, 2018, median overall survival (OS) was 105 weeks (95% CI: 37–NA). This abstract reports data from Cohorts 2 and 4.

**Methods**

Patients with advanced SS were enrolled to cohorts based on NY-ESO-1 expression (Cohort 2, low; Cohort 4, high) determined by immunohistochemistry. Treatment response (RECIST v1.1), safety (CTCAE v4.0), and GSK3377794 persistence in transduced PBMCs (transgene vector copies measured by qPCR) were assessed. Progression-free survival (PFS) was defined as the interval between first infusion and first documented disease progression or death. Safety was monitored throughout. The study was not designed/powered for cohort comparison.

**Results**

As of April 2019, 50 patients were enrolled (N=13 Cohort 2; N=15 Cohort 4). Table 2 summarizes response outcomes by Cohort. Median PFS (95% CI) was 13.1 weeks (7.9, 13.9; Cohort 2) and 22.4 weeks (11.3, 26.6; Cohort 4). Median peak (range) persistence of ~64,712 DNA copies/μg (13,364–197,546) occurred in Cohort 2 first week post-infusion versus ~16,468 DNA copies/μg (163–131,175) in Cohort 4. No significant correlation was observed between peak persistence and best overall response in either cohort (p>0.05). Grade 3/4 adverse events occurring in ≥40% of patients in both cohorts were leukopenia, neutropenia, anemia, thrombocytopenia, lymphopenia, and hypophosphatemia.

**Conclusions**

Cohorts 2 and 4 showed similar ORRs; more durable responses were observed in Cohort 4, with prolonged DoR, duration of stable disease, and PFS. Peak persistence of GSK3377794 was higher in Cohort 2, likely due to higher lymphodepletion, but this did not correlate with response, unlike data previously reported in other cohorts. Further development in SS will be based on previously reported data from Cohort 1.

**Acknowledgements**

Medical writing assistance was provided by provided by Fiona Woodward and Victoria Hunter of Fishawack Indicia Ltd. This study (NCT01343043) was funded by GlaxoSmithKline.

**Trial Registration**

NCT01343043

**Ethics Approval**

This study was approved by the appropriate institutional review boards and independent ethics committees.

**Table 1 (abstract P358).**
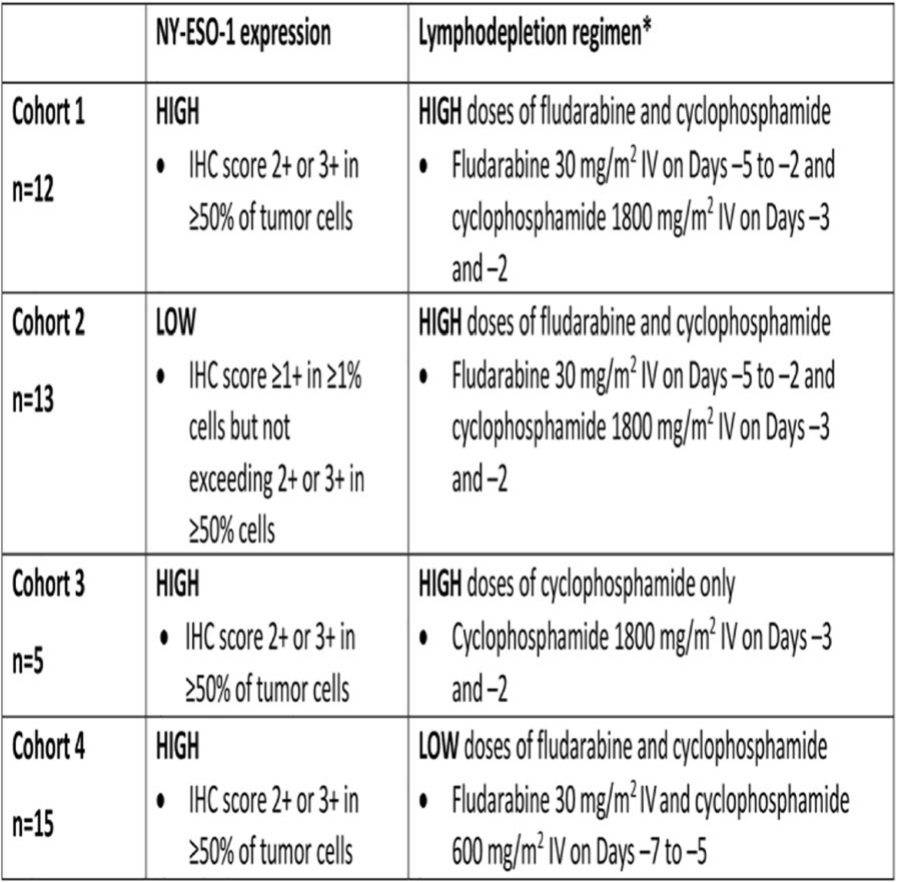
See text for description

**Table 2 (abstract P358).**
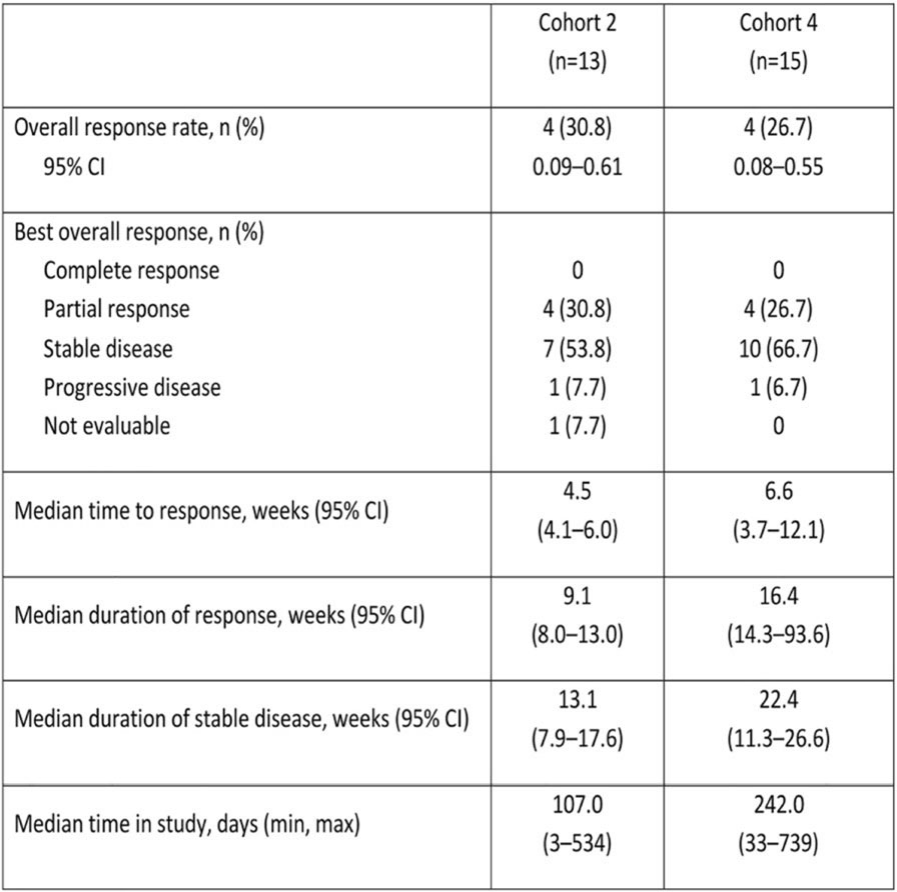
See text for description

#### P359 Tracking and profiling of NY-ESO-1 TCR-transgenic T cells upon adoptive transfer in patients with NY-ESO-1-expressing solid tumors: in vivo differentiation associated with response

##### Michael Fehlings^1^, Alessandra Nardin, DVM^1^, Faris Kairi^1^, Evan Newell, PHD^2^, Yoshihiro Mihayara^3^, Shinichi Kageyama^3^, Hiroshi Shiku, MD^3^

###### ^1^immunoSCAPE, Singapore, Singapore; ^2^Fred Hutchinson Cancer Research Center, Singapore, Singapore; ^3^Mie University School of Medicine, Tsu, Japan

####### **Correspondence:** Hiroshi Shiku (shiku@clin.medic.mie-u.ac.jp)

**Background**

NY-ESO-1 is highly expressed in the majority of synovial sarcomas as well as other solid tumors and may be an effective target for T cell-based therapies. We conducted a clinical study of adoptive transfer of lymphocytes transduced with NY-ESO-1-specific TCR in refractory cancer patients with preconditioning (TBI-1301).

**Methods**

High-dose of 5 billion autologous transduced and expanded lymphocytes, consisting of >96% T cells, was transferred into 6 patients, three of whom with synovial sarcoma. Longitudinal PBMC samples were obtained for immunomonitoring. We used high-dimensional mass cytometry and combined a 36-antibody panel with a multiplexed combinatorial peptide-MHC tetramer staining approach to longitudinally track and phenotypically characterize adoptively transferred HLA-A*02:01-NY-ESO-1 transgenic TCR T cells 14, 28, and 56 days after treatment.

**Results**

Three out of 6 patients with tumors expressing >75% NY-ESO-1 experienced an objective clinical response (PR) and had cytokine-release syndrome (CRS) with high-levels of IL-6 and MCP-1 that could be managed with tocilizumab. The infusion products had variable percentages of naive, TEMRA and EM CD8+ cells, with the three clinical responders having the highest proportion of EM T cells. NY-ESO-1 TCR transgenic T cells could be detected in the circulation of 5 out of 6 treated patients, with frequencies peaking at day 14 and day 28; specific T cells were undetectable in all patients by day 56. In responders, a substantial number of circulating NY-ESO-1-specific CD8+ T cells showed a phenotypic profile consistent with antigen-experience, activation and differentiation into an effector phenotype 28 days post transfusion.

**Conclusions**

Adoptive transfer of NY-ESO-1 TCR-transgenic T cells has shown signs of efficacy in patients with high NY-ESO-1 tumor expression, with manageable adverse events. Our study shows feasibility of tracking phenotypic profiles of adoptively transferred tumor-antigen-specific T cells in patients and derive association between adoptive T cell status and clinical read-outs.

**Ethics Approval**

The study was approved by Mie University Ethics Board, approval number H2018-092.

#### P360 The influence of Durvalumab/Tremelimumab Combination Therapy on Sarcomas Immune Microenvironment profile in a phase II clinical trial (NCT02815995)

##### Edwin Parra, MD, PhD^1^, Carmelia Barreto, PhD^1^, Ruth Salazar, MD^1^, Cara Haymaker, PhD^1^, Heather Lin^1^, Carmen Behrens, MD^1^, Mei Jiang^1^, Luisa Solis, MD^1^, Krishna Pandurenga, MS^1^, Sandesh Subramanya, PhD^1^, Young Kim, PhD^2^, Chantale Bernatchez^1^, Jack Lee, PhD^1^, Taylor Tate^1^, Teresa Simmons^1^, Alexander Lazar, MD, PhD^1^, Wei-Lien Wang^1^, Zachary Cooper, PhD^3^, Jaime Rodriguez-Canales, MD^3^, Jean Soria, MD^3^, Anthony Conley, MD^1^, Ignacio Wistuba, MD^1^, Neeta Somaiah, MD, MBBS^1^

###### ^1^MD Anderson Cancer Center, Houston, TX, United States; ^2^Translational Molecular Pathology, Houston, TX, United States; ^3^AstraZeneca, Gaithesburg, MD, United States

####### **Correspondence:** Edwin Parra (erparra@mdanderson.org)

**Background**

To determine the tumor microenvironment (TME) changes after the combination of durvalumab/tremelimumab treatment, longitudinal sarcoma tissue collections were obtained and analyzed for in-depth immunoprofiling.

**Methods**

Sixty-two patients were enrolled and 36 paired samples (Liposarcoma,LS=6; Angiosarcoma,AS=2; Leiomyosarcoma,LMS=2; Osteo/Chondrosarcoma,OS/CS=3/1; Undifferentiated Pleomorphic Sarcoma,UPS=3; Alveolar Soft Part Sarcoma,ASPS=8; Synovial Sarcoma,SS=3; Chordoma,C =2; and other types,OT=6), were evaluable for TME changes and correlated with clinical benefit (PR or SD). All patients were treated with durvalumab/tremelimumab every 4 weeks for four cycles and then continued durvalumab every 4 weeks for up to 1 year. Biopsies were collected prior to-and- during treatment (Wk6). Malignant cells (MCs) PD-L1+ was studied by immunohistochemistry. Tumor-infiltrating-lymphocytes (TILs), and macrophages were interrogated by multiplex immunofluorescence, Figure-1. The combination of three T-cell phenotypes (CD3+,CD3+CD8+ and CD3+CD8+GZB+) greater than the median density as higher TILs (TILs+) was used to define “inflamed” tumors and ≤ than the median of 1 or 2 of these as lowest TILs (TILs–) to define “non-inflamed” tumors, through baseline to Wk6. To characterize patterns of the TME and changes between baseline and Wk6 we stratified the tumors in four groups using an approach similar as Teng's criteria (1).

**Results**

Overall, all the phenotype median densities increased from baseline to Wk6, Table-1. PR or SD was observed in 17/36 with paired samples, 47% (3 LS, 1 LMS, 1 OS, 7 ASPS, 1 SS, 2 C, and 2 OT). Five ASPS showed PR and 2 SD out of 8 cases. We categorized as inflamed tumor 1 LPS, 1 AS, 1 LMS, 1 OS, and 5 ASPS at baseline. Interestingly 1 AS, 1 OS, 1 CS, 1ASPS, 1 SS and 2 OT defined as non-inflamed tumors at baseline changed to inflamed tumors at Wk6 (Figure-2) and from those, OS and ASPS showed SD and PR, respectively. Finally, 4/17 inflamed tumors showed SD and 3/17 PR. Furthermore, CD3+CD8+CD45Ro+ increase in the inflamed tumors at Wk6 than non-inflamed tumors (P=0.005). The most frequent TME pattern detected at baseline and Wk6 was the immunological ignorance (TILs-PD-L1-, 61% and 47%, respectively). Interestingly, adaptive immune resistance pattern changed from baseline to Wk6 (TILs+PD-L1+, 14% and 22%, respectively), Figure-3.

**Conclusions**

Combination of durvalumab/tremelimumab influenced the TME in the selected sarcomas cohorts. The immunologic score assessment in this longitudinal collection demonstrates the capability to distinguish non-inflamed vs inflamed tumors and relate it with a clinical benefit, showing the value of use these markers as possible immune prognostic markers in sarcomas.

**Trial Registration**

This trial is registered with ClinicalTrials.gov (NCT02815995)

**References**

1. Teng MW, Ngiow SF, Ribas A, Smyth MJ. Classifying Cancers Based on T-cell Infiltration and PD-L1. Cancer Res. 2015;75(11):2139-45.

**Ethics Approval**

The study was approved by MD Anderson Institution Ethics Board, Clinical Trail number NCT02815995

**Table 1 (abstract P360).**
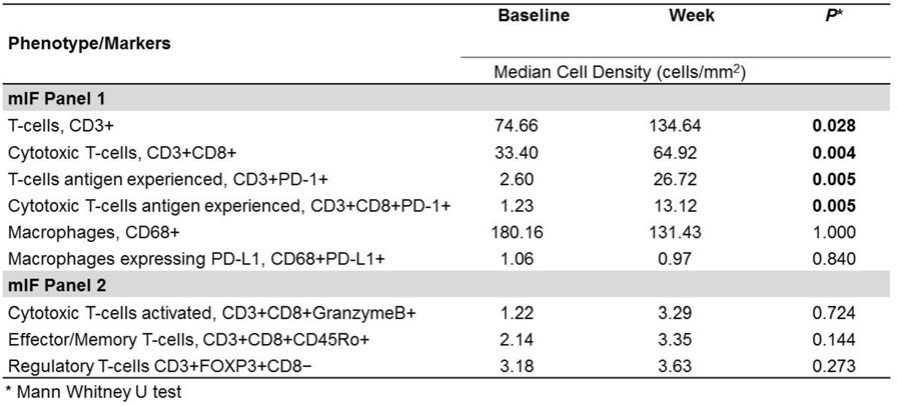
See text for description

**Fig. 1 (abstract P360).**
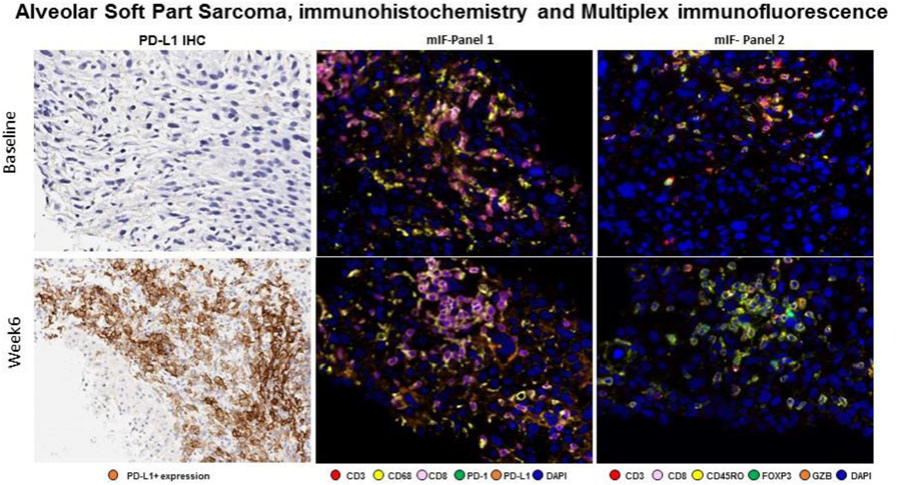
See text for description

**Fig. 2 (abstract P360).**
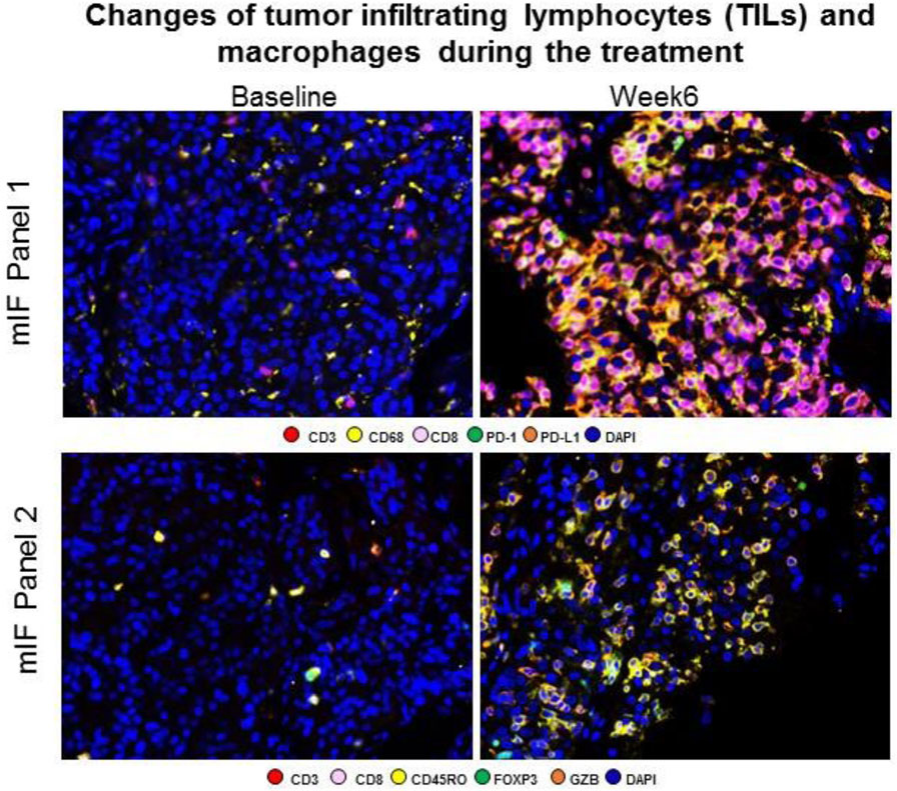
See text for description

**Fig. 3 (abstract P360).**
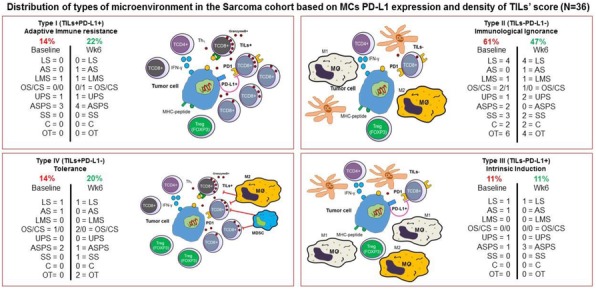
See text for description

#### P361 Molecular and immunologic profiling of CD8+ T cell responses in patients receiving a multiple antigen-engineered dendritic cell vaccine

##### Juraj Adamik, PhD^1^, Patricia Santos, PhD^2^, Samuel Du, BS^2^, Lazar Vujanovic, PhD^2^, Timothy Howes^1^, Sarah Warren, PhD^3^, Andrea Gambotto, MD^2^, John Kirkwood, MD^2^, Lisa Butterfield, PhD^1^

###### ^1^Parker Institute for Cancer Immunotherapy, San Francisco, CA, United States; ^2^University of Pittsburgh, Pittsburgh, PA, United States; ^3^NanoString Technologies, Seattle, WA, United States

####### **Correspondence:** Lisa Butterfield (lbutterfield@parkerici.org)

**Background**

Despite the immunogenicity and safety profile of dendritic cell (DC) vaccines, the importance of vaccine-induced antigen-specific T cell responses is unclear across clinical trials, and therapeutic efficacy remains low with limited clinical responses. Our comprehensive characterization of T cell responses, cell-intrinsic and soluble immune checkpoint molecules and immune-related gene expression profiles reveal novel insights into CD8+ T cells specific for melanoma-associated antigens (MAA) from patients who received autologous DC engineered to express three full length melanoma antigens: tyrosinase, MART-1 and MAGE-A6 [1].

**Methods**

MAA-specific T cell responses were examined by standardized IFNγ ELISPOT assays at baseline, day 43 (post DC vaccines) and d89 (post observation or IFNα). Luminex was used to detect serum checkpoint and costimulatory molecules, and whole blood flow cytometry was used to quantify PBMC subsets. Targeted mRNA and protein expression analyses in circulating lymphocytes and melanoma tumor samples were performed using NanoString nCounter platform (RUO).

**Results**

The majority of the 35 patients were successfully vaccinated, and the total vaccine-induced T cell responses were higher among those exhibiting a favorable clinical outcome. Patients who received checkpoint blockade treatment prior to DC vaccination had higher baseline MAA-specific CD8+ T cell responses, yet they did not respond more strongly to the vaccine. Two patients who received checkpoint blockade post-DC vaccine showed very strong amplification of their MAA-specific T cells. Molecular profiling in circulating lymphocytes and tumor biopsies showed that elevated PD-1 and CTLA-4 protein levels and gene expression signatures representing checkpoint signaling, interferon response and T-cell exhaustion were associated with unfavorable clinical outcome. Gene signatures showing positive correlation with PD-1 protein expression included CD28-dependent PI3K-AKT signaling, the IL12/STAT4 pathway and pan-semaphorin receptor interactions. CTLA-4 protein levels correlated with type I interferon response and NOTCH signaling genes. Interestingly, B cell receptor pathways negatively correlated with PD-1 expression, while gene signatures downstream of T cell receptor activation and IL-2 signaling were negatively correlated with CTLA-4 expression. Serum levels of PD-1 and PD-L2 were inversely correlated and post-vaccine serum levels PD-L2 correlated with decreased circulating Treg and favorable outcome in patients, suggesting that it may serve as a biomarker of clinical response.

**Conclusions**

Collectively, our study shows that specific checkpoint molecular pathways are critical for vaccine outcomes and for the activation of anti-tumor responses in melanoma patients. Comprehensive profiling of MAA-specific T cell responses suggests that DC-vaccine immunization followed by immune checkpoint blockade may be an optimal sequential therapy to improve antitumor immunity in melanoma.

**Trial Registration**

FDA IND #15044 and NCT01622933.

**Reference**

1. Butterfield LH, Vujanovic L, Santos PM, Maurer DM, Gambotto A, Lohr J, Li C, Waldman J, Chandran U, Lin Y, Lin H, Tawbi HA, Tarhini AA, Kirkwood JM. Multiple antigen-engineered DC vaccines with or without IFNa to promote antitumor immunity in melanoma. JITC. 2019; 7:113.

**Ethics Approval**

The clinical trial was fully approved by the Univ. Pittsburgh PRC and IRB (PRO12010416, #09–021).

#### P362 First-line avelumab treatment in patients with metastatic Merkel cell carcinoma: primary analysis after ≥15 months of follow-up from JAVELIN Merkel 200, a registrational phase 2 trial

##### Sandra D'Angelo, MD^1^, Celeste Lebbé^2^, Laurent Mortier^3^, Andrew Brohl, MD^4^, Nicola Fazio^5^, Jean-Jacques Grob^6^, Natalie Prinzi^7^, Glenn Hanna^8^, Jessica Hassel, MD^9^, Felix Kiecker^10^, Barbara Ellers-Lenz^11^, Marcis Bajars^12^, Meliessa Hennessy, MPH^12^, Paul Nghiem, MD, PhD^13^

###### ^1^Memorial Sloan Kettering Cancer Center, New York, NY, United States; ^2^Saint Louis Hospital, Paris, France; ^3^Lille Hospital–Claude Huriez Hospital, Lille Cedex, France; ^4^H. Lee Moffitt Cancer Center and Research Institute, Tampa, Florida, United States; ^5^European Institute of Oncology (IEO), IRCCS, Milan, Italy; ^6^Aix-Marseille University, AP-HM Hospital, Marseille, France; ^7^Fondazione IRCCS Istituto Nazionale dei Tumori, Milan, Italy; ^8^Dana-Farber Cancer Institute, Boston, MA, United States; ^9^Heidelberg University Hospital, Heidelberg, Germany; ^10^Charité Universitätsmedizin Berlin, Campus Charité Mitte, Berlin, Germany; ^11^Merck KGaA, Darmstadt, Germany; ^12^EMD Serono Research and Development Institut Inc, Billerica, MA, United States; ^13^University of Washington Medical Center at South Lake Union, Seattle, WA, United States

####### **Correspondence:** Sandra D'Angelo (dangelos@mskcc.org)

**Background**

Merkel cell carcinoma (MCC) is a rare, aggressive neuroendocrine carcinoma with a poor prognosis. In the pivotal phase 2 JAVELIN Merkel 200 trial (NCT02155647), avelumab, a human anti–PD-L1 monoclonal antibody, yielded durable responses in patients with metastatic MCC (mMCC) who had received prior chemotherapy (part A), and a high objective response rate (ORR) in an initial subgroup treated in the first-line metastatic setting (part B), leading to regulatory approval worldwide. Here we report the primary analysis of JAVELIN Merkel 200 part B after ≥15 months of follow-up in the full patient population.

**Methods**

Eligible patients had no prior systemic therapy for mMCC and were enrolled irrespective of biomarker status; PD-L1+ status was defined as ≥1% expression in tumor cells (PD-L1 IHC 73-10 pharmDx assay). All patients received avelumab 10 mg/kg IV every 2 weeks. The primary endpoint was durable response, defined as objective response (complete response [CR] or partial response [PR] per RECIST v1.1, adjudicated by independent endpoint review committee) lasting ≥6 months. Secondary endpoints included best overall response, duration of response (DOR), progression-free survival (PFS), overall survival (OS), and safety.

**Results**

At data cut-off on May 2, 2019, 116 patients had been treated with avelumab. Median treatment duration was 5.5 months (range, 0.5-35.4), and treatment was ongoing in 26 patients (22.4%). Median follow-up was 21.2 months (range, 14.9-36.6). The ORR was 39.7% (95% CI: 30.7%-49.2%), including 19 patients (16.4%) with a CR and 27 (23.3%) with a PR. In patients with PD-L1+ (n=21 [18.1%]) or PD-L1− (n=87 [75.0%]) tumors, ORRs were 61.9% (95% CI: 38.4%-81.9%) and 33.3% (95% CI: 23.6%-44.3%), respectively. Median DOR was 18.2 months (95% CI: 11.3 months-not estimable). 35 patients had a response lasting ≥6 months (durable response rate, 30.2% [95% CI: 22.0%-39.4%]). 6- and 12-month PFS rates were 41% (95% CI: 32%-50%) and 31% (95% CI: 23%-40%), respectively. Median OS was 20.3 months (95% CI: 12.4 months-not evaluable), and the 12-month OS rate was 60% (95% CI: 50%-68%). In PD-L1+ and PD-L1− subgroups, 12-month OS rates were 71% (95% CI: 47%-86%) and 56% (95% CI: 45%-66%), respectively. Treatment-related adverse events (TRAEs) of any grade occurred in 94 patients (81.0%), including grade ≥3 TRAEs in 21 (18.1%). No treatment-related deaths occurred.

**Conclusions**

Updated results from JAVELIN Merkel 200 confirm that first-line avelumab treatment was associated with durable responses, a clinically meaningful OS benefit, and an acceptable safety profile in patients with mMCC.

**Acknowledgements**

This study was funded by Merck KGaA, Darmstadt, Germany, as part of an alliance between Merck KGaA, Darmstadt, Germany and Pfizer Inc., New York, NY, USA.

**Trial Registration**

Registered at www.clinicaltrials.gov, NCT02155647

**Ethics Approval**

The trial was conducted in accordance with international good clinical practice standards and approved by the independent ethics committee at each participating center

#### P364 A gp100 targeting TCR-based soluble T cell engaging bispecific induces mobilisation and activation of peripheral T cells in patients with metastatic melanoma

##### Sion Lewis, BSc MSc PhD, Sion Lewis, BSc MSc PhD, Sion Lewis, BSc MSc PhD, Mariantonella Vardeu, Jacob Hurst, PhD, Cheryl McAlpine, MSN

###### Immunocore Ltd, Abingdon, United Kingdom

####### **Correspondence:** Sion Lewis (Sion.Lewis@immunocore.com)

**Background**

ImmTAC® (immune-mobilizing monoclonal TCRs Against Cancer) molecules are a new class of bispecific therapeutics, consisting of a high affinity TCR fused to an anti-CD3 single-chain variable fragment (scFv) T cell-activating moiety [1]. Tebentafusp (gp100 antigen specific ImmTAC) can elicit a polyfunctional T cell response and has demonstrated monotherapy activity in advanced metastatic melanoma [2-5]. Clinical benefit was associated with a reduction in peripheral CXCR3+ T cells and concurrent increase in serum CXCL-10 [6, 7]. The chemokine receptor CCR5 is understood to drive T cell extravasation and potentialy synsergise with CXCR3 to promote tumour infiltration [8, 9]. Therefore the aim of this study was to investigate the effect of tebentafusp on the dynamics of T cell mobilisation and activation in metastatic melanoma patients.

**Methods**

HLA-A2+ patients with metastatic melanoma were enrolled on a first-in-human, multicentre, Phase I/II, open-label, dose-finding study (NCT01211262). Immunophenotypic analysis was undertaken to assess baseline levels and pharmacodynamic changes in peripheral immune subsets. PBMC samples were analysed by flow cytometry from patients at baseline (n=38) and on-treatment (n=22) over the first dosing cycle, and from age/sex-matched healthy control subjects (n=18). Data is reported on T cell populations, markers of activation (CD25) and function (CCR5, Ki67) and represented as mean ± standard deviation of subset frequency or percentage change from baseline, relationships with overall survival (OS) assessed by univariate Cox proportional hazards model.

**Results**

Tebentafusp induced T cell extravasation within 24hrs (p

T cells from patients on-treatment exhibited an increase in activation marker expression and an expansion of memory and effector T cell subsets (p<0.05).

**Conclusions**

Tebentafusp administratation induces the rapid extravasation of chemokine receptor expressing T cells and the expansion and activation of memory T cells. The association between clinical benefit and baseline levels of peripheral immune subsets may aid our mechanistic understanding of its anti-tumour activity in metastatic melanoma patients.

**Trial Registration**

NCT01211262

**References**

1. Lowe KL, et al. Cancer Treat Rev. 2019.

2. Boudousquie C, et al. Immunology 2017;152:425–38.

3. Middleton M, et al. Presented at ASCO 2016. Abstract 3016.

4. Carvajal R, et al. Presented at SITC 2017. Abstract P208.

5. Middleton M, et al. Presented at ASCO 2019. Abstract 9523.

6. Middleton M, et al. Presented at ASCO 2019. Abstract 9530

7. Mullins et al, Cancer Res 2004; 64, 7697-7701

8. Hong M et al, Cancer Res 2011; 71, 22:6997-7009

9. Harlin, Cancer Res 2009;69(7):3077–85:

**Ethics Approval**

This study was approved by following institutions’ Ethics Boards:

- Oxfordshire Research Ethics Committee; 10/H0604/47, Approved June 4, 2010.
- Mary Crowley Cancer Research Center; MCMRC IRB # 12-06, Approved March 16, 2012.
- Human Investigation Committee, Yale University; HIC Protocol # 1302011504, Approved March 22, 2012.
- IntegReview; Protocol No IMCgp100/01, Approved November 13, 2013.
- Western Sydney Local Health District; HREC2012/7/4.1 (3552) AU RED HREC/12/WMEAD/237, Approved on October 24, 2012.
- Western Institutional Review Board; Panel 1, Study Num 1147687, WIRB Pro Num 20141184, Approved July 15, 2014.
- Memorial Sloan Kettering Cancer Center, Institutional Review Board; Protocol # 14-152, August 28, 2014.

#### P365 A trial to evaluate the immunogenicity and safety of a melanoma helper peptide vaccine plus incomplete Freund’s adjuvant, cyclophosphamide, and polyICLC (Mel63)

##### Craig Slingluff, MD^1^, Gina Petroni, PhD^1^, Kimberly Chianese-Bullock, PhD^1^, Nolan Wages, PhD^1^, Walter Olson, PhD^1^, Kelly Smith^1^, Lynn Dengel, MD^1^, Anna Dickinson^1^, Caroline Reed^2^, Elizabeth Gaughan, MD^1^, William Grosh, MD^1^, Varinder Kaur, MD^1^, Nikole Varhegyi^1^, Mark Smolkin^1^, Nadejda Galeassi^1^, Donna Deacon, BS^1^

###### ^1^University of Virginia, Charlottesville, VA, United States; ^2^Emory University School of Medicine, Atlanta, GA, United States

####### **Correspondence:** Craig Slingluff (CLS8H@hscmail.mcc.virginia.edu)

**Background**

Cancer vaccines require adjuvants to induce effective and durable protective immunity. However, there is no consensus on optimal vaccine adjuvants to support T cell responses to peptide vaccines. We hypothesized that toll-like receptor (TLR)3 agonist polyICLC and/or low-dose metronomic cyclophosphamide (mCy) would be safe and would support strong and durable CD4+ T cell responses in combination with an incomplete Freund’s adjuvant (IFA).

**Methods**

An adaptive design based upon toxicity and durable immune response (dRsp) was used to assign participants with resected stage IIA-IV melanoma to one of four study regimens, including a vaccine comprising 6 melanoma peptides restricted by Class II MHC (6MHP), administered in an emulsion with IFA (Montanide ISA-51), with or without the TLR3 agonist polyICLC and with or without systemic mCy. Toxicities were recorded (CTCAE v4). T cell responses were measured in peripheral blood lymphocytes (PBL) and in vaccine-site draining lymph node (sentinel immunized node, SIN) with IFNγ ELIspot assay ex vivo. Serum antibody responses to 6MHP were measured by ELISA, and changes in circulating regulatory T cells were assessed by flow cytometry.

**Results**

Forty-eight eligible patients were enrolled and treated. Following an adaptive design, early safety data and T cell response data favored enrollment on arm D. At study conclusion, total enrollment was 3, 7, 6, and 32 individuals for arms A-D, respectively. Treatment-related dose-limiting toxicities (DLTs) were observed in 1/7 (14%) patients on arm B and 2/32 (6%) on arm D, with no treatment arm exceeding the DLT 25% threshold for early stopping. Strong and durable T cell responses to 6MHP were detected ex vivo in 0%, 29%, 50%, and 50% of patients enrolled on arms A-D, respectively (Table 1). IgG antibody responses were also induced and were greatest for arms C and D (Figure 1). Circulating regulatory T cell frequencies were not altered by use of mCy.

**Conclusions**

Combination vaccine adjuvants with IFA, polyICLC, and mCy were well-tolerated. The dRsp rate for arm D (IFA + polyICLC + mCy) of 50% (90% CI: [34,66]) exceeded the 18% dRsp rate (90% CI: [11,26]) from prior experience with 6MHP in IFA alone. The regimen with IFA + pICLC alone also showed promise for enhancing T cell and antibody responses. Addition of mCy does not alter circulating T reg frequencies but shows some promise as a systemic vaccine adjuvant.

**Acknowledgements**

We thank the Cancer Research Institute/ Ludwig Institute for Cancer Research for providing the polyICLC used in the vaccines. Funding was provided by NCI R01 CA178846 (CLS); 5K25CA181638 (NW); P30 CA044579 (Biorepository and Tissue Research Facility, Office of Clinical Research, and Biostatistics Shared Resource).

**Trial Registration**

The clinical trial Mel63 is registered with Clinicaltrials.gov (NCT02425306).

**Ethics Approval**

The clinical trial Mel63 was performed with IRB (#17860) and FDA approval (IND #10825).

**Table 1 (abstract P365).**
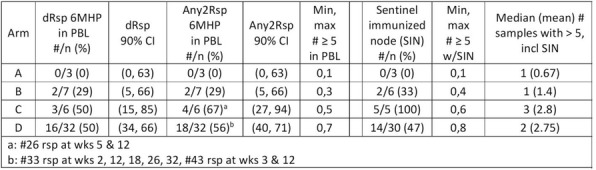
T cell responses to 6MHP

**Fig. 1 (abstract P365).**
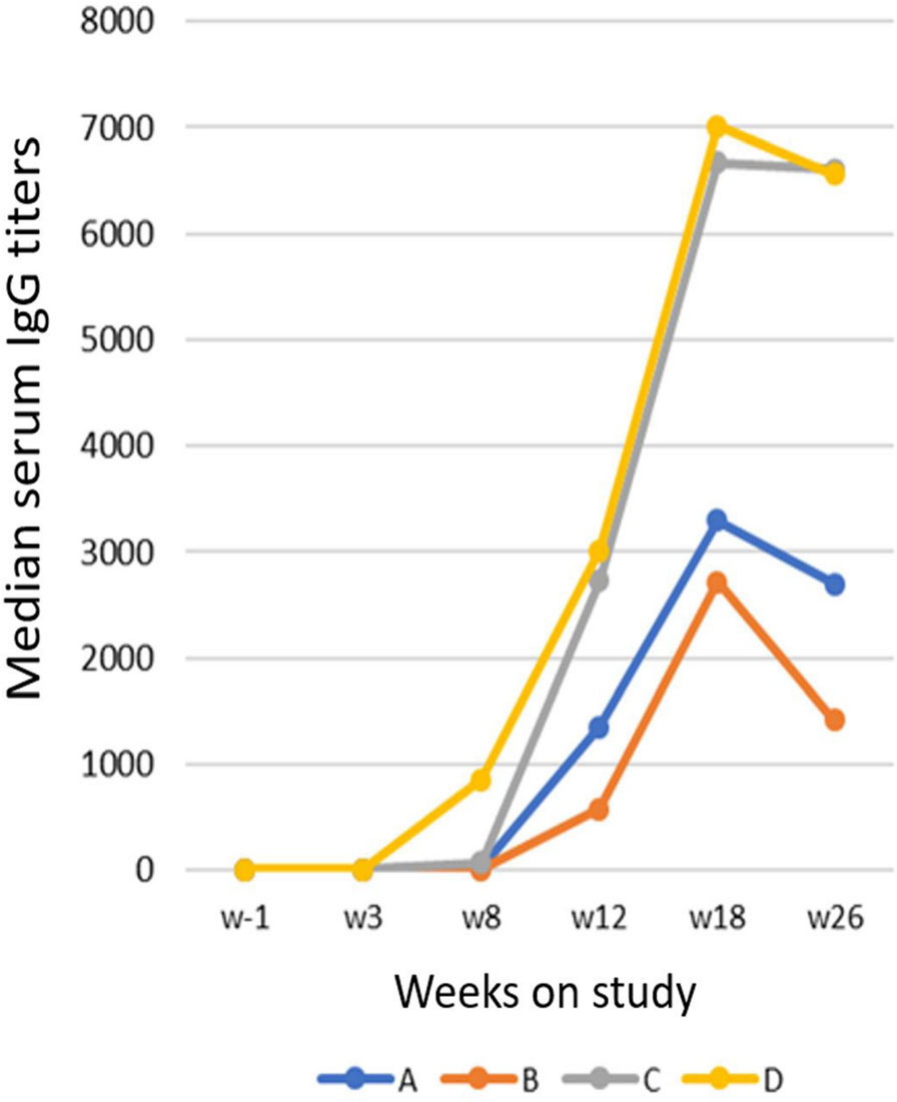
Antibody responses to 6MHP

#### P366 A phase 1 study of NY-ESO-1 vaccine + ipilimumab (ipi) in patients with unresectable or metastatic melanoma

##### Craig Slingluff, MD^1^, Hassane Zarour, MD^2^, Michael Postow, MD^3^, Philip Friedlander, MD PhD^4^, Craig Devoe, MD^5^, Ileana Mauldin, PhD^1^, Kelly Smith^1^, Mary Macri, BSc^6^

###### ^1^University of Virginia, Charlottesville, VA, United States; ^2^University of Pittsburgh Cancer Center, Pittsburgh, PA, United States; ^3^Memorial Sloan Kettering Cancer Center, New York, NY, United States; ^4^Mount Sinai Medical Center, New York, NY, United States; ^5^Northwell Health Cancer Institute, Lake Success, NY, United States; ^6^Ludwig Institute for Cancer Research, New York, NY, United States

####### **Correspondence:** Craig Slingluff (CLS8H@hscmail.mcc.virginia.edu)

**Background**

Ipilimumab (IPI) is an approved immunotherapy for advanced melanoma. It can enhance immunity to cancer-testis antigen NY-ESO-1. Vaccines with NY-ESO-1 protein or NY-ESO-1 overlapping long peptides (OLP4) have enhanced immunity when administered with Montanide ISA-51 (Montanide) and/or Poly-ICLC (pICLC) adjuvants. This trial assessed safety, immunogenicity, clinical responses (irRC), and effects of IPI + NY-ESO-1 vaccines on the tumor microenvironment (TME).

**Methods**

This Phase 1, open-label study enrolled patients among 3 arms: IPI (3 mg/kg i.v. q3 wks x 4) + NY-ESO-1 protein + pICLC + Montanide (Arm A); IPI + NY-ESO-1 OLP4 + pICLC + Montanide (Arm B); and IPI + NY-ESO-1 OLP4 + pICLC (Arm C). Patients had measurable NY-ESO-1+ tumors. Treatments were administered days 1, 22, 43, 64. Circulating T cell responses were assessed by ex vivo IFN-gamma ELIspot assay. Circulating antibody (Ab) responses to overlapping NY-ESO-1 peptides were detected by ELISA. Tumor biopsies obtained pre-treatment and day 85 were evaluated for immune infiltrates by multispectral immunofluorescence histology.

**Results**

Target enrollment was 27; study closed early for slow enrollment. Eight patients enrolled and were treated (Table 1). All had ≥ 1 treatment emergent adverse event (TEAE); most common (≥50%): rash, fatigue, injection site reaction, pruritus, and diarrhea. Two patients had Gr3 TEAEs related to IPI but not to vaccine. There were no DLTs. Best responses: SD (n=4); PD (n=4). T cell responses to NY-ESO-1 were detected in 6 of 8 (75%) patients.[1] Both patients without T cell response had PD as best response. Ab responses were detected in 7/8 (88%) patients (Table 1). The patient without Ab response had PD as best response. The breadth of Ab responses to NY-ESO-1 was greater for patients with SD than those with PD (p = 0.02). Evaluation of TME of 5 patients revealed increases in proliferating (Ki67+) CD8 T cells, decreases in RORγt+ CD4+ T cells (Figure 1). Interestingly, there were increases in density of CD8+ and CD4+ cells for those with SD (n=3), but decreases for those with PD (n=2, not shown).

**Conclusions**

T cell responses and Ab responses to NY-ESO-1 were induced in most patients and were evident ex vivo, suggesting that IPI may have enhanced the T cell responses to NY-ESO-1 protein and OLP4. Integrated T cell and antibody responses were associated with tumor control. Preliminary data of the TME suggests increased activating and proliferating T cells after vaccination plus IPI, especially in patients with tumor control.

**Acknowledgements**

The trial was supported by the Ludwig Institute for Cancer Research, the Cancer Research Institute, and by the National Institutes of Health, including support from the University of Virginia Cancer Center Support Grant (NIH/NCI P30 CA44579:, Clinical Trials Office, Biorepository and Tissue Procurement Facility, Flow Cytometry Core, and Biomolecular Core Facility).

Earlier presentation of results of this clinical trial [1] has been expanded in the present abstract with additional biologic correlates.

**Trial Registration**

This trial was registered at ClinicalTrials.gov (NCT01810016).

**Reference**

1. Slingluff CL Jr, Zarour HM, Postow MA, Friedlander P, Devoe CE, Macri M, Ryan A, Venhaus R, Wolchok J. J Clin Oncol. 2018; 36(suppl; abstr e15175).

**Ethics Approval**

The study was approved by each institution's Ethics Board, with approval numbers: IRB#12-253 (Memorial Sloan Kettering), HS#13-00471 (Mount Sinai), IRB#14-133B (Northwell Health), MOD13030240-02/PRO13030240(University of Pittsburgh), and HRS#16347(University of Virginia), and to the FDA with IND 10369.

**Table 1 (abstract P366).**
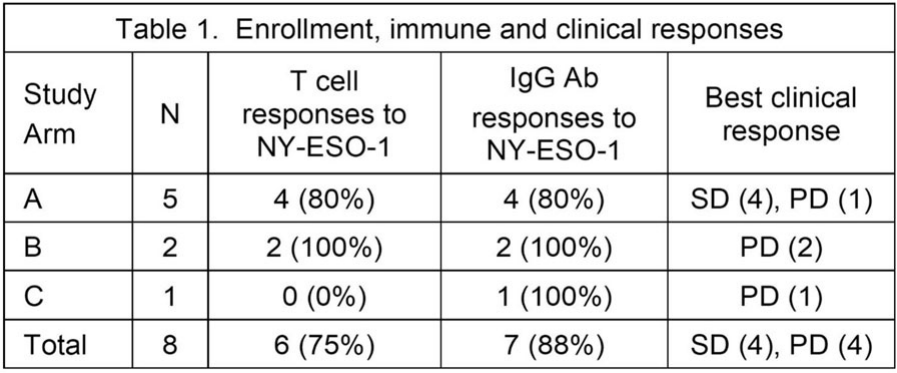
Enrollment, immune and clinical responses

**Fig. 1 (abstract P366).**
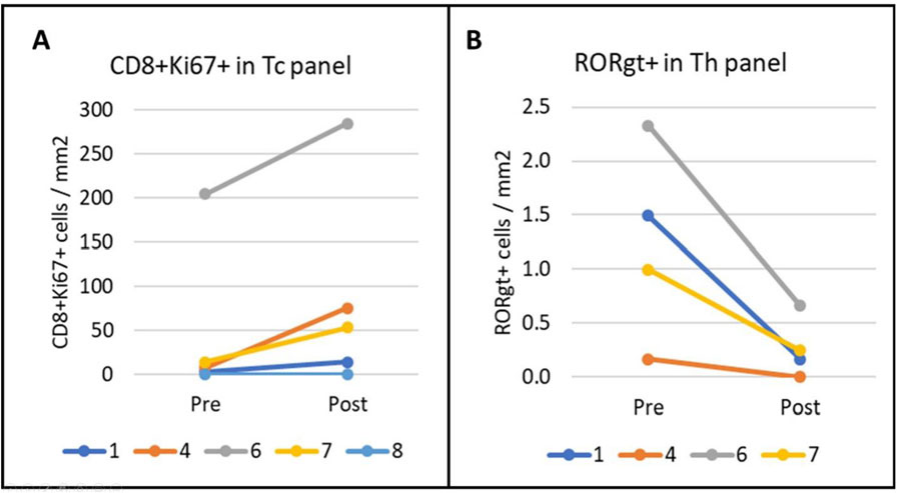
See text for description

#### P367 A multicenter, double-blind, placebo-controlled trial of seviprotimut-L polyvalent melanoma vaccine in post-resection melanoma patients at high risk of recurrence

##### Craig Slingluff, MD^1^, Brent Blumenstein, PhD^2^, Karl Lewis, MD^3^, Robert Andtbacka, MD, CM, FACS, FRCSC^4^, John Hyngstrom, MD^5^, Mohammed Milhem, MBBS^6^, Svetomir Markovic, MD, PhD^7^, Omid Hamid, MD^8^, Leonel Hernandez-Aya, MD PhD^9^, Tawnya Bowles, MD^10^, Prejesh Philips, MD^11^, Joel Claveau, MD^12^, Sekwon Jang, MD^13^, Jose Lutzky, MD, FACP^14^, Anna Bar, MD^15^, Peter Beitsch, MD^16^

###### ^1^University of Virginia, Charlottesville, VA, United States; ^2^Tri Arc Consulting, Washington, DC, United States; ^3^University of Colorado, Aurora, CO, United States; ^4^Seven and Eight Biopharmaceuticals, Salt Lake City, UT, United States; ^5^Huntsman Cancer Institute/ Univ of Utah, Salt Lake City, UT, United States; ^6^University of Iowa Hospitals and Clinics, Iowa City, IA, United States; ^7^Mayo Clinic Rochester, Rochester, MN, United States; ^8^The Angeles Clinic & Research Institute, Los Angeles, CA, United States; ^9^Washington University School of Medicine, Saint Louis, MO, United States; ^10^Intermountain Medical Center, Murray, UT, United States; ^11^University of Louisville, Louisville, KY, United States; ^12^CHU de Quebec, L'Hotel Dieu de Quebec, Quebec, Canada; ^13^Inova Melanoma and Skin Center, Fairfax, VA, United States; ^14^Mount Sinai Medical Center, Miami Beach, FL, United States; ^15^Oregon Health and Science University, Portland, OR, United States; ^16^Cancer Solutions, Dallas, TX, United States

####### **Correspondence:** Craig Slingluff (CLS8H@hscmail.mcc.virginia.edu)

**Background**

Seviprotimut-L is a vaccine prepared from antigens shed by 3 human melanoma cell lines, administered with alum. Prior formulations showed promising immunogenicity for T cell and antibody responses and improved survival in a small phase II clinical trial[1]. Part B1 of MAVIS (Melanoma Antigen Vaccine Immunotherapy Study, a three part, Phase III clinical program), was a multicenter, double-blind, placebo-controlled trial to assess the efficacy of seviprotimut-L, with the primary endpoint of relapse-free survival (RFS) in patients at high risk of recurrence after definitive surgical resection.

**Methods**

For MAVIS Part B1, patients with AJCC v7 stage IIB-III cutaneous melanoma, after surgical resection, age 18-75, ECOG PS 0-1, were randomized 2:1 to seviprotimut-L 40 mcg or placebo, administered subcutaneously every 2 weeks x 5, then monthly x 4, then every 3 months to month 24. Patients were stratified by stage (IIB/C, IIIA, IIIB/C). Target enrollment was 325. The study was powered for assessment of RFS, with target hazard ratio (HR) of 0.625, one-sided alpha of 0.10, and power 80%.

**Results**

347 patients were randomized, and arms were well-balanced. Treatment-emergent adverse events (AEs) were similar for seviprotimut-L and placebo patients (Table 1). By intent-to-treat (ITT) analysis, RFS was not significantly enhanced for seviprotimut-L in the full study population, but trended slightly higher (Figure 1A). Analysis of subgroups based on pre-planned stratification suggested enhanced RFS for seviprotimut-L among Stage IIB/IIC patients (HR 0.59, 95% CI[0.33,1.07], Figure 1B). Age has been identified as a cause of decreased immune competence[2]; thus, outcomes were assessed as a function of age as an effect modifier. Figures 1C and 1D show all randomized patients (Figure 1C) and Stage IIB/IIC subset (Figure 1D) by arm and age split at

**Conclusions**

Seviprotimut-L is very well tolerated. Subgroup efficacy analyses identified two populations who may benefit from Seviprotimut-L: those with AJCC stage IIB/IIC melanoma and those under age 60. These data support proceeding to the definitive final part of the MAVIS phase III trial testing seviprotimut-L for stage IIB/C patients, in particular those under age 60.

**Acknowledgements**

We acknowledge the support of all investigators and clinical coordinators responsible for enrolling patients to this trial.

**Trial Registration**

This trial was registered at ClinicalTrials.gov: NCT01546571.

**References**

1. Bystryn JC, Zeleniuch-Jacquotte A, Oratz R et al. Double-blind trial of a polyvalent, shed-antigen, melanoma vaccine.[see comment]. Clinical Cancer Research 2001; 7: 1882-1887.

2. Dorshkind K, Swain S. Age-associated declines in immune system development and function: causes, consequences, and reversal. Curr Opin Immunol 2009; 21: 404-407.

**Ethics Approval**

The study was approved by the Ethics Board at each participating institution (IRB#), as follows: University of Virginia Hospital (16223); Anschutz Cancer Pavilion, UC Denver(1134601); Huntsman Cancer Institute, / Univ of Utah Health Care (55911); University of Iowa Hospitals and Clinics (1133782); Mayo Clinic Cancer Center / Mayo Clinic Rochester (12-002308); The Angeles Clinic and Research Institute (1196134); Washington University School of Medicine (201205056); Intermountain Medical Center (1024288); University of Louisville (15.0039); CHU de Quebec, L'Hotel Dieu de Quebec (MP-20-2015-2318); Inova Melanoma and Skin Cancer Center (1152774); Mount Sinai Medical Center (12-16-H-03); Oregon Health and Science University (IRB00011848); Cancer Solutions (1197259).

**Table 1 (abstract P367).**
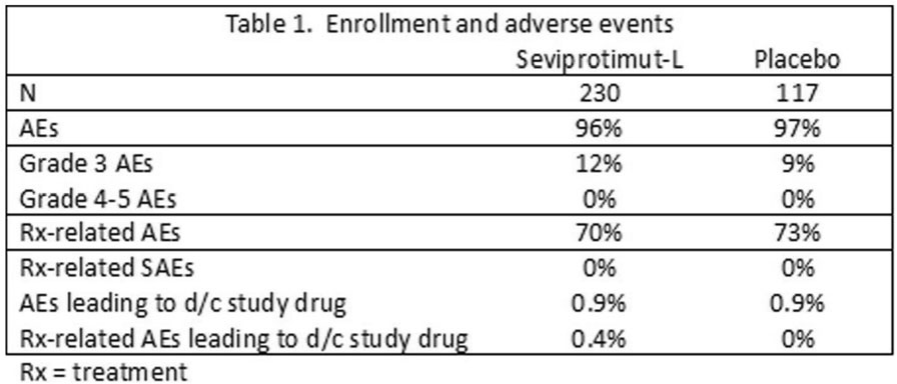
Enrollment and adverse events

**Fig. 1 (abstract P367).**
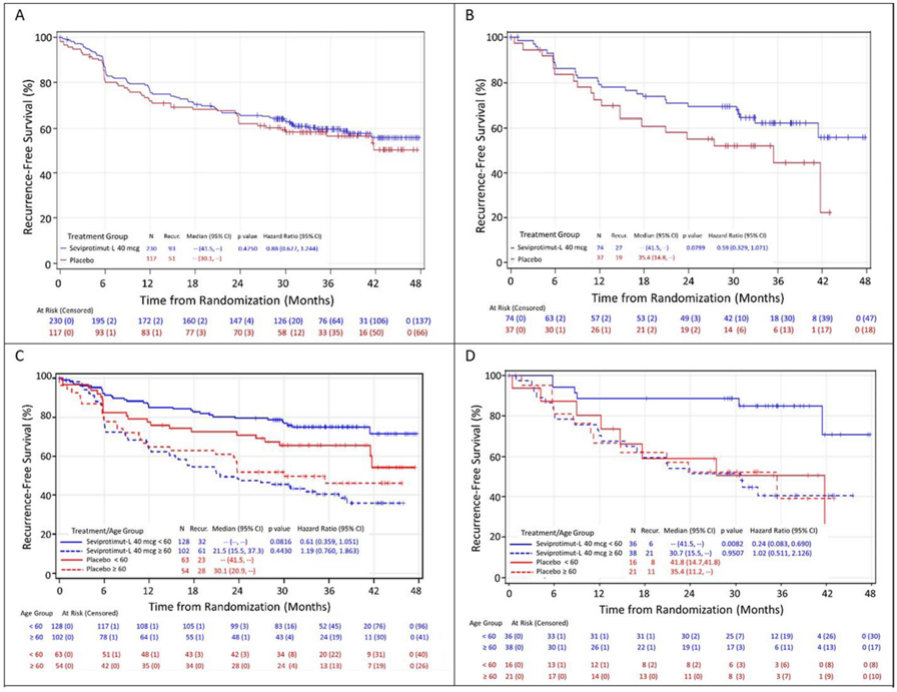
Clinical outcome

### Clinical Trial In Progress

#### P368 ZI-H04 - A novel MHC class II restricted TCR based cellular therapy targeting hTERT to treat solid tumours

##### Jens-Peter Marschner, MD, Mona Welschof, PhD, Miguel Forte, Eva Kristine Klemsdal, Sylvie Pollmann, Namir Hassan

###### Zelluna, Seeheim-Jugenheim, Germany

####### **Correspondence:** Jens-Peter Marschner (jenspeter.marschner@zelluna.com)

**Background**

Chimeric Antigen Receptor T-cells (CAR-T) are highly effective in the treatment of some hematological malignancies but solid tumors remain a challenge for cellular therapies. A few T-cell Receptor T-cells (TCR-T) have been investigated in solid tumors. To our knowledge, there is only one published study and one case report using MHC Class II restricted TCRs targeting MAGE-A3 and NY-ESO-1, respectively [1,2].

**Methods**

ZI-H04 represents a novel approach of TCR based therapies. Autologous T-cells from patients are genetically modified by lentiviral transduction to express the TCR targeting hTERT in the context of the MHC Class II allele, HLA-DPB1*04:01. The TCR was isolated from a pancreatic cancer patient who experienced clinical benefit following a peptide-based cancer vaccination against hTERT [3]. The TCR clone responded to autologous tumor and preclinical data demonstrate that ZI-H04 exhibits high sensitivity to hTERT peptide as well as recognition of processed antigen. Furthermore, specificity analysis supports the safety of the TCR-T. The restricted combined expression of hTERT plus HLA class II on normal cells limits the potential for on-target off tumor toxicity. A first-in-human study is designed to treat patients with relapsed/refractory solid tumors lacking an option of further treatments. Patients must be tested positive for HLA-DPB1*04:01 and the tumors must express hTERT. Adequate organ function and lab parameters are required. CNS involvement, autoimmune diseases, infections and immunosuppressive medication are main exclusion criteria. Primary objectives are safety and tolerability. Part 1 of the study, starting in 2020, will be a dose finding part, Part 2 a dose extension part with 5 cohorts. Prior to adoptive cell infusion patients will receive a low dose conditioning regimen consisting of 2 x 600 mg/m² cyclophosphamide followed by 3 x 25 mg/m² fludarabine. Patients will be observed for safety, efficacy and exploratory biomarkers.

**Conclusions**

ZI-H04 is a novel TCR-T with potentially favourable characteristics. The TCR was isolated from an hTERT vaccinated pancreatic cancer patient that experienced clinical benefit. A lower probability of off-target activity is expected since no engineering was done to the TCR. The MHC Class II restriction provides the possibility to induce a multi-pronged immune response including antigen spreading as demonstrated in a case report using an MHC Class II TCR-T in melanoma [2]. More than 50% of patients are HLA-DPB1*04:01 positive and the expression rate of hTERT is >80% in many tumors. Therefore, a substantial population may benefit from ZI-H04 treatment.

**References**

1. Lu Y-C, Parker LL, Lu T, et al. Treatment of patients with metastatic cancer using a major histocompatibility complex class II-restricted T-cell receptor targeting the cancer germline antigen MAGE-A3. J Clin Oncol. 2017;35:3322-3329

2. Hunder NN, Wallen H, Cao J, et al. Treatment of metastatic melanoma with autologous CD4+ T cells against NY-ESO-1. N Engl J Med. 2008;358:2698-703

3. Bernhardt SL, Gjertsen MK, Trachsel S, et al. Telomerase peptide vaccination of patients with non-resectable pancreatic cancer: a dose escalating phase I/II study. Br J Cancer. 2006;95:1474-1482

#### P369 Feasibility of a phase I personalized adoptive T-cell therapy in patients with relapsed/refractory solid tumors

##### Apostolia Tsimberidou, MD, PhD^1^, Ali Mohamed^3^, Stephen Eck, MD, PhD^4^, Harpreet Singh^4^, Patrick Hwu, MD^1^, Cassian Yee, MD^1^, Borje Andersson, MD, PhD^1^

###### ^1^MD Anderson Cancer Center, Houston, TX, United States; ^2^MD Anderson, Houston, TX, United States; ^3^Immatics Biotechnology, Tubingen, Germany; ^4^Immatics, Houston, TX, United States

####### **Correspondence:** Apostolia Tsimberidou (atsimber@mdanderson.org)

**Background**

Adoptive cellular therapy (ACT) is limited in solid tumors due to lack of suitable immunotherapy targets with high specificity and frequent relapse following immunotherapy to single targets often associated with loss of target expression in the tumor. ACTolog® is a personalized, multi-targeted ACT approach in which autologous T-cell products are manufactured against the most relevant tumor target peptides for individual patients whose tumors are positive against predefined targets.

**Methods**

Patients with advanced metastatic cancers and HLA-A*02:01 phenotype, undergo a tumor biopsy. Patients whose tumors express >1 of 8 cancer targets undergo leukapheresis. Autologous T cells are primed against the expressed ACTolog targets in the presence of IL-21 followed by HLA tetramer-guided cell sorting and rapid expansion. Patients who meet criteria for treatment receive lymphodepletion with Fludarabine 40 mg/m2 i.v. and Cyclophosphamide 500 mg/m2 i.v. (Days, -6 to -3). T-cells are infused on Day 0, followed by low-dose of IL-2 for 14 days (www.clinicaltrials.gov NCT02876510).

**Results**

From 7/2017 to 7/2019, 203 patients signed an informed consent to participate in the study, 91 had HLA-A*02:01 phenotype, 52 had a tumor biopsy and 34 patients underwent leukapheresis. To date, 9 patients have received treatment (median age, 38 yrs; range, 25-58 yrs; 2 men and 7 women; breast cancer, 3; sarcoma, 3; ovarian cancer, 1; nasopharyngeal, 1; anal carcinoma, 1; median time from diagnosis 4 years, range, 2-18 years; median number of prior therapies 6, range 3-12). Very high ACTolog cell doses could be administered. Patients received a median of 2 target-specific ACTolog products (range 1-3). Treatment was overall well tolerated. The most common adverse events were cytopenias and cytokine release syndrome. All patients are alive to date. At 6 weeks, restaging imaging studies demonstrated stable disease in all patients. One patient with squamous cell carcinoma of the anus treated with T cells directed to COL6A3, exon 6, and PRAME had 26% decrease in tumor measurements at week 6 associated with high T-cell frequencies at 2 weeks but her disease subsequently progressed. Another patient with nasopharyngeal cancer treated with COL6A3 tumor stroma-specific T cells had resolution of tumor associated pain and has not required further treatment for 11 months. A recent tumor biopsy demonstrated necrotic cells and no tumor cells could be identified.

**Conclusions**

ACTolog IMA101 is well-tolerated and no safety issues have been noted to date. The study is ongoing.

**Trial Registration**

www.clinicaltrials.gov NCT02876510

**Ethics Approval**

The study was approved by MD Anderson's IRB.

**Consent**

Written informed consent was obtained from the patient for publication of this abstract and any accompanying images. A copy of the written consent is available for review by the Editor of this journal.

#### P370 The positive correlation between baseline absolute eosinophil count (AEC) in blood and clinical benefit to PD-(L)1 inhibition monotherapy

##### Anna Szpurka, PhD^1^, Danni Yu, PhD^1^, Michelle Carlsen^1^, Antoine Hollebecque, MD^2^, Hyun Cheol Chung, MD, PhD^3^, Amita Patnaik^4^, Johanna Bendell, MD^5^, Antoine Italiano, MD^6^, Yung-Jue Bang, MD PhD^7^, Chia-Chi Lin, MD, PhD^8^, Marcus Butler, MD^9^, Timothy Yap, MD PhD^10^, María José de Miguel, MD^11^, María José de Miguel, MD^11^, Jean-Pascal Machiels, MD, PhD^12^, Marc Peeters, MD, PhD^13^, Wu-Chou Su, MD^14^, Victor Moreno, MD^15^, Yumin Zhao, PhD^1^, Erik Rasmussen, PhD^1^, Xiaojian Xu, MD^1^

###### ^1^Eli Lilly and Company, Indianapolis, IN, United States; ^2^University of Paris Sud, Villejuif, France; ^3^Yonsei University College of Medicine, Seoul, Korea, Republic of; ^4^South Texas Accelerated Research Therape, San Antonio, TX, United States; ^5^Sarah Cannon Research Institute, Nashville, TN, United States; ^6^Institut Bergonié, Bordeaux, France; ^7^Seoul National University Hospital, Seoul, Korea; ^8^National Taiwan University Hospital, Taipei, Taiwan, Province of China; ^9^Princess Margaret Cancer Center, Toronto, Canada; ^10^The University of Texas, Houston, TX, United States; ^11^START-HM Sanchinarro, Madrid, Spain; ^12^jean-pascal.machiels@uclouvain.be, Brussels, Belgium; ^13^Antwerp University Hospital,, Antwerp, Belgium; ^14^National Cheng Kung University Hospital, Tainan, Taiwan, Province of China; ^15^START Madrid-FJD, Madrid, Spain

####### **Correspondence:** Danni Yu (yu_danni@lilly.com)

**Background**

Anti-PD-(L)1 immunotherapies have increased the response rate in certain cancer subtypes however, some patients who may have clinical benefit are not identifiable with existing predictive biomarkers. Research is ongoing to identify routinely available blood and clinical markers to predict response to PD-(L)1 therapies. In this study, we explored absolute eosinophil count (AEC) as a biomarker in patient’s response to PD-(L)1 treatment.

**Methods**

This is a phase 1a/1b study of an anti-PD-L1 antibody (LY3300054) administered alone or in combination with other agents in patients with advanced refractory solid tumors. Eligible patients were ≥18 years old, had ECOG status ≤1 and had at least 1 measurable lesion per RECIST v1.1. We assessed the association of AEC with confirmed best overall response (BORc). The AEC cutoff 0.155 (10^9/L) maximized the difference in ORR, similar to previous reports (Tanizaki etal, 2018, JTO [13] e85-e86) . The impact of AEC status was demonstrated by a 3-dimensional waterfall plot depicting the best change in tumor size and overall survival (OS).

We also tested the correlation between AEC and OS/proxy-progression-free survival (PFS; time to next treatment, TTNT) in NSCLC patients (n=455) who received anti-PD-1 therapy with same cutoff from an independent Flatiron database.

**Results**

As of 8 December 2017, 30 patients (MSI-H: n=22, M: n=8) were treated. There were no deaths due to adverse events. Two patients in MSI-H cohort experienced grade 3 treatment-related AEs (TRAEs): diarrhea (n=1, 4.5%), blood creatinine phosphokinase increased (n=1, 4.5%), and hyponatraemia (n=1, 4.5%). No grade 3 events were reported in M cohort, and no grade 4/5 TRAEs were reported in either cohorts. There were no TRAEs leading to discontinuation of study treatment. Preliminary efficacy data in MSI-H cohort showed ORR of 36% [CR in 1 pt (5%)(ovarian), PR in 7 pts (32%)(small intestine adenocarcinoma [1 pt], endometrial [3 pts], colon [3 pts])], DCR in 64% [SD in 6 pts (27%)]; mPFS was 7.39 months (95% CI 1.7, NR). In the M cohort, DCR was 63% [PR in 1 pt (13%), SD in 4 pts (50%)]. As of data cut-off, 16 pts (53%) remain on treatment. Preliminary biomarker analysis, including but not limited to, PD-L1 and CD8 expression and circulating markers will be presented.

**Conclusions**

LY3300054 was well-tolerated and demonstrated antitumor activity in patients with MSI-H solid tumors; combination expansions are ongoing.

**Trial Registration**

NCT02791334

**Reference**

Tanizaki, Junko, Koji Haratani, Hidetoshi Hayashi, Yasutaka Chiba, Yasushi Nakamura, Kimio Yonesaka, Keita Kudo, et al. "Peripheral Blood Biomarkers Associated with Clinical outcome in Non–Small Cell Lung Cancer Patients Treated with Nivolumab." Journal of Thoracic Oncology 13, no. 1 (2018/01/01/ 2018): 97-105.

**Ethics Approval**

The study included multiple investigator sites, and some of these had approval dates instead of approval numbers. The following are listed showing approving committee followed by approval date or approval # (if available): IntegReview, 15Jun2016; IntegReview, 21June2016; MD Anderson Office of Protocol Approval IRB, 26May2016; Princess Margaret Cancer Centre, University Health Network Research Ethics Board, 16-5824 (initial approval date 01Feb2017); Comite de protection des Personnes (CPP), EudraCT number 2016-000440-33.

**Fig. 1 (abstract P370).**
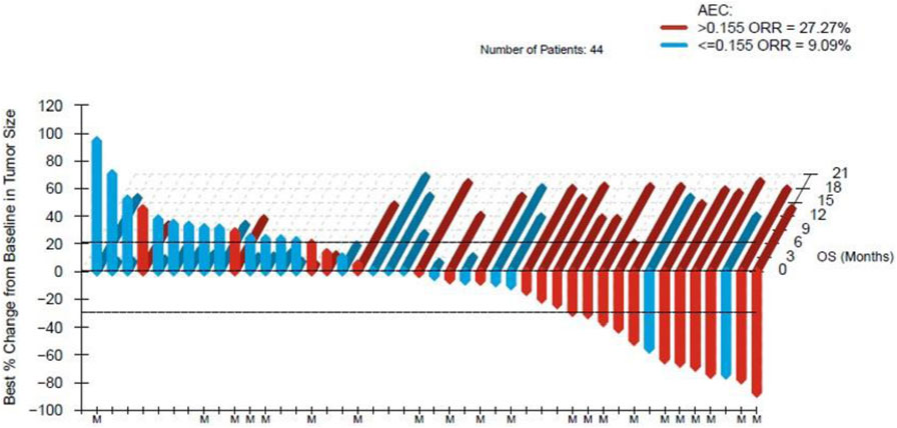
See text for description

#### P371 Cytokine Microdialysis for real-time immune monitoring in Glioblastoma patients undergoing Checkpoint Blockade

##### John Lynes, MD^1^, Victoria Sanchez^2^, Anthony Nwankwo^2^, Gifty Dominah^2^, Xiang Wang, MS^2^, Isac Kunnath^2^, Samantha Dill^2^, Gretchen Scott^2^, Christi Hayes^2^, Tianxia Wu^2^, Marta Penas-Prado^2^, Jing Wu^2^, Eric Burton^2^, John Heiss^2^, Christopher Hourigan, MD, PhD^2^, Mark Gilbert^2^, Edjah Nduom, MD^2^

###### ^1^Georgetown University, Bethesda, MD, United States; ^2^National Institutes of Health, Bethesda, MD, United States

####### **Correspondence:** Edjah Nduom (edjah.nduom@nih.gov)

**Background**

Glioblastoma is the most common primary malignancy of the brain, with a dismal prognosis. Immunomodulation via checkpoint inhibition has provided encouraging results in non-CNS malignancies, but prediction of responders has proven to be challenging in glioblastoma patients. OBJECTIVES: To determine the proportion of patients who have a measurable increase of interferon gamma levels in brain tumor tissue after their first dose of nivolumab; to evaluate the safety of using brain tumor microdialysis to monitor for immune response; to evaluate the safety of the combination of anti-programmed death 1 (PD-1) and anti-lymphocyte activation gene 3 (LAG-3) checkpoint inhibition in recurrent glioblastoma patients.

**Methods**

The study design is a single-center, nonrandomized phase 1 clinical trial. Up to 20 adult patients with recurrent glioblastoma will be enrolled with the goal of 10 patients completing the trial over an anticipated 18 months. Patients will undergo biopsy; placement of microdialysis catheters and lumbar drains; treatment with anti-PD-1 checkpoint inhibition; comprehensive immune biomarker collection; tumor resection; and then treatment with anti-PD-1 and anti-LAG-3 checkpoint inhibition until progression (Figure 1). Three patients have undergone all study procedures (Figures 2 and 3). There have been no serious adverse events related to the research surgical procedure, nor during the microdialysis portion of the trial. Enrollment is ongoing.

EXPECTED OUTCOMES: We expect interferon gamma levels to increase in the brain as measured via microdialysis in treated patients. Based on published reports, microdialysis in this patient population is expected to be safe, and anti-LAG-3 and anti-PD-1 combined will likely have a similar side effect profile to other checkpoint inhibitor combinations.

The failure of recent trials of immune therapies in glioblastoma underscores the need to appropriately measure response in the treated tissue. This trial may provide insight on indicators of which patients will respond to immune therapy.

**Acknowledgements**

Funding and support came from: Intramural Research Program of the National Institute of Neurological Disorders and Stroke

**Trial Registration**

Clinicaltrials.gov: NCT03493932 (Registration Date: April 11, 2018)

**Ethics Approval**

Institutional Approvals: National Institutes of Health Combined Neurosciences Internal Review Board number - 18-N-0077

**Fig. 1 (abstract P371).**
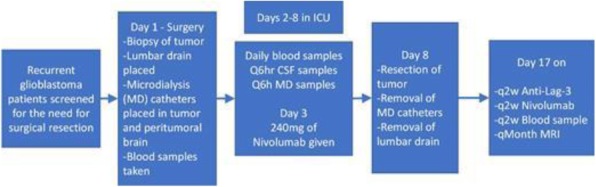
See text for description

**Fig. 2 (abstract P371).**
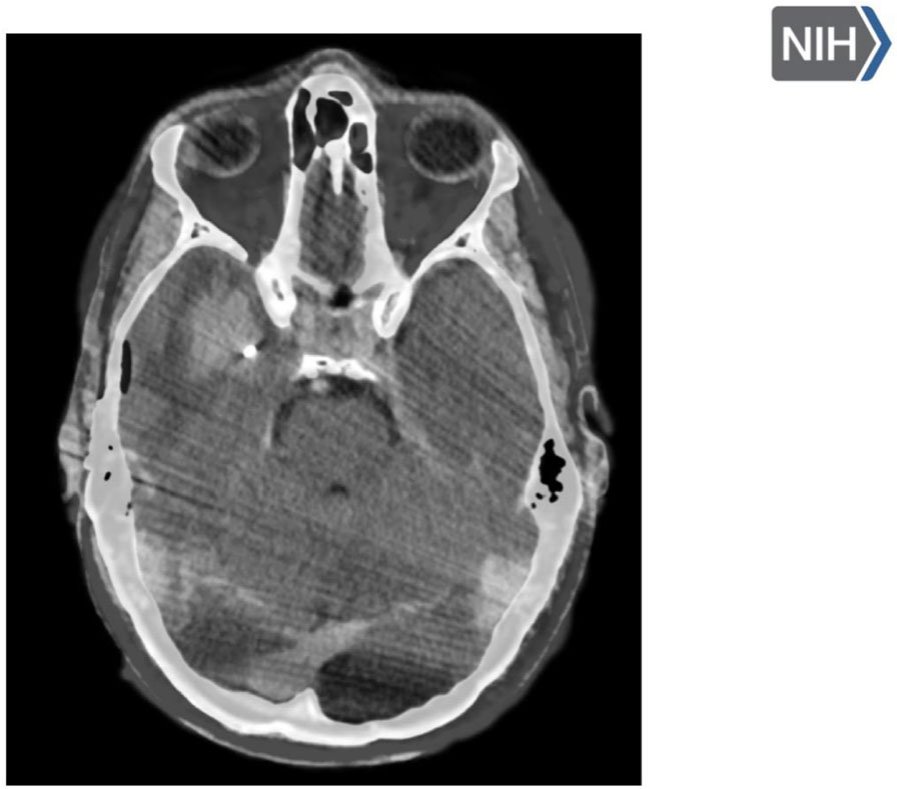
See text for description

**Fig. 3 (abstract P371).**
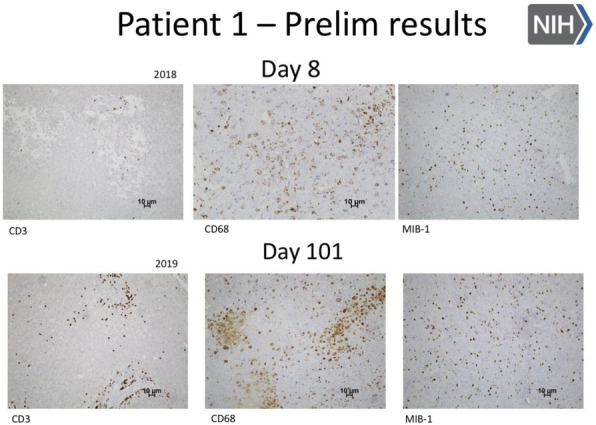
See text for description

#### P372 Circulating immune cell biomarkers predict response to immune checkpoint inhibitor therapy in metastatic breast cancer

##### Jin Sun Bitar, MD, Colt Egelston, PhD, Susan Yost, Wanqiu Hou, Padam Simran, Paul Frankel, PhD, Mina Sedrak, Jana Portnow, MD, Joanne Mortimer, MD, Christina Yeon, MD, Arti Hurria, MD, Aileen Tang, Norma Martinez, Peter Lee, MD, Yuan Yuan

###### City of Hope, Duarte, CA, United States

####### **Correspondence:** Yuan Yuan (yuyuan@coh.org)

**Background**

The role of immune checkpoint PD-1/PD-L1 inhibitor (ICI) in breast cancer (BC) is being investigated in clinical trials. Preclinical evidence strongly supports the synergistic effects of CDK4/6 inhibitor and ICI [1]. A phase II trial is testing the safety and efficacy of the combination of letrozole, palbociclib and pembrolizumab in patients with hormone receptor positive (HR+) BC (NCT02778685). Currently, there is no well-defined circulating biomarker to predict response to ICI.

**Methods**

Peripheral blood mononuclear cells (PBMC) were collected at day 1 of cycles 1 (pre-treatment), 2, 4, 6 and 8. The comprehensive characterization of circulating immune cell composition was performed using 15-color flow cytometry.

**Results**

Preliminary analysis included 9 patients with the following responses by RECIST 1.1: 1 complete response, 4 partial response, 2 stable disease, and 2 progressive disease.

Higher baseline frequencies of CD4+ effector memory (p=0.01) and CD8+ CD45RA+ effector memory cells (p=0.01) were observed in patient responders. Additionally, patient responders demonstrated higher frequencies of T cells expressing KLRG1, a marker of effector T cell differentiation, on both CD4+ (p=0.001) and CD8+ T cells (p=0.004) at baseline. An increase in the frequency of circulating CXCR5+ CD8+ T cells (p=0.01) at cycle 2 was identified in all treated patients and an increase in CCR10+ CD8+ T cells (p=0.03) was detected at cycle 2 in patient responders indicating changes in T cell trafficking. Finally, a shift in myeloid cell composition from predominantly classical to non-classical monocytes was observed in patient responders between baseline and cycle 2 (p=0.007).

**Conclusions**

High baseline levels of both CD4+ and CD8+ effector T cells indicate the necessity for a pre-existing favorable T cell composition in checkpoint blockade responders. Temporal changes in T cell trafficking molecules and shifts in myeloid cell composition over the course of therapy indicate potential changes in T cell priming and regulation. Further analysis is currently ongoing to understand correlates of systemic immune changes and changes in the tumor microenvironment.

**Trial Registration**

NCT02778685

**Reference**

1. Goel S, DeCristo MJ, Watt AC, et al. CDK4/6 inhibition triggers anti-tumour immunity. Nature. 2017;548(7668):471–475.

**Ethics Approval**

The study was approved by City of Hope National Cancer Center‘s Ethics Board, approval number 16058

#### P373 A window-of-opportunity study of pelareorep in early breast cancer (AWARE-1)

##### Luis Manso^1^, Patricia Villagrasa^2^, Nuria Chic^3^, Juan Cejalvo^4^, Yann Izarzugaza^5^, Blanca Cantos^5^, Salvador Blanch^6^, Manel Juan^3^, Blanca Gonzalez^3^, Rita Laeufle^7^, Gerard Nuovo^8^, Grey Wilkinson, PhD^7^, Matt Coffey^7^, Azucena Gonzalez^3^, Patricia Galvan^3^, Laia Paré^2^, Jordi Canes^2^, Xavier Gonzalez^9^, Aleix Prat, MD PhD^3^, Joaquín Gavilá^6^

###### ^1^Hospital Universitario 12 de Octubre, Madrid, Spain; ^2^SOLTI Breast Cancer Research Group, Barcelona, Spain; ^3^Hospital Clinic, Barcelona, Spain; ^4^Hospital Clínico, Valencia, Spain; ^5^Hospital Universitario, Madrid, Spain; ^6^Instituto Valenciano de Oncología (IVO), Valencia, Spain; ^7^Oncolytics Biotech Inc., San Diego, CA, United States; ^8^Ohio State University, Columbus, OH, United States; ^9^Hospital Universitari, Sant Cugat del Vallés, Spain

####### **Correspondence:** Aleix Prat (ALPRAT@clinic.cat)

**Background**

Pelareorep is an intravenously delivered (IV) unmodified oncolytic reovirus. Clinical studies have demonstrated that IV delivered pelareorep can replicate in tumor tissue and promote an inflamed tumor phenotype characterized the recruitment of CD8+ T cells and upregulation of PD-L1 [1]. Consistent with pelareorep’s role in promoting adaptive anti-tumor immunity, a randomized phase 2 study in metastatic breast cancer demonstrated a statistically significant improvement in overall survival when pelareorep was combined with paclitaxel [2]. We hypothesize that pelareorep mediated anti-tumor immune responses, such as those mediated by T cells, represent a novel strategy for the control or elimination of tumor cells in breast cancer. Specifically, in the preoperative setting for early breast cancer, we examined if pelareorep in combination with anti-PD-L1 therapy, atezolizumab, and other breast cancer therapies offers clinical benefit in terms of CeLTIL score, a metric for quantifying tumor cellularity (Cel) and tumor-infiltrating lymphocytes (TIL) [3].

**Methods**

This exploratory, non-randomized, window of opportunity study, will evaluate the safety and effect of pelareorep ± atezolizumab on the tumor microenvironment in 38 women with early breast cancer. Patients will receive study treatment for ~21 days prior to definitive surgery or neoadjuvant chemotherapy. Five cohorts will be examined (Figure 1): Cohort 1: HR+/HER2-neg (10 patients), pelareorep + letrozole. Cohort 2: HR+/HER2-neg (10 patients): pelareorep + letrozole + atezolizumab. Cohort 3: TNBC (6 patients): pelareorep + atezolizumab. Cohort 4: HER2+/HR+ (6 patients): pelareorep + trastuzumab + atezolizumab. Cohort 5: HER2+/HR- (6 patients): pelareorep + trastuzumab + atezolizumab. CelTIL, viral replication, and other immune-based biomarkers will be used to examine treatment-related changes within the tumor microenvironment. Blood and tumor tissue biopsies will be collected at screening, Day 3 (after pelareorep but before atezolizumab), and at surgery (Day ~21).

**Trial Registration**

Spanish clinical studies registry: 2018-003345-42

**References**

1. Samson A, Scott KJ, Taggart D, et al. Intravenous delivery of oncolytic reovirus to brain tumor patients immunologically primes for subsequent checkpoint blockade. Sci Transl Med 2018;10.

2. Bernstein V, Ellard SL, Dent SF, et al. A randomized phase II study of weekly paclitaxel with or without pelareorep in patients with metastatic breast cancer: final analysis of Canadian Cancer Trials Group IND.213. Breast Cancer Res Treat 2018;167:485-93.

3. Nuciforo, P., Pascual, T., Cortés, J., Llombart-Cussac, A., Fasani, R., Paré, L., … Holgado, E. (2018). A predictive model of pathologic response based on tumor cellularity and tumor-infiltrating lymphocytes (CelTIL) in HER2-positive breast cancer treated with chemo-free dual HER2 blockade. Annals of Oncology. https://doi.org/10.1093/annonc/mdx647

**Ethics Approval**

This study was approved by the Spanish Health Authority, protocol number 2018-003345-42.

**Fig. 1 (abstract P373).**
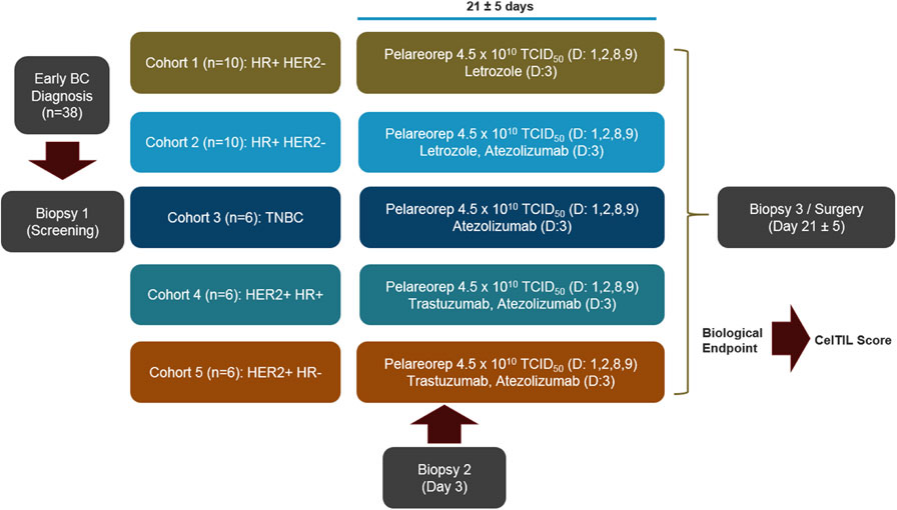
See text for description

#### P374 TCR repertoires from peripheral blood correlate with prognostic response in TNBC cancer vaccine immunotherapy

##### Sadanand Vodala, PhD, Andrew Nguyen, PhD, Noe Rodriguez, Peter Sieling, Charles Vaske, Jon Van Lew, Kayvan Niazi, John Lee, MD, Patrick Soon-Shiong, MD, FRCS, FACS, Shahrooz Rabizadeh

###### ImmunityBio, Inc, Culver City, CA, United States

####### **Correspondence:** Kayvan Niazi (kayvan.niazi@immunitybio.com)

**Background**

TNBC is an aggressive, heterogeneous, and high-grade subtype that represents 10-20% of breast carcinomas. Recently, TECENTRIQ and Abraxane were approved for the treatment of PD-L1+ unresectable locally advanced or mTNBC suggesting a role for immunotherapy in the treatment of this disease. Growing evidence suggests that chemotherapeutic agents and immunotherapies synergize in patients. Cancer vaccines are also a promising option for TNBC due to the discovery of neo-antigens and tumor associated antigens that mobilize anti-tumor T cells.

**Methods**

Here, we characterize T cell receptor repertoires in patients enrolled in a Phase Ib/2 clinical trial (NCT03387085) following treatment by a regimen intended to induce a synchronized, multi-compartment, anti-tumor immune response by mitigating the immune-suppressive effects of the tumor microenvironment and activating immunogenic tumor cell death. The trial combined metronomic low-dose chemotherapy, SBRT, an allogeneic NK cell line expressing high affinity CD16, yeast and adenoviral tumor-associated antigen vaccines, an IL15RαFc super-agonist, checkpoint inhibition, and an anti-angiogenic agent in a manner predicted to maximize cytotoxic T-cell mediated immunological recognition of tumor cells. Tumor associated antigens included adenoviral vector-based CEA, MUC1, brachyury, and yeast-based brachyury and CEA vaccines. Blood was collected pre- and post-treatment and target lesion analysis was performed using irRC and Recist1.1. Total RNA from PBMCs was used to generate sequencing libraries from each longitudinal sample. Using NGS, TCR-α and -β CDR3s were clonotyped and tracked over serial blood draws. Additionally the Shannon-Wiener Diversity Index (SWDI) was calculated for each time point.

**Results**

Patient samples showing consistent positive responses by irRC/Recist1.1 showed emergence and persistence of new TCR clones post-induction. This was further reflected in acute surges in SWDI. Furthermore, a high SWDI at baseline and post-treatment indicated clinical benefit suggesting an inverse correlation between disease severity and peripheral repertoire diversity. A TNBC super responder showed dramatic increases in mean SWDI index from 74 prior treatment to 1177 at first biopsy post treatment (34% decrease by irRC and 26% by Recist1.1 analysis) and achieved an index as high as 3516 in a subsequent biopsy (83% and 64% decrease by irRC and Recist 1.1 respectively).

**Conclusions**

Our findings strongly suggest that peripheral blood TCR repertoires are prognostic indicators/biomarker for TNBC cancer vaccine immunotherapy and for T cell-based immunotherapy in general. Taken together, these results strongly indicate activation and expansion of anti-tumor T cell clones following combination therapy. Further functional studies will expand our understanding of T cell based cancer vaccine immunotherapy in TNBC.

#### P375 Margetuximab combined with anti-PD-1 (MGA012) or anti-PD-1/LAG-3 (MGD013) +/- chemotherapy in first-line therapy of advanced/metastatic HER2+ gastroesophageal junction (GEJ) or gastric cancer (GC)

##### Daniel Catenacci, MD^1^, Minori Rosales^2^, Jon Wigginton, MD^2^, Hyun Cheol Chung, MD, PhD^3^, Harry Yoon^4^, Lin Shen^5^, Yoon-Koo Kang^6^, Markus Moehler^7^

###### ^1^University of Chicago, Chicago, IL, United States; ^2^MacroGenics, Inc. Rockville, MD, United States; ^3^University College of Medicine, Seoul, Korea, Republic of; ^4^Mayo Clinic Cancer Center, Rochester, MN, United States; ^5^Beijing Cancer Hospital, Beijing, China; ^6^Asan Medical, Seoul, Korea, Republic of; ^7^University Medical Center Mainz, Mainz, Germany

####### **Correspondence:** Daniel Catenacci (dcatenac@bsd.uchicago.edu)

**Background**

Trastuzumab (T), a monoclonal antibody (mAb) targeting the human epidermal growth factor receptor 2 (HER2) is the standard of care palliative first-line therapy for advanced HER2+ GEJ and GC patients. Margetuximab (M) is an Fc-engineered anti-HER2 mAb targeting the same HER2 epitope, but with higher affinity for both 158V (high binding) and 158F (low binding) alleles of the activating Fc receptor CD16A. Even more, M coordinately enhanced both innate and adaptive immunity, including antigen-specific T-cell responses to HER2 [1,2]. Programmed cell death receptor 1 (PD-1) and lymphocyte-activation gene 3 (LAG-3) are both T-cell checkpoint molecules that suppress T-cell function. MGA012 (INCMGA00012) is a humanized, hinge-stabilized, IgG4κanti-PD-1 mAb blocking binding of PD-L1 or PD-L2 to PD-1. MGD013 is a humanized Fc-bearing bispecific tetravalent protein that concomitantly binds to PD-1 and LAG-3, inhibiting their respective ligand-binding. We previously reported that a chemotherapy (CTX)-free regimen consisting of M+PD-1 blockade was well tolerated in GEJ/GC patients, and induced a 30% objective response rate (ORR) [3]. This was 2- to 3-fold greater than in historical controls with checkpoint inhibitors alone [4,5]. This registration-directed trial investigates the efficacy, safety, and tolerability of M+checkpoint inhibition ± CTX in metastatic/locally advanced, treatment-naïve, HER2+ GEJ/GC patients.

**Methods**

This adaptive open-label phase 2/3 study includes 2 cohorts. In the first single arm, CTX-free cohort A, M+MGA012 is evaluated in HER2+ (immunohistochemistry [IHC] 3+) and PD-L1+ (excluding microsatellite instability high) patients. After 40 patients are evaluated for response/safety, 60 more patients will be enrolled if the threshold for study continuation is met. In the randomized cohort B, HER2+ (IHC 3+ or IHC 2+/fluorescent in situ hybridization+) patients, are enrolled irrespective of PD-L1 status. Part 1 randomizes patients to 1 of 4 arms (50 patients each): control arm (T+CTX) or 1 experimental arm (M+CTX; M+CTX+MGA012; M+CTX+MGD013). CTX is investigator’s choice of XELOX or mFOLFOX-6. Part 2 (pick-the-winner) consists of the control (T+CTX) versus 1 experimental arm (M+CTX) + either MGA012 or MGD013, depending on the interim analysis from part 1; with 250 patients each. The primary efficacy endpoint for cohort A (both parts) is ORR per RECIST 1.1; for cohort B part 2 it is overall survival.

**Acknowledgements**

The authors thank all the patients, their families, and the entire staff who are participating in this trial. Professional medical writing support was provided by Meredith Rogers, MS, CMPP, of The Lockwood Group (Stamford, Connecticut, USA), in accordance with Good Publication Practice (GPP3) guidelines, with funding by MacroGenics, Inc. (Rockville, MD, USA).

**Trial Registration**

NCT number to come

**References**

1. Nordstrom JL, Gorlatov S, Zhang W, et al. Anti-tumor activity and toxicokinetics analysis of MGAH22, an anti-HER2 monoclonal antibody with enhanced Fcγ receptor binding properties. Breast Cancer Res. 2011;13:R123.

2. Nordstrom JL, Muth J, Erskine CL, et al. High frequency of HER2-specific immunity observed in patients (pts) with HER2+ cancers treated with margetuximab (M), an Fc-enhanced anti-HER2 monoclonal antibody (mAb). J Clin Oncol. 2019;37(suppl; abstr 1030).

3. Catenacci DVT, Limet KH, Uronis HE, al. Antitumor activity of margetuximab (M) plus pembrolizumab (P) in patients (pts) with advanced HER2+ (IHC3+) gastric carcinoma (GC). J Clin Oncol. 2019;37(suppl 4; abstr 65).

4. Fuchs CS, Doi T, Jang RW, et al. Safety and efficacy of pembrolizumab monotherapy in patients with previously treated advanced gastric and gastroesophageal junction cancer: phase 2 clinical KEYNOTE-059 trial. JAMA Oncol. 2018;4:e180013.

5. Kang YK, Boku N, Satoh T, et al. Nivolumab in patients with advanced gastric or gastro-oesophageal junction cancer refractory to, or intolerant of, at least two previous chemotherapy regimens (ONO-4538-12, ATTRACTION-2): a randomised, double-blind, placebo-controlled, phase 3 trial. Lancet. 2017;390:2461-2471.

**Ethics Approval**

Each investigator’s institutional review/ethics board approved the study.

#### P376 NOUS-209: A phase I, first-in-human, multicenter, open-label study of Nous-209 genetic vaccine for the treatment of microsatellite unstable solid tumors

##### Anna Gorrasi, PhD, Elisa Scarelli, Maria Teresa Catanese, Cinzia Traboni, Paola Antonini, Denis Brkic

###### Nouscom Srl, Rome, Italy

####### **Correspondence:** Elisa Scarelli (booking@nouscom.com)

**Background**

MSI-H tumors are caused by a defective DNA mismatch repair (dMMR) system that leads to the accumulation of mutations within microsatellite regions. Insertions or deletions (indels) in microsatellites of coding regions can result in the synthesis of tumor-specific frameshift peptides (FSPs). FSPs are considered safe and potent neoantigens because they are not expressed in the normal human proteome. We selected shared FSPs among patients with MSI cancers with the aim of developing an off-the-shelf vaccine for the cure of MSI tumors. 209 FSPs were assembled into 4 artificial genes and cloned into 4 Great Apes Adenoviral (GAd) and 4 Modified Vaccinia Ankara (MVA) vectors to generate a viral vectored vaccine called Nous-209. We showed that treatment of tumor-bearing mice with GAd/MVA-based neoantigen vaccines synergizes with Checkpoint Inhibitors (CPI), resulting in a 3-fold-increase of cured animals over CPI monotherapy.

**Methods**

A Phase-I, FIH study was designed to evaluate the safety, tolerability, and immunogenicity of Nous-209 genetic polyvalent vaccine in combination with the licensed programmed death receptor-1 (PD-1)-blocking antibody pembrolizumab and to detect any preliminary evidence of anti-tumor activity. Nous-209 is administered intramuscularly, with a heterologous prime/boost regimen composed of 1 prime with the mixture of 4 GAd vectors (GAd20-209-FSP) and 3 boosts with 4 MVA vectors (MVA-209-FSP). The target population includes adult patients with unresectable or metastatic dMMR or MSI-H colorectal cancer (CRC), gastric, and gastroesophageal (G-E) junction tumors.

The study is composed of two sequential cohorts. In the first cohort (dose escalation), the Recommended Phase 2 Dose (RP2D) will be established. In the second part (dose expansion), additional patients will be evaluated to consolidate the safety of the RP2D and establish the immunogenicity of the vaccination.

NOUS-209 IND has been cleared by the US Food and Drug Administration (FDA). The trial will be enrolling up to 30 patients at US clinical sites. Preliminary results from the study are expected in early 2020.

#### P377 Safety and anti-tumor activity of the transforming growth factor β receptor I kinase inhibitor, vactosertib, in combination with pembrolizumab in patients with metastatic colorectal or gastric cancer

##### Keun-Wook Lee, MD^1^, Young Suk Park, MD, PhD^2^, Joong Bae Ahn^3^, Sun Young Rha^3^, Hark Kyun Kim^4^, Park Young Lee^4^, Min-Hee Ryu^5^, Jeeyun Lee, MD, PhD^2^, Jin Kyung Lee^6^, Sunjin Hwang^6^, Seong-Jin Kiim^6^, Tae Won Kim, MD, PhD^5^

###### ^1^Seoul National University Bundang Hospit, Seongnam, Korea, Republic of; ^2^Samsung Medical Center, Seoul, Korea, Republic of; ^3^Severance Hospital, Seoul, Korea, Republic of; ^4^National Cancer Center, Kyunggi-do, Korea, Republic of; ^5^Asan Medical Center, Seoul, Korea, Republic of; ^6^Medpacto, Inc, Seoul, Korea, Republic of

####### **Correspondence:** Tae Won Kim (twkimmd@amc.seoul.kr)

**Background**

Vactosertib is a highly selective and potent inhibitor of transforming growth factor beta (TGF-β) receptor type 1. Recent studies have revealed that inhibition of TGF-β signaling reverses immunosuppressive tumor microenvironment and poor responses to cancer immunotherapy. To date, antitumor efficacy by immune check point inhibitors in colorectal or gastric/gastroesophageal cancer as monotherapy is known to be limited. A combination of TGF-β and PD-1 inhibition may induce immune restoration and improve antitumor responses. We are reporting Dose Finding part of Phase 1b/2a study evaluating the combination of vactosertib plus pembrolizumab in metastatic colorectal cancer (CRC) or diffuse gastric cancer (GC).

**Methods**

Eligible patients (pts) are ≥19 years old, have ECOG status ≤1, and have no prior exposure to immunotherapy including anti-CTLA-4, anti-PD-1, anti-PD-L1, and TGFβR1 kinase inhibitors. The primary objective is to assess the safety and the recommended dose of vactosertib given 5 days on/2 days off in combination with pembrolizumab 200 mg every 3 weeks. The Dose Finding part starts with vactosertib 200 mg BID plus pembrolizumab. Secondary objectives include characterization of vactosertib pharmacokinetics and anti-tumor activity by response rate.

**Results**

As of July 8, 2019, among 10 patients enrolled to 200 mg BID cohort, 6 were with CRC and 4 diffuse type GC. Median age was 51 (range 31-71), 50% were male, median number of previous lines of chemotherapy was 4 (range 2-6). All patients were immune checkpoint inhibitor naïve. No dose limiting toxicity was reported. Common adverse events (AE) were anorexia (33%), fatigue (33%), abdominal pain (33%), and fever (33%). There were 3 serious adverse events (SAE) reported; bilirubin elevations (1), pleural effusion (1), and ileus (1). All SAEs were not related to the study drugs. One patient (1/6) with microsatellite stable (MSS) metastatic CRC achieved partial response. Biomarker data will be presented at the meeting.

**Conclusions**

The combination of vactosertib plus pembrolizumab was tolerable with no additional safety concern. The activity of this combination in CRC and GC patients will be further evaluated in the Dose Expansion part of the study. Clinical trial information: NCT03724851

**Trial Registration**

NCT03724851

**Ethics Approval**

The study was approved by Ethics Board from Asan Medical Center, Samsung Seoul Hospital, Severance Hospital, Seoul National University Bundang Hospital, and National Cancer Center, with approval number 2018-1215, SMC 2018-07-146-006, 4-2018-0728, B-1808/487-003 and NCC2019-0042, respectively.

#### P378 Phase II study of combination ipilimumab, nivolumab, and panitumumab in patients with KRAS, NRAS, and BRAF wild-type (WT) microsatellite stable (MSS) metastatic colorectal cancer (mCRC)

##### Michael Lee, MD^1^, Patrick Loehrer, MD^2^, Iman Imanirad, MD^3^, Stacey Cohen, MD^4^, Kristen Ciombor, MD^5^, Cheryl Carlson, MD,PhD^1^, Hanna Sanoff, MD^1^, Autumn McRee, MD^1^

###### ^1^Univ of North Carolina at Chapel Hill, Chapel Hill, NC, United States; ^2^Indiana University, Indianapolis, IN, United States; ^3^Moffitt Cancer Center, Tampa, FL, United States; ^4^University of Washington, Seattle, WA, United States; ^5^Vanderbilt University, Nashville, TN, United States

####### **Correspondence:** Michael Lee (michael_s_lee@med.unc.edu)

**Background**

Panitumumab is an IgG2 monoclonal antibody (mAb) targeting the epidermal growth factor receptor (EGFR) and is a standard therapy for patients with KRAS, NRAS, and BRAF WT mCRC. Preclinical data shows that anti-EGFR mAbs require functional innate and adaptive immunity to mediate efficacy. Anti-EGFR therapy causes a tumor-specific adaptive immune response and immunogenic apoptosis [1,2], and anti-EGFR antibodies require functional T cells for in vivo efficacy [3]. However, resistance to anti-EGFR therapy inevitably develops and is associated with increased regulatory T cells expressing CTLA-4 [4] and activated immunosuppressive macrophages with upregulation of PD-L1 [5]. Thus, resistance to anti-EGFR antibody therapy is associated with increased expression of both CTLA-4 and PD-L1. We hypothesized that treatment with ipilimumab (anti-CTLA-4) and nivolumab (anti-PD-1) synergizes with panitumumab to significantly improve the response rate in patients with KRAS, NRAS, and BRAF WT MSS mCRC.

**Methods**

LCCC1632 is a multicenter, single-arm, phase II clinical trial with a pre-specified safety run-in of panitumumab, ipilimumab, and nivolumab in KRAS/NRAS/BRAF WT, MSS mCRC (NCT03442569). Eligible patients must have received 1-2 prior lines of therapy and no prior anti-EGFR or immune checkpoint inhibitor therapy. A 6-subject safety run-in was treated with ipilimumab 1 mg/kg IV q6wk, nivolumab 240 mg IV q2wk, and panitumumab 6 mg/kg IV q2wk and observed for 12 weeks for dose-limiting toxicities (DLTs), followed by expansion into a Simon’s two stage phase II trial, with 26 more subjects enrolled in the first phase and 56 total subjects planned. The primary endpoint is response rate defined by RECIST 1.1. Secondary endpoints include response rate by irRECIST, progression-free survival (PFS), overall survival (OS), and duration of response. Correlative studies include Consensus Molecular Subtype analysis by archival tissue and assessment of peripheral immune cell activation.

**Results**

Within the 12-week DLT period, only one grade 3-4 toxicity (grade 3 increased lipase) and no DLTs were observed. The most common grade 1-2 treatment-related adverse events within the DLT period included acneiform rash, hypomagnesemia, decreased lymphocyte count, anemia, nausea, vomiting, hypothyroidism, fatigue, cough, oral mucositis, and elevated AST. No dose modifications were required. Five of the 6 subjects (83%) had disease control (1 unconfirmed partial response, 4 stable disease) at 12 weeks.

**Conclusions**

The combination of panitumumab, ipilimumab, and nivolumab was well-tolerated, without unexpected toxicities encountered in the safety run-in cohort. The response rate and disease control rate demonstrate early signs of clinical activity. Enrollment in the phase II expansion is ongoing.

**Trial Registration**

ClinicalTrials.gov Identifier: NCT03442569

**References**

1. Garrido G, Rabasa A, Sanchez B, et al. Induction of immunogenic apoptosis by blockade of epidermal growth factor receptor activation with a specific antibody. J Immunol 2011;187:4954-66.

2. Pozzi C, Cuomo A, Spadoni I, et al. The EGFR-specific antibody cetuximab combined with chemotherapy triggers immunogenic cell death. Nat Med 2016;22:624-31.

3. Garrido G, Lorenzano P, Sanchez B, et al. T cells are crucial for the anti-metastatic effect of anti-epidermal growth factor receptor antibodies. Cancer Immunol Immunother 2007;56:1701-10.

4. Jie HB, Schuler PJ, Lee SC, et al. CTLA-4(+) Regulatory T Cells Increased in Cetuximab-Treated Head and Neck Cancer Patients Suppress NK Cell Cytotoxicity and Correlate with Poor Prognosis. Cancer Res 2015;75:2200-10.

5. Pander J, Heusinkveld M, van der Straaten T, et al. Activation of tumor-promoting type 2 macrophages by EGFR-targeting antibody cetuximab. Clin Cancer Res 2011;17:5668-73.

**Ethics Approval**

The study was approved by the Institutional Review Board of the University of North Carolina at Chapel Hill (IRB number 17-1832) and by the IRB of each subsite.

#### P379 Phase 1 safety study in healthy volunteers of AB680, a small-molecule inhibitor of CD73 and rationale for combination therapy in patients with gastrointestinal malignancies

##### Devika Ashok, PhD, Irene Luu, Akshata Udyavar, PhD, Lixia Jin, Lijuan Fu, Elaine Ginn, Ken Lawson, Jenna Jeffrey, PhD, Manmohan Leleti, PhD, Jay Powers, PhD, Eric Connor, Andy Pennell, Daniel DiRenzo, PhD, Dana Piovesan, MSc, Joanne Tan, PhD, Amanda Garofalo, Wade Berry, BA, Matthew Walters, PhD, Steve Young, PhD, Fangfang Yin, PhD, Dominic Lai, Lisa Seitz, MA

###### Arcus Biosciences, Hayward, CA, United States

####### **Correspondence:** Dominic Lai (dlai@arcusbio.com)

**Background**

Extracellular adenosine, present at high concentrations in the tumor microenvironment (TME), suppresses immune function. The enzymes ecto-5’-nucleotidase (CD73) and tissue non-specific alkaline phosphatase (TNAP) catalyze extracellular conversion of adenosine monophosphate (AMP) into adenosine. Inhibition of CD73 eliminates a major pathway of adenosine production in the TME and can reverse adenosine‐mediated immune suppression. Here we present the first results from a Phase 1 healthy volunteer (HV) study of AB680, a potent, reversible and selective small-molecule CD73 inhibitor. This placebo-controlled HV study assessed the safety, tolerability, pharmacokinetic (PK) and pharmacodynamic (PD) profile of AB680.

**Methods**

Male or female healthy volunteers aged 18-55 with a body mass index of 18-30 kg/m2 were eligible for enrollment in the AB680CSP0001 study (NCT03677973). Escalating doses of AB680 were evaluated in a single ascending dose (SAD) and repeat dosing study. Post dosing, participants were admitted for evaluation and serially assessed for adverse events. Blood samples were collected at various timepoints to elucidate the PK and PD profiles of AB680. AB680 plasma concentrations were determined using LC-MS/MS and PD effects were evaluated by monitoring AMP-ase activity in serum. Linear models were used to predict tumor up-regulation of CD73 in multiple tumor types with the assumption that tumors expressing higher CD73 levels will derive greater benefit from CD73 inhibition. Immunohistochemistry analyses were performed on serial sections of tumor tissue to correlate protein and gene expression.

**Results**

The HV study enrolled more than 50 participants, randomized 3:1 (active: placebo). AB680 exhibited a good safety profile and displayed a long half-life following a 30-60 minute intravenous (IV) infusion, consistent with the intended Q2W dosing schedule in cancer patients. Doses were identified that provided maximal inhibition of peripheral AMP-ase activity. Our bioinformatics analyses identified tumors that have high CD73 expression relative to TNAP and identified pan-RAS mutations that correlate with upregulated CD73 and poor prognosis.

**Conclusions**

AB680 is the first potent and selective small-molecule CD73 inhibitor to be tested in humans. This first-in-human study demonstrates that AB680 is well tolerated and has optimal PK/PD to support its continued evaluation in cancer patients.

**Trial Registration**

ClinicalTrials.gov NCT03677973

**Ethics Approval**

The study was approved by Bellberry Limited Ethics Board, approval number 2018-08-673

#### P380 Phase 1b/2 study of BXCL701, a small molecule inhibitor of dipeptidyl peptidases, with bempegaldesleukin (bempeg, NKTR-214) and avelumab (anti-PD-L1) in unresectable or metastatic pancreatic cancer

##### Louis Weiner, MD^1^, Benjamin Weinberg, MD^1^, Stina Singel^2^, Cedric Burg^3^, Diane Healey^3^, Jonathan Zalevsky, PhD^2^, Chetan Lathia^3^, Willem Overwijk, PhD^2^, Cristian Massacesi^4^, Joyce Acbay^2^, John MacDougall, PhD^3^, Vincent O'Neill^3^

###### ^1^Georgetown Lombardi Comprehensive Cancer Center, Washington, DC, United States; ^2^Nektar Therapeutics, San Francisco, CA, United States; ^3^BioXcel Therapeutics, New Haven, CT, United States; ^4^Pfizer, New York, NY, United States

####### **Correspondence:** Diane Healey (dhealey@bioxceltherapeutics.com)

**Background**

Treatment of pancreatic cancer continues to have poor outcomes with currently available therapies including checkpoint inhibitors. BXCL701 (talabostat, previously PT100) is an orally administered, small molecule inhibitor of dipeptidyl peptidases (DPP) specifically DPP4, DPP8 and DPP9. Inhibition of DPP8 and DPP9 triggers a process in macrophages called pyroptosis leading to innate immune proinflammatory stimulation of the tumor microenvironment[1,2,3]. BXCL701 also inhibits fibroblast activation protein (FAP) releasing the FAP-mediated block of T-cell migration into the tumor[4]. In syngeneic animal models, significant tumor responses were observed when BXCL701 was used with checkpoint inhibition[2]. Bempegaldesleukin (bempeg, NKTR-214) is a CD122-preferential interleukin-2 (IL-2) pathway agonist being investigated for its potential to leverage the clinically validated IL-2 pathway and selectively stimulate an immune response, without overacting the immune system. Bempeg has demonstrated robust anti-cancer activity when used with checkpoint inhibition in multiple murine tumor models and recently in multiple human cancers[5,6]. Avelumab is a checkpoint inhibitor that binds PD-L1 resulting in the release of immune inhibitory effects of this pathway thereby restoring immune responses, including anti-cancer immune responses. In a syngeneic mouse model of pancreatic cancer (Pan02), the triple combination demonstrated potent anti-cancer activity, including long-lasting anti-cancer immunity[7]. These results provide therapeutic rationale for testing of this combination in patients with pancreatic cancer.

**Methods**

This is an open-label, multicenter study to determine the safety and efficacy of the triple combination therapy of BXCL701, bempeg and avelumab. Patients with pancreatic cancer should have received at least 1 line of gemcitabine-based therapy and no more than 2 lines of chemotherapy for unresectable or metastatic disease, received no prior anti-PD-1/PD-L1, IL-2 based or other T-cell directed anti-cancer therapy, and have ECOG 0-1. Patients must agree to biopsy of metastatic disease. Part 1 (Phase 1b), is the 3+3 dose escalation phase designed to evaluate the safety of escalating doses of BXCL701 with bempeg and avelumab. Part 2 (Phase 2) will begin once the recommended combination dose is determined. A Simon two-stage design will be used in Phase 2, initially enrolling 13 patients. If 2 or more responses are observed, the cohort will expand to 34 patients. The primary efficacy parameter is objective response by RECIST 1.1. The study will also assess other parameters measuring clinical benefit and mechanistic effects on the immune system and tumor microenvironment. The study is not yet recruiting in the US.

**Trial Registration**

Pending

**References**

1. Rastelli L, Gupta S, Dahiya A, et al. The synergy between BXCL701, a DPP inhibitor, and immune checkpoint inhibitors discovered using AI and Big Data analytics. Cancer Research 2017;77(13 Suppl):Abstract nr 2629.

2. Okondo M, Johnson D, Sridharan R, et al. DPP8/9 inhibition induces pro-caspase-1-dependent monocyte and macrophage pyroptosis. Nature Chemical Biology. 2017;13(1):46-53

3. Okondo M, Rao S, Taapazuing C, et al. Inhibition of DPP8/9 Activates the Nlrp1b Inflammasome. Cell Chemical Biology. 2018;25:1-6

4. Lo A, Wang LC, Scholler J, et al. Tumor-Promoting Desmoplasia Is Disrupted by Depleting FAP-Expressing Stromal Cells. Cancer Research. 2015;75(14):2800-2810.

5. Charych D, Khalili S, Dixit V et al. Modeling the receptor pharmacology, pharmacokinetics, and pharmacodynamics of NKTR-214, a kinetically-controlled interleukin-2 (IL2) receptor agonist for cancer immunotherapy. PLOS ONE 2017; 12(7): e0179431.

6. Diab A, Tannir N, Bernatchez C, et al. A phase ½ study of a novel IL-2 cytokine, NKTR-214, and nivolumab in patients with select locally advanced or metastatic solid tumors. J Clin Oncol. 2017;35(suppl):e14040.

7. Rastelli L, Gupta S, Jagga Z, et al. Efficacy and immune modulation by BXCL701 and dipeptidyl peptidase inhibitor, NKTR-214 a CD122-biased immune agonist with PD1 blockage in murine pancreatic tumors [abstract]. J Clin Oncol. 2018;36(suppl):3085

**Ethics Approval**

This study was approved by Institution Review Boards or Ethics Committees affiliated with participating institutions

#### P381 Phase 1b/2 study of BXCL701, a small molecule inhibitor of dipeptidyl peptidases (DPP), with pembrolizumab, (anti-PD-1) monoclonal antibody, in small cell neuroendocrine prostate cancer (SCNC, NEPC)

##### Diane Healey^1^, Rahul Aggarwal, MD^2^, Rahul Aggarwal, MD^2^, Vincent O'Neill^1^, Cedric Burg^1^, Diane Healey^1^, Jiaoti Huang^3^, Johann De Bono, MD^4^, Eric Small^2^

###### ^1^BioXcel Therapeutics, New Haven, CT, United States; ^2^UCSF Helen Diller Family Comprehensive Cancer Center, San Francisco, CA, United States; ^3^Duke University School of Medicine, Durham, NC, United States; ^4^Institute for Cancer Research Royal Marsden NHS Foundation Trust, London, United Kingdom

####### **Correspondence:** Diane Healey (dhealey@bioxceltherapeutics.com)

**Background**

Treatment emergent Small Cell Neuroendocrine Prostate Cancer (t-SCNC) is an aggressive with poor survival outcomes on standard therapies given for metastatic castration-resistant disease[1 ]. BXCL701 (talabostat previously PT100) is an orally administered, small molecule inhibitor of dipeptidyl peptidases (DPP) specifically DPP4, DPP8 and DPP9 triggering macrophage cell death via pyroptosis resulting in proinflammatory stimulation of the innate immunity pathway[2,3,4]. BXCL701 also inhibits fibroblast activation protein (FAP) releasing the FAP-mediated block of T-cell migration into the tumor[5]. FAP, DPP8 and DPP9 are expressed and activated in neuroendocrine CRPC[6]. Correlation is robust between the expression of PD-L1 and the targets of BXCL701, particularly FAP, DPP8 and DPP9[2]. In syngeneic animal models, significant tumor responses were observed when BXCL701 was used in combination with checkpoint inhibition[2]. Therefore, it is believed BXCL701 mediated activation of the innate immune system via macrophage pyroptosis inflames the cancer microenvironment and t-SCNC might become responsive to checkpoint inhibition combined with BXCL701.

**Methods**

This is an open-label, multicenter study in patients with progressive, metastatic castration resistant prostate cancer (CRPC) as defined by PCWG3. Patients should have received at least 1 line of systemic therapy and no more than 2 lines of cytotoxic chemotherapy for CRPC, received no prior anti-PD-1/PD-L1 or other T-cell directed anti-cancer therapy, and have ECOG 0-2. Patients in Phase 2 must also have evidence of SCNC,NEPC by central pathology and agree to biopsy of metastatic disease. Phase 1b, is the 3+3 dose escalation phase designed to evaluate the safety of 0.4 mg and 0.6mg BXCL701 QD on days 1 to 14 of 21-day cycle plus fixed dose pembrolizumab 200 mg administered IV on day 1 every 21 days to determine the recommended dose for phase 2. A Simon’s two-stage design will be used in Phase 2 and initially 15 patients with SCNC/NEPC will be enrolled. If more than 2 responses are observed, then the cohort will expand to 28 patients. Primary efficacy parameter is the composite response defined as achieving 1 or more of the following: • Objective response by RECIST 1.1 • CTC conversion from > 5/7.5 mL to < 5/7.5 mL per Veridex assay by Week 12 • Greater than 50% PSA decline from baseline by Week 12. The study is open in the US with expansion to the UK underway.

**Trial Registration**

NCT03910660

EUDRACT:2018-003734-32

**References**

1. Aggarwal R, Huang J, Alumkal JJ, et al. Clinical and Genomic Characterization of Treatment-Emergent Small-Cell Neuroendocrine Prostate Cancer: A Multi-institutional Prospective Study. J Clin Oncol. 2018; 36(24): 2492-2505

2. Rastelli L, Gupta S, Dahiya A, et al. The synergy between BXCL701, a DPP inhibitor, and immune checkpoint inhibitors discovered using AI and Big Data analytics [abstract]. In: Proceedings of the American Association for Cancer Research Annual Meeting 2017; 2017 Apr 1-5; Washington, DC. Philadelphia (PA): AACR; Cancer Res 2017;77(13 Suppl):Abstract nr 2629.

3. Okondo M, Johnson D, Sridharan R, et al. DPP8/9 inhibition induces pro-caspase-1-dependent monocyte and macrophage pyroptosis. Nature Chemical Biology. 2017;13(1):46-53

4. OkondoM, Rao S, Taapazuing C, et al. Inhibition of DPP8/9 Activates the Nlrp1b Inflammasome. Cell Chemical Biology. 2018;25:1-6

5. Lo A, Wang LC, Scholler J, et al. Tumor-Promoting Desmoplasia Is Disrupted by Depleting FAP-Expressing Stromal Cells. Cancer Research. 2015;75(14):2800-2810.

6. cBioPortal version 1.4.3 (dataset accessed on 9th March, 2017)

**Ethics Approval**

The study was approved by Institution Review Boards or Ethics Committees affiliated with participating institutions.

#### P382 KEYNOTE-365 cohort D: phase 1b/2 study of pembrolizumab plus abiraterone acetate and prednisone in metastatic castration-resistant prostate cancer

##### Leonard Appleman, MD, PhD^1^, Josep Piulats^2^, Nataliya Mar^3^, José Arranz^4^, Anthony Joshua, MD^5^, Tina Mayer^6^, Neal Shore, MD^7^, Haiyan Wu, PhD^8^, Charles Schloss^8^, Evan Yu^9^

###### ^1^UPMC Hillman Cancer Center, Pittsburgh, PA, United States; ^2^Catalan Cancer Institute, Barcelona, Spain; ^3^UC Irvine Medical Center, Orange, CA, Uunited States; ^4^Hospital General Universitario Gregorio Marañon, Madrid, Spain; ^5^St. Vincent’s Hospital Sydney, Sydney, NSW, Australia; ^6^Rutgers Cancer Institute of New Jersey, New Brunswick, NJ, United States; ^7^Carolina Urologic Research Center, Myrtle Beach, SC,United States; ^8^Merck & Co. Inc., Kenilworth, NJ, United States; ^9^University of Washington, Seattle, WA, United States

####### **Correspondence:** Leonard Appleman (applemanlj@upmc.edu)

**Background**

For patients with metastatic castration-resistant prostate cancer (mCRPC), additional therapeutic options are needed to improve overall outcomes and to delay the use of chemotherapy. In early-phase trials, pembrolizumab, an anti–PD-1 antibody, showed some activity as monotherapy in heavily pretreated patients with mCRPC. Cohort D of the nonrandomized, multicohort, open-label, phase 1b/2 KEYNOTE-365 (NCT02861573) study has been designed to evaluate the safety and efficacy of pembrolizumab combined with abiraterone acetate and prednisone in patients with mCRPC who have not received chemotherapy for mCRPC.

**Methods**

Adults (≥18 years) with histologically or cytologically confirmed prostate cancer, without small cell histology, and who experience progression ≤6 months before screening and have an ECOG PS score of 0 or 1 are eligible. Patients must be chemotherapy naïve for mCRPC and must not have received second-generation hormonal therapy for mCRPC or must not have experienced failed treatment with enzalutamide or become intolerant to enzalutamide for mCRPC. Patients will receive pembrolizumab 200 mg IV every 3 weeks, abiraterone acetate 1000 mg once daily, and prednisone 5 mg twice daily. Responses will be radiographically assessed every 9 weeks during year 1 and every 12 weeks thereafter. Pembrolizumab treatment will continue for up to 35 cycles (approximately 2 years) or until disease progression, unacceptable toxicity, or patient/physician decision to withdraw. Patients who discontinue 1 of the 2 drugs in the combination because of drug-related adverse events can continue with the other combination partner. All patients who discontinue treatment will be monitored until trial completion. Primary end points are prostate-specific antigen (PSA) response rate, defined as a PSA decrease of ≥50% from baseline measured on 2 occasions at least 3 weeks apart for confirmation, objective response rate (ORR) per RECIST v1.1 by blinded independent central review (BICR), and safety. Secondary end points include time to PSA progression, ORR based on Prostate Cancer Working Group 3 (PCWG3)–modified RECIST v1.1 assessed by BICR, duration of response based on RECIST 1.1 and PCWG3-modified RECIST 1.1 assessed by BICR, radiographic progression-free survival based on PCWG3-modified RECIST 1.1 assessed by BICR, and overall survival. Recruitment began in December 2018 and will continue until ~100 patients are enrolled.

**Ethics Approval**

The study and the protocol were approved by the Institutional Review Board or ethics committee at each site.

**Consent**

All patients provided written informed consent to participate in the clinical trial.

#### P383 Withdrawn

#### P384 A phase I dose escalation and expansion study of HPN424, a PSMA-targeting T cell engager, in patients with advanced prostate cancer refractory to androgen therapy

##### Johanna Bendell, MD^1^, Mark Stein, MD^2^, Johann de Bono, MD^3^, Richard Austin, PhD^4^, Sue Hirabayashi^4^, Che-Leung Law, PhD^4^, Bryan Lemon, PhD^4^, Holger Wesche, PhD^4^, Aaron Weitzman, MD FACP^5^, Lawrence Fong, MD^6^

###### ^1^Sarah Cannon Research Institute/Tennessee Oncology, Nashville, TN, United States; ^2^Columbia University, New York, NY, United States; ^3^Royal Marsden Hospital and The Institute of Cancer Research, Sutton, United Kingdom; ^4^Harpoon Therapeutics, South San Francisco, CA, United States; ^5^Weitzman Consulting Group, Los Altos Hills, CA, United States; ^6^University of California, San Francisco, San Francisco, CA, United States

####### **Correspondence:** Johanna Bendell (jlee@samornbiosciences.com)

**Background**

HPN424 is a PSMA-targeting T cell engager derived from the TriTAC platform (Tri-specific T Cell-Activating Construct). PSMA is a well-validated antigen specific to prostate epithelial cells upregulated upon malignant prostate cancer with limited expression in normal tissues. HPN424 is a recombinant polypeptide of ~50kDa containing three humanized antibody-derived binding domains, targeting PSMA (for tumor binding), albumin (for half-life extension) and CD3 (for T cell engagement). It has been engineered to be a small, globular protein to enable efficient exposure in solid tumor tissue with prolonged half-life and excellent stability under physiological conditions. HPN424 binds monomerically to CD3 and PSMA, minimizing non-specific T-cell activation. These features are designed to increase the therapeutic index compared to earlier generations of T cell engagers by minimizing off-target toxicities. HPN424 mediates potent target tumor cell killing in a PSMA-specific manner in vitro and in xenograft models in the presence of T cells, demonstrated at very low antigen densities. Consistent with its mechanism of action (MOA), tumor cell killing is accompanied by T cell activation, cytokine induction, and T cell expansion.

**Methods**

This is a Phase 1, open-label, multicenter, dose escalation and dose expansion study to evaluate the safety, tolerability, clinical activity, and pharmacokinetics of HPN424 in adult patients with metastatic castrate-resistant prostate cancer (mCRPC) who have progressed on the prior regimen (per PCWG3 criteria) and received at least 2 prior systemic therapies approved for mCRPC. HPN424 is administered once weekly as one-hour IV infusion by single-patient cohorts until either a Grade ≥2 adverse event (AE) that is possibly related to HPN424 is observed or an estimated therapeutic dose level has been reached. Then a conventional 3+3 design is implemented. Dose escalation will continue until a recommended phase 2 dose (RP2D) is determined. In dose expansion, up to 18 patients receive HPN424 at the established RP2D. Additional expansion cohorts may be added. Patients may continue weekly HPN424 treatment as long as they are receiving clinical benefit. Primary endpoints are number and severity of DLTs following treatment with escalating doses of HPN424 during escalation, and overall response rate (per PCWG3 criteria) in dose expansion. Secondary endpoints include AEs, preliminary anti-tumor activity, pharmacokinetic and pharmacodynamic parameters based on the proposed MOA of HPN424.

**Trial Registration**

NCT03577028

**Ethics Approval**

This study was approved by each participating institution's Institutional Review Board.

#### P385 Pembrolizumab plus enzalutamide versus placebo plus enzalutamide for metastatic castration-resistant prostate cancer: phase 3 KEYNOTE-641 study

##### Julie Nicole Graff^1^, Joseph Burgents^2^, Li Wen Liang^3^, Arnulf Stenzl^4^

###### ^1^Knight Cancer Institute, Oregon Health & Science University, Portland, OR, United States; ^2^Merck & Co., Inc., Kenilworth, NJ, USA, Kenilworth, NJ, United States; ^3^MSD, China, Beijing, China, Beijing, China; ^4^University of Tuebingen Medical School, Tuebingen, Germany, Tübingen, Germany

####### **Correspondence:** Julie Nicole Graff (graffj@ohsu.edu)

**Background**

Treatment options for patients with metastatic castration-resistant prostate cancer (mCRPC) are noncurative, and life expectancy is only about 3 years. Enzalutamide is an androgen receptor inhibitor used for the treatment of patients with mCRPC. Pembrolizumab is a programmed death 1 (PD-1) inhibitor with antitumor activity as monotherapy in mCRPC. Results of clinical studies have shown that the mechanisms of action of pembrolizumab and enzalutamide may be synergistic. In the phase 1b/2 KEYNOTE-365 (NCT02861573) study, antitumor activity of pembrolizumab plus enzalutamide was observed in mCRPC patients pretreated with abiraterone acetate. Also, in a single-arm, phase 2 study (NCT02312557) of patients who progressed on enzalutamide, some patients had profound anticancer response when pembrolizumab was added to enzalutamide that lasted years. KEYNOTE-641 (NCT03834493) is a randomized, double-blind, phase 3 trial to evaluate efficacy and safety of pembrolizumab plus enzalutamide versus placebo plus enzalutamide for patients with mCRPC.

**Methods**

Approximately 1200 patients will be randomly assigned 1:1 to receive enzalutamide 160 mg/day plus pembrolizumab 200 mg Q3W or enzalutamide 160 mg/day plus placebo. Treatment will be stratified per prior abiraterone acetate treatment (yes/no), metastases (bone only/liver/other), and prior docetaxel treatment for metastatic hormone-sensitive prostate cancer (yes/no). Adults (≥18 years) with histologically or cytologically confirmed prostate cancer and mCRPC who experienced biochemical or radiographic progression are eligible. Patients who received chemotherapy for mCRPC, checkpoint inhibition, or any treatment with a second-generation androgen receptor inhibitor (eg, enzalutamide, apalutamide, or darolutamide) are excluded. Patients intolerant of or experiencing progression with prior abiraterone acetate therapy are included. Patients must have ECOG PS 0/1, adequate organ function, and tissue for biomarker analysis. Responses will be assessed by CT/MRI and radionuclide bone imaging per PCWG-modified RECIST v1.1 every 9 weeks during the first year and every 12 weeks thereafter. Treatment will continue with enzalutamide plus pembrolizumab/placebo until radiographic disease progression, unacceptable toxicity, or consent withdrawal, with a maximum of 2 years of treatment for the pembrolizumab/placebo component of the combination. Dual primary end points are overall survival and radiographic progression-free survival by blinded independent central review. The key secondary efficacy end point is time to subsequent anticancer therapy or death. Additional secondary end points include objective response rate, duration of response, prostate specific antigen (PSA) response rate, PSA-undetectable rate, time to PSA progression, time to pain progression, and time to radiographic soft tissue progression. Safety and tolerability will also be reported.

**Trial Registration**

ClinicalTrials.gov; NCT03834493

**Ethics Approval**

The study and the protocol were approved by the Institutional Review Board or ethics committee at each site.

**Consent**

All patients provided written informed consent to participate in the clinical trial.

#### P386 Economic benefits associated with treatment-free survival of immuno-oncology agents among untreated patients with intermediate/poor-risk advanced or metastatic renal cell carcinoma

##### Michael Harrison, MD^1^, Meredith Regan, PhD^2^, Michael Atkins, MD^3^, Sumati Rao, PhD^4^, Shuo Yang, PhD^4^, Jennifer Johansen, PharmD, BCPS^4^, Ella Du, MESc^5^, Chenyang Gu^5^, Ela Fadli^5^, Keith Betts, PhD^5^, David McDermott, MD^6^

###### ^1^Duke Cancer Institute, Chapel Hill, NC, United States; ^2^Dana-Farber Cancer Institute, Boston, MA, United States; ^3^Georgetown Lombardi Comprehensive Cancer, Washington, DC, United States; ^4^Bristol-Myers Squibb, Princeton, NJ, United States; ^5^Analysis Group, Inc, Los Angeles, CA, United States; ^6^Beth Israel Deaconess Medical Center, Milton, MA, United States

####### **Correspondence:** Michael Harrison (Michael.Harrison@Duke.edu)

**Background**

Nivolumab plus ipilimumab (NIVO+IPI) was associated with significantly longer treatment-free survival (TFS) compared with sunitinib in intermediate/poor-risk patients with previously untreated advanced or metastatic renal cell carcinoma (aRCC) in the CheckMate 214 trial [1]. To further assess the economic impact of NIVO+IPI associated with TFS, this study compared healthcare costs among untreated intermediate/poor-risk aRCC patients with different lengths of TFS.

**Methods**

This study used individual patient data from the NIVO+IPI arm in CheckMate 214 (database lock, August 6, 2018; minimum follow-up, 30 months). TFS is defined as the time from last dose of NIVO+IPI to the start of subsequent systemic therapy or death, whichever occurs first. All intermediate/poor-risk aRCC patients who received NIVO+IPI and provided consent were classified into 3 cohorts based on the length of TFS: cohort 1 remained on NIVO monotherapy maintenance; cohort 2 had TFS ≤6 months; and cohort 3 had TFS >6 months. Patient characteristics and overall survival from randomization were described for the 3 cohorts. Monthly costs from randomization to last known date alive, including study treatment costs, all-cause grade 3/4 adverse event costs, terminal care costs, and subsequent treatment costs were compared between cohort 3 versus cohort 1 and cohort 3 versus cohort 2 using Wilcoxon rank-sum tests. All costs were adjusted to 2019 United States dollars.

**Results**

Of the 420 eligible patients, 16.4% (N=69) remained on NIVO monotherapy maintenance, 60.2% (N=253) had TFS ≤6 months, and 23.3% (N=98) had TFS >6 months by the end of patient follow-up. Patient characteristics were mostly similar between cohort 3 versus cohort 1 or 2. By definition, all patients (100%) in cohort 1 were alive at the end of follow-up. The survival probabilities were 94% for cohort 3 and 60% for cohort 2 by 18 months and 83% for cohort 3 and 39% for cohort 2 by 30 months. Patients with TFS >6 months had significantly lower monthly costs ($8,318) compared with those who never discontinued NIVO+IPI ($16,374) and those with TFS ≤6 months ($22,811) (Figure 1).

**Conclusions**

This retrospective healthcare cost assessment of 3 patterns of patient outcomes during and after treatment with NIVO+IPI suggests an economic value of achieving prolonged TFS (>6 months). Thus, management strategies that would lead to prolonged TFS could be beneficial both clinically and economically.

**Acknowledgements**

Writing support was provided by Analysis Group, Inc. and editorial support was provided by Parexel, funded by Bristol-Myers Squibb.

**Trial Registration**

NCT02231749.

**Reference**

1. McDermott DF, Rini BI, Motzer RJ, Tannir NM, Escudier B, Kollmannsberger CK, Hammers HJ, Porta C, George S, Donskov F, Gurney HP. 874P Treatment-free interval (TFI) following discontinuation of first-line nivolumab plus ipilimumab (N+ I) or sunitinib (S) in patients (Pts) with advanced renal cell carcinoma (aRCC): CheckMate 214 analysis. Ann Oncol. 2018; 29(suppl 8):mdy283.083.

**Ethics Approval**

This trial was approved by the institutional review board or ethics committee at each site.

**Fig. 1 (abstract P386).**
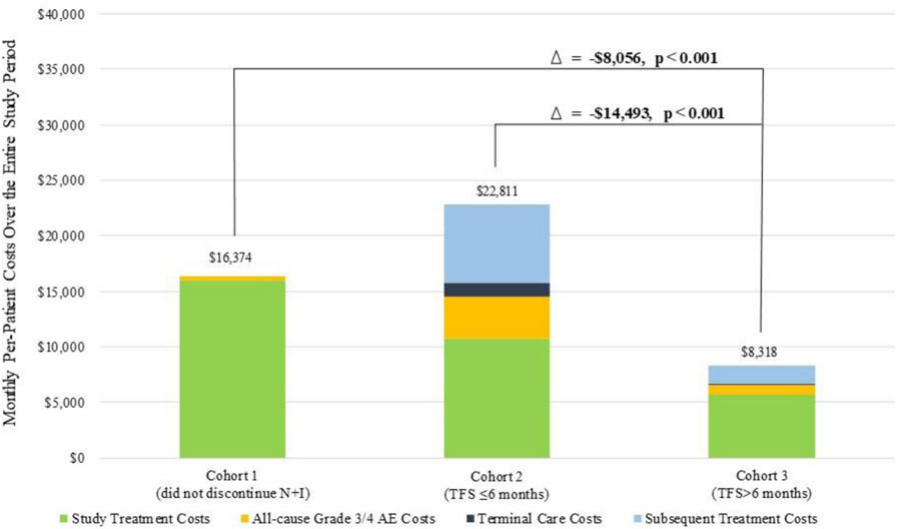
See text for description

#### P387 A multicenter, open-label, exploratory platform study to evaluate biomarkers and immunotherapy combinations for the treatment of patients with metastatic castration-resistant prostate cancer (PORTER)

##### Leo Nissola, MD^1^, Karen Autio, MS, MD^2^, Nina Bhardwaj, MD, PhD^3^, Matthew Galsky, MD^3^, Kristopher Wentzel, MD^4^, Vanessa Lucey^5^, Cheryl Selinsky, PhD^1^, Christopher Perry^1^, Christopher Cabanski, PhD^1^, Ari Bitton^1^, Justin Fairchild^1^, Christine Horak, PhD^6^, Jeffrey Skolnik, MD^7^, Michael Yellin, MD^8^, Ute Dugan, MD, PhD^6^, Ramy Ibrahim, MD^1^, Lawrence Fong, MD^9^

###### ^1^Parker Institute for Cancer Immunotherapy, San Francisco, CA, United States; ^2^Memorial Sloan Kettering Cancer Center, New York, NY; ^3^The Mount Sinai Hospital, New York, NY, United States; ^4^The Angeles Clinic & Research Institute, Los Angeles, CA, United States; ^5^Cancer Research Institute, New York, NY, United States; ^6^Bristol-Myers Squibb, Lawrenceville, NJ, United States; ^7^Inovio Pharmaceuticals, Plymouth Meeting, PA, United States; ^8^Celldex Therapeutics, Hampton, NJ, United States; ^9^University of California San Francisco, San Francisco, CA, United States

####### **Correspondence:** Leo Nissola (lnissola@parkerici.org)

**Background**

Metastatic castration resistant prostate cancer (mCRPC), the lethal form of prostate cancer, has shown limited benefit from immune checkpoint inhibition as monotherapy, with two randomized phase 3 trials with ipilimumab failing to show a survival benefit, and a large phase 2 trial with pembrolizumab demonstrating an overall response rate (ORR) of 3-5%. Clearly novel combinations are needed and a deeper understanding of immune resistance.

Using a multi-arm, multi-stage platform design, the PORTER study will adaptively test multiple immunotherapeutic combinations to activate the innate and adaptive immune systems. Coupled with deep immune biomarker profiling, this design will enable rapid insights into the immune responses for each combination, providing data for potential larger definitive trials, while generating hypotheses for new cohorts.

**Methods**

PORTER is an open-label, non-randomized, exploratory platform study designed to assess the safety and antitumor activity of multiple immunotherapy combinations in participants with mCRPC who have received prior secondary androgen inhibition therapy. Each cohort has a two-stage design (initial n = 15, expansion n = 15) with a decision to expand based on the safety, clinical activity, and biomarker results observed in the initial stage.

Cohort A is open and recruiting, testing the combination of bempegaldesleukin (“BEMPEG”, NKTR-214; a CD-122 preferential IL-2 pathway agonist) with nivolumab (PD-1 inhibitor), postulating that this will increase PD-L1 expression, intratumoral T and NK cells, and induce an IFN gamma signature.

Cohort B will combine CDX-301 (Flt3L), poly-ICLC (PAMP-adjuvant), nivolumab and stereotactic body radiation therapy, in 1-5 metastatic sites, inducing immunogenic cell death, mobilizing and activating dendritic cells increasing tumor antigen presentation, and overcoming adaptive immune resistance in mCRPC.

Cohort C will evaluate INO-5151, a DNA vaccine encoding PSA, PSMA, and IL-12 delivered via intramuscular electroporation, in addition to CDX-301 and nivolumab. This is a multi-pronged approach to mobilize and activate dendritic cells, stimulate anti-tumor CD8 T cells, and circumvent adaptive immune resistance.

Inclusion criteria include: histologically-confirmed mCRPC that is measurable or non-measurable by Prostate Cancer Clinical Trials Working Group 3 and progressing despite secondary androgen receptor signaling inhibitor therapy.

The primary endpoint: safety, as assessed by the incidence and severity of adverse events. Secondary endpoints: Composite ORR (PSA reduction >50%, confirmed CR or PR per RECIST 1.1, or change in circulating tumor cell (CTC) from >5 cells/7.5 ml to

**Trial Registration**

https://clinicaltrials.gov/ct2/show/NCT03835533

**References**

1. Alexandrov LB, Nik-Zainal S, Wedge DC, Aparicio SAJR, Behjati S, Biankin AV, et al. Signatures of mutational processes in human cancer. Nature 2013;500(7463):415–21. Beer TM, Kwon ED, Drake CG, Fizazi K, Logothetis C, Gravis G, et al. Randomized, Double-Blind, Phase III Trial of Ipilimumab Versus Placebo in Asymptomatic or Minimally Symptomatic Patients With Metastatic Chemotherapy-Naive Castration-Resistant Prostate Cancer. J Clin Oncol 2017;35(1):40–7.

2. Brahmer JR, Drake CG, Wollner I, Powderly JD, Picus J, Sharfman WH, et al. Phase I study of single-agent anti-programmed death-1 (MDX-1106) in refractory solid tumors: safety, clinical activity, pharmacodynamics, and immunologic correlates. J Clin Oncol 2010;28(19):3167–75.

3. Di Lorenzo G, Buonerba C, Kantoff PW. Immunotherapy for the treatment of prostate cancer. Nat Rev Clin Oncol 2011;8(9):551–61. Drake CG. Prostate cancer as a model for tumour immunotherapy. Nat Rev Immunol

2010;10(8):580–93.

4. Eisenhauer EA, Therasse P, Bogaerts J, Schwartz LH, Sargent D, Ford R, et al. New response evaluation criteria in solid tumours: Revised RECIST guideline (version 1.1). Eur J Cancer 2009;45(2):228–47.

5. Flammiger A, Bayer F, Cirugeda-Kühnert A, Huland H, Tennstedt P, Simon R, et al. Intratumoral T but not B lymphocytes are related to clinical outcome in prostate cancer. APMIS 2012;120(11):901–8.

6. Gao J, Ward JF, Pettaway CA, Shi LZ, Subudhi SK, Vence LM, et al. VISTA is an inhibitory immune checkpoint that is increased after ipilimumab therapy in patients with prostate cancer. Nat Med 2017;23(5):551–5.

7. Graff JN, Alumkal JJ, Drake CG, Thomas GV, Redmond WL, Farhad M, et al. Early evidence of anti-PD-1 activity in enzalutamide-resistant prostate cancer. Oncotarget 2016;7(33):52810–7. Kantoff PW, Higano CS, Shore ND, Berger ER, Small EJ, Penson DF, et al. Sipuleucel-Timmunotherapy for castration-resistant prostate cancer. N Engl J Med 2010;363(5):411–22.

8. Kwon ED, Drake CG, Scher HI, Fizazi K, Bossi A, van den Eertwegh AJM, et al. Ipilimumab versus placebo after radiotherapy in patients with metastatic castration-resistant prostate cancer that had progressed after docetaxel chemotherapy (CA184-043): a multicentre, randomised, double-blind, phase 3 trial. Lancet Oncol 2014;15(7):700–12.

9. Lee P, Gujar S. Potentiating prostate cancer immunotherapy with oncolytic viruses. Nat Rev Urol 2018;15(4):235–50.

10. Lopez-Bujanda Z, Drake CG. Myeloid-derived cells in prostate cancer progression: phenotype and prospective therapies. J Leukoc Biol 2017;102(2):393–406.

11. Martin AM, Nirschl TR, Nirschl CJ, Francica BJ, Kochel CM, van Bokhoven A, et al. Paucity of PD-L1 expression in prostate cancer: Innate and adaptive immune resistance. Prostate Cancer Prostatic Dis 2015;18(4):325–32.

**Ethics Approval**

The study was approved by WIRB‘s Ethics Board, IRB Tracking Number: 20183376.

#### P388 Pembrolizumab plus docetaxel and prednisone for enzalutamide- or abiraterone acetate–pretreated patients with metastatic castration-resistant prostate cancer: phase 3 KEYNOTE-921 study

##### Neal Shore, MD^1^, Daniel Petrylak, MD^2^, Mostefa Bennamoun^3^, Raffaele Ratta^4^, Josep Piulats^5^, Ben Li^6^, Charles Schloss^6^, Karim Fizazi^7^

###### ^1^Carolina Urologic Research Center, Myrtle Beach, SC, USA, Myrtle Beach, SC, United States; ^2^Smilow Cancer Hospital at Yale University, New Haven, CT, USA, New Haven, CT, United States; ^3^Institut Mutualiste Montsouris, Paris, France, Paris, France; ^4^Hopital Foch, Suresnes, France, Suresnes, France; ^5^Catalan Cancer Institute, Barcelona, Spain, Barcelona, Spain; ^6^Merck & Co., Inc., Kenilworth, NJ, USA, Kenilworth, NJ, United States; ^7^Gustave Roussy, Villejuif, France, Villejuif, France

####### **Correspondence:** Neal Shore (nshore@gsuro.com)

**Background**

Docetaxel is an established treatment for patients with metastatic castration-resistant prostate cancer (mCRPC). Pembrolizumab is a programmed death 1 inhibitor that was found to have antitumor activity as monotherapy in mCRPC. In the phase 1b/2 KEYNOTE-365 study (NCT02861573), docetaxel plus pembrolizumab and prednisone had activity in patients treated with abiraterone acetate or enzalutamide for mCRPC, warranting further evaluation of this treatment combination. KEYNOTE-921 (NCT03834506) is a randomized phase 3 trial to evaluate the efficacy and safety of pembrolizumab plus docetaxel and prednisone in chemotherapy-naïve patients who were previously treated with enzalutamide or abiraterone acetate for mCRPC and experienced progression while on therapy.

**Methods**

Approximately 1000 patients will be randomly assigned 1:1 to receive docetaxel 75 mg/m2 every 3 weeks (Q3W) plus prednisone/prednisolone 5 mg twice daily (BID) and pembrolizumab 200 mg Q3W or docetaxel 75 mg/m2 Q3W plus prednisone/prednisolone 5 mg BID plus placebo Q3W. Treatment will be stratified per previous treatment with a next-generation hormonal agent (abiraterone acetate or enzalutamide) and metastases (bone only, liver, other). Adult (≥18 years) patients with chemotherapy-naïve histologically or cytologically confirmed mCRPC who experienced progression while receiving androgen deprivation therapy (or postbilateral orchiectomy) within 6 months before screening were eligible. Patients must have experienced progression after ≥8 weeks (≥14 weeks for those with bone progression) or become intolerant after ≥4 weeks of abiraterone acetate or enzalutamide treatment (but not both) with androgen-deprivation therapy in the chemotherapy-naïve mCRPC state. Patients must have ECOG PS 0 or 1, adequate organ function, and tissue for biomarker analysis. Responses will be assessed by CT or MRI and radionuclide bone imaging per Prostate Cancer Working Group–modified RECIST v1.1 by blinded independent central review (BICR) Q9W during the first year and Q12W thereafter. Treatment will continue with docetaxel and prednisone for up to 10 cycles and with pembrolizumab for up to 35 cycles or until radiographic disease progression, unacceptable toxicity, or consent withdrawal. Primary end points are radiographic progression-free survival by BICR and overall survival. The key secondary efficacy end point is time to initiation of subsequent anticancer therapy or death. Additional secondary end points include prostate-specific antigen response rate (decline of ≥50% from baseline, measured on 2 occasions at least 3 weeks apart), time to PSA progression, and objective response rate and duration of response per PCWG-modified RECIST 1.1 as assessed by BICR. Safety and tolerability will also be reported.

**Trial Registration**

ClinicalTrials.gov, NCT03834506

**Ethics Approval**

The study and the protocol were approved by the Institutional Review Board or ethics committee at each site.

**Consent**

All patients provided written informed consent to participate in the clinical trial.

#### P389 A phase I trial of Interleukin-2 (IL-2) and Pembrolizumab (Pembro) Combination Therapy for patients with advanced renal cell carcinoma

##### Scott Tykodi, MD, PhD, Johanna Whitney, Sumia Dakhil, Eleanor Bergren, Vivian Nguyen, Samantha Kiriluk, Shailender Bhatia, MD, John Thompson, MD

###### University of Washington, Seattle, WA, United States

####### **Correspondence:** Scott Tykodi (stykodi@fredhutch.org)

**Background**

Background: Cellular immune responses play a key role modulating renal cell carcinoma (RCC) progression. IL-2 (aldesleukin) is a potent growth and differentiation factor for T and NK cells with anti-tumor activity for advanced RCC across a broad dose range. A high dose (HD) IL-2 regimen has demonstrated superior overall response rate (ORR), depth and durability of response versus lower dose alternatives. Resistance mechanisms exploited by tumors may play a dominant role in limiting the effectiveness of T-cell mediated cancer therapies. The PD-1/PD-L1 interaction is a major pathway hijacked by RCC tumors to suppress immune control. Antibody-mediated PD1 blockade with pembro results in spontaneous and durable regressions for a subset of RCC tumors (SS Tykodi et al., ASCO 2019, abstract #4570). PD1 blockade has entered clinical practice for advanced RCC as both a front-line and salvage therapy option. A favorable safety profile for PD1 blockade has encouraged exploration of novel immuno-oncology combinations.

**Methods**

Methods: This is an investigator-initiated, phase I trial of IL-2 plus pembro in patients with advanced, clear cell RCC. The study will use a 3 + 3 trial design to test three IL-2 dose levels in combination with pembro given every 3-weeks at 200 mg flat dosing. Cohorts will receive subcutaneous IL-2 given once daily, 5 days per week for 6 weeks (250,000 U/kg week 1, 125,000 U/kg weeks 2-6); or IV bolus dosing at 72,000 U/kg or 600,000 U/kg (HD IL-2) every 8 hours to a maximum of 14 doses on week 1 and 4 of a 12-week treatment course. Patients with stable or responding disease and without treatment-limiting toxicity can receive up to 3 courses of therapy. The HD IL-2 cohort will enroll an additional 9 patients to gain further insight into anti-tumor efficacy. The primary objective is to evaluate safety and tolerability for IL-2 plus pembro. The secondary objective is to assess antitumor activity by RECIST 1.1 for ORR, disease control rate, and progression free survival. Exploratory endpoints will include pretreatment tumor analysis for PD-L1 expression, and quantitation of regulatory T cell frequency in peripheral blood and tumor microenvironment.

**Trial Registration**

Trial Registration: ClinicalTrials.gov, NCT03260504

**Ethics Approval**

Ethics Approval: This study was approved by the Fred Hutchinson Cancer Research Center Institutional Review Board, approval number 9611.

#### P390 Pembrolizumab plus olaparib vs enzalutamide or abiraterone in patients with metastatic castration-resistant prostate cancer who experienced progression on chemotherapy: phase 3 KEYLYNK-010 study

##### Evan Yu^1^, Se Hoon Park^2^, Yi-Hsiu Huang^3^, Mostefa Bennamoun^4^, Lu Xu^5^, Jeri Kim^5^, Emmanuel Antonarakis, MD^6^

###### ^1^University of Washington, Seattle, WA, United STates; ^2^Samsung Medical Center, Seoul, South Korea, Seoul, Korea, Republic of; ^3^Taipei Veterans General Hospital, Taipei, Taiwan, Taipei, Taiwan, Province of China; ^4^Institut Mutualiste Montsouris, Paris, France, Paris, France; ^5^Merck & Co., Inc., Kenilworth, NJ, USA, Kenilworth, United States; ^6^Johns Hopkins University, Baltimore, MD, United States

####### **Correspondence:** Evan Yu (evanyu@u.washington.edu)

**Background**

The docetaxel-pretreated, metastatic castrate-resistant prostate cancer (mCRPC) disease state remains an unmet need for new therapeutics. The programmed death 1 (PD-1) inhibitor pembrolizumab and the polyadenosine diphosphate ribose polymerase (PARP) inhibitor olaparib have some independent antitumor monotherapy activity for mCRPC. In patients with mCRPC who were unselected for homologous recombination deficiency (HRD), promising activity was seen with the combination of pembrolizumab and olaparib in the phase 1b/2 KEYNOTE-365 study (NCT02861573), warranting further investigation in this population. KEYLYNK-010 (NCT03834519) is a randomized, open-label, phase 3 trial to evaluate the efficacy and safety of pembrolizumab plus olaparib in molecularly unselected enzalutamide-pretreated or abiraterone acetate–pretreated patients with mCRPC whose disease progressed on or after taxane chemotherapy.

**Methods**

Approximately 780 patients will be randomly assigned 2:1 to receive pembrolizumab 200 mg intravenously Q3W plus olaparib 300 mg orally twice daily or abiraterone acetate 1000 mg orally once daily plus prednisone/prednisolone 5 mg orally twice daily (for enzalutamide-pretreated patients) or enzalutamide 160 mg/day orally (for abiraterone acetate–pretreated patients). Arms will be stratified per prior treatment (abiraterone acetate/enzalutamide) and presence of measurable disease (yes/no). Eligible patients (≥18 years) must have histologically confirmed mCRPC, experienced progression while receiving androgen deprivation therapy within 6 months before screening, previously received treatment with abiraterone acetate or enzalutamide (but not both), and previously received treatment with chemotherapy (1 prior docetaxel-based regimen). Patients must have an ECOG PS of 0 or 1, adequate organ function, and tumor tissue suitable for biomarker analysis. Responses will be assessed by CT/MRI and radionuclide bone imaging per Prostate Cancer Working Group (PCWG)–modified RECIST v1.1 by blinded independent central review (BICR) Q9W during the first year and Q12W thereafter. Treatment will continue with up to 2 years of pembrolizumab (35 cycles) and olaparib or abiraterone acetate/enzalutamide until radiographic disease progression, unacceptable toxicity, or consent withdrawal. Primary end points are overall survival and radiographic progression-free survival. The key secondary efficacy end point is time to initiation of subsequent anticancer therapy. Other secondary end points are objective response rate and duration of response per PCWG-modified RECIST v1.1 by BICR, time to prostate-specific antigen progression, time to first symptomatic skeletal event, and safety and tolerability. Prognostic or predictive molecular biomarkers (eg, genomic HRD status, microsatellite instability) and patient-reported outcomes will be explored.

**Trial Registration**

ClinicalTrials.gov, NCT03834519

**Ethics Approval**

The study and the protocol were approved by the Institutional Review Board or ethics committee at each site.

**Consent**

All patients provided written informed consent to participate in the clinical trial.

#### P391 Randomized phase II trial of autologous dendritic cells loaded with autologous tumor cell antigens from self-renewing cancer cells in patients with newly diagnosed stage III or IV ovarian cancer

##### Lisa Abaid, MD^1^, Richard Friedman, MD, PhD^2^, John Brown, MD^1^, Alberto Mendivil, MD^1^, Tiffany Beck, MD^1^, Bradley Corr^3^, Leslie Randall^4^, James Mason^5^, Candace Hsieh^6^, Gabriel Nistor, MD^6^, Robert Dillman, MD, FACP^6^

###### ^1^Hoag Hospital, Newport Beach, CA, United States; ^2^Disney Family Cancer Center, Burbank, CA, United States; ^3^University of Colorado, Aurora, CO, United States; ^4^University of California Irvine, Orange, CA, United States; ^5^Scripps Green & Memorial Hospitals, La Jolla, CA, United States; ^6^AiVita Biomedical, Inc., Irvine, CA, United States

####### **Correspondence:** Robert Dillman (bob@aivitabiomedical.com)

**Background**

Despite recent advances, the 5-year survival rate for patients who present with stage III or IV ovarian cancer remains less than 40%. Standard therapy includes surgical debulking and neoadjuvant and/or adjuvant combination chemotherapy, with or without bevacizumab, with or without intraperitoneal therapy in certain stage III patients, and increasingly the use of PARP inhibitors. So far advanced ovarian cancer has been relatively refractory to anti-checkpoint therapy, presumably because of limited host anti-tumor immune responses. Adjunctive treatment with an effective vaccine could increase immune responses and improve survival.

**Methods**

This randomized phase II trial was approved by Western IRB. AV-OVA-1, patient-specific dendritic cell vaccines loaded with autologous tumor antigens from self-renewing cancer cells, is administered as an adjunctive therapy after completion of standard optimal therapy. Key eligibility criteria are a diagnosis of stage III or IV primary epithelial ovarian cancer, successful establishment of a short-term cancer cell line, a successful leukapheresis collection of monocytes, and a Karnofsky Performance Status of 70 or greater at the time of randomization, which takes place shortly after completion of primary therapy. Tumor is collected at the time of surgery from which a short-term cell line is derived. Dendritic cells are produced by incubating peripheral blood monocytes in the presence of GM-CSF and IL-4. The antigen source is a lysate of irradiated tumor cells from the cell culture. Six to seven months after tumor collection, after completion of concurrent surgery and primary systemic therapy, patients are stratified by whether they have detectable residual disease, and then randomized 2:1 to receive the dendritic cell vaccine or autologous monocytes. Both are admixed with GM-CSF and injected subcutaneously at weeks 1, 2, 3, 8, 12, 16, 20, and 24 for up to eight doses. The objective is to achieve a 50% reduction in the risk of death in the vaccine arm.

**Results**

Cell line success rate for submitted tumor samples is 22/22 with 1 in progress. A satisfactory leukapheresis product has been obtained for 14/14 patients, but was repeated for 2. 12 of a planned 99 patients have been randomized. 11 have started treatment; 7 have completed all 8 doses, 1 discontinued early for disease progression, 3 are currently in treatment. A total of 77 doses have been administered. No significant toxicity directly attributed to the vaccine has been reported.

**Conclusions**

Although logistically complex, this patient-specific vaccine approach is feasible, and has been well-tolerated. [NCT02033616]

**Trial Registration**

ClinicalTrials.gov NCT02033616

**Ethics Approval**

This study was approved by the Western Institutional Review Board 20171661

#### P392 Phase 1 combination study of the CHK1 inhibitor prexasertib (LY2606368) and anti-PD-L1 antibody LY3300054, in patients with high-grade serous ovarian cancer and other advanced solid tumors

##### Khanh Do^1^, Claire Manuszak^1^, Sarah Kelland^1^, Allison Powers^1^, Adrienne Anderson^1^, Alona Muzikansky^2^, Andrew Wolanski^1^, Mariano Severgnini, MSc^1^, Geoffrey Shapiro, MD, PhD^1^, Khanh Do, MD^1^

###### ^1^Dana-Farber Cancer Institute, Boston, MA, United States; ^2^Massachusetts General Hospital, Boston, MA, United States

####### **Correspondence:** Khanh Do (Khanh_Do@dfci.harvard.edu)

**Background**

Ovarian cancers are characterized by defects in DNA damage repair and high levels of replication stress, creating susceptibility to inhibition of checkpoint kinase 1 (CHK1). CHK1 inhibition results in intratumoral DNA damage that can drive T cell infiltration, as well as PD-L1 expression. Combined CHK1 inhibition and immune checkpoint blockade therefore has the potential to enhance T cell activation against tumors.

**Methods**

We conducted an open-label phase 1 study of prexasertib-mediated CHK1 inhibition combined with LY3300054-mediated PD-L1 blockade following a 3+3 design evaluating 3 administration schedules: lead-in of LY3300054 alone (Arm A), lead-in of prexasertib alone (Arm B), and combined LY3300054 and prexasertib at outset (Arm C). Both agents were administered on days 1 and 15 of a 28-day cycle. The MTD was defined as the highest dose level at which less than one-third of at least 6 patients experienced a DLT during C0+C1. Flow cytometry of peripheral blood mononuclear cells was performed for analysis of T cell subsets. Plasma cytokine and chemokine analyses were conducted using the Luminex platform. Patients enrolled to the currently ongoing expansion phase of the study undergo mandatory tumor biopsies during C0 and on C1D16 after the combination.

**Results**

Fifteen patients have been treated in the dose escalation phase. The combination of both agents is tolerable at the RP2D with prexasertib at 105mg/m2 IV on days 1 and 15 in combination with LY3300054 at 700mg flat dosing. Two DLTs occurred, including febrile neutropenia (Arm C) and prolonged grade 4 neutropenia lasting > 5 days (Arm B). Most common drug-related adverse events included leukopenia, neutropenia, thrombocytopenia, and anemia. Confirmed partial responses have been observed in 2 patients with CCNE1-amplified HGSOC ongoing for 9 and 10 months, respectively. One additional CCNE-1 amplified HGSOC patient has had prolonged SD for 11 months. Preliminary data on T-cell subset analysis and cytokine profile show immune modulatory effect of prexasertib, confirming proof-of-mechanism.

**Conclusions**

Full-dose prexasertib in combination with immune checkpoint blockade is tolerable and has preliminary clinical activity in patients with HGSOC with durable responses. An expansion cohort in this population is currently being enrolled utilizing schedule B. Comprehensive characterization of the immune microenvironment will be performed in paired tumor biopsies, with attention to pharmacodynamic proof-of-mechanism endpoints, including T cell infiltration and PD-L1 expression and their correlation with the induction of DNA damage. Additionally, immune signatures will be correlated with genomic profile and response duration.

**Ethics Approval**

This study was approved by Dana-Farber Cancer Institute's Ethics Board.

**Consent**

Written informed consent was obtained from the patient for publication of this abstract and any accompanying images. A copy of the written consent is available for review by the Editor of this journal.

#### P393 Single agent activity of a novel PD-1 inhibitor AGEN2034 in recurrent ovarian cancer:Subset analysis of phase I dose escalation NCT03104699 study

##### David O'Malley^1^, John Hays^1^, Charles Drescher^2^, Jasgit Sachdev^3^, Wilberto Nieves-Neira^4^, Breelyn Wilky^5^, Marylin Huang^6^, Kathleen Moore^7^, Waldo Ortuzar^8^, Anna Wijatyk^8^, Hagop Youssoufian^8^, Remigiusz Kaleta, MD^8^, Inbal Sapir^8^, Christopher Dupont, PhD^8^, Irina Shapiro^8^, Debra Richardson, MD^7^

###### ^1^The James Cancer Center Hospital, Columbus, OH, United States; ^2^Swedish Cancer Institute, Seattle, WA, United States; ^3^HonorHealth Research Institute, Scottsdale, AZ, United States; ^4^Northwestern University, Stroger Hospital, Chicago, IL, United States; ^5^University of Colorado Anschultz Medica, Aurora, CO, United States; ^6^Sylvester Comprehensive Cancer Center, Miami, FL, United States; ^7^Stephenson Cancer Center, Oklahoma City, OK, United States; ^8^Agenus Inc, Lexington, MA, United States

####### **Correspondence:** Christopher Dupont (Christopher.Dupont@Agenusbio.com)

**Background**

AGEN2034 is a novel, fully human monoclonal immunoglobulin G4 (IgG4) antibody, designed to block PD-1 from interacting with its ligands PD-L1 and PD-L2 with high affinity. The overall objective of the study was to assess safety, MTD, and pharmacokinetic (PK) and pharmacodynamic (PD) characteristics of AGEN2034 monotherapy in patients with advanced, refractory malignancies.

**Methods**

Between April 2017 - April 2019, 50 patients with advanced solid tumors were enrolled in a phase 1 dose escalation study treated with infusion of AGEN2034 every 2 weeks at the dose range of 1-10 mg/kg. Within the study population a subset of patients with heavily pretreated recurrent epithelial ovarian cancer was identified.

**Results**

Twelve patients with recurrent epithelial ovarian cancer were enrolled in the Phase I dose escalation. Median age was 58 years (range 41-77) with ECOG 0-1 and a median of 4 prior lines of systemic treatment (ranging from 1 to 8). All 12 patients received platinum-based treatment prior to study entry. No DLTs were observed. The most frequent AEs regardless of relationship to study drug were fatigue (7 patients), nausea (7 patients) and UTI (5 patients). Overall, 7 patients had AEs of > grade 3, 4 of which were considered related to study drug by the investigator. Pharmacodynamic assessments included immune phenotyping at the periphery where immune activation was observed following AGEN2034 treatment. In this subset of recurrent ovarian cancer patients, 1 of 12 patients developed a durable partial response (42 wks) at the lowest dose level (1 mg/kg), 8 patients demonstrated at least stable disease lasting 8.7 -65.7 weeks, with 5 of them meeting the DCR criteria of at least 12 weeks of duration. Four patients demonstrated progressive disease at the first on treatment tumor evaluation.

**Conclusions**

AGEN2034, a PD-1 inhibitor, is well tolerated with no DLTs observed at all dose levels evaluated. The clinical activity and safety observed in the recurrent ovarian cancer subset were consistent with the overall Phase 1 study population. Biomarker evaluations (including PD-L1 status) are ongoing.

**Ethics Approval**

20170314 - IRB tracking for Copernicus

**Consent**

Written informed consent was obtained from the patient for publication of this abstract and any accompanying images. A copy of the written consent is available for review by the Editor of this journal.

#### P394 A Phase 1 study of INCMGA00012, a PD-1 inhibitor, in patients with advanced solid tumors: Preliminary results for patients with advanced cervical cancer (POD1UM-101)

##### Janice Mehnert, MD^1^, Luis Paz Ares, MD, PhD^2^, Joanna Pikiel, Dr n med^3^, Udai Banerji, PhD^4^, Anna Kryzhanivska, MD^5^, Nehal Lakhani, MD, PhD^6^, Sebastian Ochsenreiter, Dr med^7^, Tobias Arkenau, MD, PhD^8^, Nawel Bourayou, MD^9^, Deanna Kornacki, PhD^9^, Chuan Tian, PhD^9^, Thomas Condamine, PhD^9^, Itziar Gardeazabal González^10^

###### ^1^Rutgers Cancer Institute of New Jersey, New Brunswick, NJ, United States; ^2^Hospital Universitario, Madrid, Spain; ^3^Szpitale Pomorskie Sp. z o.o., Gdansk, Poland; ^4^Royal Marsden NHS Foundation Trust, Sutton, United Kingdom; ^5^Regional Clinical Oncology Center, Ivano-Frankivsk, Ukraine; ^6^START Midwest, Grand Rapids, MI, United States; ^7^Charité Comprehensive Cancer Center, Berlin, Germany; ^8^Sarah Cannon Institute, London, United Kingdom; ^9^Incyte Corporation, Wilmington, DE, United States; ^10^Hospital Vall d’Hebron, Barcelona, Spain

####### **Correspondence:** Janice Mehnert (mehnerja@cinj.rutgers.edu)

**Background**

Background

INCMGA00012 is an investigational humanized, hinge-stabilized IgG4 monoclonal antibody that binds to PD-1. At all doses tested, INCMGA00012 has an acceptable tolerability profile with no dose-limiting toxicities or maximum tolerated dose. A dose of 3 mg/kg Q2W was initially selected for the tumor specific cohorts. Multiple fixed doses were also examined in this study. The 500 mg Q4W and 375 mg Q3W doses are selected for further development based on favorable pharmacokinetics and safety.

**Methods**

Methods

The initial expansion phase contained 4 tumor-specific cohorts (endometrial [unselected], cervical, soft tissue sarcoma, and non-small cell lung) treated for up to 2 years. Eligible patients presented with a histologically proven, unresectable locally advanced or metastatic tumor, ECOG performance status (PS) ≤1, disease progression during or following ≤5 prior treatments, measurable disease per RECIST v1.1, and no prior treatment with immune checkpoint inhibitors. The primary endpoint is safety (using CTCAE v4.03 grading). Confirmed best overall response rate and duration of response were evaluated by RECIST v1.1 (investigator's assessment). Treatment past progression was allowed for patients experiencing clinical benefit. Preliminary safety and efficacy results for patients with unresectable locally advanced or metastatic cervical cancer are presented.

**Results**

Results

As of 24 APR 2019, 35 patients with cervical cancer were treated with 3 mg/kg INCMGA00012. Median age was 51 (29-81) years, 88.6% were white, 48.6% had an ECOG PS of 1. All patients were pretreated with at least 1 prior platinum-based chemotherapy for recurrent or advanced disease, 91.4% were treated with radiotherapy, and 62.9% underwent surgery. Median drug exposure was 4.4 (0.03-16.0) months. Fourteen patients (40.0%) experienced Grade (G) 3/4 AEs regardless of causality. Seven patients (20.0%) had immune-related AEs (colitis [G2, n=1; G3, n=2], infusion-related reaction [G1, n=1; G3, n=1], diarrhea [G1], hyperthyroidism [G2], and maculopapular rash [G3]). Three patients with colitis discontinued study treatment. No treatment-related deaths occurred. Confirmed responses per RECIST v1.1 were observed in 6/31 (19.4%) response evaluable patients, with 1 patient having a confirmed CR. Median duration of response was not reached as 5/6 patients remain on treatment (10.3, NE months). An additional 12 patients had stable disease for an overall disease control rate of 58.1% (18/31).

**Conclusions**

Conclusions

INCMGA00012 has been generally well tolerated with evidence of significant and durable antitumor activity in platinum-refractory cervical cancer. These data support further development of INCMGA00012 in cervical cancer.

**Trial Registration**

NCT03059823, 2017-000865-63

**Ethics Approval**

The study was approved by institutional review boards or independent ethics committees of participating institutions.

#### P395 Phase 2/3 open-label trial of enoblituzumab in combination with MGA012, with and without chemotherapy, in the treatment of patients with recurrent or metastatic head and neck squamous cell carcinoma

##### Fernanda Arnaldez^1^, Charu Aggarwal, MD MPH^2^, Scott Currence^1^, Jan Baughman, MPH^1^, Paul Moore, PhD^1^, George Blumenschein, MD^3^, Jon Wigginton, MD^1^, Robert Ferris, MD, PhD^4^

###### ^1^MacroGenics, Inc., Rockville, MD, United States; ^2^Abramson Cancer Center, Philadelphia, PA, United States; ^3^MD Anderson Cancer Center, Houston, TX, United States; ^4^UPMC Hillman Cancer Center, Pittsburgh, PA, United States

####### **Correspondence:** Fernanda Arnaldez (farnaldez@gmail.com)

**Background**

Squamous cell carcinoma of the head and neck (SCCHN) accounts for >500,000 new cases and nearly 300,000 deaths annually worldwide as of 2012 [1]. Patients with recurrent/metastatic (R/M) SCCHN have a poor prognosis with median overall survival (OS) of Enoblituzumab is an investigational Fc-modified monoclonal antibody that binds B7-H3, which is over-expressed in a wide range of cancers including SCCHN [4], but not in most normal tissues. It has increased affinity for the activating FcγR IIIA (CD16A) and decreased affinity for the inhibitory FcγRIIB (CD32B). The engineered Fc domain confers enoblituzumab with target-specific antibody-dependent cellular cytotoxicity in vitro and anti-tumor activity in preclinical studies, and in vivo and clinical data suggest that Fc-optimized antibodies such as enoblituzumab can engage both innate and adaptive immunity as mediators of anti-tumor activity [6]. Enoblituzumab was well tolerated in a Phase 1 monotherapy trial with no maximum tolerated dose defined up to 15 mg/kg.

A recent study of enoblituzumab combined with pembrolizumab showed this combination is feasible and well tolerated with minimal additive toxicity [5]. While studies of monotherapy pembrolizumab in this population report responses below 17% [6], the overall response rate of PD-1/PD-L1 inhibitor-naïve patients (post platinum) receiving enoblituzumab plus pembrolizumab was 33% (6/18) including 1 confirmed CR and 5 confirmed PRs [5]. This suggests a cooperative mechanism and provides a rationale for further development of this combination in patients with recurrent/metastatic SCCHN.

MGA012 (also known as INCMGA00012) is an investigational anti-PD-1 monoclonal antibody with a tolerable safety profile and efficacy signal consistent with other agents in its class [7] demonstrated in early studies.

**Methods**

This is a Phase 2/3, randomized, open label study in first-line treatment of patients with R/M SCCHN not curable by local therapy (Figure 1). We hypothesize that combining enoblituzumab and PD-1 inhibition (with or without chemotherapy) will improve objective response rates and OS compared to pembrolizumab/chemotherapy in patients in R/M SCCHN.

Approximately 200 patients will be randomized in a 1:1:1:1 ratio to one of four treatment arms to select the preferred enoblituzumab combination treatment for further evaluation based primarily on ORR. In subsequent Phase 3 portion, the selected enoblituzumab/MGA012 regimen (with or without chemotherapy) will be compared to pembrolizumab and chemotherapy with an endpoint of OS.

**Trial Registration**

To be registered on clinicaltrials.gov

**References**

1. Siegel R, Naishadham D, and Jemal A, Cancer statistics, 2013. CA Cancer J. Clin, 2013. 63(1): p. 11-30.

2. Price KA and Cohen EE, Current treatment options for metastatic head and neck cancer. Curr Treat Options Oncol, 2012. 13(1): p. 35-46.

3. Keynote 048. 1200/JCO.2019.37.15_suppl.6000 Journal of Clinical Oncology 37, no. 15_suppl (May 20, 2019) 6000-6000.

4. Collins M, Ling V, and Carreno BM, The B7 family of immune-regulatory ligands. Genome Biol, 2005. 6(6): p. 223.

5. Aggarwal C et al, Open-Label, Dose Escalation Study of Enoblituzumab in Combination with Pembrolizumab in Patients with Select Solid Tumors. 33rd Annual Meeting of The Society for Immunotherapy of Cancer Washington, DC, USA November 7–11, 2018

6. Cohen EE, Harrington KJ, Tourneau C, Dinis J, Licitra L, Ahn M, et al., Head and Neck Cancer, Excluding Thyroid. ESMO, 2017. 28.

7. Mehnert J et. At. 33rd Annual Meeting of The Society for Immunotherapy of Cancer Washington, DC, USA November 7–11, 2018

**Ethics Approval**

Each institution will obtain Ethics Board approval prior to enrollment

**Fig. 1 (abstract P395).**
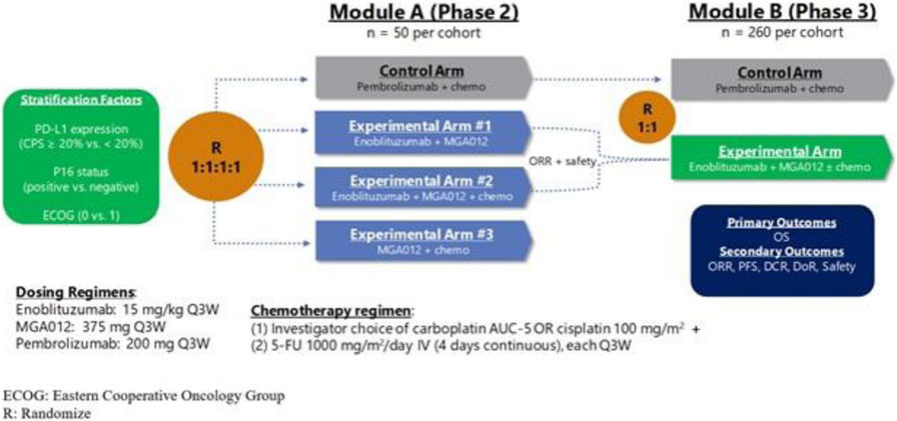
Study Schema

#### P396 A phase 2 efficacy and safety trial of ADU-S100 and pembrolizumab in adults with head and neck cancer

##### Ezra Cohen, MD^1^, Robert Ferris, MD, PhD^2^, Douglas Adkins, MD^3^, Dan Zandberg, MD^2^, Arkadiusz Dudek, MD, PhD^4^, Matthen Mathew^5^, Lara Dunn, MD^6^, Juneko Grilley-Olson, MD^7^, Ammar Sukari, MD^8^, Rebecca Redman, MD^9^, Julie Bauman, MD, MPH^10^, John Kaczmar, MD^11^, Lisle Nabell, MD^12^, Nabil Saba, MD^13^, Eric Nadler, MD^14^, Young Kim, MD^15^, Ranee Mehra, MD^16^, Nitya Nair, PhD^17^, Somayeh Honarmand, MS^17^, Richard Cutler Jr.^17^, Barbara Burtness, MD^18^

###### ^1^University of California at San Diego, La Jolla, CA, United States; ^2^University of Pittsburgh Medical Center, Pittsburgh, PA, United States; ^3^Washington University of St. Louis, St. Louis, United States; ^4^HealthPartners Regions Cancer Care Center, St. Paul, MN, United States; ^5^Columbia University Medical Center, New York, United States; ^6^Memorial Sloan-Kettering Cancer Center, New York, NY, United States; ^7^University of North Carolina at Chapel Hill, Chapel Hill, NC, United States; ^8^Wayne State University School of Medicine, Detroit, MI, United States; ^9^University of Louisville, Louisville, United States; ^10^The University of Arizona Cancer Center, Tucson, United States; ^11^MUSC Hollings Cancer Center, Charleston, SC, United States; ^12^University of Alabama at Birmingham, Birmingham, United States; ^13^Emory University, Atlanta, GA, United States; ^14^Baylor Charles A. Sammons Cancer Center, Dallas, United States; ^15^Vanderbilt University School of Medicine, Nashville, United States; ^16^University of Maryland, Greenebaum Comprehensive Cancer Center, Baltimore, United States; ^17^Aduro Biotech, Berkeley, CA, United States; ^18^Yale University School of Medicine, New Haven, CT, United States

####### **Correspondence:** Richard Cutler Jr. (rcutler@aduro.com)

**Background**

Immune checkpoint inhibitors such as the PD-1 blocking antibody pembrolizumab have demonstrated marked improvements in duration of response and long-term survival over standards of care (SOC) in head and neck squamous cell carcinoma (HNSCC) and other cancers. However, the significant percentage of patients who are nonresponsive to these immunotherapies (primary resistance) or experience disease relapse following an acquired immune resistance mechanism (secondary resistance) [1] highlights the need for new therapies. As tumor responsiveness to immunotherapy may depend, in part, on the immunophenotype of the tumor microenvironment (TME) [2-5], one exploratory approach to establish, re-establish, or enhance active immune surveillance conditions within the TME is to inject innate immune modulators directly into the tumor to promote an adaptive tumor-specific immune response. ADU-S100 (MIW815) is a novel synthetic cyclic dinucleotide that activates the stimulator of interferon genes (STING) pathway within the TME leading to activation of tumor-resident APCs and priming of tumor antigen specific CD8+ T cells. Direct activation of STING via intratumoral injection of ADU-S100 (MIW815) has been shown to overcome active tolerance mechanisms through stimulation of resident leukocyte populations. Preclinical models indicate that survival and local tumor shrinkage were significantly enhanced when ADU-S100 (MIW815) was administered with an anti-PD-1 antibody, suggesting the PD-1 blockade may act synergistically with concomitant STING activation. In phase 1 trials, tumor shrinkage and durable responses have been observed after treatment with S100 alone or in combination with a PD-1 inhibitor. The primary objective of this trial is to evaluate the clinical efficacy of intratumoral ADU-S100 (MIW815) when administered in combination with pembrolizumab.

**Methods**

This open-label, multicenter phase 2 clinical trial (NCT03937141) aims to enroll 33 adults with PD-L1 positive, recurrent or metastatic HNSCC for which pembrolizumab is indicated as SOC in the first-line setting. Patients with at least one lesion that is accessible for repeat intratumoral injection and can provide tumor tissue for eligibility determination and biomarker analyses will receive intravenous infusions of pembrolizumab (200 mg) at Day 1 and intratumoral injections of ADU-S100 (MIW815) (800 mcg/lesion) at Day 1 and 8 in 21-day dosing cycles up to 35 cycles, or until criteria for treatment discontinuation are met. The primary endpoint is the objective response per Response Evaluation Criteria in Solid Tumors v1.1. Key secondary endpoints include occurrence and severity of treatment-emergent adverse events and changes from baseline in safety assessments. This trial is currently in the recruitment phase.

**References**

1. Chen PL, Roh W, Reuben A, et al. Analysis of Immune Signatures in Longitudinal Tumor Samples Yields Insight into Biomarkers of Response and Mechanisms of Resistance to Immune Checkpoint Blockade. Cancer Discov 2016;6:827-37.

2. Gajewski TF, Fuertes MB, Woo SR. Innate immune sensing of cancer: clues from an identified role for type I IFNs. Cancer Immunol Immunother 2012;61:1343-7.

3. Gajewski TF, Schreiber H, Fu YX. Innate and adaptive immune cells in the tumor microenvironment. Nat Immunol 2013;14:1014-22.

4. Gajewski TF, Woo SR, Zha Y, et al. Cancer immunotherapy strategies based on overcoming barriers within the tumor microenvironment. Curr Opin Immunol 2013;25:268-76.

5. Woo SR, Corrales L, Gajewski TF. The STING pathway and the T cell-inflamed tumor microenvironment. Trends Immunol 2015;36:250-6.

**Ethics Approval**

This study was approved or is currently under review by an institutional review board at each site.

#### P397 Interim analysis of the combination of durvalumab and cetuximab in a phase II trial of patients with recurrent and metastatic head and neck squamous cell carcinoma

##### Shuchi Gulati, MD^2^, Sarah Palackdharry^1^, Layne Weatherford^1^, Sarah Wilson^1^, Shireen Desai^1^, Aubrey Steele^1^, Kashif Riaz^1^, Vinita Takiar^3^, Trisha Draper^1^

###### ^1^University of Cincinnati, Cincinnati, OH, United States; ^2^University of Cincinnati Cancer Institute, Cincinnati, OH, United States; ^3^University of Cincinnati/Barrett Cancer, Cincinnati, OH, United States

####### **Correspondence:** Shuchi Gulati (gulatisi@ucmail.uc.edu)

**Background**

Cetuximab ( IgG1 isotype monoclonal antibody) monotherapy is considered standard of care therapy for recurrent and metastatic head and neck squamous cell carcinoma (HNSCC).[1] Cetuximab results in NK cell mediated ADCC and inhibition of the EGFR signaling pathway.[1] NK cell activation increases secretion of plasma transforming growth factor β (TGFβ) and interleukin 10 (IL-10), resulting in increased expression of PD-1 on T cells and PD-L1 expression on tumor cells. Blocking the PD-1/PD-L1 check-point receptor pathway increases the cytotoxic response of NK cells in mice.[2] Therefore, it was hypothesized that cetuximab and PD-1/PD-L1 blockade would be synergistic. Here we report our findings on the combination of cetuximab with a PD-L1 inhibitor, durvalumab on T cells, NK cells and cytokines from a phase II open-label single site clinical trial in HNSCC patients with recurrent or metastatic disease. (NCT03691714.)

**Methods**

Interim analysis includes a total of 15 enrolled patients. Using flow cytometry and Luminex, we evaluated the immune cell phenotypes and cytokine profiles of peripheral blood in patients before and after treatment with the combination of cetuximab and durvalumab with respect to overall response rate (ORR).

**Results**

Fourteen patients who received at least 2 cycles of treatment were included in the interim analysis. Median age was 66 years (range 47-75), majority of patients were male (79%). Eight patients (57%) had received 1 line of prior chemotherapy, while 3 (21%) had received 2 prior chemotherapies. Seven patients (50%) had received prior immunotherapy. Of the 7 patients who had next generation sequencing completed, 1 was PDL1 positive (14%), all had MSI-high tumors (100%) and all had TP53 mutations (100%). One patient achieved a partial response, and 3 were noted to have stable disease; overall response rate (ORR) was noted as 27%. No grade 3/ 4 adverse events attributable to study drugs were reported. Results from peripheral blood flow cytometry analyses showed an increase in cytokine producing NK cells and CD3+ T cells in all responders. Luminex assay revealed that all responders had a drop in their GM-CSF levels, and an increase in their TNF-alpha and CXCL-10 levels.

**Conclusions**

The combination of cetuximab and durvalumab results in a pro-tumorigenic profile with a modest ORR.

**References**

1. Ferris, R. L. et al. Rationale for combination of therapeutic antibodies targeting tumor cells and immune checkpoint receptors: Harnessing innate and adaptive immunity through IgG1 isotype immune effector stimulation. Cancer Treat. Rev. 63, 48–60 (2018).

2. Hsu, J. et al. Contribution of NK cells to immunotherapy mediated by PD-1/PD-L1 blockade. J. Clin. Invest. 128, 4654–4668

**Ethics Approval**

The study was approved by University of Cincinnati's Ethics Board, approval no. FWA #: 000003152

#### P398 miRNA-A and programmed death ligand 1 (PD-L1) expression in oral squamous cell carcinoma

##### Hong Hyung, MD PhD^1^, Yoon Ho Ko^2^, Lee Hee JIn^2^, Sang Hoon Jeon^2^

###### ^1^Catholi, Seoul, Korea, Republic of; ^2^Catholic Universtiy, Uijeongbu-si, Korea, Republic of

####### **Correspondence:** Yoon Ho Ko (koyoonho@catholic.ac.kr)

**Background**

Overexpression of PD-L1 in cancer cells is involved not only in the immune evasion but also in cancer progression. Increasing evidence indicates that dysregulation of miRNA(miR)s contributes to the pathogenesis of oral squamous cell carcinoma (OSCC). Here, we identified miR-C that regulate the expression of PD-L1 and elucidated whether miR-C affects chemotherapy responsiveness via regulating PD-L1 expression in OSCC.

**Methods**

To further verify the role of miRNAs on PD-L1 in OSCC, we carried out the functional study in human head and neck cancer cell line CAL27 and YD8. After transfection with scrambled miRNA-A, B, C (Scr), miRNAs-A, B, C for 48 h, PD-L1 mRNA and PD-L1 protein levels were assessed by RT-qPCR and western blot analysis. To perform EGFP reporter assay, cells were transfected with miRNA-C and reporter constructs, containing the putative PD-L1 3’-UTR target sites, along with a control vector, EGFP levels were assessed by western blotting. Cell viability was assessed after treatment with 5-FU for 72 h using by MTT solution. GAPDH mRNA and its protein level were used for normalization and as a loading control.

**Results**

We investigated various miRs that were negatively correlated with PD-L1 in The Cancer Genome Atlas head and neck squamous cell carcinoma (HNSCC) dataset and could recognize PD-L1 3'-UTR by analyzing TargetScan. Three miRs (miR-A, miR-B, miR-C) were identified which had not been reported to be as associated with PD-L1 before. EGFP reporter assay of only miR-C out of three miRs showed a decrease in the relative PD-L1 expression. This would indicate that only miR-C can recognize target sites in the 3’-UTR of PD-L1 mRNA in OSCC cells. Overexpression of miR-C induced the decrease of PD-L1 mRNA and protein (Figure 1A,1B). The sensitivity of CAL27 and YD8 cells to 5-FU was increased when miR-C was overexpressed (Figure 2). Also, the level of cleaved PARP, one of the apoptotic markers, was increased according to miR-A overexpression (Figure 3).

**Conclusions**

Our data suggest that miR-C can regulate PD-L1 expression by targeting PD-L1 mRNA, and our present findings shed new light on the complex regulation of PD-L1 in human tumors, and on miR-C in cancer immuno-based therapy.

**Fig. 1 (abstract P398).**
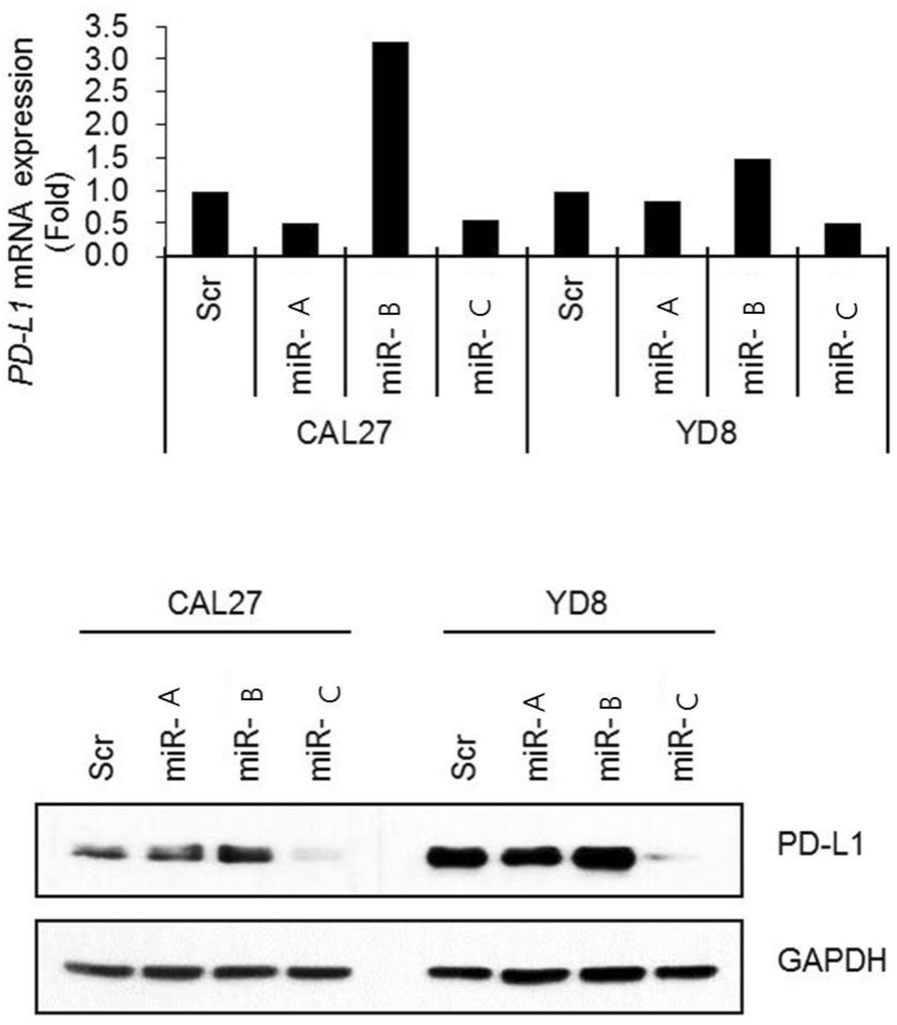
A and B. See text for description

**Fig. 2 (abstract P398).**
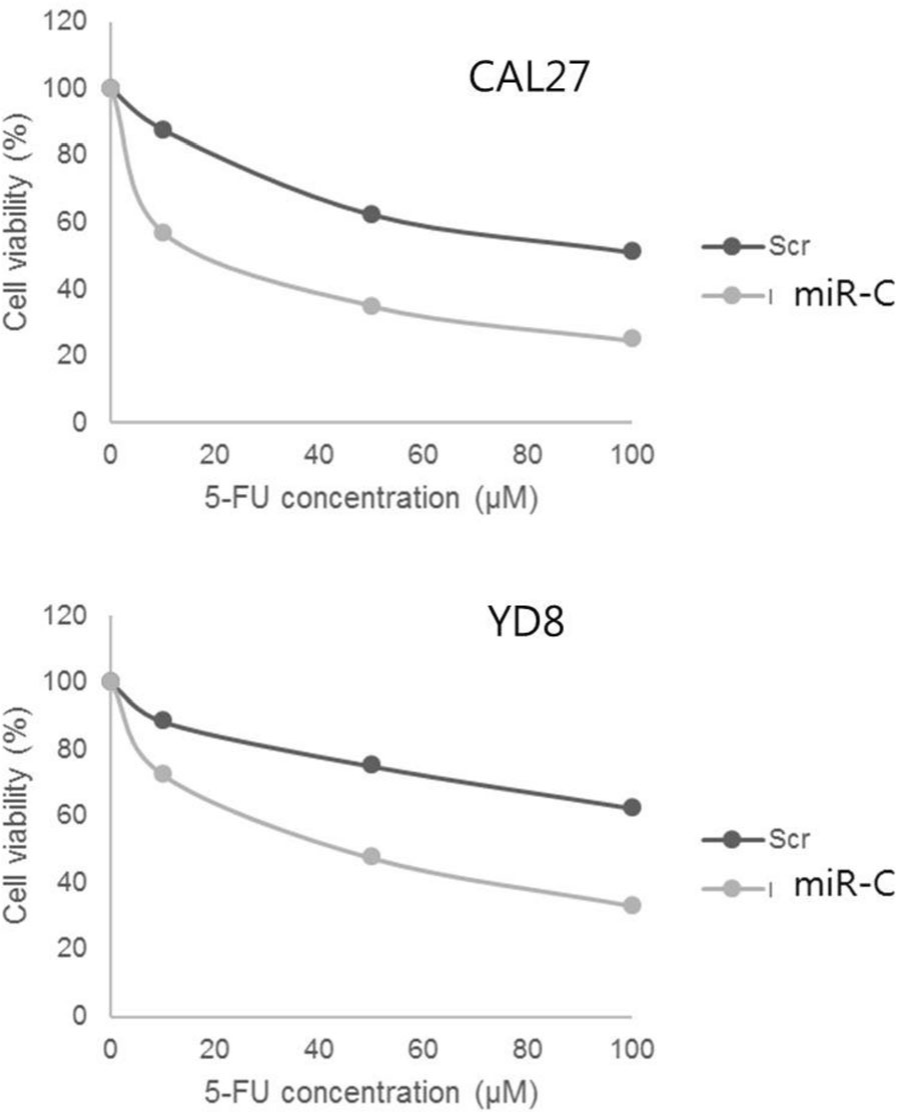
See text for description

**Fig. 3 (abstract P398).**
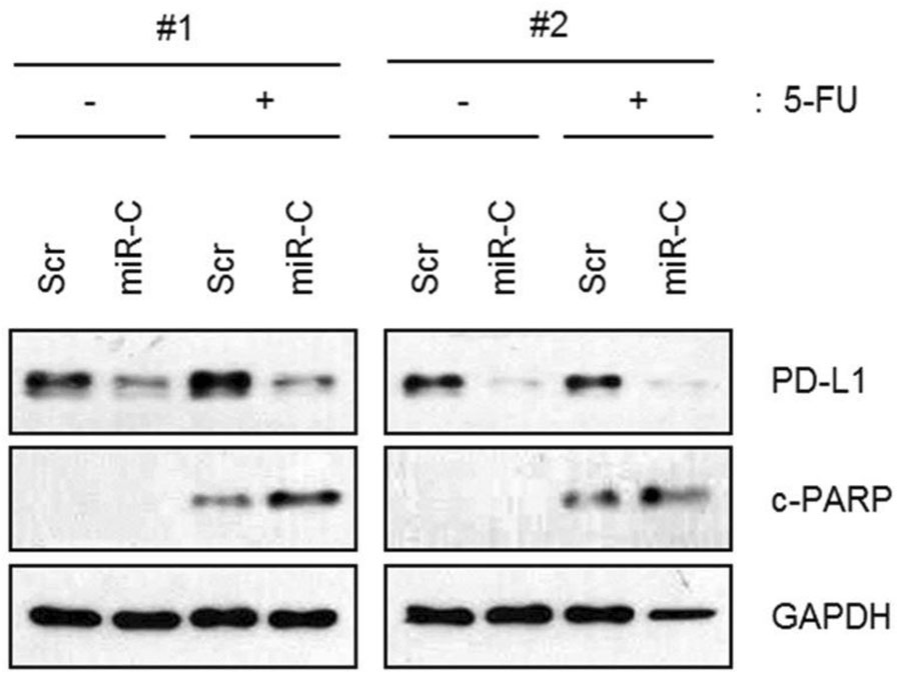
See text for description

#### P399 Instructive conclusions from performing immune correlatives on IO trials: a meta-analysis of patient tumor and blood samples

##### Patrick Lizotte, PhD, Megan Cavanaugh, Melissa Jean, Cloud Paweletz, PhD

###### Dana-Farber Cancer Institute, Boston, MA, United States

####### **Correspondence:** Cloud Paweletz (CloudP_Paweletz@DFCI.HARVARD.EDU)

**Background**

Blood-based immune phenotyping provides a cost-effective, minimally invasive, longitudinal, and logistically convenient assay that may provide two critical pieces of information for cancer patients receiving immunotherapy: 1.) if the therapeutic is working as intended, and 2.) if there will be any benefit to the patient.

**Methods**

Our group has performed multi-parameter flow cytometric immune-profiling on hundreds of blood and tumor samples from patients treated with immunotherapy. We have compiled this dataset of immune correlatives from patients receiving immune checkpoint blockade enrolled on Dana-Farber clinical trials in the following cancers: thyroid cancer (tumor n = 24; blood n = 180) , head & neck squamous cell carcinoma (tumor n =18; blood n = 118) , mesothelioma (tumor n = 41) , non-small cell lung cancer (tumor n = 34) , and gastric-esophageal cancer (tumor n= 55).

**Results**

Our meta-analysis of blood and tumor flow-based immune profiling confirms that tumor tissue remains the benchmark for determining therapeutic efficacy to immune checkpoint blockade. However, our profiling of blood has produced several generalizable findings: 1.) IO-relevant markers are generally expressed at very low levels by circulating T cells and only subtly change after treatment, 2.) the abundance of different leukocyte lineages is also largely static, 3.) serial blood profiling at timepoints later than three weeks after initiation of treatment are minimally informative, 4.) some immune parameters significantly correlated with therapeutic efficacy are simply indicative of immune-related adverse events, which are historically associated with improved therapeutic efficacy and also easy to diagnose without immune correlatives, and 5.) it is impossible to delineate between reinvigorated or activated tumor-specific circulating T cells and global reinvigoration or activation of, for instance, bystander T cells without more sophisticated and costly TCR deconvolution.

**Conclusions**

We conclude that blood-based immune phenotyping can be, in some contexts, highly informative, but invites over-analysis.

**Trial Registration**

NCT03246958, NCT03341936, NCT03425331, NCT02971956, NCT03075527, NCT02635061

**Ethics Approval**

The present studies were reviewed and approved by the Dana-Farber/Harvard Cancer Center (DF/HCC) institutional review board (Boston, Massachusetts, USA) and all were performed in accordance with relevant guidelines and regulations.

**Consent**

Written informed consent was obtained from all subjects prior to participation in these studies. Informed consent by patients to DF/HCC protocol 02-180 enabled collection of clinical and demographic data, and genomic characterization.

#### P400 Sitravatinib and Nivolumab for resectable Oral cavity squamous cell carcinoma Window of opportunity study (SNOW)

##### Marc Oliva Bernal, MD^1^, Douglas Chepeha, MD^1^, Amy Prawira, MD^2^, Anna Spreafico, MD PhD^1^, Scott Bratman, MD^1^, Tina Shek, MD^3^, John De Almeida, MD^1^, Ivan Yeung, MD^3^, Aaron Hansen^1^, Andrew Hope, MD^1^, David Goldstein, MD^1^, Ralph Gilbert, MD^1^, Doug Vines, BSc, MRT(N), CNMT^3^, Patrick Gullane^1^, Dale Brown, MD^1^, Ilan Weinreb, MD^1^, Bayardo Perez-Ordoñez, MD^1^, Trevor Pugh, PhD^4^, Pamela Ohashi, PhD^4^, Ben Wang, PhD^4^, Jonathan Irish, MD^1^, Hirak Der-Torossianh, MD^5^, Isan Chen, MD^5^, Lillian Siu, MD^1^

###### ^1^Princess Margaret Cancer Centre, University of Toronto, Toronto, Canada; ^2^The Kinghorn Cancer Centre, St Vincent’s Hospital, Darlinghurst, Australia; ^3^Princess Margaret Cancer Centre, University of Toronto; Quantitative Imaging for Personalized Cancer Medicine, TECHNA Institute, University Health Network., Toronto, Canada; ^4^University of Toronto, Toronto, Canada; ^5^Mirati Therapeutics, San Diego, CA, United States

####### **Correspondence:** Lillian Siu (lillian.siu@uhn.ca)

**Background**

Sitravatinib is a receptor tyrosine kinase inhibitor that blocks TAM and VEGF family of receptors. Based on non-clinical findings, it is predicted to increase M1 macrophage response and decrease immunosuppressive Tregs and MDSCs in the tumor microenvironment. Sitravatinib combined with nivolumab showed a safe toxicity profile and promising antitumor activity in non-small cell lung cancer patients (pts) progressing on anti-PD-1 agents [1]. The CheckMate-358 study revealed that preoperative nivolumab was safe and active in oral cavity squamous cell carcinoma (OCSCC) [2]. We hypothesize that preoperative sitravatinib and nivolumab have synergistic immunogenic and antitumor effects in OCSCC.

**Methods**

SNOW is an investigator-initiated, single-center, non-randomized, window-of-opportunity study evaluating preoperative sitravatinib and nivolumab in pts with resectable, previously untreated OCSCC. Pts with T2-4a, N0-2 or T1 (>1cm)-N2 tumors as per AJCC 8th edition, ECOG >/=1, adequate organ function and no autoimmune disorders are eligible. Figure 1 summarizes study design and treatment. Primary objective is to evaluate the immune and pharmacodynamic effects of the treatment combination. Secondary objectives are: (a) safety, including rate of treatmen-related adverse events (TRAEs), surgery completion within the planned window and postoperative complications; (b) antitumor activity, including clinical and pathologic responses; rate of pathological extranodal extension (ENE) and positive margins; (c) pharmacokinetics/pharmacodynamics of sitravatinib alone and combined with nivolumab. Correlative studies include: immune biomarkers by multiplex immunohistochemistry, tumor and blood immunophenotyping; tumor genome and transcriptome analyses; intratumoral hypoxia changes using 18FAZA-PET. Preliminary results as of June 30th, 2019 are reported.

**Results**

Seven out of the 12 planned evaluable pts have been enrolled: 1 pt is currently undergoing study treatment and thus excluded from this analysis. Median follow-up: 19.5 weeks. All pts completed study treatment and had surgery within the planned window. None required sitravatinib dose reduction/hold or nivolumab delay. No G3/G4 TRAEs occurred pre-surgery. One pt had G3 neck infection and G3 bleeding from the tracheostomy site 11 days post-surgery, both resolved and deemed possibly related to study drugs. Tumor reduction as per investigator’s assessment was observed in all pts. Five pts had pathological downstaging, including 1 complete pathological response (Table 1); all pts had clear margins and no ENE. All pts received standard of care postoperative radiotherapy based on clinical stage. None required postoperative chemotherapy. All pts are alive with no recurrence to date.

**Conclusions**

These preliminary results suggest that preoperative sitravatinib and nivolumab is a safe and active combination in OCSCC. Ongoing biomarker and tumor immunophenotyping analyses will be presented

**Acknowledgements**

The authors would like to thank patients and their families for their participation and Mirati Therapeutics for drug supply and their support of this study.

**Trial Registration**

NCT03575598

**References**

1. Leal et al. ESMO Meeting 2018, Abstract 1129O.

2. Ferris et al. ESMO Meeting 2017, Abstract LBA46.

**Ethics Approval**

This study was approved by the University Health Network Research Ethics Board (Study number: 18-5537) on July 12th 2018.

**Fig. 1 (abstract P400).**
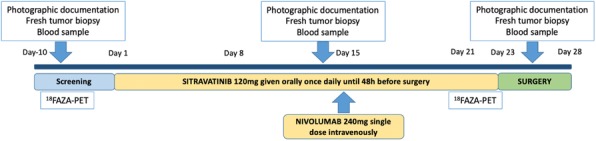
SNOW study design and treatment plan

**Table 1 (abstract P400).**
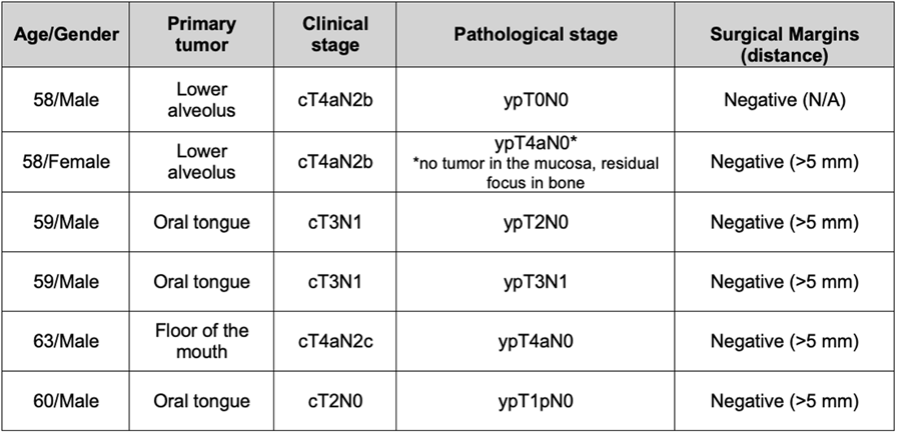
Tumor downstaging following study treatment

#### P401Neoadjuvant and adjuvant pembrolizumab plus standard of care (SOC) in patients with resectable, locally advanced head and neck squamous cell carcinoma (HNSCC): the phase 3 KEYNOTE-689 study

##### Ravindra Uppaluri, MD, PhD^1^, Nancy Lee, MD^2^, William Westra^3^, Ezra Cohen, MD^4^, Robert Haddad^5^, Stephane Temam^6^, Christophe Le Tourneau^7^, Rebecca Chernock^8^, Sufia Safina^9^, Arkadiy Klochikhin^10^, Amichay Meirovitz^11^, Irene Brana, MD^12^, Joy Yang Ge^13^, Ramona Swaby, MD^13^, Cecilia Pinheiro^13^, Douglas Adkins, MD^8^

###### ^1^Dana-Farber Cancer Institute and Brigham and Women’s Hospital, Boston, MA, United States; ^2^Memorial Sloan Kettering, New York, NY, USA, Sylmar, CA, United States; ^3^Icahn School of Medicine, New York, NY, USA, New York, United States; ^4^University of California San Diego, La Jolla, CA, USA, La Jolla, CA, United States; ^5^Dana-Farber Cancer Institute and Brigham and Women's Hospital, Boston, MA, United States; ^6^Gustave Roussy, Villejuif, France, Villejuif, France; ^7^Institut Curie, Paris, France, Paris & Saint-cloud, France; ^8^Washington University School of Medicine, St. Louis, MO, United States; ^9^Republican Dispensary of Tatarstan MoH, Kazan, Russia, Kazan, Russian Federation; ^10^Yaroslavl Regional Clinical Oncology, Ulitsa Chkalov, Yaroslavl, Russia, Yaroslavl, Russian Federation; ^11^Hadassah-Hebrew University Medical Center, Jerusalem, Israel, Jerusalem, Israel; ^12^Hospital Vall d’Hebron, Barcelona, Spain, Barcelona, Spain; ^13^Merck & Co., Inc., Kenilworth, NJ, USA, Kenilworth, NJ, United States

####### **Correspondence:** Ravindra Uppaluri (ravindra_uppaluri@dfci.harvard.edu)

**Background**

Neoadjuvant and adjuvant pembrolizumab showed evidence of pathological response (PR) and acceptable safety in patients with high-risk, resectable, locally advanced (LA) HNSCC in phase 2 studies (NCT02296684 and NCT02641093). KEYNOTE-689 (NCT03765918), a randomized, open-label, phase 3 trial, will assess efficacy and safety of neoadjuvant pembrolizumab and adjuvant pembrolizumab plus SOC in patients with previously untreated, resectable LA HNSCC.

**Methods**

Eligible patients are adults with newly diagnosed, resectable HNSCC (stage III oropharyngeal p16-positive disease [T4 (N0-N2), M0]; stage III/IVA oropharyngeal p16 negative; or stage III/IVA larynx or hypopharynx or oral cavity, independent of p16 status) [1] and ECOG performance status 0 or 1. Patients will be randomly assigned 1:1 to arms A and B, with randomization stratified by primary tumor site (oropharynx/oral cavity vs larynx vs hypopharynx), tumor stage (III vs IVA), and PD-L1 status defined by tumor proportion score 50% (TPS≥50% vs TPS

**Trial Registration**

ClinicalTrials.gov, NCT03765918

**Reference**

1. American Joint Committee on Cancer. AJCC Cancer Staging Manual, Eight Edition. Amin MB, ed. Chicago, IL: American College of Surgeons; 2018.

**Ethics Approval**

The study and the protocol were approved by the Institutional Review Board or ethics committee at each site.

**Consent**

All patients provided written informed consent to participate in the clinical trial.

#### P402 BELINDA : A phase 3 study evaluating the safety and efficacy of tisagenlecleucel versus standard of care in adult patients with relapsed/refractory aggressive B-cell non-Hodgkin lymphoma

##### Michael Bishop, MD^1^, Ian Flinn, MD^3^, Peter Borchmann^4^, Ulrich Jaeger^5^, Jason Westin, MD^6^, Nada Hamad^7^, Duncan Purtill^8^, Richard Greil^9^, Simone Thomas^10^, Takanori Teshima^11^, Hideo Harigae^12^, Carlos Garcia^13^, Pere Barba^14^, Abhinav Deol, MD^15^, Paul Shaughnessy^16^, Jessie Gu^17^, Giovanna Andreola^18^, Marcela Martinez Prieto^17^, Lida Pacaud^17^, Stephen Schuster, MD^19^

###### ^1^University of Chicago, Chicago, IL, United States; ^2^Univeristy of Chicago, Chicago, IL, United States; ^3^Sarah Cannon Research Institute, Nashville, TN, United States; ^4^University Hospital of Cologne, Cologne, Germany; ^5^Medical University of Vienna, Vienna, Austria; ^6^University of Texas, MD Anderson Cancer Center, Houston, TX, United States; ^7^St Vincent’s Hospital, Sydney, Australia; ^8^Fiona Stanley Hospital, Murdoch, Australia; ^9^Paracelsus Medical University, Salzburg, Austria; ^10^University Hospital Regensburg, Regensburg, Germany; ^11^Hokkaido University, Hokkaido, Japan; ^12^Tohoku University Graduate School of Med, Miyagi, Japan; ^13^University Hospital "12 de Octubre", Madrid, Spain; ^14^Universitat Autònoma de Barcelona, Barcelona, Spain; ^15^Karmanos Cancer Institute, Wayne State University, Detroit, MI, United States; ^16^Texas Transplant Institute, San Antonio, TX, United States; ^17^Novartis Pharmaceuticals Corporation, East Hanover, NJ, United States;^18^Novartis Pharma AG, Basel, Switzerland: ^19^Abramson Cancer Center, University of Pennsylvania, Philadelphia, PA, United States

####### **Correspondence:** Michael Bishop (mbishop@medicine.bsd.uchicago.edu)

**Background**

Around one-third of aggressive B-cell non-Hodgkin lymphoma (NHL) patients will not respond to, or will relapse or progress after frontline treatment; >50% of treatment failures occur within one year. Prognosis is particularly poor in these patients, regardless of salvage chemotherapy and autologous hematopoietic stem cell transplant (auto-HSCT). Novel therapies are therefore needed for refractory or early-relapsed patients with NHL.

**Methods**

BELINDA (NCT03570892) is a randomized, open-label, multicenter, phase 3 study to compare the safety and efficacy of two treatment strategies: tisagenlecleucel with standard of care (SOC) immunochemotherapy followed by auto-HSCT in adult patients with aggressive B-cell NHL whose disease relapsed or progressed after frontline immunochemotherapy. Eligible patients are aged ≥18 years, have histologically confirmed aggressive B-cell NHL relapsed/refractory to frontline therapy containing rituximab and anthracycline within one year of last dose, and are eligible for auto-HSCT. Patients are apheresed prior to enrollment and randomized 1:1 to receive tisagenlecleucel (Arm A) or SOC (Arm B) (Figure 1). Randomization is stratified by remission duration (refractory or relapse

**Trial Registration**

NCT03570892

**Ethics Approval**

The study is done in accordance with the principles of Good Clinical Practice, the Declaration of Helsinki, and all local regulations. The study protocol and all amendments were reviewed and approved by independent ethics committees or institutional review boards for each center. All patients provided written informed consent.

**Fig. 1 (abstract P402).**
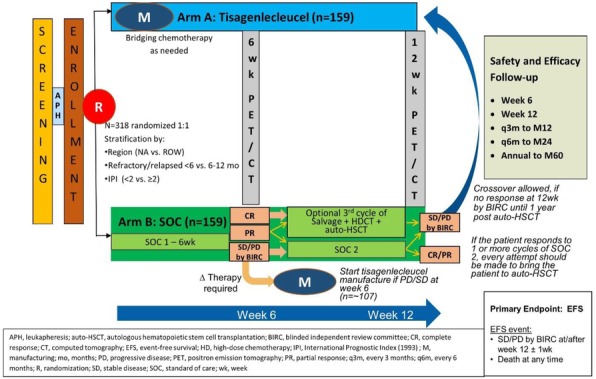
See text for description

#### P403 Serum soluble CD25 may predict the early therapeutic response in pediatric patients with B-cell non-Hodgkin's lymphoma(B-NHL)during autologous chimeric antigen receptor T cell(CAR-T) therapy

##### Jing Guo, Yonghong Zhang, Professor, Juan Du, MD

###### Beijing Boren Hospital, Beijing, China

####### **Correspondence:** Yonghong Zhang (yhzhang58@126.com)

**Background**

CAR-T therapies have been widely employed in B-NHL.Immune activation induced by CAR-T therapies includes significant changes of some inflammatory cytokines. In this study we analyzed the changes of serum cytokine levels in 12 pediatric patients with B-NHL during CAR-T therapy, so as to explore the relationship between serum cytokine levels and early treatment response. The association between cytokine levels and cytokine-release syndrome(CRS) grade was also investigated.

**Methods**

12 B-NHL pediatric patients aging from 4 to 14 in stage II to IV according to st.jude stage were enrolled .Each patient received 1 to 3 rounds sequential CAR-T treatment, consisting of CD19, CD20, CD22 CAR-T treatment. A total of 18 rounds CAR-T were performed, including 12 CD19, 3 CD20 and 3 CD22. Early tumor response were evaluated on day 15, 30 and 60 of each round of CAR-T and early side effect known as CRS were observed and graded. Serum samples were collected at baseline and on day 3,7,11,15,20,30,60 of each round of CAR-T. Levels of Interleukin-6 (IL-6), soluble CD25 (sCD25) and interferon-γ (IFN-γ) interleukin-10 (IL-10), tumor necrosis factor- α(TNF-α)were measured by ELISA(enzyme-linked immunosorbent assay). We analyzed the cytokine changes in different treatment outcomes, explored the predictive value of different cytokines using ROC curve .The relationship between cytokine level and CRS grade was also analyzed. Tumor response was assessed per RECIST 1.1. The ROC curve take” response “and “no response” as the outcome indicators ,stable disease (SD) and progressive disease (PD)were defined as “no response”, complete response(CR) and partial response(PR )were defined as “response”. Adverse Event (AE) grade categorization is according to CTCAE 4.0.

**Results**

There were statistically significant differences in sCD25 values among different treatment responses (P<0.01), with an average decrease in SD patients and an average increase in PR patients ,it is speculated that sCD25 may play a predictive role in treatment response. The ROC curve analysis shows that sCD25 had a predictive effect on the response to treatment, and the AUC was 0.719,95%ci =(0.516, 0.922) ,excluding 0.5, indicating that the difference is statistically significant. In this study, other cytokines were not found to be predictive markers of therapeutic response. Besides,Spearman rank correlation coefficient showed that there was a positive correlation between sCD25 and CRS grade (r=0.693,P=0.001).

**Conclusions**

sCD25 may be a useful predictive marker for early response of CAR-T and level of sCD25 are correlated with CRS grade. Clinical trial information:ChiCTR18000144

#### P404 Developing canine CART-19 to fully leverage comparative oncology and inform human clinical trials

##### Kumudhini Haran, MS, Ailian Xiong, Enrico Radaelli, Patrick Savickas, Avery Posey, Donald Siegel, Nicola Mason, BVet Med PhD

###### University of Pennsylvania, Springfield, PA, United States

####### **Correspondence:** Nicola Mason (nmason@vet.upenn.edu)

**Background**

CD19 specific chimeric antigen receptor T cell (CART-19) therapy has resulted in unprecedented durable clinical responses in adult and pediatric patients with B-cell malignancies. However, poor quality of patient T cells, failed persistence, reduced effectiveness within an immunosuppressive microenvironment and target antigen loss, represent some of the challenges to improving CART19 efficacy. Furthermore, correlative biomarkers that predict CART-19 response remain elusive.

Pet dogs spontaneously develop B-NHL and B cell leukemias that share oncogenic pathways and similar immunosuppressive features to human B cell malignancies. Therefore, they provide an immunologically intact, parallel patient population in which to evaluate next generation CAR T cell strategies and combination approaches that address current CART19 challenges. Previously, we have demonstrated the ability to generate functional CD20-targeting canine CAR T cells. Their use in client owned animals with B-NHL can lead to the development of canine anti-mouse antibody (CAMA) formation and target antigen escape. To address these issues and provide a parallel reagent that can inform human CAR T cell strategies, we have developed a fully canine CD19 targeting CAR and confirmed its function in vitro against CD19 expressing targets.

**Methods**

We employed a canine scFv phage display library to isolate canine CD19-specific scFvs following 3-4 rounds of panning against the soluble extracellular domain of canine CD19. Twelve unique scFvs were isolated and their binding to soluble canine CD19 and cell surface expressed CD19 was confirmed by ELISA and flow cytometry respectively. One of the highest binding candidates was cloned into a fully canine CD28ζ CAR in a pMX retroviral plasmid. Retroviruses pseudotyped with both RD114 and VSV-G envelope proteins were generated using standard protocols and used to transduce primary canine T cells activated using anti-canine CD3/CD28 beads in the presence of RetroNectin®. Successful transductions of canine T cells were obtained with 45% of T cells expressing CAR on their cell surface by flow cytometry. Canine CART-19 cells demonstrated antigen-specific proliferation and cytokine production in vitro. We now aim to perform a pilot study to evaluate the safety and efficacy of this approach in canine patients with relapsed, refractory B-NHL. This work will serve the dual purpose of enabling pet dogs with spontaneous B cell malignancies to accelerate application of next generation CART cell therapies into the human clinics as well as provide much needed immunotherapeutics for canine patients with B cell malignancies for which there are no effective therapies that induce durable remissions.

#### P405 CASSIOPEIA: A phase 2 study evaluating efficacy and safety of tisagenlecleucel in first-line therapy for high-risk pediatric and young adult patients with B-ALL who are MRD positive at the EOC

##### Shannon Maude, MD, PhD^1^, Hunger Stephen, MD^1^, Jochen Buechner, MD^2^, Stephen Grupp, MD^1^, Susana Rives^3^, Andre Baruchel^4^, John Levine^5^, Joerg Krueger^6^, Theodore Laetsch^7^, Marianne Ifversen^8^, Aiesha Zia^9^, Jaclyn Davis^10^, Eric Bleickardt^10^, Mignon Loh^11^

###### ^1^University of Pennsylvania; Children’s Hospital of Philadelphia, Philadelphia, PA, United States; ^2^Oslo University Hospital, Oslo, Norway; ^3^Sant Joan de Deu Hospital, Barcelona, Spain; ^4^Hôpital Robert Debré&Université de Paris, Paris, France; ^5^Mount Sinai School of Medicine, New York, NY, United States; ^6^The Hospital for Sick Children, Toranto, Canada; ^7^UT Southwestern Medical Center and Child, Dallas, TX, United States; ^8^Copenhagen University Hospital Rigshospi, Copenhagen, Denmark; ^9^Novartis Pharma AG, Basel, Switzerland; ^10^Novartis Pharmaceuticals Corporation, East Hanover, NJ, United States; ^11^University of California, San Francisco, CA, United States

####### **Correspondence:** Shannon Maude (maude@email.chop.edu)

**Background**

Survival is compromised for patients with high-risk (HR) B-cell acute lymphoblastic leukemia (B-ALL) who have a poor response to first-line chemotherapy. A Children’s Oncology Group (COG) phase-3 study for HR B-ALL, AALL0232, showed poor 5-year disease free survival (DFS) of 39% in patients with minimal residual disease (MRD) ≥0.1% at end of induction (EOI) and MRD ≥0.01% at the end of consolidation (EOC) [1]. The objective of this trial is to determine the efficacy and safety of tisagenlecleucel in pediatric and young adult patients with de novo HR B-ALL who received first-line treatment and remain MRD-positive after the EOC therapy.

**Methods**

CASSIOPEIA (NCT03876769) is a phase-2, single-arm, global, multicenter, open-label study being conducted in collaboration with COG. Patients aged 1-25 years with de novo National Cancer Institute defined HR B-ALL (presenting white blood count >50,000/μL or over the age of 10 years) who are in first complete remission (CR1) but remain MRD-positive (≥0.01% by flow cytometry determined at a central reference laboratory) at EOC are eligible. Prior to screening, patients will complete a standard of care first-line 4-drug induction, MRD assessment at EOI, a Berlin-Frankfurt-Münster phase-1b consolidation, and MRD assessment at EOC. Eligible patients undergo leukapheresis either at the EOI or EOC. Prior to tisagenlecleucel infusion, patients will receive interim maintenance including high-dose methotrexate. Following lymphodepleting chemotherapy, patients receive a single infusion of tisagenlecleucel based on body weight; 0.2-5.0x10^6 chimeric antigen receptor (CAR)-positive viable T-cells per kg in patients ≤50 kg or 0.1-2.5x10^8 CAR-positive viable T-cells in patients >50 kg. Patients may receive a second infusion based on B-cell recovery and MRD status. Efficacy will be assessed at day 29, then every 3 months for the first year, every 6 months for the second year, then yearly until the end of study. The primary outcome is 5-year DFS rate by local investigator assessment, defined as the time from tisagenlecleucel infusion to morphologic relapse, occurrence of secondary malignancy or death from any cause, whichever occurs first. Secondary outcomes include percentage of patients in remission without allogeneic transplantation at 1 year, MRD negativity at month 3, overall survival, cellular kinetics, and safety. The primary analysis of DFS will be undertaken when 40 DFS events are observed or 6 years after first-patient-first-treatment, whichever occurs later. The estimated enrollment for this study is 160 patients (with 140 infused). The study is currently enrolling patients in the U.S., Europe and Canada.

**Trial Registration**

NCT03876769

**Reference**

1. Borowitz MJ, Wood BL, Devidas M et al. Prognostic significance of minimal residual disease in high risk B-ALL: a report from Children's Oncology Group study AALL0232. Blood. 2015;126(8):964-971.

**Ethics Approval**

The study is done in accordance with the principles of Good Clinical Practice, the Declaration of Helsinki, and all local regulations. The study protocol and all amendments were reviewed and approved by independent ethics committees or institutional review boards for each center. All patients provided written informed consent.

#### P406 Interleukin-1 blockade to prevent severe immune effector cell-associated neurotoxicity syndrome; Trial in progress

##### Caspian Oliai, MD, Anna Crosetti, John Timmerman, MD

###### UCLA, Los Angeles, CA, United States

####### **Correspondence:** John Timmerman (jtimmerman@mednet.ucla.edu)

**Background**

CAR T-cell therapy targeting CD19 is a promising new treatment for relapsed/refractory B-cell lymphomas and leukemias. However, severe grade 3 neurotoxicity (immune effector cell-associated neurotoxicity syndrome, or ICANS) is seen in up to one-third of patients. Recently, preclinical animal studies of human CD19 CAR T-cell therapies have shown that while IL-6 and IL-1 receptor antagonists could prevent cytokine release syndrome (without impairing anti-tumor efficacy), only IL-1 blockade could prevent neurotoxicity [1,2]. The recombinant IL-1 receptor antagonist Anakinra was used successfully to avert lethal neurotoxicity in mice. Anakinra crosses the blood brain barrier and has been shown to be safe and efficacious in rheumatologic conditions driven by high levels of monocyte lineage-associated IL-1 including rheumatoid arthritis and neonatal onset multisystem inflammatory disease, for which it is FDA approved. We are conducting the first human trial of Anakinra to treat ICANS in B-cell lymphoma patients treated with anti-CD19 CAR T-cells. The trial has been approved by the U.S. FDA under an investigator-sponsored IND, and Anakinra is supplied by the agent’s manufacturer (Sobi Pharmaceuticals).

**Methods**

Patients with diffuse large B-cell lymphoma receiving standard of care CAR T-cells are eligible for enrollment. The primary objectives are to: 1) Evaluate the effectiveness of IL-1 blockade in reducing the incidence and duration of severe ICANS in participants receiving anti-CD19 CAR T-cells, 2) Assess the safety of Anakinra in CAR T-cell patients, 3) Measure cytokines (including IL-1, IL-6, IL-15, TNF-alpha, interferon-gamma) and nitric oxide in the serum and CSF of treated patients prior to and during CAR T-cell therapy for correlation with ICANS events, and 4) Determine the tumor response rate in comparison to historical controls. Upon development of grade 1 ICANS, or grade 3 CRS (which is often followed by ICANS), participants will receive Anakinra 100 mg subcutaneously every 6 hours for at least 12 doses, or until ICANS returns to grade 1 in participants who develop grade 2 neurotoxicity. Patients will be continuously evaluated for toxicity, and assessed for overall tumor response by day 120 with PET/CT scanning. Thirty-six participants will be treated in this multicenter trial, at four centers within the University of California Hematologic Malignancies Consortium (UC Los Angeles, UC San Francisco, UC San Diego, and UC Davis). The trial is powered to detect a 50% reduction in the rate of severe ICANS compared to historical rates.

**References**

1. Norelli M, Camisa B, Barbiera G, Falcone L, Purevdorj A, Genua M, Sanvito F, Ponzoni M, Doglioni C, Cristofori P, Traversari C, Bordignon C, Ciceri F, Ostuni R, Bonini C, Casucci M, Bondanza A. Monocyte-derived IL-1 and IL-6 are differentially required for cytokine-release syndrome and neurotoxicity due to CAR T cells. Nat Med. 2018 Jun;24(6):739-748.

2. Giavridis T, van der Stegen SJC, Eyquem J, Hamieh M, Piersigilli A, Sadelain M. CAR T cell-induced cytokine release syndrome is mediated by macrophages and abated by IL-1 blockade. Nat Med. 2018 Jun;24(6):731-738.

**Ethics Approval**

UCLA IRB #19-000604

#### P407 Phase 3 KEYNOTE-937: adjuvant pembrolizumab versus placebo in patients with hepatocellular carcinoma and complete radiologic response after surgical resection or local ablation

##### Masatoshi Kudo, MD, PhD^1^, Andrew Zhu^2^, Arndt Vogel^3^, Thomas Yau^4^, Jian Zhou^5^, Erluo Chen^6^, Usha Malhotra^6^, Abby Siegel^6^, Ann-Lii Cheng, MD PhD^7^

###### ^1^Kindai University School of Medicine, Osaka, Japan, Osaka-Sayama, Japan; ^2^Massachusetts General Hospital Cancer Center, Harvard Medical School, Boston, MA, United States; ^3^Medizinische Hochschule, Hannover, Germany, Hannover, Germany; ^4^University of Hong Kong, Queen Mary Hospital, Hong Kong, Hong Kong PRC; ^5^Zhongshan Hospital, Fudan University, Shanghai, China, Shanghai, China; ^6^Merck & Co., Inc., Kenilworth, NJ, USA, Kenilworth, NJ, United States; ^7^National Taiwan University Hospital Cancer Center, Taipei, Taiwan, Taipei, Taiwan

####### **Correspondence:** Masatoshi Kudo (m-kudo@med.kindai.ac.jp)

**Background**

For patients with hepatocellular carcinoma (HCC) who are undergoing potentially curative surgical resection or local ablation, 5-year recurrence rates are up to 50%-80%; there is no standard of care for adjuvant treatment. The programmed death 1 inhibitor pembrolizumab is approved for the treatment of patients with HCC previously treated with sorafenib. There is no direct evidence of benefit with pembrolizumab in the HCC adjuvant setting, but a favorable benefit/risk profile is anticipated based on data from other indications. KEYNOTE-937 (NCT03867084) is a randomized, double-blind, phase 3 trial to examine the safety and efficacy of adjuvant pembrolizumab versus placebo in patients with complete radiologic response after surgical resection or local ablation of HCC.

**Methods**

Eligible patients are aged ≥18 years and have confirmed HCC, complete radiologic response after complete resection or local ablation, Eastern Cooperative Oncology Group performance status of 0, and class A Child-Pugh score. Patients with past or ongoing HCV or controlled HBV are eligible if they meet certain criteria. Patients (N=~950) will be randomly assigned 1:1 to receive pembrolizumab 200 mg or placebo every 3 weeks and stratified by geographic region, prior local therapy (resection vs ablation), recurrence risk, and alpha-fetoprotein level at diagnosis. Treatment will continue for up to 17 cycles (~1 year) or until documented disease recurrence, unacceptable toxicity, or investigator/patient decision to withdraw. Dual primary end points are recurrence-free survival (RFS) and overall survival. Secondary end points are safety, tolerability, and quality of life. Exploratory end points include distant metastases–free survival (DMFS); time to recurrence (TTR); and genomic, metabolic, and/or proteomic biomarkers. RFS, DMFS, and TTR will be assessed radiographically by the investigator and/or by subsequent biopsy and confirmed by blinded independent central review. Adverse events (AEs), graded per National Cancer Institute Common Terminology Criteria for Adverse Events version 4.0, will be recorded up to 30 days after last dose (90 days for serious AEs).

**Trial Registration**

ClinicalTrials.gov, NCT03867084

**Ethics Approval**

The study and the protocol were approved by the Institutional Review Board or ethics committee at each site.

**Consent**

All patients provided written informed consent to participate in the clinical trial.

#### P408 LEAP-002: phase 3 study of first-line lenvatinib plus pembrolizumab for patients with advanced hepatocellular carcinoma

##### Josep Llovet^1^, Masatoshi Kudo, MD, PhD^2^, Ann-Lii Cheng, MD PhD^3^, Richard Finn^4^, Peter Galle^5^, Shuichi Kaneko, MD PhD^6^, Tim Meyer^7^, Shukui Qin^8^, Corina Dutcus^9^, Erluo Chen^10^, Leonid Dubrovsky^10^, Abby Siegel^10^, Andrew Zhu^11^

###### ^1^Icahn School of Medicine at Mount Sinai, New York, NY, USA; ^2^Kindai University School of Medicine, Higashiosaka, Japan, Osaka-Sayama, Japan; ^3^National Taiwan University Hospital Cancer Center, Taipei, Taiwan, Taipei, Taiwan; ^4^David Geffen School of Medicine at UCLA, Los Angeles, CA, USA, Los Angeles, CA, United States; ^5^University of Mainz Medical Center, Mainz, Germany, Mainz, Germany; ^6^Kanazawa University Hospital, Kanazawa, Japan, Kanazawa, Japan; ^7^University College London Cancer Institute, London, United Kingdom, London, United Kingdom; ^8^Jinling Hospital, Nanjing, China, Nanjing, China; ^9^Eisai Inc., Woodcliff Lake, NJ, USA, Woodcliff Lake, NJ, United States; ^10^Merck & Co., Inc., Kenilworth, NJ, USA, Kenilworth, NJ, United States; ^11^Massachusetts General Hospital Cancer Center, Harvard Medical School, Boston, MA, USA, Boston, MA, United States

####### **Correspondence:** Josep Llovet (Josep.Llovet@mountsinai.org)

**Background**

Lenvatinib, a multikinase inhibitor, is approved for first-line treatment of unresectable hepatocellular carcinoma (HCC). Pembrolizumab, a programmed death 1 inhibitor, is approved for second-line treatment of advanced HCC in patients previously treated with sorafenib. The phase 1b KEYNOTE-524 trial showed that lenvatinib plus pembrolizumab was well tolerated and demonstrated promising antitumor activity in patients with unresectable HCC. LEAP-002 (NCT03713593) is a phase 3 study to evaluate the safety and clinical benefit of lenvatinib plus pembrolizumab in patients with previously untreated advanced HCC.

**Methods**

Eligible patients are aged ≥18 years and have confirmed HCC, Eastern Cooperative Oncology Group performance status (ECOG PS) 0 or 1, Barcelona Clinic Liver Cancer stage C or stage B disease not amenable to locoregional or curative therapy, class A Child-Pugh score ≤7 days before study day 1, and ≥1 measurable lesion (per RECIST v1.1 by blinded independent central review [BICR]). Past or ongoing HCV infection and controlled HBV are allowed. Patients will be randomly assigned 1:1 to receive oral lenvatinib 12 mg (body weight [BW] ≥60 kg) or 8 mg (BW 400 ng/mL); and ECOG PS (0 vs 1). Tumor imaging will be performed every 9 weeks. Dual primary end points are progression-free survival (PFS), assessed per modified RECIST v1.1 by BICR, and overall survival. Secondary end points are objective response rate (ORR), duration of response (DOR), disease control rate (DCR), and time to progression (TTP) per RECIST v1.1 by BICR, efficacy outcomes (PFS, ORR, DOR, DCR, and TTP) per modified RECIST v1.1 by BICR, pharmacokinetics, and safety. Exploratory end points are efficacy outcomes evaluated per RECIST v1.1 and iRECIST assessed by the investigator. Adverse events (AEs), graded per National Cancer Institute Common Terminology Criteria for Adverse Events version 4.0, will be monitored throughout the treatment period and for 90 days after the last dose (120 days for serious AEs).

**Trial Registration**

ClinicalTrials.gov, NCT03713593

**Ethics Approval**

The study and the protocol were approved by the Institutional Review Board or ethics committee at each site.

**Consent**

All patients provided written informed consent to participate in the clinical trial.

#### P409 A case report of personalized neoantigen peptide vaccine in treating patients with biliary tract cancer

##### Fang Yong^1^, Fan Mo^2^, Jiawei Shou^1^, Huimin Wang^3^, Lin Chen^3^, Shanshan Zhang^4^, Hongsen Li^1^, Weidong Han^1^, Hongming Pan^1^, Shuqing Chen^4^

###### ^1^Sir Run Run Shaw Hospital, Hangzhou, China; ^2^Vancouver Prostate Centre, UBC, Hangzhou, China; ^3^Hangzhou Neoantigen Therapeutics Co., Hangzhou, China; ^4^Zhejiang University, Hangzhou, China

####### **Correspondence:** Shuqing Chen (chenshuqing@zju.edu.cn)

**Background**

Despite recent advance in immune checkpoint blockade therapies in cancer, the overall response rate is still low in malignancy treatment. Arisen from tumor somatic mutations, neoantigens provide tumor specific targets for developing personalized cancer vaccines, further eliciting strong T cell-mediated immune response. A single-arm, open-labelled, investigator-initiated clinical study was carried out to examine the safety and efficacy of personalized peptide vaccine (iNeo-Vac-P01). Total of 22 patients with solid tumors had been enrolled in the trial from Feb 7th, 2018 to May 31st, 2019. A biliary tract cancer patient achieved unique neoplastic changes.

**Methods**

The 63-year-old male, initially diagnosed with intrahepatic biliary tract cancer in 2013, was treated with surgical excision in Jun. 2013 and postoperative chemotherapy in Apr. 2014 respectively. Both tumor recurrence and metastases were confirmed with CT scan in Nov. 2017. And then, he was treated with 6 cycles of PD-1 antibody in a clinical trial and dropped out due to disease progression. Under his consent, his biopsy and blood samples were obtained for whole exome sequencing (WES), RNA sequencing (RNA-seq), and neoantigen identification [1-4]. Finally the total of 7 peptides were synthesized and pooled into 2 groups.

On Mar 22th, 2018, he started to receive iNeo-Vac-P01 subcutaneously (s.c.). The injection sites were the two upper arms. He was scheduled to receive vaccinations with GM-CSF as adjuvant on day 1, 4, 8, 15 and 22 (i.e. priming phase), as well as 6 subsequent boosters [5-8].

**Results**

After the last booster vaccination, a grade 3~4 allergic reaction (under NCI-CTCAE 4.03) happened, while clinical manifestations were nausea, vomiting and rash. The treatment-relating allergic reaction maybe result from peptide-specific antibody accumulation, however, this hypothesis needs experimental validation by enzyme-linked immunosorbent assay. The CT scans indicated an evident increase of tumor size at 5th month, and a surprisingly decrease of tumor size at 8th month, implying a pseudo-progression. The duration of stable disease was 14.5+ months, and he had been keeping progression-free since then. The results of IFN-γ ELISPOT assay shows that the neoantigen peptides stimulated highest number of IFN-γ spots and induced significant peptide-specific T-cell response. TCR sequencing demonstrated the evident increase of peripheral T cells with three TCRs (data unshown).

**Conclusions**

The preliminary results demonstrated that iNeo-Vac-P01 treatment was feasible and safe, and can prolong progression-free survival and overall survival.

**Acknowledgements**

The authors would like to gratitude all the patients who participated in the trial and their families, as well as the Sir Run Shaw clinical site. This study was funded by Hangzhou Neoantigen Therapeutics Co.

**Trial Registration**

This trial had been registration on ClinicalTrials.gov, the identifier number was NCT03662815.

**References**

1. Chen F, Zou Z, Du J, Su S, Shao J, Meng F, Yang J, Xu Q, Ding N, Yang Y et al. Neoantigen identification strategies enable personalized immunotherapy in refractory solid tumors. J Clin Invest. 2019; 130.

2. Hundal J, Carreno BM, Petti AA, Linette GP, Griffith OL, Mardis ER, Griffith M. pVAC-Seq: A genome-guided in silico approach to identifying tumor neoantigens. Genome Med. 2016; 8(1):11.

3. Ng AWR, Tan PJ, Hoo WPY, Liew DS, Teo MYM, Siak PY, Ng SM, Tan EW, Abdul Rahim R, Lim RLH et al. In silico-guided sequence modifications of K-ras epitopes improve immunological outcome against G12V and G13D mutant KRAS antigens. PeerJ. 2018; 6:e5056.

4. Ott PA, Hu Z, Keskin DB, Shukla SA, Sun J, Bozym DJ, Zhang W, Luoma A, Giobbie-Hurder A, Peter L et al. An immunogenic personal neoantigen vaccine for patients with melanoma. Nature. 2017; 547(7662):217-221.

5. Gjertsen MK, Buanes T, Rosseland AR, Bakka A, Gladhaug I, Soreide O, Eriksen JA, Moller M, Baksaas I, Lothe RA et al. Intradermal ras peptide vaccination with granulocyte-macrophage colony-stimulating factor as adjuvant: Clinical and immunological responses in patients with pancreatic adenocarcinoma. Int J Cancer. 2001; 92(3):441-450.

6. Keskin DB, Anandappa AJ, Sun J, Tirosh I, Mathewson ND, Li S, Oliveira G, Giobbie-Hurder A, Felt K, Gjini E et al. Neoantigen vaccine generates intratumoral T cell responses in phase Ib glioblastoma trial. Nature. 2019; 565(7738):234-239.

7. Weden S, Klemp M, Gladhaug IP, Moller M, Eriksen JA, Gaudernack G, Buanes T. Long-term follow-up of patients with resected pancreatic cancer following vaccination against mutant K-ras. Int J Cancer. 2011; 128(5):1120-1128.

8. Kirner A, Mayer-Mokler A, Reinhardt C. IMA901: a multi-peptide cancer vaccine for treatment of renal cell cancer. Hum Vaccin Immunother. 2014; 10(11):3179-3189.

**Ethics Approval**

This study was approved by the institutional review board and independent ethics committee of Sir Run Run Shaw Hospital, Zhejiang University School of Medicine; approval number 20180109-9.

**Consent**

Written informed consent was obtained from the patient for publication of this abstract and any accompanying images. A copy of the written consent is available for review by the Editor of this journal.

#### P410 Safety and anti-tumor activity of the transforming growth factor β receptor I kinase inhibitor, vactosertib, in combination with durvalumab in patients with advanced non-small cell lung cancer (NSCLC)

##### Ji-Youn Han, MD, PhD^1^, Kyoung-Ho Pyo^2^, Jea Hwan Kim^2^, Chun-Feng Xin^2^, Jin Kyung Lee^3^, Sunjin Hwang^3^, Seong-Jin Kim^3^, Byoung Chul Cho, MDphD^2^, Byoung Chul Cho, MDphD^2^

###### ^1^National Cancer Center, Goyang-si, Korea; ^2^Severance Hospital, Seoul, Korea, Republic of; ^3^Medpacto, Inc, Seoul, Korea, Republic of

####### **Correspondence:** Byoung Chul Cho (cbc1971@yuhs.ac)

**Background**

TGF-β signaling is known to be associated with poor response to single-agent immune checkpoint inhibitors by immunosuppressive microenvironment through strong epithelial-mesenchymal transition (EMT) induction. Combined inhibition of immune checkpoint and TGF-β signal is anticipated as a promising therapeutic strategy because these two key pathways have independent and complementary immunosuppressive functions. We are reporting the Dose Finding part of a Phase 1b/2a study evaluating the combination of vactosertib, a highly selective and potent TGF-β inhibitor, with durvalumab in patients with advanced non-small cell lung cancer (NSCLC) who progressed following platinum-based chemotherapy.

**Methods**

Eligible patients (pts) are ≥19 years old, have ECOG status ≤1, and have no prior exposure to immune checkpoint inhibitors, or TGFβ R1 kinase inhibitors. The primary objective is to assess the safety and the recommended dose of vactosertib given 5 days on/2 days off in combination with durvalumab 1500 mg every 4 weeks. Two dose levels of vactosertib (100 mg BID and 200 mg BID) were tested in the dose finding part. Secondary objectives include characterization of vactosertib pharmacokinetics and anti-tumor activity by response rate (RECIST v1.1).

**Results**

As of July 18, 2019, 13 patients were enrolled to the study (7 in 100 mg BID cohort and 6 in 200 mg BID cohort). Median age was 66 (range 45-76), 62% were male, median number of previous lines of chemotherapy was 4 (range 2-8). All patients were PD-L1 less than 25% by SP263 antibody assay. At 100 mg BID cohort, no dose limiting toxicity was observed. The most frequently reported adverse events (AE) were skin rash (30.8%), nausea (23.1%), and pruritis (23.1%). There were 3 serious adverse events (SAE) reported; pleural effusion (1), skin eruption (1), and empyema (1), and no patients with reported cardiotoxicity. Among 7 tumor response evaluable patients, best responses to treatment were SD in 3 patients; 5.9%, 10.4%, and 26.4% decreases from baseline. Biomarker data will be presented at the meeting.

**Conclusions**

The combination of vactosertib plus durvalumab has been tolerated thus far with no safety concerns; the study is ongoing. The anti-tumor activity of this combination in patients with advanced NSCLC will be further explored. Clinical trial information: NCT03732274

**Trial Registration**

NCT 03732274

**Ethics Approval**

The study was approved by Ethics Board of Severance Hospital (approval number 4-2018-0892) and National Cancer Center (approval number NCC2019-0057)

#### P411 Treating advanced non-small lung cancer (NSCLC) patients after checkpoint inhibitor treatment failure with a novel combination of Viagenpumatucel-L (HS-110) plus nivolumab

##### Daniel Morgensztern, MD^1^, Saiama Waqar, MD^1^, Lyudmila Bazhenova, MD^2^, Rachel Sanborn, MD^3^, Lori Mcdermott, RN, MSc^4^, Jeff Hutchins, PhD^4^, Luis Raez, MD, FACP, FCCP^5^, Corey Langer, MD^6^, Roger Cohen, MD^6^

###### ^1^Washington University School of Medicine, St. Louis, MO, United States; ^2^Moores Cancer Center, La Jolla, CA, United States; ^3^Earle A. Chiles Research Institute, Portland, OR, United States; ^4^Heat Biologics, Tampa, FL, United States; ^5^Memorial Cancer Institute, Pembroke Pines, FL, United States; ^6^Perelman School of Medicine, Philadelphia, PA, United States

####### **Correspondence:** Lori Mcdermott (lmcdermott74@yahoo.com)

**Background**

Viagenpumatucel-L (HS-110) is an allogeneic cellular vaccine derived from a human lung adenocarcinoma cell line transfected with the gp96-Ig fusion protein that functions as an antigen chaperone for cross presentation and dendritic cell activation. DURGA is a multi-cohort study evaluating the combination of HS-110 and anti-PD-1 monoclonal antibodies in patients with advanced NSCLC. We report on Cohort B, which enrolled patients with progressive disease (PD) after receiving a minimum of 4 months of treatment with a checkpoint inhibitor (CPI) at any time prior to study entry.

**Methods**

Patients with previously treated NSCLC received weekly HS-110 (1 X 107 cells) intradermally for 18 consecutive weeks and nivolumab IV 240 mg every 2 weeks, followed by nivolumab maintenance until tumor progression or intolerable toxicity. Tissue was tested at baseline for PD-L1 expression (≥ 1% or < 1%) and tumor infiltrating lymphocytes (TILs). TIL high was defined as >10% CD8+ lymphocytes in the tumor stroma. The primary endpoint was objective response rate (ORR) by RECIST 1.1. Secondary endpoints included ORR and clinical benefit rate using iRECIST, progression-free survival (PFS), overall survival (OS) and adverse events (AEs).

**Results**

As of March 2019, 56 patients were enrolled and evaluated for efficacy. The median number of prior treatment lines was 2 [range 1 to 6]. Seven patients (13%) achieved partial response and 26 patients (46%) had stable disease. Median PFS and median OS were 3.2 months and 11.8 months, respectively. Immune ORR and clinical benefit rate by iRECIST were 14% and 61%, respectively. Patients experiencing injection site reactions (ISR) had improved PFS (3.7 vs 1.8 months; HR 0.21, p =0.0021) and improved OS (12 vs 5 months; HR 0.16, p=0.0005) compared to those without ISR. 96% of patients experienced at least one adverse event, and 92% of all AEs were grade 1 or 2. The most common AEs were fatigue (34%), hypocalcemia (18%), cough (16%) and diarrhea and dyspnea (14% each). There were four grade 4 events: QTc prolongation, stroke, pericardial tamponade, and hyponatremia, none of which were deemed related to treatment. There were no grade 5 AEs.

**Conclusions**

The combination of HS-110 and nivolumab is well tolerated, and does not appear to increase the incidence of immune-related AEs as compared to CPI monotherapy. Patients continue to be enrolled into this cohort. Data suggest that re-challenging the immune system with nivolumab and HS-110 after CPI treatment failure restores responsiveness and clinical benefit for some patients.

**Acknowledgements**

Thank you to the Investigators, their staff, and the patients and family members that made this research possible.

**Trial Registration**

NCT 02439450

**Ethics Approval**

This study was approved by Advarra IRB, Western IRB, Washington University IRB, Cleveland Clinic IRB, UCSD IRB, Providence Portland IRB, NYU Winthrop IRB, Baptist Health Louisville IRB, Lifespan IRB, and US Oncology IRB.

#### P412 Validation of a single-blinded (patients only) study design for the prevention of premature patient consent withdrawal in the immuno-oncology trial DUBLIN-3

##### Ramon Mohanlal, MD, PhD, MBA, Huang Lan, PhD

###### BeyondSpring Pharmaceuticals, Inc., New York, NY, United States

####### **Correspondence:** Ramon Mohanlal (rmohanlal@beyondspringpharma.com)

**Background**

Patients (pts) generally prefer immunotherapy (IO) over chemotherapy (Chemo) in clinical trials and may prematurely withdraw consent if allocated to Chemo. This may impact study outcome (Barlesi Lancet Onc 2018). Due to pts awareness of their treatment allocation in unblinded IO trials, ‘premature’ consent withdrawal (thus before receiving first dose of study drug) is consistently and significantly (p

**Methods**

‘Premature’ pts consent withdrawal rate was calculated for the Plin/Doc (n=174) and Doc (n=181) arms in DUBLIN-3 (NCT02504489) around the time of the first pre-planned Interim Analysis (IA).

**Results**

‘Premature’ consent withdrawal rate in DUBLIN-3 was 1.1 % for Doc and 2.3 % for Plin/Doc (p=0.53; NS). Premature consent withdrawal rate of the Doc arm was significantly (p

**Conclusions**

A single-blinded design (for pts only) is effective in preventing premature and imbalanced patient consent withdrawal. This finding may have relevance for the design of future IO trials. A second pre-planned IA for DUBLIN-3 to evaluate OS is projected for end 2019.

**Trial Registration**

NCT02504489

**References**

Fabrice Barlesi et al., Avelumab versus docetaxel in patients platinum-treated advanced non-small-cell lung cancer (JAVELIN Lung 200): an open-label, randomised, phase 3 study. The Lancet. 2018; 19 (11): 1468-1479

Roy S Herbst et al., Pembrolizumab versus docetaxel for previously treated, PD-L1-positive, advanced non-small-cell lung cancer (KEYNOTE-010): a randomised controlled trial. The Lancet. 2016; 387 (10027): 1540-1550

Hossein Borghaei et al., Nivolumab versus Docetaxel in Advanced Nonsquamous Non–Small-Cell Lung Cancer. The New England Journal of Medicine. 2015; 373:1627-1639

Achim Rittmeyer et al., Atezolizumab versus docetaxel in patients with previously treated non-small-cell lung cancer (OAK): a phase 3, open-label, multicentre randomised controlled trial. The Lancet. 2017; 389 (10066): 255-265

**Ethics Approval**

The last amendment was approved in June 2019 by WIRB and Copernicus IRB, approval number 420160463

#### P413 A Phase 1b/2 study of galunisertib in combination with nivolumab in solid tumors and NSCLC

##### Ernest Nadal, MD, PhD^1^, Mansoor Saleh, MD^2^, Santiago Ponce Aix, MD^3^, Maria Ochoa de Olza^4^, Sandip Patel, MD^5^, Scott Antonia, MD, PhD^6^, Yumin Zhao, PhD^7^, Ivelina Gueorguieva, PhD^7^, Michael Man, PhD^7^, Shawn Estrem, PhD^7^, Emin Avsar^7^, Wen Hong Lin^8^, Karim Benhadji^7^, Susan Guba^7^, Inmaculada Ales Diaz^9^, Ernest Nadal, MD, PhD^1^

###### ^1^Catalan Institute of Oncology, L'Hospitalet, Spain; ^2^University of Alabama, Birmingham, AL, United States; ^3^Hospital 12 de Octubre, Madrid, Spain; ^4^Hospital Universitario Vall d'Hebron, Barcelona, Spain; ^5^University of California, La Jolla, CA, United States; ^6^H. Lee Moffitt Cancer Center and, Tampa, FL, United States; ^7^Eli Lilly and Company, Indianapolis, IN, United States; ^8^Bristol-Myers Squibb, New York, NY, United States; ^9^Hospital Universitario Regional de Mala, Malaga, Spain

####### **Correspondence:** Ernest Nadal (esnadal@iconcologia.net)

**Background**

TGF-β promotes immune suppression. In this study, both TGF-β and PD-1 were targeted in patients with advanced refractory solid tumors and recurrent/refractory NSCLC using galunisertib, an oral small molecule inhibitor of TGF-β receptor I, in combination with nivolumab, a monoclonal antibody that binds PD-1.

**Methods**

This is a Phase 1b/2 open-label study. Eligible patients were ≥18 years old, had ECOG status ≤1, and were treatment-naive for anti-PD-1/PD-L1, or TGFβ R1 kinase inhibitor. Patients had advanced solid tumors that were refractory to standard systemic therapy (Phase 1b). NSCLC patients (Phase 2) were required to have received prior platinum-based treatment. Phase 2 portion of the trial evaluated the safety of 150 mg BID galunisertib administered on a 14 days on, 14 days off dosing schedule in combination with nivolumab given at 3 mg/kg Q2W. Efficacy, pharmacokinetics (PK) and pharmacodynamic data were also evaluated.

**Results**

15 patients were enrolled in Phase 1b and 25 in Phase 2. No dose-limiting toxicities were observed in the Phase 1 portion of the study. In the Phase 2 NSCLC cohort, the most frequent treatment-related grade 3 AEs included immune-related encephalitis, diarrhea, fatigue, ALT/ AST/GGT increase, blood alkaline phosphatase increase, abdominal distension, cutaneous rash (n=1 each), and cholestasis (n=2) that resolved or were resolving at the time of data cutoff. Two deaths on treatment (multi-organ failure and myocardial infarction), both unrelated to study treatment, were observed. 6 (24%) patients had confirmed partial response (PR) and 4 (16%) had stable disease; 1 patient had confirmed PR after initial pseudo-progression. Among the 6 responders, 5 had low or negative PD-L1 expression (≤50%). Median PFS was 5.26 months (95% CI: 1.77, 9.20) and median OS was 11.99 months (95% CI: 8.15, NR). Phase 1b PK data showed rapid absorption (1-3h) and elimination of galunisertib within 48h. Additional biomarker data including tumor mutational burden and gene-expression data will be presented.

**Conclusions**

Combination treatment of galunisertib at the RP2D of 150 mg BID for 14 days on 14 days off schedule with nivolumab 3 mg/kg Q2W was well tolerated. Preliminary efficacy was observed in a subset of patients.

**Trial Registration**

NCT02423343

**Ethics Approval**

The study was performed in accordance with the Declaration of Helsinki and was approved by ethics committees in multiple investigator sites.

#### P414 Interim results from CLASSICAL-Lung, a phase 1b/2 study of pepinemab (VX15/2503) in combination with avelumab in advanced non-small cell lung cancer patients

##### Michael Shafique, MD^1^, Terrence Fisher, PhD^2^, Elizabeth Evans, PhD^2^, John Leonard, MD PhD^2^, Desa Rae Pastore^2^, Crystal Mallow, BS^2^, Ernest Smith, PhD^2^, Andreas Schroeder, MD, PhD^3^, Kevin Chin^4^, Joseph Beck^5^, Megan Baumgart, MD^6^, Ramaswany Govindan, MD^7^, Nashat Gabrail, MD^8^, Jonathan Goldman, MD^9^, Rachel Sanborn, MD^10^, Alexander Spira, MD, PhD, FACP^11^, Nagashree Seetharamu^12^, Yanyan Lou, MD^13^, Aaron Mansfield, MD^13^, Maurice Zauderer, PhD^2^, Terrence Fisher, PhD^2^

###### ^1^Moffitt Cancer Center, Tampa, FL, United States; ^2^Vaccinex, Inc, Rochester, NY, United States; ^3^Merck KGaA, Darmstadt, Germany; ^4^EMD Serono, Rockland, MA, United States; ^5^Highlands Oncology Group, Fayetteville, AZ, United States; ^6^University of Rochester, Rochester, NY, United States; ^7^Washington University School of Medicine, St. Louis, MO, United States; ^8^Gabrail Cancer Center, Canton, OH, United States; ^9^UCLA Medical Center, Paramus, NJ, United States; ^10^Earle A. Chiles Research Institute, Portland, OR, United States; ^11^Virginia Cancer Specialists, Fairfax, VA, United States; ^12^Northwell Health, New York, NY, United States; ^13^Mayo Clinic, Jacksonville, FL, United States

####### **Correspondence:** Terrence Fisher (tfisher@vaccinex.com)

**Background**

Despite progress of immune checkpoint blockade therapies, many patients with non-small cell lung cancer (NSCLC) do not receive durable clinical benefit from these agents, and even in those who do respond initially, acquired resistance and tumor recurrence can develop. Pepinemab is an IgG4 humanized monoclonal antibody targeting semaphorin 4D (SEMA4D, CD100). In vivo preclinical studies demonstrated antibody blockade of SEMA4D promoted immune infiltration and reduced function and recruitment of immunosuppressive myeloid cells within the tumor [1,2]. Importantly, preclinical combinations of anti-SEMA4D with various immunotherapies enhanced T cell infiltration and activity, as well as durable tumor regression.

**Methods**

The CLASSICAL-Lung clinical trial evaluates the combination of pepinemab with anti-PD-L1 antibody avelumab to couple beneficial modifications of the immune microenvironment via pepinemab with immune activation via checkpoint inhibition. This ongoing phase 1b/2, open label, single arm, first-in-human combination study is designed to evaluate the safety, tolerability and efficacy of the combination in patients with advanced (stage IIIB/IV) NSCLC, including a dose escalation cohort and expansion cohorts consisting of 1) 17 immunotherapy-naïve patients and 2) 33 patients whose tumors progressed during or following immunotherapy (IO failure).

**Results**

The combination was well tolerated with no concerning safety signals identified to date. No patient experienced a treatment-related adverse event leading to permanent treatment discontinuation or death and the most frequent related AEs were grades 1 or 2 fatigue, pyrexia, or chills. Interim analysis focused on the IO failure cohort which included 22 evaluable patients. Two patients experienced a partial response (PR) with 49% and 37% tumor reduction on study following acquired resistance to prior treatment with pembrolizumab. In addition, stable disease of at least 8 weeks was observed in 11 patients and 4 patients have remained on study for ≥20 weeks. Analysis of pre- and on-treatment lung biopsies demonstrated no or low tumor burden detected in 2 patients with PR, and interestingly no detectable tumor was observed in the biopsies from 3 of 4 patients with stable disease.

**Conclusions**

Preliminary data suggest the combination of pepinemab plus avelumab is well tolerated and shows initial signals of antitumor activity in patients with IO failure. We will present updated clinical response data, as well as additional immunophenotyping of tissue biopsies, including but not limited to activated T cells, regulatory T cells, DCs, monocytes, macrophages, and importantly myeloid-derived suppressor cells (MDSCs).

**Acknowledgements**

All of the CLASSICAL-Lung investigators, site staff, and patients

**Trial Registration**

NCT03268057

**References**

1. Evans EE et al. Antibody blockade of semaphorin 4D promotes immune infiltration into tumor and enhances response to other immunomodulatory therapies. Cancer Immunol Res. 2015; 3: 689-701

2. Clavijo PE et al. Semaphorin4D inhibition improves response to immune checkpoint blockade via attenuation of MDSC recruitment and function. Cancer Immunol Res. 2019; 7(2):282-291.

**Ethics Approval**

This protocol and its amendments were approved by the appropriate IRBs at each site.

#### P415 Tumor Treating Fields (TTFields, 150 kHz) concurrent with standard of care treatment for stage 4 non-small cell lung cancer (NSCLC) in Phase 3 LUNAR Study

##### Ori Farber, Moshe Giladi, Ze'ev Bomzon, Eilon Kirson, Uri Weinberg, MD PhD

###### Novocure Ltd., Haifa, Israel

####### **Correspondence:** Uri Weinberg (weinberg@novocure.com)

**Background**

Tumor Treating Fields (TTFields) are a non-invasive, anti-mitotic treatment that disrupts the formation of the mitotic spindle and dislocation of intracellular constituents. TTFields plus temozolomide significantly extended survival in newly diagnosed glioblastoma. Efficacy of TTFields in NSCLC has been shown in preclinical models, and safety in combination with pemetrexed in a pilot study. In the Phase 3 LUNAR study [NCT02973789], we investigated if the addition of TTFields to immune checkpoint inhibitors or docetaxel increases overall survival (OS).

**Methods**

Trial Design:

Patients (N=534), with squamous or non-squamous NSCLC, are stratified by their selected standard therapy (immune checkpoint inhibitors or docetaxel), histology and geographical region. Key inclusion criteria are disease progression, ECOG 0-2, no electronic medical devices in the upper torso, and absence of brain metastasis. TTFields (150 kHz) are applied to the upper torso for at >18 hours/day until progression in the thorax and/or liver. The primary endpoint is superiority in OS between patients treated with TTFields in combination with the standard of care treatments versus standard of care treatments alone. Key secondary endpoints compare the OS in patients treated with TTFields and docetaxel versus docetaxel alone, and patients treated with TTFields and immune checkpoint inhibitors vs those treated with immune checkpoint inhibitors alone. An exploratory analysis will test non-inferiority of TTFields with docetaxel compared to checkpoint inhibitors alone. Secondary endpoints include progression-free survival, radiological response rate, quality of life based on the EORTC QLQ C30 questionnaire. The sample size is powered to detect a HR of 0.75 in TTFields-treated patients versus control group. In January 2019, an independent Data Monitoring Committee (DMC) performed a review of the LUNAR trial data collected to that point. The DMC concluded that no unexpected safety issues could be found in patients treated with the combination of immune checkpoint inhibitors and TTFields, and recommended to continue the LUNAR study as planned.

**Trial Registration**

NCT02973789

#### P416 A phase 1 dose escalation with expansion study to evaluate the safety, tolerability, pharmacokinetics, and efficacy of AMV564 in subjects with advanced solid tumors

##### Raghad Abdul Kairm, MD^1^, Anthony Tolcher^1^, Victoria Smith^2^, Sterling Eckard, PhD^2^, Jeanmarie Guenot^2^, Patrick Chun^2^

###### ^1^NEXT Oncology and Texas Oncology, San Antonio, TX, United States; ^2^Amphivena Therapeutics, Inc., South San Francisco, CA, United States

####### **Correspondence:** Patrick Chun (pchun@amphivena.com)

**Background**

Overcoming the suppressive tumor microenvironment is a major challenge in immune therapy. The critical cellular effectors of the suppressive tumor microenvironment are the myeloid-derived suppressor cells (MDSC). MDSC are elevated in both the tumor microenvironment and periphery in cancer patients and are associated with immune dysfunction, repression of anti-tumor immunity and poor response to immunotherapy. MDSC secrete a variety of immunosuppressive factors that directly inhibit both the cytolytic activity and proliferative capacity of anti-tumor T cells. AMV564 is a bivalent, bispecific antibody that engages both CD3 and CD33. Preferential binding of AMV564 to regions of high CD33 density enables the selective targeting of MDSC. Data from both *ex vivo* studies [1] and an ongoing clinical trial in acute myeloid leukemia (AML) support the ability of AMV564 to selectively deplete monocytic and granulocytic MDSC while sparing monocytes and neutrophils.

**Methods**

AMV564-301 is an open label, phase 1, multicenter, dose-escalation with expansion trial of AMV564 in patients with advanced solid tumors for which no recognized standard curative therapy options are available. The key objectives of the dose-escalation stage of the study are to characterize the safety and tolerability of AMV564 and identify a maximum tolerated dose (MTD) or a recommended phase 2 dose (RP2D) for further study. In the dose expansion stage of the study, the safety and tolerability of AMV564 will be further characterized in addition to evaluating the preliminary efficacy of AMV564. Other objectives include characterization of AMV564 pharmacokinetics, pharmacodynamics, and immunogenic potential.

Approximately 90 patients with locally advanced or metastatic solid tumors will be enrolled. The Dose Escalation Stage will include up to approximately 40 patients, depending on the dose at which the MTD/RP2D is determined, and approximately 50 additional patients will be enrolled in the Expansion Stage. AMV564 will be administered once daily as a subcutaneous injection for 14 days in each 21-day cycle. Patients will be treated until disease progression, unacceptable toxicity, or withdrawal of consent.

**Trial Registration**

NCT pending

**Reference**

1. Cheng P, Eksioglu E, Chen X, Wei M, Guenot J, Fox J, List A, Wei S. Immunodepletion of MDSC by AMV564, a novel tetravalent bispecific CD33/CD3 T cell engager restores immune homeostasis in MDS in vitro. Blood. 2017;130:51.

**Ethics Approval**

This study will be approved by the Institutional Review Board (IRB) or Independent Ethics Committee (IEC) at each participating institution prior to patient enrollment.

#### P417 ATLAS™ identifies relevant neoantigens for therapeutic anti-tumor vaccination and may serve as a biomarker for efficacy of immunotherapy of solid tumors

##### Parul Agnihotri, Tulin Dadali, Parul Agnihotri, PhD

###### Genocea Biosciences Inc, Cambridge, MA, United States

####### **Correspondence:** Tulin Dadali (tulin.dadali@genocea.com)

**Background**

Mutation-derived neoantigen cancer vaccines are promising as next generation cancer therapies. However, the success of vaccination is dependent on the ability to identify the right neoantigens for vaccine inclusion, which remains a critical challenge. Computationally-identified neoantigens do not necessarily generate immunogenic responses. Recently, we reported interim immunogenicity results from the ongoing GEN-009 personalized immunotherapy Phase 1/2a clinical trial (NCT03633110). For GEN-009, ATLAS, an ex vivo, cell-based assay selects neoantigens for vaccine inclusion based on a patient’s own pre-existing T cell responses. The interim results revealed that vaccination elicited T cell responses to over 98% of administered peptides. Here, we explore the relationship between ATLAS readouts and immunogenicity outcomes in the same subjects.

**Methods**

Antigens were profiled by expressing each mutation, identified by whole exome sequencing, as individual clones in E. coli which are subsequently processed by each subject’s own antigen presenting cells and presented to autologous CD4+ or CD8+ T cells. Antigen-specific responses were determined based on cytokine secretion in the supernatant after overnight incubation. GEN-009, composed of 4 pools of 1-5 unique ATLAS-identified neoantigen-specific peptides combined with Hiltonol® was administered to each subject. Both ex vivo and ten day in vitro stimulated FluoroSpot assays were performed on unsorted PBMC and CD4- and CD8-sorted T cells at baseline and 50 days post vaccination to identify peptides to which T cells from the vaccinated patients responded.

**Results**

In the first cohort of six patients, ATLAS identified neoantigens by recalling both stimulatory and inhibitory neoantigen-specific T cell responses. One subject, who had a greater proportion of inhibitory to stimulatory responses detected, progressed prior to vaccination while no vaccinated patients have experienced progressive disease. Compared to NetMHCPan results, more than half of the ATLAS-identified neoantigens were not predicted. Moreover, the predicted epitopes did not result in better immunogenicity outcomes post-vaccination than the non-predicted ATLAS-identified neoantigens. Comprehensively profiling T cell responses over time shows consistency of results in patients with no evidence of disease.

**Conclusions**

Neoantigens selected by immune response data from ATLAS and included in the GEN-009 vaccine were immunogenic and many were not algorithm-predicted, confirming that ATLAS identifies relevant neoantigens. ATLAS will be useful for profiling epitope spread in tumor-bearing subjects post-vaccination. The proportion of inhibitory to stimulatory neoantigen-specific responses may be a biomarker of immunotherapy success. Combination of GEN-009 with standard-of-care checkpoint blockade therapy is currently ongoing.

**Trial Registration**

ClinicalTrials.gov NCT03633110

**Ethics Approval**

The study was approved by Western Institutional Review Board, approval number 1-1078861-1.

#### P418 Intratumoral IL-12 plus pembrolizumab combination therapy in treatment refractory solid tumors: a safety and biomarker analysis

##### Pablo Fernandez-Penas, MD, PhD^1^, Matteo Carlino, MBBS, PhD, BMedSC, F^1^, Victoria Atkinson, MD^2^, Melinda Telli^3^, Rohit Joshi^4^, Sajev Thomas^5^, Katy Tsai, MD^6^, Rachel Roberts-Thomson^4^, Andrew Haydon, MBBS PhD^7^, Andrew Mant^8^, Tom Van Hagen^9^, Katharine Cuff^10^, Bianca Devitt^11^, Igor Puzanov, MD, MSCI, FACP^12^, Marcus Butler, MD^13^, Catalin Mihalcioiu^14^, Hatem Soliman, MD^15^, John Hyngstrom, MD^16^, Mecker Moller^17^, Gregory Daniels, MD, PhD^18^, Eric Whitman, MD, FACS^19^, Erica Browning, BS^20^, Reneta Hermiz^20^, Lauren Svenson^20^, Jack Lee^20^, Donna Bannavong^20^, Jendy Sell^20^, Kellie Malloy^20^, David Canton, PhD^20^, Christopher Twitty^20^, Adil Daud, MBBS MD^6^, Alain Algazi, MD^6^

###### ^1^Westmead Hospital, University of Sydney, Westmead, Australia; ^2^Princess Alexandra Hospital, University of Queensland, Woolloongabba, Australia; ^3^Stanford University Medical School, Stanford, CA, United States; ^4^Adelaide Oncology and Haematology, Adelaide SA, Australia; ^5^UF Health Cancer Center, Orlando Health, Orlando, FL, United States; ^6^University of California San Francisco, San Francisco, CA, United States; ^7^The Alfred Hospital, Melbourne, Australia; ^8^Box Hill Hospital, Box Hill, Victoria, Australia; ^9^St. John of God Hospital, Subiaco, Australia; ^10^Princess Alexandra Hospital, Woolloongabba QLD, Australia; ^11^Eastern Health Clinical School, Box Hill, VIC, Australia; ^12^Roswell Park Cancer Institute, Buffalo, NY, United States; ^13^Princess Margaret Cancer Centre, Toronto, Canada; ^14^McGill University Health Centre, Montreal, QC, Canada; ^15^Moffitt Cancer Center, Tampa, FL, United States; ^16^Huntsman Cancer Institute and Hospital, Salt Lake City, UT, United States; ^17^Sylvester Comprehensive Cancer Center, University of Miami, Miami, FL, United States; ^18^University of California San Diego, La Jolla, CA, United States; ^19^Atlantic Health System, Morristown, NJ, United States; ^20^OncoSec Medical Inc., San Diego, CA, United States

####### **Correspondence:** Alain Algazi (Alain.algazi@ucsf.edu)

**Background**

Intratumoral inflammation, including IL-12 expression and intratumoral T cell infiltration, is a prerequisite for response to anti-PD-1 therapies. Previously, we demonstrated that enhanced intratumoral IL-12 expression via injection of plasmid IL-12 (tavokinogene telseplasmid; TAVO) followed by electroporation (IT-tavo-EP) can increase TIL infiltration, ratios of CD8+ T cell:suppressive immune subsets, and IFN-gamma gene signatures, converting weakly immunogenic tumors into highly inflamed, immunologically active lesions that regress with anti-PD-1 antibody therapy. Here, we present further support for our hypothesis that local IT-tavo-EP induces local and systemic immune modulation with minimal systemic toxicity.

**Methods**

Melanoma (KEYNOTE-695) and mTNBC (KEYNOTE-890) patients were treated every three weeks with IT-tavo-EP on days 1, 5, and 8 of every odd numbered cycle. Clinical toxicity was assessed at 3-week intervals and graded by CTCAE v4. In addition, pre- and post-treatment tumor biopsies and peripheral blood samples were interrogated for treatment-related changes in the frequency of CD8+ TIL and other key IL-12-driven peripheral immune cell populations. In particular, we examined circulating short-lived effector T cells (SLECs, KLRG1+/CD127-), which are induced by IL-12 exposure, and granulocytic myeloid derived suppressor cells (gMDSCs or PMN-MDSCs), which serve a regulatory function, inhibiting effective anti-tumor immune responses.

**Results**

62 patients were assessed including 46 patients with anti-PD-1 antibody-refractory melanoma, and 16 patients with chemotherapy-refractory mTNBC. TAVO in combination with pembrolizumab was well tolerated with only 2 of 46 (4.3%) patients from KEYNOTE-695 (cellulitis and presyncope) and 1 of 16 (6.3%) from KEYNOTE-890 (acute renal failure) experiencing grade 3 treatment-related adverse events (TRAEs). Paired biopsies were available from both advanced melanoma patients and mTNBC patients. Flow cytometry on fresh biopsies from the KEYNOTE-695 revealed significant increases in CD8+ T cells after 1 cycle of treatment. Despite previous data demonstrating non-detectable circulating IL-12 levels after treatment with TAVO, paired peripheral blood analysis revealed a treatment-related increase of KLRG1+/CD127- SLECs as well as a treatment-related reduction of MDSCs in the periphery predominantly in responding patients.

**Conclusions**

TAVO + pembrolizumab continues to be well tolerated in patients with advanced solid tumors and peripheral blood analyses demonstrates both local and most importantly, systemic signals of IL-12 mediated anti-tumor immunity in the absence of clinical signs of systemic IL-12 exposure. Thus, TAVO acts as an in situ vaccine to potentiate the anti-tumor activity of pembrolizumab with a favorable toxicity profile.

**Trial Registration**

NCT03132675; NCT03567720

**Ethics Approval**

These studies were approved by the appropriate ethics committees.

**Consent**

Written informed consent was obtained from the patient for publication of this abstract and any accompanying images. A copy of the written consent is available for review.

#### P419 A phase 1/2 study of GB1275, a novel CD11b modulator, as monotherapy and with an anti-PD-1 antibody in specified advanced solid tumors or with chemotherapy in metastatic pancreatic cancer (mPDAC)

##### Johanna Bendell^1^, Drew Rasco, MD^2^, Wungki Park, MD^3^, Lei Zhou, MD, MS^4^, Anna Galkin, PhD^4^, Debbie Slee, PhD^4^, Laura Carter, PhD^4^, David Nickle, PhD^4^, Rebecca Tran, MS^4^, Jack Li, PhD^4^, Beatrice Ferguson, MS^4^, Jakob Dupont, MD^4^, Vineet Gupta, PhD^5^, Eileen O'Reilly^3^

###### ^1^Sarah Cannon Research Institute, Nashville, TN, United States; ^2^The START Center for Cancer Care, San Antonio, TX, United States; ^3^Memorial Sloan Kettering Cancer Center, New York, NY, United States; ^4^Gossamer Bio, San Diego, CA, United States; ^5^Rush University Medical Center, Chicago, IL, United States

####### **Correspondence:** Johanna Bendell (ttobore@samornbiosciences.com)

**Background**

Tumor influx of CD11b-expressing Myeloid-Derived Suppressor Cells (MDSCs) and M2 Tumor-Associated Macrophages (TAMs) creates an immunosuppressive tumor microenvironment that is associated with resistance to anti-PD-1 antibody therapy [1, 2, 3]. GB1275 is a novel, first-in-class, CD11b modulator that in vivo led to reduced MDSCs and TAMs at the tumor site, repolarized M2 immuno-suppressive TAMs towards an M1 phenotype, and subsequently increased tumor infiltration of activated CD8+ T cells [4]. In combination settings with an anti-PD-1 antibody or chemotherapy, these immunomodulatory effects translated into potent anti-tumor effects and prolonged survival in orthotopic PDAC models [4]. We hypothesize that GB1275 administration can alleviate myeloid cell-mediated immunosuppressive effects and improve cancer treatment outcomes.

**Methods**

This is an open-label, first-in-human study consisting of a Phase 1 Dose Escalation phase with Regimen A using GB1275 monotherapy and Regimen B using GB1275 with an anti-PD-1 antibody in pts with pancreatic, esophageal, gastric/GEJ, triple negative breast, castration resistant prostate, or Microsatellite Stable Colorectal Cancer (MSS CRC) and Regimen C (GB1275 with Nab-paclitaxel + Gemcitabine (Nab-P+Gem)) in mPDAC, followed by a Phase 2 Expansion phase with three cohorts planned: newly diagnosed stage IV mPDAC, MSS CRC and PD-L1+ gastric/GEJ cancer. The study starts with Regimen A with Regimen B starting after the completion of the first few cohorts of Regimen A. Regimen C will start when Regimen A is completed. Key Inclusion Criteria: Age ≥18 years, histologically confirmed locally advanced/metastatic tumor specified, ECOG 0-1, prior immunotherapy is permissible in Dose Escalation phase for Regimen A and B, but not for the expansion or Regimen C. Key Exclusion Criteria: untreated or symptomatic CNS metastasis, received prior myeloid targeting agent or other prohibited medications, history of clinical significant cardiovascular disease. Pts with active autoimmune disease requiring systemic therapy will be excluded from Regimen B. Primary objectives for the Dose Escalation phase are to determine the MTD/RP2D and PK profile of GB1275 monotherapy and in combination with an anti-PD-1 antibody, and safety in combination with Nab-P+Gem. The primary objective for the Basket Expansion phase is to assess efficacy.

Statistical Considerations: 3+3 design for the Dose Escalation Phase and Simon’s 2-stage design for Expansion Phase. AEs graded per CTCAE v5.0, responses per RECIST v1.1. The study is open for recruitment and clinical trial registration on clinicaltrials.gov is pending (NCTxxxxx).

**References**

1. Fleming, V., Hu, X., Weber, R., Nagibin, V., Groth, C., Altevogt, P., et al. Targeting myeloid-derived suppressor cells to bypass tumor-Induced immunosuppression. Front Immunol. 2018; 9: 398.

2. Kumar, V., Patel, S., Tcyganov, E. and Gabrilovich, D. I. The nature of myeloid-derived suppressor cells in the tumor microenvironment. Trends Immunol. 2016; 37(3): 208-220.

3. Mantovani, A., Sozzani, S., Locati, M., Allavena, P. and Sica, A. Macrophage polarization: tumor-associated macrophages as a paradigm for polarized M2 mononuclear phagocytes. Trends Immunol. 2002; 23(11): 549-555.

4. Panni R., Herndon J., Zuo C., et al. Agonism of CD11b reprograms innate immunity to sensitize pancreatic cancer to immunotherapies. Sci Transl Med. 2019; 11: eaau9240.

**Ethics Approval**

The study was approved by the local IRB at each participating study site.

#### P420 Broad immunogenicity from GEN-009, a neoantigen vaccine using ATLAS™, an autologous immune assay, to identify immunogenic and inhibitory tumor neoantigens

##### Roger Cohen, MD^1^, Melissa Johnson, MD^2^, Przemyslaw Twardowski, MD^3^, Mark Stein, MD^4^, Ulka Vaishampayan, MD^5^, Maura Gillison, MD, PhD^6^, Lisa McNeil, PhD^7^, Louisa Dowal, PhD^7^, James Foti, PhD^7^, Parul Agnihotri, PhD^7^, Daniel DeOliveira, PhD^7^, Manish Jain, MS^7^, Jessica Price^7^, Richard Hernandez^7^, Arthur DeCillis, MD^7^, Narinderjeet Singh, MS, MBA^7^, Thomas Davis, MD^7^, Jessica Flechtner, PhD^7^

###### ^1^University of Pennsylvania, Philadelphia, PA, United States; ^2^Sarah Cannon Research Institute, Nashville, TN, United States; ^3^John Wayne Cancer Institute, Duarte, CA, United States; ^4^Columbia University Medical Center, New York, NY, United States; ^5^Karmanos Cancer Institute, Detroit, MI, United States; ^6^MD Anderson Cancer Center, Houston, TX, United States; ^7^Genocea, Cambridge, MA, United States

####### **Correspondence:** Thomas Davis (tom.davis@genocea.com)

**Background**

Tumor-specific neoantigens provide personalized targets for immunotherapy. Vaccines against epitopes predicted by in silico approaches very rarely induce CD4+ and CD8+ ex vivo T cell responses regardless of formulation. ATLAS selects neoantigens for vaccine inclusion using ex vivo screening of all patient-specific mutations to identify pre-existing CD4+ or CD8+ T cell responses and to exclude inhibitory peptides that may suppress immunity and potentially accelerate tumor progression. Preliminary data suggest that the inhibitory peptide profile may predict tumor response to immunotherapy.

**Methods**

GEN-009-101 is a phase 1/2a study testing safety, immunogenicity and clinical activity in immune responsive tumors (NCT03633110). After next-generation tumor sequencing and ATLAS testing of autologous leukocytes, each personalized vaccine is created using up to 20 stimulatory synthetic long peptides adjuvanted with poly-ICLC. The immunogenicity pilot includes 8 patients in remission (NED), who received a course of GEN-009 monotherapy.

**Results**

Eight patients have participated and reached the primary immunogenicity readout at day 50 (some data pending). The 24 doses given across all patients have induced only grade 1/2 adverse events consistent with those expected from the poly-ICLC adjuvant alone, and no DLTs. ATLAS results show high interpatient variability as described previously. In an interim analysis of patients, vaccination has generated both CD8 and CD4 T cell responses measured by ex vivo fluorospot (Table 1). Ten-day in vitro stimulation (IVS) fluorospot assays confirm even broader immune responses. Overall, T cell responses were measured to 98% of administered peptides.

**Conclusions**

GEN-009 is a neoantigen vaccine that targets tumor specific immune antigens recognized by the individual patient’s lymphocytes and likely expressed by tumor cells. Immunogenicity data show that ATLAS can, with very high frequency, identify relevant neoantigens and exclude putatively deleterious (immune inhibitory) antigens. Clinical vaccination together with Standard of Care PD-1 blockade-based regimens is in progress.

**Trial Registration**

ClinicalTrials.gov NCT03633110

**Ethics Approval**

The study was approved by Western Institututional Review Board, approval number 1-1078861-1.

**Table 1 (abstract P420).**
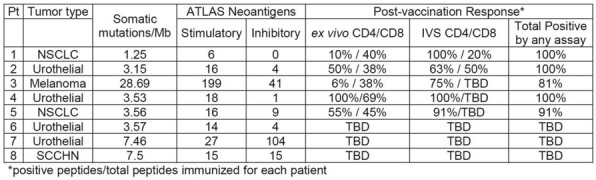
See text for description

#### P421 Phase 1 study of the safety, tolerability and preliminary anti-tumor activity of COM701 monotherapy in patients with advanced solid tumors

##### Ecaterina Dumbrava, MD^1^, Gini Fleming, MD^2^, Erika Hamilton, MD^3^, Ryan Sullivan, MD^4^, Amita Patnaik, MD FRCP(C)^5^, Kyriakos Papadopoulos, MD^5^, Adam ElNaggar, MD^6^, John Hunter, PhD^7^, Judy Olweny^7^, Adeboye Adewoye, MD^7^, Bartosz Chmielowski, MD, PhD^8^, Dale Shepard, MD PhD^9^, Manish Sharma, MD^10^, Emerson Lim, MD^11^, Daniel Vaena, MD^6^, Drew Rasco, MD^5^

###### ^1^The MD Anderson Cancer Center., Houston, TX, United States; ^2^The University of Chicago, Chicago, IL, United States; ^3^Sarah Cannon Research Institute/TN Oncology, Nashville, TN, United States; ^4^Massachusetts General Hospital, Needham, MA, United States; ^5^The START Center for Cancer Care, San Antonio, TX, United States; ^6^West Cancer Center and Research Institute, Memphis, TN, United States; ^7^Compugen USA Inc, South San Francisco, CA, United States; ^8^University of California Los Angeles, Los Angeles, CA, United States; ^9^Cleveland Clinic, Cleveland, OH, United States; ^10^START - Midwest Cancer Center, Chicago, IL, United States; ^11^Columbia University Medical Center, New York City, NY, United States

####### **Correspondence:** Ecaterina Dumbrava (EEIleana@mdanderson.org)

**Background**

COM701 is a novel first-in-class immune checkpoint inhibitor (ICI) of poliovirus receptor related immunoglobulin domain (PVRIG) [1]. It inhibits the binding of PVRIG with its ligand, PVRL2. PVRIG is a member of the DNAM/TIGIT signaling axis regulating the activity of T/NK-cells. In preclinical experiments we have demonstrated that PVRIG inhibition alone and in combination with anti-PD-1 and/or TIGIT blockers leads to activation of T cells in the tumor microenvironment generating an anti-tumor immune response and tumor growth inhibition [1]. Although ICI revolutionized cancer treatment, there is an urgent need to develop treatments for patients who are refractory or relapse after treatment with ICI. We hypothesized that COM701 will be safe, tolerable and demonstrate preliminary anti-tumor activity.

**Methods**

A phase 1a, dose-escalation of COM701 monotherapy utilizing a hybrid accelerated and 3+3 study design was conducted to determine safety, tolerability, to assess the pharmacokinetics (PK), pharmacodynamics, to determine the recommended phase 2 dose and to evaluate preliminary anti-tumor activity of COM701. Patients with performance status ECOG 0-1 and advanced solid tumors who failed standard of care treatments were eligible for inclusion. Prior ICIs were permissible. COM701 0.01, 0.03, 0.1, 0.3, 1, 3 and 10 mg/kg IV every 3 weeks were administered until progression, intolerable toxicity or investigator or patient discretion. Adverse events were reported per CTCAE v4.03 and anti-tumor activity was evaluated using RECIST v1.1. Dose-limiting toxicities (DLTs) were evaluated within a 21-day window. Data cutoff date was August 09, 2019.

**Results**

A total of 13 patients were enrolled and treated during dose escalation of COM701, including 6 patients with metastatic colorectal cancer (CRC), 5 with microsatellite stable status (MSS) and 1 unknown. Patients were heavily pretreated with a median of 7 prior anticancer therapies (range 2-15). No DLTs have been reported up to 10 mg/kg COM701 dose level. The most frequent toxicities were fatigue (8%), abdominal pain (6%). Likely immune-related adverse events: elevated TSH and rash were observed in 2 patients. Overall 7/13 patients (54%) maintained best response of stable disease (SD) ≥12 weeks (13.6 – 43 weeks), including 5/6 (83%) of patients with CRC. Five patients continue on study treatment. Peripheral PVRIG receptor occupancy (≥90%) was demonstrated at ≥1mg/kg dose of COM701 and PK profile supports Q3 weekly dosing.

**Conclusions**

COM701 monotherapy demonstrates an acceptable safety and tolerability profile with preliminary anti-tumor activity in a patient population that had received multiple prior anti-cancer therapies. Updated data will be presented at the conference.

**Trial Registration**

Clinical trial identification: NCT03667716.

**Reference**

1. Spencer L, Ofer L et al, Discovery of COM701, a therapeutic antibody targeting the novel immune checkpoint PVRIG, for the treatment of cancer. J Clin Oncol. 2017; (suppl; abstr 3074)

**Ethics Approval**

The study was approved by each site's ethics board.

**Consent**

Written informed consent was obtained from the patient for publication of this abstract and any accompanying images. A copy of the written consent is available for review by the Editor of this journal.

#### P422 Phase 1 study of COM701 monotherapy and in combination with nivolumab in patients with advanced solid tumors

##### Ecaterina Dumbrava, MD^1^, Gini Fleming, MD^2^, Erika Hamilton, MD^3^, Ryan Sullivan, MD^4^, Amita Patnaik, MD FRCP(C)^5^, Kyriakos Papadopoulos, MD^5^, Adam ElNaggar, MD^6^, John Hunter, PhD^7^, Judy Olweny^7^, Adewoye Adewoye, MD^7^, Bartosz Chmielowski, MD, PhD^8^, Dale Shepard, MD PhD^9^, Manish Sharma, MD^10^, Emerson Lim, MD^11^, Daniel Vaena, MD^6^, Drew Rasco, MD^5^

###### ^1^The University of Texas MD Anderson Cancer Center, Houston, TX, United States; ^2^The University of Chicago, Chicago, IL, United States; ^3^Sarah Cannon Research Institute/Tennessee Oncology, Nashville, TN, United States; ^4^Massachusetts General Hospital, Harvard Medical School, Needham, MA, United States; ^5^The START Center for Cancer Care, San Antonio, TX, United States; ^6^West Cancer Center and Research Institute, Memphis, TN, United States; ^7^Compugen USA Inc, South San Francisco, CA, United States; ^8^University of California Los Angeles, Los Angeles, CA, United States; ^9^Cleveland Clinic, Cleveland, OH, United States; ^10^The START-Midwest Center for Cancer Care, Chicago, IL, United States; ^11^Columbia University Medical Center, New York City, NY, United States

####### **Correspondence:** Ecaterina Dumbrava (EEIleana@mdanderson.org)

**Background**

COM701 is a novel first-in-class immune checkpoint inhibitor (ICI) of poliovirus receptor related immunoglobulin domain (PVRIG) [1]. It inhibits the binding of PVRIG with its ligand, PVRL2. Nivolumab is an anti-PD-1 antibody approved in patients with several malignancies [2]. PVRIG is a member of the DNAM/TIGIT signaling axis regulating the activity of T/NK-cells. PD-1 inhibitors play an important role in this axis by modulating DNAM activation [3]. In preclinical experiments we have demonstrated that PVRIG inhibition alone and in combination with anti-PD-1 leads to activation of T cells in the tumor microenvironment generating an anti-tumor immune response and tumor growth inhibition [1]. Although ICI revolutionized cancer treatment there is an urgent need to develop treatments for patients who are refractory or relapse after treatment with ICI. We hypothesized that COM701 will be safe and tolerable and demonstrate preliminary antitumor activity as monotherapy and in combination with nivolumab in patients with advanced solid tumors.

**Methods**

This is a phase 1 study with single patient cohorts and 3+3 study design of COM701 in escalating doses as monotherapy IV Q3 weeks and in combination with nivolumab 360mg IV Q3 weeks. Key Inclusion Criteria: Age ≥18 years, histologically confirmed advanced solid tumor, performance status ECOG 0-1, prior anti-PD-1, anti-PD-L1, anti-CTLA-4, OX-40, CD137 treatments are permissible. Key Exclusion Criteria: Active autoimmune disease requiring systemic therapy in the last 2 years, symptomatic interstitial or inflammatory lung disease, untreated or symptomatic central nervous system metastases. Primary objectives: to evaluate the safety and tolerability of COM701 monotherapy and in combination with nivolumab measured by the incidence of adverse events and dose-limiting toxicities (21-day window), to evaluate the pharmacokinetics of COM701, and to identify the maximum tolerated dose and/or the recommended dose for expansion as monotherapy and in combination with nivolumab. Secondary objectives: to characterize the immunogenicity and preliminary antitumor activity of COM701 in combination with nivolumab. Statistical Considerations: AEs will be reported as per CTCAE v4.03 and tumor responses will be evaluated per RECIST v1.1. Analyses of objectives are descriptive and hypothesis generating.

**Results**

At the time of submission no DLTs have been observed up to dose level 7 of COM701 monotherapy and dose level 1 of COM701 in combination with nivolumab 360mg IV Q3 weeks.

**Conclusions**

Assessment of safety and tolerability is ongoing for all patients. Updated results will be presented at the congress.

**Trial Registration**

Clinical trial identification: NCT03667716.

**References**

1. Spencer L, Ofer L et al, Discovery of COM701, a therapeutic antibody targeting the novel immune checkpoint PVRIG, for the treatment of cancer. J Clin Oncol. 2017; (suppl; abstr 3074)

2. Nivolumab package insert. http://packageinserts.bms.com/pi/pi_opdivo.pdf. Accessed 07/22/2019.

3. Wang B, Zhang W et al., Combination cancer immunotherapy targeting PD-1 and GITR can rescue CD8+ T cell dysfunction and maintain memory phenotype. Sci. Immunol. 2018; Nov 2:3(29).

**Ethics Approval**

The study was approved by the Investigational Review Board/Ethics Committee of the participating sites.

#### P423 SURPASS trial design: A phase 1 dose escalation trial to assess safety and efficacy of ADP-A2M4CD8 in HLA-A2+ patients with MAGE-A4+ tumors

##### David Hong, MD^2^, Marcus Butler^3^, Melissa Johnson, MD^4^, Tanner Johanns^5^, Francine Brophy^1^, Rebecca Dryer-Minnerly, PhD^1^, Trupti Trivedi, MS^1^, Rafael Amado, MD^1^, Paula Fracasso, MD, PhD^1^

###### ^1^Adaptimmune, Philadelphia, PA, United States; ^2^MD Anderson Cancer Center, Houston, TX, United States; ^3^Princess Margaret Cancer Centre, Toronto, Canada; ^4^Sarah Cannon, Nashville, TN, United States; ^5^Washington University in St. Louis, St. Louis, MO, United States

####### **Correspondence:** Paula Fracasso (paula.fracasso@adaptimmune.com)

**Background**

ADP-A2M4CD8 specific peptide enhanced affinity receptor (SPEAR) T-cells are genetically engineered to target MAGE-A4+ tumors in the context of HLA-A*02. ADP-A2M4CD8 are autologous CD4+ and CD8+ T-cells that express a high affinity MAGE-A4-specific T-cell receptor (TCR) and an additional CD8α co-receptor. The ADP-A2M4 TCR is being explored in a pilot study (NCT03132922), where clinical responses have been observed and the TCR has been well tolerated in doses up to 10 × 10^9 transduced cells. Because CD4+ T-cells have a weak effector function in response to class I antigens, a CD8α co-receptor was genetically engineered alongside the TCR in ADP-A2M4CD8, to increase TCR binding avidity and enhance the polyfunctional response of engineered CD4+ T-cells against MAGE-A4+ tumors. This approach is intended to widen the immune response to the tumor and improve depth and durability of clinical responses.

**Methods**

This phase 1, dose-escalation, open-label trial (SURPASS Trial) will characterize safety, tolerability, and antitumor activity across multiple tumor types. Patients who are HLA-A*02+ with MAGE-A4+ advanced esophageal, esophagogastric junction, gastric, head and neck cancers, non-small cell lung, ovarian and urothelial carcinoma, melanoma, myxoid/round cell liposarcoma, or synovial sarcoma and who meet all other inclusion criteria are eligible. Up to 30 patients will be enrolled.

Following apheresis, T-cells are isolated, transduced with CD8α_MAGE-A4c1032TCR, and expanded. Prior to ADP-A2M4CD8 infusion, patients will receive lymphodepletion consisting of fludarabine (30 mg/m2/day x 4 days) and cyclophosphamide (600 mg/m2/day x 3 days). During dose escalation, patients will be treated in one of three ADP-A2M4CD8 dose groups. The initial dose of ADP-A2M4CD8 will be 0.8 × 10^9 - 1.2 × 10^9 to be escalated to 1.2 × 10^9 - 3 × 10^9 and then to 3.0 × 10^9 - 6.0 × 10^9 transduced cells in a modified 3 + 3 dose escalation scheme. Patients will be monitored for dose-limiting toxicities (DLTs). Once the tolerability and safety of the lymphodepletion regimen and cell dose has been demonstrated, the dose range will increase to a maximum of 10 × 10^9 transduced cells in the expansion phase. Disease will be assessed per RECIST v1.1 by CT/MRI at weeks 4, 8, 16, and 24, and every 3 months for 2 years, then every 6 months up to 15 years or until progression. Blood and tumor biopsy samples will be obtained pre- and post-infusion to evaluate safety, monitor persistence of transduced T-cells, and identify tumor-intrinsic correlates of response or resistance to therapy.

**Trial Registration**

NCT04044859

**Ethics Approval**

This trial is under review by the local institutional review boards for each proposed study center.

#### P424 Preliminary safety, efficacy and immunogenicity results from a phase 1/2a study (DIRECT-01) of cancer neoantigen DNA vaccine VB10.NEO in patients with locally advanced or metastatic solid tumors

##### Jürgen Krauss^1^, Angela Krackhardt^2^, Elke Jaeger^3^, Anja Williams^1^, Reza Rafiyan^3^, HEDDA WOLD, MSc^4^, LIsa Gerner^4^, Monika Sekelja^4^, Agnete Fredriksen, PhD^4^, Karoline Schjetne^4^, Mads Axelsen^4^, Agnete Fredriksen, PhD^4^, Hedda Wold, MSc^4^

###### ^1^NCT, University Hospital Heidelberg, Heidelberg, Germany; ^2^Klinicum rechts der Isar, TUM, Munich, Germany; ^3^Krankenhaus Nordwest, Frankfurt, Germany; ^4^Vaccibody AS, OSLO, Norway

####### **Correspondence:** Hedda Wold (hwold@vaccibody.com)

**Background**

Generation of potent neoantigen-specific T-cell responses has shown promising preclinical efficacy as well as clinical responses, especially in patients with high tumor mutational burden (TMB). VB10.NEO is a DNA vaccine with intrinsic adjuvant designed for delivery of 20 personalized neoepitopes to antigen presenting cells. Preliminary results from the ongoing phase 1/2a study treating patients with solid tumors are presented. The study (NCT03548467) was approved by Central Ethics Committee in Heidelberg, Germany.

**Methods**

Patients with melanoma, NSCLC, clear RCC, urothelial cancer or SCCHN who did not reach complete responses after >12 weeks of immune checkpoint inhibitor (CPI) therapy as standard of care were eligible. After patient-specific vaccine production, patients receive up to 14 vaccinations as intramuscular jet injections over a one-year period. CPI treatment continued. CT/MRI scans were performed according to hospitals’ routine. Immune responses were assessed by IFN-γ ELISpot.

**Results**

At July 22, 2019 data cut-off, 15 patients (9 RCC, 4 SCCHN, 1 Melanoma, 1 NSCLC) had received ≤ 11 VB10.NEO vaccinations. Most common AEs were injection site reactions which all subsided within days. 6 patients reported ≥ Grade 3 AEs, the most frequent ones were injection-related hypertensive episodes normalizing within hours. In the 4 patients assessed with low TMB (2 RCC, 2 SCCHN) strong T-cell responses towards 63% of selected neoepitopes were observed after 3-6 vaccinations. An amplification of existing neoepitope-specific T-cells (average of 250-fold increase) as well as de novo responses were observed suggesting that VB10.NEO increases both the breadth and strength of the immune responses.

10 patients were evaluable with >1 scan after VB10.NEO start (after being on CPI for 9-32 months). 4 patients (3 low TMB, 1 medium TMB) started VB10.NEO with progressive disease (PD) development, of which 3 showed as stable disease (SD) in target lesions after vaccination (followed up to 7 months), one developed PD after 5 months. New lesions were detected in 2 patients. 6 patients (5 low TMB, 1 medium TMB) had SD at VB10.NEO start, 5 remained SD (followed up to 9 months), while one had a best target lesion reduction of 40%. Updated data will be presented.

**Conclusions**

Vaccinations with VB10.NEO in addition to CPI were well tolerated. VB10.NEO induces strong T cell responses towards personalized neoepitopes; both novel T cell specificities and amplification of pre-existing T cell responses were observed. Clinical signs of effect on tumor size will continuously be monitored in the trial and early signs are promising.

**Trial Registration**

NCT03548467

**Ethics Approval**

The study was approved by Central Ethics Committee in Heidelberg.

#### P425 Phase 1b study of GX-I7, a long-acting interleukin-7, evaluating the safety, pharmacokinetics and pharmacodynamics profiles in patients with advanced solid cancers

##### Minkyu Heo^1^, Minkyu Heo^1^, Tae Won Kim, MD, PhD^2^

###### ^1^Genexine, Inc, New York, NY, United States; ^2^Asan Medical Center, Seoul, Korea

####### **Correspondence:** Tae Won Kim (twkimmd@amc.seoul.kr)

**Background**

Cancer and treatment-related lymphopenia is associated with higher mortality in patients with various oncologic malignancies. Interleukin-7(IL-7), a homeostatic cytokine of T lymphocytes, plays a critical and non-redundant role in T cell development and homeostasis of mature T lymphocytes. IL-7 is a potent amplifier of naïve and memory T cells, thereby correcting T cell deficiency and contributing to immune reconstitution. This may result in significant clinical benefit when combined with lymphopenia-inducing radiation/chemotherapy or immunotherapy where anti-tumor effects are mediated by T cells.

**Methods**

A phase 1b study was conducted to assess the safety, pharmacokinetics and pharmacodynamics of single-agent GX-I7(human IL-7 fused to the half-life extension hyFcTM) administered intramuscularly q3w to advanced solid cancer patients who have no available effective treatments (n=21). The dose escalation phase followed the 3+3 design of GX-I7 doses ranged from 60 to 1,200 μg/kg. Adverse events, PK, and subset analysis of peripheral blood monocytes(PBMCs) were evaluated.

**Results**

GX-I7 was well tolerated without DLT and cytokine release syndrome. Injection site reactions were the most common treatment-emergent adverse events, which were Grade1 or 2 and resolved. GX-I7 was slowly but steadily absorbed with a Tmax range of 12-48 hours with delay in higher doses. Following GX-I7 administration, up to 4-fold increase in absolute lymphocyte count(ALC) were demonstrated. Importantly, the number of various subsets(naïve, TEM, TCM and TEMRA) of both CD4+ and CD8+ T cells was in a greater magnitude than that of ALC, coupled with enhanced expression of Ki-67 peaked at day 7. Among T cell subsets, increase in naïve CD4+ and CD8+ T cells was most prominent. IL-7 receptor alpha(CD127) expression was reduced during the first week, and recovered to baseline after 2~3 weeks post GX-I7 administration. CCR5 expression in both CD4+ and CD8+ T cells increased transiently in a dose dependent manner, suggesting GX-I7 promotes migration of T cell to tumor environment. No apparent increases in the number of other immune cells (NK cell, monocytes, B cells) were observed.

**Conclusions**

A 3-week interval repeated IM administration of GX-I7 appears to be well tolerated in dose range of 60 – 1,200 μg/kg in advanced solid cancer patients. Following GX-I7 administration, dose-dependent increase of ALC and T cell subsets(not Treg) were observed. These findings suggest that GX-I7 can be an excellent combination partner for chemo-radiation, cancer vaccines and immune checkpoint inhibitors such as anti-PD-1/PD-L1 antibodies, by increasing T lymphocytes and thereby contributing to enhanced anti-tumor effects.

**Trial Registration**

ClinicalTrials.gov Identifier: NCT03478995

**Ethics Approval**

The study was approved by the Severance Hospital, Asan Medical center, and Catholic Medical Center Institutional Review Board, protocol number GX-I7-CA-003.

#### P426 A first-in-human phase 1, multicenter trial of toll-like receptor (TLR) 7 agonist DSP-0509 as monotherapy and in combination with pembrolizumab in adult patients with advanced solid tumors

##### Jared Weiss, MD^2^, Anthony Olszanski, MD, RPh^3^, Jordan Berlin, MD^4^, Makoto Origuchi^5^, Zhonggai Li^5^, Bella Ertik^5^, Hongliang Cai^5^, Daniel Clancy^5^, Jose Iglesias^6^, Vivek Subbiah, MD^7^, Shadia Jalal, MD^1^

###### ^1^Indiana University School of Medicine, Indianapolis, IN, United States; ^2^UNC School of Medicine, Chapel Hill, NC, United States; ^3^Fox Chase Cancer Center, Phildelphia, PA, United States; ^4^Vanderbilt University Medical Center, Nashville, TN, United States; ^5^Boston Biomedical Inc, Cambridge, MA, United States; ^6^Former employee, Boston Biomedical Inc, Cambridge, MA, United States; ^7^University of Texas, Houston, TX, United States

####### **Correspondence:** Shadia Jalal (sjalal@iu.edu)

**Background**

DSP-0509 is a TLR7 agonist designed to have high water solubility allowing for intravenous (IV) administration and has rapid elimination, partially due to excretion via organic anion transporting peptide transporters. In preclinical models, DSP-0509 suppressed tumor volume and lung metastasis versus vehicle control. Furthermore, DSP-0509 in combination with anti–programmed cell death protein 1 (PD-1) antibody suppressed tumor growth versus vehicle, DSP-0509 alone, or anti–PD-1 antibody alone. This 3-part dose escalation (Part A), dose expansion (Part B), and maintenance dose schedule evaluation (Part C) study will investigate DSP-0509 alone or in combination with PD-1 inhibitor pembrolizumab (Part A only) (NCT03416335). Primary objectives are to evaluate DSP-0509 safety/tolerability, determine the maximum tolerated dose (Part A monotherapy), and identify DSP-0509 monotherapy recommended phase 2 dose (RP2D) and the RP2D of DSP-0509 in combination with pembrolizumab for future studies. Secondary objectives include pharmacokinetics and antitumor activity.

**Methods**

Eligible patients are aged ≥18 years with advanced solid tumors (Part A and C) or melanoma or head and neck squamous cell carcinoma with acquired immune checkpoint inhibitor resistance (grouped high or low CD8+ cell density in tumor tissue; Part B). In Part A, approximately 21–30 patients will be enrolled in each of the monotherapy and combination arms. DSP-0509 will be given as a constant rate IV infusion over 3 minutes at a fixed dose. Five provisional dose levels of DSP-0509 may be tested, with approximately 3–6 patients at each level (3 escalation levels at target doses of 0.3, 1, and 3 mg; 2 de-escalation levels at target doses of 0.6 and 1.8 mg). During induction treatment, patients will receive 5 doses of DSP-0509 every week for 4 weeks on days 1, 8, 15, 22, and 29 followed by every 2-weeks until discontinuation. In the combination arm, pembrolizumab will be administered IV at 200 mg every 3 weeks (Q3W). Dose limiting toxicities will be monitored within the first 6 weeks of dosing. The mono- and combination DSP-0509 RP2Ds will be determined using a Bayesian logistic regression model. In Part B, approximately 20–40 patients will receive DSP-0509 at the RP2D using the same dosing schedule as Part A. In Part C, approximately 3–6 patients will be treated using the RP2D with the same induction treatment schedule as Part A, but followed by Q3W maintenance dosing. This study is currently recruiting patients.

#### P427 A phase 1 study of IMC-001, novel anti-PD-L1 antibody, in patients with advanced solid tumors

##### Bhumsuk Keam, MD, PhD^1^, Tae Min Kim, MD, PhD^1^, Do-Youn Oh, MD, PhD^1^, Chan-Young Ock, MD, PhD^1^, Won Ki Kang, MD, PhD^2^, Yeon Hee Park, MD, PhD^2^, Jeeyun Lee, MD, PhD^2^, Ji Hye Lee, MD^3^, Yun Jeong Song, MD^3^, Young Suk Park, MD, PhD^2^

###### ^1^Seoul National University Hospital, Seoul, Korea, Republic of; ^2^Samsung Medical Center, Sungkyunkwan University School of Medicine, Seoul, Korea, Republic of; ^3^ImmuneOncia Therapeutics Inc., Gyeonggi, South Korea

####### **Correspondence:** Young Suk Park (pys27hmo@skku.edu)

**Background**

IMC-001 is a fully human IgG1 monoclonal antibody that binds to human PD-L1 and mediate the antibody-dependent cell-mediated cytotoxicity. The main objectives of this study were to evaluate the safety, pharmacokinetics, and pharmacodynamics of IMC-001 in patients with advanced solid tumors. Additional objectives were to explore the anti-tumor activity and identify the maximum tolerated dose (MTD) of IMC-001.

**Methods**

This is a phase 1, open-label study of IMC-001 in patients with metastatic or advanced solid tumors. IMC-001 was administered intravenously every two weeks with a standard 3+3 dose-escalation design until disease progression or unacceptable toxicity. Dose limiting toxicity (DLT) window was defined as 21 days from the first dose. Adverse events (AEs) were assessed using CTCAE v4.03, and tumor response was assessed by the Response Evaluation Criteria In Solid Tumors, version v1.1.

**Results**

Fifteen patients (8 Male, 7 Female; Median age: 58 [range 39-69]) were included in 5 dose escalation cohorts, dose ranging from 2 to 20 mg. Of the 15 subjects, 5 colorectal cancers, 3 biliary tract cancers and 2 thymic cancers were included. No DLT was observed and the maximum tolerated dose was not reached. Most common AEs were decreased appetite, pyrexia, and cough. No Grade 4 or 5 treatment emergent AEs were reported during the study and no TEAE or serious AE led to treatment discontinuation or death. There were no infusion-related reactions during this study. Two grade 2 serious AEs suspected to be related to IMC-001 were seen in one subject at 2mg/kg cohort. Over the dose range 2 to 20 mg/kg IMC-001, AUC_0-14d_, AUC_0—∞_, and C_max_ generally appeared to increase in a dose proportional manner for each step of dose escalation. Efficacy evaluation is ongoing and will be presented in the future.

**Conclusions**

IMC-001 demonstrated a favorable safety profile up to 20mg/kg given IV every 2 weeks in patients with advanced solid tumors.

**Trial Registration**

Clinical trial identification : NCT03644056

**Ethics Approval**

This study was approved by Institutional Review Board; approval number SMC 2018-01-007-001 and H-1801-042-913.

#### P428 A phase 1 study evaluating BI 765063, a first in class selective myeloid SIRPa inhibitor, as stand-alone and in combination with BI 754091, a PD-1 inhibitor, in patients with advanced solid tumours

##### Nuria Kotecki, MD^1^, Philippe Cassier^2^, Jean-Pierre Delord, MD^3^, Stéphane Champiat^4^, Christiane Jungels^1^, Armelle Vinceneux^2^, Iphigenie Korakis^3^, Richard Huhn^5^, Nicolas Poirier^6^, Dominique Costantini^6^, Bérangère Vasseur^6^, Aurélien Marabelle^4^

###### ^1^Institut Jules Bordet, Brussels, Belgium; ^2^Centre Léon Bérard, Lyon, France; ^3^IUCT, Oncopole, Toulouse, France; ^4^Gustave Roussy, Villejuif, France; ^5^Boehringer Ingelheim, Ridgefield, CT, United States; ^6^OSE Immunotherapeutics, Nantes, France

####### **Correspondence:** Nuria Kotecki (nuria.kotecki@bordet.be)

**Background**

Signal Regulatory Protein α [SIRPα] is a polymorphic protein, strongly expressed on myeloid suppressive cells. BI 765063 (OSE172), a humanized IgG4 monoclonal antibody (mAb), is a selective antagonist of SIRPα/CD47 interaction, it does not bind to SIRPɣ, known to assist T cell co-stimulation and migration. BI 765063 strongly binds V1 allele, one of the 2 major functional allele of SIRPα expressed in more than 80% of general population and Asian (in 60%).

Anti-tumor effect was shown in various in vivo cancer models using the validated anti-mouse SIRPα mAbs surrogate, as single agent. The effect was more pronounced in combination with T checkpoint inhibitors [1]. BI 765063 mechanism of action includes promotion of tumor-antigen-presentation while preserving T-cell activation and increase tumor phagocytosis.

The trial plans to assess the safety profile and preliminary efficacy of BI 765063, a first in class myeloid check point inhibitor antagonist of SIRPα on myeloid cells.

**Methods**

This study comprises a dose escalation (step 1) to determine the Dose-Limiting Toxicities, Maximum Tolerated Dose (MTD), and Recommended Phase 2 Dose (RP2Ds) of BI 765063 monotherapy and with BI 754091, and dose-confirmation expansion cohorts (step 2).

In Step 1, ascending dose of BI 763063 once every 3 weeks intravenously (iv) using a Bayesian approach with overdose control are tested. When MTD determined, BI 763063 will be tested with BI 754091, a PD-1 mAb inhibitor. In step 2, 2 parallel randomized, non-comparative mono and combination cohorts will further confirm the RP2Ds and assess the safety and preliminary efficacy (RECIST 1.1 and iRECIST).

Patients ≥ 18 years, PS:0-1, with advanced solid tumor who failed or are not eligible to standard therapy will be included. V1/V1 and V1/V2 patients (central testing) are evaluated in separate cohorts in step 1. In step 2, a selected population of V1/V1 patients with advanced-stage cancers (e.g. non-small cell lung cancer, triple negative breast cancer, or gastro-intestinal cancers) will be included.

Pharmacokinetics (PK), SIRPα receptor occupancy (RO) and a comprehensive translational program (in blood and tumour) will assess PK/PD profile and biomarkers of activity.

A total of 116 (56 in step 1 and 60 in step 2) patients will be enrolled.

**Acknowledgements**

This study was funded by OSE Immunotherapeutics (the sponsor of the study) in collaboration with Boehringer Ingelheim.

**Trial Registration**

This study is registered under ClinicalTrials.gov Identifier: NCT03990233

**Reference**

1. Gauttier V, Pengam S, Durand J, Morello A, Conchon S, Vanhove B and Poirier N. Selective SIRPa blockade potentiates dendritic cell antigen cross-presentation and triggers memory T-cell antitumor responses. Cancer Res 2018;78(13 Supplement): abstr 1684. doi:10.1158/1538-7445.AM2018-1684.

**Ethics Approval**

The study protocol and its related documents (including the patient information and informed consent form) received approval from the Ethics Committees, and the Competent Authority prior to study initiation. Each patient gave his/her written informed consent prior to study enrolment.

#### P429 A phase 1/2a dose escalation and expansion study of HPN536, a mesothelin-targeting T cell engager, in patients with advanced cancers expressing mesothelin who have failed standard therapy

##### Erika Hamilton, MD^1^, Richard Austin, PhD^2^, Sue Hirabayashi^2^, Che-Leung Law, PhD^2^, Bryan Lemon, PhD^2^, Holger Wesche, PhD^2^, Debra Richardson, MD^3^

###### ^1^Sarah Cannon Research Institute/Tennessee Oncology, Nashville, TN, United States; ^2^Harpoon Therapeutics, South San Francisco, CA, United States; ^3^Sarah Cannon Research Institute/Stephenson Cancer Center at the University of Oklahoma Health Sciences Center, Oklahoma City, OK, United States

####### **Correspondence:** Erika Hamilton (ehamilton@tnonc.com)

**Background**

HPN536 is a mesothelin-targeting T cell engager derived from the TriTAC platform (Tri-specific T Cell-Activating Construct). Mesothelin (MSLN) is a tumor antigen overexpressed in malignant mesothelioma, ovarian carcinoma, pancreatic carcinoma, lung cancer, and triple negative breast cancer with limited expression in normal tissues. HPN536 is a recombinant polypeptide of ~50kDa containing three humanized antibody-derived binding domains, targeting mesothelin (for tumor binding), albumin (for half-life extension) and CD3 (for T cell engagement). It has been engineered to be a small, globular protein to enable efficient exposure in solid tumor tissue with prolonged half-life and excellent stability under physiological conditions. HPN536 binds monomerically to CD3 and MSLN, minimizing non-specific T-cell activation. These features are designed to widen the therapeutic index compared to earlier generations of T cell engagers by minimizing off target toxicities. HPN536 mediates potent target tumor cell killing in a MSLN-specific manner in vitro and in xenograft models in the presence of T cells. Consistent with its mechanism of action (MOA), tumor cell killing is accompanied by T cell activation, cytokine induction, and T cell expansion.

**Methods**

This is a Phase 1/2a, open-label, multicenter, dose escalation and expansion study to evaluate the safety, tolerability, clinical activity, and pharmacokinetics of HPN536 in adult patients with advanced cancers expressing mesothelin who have failed standard available therapy. This study will be divided into 2 parts: Dose Escalation (Part 1) and Expansion (Part 2). Eligible patients with ovarian cancer will be enrolled in Dose Escalation. Dose expansion will include patients with ovarian cancer, pancreatic carcinoma and mesothelioma. HPN536 is administered once weekly as one-hour IV infusion by single-patient cohorts until either a Grade ≥2 adverse event (AE) that is possibly related to HPN536 is observed or an estimated therapeutic dose level has been reached. Then a conventional 3+3 design will be implemented. Dose escalation will continue until a recommended phase 2 dose (RP2D) is determined. In dose expansion, up to 20 patients per group receive HPN536 at the established RP2D based on a Simon 2-stage design. Patients may continue weekly HPN536 treatment cycles until disease progression. Primary endpoints are number and severity of DLTs following treatment with escalating doses of HPN536 during escalation, and overall response rate (by RECIST v1.1 for ovarian and pancreatic, mRECIST v1.1 for mesothelioma) in dose expansion. Secondary endpoints include AEs, preliminary anti-tumor activity, pharmacokinetic and pharmacodynamic parameters based on the proposed MOA of HPN536.

**Trial Registration**

NCT03872206

**Ethics Approval**

This study was approved by each participating institution's Institutional Review Board.

#### P430 Open-label, multicenter phase 1/2 dose escalation and expansion study of THOR-707 as a single agent and in combination with a PD-1 inhibitor in adult subjects with advanced or metastatic solid tumors

##### David Luo^1^, Raghad Abdul-Karim, MD^2^, Arun Azad, MD^3^, Joanna Bendell, MD^4^, Hui Gan, MBBS PhD^5^, Filip Janku, MD, PhD^6^, Shiraj Sen, MD, PhD^7^, Tira Tan, MBBS^8^, Judy Wang, MD^9^, Lisa Schechet^1^, Lauren Baker, PhD^1^ , Joseph Leveque, MD^1^, Tarek Meniawy, MBBS FRACP^10^

###### ^1^Synthorx Inc, La Jolla, CA, United States; ^2^NEXT Oncology, Texas Oncology, San Antonio, TX, United States; ^3^Peter MacCallum Cancer Centre, Melbourne, Austrailia; ^4^Sarah Cannon Research Institute, Nashville, TN, United States; ^5^Austin Hospital, Melbourne, Victoria, Australia; ^6^University of TX, MD Anderson Cancer Center, Houston, TX, United States; ^7^Sarah Cannon Research Institute at HealthONE, Houston, TX, United States; ^8^National Cancer Centre Singapore, Toronto, Canada; ^9^Florida Cancer Specialists, Sarasota, FL, United States; ^10^Linear Clinical Research, Nedlands, WA, Australia

####### **Correspondence:** Lauren Baker (lbaker@synthorx.com)

**Background**

Recombinant interleukin-2 (aldesleukin), an approved immunotherapy in metastatic melanoma and renal cell carcinoma, can induce complete durable responses in some patients. The anti-neoplastic properties of IL-2 are mediated by activation of effector memory T cells and newly recruited naïve CD8+ T cells against the tumor. The widespread use of IL-2 has been limited due to its high affinity bias for the IL-2 receptor alpha chain (IL-2R⍺) on regulatory CD4+ T cells, leading to immunosuppression and, eosinophilic recruitment and activation on innate lymphoid cells in the vascular endothelium causing vascular leak syndrome (VLS). THOR-707 is a recombinant human IL-2 variant that is site-specifically pegylated, providing a “not alpha” pharmacologic profile designed to prevent engagement of IL-2R⍺, thereby providing an improved safety profile while still promoting newly recruited and effector memory T cell anti-tumor activity In preclinical studies, eosinophilia was not observed at a doses 10-fold higher than the dose responsible for eliciting maximal expansion of peripheral CD8+ T cells. Based on these findings, a first-in-human study of THOR-707 was started in June 2019.

**Methods**

This open-label, multicenter, dose escalation and expansion study in adult subjects with advanced or metastatic solid tumors will evaluate THOR-707 as a single agent and in combination with a PD-1 inhibitor. Study objectives are to define the maximum tolerated dose (MTD) and/or recommended phase 2 dose (RP2D) of THOR-707 as single agent and in combination with a PD-1 inhibitor; and to evaluate the overall safety and tolerability as well as, pharmacokinetics, pharmacodynamics, and preliminary anti-tumor activity. The study will be conducted in 3 parts.

- Part 1 will evaluate THOR-707 as a single agent across different dosing schedules (e.g., dosing every 2 weeks [Q2W] or 3 weeks [Q3W]).
- Part 2 will evaluate THOR-707 (Q3W) in combination with a PD-1 inhibitor.
- Part 3: Dose expansion will begin after the RP2D for THOR-707 as a single agent or in combination with a checkpoint inhibitor has been determined and will enroll selected populations (e.g., specific tumor types, treatment history, and/or biomarker profile).

Between 50-100 subjects may be enrolled in the dose escalation phase to determine the MTD and/or RP2D as a single agent and in combination with a PD-1 inhibitor. After defining the MTD and/or RP2D, additional subjects may be enrolled at the respective dose schedules to further evaluate safety, pharmacodynamic effects, and anti-tumor activity

**Trial Registration**

NCT04009681

**Table 1 (abstract P430).**
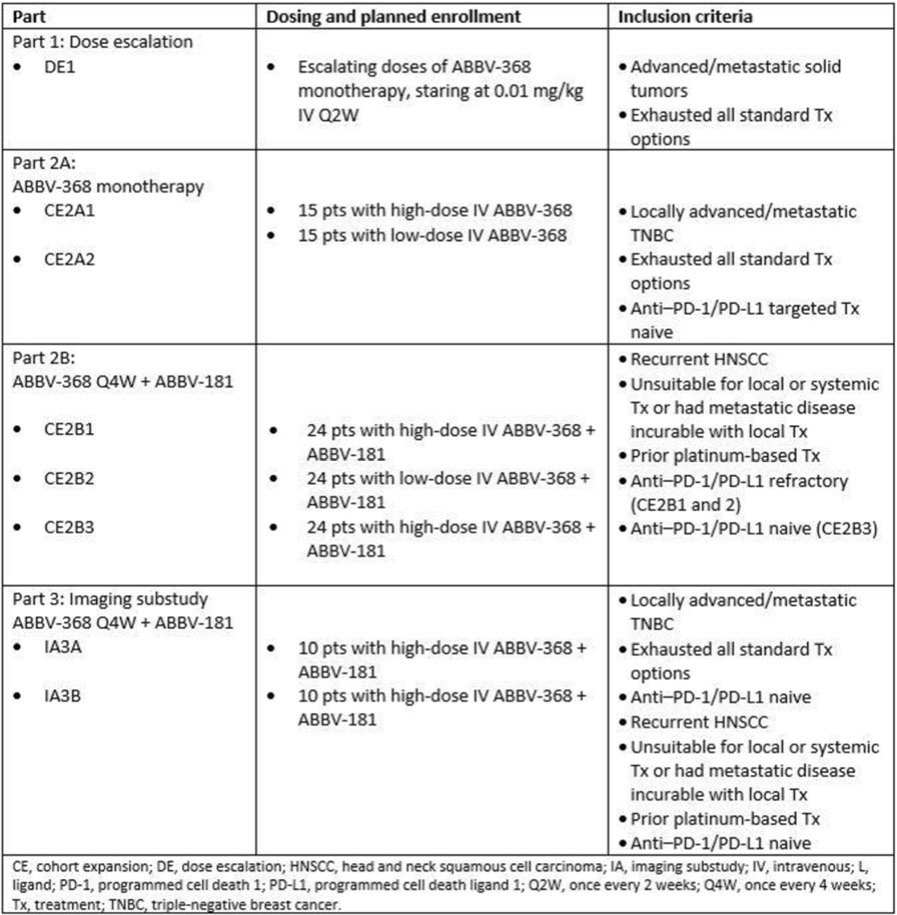
See text for description

#### P431 Semi-mechanistic PK and target-occupancy modeling to support dose justification for anti-PD-L1 clinical candidate CK-301 (TG-1501) in oncology patients

##### Lin Lin, PhD^1^, James Hilbert^1^, Leonid Gorelik^2^, Jian-Ping Tang^3^, James Oliviero^4^, Joshua Apgar^1^, Lore Gruenbaum, PhD^1^, John Burke^1^

###### ^1^Applied BioMath, LLC, Concord, MA, United States; ^2^Fortress Biotech, Waltham, MA, United States; ^3^TG Therapeutics, New York, NY, United States; ^4^Checkpoint Therapeutics, New York, NY, United States

####### **Correspondence:** Lore Gruenbaum (lgruenbaum@gothamtx.com)

**Background**

Mathematical modeling was used in conjunction with in vitro, preclinical and clinical data to facilitate dose selection of CK-301 (also known as TG-1501), an anti-PD-L1 monoclonal antibody (mAb), for ongoing and future clinical trials in oncology patients.

**Methods**

A semi-mechanistic pharmacokinetic/target-occupancy (PKTO) model was developed to predict pharmacokinetics (PK) of CK-301 at steady state and its tumor target occupancy (TO) under various dosing regimens. The model captures the interactions between CK-301, PD-L1, soluble PD-L1 and PD-1 in 3 compartments: tumor, circulation (central) and other tissues (peripheral). The model was calibrated with CK-301 PK data from the first 5 patients in a clinical study, CK-301-101, and PK data from published Phase 1 studies of 3 marketed anti-PD-L1 mAbs: atezolizumab, avelumab and durvalumab. Additionally, the model incorporated experimentally determined binding affinities for the 3 marketed anti-PD-L1 mAbs and CK-301.

**Results**

Using the PKTO model, plasma Ctrough values and tumor TO of CK-301 at steady-state with 800 and 1200 mg q2w or q3w were projected. The TO of CK-301 were compared with predicted steady-state Ctrough TOs of atezolizumab, avelumab and durvalumab at their marketed doses. The steady-state Ctrough values of CK-301 are predicted to give >99% tumor TO for patients with a nominal or a 10-fold greater than nominal PD-L1 tumor burden. This is similar to predicted TO for atezolizumab and durvalumab. The PKTO model was used to simulate PK and TO of CK-301 in 1000 virtual patients. The simulations predicted that, at 800 and 1200 mg q2w or q3w, ≥93.0% of patients with a nominal PD-L1 tumor burden or ≥80.1% of patients with 10-fold higher than nominal PD-L1 tumor burden would have a >99% tumor TO at steady-state Ctrough.

**Conclusions**

At the proposed CK-301 dosing regimens of 800 and 1200 mg q2w or q3w, a >99% TO is expected throughout the dosing interval. Relative to atezolizumab and durvalumab treatments, similar percentages of patients would possibly benefit from CK-301 treatment.

#### P432 A phase 1 dose-escalation study of safety, tolerability, and pharmacokinetics (PK) of ABBV-368 monotherapy and combination in patients (pts) with locally advanced or metastatic solid tumors

##### Christophe Le Tourneau^1^, Wu-Chou Su, MD^2^, Ki Chung, MD^3^, Patricia LoRusso, DO^4^, Chia-Chi Lin, MD, PhD^5^, Fabrice Barlesi, MD, PhD^6^, Her-Shyong Shiah^7^, Eric Angevin, MD^8^, Alexander Spira, MD, PhD, FACP^9^, Amita Patnaik, MD FRCP(C)^10^, John Powderly, MD, CPI^11^, Dimitrios Colevas^12^, Helen Chew, MD^13^, Maulik Patel, PharmD, PhD^14^, Stacie Lambert^14^, Yan Li^14^, Daniel Da Costa^14^, Martha Blaney, PharmD^14^, Michael McDevitt, MD, PhD^14^, Philippe Cassier^15^

###### ^1^Institut Curie, Paris & Saint-cloud, France; ^2^National Cheng Kung University Hospital, Tainan, Taiwan, Province of China; ^3^GHS Cancer Institute, Spartanburg, SC, United States; ^4^Yale Cancer Center, New Haven, CT, United States; ^5^National Taiwan University Hospital, Taipei, Taiwan, Province of China; ^6^Aix Marseille Univ Hôpitaux de Marseille, Livon, France; ^7^Taipei Medical University, Taipei, Taiwan, Province of China; ^8^Gustave Roussy, Villejuif, France; ^9^Virginia Cancer Specialists Research Ins, Fairfax, VA, United States; ^10^South Texas Accelerated Research Thera, San Antonio, TX, United States; ^11^Carolina BioOncology Institute, Huntersville, NC, United States; ^12^Stanford University, Stanford, United States; ^13^UC Davis, Sacramento, United States; ^14^Abbvie Inc, North Chicago, IL, United States; ^15^Léon Bérard Cancer Center, Lyon, France

####### **Correspondence:** Michael McDevitt (michael.mcdevitt@abbvie.com)

**Background**

ABBV-368 is a humanized anti-OX40 monoclonal antibody. OX40 is a member of the TNF receptor superfamily, which exerts its action via activating T effector cells and inhibiting the suppressive capacity of T reg cells. Preclinical data have shown ABBV-368 antitumor activity in animals.

**Methods**

This is a multicenter, phase 1, dose-escalation study (NCT03071757) in pts (≥18 years; Eastern Cooperative Oncology Group performance status 0–2) with locally advanced or metastatic solid tumors. The study consisted of 3 parts: 1) dose escalation (DE1); 2) cohort expansion (CE2); and 3) imaging substudy (IA3). Specific inclusion criteria and dosing schedules for each cohort are shown in the table (Table 1). In DE1, the primary endpoints are safety, tolerability, and PK of ABBV-368 monotherapy to establish the maximum tolerated dose or reach the maximally administered dose; the secondary endpoint is preliminary antitumor activity. Preliminary results for DE1 have been reported (Powderly et al. ESMO 2018). For CE2, the primary endpoints are safety, tolerability, and PK of ABBV-368 monotherapy and in combination with ABBV-181 (a humanized anti-programmed cell death 1 monoclonal antibody), and to establish the recommended phase 2 dose; the secondary endpoint is preliminary antitumor activity of ABBV-368 monotherapy and combination therapy. The primary endpoints of the IA3 part are safety and tolerability. For all cohorts, AEs will be assessed according to the NCI CTCAE v4.03; response will be assessed Q2 months (mo) for 12 mo, and Q3 mo thereafter, as per the immunotherapy Response Evaluation Criteria in Solid Tumors (iRECIST), and RECIST v1.1. As of 12 Jul 2018, enrollment into DE1 was completed and CE2 has started.

**Trial Registration**

NCT03071757

#### P433 Initial results of the phase 1 portion of an ongoing phase 1/2 study of RP1 as a single agent and in combination with nivolumab in patients with solid tumors

##### M Middleton, MD PhD^1^, Joseph Sacco^2^, Jaime Merchan^3^, Amber Thomassen^3^, Brendan Curti, MD^4^, Ari VanderWalde, MD, MPH, MBioeth^5^, Anna Olsson-Brown, MBChB (Hons), BSc (Hons)^2^, Francesca Aroldi^1^, Nicos Fotiadis^6^, Scott Baum^5^, Howard Kaufman, MD, FACS^7^, Kevin Harrington, MD^6^

###### ^1^University of Oxford, Childrey, United Kingdom; ^2^University of Liverpool, Liverpool, United Kingdom; ^3^University of Miami, Miami, United States; ^4^Providence Medical Center, Portland, OR, United States; ^5^West Cancer Center, Germantown, TN, United States; ^6^The Institute of Cancer Research, London, United Kingdom; ^7^Replimune, Woburn, MA, United States

####### **Correspondence:** Howard Kaufman (Howard.Kaufman@replimune.com)

**Background**

Background: RP1 is an enhanced-potency oncolytic HSV-1 expressing a fusogenic glycoprotein (GALV-GP R-) and GM-CSF which is being tested in a Phase 1/2 clinical trial in ~150 patients with a range of solid tumors (NCT03767348).

**Methods**

Methods: The objectives were to define the safety of RP1 alone and with nivolumab, determine the recommended phase 2 dose (RP2D), and in 30 patient phase 2 cohorts, assess efficacy in melanoma, non-melanoma skin cancer, urothelial carcinoma and MSI-H tumors. Initial phase 1 results will be reported where patients were treated by intra-patient dose escalation of RP1 (up to 10mL of 104-108PFU/mL) by intratumoral injection into a single tumor Q2W up to 5 times followed by 12 patients dosed 8 times at the RP2D combined with nivolumab (240mg Q2W for 4 months from the second RP1 dose, then 480 mg Q4W for 20 months). Clinically accessible lesions were directly injected, with imaging guidance for deep/visceral lesions. Pre- and on-treatment tumor biopsies were obtained for biomarker analysis. Viral shedding and anti-HSV antibody titers were also monitored.

**Results**

Results: 22 heavily pretreated patients with advanced tumors were enrolled into the dose-rising phase with largely low-grade adverse events, including febrile and other constitutional symptoms, local inflammation and erythema. No clear differences were seen between superficial and visceral dosing. RP1 was detected at the injection site and in blood for up to 14 days (next injection), suggesting virus replication. All HSV seronegative patients seroconverted after three injections. Biological activity was demonstrated including tumor necrosis and shrinkage, with extended clinical benefit and delayed (post-treatment termination and initial PD) systemic reduction in multiple tumors in two patients (ipilimumab/nivolumab-refractory melanoma and chemotherapy-refractory cholangiocarcinoma) without intervening treatment. The RP2D was selected as up to 10mL of 106PFU/mL followed Q2W by multiple doses of 107PFU/mL. Twelve evaluable patients (6 direct injection, 6 image-guided) were then enrolled into the phase 1 expansion combined with nivolumab. This demonstrated tolerability and clinical activity, including complete and partial responses in patients with chemotherapy-refractory cutaneous squamous carcinoma, and ipilimumab/nivolumab-refractory melanoma. Treatment remains ongoing, and current data will be presented, including biomarker data (CD8, PD-L1 staining and Nanostring analysis from tumor biopsies).

**Conclusions**

Conclusions: The Phase 1 clinical data supports the safety and efficacy of RP1 alone and when combined with nivolumab, including demonstration of abscopal anti-tumor effects in patients refractory to prior checkpoint inhibitors. The Phase 2 portion of this clinical trial is open in the US and the UK.

**Trial Registration**

NCT03767348

**Ethics Approval**

The study was approved by applicable institutional review or ethics boards.

#### P434 A phase 1/1b study to evaluate the humanized anti-CD73 antibody, CPI-006, as a single agent, in combination with CPI-444, and in combination with pembrolizumab in adult patients with advanced cancers

##### Mehrdad Mobasher^1^, Richard Miller^1^, Brian Munneke^1^, Deborah Strahs^1^, Gabriel Luciano^1^, Emily Piccione, PhD^1^, Suresh Mahabhashyam^1^, Jaime Merchan^2^, John Powderly, MD, CPI^3^, Lauren Harshman, MD^4^, Minal Barve^5^, Walter Stadler, MD^6^, Patricia LoRusso, DO^7^, Melissa Johnson^8^, Abhishek Tripathi, MD^9^, Sumanta Pal, MD^10^, Ben Markman, MBBS FRACP^11^, Jason Luke, MD, FACP^12^, Thomas Marron, MD PhD^13^

###### ^1^Corvus Pharmaceuticals Inc, Burlingame, CA, United States; ^2^University of Miami, Miami, United States; ^3^Carolina BioOncology Institute, Huntersville, NC, United States; ^4^Dana-Farber Cancer Institute, Boston, MA, United States; ^5^Mary Crowley Cancer Research Center, Dallas, TX, United States; ^6^University of Chicago Comprensive Cancer Center, Chicago, IL, United States; ^7^Yale University School of Medicine, New Haven, CT, United States; ^8^Sarah Cannon Research Institute, Nashville, TN, United States; ^9^University of Oklahoma, Stephenson Cancer Center, Oklahoma City, OK, United States; ^10^City of Hope, Duarte, CA, United States; ^11^Monash Health, Melbourne, Australia; ^12^UPMC, Pittsburgh, PA, United States; ^13^Icahn School of Medicine at Mount Sinai, New York, NY, United States

####### **Correspondence:** Mehrdad Mobasher (mmobasher@corvuspharma.com)

**Background**

CD73 expression is elevated in tumors and contributes to increasing levels of immunosuppressive adenosine in the tumor microenvironment. CD73 knockout mice exhibit reduced tumor growth and resistance to experimental metastasis. Inhibition of CD73 activity with an anti-CD73 antibody blocks adenosine production, shown to inhibit tumor growth in syngeneic models. Dual inhibition of CD73 and A2aR improves anti-tumor immune responses in mouse tumor models[1]. CPI-006 is a humanized IgG1 Fc gamma receptor binding-deficient anti-CD73 antibody that has a dual mechanism of action. It blocks CD73 catalytic activity and adenosine production. In addition, it has immunomodulatory activity on CD73 positive immune cells including B cells, T cells and antigen presenting cells. CPI-006 relieves adenosine-mediated immunosuppression in vitro as a single agent and in combination with ciforadenant[2]. CPI-006 is now being investigated in this Phase 1/1b multicenter, open label trial as single agent (SA), in combination with ciforadenant, an oral, small molecule, selective A2aR antagonist and in combination with pembrolizumab, an anti-PD1 indicated for the treatment of patients across a number of malignancies (NCT03454451).

**Methods**

Up to 462 subjects will be enrolled at approximately 35 sites in the US, Canada and Australia. Eligible patients with: non-small cell lung cancer (NSCLC), renal cell carcinoma cancer (RCC), urothelial bladder cancer, cervical cancer, colorectal cancer, ovarian cancer, pancreatic cancer, prostate cancer, head and neck cancer, triple-negative breast cancer, endometrial cancer, select sarcomas and non-Hodgkin lymphoma (NHL) who are relapsed, refractory or intolerant to 1 to 5 standard therapies; aged ≥ 18 yo; with adequate organ function and measurable disease. Study details is presented in Figure 1.

The primary objective of the dose escalation is to assess safety/ tolerability, MTD or MDL of CPI-006 SA, in combination with ciforadenant and with pembrolizumab in ascending dose levels. Secondary objectives are to evaluate the PK of CPI-006 as SA or in combinations and analyze potential predictive biomarkers. In dose escalation, the primary objective is to assess the safety and tolerability of CPI-006 SA and in combinations in patients with selected advanced cancers. Secondary endpoints include efficacy; PK of CPI-006 as SA and in combinations and to evaluate the relationship between biomarkers and clinical activity.

**References**

1. Young et al. Co-inhibition of CD73 and A2AR adenosine signaling improves anti-tumor immune responses. Cancer Cell. 2016; 30(3):391-403.

2. Piccione et al. Preclinical and initial phase I clinical characterization of CPI-006: an anti-CD73 monoclonal antibody with unique immunostimulatory activity. Presented at Society for Immunotherapy of Cancer Meeting; November 7-11, 2018; Washington, DC, USA: Abstract P205.

**Ethics Approval**

The study was approved by Western IRB, approval number 1-1066703-1.

**Fig. 1 (abstract P434).**
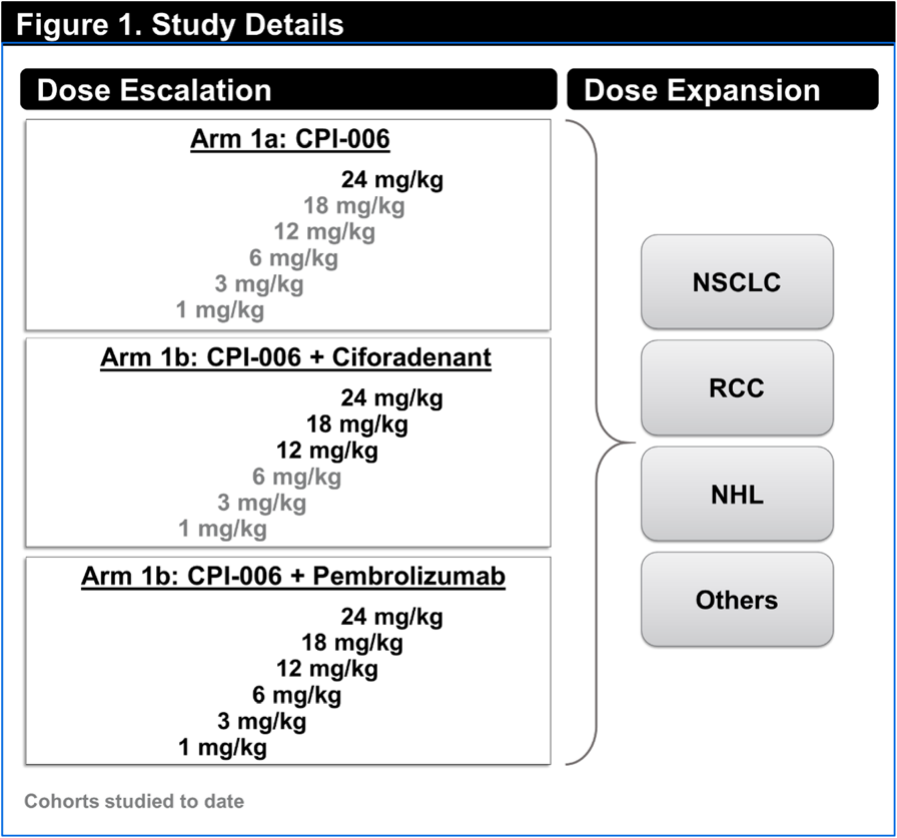
See text for description

#### P435 A window of opportunity trial using intratumoral injection of glatiramer as an immune modulator in patients with resectable head and neck and cutaneous squamous cell cancer

##### Ghulam Rehman Mohyuddin, MD, Joaquina Baranda, MD, Andres Bur, Lisa Shnayder, Kiran Kakarala, Terry Tsue, Prakash Neupane, Gregory Gan, Joshua Mammen, Daniel Aires, Sufi Thomas, Stephen Williamson, Nelli Lakis, Rashna Madan, Prabhakar Chalise, Scott Weir, Andrew Godwin, Greg Reed, Cory Berkland

###### Kansas University Medical Center, Kansas City, KS, United States

####### **Correspondence:** Joaquina Baranda (gioncol@gmail.com)

**Background**

Immunotherapy using checkpoint inhibition improves outcome of patients with melanoma, lung, bladder, microsatellite instability-high and other tumors[1]. However, systemic administration of immunotherapy may have limited activity in some tumors partly due to failure of activated T cells to migrate to tumor[1]. Intratumoral injections (ITI) may allow high concentration of immunostimulatory products locally while using small amounts of drugs. This may also facilitate multiple combination therapies and avoid systemic off-target toxicities. By capitalizing on existing data and experience, repurposing approved drugs for cancer represents an opportunity to rapidly advance promising therapies.

Glatiramer acetate is an agent commonly used for multiple sclerosis[2]. It acts as an immunomodulator and has essentially no systemic bioavailability but exhibits a high prevalence of injection site reactions[2]. It upregulates the activity of natural killer cells in leukemia cell lines [3], suggesting potential for immunostimulatory effect for ITI. For percutaneously accessible tumors for which the standard of care is surgical resection without any neoadjuvant therapy, there exists a window of opportunity where ITI of glatiramer can be performed before surgery.

**Methods**

This is a proof-of-concept, investigator-initiated, window of opportunity trial in subjects with percutaneously accessible head and neck or cutaneous squamous cell cancer who are to undergo surgery. Subjects will receive glatiramer 40 mg by ITI 3 times a week prior to surgery. Subjects will receive at least one dose and up to 3 doses of glatiramer. About 10 eligible patients will be included in this trial. Safety data will be collected. Tumor tissue at the time of diagnosis and at the time of surgery will be collected and compared for biomarkers. Primary endpoint is safety. Secondary endpoint is effect of ITI of glatiramer on biomarker levels. Pre- and post-treatment tumor samples will be tested using an immunology panel that profiles immunology genes and proteins including major classes of cytokines, interferons, KIR family, and TNF-receptor. Tumors samples will also be evaluated for the Ki-67 proliferative index and for caspase-3 and cleaved caspase-3 immunoexpression. Paired T-test or the Wilcoxon signed rank test will be used to assess the changes in the variables. We hypothesize that this approach will break the immunosuppressive tumor microenvironment as evidenced by an increase in inflammatory cytokines, chemokines, and immune cell infiltration. Decline in Ki-67 and increase in caspase-3 may be a signal of anti-tumor activity.

**Trial Registration**

NCT03982212

**References**

1. Whiteside, T.L., et al., Emerging Opportunities and Challenges in Cancer Immunotherapy. Clin Cancer Res, 2016. 22(8): p. 1845-55.

2. Wingerchuk, D.M. and J.L. Carter, Multiple sclerosis: current and emerging disease-modifying therapies and treatment strategies. Mayo Clin Proc, 2014. 89(2): p. 225-40.

3. Maghazachi, A.A., K.L. Sand, and Z. Al-Jaderi, Glatiramer Acetate, Dimethyl Fumarate, and Monomethyl Fumarate Upregulate the Expression of CCR10 on the Surface of Natural Killer Cells and Enhance Their Chemotaxis and Cytotoxicity. Front Immunol, 2016. 7: p. 437.

**Ethics Approval**

This study was approved by Kansas University Medical Center Institutional Review Board, approval number: HSC00144030

#### P436 Phase 2 study of lenvatinib plus pembrolizumab in previously treated patients with solid tumors: LEAP-005

##### Ravit Geva^1^, Seock-Ah Im^2^, Zarnie Lwin^3^, Susan Weil^4^, Lei Xu^5^, Anne Morosky^5^, Kevin Norwood, MD^5^, Hyun Cheol Chung, MD, PhD^6^

###### ^1^Tel-Aviv Sourasky Medical Center, Tel-Aviv, Israel; ^2^Seoul National University Hospital, Seoul, Korea, Republic of; ^3^University of Queensland, Queensland, Australia; ^4^Eisai Inc., Woodcliff Lake, NJ, United States; ^5^Merck & Co., Inc., Kenilworth, NJ, United States; ^6^Yonsei University College of Medicine, Seoul, Korea, Republic of

####### **Correspondence:** Ravit Geva (ravitg@tlvmc.gov.il)

**Background**

Lenvatinib (multiple receptor tyrosine kinase inhibitor of vascular endothelial growth factor receptors 1–3, fibroblast growth factor receptors 1–4, platelet-derived growth factor receptor α, RET, and KIT) and anti–PD-1 inhibitor pembrolizumab have shown clinical benefit as monotherapies across multiple cancers. In preclinical studies, lenvatinib plus PD-1 blockade improved antitumor activity vs either agent alone. LEAP-005 (NCT03797326) evaluates the efficacy and safety of lenvatinib plus pembrolizumab in patients with previously treated selected advanced tumors.

**Methods**

This global, open-label, phase 2 study enrolls patients ≥18 years with the following previously treated histologically/cytologically confirmed advanced tumors: triple negative breast, ovarian, gastric, colorectal (non-MSI-H/pMMR), glioblastoma multiforme (GBM), or biliary tract (excluding ampulla of Vater). Patients must have progressed on or since last treatment; have measurable disease per RECIST v1.1 (modified to follow ≤5 target lesions/organ [10 total]) or the RANO criteria (GBM only), assessed locally and confirmed by blinded independent central review (BICR); ECOG performance score 0–1, adequately controlled blood pressure, and must have provided a tumor sample evaluable for PD-L1; and must not have immunodeficiency, active central nervous system metastases (except in GBM cohort), or be on steroid therapy (patients with GBM may be on dexamethasone ≤2 mg/day orally or equivalent and stable for 5 days at the time of enrollment). Patients will receive lenvatinib 20 mg daily and pembrolizumab 200 mg every 3 weeks (Q3W) for ≤2 years, or until confirmed disease progression (may continue lenvatinib if receiving clinical benefit), unacceptable toxicity, or study withdrawal. Patients with confirmed complete response may discontinue after ≥24 weeks combination therapy and ≥2 pembrolizumab doses after initial complete response date. Tumor imaging will occur at baseline, Q9W (or for GBM patients: Q6W until 18 weeks, then Q9W) for the first 54 weeks, Q12W until week 102 (~2 years), and Q24W thereafter using RECIST v1.1/RANO by investigator assessment in the initial cohorts and by BICR after cohort expansion. Primary endpoints are objective response and safety (adverse events graded using NCI CTCAE v4.0, and discontinuation due to adverse events). Secondary endpoints include disease control, duration of response, progression-free survival, and overall survival. Initially, approximately 180 patients will be enrolled (30/cohort; each cohort may be expanded to 100 after planned interim analysis). Enrollment is ongoing at 44 sites in 10 countries across North America, South America, Europe, Asia, and Australia.

**Acknowledgements**

Writing support was provided by Shilpa Aggarwal, PhD, of C4 MedSolutions, LLC (Yardley, PA, USA), a CHC Group company, funded by Eisai Inc. and Merck Sharp & Dohme Corp., a subsidiary of Merck & Co., Inc., Kenilworth, NJ, USA.

Legal Entity Responsible for the Study: Eisai Inc. and Merck Sharp & Dohme Corp., a subsidiary of Merck & Co., Inc., Kenilworth, NJ, USA

Funding Source

Funding for this research was provided by Eisai Inc. and Merck Sharp & Dohme Corp., a subsidiary of Merck & Co., Inc., Kenilworth, NJ, USA.

**Trial Registration**

NCT03797326

**Ethics Approval**

An independent institutional review board or ethics committee approved the protocol at each study site, and the trial is being conducted in compliance with Good Clinical Practice guidelines and the Declaration of Helsinki.

#### P437 Disease-related biomarkers are associated with extended progression free survival after treatment with NEO-PV-01 in combination with anti-PD1 in patients with metastatic cancers

##### Patrick Ott, MD, PhD^1^, Ramaswamy Govindan, MD^2^, Aung Naing, MD, FACP^3^, Terence Friedlander, MD^4^, Kim Margolin, MD^5^, Jessica Lin, MD^6^, Nina Bhardwaj, MD, PhD^7^, Matthew Hellmann, MD^8^, Mark Awad, MD PhD^1^, Amy Wanamaker^9^, Lisa Cleary^9^, Michael Rooney^9^, Julian Scherer, PhD^9^, Meghan Bushway^9^, Melissa Moles^9^, Zakaria Khondker^9^, Richard Gaynor, MD^9^, Lakshmi Srinivasan, PhD^9^, Andrew Chi^9^, Joel Greshock^9^, Siwen Hu-Lieskovan, MD, PhD^10^

###### ^1^Dana Farber Cancer Institute, Boston, MA, United States; ^2^Washington University, Saint Louis, MO, United States; ^3^MD Anderson Cancer Center, Houston, TX, United States; ^4^University of California San Francisco, San Francisco, CA, United States; ^5^City Of Hope, Duarte, CA, United States; ^6^Massachusetts General Hospital, Boston, MA, United States; ^7^Mt. Sinai Medical Center, New York, NY, United States; ^8^Memorial Sloan Kettering Cancer Center, New York, NY, United States; ^9^Neon Therapeutics, Cambridge, MA, United States; ^10^Huntsman Cancer Institute, Los Angeles, CA, United States

####### **Correspondence:** Joel Greshock (jgreshock@neontherapeutics.com)

**Background**

Neoantigens arise from mutations in cancer cell DNA and are important targets for T cell mediated anti-tumor immunity. NEO-PV-01 is a personal neoantigen vaccine of up to 20 peptides designed by the RECON® bioinformatics platform using patient neoantigen and HLA profiles. Here we report biomarker correlates of clinical benefit for NT-001, a Phase 1b study of NEO-PV-01 + adjuvant in combination with nivolumab in anti-PD1 naïve metastatic melanoma, NSCLC and bladder cancer patients (NCT02897765).

**Methods**

Patients received 12 weeks of nivolumab monotherapy (240 mgs Q2W), then NEO-PV-01 in a prime-boost format spanning 12 weeks, nivolumab continued for up to 2 years. The primary objective was safety, secondary objectives were overall response rate (ORR), progression-free survival (PFS), and overall survival. Comprehensive comparisons of baseline and serial molecular and immunological characteristics between patients with vs. without durable PFS were performed for all tumor cohorts.

**Results**

A total of 34 melanoma, 27 NSCLC and 21 bladder cancer patients received nivolumab therapy, of which 27, 18 and 15 initiated vaccine respectively. The median follow up time was 13.4, 12.0 and 14.7 months for melanoma, NSCLC and bladder cancer respectively. No treatment-related serious adverse events were noted. The median PFS for the melanoma cohort was not reached (95% CI: 3.3, NE), and the ORR was 47%. The median PFS in both the NSCLC and bladder cohort was 5.6 months (95% CI’s: 2.3, 8.7; 2.0, 8.1 respectively) with ORR’s of 22% and 24% respectively. RECON tumor neoantigen abundance was predictive of durable PFS in melanoma patients. Analyses of pre-treatment peripheral TCR repertoires reveal a more clonal T cell population in melanoma patients with extended PFS. Other factors that associated with durable PFS included the abundance of B cells and CD8+ T cells in the tumor microenvironment. Finally, across cohorts, longitudinal tumor biopsies from patients with extended PFS showed higher rates of initial pathologic responses after vaccination vs. biopsies from patients with shorter PFS, suggesting vaccine-related anti-tumor responses in this subset.

**Conclusions**

NEO-PV-01 in combination with nivolumab is safe and leads to post-vaccine immune and pathologic responses, indicating further clinical evaluation is warranted. The association of baseline disease characteristics with prolonged PFS suggests future patient enrichment strategies.

**Trial Registration**

NCT02897765

**Ethics Approval**

This trial has been approved by all institutional Review Boards of every clinical trial site involved with this study.

#### P438 Phase 2 Multicenter Trial of ICOS agonist vopratelimab and a CTLA-4 inhibitor in PD-1/PD-L1 inhibitor experienced adult subjects with Non-small Cell Lung Cancer or Urothelial Cancer (EMERGE)

##### Russell Pachynski, MD^1^, Ramaswamy Govindan, MD^1^, Ellen Hooper, MD^2^, Christopher Harvey, PhD^2^, Amanda Hanson^2^, Sean Lacey, MA^2^, Rachel McComb^2^, Courtney Hart^2^, Haley Laken^2^, Johan Baeck^2^, Elizabeth Trehu, MD^2^

###### ^1^Washington University School of Medicine, St. Louis, MO, United States; ^2^Jounce Therapeutics, Cambridge, MA, United States

####### **Correspondence:** Russell Pachynski (rkpachynski@wustl.edu)

**Background**

ICOS is a costimulatory molecule upregulated on activated T cells. Vopratelimab (JTX-2011) is an IgG1 ICOS agonist monoclonal antibody known to activate and proliferate primed CD4 T effector cells in vitro, with established preclinical efficacy in multiple tumor models. In the Phase 1/2 ICONIC trial (NCT02904226), vopratelimab in patients with advanced solid tumors (Yap 2019) has shown to be safe and well tolerated as monotherapy and in combination with nivolumab. The ICONIC study showed no correlation between tumor reductions and ICOS and PD-L1 levels in pre-treatment tumor samples by IHC. However, emergence of peripheral blood ICOS High (hi) CD4 T effector cells following treatment with vopratelimab +/- nivolumab was associated with tumor reductions and improved PFS and OS. In addition, ex vivo antigen recall studies (Hanson 2018) showed that soluble vopratelimab stimulated a polyfunctional cytokine response only in CD4 T cells that were ICOS hi, further supporting the hypothesis that vopratelimab induces activation and proliferation of CD4 T effector cells only after an initial priming event induces an ICOS hi CD4 T cell phenotype. Furthermore, in melanoma patients treated with ipilimumab, a sustained increase in the frequency of ICOS-positive CD4 T cells correlated with clinical benefit and survival (Carthon 2010). In contrast, emergence of these ICOS hi cells has not been noted with PD-1/PD-L1 inhibitors (Hanson 2018). We hypothesized that the combination of vopratelimab with ipilimumab will enhance the presence and functionality of ICOS hi CD4 T effector cells, thereby potentially increasing the likelihood of clinical benefit.

**Methods**

This open label, multi-center, phase 2 study will evaluate efficacy, safety, PK, and exploratory pharmacodynamics of vopratelimab in combination with ipilimumab in adult patients with non-small cell lung cancer or urothelial cancer who have been previously treated with PD-1/PD-L1 inhibitors. We expect to enroll approximately 200 evaluable subjects in total. Primary endpoint is ORR. Secondary endpoints include safety and tolerability, PFS, OS as well as PK/PD.

**Trial Registration**

ClinicalTrials.gov NCT03989362

**Ethics Approval**

Study was approved by the applicable Institution Ethics Boards

#### P439 Phase 1 first in human study of programmed cell death receptor-1(PD-1) inhibitor monoclonal antibody (mAb) JTX-4014 in adult subjects with advanced refractory solid rumor malignancies

##### Kyriakos Papadopoulos, MD^1^, Gerald Falchook, MD^2^, Nehal Lakhani, MD, PhD^3^, Gosia Riley^4^, Jian Xu, PhD^4^, Johan Baeck^4^, Gilad Gordon^4^, Elizabeth Trehu, MD^4^, Judy Wang, MD^5^

###### ^1^START, San Antonio, TX, United States; ^2^Sarah Cannon Research Inst. at Healthone, Denver, CO, United States; ^3^START-Midwest, Grand Rapids, MI, United States; ^4^Jounce Therapeutics, Cambridge, MA, United States; ^5^Florida Cancer Specialists - SCRI, Sarasota, FL, United States

####### **Correspondence:** Kyriakos Papadopoulos (Kyri.Papadopoulos@startsa.com)

**Background**

JTX-4014 is a fully human mAb consisting of 2 identical hinge-stabilized immunoglobulin gamma 4 (IgG4, S228P) heavy and two identical kappa (Igκ) light chains, that specifically binds to PD-1. The mechanism of action of JTX-4014 is to block the interaction of PD-1 with its ligands, PD-L1 and PD-L2, and augment anti-tumor T-Cell activity. This Phase 1 trial objectives were to evaluate the safety and tolerability of the drug along with its maximum tolerated dose (MTD) and recommended Phase 2 dose.

**Methods**

Key inclusion criteria included age ≥18 yrs, histologically or cytologically confirmed extracranial solid tumor refractory to at least one prior line of therapy, no concurrent anticancer treatment, no prior anti-PD-1 or anti-PD-L1 therapy, no requirement for selection based on PD-L1 expression, no history of immune-mediated conditions, and adequate renal, hepatic, and bone marrow function. The trial was a standard 3+3 design with 5 fixed dose levels ranging from 80 mg Q3wk to 1200 mg Q3wk given by IV infusion. In addition, there was one arm of 800 mg Q6wk.

**Results**

18 patients were enrolled in the trial (10 males, 8 females) with an average age of 66.3 yrs. Tumor types included ovarian (n=4), salivary gland, sarcoma, prostate and mesothelioma (n=2 each). The maximum administered dose was 1200mg; MTD was not reached. There were no deaths, no dose limiting toxicities. One treatment-related serious adverse event of pneumonitis occurred after the second dose at 1200 mg Q3wk. Adverse events occurring in > 15% of patients included fatigue, anemia, AST increased, dizziness, and tumor pain. Only fatigue was noted as related in more than one patient (all Grade 1 and 2). Grade 3 related AEs included increase alkaline phosphatase and pneumonitis. At time of data cutoff, median number of doses administered was 3 (range 1-11). Preliminary investigator assessed antitumor activity included: confirmed partial response (PR) in 1 patient with salivary gland carcinoma, unconfirmed PR in 1 patient with ovarian cancer (both PD-L1+ by IHC) and best response of stable disease in 6 patients. Systemic exposure of JTX-4014 increased dose proportionally; mean terminal half-life ranged from 11 to 17 days. JTX-4014 pharmacokinetics was comparable to other approved anti-PD-1 mAbs. No anti-drug antibodies were observed.

**Conclusions**

JTX-4014 is well-tolerated and appears to have similar qualities to known anti-PD-1 inhibitors in terms of pre-clinical and clinical characteristics. Antitumor activity was observed in the difficult to treat population enrolled. Phase 2 testing JTX-4014 is planned.

**Trial Registration**

NCT03790488

**Ethics Approval**

The study was approved by the relevant Institutions' Ethics Board

#### P440 Phase 1/1b multicenter trial of TPST-1120, a peroxisome proliferator-activated receptor alpha (PPARα) antagonist as a single agent (SA) or in combination in subjects with advanced cancers

##### John Powderly, MD, CPI^1^, Saurin Chokshi, MD^2^, Johanna Bendell, MD^2^, Leisha Emens, MD, PhD^3^, Jason Luke, MD, FACP^3^, Brian Francica^4^, Chan Whiting, PhD^4^, Thomas Dubensky, PhD^4^, Ginna Laport, MD^4^

###### ^1^Carolina BioOncology, Huntersville, NC, United States; ^2^Sarah Cannon Research Institute, Nashville, TN, United States; ^3^University of Pittsburgh, Pittsburgh, PA, United States; ^4^Tempest Therapeutics, South San Francisco, CA, United States

####### **Correspondence:** Thomas Dubensky (tdubensky@tempesttx.com); Ginna Laport (glaport@tempesttx.com)

**Background**

Tumor cells initially rely on glucose consumption via aerobic glycolytic pathways. However, as tumor cells proliferate and metastasize in an increasingly hypoxic tumor microenvironment (TME), tumors increasingly utilize fatty acid oxidation (FAO) as glucose stores are depleted. FAO supports both tumor growth and suppressive immune cells in the TME, facilitating tumor progression. PPARα is a ligand-activated nuclear transcription factor which regulates lipid metabolism, FAO and inflammation. TPST-1120 is a first in class, oral, selective PPARα antagonist that blocks transcription of PPARα target genes leading to a metabolic shift from FAO to glycolysis. Antagonism of FAO in the TME leads to direct killing of tumor cells dependent on FAO and facilitates the cytotoxicity of effector cells. Preclinical studies with various tumor models demonstrate efficacy of TPST-1120 as monotherapy and in combination with anti-PD1 antibodies and chemotherapy. TPST-1120 has an IC50 of 0.04 nM with a >35 fold selectivity over other PPAR isoforms.

**Methods**

We have initiated a phase 1/1b multicenter, open label trial to evaluate TPST-1120 as a SA and in combination (combo) with other systemic therapies including nivolumab, an anti-PD1 monoclonal antibody; docetaxel, a cytotoxic chemotherapeutic agent and cetuximab, an anti-EGFR monoclonal antibody. The objectives are to 1) evaluate safety and tolerability of continuous dosing of TPST-1120 2) identify a recommended phase 2 dose (RP2D) 3) evaluate efficacy, and 4) evaluate PK/PD parameters. Eligibility criteria: 1) patients with advanced non-small cell lung, hepatocellular, renal cell, triple-negative breast, urothelial, pancreatic, gastro-esophageal, castration-resistant prostate, head and neck, or MSS colorectal cancer, or cholangiocarcinoma, or sarcoma; and 2) 1-5 prior therapies for metastatic disease. This phase 1/1b adaptive design is composed of Dose Escalation (DEs) and Dose Expansion (DEx) cohorts. DEs consist of 4 arms, 1 SA arm and 3 combination arms in which TPST-1120 is combined with nivolumab, docetaxel or cetuximab. The RP2D of TPST-1120 to proceed to DEx will be determined by safety and biomarkers including analysis of FAO/PPARα gene expression in the peripheral blood and in tumor biopsies. The DEx arms will follow a 2-stage expansion design. This trial began accrual in May 2018 at U.S sites and is currently enrolling into the Monotherapy/Dose Escalation cohort. Expansion cohorts are projected to open in early 2019. The total sample size is up to 338 pts.

**Trial Registration**

NCT03829436

**Ethics Approval**

This study is being conducted in accordance with Good Clinical Practice and the Helsinki Declaration and has been approved by the Western IRB/Copernicus Group, tracking # 20190182.

#### P441 ARTISTRY-2: a phase 1/2 study of subcutaneously administrated ALKS 4230 as monotherapy and in combination with pembrolizumab in patients with advanced solid tumors

##### John Powderly, MD, CPI^1^, Bradley Carthon, MD, PhD^2^, Marc Ernstoff, MD^3^, Anthony Olszanski, MD, RPh^4^, Stephen Liu, MD^5^, Kelly Curtis, MD^6^, Yangchun Du, PhD^7^, Lei Sun, PhD^7^, Emily Putiri, PhD^7^, Yan Wang, PhD^7^, Heather Losey, PhD^7^, Bruce Dezube, MD^7^, Ulka Vaishampayan, MD^8^

###### ^1^Carolina BioOncology, Huntersville, NC, United States; ^2^Emory University, Atlanta, GA, United States; ^3^Roswell Park Comprehensive Cancer Center, Buffalo, NY, United States; ^4^Fox Chase Cancer Center, Phildelphia, PA, United States; ^5^Georgetown University, Washington, DC, United States; ^6^Syneos Health, Phoenix, AZ, United States; ^7^Alkermes, Inc., Waltham, MA, United States; ^8^Barbara Ann Karmanos Cancer Institute, Detroit, MI, United States

####### **Correspondence:** Bruce Dezube (bruce.dezube@alkermes.com)

**Background**

ALKS 4230 is an engineered fusion protein of circularly permuted interleukin-2 (IL-2) and IL-2 receptor α (IL-2Rα) designed to selectively bind the intermediate-affinity IL-2R for selective expansion of natural killer (NK) and CD8+ T cells (Figures 1 and 2). Compared with recombinant human IL-2, ALKS 4230 exhibited enhanced pharmacokinetic and selective pharmacodynamic properties in mice, resulting in improved antitumor efficacy [1]. Intravenous dosing of ALKS 4230 is being studied in the ARTISTRY-1 trial of patients with advanced solid tumors (NCT02799095), which has more than 50 patients enrolled to date [2]. Here, we present a study investigating ALKS 4230 administered subcutaneously. Potential advantages of subcutaneous dosing over intravenous include: (i) lower peak serum drug concentrations with a prolonged exposure profile, which may result in a milder safety profile and improved tolerability; (ii) lymphatic absorption, which may facilitate direct immunologic effects; and (iii) a more convenient dosing schedule than daily inpatient intravenous dosing.

**Methods**

ARTISTRY-2 (NCT03861793) is a phase 1/2 study of ALKS 4230 administered subcutaneously as monotherapy and in combination with pembrolizumab in patients with advanced solid tumors. The study will be conducted in 2 parts. In the first part (dose escalation; phase 1), multiple ascending doses of ALKS 4230 will be administered subcutaneously every 7 days (q7d) or every 21 days (q21d) during a 6-week lead-in period. Injection site locations will include the back of the arm, the thigh, or the abdomen. If the patient has tolerated ALKS 4230 monotherapy treatment, combination therapy with pembrolizumab (200 mg) administered as an intravenous infusion over 30 minutes q21d will be added to the ongoing ALKS 4230 regimen. In the second part (dose expansion; phase 2), ALKS 4230 will be administered subcutaneously at the selected recommended phase 2 dose (RP2D) and dosing schedule from phase 1 in combination with pembrolizumab in 5 tumor-specific cohorts of patients with non-small-cell lung cancer, small-cell lung cancer, hepatocellular carcinoma, squamous cell carcinoma of the head and neck, and squamous cell carcinoma of any tissue origin. Additional eligibility criteria for the study include Eastern Cooperative Oncology Group performance status of 0 to 1 and adequate bone marrow, liver, and kidney function. Outcomes include RP2D, safety, pharmacokinetics/pharmacodynamics, immunogenicity, and antitumor activity. Efficacy endpoints include overall response rate, disease control rate, duration of response, time to response, and progression-free survival and overall survival at 6- and 12-month milestones.

**Acknowledgements**

The study is sponsored by Alkermes, Inc. Medical writing and editorial support was provided by Parexel and funded by Alkermes, Inc.

**Trial Registration**

ClinicalTrials.gov NCT03861793

**References**

1. Losey HC, Lopes JE, Dean RL, Huff MR, Moroso RA, Alvarez JC. Efficacy of ALKS 4230, a novel immunotherapeutic agent, in murine syngeneic tumor models alone and in combination with immune checkpoint inhibitors. Cancer Res. 2017;77(13 Suppl). Abstract 591.

2. Vaishampayan UN, Fishman MN, Cho DC, Hoimes CJ, Velcheti V, McDermott DF, et al. Intravenous administration of ALKS 4230 as monotherapy and in combination with pembrolizumab in a phase I study of patients with advanced solid tumors. J Clin Oncol. 2019:37(Suppl). Abstract TPS2649.

**Ethics Approval**

This study was approved by Ethics and Institutional Review Boards (IRBs) at all study sites; IRB reference numbers 20182543 (Western IRB), 00006731 (Roswell Park Comprehensive Cancer Center), STUDY00000056 (Georgetown University, MedStar Health Research Institute).

**Fig. 1 (abstract P441).**
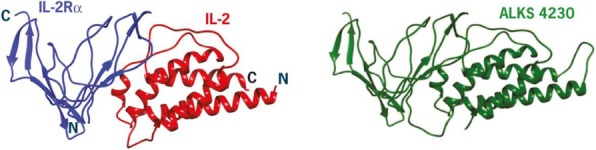
ALKS 4230 is a fusion of IL-2 and IL-2Rα

**Fig. 2 (abstract P441).**
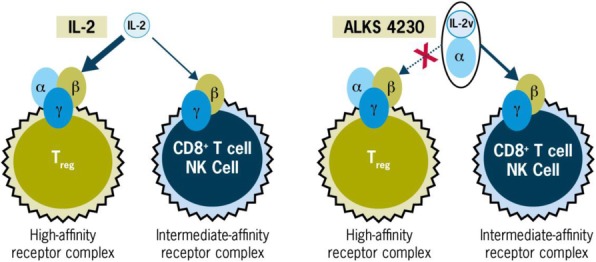
Cell activation by IL-2 and ALKS 4230

#### P442 A phase 1 study of FPT155, a first-in-class CD80 extracellular domain-Fc fusion protein, in patients with advanced solid tumors

##### Jermaine Coward^2^, Hui Gan, MBBS PhD^3^, James Kuo^4^, Michael Millward^5^, Gary Richardson^6^, Wei Deng^7^, Siddhartha Mitra^7^, Maike Schmidt^7^, Hong Xiang, PhD^7^, Lisa Horvath^8^, Amy Prawira, MD^1^

###### ^1^St. Vincent’s Hospital Sydney, Darlinghurst, Australia; ^2^Icon Cancer Centre, Brisbane, Australia; ^3^Olivia Newton-John Cancer Center, Melbourne, Victoria, Australia; ^4^Scientia Clinical Research, Randwick, Australia; ^5^Linear Clinical Research, Nedlands, Australia; ^6^Cabrini Hospital, Malvern, Australia; ^7^Five Prime Therapeutics, Inc, South San Francisco, CA, United States; ^8^Chris O’Brien Lifehouse, Camperdown, Australia

####### **Correspondence:** Amy Prawira (amy.prawira@svha.org.au)

**Background**

T-cell activation requires effective co-stimulation along with T-cell receptor (TCR) engagement. CD80 provides a well-characterized costimulatory signal by binding to CD28 on the surface of T cells. Following activation, T cells upregulate CTLA4 on the cell surface which binds to CD80 with higher affinity than CD28, disrupts effective CD80-CD28 signaling, and inhibits T-cell activation. FPT155 is a novel CD80 extracellular domain-Fc fusion protein that directly induces T-cell activation and cytokine production by binding to CD28 and de-represses endogenous CD80-CD28 activity in the tumor microenvironment by binding to CTLA4. FPT155 has potent efficacy in syngeneic preclinical tumor models, including some that are not responsive to agents targeting PD-1. FPT155 is not a superagonist as it requires separate, concurrent TCR engagement.

**Methods**

FPT155 is being investigated in a multi-center, open-label, first-in-human phase 1 trial. The dose escalation portion of the trial is currently enrolling patients with advanced solid tumors that have progressed after treatment with available therapies. A minimum anticipated biological effect level (MABEL) based approach was used to select the initial dose in humans. Patients receive a fixed dose of FPT155 every three weeks with single-patient accelerated titration cohorts through the first four dose levels of 0.07, 0.21, 0.7 and 2.1 mg and a standard 3+3 dose-escalation design for the subsequent 7, 21, 42, and 70mg dose levels. The primary objective of the phase 1a portion of the trial is to determine the recommended dose and evaluate the safety and tolerability of FPT155.

**Results**

As of June 17, 2019, 7 patients have been treated on study with FPT155 doses ranging from 0.07-7mg; median age was 58 years, 57% had ECOG PS 1 and median number of prior therapies was 4 (range: 2-8). To date, no dose-limiting toxicities or ≥Grade 3 treatment-emergent adverse events (TEAEs) from causes other than disease progression have been reported. There have been no serious adverse events or ≥grade 3 TEAEs attributed to FPT155 and the only TEAE attributed to FPT155 in more than one patient has been fatigue (Gr1, Gr2; 1 pt each). 2/7 patients continue on treatment.

**Conclusions**

FPT155 as monotherapy has been well tolerated to date. Enrollment of accelerated titration cohorts is complete with dose-escalation in 3+3 cohorts ongoing.

**Trial Registration**

ACTRN12618001955202

**Ethics Approval**

The study was approved by IRBs at all participating study sites.

#### P443 Expansion cohorts of non-small cell lung cancer (NSCLC) and castration resistant prostate cancer (CRPC) in COSMIC-021, a phase 1b study of cabozantinib plus atezolizumab

##### Nick Salgia, PhD^1^, Sumanta Pal, MD^1^, Santiago Ponce Aix, MD^2^, Yohan Loriot^3^, Robert Dreicer^4^, Ulka Vaishampayan, MD^5^, Toni Choueiri^6^, Patrick Schöffski^7^, Giri Ramsingh^8^, Amy Liu^8^, Farah Lim^9^, Joel Neal, MD, PhD^10^, Neeraj Agarwal, MD^11^

###### ^1^City of Hope, Duarte, CA, United States; ^2^University Hospital 12 de Octubre, Madrid, Spain; ^3^Institut de Cancérologie Gustave Roussy, Villejui, France; ^4^University of Virginia School of Medicin, Charlottesville, VA, United States; ^5^Karmanos Cancer Institute, Detroit, MI, United States; ^6^Dana-Farber Cancer Institute, Boston, MA, United States; ^7^Leuven Cancer Institute, Leuven, France; ^8^Exelixis, Alameda, CA, United States; ^9^Barts Cancer Institute, London, United Kingdom; ^10^Stanford University Medical Center, Standford, CA, United States; ^11^Huntsman Cancer Institute, Salt Lake City, UT, United States

####### **Correspondence:** Nick Salgia (jennifer.humbert@fishawack.com)

**Background**

Cabozantinib inhibits tyrosine kinases involved in tumor growth, angiogenesis, and immune regulation, including MET, VEGFR, RET, ROS1, and TAM family kinases (TYRO3, AXL, MER). Encouraged by preclinical and clinical studies that suggested that cabozantinib promotes an immune-permissive environment, the safety and efficacy of cabozantinib or cabozantinib in combination with atezolizumab are being evaluated in the COSMIC 021 phase 1b study (NCT03170960) in solid tumors including NSCLC and CRPC. Cabozantinib has demonstrated clinical activity as monotherapy in advanced NSCLC and in previously treated CRPC [1,2]. Here we provide updated trial details for expansion cohorts of NSCLC and CRPC patients.

**Methods**

The dose-escalation stage of this global, open-label trial is completed; in the expansion stage, 20 combination cohorts are being enrolled at the recommended dose of cabozantinib 40 mg QD PO + atezolizumab 1200 mg Q3W IV.

NSCLC cohorts include patients with: (1) nonsquamous (nsq)NSCLC with prior immune checkpoint inhibitor (ICI) therapy (anti–PD-1 or anti–PD-L1); (2) nsqNSCLC without prior systemic anticancer therapy for metastatic disease; (3) EGFR-mutant nsqNSCLC with prior EGFR-targeting therapy. An additional exploratory cohort will assess cabozantinib monotherapy (60 mg) in nsqNSCLC patients with prior ICI therapy.

CRPC cohorts include patients with: (1) metastatic CRPC adenocarcinoma with measurable disease and prior enzalutamide and/or abiraterone therapy; (2) high-risk (measurable visceral metastasis or prostate-specific antigen doubling time of

The study allows an initial enrollment of 30 patients in each cohort with potential for expansion per recommendation by the Study Oversight Committee. Based on preliminary efficacy per RECIST v1.1 and safety, the original cohorts of nsqNSCLC with prior ICI therapy and metastatic CRPC adenocarcinoma with measurable disease and prior enzalutamide and/or abiraterone therapy are being expanded to 80 patients each.

The primary endpoint of the expansion stage is the objective response rate for each cohort. Exploratory objectives include correlation of tumor and plasma biomarkers and immune cell profiles with clinical outcome.

**Trial Registration**

NCT03170960

**References**

1. Smith DC, Smith MR, Sweeney C, Elfiky AA, Logothetis C, Corn PG, Vogelzang NJ, Small EJ, Harzstark AL, Gordon MS, Vaishampayan UN. J Clin Oncol. 2013; 31:412-419.

2. Drilon A, Rekhtman N, Arcila M, Wang L, Ni A, Albano M, Van Voorthuysen M, Somwar R, Smith RS, Montecalvo J, Plodkowski A. Lancet Oncol. 2016; 17:1653-60.

#### P444 The quest for highly potent human papillomavirus-specific T Lymphocytes for Adoptive Immunotherapy of HPV-associated malignancies

##### Pei Yun Teo, PhD^1^, Sandhya Sharma, BSc^2^, Alex Salyer^2^, Dimitrios Wagner^2^, Benjamin Shin^2^, Sachin Thakar^2^, Li-Chun Huang^3^, Shian Jiun Shih^3^, Carlos Ramos^2^, Cliona Rooney, PhD^2^

###### ^1^Tessa Therapeutics/ Baylor College of Medicine, Houston, TX, United States; ^2^Baylor College of Medicine, Houston, TX, United States; ^3^Tessa Therapeutics, Singapore, Singapore

####### **Correspondence:** Cliona Rooney (crooney@bcm.edu)

**Background**

The human papillomavirus is linked to 42,700 new cases of cancers each year [1]. While many HPV-associated cancers can be eradicated by multimodal therapies, recurrent diseases have dismal prognosis [2,3]. HPV-positive tumors express viral antigens (E6 and E7) that are recognized by HPV-specific T cells (HPVST). We are evaluating the adoptive transfer of ex vivo expanded autologous HPVSTs for the treatment of HPV-positive cancers in a phase I clinical trial (HESTIA). To date, 12 patients have been treated and promising outcomes have been attained- 1 complete response and 1 partial response, with minimal toxicities at the dose levels studied (1×108 HPVSTs/m2) so far. However, most patients remained with disease after infusion. A great challenge for HPVST therapy is to generate more specific and potent HPVSTs as ~30% of our HPVST products failed the potency criterion, evaluated by γ-IFN ELISpot assay. The aim of this work was to increase the potency and success rate of HPVST manufacturing.

**Methods**

The current manufacturing strategy uses peripheral blood mononuclear cells (PBMCs) as starting material for the enrichment and expansion of HPVSTs, in the presence of dendritic cells and cytokines. Either low frequency or anergy of HPVSTs, even in HPV-exposed donors, impede growth, and manufacturing failure is largely attributed to non-specific T cell outgrowth. To overcome this problem, we evaluated CD45RA depletion of PBMCs to remove the bulk of non-specific cells (naïve T cells and natural killer (NK) cells). The CD45RA fraction also contains B-cells and T regulatory cells that may inhibit specific outgrowth. We also evaluated the use of an HLA-negative universal lymphoblastoid cell line (uLCL), developed in our center, as a co-stimulatory cell line to rapidly expand the cells while maintaining HPV specificity.

**Results**

HPVSTs manufactured using CD45RA negative PBMC populations as starting material consistently displayed overarchingly higher specificity than HPVSTs manufactured from PBMCs. Interestingly, uLCLs not only supported exponential growth of HPVSTs, but increased their HPV specificity, opening the possibility of producing sufficient HPVSTs for higher dose levels. We reported successful production using PBMC from HPV-exposed healthy donors and cancer patients, and greatly improved HPVST specificity in all.

**Conclusions**

These changes will be incorporated in our HPVST manufacturing protocol with the goal of improving the anti-tumor activity of our product.

**References**

1. Centers for Disease Control and Prevention https://www.cdc.gov/cancer/hpv/index.htm

2. Forastiere AA, Ang KK, Brizel D, et al. Head and neck cancers. J Natl Compr Canc Netw. 2008; 6:646–695.

3. 11. Greer BE, Koh WJ, Abu-Rustum N, et al. Cervical cancer. J Natl Compr Canc Netw. 2008; 6:14–36

**Ethics Approval**

This study was approved by Baylor College of Medicine Institutional Review Board; approval number H7634, H7666 and HESTIA and HESTIA IND.

#### P445 Preliminary results of a Phase 1 trial with a personalized neoantigen vaccine (ADXS-NEO) in advanced and refractory cancer patients

##### Frank Tsai, MD^1^, Jonathan Goldman, MD^2^, Marc Matrana^3^, Sumitra Sheeri^4^, John Heyburn^4^, Megan Parsi^4^, Andres Gutierrez, MD PhD^4^, Joel Hecht^2^, Joel Hecht^2^

###### ^1^Honor Health Virginia Piper Cancer Care, Scottsdale, AZ, United States; ^2^UCLA Jonsson Comprehensive Cancer Center, Paramus, NJ, United States; ^3^Ochsner Cancer Center, New Orleans, LA, United States; ^4^Advaxis Inc, Princeton, NJ, United States

####### **Correspondence:** Joel Hecht (JRHecht@mednet.ucla.edu)

**Background**

ADXS-NEO is a personalized Listeria monocytogenes (Lm)-based immunotherapy. This vaccine is a bioengineered Lm vector that secretes an antigen-adjuvant fusion protein consisting of up to 40 unique (personal) neoantigens and a truncated fragment of listeriolysin O (tLLO), which has adjuvant properties. Preliminary clinical and immunogenicity results from two dose-levels of ADXS-NEO monotherapy evaluated in the ongoing Phase 1 trial are herein reported.

**Methods**

ADXS-NEO-02 is a phase 1 dose-escalation study of ADXS-NEO monotherapy in subjects with advanced and refractory metastatic microsatellite stable-colon cancer (MSS-CRC), metastatic squamous histology head and neck cancer, and metastatic non-small cell lung cancer (NSCLC). Manufacturing of ADXS-NEO starts with whole exome sequencing of each pt-matched normal and tumor samples to detect genetic alterations in the coding regions of the genome followed by its production under GMP specifications. ADXS-NEO is infused intravenously every 3 weeks until disease progression or limiting toxicity. Main endpoints include safety, tolerability, preliminary efficacy and immune-correlative data.

**Results**

The turnaround time for manufacturing ADXS-NEO has consistently been 7-8 weeks from biopsy to first dose. Two pts treated at 1X109 CFU (dose level 1) experienced dose limiting toxicities (i.e., Gr 3 hypoxia ± Gr 3 hypotension) within 4 hours of completing the infusion of the second dose. These acute adverse events were manageable and reversible with tocilizumab and/or steroids. A de-escalated dose of 1X108 CFU, has been found to be safe, tolerable and immunogenic in a cohort of 3 pts. ADXS-NEO at both doses induced: 1) activation and proliferation of CD4+ / CD8+ T cells; 2) neoantigen-specific T cell responses -including hotspot mutations- after 1 week of the initial priming dose in pooled ELISPot analysis and 3) T cell responses to neoantigens found in the pts’ tumor, but not included in the construct (i.e., antigen spreading). Deconvolution ELISPot data from the first MSS-CRC pts. analyzed, showed T cell responses to 90% of the targets in the ADXS-NEO construct. Two out of 4 initial pts treated had stable disease.

**Conclusions**

A safe and tolerable dose of ADXS-NEO monotherapy has been established (1X108 CFU) which elicited fast and broad antitumor immunity, including T cell responses to neoantigens and antigen spreading. Enrollment in a combination therapy arm with pembrolizumab is due to start in 4Q2019.

**Trial Registration**

NCT03265080

**Ethics Approval**

This clinical tria has been performed in accordance with the Declaration of Helsinki and has been approved by appropriate ethics committee at UCLA LA, Ochsner Cancer Center LA and Honor Health Virginia G Piper Cancer Care AX.

#### P446 A novel regulatory T Cell-Targeted Immunotherapy by targeting their crucial signal by HSP90 inhibitors

##### Ayaka Tsuge, MD, Yosuke Togashi, MD, PhD, Kohei Shitara, MD, Hiroyoshi Nishikawa, MD, PhD

###### National Cancer Center, Kashiwa, Japan

####### **Correspondence:** Hiroyoshi Nishikawa (hnishika@east.ncc.go.jp)

**Background**

Cancer immunotherapy, particularly immune checkpoint inhibitors opened a new era of cancer therapy. Yet, the clinical efficacy is limited due to the complexed immune suppressive mechanisms in the tumor microenvironment (TME). Regulatory T (Treg) cells, an immune suppressive subset of CD4+ T cells, are abundant in tumor tissues and play a key role as an immune suppressive mechanism in the TME via inhibiting effective antitumor immunity. While various Treg cell-targeted reagents is under development, none of them have not been translated into the clinic due to the difficulty of specific Treg cell depletion in the TME. The major obstacle to develop effective Treg cell-targeted reagents was the lack of molecules specifically expressed by Treg cells in the TME. We therefore focused on the specific signal(s) used in Treg cells in the TME.

**Methods**

We focused on HSP90 inhibitor, TAS-116 as a Treg cell regulator, especially terminally-differentiated effector Treg cells. Peripheral blood mononuclear cells (PBMCs) were treated with TAS-116, and the changes in T cell populations including Treg cells were analyzed. In addition, we explored the mechanism(s) of Treg cell reduction using PBMCs and FoxP3+ T cell lines. The effect of TAS-116 on tumor antigen (NY-ESO-1)-specific CD8+ T cells was also examined. Furthermore, the possibility of combination treatment of TAS-116 and anti-PD-1 mAb were investigated in animal models

**Results**

TAS-116 significantly reduced Treg cells, particularly effector Treg cells in both peripheral blood and the TME, resulting in augmentation of tumor antigen-specific CD8+　T cells. STAT5, one of the　HSP90 client proteins that is important for Treg cell development, maintenance and function was degraded by TAS-116, thereby reducing FoxP3 expression in effector Treg cells. TAS-116 augmented tumor antigen-specific CD8+　T cells in animal models with reduction of Treg cells in the TME. Additionally, combination treatment with PD-1 blockade exhibited a far stronger antitumor effect than either treatment alone. Moreover, in a phase I trial, the combination of an anti-PD-1 mAb and TAS-116 exhibited a notable clinical efficacy in patients with microsatellite-stable (MSS) colorectal cancer accompanied by effector Treg cell reduction in the TME.

**Conclusions**

We propose a novel concept to control eTreg cells by targeting a Treg cell-critical signaling pathway and the potential as a combination therapy with PD-1 blockade.

**Trial Registration**

UMIN000032801

**Ethics Approval**

This study was approved by the institutional review boards of the National Cancer Center and was conducted in accordance with ethical guidelines, including the Declaration of Helsinki.All mouse experiments were approved by the Animals Committee for Animal Experimentation of the National Cancer Center, Japan, and Taiho Pharmaceutical Co. Ltd.

#### P447 ALKS 4230, an engineered IL-2 fusion protein, in monotherapy dose-escalation and combination therapy with pembrolizumab in patients with solid tumors: ARTISTRY-1 trial

##### Ulka Vaishampayan, MD^1^, Jameel Muzaffar, MD^2^, Vamsidhar Velcheti, MD, FACP^3^, Christopher Hoimes, DO^4^, Lucy Gilbert, MD^5^, David McDermott, MD^6^, Anna Spreafico, MD PhD^7^, Quincy Chu, MD^8^, Kelly Curtis, MD^9^, Yangchun Du, PhD^10^, Harald Mackenzie, MB^10^, Lei Sun, PhD^10^, Emily Putiri, PhD^10^, Heather Losey, PhD^10^, Bruce Dezube, MD^10^, Marc Ernstoff, MD^11^

###### ^1^Barbara Ann Karmanos Cancer Institute, Detroit, MI, United States; ^2^Moffitt Cancer Center, Tampa, FL, United States; ^3^Perlmutter Cancer Center, NYU Langone Health, New York, NY, United States; ^4^UH Cleveland Medical Center, Cleveland, OH, United States; ^5^Cedars Cancer Center, Montreal, QC, Canada; ^6^Beth Israel Deaconess Medical Center, Milton, MA, United States; ^7^Princess Margaret Cancer Centre, Toronto, ON, Canada; ^8^Cross Cancer Institute, University of Alberta/Alberta Health Services, Edmonton, AB, Canada; ^9^Syneos Health, Phoenix, AZ, United States; ^10^Alkermes, Inc., Waltham, MA, United States; ^11^Roswell Park Comprehensive Cancer Center, Buffalo, NY, United States

####### **Correspondence:** Bruce Dezube (bruce.dezube@alkermes.com)

**Background**

ALKS 4230 is an engineered fusion of IL-2 and IL-2Rα designed to selectively expand NK and CD8+ T cells (Figures 1 and 2). In preclinical studies, ALKS 4230 exhibited enhanced pharmacokinetic and selective pharmacodynamic properties with improved antitumor efficacy relative to IL-2 [1].

**Methods**

ARTISTRY-1 (NCT02799095) is a phase 1/2 study investigating ALKS 4230 as monotherapy and in combination with pembrolizumab in adults with advanced solid tumors [2]. For monotherapy dose escalation, ALKS 4230 is administered intravenously over 30 minutes once daily for 5 days every 14 or 21 days. For combination therapy, the same regimen of ALKS 4230 is administered with pembrolizumab every 21 days in cohorts based on tumor type, prior anti-PD-1 therapy, and rollover from monotherapy. Outcomes include the monotherapy recommended phase 2 dose (RP2D), safety, pharmacodynamics, and antitumor activity (RECIST 1.1). Results of the completely enrolled cohorts of dose-escalation phase and of combination therapy in anti-PD-1-unapproved tumors as of June 21, 2019, are presented.

**Results**

For dose escalation, 36 patients received ALKS 4230 monotherapy ≤6 μg/kg/d. Maximum tolerated dose has not been reached. Most frequent adverse events (AEs), regardless of relationship, were pyrexia (75%) and chills (72%); the majority were grades 1 or 2. Grade ≥3 AEs related to ALKS 4230 occurred in 11 patients (31%) and were mainly transient leukopenia. One death from aspiration pneumonia was considered unrelated to ALKS 4230 by the investigator. ALKS 4230 induced dose-dependent increases in circulating NK and CD8+ T cells with minimal, non-dose-dependent effects on regulatory T cells (Tregs). At 3 and 6 μg/kg/d, 8 of 14 patients with evaluable scans had stable disease. One patient with heavily pretreated pancreatic adenocarcinoma had prolonged stable disease with 6+ months of monotherapy; CA19-9 decreased from 2571 U/mL (pretherapy) to 673 U/mL (nadir). Data from 20 patients enrolled in the combination therapy cohort of PD-1-unapproved tumors indicate no new toxicities; 7 of 11 patients with evaluable scans had stable disease or better. One patient (ovarian cancer) had confirmed partial response; CA-125 normalized from a peak of 282 to 24.5 U/mL after 2 months of therapy.

**Conclusions**

ALKS 4230 is a promising agent with acceptable tolerability and preliminary clinical benefit. It selectively expanded CD8+ T cells and NK cells with minimal Treg expansion. The intravenous monotherapy RP2D was established as 6 μg/kg/d. Safety and pharmacodynamic data enabled selection of the 3 μg/kg dose for initial evaluation in combination with pembrolizumab.

**Acknowledgements**

The authors would like to thank all the patients who are participating in this study. The study is sponsored by Alkermes, Inc. Medical writing and editorial support was provided by Parexel and funded by Alkermes, Inc.

**Trial Registration**

ClinicalTrials.gov NCT02799095

**References**

1. Losey HC, Lopes JE, Dean RL, Huff MR, Moroso RA, Alvarez JC. Efficacy of ALKS 4230, a novel immunotherapeutic agent, in murine syngeneic tumor models alone and in combination with immune checkpoint inhibitors. Cancer Res. 2017;77(13 Suppl). Abstract 591.

2. Vaishampayan UN, Fishman MN, Cho DC, Hoimes CJ, Velcheti V, McDermott DF, et al. Intravenous administration of ALKS 4230 as monotherapy and in combination with pembrolizumab in a phase I study of patients with advanced solid tumors. J Clin Oncol. 2019:37(Suppl). Abstract TPS2649.

**Ethics Approval**

This study was approved by Ethics and Institutional Review Boards (IRBs) at all study sites; IRB reference numbers 16-229 (Dana-Farber Cancer Institute), MOD00003422/PH285316 (Roswell Park Comprehensive Cancer Center), 20160175 (Western IRB), i15-01394_MOD23 (New York University School of Medicine), STUDY20190090 (Cleveland Clinic), 0000097 (ADVARRA).

**Fig. 1 (abstract P447).**
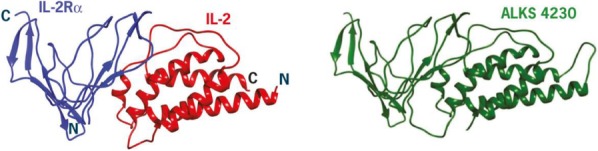
ALKS 4230 structure and activity

**Fig. 2 (abstract P447).**
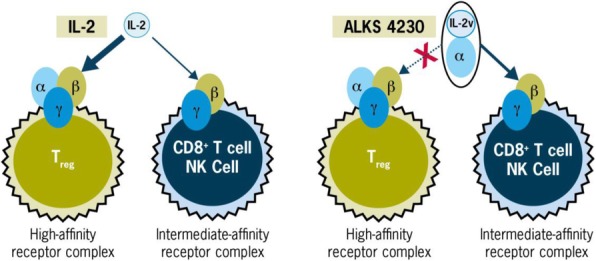
ALKS 4230 structure and activity

#### P448 Phase I study of Veliparib and Nivolumab in adults with refractory advanced solid tumor and lymphoma

##### Young Kwang Chae, MD, Pedro Viveiros, MD, Sheetal Kircher, Valerie Nelson, Aparna Kalyan, Devalingam Mahalingam

###### Northwestern University, Chicago, IL, United States

####### **Correspondence:** Young Kwang Chae (ychae@nm.org)

**Background**

Tumors with high mutation burden often respond to immunotherapy. Veliparib, a Poly (ADP-ribose) polymerase (PARP) inhibitor carries anti-neoplastic activity by accumulating DNA damage, possibly enhancing the effect of checkpoint inhibitors. Here we report safety and preliminary efficacy of veliparib and nivolumab combination from the dose escalation phase of [NCT03061188] clinical trial.

**Methods**

We conducted a phase I study of veliparib in combination with nivolumab in chemo-refractory stage IV/ unresectable solid cancer patients. Nivolumab 240mg IV day 1 and 15 q28 days for 4 cycles; 480mg IV q28 days Cycle 5 onwards, combined with veliparib starting 300mg PO bid 3+3 dose escalation until maximum tolerated dose (MTD) was established. Treatment continued until disease progression or limiting toxicity. Primary objective was establishing MTD for veliparib, defined as the highest dose causing dose-limiting toxicity (DLT) in < 2 of 6 patients. Secondary objective was to assess safety, tolerability and early efficacy of combination.

**Results**

Nine patients with adequate end-organ function and performance status were enrolled. Tumor types included colon cancer (n = 3) and pancreatic cancer (n = 2). Four patients (44%) had BRCA-related somatic mutations (BRCA1, BRCA2, ATM and BRIP1). Most common adverse events categorized as possibly or probably related to treatment were fatigue (n = 6, 67%), anemia (n = 5, 56%), nausea (n = 4, 44%) and diarrhea (n = 3, 33%). Grade 3 and 4 events were anemia (n = 3, 33%), fatigue and alkaline phosphatase elevations (n = 2, 22% each), AST, ALT elevations, low platelet count, hypokalemia and hypertension (n = 1, 11% each). One patient experienced DLT, grade 3 fatigue, at dose level 2 (400 mg BiD) and the MTD was established as 400mg bid. Disease control rate after 24 week follow up was 11%. Five patients presented disease progression (56%). One patient withdrew consent at Cycle 3 and another developed limiting fatigue at Cycle 3, both had stable disease (SD). A patient succumbed due to complications of disease before first assessment. One patient with refractory metastatic pancreatic carcinoma harboring a BRIP1 L680fs*9 mutation had SD after a 35-week follow-up. Median progression-free survival and overall survival were 9 and 25 weeks, respectively.

**Conclusions**

The recommend phase 2 dose of Veliparib is 400mg bid when combined with Nivolumab. The side effect profile is on par with the ones previously described for veliparib and nivolumab in monotherapy. We are expanding the cohort to now include tumors harboring DNA repair defects.

**Trial Registration**

NCT03061188

**Ethics Approval**

The study was approved by Northwestern University Ethics Board. STU00204250.

#### P449 Pharmacodynamic biomarker characterization of ALX148, a CD47 blocker, in combination with established anticancer antibodies in patients with advanced malignancy

##### Hong Wan, PhD^1^, Laura Chow, MD^2^, Justin Gainor, MD^3^, Nehal Lakhani, MD, PhD^4^, Hyun Chung, MD, PhD^5^, Keun-Wook Lee, MD^6^, Jeeyun Lee, MD, PhD^7^, Patricia LoRusso, DO^8^, Yung-Jue Bang, MD PhD^9^, Stephen Hodi^10^, Wells Messersmith, MD^11^, Philip Fanning, PhD^1^, Pierre Squifflet^12^, Feng Jin^1^, Tracy Kuo^1^, Sangeetha Bollini^1^, Jaume Pons, PhD^1^, Sophia Randolph, MD, PhD^1^

###### ^1^ALX Oncology, Burlingame, CA, United States; ^2^University of Washington, Seattle, WA, United States; ^3^MGH Cancer Center, Boston, MA, United States; ^4^START Midwest, Grand Rapids, MI, United States; ^5^Yosei Cancer Center, Seoul, Korea, Republic of; ^6^Seoul University Bundang Hospital, Seongnam, Korea, Republic of; ^7^Samsung Medical Center, Seoul, Korea; ^8^Yale Cancer Center, New Haven, CT, United States; ^9^Seoul National University Hospital, Seoul, Korea; ^10^Dana Farber Cancer Center, Boston, MA, United States; ^11^University of Colorado Cancer Center, Aurora, CO, United States; ^12^International Drug Development Institute, Brussels, Belgium

####### **Correspondence:** Hong Wan (Hong@alxoncology.com)

**Background**

CD47 is a myeloid checkpoint upregulated by tumor cells to evade immune destruction. ALX148 (A) is a fusion protein comprised of a high affinity CD47 blocker linked to an inactive human immunoglobulin Fc region [1]. We have previously shown in the first-in-human clinical trial, that ALX148 is well tolerated in combination with trastuzumab (T) or pembrolizumab (P) with no maximum tolerated dose (MTD) identified [2, 3]. Antitumor activity of ALX148 in combination with T or P was observed in patients with advanced gastric/ gastroesophageal junction (G/GEJ), head and neck squamous cell carcinoma (HNSCC) and non-small cell lung cancer (NSCLC) [4]. The objective of this exploratory analysis was to characterize tumor infiltrating immune cells and molecular signatures from tumor biopsies and peripheral blood samples obtained from this trial.

**Methods**

Patients with HER2-positive malignancy (including G/GEJ cancers progressed on T + fluoropyrimidine and platinum-based therapy) received A+T. Patients with advanced malignancy including NSCLC [checkpoint inhibitor (CPI)-resistant/refractory or PD-L1 tumor proportion score (TPS)

**Results**

Eighty-two patients received A+T (n=30) or A+P (n=52) as of April 18, 2019. In dose expansion cohorts (N=60), anticancer activity was observed in response-evaluable patients [G/GEJ (n=18) 4PR, 5SD; HNSCC (n=19) 3PR, 6SD and NSCLC (n=18) 3SD]. Near complete CD47 TO was maintained throughout the dosing interval. No dose-dependent changes were apparent in peripheral lymphocyte populations. Preliminary results from paired biopsies (n=15) demonstrated increased tumor-associated macrophages and lymphocytes in both intra-tumoral and peri-tumoral regions after treatment with ALX148 combinations. Gene expression signatures of tumor inflammation and immune cell subsets are being investigated. Results will be updated at presentation.

**Conclusions**

ALX148 demonstrates excellent tolerability with objective responses observed in patients with advanced G/GEJ cancer and HNSCC that have progressed on prior systemic and HER2-targeted therapies. Effects on tumor infiltrating immune cells and molecular signatures in correlative biomarker analyses provide insights to ALX148’s mechanism as a myeloid checkpoint inhibitor.

**Acknowledgements**

We would like to thank all of the participating patients and their families as well as site research staff.

**Trial Registration**

ClinicalTrials.gov identifier NCT03013218.

**References**

1. Kauder et al., ALX148 blocks CD47 and enhances innate and adaptive antitumor immunity with a favorable safety profile. PLOS ONE. 2018 13(8): e0201832

2. Lahkani et al., A phase 1 study of ALX148, a CD47 blocker, alone and in combination with established anticancer antibodies in patients with advanced malignancy and non-Hodgkin lymphoma. Journal of Clinical Oncology 2018 36:15_suppl, 3068-3068

3. Lahkani et al., A phase 1 study of ALX148: CD47 blockade in combination with anticancer antibodies to bridge innate and adaptive immune responses for advanced malignancy. Journal for ImmunoTherapy of Cancer 2018 6 (Suppl 1):114. Abstract 335.

4. Chow et al., A phase I study of ALX148, a CD47 blocker, in combination with established anticancer antibodies in patients with advanced malignancy. Journal of Clinical Oncology 2019 37:15_suppl, 2514-2514.

**Ethics Approval**

The study was approved by institutional review boards or independent ethics committees of participating institutions (approval numbers on file at ALX Oncology).

#### P450 A phase I/IIa, open-label, dose-escalation and expansion study to investigate the safety, tolerability, pharmacokinetics and pharmacodynamics of TJ107 in Chinese patients with advanced solid tumors

##### Jin Li^1^, Ye Guo^1^, Wei Peng^1^, Junli Xue^1^, Wei Zhao^1^, Xiaoxiao Ge^1^, Liqiong Xue^1^, Wenbo Tang^1^, Li Zhou^1^, Min Zhang^1^, Bingshi Guo^2^, Liping Wang, MD^2^, Jiyuan Guo^2^, Feifei Cui, PhD^2^, Haiyun Suo^2^

###### ^1^Shanghai East Hospital, Shanghai, China; ^2^I-Mab Biopharma, Shanghai, China

####### **Correspondence:** Jin Li (lijin@csco.org.cn)

**Background**

TJ107, an immuno-oncology agent also known as Hyleukin, is a T cell amplifier comprising a homodimer of engineered human interleukin-7 (IL-7) fused with Genexine’s proprietary long-acting platform hybrid Fc. IL-7 is a critical homeostatic factor for T cells, acting on T cells to increase their number, diversity and functionality. TJ107 could play a pivotal role in reconstitution and reinvigoration of T cell immunity in cancer patients, providing unique opportunities for immuno-oncology combination strategies. The aim of this study (NCT04001075) is to determine the safety, tolerability and PKPD profile of TJ107 in Chinese cancer patients.

**Methods**

This ongoing study is to evaluate the safety, tolerability, PK profile, and anti-tumor activity of TJ107 in patients with advanced solid tumors who failed standard therapy. Patients receive TJ107 every 4 weeks by intramuscular (IM) injection. Dose escalation is aided by a 3+3 scheme from 240μg/kg to 1200μg/kg. A dose expansion cohort is being planned after the RP2D is determined. Safety is assessed by monitoring AEs and the associated grades per NCI CTCAE v5.0. Tumor response will be assessed per RECIST v1.1. Samples will be collected for PK, PD, ADA, immunophenotyping and TCR repertoire analysis.

**Results**

Three patients with colorectal cancer were enrolled in the first cohort (240μg/kg).TJ107 was well tolerated and no DLTs were reported during the first cycle at this dose level. The preliminary PK results shows that TJ107 was rapidly absorbed and reached serum peak concentration around 24 hours post-dose. TJ107 was slowly cleared from the body and remained detectable in serum until Day 14 post-dose. A substantial increase in absolute lymphocyte count (ALC) from baseline was observed and peaked around 3 to 4 weeks post first dose. FACS analysis revealed increases in CD3+, CD4+ and CD8+ T cells. The numeric increase in T cells is consistent with increased Ki67 expression on Day 8 post first dose. There were no notable changes in B cells, monocytes, NK cells, neutrophils, nor Tregs, as expected.

**Conclusions**

Preliminary results from this trial suggest that TJ107 activated IL-7 pathway and expanded T cells in cancer patients in a similar way to data previously reported in healthy subjects. TJ107 exhibits a promising safety and tolerability profile in cancer patients under current dose. These findings support further clinical investigation.

**Trial Registration**

Investigate the Safety, Tolerability, Pharmacokinetics and Pharmacodynamics of TJ107 in Chinese Patients With Advanced Solid Tumors. ClinicalTrials.gov Identifier: NCT04001075

**Ethics Approval**

The study was approved by the Ethics Committee Shanghai East Hospital's, approval number 2018 (058).

#### P451 First-in-human study of CD40 agonist MEDI5083 in advanced solid tumors with durvalumab administered sequentially or concurrently

##### Ben Tran, MBBS FRACP^1^, Mark Voskoboynik^2^, Johanna Bendell, MD^3^, Martin Gutierrez, MD^4^, Charlotte Lemech, MBBS BSc(med) MD(res)^5^, Daphne Day^6^, Sophia Frentzas^6^, Ignacio Garrido-Laguna^7^, Chris DelNagro^8^, Fujun Wang^8^, Charles Ferte, MD, PhD^8^, Mayukh Das^8^, Benedito Carneiro, MD^9^

###### ^1^Peter MacCallum Cancer Center, Melbourne, Australia; ^2^Nucleus Network, Melbourne, Australia; ^3^Sarah Cannon Research Institute, Nashville, TN, United States; ^4^Hackensack University Medical Center, Hackensack, NJ, United States; ^5^Scientia Clinical Research, Sydney, Australia; ^6^Monash Medical Centre, Clayton, Australia; ^7^Huntsman Cancer Institute, Salt Lake City, UT, United States; ^8^AstraZeneca, San Francisco, CA, United States; ^9^The Warren Alpert Medical School, Providence, RI, United States

####### **Correspondence:** Mayukh Das (mayukh.das@medimmune.com)

**Background**

MEDI5083 is a homodimeric fusion protein of three single-chain CD40L domains linked to an immunoglobulin G fragment crystallizable domain, and activates the CD40 pathway to promote immune responses. This first-in-human study evaluated the safety and clinical activity of MEDI5083 given sequentially or concurrently with the PD-L1 antibody durvalumab in patients with advanced solid tumors.

**Methods**

Eligible patients with metastatic or recurrent tumor types progressing on or refractory to prior therapy were enrolled in multiple cohorts of MEDI5083 (3mg, 4mg, 5mg, 6mg, and 7.5mg) administered subcutaneously (SC) Q2W for 4 doses. In the sequential-treatment cohort, MEDI5083 was followed by a 4-week wash-out, then durvalumab 1500 mg intravenously (IV) Q4W. In the concurrent-treatment cohort, MEDI5083 was administered with concurrent durvalumab 1500 mg IV Q4W for 2 doses, followed by durvalumab 1500 mg IV Q4W. The primary endpoint was safety. Secondary endpoints included pharmacokinetics, immunogenicity, and efficacy based on investigator-assessed RECIST V1.1.

**Results**

As of June 30, 2019, 38 patients were treated; 29 received sequential treatment (MEDI5083 3mg, n=4; 4mg, n=4; 5mg, n=18; 7.5mg, n=3) and 9 patients received concurrent treatment (MEDI5083 3mg, n=3; 4mg, n=6). Two patients (sequential cohort, MEDI5083 5mg and 7.5mg) had MEDI5083-related G3/4 dose-limiting toxicities (injection-site reaction [ISR], lymph node pain; ISR). Among all treated patients, the most common adverse events (AEs) were ISR (89.5%), fatigue (39.5%), nausea (28.9%), constipation, and decreased appetite (23.7% each). Nine (23.7%) patients had MEDI5083-related ≥G3 events, most commonly ISR. Six (15.8%) patients discontinued due to a MEDI5083-related AE. There were 4 (10.5%) deaths due to AEs (sequential cohort, MEDI5083 5mg, n=2 and MEDI5083 7.5mg, n=1; concurrent cohort, MEDI5083 4mg, n=1), 3 unrelated to treatment and 1 possibly related to MEDI5083. The maximum tolerated dose for MEDI5083 was 5mg. In the response evaluable population (N=36), a PR was observed (head and neck squamous cell carcinoma; sequential cohort, MEDI5083 3mg; time to response 5.7 months) and 11 (30.6%) patients had SD (sequential cohort, n=7; concurrent cohort, n=4). Six patients had SD ≥24 weeks. The ORR (95% CI) was 2.8% (0.1–14.5%). MEDI5083 showed dose-dependent pharmacological activity in the mobilization of peripheral blood B cells, and induced measurable increases in activated proliferative Ki-67+ CD8+ T cells in peripheral blood.

**Conclusions**

Subcutaneous administration of MEDI5083 caused high rates of injection site reactions. The toxicity profile does not support further development of the subcutaneous formulation of this drug.

**Trial Registration**

ClinicalTrials.gov NCT03089645

**Ethics Approval**

This study was approved by the Institutional Review Board/Independent Ethics Committee at each investigational site participating in the study

#### P452 SPEARHEAD-1 trial design: A phase 2, single arm, open-label clinical trial of ADP-A2M4 SPEAR T-cells in patients with advanced synovial sarcoma or myxoid/round cell liposarcoma

##### Dejka Araujo, MD^1^, Jean-Yves Blay^2^, Sandra Strauss^3^, Claudia Valverde^4^, Erin Van Winkle^5^, Malini Iyengar, PhD^5^, Rafael Amado, MD^5^

###### ^1^MD Anderson Cancer Center, Houston, TX, United States; ^2^Leon Berard, Lyon, France; ^3^University College London Hospitals, London, United Kingdom; ^4^Vall D'Hebron University Hospital, Barcelona, Spain; ^5^Adaptimmune, Philadelphia, United States

####### **Correspondence:** Erin Van Winkle (erin.vanwinkle@adaptimmune.com)

**Background**

ADP-A2M4 specific peptide enhanced affinity receptor (SPEAR) T-cells are genetically engineered to target MAGE-A4+ tumors in the context of HLA-A*02. MAGE-A4 has been described as having high expression in synovial sarcoma (SS) and myxoid/round cell liposarcoma (MRCLS). In recent studies [1, 2], immunohistochemistry (IHC) analyses showed that 82% of SS samples and 68% of MRCLS samples expressed MAGE-A4. A pilot study (NCT03132922) of ADP-A2M4 induced clinical responses in patients with SS.

**Methods**

This phase 2, open-label trial (SPEARHEAD-1 Trial) will evaluate the efficacy, safety and tolerability of ADP-A2M4 SPEAR T-cells. Patients who are HLA-A*02+ (excluding A*02:05, and A*02:07 and A*02 null as sole A*02 alleles), who have advanced/metastatic SS or MRCLS who have received prior chemotherapy, and have MAGE-A4 expression assessed by IHC at ≥2+ in ≥ 30% of tumor cells, and who meet all other inclusion criteria are eligible for treatment. Up to 60 patients will be treated.

Following apheresis, T-cells are isolated, transduced with MAGE-A4c1032TCR, and expanded. Prior to infusion, patients will receive lymphodepletion consisting of fludarabine (30 mg/m2/day x 4 days) and cyclophosphamide (600 mg/m2/day x 3 days). Patients will receive 1 – 10 × 10^9 transduced T-cells. Futility analysis will be conducted after 15 patients are dosed and have been followed for at least 4 months from the time of T-cell infusion. An independent Data Safety Monitoring Board will review ongoing safety and benefit:risk during the interventional phase of the study. Disease will be assessed by independent review per RECIST v1.1 by CT/MRI at weeks 4, 8, 12, 16, 24, and every 2 months thereafter until confirmed disease progression. Once disease progression is established, patients will enter the long-term follow-up phase of the study, with visits every 6 months through Year 5, and annually thereafter for Years 6-15.

**Trial Registration**

NCT04044768

**References**

1. Iura K, et al. Cancer-testis antigen expression in synovial sarcoma: NY-ESO-1, PRAME, MAGEA4, and MAGEA1. Human Pathology 2017a; 61:130-139.

2. Iura K, et al. MAGEA4 expression in bone and soft tissue tumors: its utility as a target for immunotherapy and diagnostic marker combined with NY-ESO-1. Virchow Archiv 2017b;471:383–392.

**Ethics Approval**

This trial is under review by the institutional review board of the trial sites

#### P453 Autologous T cells with NY-ESO-1-specific T-cell receptor (GSK3377794) in HLA-A*02+ previously-treated and -untreated advanced metastatic/unresectable synovial sarcoma: A master protocol study design

##### Sandra D'Angelo, MD^1^, Jean-Yves Blay^2^, Warren Chow^3^, George Demetri^4^, Fiona Thistlethwaite, MD, PhD^5^, Michael Wagner^6^, David Loeb^7^, Steven Attia^8^, Albiruni Razak^9^, John Haanen, MD PhD^10^, Aisha Hasan, MBBS MD^11^, Julia Billiard^11^, Laura Pearce^11^, Yuehui Wu^11^, Ran Ji^11^, Laura Johnson^11^, Chandra Srinath^11^, Aiman Shalabi^11^, Sandra Strauss^12^, Katherine Thornton^4^, Crystal Mackall, MD^13^, William Tap^1^, Brian Van Tine, MD, PhD^14^

###### ^1^Memorial Sloan Kettering Cancer Center, New York, NY, United States; ^2^Centre Léon Bérard, Lyon, France; ^3^City of Hope Comprehensive Cancer Center, Duarte, CA, United States; ^4^Dana-Farber Cancer Institute, Boston, MA, United States; ^5^The Christie NHS Foundation Trust, Manchester, United Kingdom; ^6^Fred Hutchinson Cancer Research Center, Seattle, WA, United States; ^7^Montefiore Medical Center, New York, NY, United States; ^8^Mayo Clinic in Florida, Jacksonville, FL, United States; ^9^Princess Margaret Cancer Centre, Toronto, Canada; ^10^Antoni van Leeuwenhoek Ziekenhuis, Amsterdam, Netherlands; ^11^GlaxoSmithKline, Collegeville, PA, United States; ^12^University College London Hospitals, London, United Kingdom; ^13^Stanford University, Stanford, CA, United States; ^14^Washington University in St. Louis, St. Louis, MO, United States

####### **Correspondence:** Sandra D'Angelo (dangelos@mskcc.org)

**Background**

Synovial sarcoma (SS) comprises ~5%–10% of soft-tissue sarcomas [1]. Anthracycline-based chemotherapy is a 1st-line treatment in advanced metastatic/unresectable disease, but response rates are low

**Methods**

A clinical trial is underway utilizing a Master Protocol design allowing investigation of GSK3377794 in multiple tumor types (NCT03967223). The first 2 sub-studies are single-arm trials evaluating treatment in previously-untreated (sub-study 1) and previously-treated (sub-study 2) HLA A*02+ patients with NY-ESO-1+ metastatic SS. Sub-study 1 is a pilot study evaluating efficacy as 1st-line treatment (N=10). Sub-study 2 plans to enroll 55 patients with metastatic/locally advanced unresectable SS who have progressed following anthracycline-based chemotherapy. Inclusion criteria include: ≥10 years of age; measurable disease; adequate organ function; ECOG performance status 0–1. Exclusion criteria include: CNS metastases; clinically significant systemic illness; prior gene therapy with integrating vector or NY-ESO-1-specific T cells, vaccine or targeting antibody; prior autoimmune disease or allogeneic hematopoietic stem-cell transplant. Patients will undergo eligibility screening; leukapheresis and manufacture of GSK3377794; lymphodepletion and infusion of GSK3377794 followed by safety follow-up and disease assessments; and a 15-year follow-up under a separate protocol (Figure 1).

**Results**

The primary objective of sub-study 2 is to evaluate GSK3377794 efficacy by overall response rate per RECIST v1.1 (central independent review). Secondary objectives include: time to and duration of response; disease control rate; progression-free survival; overall survival; potential immune response to GSK3377794; safety and tolerability. Exploratory objectives include: correlation of T-cell persistence with safety, clinical responses, and phenotype of infused T cells. Impact on quality of life and daily functioning will also be assessed.

**Conclusions**

Based on the encouraging clinical activity of GSK3377794 observed in earlier trials, this larger clinical trial is being initiated to establish and further discern the efficacy and safety of GSK3377794 in this biomarker-selected metastatic SS patient population. This innovative Master Protocol study design permits evaluation of GSK3377794 treatment in other NY-ESO-1+ tumor types in HLA A*02+ patients within separate sub-studies.

**Acknowledgements**

Medical writing assistance was provided by Fiona Woodward of Fishawack Indicia Ltd, UK, funded by GlaxoSmithKline (GSK). This study (NCT03967223) was funded by GSK.

**Trial Registration**

NCT03967223

**References**

1. Riedel RF, Jones RL, Italiano A, et al. Systemic anti-cancer therapy in synovial sarcoma: A systematic review. Cancers 2018; 10:E417.

2. Vlenterie M, Litière S, Rizzo E, et al. Outcome of chemotherapy in advanced synovial sarcoma patients: Review of 15 clinical trials from the European Organisation for Research and Treatment of Cancer Soft Tissue and Bone Sarcoma Group; setting a new landmark for studies in this entity. Eur J Cancer 2016; 58:62–72.

**Ethics Approval**

This Master Protocol will be conducted under approval by the appropriate institutional review boards and independent ethics committees.

**Fig. 1 (abstract P453).**
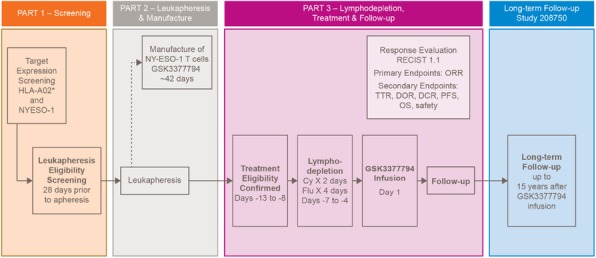
Study Design

#### P454 Induction of serum CXCL10 by tebentafusp, a gp100-CD3 bispecific fusion protein, was associated with survival in uveal melanoma in a Phase I/II Study

##### Marcus Butler, MD^1^, Brandon Higgs^2^, Cheryl McAlpine, MSN^2^, Joseph Sacco^3^, Jessica Hassel, MD^4^, Shaad Abdullah, MD^5^, Koustubh Ranade, PhD^5^, Richard Carvajal, MD^6^

###### ^1^Princess Margaret Cancer Centre, Toronto, Canada; ^2^Immunocore, Ltd, Oxfordshire, United Kingdom; ^3^Clatterbridge Cancer Centre, Liverpool, United Kingdom; ^4^University Hospital Heidelberg, Heidelberg, Germany; ^5^Immnuocore, Ltd, Rockville, MD, United States; ^6^Columbia University Medical Center, New York, NY, United States

####### **Correspondence:** Brandon Higgs (brandon.higgs@immunocore.com)

**Background**

Tebentafusp (formerly IMCgp100) is a TCR–anti-CD3 bispecific fusion protein targeting melanocyte-expressed gp100 antigen; Phase I/II clinical studies showed monotherapy activity in metastatic melanoma including uveal (UM) [1]. Historical 1-year survival (OS) rate for 2L metastatic UM is ~35% [2]. In a phase I clinical study of tebentafusp in cutaneous and 19 UM patients, serum IFNg-induced chemokines, particularly CXCL10, were induced by tebentafusp and high induced levels correlated with OS and tumor reduction. In a subsequent phase I/II trial (NCT02570308) of 2L UM, we sought to confirm this and increase mechanistic understanding with blood mRNA analysis.

**Methods**

NCT02570308 was conducted in HLA-A*0201+ patients with advanced 2L UM; this exploratory analysis focused on further investigation of an initial 40 patient cohort [3]. Intra-patient escalation dosing regimen used low initial dosing at Cycle1, Day1 (C1D1, 20 mcg) and C1D8 (30 mcg). From C1D15, 19 patients received between 54-73 mcg , 22 patients received 68 mcg (expansion phase). Sera from 18 and 22 patients in escalation and expansion phases, respectively were profiled pre-treatment and post first and third dose with 11 immune markers (Luminex); whole blood from 19 escalation phase patients was analysed for gene expression (NanoString). Low/High groups were defined at the median for OS and Mann-Whitney tests were used for time contrasts.

**Results**

In an updated analysis of rash (on-target, off-tumor toxicity), 31 of 40 patients with Grade 2+ rash had 1-year OS rate ~77%; 1-year OS in remaining patients was ~35%. Tebentafusp induced a transient response in cytokines, reaching maximal changes at 8-24 hours post first and third doses. High levels of CXCL10 induced at first dose correlated with improved OS (HR=0.37 95%CI=[0.15, 0.89]); high induced CXCL9 levels also trended with OS (HR=0.52 95%CI=[0.21,1.3]). CXCL9/CXCL10 transcripts increased at the same time points, as did signatures for neutrophils, type I IFN, and eosinophils (folds>1.5, p<-2, p<-1.5, p

**Conclusions**

In this exploratory analysis, high CXCL10 levels induced by tebentafusp associated with improved OS in UM. Tebentafusp reduced CD8+ and CD4+ gene signatures in the blood and induced systemic cytokine and gene expression responses, consistent with T cell redirection and immune activation. Patients who develop tebentafusp-induced Grade 2+ rash appear to have better survival than those who do not.

**Trial Registration**

NCT02570308

**References**

1. Middleton MR. J Clin Oncol 37, 2019 (suppl; abstr 9523)

2. Rantala ES, Hernberg M, Kivelä TT. Overall survival after treatment for metastatic uveal melanoma: a systematic review and meta-analysis. Melanoma Res. 2019 Jan 16. doi: 10.1097/CMR.0000000000000575.

3. Sato T DOI: 10.1200/JCO.2017.35.15_suppl.9531 Journal of Clinical Oncology 35, no. 15_suppl (May 20 2017) 9531-9531.

**Ethics Approval**

This study was in accordance with the Declaration of Helsinki and was approved by all IRBs/ethics committees from each clinical sites participating in the study. Specific approval numbers can be provided upon request.

#### P455 A randomized phase 2 study of neoadjuvant talimogene laherparepvec (T-VEC) plus surgery vs surgery for resectable stage IIIB-IVM1a melanoma: 2-year primary analysis of recurrence-free survival (RFS)

##### Reinhard Dummer, MD^1^, David Gyorki^2^, John Hyngstrom, MD^3^, Adam Berger, FACS, MD^4^, Robert Conry, MD^5^, Lev Demidov^6^, Anjali Sharma^7^, Sheryl Treichel^7^, Kevin Gorski^7^, Abraham Anderson, PhD^7^, Mark Faries^8^, Merrick Ross, MD^9^

###### ^1^University Hospital of Zurich, Zurich, Switzerland; ^2^Olivia Newton-John Cancer Centre, Melbourne, Australia; ^3^University of Utah Huntsman Cancer Inst, Salt Lake City, UT, United States; ^4^Rutgers Cancer Institute of New Jersey, New Brunswick, United States; ^5^University of Alabama School of Medicine, Birmingham, AL, United States; ^6^N.N. Blokhin Russian Cancer Research Ce, Moscow, United States; ^7^Amgen Inc, Thousand Oaks, United States; ^8^John Wayne Cancer Institute, Santa Monica, United States; ^9^University of Texas MD Anderson Cancer, Houston, TX, United States

####### **Correspondence:** Reinhard Dummer (Reinhard.Dummer@usz.ch)

**Background**

Risk of recurrence and death after resection of stage IIIB-IVM1a melanoma is high. In the previous 1-year interim analysis, T-VEC plus surgery demonstrated a pathological complete response rate of 22.8% and improved RFS compared to upfront surgery (Dummer et al, ASCO 2019). Here, we present results from the primary 2-year RFS and biomarker analyses (CT.gov identifier: NCT02211131).

**Methods**

Patients with resectable stage IIIB-IVM1a melanoma, ≥ 1 injectable cutaneous, subcutaneous, or nodal lesions ≥10 mm, and no systemic treatment 3 months prior were randomized 1:1 to 6 doses/12 wks of neoadjuvant T-VEC followed by surgery during weeks 13-18 (Arm 1) versus surgery during weeks 1-6 (Arm 2). T-VEC was given at standard dosing until surgery, no remaining injectable tumors, or intolerance. The primary analysis estimated a between-group difference in 2-yr RFS on the intent-to-treat set. RFS event was defined as the first local, regional, or distant recurrence or death due to any cause after surgery. Per protocol, patients who withdrew prior to surgery or had an R1 or R2 resection were counted as an RFS event at randomization. An additional analysis calculated RFS from randomization to the date of first post-surgery event regardless of surgical margin status.

**Results**

150 pts were randomized (76 arm 1, 74 arm 2). Median (range) follow-up time was 31.2 (0.1–49.9) months. 75% in Arm 1 and 93% in Arm 2 had surgery as planned. In the per protocol analysis, 29.5% of patients in Arm 1 and 16.5% of patients in Arm 2 remained recurrence free (HR: 0.75, P=0.07). In the additional analysis, 50.5% of pts in Arm 1 and 30.2 % in Arm 2 remained recurrence free (HR: 0.66, P=0.038). 2-year overall survival rates were 88.9% in Arm 1 and 77.4% in Arm 2 (HR: 0.49, P=0.050). In arm 1, T-VEC treatment resulted in a 3-fold increase (P

**Conclusions**

Neoadjuvant T-VEC improved 2-year RFS and OS in resectable stage IIIB-IVM1a melanoma. T-cell influx and PD-L1 upregulation after T-VEC treatment support a role for the adaptive immune system consistent with the mechanisms of action. Additional biomarker results including clinical correlations will be presented at the congress.

**Trial Registration**

CT.gov identifier: NCT02211131

**Reference**

1. Dummer R, et al. Presented at The American Society of Clinical Oncology; May 31–June 3, 2019; Chicago IL, USA. J Clin Oncol 37, 2019 (suppl; abstr 9520)

**Ethics Approval**

The study was approved by participating institutions' Ethics Board.

#### P456 KEYNOTE-630: phase 3 study of adjuvant pembrolizumab versus placebo in patients with high-risk, locally advanced cutaneous squamous cell carcinoma

##### Jessica Geiger^1^, Gregory Daniels, MD, PhD^2^, Ezra Cohen, MD^2^, Joy Yang Ge^3^, Burak Gumuscu, MD PhD^3^, Ramona Swaby, MD^3^, Anne Lynn Chang^4^

###### ^1^Cleveland Clinic, Cleveland, OH, United States; ^2^University of California, San Diego, La Jolla, CA, United States; ^3^Merck & Co., Inc., Kenilworth, NJ, United States; ^4^Stanford University Medical Center, Stanford, CA, United States

####### **Correspondence:** Jessica Geiger (GEIGERJ@ccf.org)

**Background**

Despite undergoing current standard-of-care surgical resection and adjuvant radiotherapy, ~20% of patients with high-risk, locally advanced cutaneous squamous cell carcinoma develop local recurrence within 5 years [1]. Recent data show effective antitumor activity and acceptable safety of programmed death 1 inhibitors in patients with locally advanced or metastatic cutaneous squamous cell carcinoma. KEYNOTE-630 (NCT03833167), a randomized, double-blind, placebo-controlled phase 3 trial, will evaluate the efficacy and safety of adjuvant pembrolizumab in patients with high-risk locally advanced or metastatic cutaneous squamous cell carcinoma.

**Methods**

Patients with high-risk locally advanced cutaneous squamous cell carcinoma who have undergone surgical resection and radiotherapy will be randomly assigned 1:1 to intravenous pembrolizumab (400 mg every 6 weeks) or placebo for up to 9 cycles (~1 year) or until disease recurrence, unacceptable toxicity, or investigator or patient decision to withdraw. Randomization will be stratified by extracapsular extension (yes vs no), cortical bone invasion (yes vs no), and prior systemic therapy (yes vs no). Eligible patients are adults with histologically confirmed locally advanced cutaneous squamous cell carcinoma with ≥1 high-risk features at the primary site of malignancy and macroscopic resection with or without microscopic positive margins who completed adjuvant radiotherapy, were disease free ≤28 days from randomization, and have Eastern Cooperative Oncology Group performance status 0 or 1. The primary efficacy end points are investigator-assessed and biopsy-confirmed recurrence-free survival. Secondary end points are overall survival, health-related quality of life, and safety. To assess treatment response, radiographic imaging will be performed at least every 12 weeks in year 1, then every 6 months until the end of year 5. All patients meeting crossover or retreatment criteria at first disease recurrence may receive pembrolizumab 400 mg every 6 weeks for up to 18 cycles. Adverse events will be recorded until 30 days (90 days for serious adverse events) after study end and will be graded per NCI CTCAE v4.0. Enrollment of ~570 patients is planned, and recruitment is ongoing in 18 countries.

**Trial Registration**

ClinicalTrials.gov, NCT03833167

**Reference**

1. Porceddu SV, Bressel M, Poulsen MG, et al. Postoperative concurrent chemoradiotherapy versus postoperative radiotherapy in high-risk cutaneous squamous cell carcinoma of the head and neck: the randomized phase III TROG 05.01 trial. J Clin Oncol. 2018;36:1275-1283.

**Ethics Approval**

The study and the protocol were approved by the Institutional Review Board or ethics committee at each site.

**Consent**

All patients provided written informed consent to participate in the clinical trial.

#### P457 Randomized phase II neoadjuvant study: PD-1 inhibitor TSR-042 vs. combination PD-1 inhibitor TSR-042 and Tim-3 inhibitor TSR-022 in borderline resectable stage III or oligometastatic stage IV melanoma

##### Zahra Kelly, DO^1^, Yana Najjar, MD^2^, Hassane Zarour, MD^2^, John Kirkwood, MD^2^, Suthee Rapisuwon, MD^3^, Hong Wang^2^, Marc Ernstoff, MD^4^, Joseph Drabick, MD, FACP, FIDSA^5^, Diwakar Davar, MD^2^, Mohan Bala^6^

###### ^1^University of Pittsburgh Medical Center, Pittsburgh, PA, United States; ^2^University of Pittsburgh, Pittsburgh, PA, United States; ^3^Georgetown University, Washington, DC, United States; ^4^Roswell Park Comprehensive Cancer Center, Buffalo, NY, United States; ^5^Penn State Cancer Institute, Palmyra, PA, United States; ^6^Tesaro, Inc, Wltham, MA, United States

####### **Correspondence:** Diwakar Davar (davard@upmc.edu)

**Background**

Neoadjuvant PD-1 blockade produces pathological responses in ~30% of patients (pts) with high-risk resectable melanoma (MEL) with durable relapse-free benefit, and increased circulating activated CD8+ T cells (1,2). TIM-3 is an inhibitory immune checkpoint and mediates immune escape; blockade of which produces anti-tumor immune responses and synergizes with anti-PD-1 (3–5). TSR-042/dostarlimab is an IgG4 humanized monoclonal antibody that binds with high affinity to PD-1, inhibiting binding to PD-L1 and PD-L2. TSR-042 has been studied in patients with advanced non-small cell lung (NSCLC) and endometrial cancers with promising results (6). TSR-022 is an IgG4-k isotype humanized monoclonal antibody that binds with high affinity to TIM-3, thus enhancing T cell activity. TSR-042/TSR-022 combination has been studied in a phase I/II study that demonstrated promising efficacy of combination in PD-1 refractory melanoma and NSCLC (7). We hypothesized that neoadjuvant therapy with TSR-042/TSR-022 combination may improve pathologic response rates compared to TSR-042 monotherapy in high-risk resectable MEL.

**Methods**

Pts with regionally advanced (stage IIIB-D) or oligometastatic (stage IVA-B) melanoma who have yet to undergo definitive surgery are eligible. Primary endpoints are rate of major pathologic response (MPR), safety, and incidence of dose-limiting toxicities (DLT). Secondary endpoints are radiographic response, relapse-free survival (RFS) and overall survival (OS). Pathological response will be assessed depending on residual volume of tumor (RVT) using prior cutoffs: 0% (complete response, pCR); 0%<RVT<RVT50% (non-response, pNR) (8–10). Sample size is 28 patients per arm. Patients will receive neoadjuvant therapy for 6 weeks prior to planned surgery. Surgery will occur 1-3 weeks after completion of pre-operative therapy. After surgery, subjects will receive additional maintenance TSR-042 for approximately 48 weeks (Figure 1). Sample size provides 80% power to detect an improvement of 30% upon MPR rate of 30% with anti-PD-1 monotherapy.

**References**

1. Amaria, R. N. et al. Neoadjuvant immune checkpoint blockade in high-risk resectable melanoma. Nature Medicine 24, 1649–1654 (2018).

2. Huang, A. C. et al. A single dose of neoadjuvant PD-1 blockade predicts clinical outcomes in resectable melanoma. Nature Medicine 25, 454–461 (2019).

3. Fourcade, J. et al. Upregulation of Tim-3 and PD-1 expression is associated with tumor antigen–specific CD8+ T cell dysfunction in melanoma patients. The Journal of Experimental Medicine 207, 2175–2186 (2010).

4. Chauvin, J.-M. et al. TIGIT and PD-1 impair tumor antigen–specific CD8+ T cells in melanoma patients. Journal of Clinical Investigation 125, 2046–2058 (2015).

5. Woo, S.-R. et al. Immune Inhibitory Molecules LAG-3 and PD-1 Synergistically Regulate T-cell Function to Promote Tumoral Immune Escape. Cancer Research 72, 917–927 (2012).

6. Moreno, V. et al. Abstract CT053: Preliminary safety, efficacy, and PK/PD characterization from GARNET, a phase 1 clinical trial of the anti-PD-1 monoclonal antibody, TSR-042, in patients with recurrent or advanced NSCLC and MSI-H endometrial cancer. Cancer Research 78, CT053–CT053 (2018).

7. Davar, D. A phase 1 study of TSR-022, an anti-TIM-3 monoclonal antibody, in combination with TSR-042 (anti-PD-1) in patients with colorectal cancer and post-PD-1 NSCLC and melanoma. (2018).

8. Cottrell, T. et al. Pathologic Features of Response to Neoadjuvant Anti-PD-1 in Resected Non-Small Cell Lung Carcinoma: A Proposal for Quantitative Immune-Related Pathologic Response Criteria (irPRC). Annals of oncology : official journal of the European Society for Medical Oncology (2018). doi:10.1093/annonc/mdy218

9. Stein, J. et al. Major pathologic response on biopsy (MPRbx) in patients with advanced melanoma treated with anti-PD-1: evidence for an early, on-therapy biomarker of response. Annals of oncology : official journal of the European Society for Medical Oncology 30, 589–596 (2019).

10. Tetzlaff, M. et al. Pathological assessment of resection specimens after neoadjuvant therapy for metastatic melanoma. Annals of oncology : official journal of the European Society for Medical Oncology 29, 1861–1868 (2018).

**Fig. 1 (abstract P457).**
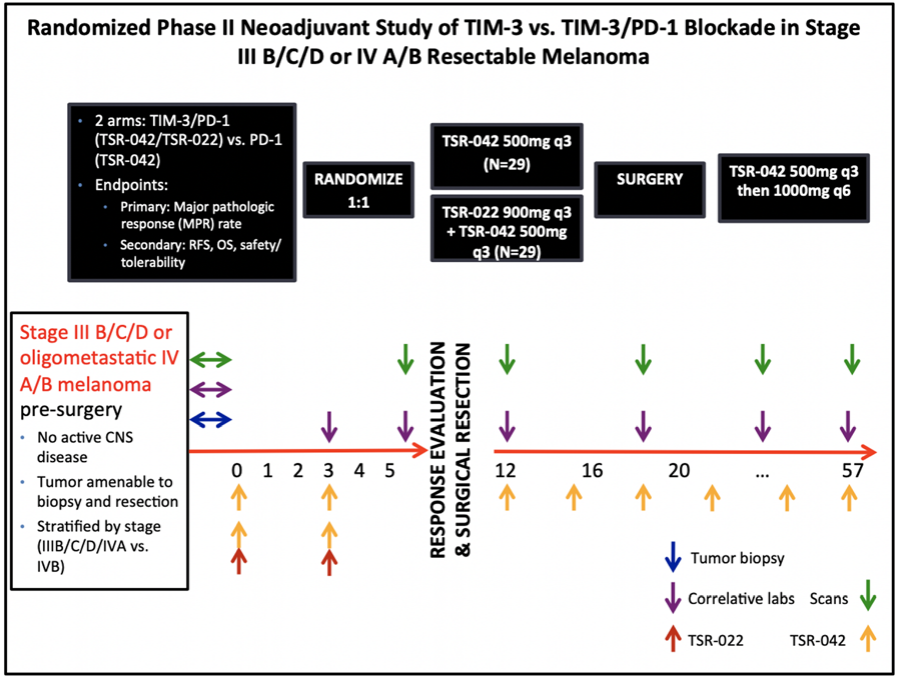
See text for description

#### P458 A phase I clinical trial on intratumoral administration of autologous CD1c (BDCA-1)+ myeloid dendritic cells plus talimogene laherparepvec (T-VEC) in patients with advanced melanoma

##### Julia Katharina Schwarze, MD, MSc, Gil Awada, Louise Cras, Inès Dufait, Ramses Forsyth, Ivan Van Riet, Bart Neyns

###### UZ Brussel, Brussels, Belgium

####### **Correspondence:** Julia Katharina Schwarze (juliakatharina.schwarze@uzbrussel.be)

**Background**

Intratumoral (IT) myeloid dendritic cells (myDC) play a pivotal role in initiating antitumor immune responses and re-licensing of anti-tumor cytotoxic T-lymphocytes within the tumor microenvironment. IT injection of the oncolytic virus T-VEC leads to the release of maturation signals and tumor antigens that can be captured and processed by IT co-administered CD1c (BDCA-1)+ myDC, reinvigorating the cancer immunity cycle.

**Methods**

Patients with advanced melanoma who failed standard therapy were eligible for IT injections of ≥1 non-visceral metastasis with T-VEC (10^6 PFU/mL; max total volume of 4 mL) on day 1 followed by IT injection of autologous, non-substantially manipulated CD1c (BDCA-1)+ myDC on day 2. Injection of T-VEC (10^8 PFU/mL; max total volume of 4 mL) was repeated on day 21 and every 14 days thereafter. Patients were treated with 0.5x10^6, 1x10^6, or 10x10^6 CD1c (BDCA-1)+ myDC in cohort-1, -2, and -3, respectively. Primary objectives were safety and feasibility. Repetitive biopsies of treated lesions were performed.

**Results**

In this ongoing trial, 2 patients were treated in cohort-1, 2 patients in cohort-2, and 3 patients in cohort-3. Patients received a median of 6 (range 3-10) injections of T-VEC. All patients are evaluable for response. The best overall tumor response (according to iRECIST) was a CR (pathologic CR) and one PR (confirmation pending; pathologic CR of treated lesions). Both patients were treated in cohort-3 and had previously progressed on anti-PD-1 checkpoint inhibition, and one patient also on anti-CTLA-4 therapy. Adverse events include G1 fever in 4 patients, G1-2 flu-like symptoms in 5 patients, transient G1-2 local pain and redness at the injection-site in 3 patients, and G1 gastrointestinal symptoms in 4 patients. The patient with CR developed an asymptomatic G3 eosinophilia during treatment; the patient with PR developed a transient purpuric rash at the site of skin metastases after the first treatment. Multiplexed immune-profiling (Ultivue) of baseline and on-treatment tumor biopsies is ongoing.

**Conclusions**

IT co-injection of autologous CD1c (BDCA-1)+ myDC with T-VEC is feasible and tolerable and resulted in encouraging early signs of anti-tumor activity in patients with immune checkpoint inhibitor refractory melanoma who received high dose CD1c (BDCA-1)+ myDC.

**Trial Registration**

ClinicalTrials.gov: NCT03747744

**Ethics Approval**

This study was approved by the Ethics Board of Universitair Ziekenhuis Brussel.

**Consent**

Written informed consent was obtained from the patient for publication of this abstract and any accompanying images. A copy of the written consent (NL,FR) is available for review by the Editor of this journal.

#### P459 New generation chimeric antigen receptor T-Cell Therapy (CoupledCAR) induces high rate remissions in solid tumor

##### Chengfei Pu, Lei Xiao

###### Innovative Cellular Therapeutics, Shanghai, China

####### **Correspondence:** Lei Xiao (xiaolei@ictbio.com)

**Background**

Conventional CAR T cell therapy showed weak CAR T expansion in patients, thus achieved no or little response for treating solid tumors. Here, we generated "CoupledCAR" T cells including an anti-TSHR CAR molecule. Compared with conventional CART cells, "CoupledCAR" T cells successfully improved the expansion of CART cells more than 100 times and enhanced CAR T cells’ migration ability, allowing the CAR T cells to resist and infiltrate the tumor microenvironment and killed tumor cells.

**Methods**

We designed a “CoupledCAR” lenti-vector containing a scFv targeting hTSHR. Patient‘s CD3 T cells were isolated and transduced with the lentivirus. Then,transduction efficiency was evaluated. After infusion, peripheral blood samples were collected to analyze expansion and cytokine release. The evaluation of response level for patients were performed at month 1,month 3,and month 6 by PET/CT.

**Results**

To verify the effect of “CoupledCAR” T cells on solid tumors, we have completed several clinical trials for different solid tumors, including two patients with thyroid cancer. Immunohistochemistry (IHC) results showed thyroid stimulating hormone receptors (TSHR) were highly expressed in thyroid cancer cells. In vitro co-culture experiments showed TSHR CAR T cells specifically recognized and killed TSHR-positive tumor cells. Animal experiments showed TSHR CAR T cells inhibited the proliferation of TSHR-positive tumor cells. Therefore, we designed "CoupledCAR" T cells expressing a binding domain against TSHR. Further,we did clinical trials of two group patients that were successfully treated using conventional TSHR CAR T cells and "CoupledCAR" T cells, respectively. In the group using conventional TSHR CAR T cells, patients showed weak cell expansion and less migration ability. In the group using TSHR "CoupledCAR" T cells, patients showed rapid expansion of CAR T cells and killing of tumor cells. One month after infusion (M1), the patient was evaluated as PR(Partial Response): the lymph node metastasis disappeared, and thoracic paratracheal tumors decreased significantly. Three months after infusion (M3), the patient was evaluated as a durable response, and the tumor tissue was substantially smaller than M1.

Further, two patients with colorectal cancer were enrolled in this trial and infused "CoupledCAR" T cells. One patient achieved PR and the other one achieved SD.

**Conclusions**

“CoupledCAR” T cells can effectively promote expansion, migration and killing ability of CAR T cells in patients with thyroid cancer. “CoupledCAR” T cell technology is a technological platform, which may be used to treat other cancer types. Next, we are recruiting more patients for clinical trials using “CoupledCAR” T cells.

#### P460 A randomized, placebo-controlled phase II study of multi-epitope TARP peptide autologous dendritic cell vaccination in men with stage D0 prostate cancer

##### Hoyoung Maeng, MD^1^, Lauren Wood, MD^3^, Seth Steinberg^1^, David Stroncek, MD^2^, Masaki Terabe, PhD^1^, Jay Berzofsky, MD, PhD^1^

###### ^1^National Cancer Institute, Bethesda, MD, United States; ^2^National Institute of Health, Bethesda, MD, United States; ^3^PDS Biotechnology, Berkeley Heights, NJ, United States

####### **Correspondence:** Hoyoung Maeng (hoyoung.maeng@nih.gov)

**Background**

TARP is a 58 amino acid protein expressed in normal and malignant prostate tissue [1]. TARP is highly expressed in 95% of prostate cancers including all Gleason types and in both castration sensitive (CSPC) and castration resistant prostate cancer (CRPC). In the pilot study of 1st generation TARP peptide-pulsed autologous dendritic cell vaccination (TARP DC vaccine, NCT00972309, N= 41) utilizing TARP WT 27-35 and epitope enhanced EE29-37-9V in HLA-A*0201 positive men with stage D0 prostate cancer (PSA biochemical recurrence) published by Wood et al, the vaccine was found to be immunogenic and safe [2]. TARP vaccination was also associated with a decreased slope log PSA in more than 70 % of the patients at 24 and 48 weeks and a decrease in calculated tumor growth rate constant. Standard of care in D0 prostate cancer ranges from watchful waiting, salvage radiation and anti-androgen therapy without strong evidence to support one or the other. In the current study of multi-epitope (ME)-TARP DC vaccines, 5 overlapping 18-20-mer peptides encompassing the full sequence of TARP are added to 1st generation TARP peptides to pulse the autologous DCs. The use of synthetic long peptides can increase the chance of a durable multi-valent anti-TARP response.

**Methods**

This is a single-blinded, randomized, placebo-controlled phase II study in men with Stage D0 prostate cancer. Men with a PSADT between 3 and 15 months will be randomized 2:1 to receive a ME TARP DC vaccine or a monocyte placebo. HLA restriction is not required. Patients will receive a total of 6 doses of vaccine or a placebo, 20e6 cells/dose intradermally every 3 weeks. Males older than 18 with adenocarcinoma and documented D0 prostate cancer whose PSADT is between 3 and 15 months are eligible. The participant should have normal organ functions, ECOG 0-1. Patient should not be on any other cancer treatment at enrollment. The primary objective is to assess the PSA slope log change at week 24 and 48 compared to baseline. Secondary objectives are 1) to assess the safety of ME-TARP DC vaccines 2) To characterize cellular and humoral immune responses associated with vaccination.

**Results**

The lead-in safety cohort (N=6) completed the treatment without DLT and we are currently enrolling the randomized arms (N=66).

**Conclusions**

Multi-Epitope TARP Peptide Autologous Dendritic Cell Vaccination in Men with Stage D0 Prostate is safe and open for further accrual in randomized arms to compare vaccine versus placebo.

**Acknowledgements**

This study is supported by the Center for Cancer Research, National Cancer Institute, National Institute of Health.

**Trial Registration**

NCT02362451

**References**

1. Wolfgang CD, Essand M, Vincent JJ et al. TARP: a nuclear protein expressed in prostate and breast cancer cells derived from an alternate reading frame of the T cell receptor gamma chain locus. Proc Natl Acad Sci U S A. 2000;97(17):9437–9442.

2. Wood LV, Fojo A, Roberson BD, et al. TARP vaccination is associated with slowing in PSA velocity and decreasing tumor growth rates in patients with Stage D0 prostate cancer. Oncoimmunology. 2016;5(8):e1197459.

**Ethics Approval**

The study was approved by National Cancer Institute Ethics Board, approval number P121083 and assigned a local number 15C0075.

#### P461 Phase 1b study of INCMGA00012, a programmed cell death-1 (PD-1) inhibitor, in combination with chemotherapy in patients with advanced solid tumors (POD1UM-105)

##### David Planchard, MD, PhD^1^, Jill Bowman^2^, Nawel Bourayou, MD^2^

###### ^1^Gustave Roussy Institute, Villejuif, France; ^2^Incyte Corporation, Wilmington, DE, United States

####### **Correspondence:** David Planchard (david.planchard@gustaveroussy.fr)

**Background**

Background: The combination of PD 1/programmed death-ligand 1 (PD-L1) checkpoint inhibitors with chemotherapy has proven clinically meaningful efficacy and manageable safety profile based on several randomized trials in treatment-naive advanced non-small cell lung cancer (NSCLC) [1,2,3] and encouraging data are emerging in unresectable advanced malignant pleural mesothelioma (MPM) [4]. INCMGA00012 is an investigational humanized immunoglobulin G4 (IgG4) monoclonal antibody against human PD-1. In the phase 1 POD1UM-101 study, INCMGA00012 has demonstrated acceptable tolerability with clinical activity observed in multiple tumor types, including a confirmed objective response rate (by Response Evaluation Criteria in Solid Tumors [RECIST] version1.1) of 19% in an interim analysis of a cohort of patients with platinum-refractory NSCLC [5]. The POD1UM-105 trial aims to investigate INCMGA00012 in combination with standard-of-care chemotherapy regimens in patients with advanced NSCLC or MPM.

**Methods**

Methods: POD1UM-105 is a phase 1b, global, multicenter study, in patients with histologically or cytologically confirmed advanced/metastatic NSCLC or unresectable MPM and regardless of PD-L1 expression, not previously treated with systemic therapy. Key eligibility criteria include no prior systemic treatment (except for patients [with known sensitizing mutations] who have disease progression on or following an approved targeted tyrosine kinase inhibitor, or chemotherapy completed > 6 months before enrollment), no prior checkpoint inhibitor therapy, measurable or nonmeasurable disease by RECIST version 1.1, and Eastern Cooperative Oncology Group performance status ≤1.

Patients will be assigned to 1 of 4 treatment groups (12-24 patients each), and will receive INCMGA00012 every 3 weeks for up to 2 years in combination with standard doses of chemotherapy agents (4 to 6 cycles) (Treatment Group A: gemcitabine/cisplatin; Group B: pemetrexed/cisplatin; Group C: pemetrexed/carboplatin; Group D: paclitaxel/carboplatin) (Figure 1).

The primary study objective is to evaluate safety, tolerability (DLTs), and determine a recommended phase 2 dose of INCMGA00012 in combination with chemotherapy. Secondary objectives include determining preliminary clinical activity (measured by objective response rate, duration of response, and disease control rate) and pharmacokinetics. Exploratory objectives include assessment of additional efficacy measures (progression-free survival and overall survival), and relevant biomarkers.

**Acknowledgements**

This study is sponsored by Incyte Corporation (Wilmington, DE).

**Trial Registration**

NCT03920839

**References**

1. Gandhi L, Rodriguez-Abreu D, Gadgeel S, et al. Pembrolizumab plus chemotherapy in metastatic non-small-cell lung cancer. N Engl J Med. 2018;378:2078-2092.

2. Langer CJ, Gadgeel SM, Borghaei H, et al. Carboplatin and pemetrexed with or without pembrolizumab for advanced, non-squamous non-small-cell lung cancer: a randomised, phase 2 cohort of the open-label KEYNOTE-021 study. Lancet. 2016;17:1497-1508.

3. Socinski MA et al. Atezolizumab for first-line treatment of metastatic nonsquamous NSCLC. N Engl J Med. 2018;378:2288-2301

4. Nowak AK, Lesterhuis WJ, Hughes BGM, et al. DREAM: a phase II study of durvalumab with first line chemotherapy in mesothelioma─first results. J Clin Oncol. 2018;36(suppl):8503

5. Mehnert JM, Joshua AM, Lakhani N, et al. First-in-human phase 1 study of INCMGA00012 in patients with advanced solid tumors: interim results of the cohort expansion phase. J Immunother Cancer. 2018;6(suppl 1):115. Abstract P669.

**Ethics Approval**

The study was approved by institutional review boards or independent ethics committees of participating institutions.

**Fig. 1 (abstract P461).**
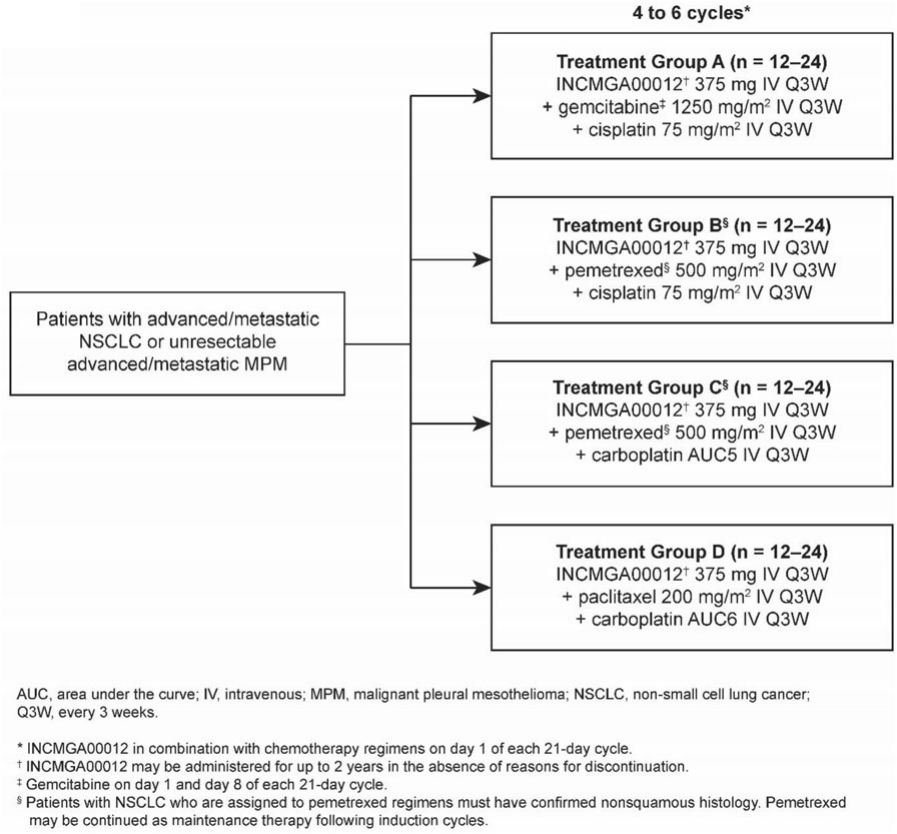
See text for description

### Combination Immunotherapies

#### P462 ImmTAC®-chemotherapy combination: A preclinical evaluation shows potential benefits

##### Filipa Bravo-Lopes, Nora Rippaus, Kristina Petrovic, Francesca Amicarella, Adam Taylor, Laure Humbert, Rupert Kenefeck, Adel Benlahrech, BSc PhD

###### Immunocore Ltd, Abingdon, United Kingdom

####### **Correspondence:** Rupert Kenefeck (rupert.kenefeck@immunocore.com); Adel Benlahrech (adel.benlahrech@immunocore.com)

**Background**

ImmTAC molecules are bispecific T cell redirectors comprised of an affinity‐enhanced TCR recognizing tumour antigen in the context of HLA molecules and a T cell engaging anti‐CD3 domain. Paclitaxel is a chemotherapy drug which stabilises microtubules and it is widely used in combination with platinum-based chemotherapies against multiple indications. Building on the recent evidence that chemotherapy positively combines with immunotherapy [1], this study was designed to evaluate the potential of combining Paclitaxel with ImmTAC-mediated T cell activation.

**Methods**

In vitro assays to assess T cell-mediated lysis, T cell expansion, and cytokine release in response to ImmTAC-mediated T cell redirection were performed with a range of ImmTAC concentrations and/or Paclitaxel. T cell-mediated lysis of antigen positive cells was monitored in real time while Granzyme A and B, IL-2, IL-8, IL-10, IFN-γ, TNF-α, MIP-1α, IP-10, and MIG were measured in cell culture supernatants. T cell expansion was measured by flow cytometry.

**Results**

ImmTAC molecules were highly effective at redirecting T cells to proliferate, produce pro-inflammatory cytokines, chemokines and granzymes, and to lyse antigen positive targets. ImmTAC-Paclitaxel combination showed substantially enhanced lysis of target cells compared to either agent alone (in both magnitude and kinetics). With 100 pM ImmTAC and 25 nM Paclitaxel, the median cytolysis area under the curve using the compounds in combination was 6269 compared to 3782 for Paclitaxel alone or 3196 for ImmTAC alone. Additionally, the time it took to lyse 80% of target cells decreased from 84 hours for ImmTAC alone to 51 hours when in combination with Paclitaxel. Despite enhanced killing, some reduction of cytokine release and T cell proliferation were observed when ImmTAC and Paclitaxel were combined. Notably, cytokine secretion and T cell proliferation were restored, and improved killing maintained by pre-treating target cells with Paclitaxel. Similarly, pre-treatment of effector T cells with Paclitaxel did not impact their ability to kill and proliferate in response to ImmTAC.

**Conclusions**

These in vitro studies show enhanced ImmTAC-mediated tumour cell lysis with Paclitaxel when administered either in combination with ImmTAC or sequentially. These data support the premise that Paclitaxel positively combines with ImmTAC and provide strong rationale for further clinical investigation.

**Reference**

1. Liu SV, Camidge DR, Gettinger SN, Giaccone G, Heist RS, Hodi FS, Ready NE, Zhang W, Wallin J, Funke R, Waterkamp D, Foster P, Iizuka K, Powderly J. Long-term survival follow-up of atezolizumab in combination with platinum-based doublet chemotherapy in patients with advanced non-small-cell lung cancer. Eur J Cancer. 2018;101:114-122.

**Ethics Approval**

The study was approved by the South Central - Oxford A Research Ethics Committee (UK), REC reference 13/SC/0226.

#### P463 Immunotherapy combinations for betel-nuts related HNSCC: one institutional experiences in Taiwan

##### Jo-Pai Chen, MD, Ruey-Long Hong, MD, PhD, Wei-Chen Lu, MD

###### National Taiwan University Hospital, Taipei, Taiwan, Province of China

####### **Correspondence:** Ruey-Long Hong (rlhong@ntu.edu.gov.tw)

**Background**

Betel-nuts chewing might contribute to (1) strong inflammation, invasion, and angiogenesis; (2) poor response to traditional therapeis. In the 1st line setting of R/M HNSCC, EPF offers survival benefit and immune checkpoint inhibitor(anti-PD1 monoclonal antibody), like nivolumab or pembrolizumab, has already brought survival benefit in the 2nd line treatment.

**Methods**

From 2016 to early 2019, 30 R/M HNSCC patients receiving immunotherapy-containing regimens in Yun-lin Branch of National Taiwan University Hospital were reviewed.

**Results**

These patients consisted of 1 HPV and 29 non-HPV; 18 pembrolizumab and 12 nivolumab; 10 with afatinib(6 pembrolizumab & 4 nivolumab); 5 with bevacizumab; 6 with chemotherapy. The objective response rate was 47%(14/30) and clinical benefit was 80%(24/30). 16 patients were still under use(4 afatinib with pembrolizumab; 4 afatinib with nivolumab). 1 patient under afatinib and pembrolizumab presented hyperprogression but then got pCR after bevacizumab combined with strong chemotherapy. 1 patient under low dose nivolumab in 20 mg biweekly with oral metronomic cyclophosphamide had mild tumor response. 1 patient under nivolumab with high dose ifosfamide developed nephritis. 1 patient had rapid skin metastasis over previous radiation fields after pembrolizumab, bevacizumab, and chemotherapy. 3 patients under afatinib & pembrolizumab developed autoimmune cholestasis(2 also with pneumonitis). Afatinib & nivolumab had similar efficacy but less toxicity. 10 pateints receiving afatinib combined with anti-PD1(8 faliling EPF, 7 with pleural/pericardial/skin metastasis. 5 rapid progression within 3 months after definite CCRT) had 70% response rate(7/10) and 90% clincial benefit(9/10). Post-progression use of anti-PD1 with other treatments were seen in 4 patients(esp. 1 with nivolumab & ipilimumab for sarcomatous change). 3 patients got benefits and had longer survival.

**Conclusions**

Immunotherapy-containing combinations are of clinical significance in refractory betel-nuts related HNSCC in Taiwan. Afatinib has several immuno-modulatory effects. In high risk patients(pleural/pericardial/skin metastasis failing EPF and rapid progression within 3 months after definite CCRT) in Taiwan, afatinib with anti-PD1 may be a good option to avoid hyperprogression. Earlier use, well biomarkers, best combinations, and optimal sequencing will be future goals.

#### P464 IFN-α and 5-Aza-2’-deoxycytidine enhance the anti-tumor efficacy of a dendritic-cell targeting MIP3α-Gp100-Trp2 DNA vaccine by affecting T-cell and Dendritic Cell recruitment into tumor

##### James Gordy, PhD, Richard Markham, BS MD

###### Johns Hopkins Bloomberg School of Public, Baltimore, MD, United States

####### **Correspondence:** Richard Markham (rmarkha1@jhu.edu)

**Background**

The chemokine MIP-3α (CCL20) binds to CCR6 on immature dendritic cells (DCs). DNA vaccines fusing MIP-3α to melanoma-associated antigens have shown improved efficacy and immunogenicity, compared to vaccines lacking the chemokine. To optimize the therapy, our laboratory has added agents designed to further enhance the T cell activating function of DCs and overcome immunoregulatory mechanisms of the tumor microenvironment. Here, we report that the combination of type-I interferon therapy (IFNα) with 5-Aza-2’-deoxycitidine (Aza) profoundly enhanced the therapeutic anti-melanoma efficacy of a MIP-3α-Gp100-Trp2 DNA vaccine, correlating with increases of T-cells and CD8α+ DCs

**Methods**

Utilizing the B16F10 syngeneic mouse melanoma model, vaccinations are administered by intramuscular electroporation with CpG adjuvant three times at one-week intervals beginning five days post lethal tumor implantation. Aza is given intraperitoneally at 1mg/kg, and IFNα therapy is given in a series of one high followed by three low doses, as noted. Tumor sizes, growth, and survival were all assessed. Tumor-infiltrating lymphocytes (TILs) were assessed by stimulating the purified lymphocyte fraction of tumors with vaccine antigens followed by intracellular cytokine staining flow cytometry. Dendritic Cells were assessed by flow cytometry.

**Results**

We demonstrate that the addition of IFNα and Aza treatments to mice vaccinated with the MIP-3α-Gp100-Trp2 vaccine led to significantly reduced tumor burden and overall increases in mouse survival, increasing median survival by 39% over vaccine. Importantly, this increase in efficacy was dependent on the presence of all three components, as vaccine plus IFNα or vaccine plus Aza did not differ significantly from vaccine alone. The addition of Aza and IFNα to the vaccine increased T-cell tumor infiltration, altered the proportion of CD8+T-cells, and increased CD8α+ DC infiltration.

**Conclusions**

Efficient targeting of antigen to immature dendritic cells with a chemokine-fusion vaccine offers a potential alternative approach to classic and dendritic cell-based vaccines currently undergoing clinical investigation. Combining this approach with IFNα and Aza treatments significantly improved vaccine efficacy, with efficacy correlating with changes in TILs and tumor infiltrating DCs. Further potential therapy optimization currently undergoing investigation offers promise for this line of investigation to become a novel melanoma therapy.

**Ethics Approval**

All procedures performed in studies involving animals were in accordance with the ethical standards of the IACUC of the Johns Hopkins University under Protocols #MO16H147 and MO19H319.

#### P465 Immune-mediated mechanisms involved in the synergistic anti-tumor efficacy of NHS-IL12 combined with the class I HDAC inhibitor entinostat

##### Kristin Hicks, PhD, Yohei Ozawa, MD, PhD, Karin Knudson, PhD, Jeffrey Schlom, Sofia Gameiro, PharmD, PhD

###### National Cancer Institute, NIH, Bethesda, MD

####### **Correspondence:** Sofia Gameiro (Sofia.Gameiro@nih.gov)

**Background**

Combining epigenetic agents with immunotherapy has shown clinical promise. Preclinically, we and others have shown that the class I histone deacetylase (HDAC) inhibitor entinostat can potentiate the anti-tumor efficacy of immunotherapies. Mechanistically, this is partially mediated by tumor MHC Class I upregulation, impairing regulatory CD4+ T cells (Tregs) and monocyte derived suppressive cells (MDSCs) and/or enhancing CD8+ T cells and granzyme B levels in the tumor microenvironment (TME). In these studies, we observed that entinostat induced murine tumor necrosis.

**Methods**

Therefore, we investigated if this treatment-induced necrosis could be targeted by NHS-IL12, an IL-12 NHS76 conjugate that binds free DNA in regions of tumor death/necrosis. NHS-IL12 has been shown to have significant anti-tumor efficacy in preclinical murine models and has been demonstrated to be safe in patients. We hypothesize that increasing the pro-inflammatory, immune stimulatory cytokine IL-12 in the TME will synergize with the entinostat-mediated immune effects to induce significant tumor control.

**Results**

In the EMT6 breast cancer murine model, administration of NHS-IL12 at the onset of entinostat-induced necrosis synergized to produce significant anti-tumor efficacy, including resolving well-established tumors and enhancing survival. These studies are now being extended to additional murine models of solid carcinomas. Examination of immune subsets in the TME by flow cytometry revealed that the combination increased infiltration of granzyme B+ CD8+ T cells and M1-like CD38 expressing tumor macrophages. Currently, we are using depletion studies to assess the relative contribution of these immune subsets to the anti-tumor efficacy. Further, we are examining the effect of this combination on CD8+ T cell function and macrophage polarization, phenotype, and function.

**Conclusions**

Overall, the combination of NHS-IL12 and entinostat is showing encouraging results in a preclinical solid tumor model, providing a rationale to examine this combination clinically.

**Acknowledgements**

The authors thank Curtis Randolph for excellent technical assistance.

#### P466 Carboplatin and paclitaxel after anti-PD1 or anti-PDL1 therapy: A retrospective study in patients with squamous cell carcinoma of the head and neck

##### Audrey Humphries, BS, Madeleine Welsh, BA, Alain Algazi, MD

###### UCSF, San Francisco, CA, United States

####### **Correspondence:** Alain Algazi (Alain.algazi@ucsf.edu)

**Background**

Historically, cytotoxic chemotherapy has been the first line standard of care for patients with RM-SCCHN. More recently, a randomized phase 3 clinical trial demonstrated improved overall survival in RM-SCCHN patients receiving pembrolizumab in the first line versus those receiving the prior standard carboplatin, 5FU, cetuximab. Additional studies have suggested that sequencing of therapies impacts outcomes. Several small case series have described clinical outcomes in patients treated with chemotherapy after immunotherapy, but these have often included patients treated with a variety of cytotoxic regimens such that comparison to historical controls is difficult. Here we present clinical outcomes in patients treated with carboplatin and paclitaxel immediately following disease progression on anti-PD1 or anti-PDL1 therapy.

**Methods**

A chart review was performed to identify all patients with RM-SCCHN, including tumors originating in the oral cavity, oropharynx, hypopharynx, larynx, and sinuses treated with an anti-PD1 or anti-PDL1 antibody immediately followed by carboplatin and paclitaxel through December 2018. Patients were required to have both pre-treatment and post-treatment imaging associated with treatment with both immunotherapy and chemotherapy or documented death from disease after administration of at least 1 cycle of chemotherapy. Anti-PD1 / PDL1 treatment history, p16 IHC (for OPC), and baseline PD-L1 IHC (where available) were assessed. The best overall response was assessed by RECIST v1.1 and PFS and OS were determined using the Kaplan-Meier method.

**Results**

A total of 11 patients meeting the inclusion criteria were identified including 5 patients with p16+ SCC of the oropharynx, and 3 with SCC of the oral cavity. 5 patient has detectable staining for PD-L1 at baseline, 2 did not, and 3 patients had no PD-L1 IHC available. 6 patients receive pembrolizumab alone or in combination, 4 received durvalumab, and 1 patient was treated with nivolumab. The median duration of prior PD1 / PDL1 exposure prior to chemotherapy was 2.7 months (range 1.4 to 18.2 months). The BORR to carboplatin and paclitaxel was 54.5% (6/11), the median PFS was 6.4 months (95% CI 3.7 to 9.2 months), and the median OS was 14.1 months (95% CI 3.2 to 25.0 months).

**Conclusions**

Our preliminary data suggest that carboplatin and paclitaxel often induce objective responses in patients with prior treatment with anti-PD1/PD-L1 antibodies and that the clinical outcomes compare favorably to those seen in checkpoint inhibitor naïve patients. If confirmed, these data suggest that there is no “opportunity cost” for administering immunotherapy in the first line.

**Ethics Approval**

Approval by the UCSF IRB has been requested and will be obtained before this work is presented.

#### P467 ALPN-202, a conditional CD28 costimulator and dual checkpoint inhibitor, enhances the activity of multiple standard of care modalities

##### Katherine Lewis, PhD, Mark Maurer, BS, Sherri Mudri, BS, Kayla Susmilch, MS, Fariha Ahmed-Qadri, MS, Chelsea Gudgeon, BS, Steven Levin, PhD, Martin Wolfson, BS, Stacey Dillon, PhD, Kristine Swiderek, PhD, Stanford Peng, MD, PhD

###### Alpine Immune Sciences, Seattle, WA, United States

####### **Correspondence:** Katherine Lewis (katherine.lewis@alpineimmunesciences.com)

**Background**

Checkpoint inhibitors targeting the PD-1 axis have transformed cancer treatment. However, objective response rates remain low, suggesting that novel therapeutics and/or combination treatments are needed. At the same time, non-immuno-oncology therapeutic approaches such as chemotherapy remain standard of care for many malignancies. ALPN-202 is a variant CD80 vIgD™-Fc fusion that mediates PD-L1-dependent CD28 costimulation and inhibits the PD-L1 and CTLA-4 checkpoints. This novel mechanism of action provides potent single agent immunomodulatory activity in mouse tumor models, and thus has the potential to complement other therapeutic modalities, such as checkpoint inhibitors or chemotherapies.

**Methods**

Mice were implanted subcutaneously with human (hu) PD-L1-transduced MC38 colon carcinoma and B16-F10 melanoma cell lines. Once measurable tumors were established, mice were treated with anti-mouse checkpoint (i.e. PD-1 or CTLA-4) blocking monoclonal antibodies (mAbs) or oxaliplatin, a platinum-based chemotherapeutic agent, alone or in combination with ALPN-202, to evaluate compatibility of the novel ALPN-202 protein with existing cancer therapies. Anti-tumor responses were evaluated by serial tumor volume measurements and RNA-Seq analysis of tumors isolated from treated mice.

**Results**

Anti-PD-1, anti-CTLA-4, or oxaliplatin alone were only modestly effective as monotherapy in huPD-L1+ MC38 tumor-bearing mice, while ALPN-202 has potent anti-tumor activity in this model. When the checkpoint inhibitors or chemotherapy were administered in combination with ALPN-202, significantly greater reductions in tumor growth over time were observed than with any of these agents alone (Figure 1 and Figure 2, respectively). Furthermore, ALPN-202 was extremely effective (92% tumor growth inhibition) in improving the anti-tumor activity of anti-PD-1 mAb in mice bearing huPD-L1+ B16-F10 tumors, a tumor that is known to be poorly immunogenic and treatment-recalcitrant (Figure 3). RNA-Seq analysis of tumors from the MC38 studies was performed to explore in-depth the mechanisms, including enhancement of T cell effector transcript expression, that play a role in the ability of ALPN-202 to provide anti-tumor immunity and to enhance the activity of checkpoint inhibitors and the chemotherapeutic oxaliplatin.

**Conclusions**

ALPN-202 demonstrates potent anti-tumor efficacy as monotherapy and significantly improves the anti-tumor activity of other only modestly effective treatment modalities, such as checkpoint-only blockade mAbs and chemotherapy. ALPN-202 has the potential to be significantly effective as a monotherapy, and its compatibility with checkpoint inhibitors and chemotherapeutics suggest versatility in its potential to improve outcomes in the frontline setting alone and/or in combination with standard of care of multiple cancer types. A first-in-human clinical study with ALPN-202 is in preparation.

**Ethics Approval**

The study was approved by the vivarium's International Animal Care and Use Committee (IACUC), IR# 17-01.

**Fig. 1 (abstract P467).**
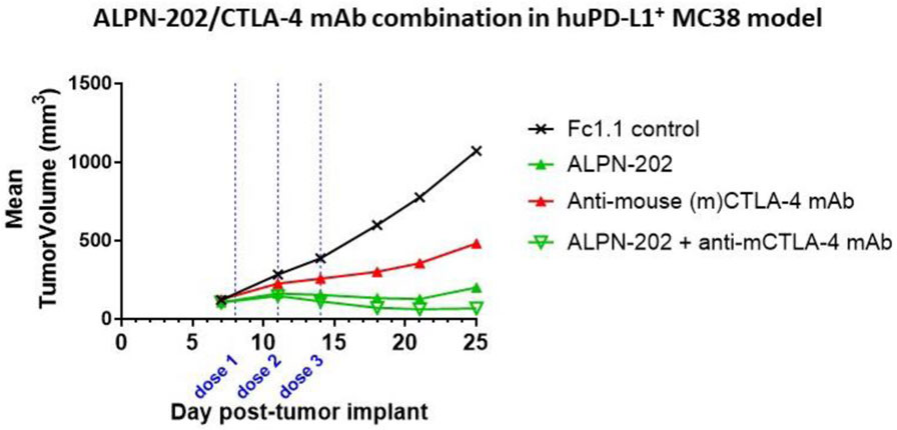
See text for description

**Fig. 2 (abstract P467).**
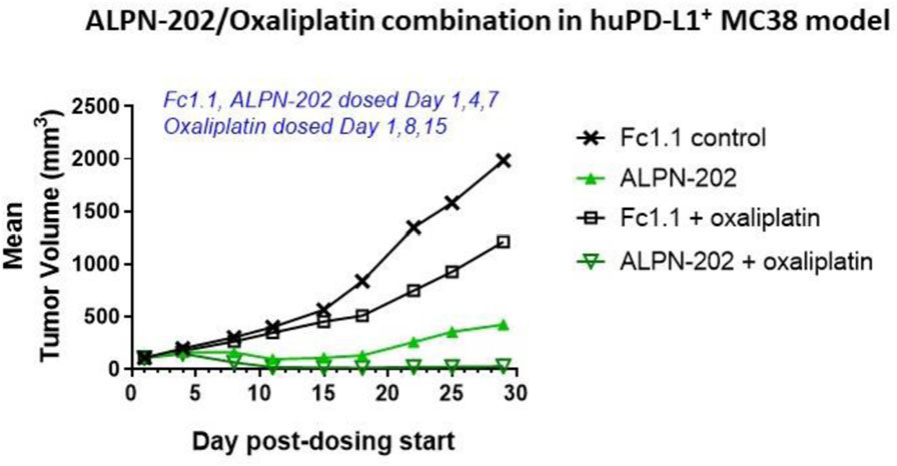
See text for description

**Fig. 3 (abstract P467).**
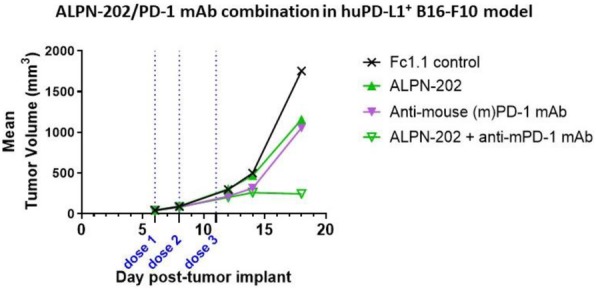
See text for description

#### P468 Targeting the ICOS pathway in combination with chemotherapy to enhance T-cell mediated anti-tumor immune responses

##### Tamer Mahmoud, PhD, Jeffrey Riggs, Madhu Ramaswamy, PhD, Leigh Hostetler, Brian Naiman, Ilyssa Ramos, Sean Turman, Fernanda Pilataxi, Christopher Morehouse, MD, Alex Alfaro, Norman Peterson, Ryan Fleming, Nazzareno Dimasi, Kyle Kuszpit, Ronald Herbst, Gianluca Carlesso, PhD

###### AstraZeneca, Gaithersburg, MD, United States

####### **Correspondence:** Gianluca Carlesso (carlessog@medimmune.com)

**Background**

ICOS (Inducible T-cell Costimulator) is a member of the CD28 superfamily that is detected on activated and memory T lymphocytes. Treatment with Immune checkpoint inhibitors (ICI) in clinical studies has shown that expansion of ICOS-expressing T cells is associated with positive patient outcome. Preclinical studies have validated the rationale for targeting ICOS and the clinical investigation of ICOS agonist antibodies (mAbs) combined with ICI is under way. To enhance tumor immunogenicity and increase the response rate to immunotherapy, we examined the efficacy of combination therapy of ICOS mAbs with standard of care chemotherapy using a colorectal tumor syngeneic animal model.

**Methods**

For the microarray analysis, freshly isolated normal primary human T cells were stimulated with sub-optimal concentration of anti-CD3 mAb with either ICOS, OX40 mAbs, or GITRL-FP for 4 hours. ¬In-vitro proliferation and cytokine secretion assays were performed using primary human T cells stimulated with anti-CD3 mAb and ICOS mAb in the presence of different inhibitors. For in-vivo studies, CT26 tumor cells were subcutaneously injected into BALB/c mice and ICOS or control mAbs were administered intraperitoneally (ip) or in combination with an intravenous injection of 5-Fluorouracil (5-FU) at day 10 post tumor implantation. For the biodistribution study, CT26 tumor-bearing mice were dosed ip with 89Zr-labeled anti-ICOS or control mAb and imaged using positron emission tomography (PET)/SCAN. For the depletion study, CT26 tumor-bearing mice were injected ip with either CD4 or CD8 mAb twice a week starting one day before tumor implantation, whereas sphingosine-1 phosphate receptor (S1PR) modulator (FTY720) was orally administered daily starting at day 9 post-implantation. Tumor growth was assessed three times a week.

**Results**

Compared to other T-cell agonists, in-vitro co-stimulation of human T cells with ICOS mAb lead to enhanced metabolic T cell reprogramming, activation of the PI3K/mTOR pathways, and secretion of IFN-gamma and IL-10. Immuno-PET scan imaging analysis revealed ICOS expression across tumor and secondary lymphoid tissues. ICOS mAb and 5-FU combination therapy on CT26 tumor-bearing mice resulted in a significant increase in anti-tumor responses compared to single-arm treatment or control groups. Moreover, the efficacy of either anti-ICOS or 5-FU monotherapy or combination required cytotoxic T cell activity and was dependent on the S1P-mediated egress of immune cells from peripheral lymph nodes.

**Conclusions**

Collectively, the results of these studies show that ICOS agonism in combination with chemotherapy may provide an effective therapeutic option for the activation of T cells in solid tumor indications.

#### P469 Chemotherapy enhances effector T cell responses to tumor associated antigens in human and mouse pancreatic cancer

##### Giorgia Mandili, PhD^1^, Claudia Curcio, PhD^1^, Sara Bulfamante, MS^1^, Laura Follia, MS^1^, Daniele Giordano, MD^1^, Rossella Spadi, MD^2^, Maria Antonietta Satolli, MD^2^, Paola Cappello, PhD MS^1^, Francesco Novelli, PhD^1^

###### ^1^University of Turin, Turin, Piedmont, Italy; ^2^Azienda Ospedaliera Universitaria Città della Salute e della Scienza di Torino, Turin, Piedmont, Italy

####### **Correspondence:** Francesco Novelli (franco.novelli@unito.it)

**Background**

Pancreatic Ductal Adenocarcinoma (PDA) is one of the most lethal cancer, both for lack of effective screening method and resistance to chemotherapy (CT). Immunotherapy (IT) trials with immune check-point inhibitors did not achieve significant gain of survival yet [1]. However, some CT agents, such as gemcitabine (GEM), have several immune modulating effects [2] and starting from the hypothesis that more immunogenic antigens can be induced by CT treatment, its ability to increase the susceptibility of PDA to IT was evaluated.

**Methods**

Sera from 28 PDA patients before and after CT (BCT and ACT respectively) were profiled by serological proteome analysis (SERPA) [3]; the recognized TAAs were identified by mass spectrometry and confirmed by ELISA. The proliferation, phenotype and cytokine production of T cells were evaluated on patients’ PBMCs from the same cohort after *in vitro* stimulation with four selected TAAs (ENO1, G3P, K2C8 and FUBP1). Mice that spontaneously develop PDA (KC) were treated with GEM prior of DNA vaccination against ENO1 [4]. Tumor lesions, immune infiltration and the titer of TAAs-specific antibody were evaluated. TAAs-specific IFNγ production from splenocytes was analyzed.

**Results**

The number of TAAs recognized by IgG in PDA patients’ sera, as well as their ability to induce a complement dependent cytotoxicity against PDA cells, was increased in ACT sera. Some identified TAAs showed a positive correlation between the increase of ACT antibody titer and longer patients’ survival. An increased T cell TAAs-specific proliferative response after CT and the evaluation of IFNγ/IL10 ratio was detected, revealing that ATC T cells shifted TAAs-specific responses from regulatory to effector one. After stimulation with TAAs, in mostly patients the ratio between CD8 and CD4 Treg cells was increased in ATC T cells. The role of CT to enhance the TAAs-specific adaptive response prompt us to exploit its effect in combination with the DNA vaccination. Of clinical relevance, KC mice treated with GEM prior of ENO1 DNA vaccination displayed smaller tumor lesions together with an increase of tumor-infiltrating CD4 and CD8, in comparison to mice vaccinated or GEM-treated only. Furthermore, CT increased specific antibodies and IFNγ-producing T cells in vaccinated mice not only against ENO1, the target TAA of vaccination, but also against G3P, suggesting an antigen spreading effect of the combinatory treatment.

**Conclusions**

Overall these data indicated that in pancreatic cancer CT effectively ameliorates T cell responses against TAA and it might be reconsidered to render them suitable targets for IT.

**References**

1. Brahmer JR, et al. Safety and activity of anti-PD-L1 antibody in patients with advanced cancer. N Engl J Med. 2012;366:2455–65.

2. Cappello P, et al. Next generation immunotherapy for pancreatic cancer: DNA vaccination is seeking new combo partners. Cancers. 2018;10(2):51

3. Tomaino B, et al. Autoantibody signature in human ductal pancreatic adenocarcinoma. J Proteome Res. 2007;6:4025–31.

4. Cappello P, et al. Vaccination with ENO1 DNA prolongs survival of genetically engineered mice with pancreatic cancer. Gastroenterology. 2013;144:1098–106.

**Ethics Approval**

The study was approved by the local research ethical committee (Azienda Ospedaliera Città della Salute e della Scienza di Torino, Turin) and investigations were performed according to the Helsinki Declaration principles.

#### P470 Impact of treatment-induced necrosis in the anti-tumor efficacy of Entinostat combined with the immunocytokine NHS-IL12

##### Yohei Ozawa, MD, PhD, Kristin Hicks, PhD, Karin Knudson, PhD, Jeffrey Schlom, Sofia Gameiro, PharmD, PhD

###### National Cancer Institute, NIH, Bethesda, MD, United States

####### **Correspondence:** Sofia Gameiro (Sofia.Gameiro@nih.gov)

**Background**

Tumor necrosis resulting from hypoxia, inflammation, or abnormal angiogenesis is associated with poor outcome in several solid malignancies. However, the role of treatment-induced necrosis is still controversial. The class I HDAC inhibitor entinostat (Syndax) has been shown to promote significant tumor control in combination with immunotherapies through immune-mediated mechanisms. In addition, we observed that entinostat induces tumor necrosis in murine solid tumors. We hypothesize that entinostat-induced necrosis could be targeted by NHS-IL12 (M9241, Merck KGaA), an IL-12 NHS76 conjugate designed to bind free DNA in regions of tumor death/necrosis, therefore promoting a synergistic anti-tumor effect. NHS-IL12 has demonstrated significant anti-tumor efficacy in preclinical solid tumor models and has been safely administered to cancer patients.

**Methods**

The amount of necrosis entinostat induced over time was examined in three distinct murine solid tumor models: MC38 (colon), 4T1 (triple-negative breast) and EMT6 (breast). Necrosis was evaluated by histological analysis of H&E staining and a terminal deoxynucleotidyl transferase dUTP nick end labeling (TUNEL) assay. To assess if NHS-IL12 could bind to necrotic tumor regions, tumor sections from mice treated with PBS or entinostat were incubated in vitro with NHS-IL12. The binding and distribution of NHS-IL12 in the tumor was then examined by immunofluorescence staining using an anti-human secondary antibody.

**Results**

Entinostat promoted tumor control in all three tumor models, albeit to different degrees. The onset of entinostat-induced necrosis was observed after two weeks of continuous dosing. In EMT6 tumors, areas of tumor necrosis identified via H&E staining also displayed DNA fragmentation measured by the TUNEL assay. Of note, in the entinostat-treated tumors there were areas of tumor death/necrosis with increased immune cell infiltration identified by H&E staining. Furthermore, initial immunofluorescence studies indicate that NHS-IL12 binds to these areas in the entinostat-treated tumors in vitro. These preliminary findings are being confirmed utilizing specimens from mice treated with all the agents.

Additionally, in the EMT6 model, entinostat and NHS-IL12 synergized to produce significant anti-tumor efficacy, including resolving well-established tumors and enhancing survival. The combination therapy promoted tumor infiltration of CD8 T cells and M1-like macrophages. Ongoing correlative studies in tumor specimens are examining the spatial distribution between entinostat-induced tumor necrosis, NHS-IL12 binding, and CD8 and M1 infiltration.

**Conclusions**

Overall, our results suggest that entinostat-induced necrosis may promote a tumor microenvironment conducive to NHS-IL12 binding, resulting in significant anti-tumor efficacy. These results inform a rationale to examine this combination in the clinic for patients with solid carcinomas.

**Acknowledgements**

The authors thank Curtis Randolph for his excellent technical assistance.

#### P471 Combination of BL-8040, anti PD-1 and chemotherapy significantly reduced pancreatic tumor growth and changed the balance between CD4+/FOXP3+ cells and CD8+ cells in the tumor

##### Amnon Peled, PhD (peled@hadassah.org.il)

###### Hadassah University Hospital, Jerusalem, Israel

**Background**

Cancer cells shape the tumor microenvironment (TME) to support their growth by recruiting immune suppressing cells such as T regulatory cells, as well as inhibiting the recruitment and activation of effector CD8+ T cells. In this study, we investigated the effect of combining the CXCR4 antagonist BL-8040 [1, 2], anti PD-1 immune checkpoint inhibitor and chemotherapy of Irinotecan, Fluorouracil and Leucovorin) IFL (on pancreatic tumor immune cell composition and growth.

**Methods**

The effect of BL-8040, anti PD-1 and IFL on tumor growth and immune cell constitution was assessed using the syngeneic Panc02 tumor mouse model. Tumors were established by s.c. injection . The accumulation of immune cells in the TME was assessed by immunohistochemical staining for CD8, CD4, Foxp3, and CD69.

**Results**

Treatment of tumors with anti PD-1 or BL-8040 alone, had no effect on tumor growth, whereas, IFL treatment had significant effect on tumor growth (67% inhibition). Combination of anti PD-1 + IFL, had no significant better effect on tumor growth compared to IFL (p<0.09), whereas, BL-8040 + IFL, had a significantly better effect on tumor growth compared to IFL (p<0.04). Moreover, IFL + BL-8040 + anti PD-1, had a highly significantly better effect on tumor growth, compared to IFL alone (p<0.004) (Figure 1). The triple combination treatment (TCT), further reduced tumor growth, compared to chemotherapy alone, by 58%. In the TCT, 3 out of 8 mice did not develop tumor at all, compared to 1 mouse that did not develop tumor in the IFL alone group. The TCT did not significantly change the number of CD8+ T cells accumulating in the tumor but increased their activation status (Figure 2A,B). Only in the TCT, the CD8+ T cells were larger in size inside the tumor parenchyma (p<0.01, Fig. 2C) and expressed CD69. Interestingly, we found that tumors treated with the TCT, had significantly reduced numbers of CD4+ and CD4+, Foxp3+ cells (Figure3).

**Conclusions**

TCT reduced significantly the number CD4+ and CD4+FOXP3+ cells and increased the numbers of activated CD8+, CD69+ cells in the TME. We hypothesize, that the ability of BL-8040 to modulate the TME may allow better activation of immune effector cells, contributing to the effect of chemotherapy and immunotherapy on tumor growth .A Phase IIa, multicenter, open label trial in patients with metastatic pancreatic cancer (the COMBAT study cohort II, [3]) is currently ongoing to assess the effect of the triple combination with BL-8040, +Pembrolizumab and ILF on disease progression.

**References**

1. Michal Abraham et al. Effect of BL-8040, high-affinity CXCR4 antagonist, on T-cell infiltration, tumor growth, and synergy with immunomodulatory agents.

Journal of Clinical Oncology, ASCO, 2017 35, e14544.

2. Pankaj Gaur et al. CXCR4 antagonist (BL-8040) to enhance antitumor effects by increasing tumor infiltration of antigen-specific effector T-cells. Journal of Clinical Oncology, ASCO, 2018, 36, 73.

3. Manuel M. Hidalgo et al. Evaluation of pharmacodynamic (PD) biomarkers in patients with metastatic pancreatic cancer treated with BL-8040, a novel CXCR4 antagonist.

Journal of Clinical Oncology, ASCO, 2018 36, 88-88.

**Ethics Approval**

The study was approved by the Hebrew University Ethics Board, approval number 18-15644-4.

**Fig. 1 (abstract P471).**
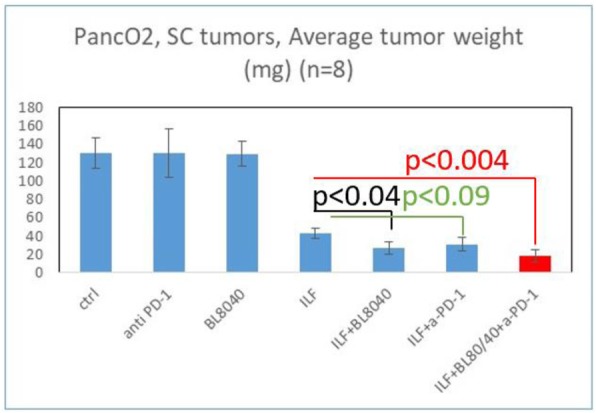
Tumor growth

**Fig. 2 (abstract P471).**
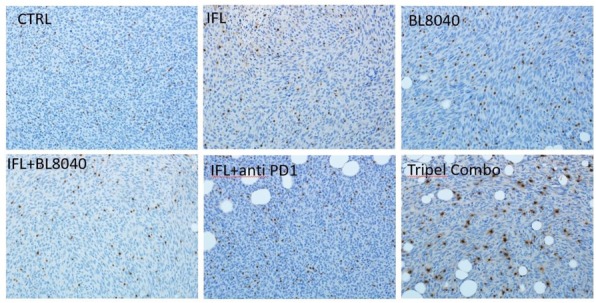
A, B, C. See text for description

**Fig. 3 (abstract P471).**
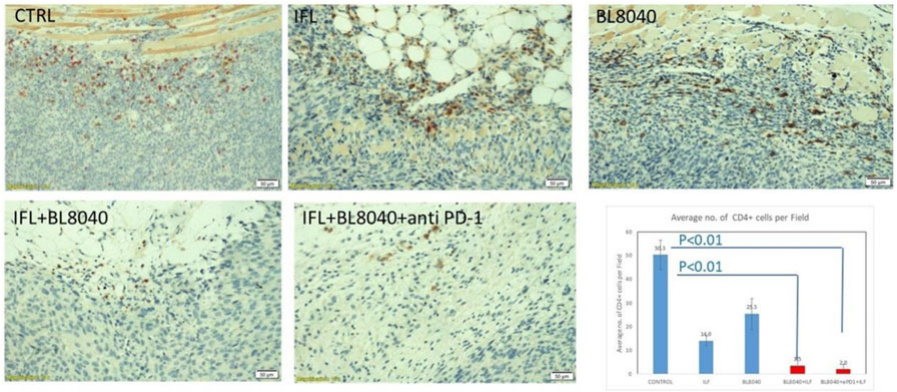
See text for description

#### P472 STAT3 ASO and Cisplatin combination sensitizes protected tumor microenvironments to checkpoint-mediated therapy

##### Theresa Proia, PhD, Maneesh Singh, PhD, Nanhua Deng, Minwei Ye, Frank McGrath, Douglas Ferguson, Simon Barry

###### AstraZeneca, Waltham, MA, United States

####### **Correspondence:** Simon Barry (simon.t.barry@astrazenea.com)

**Background**

Chemotherapy-immunotherapy (chemo-IO) combinations are being explored in the clinic. Immune responses mediated by PD-1 or PD-L1 inhibition may be enhanced by the immunogenic effects of cytotoxic agents, which can increase tumor antigens. Chemo-IO combination strategy relies on drug dose and schedule optimization, to minimize direct T cell killing with chemotherapy, enhance antigen presentation, and promote T cell activation.

Danvatirsen is a therapeutic antisense oligonucleotide (ASO) that selectively targets STAT3, a master regulator of immune suppression, and is currently in Ph 1/2 clinical trials in combination with an anti-PD-L1 antibody, durvalumab. In preclinical tumor models, we demonstrated that mouse surrogate STAT3 ASO remodels the suppressive tumor microenvironment to enhance cytotoxic T cell activity in combination with anti-PDL1 (Singh et al. SITC 2019).

**Methods**

We sought to maximize the therapeutic benefits of chemo-IO by dose and schedule optimization of adding cisplatin to STAT3 ASO and anti-PDL1. In patients, cisplatin dose ranges from 50mg/m2-100mg/m2. We modeled relevant preclinical doses in the range of 5-10 mg/kg based on mouse plasma exposure, and proceeded with 5 mg/kg to represent a low clinical therapeutic (~60mg/m2) dose.

Using the immunogenic MC38 model, various schedules of chemo-IO were explored; (1) Cisplatin priming on day 3 (to increase antigens), followed by STAT3 ASO/anti-PDL1 on day 7. (2) STAT3 ASO pre-treatment on day 3 (to remodel the suppressive tumor microenvironment), followed by cisplatin/anti-PDL1 on day 7. (3) All 3 agents dosed simultaneously on day 7 post implant.

**Results**

Cisplatin treatment in MC38-tumor bearing mice resulted in variable tumor growth inhibition, with one tumor regression. Flow cytometry analysis confirmed that cisplatin treatment had no detrimental effect on T cell number or functionality, and showed a trend of increased CD11b+/Ly6C+ dendritic cells, providing confidence to explore efficacy of the triplet combination.

Regardless of schedule, we observed 20% complete response rate in the triplet combinations, compared with 0 complete responses in any other treatment group; but interestingly, schedule 1 required dose holidays. Flow cytometry analysis of the triplet compared with vehicle revealed enhanced CD4 T cell functionality (1.6x increase IFNγ, p<0.001, and 1.2x increase IL-2, p=0.001), and enhanced NK functionality (1.3x increase Granzyme B+, p<0.01; 4.3x increase TNFα, p<0.001).

**Conclusions**

Collectively, we have generated data to support the combination of low-dose chemotherapy and immunotherapy to enhance the anti-tumor immune response. As chemo-IO combinations are being explored in the clinic, it will be important to optimize dose and schedule to minimize toxicity and maximize therapeutic benefit.

#### P473 Adoptive transfer of Deep IL-12 Primed T cells increases sensitivity to PD-L1 blockade for superior efficacy in checkpoint refractory tumors

##### Gulzar Ahmad, PhD^1^, Jonathan Nardozzi, PhD^1^, Lars Petersen, PhD^2^, Esben Christensen, MSc^2^, Ditte Jaegher^2^, James Suchy, PhD^1^, Karsten Sauer, PhD^1^, Douglas Jones, PhD^1^, Thomas Andresen, PhD^1^

###### ^1^Torque Therapeutics, Cambridge, MA, United States; ^2^Denmark Technical University, Kgs. Lyngby, Denmark

####### **Correspondence:** Thomas Andresen (tandresen@torquetx.com)

**Background**

While immune checkpoint blockade has revolutionized cancer care, many patients remain refractory to checkpoint inhibition. Converting checkpoint-refractory tumors into checkpoint-responsive tumors is a major challenge for cancer immunotherapy. Interleukin-12 (IL-12) is a potent cytokine that holds potential to reshape the immune environment in solid tumors. Its clinical utility, however, has been limited by severe toxicities both from systemic administration and from expression by adoptively transferred gene engineered T cells. We report here that adoptive cell therapy (ACT) with T cells carrying surface-tethered DeepTM IL-12 overcomes these challenges, enhances T cell therapeutic efficacy, activates immune cells in the tumor and overcomes resistance to checkpoint blockade.

**Methods**

Activity of Deep IL-12 Primed T cells were evaluated in B16-F10 melanoma tumors, a cell line resistant to checkpoint inhibition. Mouse PMEL CD8 T cells, which are reactive against the B16-F10 melanoma antigen gp100, were surface-tethered with Deep IL-12 and evaluated for anti-tumor activity in mice bearing B16-F10 melanoma. We additionally evaluated effects in the tumor of Deep IL-12 Primed T cells and PMEL T cells alone or co-administered with IL-12. Further studies evaluated combinations of Deep IL-12 Primed T cells with checkpoint inhibition.

**Results**

ACT of Deep IL-12 Primed PMEL T cells significantly improved tumor growth inhibition and overall survival of B16-F10 tumor bearing mice compared to PMEL T cells alone or combined with systemic co-administration of IL-12. In the tumor, Deep IL-12 repolarized immunosuppressive monocytic myeloid-derived suppressor cells (M-MDSC) into an immune-activating antigen-presenting cell (APC) phenotype. Consistent with an anti-tumor role for the repolarized M-MDSC, administration of an antibody to deplete these cells reduced the efficacy of Deep IL-12 Primed T cells. Further evaluation revealed high expression of the checkpoint ligand PD-L1 on the repolarized M-MDSC. To test the hypothesis that this limits efficacy of Deep IL-12 Primed T cell ACT, Deep IL-12 Primed T cells were co-administered with PD-L1 blockade. This further improved anti-tumor efficacy, resulting in durable long-term responders. Efficacy was further improved by repeat dosing of Deep IL-12 Primed PMEL T cells.

**Conclusions**

Our data demonstrates that ACT with tumor-specific T cells carrying surface-tethered Deep IL-12 repolarizes suppressive M-MDSC in the tumor, enhances anti-tumor efficacy, and synergizes with checkpoint inhibition in a checkpoint refractory cancer model. Torque is applying this approach to develop a novel adoptive cell therapy for cancer, Deep IL-12 Primed multi-targeted T cells (TRQ12-01), which is expected to start clinical evaluation in 2019.

#### P474 Efficacy and toxicity evaluation of anti-human CD47 and SIRPa antibodies in genetically humanized B-hSIRPa/hCD47 mice

##### Frank An, Yanan Guo, Jie Xiang, Chaoshe Guo

###### Biocytogen, Boston, MA, United States

####### **Correspondence:** Jie Xiang (info@biocytogen.com); Chaoshe Guo (info@Biocytogen.com)

**Background**

CD47 is a transmembrane protein expressed ubiquitously on the surface of human cells, including. SIRPa, one of the binding partners of CD47, is a member of the signal-regulatory-protein (SIRP) family which is expressed on many myeloid cells including phagocytic cells. Engagement of SIRPa by CD47 elicits the “do not eat me” signal to prevent phagocytosis of “self” cells by macrophages. Anti-CD47 and anti-SIRPa antibodies that interfere with the CD47-SIRPa interaction have demonstrated promise in clinical trials as new class of therapeutics. Despite such exciting potential, side effects associated with CD47/SIRPa blockade such as anemia may represent a significant toxicity concern. Therefore, evaluation of both efficacy and toxicity of human CD47/SIRPa antibody candidates emerges as one of most actively investigated areas in immuno-oncology in recent years. However, lack of animal models that enable expedient testing of anti-human CD47 or anti-human SIRPa antibodies in vivo has been a limiting factor for CD47/SIRPa antibody development.

**Methods**

To accelerate direct efficacy and toxicity testing of anti-human CD47 and SIRPa antibodies, Biocytogen has generated the double humanized mice, B-hSIRPa/hCD47, where the human extracellular domains of SIRPa and CD47 replace their respective murine counterparts. Homozygous B-hSIRPa/hCD47 mice express humanized but not the wild type mouse SIRPa and CD47. Further, we also created two triple humanized mouse strains, B-hPD1/hSIRPa/hCD47 and B-hPD-L1/hSIRPa/hCD47, where humanized PD1 and PD-L1 extracellular domains replace their mouse counterparts, respectively, in the B-hSIRPa/hCD47 background.

**Results**

We present here that B-hSIRPa/hCD47 mice were successfully used for screening anti-human CD47 and anti-human SIRPa antibodies for efficacy and toxicity in tumor models of the engineered MC38-hCD47 cell line that expresses human CD47 in MC38 cells. Anti-human CD47 and anti-human SIRPa antibodies were efficacious in controlling MC38-hCD47 tumor growth in B-hSIRPa/hCD47 mice. Varied toxicity profiles were observed in terms of body weight loss, blood cell counts, and blood liver enzyme levels in anti-human CD47 antibody treatments. Anti-human PD-1 and anti-human CD47 antibodies showed single agent and combination anti-tumor effect in B-hPD1/hSIRPa/hCD47 mice. So did anti-human PD-L1 and anti-human CD47 antibodies in B-hPD-L1/hSIRPa/hCD47 mice.

**Conclusions**

Taken together, we have validated three double and triple humanized CD47/SIRPa mouse models and demonstrate that these humanized mice are useful tools in facilitating development of therapeutics targeting human CD47/SIRPa.

#### P475 PD1 and LAG3 converge to limit polyfunctionality and systemic immunity

##### Lawrence Andrews, PhD^1^, Sasikanth Manne^2^, E. John Wherry, PhD^2^, Creg Workman, PhD^1^, Dario Vignali, PhD^1^

###### ^1^University of Pittsburgh, Pittsburgh, PA, United States; ^2^University of Pennsylvania, Philadelphia, PA, United States

####### **Correspondence:** Dario Vignali (dvignali@pitt.edu)

**Background**

Targeting PD1 has yielded clinical success across a variety of tumor types, yet a significant proportion of patients remain unresponsive to treatment. Thus, overcoming inhibitory receptor (IR)-mediated tolerance is essential to improve immunotherapeutic responses. Co-expression of PD1 and LAG3 on CD8+ tumor-infiltrating T cells (TIL) is associated with an exhausted phenotype, exemplified by a severe defect in cytokine production, cytolytic activity and inability to proliferate. In a number of murine tumor models, dual PD1/LAG3 blockade synergistically limits tumor growth greater than targeting PD1 alone, yet the relative and synergistic contributions of PD1 and LAG3 on CD8+ T cells in preventing effective anti-tumor immunity is unknown.

**Methods**

To understand the cellular and mechanistic basis for PD1/LAG3 synergy, conditional knockin mice “surgically dissect” Pdcd1 and/or Lag3 floxed alleles restricted to CD8+ T cells expressing E8ICre.GFP. To allow for intrinsic analysis of PD1 and/or LAG3 on antigen-specific CD8+ T cells, these mice have been generated as a pmel-1 transgenic background, with each mutant strain uniquely congenically marked for use in a co-adoptive transfer system allowing analysis of PD1 and/or LAG3-deficient CD8+ T cells, and controls, in the same host.

**Results**

Mice with CD8+ T cells deficient in PD1 or PD1 and LAG3 (Pdcd1L/L E8ICre.GFP and Pdcd1L/L Lag3L/L-yfp E8ICre.GFP, respectively) show attenuation of B16-F10 tumor growth with improved survival, compared to LAG3-deficient mice (Lag3L/L-yfp E8ICre.GFP) and controls (E8ICre.GFP). CD8+ TIL frequency is increased with loss of PD1, and further increased with loss of both PD1 and LAG3, as a result of enhanced proliferation (Ki67/BrdU) – a phenotype demonstrated to be intrinsically regulated in the co-adoptive transfer system. Although expression of TIM3, TIGIT and 2B4 IRs that normally co-express with PD1 are maintained, CD8+ TIL isolated from Pdcd1L/L E8ICre.GFP and Pdcd1L/L Lag3L/L-yfp E8ICre.GFP show increased functionality (IFNg, TNFa and GzmB release) by flow cytometry. Moreover, CD8+ TIL polyfunctionality is evident with PD1/LAG3 loss that was largely driven by effector and chemoattractive secretions by analysis with a 28-plex single-cell cytokine response panel (Isoplexis).

**Conclusions**

Overall, these data suggest that PD1 and LAG3 synergize to have a dominant effect on CD8+ TIL and limit antitumor immune effects, as intrinsic removal of both IRs results in reduced B16-F10 tumor growth which has a substantive impact on the development of systemic anti-tumor immunity. These results are encouraging for the continued development of LAG3 targeting agents in the clinic, which would hopefully yield improved clinical responses in combination with anti-PD1.

#### P476 The combination of a STING agonist with cytokines results in robust anti-tumor effects in autochthonous tumor models

##### Cristina Blaj, PhD^1^, Yingjoy Li^1^, Allen Chen^1^, Anthony Descien, PhD^2^, Brian Francica, PhD^2^, Sarah McWhirter^2^, Lora Picton^3^, K. Christopher Garcia^3^, David Raulet, PhD^1^

###### ^1^University of California Berkeley, Berkeley, CA, United States; ^2^Aduro Biotech Inc, Berkeley, CA, United States; ^3^Stanford University School of Medicine, Stanford, CA, United States

####### **Correspondence:** Cristina Blaj (cristina.blaj@berkeley.edu)

**Background**

Cancer immunotherapies based on immune checkpoint blockade are highly effective, but only in a limited number of tumor types. Even in transplanted preclinical models, monotherapy rarely results in cures. Moreover, preclinical subcutaneous models appear to be more responsive to therapies than preclinical carcinogen or genetically engineered mouse (GEM) autochthonous models of cancer, or human tumors. To extend immunotherapy to a broader range of tumor types, our strategy is to rationally design combination immunotherapies with the potential to boost innate and adaptive immune responses and overcome immunosuppressive environments characteristic of human tumors. Our regimen includes a stimulator of interferon genes (STING) agonist, cytokines and checkpoint inhibitors.

**Methods**

Autochthonous tumors induced by a carcinogen or in GEM models, as well as subcutaneous models derived from the GEM models, were treated with combination immunotherapies. We used flow cytometry, immunofluorescence, Luminex assays and qPCR to characterize the immune cell infiltration, activation status, receptor expression and secretion of cytokines in response to therapy.

**Results**

Intratumoral injection of cyclic dinucleotides (CDNs, specifically ADU-S100, a STING agonist) resulted in ~20% stable regressions of established transplanted tumors and immunity to re-challenge in a subcutaneous sarcoma model. The same protocol resulted in significant tumor growth delay in autochthonous GEM models and a carcinogen model. Antibody-mediated depletion studies revealed that natural killer (NK) cells as well as CD4 and CD8 T-cells played an important role in mediating the anti-tumor effects. The combination of CDNs and an IL-2 superkine resulted in synergistic anti-tumor efficiency in all models tested.

Autochthonous tumors are clinically more relevant, as they resemble the tumor resistance signature observed in human cancers. Our sarcoma models represent excellent platforms to dissect the differences between the refractory GEM models and the more responsive transplanted tumors. Flow cytometry and immunofluorescence analysis revealed the prevalence of tumor associated macrophages in the GEM models, consistent with the established role of macrophages in promoting an immunosuppressive environment. Additionally, we discovered a number of differentially secreted cytokines that might play a role in the anti-tumor response. The roles of macrophages and cytokines are being tested.

**Conclusions**

The combination of cyclic dinucleotides with an IL-2 superkine produced strong antitumor effects in transplanted and autochthonous tumor models and may translate into an efficacious approach to treat human cancers. Our studies provide indications for additional targets that might modulate the tumor microenvironment to enhance antitumor responses and synergize to enhance immunotherapy effects.

**Ethics Approval**

The study was approved by the University of California Berkeley Institutional Animal Care And Use Committee, approval number AUP-2015-1-8058-1.

#### P477 Isoform-specific blockade of active TGFb1 with mAb 13A1 enhances the efficacy of PD-L1 checkpoint therapy in a EMT6 mouse tumor model

##### Matteo Brioschi^1^, Jacques Van Snick, PhD^2^, Catherine Uyttenhove^2^, Pamela Cheou^3^, George Coukos, MD, PhD^4^, Gerd Ritter^5^, Steven Dunn, PhD^4^

###### ^1^UNIL/Ludwig Cancer Research - Lausanne, Epalinges, Switzerland; ^2^Ludwig Cancer Research - Brussels, Brussels, Belgium; ^3^University of Louvain, Brussels, Belgium; ^4^CHUV/Ludwig Cancer Research - Lausanne, Epalinges, Switzerland; ^5^Ludwig Cancer Research, New York, NY, United States

####### **Correspondence:** Steven Dunn (steven.dunn@chuv.ch)

**Background**

TGFb is a highly pleiotropic cytokine implicated in tumor escape and progression. Targeting TGFb has recently emerged as an exciting new approach to overcome TGFb-mediated resistance to checkpoint cancer immunotherapy [1]. In humans, three isoforms of TGFb (TGFb1, -2 and -3) have been shown to individually drive context-dependent physiological and phenotypic responses [2]. Most current therapeutic TGFb reagents do not adequately distinguish among the three TGFb isoforms and could give rise to on-target off-tumor toxicity, undesirable inflammatory adverse events or lack of activity. To address this unmet need for more selective therapeutic reagents, we generated a panel of murine antibodies with mono-isoform specificity for TGFb1 (13A1) or TGFb3 (1901) and successfully humanized these antibodies, carefully maintaining their selective specificity and neutralization potency [3]. The murine TGFb isoform-specific antibodies enhanced anti-tumor efficacy in-vivo in B16 melanoma and 4T1 breast cancer models [4]. We now expanded our in-vivo studies into the immune-exclusion-type tumor model EMT6, in which a pan-specific TGFb antibody was shown to overcome TGFb mediated resistance to PD-L1 checkpoint therapy [5].

**Methods**

Efficacy of the murine TGFb1 antibody 13A1 and a pan-TGFb antibody (1D11) in combination with anti-mPD-L1 were evaluated in established orthotopic EMT6 murine breast carcinomas. Antibody humanization was performed using molecular engineering, combining framework grafting, competitive screening and selective back mutations guided by assaying for TGFb neutralization potency in TMLEC reporter cells. Further humanized 13A1 variants with distinct kinetic properties were generated by error-prone mutagenesis and selective library screening.

**Results**

We found that isoform-specific neutralization of active TGFb1 with mAb 13A1 was highly efficacious in overcoming the low efficacy of PD-L1 checkpoint mono-therapy, enhancing control of tumor growth and increasing survival of mice with established EMT6 tumors. Further, 13A1 appeared at least as potent as the pan-TGFb antibody when combined with anti-PD-L1, highlighting the dominant role played by TGFb1 in this model. Based on these promising data, we have generated novel humanized 13A1 variants with in-vitro characteristics similar to the parental antibody.

**Conclusions**

Isoform-specific blockade of active TGFb1 with antibody 13A1 is as efficacious or better than pan-TGFb blockade in enhancing the anti-tumor efficacy of PD-L1 checkpoint therapy in mice with established EMT6 tumors. Humanized 13A1 antibodies that maintain the in-vitro features of the biologically active parental murine 13A1 antibody are therefore attractive clinical candidates for combination therapy with checkpoint antibodies, especially in patients with low response rates to current anti-checkpoint mono-therapies.

**References**

1. Neuzillet C, Tijeras-Raballand A, Cohen R, Cros J, Faivre S, Raymond E, de Gramont A. Targeting the TGFβ pathway for cancer therapy. Pharmacol Ther. 2015;147:22-31; Colak S. and Ten Dijke P. Targeting TGFb signaling in cancer. Trends Cancer 2017; 3(1):56-71.; Tauriello, D.V.D., Palomo-Ponce S., et al. TGFb drives immune evasion in genetically reconstituted colon cancer metastasis. Nature, 2018; 554:538-543

2. Poniatowski LA, Wojdasiewicz P, Gasik R, Szukiewicz D. Transforming growth factor beta family: Insight into the role of growth factors in regulation of fracture healing biology and potential clinical applications. Mediators Inflamm. 2015; 2015:137823

3. Uyttenhove C, Marillier RG, Tacchini-Cottier F, Charmoy M, Caspi RR, Damsker JM, Goriely S, Su D, Van Damme J, Struyf S, Opdenakker G, Van Snick J. Amine-reactive OVA multimers for auto-vaccination against cytokines and other mediators: perspectives illustrated for GCP-2 in L. major infection. J Leukoc Biol. 2011 Jun; 89(6):1001-1007 AND Brioschi M., Cheou P., van Snick, J., Uyttenhove C., Coukos, G., Ritter, G., Dunn, S. Journal for ImmunoTherapy of Cancer 2018, 6(Suppl 1):115 Abstr. Nr. 482

4. Gupta A, Budhu S, Giese R, van Snick J, Uyttenhove C, Ritter G, Wolchok J, Merghoub T. Isoform specific TGF-β inhibition in combination with radiation therapy as a novel immune therapeutic approach to cancer therapy. Journal for ImmunoTherapy of Cancer 2017; 5 (suppl 2):87 Abstract 326 AND Gupta A, Budhu S, Giese R, van Snick J, Uyttenhove C, Ritter G, Wolchok J, Merghoub T. Targeting specific TGF-β isoforms in combination with radiation therapy leads to differential antitumor effects in mouse models of cancer. Cancer Res. 2018; 78(13 suppl): Abstract 4716

5. Mariathasan S., Turley S.J. et al. TGFb attenuates tumor response to PD-L1 blockade by contributing to exclusion of T cells. Nature, 2018; 554:544-548

#### P478 SEMA4D antibody blockade overcomes mechanisms of immune suppression and combination immunotherapy including TGFβ blockade promotes efficient tumor regression

##### Terrence Fisher, PhD^1^, Crystal Mallow, BS^1^, Holm Bussler, PhD^1^, Sebold Torno, BS^1^, Desa Rae Pastore^1^, Alan Howell, MS^1^, Luis Ruffolo, MD^2^, Nicholas Ullman^2^, Brian Belt, JD^2^, Joe Bucukovski^1^, Christine Reilly, BS^1^, Benjamin Dale^2^, Ernest Smith, PhD^1^, David Linehan, MD^2^, Maurice Zauderer, PhD^1^, Elizabeth Evans, PhD^1^

###### ^1^Vaccinex, Rochester, NY, United States; ^2^University of Rochester, Rochester, NY, United States

####### **Correspondence:** Elizabeth Evans (eevans@vaccinex.com)

**Background**

Despite progress of immune checkpoint blockade therapies, resistance mechanisms including myeloid suppression and upregulation of TGFβ signaling prevent durable clinical benefit in many cancer patients. Anti-semaphorin 4D (SEMA4D, CD100) blocking antibody promotes immune infiltration, reduces immunosuppression, and enhances T cell activity in the tumor microenvironment (TME), resulting in increased tumor control in preclinical models when combined with various immunotherapies [1,2]. Clinical trials of immune checkpoint inhibitors (ICI) in combination with pepinemab (VX15/2503), a humanized anti-SEMA4D antibody, are currently underway in several cancer indications.

**Methods**

Activity of anti-SEMA4D antibody in combination with immune checkpoint inhibitors and TGFβ blockade was evaluated in preclinical mouse tumor models. Ongoing clinical trials of immune checkpoint inhibitors (ICI) in combination with pepinemab include: (i) a Phase 1b/2a combination trial of pepinemab with avelumab in ICI naïve or ICI refractory or relapsed NSCLC (CLASSICAL-Lung) (NCT03268057, N=65); (ii) neoadjuvant integrated biomarker trials in patients with metastatic melanoma (NCT03769155, n=36), metastatic colorectal, pancreatic (NCT03373188, n=32) and head and neck (NCT03690986, n=36) cancers treated with pepinemab in combination with nivolumab or ipilimumab. Gene expression and immunohistochemical analysis are employed to evaluate changes in immunophenotype as well as TGFβ-induced effects on TME and tumor progression.

**Results**

Anti-SEMA4D antibody enhanced tumor regression when combined with antibodies targeting CTLA-4, PD-1, PD-L1, LAG3, and TGFβ in several preclinical models. For example, anti-SEMA4D plus anti-TGFβ treatment resulted in maximal tumor growth delay (TGD) of 239% (p<0.01) and 10/15 complete tumor regressions (CR) (p<0.05), compared to 10% TGD and 0/13 CR with single agent anti-TGFβ or 29% TGD and 1/10 CR with anti-SEMA4D alone in MC38 colon carcinoma model. SEMA4D blockade reversed expression of genes related to EMT. Additionally, the combination of anti-SEMA4D, folfirinox, and ICI improved survival in KP2-tumor bearing mice, a KPC-derived pancreatic adenocarcinoma model of immune exclusion, myeloid suppression and active TGFb signaling. In clinical trials, pepinemab was well-tolerated and analysis of pre- and on-treatment biopsies revealed increased CD8:FoxP3 ratios and reduced presence of myeloid derived suppressor cells within TME.

**Conclusions**

SEMA4D antibody blockade modulates the TME to enhance anti-tumor immunity and combination therapies further enhance anti-tumor activity and overcome important resistance mechanisms. Preliminary data suggest the combination of pepinemab plus immune checkpoint therapy is well tolerated and shows initial signals of antitumor activity in patients. Ongoing analysis of various therapeutic combinations and immunophenotyping of tissue biopsies will shed light on mechanism of action of SEMA4D antibody blockade in several combination therapies.

**Acknowledgements**

We would like to thank the clinical and research teams at Emory University, including Doctors Greg Lesinsky, Christina Wu, Conor Steuer, Nabil Saba, Michael Lowe, Ragini Kudchadkar, and Brian Olson. We also extend gratitude to the Avelumab team at EMD Serono, as well as clinical investigators and their teams related to the CLASSICAL-Lung trial.

**Trial Registration**

NCT03268057

NCT03769155

NCT03373188

NCT03690986

**References**

1. Evans EE, et al. Antibody Blockade of Semaphorin 4D Promotes Immune Infiltration into Tumor and Enhances Response to Other Immunomodulatory Therapies. Cancer Immunol Res. 2015;3(6):689-701.

2. Clavijo PE, Friedman J, Robbins Y, Moore EC, Smith ES, Zauderer M, Evans EE, Allen CT. Semaphorin4D inhibition improves response to immune checkpoint blockade via attenuation of MDSC recruitment and function. Cancer Immunol Res. 2019;7(2):282-291

#### P479 Evaluation of a TNFR2 antibody with and without anti-PD-1 therapy in two murine colon cancer models

##### Katie Case, Lisa Tran, Hui Zheng, Michael Yang, Denise Faustman, MD, PhD

###### Massachusetts General Hospital/Harvard Medical School, Charlestown,, MA, United States

####### **Correspondence:** Denise Faustman (faustman@helix.mgh.harvard.edu)

**Background**

Tumor necrosis factor receptor 2 (TNFR2) is central to immune balance control in humans and mice. We created human-directed anti-TNFR2 antibodies as a therapeutic approach in cancer, with positive findings [1]. We have now identified a murine-directed surrogate antagonistic antibody to TNFR2. This antibody shares traits identified in our human-directed antibodies as critical to limiting regulatory T cell (Treg) expansion and activating T effector (Teff) cells. Here we present data on this surrogate anti-TNFR2 antibody in two syngeneic mouse models of colon cancer.

**Methods**

We studied the therapeutic effects of solo and combined immunotherapy using the murine-directed anti-TNFR2 antibody in CT26 and MC38 colon tumor models. We compared the new mouse surrogate anti-TNFR2 antibody therapy, a commercially available anti-PD1 therapy, and anti-TNFR2/anti-PD1 combination immunotherapy. Mice were dosed bi-weekly (100ug/mouse antibody). Antigen-specific CD8 and Treg infiltrates were also studied.

**Results**

In the CT26 model, anti-TNFR2 antagonism alone or co-treatment with anti-PD1 and anti-TNFR2 was highly efficacious (55-62% of mice cured). Anti-PD1 alone was less efficacious (25% cured). In the MC38 model, therapy with anti-TNFR2 alone showed some efficacy (20% cured) and anti-PD1 alone had the least efficacy (10% cured), but anti-PD1 in combination with anti-TNFR2 yielded the best overall survival (70% cured). Sequential antibody dosing with anti-PD1 followed by anti-TNFR2 yielded no synergy. In contrast, sequential treatment with anti-TNFR2 first followed by anti-PD1 or the combination of anti-TNFR2 plus anti-TNFRF2 showed synergy and highest efficacy. Anti-TNFR2 therapy was distinct from anti-PD1 therapy in showing pronounced regulatory Treg depletion and enhanced Teff infiltration in the tumor microenvironment, demonstrating in vivo specificity for disease-causing cells only in the tumor.

**Conclusions**

Anti-TNFR2 immunotherapy provides benefits in two colon cancer models, both as a single agent and when administered in combination with anti-PD1. Anti-PD1 before anti-TNFR2 was associated with poor outcomes for survival, histology and lack of long-term cure, suggesting that non-specific unleashing of the immune system with anti-PD1 destroys the tumor microenvironment specificity of anti-TNFR2. These results highlight the value of anti-TNFR2 antagonism in vivo in mouse tumor models as solo therapy or as a combination therapy, administered first or concurrently with anti-PD1. This study of new immunotherapy combinations highlights the need to test both single agent therapy as well as the sequencing of combination therapy as new agents are brought forward to the clinic.

**References**

1. Torrey H, Butterworth J, Mera T, et al.Targeting TNFR2 with antagonistic antibodies inhibits proliferation of ovarian cancer cells and tumor-associated Tregs. Sci Signal. 2017;10(462). pii: eaaf8608.

**Ethics Approval**

Mice were tested and monitored for tumor growth by either Champions Oncology (Hackensack, NJ) or a third-party pharmaceutical company in accordance with their animal welfare guidelines.

#### P480 Heterologous prime-boost vaccination safely enhances antitumor immunity to the colorectal antigen GUCY2C

##### John Flickinger, BS, Robert Carlson, Jagmohan Singh, Trevor Baybutt, BS, Elinor Leong, Alicja Zalewski, Amanda Pattison, Jeffrey Rappaport, Joshua Barton, Scott Waldman, Adam Snook, PhD

###### Thomas Jefferson University, Philadelphia, PA, United States

####### **Correspondence:** Adam Snook (adam.snook@jefferson.edu)

**Background**

The transmembrane receptor guanylyl cyclase C (GUCY2C) is an emerging target for colorectal cancer immunotherapy. Recently, an adenovirus-based vaccine against GUCY2C was tested in a phase I clinical trial where it was found to safely induce GUCY2C-specific immune responses [1]. However, GUCY2C immune responses following immunization wane over time and optimal GUCY2C immunity may require multiple GUCY2C vaccinations. Moreover, repeated vaccination utilizing adenovirus-based vectors is hindered by the production of adenovirus-specific antibodies following first vaccination. For this reason, we have generated a recombinant strain of Listeria monocytogenes secreting GUCY2C (Lm-GUCY2C) to boost GUCY2C immune responses. These studies assess the immunogenicity, therapeutic efficacy, and safety of a heterologous prime-boost immunization utilizing adenovirus and Listeria monocytogenes vectors.

**Methods**

T-cell responses following vaccination were assessed by IFNy ELISpot. Tumor protection following vaccination was assessed by challenging mice with a luciferase-expressing CT26 colorectal cancer cell line. Luminescence following luciferin injection and overall survival were quantified. Safety was assessed by histopathologic evaluation of known GUCY2C-expressing tissues following vaccinations.

**Results**

Construction of Lm-GUCY2C was validated by GUCY2C western blot on J774A.1 macrophage cells infected with Lm-GUCY2C. Optimal GUCY2C immunogenicity was achieved utilizing adenovirus-GUCY2C to ‘prime’ GUCY2C immune responses with Lm-GUCY2C to ‘boost’ and was found to be superior to homologous administration using either adenovirus-GUCY2C or Lm-GUCY2C vectors. Similarly, anti-tumor studies found heterologous administration of adenovirus-GUCY2C and Lm-GUCY2C to be superior to homologous administrations. Importantly, histopathologic evaluation of mice following heterologous prime-boost revealed no toxicity.

**Conclusions**

Heterologous prime-boost vaccination utilizing adenovirus and Listeria vectors expressing the tumor antigen GUCY2C demonstrate superior immunogenicity and antitumor efficacy over homologous immunization with either vector, a strategy that can be translated to colorectal cancer patients.

**Acknowledgements**

The authors thank the Center for Cell and Gene Therapy, Baylor College of Medicine for assistance in adenovirus vaccine manufacturing.

**Reference**

1. Snook AE, Baybutt TR, Xiang B, Abraham TS, Flickinger JC, Hyslop T, Zhan T, Kraft WK, Sato T, and Waldman SA. Split tolerance permits safe Ad5-GUCY2C-PADRE vaccine-induced T-cell responses in colon cancer patients. J. Immunother Cancer. 2019;7, 104.

**Ethics Approval**

Studies were approved by the Thomas Jefferson University IACUC (Protocol # 01956).

#### P481 Obesity impairs immunotherapeutic efficacy in pre-clinical breast cancer

##### Justin Gibson, BS, Rachael Orlandella, BS, William Turbitt, Robert Sorge, PhD, Lyse Norian, PhD

###### University of AlabamaBirmingham, AL, United States

####### **Correspondence:** Lyse Norian (lnorian@uab.edu)

**Background**

Obesity has long been known to worsen prognosis and survival for breast cancer patients. Recent reports further indicate that obesity negatively impacts response to targeted anti-VEGF therapy [1] and efficacy of chemotherapeutics [2,3]. However, no studies have yet investigated the impact of obesity on response to immunotherapy in the context of breast cancer. Although paradoxically, obesity has been found to improve response to immunotherapy in a subgroup of patients with melanoma [4,5].

**Methods**

Wildtype C57BL/6 female mice were randomized to a high-fat (60%) or low-fat standard chow (14%) diet for 16 weeks to generate diet-induced obese (DIO) or age-matched lean controls, respectively. Animals were then challenged with the syngeneic E0771 mammary carcinoma cell line. Tumor outgrowth was quantified by caliper measurements, bioluminescent imaging (BLI) via firefly luciferase-expressing E0771 (E0771-fLUC) cells, and endpoint tumor weights. Once tumors were palpable, animals were randomized to receive no therapy or immunotherapy consisting of intratumoral CpG co-administered with non-replicative adenovirus (Ad) encoding murine TNF-related apoptosis inducing ligand (TRAIL; AdT). Whole tumor immunogenetic gene expression profiles were evaluated using nanoString and immune populations were assessed via multi-parameter flow cytometry. T cell cytokine production was evaluated via flow cytometry following ex vivo CD3/CD28 stimulation.

**Results**

DIO mice had significantly increased body weights at tumor challenge versus lean controls (45 versus 25 grams, p <0.0001) All methodologies demonstrated that obesity significantly increases primary mammary tumor outgrowth and alters cellular and immunogenetic profiles within the tumor microenvironment. Notable alterations include significant reductions in the frequency of CD4+ T cells, CD8+ T cells, and CD19+ B cells; with a simultaneous increase in the frequency of myeloid-derived suppressor cells (MDSCs). Following immunotherapy administration, lean animals controlled tumor growth whereas DIO animals experienced progressive tumor growth. Despite these differential tumor outcomes, both lean and DIO animals displayed robust intratumoral effector CD8+ T cell accumulation and ex vivo function. In contrast, immunotherapy reduced the intratumoral accumulation of monocytic and granulocytic MDSCs only in lean animals. Both MDSC populations persisted in the tumors of animals with DIO, resulting in less favorable effector CD8+ T cell to MDSC ratios.

**Conclusions**

Our data implicate obesity as a causal factor in impairing immunotherapeutic efficacy in a pre-clinical model of breast cancer, potentially via accumulation of MDSCs. Our data suggest that clinical investigation and consideration is needed for factors such as body composition and body mass index when treating breast cancer patients with immunotherapy.

**Acknowledgements**

This study was supported in part by NIH-NIGMS training grant T32GM008111 to JTG.

**References**

1. Incio, J., et al., Obesity promotes resistance to anti-VEGF therapy in breast cancer by up-regulating IL-6 and potentially FGF-2. Sci Transl Med, 2018. 10(432).

2. Sheng, X., et al., Adipocytes Sequester and Metabolize the Chemotherapeutic Daunorubicin. Mol Cancer Res, 2017. 15(12): p. 1704-1713.

3. Lehuede, C., et al., Adipocytes promote breast cancer resistance to chemotherapy, a process amplified by obesity: role of the major vault protein (MVP). Breast Cancer Res, 2019. 21(1): p. 7.

4. McQuade, J.L., et al., Association of body-mass index and outcomes in patients with metastatic melanoma treated with targeted therapy, immunotherapy, or chemotherapy: a retrospective, multicohort analysis. Lancet Oncol, 2018. 19(3): p. 310-322.

5. Wang, Z., et al., Paradoxical effects of obesity on T cell function during tumor progression and PD-1 checkpoint blockade. Nat Med, 2019. 25(1): p. 141-151.

#### P482 STACT: A novel therapeutic platform that delivers immunomodulatory payloads to tumor-resident myeloid cells After IV dosing and demonstrates potent anti-tumor efficacy in preclinical studies

##### Laura Glickman, PhD, Christopher Rae, PhD, Alexandre Iannello, PhD, Anastasia Makarova, PhD, Haixing Kehoe, MS, John Faulhaber, Bill Hanson, Christopher Thanos, PhD

###### Actym Therapeutics, Inc, Berkeley, CA, United States

####### **Correspondence:** Christopher Thanos (cthanos@actymthera.com)

**Background**

Many experimental therapies developed to promote proper T-cell infiltration in immune-excluded tumors are too toxic for systemic administration, which will be required in a metastatic disease setting. These include innate targets such as STING and TLR agonists, co-stimulatory receptor agonists, and type I/II cytokine receptor combinations. To address these limitations, we have engineered a highly attenuated, microbial-based immunotherapy platform called STACT (S. Typhimurium Attenuated Cancer Therapy). Upon IV administration, the microbe traffics to and enriches in the tumor microenvironment. There, it is specifically phagocytosed and lysed by tumor-resident myeloid cells, enabling efficient delivery of plasmids encoding immunomodulatory payloads. Using our proprietary platform, we have generated multiple systemically-administered therapies that target several well-characterized, yet intractable immune pathways. Characterization of STACT microbes encoding constitutively active STING variants (STACT-STING) and IL-2 (STACT-IL2) are provided as examples.

**Methods**

The STACT platform strain has been engineered using precision genome modifications for enhanced tolerability, reduced immunosuppressive inflammation, and tumor specificity. STACT-mediated delivery of immunomodulatory proteins in primary mouse and human cells was confirmed by in vitro functional assays. STACT strains were evaluated in vivo for tumor-specific enrichment, payload delivery, tolerability, and therapeutic efficacy following IV administration in several subcutaneous syngeneic tumor studies.

**Results**

STACT was found to be 100,000-fold enriched in tumors, relative to spleen, after tail vein injections in mice. Flow cytometry staining revealed that STACT does not infect stromal or tumor cells and is specifically targeted by tumor-resident myeloid cells (TAMs, DCs, and monocytes). STACT is rapidly phagocytosed and then destroyed by these cells, delivering its plasmid DNA contents encoding immunomodulatory protein expression cassettes. We have measured highly efficient heterologous gene transfer and protein expression within primary mouse and human M2 macrophages treated with STACT, at levels comparable to DNA transfection. Therapeutically relevant levels of IL-2 were measured in the tumor microenvironment of STACT-IL2 treated mice several weeks after dosing. For STACT-STING, significant tumor growth inhibition, including complete tumor regressions were observed, and the therapy was well tolerated. Immune correlates were consistent with on-target expression in the tumor microenvironment, and the anti-tumor effect was adaptive immune mediated.

**Conclusions**

STACT is a highly attenuated, microbial-based therapeutic platform engineered to deliver immunomodulatory payloads, alone or in combination, to phagocytic cells of the solid tumor microenvironment after systemic administration. The goal of STACT therapy is to promote immune-mediated tumor clearance of T-cell excluded solid tumors and elicit durable anti-tumor immunity.

**Ethics Approval**

All animals were used according to protocols approved by an Institutional Animal Care and Use Committee and maintained in specific pathogen-free conditions in a barrier facility.

#### P483 TLR enhanced GVAX elicits tumor-specific tissue resident memory T cells independent of T cell priming

##### Michael Korrer, Young Kim, MD, PhD, David Taylor

###### Vanderbilt University Medical Center, Nashville, TN, United States

####### **Correspondence:** Young Kim (young.j.kim@vanderbilt.edu)

**Background**

GVAX, a genetically modified whole cell vaccine, is proposed to work by recruiting and activating antigen-presenting cells which then traffic to the draining lymph node and elicit tumor-specific T cells. In mouse models GVAX has little therapeutic benefit as a monotherapy, but when combined with a Toll-like receptor (TLR) 4 adjuvant, TLR Enhanced GVAX (TEGVAX) significantly reduces tumor burden. Paradoxically, the increased therapeutic benefit of TEGVAX corresponds with a decrease in delivery of tumor antigen to the draining lymph node. In order to improve the efficacy of the TEGVAX platform, it is critical to understand the mechanism by which it induces anti-tumor immune responses. Since TEGVAX requires T cells to work, yet significantly reduces antigen delivery to the draining lymph node, we hypothesize that TEGVAX functions independent of lymph node priming.

**Methods**

B16-mOVA cells injected s.c. into B6 mice. TEGVAX (1e6 B16-mOVA + 1e5 B78H1-GM Irradiated cells + 20ug MPLA) injected s.c. into opposite flank 5 days after tumor injection. 10ug daily FTY720 i.p. on day 4.

**Results**

To determine if TEGVAX alters the priming of CD8 T cells, we performed longitudinal studies of OT-1 CD8 T cell proliferation in vivo comparing TEGVAX to GVAX. We found that GVAX induced rapid proliferation of OT-1 cells, whereas TEGVAX failed to induce OT-1 cell proliferation as shown by FACS and in vivo imaging. We then determined if TEGVAX required myeloid or NK cells to reduce tumor burden. We found that TEGVAX reduced tumor burden in NK depleted, but not myeloid cell depleted mice. These results raised the question if priming in the draining lymph node was required for therapeutic efficacy of TEGVAX. To determine this, we performed tumor growth studies with mice administered FTY720, which sequesters circulating T cells in lymph nodes. We demonstrated that TEGVAX significantly reduced B16-mOVA tumor growth even in the presence of FTY720 treatment, suggesting T cell priming was not required. Immune phenotyping of TEGVAX treated mice, showed a significant increase in tumor infiltrating tissue-resident memory (Trm) T cells.

**Conclusions**

Our results demonstrate that combining TLR4 agonist with GVAX (TEGVAX) completely alters the immune response to vaccination. TEGVAX does not prime naïve T cells nor require trafficking of T cells from LN to tumor to function. We observed an increased number of Trm CD8 T cells infiltrating the tumor leading us to conclude that TEGVAX is functioning by eliciting a tumor-specific Trm T cell response independent of lymph node priming.

**Ethics Approval**

The study was approved by Vanderbilt University Animal care and use board

#### P484 CB-708, an orally bioavailable small molecule inhibitor of CD73 with immunostimulatory and anti-tumor activity

##### Clarissa Lee, PhD, Deepthi Bhupathi, Roland Billedeau, Jason Chen, Lijing Chen, Rosalyn Dang, Matthew Gross, Tony Huang, Weiqun Li, PhD, Yong Ma, Andrew MacKinnon, Gisele Marguier, MS, Silinda Neou, MS, Francesco Parlati, PhD, Natalija Sotirovska, MS, Sandra Spurlock, Timothy Stanton, Susanne Steggerda, PhD, Jing Zhang, Winter Zhang, Jim Li

###### Calithera Biosciences, South San Francisco, CA, United States

####### **Correspondence:** Jim Li (jli@calithera.com)

**Background**

High adenosine (ADO) in the tumor microenvironment suppresses the immune response against cancer cells by inhibiting immune effector functions and promoting the development of immunosuppressive cells. Extracellular ADO can be generated from ATP released by cells undergoing stress or death through the combined actions of the ectonucleotidases CD39 (ATP to AMP) and CD73 (AMP to ADO). Inhibition of ADO production via CD73 is a promising therapeutic approach for the treatment of cancer.

**Methods**

We developed CB-708, a potent and selective small molecule inhibitor of CD73. The potency of CB-708 was evaluated against recombinant CD73 and CD73-expressing cells using a malachite green assay. Selectivity against related ectonucleotidases was also assessed. Inhibition of CD73 in plasma was measured using LC/MS to assess conversion of 15N5-AMP into 15N5-ADO. Reversal of AMP-mediated immune suppression of human CD8+ T cells was determined by measuring T cell activation in the presence of exogenous AMP. T cell proliferation was assayed by flow cytometry and cytokine levels were measured by ELISA. The EG7 and CT26 syngeneic tumor models were used to assess the therapeutic effect of CB-708.

**Results**

CB-708 potently and completely inhibited soluble human CD73 (IC50 = 170 pM) and cell-bound human CD73 (IC50 = 210 pM), but did not inhibit human CD39, ENTPD2, or ENTPD3. CB-708 retained high potency in the presence of whole human plasma (IC50 = 380 pM) and reversed AMP-mediated suppression of human CD8+ T cell proliferation and production of IFNγ and granzyme B in vitro. Oral administration of CB-708 was well-tolerated in tumor-bearing mice, resulted in sustained exposure above mouse plasma IC50, and exhibited single-agent tumor growth inhibition in syngeneic tumor models including established EG7 tumors. Efficacy in the EG7 model was dependent on CD8+ T cells and was correlated with pharmacodynamic inhibition of CD73. Enhanced tumor growth inhibition was observed when CB-708 was combined with checkpoint inhibition (anti-PD-L1) or with chemotherapy (oxaliplatin, doxorubicin, docetaxel) in the EG7 model.

**Conclusions**

CB-708 is an orally bioavailable and highly potent small molecule inhibitor of CD73. CB-708 reverses the immunosuppressive effects of AMP-derived ADO in vitro and in vivo and has anti-tumor activity. CB-708 is expected to enter clinical development in 2019.

#### P485 Synergistic efficacy of anti-PD-L1/IL-15 fusion protein in combination with anti-CTLA-4 antibody in a murine orthotopic 4T1 breast carcinoma model

##### Stella Martomo, PhD^1^, Dan Lu, MA^2^, Jeegar Patel^2^, Zhanna Polonskaya^2^, Xenia Luna^2^, Kevin McCracken^2^

###### ^1^Kadmon Corporation, New York, NY, United States; ^2^Kadmon, New York, NY, United States

####### **Correspondence:** Stella Martomo (stella.martomo@kadmon.com)

**Background**

Administration of immune checkpoint inhibitors anti-PD1/PD-L1 have led to durable objective responses in select cancers. However, a substantial number of patients fail to respond or become resistant to these therapies. We have generated a therapeutic fusion protein (KD033) by combining a proprietary high affinity anti-human-PD-L1 (or anti-murine-PD-L1, (KD033-surrogate)) antibody with human IL-15. Initial assessment of this fusion antibody showed enhanced tolerability relative to a non-targeted IL-15 fusion protein in addition to its potent anti-tumor activity. In the CT26 murine colorectal tumor model, a single dose of KD033-surrogate consistently resulted in antitumor response that included tumor clearance and long-term tumor-free survival. Initial analysis of KD033-surrogate treatment showed robust adaptive and cytotoxic immune gene signatures in tumors leading to tumor inhibition and memory responses. We further analyzed tumors from KD033-surrogate responders and non-responders to evaluate possible therapeutic combinations to broaden the response of KD033.

**Methods**

CT26 tumor measurement seven days after treatment was used to define tumors as KD033-surrogate responders (decreasing tumor volumes), non-responders (no change or increasing tumor volumes) and non-targeted IL-15 best responders. RNA was isolated from these tumors and analyzed using the Nanostring PanCancer IO 360 Gene Expression Panel for immune cell responses. Combination therapy with genes identified through Nanostring analysis was evaluated in a tumor model where KD033-surrogate monotherapy showed minimal efficacy such as 4T1, an aggressive breast carcinoma murine model involving spontaneous metastases to other organs. 4T1 cells were injected into the mammary gland of Balb/c mice and grown to 100 mm3 prior to treatments. Tumors and metastasis nodules in the lung were evaluated.

**Results**

Transcriptional analysis showed that CTLA-4 was one of the top genes that was differentially upregulated after KD033-surrogate treatment in comparison to non-targeting IL-15. In the 4T1 tumor model, monotherapies of both KD033-surrogate or anti-CTLA-4 did not have any effect on 4T1 tumor growths; however, the combination therapy with single dose of KD033-surrogate and repeat dose of anti-CTLA-4 showed a decrease in the average number of lung metastases and a significant tumor growth inhibition compared to vehicle-treated animals.

**Conclusions**

Analysis of murine tumors treated with KD033 surrogate in vivo resulted in combination strategies, including KD033 in combination with CTLA-4, that can be exploited in targeting resistant and refractory cancers. Based on the therapeutic activity and improved safety of the fusion protein, Kadmon plans to initiate clinical studies of KD033 in 2019.

**Ethics Approval**

Animal studies were conducted for Kadmon by Crown Bioscience Inc. with approved SOP and IACUC protocol.

#### P486 Releasing the break on T cell activation through novel small molecule inhibition of HPK1

##### Minhui Shen, Gayathri Bommakanti, PhD, Deanna Mele, PhD, Neil Grimster, PhD

###### AstraZeneca, Waltham, MA, United States

####### **Correspondence:** Deanna Mele (Deanna.Mele@astrazeneca.com)

**Background**

Loss of immune surveillance is required for cancer cell growth and metastasis, tumors co-opt suppressive mechanisms to evade detection by the immune system. The immune system is equipped with multiple feedback mechanisms to limit inflammation in order to prevent autoimmunity, tumors activate these pathways to escape immunity. HPK1 (hematopoietic progenitor kinase 1) is a negative regulator of T cell activation, the kinase activity limits T cell signaling and tumoricidal cytokine production. Recent literature has shown inactivation of the kinase function of HPK1 prevents tumor progression in murine tumor models [1].

**Methods**

We used lentiviral delivered shRNAs and CRISPR/Cas9 technology to delete HPK1 in Jurkat cells and primary human T cells, respectively. Jurkat T cells (wt/KO) were activated with anti-CD3 antibody and cell lysates were assessed by western blot to examine signaling events downstream of the T cell receptor (TCR). Primary human T cells or CRISPR/Cas9 KO T cells were activated in vitro using anti-CD3/anti-CD28 antibodies in the presence or absence of HPK-1 inhibitors +/- prostaglandin E2 (PGE2). Cell viability and numbers were assessed by flow cytometry. Cytokines were quantified by ELISA.

**Results**

We present novel findings demonstrating that the role of HPK1 is conserved in human Jurkat cells as well as in primary human T cells. Loss of HPK1 enhanced T cell receptor signaling in Jurkat cells. In CRISPR/Cas9 KO HPK1 primary human T cells, TCR activation resulted in enhanced cytokine secretion and proliferation concomitant to a decrease in pSLP76, the target molecule phosphorylated by HPK1. Consistent with the KO phenotype, our HPK1 inhibitors resulted in enhanced T cell activation, cytokine secretion and proliferation. In addition, the inhibitors were able to rescue T cells from PGE2 mediated suppression. In vivo studies are currently underway to examine anti-tumor activity of these compounds in various syngeneic models.

**Conclusions**

In summary our small molecule inhibitors of HPK1 could enhance anti-tumor immunity through increased T cell function overcoming suppressive signals in the tumor microenvironment and thus broaden the response to check point inhibitors for cancer immunotherapy.

**Acknowledgements**

Minhui Shen1, Gayathri Bommakanti1, Kevin Xu1, Kun Song1, Rob Ziegler1, Jason Kettle2, Adelphe Mfuh1 Jason Sheilds2, Neil Grimster1, Lisa Drew1, Stephen Fawell1, Deanna A. Mele1

1AZ Discovery Early Oncology, Boston, MA, 2AZ Discovery Early Oncology, Cambridge UK, MA

**Reference**

1. Hernandez S, Qing J et al. The Kinase Activity of Hematopoietic Progenitor Kinase 1 is Essential for the Regulation of T cell Function Cancer. Cell. 2018; 25: 80-94.

#### P487 Combining an engineered costimulatory vaccine with NK cells induces an anti-tumor effect against murine neuroblastoma in vitro and after bone marrow transplant in vivo

##### Nicholas Mohrdieck, BS, Paul Bates, Sean Rinella, Katharine Tippins, Christian Capitini, MD

###### University of Wisconsin-Madison, Madison, WI, United States

####### **Correspondence:** Christian Capitini (ccapitini@pediatrics.wisc.edu)

**Background**

High risk neuroblastoma remains a challenge to cure with only 50% survival, despite multi-modality treatment. Natural killer (NK) cells have been previously shown to have activity versus neuroblastoma but have not been consistently successful in clinical trials. NK cell activation via co-culture with a vaccine engineered to express CD54, CD80, CD86, and CD137L, called AgN2a 4P, was studied to investigate NK cells’ ability to induce cytotoxicity of murine neuroblastoma tumor cells in vitro and in vivo.

**Methods**

NKs and irradiated AgN2a 4P were co-cultured in ratios of 1 (NKs):0.5 (AgN2a 4P) and 1:1, and compared to NK only and AgN2a 4P only controls, with all groups receiving IL-15/IL-15Ralpha, and then analyzed by flow cytometry, multiplex cytokine analysis, and cytotoxicity in vitro after 1, 3, 5, 7, and 9 days. To study the efficacy of in vivo vaccination with AgN2a 4P after bone marrow transplant (BMT), C57BL/6 or B6AJ recipients were lethally irradiated, followed by transplantation of T-cell depleted C57BL/6 donor bone marrow on day +0. BMT recipients were then treated with the AgN2a 4P vaccine for 2 versus 3 weekly doses, and with or without adoptive transfer of donor NK cells to accelerate immune reconstitution. All recipients were then challenged with NXS2 neuroblastoma tumor, and followed for tumor growth and survival.

**Results**

The NK:AgN2a 4P co-culture at 1:0.5 and 1:1 increases Ly49A+ NKs from 6% to 21%, and Ly49D+ NKs from 3% to 30% from day +0 to day +9. pSTAT1 activation remains consistently high between 80%-98%, and pSTAT3 activation remains 20%-50%, across the co-culture period. NK cells release increased levels of IFN-gamma and IL-6 at the co-cultured ratios of 1:0.5 and 1:1, and CXCL1 at the 1:1 ratio, as compared to the NK with IL-15/IL-15Ralpha controls. The NK:AgN2a 4P ratios of 1:0.5 and 1:1 induce significantly increased apoptosis of Neuro2a neuroblastoma cells than NK cells with IL-15/IL-15Ralpha alone. In vivo, injection of 2 doses of AgN2a 4P vaccine leads to 100% lethality by 60 days, but 3 doses of AgN2a 4P leads to 100% survival at 50 days after both allogeneic and syngeneic BMT.

**Conclusions**

Co-culture of NK cells with an engineered costimulatory vaccine is an effective strategy to induce apoptosis of neuroblastoma tumor cells by increasing NK-mediated cytokine production and cytotoxicity, and enhances anti-tumor effects after BMT. Usage of cell-based vaccines after BMT could be an effective strategy to augment NK cell activity against neuroblastoma.

**Acknowledgements**

AgN2a 4P was a gift from Dr. Bryon Johnson at Medical College of Wisconsin. This work was supported by grants from the St. Baldrick’s – Stand up to Cancer Pediatric Dream Team Translational Research Grant SU2C-AACR-DT-27-17, NCI/NIH R01 CA215461, American Cancer Society Research Scholar grant RSG-18-104-01-LIB, Hyundai Hope on Wheels and the MACC Fund (C.M.C). We would like to thank the UWCCC core facilities, who are supported in part through NCI/NIH P30 CA014520. Stand Up to Cancer is a division of the Entertainment Industry Foundation. Research Grants are administered by the American Association for Cancer Research, the Scientific Partner of SU2C.

**Ethics Approval**

The study was approved by University of Wisconsin-Madison Animal Care and Use Committee, approval number M005915.

#### P488 IPH5201, a blocking antibody targeting the CD39 immunosuppressive pathway, unleashes immune responses in combination with cancer therapies

##### Pascale Andre^1^, Ivan Perrot^1^, Caroline Denis, PhD^1^, Marc Giraudon-Paoli^1^, Severine Augier^1^, Rachel Courtois^1^, Diana Jecko^1^, Thomas Arnoux^1^, Violette Breso^1^, Nicolas Gourdin, PhD^1^, Nadia Luheshi, PhD^2^, Ariane Morel, PhD^1^, Yannis Morel, PhD^1^, Eric Vivier^1^, Carine Paturel, PhD^1^

###### ^1^Innate Pharma, Marseille, France; ^2^AstraZeneca, Milton, Cambridge, United Kingdom

####### **Correspondence:** Pascale Andre (pascale.andre@innate-pharma.fr)

**Background**

CD39 is an extracellular ectonucleotidase highly expressed in the tumor microenvironment, by stromal cells and some immune infiltrating cells. CD39 contributes to the production of adenosine, an inhibitor of immune response, via sequential hydrolysis of adenosine triphosphate (ATP) and adenosine diphosphate into adenosine monophosphate, which then is degraded into adenosine by CD73 enzyme. In contrast, ATP has immune-stimulatory activity through promoting dendritic cell (DC) maturation. Blockade of CD39-mediated degradation of ATP may therefore stimulate anti-tumor immunity across a wide range of tumors by preventing production of immunosuppressive adenosine and by promoting accumulation of immunostimulatory ATP in the tumor microenvironment. IPH5201 is a humanized monoclonal antibody that selectively binds to and inhibits the activity of both membrane-bound and soluble human CD39. Here, we explored the efficacy of IPH5201 in vitro and in vivo in immunocompetent human CD39 knockin (huCD39KI) mouse model in combination with immune checkpoint inhibitor.

**Methods**

In vitro, efficacy of IPH5201 was evaluated (1) on the phenotypic changes and stimulatory potential of monocyte-derived DC, (2) on the inflammasome pathway by assessing interleukin-1b secretion from in vitro-derived M1 macrophages, and (3) on T cell proliferation. HuCD39KI mice were characterized for the expression and function of human CD39. To assess CD39 blockade in vivo, a mouse IgG1 version of IPH5201 was produced (moIPH5201), which contained key point mutations in the Fc region to abrogate Fc receptor interactions. Antitumor efficacy of CD39 blockade was assessed in huCD39KI mice grafted with mouse tumor cells not expressing mouse CD39. HuCD39KI mice were treated with blocking anti-human CD39 Ab, either alone or in combination with a blocking anti-mouse PD-L1 Ab.

**Results**

As hypothesized, in vitro IPH5201 enhanced the phenotypic maturation and the activation of DCs and macrophages by inhibiting ATP hydrolysis. IPH5201 also efficiently restored T cell proliferation in a dose-dependent manner to the levels observed in the absence of ATP addition. Thus, IPH5201 preserved extracellular ATP, thereby promoting the activation of DCs and macrophages and limiting adenosine accumulation and its immunosuppressive effect on T cells. Furthermore, moIPH5201 mice treatment efficiently inhibits membrane and soluble human CD39, from huCD39KI mice ex vivo without mediating CD39-expressing cell depletion in vivo. Finally, blockade of CD39 potentiates the anti-tumor efficacy of anti-PD-L1 Ab monotherapy.

**Conclusions**

Together these data indicate that blocking CD39 in conjunction with PD-1/PD-L1 checkpoint inhibitors provides increased anti-tumor efficacy and support the rational for assessing this combination in clinical trials.

#### P489 Identification and characterization of MCLA-145 (CD137 x PD-L1): a bispecific antibody that requires PD-L1 binding to activate CD137

##### Simon Plyte^1^, Cecile Geuijen^1^, John de Kruif^1^, Pieter Fokko van Loo, PhD^1^, Paul Tacken^1^, vanessa Zondag-vander Zande^1^, Rinse klooster^1^, hans van Maaden^1^, Erik Rovers^1^, steef engels^1^, floris franzen^1^, abdul basmeleh^1^, willem bartelink^1^, Mark Throsby^1^, Patrick Mayes^2^, Horacio Nastri^2^, shaun stewart^2^, jing zhou^2^, steve wang^2^, Chen-yen Huang^2^, thomas codamine^2^, ashwini kularni^2^, yao bin lui^2^, arpita mondal^2^, leslie hall^2^, soeon kim^2^, marina martinez^2^, shaun o'brien^2^, edmund moon^2^, steven albelda^2^

###### ^1^Merus, Utrecht, Netherlands; ^2^Incyte Research Institute, Wilmington, DE, United States

####### **Correspondence:** Simon Plyte (simon.plyte@merus.nl)

**Background**

CD137 (4-1BB) is costimulatory receptor on T and NK cells that requires clustering to elicit its effects on target cells and enhance adaptive immune responses against tumors. The development of CD137 targeted agents for cancer therapy has been hampered by on-target off-tumor toxicity in the case of agonistic monospecific antibodies, or limited antitumor activity in the case of Fcγ-mediated crosslinking of mAbs.

**Methods**

To address the issues of toxicity and efficacy, we have identified a highly selective and potent CD137xPD-L1 bispecific antibody, MCLA-145. Collections of common light chain Fabs recognizing CD137 and PD-L1 were produced based on antibody panels from immunized MeMo® mice. Unbiased, combinatorial, functional screening was then performed on a large and diverse panel of CD137xPD-L1 bAbs to identify those for which CD137 mediated activation is dependent on the presence of PD-L1 on a neighboring cell

**Results**

Both the CD137 and PD-L1 Fab arms block the interaction with their respective ligands as demonstrated in competition flow cytometry or ELISA assays, respectively. MCLA-145 drives transactivation of CD137 in the vicinity of cells expressing PD-L1 and the degree of CD137 agonistic activity in T cells correlated with the expression level of PD-L1 on neighboring cells. CD137 signaling was induced by MCLA-145 in multiple primary human immune cell assays and reversed T cell suppression mediated by M2 macrophages or Tregs, in vitro. In one humanized mouse tumor model, human T cells expressing NY-ESO specific TCR were adoptively transferred to mice bearing A549 tumors, which expressed NY-ESO antigen and human PD-L1. MCLA-145 treatment at 5 mg/kg resulted in 54% tumor growth inhibition (TGI) as compared to T cell only–treated mice. In the tumors of MCLA-145–treated mice, the percentage of NY-ESO specific CD8+ T cells were significantly increased compared with controls. In a second model, mice engrafted with human CD34+ cells were implanted with the breast tumor cell line MDA-MB-231. MCLA-145 at 0.5 mg/kg and 5 mg/kg induced significant tumor growth inhibition (55% and 57%, respectively) as compared to vehicle control or Fc-silenced huIgG1 controls. Additionally, 2 out of 9 animals in the 5 mg/kg MCLA-145–treated group had complete tumor regression. MCLA-145 increased the number of infiltrating CD8+ T cells, as well as the percentage of central memory CD8+ T cells.

**Conclusions**

MCLA-145 is currently undergoing clinical development (NCT03922204).

#### P490 Antibody derived from an elite responder to checkpoint inhibitor therapy relieves immunosuppression by tumor associated macrophages

##### Randi Simmons, Siddarth Chandrasekaran, Melissa Conerly, Tyrel Smith, Sam Lam, Jacqueline Pham, Ray Fox, Darbie Whitman, Meghan Zuck, Sara Carbonetti, Kamal Puri, PhD

###### Oncoresponse Inc, Seattle, WA, United States

####### **Correspondence:** Kamal Puri (kpuri@oncoresponseinc.com)

**Background**

Tumor-associated macrophages (TAM)s in the tumor microenvironment (TME) contribute to tumor immune evasion by suppressing anti-tumor immune responses and by promoting a tumorigenic milieu. High infiltration of immunosuppressive myeloid cells generally predicts unfavorable prognosis. Reduction or repolarization of suppressive myeloid cells is an attractive strategy to enhance clinical responses to immune checkpoint inhibitor (CPI) therapy. Cancer patients who achieved durable response to CPI therapy (elite responders) may harbor antibodies that contribute to clinical response by promoting an anti-tumor TME.

**Methods**

B cells derived from elite responders were cloned and screened for IgG antibodies binding to myeloid derived suppressor cells. Hits were prioritized based on myeloid binding profiles and their variable-regions sequenced, cloned, and expressed as recombinant IgG1. Cloned antibodies underwent further characterization to evaluate their ability to reverse the immunosuppressive effects of myeloid cells in assays modelling the TME. Primary human monocytes and T cells were used to interrogate antibody-dependent immunomodulatory responses in vitro. A humanized mouse model was used to evaluate the anti-tumor activity of the lead antibody, OR2805.

**Results**

The target of OR2805 is highly expressed on TAMs and M2-like macrophages. OR2805 does not bind to other hematopoietic cells nor a panel of human primary non-immune cells. The antibody stains positively on M2-like TAMs from primary human lung tumor samples. OR2805 treatment reduces expression of cell-surface markers associated with tumor-promoting M2c-like macrophages. In co-culture assays, OR2805 relieves the suppressive effect of M2 macrophages and resulted in increased T cell activation and proliferation, upregulation of T cell activation markers, and enhanced T cell-mediated tumor cell killing. Administration of OR2805 in humanized NSG-SGM3 mouse tumor models resulted in approximately 50% reduction in A549 tumor growth and a 60% reduction in H1975 tumor growth. In this model, OR2805 treatment significantly increased the proportions of human CD8+ T cells and human CD11b+ myeloid cells in the spleen as well as significantly enhanced expression of activation markers (ICOS, OX-40) by human CD8+ T cells.

OR2805 reduces TAM-mediated immunosuppression and enhances anti-tumor immune responses. OR2805 treatment induces robust anti-tumor activity in lung cancer xenograft models in humanized mice. This data justifies further development of OR2805 as anti-cancer therapy in combination with other CPI treatments. OR2805 has the potential to increase the number of patients who may benefit from current CPI therapy.

#### P491 Preclinical development of a novel TNFRSF25 agonist antibody, PTX-35, for cancer immunotherapy combinations

##### Matthew Seavey, PhD, Jayalakshmi Miriyala, MS, Jason Rose, MS, Vikas Tahiliani, PhD, Patrick Dillon, PhD, Elena Gorovits, PhD, Jeff Hutchins, PhD, Rahul Jasuja, PhD

###### Heat Biologics, Inc., Durham, NC, United States

####### **Correspondence:** Matthew Seavey (mseavey@heatbio.com)

**Background**

Tumor Necrosis Factor Receptor Super Family 25 (TNFRSF25), also known as Death Receptor 3 (DR3), is preferentially expressed by activated and antigen-experienced T-cells. TNFRSF25 is a potent costimulatory molecule, similar to OX40, 4-1BB and GITR. PTX-35 was developed as a humanized, affinity matured, IgG2 mAb against TNFRSF25 for use with current cancer immunotherapy options, including cancer vaccines. All pharmacology and IND-enabling PK and toxicology has been completed for PTX-35 [1-6].

**Methods**

Previous proof-of-concept studies were completed elsewhere. Pharmacology studies described here were completed with a surrogate antibody, mouse-IgG1-PTX-35 (mPTX-35). Regulatory T-cell expansion studies were completed with Foxp3-RFP+ transgenic mice (FIR). CD8+ T-cell expansion studies were conducted with adoptively transferred OVA-specific, TCR-transgenic, CD8+ T-cells (OT-1). Human PTX-35 was tested in 28-day mouse, and 2-week and 8-week non-human primate, toxicology studies. Human, mouse, and monkey, in vitro, tissue-cross reactivity tests were also performed to check species cross-reactivity. Human PTX-35 was also tested in a human PBMC stimulation assay, in vitro, to check for impact on proliferation and cytokine release.

**Results**

TNFRSF25-engagement in mice expanded antigen specific CD8+ T-cells when given in the context of vaccination (6 and 19-fold increase in CD8+ T-cells in blood over vaccination alone at peak and boost, respectively), and Tregs were expanded in vaccine absence in FIR animals (2-fold increase in CD4+ Foxp3+ T-cells in blood). The MABEL and NOEL, in FIR mice, using mPTX-35, was determined to be 0.01 mg/kg and 0.001 mg/kg, respectively. The 2-week PTX-35 treatment of cynomolgus monkeys confirmed findings in mice, that treatment results in the expansion of Teff and Treg cell subsets. Intravenous bolus injection once every 2-weeks over an 8-week period was well tolerated giving a NOAEL of 100 mg/kg. Species cross-reactivity was observed for mouse, human and monkey tissues. No adverse events were recorded for the 28-day mouse toxicology study. Testing PTX-35 in a human PBMC, anti-CD3 proliferation assay, in vitro, showed that TNFRSF25-engagement provided the necessary costimulation to drive cellular division at sub-optimal concentrations of anti-CD3 (2-fold increase), providing the necessary in vitro, human proof-of-concept data, which was consistent across four different human blood donors.

**Conclusions**

PTX-35 is a potent costimulatory agonist targeting a novel pathway that can work in concert with cancer vaccines. Due to the ability of PTX-35 to possibly stimulate both pro-inflammatory and anti-inflammatory pathways, depending on treatment context, therapeutic modulation can provide numerous opportunities for cancer and inflammatory diseases.

**Acknowledgements**

Pelican Therapeutics would like to thank the Cancer Prevention and Research Institute of Texas (CPRIT) that helped fund these studies. We would also like to thank Dr. Natasa Strbo for 4C12 antibody and Dr. Robert Levy for mouse TL1A-Ig and advice, both located at the University of Miami.

**References**

1. Schreiber TH, Podack ER. Immunobiology of TNFSF15 and TNFRSF25. Immunologic Research, December 2013, Volume 57 (issue 1-3), pp. 3-11

2. Schreiber T.H., Levy R.B., and Podack E.R.; (2010). Therapeutic Treg expansion in mice by TNFRSF25 prevents allergic lung inflammation. J Clin Invest.; 120(10):3629-3640

3. Melero I., Hirschhorn-Cymerman D., Morales-Kastresana A., Sanmamed M.F., and Wolchok J.D.; (2013). Agonist Antibodies to TNFR Molecules That Costimulate T and NK Cells. Clin Cancer Res.; 19(5); 1044–53

4. Slebioda T.J. et al.; (2011). Triggering of TNFRSF25 promotes CD8+ T-cell responses and anti-tumor immunity. Eur J of Immunol 41 (9), 2606-2611

5. Schreiber T.H., et al.; (2014). Comparative combination cancer immunotherapy with vaccination and TNFRSF stimulation. Society of Immunotherapy of Cancer (SITC) Conference, Poster: https://d1io3yog0oux5.cloudfront.net/_675812118f795f2435017262bf9d3804/heatbio/db/527/5588/pdf/2014-AACR_gp96-Ig-and-Costim-Combos.pdf

6. Nishikii H. et al.; (2016). DR3 signaling modulates the function of Foxp3+ regulatory T cells and the severity of acute graft-versus-host disease. Blood 128 (24), 2846-2858

#### P492 A new generation anti-TGFβ antibody, SAR439459, relieves immunosuppression and improves anti-tumor efficacy of PD1 blockade

##### Rita Greco, Hongjing Qu, Joachim Theilhaber, Gary Shapiro, Richard Gregory, PhD, Christopher Winter, Natalia Malkova, Lily Pao, Mikhail Levit, Alexei Protopopov, Jack Pollard, PhD, Tun Tun Lin, MD, Dmitri Wiederschain, Sharad Sharma, PhD

###### Sanofi, Cambridge, MA, United States

####### **Correspondence:** Sharad Sharma (sharad.sharma@sanofi.com)

**Background**

TGFβ is a potent immunosuppressive cytokine that acts on multiple cell types of the innate and adaptive arms of the immune system within the tumor microenvironment. Emerging data implicates role of TGFβ in tumor immune evasion, resistance to cancer therapy and poor prognosis. We report preclinical data on SAR439459, a new pan anti-TGFβ antibody, which is capable of neutralizing all active isoforms of human and murine TGFβ.

**Methods**

Cancer patient databases were analyzed for various gene signatures. In vitro experiments were performed to examine the efficacy of SAR439459 in preventing TGFβ-mediated suppression of primary human T and NK cells. To evaluate anti-tumor efficacy, MC38 and EMT6 mouse tumor models were treated with SAR439459, anti-PD1, or combination. MC38 model was used to examine immune cell functions ex vivo.

**Results**

TGFβ was found to be upregulated in cancer patients and its increased activation correlated with reduced overall survival (OS) in PD-1 refractory cancer patients. SAR439459 blocked TGFβ-mediated suppression of human T and NK cells. TGFβ impaired the activity of anti-PD1 mediated T cell response, while SAR439459 restored this activity. In MC38 and EMT6 tumor models, treatment with SAR439459 resulted in anti-tumor efficacy; the combination of SAR439459 with anti-PD1 enhanced this activity and resulted in complete tumor regression and elicited a prolonged anti-tumor response.

**Conclusions**

This data demonstrates that combination of SAR439459 with anti-PD1 generates a strong anti-tumor immune response and improves anti-tumor efficacy in several tumor models. The preclinical data presented here formed the basis for the ongoing clinical investigation of SAR439459 in cancer patients.

#### P493 High-dimensional analysis delineates modulation of myeloid and lymphoid compartments with STAT3 ASO and PDL-1 combination therapy

##### Theresa Proia, PhD, Maneesh Singh, PhD, Larissa Carnevalli, PhD, Gayathri Bommakanti, PhD, Nanhua Deng, Matthew Griffin, Lukasz Magiera, Adina Hughes, Laura Prickett, Patricia McCoon, PhD, Corinne Reimer, Simon Barry, PhD

###### AstraZeneca, Waltham, MA, United States

####### **Correspondence:** Theresa Proia (theresa.proia@astrazeneca.com); Gayathri Bommakanti (gayathri.bommakanti@astrazeneca.com)

**Background**

STAT3 is a ubiquitously expressed transcription factor and master regulator of immune suppression in the tumor microenvironment (TME). Danvatirsen, a therapeutic antisense oligonucleotide (ASO) that selectively targets STAT3, has shown clinical benefit alone and in combination with durvalumab (anti-PDL1) and is currently in Phase 1/2 clinical studies.

**Methods**

To gain mechanistic insight into the therapeutic response induced by mouse surrogate STAT3 ASO in the CT26 syngeneic mouse tumor model, we have used two complementary forms of high-dimensional profiling; mass cytometry (CyTOF) and flow cytometry. We supported the in vivo mouse findings with in vitro studies in human macrophages treated with danvatirsen.

**Results**

Multidimensional immune profiling studies provided key mechanistic observations: (1) Robust reduction of total STAT3 protein in myeloid lineage cells, including an 80% reduction in macrophages and 50% reduction in dendritic cells, but not in CD8+ T cells. (2) In the combination treatment arm, STAT3 ASO treatment promoted a two-fold reduction of intratumoral immunosuppressive macrophages, doubling of iNOS positive activated macrophages and enhanced proliferation and IFNγ production from tumor antigen specific T cells. (3) The tumor-associated monocyte/macrophage compartment is highly complex and dynamic and displays a spectrum of activation states ranging from a predominantly anti-inflammatory phenotype (F4/80+ CD206+ IL4r+ MerTK+; six fold higher) in progressively growing control tumors to a predominantly proinflammatory phenotype (F4/80+ iNOS+ CCR2+; three fold higher) in responding tumors from combination treated groups.

In vitro, human macrophages were highly sensitive to danvatirsen treatment, with an IC50 of 60nM for total STAT3. Human ‘M2-like’ macrophages were generated in the presence of IL10 and M-CSF and treated with danvatirsen, which promoted an increase in IFNγ, IL-12, and TNFα, as well as increased CD80/CD86 expression, consistent with polarization from a suppressive phenotype to a pro-inflammatory phenotype.

**Conclusions**

Our data support the hypothesis that STAT3 reduction in the myeloid lineage results in activation of macrophages entering the TME and enhanced effector T cell responses in combination with checkpoint inhibition. Our ongoing work is focused on exploring the effects of STAT3 reduction in other key immune cells in which we have observed robust knockdown including Tregs, endothelial cells, and CAFs.

#### P494 A novel TNFRSF25 agonist, PTX35, synergizes with Gp96-Ig/OX40L-Ig to enhance effector and memory anti-tumor CD8+ T cell responses and delay tumor growth

##### Vikas Tahiliani, Patrick Dillon, PhD, Jayalakshmi Miriyala, MS, Jason Rose, MS, Anh Trinh, Rahul Jasuja, PhD, Jeff Hutchins, PhD, Matthew Seavey, PhD

###### Heat Biologics, Inc., Durham, NC, United States

####### **Correspondence:** Matthew Seavey (mseavey@heatbio.com)

**Background**

Heat (Heat) Biologics has developed a next generation cellular vaccine platform that incorporates a tumor antigen chaperone (gp96-Ig) in a tumor cell line and a host of over-expressed cancer associated neoantigens. Viagenpumatucel-L (HS110), a human lung adenocarcinoma cell line, stably transfected to express gp96-Ig, is being tested in a phase 1/2 clinical trial (NCT#02439450) for NSCLC. Heat has recently developed HS130, an allogeneic cell-based vaccine, designed to secrete tumor-associated antigens along with a costimulatory molecule, OX40L. Preclinical results of mouse HS130 (mHS130) in combination with mouse HS110 (mHS110) has shown a potent anti-tumor effector and memory CD8+ T cell response, followed by tumor regression. In our current study, we further characterized the role of mHS110 and mHS130 in combination with an agonist TNFRSF25 monoclonal antibody, PTX35. PTX35 is a potent stimulator of effector and memory CD8+ T cell responses, which taken-together with HS110 and HS130 has the potential of treating human cancers [1-7].

**Methods**

To study expansion, contraction, and maintenance of tumor-specific CD8+ T cell responses, mHS110 and/or mHS130, in combination with different doses of mouse-IgG1-PTX35 (mPTX35) was administered to C57BL/6 mice that were adoptively transferred with syngeneic OVA-specific T cells (OT-I). Mice were then challenged with murine melanoma tumors (B16F10-OVA) to characterize the tumor-specific immune cells in the periphery, spleen, and tumor-microenvironment that were involved in tumor regression.

**Results**

Combination of mPTX-35 with mHS-110 and mHS130 increased the expansion of tumor-specific CD8+ T-cells, in a mPTX-35 dose-dependent manner. This cellular expansion was significantly higher in the 1 mg/kg dose of mPTX-35 and far exceeded the additive value of mPTX-35, mHS130, and mHS110 treatment alone. Systemic administration of mPTX35, in combination with mHS110 and mHS130, led to a significant increase in the expansion of activated CD8+ T cells in the blood and stimulated activation of KLRGhi IL7Rlo short-lived effector cells (SLECs). Importantly, this combination resulted in higher frequencies of tumor infiltrating lymphocytes (TILs), which enhanced regression of established B16F10-OVA tumors and increased overall survival.

**Conclusions**

These results strongly suggest that mPTX35 synergizes with mHS110 and mHS130 to amplify activated tumor-specific CD8+ T cells, program a strong memory response, and allow for tumor regression. The combinations of these three treatments in the clinic may translate into an efficacious approach to treating human cancers.

**Acknowledgements**

Pelican Therapeutics would like to thank the Cancer Prevention and Research Institute of Texas (CPRIT) that helped fund these studies. We would also like to thank Dr. Natasa Strbo for 4C12 antibody and Dr. Robert Levy for mouse TL1A-Ig and advice, both located at the University of Miami.

**References**

1. Schreiber TH, Podack ER. Immunobiology of TNFSF15 and TNFRSF25. Immunologic Research, December 2013, Volume 57 (issue 1-3), pp. 3-11

2. Schreiber T.H., Levy R.B., and Podack E.R.; (2010). Therapeutic Treg expansion in mice by TNFRSF25 prevents allergic lung inflammation. J Clin Invest.; 120(10):3629-3640

3. Melero I., Hirschhorn-Cymerman D., Morales-Kastresana A., Sanmamed M.F., and Wolchok J.D.; (2013). Agonist Antibodies to TNFR Molecules That Costimulate T and NK Cells. Clin Cancer Res.; 19(5); 1044–53

4. Slebioda T.J. et al.; (2011). Triggering of TNFRSF25 promotes CD8+ T-cell responses and anti-tumor immunity. Eur J of Immunol 41 (9), 2606-2611

5. Schreiber T.H., et al.; (2014). Comparative combination cancer immunotherapy with vaccination and TNFRSF stimulation. Society of Immunotherapy of Cancer (SITC) Conference, Poster: https://d1io3yog0oux5.cloudfront.net/_675812118f795f2435017262bf9d3804/heatbio/db/527/5588/pdf/2014-AACR_gp96-Ig-and-Costim-Combos.pdf

6. Nishikii H. et al.; (2016). DR3 signaling modulates the function of Foxp3+ regulatory T cells and the severity of acute graft-versus-host disease. Blood 128 (24), 2846-2858

7. Fromm G, de Silva S, Giffin L, Xu X, Rose J, Schreiber TH. Gp96-Ig/Costimulator (OX40L, ICOSL, or 4-1BBL) Combination Vaccine Improves T-cell Priming and Enhances Immunity, Memory, and Tumor Elimination. Cancer Immunol Res. 2016;4(9):766-778. doi:10.1158/2326-6066.CIR-15-0228

#### P495 Secondary immune resistance mechanisms induced by therapeutic cancer vaccines which prevent tumor regression and foster recurrences

##### Sjoerd Van der Burg, PhD, Elham Beyranvand Nejad, Camilla Labrie, Suzanne van Duikeren, Ing, Ramon Arens, PhD, Thorbald van Hall, PhD, Sjoerd van der Burg, PhD

###### Leiden University Medical Center, Leiden, Netherlands

####### **Correspondence:** Sjoerd van der Burg (shvdburg@lumc.nl)

**Background**

Immunotherapy may induce complete tumor regressions but often tumors partially regress followed by tumor recurrence. Here, we focused on the underlying mechanisms.

**Methods**

The TC-1 mouse tumor model in which different formulation and application of an HPV16 SLP vaccine results in full cure or tumor recurrence and therapy resistance after initial full tumor regression. Tumors, spleens and lymph nodes were analyzed by mass- and flow-cytometry. Mice were treated with antibodies to PD-1, PD-L1, OX-40, 4-1BB, NKG2A and TGFβ. Cell-sorted tumor cells were RNA sequenced. Immune parameters were assessed in 5-10 mice, survival analyses were performed on at least 10 mice per group. All experiments were performed 2-3 times.

**Results**

Optimal vaccination resulted in about 7% circulating tumor-specific CD8+ T cells and complete cure of all mice, whereas suboptimal vaccination led on average to 1.7% tumor-specific T cells and tumor regression followed by recurrence in all mice. Neither booster vaccinations, which increased the numbers of circulating tumor-specific type 1 cytokine-producing CD4+ and CD8+ T cells (p<0.01), nor the co-administration of (combinations of) antibodies against PD-1, PD-L1, 4-1BB, or OX-40 prevented tumor recurrence or improved survival after vaccination. Immune escape was intrinsic to the tumor cells as the direct reinjection of ex-vivo cell-sorted recurrent tumor cells into groups of 10 naïve hosts did not result in any response to vaccination while in all cases the reinjected ex-vivo cell-sorted nontreated tumor cells displayed vaccine-induced tumor regression followed by relapse (p<0.01). Ex-vivo analyses of escaped tumor cells showed no alterations in the expression of MHC-I or the E7 tumor-antigen or their sensitivity to tumor-specific CTL mediated killing. RNA sequencing on bulk sorted (CD45-) tumor cells from non-treated (n=4) and escaped (n=4) tumors, revealed a specific vaccine-induced downregulation of the TNF- and P53-signaling pathways and upregulation of TGFβ signaling. Indeed, more TGFβ positive fibroblasts surrounded the escaped tumors (p<0.05). Recurrent tumors displayed strongly reduced numbers of infiltrated CD8+ T cells (p<0.001), whereas this was not the case for escaped tumor cells-reinjected tumors. However, both types of escaped tumors displayed lower numbers of tumor-infiltrating Ly6C+MHCI-II+ inflammatory macrophages (p<0.01). TGFβ-blockade delayed but did not prevent relapses to recur (p=0.07). The use of inflammation inducing chemotherapy reinstalled the infiltration of tumors with inflammatory myeloid cells after vaccination, prevented relapse and reinstalled sensitivity of escaped tumors to therapeutic vaccination (p=0.01).

**Conclusions**

The sequential clinical phases during non-curative immunotherapy may involve several distinct secondary escape mechanisms.

#### P496 Inhibition of autophagy enhances multifunctional genetically-engineered NK cell-based immunotherapy of glioblastoma

##### Jiao Wang, PhD, Sandro Matosevic, PhD

###### Purdue University, West Lafayette, IN, United States

####### **Correspondence:** Sandro Matosevic (sandro@purdue.edu)

**Background**

Despite aggressive treatments, the median life expectancy for GBM patients is only around 15 months, highlighting the need for new therapeutic approaches. NK cells are showing potential for immunotherapy of GBM. However, NK cells struggle to cross the blood brain barrier (BBB) and infiltrate into GBM [1]. Moreover, the immunosuppressive tumor microenvironment (TME) impairs NK cell activity, for instance due to adenosine-mediated downregulation of NKG2D expression [2].

**Methods**

We developed an innovative NK cell-based immunotherapy for GBM that targets multiple “checkpoints” simultaneously, by combining 1) multifunctional NK cells which consist of a cleavable scFv targeting CD73 alongside dual chimeric antigen receptors directed against GD2 and NKG2D ligands and 2) inhibition of autophagy in GBM cells to sensitize them to NK cell lysis and promote NK cell infiltration into GBM via the secretion of GBM-specific chemoattractants.

**Results**

We have designed and synthesized a multifunctional CAR construct that expresses an anti-CD73 scFv which is cleavable by GBM-associated proteases, and a dual CAR that enables NK cells to avoid antigen escape common to GBM (Figure 1A). We have generated engineered NK-92 or primary human NK cells that efficiently express the construct, from which the anti-CD73 scFv could be functionally released via uPA treatment (Figure 1B and C). Engineered NK cells showed a significantly higher in vitro ability to kill patient-derived GBM43 targets (Figure 1D and E). To target autophagy, BECN1- GBM43 cells were generated, and their in vivo subcutaneous growth in RAG-1-/- mice validated the critical role of autophagy in GBM onset and progression (Figure 1F). We further showed that targeting autophagy inhibited the in vitro proliferation of GBM43 itself (Figure 1G), sensitized GBM to NK cell lysis (Fig. 1H), and induced elevated chemokine secretion (CCL5), which in turn increased NK cell migration across the BBB using an in vitro BBB model (Figure 1I).

**Conclusions**

We have generated multifunctional NK cells that can target multiple “checkpoints” at once showing improved cytotoxicity against GBM through increased resistance to the immunosuppressive TME via adenosinergic CD73 blockade and the ability to avoid antigen escape by GBM via dual CARs. We have also found that blocking the autophagy pathway in GBM displayed potent synergy with NK cell-mediated immunotherapy. Based on these results, to achieve combined therapeutic effects in vivo, we are currently evaluating this immunotherapy in an orthotopic GBM mouse model. Taken together, this approach provides a promising platform for the combination treatment of GBM with engineered NK cells.

**References**

1. Kmiecik J, Zimmer J, Chekenya M. Natural killer cells in intracranial neoplasms: presence and therapeutic efficacy against brain tumours. J Neurooncol. 2014 ;116(1):1-9.

2. Wang J, Lupo KB, Chambers AM, Matosevic S. Purinergic targeting enhances immunotherapy of CD73+ solid tumors with piggyBac-engineered chimeric antigen receptor natural killer cells. J Immunother Cancer. 2018;6(1):136.

**Fig. 1 (abstract P496).**
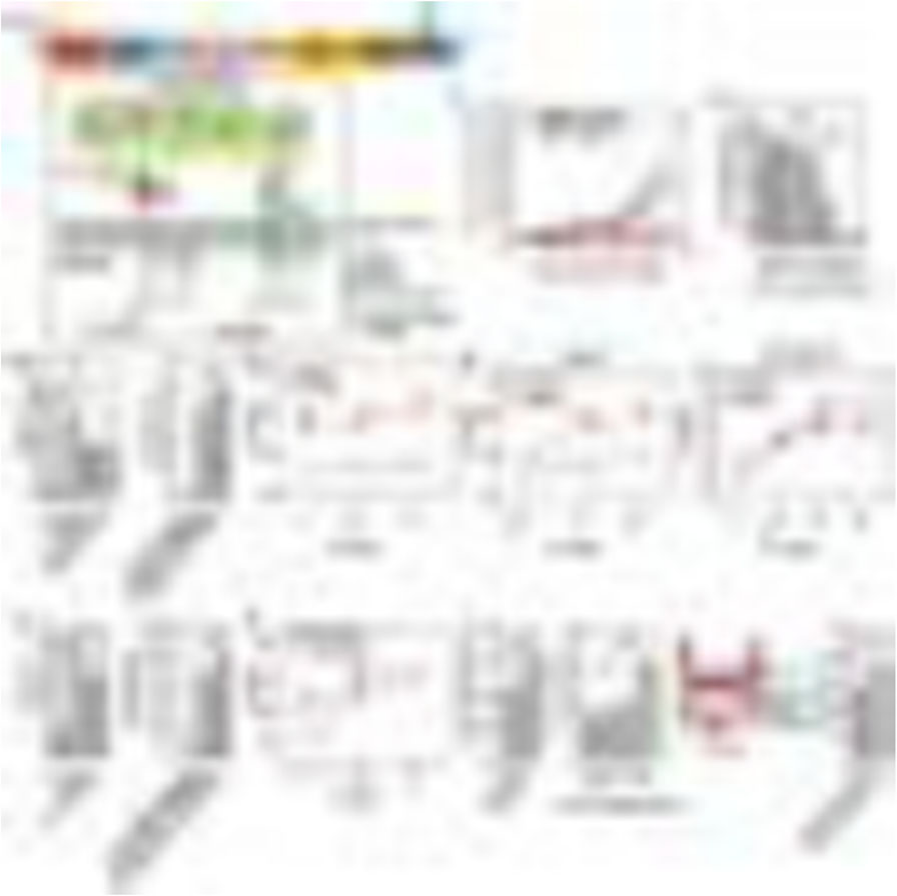
See text for description

#### P497 DRP-104, a novel broad acting glutamine antagonist, induces distinctive immune modulation mechanisms and synergistic efficacy in combination with immune checkpoint blockade

##### Yumi Yokoyama, PhD, Michael Nedelcovych, PhD, Robert Wild, PhD

###### Dracen Pharmaceutical, New York,, NY, United States

####### **Correspondence:** Robert Wild (rwild@dracenpharma.com)

**Background**

Glutamine is an essential amino acid for rapidly proliferating cancer cells, thus depriving the same fuel from immune cells and contributing to tumor immune evasion. DRP-104 was designed as a novel prodrug of the broad acting glutamine antagonist 6-Diazo-5-oxo-L-norleucine (DON). DRP-104 is inert in its prodrug form, affords high levels of plasma and gastro-intestinal (GI) tissue stability; has high tumor cell permeability and preferential tumor versus plasma/GI tissue distribution for DON. Here we sought to (1) compare immunological modulation of DRP-104 to anti-PD-1Ab, and (2) evaluate the combination effect of DRP-104 with PD-1/PD-L1 checkpoint inhibitors.

**Methods**

Immunomodulatory effects of DRP-104 as a single agent and combination with anti-PD-1Ab was evaluated in the CT26 mouse colon carcinoma model by flow cytometry and Luminex assay. In vivo anti-tumor efficacy of combination with anti-PD-1/PD-L1Ab was evaluated in CT26 and H22 hepatocellular carcinoma models.

**Results**

DRP-104 treatment showed broad immune cell modulation effects including increased T, NK, and macrophages; while anti-PD-1Ab affected mainly CD8+T cells. Cytokine modulation in tumor and plasma revealed that DRP-104 decreased pro-tumorigenic cytokines such as VEGF and KC(IL-8) while anti-PD-1Ab showed either no change or slight increase in these cytokines. CT26 bearing mice treated with anti-PD-1Ab alone, DRP-104, and the combination showed tumor growth inhibition at day 12 of 48%, 90%, and 94%, respectively. Median survival days were 31.5, 36, and 56 days, respectively (vehicle; 17.5 days). Notably 9 mice treated with combination of anti-PD-1 with DRP-104 were tumor free at end of the experiment (day 77) and 100% of these mice rejected a CT26 tumor re-challenge. In the H22 model, mice were treated with either anti-PD-L1 Ab, DRP-104, or combination. While anti-PD-L1Ab did not show tumor growth inhibition in this model, DRP-104 significantly inhibited tumor growth and the combination further enhanced efficacy, illustrated by extended survival for both DRP-104 alone (50 days) and combination (96 days) treatment groups compared to vehicle (33days) and anti-PD-L1 alone (33days). Combination treatment also resulted in long term durable cures in 50% of the mice.

**Conclusions**

DRP-104 treatment results in dramatic remodeling of the tumor micro environment, leading to enhanced function of multiple immune cells distinct from activities obtained by anti-PD-1 Ab. Combination therapy of DRP-104 with anti-PD-1/PD-L1 achieved significantly enhanced anti-tumor efficacy including long-term durable cures even in checkpoint inhibitor resistant models. This unique and non-overlapping mechanism of action supports clinical development of DRP-104 alone and in combination with PD-1/PD-L-1 checkpoint inhibitors.

#### P498 Blockade of PD-1 and LAG-3 on CD8+ T cells, induced by vaccination, elicits superior anti-tumor efficacy

##### Christopher Zahm, PhD, Douglas McNeel, MD, PhD, Laurne Delmastro

###### UW Carbone Cancer Center, Madison, WI, United States

####### **Correspondence:** Douglas McNeel (dm3@medicine.wisc.edu)

**Background**

T cell immune checkpoint receptors (ICR) and their ligands have emerged as a major mechanism by which tumors avoid immune detection. ICR blockade targeting PD-1/PD-L1 and/or CTLA-4 have revolutionized the treatment of many cancer types. However, not all cancers respond to ICR blockade, in large part mediated by the presence or absence of tumor infiltrating CD8+ T cells. We have focused on tumor vaccines as a means to increase the number of tumor-specific CD8+ T cells. We have previously demonstrated that activation of CD8+ T cells by vaccination leads to increased expression of specific ICR, and that blockade of these ICR with vaccination leads to better anti-tumor response than either alone. Differences in expression of specific ICR, notably PD-1 and LAG-3, appeared dependent on presentation of antigen by professional versus non-professional APC, hence we hypothesized that blockade of both of these ICR with vaccination should be superior to either alone.

**Methods**

In these studies we directly assessed the expression of PD-1, LAG-3, CTLA-4, and TIM-3 on CD8+ T cells following activation in the presence or absence of professional APC. Next, we transferred these cells into tumor bearing mice, alone or in combination ICR blocking antibodies, to directly evaluate their anti-tumor efficacy. Finally, we immunized tumor-bearing HLA-A2-transgenic mice with different anti-tumor DNA vaccines that have previously been shown to elicit CD8+ T cells preferentially expressing either PD-1 or LAG-3, and used each vaccine alone or in combination with ICR blockade.

**Results**

We found that PD-1, LAG-3, CTLA-4 and TIM-3 are all increased on CD8+ T cells after activation by professional APC, however LAG-3 alone was increased on CD8+ T cells activated in the absence of professional APC (Figure 1). When these cells were adoptively transferred into tumor bearing mice, LAG-3 blockade improved the anti-tumor efficacy of CD8+ T cells activated without APC, and PD-1 blockade improved the anti-tumor efficacy of CD8+ T cells activated by APC (Figure 2). Immunization with different DNA constructs [1-3] in combination with ICR blockade led to improved anti-tumor responses, however combining LAG-3 blockade with PD-1 blockade showed no benefit over PD-1 blockade alone (Figure 3).

**Conclusions**

These data support our previous finding that PD-1 blockade improves the efficacy of CD8+ T cells activated by vaccination. In this model, we detected no additional benefit to concurrent LAG-3 blockade. The role of other ICR in limiting anti-tumor immunity, and strategic blockade of these receptors following T-cell activation, is an area of active investigation.

**References**

1 Smith, H. A., Rekoske, B. T. & McNeel, D. G. DNA vaccines encoding altered peptide ligands for SSX2 enhance epitope-specific CD8+ T-cell immune responses. Vaccine 32, 1707-1715, doi:10.1016/j.vaccine.2014.01.048 (2014).

2 Rekoske, B. T., Smith, H. A., Olson, B. M., Maricque, B. B. & McNeel, D. G. PD-1 or PD-L1 Blockade Restores Antitumor Efficacy Following SSX2 Epitope-Modified DNA Vaccine Immunization. Cancer Immunol Res 3, 946-955, doi:10.1158/2326-6066.CIR-14-0206 (2015).

3 Colluru, V. T., Zahm, C. D. & McNeel, D. G. Mini-intronic plasmid vaccination elicits tolerant LAG3+ CD8 T cells and inferior anti-tumor responses. OncoImmunology, 0-0, doi:10.1080/2162402X.2016.1223002 (2016).

**Fig. 1 (abstract P498).**
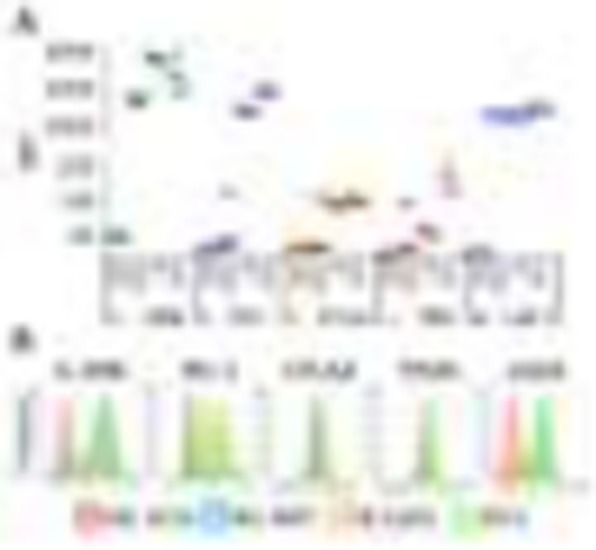
Priming with professional APCs leads to ICR expression

**Fig. 2 (abstract P498).**
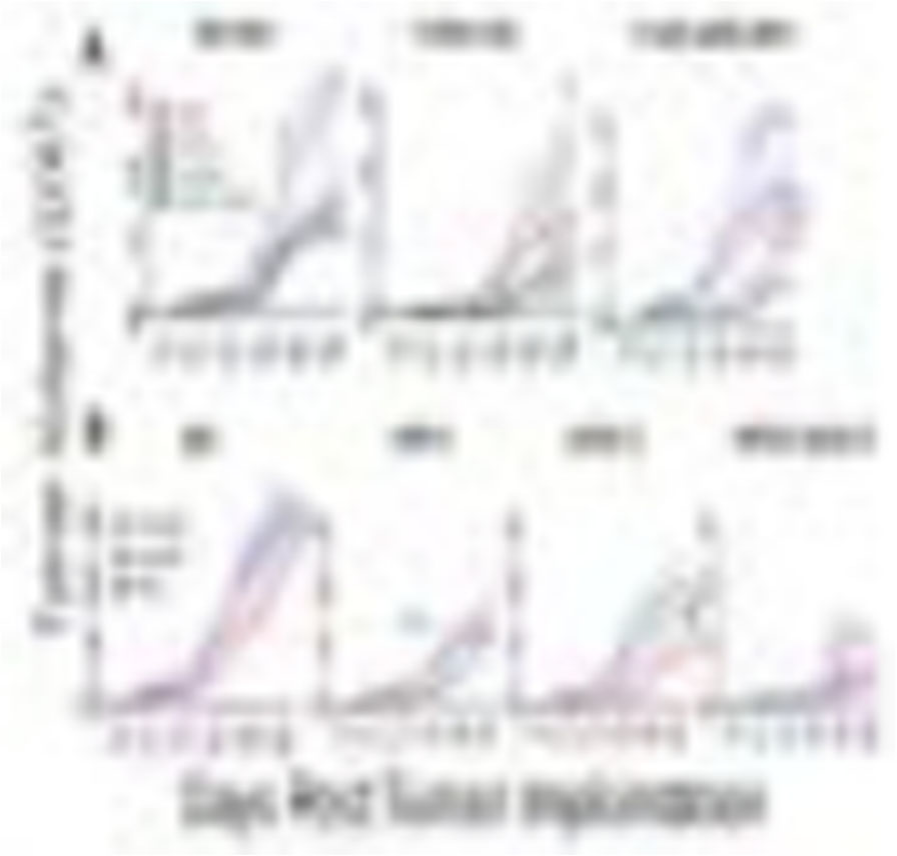
APC-induced ICR signaling compromised anti-tumor response

**Fig 3 (abstract P498).**
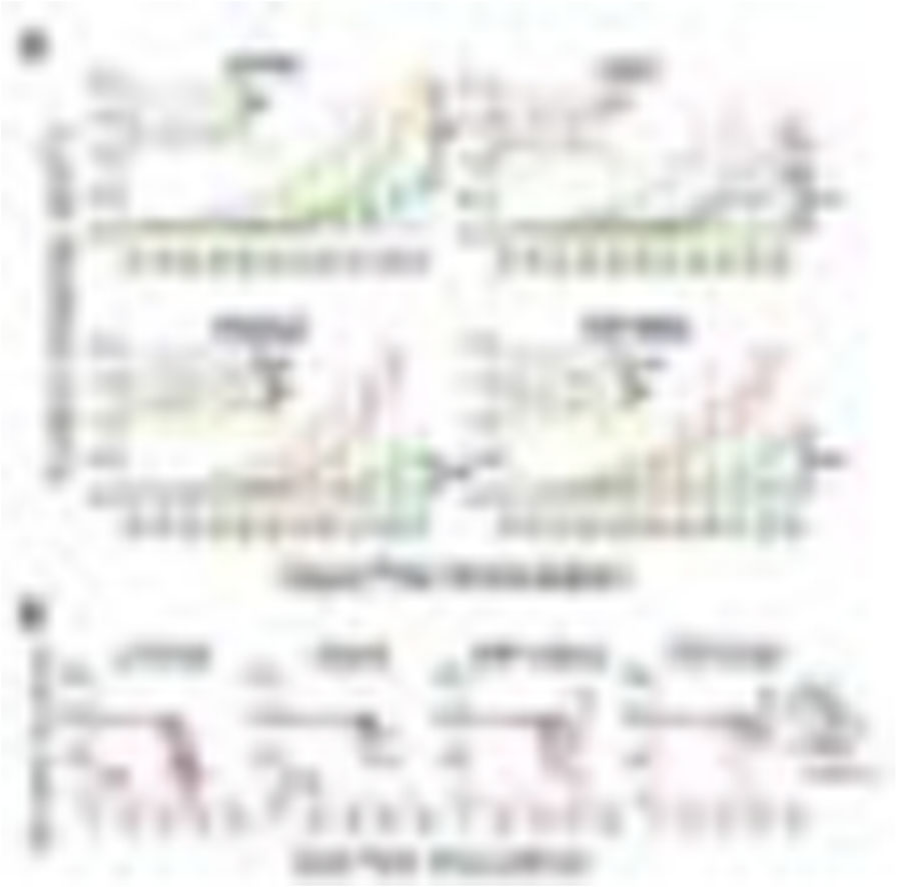
Some vaccines only effective when combined with ICR blockade

#### P499 Nano-Pulse Stimulation in combination with the TLR 7/8 agonist, resiquimod, synergizes to eliminate murine melanoma through innate and adaptive immune responses

##### Joel Benjamin, PhD, Amanda McDaniel, BA, Kristin von Rothstein, Sasha Farina, Bruce Freimark, PhD, Richard Nuccitelli, MS, PhD

###### Pulse Biosciences, Inc, Hayward, CA, United States

####### **Correspondence:** Richard Nuccitelli (rnuccitelli@pulsebiosciences.com)

**Background**

Nano-Pulse Stimulation (NPS) is a non-thermal treatment modality that provides high amplitude electrical energy pulses in the nanosecond range that is focal and directly acts on cellular structures and membranes to initiate regulated cell death. Previous work has shown that NPS induces release of tumor antigen and stimulates an in situ anti-tumor immune response [1, 2, 3]. The TLR 7/8 agonist resiquimod (RES) has been used as an immune adjuvant in previous cancer vaccine treatments in murine models to aid in antigen processing and presentation [4]. We have evaluated the combination of NPS and RES treatment to inhibit tumor growth and induce innate and adaptive immune responses.

**Methods**

The B16-F10 melanoma in C57BL/6j mice was used to investigate the potential combined effects of NPS and RES. B16-F10 tumor cells (2x10e5) were injected intradermally on the left flank and treated with NPS 5 days after inoculation. RES (50 μg) was then dosed in multiple combinations and timing to assess optimal tumor cell elimination and immune stimulation from the combination treatment. Tumor efficacy (volume) was measured twice per week. Immune biomarkers included flow cytometry and IHC of T cell and myeloid immune cells from tumors, draining lymph nodes and spleens.

**Results**

Low energy NPS and up to 3 doses of RES as monotherapies partially inhibit tumor growth. However, combination of NPS with 24-hour, post treatment of RES resulted in complete regression in a large fraction of treated animals. This efficacy is concomitant with increased antigen-specific and memory CD8 populations in the spleen and lymph node, as well as an increase in certain innate immune cell populations. Tumor regressions showed no sign of regrowth 90 days after treatment.

**Conclusions**

Compared to monotherapy treatments, the combination of NPS and RES treatments induce stronger tumor growth inhibition which persists as long-term tumor regression. Persistent tumor regression by combination treatment is associated with increases in B16F10 antigen-specific and memory CD8 populations, as well as increases in innate immune populations with potential for antigen presentation. These data support a mechanism by which NPS combined with TLR7/8 agonists activates an enhanced immune response.

**References**

1. Nuccitelli R, Berridge JC, Mallon Z, Kreis M, Athos B, Nuccitelli P. Nanoelectroablation of murine tumors triggers a CD8-dependent inhibition of secondary tumor growth. PLoS ONE. 2015; 10(7): e0134364 1-17.

2. Nuccitelli R, McDaniel A, Anand S, Mallon Z, Berridge JC, Uecker D. Nano-Pulse Stimulation is a physical modality that can trigger immunogenic tumor cell death. J Immunotherapy Cancer. 2017; 5:32 DOI 10.1186/s40425-017-0234-5.

3. Skeate JG, DaSilva DM, Chavez-Juan E, Anand S, Nuccitelli R, Kast W.M. Nano-Pulse Stimulation induces immunogenic cell death in human papillomavirus-transformed tumors and initiates an adaptive immune response. 2018; PLoS ONE. 13(1): e0191311

4. Caisova V, Vieru A, Kumzakova Z, Glaserova S, Husnikova H, Vacova N, Krejcova G, Padoukova L, Jochmanova I, Wolf KI, Chmelar J, Kopecky J, Zenka J. Innate immunity-based cancer immunotherapy: B16-F10 murine melanoma model. 2016; BMC Cancer.16(1); 940; DOI:10.1186/s12885-016-2982-x.

#### P500 Targeting PARP-1 with metronomic therapy as a new approach to modulate MDSC function and enhance anti-PD1 immunotherapy in colon cancer

##### Salome Valentina Ibba^1^, Mohamed Ghonim^1^, Abdelmetalab Tarhuni^1^, Matthew Dean^1^, Hamid Boulares, PhD^1^, Augusto Ochoa, MD^1^, Youssef Errami^1^, Ali Elbahraway^1^, Ilyes Benslimane^1^, Dorota Wyczechowska^1^, Luis Del Valle, MD^1^, Amir Al-Khami^2^, Hanh Luu^1^

###### ^1^Louisiana State University-Health Science Center, New Orleans, LA, United States; ^2^Tanta University, Tanta, Egypt

####### **Correspondence:** Hamid Boulares (hboulr@lsuhsc.edu)

**Background**

PARP inhibitors (PARPi) have important anti-tumor effects in BRCA-defective cancers but efficacy requires their use at/near maximum-tolerated-doses to achieve trapping of the enzyme on damaged chromatin. However, the benefits of targeting non-DNA repair aspects of PARP with low metronomic doses have not been explored.

**Methods**

Three models of colon tumorigenesis were used to conduct the present study (IACUC#3408), which included a colitis (AOM/DSS –induced), a spontaneous (APCMin-driven), and a syngeneic (engrafted with MC-38 or CT-26 cells) model. Several mutant mice were used in these models including PARP-1-/-, PARP-1-/+ APCMin/+, APCMin/+PARP-1-/-+/−, APCMin/+PARP-1-/+ as well as WT mice. Mice were randomized and assigned to the different experimental groups. Some groups of mice were administered olaparib, anti-mouse PD-1 antibodies, or a combination of the two agents. Mice were scarified according to the requirements of each model and tumor and tissues were collected for the analysis. MDSCs were generated by incubating bone marrow cells with GM-CSF, G-CSF, and IL-6. Tumor MDSCs were generated by enzymatic digestion of MC-38-engrafted tumors followed by positive selection. The suppression assay was performed by co-cultured with CD3/CD28-stimulated CFSE-labeled T cells and proliferation was assessed by FACS.

**Results**

Here, we show that partial PARP-1 inhibition via gene heterozygosity or a moderate olaparib dose was sufficient to protect against colitis- or APCMin-mediated intestinal tumorigenesis, while extensive inhibition via gene knockout or a high olaparib dose was ineffective or aggravated the burden despite anti-inflammatory effects and promotion of a tumor-suppressive microenvironment. A sub-IC50 metronomic dose of olaparib or PARP-1 heterozygosity was also sufficient to block tumorigenesis in syngeneic colon cancer models by modulating the suppressive function, but not differentiation or intratumoral migration, of myeloid-derived suppressor cells (MDSCs). These effects occurred through a reduction of arginase-1, iNOS, and COX-2 expression but independently of PARP-1-trapping on chromatin. Interestingly, the metronomic olaparib dose increased the intratumoral numbers, but not percentages, of CD8+ T cells. Adoptive transfer of WT bone marrow-derived MDSCs abrogated the protective effects of PARP-1 heterozygosity against the tumor burden. A metronomic olaparib dose was highly synergistic with anti-PD1-based immunotherapy leading to almost complete eradication of tumors on mice.

**Conclusions**

Our results support a paradigm-shifting concept that expands the utility of PARPi and encourage testing metronomic dosing of PARPi to enhance efficacy of check-point inhibitor-based immunotherapies not only in cancer of the colon but also that of other tissues ultimately benefiting a large proportion of cancer patients.

**Ethics Approval**

IACUC#3408

